
JOURNAL INFORMATION
==============================
NLM Title Abbreviation: Insights Imaging
Journal ID: Insights Imaging
NLM Journal ID: 101532453

Insights into Imaging

EISSN: 1869-4101
Publisher: Springer

ARTICLE INFORMATION
==============================
PMCID: PMC12270984
PMID: 40676413
DOI: 10.1186/s13244-025-02003-8
Article ID: 2003
Article version: 1
Subjects: Meeting Abstracts

ECR 2025 Book of Abstracts

Electronic publication date: 2025 Jul 17
Publication date: 2025 Dec
Volume: 16
Issue: Suppl 1
Issue sponsor: Publication of this supplement was funded by the European Congress of Radiology.
Electronic Location ID: 147
Copyright: © The Author(s) 2025
Copyright year: 2025
License: Open Access This article is licensed under a Creative Commons Attribution 4.0 International License, which permits use, sharing, adaptation, distribution and reproduction in any medium or format, as long as you give appropriate credit to the original author(s) and the source, provide a link to the Creative Commons licence, and indicate if changes were made. The images or other third party material in this article are included in the article’s Creative Commons licence, unless indicated otherwise in a credit line to the material. If material is not included in the article’s Creative Commons licence and your intended use is not permitted by statutory regulation or exceeds the permitted use, you will need to obtain permission directly from the copyright holder. To view a copy of this licence, visit http://creativecommons.org/licenses/by/4.0/.
License URL: https://creativecommons.org/licenses/by/4.0/

Conference name: European Congress of Radiology 2025
Conference location: Vienna, Austria
Conference date: 26 February - 2 March 2025
issue-copyright-statement: © European Society of Radiology (ESR) 2025

==============================

ECR 2025

Insights into Imaging
Volume 16
Supplement 1

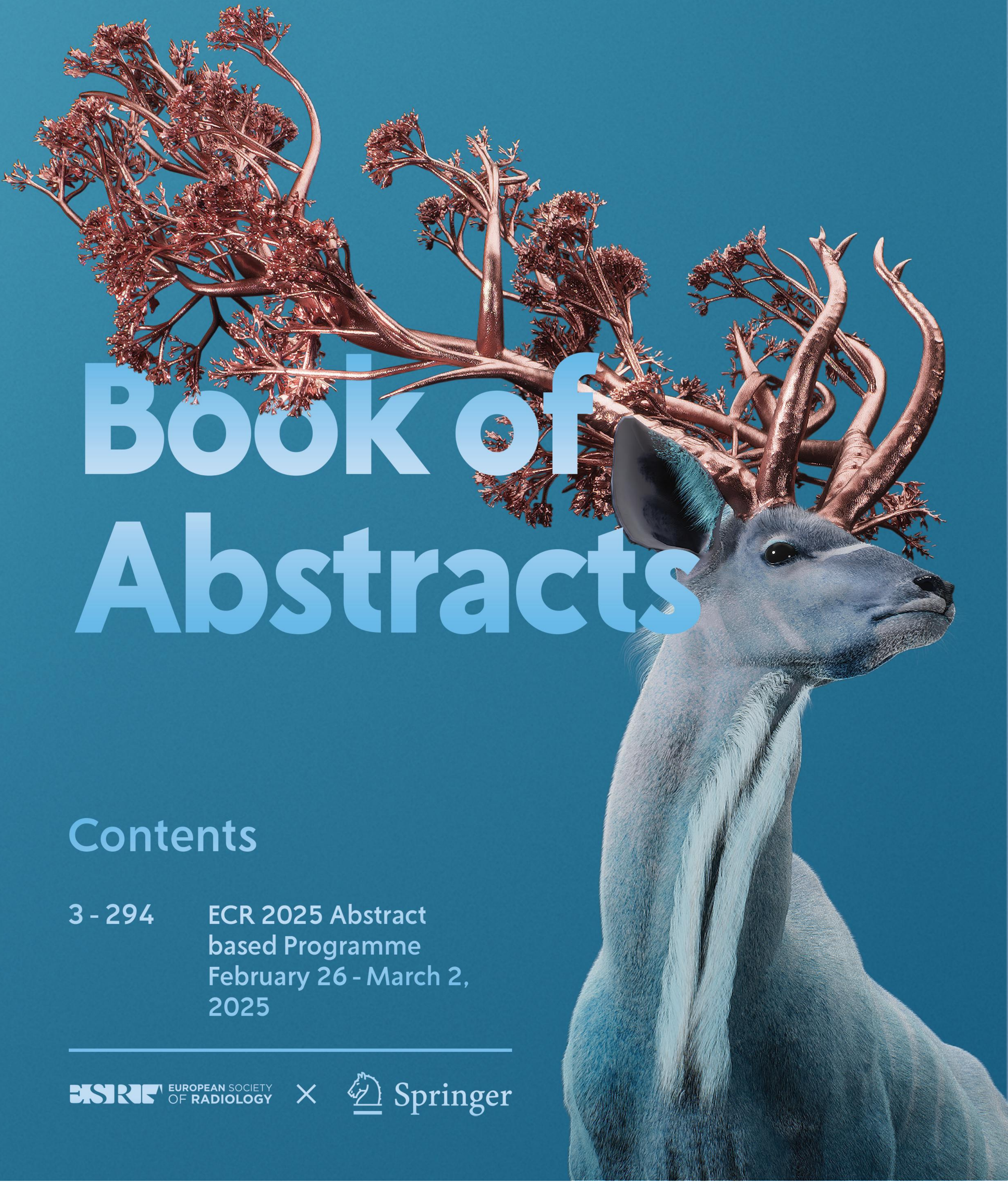

### Book of Abstracts

### Contents

3 - 294 ECR 2025 Abstract based Programme February 26 - March 2, 2025

ESR EUROPEAN SOCIETY OF RADIOLOGY × Springer

**Disclaimer**

The ECR 2025 Book of Abstracts is published by the European Society of Radiology (ESR) and summarises the presentations accepted to be held at the European Congress of Radiology 2025 (Vienna, Austria, February 26 - March 2, 2025).

Abstracts were submitted by the authors warranting that good scientific practice, copyrights and data privacy regulations have been observed and relevant conflicts of interest declared.
Abstracts reflect the authors' opinions and knowledge. The ESR does not give any warranty about the accuracy or completeness of medical procedures, diagnostic procedures or treatments contained in the material included in this publication. The views and opinions presented in ECR abstracts and presentations, including scientific, educational and professional matters, do not necessarily reflect the views and opinions of the ESR.

In no event will the ESR be liable for any direct or indirect, special, incidental, consequential, punitive or exemplary damages arising from the use of these abstracts.

The Book of Abstracts and all of its component elements are for general educational purposes for health care professionals only and must not take the place of professional medical advice. Those seeking medical advice should always consult their physician or other medical professional.

In preparing this publication, every effort has been made to provide the most current, accurate, and clearly expressed information possible. Nevertheless, inadvertent errors in information can occur. The ESR is not responsible for typographical errors, accuracy, completeness or timeliness of the information contained in this publication.

The ECR 2025 Book of Abstracts is a supplement to Insights into Imaging (1869-4101) and published under Creative Commons Attribution 4.0 International License.

Insights Imaging (2025) Vol 16 (Suppl 1)
https://doi.org/10.1186/s13244-025-02003-8

### ECR 2025
### Abstract-based Programme

Research Presentation Sessions (RPS)

**Wednesday, February 26 .............. 3**
**Thursday, February 27................ 72**
**Friday, February 28 ....................134**
**Saturday, March 1 ......................193**
**Sunday, March 2........................260**

### Wednesday, February 26

08:00-09:30 **Research Stage 1**

### Research Presentation Session: Abdominal and Gastrointestinal

### RPS 101

### What's going on in the pancreas?

**Moderator**
C. Ewertsen; Copenhagen/DK
*(caroline.ewertsen@dadlnet.dk)*
**Author Disclosures:**
Caroline Ewertsen: Speaker: Bracco 2023

**Improved pancreatic imaging with photon-counting ct**
*E. G. S. Brandt*[1], C. F. Müller[1], A. M. Ewald[1], Y. Wirenfeldt Nielsen[1], H. S. S. Thomsen[1], B. Ibragimov[2], M. Andersen[1]; [1]Herlev/DK, [2]Copenhagen/DK
*(Erikbr1980@gmail.com)*

**Purpose or Learning Objective:** The aim of this study is to investigate the image quality of pancreatic late arterial (LA) and portovenous phase (PV) pancreatic images from Photon-Counting CT (PCCT) scanners in comparison to conventional CT (EID-CT).
**Methods or Background:** We retrospectively identified 35 patients without suspicion of pancreatic pathology scanned on both EID-CT and PCCT in the period from October 2021 until December 2023. IV contrast was given according to patient weight and both a late arterial phase (LA) and a portovenous phase (PV) was performed. Image quality was rated on a 5-point Likert-scale (from 1=nondiagnostic to 5=optimal). Eleven different pancreatic parameters were scored by four radiology consultants. One reader made quantitative measurements of density and noise. All data analysis was performed with RStudio, version 2022.07.1. Continuous parameters were compared with a paired t-test and mean image quality ratings with a Wilcoxon signed rank test.
**Results or Findings:** Image quality was rated significantly higher on PCCT for the pancreatic parenchyma in the LA (3.87 vs 2.77, p<.001), the pancreatic parenchyma in the PV (3.31 vs 2.53, p<.001), pancreatic ducts (2.88 vs 2.62, p<.001), SMA (4.10 vs 2.74, p<.001), celiac axis (4.04 vs 2.70, p<.001) and portal vein (3.29 vs 2.52, p<.001). Noise levels were significantly lower with PCCT with a mean reduction of 5.8 HU across all parameters. DLP was significantly reduced with a 31.8% reduction (p< 0.01) for the LA and 21.5% (p< 0.01) for the PV.
**Conclusion:** Image quality was significantly improved for all evaluated pancreatic and peripancreatic structures with PCCT. Additionally, image noise and radiation dose were significantly reduced. The improved image quality with PCCT could potentially lead to improvements in the currently difficult evaluation of pancreatic diseases.
**Limitations:** No limitations were identified.
**Funding for this study:** Innovation Fund Denmark, Grant No. 1044-00015B.
**Ethics committee - additional information:** The study was approved by the national ethics committee with the number: nvk2215338.
**Author Disclosures:**
Michael Andersen: Speaker: Different Vendor Seminars, GE, Philips and Siemens
Anne Marie Ewald: Nothing to disclose
Henrik S. S. Thomsen: Nothing to disclose
Bulat Ibragimov: Nothing to disclose
Erik Gudmann Steuble Brandt: Nothing to disclose
Yousef Wirenfeldt Nielsen: Nothing to disclose
Christoph Felix Müller: Nothing to disclose

**Development and Validation of Contrast-enhanced CT-based Imaging Intratumor Heterogeneity of Pancreatic Ductal Adenocarcinoma**
*B. Zhao*, S. Ju; Nanjing/CN
*(zhaoben1207@163.com)*

**Purpose or Learning Objective:** To construct an imaging ITH (IITH) through radiomics methodology to effectively reflect the ITH of PDAC and explore its prognostic value.
**Methods or Background:** This study enrolled 961 patients with pathologically confirmed PDAC who had undergone preoperative contrast-enhanced computed tomography (CT) in two cohorts. Firstly, Tumor regions of interest were automatically segmented in both arterial and venous phase images. Radiomics features from these 2 phasea images were extracted based on PyRadiomics. Highly variable radiomic features with median absolute deviation > 1, were selected to assess the ITH. Similarity Network Fusion (SNF) was employed to identify distinct imaging heterogeneity phenotypes in the discovery cohort (Cohort 1), and the identical criteria were applied to Cohort 2. Kaplan-Meier analysis was utilized to investigate the association between the identified imaging phenotypes and overall survival (OS).
**Results or Findings:** A total of 961 patients (mean age, 63.1 years ± 9.2 standard deviation; 460 men) from 2 cohorts were enrolled. And 3378 radiomics features were extracted for each patient. In the discovery cohort (Cohort 1, n = 637), 241 highly variable heterogeneity-related features were selected to identify IITH, and 283 patients were classified into a high-IITH subgroup. Patients with high-IITH in the discovery cohort presented significantly poorer OS compared to those with low IITH (median time, 20.6 vs. 37.1 months, P < 0.001). The identical criteria were applied to the validation cohort (Cohort 2, n = 324). Kaplan-Meier analysis also confirmed that patients with high-IITH (n =149) had shorter OS (median time, 16.4 vs. 26.3 months, P < 0.001).
**Conclusion:** We established a noninvasive radiomics method to evaluate the ITH of PDAC. Furthermore, we demonstrated the prognostic power of IITH.
**Limitations:** This study requires further multi-omics validation in the future.
**Funding for this study:** NSFC, No. 82330060, 92059202, 823B2040, 61821002 and 82372024)
**Ethics committee - additional information:** IEC for Clinical Research ofZhongda Hospital, Affliated to Southeast University
**Author Disclosures:**
Ben Zhao: Nothing to disclose
Shenghong Ju: Nothing to disclose

**Interobserver agreement of pancreatic tumor size measurement before and after neoadjuvant therapy: is MRI as reproducible as CT?**
*A. Licha*, C. Touloupas, A. Delpla, A. Pouvelle, M. Zins; Paris/FR

**Purpose or Learning Objective:** Assess inter-observer agreement of pancreatic ductal adenocarcinoma (PDAC) tumor size measurement on CT and MRI, before and after neoadjuvant therapy (NAT)
**Methods or Background:** We reviewed all patients with a histological diagnosis of PDAC at Paris Saint-Joseph Hospital, between 2010 and 2022, and who underwent CT and MRI, both before and after NAT. Three independent radiologists anonymously evaluated the large axial tumor axis on 2 CT acquisitions and 6 MRI sequences. Inter-observer agreement was assessed by intra-class correlation coefficients (ICCs) and by LOAM graphs (Bland & Altmann extension for multiple observers).
**Results or Findings:** The final population consisted of 50 patients. On CT exams, inter-observer agreement was excellent before NAT (ICC of 0.83 [0.73;0.90] at arterial phase and 0.84 [0.74;0.90] at portal-venous phase) and decreased but remained good after NAT (ICC of 0.66 [0.52;0.78] at arterial phase and 0.65 [0.51;0.77] at portal-venous phase). On MRI exams, inter-observer agreement was moderate to good before NAT (best sequence being T1 at arterial phase with ICC of 0.67 [0.53;0.79]) and decreased becoming moderate for all sequences after NAT (best sequence being T1 at late phase with ICC of 0.55 [0.37;0.71]).
**Conclusion:** Inter-observer agreement of PDAC great axe measurement is better on CT than on MRI, and decreases between pre-NAT and post-NAT imaging, both on CT and MRI. These results encourage to keep on performing technically perfect CT scans, without questioning the need for pre-operative hepato-pancreatic MRI in non-metastatic patients.
**Limitations:** This is a monocentric retrospective study.
**Funding for this study:** Inter-observer agreement of tumor size measurement is higher on CT than on MRI, both before and after NAT. Inter-observer agreement of tumor size measurement decreases following NAT, both on CT and MRI.
**Ethics committee - additional information:** This study was approved by our organization's Medical Research Ethics Group.
**Author Disclosures:**
Caroline Touloupas: Nothing to disclose
Marc Zins: Nothing to disclose
Arié Licha: Nothing to disclose
Alexandre Delpla: Nothing to disclose
Arnaud Pouvelle: Nothing to disclose

**A Radiomics-Based Model for Predicting Lymph Node Metastasis of Pancreatic Ductal Adenocarcinoma: A Multi-Center Study**
*B. Zhao*, S. Ju; Nanjing/CN
*(zhaoben1207@163.com)*

**Purpose or Learning Objective:** To develop a radiomics model to predict lymph node metastasis (LNM) in patients with pancreatic ductal adenocarcinoma (PDAC) and assess its value for clinical management.
**Methods or Background:** Patients with pathologically confirmed PDAC were retrospectively enrolled from four centers and divided into a training (n = 192), validation (n = 82), testing (n = 100), and clinical utilization cohort (n = 163).A radiomics model was constructed based on the arterial phase of computed tomography (CT) for predicting LNM. The areas under the curve (AUCs) were used to compare the performance between the radiomics model and other models. Subsequently, Kaplan-Meier analysis was used to validate the model's value for prognosis and therapy decisions.
**Results or Findings:** A total of 437 patients (mean age, 63.1 years ± 9.2 standard deviation; 253 men) were included. The radiomics model demonstrated AUCs of 0.84, 0.82, and 0.78 in the training, validation, and testing cohorts, respectively, superior to other models (all P < 0.05). Besides, LNM predicted by the radiomics model was strongly associated with overall survival (OS) (P < 0.001). Kaplan-Meier analysis also demonstrated that patients with a high risk of LNM had a worse prognosis (all P < 0.05). Furthermore, patients who were dissected with ≥ 15 LNs had a longer OS than those with fewer LNs dissected in the high-risk subgroup predicted by the radiomics model in the clinical utilization cohort (P = 0.002).
**Conclusion:** The radiomics model demonstrated impressive performance in predicting LNM and prognosis, indicating its potential for. therapy decisions.
**Limitations:** The model we developed should be validated in a prospective study.
**Funding for this study:** NSFC, No. 82330060, 92359304, 92059202, 823B2040, 61821002 and 82372024
**Ethics committee - additional information:** IEC for Clinical Research ofZhongda Hospital, Affliated to Southeast University
**Author Disclosures:**
Ben Zhao: Nothing to disclose
Shenghong Ju: Nothing to disclose

### CT-Based Early Indicators of Severe Pancreatic Fistula and Hemorrhage After Pancreatoduodenectomy

D. Palumbo, *A. Campisi*, V. Andreasi, F. Prato, S. Partelli, D. Tamburrino, M. Falconi, F. De Cobelli; Milan/IT

**Purpose or Learning Objective:** Postoperative pancreatic fistula (POPF) and postpancreatectomy hemorrhage (PPH) are major complications following pancreatoduodenectomy (PD). Despite their clinical importance, no tool currently exists to predict their occurrence or severity. This study aims to identify radiological characteristics that can aid in the early prediction and stratification of POPF and PPH.
**Methods or Background:** We retrospectively reviewed 399 patients who underwent PD at San Raffaele Hospital between January 2015 and December 2021. Patients included had at least one contrast-enhanced computed tomography (CE-CT) scan within 14 days post-surgery. Several radiological features were systematically assessed, including pancreaticojejunostomy (PJ) dehiscence, PJ defects, fluid collections, perianastomotic air bubbles, and pancreatic remnant density.
**Results or Findings:** Clinically relevant POPF occurred in 230 patients (57.9%), with 185 classified as grade B and 45 as grade C. PPH occurred in 61 patients (15.3%). PJ dehiscence was significantly associated with clinically relevant POPF (31% vs. 22%, p = 0.035), and PJ defects were more extensive in patients with severe POPF (median 7 mm vs. 5 mm, p = 0.001). Fluid collections, particularly above the PJ site, were also linked to the development of severe POPF (p < 0.001). Additional markers, such as stump pancreatitis, perianastomotic air bubbles, and lower pancreatic remnant density, were significantly more frequent in severe POPF cases.
**Conclusion:** These findings support the use of postoperative CT scans to prospectively identify patients at risk of developing severe POPF and PPH, allowing for better clinical management.
**Limitations:** Retrospective study design.
**Funding for this study:** None.
**Ethics committee - additional information:** Ethics committee approval number: 28/INT/2015
**Author Disclosures:**
Domenico Tamburrino: Nothing to disclose
Valentina Andreasi: Nothing to disclose
Diego Palumbo: Nothing to disclose
Massimo Falconi: Nothing to disclose
Antonino Campisi: Nothing to disclose
Stefano Partelli: Nothing to disclose
Francesco De Cobelli: Nothing to disclose
Francesco Prato: Nothing to disclose

### Correlation between celiac axis stenosis and complications after pancreatoduodenectomy

*Y. Shu*, Y. Dai, J. Wei, Q. Xu; Nanjing/CN
*(18227237972@163.com)*

**Purpose or Learning Objective:** We aimed to explore the correlation between celiac axis stenosis and complications after pancreatoduodenectomy.
**Methods or Background:** Patients who underwent pancreatoduodenectomy in our hospital pancreas center from January 2021 to December 2023 were retrospectively collected. The stenosis rate of celiac axis was measured on pre-operation arterial phase imaging of routine enhanced CT, and graded the severity of celiac trunk stenosis: no stenosis (< 30%), mild stenosis (30%-50%), and significant stenosis (≥50%). The incidence of postoperative complications was evaluated, and both univariate and multivariate logistic regression analysis were conducted.
**Results or Findings:** A total of 774 patients were included in the study, 205 (26.5%) had celiac axis stenosis: 144 (18.6%) with mild stenosis, and 61 (7.9%) with significant stenosis. Celiac axis stenosis was associated with pancreatic fistula (p<0.001), postoperative bleeding (p=0.033), and postoperative biliary leakage (p= 0.006). In multivariate logistic regression analysis, mild stenosis of the celiac axis was an independent risk factor for postoperative pancreatic fistula (OR 2.81, 95%CI 1.82-4.33, p<0.001), and significant stenosis of the celiac axis was an independent risk factor for postoperative biliary leakage (OR 4.91, 95%CI 1.27-19.04 , p=0.021).
**Conclusion:** Celiac axis stenosis was associated with the risk of complications after pancreatoduodenectomy. Surgeons may need to pay attention to the condition of celiac axis stenosis before pancreatoduodenectomy.
**Limitations:** Retrospective study. Single center.
**Funding for this study:** There was no funding for this study.
**Ethics committee - additional information:** None
**Author Disclosures:**
Yuping Shu: Nothing to disclose
Qing Xu: Nothing to disclose
Yuran Dai: Nothing to disclose
Jishu Wei: Nothing to disclose

### Cystic fluid non-invasive evaluation based on photon-counting detector CT spectral imaging in patients with pancreatic cystic lesions

*I. Dudás*, B. Lovász, M. Benke, Á. Szücs, P. N. Kaposi-Novák, A. Szijártó, P. Maurovich-Horvat, B. K. Budai; Budapest/HU

**Purpose or Learning Objective:** Differentiation between pancreatic cystic lesions is a challenging task for clinicians. Spectral imaging via photon-counting detector CT (PCD-CT) scanners allows the reconstruction of virtual monoenergetic images (VMI) enabling the measurement of Hounsfield unit (HU) densities at different keV and the generation of spectral absorption curves. Our study aimed to investigate whether mucinous and non-mucinous pancreatic cystic lesions (PCL) have different spectral absorption curves that could help the differential diagnostics.
**Methods or Background:** Our study included 74 patients with PCLs, 53 patients with mucinous cystic neoplasms and 21 with non-mucinous cystic pancreatic lesions diagnosed based on current practice guidelines. The spectral absorption curves were generated from the pancreatic-phase scans. The average densities were measured on the 70keV (HU70keV) and 40keV (HU40keV) virtual monoenergetic images (VMIs), by placing 3-3 circular regions of interest in PCL's cystic component parts and the density differences were calculated (HUdiff(40keV-70keV)). Kruskal-Wallis test with post-hoc Dunn's test was used for comparing the groups. The discrimination performance was assessed by receiver operating characteristic (ROC) curve analysis. The intraobserver reproducibility and interobserver reproducibility were evaluated by the intraclass correlation coefficient (ICC).
**Results or Findings:** On 70keV VMIs, no significant differences were found between the average densities of mucinous and non-mucinous PCLs cystic components, however, a significant difference was found in HUdiff(40keV-70keV) values (p<0.0001). The diagnostic performance of HUdiff(40keV-70keV) in differentiating between mucinous vs. non-mucinous PCLs had AUCs of 0.92 and 0.92 on the training and test datasets, respectively, with a good interobserver (ICC=0.82) and excellent intraobserver reproducibility (ICC=0.94).
**Conclusion:** Spectral absorption curve assessment of cystic components could be a useful additional measurement to facilitate the non-invasive differential diagnosis between mucinous and non-mucinous pancreatic cystic lesions.
**Limitations:** This was a single-center study with a retrospective study design.
**Funding for this study:** No funding was received for this study.
**Ethics committee - additional information:** The presented study was approved by the institutional ethics committee of our University (SE RKEB 256/2023).
**Author Disclosures:**
Attila Szijártó: Nothing to disclose
Pál N. Kaposi-Novák: Nothing to disclose
Pál Maurovich-Horvat: Nothing to disclose
Ákos Szücs: Nothing to disclose
Ibolyka Dudás: Nothing to disclose
Márton Benke: Nothing to disclose
Borbála Lovász: Nothing to disclose
Bettina Katalin Budai: Nothing to disclose

**CT-Derived Body Composition Metrics Predict Severity in Acute Pancreatitis: A Post-Hoc Multicenter Study**

*R. Z. Borbély*[1], B. Teutsch[1], V. Vass[1], K. Márta[1], B. Erőss[1], A. Vincze[2], A. Szentesi[1], P. Hegyi[1], N. Faluhelyi[2]; [1]Budapest/HU, [2]Pécs/HU
*(drborbelyruben@gmail.com)*

**Purpose or Learning Objective:** Accurately predicting the severity of acute pancreatitis (AP) remains a significant clinical challenge. CT scans, traditionally used for diagnosis, also provide valuable insights into body composition. However, the prognostic utility of CT-derived body composition metrics has been inconsistently reported. This study aimed to evaluate whether key CT body composition metrics can effectively predict the severity of AP.
**Methods or Background:** A post-hoc analysis was conducted on a multicenter study involving 437 AP patients who underwent CT scans within the first 24 hours of hospital admission. Measurements of visceral adipose tissue (VAT), subcutaneous adipose tissue (SAT), and skeletal muscle area (SMA) were obtained at the third lumbar vertebra level. These areas were normalized for patient height to calculate the Visceral Adipose Tissue Index (VATI), Subcutaneous Adipose Tissue Index (SATI), and Skeletal Muscle Index (SMI). Muscle radiodensity in Hounsfield Units (HU) was assessed to determine fatty infiltration. The fat-to-muscle volume ratio was also calculated. These metrics were analyzed as potential predictors of severe AP using receiver operating characteristic (ROC) curves and area under the curve (AUC) values. Severity was determined using the Modified CT Severity Index (mCTSI). Statistical analyses were performed using IBM SPSS Statistics.
**Results or Findings:** The fat-to-muscle ratio demonstrated the highest predictive accuracy for severe AP (AUC = 0.68), followed by VATI (AUC = 0.65). Other indices did not show significant predictive potential (AUC < 0.6).
**Conclusion:** CT-derived body composition metrics, particularly the fat-to-muscle ratio and VATI, are valuable predictors of severe acute pancreatitis. Incorporating body composition analysis into routine CT evaluations may enhance prognostic assessments for AP patients.
**Limitations:** As a post-hoc analysis of retrospective data, the study may be subject to selection bias, potentially limiting the generalizability of the findings.
**Funding for this study:** Funding for Ruben Zsolt Borbély was supported by the EKÖP-2024-239 New National Excellence Program of the Ministry for Culture and Innovation from the source of the National Research Development and Innovation Fund. Funding for Brigitta Teutsch was provided by the ÚNKP-22-3 New National Excellence Program of the Ministry for Innovation and Technology from the source of the National Research, Development and Innovation Fund (to BT - ÚNKP-22-3-IPTE-1693). Center costs were covered by the University of Pécs, the Momentum Grant of the Hungarian Academy of Sciences (LP2014-10/2014), and grants from the National Research, Development, and Innovation Office (GINOP-2.3.2-15-2016-00015, KH-125678). The funders had no influence on the study design, data collection, analysis, or manuscript preparation.
**Ethics committee - additional information:** This post-hoc analysis is based on a study that received ethical approval from the Scientific and Research Ethics Committee of the Medical Research Council (ISRCTN63827758, decision 55961-2/2016/EKU).
**Author Disclosures:**
Bálint Erőss: Nothing to disclose
Nándor Faluhelyi: Nothing to disclose
Péter Hegyi: Nothing to disclose
Katalin Márta: Nothing to disclose
Ruben Zsolt Borbély: Nothing to disclose
Aron Vincze: Nothing to disclose
Brigitta Teutsch: Nothing to disclose
Vivien Vass: Nothing to disclose
Andrea Szentesi: Nothing to disclose

**Role of incidental pancreatic calcifications on computed tomography as opportunistic biomarker for chronic pancreatitis**

*A. Pata*, F. Rizzetto, C. B. Monti, A. Vanzulli; Milan/IT
*(annamaria.pata@unimi.it)*

**Purpose or Learning Objective:** To evaluate whether incidentally detected pancreatic calcifications on computed tomography (CT) serve as a reliable biomarker for chronic pancreatitis as defined by clinical criteria.
**Methods or Background:** We retrospectively reviewed CT scans from adult patients between 2014 and 2024, identifying cases where "pancreatic calcifications" were mentioned in the radiology report. Patients with known history of pancreatitis or pancreatic surgery were excluded. For each patient, we recorded pancreatic size, calcification characteristics (number, size, and location), and other features of chronic pancreatitis, such as duct dilatation or intraductal calculi. Clinical data, including pain, abdominal symptoms, and risk factors for chronic pancreatitis, were also collected.
**Results or Findings:** A total of 137 patients with incidental pancreatic calcifications were identified. A small subset had coarse calcifications (n=9, 7%), while the majority had both coarse and punctiform calcifications (n=102, 74%), with calcification numbers ranging from 7 to 50 in over half of the cases. In 121 patients (88%), at least two pancreatic segments were involved, most frequently the head (n=125, 91%) and the body (n=112, 81%). When coarse calcifications were present alongside duct dilation, the duct caliber was significantly larger (6.7 mm vs 4.1 mm; p=0.026), while the tail was slightly smaller (17 mm vs 19 mm, p=0.018), with no significant differences in the size of the head or body (p>0.198). Among patients with available clinical information (n=38), only 3 (11%) reported symptoms such as abdominal pain, diarrhea, or bloating, and none (0%) reported weight loss. Elevated alcohol consumption or smoking history was not associated with calcification type or location (p>0.186), pancreatic size, or duct dilation (p>0.317).
**Conclusion:** Incidentally detected pancreatic calcifications are not a reliable biomarker for screening for chronic pancreatitis.
**Limitations:** The main study limitation is its retrospective design.
**Funding for this study:** No fundings were received for this study
**Ethics committee - additional information:** Institutional Review Board approved the retrospective data collection in anonymous form
**Author Disclosures:**
Francesco Rizzetto: Author: nothing to disclose
Caterina Beatrice Monti: Author: nothing to disclose
Angelo Vanzulli: Author: nothing to disclose
Annamaria Pata: Author: nothing to disclose

**The diagnostic potential of unenhanced dual-layer spectral CT quantitative parameters in diabetic pancreas**

*L. Ge*, Y. Li, Y. Gao, X. Zhang, X. Yu; Xi An/CN
*(1051394676@qq.com)*

**Purpose or Learning Objective:** To investigate pancreas characterizing for type 2 diabetes mellitus (T2DM) using unenhanced dual-layer Spectral CT.
**Methods or Background:** This retrospective study included patients who underwent abdominal unenhanced dual-layer spectral CT between March 2023 and April 2024. The patients were divided into T2DM group and control group. Nine regions of interest (ROIs) were drawn (three for head, three for body and three for tail). Mean attenuation on conventional 120-kVp CT images (CTconv), effective atomic number maps (Z-eff), iodine density maps (ID), virtual non-contrasted (VNC), and mean attenuation on virtual monoenergetic images (VMIs) at 40-200keV were measured. The Mann -Whitney U test was used to compare the differences between the two groups. The receiver operating curve (ROC) was used to evaluate the diagnostic efficacy of the above parameters.
**Results or Findings:** A total of 84 patients, including 44 T2DM patients and 40 controls, were evaluated. There was a statistically significant difference in the CTconv (46.7 ± 5.9 HU vs. 50.7 ± 4.5 HU, p < 0.01), Z-eff (7.43 ± 0.05 vs. 7.46 ± 0.03, p < 0.01), ID (103.9 ± 0.6 vs. 104.3 ± 0.4, p < 0.01), VNC (39.2 ± 5.5 HU vs. 42.3 ± 4.2 HU, p < 0.01) and VMIs (62.1 ± 9.7 HU vs. 68.6 ± 6.2 HU at 40 keV, p < 0.001) between the T2DM group and the control group. The AUCs of the CTconv, Z-eff, ID, VNC and 40 keV were 0.722,0.671,0.695, 0.691 and 0.734. Histogram analysis found that the 10th percentile value of the above parameters had higher diagnostic efficiency (0.757,0.736,0.702,0.734 and 0.805). The AUC of VMIs increased with decreasing monoenergeic levels.
**Conclusion:** The 10th percentile value of 40 keV was the best indicator for distinguishing T2DM patients from the controls.
**Limitations:** Not applicable
**Funding for this study:** Not applicable
**Ethics committee - additional information:** Not applicable
**Author Disclosures:**
Xiao Yu: Nothing to disclose
Yishan Li: Nothing to disclose
Xiaoyue Zhang: Nothing to disclose
Yanjun Gao: Nothing to disclose
Liu Ge: Nothing to disclose

**Abdominal mapping with MOLLI and T2p-SSFP: can you do without the cardiac trigger?**

A. Lupi, P. Dardeshi, I. Toniolo, *G. Beggiato*, M. Pizzi, D. Negro, E. Quaia, A. Pepe; Padua/IT
*(giulia.beggiato.1@studenti.unipd.it)*

**Purpose or Learning Objective:** Quantitative abdominal imaging is increasingly emerging in the radiology arena, drawing inspiration from cardiac mapping, which is significantly changing patient management. In fact, cardiac mapping sequences offer the possibility of obtaining abdominal mapping values, although with long acquisition times, due to the cardiac trigger. The aim of our study is to evaluate the differences between abdominal mapping values obtained with and without cardiac trigger.
**Methods or Background:** Ten patients who were candidates for the abdominal mapping study as part of a research protocol were included. MOLLI and T2p-SSFP sequences were acquired for T1 and T2 mapping, respectively, with (T) and without (NT) cardiac trigger and with simulated trigger (ST, HR 60 bpm). T1/T2 mapping values were extracted using manually traced regions of interest (ROIs) at the hepatic, pancreatic and renal cortical levels, using the cvi42 software. The results obtained were compared using paired t-tests.
**Results or Findings:** Liver T1/T2 mapping showed statistically significant differences between NT and ST sequences (p=0.003 and 0.004, respectively), and T2 mapping values turned out to be significantly different between NT and T acquisition also (p<0.001). Pancreas T1mapping values were different between NT vs T (p=0.016) and ST (p=0.037), while T2mapping values were different between NT and ST only (p=0.029). No differences between T and ST sequences were found in liver and pancreas T1/T2 mapping (p>0.05). Renal T1/T2 mapping did not show significant differences among the three acquisition strategies.
**Conclusion:** Our results show that cardiac trigger should not be avoided in liver and pancreas mapping with MOLLI and T2p-SSFP sequences, but in order to reduce acquisition time, a simulator could be used. Further analysis on larger sample and with standard ROIs are needed to confirm these data.
**Limitations:** Sample size
**Funding for this study:** n/a
**Ethics committee - additional information:** Azienda Ospedale Università Padova
**Author Disclosures:**
Amalia Lupi: Nothing to disclose
Alessia Pepe: Nothing to disclose
Giulia Beggiato: Nothing to disclose
Marco Pizzi: Nothing to disclose
Pajtim Dardeshi: Nothing to disclose
Donato Negro: Nothing to disclose
Emilio Quaia: Nothing to disclose
Irene Toniolo: Nothing to disclose

**08:00-09:30** **Research Stage 2**

### Research Presentation Session: Cardiac

### RPS 103

### Applications of cardiac CT

**Moderator**
R. Vliegenthart; Groningen/NL
*(r.vliegenthart@umcg.nl)*
**Author Disclosures:**
Rozemarijn Vliegenthart: Advisory Board: Lifelines, ICAN (Institute for Cardiometabolism and Nutrition); Board Member: ESCR; Grant Recipient: Siemens Healthineers (institutional research grant); Speaker: Siemens Healthineers, Bayer Healthcare, Wiley

### Long-term exposure to particulate and gaseous air pollution and coronary atherosclerotic disease assessed by cardiac CT

*F. Castillo Aravena*[1], C. Desroche[2], S. Delaney[3], R. Nethery[3], P. Thavendiranathan[1], H. Ross[1], K. Hanneman[1]; [1]Toronto, ON/CA, [2]Kingston, ON/CA, [3]Boston, MA/US
*(felipe.castilloaravena@uhn.ca)*

**Purpose or Learning Objective:** Both fine particulate matter (PM2.5) and nitrogen dioxide (NO2) are associated with cardiovascular mortality. However, the underlying pathophysiological mechanisms are unclear. The purpose of this study was to evaluate the relationship between long-term exposure to these air pollutants and extent of coronary artery disease.
**Methods or Background:** Adult patients undergoing cardiac CT between 2012-2023 were retrospectively evaluated. Coronary atherosclerosis was quantified using Agatston coronary artery calcium scores (CACS). Long-term air pollution exposures were assessed as the average of daily direct measurements of PM2.5 and NO2 in the ten-year period prior to cardiac CT. Multivariable linear regression models were adjusted for sex, age, year, distance to monitoring station, and socioeconomic status (neighborhood median household income and employment rate).
**Results or Findings:** 11,140 patients were included (52% male, mean age 59±11 years). Median 10-year exposure to PM2.5 was 7.5 (range 4.3–9.2) µg/m3 and NO2 was 13.4 (range 3.2-17.8) parts per billion (ppb). Each 1 µg/m3 increase in ten-year PM2.5 exposure was associated with 23.2 higher CACS (β-coefficient 23.2, 95%CI, 5.3-41.0, P=0.011) in unadjusted analysis and 19.2 higher CACS (β-coefficient 19.2, 95%CI, 0.7-37.7, P=0.042) in multivariable analysis. Each 1 ppb increase in ten-year NO2 exposure was associated with 5.0 higher CACS (β-coefficient 5.0, 95%CI, 1.9-8.2, P=0.002) in unadjusted analysis; however, this association was attenuated in multivariable analysis (β-coefficient 1.4, 95%CI, -1.7-4.4, P=0.38).
**Conclusion:** Higher long-term exposure to fine particulate (PM2.5) air pollution is associated with higher extent of coronary atherosclerotic disease. The relationship with NO2 was not significant in adjusted analysis. These results highlight the potential for CT to detect the sequela of long-term air pollution.
**Limitations:** CACS does not quantify non-calcified plaque and further study is needed to evaluate relationships with total plaque burden.
**Funding for this study:** None
**Ethics committee - additional information:** University Health Network (CAPCR 24-5344)
**Author Disclosures:**
Paaladinesh Thavendiranathan: Nothing to disclose
Rachel Nethery: Nothing to disclose
Kate Hanneman: Nothing to disclose
Heather Ross: Nothing to disclose
Chloe Desroche: Nothing to disclose
Felipe Castillo Aravena: Nothing to disclose
Scott Delaney: Nothing to disclose

### Video Assisted Informed Consent in Cardiac Imaging: Influence on Patient Anxiety during CT – The VAICICI-trial

*R. Gohmann*, S. Mettke, C. F. Lücke, C. D. Krieghoff, M. Gutberlet; Leipzig/DE
*(robin.gohmann@helios-gesundheit.de)*

**Purpose or Learning Objective:** CT is a non-invasive tool for the diagnosis of coronary artery disease (CAD) and preoperative planning. However, cardiac CT (cCT) can elicit anxiety, potentially impacting patient compliance and ultimately image quality. This study investigates whether video-assisted informed consent in cardiac imaging (VAICICI) reduces patient anxiety during cCT and enhances patient understanding of the examination process.
**Methods or Background:** This prospective, randomized, controlled trial enrolled 205 patients scheduled for cCT. Patients were randomized into three groups: Control (n=69), Video I (n=67), and Video II (n=69). Video I was an educational video with visuals, subtitles, and voiceover explaining the examination. Video II presented only voiceover and subtitles without visuals. All patients received a standard physician consultation. Anxiety and patient satisfaction were measured using visual analog scales immediately before and after the examination. Statistical analyses included group-comparisons and multivariate-analysis to examine the influence of demographic and anamnestic variables.
**Results or Findings:** Both Video I and II significantly improved patient understanding, and satisfaction compared to the control group (p<0.05). Patients with Video II reported the informed consent form as more important for understanding than those in the control group (p=0.023). Satisfaction was higher after watching any video (p=0.020) with significant difference between Video I and II. Anxiety levels did not differ between the groups, though female patients (p=0.008) and those having suspected CAD reported higher pre-examination-anxiety. Overall, 10 baseline demographics were found to be partially explanatory to the response and independently statistically significant, e.g. age and previous CT/MRI-experience.
**Conclusion:** VAICICI improved patient satisfaction and understanding of the cCT examination. However, its impact on reported anxiety reduction was limited. The findings suggest that VAICICI enhances the informed consent process. Its influence on compliance and thus image quality remains to be investigated.
**Limitations:** The Single-center Design And The Relatively High Familiarity With Imaging Among The Study Cohort May Limit The Generalizability Of The Results.
**Funding for this study:** None.
**Ethics committee - additional information:** This Study Was Approved By The Local Ethics Committee (Reference-No.:172/22-ek). Written Informed Consent Was Waived.
**Author Disclosures:**
Robin Gohmann: Nothing to disclose
Christian Friedrich Lücke: Nothing to disclose
Matthias Gutberlet: Nothing to disclose
Christian Dominik Krieghoff: Nothing to disclose
Sophia Mettke: Nothing to disclose

### Impact of Cardiac Computed Tomography Angiography to prevent major adverse cardiovascular events in patients undergoing diagnostic work-up for orthotopic liver transplant

*F. Santoro*, B. La Delfa, D. Tore, C. Guarnaccia, R. Faletti, G. A. Strazzarino, C. Gaetani, A. Depaoli, P. Fonio; Turin/IT
*(federicasantoro1996@gmail.com)*

**Purpose or Learning Objective:** To evaluate the impact of Cardiac CT Angiography (CCTA) in cardiovascular risk assessment to prevent major adverse cardiovascular events (MACE) in patients undergoing diagnostic work-up before orthotopic liver transplant (OLT).

Wednesday

**Methods or Background:** Monocentric retrospective study on 140 patients with intermediate to high risk of CAD who underwent CCTA during pre-OLT diagnostic work-up at our Institution from March 2021 to October 2024. All exams were performed using prospective ECG-gated single heartbeat axial acquisition (0.28 s gantry rotation time, kV and mA set depending on patient BMI, ECG window 40-80% of R-R cycle) with a whole-heart coverage CT scanner (Revolution CT, GE, USA). 95 patients were classified with non-critical CAD at CCTA. 23 patients with suspicion of critical CAD at CCTA underwent invasive coronary angiography (ICA). Sensitivity, specificity, positive predicting value (PPV) and negative predictive value (NPV) were calculated.
**Results or Findings:** In the 95 patients with negative CCTA no one developed MACEs or cardiovascular related complications while on waiting list, nor during or after OLT; 23 patients with critical disease (CAD-RADS 4-5) at CCTA subsequently underwent ICA. In 14 cases stenosis was confirmed at ICA and patients underwent percutaneous coronary intervention (PCI) with Drug Eluting Stent (DES). CCTA in this group of patients had 100% sensitivity, 91.3% specificity, 100% negative predicting value and 60.9% positive predicting value.
**Conclusion:** CCTA has an excellent diagnostic accuracy for cardiovascular risk stratification in the setting of pre-OLT work-up and it may have a role in preventing MACEs or other cardiovascular events in asymptomatic patients with intermediate to high risk of CAD.
**Limitations:** Monocentric study, small simple size.
**Funding for this study:** Nothing to discloure
**Ethics committee - additional information:** Not applicable
**Author Disclosures:**
Giulio Antonino Strazzarino: Nothing to disclose
Alessandro Depaoli: Nothing to disclose
Riccardo Faletti: Nothing to disclose
Carla Guarnaccia: Nothing to disclose
Benedetta La Delfa: Nothing to disclose
Federica Santoro: Nothing to disclose
Davide Tore: Nothing to disclose
Paolo Fonio: Nothing to disclose
Clara Gaetani: Nothing to disclose

### Matters of the heart in stroke - acute phase cardiac CT in intracranial large vessel occlusion stroke for the identification of high-risk imaging biomarkers indicative of a cardioembolic cause

*K. Mostafa*, C. Wolf, C. Wünsche, S. Krutmann, M. Both, O. Jansen, H. Seoudy, P. Langguth; Kiel/DE
*(mostafa.karim86@gmail.com)*

**Purpose or Learning Objective:** The purpose of this study is the exploration of imaging biomarkers on acute phase cardiac CT that may suggest a cardioembolic etiology in patients with large vessel occlusion (LVO) stroke in patients without intracardial thrombi or atrial fibrillation in an effort to further specify and weigh the known major and minor cardioembolic risk factors.
**Methods or Background:** A total of 317 patients with LVO stroke and acute-setting one-step cardiac CT imaging examination were retrospectively identified and included in this study. Images were assessed for a total of 15 specific imaging findings according to known minor and major cardioembolic risk factors. Final etiology (TOAST) of LVO stroke was determined by interdisciplinary consensus after full clinical workup. Multivariate regression analysis was performed to identify cardiac imaging findings associated with a cardioembolic etiology.
**Results or Findings:** Overall, 221 (70%) of the LVO strokes were found to have a cardioembolic etiology by interdisciplinary consensus. After correction for atrial fibrillation and intracardiac thrombi, multivariate regression analysis defined dilatative cardiomyopathy (adjusted odds-ratio (AOR) 37.9), right-to-left shunt (AOR 21.8), valvular implants (AOR 23.7), type II and III thrombotic aortic arch (AOR 8.1) and visible myocardial scars (AOR 6.8) as risk factors for a cardioembolic etiology (AUC 0.87, $p < 0.05$ for all factors respectively).
**Conclusion:** In patients with LVO stroke without atrial fibrillation or intracardiac thrombi on acute phase cardiac CT imaging, the presence of dilatative cardiomyopathy, right-to-left shunt, valvular implants, type II and II thrombotic aortic arch and visible myocardial scars on acute phase CT imaging findings is significantly associated with a cardioembolic stroke etiology.
**Limitations:** This is a retrospective study with its associated limitations. The number of patients was low due to the single centre design.
**Funding for this study:** None.
**Ethics committee - additional information:** Ethikkommission der Christian Albrechts Universität Kiel
**Author Disclosures:**
Cosima Wünsche: Nothing to disclose
Carmen Wolf: Nothing to disclose
Patrick Langguth: Nothing to disclose
Marcus Both: Nothing to disclose
Karim Mostafa: Nothing to disclose
Sarah Krutmann: Nothing to disclose
Olav Jansen: Nothing to disclose
Hatim Seoudy: Nothing to disclose

### The pericoronary adipose tissue attenuation in CT strongly depends on kernels and iterative reconstructions

C. Lisi[1], *K. Klambauer*[2], L. J. Moser[2], V. Mergen[2], R. Manka[2], T. Flohr[2], M. Eberhard[2], H. Alkadhi[2]; [1]Milan/IT, [2]Zürich/CH

**Purpose or Learning Objective:** To investigate the influence of kernels and iterative reconstructions on pericoronary adipose tissue (PCAT) attenuation in coronary CT angiography (CCTA)
**Methods or Background:** Twenty subjects (16 females; median age 52 years (IQR 48-61)) with atypical chest pain and low risk of coronary artery disease (CAD) who were otherwise healthy and without evidence of CAD in photon-counting detector CCTA were included. In each subject images were reconstructed with a quantitative smooth (Qr36) and three vascular kernels of increasing sharpness levels (Bv36, Bv44, Bv56). Quantum iterative reconstruction (QIR) was either switched-off (QIR off) or was used with strengths 2 and 4. The fat-attenuation-index (FAI) of the PCAT surrounding the right coronary artery was calculated in each dataset. Histograms of FAI measurements were created. Intra- and inter-reader agreement were determined. A CT edge-phantom was used to determine the edge-spread-function (ESF) for the same datasets
**Results or Findings:** Intra- and inter-reader agreement of FAI were excellent (ICCs=0.99 and 0.98, respectively). Significant differences in FAI were observed depending on the kernel and iterative reconstruction strength level (each, $p < 0.001$), with inter-individual variation up to 34HU. FAI showed also considerable intra-individual variation (average FAI difference 19HU, maximal intra-individual difference 33HU), also depending on kernels and iterative reconstruction levels. The ESFs showed a reduced range of edge-smoothing with increasing kernel sharpness, causing FAI decrease. Histogram analyses revealed a narrower peak of PCAT values with increasing iterative reconstruction levels, causing FAI increase
**Conclusion:** PCAT attenuation determined with CCTA heavily depends on kernels and iterative reconstruction levels both within and across subjects. Standardization of CT reconstruction parameters is mandatory for FAI studies to enable meaningful interpretations
**Limitations:** Single centre study, including only healthy patients. Single vendor scanner and software analysis,
**Funding for this study:** No funding was received for this study
**Ethics committee - additional information:** No additional information needed
**Author Disclosures:**
Thomas Flohr: Nothing to disclose
Costanza Lisi: Nothing to disclose
Victor Mergen: Nothing to disclose
Matthias Eberhard: Nothing to disclose
Lukas Jakob Moser: Nothing to disclose
Robert Manka: Nothing to disclose
Konstantin Klambauer: Nothing to disclose
Hatem Alkadhi: Nothing to disclose

### Inter-Observer Agreement of the Coronary Artery Disease-Reporting and Data System (CAD-RADS) 2.0

*J. H. Lund*, J. Erley, G. Adam, E. Tahir, I. Molwitz, M. Meyer; Hamburg/DE
*(lundjonas@gmx.de)*

**Purpose or Learning Objective:** In 2022, the Coronary Artery Disease-Reporting and Data System (CAD- RADSTM) was updated to standardize coronary CT angiography (CCTA) reports, requiring high inter-observer reproducibility. This study aims to assess the inter-observer agreement of CAD-RADS 2.0.
**Methods or Background:** Patients who underwent CCTA between 2022 and 2024 using a 3rd-generation-dual-source-CT were independently evaluated by three readers with varying levels of experience (1, 3, and 12 years). CAD-RADS 2.0 was used to assess visual grading of plaque burden (P1 = mild to P4 = extensive), stenosis degree (CAD-RADS 0 = no stenosis to CAD-RADS 5 = total occlusion), and modifiers (HRP for high-risk plaque features, E for exceptions, S for stents, G for grafts, and N for non-evaluable studies). Inter-observer agreement was measured using intraclass correlation coefficients (ICC).

Wednesday

**Results or Findings:** 100 patients (29% female, age 63 ± 12 years) with a median Agatston score of 267 were included. Observers 1, 2, and 3 rated 71%, 60%, and 64% of patients, respectively, as CAD-RADS 3 or above. Inter-observer agreement for plaque burden grading (ICC:0.92, 95% CI:0.88–0.94, p < 0.001) and stenosis degree (ICC:0.88, 95% CI:0.83–0.92, p < 0.001) on a per-patient level was excellent. The agreement was also excellent on a per-vessel basis, with the highest for the left anterior descending artery (ICC:0.90) and the lowest for the left main artery (ICC:0.86). Agreement on modifiers was poor (ICC:0.06, p = 0.410).
**Conclusion:** CAD-RADS 2.0 demonstrates excellent inter-observer agreement for plaque burden and stenosis grading, but agreement on modifier use is low, likely due to limited use or uncertainty in their application in routine practice.
**Limitations:** Limited assessment of the modifiers. Experienced based-bias due to limited amount of observer, thus the real-world clinical experience might not be entirely reflected.
**Funding for this study:** Nothing to disclose.
**Ethics committee - additional information:** This study was accepted by the ethics committee of the University Medial Center Hamburg-Eppendorf (UKE).
**Author Disclosures:**
Gerhard Adam: Nothing to disclose
Isabel Molwitz: Nothing to disclose
Mathias Meyer: Nothing to disclose
Jennifer Erley: Nothing to disclose
Enver Tahir: Nothing to disclose
Jonas H. Lund: Nothing to disclose

**Preliminary Experience of 60-kVp Tube Voltage Combined with Deep Learning Reconstruction Algorithm in Coronary CT Angiography**
X. Wu[1], S. Jiang[2], Y. Zou[2], *T. Wang*[2], G. Zhang[2], F. Huang[1], P. Liu[1], W. Sun[1], W. He[1]; [1]Changsha/CN, [2]Shanghai/CN
*(tiantian.wang@cri-united-imaging.com)*

**Purpose or Learning Objective:** To explore the clinical value of 60-kVp coronary CT angiography (CCTA) combined with DEep Learning Trained Algorithm (DELTA).
**Methods or Background:** Thirty-nine patients (20 male, 58.77 ± 16.26 years, 22.87 ± 3.71 kg/m^2) with suspected coronary artery disease (CAD) were prospectively enrolled. Each underwent both low- (60 kVp, 28 ml contrast medium at 2.5 ml/s) and routine-dose CCTA (100 kVp, 44 ml contrast medium at 4.0 ml/s) on a 320-row scanner within 2 weeks. The routine-dose data were reconstructed using hybrid iterative reconstruction (RD-HIR) and served as the reference standard. Low-dose data were reconstructed using both HIR (LD-HIR) and DELTA (LD-DELTA). Coronary stenosis in the right coronary artery (RCA), left anterior descending (LAD), and left circumflex (LCX) was assessed using CAD-Reporting and Data System (CAD-RADS) scores. The diagnostic performance of LD-HIR and LD-DELTA in distinguishing moderate (CAD-RADS<3) to severe (CAD-RADS≥3) stenosis was analyzed via receiver operating characteristic analysis. Signal-noise-ratio (SNR) and contrast-noise-ratio (CNR) on each vessel were also compared.
**Results or Findings:** The low-dose CCTA reduced radiation dose by 85.8% compared to the routine-dose acquisition (0.55 ± 0.09 mSv vs. 3.86 ± 1.25 mSv, p<0.001). In distinguishing moderate to severe stenosis, LD-DELTA demonstrated superior diagnostic performance compared to LD-HIR, with area under the curve (AUC) being 1.00 (95% CI, 0.91-1.00) versus 0.87 (95% CI, 0.72-0.96) in the RCA, and 1.00 (95% CI, 0.91-1.00) versus 0.78 (95% CI, 0.62-0.90) in the LCX. However, no difference was found in the LAD, with both showing an AUC of 0.98 (95% CI, 0.88-1.00). Additionally, LD-DELTA demonstrated higher SNRs and CNRs compared to LD-HIR (all p<0.001).
**Conclusion:** The 60-kVp low-dose CCTA acquisition with DELTA significantly reduces radiation dose while maintaining diagnostic performance for assessing coronary stenosis.
**Limitations:** Not applicable.
**Funding for this study:** No funding was received for this study.
**Ethics committee - additional information:** This study was approved by the local institutional review board.
**Author Disclosures:**
Peng Liu: Nothing to disclose
Tiantian Wang: Investigator: at Central Research Institute, United Imaging Healthcare
Yixuan Zou: Investigator: Central Research Institute, United Imaging Healthcare
Feng Huang: Nothing to disclose
Guozhi Zhang: Investigator: Central Research Institute, United Imaging Healthcare
Weiling He: Nothing to disclose
Wenjie Sun: Nothing to disclose
Senyang Jiang: Investigator: Central Research Institute, United Imaging Healthcare
Xi Wu: Nothing to disclose

**Quantitative assessment of early changes in myocardial extracellular volume during postoperative adjuvant chemotherapy in breast cancer patients by dual-layer spectral detector CT**
*H. Wu*, Y. Huang, W. Deng, Y. Wang, Q. Xiao, Y. Gu; Shanghai/CN
*(wuhonglinsimple@yeah.net)*

**Purpose or Learning Objective:** This study utilized dual-layer spectral detector CT (DLCT) technology to simultaneously assess cardiac function and myocardial myocardial extracellular volume (ECV) , characterizing the changes in parameters early during breast cancer therapy.
**Methods or Background:** Forty female breast cancer patients who underwent postoperative adjuvant chemotherapy were prospectively enrolled and underwent baseline and 3-month-postchemotherapy cardiac CT (CCT) and ultrasound cardiography (UCG). Global ECV of the left ventricle (LV) were measured based on an iodine map of the late enhancement phase of DLCT. Changes in cardiac function parameters and global ECV from baseline to the 3-month follow-up were analyzed. Correlation coefficients between the changes in cardiac function parameters and global ECV were calculated.
**Results or Findings:** LV ejection fraction by UCG (UCG-LVEF) and by CCT (CCT-LVEF) did not significantly change between baseline and 3 months. Heart rate (HR) increased over 3 months of follow-up. After normalization to body surface area (BSA), cardiac output (CCT-CO indexed) and LV late (active) filling volume (LVLFV indexed) significantly increased (P<0.01), while LV early (passive) filling volume (LVEFV indexed) and LVEFV/LVLFV decreased significantly at the 3-month follow-up (P<0.05). Global ECV were elevated significantly at 3 months (25.4±2.4 vs. 27.3±2.7, P<0.01). Although changes in global ECV were not associated with changes in LVEFs, global ECV change were moderately correlated with changes in LV end-diastolic volume / BSA (CCT-LVEDV indexed) (r=0.52, P<0.01), LV stroke volume / BSA (CCT-LVSV indexed) (r=0.56, P<0.01), CCT-CO indexed (r=0.40, P=0.01) and LVEFV indexed (r=0.41, P<0.01) .
**Conclusion:** CCT-derived ECV can be used to evaluate myocardial changes in the early stage of chemotherapy before LVEF significantly decreases. The increases in global ECV were not correlated with LVEFs. The changes in myocardial global ECV were moderately correlated with cardiac function parameters.
**Limitations:** The small sample.
**Funding for this study:** None
**Ethics committee - additional information:** The study was approved by the Ethics Committee of Fudan University Shanghai Cancer Center, and written informed consent was obtained from all subjects in the study.
**Author Disclosures:**
Honglin Wu: Nothing to disclose
Yan Huang: Nothing to disclose
Yajia Gu: Nothing to disclose
Yu Wang: Nothing to disclose
Qin Xiao: Nothing to disclose
Weiwei Deng: Nothing to disclose

**Cardiac calcifications detected on planning CT are major predictors of long-term cardiotoxicity after radiotherapy for breast cancer**
K. B. Dimayuga, *A. Belardo*, L. Perna, A. Fodor, P. Mangili, A. Del Vecchio, N. Di Muzio, C. Fiorino; Milan/IT
*(belardo.alfonso@hsr.it)*

**Purpose or Learning Objective:** Breast cancer (BC) patients undergoing radiotherapy (RT) may experience long-term cardiotoxicity. In modern series, delivering low dose to the heart, non-dosimetry predictors are emerging. The purpose was to test if cardiac calcifications (CAC) at planning CT, suggested as potential predictors, are associated with long-term cardiac events.
**Methods or Background:** Planning CT and clinical information of 1172 consecutive patients treated at our hospital (2009-2017) were available (right:569, left:603). The heart of all patients was automatically segmented using a previously validated AI-based tool (MIM Protegé & MIM assistant) and the mean heart dose (MHD) was assessed. CAC were automatically extracted by applying a home-made, validated, Python script extracting the Agatson score (AS) and the CAC overall volume. Their association with the risk of cardiac events was tested by logistic regression, including the potential combined effect of MHD and available clinical parameters.
**Results or Findings:** With a median follow-up of 8 years (range: 5-15), 32 patients experienced cardiac events. AS/CAC volumes were the most significant predictors (p<0.0001), with similar performances. Age, laterality (left/right), concomitant chemotherapy, obesity and hypertension were also significant at univariate analysis. MHD encoded using the best cut-off (1Gy, mostly representing laterality) was also predictive. The best multivariate model combined MHD>1Gy, age and CAC volume (AUC=0.79, p<0.0001, calibration plot: m=1.506, q=-0.007), being CAC volume the strongest predictor (OR: 1.0008/mm3, p<0.0001).
**Conclusion:** CAC load was the most important factor in cardiac risk stratification after BC RT in a modern series.

**Limitations:** Events are not recovered from a registry. Then, the risk of missing events is not negligible.
**Funding for this study:** None
**Ethics committee - additional information:** All respected.
**Author Disclosures:**
Lucia Perna: Nothing to disclose
Nadia Di Muzio: Nothing to disclose
Claudio Fiorino: Nothing to disclose
Alfonso Belardo: Nothing to disclose
Antonella Del Vecchio: Nothing to disclose
Paola Mangili: Nothing to disclose
Kerby Bjorn Dimayuga: Nothing to disclose
Andrei Fodor: Nothing to disclose

**One-Scan Acquisition of Coronary CT Angiography and CT Aortography Using Photon Counting Detector CT**
*H. Kato*, S. Araki, S. Nakamura, A. Yamazaki, N. Kato, Y. Ichikawa, H. Sakuma, K. Kitagawa; Tsu/JP

**Purpose or Learning Objective:** For patients with aortic diseases, Coronary CT angiography (CCTA) is performed for preoperative evaluation of coronary artery disease (CAD), often in combination with CT aortography (CTAO) within the same examination. However, the shared imaging field in both CCTA and CTAO results in overlapping radiation exposure to the thoracic region. Recently, a dual-source photon-counting detector CT (PCD-CT) has emerged, with its capability to perform high-pitch helical scanning to enable one-scan acquisition of CCTA and CTAO. This study aimed to compare the radiation dose and image quality of one-scan CCTA and CTAO imaging with those of separate CCTA and CTAO imaging.
**Methods or Background:** This study included 40 patients who underwent CCTA and CTAO for preoperative CAD screening for aortic disease: 22 had separate, 18 had one-scan CCTA and CTAO. A low tube potential of 70 or 90 kVp was used for all patients. CT dose-length product (DLP) data for CCTA and CTAO was collected. Image quality for each coronary segment and the aorta was assessed using a four-point scale (excellent, good, fair, non-diagnostic).
**Results or Findings:** The mean DLP was significantly lower with the one-scan CCTA and CTAO protocol (160.5±36.5 mGy*cm) compared to the separate scans (716.0±203.9 mGy*cm) (P<0.001). There was no significant difference between the separate and one-scan protocols in the assessment of image quality for CCTA (P=0.17) and CTAO (P=0.92), and in both protocols, over 95% segments/cases in the CCTA and CCAO images were rated as having good or excellent image quality.
**Conclusion:** PCD-CT, with high-pitch helical scanning and low tube voltage, enabled one-scan acquisition of CCTA and CTAO, significantly reducing radiation exposure while maintaining high image quality.
**Limitations:** There is no reference standard such as coronary angiography.
**Funding for this study:** No funding was provised for this study.
**Ethics committee - additional information:** Clinical Research Ethics Review Committee of Mie University Hospital (approval No. H2019-207)
**Author Disclosures:**
Kakuya Kitagawa: Nothing to disclose
Hajime Sakuma: Nothing to disclose
Suguru Araki: Nothing to disclose
Yasutaka Ichikawa: Nothing to disclose
Noriyuki Kato: Nothing to disclose
Hiroaki Kato: Nothing to disclose
Akio Yamazaki: Nothing to disclose
Satoshi Nakamura: Nothing to disclose

**08:00-09:30** **Research Stage 3**

### Research Presentation Session: Oncologic Imaging

### RPS 116

### Staging, metastases and response assessment

**Moderator**
R. Perez-Lopez; Barcelona/ES

**Undifferentiated pleomorphic sarcoma: Building an effective multi-parametric MRI (mpMRI) predictive treatment response model to replace RECIST**
*R. F. Valenzuela*, B. Amini, E. Duran-Sierra, J. E. Madewell, M. Antony, C. M. Costelloe, W. Murphy; Houston, TX/US
*(raulvp@estigia.cl)*

**Purpose or Learning Objective:** Undifferentiated pleomorphic sarcoma (UPS) is the largest soft-tissue sarcoma subgroup. Post-therapeutically, UPS demonstrates hemosiderin deposition, fibrosis, and calcification. This study aimed to establish the clinical value of multiparametric MRI (mpMRI) for predicting UPS response.
**Methods or Background:** An IRB-approved retrospective study included 33 extremity UPS patients with pre-operative mpMRI, including diffusion-weighted imaging (DWI), contrast-enhanced susceptibility-weighted imaging (CE-SWI), and perfusion-weighted imaging with dynamic contrast-enhancement (PWI/DCE), and surgical resection February 2021-May 2023. Lesions were visually classified on CE-SWI into one of 6 morphology patterns. On PWI/DCE, lesions were classified into one of 6 patterns, and time-intensity curves (TICs) were classified as types I-V. Patients were divided into three groups based on the percentage of pathology-assessed treatment effect (PATE) in the surgical specimen: Responders (>=90% PATE, n=16), partial-responders (31-89% PATE, n=10), and non-responders (<=30% PATE, n=7). Receiver operating characteristic (ROC) analysis of classification models based on CE-SWI and PWI/DCE patterns and TICs compared responders vs. partial/non-responders.
**Results or Findings:** At post-radiation therapy (PRT), a CE-SWI Complete Ring pattern was observed in 71% of responders (p=7.71x10-6). On PWI/DCE images, 79% of responders displayed a Capsular pattern (p=1.49x10-7), and 100% demonstrated a TIC-type II (p=8.32x10-7). RECIST could not separate responders from partial/non-responders; all demonstrated 100% stability at PRT and pseudoprogression at PC. ROC analysis comparing responders (n=14) vs. partial/non-responders (n=16) at PRT showed that the model combining the PWI/DCE TIC-type II, PWI/DCE Capsular pattern and CE-SWI Complete Ring pattern yielded the highest classification performance (AUC=0.99).
**Conclusion:** mpMRI-derived features can help assess UPS treatment response. Observing a pre-operative PWI/DCE TIC-type II, PWI/DCE Capsular pattern, and CE-SWI Complete Ring pattern can potentially predict successfully treated UPS patients with >=90% PATE, outperforming RECIST.
**Limitations:** Limitations include a small sample (n=33) and manual, time-consuming tumor VOI segmentation.
**Funding for this study:** The John S. Dunn, Sr. Distinguished Chair in Diagnostic Imaging.
M.R Evelyn Hudson Foundation Endowed Professorship.
**Ethics committee - additional information:** Not applicable
**Author Disclosures:**
Elvis Duran-Sierra: Nothing to disclose
Behrang Amini: Nothing to disclose
Colleen M Costelloe: Nothing to disclose
Raul Fernando Valenzuela: Nothing to disclose
William Murphy: Nothing to disclose
Mathew Antony: Nothing to disclose
John Edward Madewell: Nothing to disclose

**Redefining radiologic responses in high-risk soft-tissue sarcomas treated with neoadjuvant chemotherapy. Final results of ISG-STS 1001, a randomized clinical trial**
*A. Vanzulli*, R. Vigorito, C. Buonomenna, P. Verderio, S. Pasquali, P. G. Casali, C. Morosi, S. Stacchiotti, A. Gronchi; Milan/IT
*(andrea.vanzulli@unimi.it)*

**Purpose or Learning Objective:** We report the results of the pre-planned secondary analysis of radiologic responses (RR) of ISG-STS 1001, a randomized clinical trial comparing anthracycline + ifosfamide (AI) vs. histology-tailored (HT) neoadjuvant chemotherapy for primary localized high-risk soft-tissue sarcomas of the extremities/trunk wall.
**Methods or Background:** Patients with undifferentiated pleomorphic sarcoma (UPS), leiomyosarcoma (LMS), malignant peripheral nerve sheath tumor, synovial sarcoma or myxoid liposarcoma (MLPS) were randomized, whereas patients with myxofibrosarcoma, pleomorphic liposarcoma, pleomorphic rhabdomyosarcoma or unclassified sarcoma were allocated in the observational arm (O) and treated with AI. Patients with UPS, LMS or MLPS needing concurrent preoperative radiotherapy were included in O. We evaluated associations between: Disease-Free Survival (DFS)/Overall Survival (OS) and centrally reviewed RR, assessed with RECIST 1.1 and as percent dimensional variation (D; both dichotomized and continuous); DFS/OS and histology; RR and histology.
**Results or Findings:** 435 patients were included (287 randomized, 148 observed). The analysis of RR comprised 236 patients (154 randomized, 82 observed) with measurable disease and available for central review. RECIST best responses were: 28 (11.9%) partial response (PR), 195 (82.6%) stable disease (SD) and 13 (5.5%) progressive disease (PD). RECIST significantly correlated with DFS (PD-vs-PR: HR 8.18, 95% CI 2.96-22.58; SD-vs-PR: HR 2.96, 95% CI 1.30-6.75) and OS (PD-vs-PR: HR 12.61, 95% CI 3.40-46.84; SD-vs-PR: HR 4.24, 95% CI 1.34-13.47). The median value of D was -1.6%. Patients with D > -1.6% had worse clinical outcomes than those with D < -1.6% (DFS: HR 1.73, 95% CI 1.19-2.50; OS: HR 1.86, 95% CI 1.21-2.86). D in continuous scale inversely correlated with DFS (HR 1.53, 95% CI 1.25-1.87) and OS (HR 1.78, 95% CI 1.41-2.25).
**Conclusion:** Dimensional variation in continuous scale predicted the proportional efficacy of treatment irrespective of tumor histology.
**Limitations:** Nothing to disclose.
**Funding for this study:** Pharmamar® provided trabectedin for the HG-MLPS cohort. The study was partially funded through a European Union grant (EUROSARC FP7 278472). In addition, the French sites were supported by NETSARC, LYRICAN (LYRICAN [INCA-DGOS-INSERM 12563]) and DEPGYN (RHU4).
**Ethics committee - additional information:** The trial protocol and all amendments were approved by the independent ethics committee at each trial center.
**Author Disclosures:**
Sandro Pasquali: Nothing to disclose
Paolo Giovanni Casali: Nothing to disclose
Paolo Verderio: Nothing to disclose
Alessandro Gronchi: Nothing to disclose
Andrea Vanzulli: Nothing to disclose
Carlo Morosi: Nothing to disclose
Ciriaco Buonomenna: Nothing to disclose
Raffaella Vigorito: Nothing to disclose
Silvia Stacchiotti: Nothing to disclose

**Can a fast T2-Dixon sequence surpass the time obstacle of whole-body MRI in the evaluation of skeletal metastases?**
N. Magdi, *M. Elmansy*, M. Elhawary, A. Sultan; Mansoura/EG

**Purpose or Learning Objective:** Our study was conducted to elucidate the role of the T2-Dixon sequence as a rapid alternative to the standard Whole-body magnetic resonance imaging (WB-MRI) protocol with the assessment of its diagnostic accuracy and comparability to the established methodology.
**Methods or Background:** This prospective study included 30 patients with primary solid malignancies who underwent WB-MRI. The sequences obtained were T1WI, STIR, and T2-Dixon (fat-only and water-only images). Skeletal metastases were evaluated in each sequence. Results were compared between the T1-STIR combination and T2-Dixon fat and water reconstructions.
**Results or Findings:** The sensitivity of fat and water reconstructions from a single T2-Dixon in the detection of lytic skeletal metastases was marginally superior to a combination of T1WI and STIR sequences (0-7%). Detection of mixed lesions demonstrated equally high sensitivity in both protocols. Sclerotic metastases detection in WB-MRI showed low sensitivity in both protocols. However, specificity surpassed 95% for all lesion types in both protocols. Overall image quality was favored (in 87-90% of patients ) in T2-Dixon images. The overall estimated acquisition timing using T2-Dixon appeared to be approximately half that of the standard T1-STIR combination.
**Conclusion:** WB-MRI using T2-Dixon fat and water reconstructions showed similar accuracy to T1WI and STIR combination in the evaluation of skeletal metastases in patients with primary solid cancers with significantly shorter acquisition time.
**Limitations:** Few skull and humeri lesions with limited assessment of the sclerotic lesions due to high fals negative results.
Lack of quantitative analysis of signal to noise and contrast to noise ratio.
**Funding for this study:** No funding
**Ethics committee - additional information:** This study was approved by the Research Ethics Committee of the Faculty of Medicine at Mansoura University in Egypt on 10 /1 /2023; reference number of approval: MS.22.12.2250
**Author Disclosures:**
Amina Sultan: Nothing to disclose
Noha Magdi: Nothing to disclose
Mohammed Elhawary: Nothing to disclose
Mostafa Elmansy: Nothing to disclose

**Impact of reader experience on reader agreement for whole-body MRI staging of oesophageal cancer**
*P. Chapellier*[1], S. W. Soo[2], O. Westerland[2], A. Green[2], S. Gourtsoyianni[3], V. Goh[2]; [1]Lausanne/CH, [2]London/UK, [3]Athens/GR

**Purpose or Learning Objective:** Whole-body MRI (WB-MRI) may be valuable alternative to standard imaging pathways for staging. We evaluated how reader experience impacts agreement for tumour-node-metastasis (TNM) staging of oesophageal cancer.
**Methods or Background:** Following ethical approval, prospective patients underwent WB-MRI (T2-weighted, diffusion-weighted, T1-weighted post-contrast) alongside standard imaging (contrast-enhanced CT, 18F-FDG PET/CT ± EUS). WB-MRI was staged using AJCC TNMv8 by four readers from different countries with different levels of experience: subspecialty vs. non-subspecialty trained; in-training vs. staff radiologists. Inter-reader agreement was assessed using kappa statistics. For each reader, agreement with a reference standard of final tumour board stage, surgical pathology and clinical follow up was obtained.
**Results or Findings:** 29/30 (97%) patients had adenocarcinoma; 25/30 (83%) had ≥T3 stage; 27/30 (90%) had locoregional lymphadenopathy; 12/30 (40%) were metastatic. 22/30 (74%) received chemotherapy only; 8/30 (27%) had surgery, 63% with neoadjuvant treatment. Compared to reference standard, agreement for T- and N-stage was highest for the two gastrointestinal-trained radiologists (T-stage: $\kappa$ =0.516, $\kappa$ =0.824; N-stage: $\kappa$ =0.434, $\kappa$ =0.589, respectively). Agreement for M-stage was highest for the oncology-trained radiologist ($\kappa$ =0.795). Detection of lung metastases was limited on MRI. Inter-reader agreement was also highest for gastrointestinal-trained radiologists (T-stage: $\kappa$ = 0.624; N-stage: $\kappa$ = 0.822). Agreement across TNM staging was lowest with the in-training radiologist who had no exposure to WB-MRI staging.
**Conclusion:** WB-MRI could be valuable alternative for initial TNM staging of oesophageal cancer, but reliable interpretation appears to be related to subspecialty experience and level of training.
**Limitations:** WB-MRI was a relatively long acquisition, and not suit all patients. Nodal disease was not just based on size measurement. MRI is limited for some sites eg.lung
**Funding for this study:** This project was supported by the National Institute for Health and Care Research (MIHR) Biomedical Research Centre at Guy's & St Thomas' Hospitals and King's College London.
**Ethics committee - additional information:** The study was approved by the Research Ethics Committee (IRAS ID 107508, 12/LO/1754).
**Author Disclosures:**
Olwen Westerland: Nothing to disclose
Adrian Green: Nothing to disclose
Sofia Gourtsoyianni: Nothing to disclose
Pauline Chapellier: Nothing to disclose
Vicky Goh: Nothing to disclose
Suet Woon Soo: Nothing to disclose

**Observer variability and reproducibility of bone marrow metastasis biomarkers on MRI**
*C. Sattin*[1], C. Pizzi[1], M. Kosmin[2], W. McGuire[3], A. Makris[2], N. J. Taylor[2], G. Petralia[1], A. R. R. Padhani[3]; [1]Milan/IT, [2]London/UK, [3]Northwood/UK
*(caterina.sattin@unimi.it)*

**Purpose or Learning Objective:** To document inter- and intra-observer variability and test-retest reproducibility of quantitative MRI metastasis biomarkers.
**Methods or Background:** Whole-body MRI was performed in women with bone-predominant metastatic breast cancer on a 1.5T MRI system. Paired reproducibility scans were done in 14 patients after repositioning. Observer variability was assessed in 10 patients after a two-week washout period. Two trained radiologists delineated up to 5 bone metastases per patient. Quantitative biomarkers (lesion sizes, apparent diffusion coefficient (ADC), relative fat fraction (rFF%) and b-900 s/mm2 signal-to-muscle ratio [SMR]) for each lesion were averaged per patient. Data logn transformed after testing for

Wednesday normality. Within-patient coefficient of variation (wCV) and variance ratio were calculated. The repeatability (R-value; mean 95% confidence) for a single patient was calculated.
**Results or Findings:** Reproducibility analysis (2 radiologist consensus; 14 patients; 41 lesions): Variance ratios were >15 for all biomarkers. Size had wCV of 3.5% and R-value of 9.5%; SMR had wCV of 7.4% and R-value of 19.8%; ADC had wCV of 2% and R-value of 5.2%; rFF% had wCV of 9.4% and R-value of 24.9%. Inter- and intra-observer variability (for 2 independent radiologists; 10 patients; different lesions) were similar (ICC >0.8) indicating consistent reader performance.
**Conclusion:** Higher reproducibility test variance ratios and interclass correlations of clinically relevant biomarkers indicate the reliability of radiological assessments regardless of the observer. Changes in aggregated lesion size of >3mm, SMR of > 1.4 au, ADC of >53 µm2/s and rFF% of > 2.7% can be used as bone tumour response biomarkers in women with metastatic breast cancer.
**Limitations:** This abstract represents a preliminary part of a larger study that aims to evaluate whether changes in quantitative and qualitative WB-MRI biomarkers could be predictive of outcomes in patients with bone-predominant MBC on SACT.
**Funding for this study:** This work was funded by the Paul Strickland Scanner Centre Charity (UK registered charity number 298867) and the Fighting Breast Cancer (UK registered charity number 1091882).
**Ethics committee - additional information:** All procedures permorfed in this study (ClinicalTrials.gov identifier: NCT03266744) followed the ethical standards of the National Health Service Health research Authority East of England-Cambridge East Research Committee and the 1964 Helsinki Declaration and its later amendments or comparable ethical standards.
**Author Disclosures:**
N. Jane Taylor: Nothing to disclose
Michael Kosmin: Nothing to disclose
Giuseppe Petralia: Nothing to disclose
Will McGuire: Nothing to disclose
Caterina Pizzi: Nothing to disclose
Anwar R. R. Padhani: Nothing to disclose
Caterina Sattin: Nothing to disclose
Andreas Makris: Nothing to disclose

**Spectral CT imaging for assessment of metastases in melanoma patients: Multi-reader evaluation**
*C. Nelles*, P. Rauen, T. M. Dratsch, D. Maintz, J. Kottlors, N. Große Hokamp, D. Zopfs, T. Persigehl, S. Lennartz; Cologne/DE

**Purpose or Learning Objective:** To investigate the sensitivity, specificity and qualitative assessment of spectral image reconstructions for metastases in melanoma patients in a large-scale, multi-reader evaluation.
**Methods or Background:** In total, 308 patients with melanoma, 95 patients with metastases and a control group of 213 patients without metastases, who underwent staging CT of the chest and abdomen on a dual-layer dual-energy CT system (dlDECT) were retrospectively included. Conventional images (CI), iodine overlays (IO) and virtual monoenergetic images at 40 keV (VMI40keV) were reconstructed. Six radiologists (three experienced, three less experienced) evaluated all cases in a CI-based session and a session based on a combination of CI, IO and VMI40keV. Readers were asked to binarily determine presence of metastases in specific tissues and to indicate diagnostic certainty and lesion delineation on 5-point Likert scales.
**Results or Findings:** Sensitivity for detection of metastases in skeletal muscle and peritoneum was significantly higher for the spectral assessment (for skeletal muscle 70% vs. 61%; for peritoneum 76% vs. 62%, both: $p < 0.05$). For subcutaneous metastases, there was a significant increase in specificity (92% vs. 89%, $p < 0.05$), however accompanied with a significant decrease in sensitivity (79% vs. 85%, $p < 0.05$). Diagnostic certainty was rated significantly higher for spectral images than CI for 100% (6/6) of the assessed tissues, whereas improvements in lesion delineation were noted for skeletal muscle, subcutaneous tissue and pancreas.
**Conclusion:** In melanoma patients, the benefit of dlDECT-derived spectral reconstructions depends on the assessed tissue. While assessment of skeletal muscle and peritoneal metastases was significantly improved, low or absent iodine uptake of subcutaneous lesions led to false negatives and a consecutive decrease in sensitivity.
**Limitations:** -Retrospective, monocenter study design -The results may not be generalisable to all different DECT platforms
**Funding for this study:** Funding was provided by the German Research Foundation (DFG, FI 773/15-1).
**Ethics committee - additional information:** After reviewing the study design, the institutional review board waived the need for informed patient consent for this retrospective single-center study.
**Author Disclosures:**
Simon Lennartz: Speaker: Amboss GmbH Author: Amboss GmbH
Nils Große Hokamp: Consultant: Bristol-Myers Squibb; BeiGene Speaker: Philips Healthcare; Amboss GmbH Research/Grant Support: Philips Healthcare
David Zopfs: Research/Grant Support: Philips Healthcare
David Maintz: Nothing to disclose
Christian Nelles: Nothing to disclose
Philip Rauen: Nothing to disclose
Jonathan Kottlors: Nothing to disclose
Thomas Markus Dratsch: Nothing to disclose
Thorsten Persigehl: Nothing to disclose

**Accuracy of DOTATATE PET CT versus DOTATATE PET MR in the Assessment of Multifocal Small Bowel Neuroendocrine Neoplasm**
*A. Keane*[1], H. Takahashi[2], C. Zhang[3], C. Thiels[2], P. Navin[2]; [1]Dublin/IE, [2]Rochester, MN/US, [3]Pheonix, AZ/US
*(annakeane@rcsi.ie)*

**Purpose or Learning Objective:** Small bowel neuroendocrine neoplasms (sbNENs) provide challenges in diagnosis and management. Current gold standard treatment is open resection with manual small bowel palpation as 45-50% of sbNEN have synchronous lesions. Advancements in nuclear medicine have improved preoperative assessment, potentially negating manual palpation and allowing for laparoscopic approach. DOTATATE PET CT (DPCT) and PET MR can identify somatostatin receptors, frequently expressed on sbNENs. We aim to assess the accuracy of DPCT versus PET MR in determining multifocality of sbNENs.
**Methods or Background:** Multicentre retrospective analysis was performed on patients with sbNEN who underwent open small bowel resection between January 2016 to August 2022 and had either preoperative DPCT or PET MR. Blinded retrospective review of images for small bowel lesions was performed by two fellowship-trained radiologists and compared to postoperative pathology reports. Final radiology diagnosis was attained by consensus agreement. Descriptive statistics, sensitivity, specificity, positive predictive value (PPV), negative predictive value (NPV), accuracy, and area under the receiver operator characteristic curve (AUROC) of DPCT and PET MR were compared.
**Results or Findings:** Seventy-eight patients met inclusion criteria. Fifty-six (71.8%) had preoperative DPCT, twenty-two (28.2%) had PET MR. Forty-eight patients (61.5%) had multiple (> 1)sbNENs on final pathology. Thirty-six (75.0%) were identified on preoperative imaging. DPCT and PET MR demonstrated sensitivity of 85.0% and 58.0% respectively, specificity of 81.0% and 60.0%, PPV of 87.9% and 63.6%, NPV of 77.3% and 54.6%, and accuracy of 83.6% and 59.0% for the presence of multifocal disease. AUROC was 0.8 for DPCT and 0.5 for PET MR.
**Conclusion:** DPCT demonstrated higher accuracy in identifying multifocal sbNEN versus PET MR. However, as the NPV is insufficient, we conclude that preoperative DPCT should not replace open exploration.
**Limitations:** Retrospective study, underpowered due to rarity of sbNENs.
**Funding for this study:** Nil
**Ethics committee - additional information:** IRB (Institutional Review Board)
**Author Disclosures:**
Cornelius Thiels: Nothing to disclose
Hiroaki Takahashi: Nothing to disclose
Anna Keane: Nothing to disclose
Chi Zhang: Nothing to disclose
Patrick Navin: Nothing to disclose

**Discord Dilemmas in Lung Cancer Clinical Trials: Navigating Reader Variability in Response Assessment**
*H. Beaumont*[1], R. Gill[2], N. Faye[1], A. Iannessi[1]; [1]Valbonne/FR, [2]Boston, MA/US
*(hubertbeaumont@hotmail.com)*

**Purpose or Learning Objective:** In lung cancer trials, blinded independent central response assessment with double reads is challenging and prone to interobserver variability. We analyzed the patterns of discordance in reporting Progressive Disease (PD) and performed a root cause analysis.
**Methods or Background:** We retrospectively analyzed data from five clinical trials evaluating 1932 lung cancer patients treated with targeted and immune therapies, read by 17 central readers. Progressive Disease was defined based on RECIST 1.1 criteria. The RECIST components were the Sum of tumors Diameter (SOD), the unequivocal progression of the non-Target Lesions (NTL) and the detection of New Lesion (NL). We analyzed the RECIST components according to 1) Concordant/discordant PD detection; 2) Positive Predictive Value (PPV) of declaring PD; 3) Offset versus single reader detection.
**Results or Findings:** Discordance in PD was observed in 39.2% (675/1718) of patients, with adjudication of PD for 62.5% (422/675) based on 44.8% (95%CI: 39.9, 49.8) new lesion detection, 28.3% (95%CI: 24.0, 33.0) significant increase of SOD and 12.6% (95%CI: 9.5, 16.2) unequivocal non-target

Wednesday progression. For 54.2% of concordant PD, at least one reader involved more than one RECIST component. The PPV for increased SOD was 0.59, rising to 0.89 when multiple RECIST components were involved. In 49.2% of discrepant cases, PD was reported with a delay of one cycle in majority of cases (80%). Confirmation rate for NLs in the lungs was lowest (40.6%) and new nodal lesions was highest (88.4%).
**Conclusion:** Discordance among trained central readers in lung cancer trials is common. New lesion detection is pivotal in the detection of PD, also the main cause of discordances. Involving multiple RECIST components improves the reliability of assessments. When relying on NL only, the detection of extra pulmonary lesion is more reliable.
**Limitations:** No limitations
**Funding for this study:** No funding
**Ethics committee - additional information:** Median Technologies institutional ethics committee approved the study, informed consent was not required for this retrospective analysis
**Author Disclosures:**
Nathalie Faye: Consultant: Median Technologies
Madam Ritu Gill: Nothing to disclose
Antoine Iannessi: Employee: Median Technologies
Hubert Beaumont: Employee: Median Technologies

**Detection of abdominal metastases in brain tumor patients following ventriculoperitoneal shunting**
*N. Plakhotina*, A. V. Smirnova, K. Boiko, V. Bikulov, P. Ivanov; Saint-Petersburg/RU
*(plahotinadezhda@gmail.com)*

**Purpose or Learning Objective:** To develop a diagnostic algorithm and identify imaging patterns for metastatic abdominal lesions in children with brain tumors following shunt placement.
**Methods or Background:** Clinical cases of shunt-associated metastasis of primary brain tumors in children, identified through CT, MRI, and laparoscopy, with morphological confirmation.
**Results or Findings:** Acute abdominal pain developed in children with embryonal tumors (2 patients with medulloblastoma, 1 patient with ATRT) and 1 child with ependymoma in remission, as well as during chemotherapy. CT scans revealed multiple nodules of varying sizes within the heterogeneous adipose tissue of the abdominal cavity. MRI identified multiple solid isointense tumors on both T1- and T2-WI, exhibiting significant contrast enhancement and diffusion restriction. Tumor sizes ranged from 3 to 15 mm. A notable feature was the predominant spread in the interintestinal spaces, as well as the subdiaphragmatic and subhepatic regions, which complicates visualization. In one of the cases, imaging did not yield conclusive results, and metastases were confirmed only at autopsy.
**Conclusion:** Although shunt-associated metastasis in CNS tumors is extremely rare, it carries a very poor prognosis. Dynamic monitoring of children with brain tumor with a shunt system should include abdominal examinations (ultrasound or MRI). The development of an acute abdomen requires urgent evaluation to rule out tumor presence, including laparoscopy if indicated. Reports of such cases suggest that ventriculoperitoneal shunting for occlusive hydrocephalus should be considered only as a last resort when temporary external drainage is not feasible.
**Limitations:** N/A
**Funding for this study:** N/A
**Ethics committee - additional information:** N/A
**Author Disclosures:**
Konstantin Boiko: Nothing to disclose
Vyacheslav Bikulov: Nothing to disclose
Nadezhda Plakhotina: Nothing to disclose
Pavel Ivanov: Nothing to disclose
Alina Vyacheslavovna Smirnova: Nothing to disclose

**Imaging treatment response in High Grade Serous Ovarian Cancer: Metabolic imaging vs Cell death imaging**
*M. L. Chia*, K. Brindle; Cambridge/UK

**Purpose or Learning Objective:** Given the poor therapy response noted in advanced stage High Grade Serous Ovarian Cancer (HGSOC) patients and the lack of fast and reliable treatment response monitoring methods, there is a need to predict treatment response earlier. This project investigated the potential of metabolic and cell death imaging techniques to detect early treatment response to standard-of-care chemotherapy in HGSOC patients.
**Methods or Background:** HGSOC cells, derived from the ascites of stage 3-4 HGSOC patients, were maintained as patient derived organoids(PDO) and implanted into mice subcutaneously. The resulting tumours were imaged with various imaging techniques. Metabolic imaging techniques included MRS (hyperpolarized [1-13C]pyruvate metabolism) and PET(measurements of 2-deoxy-2-[fluorine-18]fluoro-D-glucose uptake). Cell death imaging techniques included diffusion-weighted 1H MRI (DWI) and 2H MRSI measurements of [2,3-2H2]fumarate metabolism. PDO 2(carboplatin sensitive) and PDO 5(Carboplatin resistant) tumour models were treated with i.v. Carboplatin (50mg/kg) or drug vehicle weekly, with imaging at baseline and weekly thereafter.
**Results or Findings:** Both metabolic imaging techniques were successful in discriminating responding from non-responding tumours to Carboplatin before there was a change in tumour volume. The techniques for detecting cell death were not as sensitive for detecting treatment response, which may reflect a slow accumulation of dead cells post treatment, a lack of knowledge of when the rate of cell death increases post treatment and immune clearance of dead cells.
**Conclusion:** Imaging with hyperpolarized [1-13C]pyruvate has the potential to be used in the clinic to detect the early treatment response in HGSOC patients.
**Limitations:** We only tested Carboplatin but other chemotherapies or combination treatment regimens would also be important for investigation as they might produce a greater and faster increase in cell death, possibly allowing cell death detection techniques to be more successful. This will be part of future work.
**Funding for this study:** Cancer Research UK Cambridge Institute Core funding
**Ethics committee - additional information:** Na
**Author Disclosures:**
Ming Li Chia: Nothing to disclose
Kevin Brindle: Nothing to disclose

**08:00-09:30 Research Stage 4**

### Research Presentation Session: Musculoskeletal

### RPS 110

### AI, radiomics and other technologies supporting MSK diagnostics

**Moderator**
V. Mascarenhas; Lisbon/PT
*(vmascarenhas@me.com)*

**AI-Driven SuperResolution reconstruction for high-quality, fast MR imaging of the lumbar spine: enhanced image clarity for pathology detection**
*R. Hahnfeldt*[1], R. A. Terzis[1], T. M. Dratsch[1], J. Bremm[1], P. Rauen[1], K. Weiss[2], D. Maintz[1], G. Bratke[1], A-I. Iuga[1]; [1]Cologne/DE, [2]Hamburg/DE

**Purpose or Learning Objective:** The aim of this study was to investigate whether a 2D MRI lumbar spine protocol with an AI-based SuperResolution reconstruction method meets the requirements for clinical diagnostic purposes.
**Methods or Background:** In this retrospective study, 25 patients underwent MRI examinations of the lumbar spine using a 1,5T MRI scanner (Philips Ingenia 1.5T, Best, NL). The MRI protocol included three sagittal sequences (STIR, T1 TSE, T2 TSE), and an axial T2 TSE sequence. The images were acquired in both standard and low resolution. Both the clinical standard (Compressed SENSE (CS)) and the new AI-based SuperResolution reconstruction method (SuperRes-AI) were applied. Four experienced readers (two radiologists and two orthopedic surgeons) evaluated the sequences for pathologies (bone marrow edema, neuroforaminal stenosis, disc herniation).
**Results or Findings:** The acquisition time for the clinical standard sequences was 11 minutes and 5 seconds. In contrast, the acquisition time for the low resolution SuperRes-AI sequences was 7 minutes and 37 seconds (31% scan time reduction). A generalized estimating equations (GEE) analysis revealed no significant differences in the sensitivity for detecting edema between reader groups and reconstruction algorithms (all p>0.99). Bonferroni-corrected post-hoc tests in a GEE analysis revealed significantly higher sensitivity for detecting neuroforaminal stenosis with AI-powered reconstruction compared to conventional algorithms among radiologists (p=0.001), with no other significant differences observed.
**Conclusion:** The new AI-based SuperResolution reconstruction of low-resolution 2D MRI sequences of the lumbar spine allows for a reduction in acquisition time of approximately 31% without compromising diagnostic quality, showing significantly higher sensitivity for detecting neuroforaminal stenosis. The AI-based SuperResolution method improves MRI efficiency by significantly reducing scan times without compromising image quality, potentially enhancing sensitivity in pathology detection, offering advantages for patient comfort and clinical workflow.
**Limitations:** Not applicable.

**Funding for this study:** No funding was provided for this study.
**Ethics committee - additional information:** The ethics committee notification can be found under the number DRKS00024156
**Author Disclosures:**
Robert Hahnfeldt: Nothing to disclose
Andra-Iza Iuga: Nothing to disclose
David Maintz: Nothing to disclose
Philip Rauen: Nothing to disclose
Johannes Bremm: Nothing to disclose
Thomas Markus Dratsch: Nothing to disclose
Kilian Weiss: Employee: Philips GmbH Market DACH
Robert Angelo Terzis: Nothing to disclose
Grischa Bratke: Nothing to disclose

**AI-based Detection of Postoperative Abnormalities Following Lumbar Fusion Surgery in Spine Radiographs**
M. Kim[1], *J. Song*[1], K. Sung[2], E. Oh[1]; [1]Seoul/KR, [2]Los Angeles, CA/US
*(jmsong@promedius.ai)*

**Purpose or Learning Objective:** The purpose of this study is to develop a deep learning-based system to detect postoperative abnormalities in spine radiographs following lumbar fusion surgery. This system aims to assist radiologists by detecting postoperative abnormalities.
**Methods or Background:** A total of 1,505 spine radiographs from 85 patients who underwent lumbar fusion surgery were collected at a secondary healthcare facility between February 2018 and January 2022. These radiographs, taken post-operation and during follow-up visits, included anteroposterior, lateral, flexion, and extension views. Annotations for periprosthetic loosening, cage subsidence, and compression fracture were performed by a musculoskeletal radiologist, and verified with CT scans. The Co-DETR model was trained on a subset of 634 radiographs from 74 patients with 726 annotations. The class distribution included 58, 24, and 17 patients yielding 278, 215, and 168 images respectively, with each image averaging 1.10 annotations. Initial training was conducted on a public dataset (FracAtlas), followed by transfer learning to enhance detection of postoperative abnormalities. Negative samples were included to boost training efficiency, and model performance was evaluated using mean Average Precision (mAP).
**Results or Findings:** Periprosthetic loosening achieved an mAP score of 0.601 with 0.5 IoU threshold. The mAP score for each class of periprosthetic loosening, cage subsidence, and compression fracture were 0.565, 0.667, 0.572, respectively.
**Conclusion:** The study demonstrates the potential of detecting postoperative abnormalities in spine radiographs after lumbar fusion surgery using deep learning. The results indicate a foundational potential for enhancing diagnostic capabilities in clinical settings. The potential of this approach to improve early detection of complications could lead to more timely interventions and better patient outcomes.
**Limitations:** Further validation is required to optimize its performance, particularly to support radiologists in settings with limited access to specialists.
**Funding for this study:** Not applicable
**Ethics committee - additional information:** IRB No. 2022-08-018
**Author Disclosures:**
Jeongmin Song: Nothing to disclose
Minjee Kim: Nothing to disclose
Kyunghyun Sung: Nothing to disclose
Eunsun Oh: Nothing to disclose

**Post-operative X-rays radiomics-based machine learning to predict two-year clinical outcome in patients with lumbar spine arthrodesis**
*I. C. Pizza*[1], M. Pedullà[2], S. Fusco[2], F. Serpi[2], D. Albano[3], C. Messina[2], S. Gitto[2], L. M. Sconfienza[2]; [1]Eboli/IT, [2]Milan/IT, [3]Cefalu'/IT
*(irene.pizza@unimi.it)*

**Purpose or Learning Objective:** The aim of this study is to predict two-year clinical outcome in patients with lumbar spine arthrodesis using machine learning and radiomics based on post-operative X-rays.
**Methods or Background:** This retrospective study was performed at a tertiary orthopaedic centre and included 162 patients with lumbar spine arthrodesis, post-operative X-rays available for analysis and minimum follow-up of two years. Clinical follow-up was evaluated at two years using Oswestry Disability Index (ODI): ODI≤20 indicated good clinical outcome (n=90), ODI>20 indicated poor clinical outcome (n=72). All X-rays were manually segmented by drawing rectangular regions of interest including the arthrodesis and one adjacent non-operated vertebra on both proximal and distal sides. Radiomic features were extracted. After feature selection and class balancing, machine learning (three ensembles of Random Forest classifiers) was trained, validated using nested 10-fold cross-validation and tested.
**Results or Findings:** After training and cross-validation, in the test dataset machine learning showed ROC-AUC (%) of 74 (majority vote), 72.9** (mean) [confidence interval 69-76.7], accuracy (%) of 68 (majority vote), 67.7** (mean) [65.9-69.5], sensitivity (%) of 60 (majority vote), 60.6** (mean) [52.7-68.6], specificity (%) of 74 (majority vote), 73.3** (mean) [67.8-78.9], PPV (%) of 65 (majority vote), 64.6** (mean) [62-67.1], and NPV (%) of 70 (majority vote), 70** (mean) [67.1-72.9] (*p<0.05, **p<0.005).
**Conclusion:** Radiomics-based machine learning may assist clinicians in predicting clinical outcome of patients with lumbar spine arthrodesis based on post-operative X-rays, thus modifying physical rehabilitation and therapeutic strategies accordingly.
**Limitations:** Retrospective study.
**Funding for this study:** No funding was provided for this study.
**Ethics committee - additional information:** Approved by Local Ethics Committee (RETRORAD protocol)
**Author Disclosures:**
Irene Carmen Pizza: Nothing to disclose
Stefano Fusco: Nothing to disclose
Carmelo Messina: Nothing to disclose
Salvatore Gitto: Nothing to disclose
Luca Maria Sconfienza: Nothing to disclose
Martina Pedullà: Nothing to disclose
Francesca Serpi: Nothing to disclose
Domenico Albano: Nothing to disclose

**AI based thoracolumbar and sacral spine fracture detection for computed tomography**
*J-B. Pialat*[1], D. Gicquel[1], A-K. Golla[2], C. Bürger[2], C. Lorenz[2], M. Villien[1], S. Gouttard[1], A. Vlachomitrou[1], T. Klinder[2]; [1]Lyon/FR, [2]Hamburg/DE
*(jean-baptiste.pialat@chu-lyon.fr)*

**Purpose or Learning Objective:** AI algorithms which detect vertebral fractures generate limited classifications which only identify vertebral body fracture. We propose a thoracolumbar and sacral spine fracture detection algorithm able to identify individual fracture locations in both the vertebral body and the posterior arch . It segments the entire spine, extracts spine-aligned sub volumes and detects spinal fractures using a convolutional neural network.
**Methods or Background:** 195 CT scans from polytraumatized patients were collected in a single-center retrospective clinical study. Dataset was split into training (n=145) and validation (n=50) sets. Accuracy for identification of injury location within the body was assessed in the validation set using a Free Response ROC (FROC) curve and performance at the vertebral body level was measured using a Receiver Operating Characteristic (ROC) curve. A subsequent test set including 173 patients ( fractured N=109, non fractured N= 64) was analyzed with the same algorithm. Performance was assessed similarly using FROC curve.
**Results or Findings:** The algorithm detected 87.3% of the 775 spinal fracture locations of the validation set using a false positive threshold of 5 per case. It detected 92.4% of the fractured vertebrae. 249 false positives were detected, most of which were easily rejected upon review by radiologists. 26 false negatives were found, most of which were transverse process fractures. There were 7 vertebral body fractures; all were single endplate stable fractures. In the test set, the algorithm detected 88.6% of the 255 fractures using a false positive threshold of 5 per case.
**Conclusion:** We have developed and validated a deep learning algorithm which determines location of fractures in the whole vertebra with reasonable accuracy.
**Limitations:** This as to be tested prospectively in routine emergency condition to assess the gain in time / sensitivity
**Funding for this study:** Collaborative study between Hospices Civils de Lyon and Philips using GOPI research fundings
**Ethics committee - additional information:** Approved by local ethics commitee
**Author Disclosures:**
Tobias Klinder: Employee: Philips Healthcare
Marjorie Villien: Employee: Philips Healthcare
Christian Lorenz: Employee: Philips Healthcare
David Gicquel: Research/Grant Support: Philips Healthcare
Sylvain Gouttard: Nothing to disclose
Anna Vlachomitrou: Employee: Philips Healthcare
Jean-Baptiste Pialat: Research/Grant Support: Philips Heathcare
Christian Bürger: Employee: Philips Healthcare
Alena-Kathrin Golla: Employee: Philips Healthcare

**The new frontier of MRI: virtual dissection with 3DPDw sequence. A pilot study on ATiFL anatomy**
*G. Del Gaudio*[1], G. Vuurberg[2], M. Dalmau-Pastor[3], G. Kerkhoffs[4], M. Maas[4]; [1]Rome/IT, [2]Weesp/NL, [3]Barcelona/ES, [4]Amsterdam/NL
*(g.d.gaudio@gmail.com)*

**Purpose or Learning Objective:** In the literature, there is much conflicting data regarding the anatomy of the anterior tibiofibular ligament (ATiFL), even in studies with anatomical specimens. Therefore, this study aims to reassess the anatomy of this ligament using MRI with a high-resolution isotropic 3D-PDw sequence.
**Methods or Background:** From February to May 2024, 72 MRI scans (3T) of the ankle were performed at Amsterdam UMC. The inclusion criterion was patients over 16 years of age. The exclusion criteria were: absence of a 3D-PDw scan, ATiFL trauma or surgery, congenital anomalies, metallic or movement artifacts. The 43 3D-PDw valid scans, allowed for aligning the planes along the individual bundles of the ligament.
**Results or Findings:** The high spatial resolution (0,23mm) of 3DPDw allowed the identification of three bundles: superior, intermediate and inferior. Regarding dimensions the superior is the thickest and widest (mean 2.68x9.28mm) and the inferior the longest (mean 15.45mm). Regarding orientation (axial from the fibula to the tibia) the superior and inferior have a transverse orientation, while the intermediate is oriented backward. Regarding the shape, they are fanned in 97.7%, 71.7%, and 25.6% respectively, while they are band-like in the remaining cases. We did not identify any anatomical variance regarding the number of bundles.
**Conclusion:** The use of volumetric isotropic sequences as the 3D-PDw, can be a very useful tool for the anatomical study of ligamentous structures in the absence of available anatomical specimens. Understanding the exact anatomy of this structure is crucial for managing both acute and chronic traumatic pathologies (impingement, overuse), especially in young patients and athletes.
**Limitations:** Sample size and lack of anatomical specimen comparison.
**Funding for this study:** No funding.
**Ethics committee - additional information:** This study received a waiver by the ethical committee according to local rules and regulations.
**Author Disclosures:**
Giovanni Del Gaudio: Nothing to disclose
Gwendolyn Vuurberg: Nothing to disclose
Miquel Dalmau-Pastor: Nothing to disclose
Ginom M.J. Kerkhoffs: Nothing to disclose
Mario Maas: Nothing to disclose

**MRI biomarker assessment of Duchenne muscular dystrophy disease progression: a 12-month longitudinal study**
*Y. Song*, H. Xu, R. Xu, K. Xu; Chengdu/CN
*(babysong020@163.com)*

**Purpose or Learning Objective:** To evaluate the disease progression in patients with Duchenne muscular dystrophy (DMD) by using multi-modal quantitative magnetic resonance imaging (qMRI), and comparing the responsiveness of these imaging indicators with the clinical function scales.
**Methods or Background:** 130 DMD patients were enrolled and underwent MRI examination of hip muscles to determine fat fraction (FF) and longitudinal relaxation time (T1). All participants returned for follow-up at an average of 12 months. According to the baseline North Star Ambulatory Assessment (NSAA) score, all patients were divided into three subgroups: mild (76-100 score), moderate (51-75 score) and severe (0-50 score) functional decline. Standardized response mean (SRM) was used as the responsiveness to the disease progression, and the responsiveness of qMRI and clinical function scales to the disease progression in different DMD stages was compared. SRM>0.8 is considered as a high response to disease progression.
**Results or Findings:** The overall SRM of MRI biomarkers is higher than that of the clinical function scales. For mild group, FF of adductors and abductors have higher responsiveness, with SRM of 0.816 and 1.043, respectively. For moderate group, FF of all muscle groups have a high responsiveness, and the SRM are between 1.004 and 1.606. For severe group, T1 of abductors and FF of all muscle groups have high responsiveness, and SRM are between 0.867 and 1.633. However, the SRM of the clinical function scales for patients with different disease stages are all less than 0.8.
**Conclusion:** The sensitivity of MRI biomarkers to DMD disease progression is higher than that of clinical function scales, especially the FF of gluteal muscles is more sensitive to disease progression, and the sensitivity indicators are different in different disease stages.
**Limitations:** This study didn't discuss whether patients received steroid therapy.
**Funding for this study:** National Natural Science Foundation of China (82271981)
**Ethics committee - additional information:** ChiCTR1800018340
**Author Disclosures:**
Ke Xu: Nothing to disclose
Huayan Xu: Nothing to disclose
Yu Song: Nothing to disclose
Rong Xu: Nothing to disclose

**Hip Imaging: Radiation-free 3D models based on 3D MRI of the hip joint for children with Slipped capital femoral epiphysis**
T. D. Lerch, *T. Kaim*, K. Ziebarth, M. K. Meier, J. D. Busch; Bern/CH
*(tilman.kaim@bluewin.ch)*

**Purpose or Learning Objective:** Slipped capital femoral epiphyses (SCFE) is a common pediatric hip disease with the risk of osteoarthritis and impingement deformities, and 3D models could be useful for patient-specific analysis. Therefore, magnetic resonance imaging (MRI) bone segmentation was investigated.
**Methods or Background:** A retrospective IRB-approved study involving 23 symptomatic pediatric patients (23 hips) with SCFE was performed. All patients underwent preoperative hip MR with pelvic axial high-resolution images (T1 VIBE DIXON images). Slice thickness was 1.2 mm. Mean age was 12 ± 2 years. All patients underwent surgical treatment. Manual and automatic MRI-based bone segmentation was compared. automatic bone segmentation was performed by machine learning algorithm, a previously used and validated convolutional neural network trained for adult pelvis bone segmentaiton was adapted to pelvis of children.
**Results or Findings:** Manual MRI-based bone segmentation was feasible (all patients, 100%, duration 4-5 hours per case). Dice coefficient was calculated to assess differences between manual and automatic bone segmentation, Dice coefficient was 82% for the pelvis and 88% for proximal femur. Precision was 80% for the pelvis and 94% for proximal femur.
**Conclusion:** MRI-based 3D models were feasible for SCFE patients. Three-dimensional models could be useful for SCFE patients for preoperative 3D printing and deformity analysis. This could aid for patient-specific diagnosis, treatment decisions, and preoperative planning. MRI-based 3D models are radiation-free and could be used instead of CT-based 3D models in the future for computer-assisted 3D simulation of surgery.
**Limitations:** MRI is expensive and access is limited
**Funding for this study:** None
**Ethics committee - additional information:** IRB approval was obtained
**Author Disclosures:**
Kai Ziebarth: Nothing to disclose
Tilman Kaim: Nothing to disclose
Till Dominic Lerch: Nothing to disclose
Malin Kristin Meier: Nothing to disclose
Jasmin D. Busch: Nothing to disclose

**Could a single isotropic 3D sequence replace a multisequence knee MRI in the new era of deep learning reconstruction?**
*E. Nikolova*[1], J. Kroschke[1], C. Obermüller[1], F. Zecca[2], K. Pawlus[1], T. Rauer[1], F. Ensle[1]; [1]Zurich/CH, [2]Cagliari/IT

**Purpose or Learning Objective:** To assess whether a single isotropic 3D proton-density-weighted fat-saturated (PDFS) sequence could replace a standard 2D multisequence MRI protocol for comprehensive examination of the knee using deep learning reconstruction (DLR).
**Methods or Background:** In this retrospective study, 95 consecutive patients > 18 years without history of prior knee surgery undergoing MRI knee examination at the same 1.5 Tesla scanner between May 2023 and July 2024 were included. Standard MRI protocol with DLR consisted of a 3D PDFS sequence and five 2D fast-spin-echo sequences in various orientations. Two radiologists separately evaluated the 3D sequence in all three planes and the 2D sequences, assessing pathologies of bone, cartilage, menisci and ligaments for all joint compartments, and overall image quality, diagnostic confidence and artifacts. Wilcoxon signed-rank test was used to compare Likert scale gradings, McNemar's test for binary grades. Interreader agreement was assessed with Cohen's kappa.
**Results or Findings:** There was no significant difference between protocols regarding assessment of medial(MC) and lateral compartment(LC) meniscus, (MC) and patellofemoral(PF) cartilage, medial and lateral collateral ligament, anterior and posterior cruciate ligament, MC and PF bone marrow edema(BME), and fractures in all compartments (p>0.05). Significant differences were shown in assessment of LC cartilage (p=0.002) and LC BME (p=0.04). Image quality and artifacts did not demonstrate significant differences. Diagnostic confidence was significantly higher for the 2D protocol(p=0.023). Interreader agreement overall was substantial for the 3D-PDFS(k=0.67) and 2D protocol (k=0.66).
**Conclusion:** Our results suggests comparable performance between a single 3D-PDFS and a multisequence 2D protocol using DLR for comprehensive assessment of knee structures, except for LC cartilage and BME. With DLR-powered image enhancement, 3D-PDFS might be able to partly replace 2D sequences for time-efficient knee MRI in the future.
**Limitations:** Retrospective study design. No arthroscopic reference standard.
**Funding for this study:** This research received no financial support.
**Ethics committee - additional information:** Not applicable

**Author Disclosures:**
Elizabet Nikolova: Nothing to disclose
Fabio Zecca: Nothing to disclose
Falko Ensle: Nothing to disclose
Karolina Pawlus: Nothing to disclose
Jonas Kroschke: Nothing to disclose
Carina Obermüller: Nothing to disclose
Thomas Rauer: Nothing to disclose

**Assessment of proximal tibial fractures with 3D FRACTURE (fast field echo resembling a CT using restricted echo-spacing) MRI – Intraindividual comparison with computed tomography**

*I. Ristow*[1], S. Zhang[2], C. Riedel[1], A. Lenz[1], M. Krause[1], G. Adam[1], P. Bannas[1], F. O. Henes[1], L. Well[1]; [1]Hamburg/DE, [2]Best/NL

**Purpose or Learning Objective:** To evaluate the feasibility and diagnostic performance of a 3D FRACTURE (fast field echo resembling a CT using restricted echo-spacing) MRI sequence for the detection and classification of proximal tibial fractures compared with CT.
**Methods or Background:** We retrospectively included 126 patients (85 male; 39.6±14.5 years) from two centers following acute knee injury. Patients underwent knee MRI at 3T including FRACTURE-MRI. Additional CT was performed in patients with tibial fractures (32.5%; n=41) as the reference standard for fracture classification. Two radiologists independently evaluated FRACTURE-MRI for the presence of fractures and classified them according to AO/OTA, Schatzker, and the 10-segment classification. Diagnostic performance of FRACTURE-MRI was assessed using crosstabulations. Inter-reader agreement was estimated using Krippendorff's alpha. Image quality was graded on a five-point scale (5=excellent; 1=inadequate definition of fracture lines and fracture displacement) and assessed using estimated marginal means.
**Results or Findings:** Fractures were detected by FRACTURE-MRI with a sensitivity of 91.5% (83.2–96.5%) and a specificity of 97.1% (93.3–99.0%). Regarding fracture classification, diagnostic performances were slightly lower, with the 10-segment classification yielding the best sensitivity of 85.7% (81.4–89.3%) and specificity of 97.4% (96.6–98.0%), and the Schatzker classification yielding the lowest sensitivity of 78.2% (67.4–86.8%) and specificity of 97.7% (94.1–99.4%). Inter-reader agreement across the whole cohort was excellent (Krippendorff's alpha 0.89–0.96) and when considering only patients with fractures, good to acceptable (0.48–0.91). Image quality was rated good (estimated marginal mean 4.3 (4.1–4.4)).
**Conclusion:** FRACTURE-MRI is feasible at 3T enabling accurate delineation of fracture lines for precise diagnosis and classification of proximal tibial fractures.
**Limitations:** Future studies need to address in a comparative intra-individual setting whether the diagnostic performance of FRACTURE-MRI is better or equivalent to other CT-like bone imaging techniques, such as UTE/ZTE, GRE, or SWI, for fracture detection.
**Funding for this study:** N/A
**Ethics committee - additional information:** The retrospective study was approved by the local institutional review board (Ärztekammer Hamburg).
**Author Disclosures:**
Gerhard Adam: Nothing to disclose
Christoph Riedel: Nothing to disclose
Matthias Krause: Nothing to disclose
Alexander Lenz: Nothing to disclose
Peter Bannas: Nothing to disclose
Frank Oliver Henes: Nothing to disclose
Shuo Zhang: Employee: Philips
Inka Ristow: Nothing to disclose
Lennart Well: Nothing to disclose

**qBone: a quantitative software for the semi-automated extraction of bone microarchitecture metrics in vivo using Photon-counting-detector CT and Artificial Intelligence**

*A. Ferrero*, J. Thorne, A. O. El Sadaney, K. Rajendran, C. Mccollough, F. Baffour; Rochester, MN/US
*(Ferrero.andrea@mayo.edu)*

**Purpose or Learning Objective:** Pathologies affecting bone health impact both mineral density (vBMD) and morphometric characteristics (thickness (Th) and spacing (Sp)) of trabecular (Tb) and cortical (Ct) bone. This work introduces a semi-automated software, qBone, which quantifies bone morphometry from in vivo CT scans of the extremities and the vertebral spine.
**Methods or Background:** protocols were optimized for extremity and spine exams using a commercial photon-counting-detector (PCD) CT system. A dedicated CNN algorithm was trained to reduce image noise of the spine CT exams while maintaining high resolution details. Adaptive segmentation algorithms automatically delineated Ct and Tb compartments allowing quantification of Th, Sp and vBMD for each. To validate the software's accuracy, 10 cadaveric wrists were scanned on HRpQCT and PCD-CT. A 3D-printed bone model (Ct.Th=2mm, Tb.Th=0.3mm and Tb.Sp=0.75mm) was used to assess the CNN denoising performance across different patient sizes. Finally, qBone was applied in vivo to multiple prospective cohorts for wrist and spine.
**Results or Findings:** optimized PCD-CT protocols for wrist (70kV, 12mGy, <0.1mSv) and spine (120kV, 40mGy, 8mSv) yielded <0.15mm in-plane resolution. Validation with cadaveric wrists and the 3D-printed bone model demonstrated excellent agreement in Ct.Th and Tb.Sp metrics. CNN denoising significantly improved trabecular morphometry accuracy in the spine for small and medium patient sizes. In vivo measurements (wrist, N=50; spine, N=14) for each metric (Tb.Th=0.3-0.45mm, Tb.Sp=0.6-1.05mm, Ct.Th=0.5-1.58mm, Ct.vBMD=450-600mg/cm3) were consistent with literature values.
**Conclusion:** qBone facilitates semi-automated quantification of bone morphometry from high resolution CT data, providing a comprehensive assessment of bone health in vivo beyond traditional mineral density.
**Limitations:** Comparisons with microCT are needed to validate metrics for vertebral bones. Additionally, qBone does not leverage spectral information to estimate vBMD in the spine.
**Funding for this study:** NIH R21ar084126-01a1
**Ethics committee - additional information:** IRB 23_005308, PI: Baffour
**Author Disclosures:**
Ahmed O. El Sadaney: Nothing to disclose
Francis Baffour: Nothing to disclose
Andrea Ferrero: Nothing to disclose
Jamison Thorne: Nothing to disclose
Kishore Rajendran: Nothing to disclose
Cynthia Mccollough: Nothing to disclose

**10:00-11:00** **Research Stage 1**

### Research Presentation Session: Head and Neck

### RPS 208

### Key insights in soft tissue neck imaging

**Moderator**
E. Loney; Halifax/UK
*(elizabeth_loney@hotmail.co.uk)*
**Author Disclosures:**
Elizabeth Loney: Consultant: DMC Healthcare Ltd

**Detection of MRI edema patterns in patients with acute neck infections: a prospective blinded multidisciplinary and multicenter interobserver human performance evaluation**

*J-P. T. Vierula*, J. Velhonoja, A. Sirén, J. Nurminen, M. J. Nyman, K. Mattila, J. Hirvonen; Turku/FI

**Purpose or Learning Objective:** In patients with acute neck infections, MRI shows reactive edema patterns that predict disease severity: retropharyngeal edema (RPE) and mediastinal edema (ME). How well radiologists and clinicians with diverse backgrounds and neck MRI experience can detect these edema patterns is unknown.
**Methods or Background:** This prospective, blinded, multidisciplinary, multicenter interobserver study evaluated human performance in detecting RPE and ME from axial in-phase and water T2-weighted Dixon images. Readers (N=28, including radiologists, neuroradiologists, radiology residents, head and neck surgeons) from all five university hospitals in Finland were briefly trained and assessed the presence of RPE and ME (yes/no) and rated their confidence (1-5) blinded to clinical data. Emergency MRI images were obtained from 60 patients with acute neck infections. Ten patients appeared twice to assess intraobserver variability. Sensitivity, specificity, accuracy, and interobserver agreement were assessed.
**Results or Findings:** Overall sensitivity, specificity, and accuracy were 0.89, 0.81, and 0.85 for RPE and 0.85, 0.81, and 0.82 for ME. ME accuracy correlated with confidence (p=0.002), whereas RPE accuracy did not (p=0.580). Radiologists achieved higher RPE sensitivity (p=0.01), RPE accuracy (p=0.04), ME sensitivity (p=0.01), and ME accuracy (p=0.02) than clinicians, whereas other group comparisons were not significant. High confidence was found for RPE (4.3) and ME (4.1). Overall, interobserver kappa was 0.61 (substantial) for RPE and 0.52 (moderate) for ME, with radiologists showing higher agreement than clinicians. Median intraobserver accuracy was 90% for both RPE and ME.

**Conclusion:** We show high diagnostic accuracy and substantial interobserver agreement for detecting clinically relevant reactive edema patterns on MRI in patients with acute neck infections. These results encourage using these biomarkers in clinical practice.
**Limitations:** Limited availability of emergency MRI.
**Funding for this study:** This study was financially supported by the Sigrid Jusélius Foundation. The funders had no role in study design, data collection and analysis, decision to publish, or preparation of the manuscript.
**Ethics committee - additional information:** A waiver for patient consent was not sought because it is not required by the national legislature for retrospective studies of existing data.
**Author Disclosures:**
Aapo Sirén: Nothing to disclose
Jarno Velhonoja: Nothing to disclose
Jussi Hirvonen: Nothing to disclose
Jari-Pekka Tapani Vierula: Nothing to disclose
Mikko Juhani Nyman: Nothing to disclose
Kimmo Mattila: Nothing to disclose
Janne Nurminen: Nothing to disclose

**Enhanced Survival Prediction in Nasopharyngeal Carcinoma Through Integrated Peritumoral and Intratumoral Radiomics**
*S. Khongwirotphan*, A. Prayongrat, S. Kitpanit, D. Kannarunimit, C. Chakkabat, V. Shotelersuk, S. Sriswasdi, C. Lertbutsayanukul, Y. Rakvongthai; Bangkok/TH
*(sararas.k@chula.ac.th)*

**Purpose or Learning Objective:** Accurately prediction of overall survival (OS) is essential for optimizing treatment strategies in nasopharyngeal carcinoma (NPC), thereby improving patient outcomes. This study aimed to enhance OS prediction by integrating radiomic features from both intra- and peritumoral areas, offering a novel biomarker approach beyond traditional clinical features.
**Methods or Background:** We analyzed 251 NPC patients treated with chemoradiotherapy between 2010 and 2019, all followed for a minimum of three years. Radiomics features were extracted from the gross tumor volume (GTV) contours and a 3-mm peritumoral area of the pre-treatment CT images using PyRadiomics v3.0.1. The robustness and predictive power of radiomic features were assessed by intraclass correlation and univariate Cox regression. Selected radiomics features were combined with clinical data (age, gender, T-stage, N-stage) for a multivariate analysis. Cox regression models were optimized with recursive feature elimination and 20 repetitions of five-fold cross-validation, reserving 20% of dataset for model testing.
**Results or Findings:** Addition of peritumoral radiomic features significantly ($P < 0.05$) improved survival predictions (C-index: 0.787±0.067 validation; 0.669 test), over intratumoral only (C-index: 0.755±0.063 validation; 0.626 test set). Integrating clinical data with intra- and peritumoral radiomics yielded the best model, with a C-index of 0.832±0.052 in validation and 0.727 in test set, that outperformed ($P < 0.05$) the model with clinical and intratumoral features (C-index of 0.769±0.066 and 0.705, respectively). The baseline clinical model yielded a C-index of 0.703±0.100 in validation and 0.618 in test set.
**Conclusion:** Integrating radiomic features from both intra- and peritumoral areas significantly improved OS predictions in NPC, surpassing traditional approaches that utilize only clinical and intratumoral radiomics. This could lead to more personalized treatment strategies, potentially improving patient outcomes.
**Limitations:** External validation is recommended for this single-center retrospective study before clinical use.
**Funding for this study:** This research project is supported by National Research Council of Thailand (NRCT) and grants for development of new faculty staff, Ratchadaphiseksomphot Fund, Chulalongkorn University
**Ethics committee - additional information:** The study received ethics approval from the Institutional Review Board of the Faculty of Medicine, Chulalongkorn University, Thailand (IRB no. 0630/66).
**Author Disclosures:**
Yothin Rakvongthai: Nothing to disclose
Sira Sriswasdi: Nothing to disclose
Anussara Prayongrat: Nothing to disclose
Danita Kannarunimit: Nothing to disclose
Chakkapong Chakkabat: Nothing to disclose
Vorasuk Shotelersuk: Nothing to disclose
Chawalit Lertbutsayanukul: Nothing to disclose
Sararas Khongwirotphan: Nothing to disclose
Sarin Kitpanit: Nothing to disclose

**Predictive potential of dynamic contrast-enhanced MRI and plasma-derived angiogenic factors for response to concurrent chemoradiotherapy in human papillomavirus-negative oropharyngeal cancer**
*A. Longo*, P. Hudler, P. Strojan, G. Plavc, L. Umek, K. Surlan Popovic; Ljubljana/SI
*(aljalongo@gmail.com)*

**Purpose or Learning Objective:** Dynamic contrast-enhanced magnetic resonance imaging (DCE-MRI) can assess tumour vascularity, which depends on the process of angiogenesis and affects tumour response to treatment. Our study explored the associations between DCE-MRI parameters and the expression of plasma angiogenic factors in human papilloma virus (HPV)-negative oropharyngeal cancer, as well as their predictive value for response to concurrent chemoradiotherapy (cCRT).
**Methods or Background:** 25 patients with locally advanced HPV-negative oropharyngeal carcinoma were prospectively enrolled in the study. DCE-MRI and blood plasma sampling were conducted before cCRT, after receiving a radiation dose of 20 Gy, and after the completion of cCRT. Perfusion parameters ktrans, kep, Ve, initial area under the curve (iAUC) and plasma expression levels of angiogenic factors (vascular endothelial growth factor [VEGF], connective tissue growth factor [CTGF], platelet-derived growth factor [PDGF]-AB, angiogenin [ANG], endostatin [END] and thrombospondin-1 [THBS1]) were measured at each time-point. Patients were stratified into responders and non-responders based on clinical evaluation. Differences and correlations between measures were used to generate prognostic models for response prediction.
**Results or Findings:** Higher perfusion parameter ktrans and higher plasma VEGF levels successfully discriminated responders from non-responders across all measured time-points, whereas higher iAUC and higher plasma PDGF-AB levels were also discriminative at selected time points. Using early intra-treatment measurements of ktrans and VEGF, a predictive model was created with cut-off values of 0.259 min-1 for ktrans and 62.5 pg/mL for plasma VEGF.
**Conclusion:** Early intra-treatment DCE-MRI parameter ktrans and plasma VEGF levels may be valuable early predictors of response to cCRT in HPV-negative oropharyngeal cancer.
**Limitations:** The small sample size and absence of healthy controls for angiogenic factors limit our findings. Perfusion parameter values in DCE-MRI can vary with different post-processing software, so comparisons between studies should be made with caution.
**Funding for this study:** This research was funded by the Slovenian Research and Innovation Agency (ARIS), grant number P3-0307.
**Ethics committee - additional information:** The study was approved by the National Medical Ethics Committee of the Republic of Slovenia (No. 0120-247/2019/4, 12 June 2019) and the Committee for Medical Ethics of the Institute of Oncology Ljubljana (OI: 28.5.2019, ERIDEK-0064/2019). Written informed consent was obtained from all patients.
**Author Disclosures:**
Lan Umek: Nothing to disclose
Gaber Plavc: Nothing to disclose
Katarina Surlan Popovic: Nothing to disclose
Petra Hudler: Nothing to disclose
Primož Strojan: Nothing to disclose
Alja Longo: Nothing to disclose

**The impact of quantification of circulating tumor HPV-DNA in the clinical and surgical management of patients with oropharyngeal squamous cell carcinoma**
*S. Ruggiero*[1], S. Lucchese[2], V. Dolcetti[1], S. Marzi[1], A. Vidiri[1]; [1]Rome/IT, [2]Naples/IT
*(s.ruggiero94@gmail.com)*

**Purpose or Learning Objective:** To conduct a multifactorial assessment in patients with oropharyngeal squamous cell carcinoma (OPSCC) that includes: 1) clinical characteristics; 2) detection of the amount of circulating tumor HPV-DNA in plasma (ctHPVDNA); 3) MRI-based volumetric analysis of both the primary tumor and lateral cervical lymph node metastases.
**Methods or Background:** A prospective study was conducted on patients with OPSCC. As controls, patients with suspected HPV-negative OPSCC were used. Both the primary tumor and lateral cervical lymph node metastases were manually delineated, slice by slice, by two expert head and neck radiologists on T2-weighted axial images at diagnosis. In patients with multiple lymph node metastases, the total volume was obtained by summing the volume of each lymph node. This measurement was correlated with the number of ctHPVDNA copies using the Mann-Whitney test.

Wednesday

**Results or Findings:** A total of 95 patients were included, of which 58 (61%) were p16+/HPV16+, 3 (3%) were p16-/HPV33+, 23 (24%) were p16-/HPV-, 1 (1%) was p16-/HPV16+, and 10 (10%) were p16+/HPV-. No association was found between the number of ctHPVDNA copies and the primary tumor volume. However, significant correlations emerged with the volume of lymph node metastases (Rho = 0.42, p = 0.004) and with the combined volume of the lymph node metastases and primary tumor (Rho = 0.51, p < 0.001).
**Conclusion:** ctHPVDNA is a promising biomarker that could potentially eliminate the need for solid biopsy for diagnosis. The data demonstrate an excellent correlation between p16+/HPV DNA testing, ctHPVDNA, and the volume of the primary tumor and lymph node metastases. This suggests that liquid biopsy could be useful in identifying the subgroup of patients with better oncological outcomes.
**Limitations:** Principal limitation of the study is the number of enrolled patients, but it was statistically appropriate.
**Funding for this study:** Partially supported by LILT 2020-21 Program and MAECI-Call for Joint Project Proposals Italy-Brazil # BR22GR03.
**Ethics committee - additional information:** All enrolled patients signed an informed consent to the protocol approved by the IRCCS Regina Elena National Cancer Institute, Istituti Fisioterapici Ospitalieri, Institu- tional Review Board (RS1647/22).
**Author Disclosures:**
Sergio Ruggiero: Nothing to disclose
Simona Marzi: Nothing to disclose
Sonia Lucchese: Nothing to disclose
Vincenzo Dolcetti: Nothing to disclose
Antonello Vidiri: Nothing to disclose

### The utility of intraoral ultrasonography in differential diagnosis of benign and malignant oral mucosal lesions

*R. Abdalla-Aslan*[1], D. E. Gaitini[1], A. Rchmiel[1], G. Merhav[1], S. Akrish[1], M. Javitt[2], D. Shilo[1], O. Emodi[1], N. Beck-Razi[1]; [1]Haifa/IL, [2]Miami/US
*(ragdaa@gmail.com)*

**Purpose or Learning Objective:** Simple yet reliable methods for differentiating between benign and malignant soft tissue tumours of the oral cavity are currently lacking. Our primary aim was to assess the correlation between pre-operative intraoral ultrasound (US) variables and malignancy of oral lesions.
**Methods or Background:** This is a cross-sectional prospective study of consecutive patients over the age of 18 years from both genders, with a clinical diagnosis of a soft tissue lesion in the oral mucosa. Within a 2-weeks interval, patients who are scheduled for biopsy and histopathological examination underwent high-resolution intraoral US obtained using a 7-15 MHz-L15-7io-linear-ultrasound-transducer-'hockey stick-probe' on a Philips-Epiq-5, 7 machines (Philips Medical, Netherlands). Sonographic variables included: size in 3-dimensions, echogenicity, presence of cystic areas, presence of calcifications, margins and vascularity. The sonographic findings were compared with histopathology.
**Results or Findings:** Full data was available for 52 patients with 53 tumors. Included were 24 females and 28 males, with a mean age 60.11±16.7 years [range 18-90]. Following histopathological results, 22 patients with 23 tumors were diagnosed with squamous cell carcinoma (SCC), 3 patients with dysplasia (1 mild, 1 moderate and 1 severe), and the remaining 26 patients with benign lesions.
Sonographic variables of maximal diameter (a cutoff of 12 mm with 0.76 sensitivity and 0.77 specificity), margins (ill-defined, p<0.001) and vascularity (high or type III/IV, p=0.002) proved to be significantly correlated to SCC group, compared to benign lesions group.
**Conclusion:** The utility of intraoral US in the differential diagnosis of benign and SCC tumors in the oral cavity is demonstrated by this prospective clinical study, using sonographic features of maximal diameter, margins and vascularity.
**Limitations:** Small sample size and operator dependent technique.
**Funding for this study:** None
**Ethics committee - additional information:** Ehics committee of Rambam Health Care Campus reference RMB-19-0596.
**Author Disclosures:**
Adi Rchmiel: Nothing to disclose
Sharon Akrish: Nothing to disclose
Marcia Javitt: Nothing to disclose
Omri Emodi: Nothing to disclose
Goni Merhav: Nothing to disclose
Diana E. Gaitini: Nothing to disclose
Dekel Shilo: Nothing to disclose
Nira Beck-Razi: Nothing to disclose
Ragda Abdalla-Aslan: Nothing to disclose

### Neoadjuvant radiochemotherapy in patients with locally advanced oral-cavity tumour: Response-predictive radiological imaging features

*I. Burck*, A. Gleich, R. Winkelmann, M. Fleischmann, E. Herrmann, J-E. Scholtz, P. Thönissen, T. Vogl, D. Pinto Dos Santos; Frankfurt/DE

**Purpose or Learning Objective:** To explore radiological MR imaging features to predict response to neoadjuvant radiochemotherapy in patients with locally advanced oral-cavity tumour.
**Methods or Background:** We included 30 patients (15 women, mean age 60±10 years) with oral cavity cancer (stage IVa) who underwent neoadjuvant radiochemotherapy (RTX) before surgery. MRI scans were performed before RTX, 15 days after its initiation and preoperatively. Two radiologists retrospectively evaluated the images for overall tumour signal intensity (SI), SI change over time, and tumour extent using a Likert scale. Quantitative analysis was performed for the absolute SI of the tumor in ADC-, DWI-, and T2-weighted sequences normalized to the spinal cord. Tumour volume (TV) was calculated manually in a contrast-enhanced T1 sequence. Differences and ratios of ADC, DWI and T2-SI and TV were calculated. Patients with a stage pT1 or T0 were classified as responders, all others as non-responders.
**Results or Findings:** ADC-SIs at 2nd and 3rd MRI differed significantly between responders and non-responders (p = 0.010 and p = 0.013), as did the ratio between baseline and preoperative DWI-SIs (p = 0.041) and the difference between normalized baseline and preoperative ADC-SIs (p = 0.049). Non-responders showed an increase in TV at the 2nd MRI, while responders showed a significant decrease in TV, so the calculated percentage decrease and ratio are significant markers of response progression.
**Conclusion:** Diffusion weighted imaging parameters as well as tumour volumetry may predict response to neoadjuvant radiochemotherapy in oral cavity cancer and may be benefical for image guided treatment potentially be used to guide treatment or extent of surgery in these patients.
**Limitations:** Limitations of this study include small sample size, single-center and retrospective study design.
**Funding for this study:** None.
**Ethics committee - additional information:** Ethics approval was obtained by the institutional review board (approvals number 208/12).
**Author Disclosures:**
Ria Winkelmann: Nothing to disclose
Thomas Vogl: Nothing to disclose
Jan-Erik Scholtz: Nothing to disclose
Daniel Pinto Dos Santos: Nothing to disclose
Philipp Thönissen: Nothing to disclose
Alexander Gleich: Nothing to disclose
Eva Herrmann: Nothing to disclose
Maximilian Fleischmann: Nothing to disclose
Iris Burck: Nothing to disclose

### Impact of Deep Learning-Based Image Reconstructions in Head and Neck MRI

*F. Albisinni*[1], C. Carbone[1], C. Zacchi[1], M. Ravanelli[1], D. Farina[1], B. Van Deberge[2]; [1]Brescia/IT, [2]Leuven/BE
*(f.albisinni@unibs.it)*

**Purpose or Learning Objective:** This study aimed to assess the impact of deep learning (DL)-based reconstructions on T2 image quality in head and neck MRI. Additionally, potential time savings from using DL in various sequences were evaluated.
**Methods or Background:** Three sequences were compared: (A) TSE T2 without DL, with three signal averages and an acquisition time of 2'35''; (B) TSE T2 with DL at intermediate strength, with two signal averages and an acquisition time of 1'25''; and (C) TSE T2 with DL at maximal strength, with one signal average and acquisition time of 43''. Images from 52 patients were randomly and blindly evaluated by three radiologists with varying levels of experience using MR scanners from different vendors. For each patient, two images were analyzed at the level of the nasopharynx and oral cavity. Four categories were assessed: overall image quality, artifacts, edge sharpness, and noise, each rated on a 3-point Likert scale. Additionally, radiologists were tasked with identifying the correct sequence for each image. The two main endpoints evaluated were inter-rater reproducibility and comparison of the sequences
**Results or Findings:** A total of 636 images were rated by three radiologists. Inter-rated reproducibility was poor across all categories. The sequences type was correctly identified in only 44% cases. The overall quality scores for sequence A, B and C were 2.63, 2.52, and 2.52 respectively (p=0.055). Artifacts scores were 2.61, 2.48, and 2.55 (p=0.07); edge sharpness scores were 2.57, 2.39, and 2.43 (p=0.02, with significant differences between sequences A and B); and noise scores were 2.51, 2.41, and 2.41 (p=0.1)
**Conclusion:** The performances of the three sequences were similar overall. DL-based sequences for head and neck MRI were shown effective and offered significant time savings, enabling potential ultra-fast imaging protocols
**Limitations:** Small sample
**Funding for this study:** No funding was provided for this study

Wednesday

**Ethics committee - additional information:** Not applicable
**Author Disclosures:**
Davide Farina: Nothing to disclose
Baptiste Van Deberge: Nothing to disclose
Flavia Albisinni: Nothing to disclose
Marco Ravanelli: Nothing to disclose
Chiara Zacchi: Nothing to disclose
Carmela Carbone: Nothing to disclose

**10:00-11:00** **Research Stage 2**

### Research Presentation Session: Imaging Informatics and Artificial Intelligence

### RPS 205

### Meta-level topics in AI: cost-effectiveness, non-interpretive use-cases and evidence

**Moderator**
E. Neri; Pisa/IT
*(emanuele.neri@med.unipi.it)*

### Early health technology assessment for an artificial intelligence tool to detect incidental pulmonary embolisms on computed tomography

*E. H. M. Kemper*[1], K. Redekop[1], F. Vos[2], M. Ijzerman[1], M. P. A. Starmans[1], J. J. Visser[1]; [1]Rotterdam/NL, [2]Delft/NL
*(e.h.m.kemper@erasmusmc.nl)*

**Purpose or Learning Objective:** Incidental pulmonary embolisms (IPE) on computed tomography (CT) are missed in up to 70% of cases. While artificial intelligence (AI) tools for IPE detection exist, an evaluation on if and how these tools can provide actual value, e.g., fit patients and end-users needs (i.e., radiologists), have never been performed. The aim of this early health technology assessment (eHTA) is to determine the requirements for a value-based AI tool for IPE detection on CT.
**Methods or Background:** A comprehensive eHTA process for radiology-AI was proposed and conducted for IPE. A literature search, structured interviews, focus group, and evaluation meetings were performed with the identified stakeholders to define criteria and scenarios for a multiple criteria decision analysis (MCDA). A representative survey was developed and circulated to weigh the importance of the criteria and assess performances of four possible AI designs. MCDA analysis on the survey help quantify the value requirements.
**Results or Findings:** Consultations with radiologists, treating physicians, patients, radiology technologists, AI specialists, legal experts, and ethicists resulted in 14 sub-criteria and five main criteria; patient impact, model performance, physician support, environmental impact, and costs. Preliminary outcomes indicate that a short follow-up time for diagnosing IPE is more important than a high sensitivity for IPE detection.
**Conclusion:** A value-based AI tool for IPE detection should be focused on triage to reduce the impact of the diagnosis of IPE on the patient, mainly because delay of diagnosis can result in progression of the IPE and preventable stress for the patient, while an improved detection rate is considered to result in significant overtreatment.
**Limitations:** The scope of this analysis has been within Europe. Outcomes might not be applicable elsewhere.
**Funding for this study:** E.H.M.K., K.R., M.P.A.S., F.V, and J.J.V. acknowledge funding by LSH-TKI (Health~Holland Dutch Top Sector Life Sciences and Health) 23024
**Ethics committee - additional information:** No applicable
**Author Disclosures:**
Maarten Ijzerman: Nothing to disclose
Ken Redekop: Nothing to disclose
Erik Hermanus Marcellinus Kemper: Nothing to disclose
Martijn Pieter Anton Starmans: Nothing to disclose
Jacob Johannes Visser: Advisory Board: Contextflow
Frans Vos: Nothing to disclose

### Potential costs and benefits of AI for fracture detection in cervical spine CT scans at hospital level

*G. Van Den Wittenboer*[1], I. M. Nijholt[1], M. Maas[2], M. F. Boomsma[1]; [1]Zwolle/NL, [2]Amsterdam/NL

**Purpose or Learning Objective:** Aim of this study was to assess healthcare costs at the hospital level for patients screened for cervical spine (CS) fractures using CT, and to estimate costs and benefits of incorporating artificial intelligence (AI) to detect CS fractures in clinical practice.
**Methods or Background:** Diagnostic accuracy of on-duty radiologists and AI in detecting CS fractures on CT scans from a retrospective database (n=2321, ≥18 years, 2007-2014) was compared with a reference standard. Healthcare costs for patients were inventoried up to 7 months after their emergency department visit. Total and average costs per patient based on the radiologist diagnosis were calculated for four categories: true positive, true negative, false positive, and false negative. Finally, a scenario-analysis was conducted to estimate the diagnostic accuracy of radiologists combined with AI, and the corresponding total healthcare costs per diagnostic category.
**Results or Findings:** Radiologists identified 193 out of 219 scans with fractures and 2085 out of 2102 scans without fractures, whereas AI identified 177 out of 219 fractures and 2065 out of 2102 scans without fractures. AI identified 23 fractures missed by the radiologists and correctly classified 16 non-fracture scans that had been misclassified as fractures by the radiologists. This resulted in a potential sensitivity of 216/219 (98%) and specificity of 2101/2102 (>99%) for the combined radiologist-AI approach. On average, €5,978 less was spent per missed fracture. The total cost for the AI-assisted scenario was €61,132 (0.3%) higher than for radiologists alone.
**Conclusion:** In this scenario-analysis, the use of AI appears to increase hospital costs by 0.3% due to more accurate diagnoses. A next step could be to complement these results with non- hospital costs and quality-adjusted life years to further investigate the cost-effectiveness of this AI.
**Limitations:** No limitations were identified.
**Funding for this study:** The radiology department of the Isala received a grant from AIDOC Medical to have a third party (THINC, Utrecht, the Netherlands) that is specialized in early health technology assessments, perform the analyses for this study. AIDOC medical had no role in the data analyses itself. Neither AIDOC Medical nor THINC had a role in data collection or drafting of the abstract.
**Ethics committee - additional information:** The study uses retrospective data.
**Author Disclosures:**
Martijn Franklin Boomsma: Other: The Department of Radiology, Isala, has established a strategic partnership with Aidoc Medical. However, Aidoc Medical had no decisive role in data collection, data analysis nor data interpretation.
Ingrid M. Nijholt: Other: The Department of Radiology, Isala, has established a strategic partnership with Aidoc Medical. However, Aidoc Medical had no decisive role in data collection, data analysis nor data interpretation.
Mario Maas: Nothing to disclose
Gaby Van Den Wittenboer: Other: The Department of Radiology, Isala, has established a strategic partnership with Aidoc Medical. However, Aidoc Medical had no decisive role in data collection, data analysis nor data interpretation.

### Cost-effectiveness of AI-assisted digital mammography – results from a Swedish model-based analysis

*P. Gialias*, J. Lyth, M. Kristoffersen Wiberg, T. Bjerner, M. Husberg, L. Bernfort, H. Gustafsson, L-Å. Levin; Linköping/SE
*(pantelis.gialias@liu.se)*

**Purpose or Learning Objective:** To evaluate the cost-effectiveness of AI-assisted biennial digital mammography (AI-DM) in comparison to conventional digital mammography (cDM) with double reading of screening mammograms (screening interval ages 40-74).
**Methods or Background:** We used a Markov decision analytic model with a life-time horizon. The analysis was conducted from a healthcare perspective. Model parameters were based on Swedish registry data and published randomized AI-DM studies. The model estimates the costs and quality-adjusted life-years (QALYs) related to mammography and breast cancer. Mammography-related costs were collected from the university hospital in Linköping. Stage-specific cancer cost,QALY-weights were obtained from the literature. Scenario analyses were performed with different screening strategies.
**Results or Findings:** Per 1000 individuals AI-DM gained 10.8 QALYs compared to cDM. The costs per 1000 individuals were USD 3,752,278 and USD 3,816,443 for AI-DM and cDM, respectively. AI-DM resulted in a cost saving of USD 64 165 which makes it a dominant strategy. The isolated screening costs were slightly higher in the used AI-DM setting, USD 597, but this was offset by reduced lifetime costs of cancer treatment. A screening strategy with AI plus one radiologist for all examinations saves USD 9128

screening costs compared to cDM, however the QALYs gained were decreased to 8.8.
**Conclusion:** AI-DM is cost saving in our setting and generates more quality-adjusted life-years. One of the add-on benefits is the possibility to free radiological time to other clinical work. These benefits could be further improved by changing the AI-DM triaging strategy.
**Limitations:** We based AI parameters in the model mainly on two Swedish randomized trials and cancer data from the population-based cancer registry from Sweden. However, cost data are highly dependent on the Swedish health care system and the generalizability to other health care systems might be limited.
**Funding for this study:** None
**Ethics committee - additional information:** Not ethics committee approval was need for this study
**Author Disclosures:**
Maria Kristoffersen Wiberg: Nothing to disclose
Magnus Husberg: Nothing to disclose
Tomas Bjerner: Nothing to disclose
Håkan Gustafsson: Nothing to disclose
Johan Lyth: Nothing to disclose
Lars Bernfort: Nothing to disclose
Lars-Åke Levin: Nothing to disclose
Pantelis Gialias: Nothing to disclose

### AI Tools to Reduce Claims and Compensation Payments of Missed Fractures on Radiographs: A Potential Game Changer?

M. Tordjman[1], L. Gracia[1], E. Guillo[1], R. Amar[1], J. Ventre[1], *N-E. Regnard*[2], R. Y. Carlier[1], J-L. Marmorat[1], J-D. Laredo[1]; [1]Paris/FR, [2]Lieusaint/FR
*(noreddine.regnard@gleamer.ai)*

**Purpose or Learning Objective:** To evaluate the potential of BoneView, an AI tool for fracture detection on radiographs, in claims files of missed fractures which led to financial compensation.
**Methods or Background:** This retrospective study included all the files of patients who submitted a claim and had financial compensation for missed fractures on radiographs from January 2013 to December 2019 in the 38 university hospitals of the Greater Paris area Hospitals (APHP, France). Of the 29 patients who claimed files, 26 were finally included (3 were not available in the system). For each patient with a claim, 5 patients with radiographs from the same anatomical areas (with or without fracture) were included from consecutive patients who had radiographs at a university hospital in 2022. Two readers (one fellow in musculoskeletal radiology and one expert radiologist in musculoskeletal imaging with more than 20 years of experience) read the radiographs, blinded from which patients had missed fractures.
**Results or Findings:** 156 patients were included (26 patients with missed fractures and 130 « control » patients). The AI software was able to detect 80.7% of fractures (21/26) for the patients who filed claims for missed fractures. The sensitivity of readers was also improved with AI for these patients: the junior reader had a sensitivity of 61.5% without AI and 69.2% with AI and the expert reader had a sensitivity of 73.1% without AI and 84.6% with AI. The total of potentially avoided financial compensation would have been 265.314 euros.
**Conclusion:** The sensitivity of the two readers is improved with AI in a cohort of patients with missed fractures who submitted claims and had financial compensations. AI was able to detect most of these fractures.
**Limitations:** A limitation was the small number of claims files.
**Funding for this study:** There was no funding for this study.
**Ethics committee - additional information:** Not applicable
**Author Disclosures:**
Raphael Amar: Nothing to disclose
Nor-Eddine Regnard: Founder: Chief Medical Officer of Gleamer
Jean-Luc Marmorat: Nothing to disclose
Mickael Tordjman: Nothing to disclose
Jean-Denis Laredo: Employee: Gleamer
Robert Yves Carlier: Nothing to disclose
Jeanne Ventre: Employee: Gleamer
Enora Guillo: Nothing to disclose
Laure Gracia: Nothing to disclose

### Overlooked and underpowered: a meta-research study addressing sample size in radiomics research

*J. Zhong*[1], J. Lu[2], Y. Xing[1], Y. Hu[1], D. Ding[1], X. Liu[1], S. Dai[1], H. Zhang[1], W. Yao[1]; [1]Shanghai/CN, [2]Stanford, CA/US
*(wal_zjy@163.com)*

**Purpose or Learning Objective:** To investigate how studies determine the sample size when developing radiomics models, and whether it is sufficient.
**Methods or Background:** We identified radiomics studies published from January to December 2023 on seven leading peer-reviewed radiological journals owned by European Society of Radiology and Radiological Society of North America. We reviewed the sample size justification methods, and actual sample size used. We calculated the minimum sample size according to 3 criteria proposed by Riley et al, and compared the estimated and the actual sample size used. We investigated which characteristics factors were associated with the sufficient sample size.
**Results or Findings:** We included 116 studies. 11/116 studies justified the sample size, in which 6/11 performed a priori sample size calculation. The mean ± standard deviation (SD), median (first and third quartile, Q1, Q3) of total sample size of models are 451 ± 871, 223 (130, 463), and those of sample size for training are 292 ± 676, 150 (90, 288). The mean ± SD, median (Q1, Q3) of difference between the total sample size and minimum sample size according to Riley et al criterion 3 are 120 ± 888, -100 (-216, 183), and those of difference between the sample size for training and minimum sample size according to Riley et al all 3 criteria are -386 ± 1264, -268 (-427, -157). The model testing method and specialty of topic were associated with sufficient sample size.
**Conclusion:** Radiomics models are often designed without sample size justification, as a consequence many models are too small to avoid overfitting, noise, and outliers. It should be encouraged to justify, perform and report sample size calculations when developing radiomics models.
**Limitations:** The limitation of the study is limited number of leading peer-reviewed radiological journals.
**Funding for this study:** Funding was provided by National Natural Science Foundation of China (82302183, 82471935, 82271934), Yangfan Project of Science and Technology Commission of Shanghai Municipality (22YF1442400), Research Found of Health Commission of Changing District, Shanghai Municipality (2023QN01), Laboratory Open Fund of Key Technology and Materials in Minimally Invasive Spine Surgery (2024JZWC-ZDA03, 2024JZWC-YBA07), and Research Fund of Tongren Hospital, Shanghai Jiao Tong University School of Medicine (TRKYRC-XX202204, TRYJ2021JC06, TRYXJH18, TRYXJH28).
**Ethics committee - additional information:** The study is a meta-research study with a protocol available on OSF (https://osf.io/pbukc/), and no human participants or animals were included in the study.
**Author Disclosures:**
Defang Ding: Nothing to disclose
Yue Xing: Nothing to disclose
Huan Zhang: Nothing to disclose
Jingyu Zhong: Board Member: Dr. Jingyu Zhong acknowledges his position as a member of the Musculoskeletal section of the Scientific Editorial Board of European Radiology, a member of Scientific Editorial Board of BMC Medical Imaging, and a guest editor of the collection “AI in radiology: revolutionizing medical imaging and interpretation” of BMC Artificial Intelligence.
Junjie Lu: Nothing to disclose
Xianwei Liu: Nothing to disclose
Yangfan Hu: Nothing to disclose
Weiwu Yao: Nothing to disclose
Shun Dai: Nothing to disclose

### Evolution of commercially available artificial intelligence in radiology: a follow-up on peer-reviewed evidence of 179 products

N. Antonissen[1], *I. B. Houben*[2], O. Tryfonos[3], M. De Rooij[1], K. G. Van Leeuwen[4]; [1]Nijmegen/NL, [2]Zwolle/NL, [3]Amsterdam/NL, [4]De Bilt/NL

**Purpose or Learning Objective:** To investigate changes in peer-reviewed evidence on commercially available radiologic artificial intelligence (AI) products from 2020 to 2023.
**Methods or Background:** A comprehensive review of the literature published between January 2015 and March 2023 of CE certified radiological AI products (according to www.healthairegister.com) was performed. Complying with the previous systematic review, this follow-up study categorized the publications according to the hierarchical model of efficacy: from technical and diagnostic accuracy (levels 1 and 2) to impacts on clinical decision-making and patient outcomes (level 3-5) or socio-economic impact (level 6).
**Results or Findings:** By March 2023, 91 vendors were identified, offering a total of 179 products, with 120 of these (67%) having peer-reviewed evidence, compared to 36% in 2020. In 2023, there were 662 publications on these 120 products, compared to 237 publications on 36 products in 2020. An increase (22 to 25%) was found in publications focusing on technical or potential clinical efficacy. The majority of publications described the diagnostic accuracy of the product (level 2), although relatively showing a decrease (55 to 52%). For the higher levels of efficacy (level 3-6) the respective contribution to the total remained the same as 2020 (23%).
**Conclusion:** While there is an increase in the amount of publications validating AI products, the majority of publications continue to describe the lower levels of efficacy. This suggests that even though the field has been maturing, we still have limited knowledge and evidence of the clinical impact of AI products in radiology.
**Limitations:** Several products have a high number of publications, which may cause them to be overrepresented in the total.
**Funding for this study:** No funding was received for this study.

**Ethics committee - additional information:** Not applicable
**Author Disclosures:**
Ignas Bernardus Houben: Nothing to disclose
Kicky Gerhilde Van Leeuwen: Owner: Romion Health Founder: Health AI Register
Noa Antonissen: Nothing to disclose
Olga Tryfonos: Nothing to disclose
Maarten De Rooij: Research/Grant Support: Siemens Healthineers

### Flexible Deep Learning MR Image Enhancement with Performance Monitoring

Z. Zhou, C. Arnold, H. Gandhi, P. Gulaka, A. Shankaranarayanan, *S. Pasumarthi Venkata*; Menlo Park, CA/US
*(srivathsa@subtlemedical.com)*

**Purpose or Learning Objective:** Deep learning (DL) MR image enhancement allows scan time reduction while maintaining the diagnostic quality. However, its performance may deteriorate over time. This study aims to develop an adaptive image enhancement DL model and investigates a non-reference-based metric without human annotation for performance monitoring.
**Methods or Background:** A single DL model with a ConvNeXt backbone was trained on 3027 paired MR data. High-quality images were enhanced by a commercial algorithm as targets. Low-quality input images were acquired with various acceleration methods (0-80%) for model to learn adaptive enhancement. The trained DL model was evaluated on another diverse set of 205 cases. Line profiles and region-of-interests (ROIs) were manually labeled for each case. The slope/gradient was extracted from line profiles to measure image sharpness, and signal-to-noise ratio (SNR) was derived from ROIs to evaluate noise level. In addition, gradient entropy (GE) as a non-reference-based metric (lower GE higher quality) was compared with line/ROI based metrics.
**Results or Findings:** Compared to inputs, over 90% of model outputs achieved 45% SNR increase and 8% sharpness increase. On average, SNR and sharpness were improved by 73% and 27%, respectively. GE measured on outputs was reduced by 0.5% for 95% of test cases. For test cases with >0.5% GE reduction, the Pearson correlation of the relative change between GE and SNR is -0.333 ($p < 0.05$), and between GE and sharpness is 0.214 ($p < 0.05$), showing a weak but significant correlation between GE and annotated image quality (IQ) metrics.
**Conclusion:** The developed DL model can adaptively improve IQ supporting flexible protocol acceleration. Its strong denoising also enables MR scans with higher acceleration/resolution. In addition, gradient entropy can be simply deployed for performance monitoring and mitigate the risk of mis-interpretation.
**Limitations:** Not applicable
**Funding for this study:** NIH SBIR grant (R44MH135725)
**Ethics committee - additional information:** Not applicable
**Author Disclosures:**
Srivathsa Pasumarthi Venkata: Employee: Subtle Medical Inc
Campbell Arnold: Employee: Subtle Medical Inc
Praveen Gulaka: Employee: Subtle Medical Inc
Ajit Shankaranarayanan: Employee: Subtle Medical Inc
Harsh Gandhi: Employee: Subtle Medical Inc
Zechen Zhou: Employee: Subtle Medical Inc

### Radiologist-Guided Active Learning for Medical Image Segmentation: Moving Beyond the Dice Score to Clinically Relevant Targets

*B. Föllmer*, V. Serafimoski, K. Schulze, F. Biavati, M. Bosserdt, M. Dewey; Berlin/DE
*(bernhard.foellmer@charite.de)*

**Purpose or Learning Objective:** Deep learning models for medical image segmentation typically require extensive pixel-wise annotations, which are costly and time-consuming. Active learning can mitigate this challenge by labeling only the most informative (i.e., uncertain) cases in multiple annotation and training rounds. However, conventional active learning methods do not account for clinically relevant segmentation targets. This study introduces a radiologist-in-the-loop approach for targeted active learning, to optimize model performance beyond standard metrics like the Dice score, focusing on clinically significant segmentation objectives.
**Methods or Background:** We propose a targeted active learning framework consisting of four iterative steps: (1) Automated identification of uncertain cases for review by the radiologist, (2) Radiologist selection of cases relevant to predefined clinical segmentation targets, (3) Combined selection of uncertain and clinically relevant cases, and (4) Efficient partial annotation and model retraining. We applied this approach to multi-class segmentation of coronary arteries using the SCCT 18-segment model, evaluating it on CTAs from 300 patients of the DISCHARGE (NCT02400229) and CAD-Man trials. Initial model training was conducted using standard active learning, followed by targeted active learning with three predefined objectives: (1) segmentation of rare vessels (e.g., Ramus Intermedius), (2) segmentation of thin vessels (e.g., R-PDA, R-PLB), and (3) segmentation of heavily calcified segments.
**Results or Findings:** Our framework demonstrated improved segmentation performance and time-efficiency over standard active learning for the three predefined targets (rare vessels, thin vessels, and calcified segments.
**Conclusion:** The proposed targeted active learning framework enables more time-efficient, radiologist-guided model training focused on clinically relevant segmentation targets, improving performance beyond conventional accuracy metrics like the Dice score.
**Limitations:** This framework was evaluated exclusively on coronary artery segmentation in cardiac CT, with only three segmentation targets considered. Broader validation is needed for other anatomical structures and imaging modalities.
**Funding for this study:** This work was funded by the German Research Foundation through the graduate program BIOQIC (GRK2260, project-ID: 289347353) and the DISCHARGE project (603266-2, HEALTH-2012.2.4.-2) funded by the FP7 Program of the European Commission.
**Ethics committee - additional information:** This study does not require any approval of the ethics committee.
**Author Disclosures:**
Kenrick Schulze: Nothing to disclose
Marc Dewey: Other: Hands-on cardiac CT courses (www.ct-kurs.de) Other: European Society of Radiology (ESR) Publications Chair (2022-2025); the opinions expressed in this abstract/presentation are the author’s own and do not represent the view of ESR Patent Holder: Patent on fractal analysis of perfusion imaging (jointly with Florian Michallek, EPO 2022 EP3350773A1, and USPTO 2021 10,991,109, approved) Author: Cardiac CT (Springer Nature) Equipment Support Recipient: Siemens, General Electric, Philips, Canon Grant Recipient: EU (EC-GA 603266 in HEALTH.2013.2.4.2-2) DFG (DE 1361/14-1, DE 1361/18-1, BIOQIC GRK 2260/1, Radiomics DE 1361/19-1 [428222922] and 20-1 [428223139] in SPP 2177/1), GUIDE-IT (DE 1361/24-1), Berlin University Alliance (GC_SC_PC 27), Berlin Institute of Health (Digital Health Accelerator).
Federico Biavati: Nothing to disclose
Maria Bosserdt: Research/Grant Support: Received funding from EU-FP7 Framework Program (DISCHARGE EU FP EC-GA 603266).
Vladimir Serafimoski: Nothing to disclose
Bernhard Föllmer: Nothing to disclose

**10:00-11:00** **Research Stage 3**

### Research Presentation Session: Vascular

### RPS 215

### Advances in peripheral imaging

**Moderator**
E. Claus; Leuven/BE

### A Novel Human Amputated Limb Model for Advancing Peripheral Artery Disease Research and Device Testing

*J. Csőre*, A. Crichton, B. Benfor, C. Karmonik, T. L. Roy; Houston, TX/US
*(csore.judit@gmail.com)*

**Purpose or Learning Objective:** Traditional animal models often fail to capture the complexity of peripheral artery disease (PAD) lesions, leading to a gap between preclinical and clinical research in percutaneous vascular interventions (PVI). To address this, we developed a human amputated limb model combined with a proprietary MRI-histology protocol for detailed plaque characterization and simulation of PVI procedures, assessing lesion-specific device impact on the vessel wall.
**Methods or Background:** Amputated limbs from end-stage PAD patients were scanned using 3T or 7T MRI, incorporating Ultrashort Echo Time and T2-weighted sequences to differentiate hard (collagen/calcium) and soft (fat/thrombus/smooth muscle) plaque components. PVI procedures were simulated in a hybrid operating room, targeting identified lesions. Device testing included balloon angioplasty, lithotripsy, atherectomy, drug-coated balloons, and novel wires/catheters. Vessel impact was evaluated intraprocedurally using intravascular ultrasound, followed by post-procedure micro-CT and 9.4T MRI. Histopathological analysis was performed with Movat’s and H&E stains.
**Results or Findings:** A total of 70 amputated limbs were collected, yielding 133 target lesions and 2500 histologic cross-sections. Key findings include: 1. Validation of the MRI protocol and human amputated limb model. 2. Successful testing of vessel preparation devices, showing plaque disruption and dissection in calcified lesions. 3. Correlation of chronic total occlusion crossing success with pre-intervention MRI histology scoring. 4. Identification of calcified lesions as barriers to effective drug delivery. 5. Collaboration with industry for device development and testing.
**Conclusion:** This human cadaveric model offers a unique platform for PAD research, providing detailed insights into plaque morphology and PVI device performance. By correlating plaque characteristics with procedural outcomes, it enables precise device testing and fosters innovation in vascular interventions. This model informs clinical decision-making, enhances new technology design, and guides personalized treatment strategies for PAD patients.
**Limitations:** Single-center study, small cohort
**Funding for this study:** Jerold B. Katz Academy of Translational Science (project ID 15790002, recipient: Trisha Roy); American Heart Association Transformational Award (project ID: 17590004, recipient: Trisha Roy); National Institutes of Health Research Project grant (R01) (award ID: R01HL174587, recipient: Trisha Roy)
**Ethics committee - additional information:** This study was approved by the Institutional Review Board under study ID PRO00027258.
**Author Disclosures:**
Alexander Crichton: Nothing to disclose
Bright Benfor: Nothing to disclose
Judit Csőre: Nothing to disclose
Christof Karmonik: Nothing to disclose
Trisha L. Roy: Research/Grant Support: Baylis Medical Technologies, Boston Scientific, Light Matter Interaction Founder: Magellan Biomedical Inc.

**Application and significance of precise CTA scanning technology in the assessment of lower extremity arterial diseases**
*J. Xing*, H. Yu, L. Zhu; Shang Hai/CN
*(13636300561@139.com)*

**Purpose or Learning Objective:** Objective: This study seeks to investigate the differences in image quality and radiation exposure between an advanced precision scanning technique and traditional scanning methods in 320-slice computed tomography angiography (CTA) of the lower limb
**Methods or Background:** Methods: A cohort of 89 patients with suspected lower limb a arteryial disease, who underwent CTA examination at our institution, were randomly allocated to either group A or group B. In group A,low-dose testing was first used. TS was obtained by subtracting the time to peak of the dorsalis pedis artery T2 and the time to peak of the main abdominal artery T1;the formal scan began at T1 + 4 seconds,and the scan was completed after adjusting the entire acquisition time to TS by the variable pitch method.Group B was scanned using standard pitch.The statistical analysis involved the assessment of image quality,radiation dose, and contrast agent dosage.
**Results or Findings:** Results:Both subjective and objective evaluations demonstrated superior image quality of lower extremity artery in group A(all P<0.001).The subjective score for group A demonstrated a significant 21% increase compared to that of group B, particularly in the assessment of ankle and dorsum images (4.32±0.79 vs. 3.57±0.94).In terms of patients' radiation dose and contrast agent dosage, group A exhibited a 16.23% reduction in radiation dose and a 12.28% reduction in contrast agent dosage compared to group B, respectively (both P< 0.001).
**Conclusion: Conclusion:** The implementation of VHP technology in lower extremity artery CTA scanning facilitates enhanced visualization of distal blood vessels and improves overall image quality, meanwhile effectively reducing radiation exposure and contrast agent consumption, which presents substantial clinical value.
**Limitations:** The sample size is relatively small and warrants expansion for further validation of the derived conclusions.
**Funding for this study:** Young Scientists Fund of the National Natural Science Foundation of China (82302188)
**Ethics committee - additional information:** No:2019tjdx123
**Author Disclosures:**
Jun Xing: Nothing to disclose
Hong Yu: Nothing to disclose
Lin Zhu: Nothing to disclose

**Run-off CT angiography with a patient-tailored post-trigger delay: Optimized scan timing compared with a fixed delay**
*K. Qi*, J. Liu; Zhengzhou/CN
*(qk_kkkkkkk@163.com)*

**Purpose or Learning Objective:** To validate the feasibility of using bolus tracking with a patient-tailored post-trigger delay (PTD) in run-off CTA and to compare image quality with that using a fixed PTD.
**Methods or Background:** Participants undergoing run-off CTA with bolus tracking were prospectively assigned at random, cohort A comprised 30 participants with a fixed 10-second PTD and cohort B comprised 30 participants with a patient-tailored PTD. The attenuation of abdominal and lower limb arteries was measured in 11 different anatomical positions in one leg and divided into four vascular segments according to the anatomical location: aortoiliac, femoropopliteal, tibioperoneal and foot. The mean attenuation, SNR and CNR of each vessel segment were calculated. Two readers rated subjective image quality. Two-way analysis of variance was used to assess the mean attenuation of four vascular segments. Sidak's multiple comparison was used to determine differences in attenuation between the two cohorts and at each anatomical location within each cohort. Mann-Whitney test was used to determine SNR and CNR between two groups, while the chi-square test compared subjective image quality scores.
**Results or Findings:** Cohort B using new bolus tracking algorithm predicts the mean patient-tailored PTD of 12 ± 1.8 s. The demographic and frequency of PAD revealed no statistically significant differences. Cohort B showed greater attenuation of tibioperoneal (432±76 HU vs 364±69HU, p=0.001) and foot (369±79 HU vs 281±77HU, p=0.001) segments. SNR (p<0.002), CNR(p<0.002) and subjective image quality (excellent or good image quality, 96.7% vs 74.3%, p=0.038) were higher in cohort B than in the fixed cohort.
**Conclusion:** Bolus tracking with a patient-tailored PTD provides reliable scan timing, resulting in improved image quality and optimized vessel opacification in run-off CTA .
**Limitations:** Further research is needed on the relationship between frequency of PAD and PTD.
**Funding for this study:** The Science and Technology Research Project of Henan Provincial Health Commission (No. 212102310142)
**Ethics committee - additional information:** Ethics Committee of Zhengzhou University
**Author Disclosures:**
Jie Liu: Nothing to disclose
Ke Qi: Nothing to disclose

**Shear Wave Elastography in Differentiating Acute and Subacute Thrombosis of Dialysis Arteriovenous Fistulas**
*Ö. Altun*, A. Dablan, M. Sam Özdemir, M. Karagülle, M. Cingöz, M. F. Arslan; Istanbul/TR
*(omeraltun1996@gmail.com)*

**Purpose or Learning Objective:** To evaluate the utility of shear wave elastography (SWE) in distinguishing between acute and subacute thrombi in thrombosed dialysis arteriovenous fistulas (AVFs).
**Methods or Background:** This retrospective study analyzed 32 dialysis patients with thrombosed AVFs treated between June 2022 and June 2024. All patients underwent Doppler ultrasound and SWE to determine thrombus characteristics. Based on ultrasound findings and clinical history, patients were categorized into acute or subacute thrombus groups. Thrombus stiffness was quantified using SWE in terms of average, median, and maximum kilopascal (kPa) values.
**Results or Findings:** The study included 16 patients with acute thrombi and 16 with subacute thrombi. SWE measurements revealed significantly higher stiffness values in subacute thrombi compared to acute thrombi (p < 0.001). A strong positive correlation was observed between thrombus age and SWE-derived kPa values (average: r = 0.770, median: r = 0.727, maximum: r = 0.835). Receiver operating characteristic (ROC) analysis demonstrated SWE's high accuracy in differentiating thrombus age, with an optimal average cut-off value of 31.7 kPa, resulting in a sensitivity of 90.5% and specificity of 73.9%.
**Conclusion:** Shear wave elastography shows promise as a non-invasive tool for differentiating between acute and subacute thrombi in thrombosed AVFs, aiding in personalized treatment planning for dialysis patients.
**Limitations:** This study's patient numbers was not much.
**Funding for this study:** This study was not supported by any funding.
**Ethics committee - additional information:** All procedures performed in studies involving human participants were in accordance with the ethical standarts of the instituional and/or national research commitee and with the 1964 Helsinki declaration and its later amendments or comparable ethical standarts.
**Author Disclosures:**
Merve Sam Özdemir: Nothing to disclose
Mehmet Karagülle: Nothing to disclose
Mustafa Fatih Arslan: Nothing to disclose
Ömer Altun: Nothing to disclose
Mehmet Cingöz: Nothing to disclose
Ali Dablan: Nothing to disclose

**MRI-Histology as a Predictive Tool for Crossing Failure in Below-the-Knee Peripheral Arterial Disease**
*J. Csőre*, A. Crichton, C. Karmonik, B. Benfor, T. L. Roy; Houston, TX/US
*(csore.judit@gmail.com)*

**Purpose or Learning Objective:** Recent randomized trials have challenged the traditional endovascular-first approach for treating below-the-knee arterial disease. Scoring systems like TASC and GLASS overlook lesion composition and morphology, which influence peripheral vascular intervention (PVI)

success. Conventional imaging provides limited plaque composition insight, while ultrashort echo time (UTE) MRI can distinguish between soft (e.g., fibrous tissue, thrombus) and hard (e.g., calcification, dense collagen) plaque components. This study aimed to assess if MRI-histology could better predict lesion crossing failure compared to GLASS and TASC scoring.
**Methods or Background:** Amputated limbs were collected from patients with chronic limb-threatening ischemia (CLTI) and scanned ex-vivo on a 3T MRI using UTE and T2w contrasts. Lesions were classified as 'hard' if >50% of the lumen was occluded by calcium or dense collagen based on the MRI. The distribution of hard components (eccentric, concentric, central), lumen stenosis caused by hard/soft components, and collagen density were recorded. Ex-vivo PVIs were carried out in a hybrid operating room and TASC and GLASS scoring was performed.
**Results or Findings:** Seventeen patients yielded 29 target lesions, 76% (22/29) of which were classified as 'hard.' Of these, 45% had a collagen-dominated composition. 'Hard' lesions showed a significantly higher crossing failure rate compared to 'soft' lesions (95% vs. 14%, p<.001). MRI scoring of 'hard' lesions was strongly associated with crossing success (p<.001), outperforming TASC and GLASS scoring (p=.062 and p=.112, respectively). Total vessel occlusion was not predictive of failure (p=0.64). Most crossing failures (64%) occurred in centrally distributed 'hard' lesions, though this was not significant.
**Conclusion:** This MRI-histology scoring system identifies plaque composition as a predictor of PVI failure, outperforming TASC and GLASS scoring, and highlighting MRI's potential role in preoperative assessment and device selection for CLTI patients.
**Limitations:** Single-center study, small cohort
**Funding for this study:** Jerold B. Katz Academy of Translational Science (project ID 15790002, recipient: Trisha Roy); American Heart Association Transformational Award (project ID: 17590004, recipient: Trisha Roy); National Institutes of Health Research Project grant (R01) (award ID: R01HL174587, recipient: Trisha Roy)
**Ethics committee - additional information:** This study was approved by the Institutional Review Board under study ID PRO00027258.
**Author Disclosures:**
Alexander Crichton: Nothing to disclose
Bright Benfor: Nothing to disclose
Judit Csőre: Nothing to disclose
Christof Karmonik: Nothing to disclose
Trisha L. Roy: Nothing to disclose

### Assessment of left renal vein areas ratios on CT-phlebography as surrogate parameter for pressure gradient in pelvic congestion syndrome

*T. Nemirovskaya*, R. Bredikhin, R. Akhmetzyanov, E. Fomina, D. Ryabinina, A. Yaglova; Kazan/RU
*(tanya.nemirovsky@gmail.com)*

**Purpose or Learning Objective:** Pelvic venous diseases are considered widespread problem. Among etiological factors compression of left renal vein (LRV) between aorta and superior mesenteric artery (SMA), so-called nutcracker syndrome, is considered primary for symptoms evolvement. Diagnostic workflow includes selective phlebography, but CT-phlebography is gaining popularity. Primary issue is applicability of CT-phlebography results concerning surgical correction selection. Study objective was parameters assessment that could be accounted surrogate characteristics of pressure gradient in the left renal vein.
**Methods or Background:** Prospective assessment of CT-phlebography in patients with pelvic congestion syndrome was performed. Expiratory CT scanning was performed with 130 s delay after Iodinated contrast medium administration. Following measurements were performed: maximum diameter of pelvic veins, gonadal veins diameter, aorta/SMA angle. Three LRV areas were outlined orthogonally projected to gonadal tributary, aorta/SMA angle, inferior vena cava conflux (IVC) with areas ratio calculation. LRV narrowing extension was also measured.
**Results or Findings:** 74 patients underwent IVC and tributaries delayed CT-angiography from 2022 to 2024. Mean age 37 years, 15 male, 59 female. All had pelvic veins enlargement with associated symptoms according to ultrasound examination and history. No severe development anomalies was observed except 3 cases of retroaortal LRV. Twelve cases were accompanied with May-Turner variant. Lineal regression assessment displayed correlation between aorta/SMA angle and maximum/minimum LRV areas ratio with p < 0.001, also with LRV narrowing extension (p = 0.003). May-Turner variant was contributing factor with more severe pelvic veins enlargement (p < 0.001). Correlation was found with pressure gradient according to direct phlebography, however only 19 patients underwent it.
**Conclusion:** CT-phlebography may be considered as supportive diagnostic modality to direct phlebography for selection candidates for surgical correction of pelvic congestion syndrome with LRV compression.
**Limitations:** Retrospective direct phlebography data collection with incomplete cohort coverage.
**Funding for this study:** No funding
**Ethics committee - additional information:** Institutional ethics board of Interregional Clinical Diagnostic Center
**Author Disclosures:**
Rustem Akhmetzyanov: Nothing to disclose
Alina Yaglova: Nothing to disclose
Daria Ryabinina: Nothing to disclose
Elena Fomina: Nothing to disclose
Tatiana Nemirovskaya: Nothing to disclose
Roman Bredikhin: Nothing to disclose

### Evaluation of low-dose upper extremity CTA with artificial intelligence iterative reconstruction for hemodialysis arteriovenous fistula/graft: Image quality and diagnostic value of stenosis detection

*B. Shou*[1], J. Li[2], Y. Zou[2], W. Zhang[1], G. Zhang[2], X. Hu[1], F. Jiang[1], H. Hu[1]; [1]Hangzhou, Zhejiang/CN, [2]Shanghai/CN
*(Y217180070@zju.edu.cn)*

**Purpose or Learning Objective:** To assess the image quality and diagnostic value of artificial intelligence iterative reconstruction (AIIR) in low-dose upper extremity CT angiography for hemodialysis arteriovenous fistula or graft (AVF/G).
**Methods or Background:** A total of 56 patients with suspected or known AVF/G dysfunction were prospectively enrolled and were randomly divided into two groups: routine-dose group (RD-group, n=28) and low-dose group (LD-group, n=28). RD-group employed a routine CTA protocol (tube voltage: 100kVp; contrast dosage: 1.0ml/kg) with hybrid iterative reconstruction, while LD-group used the low-dose protocol (tube voltage: 80kVp; contrast dosage: 0.6ml/kg) with AIIR. Two radiologists independently scored the overall image quality using a 4-point scale (1=poor; 4=excellent). Area under the curve (AUC), accuracy, sensitivity, and specificity of two groups for detecting significant (>50%) stenosis were calculated on a pre-segment basis, using digital subtraction angiography (DSA) as the reference standard. Signal-to-noise ratio (SNR) and contrast-to-noise ratio (CNR) in fistula were also analyzed.
**Results or Findings:** No significant differences in demographics characteristic were observed between the two groups (all p>0.05). The radiation dose and contrast dosage in LD-group were reduced by 53% (224.56mGyxcm vs. 479.24mGyxcm) and 42% (36mL vs. 63mL), respectively, compared to the RD-group. The mean subjective scores between the RD-group and LD-group showed no significant difference (3.86±0.36 vs. 3.68±0.48, p=0.12). The AUC, accuracy, sensitivity, and specificity were 0.91, 90% (47/52 segments), 92%, and 99% for RD-group and were 0.94, 90% (47/52 segments), 100%, and 98% on a pre-segment basis for LD-group. In fistula, SNR and CNR of LD-group were 130% and 140% higher than those of RD-group, respectively (both p<0.001).
**Conclusion:** Low-dose CTA with AIIR provides superior image quality and maintains high accuracy for detecting stenosis in AVF/G, while significantly reducing radiation dose and contrast dosage.
**Limitations:** N/A
**Funding for this study:** N/A
**Ethics committee - additional information:** This study was approved by the local ethics Committee at the University Hospital.
**Author Disclosures:**
Yixuan Zou: Nothing to disclose
Xi Hu: Nothing to disclose
Hongjie Hu: Nothing to disclose
Guozhi Zhang: Nothing to disclose
Beili Shou: Nothing to disclose
Feng Jiang: Nothing to disclose
Wenming Zhang: Nothing to disclose
Jing Li: Nothing to disclose

**10:00-11:00** **Research Stage 4**

### Research Presentation Session: Abdominal and Gastrointestinal

### RPS 201

### What's new in biliary diseases?

**Moderator**
J.-H. Yoon; Seoul/KR
*(jhjhry@gmail.com)*

### Multicenter validation of the DiStrict score, a novel classification and prognostic score for individuals with primary sclerosing cholangitis (PSC)

*A. Grigoriadis*[1], S. G. Hamma[1], G. Kemmerich[2], J. S. Nayagam[3], K. Horsthuis[4], M-C. Londoño[5], D. Assis[6], S. Charanjeet[7], A. Bergquist[1]; [1]Stockholm/SE, [2]Oslo/NO, [3]London/UK, [4]Amsterdam/NL, [5]Barcelona/ES, [6]Connecticut, CT/US, [7]New Haven, CT/US
*(aristeidis.grigoriadis@ki.se)*

**Purpose or Learning Objective:** To validate the reproducibility and prognostic value of the DiStrict-score in a multicenter international cohort.
**Methods or Background:** The DiStrict-score is an MRCP-based classification of the severity of ductal changes (ranging from 1 to 8) and is reproducible and associated with liver-transplantation and liver-related death. However, it has not been validated. For this retrospective multicenter study with participation of eight international PSC-centers, hepatologists from each center retrieved data of consecutive adult PSC individuals (MRCP, demographics, liver-tests, PSC diagnosis date, hepatobiliary cancer development, liver- transplantation, death, and cause of death). Two radiologists from each center applied the DiStrict-score independently to the patients of their center. Cases of disagreement were resolved in consensus. Interreader agreement was assessed for each pair of radiologists with the intraclass correlation coefficient (ICC), with a two-way random-effects model, absolute-agreement, and single-measurement. The association of the DiStrict-score with outcomes (transplant-free survival, development of hepatobiliary malignancy) was assessed with Cox-regression. Survival estimates were calculated with Kaplan-Meier curves and the curves were compared with the log-rank test.
**Results or Findings:** 415 patients (271 males, 248 with ulcerative colitis) with median diagnosis age of 39 years, were included. During a median follow-up of 84 months 101 patients developed outcomes (liver-transplantation; n=78, liver-related death; n=10, hepatobiliary cancer; n=13). The interreader agreement ranged between 0.61 and 0.91 for the different centers. Patients with high DiStrict-scores (5–8) had a higher risk of developing outcomes compared to patients with low scores (1–4) (log-rank test; p=0.0008) with a hazard ratio of 1,98 (95%CI; 1.32-2.96, p=0.001).
**Conclusion:** The DiStrict-sore is reproducible with good interreader agreement and is associated with transplant-free survival and development of hepatobiliary malignancy.
**Limitations:** The limitations of the study are its retrospective design and the non-standardized MRCP acquisition technique.
**Funding for this study:** Funding was provided by Medical Diagnostics Karolinska.
**Ethics committee - additional information:** Each center obtained ethical approval by local ethical committees .
**Author Disclosures:**
Singh Charanjeet: Nothing to disclose
Jeremy Shanika Nayagam: Nothing to disclose
Aristeidis Grigoriadis: Speaker: Have received institutional honoraria by JANSSEN-CILAG AB
Annika Bergquist: Nothing to disclose
Gunter Kemmerich: Nothing to disclose
Karin Horsthuis: Nothing to disclose
Stefan Gmail Hamma: Nothing to disclose
David Assis: Nothing to disclose
María-Carlota Londoño: Nothing to disclose

### Prevalence, prognostic value, and interreader agreement of high-grade strictures in individuals with primary sclerosing cholangitis (PSC)

*A. Grigoriadis*, S. G. Hamma, A. Bergquist; Stockholm/SE
*(aristeidis.grigoriadis@ki.se)*

**Purpose or Learning Objective:** To assess the prevalence and reproducibility of the evaluation of the presence of high-grade strictures (HGS) in MRCP, in PSC individuals. Moreover, to assess the predictive value of HGS for development of hepatobiliary malignancy, liver-transplantation, and liver-related death.
**Methods or Background:** AASLD and EASL have introduced in their guidelines for PSC the term HGS defined as a stricture seen in MRCP with >75% reduction of the lumen of common and/or biliary ducts. However, the prevalence, reproducibility of their detection, and their potential value for predicting outcomes have not been assessed. Two radiologists independently assessed the presence of HGS in MRCPs of 203 individuals with PSC recruited at Karolinska University Hospital in the SUPRIM study between 2012 and 2015. MRCP, demographic, clinical-laboratory and outcome data (hepatobiliary malignancy, liver-transplantation, and liver-related death) were retrieved for all patients. Interreader agreement of the evaluation of HGS was calculated with the intraclass correlation coefficient (ICC) using a two-way random-effects model, single-measurement, and absolute-agreement. The association of HGS with outcomes was assessed with Cox-regression. Survival estimates were calculated with Kaplan-Meier curves and the curves were compared with the log-rank test.
**Results or Findings:** After exclusion, 171 patients (103 males, 95 with ulcerative colitis) with a median diagnosis age of 40 years were included. During a median follow-up of 124 months 49 patients developed outcomes (liver-transplantation=36, liver-related death=5, hepatobiliary malignancy=8). 80 patients (47%) had HGS. The agreement was moderate with ICC=0.72 (95%CI; 0.64-0.78). Patients with HGS had a higher risk to develop outcomes (p=0.01) with a hazard-ratio of 2.08 (95%CI; 1.17-3.71).
**Conclusion:** HGS are common, can be identified with acceptable reproducibility, and are associated with outcomes.
**Limitations:** The limitations of the study are its retrospective design and that no intrareader agreement analysis was performed.
**Funding for this study:** Funding was provided by Medical Diagnostics Karolinska.
**Ethics committee - additional information:** The study was approved by the Swedish ethical review authority (2011/824-31/2, 2018/1111-32, 2018/1494-31/3).
**Author Disclosures:**
Aristeidis Grigoriadis: Speaker: Received Institutional honoraria from JANSSEN-CILAG AB
Annika Bergquist: Nothing to disclose
Stefan Gmail Hamma: Nothing to disclose

### Deep Learning of Preoperative Gadoxetic Acid-Enhanced MRI for Prediction of Perineural Invasion in Intrahepatic Cholangiocarcinoma

*X. Zhou*[1], J. Hu[2], S-T. Feng[1]; [1]Guangzhou/CN, [2]Beijing/CN

**Purpose or Learning Objective:** To preoperatively predict the Perineural invasion (PNI) in intrahepatic cholangiocarcinoma (ICC) on gadoxetic acid (EOB)-enhanced MRI, the deep learning with clinical model based fusion model was developed and evaluated.
**Methods or Background:** A total of 165 patients with pathologically diagnosed ICC who underwent preoperative EOB-enhanced MRI were retrospectively recruited from two independent centers (center1, training set, n = 115; validation set, n = 14; internal test set, n = 15; center 2, external test set, n = 21). The medmanba was used to extract image features on the pre-contrast, arterial phase, portal venous phase, and hepatobiliary phase of MRI. These features combined with clinical factors (such as Neutrophils, lymphocytes, and serum tumor markers), and classified by a linear layer. For comparison, a DL model was constructed by removing clinical factors, and a clinical model was established by the random forest selection on the clinical features. Model performance was evaluated with the area under the receiver operating characteristic curve (AUC). Gradient-weighted class activation mapping (Grad-CAM) heatmaps were used to show the focus area in predicting PNI.
**Results or Findings:** The PNI positive rate was 42.4% (61/144) in center 1 and 28.6% (6/21) in center 2. On the internal test and external test set, the combined model showed the highest AUC of 0.944 and 0.844. The DL model achieved the moderate AUC of 0.926 and 0.833. The performance of clinical model is relatively low, with AUCs of 0.852 and 0.711. Grad-CAM showed the DL model focused on the solid component of the tumor, especially the margin area.
**Conclusion:** MRI based DL model can accurately predict PNI-positive ICC, and the tumor margin area may have important indications for the model.
**Limitations:** Retrospective study; limited sample size.
**Funding for this study:** National Natural Science Foundation of China (82271958)
**Ethics committee - additional information:** The Institutional Review Board of The First Affiliated Hospital, Sun Yat-sen University(approval number: [2023]014)
**Author Disclosures:**
Shi-Ting Feng: Nothing to disclose
Jing Hu: Nothing to disclose
Xiaoqi Zhou: Nothing to disclose

Wednesday

**Preoperative prediction of IDH1-mutation and perineural invasion in intrahepatic cholangiocarcinoma based on Gd-EOB-DTPA-enhanced MRI and MRI-derived habitats**

X. Zhou, M. Chen, *S-T. Feng*; Guangzhou/CN
*(fengsht@mail.sysu.edu.cn)*

**Purpose or Learning Objective:** To preoperatively predict isocitrate dehydrogenase 1 (IDH1) mutation and perineural invasion (PNI) of intrahepatic cholangiocarcinoma (ICC) based on the Gd-EOB-DTPA-enhanced MRI and MRI-derived habitat imaging to improve the reliability and interpretability.
**Methods or Background:** A total of 129 ICC patients with Gd-EOB-DTPA-enhanced MRI before resection between 2018 and 2024 were collected, and randomly assigned to training set and the test set in a ratio of 7:3. IDH1 mutation and PNI status were assessed on pathologic tissue slides. Clinical information and MRI features were qualitatively and quantitatively collected. Matchable tumors in the pre- and post-enhancement T1 mapping images were manually outlined for habitat analysis and divided into five habitats based on kmeans clustering (Habitat 1-5). A combined nomogram model was constructed based on clinical features, MRI features and habitat fraction. The diagnostic accuracy was evaluated using the area under the receiver operating characteristic curves (AUCs).
**Results or Findings:** The IDH1 nomogram model consists of age, T2 central brightness, liver ADC value, tumor T1 value reduction rate and percent of Habitat 4, with AUCs of 0.926 and 0.924 in the training and validation sets. The PNI nomogram model consists of CEA, tumor location, intrahepatic bile duct dilation and percent of Habitat 1, with AUCs of 0.854 and 0.896 in the training and validation sets. By mapping the habitats to multi-sequence MRI, Habitat1 is located predominantly at the edge of the tumor, with signals suggestive of a parenchymal component representing the aggressive edge of the tumour. Habitat 4 is located intratumorally, with signals suggestive of an intratumoral fibrotic area with little tumor component.
**Conclusion:** MRI and habitat imaging can noninvasively and preoperatively determining the IDH1 mutation and PNI of ICC with good accuracy and interpretability.
**Limitations:** Retrospective study, limited sample size.
**Funding for this study:** National Natural Science Foundation of China (82271958)
**Ethics committee - additional information:** The Institutional Review Board of The First Affiliated Hospital, Sun Yat-sen University(approval number: [2023]014)
**Author Disclosures:**
Shi-Ting Feng: Nothing to disclose
Xiaoqi Zhou: Nothing to disclose
Meicheng Chen: Nothing to disclose

**Distinguishing Bile Sludge from Physiological Bile Concentration on Abdominal MRI: Key MRI Features and Diagnostic Accuracy**

*K. Kadirli*, A. Usta, S. Sahin, A. Cantürk, S. Özkan, H. Mutlu; Istanbul/TR
*(kenankadirli7@gmail.com)*

**Purpose or Learning Objective:** Routine abdominal MRI often shows signal changes in the gallbladder on T1- and T2-weighted images, aside from gallstones. Differentiating between bile sludge and physiological bile concentration is key for accurate diagnosis, but literature shows overlapping signal characteristics with limited guidance. This study aims to identify specific MRI findings to distinguish between bile sludge and physiological bile concentration for improved diagnosis and clinical management.
**Methods or Background:** This retrospective study included patients with non-stone signal changes on upper abdominal MRI from January 2022 to April 2024, who had follow-up ultrasounds within one month. The study involved 42 patients in the sludge group and 44 in the non-sludge group according to ultrasound findings. Key parameters assessed were layering, fluid-fluid levels on T2, T1 signal characteristics, and signal loss on out-of-phase (OOP) sequences. Quantitative measures included the T2 abnormal signal/spleen ratio, ADC values, and the normal-bile/abnormal-signal ratios on T2 and ADC maps. Multivariate regression was performed on parameters with $p < 0.05$, and the model's diagnostic performance was evaluated.
**Results or Findings:** T1-weighted hypointensity and fluid-fluid levels were strongly associated with bile sludge ($p < 0.001$), while layering and OOP signal loss associated with normal bile($p<0,001$). The best multivariate model, using T1W, fluid levels, and OOP signal loss, showed 83.72% specificity, 72.22% sensitivity, and 78.48% accuracy, with an AUC of 0.889.
**Conclusion:** An abnormal signal in the gallbladder lumen on MRI that does not exhibit OOP signal loss but shows fluid-fluid levels and T1-weighted hypointensity may be indicative of bile sludge.
**Limitations:** This study is limited by its retrospective design, which may introduce selection bias. Additionally, ultrasound was used as the gold standard, biochemical evaluation of the bile was not conducted.
**Funding for this study:** No funding
**Ethics committee - additional information:** Ethics committee approval was obtained from the relevant institution.
**Author Disclosures:**
Anıl Usta: Nothing to disclose
Kenan Kadirli: Nothing to disclose
Suat Özkan: Nothing to disclose
Ali Cantürk: Nothing to disclose
Hakan Mutlu: Nothing to disclose
Serdar Sahin: Nothing to disclose

**11:30-12:30** **Research Stage 1**

### Research Presentation Session: Neuro

### RPS 311

### Illuminating the brain: neuroimaging insights into epilepsy and neuroinflammation

**Moderator**
S. Gerevini; Cremona/IT

**Expanding Language Assessment in Epilepsy Patients through the Individual Functional Connectome**

*R. Stepponat*, M. Berger, L. Schäfer, M. S. Yildirim, J. Leinkauf, F. Fischmeister, S. Bonelli, G. Kasprian; Vienna/AT
*(radheshyam.stepponat@meduniwien.ac.at)*

**Purpose or Learning Objective:** Pre-surgical language evaluation has remained largely unchanged for years, underscoring the need for improved methods. Given that epilepsy and language both operate as network functions, analyzing the individual functional connectome may offer greater clinical precision, enabling more accurate predictions and better-informed surgical decisions for enhanced patient care.
**Methods or Background:** Language is predominantly left-lateralized, and fMRI is commonly used for preoperative evaluation of lateralization by calculating the lateralization index (LI). However, a survey by Benjamin et al. highlights significant uncertainties in fMRI results among clinicians. In this study, 46 patients with temporal lobe epilepsy and 25 healthy controls underwent preoperative fMRI. Data preprocessing was performed with fMRIPrep, and correlation maps were created using the CONN toolbox. Seed-based connectivity (SBC) analysis of core language areas, based on Fredarenko et al. 2024, was conducted, and a LI based on graph measurements (degree) was calculated. This was compared to traditional LI analysis and neuropsychological data, with all analyses conducted in native space.
**Results or Findings:** Results indicated that the connectivity-based lateralization index (LI) provided a more consistent measure of language lateralization compared to standard LI methods, aligning better with neuropsychological assessments. Patients with lesional epilepsy showed greater variability in lateralization compared to MR-negative patients, while healthy controls exhibited stronger left-lateralization as expected. The use of seed-based connectivity (SBC) analysis enhanced the detection of individual differences in language network organization, underscoring its potential clinical value in preoperative assessment.
**Conclusion:** The initial findings suggest that connectivity-based LI may improve the accuracy of preoperative language assessments. This approach offers a promising enhancement over traditional LI methods, providing more reliable insights for surgical planning and potentially leading to better predictions of post-surgical cognitive outcomes.
**Limitations:** -No neuropsychological data for the controls.
-Retrospective data.
**Funding for this study:** This study has been conducted as part of a PhD-thesis at the medical university of Vienna. Nothing to disclose.
**Ethics committee - additional information:** EK-Number: 1141/2023
**Author Disclosures:**
Mehmet Salih Yildirim: Nothing to disclose
Laurin Schäfer: Nothing to disclose
Joel Leinkauf: Nothing to disclose
Florian Fischmeister: Nothing to disclose
Marc Berger: Nothing to disclose
Silvia Bonelli: Nothing to disclose
Radheshyam Stepponat: Nothing to disclose
Gregor Kasprian: Nothing to disclose

**Temporal lobe epilepsy with isolated amygdala enlargement: anatomo-electro-clinical features and long-term outcome**
M. Ferro[1], J. Ramos[2], *F. M. Doniselli*[3], G. Didato[3]; [1]Lisbon/PT, [2]Gaia/PT, [3]Milan/IT
*(fabio.doniselli@gmail.com)*

**Purpose or Learning Objective:** This study focuses on the radiological characteristics of patients with temporal lobe epilepsy and isolated amygdala enlargement (TLE-AE).We aim to assess the imaging findings, particularly in relation to amygdala size and signal alterations, and their correlation with clinical and pathological data to guide better diagnostic and therapeutic decisions.
**Methods or Background:** We conducted a retrospective analysis of 143 brain MRI scans from adult patients at a tertiary neurology center. Forty-one patients with TLE-AE were selected. Imaging was reviewed by two neuroradiologists for amygdala size and T2-hyperintensity. Fluorodeoxyglucose-PET (FDG-PET) data were also included for a subgroup of patients.Amygdala signal alterations were quantified and correlated with clinical, neurophysiological, and pathological findings. Statistical analyses assessed relationships between imaging features and clinical outcomes.
**Results or Findings:** Out of 41 patients, 32% had bilateral amygdala enlargement, while the remainder had unilateral findings. T2-hyperintensity was noted in 65.9% of cases, significantly correlating with amygdala enlargement. FDG-PET showed temporomesial hypometabolism in 64% of patients, further supporting the epileptogenic involvement of the amygdala.Surgical pathology confirmed various underlying etiologies, including gliosis, low-grade tumors, and inflammatory infiltrates. Post-surgical outcomes were favorable, with 70.6% of operated patients being seizure-free at last follow-up.
**Conclusion:** Radiologically, amygdala enlargement with or without T2-hyperintensity is a significant marker of TLE, often associated with underlying structural abnormalities or inflammation. FDG-PET is a valuable adjunct for identifying hypometabolic regions corresponding to AE, supporting its role in the epileptic network.MRI findings, combined with surgical evaluation, can improve clinical outcomes in TLE-AE patients, particularly those with drug-resistant epilepsy.
**Limitations:** The study's retrospective nature and variability in MRI protocols over time limit the consistency of radiological findings. Additionally, not all patients underwent advanced imaging techniques, such as PET or follow-up MRIs, reducing the ability to track longitudinal changes.
**Funding for this study:** None.
**Ethics committee - additional information:** Retrospective study.
**Author Disclosures:**
Fabio Martino Doniselli: Nothing to disclose
Margarida Ferro: Nothing to disclose
Joao Ramos: Nothing to disclose
Giuseppe Didato: Nothing to disclose

**The relationship of glutamate and glutamine and metabolic profiling in focal epilepsy using 7T CRT-FID-MRSI**
*S. Chambers*, H. Shayeste, P. Lazen, L. Haider, E. Pataraia, G. Kasprian, W. Bogner, S. Trattnig, G. Hangel; Vienna/AT
*(stefanie.chambers@meduniwien.ac.at)*

**Purpose or Learning Objective:** Identifying epileptogenic foci is essential in therapy-planning and predictive for post-operative seizure freedom in epilepsy. In this work we present a novel MRSI technique (CRT-FID) at 7T, allowing for ultra-high resolution whole-brain maps in focal epilepsy. We offer a qualitative analysis of its feasibility in identifying and characterizing metabolic alterations over multiple pathologies.
**Methods or Background:** Following informed written consent, forty-two patients with focal epilepsy (16-52 years, 21 females/21 males) underwent a 3D-MRSI protocol in 15min with a 3.4 mm isotropic resolution at 7T using a 32Rx/1Tx-coil. Data processing involved spectral quantification and ratio mapping of Glu, Gln, Ins, tCho, tCr and NAA normalized to NAA and tCr. Furthermore, the concentration estimates of Glu and Gln were qualitatively assessed in seizure onset zones.
**Results or Findings:** Though we could find no consistent metabolic pattern throughout pathologies, ratio maps showed high detectability of lesions, identifying alterations in seizure onset zones in 78,6% when normalized to NAA. This detection rate was reduced to 71,2% when normalized to creatine. Of the assessed ratios, Ins/tNAA and Cho/tNAA showed highest stability across patients, whereas Glu/tNAA and Gln/tNAA showed varying directionality of changes. Assessing these changes further in relation to clinical parameters such as the seizure frequency, we found a trend for relative increases of Gln/Glu in patients with low seizure frequencies and the inverse for high seizure frequencies.
**Conclusion:** 7T CRT-FID-MRSI shows promising results in identifying metabolic alterations in patients suffering from focal epilepsy, even in the absence of structural lesions. Furthermore, this orientational study may point to an altered glutamate/glutamine cycle in epilepsy, potentially the result of altered energy metabolism pathways in chronic epilepsy.
**Limitations:** This study is limited by the small cohort size and qualitative nature of the analysis.
**Funding for this study:** This research was funded by the FWF grant 10.55776/KLI1121, of the Mayor of the Federal Capital Vienna (Project Number 21186).
**Ethics committee - additional information:** Ethic committee number: EK 1039/2020
**Author Disclosures:**
Wolfgang Bogner: Nothing to disclose
Philipp Lazen: Nothing to disclose
Stefanie Chambers: Nothing to disclose
Lukas Haider: Nothing to disclose
Ekatarina Pataraia: Nothing to disclose
Siegfried Trattnig: Nothing to disclose
Gilbert Hangel: Nothing to disclose
Gregor Kasprian: Nothing to disclose
Haniye Shayeste: Nothing to disclose

**Multiparametric MR-based assessment supports the inflammatory nature of symptomatic CSF HIV Escape**
S. Capelli[1], A. Caroli[1], G. Pezzetti[2], F. Ferretti[3], R. Vercesi[4], P. Cinque[4], *S. Gerevini*[2]; [1]Ranica/IT, [2]Bergamo/IT, [3]London/UK, [4]Milan/IT

**Purpose or Learning Objective:** Symptomatic cerebrospinal fluid (CSF) HIV escape is an infrequent but severe condition occurring in persons living with HIV (PLWH) undergoing combination antiretroviral therapy (cART). It is characterized by HIV-RNA in the CSF despite being undetectable in plasma. Similarly to HIV encephalitis in cART-untreated PLWH (HIV-ENC), HIV CSF escape (HIV-ESC) is accompanied by neurological impairments and brain MRI alterations. This study aimed to investigate the neuroimaging features of HIV-ESC in comparison to HIV-ENC and neuro-asymptomatic controls.
**Methods or Background:** Brain structural and microstructural alterations were quantified in: HIV-ESC (n=12), HIV-ENC (n=10), neuro-asymptomatic PLWH (n=11) and neuro-asymptomatic HIV-negative controls (n=12). The quantitative analysis included measurements of normalized FLAIR signal intensity, Apparent Diffusion Coefficient (ADC) from diffusion-weighted MRI, and brain tissue volumes from T1-weighted MRI.
**Results or Findings:** Both HIV-ESC and HIV-ENC demonstrated significantly higher FLAIR signal intensity in white matter (WM), elevated ADC values in both white and gray matter (GM) and reduced GM volumes as compared to neuro-asymptomatic controls, while the HIV-ESC group had higher WM volumes compared to HIV-ENC. In the HIV-ESC group, GM ADC values were negatively correlated with nadir CD4+ T-cell counts, while GM volume showed a positive correlation. In contrast, in HIV-ENC, WM ADC, FLAIR signal intensity, and WM volume all positively correlated with nadir and current CD4+ T-cell counts.
**Conclusion:** WM hyperintensities and increased ADC values in HIV-ESC and HIV-ENC reflect active WM damage, while reduced GM volumes are indicative of long-term brain atrophy. However, the higher WM volume in HIV-ESC suggests persistent inflammation. These findings, along with their correlation to laboratory data, support the hypothesis that inflammation is the primary mechanism of brain damage in HIV-ESC.
**Limitations:** Retrospective study with limited patient sample and lack of a standardized MRI protocol across all subjects.
**Funding for this study:** National Institutes of Health (NIH); University of California, San Francisco (UCSF)
**Ethics committee - additional information:** The study was approved by “Comitato Etico IRCCS Ospedale San Raffaele” on 15/12/2016 (ref. 235/2015)
**Author Disclosures:**
Serena Capelli: Nothing to disclose
Giulio Pezzetti: Nothing to disclose
Anna Caroli: Nothing to disclose
Francesca Ferretti: Nothing to disclose
Riccardo Vercesi: Nothing to disclose
Paola Cinque: Nothing to disclose
Simonetta Gerevini: Nothing to disclose

**Unravelling PIRA brain atrophy pattern and progression**
*T. Fakhreddine*[1], A. Tamanti[1], C. Salvatore[2], D. Calderaro[1], S. Ziccardi[1], M. Calabrese[1], A. Fattorini[1], I. Castiglioni[2], F. B. B. Pizzini[1]; [1]Verona/IT, [2]Milan/IT
*(tom.fakhreddine@gmail.com)*

**Purpose or Learning Objective:** Progression independent of relapse activity (PIRA) is the primary factor contributing to irreversible disability accumulation in relapsing multiple sclerosis (MS). This study aimed to investigate differences in brain volumes through Magnetic Resonance Imaging (MRI).

Wednesday

**Methods or Background:** We conducted a retrospective study with MS patients with and without cognitive PIRA who performed T1-weighted 3D MRI study (at baseline and at 24-months follow-up) at the Verona University Hospital. The TRACE4AD™ medical-device software (DeepTrace Technologies, Italy) performed automatic structural brain segmentation from MR scans into 53 regions and calculated corresponding total volumes and asymmetry indices, defined as the percentage difference between brain region volumes on each side. Atrophy progression was measured as the percentage difference in volumes at follow-up versus baseline. A statistical comparison of volumes at baseline and atrophy progression was performed using a two-sample t-test between MS patients with and without PIRA.
**Results or Findings:** We collected 95 MRIs from 40 patients with PIRA (26 with follow-up) and 55 patients without PIRA (43 with follow-up). At baseline, the difference in asymmetry indices of total white matter, gray-matter occipital-lobe volume and parieto-occipital cortex volume were statistically significant ($p<0.05$): PIRA patients showed more right-side atrophy in the white matter volume and less in the right occipital lobe and parieto-occipital cortex than non-PIRA patients. Atrophy progression was statistically different ($p<0.05$) for the gray-matter volume of the right temporal lobe, being faster in patients with PIRA than in those without (-3.1% vs -1.3%).
**Conclusion:** These results indicate that atrophy progression may be faster in MS patients with PIRA in regions related to early dementia and non-verbal language functions.
**Limitations:** Cohort size and statistical power show potential for improvement.
**Funding for this study:** No funding was received for this study.
**Ethics committee - additional information:** The study is retrospective.
**Author Disclosures:**
Davide Calderaro: Nothing to disclose
Isabella Castiglioni: Nothing to disclose
Agnese Tamanti: Nothing to disclose
Christian Salvatore: Nothing to disclose
Tommaso Fakhreddine: Nothing to disclose
Massimiliano Calabrese: Nothing to disclose
Francesca Benedetta Benedetta Pizzini: Nothing to disclose
Anna Fattorini: Nothing to disclose
Stefano Ziccardi: Nothing to disclose

### Mean Upper Cervical Cord Area (MUCCA) in MOGAD compared to MS, NMOSD and healthy controls

*E. Lotan*[1], V. Anania[2], T. Billiet[2], I. Kister[1], I. Lotan[3]; [1]New York, NY/US, [2]Leuven/BE, [3]Petach Tikva/IL
*(vincenzo.anania@icometrix.com)*

**Purpose or Learning Objective:** Relatively little is known about how mean upper cervical cord area (MUCCA) changes in MOGAD compared to MS, NMOSD, and healthy controls (HC). We aim to assess MUCCA values in MOGAD as compared to MS, NMOSD, and HC.
**Methods or Background:** We retrospectively reviewed the NYU Multiple Sclerosis Care Center database to identify all adult MOGAD patients with available brain MRI performed in stable remission and compared them with NMOSD and MS patients and HC. Cross-sectional MUCCA from T1 brain MRIs was quantified using icobrain ms+ (version 5.15.0) and normalized for head size. A linear modeling analysis was used to evaluate the impact of covariates on cross-sectional MUCCA. The covariates were age, T1 slice-thickness, sex, and group. Post hoc testing was conducted using estimated marginal means (EMMEANS) to evaluate group differences while controlling for covariates.
**Results or Findings:** 20 MOGAD patients, 37 AQP4+ NMOSD patients, 40 MS patients, and 31 HC were included in the analysis. Age, sex, and group showed significant effects on MUCCA measurements. The EMMEANS of MUCCA values were lower for the NMOSD group (86.1+/-1.5), followed by MOGAD (89.3+/-1.7), MS (90.3+/-1.2), and HC (91.6+/-1.5). Pairwise comparison between groups showed no statistically significant differences between the MOGAD and other groups. In contrast, a statistically significant difference between the NMOSD and HC groups and a trend towards significance between the NMOSD and MS groups were observed.
**Conclusion:** Our proof of concept study shows the feasibility of calculating cervical volume loss from routine brain MRI using automated software. While greater spinal cord tissue loss is evident in NMOSD, the degree of spinal cord tissue loss in MOGAD is lower and not significantly different compared to MS and HC. Additional analyses on a larger cohort are underway.
**Limitations:** N/A
**Funding for this study:** No funding was provided for this study.
**Ethics committee - additional information:** The ethics committee notification can be found under study number i20-01556
**Author Disclosures:**
Eyal Lotan: Nothing to disclose
Vincenzo Anania: Employee: icometrix
Thibo Billiet: Employee: icometrix
Ilya Kister: Nothing to disclose
Itay Lotan: Nothing to disclose

### Medial lemniscus as a diagnostic marker: differentiating multiple sclerosis from small vessel disease

*W. H. E. Hamed*, D. Werring, D. S Lynch, R. Jäger, T. A. Yousry; London/UK
*(weaam.hamed@nhs.net)*

**Purpose or Learning Objective:** Evaluate the diagnostic utility of medial lemniscus (ML) in differentiating between multiple sclerosis (MS) and small vessel disease(SVD).
**Methods or Background:** Distinguishing between MS and SVD remains a significant challenge in the elderly due to their overlapping clinical presentations, emphasising the necessity for reliable, non-invasive neuroimaging markers such as ML. A retrospective study analysed 270 MRI scans(100 MS, 170 SVD). SVD subtypes included arteriosclerotic(50), cerebral amyloid angiopathy(CAA)(50), mixed(50), and genetic(20). The signal intensity of ML in the pontine tegmentum was assessed visually on T2-w and FLAIR images. Statistical analysis included univariable tests to identify differences between MS and SVD, followed by multivariable logistic regression to determine independent predictors of ML involvement.
**Results or Findings:** SVD patients were significantly older than MS patients (mean age:68±13vs43±10 years, $p<0.001$) and had lower female predominance (44.7%vs64%). MS patients had no ML involvement(87%) or unilateral involvement(13%) with no bilateral cases, while 38% of SVD patients had bilateral involvement($p<0.001$). Among SVD categories, ML involvement was most frequent in genetic(80%), followed by mixed(64%) and arteriosclerotic(46%). The least affected was CAA(20%). In MS cohort, ML hyperintensity was associated with higher lesion load($p<0.001$) but not the disease duration($p=0.4$). Among SVD subtypes, the multivariable analysis revealed that lesion load($p<0.001$) and hypertension($p=0.002$) were the strongest predictors of ML involvement, followed by age($p=0.007$), diabetes($p=0.023$), and hypercholesterolemia($p=0.048$). Factors such as alcohol, gender, and smoking were not significant predictors.
**Conclusion:** Our study establishes ML as a reliable radiological marker for distinguishing MS from SVD, particularly when there is bilateral involvement, evidenced by a statistically significant presence in SVD and a notable absence in MS. Moreover, the variation in ML involvement across SVD subtypes, specifically its minimal presence in pure CAA, suggests its potential role in differentiating specific SVD categories.
**Limitations:** Not applicable.
**Funding for this study:** No funding was provided for this study.
**Ethics committee - additional information:** A retrospective study
**Author Disclosures:**
Tarek A. Yousry: Nothing to disclose
Weaam Hamed Elsayed Hamed: Nothing to disclose
Rolf Jäger: Nothing to disclose
David Werring: Nothing to disclose
David S Lynch: Nothing to disclose

### Brain Disconnection and Atrophy Assessment Multiple Sclerosis Converters

*S. Hannoun*, S. Ghazal, L. Halawi, C. Al-Dahouk, N. El-Ayoubi, S. Khoury; Beirut/LB
*(sh156@aub.edu.lb)*

**Purpose or Learning Objective:** Differentiating patients who convert from relapsing-remitting Multiple sclerosis (RRMS) to secondary progressive MS (SPMS) remains a critical challenge, as early identification of converters can significantly impact treatment strategies. This study explores specific brain regions associated with disconnection probabilities and volume reductions, aiming to identify potential MRI biomarkers predictive of conversion. This study aimed to investigate whether distinct patterns of WM disconnection and regional brain atrophy are associated with RRMS conversion to SPMS.
**Methods or Background:** We retrospectively analyzed 47 RRMS patients (17 Converters and 30 non-converters) who underwent baseline and follow-up MRI scans approximately 1.5 years apart. Mixed-effects models evaluated the interaction between conversion status (converters vs. non-converters) and time across various brain regions, focusing on disconnection probabilities and volumetric changes measured using the Vol2Brain tool.
**Results or Findings:** Converters exhibited significant disconnection in key white matter tracts, including the uncinate fasciculus, corticobulbar tract, superior longitudinal fasciculus, and cingulum parahippocampal parietal. These disruptions are linked to cognitive, emotional, and motor functions. Additionally, grey matter atrophy was more pronounced in converters, particularly in the precentral gyrus, temporal lobe, and thalamus. Lesion burden and volume, especially in juxtacortical areas, were greater in converters, with increased third ventricle volume indicating more severe brain atrophy.
**Conclusion:** Specific patterns of white matter disconnection and regional brain atrophy are associated with conversion from RRMS to SPMS. These MRI biomarkers provide valuable insights into disease progression and offer potential therapeutic targets. Further validation in larger cohorts is needed to integrate these findings into clinical practice.

**Limitations:** A limitation of our study is its relatively small sample size and retrospective nature, which may limit the generalizability of the findings.
**Funding for this study:** No funding.
**Ethics committee - additional information:** This study was approved by the Institutional Review Board (IRB), and all participants provided informed consent.
**Author Disclosures:**
Samia Khoury: Nothing to disclose
Lean Halawi: Nothing to disclose
Salem Hannoun: Nothing to disclose
Nabil El-Ayoubi: Nothing to disclose
Sola Ghazal: Nothing to disclose
Cezar Al-Dahouk: Nothing to disclose

**11:30-12:30** **Research Stage 2**

### Research Presentation Session: Imaging Informatics and Artificial Intelligence

### RPS 305

### Career, workforce issues and radiologist visibility

**Moderator**
F. Mankertz; Greifswald/DE

### Radiological discrepancy review: a novel, customisable, cloud-based technology to implement the REALM paradigm

*P. Brennan*[1], Y. Hughes-Roberts[2], J. Richenberg[2], I. Francis[2], M. Suleiman[1]; [1]Sydney/AU, [2]West Sussex/UK
*(patrick.brennan@detectedx.com)*

**Purpose or Learning Objective:** To develop a geographically-limitless REALM infrastructure that facilitates an optimised and reflective educational activity.
**Methods or Background:** Reviewing radiological discrepancies is a well-known activity that promotes diagnostic excellence, encourages reflection and minimises future errors. Formalisation of discrepancy reviews can be seen with the Royal College of Radiology REALM (Radiology Events and Learning Meetings) program where radiologists in the UK can engage both as authors and recipients. However, to promote a clinically-realistic REALM activity, comprehensive radiologic interactions with full resolution anonymised images should occur in a geographically limitless way. The infrastructures to support this, are elusive. An existing technology DxCARES was modified for this activity. This incorporated: multi-modality viewing capabilities and 3D and multi-planar reconstructions (MPR): advanced AI-powered anonymization and masking engines to automatically remove sensitive data while maintaining diagnostic integrity; a user-friendly interface for users with different levels of expertise; a web-based architecture supporting high performance DICOM streaming and real time interaction with large data sets.
**Results or Findings:** We have built a new technology which allows clinicians to upload REALM cases from PACs systems and distribute across a geographically-limitless health enterprise to limitless recipients. Customisable authorship of each case is available so that each creator can demand from recipients the type of case-specific interaction or reflection that is required, regardless of image or pathology-type. The multifunctional cloud-based viewer allows the examination and manipulation of all cases as would occur with a primary diagnostic workstation. All REALM outputs and interactions are available for review and CPD accumulation. The new technology is currently being implemented across clinical centres.
**Conclusion:** This new technical innovation should promote widespread clinically-realistic REALM engagement by simplifying time/cost implications. The potential of radiological discrepancy review should be maximised.
**Limitations:** N/A
**Funding for this study:** N/A
**Ethics committee - additional information:** N/A
**Author Disclosures:**
Jonathan Richenberg: Nothing to disclose
Patrick Brennan: Founder: DetectedX Pty Ltd
Ian Francis: Nothing to disclose
Ynyr Hughes-Roberts: Nothing to disclose
Moayyad Suleiman: Founder: DetectedX Pty Ltd

### Sex differences in inappropriate imaging requests: Insights from the Medical Imaging Decision And Support (MIDAS) trial

*S. Dijk*[1], C. Wollny[2], T. Kroencke[2], M. G. M. Hunink[1]; [1]Rotterdam/NL, [2]Augsburg/DE
*(stijntjedijk@gmail.com)*

**Purpose or Learning Objective:** We analyzed sex-related disparities in inappropriate imaging requests using data from the Medical Imaging Decision And Support (MIDAS) trial.
**Methods or Background:** This study analyzed baseline data from the MIDAS trial, a multi-center cluster randomized trial conducted at three German academic hospitals. The study population encompassed all imaging requests submitted to the 26 participating departments via the computerized order entry system during a 15-month period. Imaging appropriateness was assessed using the ESR iGuide, a clinical decision support system (CDSS). Requests were categorized as inappropriate if imaging was deemed unlikely to be indicated or if the potential risks outweighed the benefits for the patient. Chi-square tests were employed to compare the proportion of inappropriate imaging requests between men and women, with a significance level of 0.01. Secondary analyses explored differences in inappropriate requests by age group and exam type, with a Bonferroni correction applied to account for multiple testing.
**Results or Findings:** Women had more inappropriate imaging requests (7.32%) than men (6.08%; $\chi^2 = 37.176$, $p < 0.001$, OR 1.22 [95%CI 1.12-1.33]). This disparity was particularly evident in the 25-65 age group and for MRI examinations. Further research is needed to explore the underlying causes of this discrepancy, including potential differences in physician awareness of guidelines, adherence to guidelines, or limitations in the CDSSs ability to account for female-specific factors.
**Conclusion:** In our study clinicians were 22% more likely to request inappropriate imaging for women than for men across nearly all modalities and age-groups. While the absolute percentage-point differences were small (1.24%), the disparity warrants further investigation
**Limitations:** Our analysis judges each request independently, without considering the cumulative impact of requests per individual, underuse, or time between symptom onset and imaging. Our data only distinguished male/female sexes.
**Funding for this study:** The MIDAS study was funded by the German Innovation Fund (reference: Förderkennzeichen 01VSF18008).
**Ethics committee - additional information:** Approval from the Medical Ethics Review Committee was obtained under protocol numbers 20-069 (Augsburg), B 238/21 (Kiel), 20-318 (Lübeck) and 2020-15125 (Mainz). The trial is registered in the ClinicalTrials.gov register under registration number NCT05490290.
**Author Disclosures:**
Thomas Kroencke: Nothing to disclose
Claudia Wollny: Nothing to disclose
Myriam G. M. Hunink: Nothing to disclose
Stijntje Dijk: Nothing to disclose

### Multicenter and multimodality evaluation of radiological workload and development of a benchmarking metric

*P. Dankerl*, J. Lang, A. Glaser, H. P. Beyer, M. Forsting; Dortmund/DE
*(Peter.Dankerl@evidia.de)*

**Purpose or Learning Objective:** The increasing complexity and volume of radiological examinations have led to growing concerns about radiologists' workload, diagnostic efficiency and accuracy. The aim of this study was to evaluate and compare radiological workload in a multicenter and multimodality analysis while creating a benchmarking metric.
**Methods or Background:** Over 100 days radiologists' reading times for all examinations and modalities across 34 centers have been collected utilizing RIS-export, while grouping these into 67 different body regions. In order to sort, retrieve and evaluate the various output formats from the RIS-data, a uniform nomenclature was introduced and all examinations received these additional unique identifying labels. For benchmarking, reporting times were translated into relative value units – as defined by us as the mean reporting time of all X-ray exams and termed RADPoints.
**Results or Findings:** We examined 290.748 examinations and found significant variations in average reporting times across modalities and body region, e.g. abdominal MRI 14.83min and CT 13.59min. Reporting times varied considerably, with the highest average times observed in complex examinations such as cardiac MRI (26.04min) and CT (16.59min). Conversely, regions like the fingers showed much lower averages, e.g. 10.95min for MR and 9.83min for CT. We found our relative value unit time of one RADPoint to equivalent to 2.23min which further served as common devisor in order to allocate specific RADPoints to all body region specific examinations.
**Conclusion:** The findings underscore the need for targeted workload management strategies in radiology departments, particularly when high-complexity cases are in the mix. Benchmarking reporting times across modalities and body regions by the means of presented RADPoints provides a critical reference for optimizing radiologist workload, potentially leading to enhanced diagnostic accuracy and efficiency.
**Limitations:** None
**Funding for this study:** None
**Ethics committee - additional information:** Retrospective evaluation and blinding of patinet as well as doctor identyfiers makes this not applicable
**Author Disclosures:**
Michael Forsting: Employee: Evidia
Peter Dankerl: Employee: Evidia
Haemi Phaedra Beyer: Employee: Evidia
Jochen Lang: Employee: Evidia
Andrzej Glaser: Employee: Evidia

**Assessing the perceived impact of ESOR training programs on radiologists' professional development**
*J. Gregory*[1], M. L. Kofoed-Ottesen[2], B. Lindlbauer[2], C. Loewe[2], V. Vilgrain[1]; [1]Clichy/FR, [2]Vienna/AT
*(jules.gregory@aphp.fr)*

**Purpose or Learning Objective:** This study evaluates the perceived impact of European School of Radiology (ESOR) training programs on radiologists' professional development.
**Methods or Background:** A cross-sectional survey targeted alumni who participated in ESOR fellowships from 2011 to 2023. The survey included questions on demographics, professional background, ESOR program details, and career impact. Data were collected via a web-based questionnaire and analyzed using descriptive statistics and thematic analysis.
**Results or Findings:** A total of 190 participants responded, with a median age of 31 years (range 29-33), and 54% were female. Most worked in public healthcare (62%) and were involved in academic activities (24%). Fellowship types included Visiting Scholarship Program (44%), Bracco Fellowship (32%), and Exchange Program for Fellowships (25%). The majority (59%) reported the fellowship helped them reach their current position, and 35% noted it upgraded their CV. Significant application of learned skills was reported by 69%. Ongoing cooperation with former tutors was maintained by 54%. Financial support was crucial, with 41% stating they could not have completed the training without it, 33% considering it very important, and 13% deeming it important. Participants rated the impact on clinical skills with a median score of 9 out of 10. Other areas of impact included research skills (median score 7), subspecialization (median score 9), exposure to diverse practices (median score 9), networking opportunities (median score 10), and personal and professional growth (median score 10).
**Conclusion:** ESOR training programs significantly enhance radiologists' professional development through comprehensive support, high-quality training, and substantial financial aid, ensuring participants are well-equipped for career advancement.
**Limitations:** This study has limitations, including reliance on self-reported data, potential recall-bias, and a 20% response rate. The survey may not fully represent all ESOR alumni, and program heterogeneity could influence the generalizability of results.
**Funding for this study:** None
**Ethics committee - additional information:** Given the nature of the survey involving professional feedback without sensitive personal data, ethical approval was not required. However, all participants were informed about the purpose of the survey and the anonymous handling of their data.
**Author Disclosures:**
Jules Gregory: Nothing to disclose
Brigitte Lindlbauer: Board Member: European School of Radiology Office
Valérie Vilgrain: Board Member: European School of Radiology Office
Christian Loewe: Board Member: European School of Radiology Office
Mathias Lange Kofoed-Ottesen: Board Member: European School of Radiology Office

**Expanded AI learning: AI as a tool for human learning**
S. Faghani[1], C. Tiegs-Heiden[2], M. Moassefi[2], G. Powell[2], M. Ringler[2], B. J. Erickson[2], *N. Rhodes*[2]; [1]Minneapolis, MN/US, [2]Rochester, MN/US
*(nickgrhodes@hotmail.com)*

**Purpose or Learning Objective:** To use artificial intelligence (AI) as a teaching tool to identify new imaging findings and improve the radiologist’s ability to recognize subtle imaging findings without additional AI assistance.
**Methods or Background:** We studied the learning of a new task by humans using a deep learning (DL) model that can identify sex differences from frontal knee radiographs with high accuracy. We then ascertained imaging features via occlusion interpretation maps (“heat maps”) to help human readers improve their ability to perform this task. Three human readers were tasked to classify 50 frontal knee radiographs into male and female sex. They were then informed of the patient’s sex and were given the associated AI-derived “heat maps” for subsequent study. After two weeks, the group was retested with a new set of 50 radiographs.
**Results or Findings:** The DL model categorized sex with 0.96 accuracy. The average accuracy of the 3 human readers was initially 0.59 (range: 0.56-0.66). After study of AI-derived “heat maps” and associated radiographs, the average accuracy of the human readers increased to 0.80 (range: 0.76-0.84), a statistically significant improvement (p=0.0270).
**Conclusion:** We believe this improvement serves as a proof of concept for the use of AI as a tool for discovery science to advance human learning, an idea that we have not seen advanced in the radiology literature.
**Limitations:** 1. This is an education item that does not fall well into an AI or MSK section. 2. Understanding how AI algorithms work is limited. 3. Our study is small. Large enough to prove an improvement after training, but too small to dissect more.
**Funding for this study:** None.
**Ethics committee - additional information:** N/A
**Author Disclosures:**
Bradley James Erickson: Nothing to disclose
Mana Moassefi: Nothing to disclose
Garret Powell: Nothing to disclose
Shahriar Faghani: Nothing to disclose
Christin Tiegs-Heiden: Nothing to disclose
Michael Ringler: Nothing to disclose
Nicholas Rhodes: Nothing to disclose

**From observation to interpretation: elevating reporting skills in radiology**
*S. Ramirez*; Bogota/CO
*(sandritamrt@gmail.com)*

**Purpose or Learning Objective:** The preparation of a radiological report involves integrating knowledge, skills, and training. While many articles offer recommendations for quality reports, there is a lack of literature on teaching strategies for efficiently constructing these reports. Despite this, some residents and radiologists have developed this competency over time, revealing a gap in understanding how residents acquire the critical thinking skills needed. This work aims to determine how radiology residents learn to create their reports.
**Methods or Background:** A qualitative multi-case study was conducted involving three cases represented by residents of a radiology residency program, the program's faculty, and a group of specialists in high-quality report production. The study included document reviews of the syllabus, semi-structured interviews, and a focus group to capture experiences, perspectives, and learning processes, as well as the factors and strategies that influenced the development of the knowledge and skills needed for creating radiological reports.
**Results or Findings:** Information was collected on concepts, perceptions, beliefs, emotions, interactions, and experiences to construct coherent meanings about the topic. Common patterns and challenges in learning to create radiological reports were identified. Participants described the hidden curriculum as as the main component of the educational process through which residents learn to write reports. Preparing preliminary reports was regarded as the most valuable pedagogical activity for developing critical thinking skills. All residents agreed on the need to design educational activities focused on learning to prepare radiological reports.
**Conclusion:** This study represents, to our knowledge, the first research on how the critical thinking skills necessary for creating a radiological report are acquired. By understanding how a person effectively acquires a skill, teaching strategies can be designed and adapted to maximize meaningful learning.
**Limitations:** None
**Funding for this study:** No
**Ethics committee - additional information:** Ethics committee Fundación Universitaria Sanitas
**Author Disclosures:**
Sandra Ramirez: Nothing to disclose

**The use of Instagram in medical education: gender differences and student Satisfaction and gender differences compared to traditional learning platforms (Blackboard)**
*M. Alvarez García*, M. E. Pueyo, P. Boldó, C. Urtasun Iriarte, A. Ezponda Casajus, P. Chico, J. Pueyo Villoslada; Pamplona/ES
*(malvarezgar@alumni.unav.es)*

**Purpose or Learning Objective:** This study aimed to explore the student satisfaction with Instagram as an educational tool in radiology education for fourth-year medical students, compared to a traditional learning platform.
**Methods or Background:** The study focused on gender differences and overall preferences in platform usage among fourth-year medical students (64% female). Both Instagram and the traditional platform were used to provide multiple-choice test questions with images based on radiology class content. Students chose their preferred platform for completing quizzes, and their experiences were assessed via anonymous satisfaction surveys. Statistical analysis explored gender-based differences in platform usage, preferences, and perceived learning benefits. The Mann-Whitney U test was applied to analyze the results.
**Results or Findings:** A total of 65.8% of participants chose Instagram as their preferred platform for answering clinical cases. No significant gender differences were observed in overall platform choice. The majority (71%) of students expressed moderate to high satisfaction with Instagram as a complementary learning tool. A significant gender difference (p=0.019) was found in the reasons for preferring Instagram: women favored its user-friendly interface, while men prioritized its speed. No significant differences were found between genders regarding Instagram's usefulness for personal study or whether it caused distractions (p=0.31). Additionally, 97.1% of students preferred Instagram over other social networks.
**Conclusion:** Instagram was generally preferred over traditional learning platforms and other social media platforms for radiology education, with a moderate to high level of student satisfaction.
**Limitations:** Certain students do not use Instagram. There might be missing data. Instagram tool “Stories” only allows users (students) to see the content during a period of 24h.
**Funding for this study:** None
**Ethics committee - additional information:** This study has no ethical implications
**Author Disclosures:**
Pablo Chico: Author: Study design, Reviewer
Cesar Urtasun Iriarte: Author: Data analysis, reviewer
María Elena Pueyo: Author: Data analysis
Patricia Boldó: Author: Data analysis
Jesus Pueyo Villoslada: Author: Study design, data analysis, reviewer
Miguel Alvarez García: Author: Study design, data collection, data analysis, reviewer.
Ana Ezponda Casajus: Author: Reviewer

**Stock photos lead to inaccurate representations of radiologists by the media**
*L. Hartog*[1], J. M. L. Bosmans[2], J. Bouziotis[2], P. M. Parizel[3], A. Snoeckx[2];
[1]Antwerp/BE, [2]Edegem/BE, [3]Perth/AU
*(laurahartog@live.nl)*

**Purpose or Learning Objective:** Value-based radiology requires thorough understanding by patients of the role of radiologists in healthcare. News media frequently enlighten content on radiology with seemingly outdated images from stock photo databases. We conducted a critical evaluation of the ability of stock photos in three major databases to reliably represent contemporary radiology.
**Methods or Background:** We collected two hundred consecutive photos from three major commercial databases, using the keyword ‘radiologist’. Each image was evaluated for overall quality, image description, descriptive elements, displayed body regions, diagnostic or interventional context, depicted modality, inclusion of medical professionals, and accurate display on the viewing device.
**Results or Findings:** Just 6% of the stock photos received a high overall quality score. Radiographs were the most frequently depicted modality (52%), followed by MRI (21%) and CT (19%), with a focus primarily on diagnostic imaging (99%). On 4% of the stock photos, images were displayed on a PACS workstation, while 66% showed a radiologist holding up films ‘in the air’, and 10% on an X-ray viewbox. In 48% of the photos, the lead person was shown wearing a stethoscope. In all, only 9% of the stock photos presented a realistic view of a radiologist’s job.
**Conclusion:** The vast majority of stock photos fail to accurately portray the contemporary role of radiologists, contributing to gross misinterpretation of our profession by the general public.
**Limitations:** The evaluation was done by a radiology resident but reviewed by a consultant radiologist. To our knowledge, this kind of evaluation of an essential source of information for the public has never been undertaken before.
**Funding for this study:** No funding was received for this study.
**Ethics committee - additional information:** No patient data was used for this study.
**Author Disclosures:**
Annemiek Snoeckx: Nothing to disclose
Paul M. Parizel: Nothing to disclose
Jason Bouziotis: Nothing to disclose
Jan Maria Lodewijk Bosmans: Nothing to disclose
Laura Hartog: Nothing to disclose

**11:30-12:30 Research Stage 3**

### Research Presentation Session: Interventional Radiology

### RPS 309

### Image-guided tissue biopsies and thyroid interventions

**Moderator**
G. T. Yusuf; London/UK
*(Gibran.yusuf@nhs.net)*
**Author Disclosures:**
Gibran Timothy Yusuf: Speaker: Siemens, Samsung, GE, Terumo, Bracco

**Diagnostic accuracy of core needle biopsy in patients with lymphoproliferative disorders: an optimized protocol in 478 patients**
*P. Marra*, L. Dulcetta, R. Muglia, F. S. Carbone, A. Weber, S. Ferrari, A. Rambaldi, P. A. Erba, S. Sironi; Bergamo/IT
*(pmarra@asst-pg23.it)*

**Purpose or Learning Objective:** Surgical excision biopsy of lymph nodes stands as the gold standard for histological characterization of lymphoproliferative disorders (LD). However, contemporary clinical practice increasingly leans towards core needle biopsy (CNB). This study seeks to explore the factors influencing the diagnostic yield of CNB in LD.
**Methods or Background:** This unicentric retrospective study presents data from patients referred for suspicion of new or relapsing LD. All patients underwent image-guided CNB of the target lesion based on CT/PET findings. The primary endpoint was the diagnostic outcome, comparing the ability to achieve a definitive diagnosis according to international guidelines with CNB versus the necessity for subsequent excisional biopsy.
**Results or Findings:** We enrolled 478 consecutive patients undergoing CNB, categorized into two cohorts. Cohort A comprised patients who underwent CNB using 18-20G full-core Menghini needles, with a median macroscopic fragment dimension of 1 cm. Cohort B included patients who underwent CNB with 16-18G semiautomatic guillotine needles, with a median macroscopic fragment dimension of 1.5 cm. In cohort A, the rates of diagnostic and non-diagnostic (or non-sufficiently detailed) CNBs were 95 (73%) versus 35 (27%), respectively. In cohort B, these rates were 299 (86%) versus 49 (14%).
**Conclusion:** The type and size of the needle used for CNB, as well as the histologic variant of LD, emerged as factors influencing diagnostic yield and accuracy. Given the swiftness of CNB compared to surgical excision, optimizing this technique could streamline the diagnostic and therapeutic workflow for patients with suspected LD.
**Limitations:** Retrospective study; lack of control group undergoing surgery
**Funding for this study:** None
**Ethics committee - additional information:** Comitato Etico di Bergamo - Lymphoid Cancer Registry (NCT03131531)
**Author Disclosures:**
Sandro Sironi: Nothing to disclose
Alessandro Rambaldi: Nothing to disclose
Paola Anna Erba: Nothing to disclose
Paolo Marra: Nothing to disclose
Francesco Saverio Carbone: Nothing to disclose
Riccardo Muglia: Nothing to disclose
Alessandra Weber: Nothing to disclose
Silvia Ferrari: Nothing to disclose
Ludovico Dulcetta: Nothing to disclose

**The interplay of time and angle with the incidence of Pneumothorax in a CT-guided Lung Biopsy**
*N. Maalouf*[1], M. Abou Mrad[1], R. Benayed[1], R. A. Pugliesi[2], J. C. Apitzsch[1];
[1]Pforzheim/DE, [2]Stuttgart/DE
*(nourmaalouff@gmail.com)*

**Purpose or Learning Objective:** This study evaluates the relationship between the needle-pleura angle and the duration of needle traversal (NTD) through lung tissue during CT-guided lung biopsies, and their impact on the incidence of pneumothorax.
**Methods or Background:** 96 patients (54 m, 42 f, median age: 71 years) underwent CT-guided lung biopsies between January 2020 and March 2024. Procedures were performed using a semi-automatic 18G needle and a 17G trocar. The minimum delta (δmin) was calculated as the absolute difference between a 90° angle and the measured angles to the pleura and correlated

Wednesday with pneumothorax occurrence. NTD was recorded from needle puncture to retraction. Patients with immediate intraprocedural pneumothorax were excluded. A multivariate analysis compared four patient groups, categorized by δmin (greater or less than ten degrees) and NTD (less than or more than six minutes).
**Results or Findings:** 96 biopsies were performed, with six patients excluded. Pneumothorax occurred in 22 out of 90 procedures. Six of these 22 patients had δmin greater than 10 ° and NTD over six minutes; five had δmin less than ten degrees and NTD under six minutes. In contrast, 68 patients did not experience pneumothorax. Of these, 29 had δmin less than ten degrees with NTD under six minutes, while 20 had δmin less than ten degrees with NTD over six minutes. Pneumothorax occurred in 14.7% of patients with δmin less than ten degrees and NTD under six minutes, compared to 33.3% with δmin greater than ten degrees and NTD over six minutes.
**Conclusion:** Optimizing the needle-pleura angle and minimizing needle traversal duration during CT-guided lung biopsies can reduce pneumothorax risk.
**Limitations:** A relatively small number of patients, as it was a single-center observational study.
**Funding for this study:** No funding was received for this study.
**Ethics committee - additional information:** Approved by the local ethics committee (F-2021-038)
**Author Disclosures:**
Rosa Alba Pugliesi: Nothing to disclose
Jonas Christoph Apitzsch: Nothing to disclose
Mazen Abou Mrad: Nothing to disclose
Roua Benayed: Nothing to disclose
Nour Maalouf: Nothing to disclose

### Sensitivity and specificity of FNAC with ROSE of lung lesions: a single-center experience on 643 patients

*C. Verde*, L. Tarotto, S. Stilo, R. D'Angelo, V. Stoia, V. Sanna, N. Martucci, L. Arenare, F. Fiore; Naples/IT
*(caterina.verde@libero.it)*

**Purpose or Learning Objective:** The purpose of this study was to evaluate sensitivity and specificity of FNAC with ROSE in the diagnosis of lung lesions.
**Methods or Background:** The study was conducted at the INT of Naples "Foundation Pascale" between 2013 and 2017. CT, CBCT FNAC was performed on 643 patients, of which 195 subsequently underwent surgical resection. Exclusion criteria were: platelet count (< 50,000) and INR (> 1.5). Unenhanced TC scans or CBCT were performed pre-biopsy. A 18 G coaxial needle is used and a thinner needle (23-22G) is inserted into the lesion (2-3 times, if necessary). FNAC is associated with the extemporaneous examination (ROSE), which evaluates the adequacy of the sample. Pneumothorax is the most frequent complication but it is asintomatic in most cases and only 7 % of cases requests pleural drainage. No evidence of other major complications.
**Results or Findings:** Comparing surgical cytological and histological samples, positive cytological samples are neoplastic in 99.3% of cases (152/153) and non-neoplastic in only 0.6% of cases (1/153). The sensitivity of FNAC is 86.8%, the positive predictive value 99.3% and the specificity 75%.
**Conclusion:** FNAC is a reference diagnostic tool in the characterization of lung lesions for the purpose of target therapy and immunotherapy. It is an effective procedure with high sensitivity and specificity and low complication rate. Extemporaneous testing increases sensitivity, reducing the number of inadequate samples and false negatives.
**Limitations:** Single center study.
**Funding for this study:** No funding was received for this study.
**Ethics committee - additional information:** No information provided by the submitter.
**Author Disclosures:**
Vincenzo Stoia: Nothing to disclose
Nicola Martucci: Nothing to disclose
Francesco Fiore: Nothing to disclose
Caterina Verde: Nothing to disclose
Roberto D'Angelo: Nothing to disclose
Veronica Sanna: Nothing to disclose
Luca Tarotto: Nothing to disclose
Laura Arenare: Nothing to disclose
Salvatore Stilo: Nothing to disclose

### Targeted Fine-Needle Aspiration of Thyroid Nodules Guided by Shear Wave Elastography: A Novel Diagnostic Approach

*M. Khaleghi*[1], A. Aziz Ahari[2]; [1]Tehran/IR, [2]Boston, MA/US
*(Mokh.med2025@gmail.com)*

**Purpose or Learning Objective:** The aim of this study is to investigate target thyroid nodules based on shear wave elastography.
**Methods or Background:** Suspicious nodules requiring fine-needle aspiration (FNA) can sometimes be heterogeneous. Even though they may appear homogeneous on ultrasound at times, they are heterogeneous on shear wave elastography examination. Sampling from highly suspicious areas in shear wave elastography evaluation can lead to decreased inadequate samples and increased detection of malignant thyroid diseases. In the present study, patients referred for thyroid nodule sampling undergo elastography assessment and are included in the study if they exhibit heterogeneity. They then undergo thyroid FNA under ultrasound guidance using a 22-gauge spinal needle with stylet.
**Results or Findings:** As of today, five patients have been examined. In these patients, inadequate or inappropriate samples were not observed. Suspicious areas in elastography in these patients had high indices above 80 kPa, while non-suspicious areas were below 40 kPa. All these patients were diagnosed with papillary thyroid cancer.
**Conclusion:** Thyroid nodules have always been a significant challenge. Shear wave elastography presents a new criterion for better diagnosis of suspicious nodules. Target thyroid FNA as a new concept should receive attention.
**Limitations:** One of the limitations of this study is the necessity of a highly skilled radiologist who can accurately sample high-risk areas.
**Funding for this study:** No Funding is received.
**Ethics committee - additional information:** This research has been reviewed and approved by the National Ethics Committee under the number IR.IUMS.FMD.REC.1399.177
**Author Disclosures:**
Mohammadreza Khaleghi: Nothing to disclose
Alireza Aziz Ahari: Nothing to disclose

### Comparison of radiofrequency ablation and microwave ablation in the treatment of benign thyroid nodules

*P. Glielmo*, G. Mauri, D. Albano, S. Gitto, S. Fusco, L. M. Sconfienza; Milan/IT

**Purpose or Learning Objective:** Radiofrequency ablation (RFA) and microwave ablation (MWA) are minimally invasive techniques used to treat benign thyroid tumours. This study aims to compare the efficacy and safety of these two methods.
**Methods or Background:** We retrospectively evaluated all patients with benign thyroid nodules treated with either RFA or MWA at our Istitution between January 2021 and December 2021. The primary outcomes assessed were the volume reduction rate (VRR) of the ablated areas at 1, 6, 12 and 24 months, procedure duration, and complication rates.
**Results or Findings:** A total of 56 patients were enrolled, 35 treated with RFA and 21 with MWA. At 1 month, the VRR was 57% in the MWA group and 48% in the RFA group (p=0.045). At 6 months, both groups showed a VRR of 72%. At 12 months, the VRR was 75% in the MWA group and 76% in the RFA group and at two years of 76% in MWA group and 75% in RFA group. Both techniques achieved significant volume reduction with no major complications reported.
**Conclusion:** Both RFA and MWA are effective and safe for treating benign thyroid nodules. MWA demonstrated a higher initial VRR at 1 month, while both techniques achieved similar efficacy at 6, 12 and 24 months. These findings support the use of either method as viable non-surgical alternatives for patients seeking treatment options for benign thyroid nodules.
**Limitations:** Limitations of this study include its retrospective design, relatively small sample size, and absence of a control group for direct comparison.
**Funding for this study:** None
**Ethics committee - additional information:** "This study was approved by the Ethical Committee of IRCCS Ospedale Galeazzi - Sant'Ambrogio, and all participants provided informed consent in accordance with the Declaration of Helsinki."
**Author Disclosures:**
Pierluigi Glielmo: Nothing to disclose
Stefano Fusco: Nothing to disclose
Salvatore Gitto: Nothing to disclose
Luca Maria Sconfienza: Nothing to disclose
Giovanni Mauri: Nothing to disclose
Domenico Albano: Nothing to disclose

### Image-guided thermal ablation as an alternative to surgery for papillary thyroid microcarcinoma, a 7-year experience

G. Mauri, *P. Glielmo*, D. Albano, S. Gitto, S. Fusco, L. M. Sconfienza; Milan/IT

**Purpose or Learning Objective:** Thermal ablation has emerged as a minimally invasive treatment option for thyroid microcarcinomas, offering an alternative to surgery. This study presents a 7-year experience (2018-2024) in treating thyroid microcarcinomas with thermal ablation.
**Methods or Background:** We retrospectively evaluated all patients who underwent US-guided thermal ablation for thyroid microcarcinomas between January 2018 and September 2024. We assessed the technical efficacy of the procedure, complications, and local or distant recurrences.

**Results or Findings:** Of the 59 patients referred for evaluation, 5 were deemed unsuitable for thermal ablation, and 1 opted for surgery. The remaining 53 patients (40 females, 13 males; mean age 51.4 ± 7.7 years) underwent thermal ablation successfully. The procedure was well-tolerated with no major adverse events. The follow-up period ranged from 71 to 2 months (mean 36 months). No local or distant recurrences occurred.
**Conclusion:** Thermal ablation is a safe and effective treatment for thyroid microcarcinomas, providing a viable alternative to surgery. This 7-year experience supports its role as a standard treatment option for thyroid microcarcinomas, demonstrating excellent outcomes in terms of safety and efficacy in tumour control.
**Limitations:** Limitations of this study include its retrospective design, relatively small sample size, and absence of a control group for direct comparison.
**Funding for this study:** None
**Ethics committee - additional information:** This study was approved by the Ethical Committee of IRCCS Ospedale Galeazzi - Sant'Ambrogio, and all participants provided informed consent in accordance with the Declaration of Helsinki.
**Author Disclosures:**
Pierluigi Glielmo: Nothing to disclose
Stefano Fusco: Nothing to disclose
Salvatore Gitto: Nothing to disclose
Luca Maria Sconfienza: Nothing to disclose
Giovanni Mauri: Nothing to disclose
Domenico Albano: Nothing to disclose

### Transarterial Embolization Outperforms Radiofrequency Ablation for Thyroid Goiters Exceeding 100 mL: A Study on Efficacy and Safety

W-C. Lin, *Y. J. Lee*, C-K. Wang, A-N. Lin, Y-S. Chen, C. Y. Lee, P-L. Chiang, C. Y. Lu; Kaohsiung City/TW
*(wesly128@cgmh.org.tw)*

**Purpose or Learning Objective:** This study aims to compare the efficacy and safety of radiofrequency ablation (RFA) and transarterial embolization (TAE) in managing large benign thyroid nodules (BTNs), particularly those exceeding 100 mL, where established guidelines are limited.
**Methods or Background:** This retrospective multicenter study, conducted from January 2018 to May 2022, included 70 patients with a total of 76 large BTNs. Of these, 53 underwent RFA and 17 underwent TAE. Nodules were categorized by initial volume (<50 mL, 50–100 mL, >100 mL) and diameter (<6 cm, 6–9 cm, >9 cm). Treatment efficacy was evaluated using the volume reduction rate (VRR) at 6 months. Complications, as well as improvements in symptom and cosmetic scores, were documented and analyzed.
**Results or Findings:** At 6 months, TAE demonstrated a significantly higher mean VRR than RFA ($p = 0.007$), especially for nodules larger than 100 mL (TAE: 63.34% vs. RFA: 49.71%; $p = 0.035$). The complication rate in the TAE group (5.88%) was lower than that in the RFA group, where transient hoarseness and hematoma were common complications. Both treatments resulted in significant improvements in symptom and cosmetic scores ($p < 0.001$), with TAE providing greater improvements in larger nodules.
**Conclusion:** TAE is more effective and has fewer complications than RFA for the treatment of large BTNs exceeding 100 mL. These findings suggest that TAE may serve as a minimally invasive alternative to surgery for patients with large thyroid nodules. Further prospective studies are necessary to develop size-specific guidelines for selecting between RFA and TAE.
**Limitations:** Our study's limitations include the absence of gross pathological confirmation, retrospective design, short-term follow-up, and variations in TAE protocols.
**Funding for this study:** No funding was received for this article.
**Ethics committee - additional information:** Chang Gung Medical Foundation Institutional Review Board IRB No.: 202401138B0
**Author Disclosures:**
Yun Ju Lee: Nothing to disclose
Cheng-Kang Wang: Nothing to disclose
Wei-Che Lin: Nothing to disclose
Pi-Ling Chiang: Nothing to disclose
Chia Yin Lu: Nothing to disclose
Yueh-Sheng Chen: Nothing to disclose
An-Ni Lin: Nothing to disclose
Chih Ying Lee: Nothing to disclose

**11:30-12:30 Research Stage 4**

### Research Presentation Session: Musculoskeletal

### RPS 310

### Selected applications of MSK ultrasound

**Moderator**
F. Zaottini; Genoa/IT
*(federico.zaottini.fz@gmail.com)*

### Incidence of Palmar Fibromatosis Nodules Following Carpal Tunnel Release: A Prospective High-Resolution Ultrasound Study

*S. A. Jengojan*[1], A. Piacentini[1], F. P. Papa[1], V. König[1], D. Albano[2], Ž. Snoj[3], G. Ivanac[4], G. Bodner[1]; [1]Vienna/AT, [2]Milan/IT, [3]Ljubljana/SI, [4]Zagreb/HR
*(suren.jengojan@meduniwien.ac.at)*

**Purpose or Learning Objective:** Palmar fibromatosis (PF), also known as Dupuytren's disease (DD), is characterized by fibrous nodules and thickening of the palmar fascia, potentially leading to functional impairment of the hand. While its etiology remains unclear, we have observed over and over again in our daily routine that surgical procedures, such as carpal tunnel syndrome (CTS) release may trigger the development of fibromatosis in the palmar Fascia. In this study we investigated whether patients who undergo CTS surgery are more likely to develop palmar fibromatosis nodules, compared to healthy individuals and those with non-operated CTS, using high-resolution ultrasound (HRUS) imaging.
**Methods or Background:** In this prospective study, we examined 100 patients who had previously undergone CTS surgery, screening for palmar fibromatosis nodules with HRUS. We further evaluated two control groups: 50 healthy volunteers and 30 patients diagnosed with CTS who had not yet had surgery. The prevalence of PF nodules across these groups was documented and compared.
**Results or Findings:** Out of the 100 patients who had CTS surgery, 35% (n=35) were found to have PF nodules. In contrast, only 1 individual in the group of healthy volunteers had nodules 2% (n=1). None of the patients with non-operated CTS showed evidence of nodules. These findings suggest a highly significant increase in PF among post-CTS surgery patients compared to both healthy controls and those with non-operated CTS.
**Conclusion:** Our findings suggest that CTS release surgery may be linked to a higher incidence of DD. This raises important questions about the role of surgical intervention in the development of fibromatosis.
**Limitations:** A limitation of our study is the lack of preoperative imaging, which could not exclude the possibility that the palmar fibromatosis nodules already existed before CTS surgery.
**Funding for this study:** No funding was needed
**Ethics committee - additional information:** The study was approved by the Institutional Review Board. (vote Number 2028/2024) approved the study.
**Author Disclosures:**
Alessio Piacentini: Nothing to disclose
Gerd Bodner: Nothing to disclose
Francesco Pio Papa: Nothing to disclose
Suren Armeni Jengojan: Nothing to disclose
Gordana Ivanac: Nothing to disclose
Žiga Snoj: Nothing to disclose
Viktoria König: Nothing to disclose
Domenico Albano: Nothing to disclose

### The effect of soft tissue compression on shear wave velocity of peripheral nerves

J. Peterca, Ž. Snoj, *G. Omejec*; Ljubljana/SI
*(gregor.omejec@gmail.com)*

**Purpose or Learning Objective:** The objective was to determine the effect of soft tissue compression applied by the US probe on peripheral nerve shear wave velocity (SWV) measurements
**Methods or Background:** Lower and upper limbs of 20 fresh cadavers were dissected at hip and shoulder region, strapped to inox tray and emerged into the 37°C water bath. The US probe was mounted on robotic arm Yaskawa MH6 (Yaskawa Electric, Fukuoka, Japan). Median nerve at wrist and forearm, and sciatic nerve just proximal to tibial and fibular nerve branching were scanned longitudinally using US Resona R9 (Mindray Bio-medical Electronics, Shenzhen, China) and L14-3WU transducer. The compression of the soft tissue applied by US probe was gradually increased from no contact to 10mm (wrist), 20mm (forearm), and 30mm (posterior thigh) in successive steps of 1,

Wednesday

2, 3, 5, 10, 15, 20, 25 and 30mm. After every successive step, SWV was measured three times and median was used for statistical analysis.
**Results or Findings:** Average distances between the US probe and peripheral nerve at baseline were 5.4mm for median nerve at the wrist, 18.9mm for median nerve at the forearm, and 24.8mm for sciatic nerve. For median nerve at wrist, significant changes in SWVs were found for compression distances of ≥2mm, and for median nerve at forearm for compression distances of ≥15mm ($p <0.05$). No changes in SWV were found for sciatic nerve.
**Conclusion:** The influence of soft tissue compression on SWV measurements depends on the thickness of the soft tissue between the probe and peripheral nerve. No or only minor pressure is advised when measuring SWV of superficial peripheral nerves and more pressure can be applied when measuring SWV of deeper peripheral nerves.
**Limitations:** Possible small changes in ROI position after robotic arm movement
**Funding for this study:** The authors report grants from The Republic of Slovenia Research Agency (Grant No. P3-0338 and J3-4507)
**Ethics committee - additional information:** The study was approved by the National Medical Ethics Committee of Slovenia (Nr. 0120-25/2023/3).
**Author Disclosures:**
Gregor Omejec: Nothing to disclose
Jošt Peterca: Nothing to disclose
Žiga Snoj: Nothing to disclose

**Dominance differences in hamstring stiffness among athletes: Insights from ultrasound shear wave elastography**
*B. S. Alvarez De Sierra*, P. Nieto; Madrid/ES

**Purpose or Learning Objective:** Hamstring injuries are prevalent among football players, necessitating a deeper understanding of muscle stiffness and its implications. This study investigates the differences in hamstring stiffness between dominant and non-dominant legs in athletes using Shear Wave Elastography (SWE).
**Methods or Background:** A retrospective study was conducted involving 30 football players (15 males, 15 females). Ultrasound SWE was used to measure shear wave velocities (SWV) in the semitendinosus, semimembranosus, and biceps femoris muscles. Data were analyzed based on leg dominance and sex, with statistical significance set at $p < 0.05$.
**Results or Findings:** Significant differences were found in semitendinosus stiffness between sexes, particularly in the non-dominant leg ($p = 0.02$). Males exhibited higher SWV in the semitendinosus muscle compared to females, with mean SWV of 3.8 m/s (dominant) and 4.1 m/s (non-dominant) for males versus 2.9 m/s (dominant) and 2.8 m/s (non-dominant) for females. No significant SWV differences were observed in the semimembranosus and biceps femoris muscles between sexes or based on leg dominance.
**Conclusion:** SWE effectively identifies dominance-related differences in hamstring stiffness, offering valuable insights for optimizing athletic performance and reducing injury risks. Future research should explore the implications of these findings on long-term injury prevention and rehabilitation strategies.
**Limitations:** The sample size was small (30 players), the exact degree of stiffness remains uncertain without a histological correlation serving as a standard of reference for the hamstrings. Other factors such as muscle geometry, fiber orientation, and intramuscular pressure may influence SWV.
**Funding for this study:** No funding
**Ethics committee - additional information:** The study was approved by the Clinical Research Ethics Committee. CEIC 2024.232
**Author Disclosures:**
Patricia Nieto: Nothing to disclose
Beatriz Sierra Alvarez De Sierra: Nothing to disclose

**Ultrasound-Guided Interphalangeal Injection (US-IPI) of Mucoid Cysts: Technical Notes and Clinical Efficacy**
E. Faiella, *E. Vergantino*, D. Santucci, A. Bruno, G. Pacella, R. F. Grasso; Rome/IT
*(elva.vergantino@unicampus.it)*

**Purpose or Learning Objective:** Digital mucous cysts (DMCs) are common soft tissue tumors affecting interphalangeal joints. Various treatment options exist, with surgical excision being the standard. Ultrasound-guided cortisone injection into the distal interphalangeal (US-IPI) joint has been proposed as a therapeutic alternative. This study aims to assess the technical success and clinical efficacy of US-IPI in terms of swelling resolution and pain control.
**Methods or Background:** This study assessed corticosteroid infiltration for mucoid cysts in interphalangeal joints. Fifty-two patients (16 males, 36 females; median age 53, range 45-73) were treated between January 2020 and March 2023. Inclusion criteria included joint swelling, growth, and chronic pain >3 months; infections were excluded. The ultrasound-guided procedure used a 26-gauge needle and Triamcinolone acetonide (Kenacort). Pain and swelling were evaluated via the Numeric Rating Scale (NRS) at 2 weeks, 1 month, 3 months, and 6 months. Statistical analysis was performed using SPSS (v.22)
**Results or Findings:** The study assessed pain relief from ultrasound-guided corticosteroid infiltration for mucoid cysts. NRS scores decreased significantly from a median of 6.8 pre-procedure to 3.2 at two weeks ($p < 0.01$) and to 0 by one month ($p < 0.01$). Eighty-three percent of patients responded positively; 17% experienced persistent pain (average NRS 6.2). A secondary infiltration reduced their scores to 3.5 at two weeks and 0 by one month ($p < 0.01$). For joint swelling, 68% had a 50% reduction within one month, with complete resolution by three months. No recurrences were observed at the six-month follow-up.
**Conclusion:** Ultrasound-guided injection for digital mucous cysts offers effective pain relief and aesthetic improvement. Future studies should assess long-term outcomes and compare efficacy with other treatments.
**Limitations:** The lack of extended follow-up beyond one year post-treatment limits the assessment of long-term outcomes.
**Funding for this study:** No funding.
**Ethics committee - additional information:** The study was conducted according to the guidelines of the Declaration of Helsinki. Ethical review and approval were waived for this study due to its retrospective nature.
**Author Disclosures:**
Rosario Francesco Grasso: Nothing to disclose
Amalia Bruno: Nothing to disclose
Eliodoro Faiella: Nothing to disclose
Elva Vergantino: Nothing to disclose
Giuseppina Pacella: Nothing to disclose
Domiziana Santucci: Nothing to disclose

**Clinical and Radiological Outcomes of Ultrasound Guided Closed- Circuit Irrigation of Calcific Tendinitis of the Shoulder: a prospective study**
*A. De Grandis*[1], C. D'Alessandro[1], G. Sussan[1], A. Crimì[1], D. Coraci[1], S. Masiero[1], R. Ragazzi[2], E. Quaia[1], F. Crimì[1]; [1]Padova/IT, [2]Venice/IT
*(andrea.degrandis96@gmail.com)*

**Purpose or Learning Objective:** Ultrasound-guided percutaneous treatments are a recognized and effective option for calcific tendinopathy of the shoulder. In this study, we enhanced the standard double-needle technique with a closed-circuit irrigation system and evaluated the clinical/radiological outcomes of the procedure.
**Methods or Background:** We prospectively enrolled 24 patients (14 females; median age 54years, IQR:50-62) with painful calcific tendinopathy of the shoulder between October 2023 and March 2024. All patients had a calcification >5 mm treated with ultrasound-guided closed-circuit irrigation, and the procedure duration was recorded. Ultrasound, radiography evaluation, and OSS and SPADI clinical questionnaires were administered before and 3 months after the procedure.
**Results or Findings:** After the procedure, there was a significant reduction in the size of the calcifications (12 mm, IQR:10-20 mm vs. 5.5 mm, IQR:2-10 mm; $p=0.0001$). The median duration of the procedure was 41 minutes (IQR:39-45 minutes). After the procedure, none of the patients experienced infections while two developed bursitis. There was a significant improvement in the OSS score 3 months after the procedure (16.5, IQR:10-23 vs. 32, IQR:36-45.5; $p<0.0001$) and a significant reduction in SPADI scores: pain (88, IQR:74-95 before vs. 13, IQR:4-24; $p<0.0001$), disability (72, IQR:60-90 before vs. 8, IQR:4-20; $p<0.0001$), and total score (78, IQR:66-91 before vs. 11, IQR:4-20; $p<0.0001$). The improvement of SPADI total score was higher, although not significantly ($p=0.2891$), compared to a 2015 review.
**Conclusion:** The closed-circuit double-needle barbotage for calcific tendinopathy of the shoulder is an effective treatment that improves both shoulder pain and function with a very low risk of short-term complications.
**Limitations:** No limitations were identified.
**Funding for this study:** No founding were received for this study.
**Ethics committee - additional information:** CET-ACEV code: 471n/AO/24
**Author Disclosures:**
Giovanni Sussan: Nothing to disclose
Carlo D'Alessandro: Nothing to disclose
Daniele Coraci: Nothing to disclose
Roberto Ragazzi: Nothing to disclose
Filippo Crimì: Nothing to disclose
Alberto Crimì: Nothing to disclose
Emilio Quaia: Nothing to disclose
Andrea De Grandis: Nothing to disclose
Stefano Masiero: Nothing to disclose

**Real-Time Elastosonography of the Achilles Tendon and Plantar Fascia: Predictive Insights for Diabetic Foot Ulcers**

V. Burulday[1], S. Ceylan Durmaz[2], A. Gungunes[3], *A. Tezcan*[1]; [1]Malatya/TR, [2]Ankara/TR, [3]Kirikkale/TR
*(alperentezcan@hotmail.com)*

**Purpose or Learning Objective:** This study aims to evaluate the plantar fascia and Achilles tendon in patients with diabetic foot ulcers using two elastosonography methods: strain elastography (SE) and shear wave elastography (SWE). The goal is to identify biomechanical changes in these structures, which may predict the risk of diabetic foot ulcer development. This study is the first in the literature to evaluate both the Achilles tendon and plantar fascia together using both SE and SWE methods.
**Methods or Background:** Twenty-five patients with type 2 diabetes and diabetic foot ulcers, along with 30 healthy individuals, were evaluated. B-mode ultrasound, SE, and SWE methods were used to assess the thickness and stiffness of the Achilles tendon and plantar fascia. Diabetic foot ulcers were staged, and measurements were performed by a single experienced radiologist. The study compared patients with diabetic ulcers to those without, analyzing changes in tissue stiffness and structure.
**Results or Findings:** The study found significant increases in Achilles tendon thickness and stiffness in diabetic foot patients compared to the control group ($p<0.0001$). Plantar fascia stiffness was also significantly higher in diabetic patients ($p<0.0001$). Subgroup analysis revealed that patients with foot ulcers had even greater Achilles tendon and plantar fascia stiffness than those without ulcers. No significant difference in plantar fascia thickness was observed between groups ($p=0.539$), but stiffness changes were evident.
**Conclusion:** The elastosonographic evaluation of the Achilles tendon and plantar fascia provides valuable insights into the biomechanical changes in diabetic foot patients. Both SE and SWE are complementary methods that may help predict the development of diabetic foot ulcers. Early detection of stiffness and tissue changes through these methods can be crucial in preventing ulcer formation.
**Limitations:** Operator dependence, lack of MRI comparison, small sample size, absence of reliability assessment
**Funding for this study:** None
**Ethics committee - additional information:** Decision number 15/03 dated 01.10.2018
**Author Disclosures:**
Alperen Tezcan: Nothing to disclose
Askin Gungunes: Nothing to disclose
Veysel Burulday: Nothing to disclose
Senay Ceylan Durmaz: Nothing to disclose

| 11:30-12:30 | Room G1 |
|---|---|

### Research Presentation Session: Radiographers

### RPS 314

### Innovative imaging practices and patient-centred care: radiographers at the forefront of diagnostic excellence

**Moderators**
C. Messina; Milan/IT
H. Precht; Middelfart/DK
*(hepr@ucl.dk)*
**Author Disclosures:**
Carmelo Messina: Grant Recipient: Bracco Imaging Italia, Echolight

**Analysis of a focus group survey on person-centred care (PCC) practices in Nuclear Medicine: insights from the European Congress of Radiology 2024**

*P. S. Costa*[1], D. Fonseca Ribeiro[2], M. Champendal[3], S. Murphy[4], C. Baun[5], C. Andersson[6], A. Karangelis[7], K. Borg Grima[8], A. Geão[9]; [1]Porto/PT, [2]London/UK, [3]Lausanne/CH, [4]Dublin/IE, [5]Odense/DK, [6]Uppsala/SE, [7]Patra/GR, [8]Naxxar/MT, [9]Lisbon/PT
*(psc@ess.ipp.pt)*

**Purpose or Learning Objective:** This study aimed to gather insights into the understanding, implementation, and challenges of Person-Centered Care (PCC) practices among Nuclear Medicine professionals in Europe. Additionally, the focus group validated the questions used in the survey, serving as the ground work for a forthcoming European-wide study involving Nuclear Medicine Radiographers.
**Methods or Background:** A focus group survey was conducted during the European Congress of Radiology 2024. The participants included Radiographers and Nuclear Medicine Radiographers/Technologists from various European countries. The survey covered demographics, professional background, understanding of PCC, its application in clinical settings, and factors aiding or hindering its implementation. The focus group also provided some informal feedback to validate the survey questionnaire for future use.
**Results or Findings:** Thirty-two participants participated in this focus group and contributed to the preliminary survey, with n=5; 45% Radiographers and n=6; 55% NM Radiographers/Technologists . Most participants had between 1-10 years of experience. Participants were from Albania (9%), Italy (27%), Denmark (36%), Malta (9%), and Portugal (3%). Reported Key factors aiding PCC implementation included good communication (45%), empathy (40%), and sensitivity to patient characteristics (36%). The reported barriers to the implementation of PCC included burnout (82%), complexity of procedures (36%), and time constraints (27%). Additionally, the focus group provided feedback on the questions used while aiding to improve the survey set-up.
**Conclusion:** The findings revealed a strong recognition of the importance of PCC among Nuclear Medicine professionals, but also significant challenges such as burnout and time constraints which could hinder its implementation. Recommendations included, amongst others, the use of the validated questionnaire to gain a broader understanding of PCC practices in Nuclear Medicine across a wider spectrum of European countries.
**Limitations:** The small sample size and the limited geographical spread of the participants.
**Funding for this study:** No funding was received for this study.
**Ethics committee - additional information:** Not applicablw
**Author Disclosures:**
Daniela Fonseca Ribeiro: Nothing to disclose
Karen Borg Grima: Nothing to disclose
Apostolos Karangelis: Nothing to disclose
Ana Geão: Nothing to disclose
Camilla Andersson: Nothing to disclose
Christina Baun: Nothing to disclose
Pedro Silva Costa: Nothing to disclose
Shauna Murphy: Nothing to disclose
Mélanie Champendal: Nothing to disclose

**Comparison of Virtual Monoenergetic Images of a clinical Photon-Counting-Detector CT with a state-of-the-art Energy-integrated-Detector CT in Dual-Energy Mode**

*M. Scheweder*[1], D. Ecker[1], E. Wurzinger[2], A. Gratzer[2], P. Kullnig[2]; [1]Linz/AT, [2]Graz/AT
*(mario.scheweder@fhgooe.ac.at)*

**Purpose or Learning Objective:** Photon-counting detector computed tomography (PCD-CT) is a promising novel technique for clinical CT, with new opportunities for image optimization while reducing radiation dose compared to conventional energy-integrated detector CT (EID-CT). Therefore, a PCD-CT and an EID-CT were compared to assess the image quality on spectral data and image reconstructions. The goal was to explore technical potentials for clinical practice.
**Methods or Background:** A whole-body phantom was scanned on an EID-CT in Dual-Energy (DE) Mode and a PCD-CT with similar CTDIvol. Virtual monoenergetic images (VMI) were processed at 16 keV levels (40-190 keV) and different reconstructions. Signal-to-noise Ratio (SNR) and contrast-to-noise ratio (CNR) ROIs were evaluated from liver equivalent tissue for each level and reconstruction. Mann-Whitney U test was used to compare image quality. Besides, a dose-reduced PCD-CT scan was compared to the EID-CT scan.
**Results or Findings:** PCD-CT-VMI data show significantly higher (all $p<.05$) SNR and CNR (all $p<.001$) than EID-CT across all keV levels and reconstruction methods. However, SNR and CNR highly depended on the reconstruction method and keV level. PCD-CT SNR/CNR exceeded ≥80 keV (SNR: +7% to +502% / CNR: +3% to +801%). A 40% dose-reduced PCD-CT scan provided higher dose-normalized SNR against the EID-CT scan at 100-190 keV (+35% to +272%). However, at 40-90 keV, PCD-CT had lower SNR (-53% to -1%).
**Conclusion:** PCD-CT-VMI demonstrate higher SNR/CNR capabilities compared to EID-CT in DE-Mode. This advantage can be used to optimize scan setups regarding radiation exposure. Few studies have compared the VMI data of PCD-CT and EID-CT for this purpose. However, in addition to these promising objective results, subjective image assessment by radiologists is necessary to clarify diagnostic accuracy.
**Limitations:** This project is a phantom study and requires further clinical research to apply findings in practice.
**Funding for this study:** This project was internally funded by the University of Applied Sciences for Health Professions Upper Austria. Project-Number: P-2002-003

**Ethics committee - additional information:** The project was evaluated by the core team of the Institutional Review Board of the University of Applied Sciences for Health Professions Upper Austria, with the following conclusion: "There are no objections to the execution of this study in its current form". IRB-Number.: A-2022-018
**Author Disclosures:**
Peter Kullnig: Nothing to disclose
Alexander Gratzer: Nothing to disclose
Eric Wurzinger: Nothing to disclose
Mario Scheweder: Nothing to disclose
Dominik Ecker: Author: DE is an employee at Siemens Healthineers. During the course of this work, he was a master's student at the University of Applied Sciences for Health Professions Upper Austria and worked on the project.

**Radiographer-operated urgent diagnostic imaging in hybrid mobile stroke unit: a pilot technical study in challenging conditions**
*D. Pakizer*[1], A. Chalánková[2], R. Líčeník[3]; [1]Ostrava/CZ, [2]Olomouc/CZ, [3]Peterborough/UK
*(davidpakizer@gmail.com)*

**Purpose or Learning Objective:** Diagnostic assessment shift and treatment at emergency site proved beneficial for acute stroke or other neurological patients by using hybrid-mobile stroke unit (h-MSU) ambulance with mobile computed tomography (CT), portable ultrasound (US), and telemedicine onboard. We aimed to determine feasibility, safety, and efficacy of radiographer-operated h-MSU CT/US in advanced prehospital work-up for patients with acute neuroemergencies in challenging geographical/weather conditions.
**Methods or Background:** In our pilot prospective open-label cohort study, h-MSU was available constantly for 19 consecutive days (November/December 2023) in Czechia mountain/rural region. Patients were examined by CT intracranially in standby ambulance. Extracranial carotid US underwent selected stable patients with time of transport >30min to stroke center (ambulance on move). The h-MSU CT/US efficacy was compared with standard in-hospital modalities; feasibility and safety were assessed.
**Results or Findings:** Of 46 patients, 37 brain CT, 1 intracranial CT angiography, and 8 extracranial carotid US examinations were conducted. CT helped find contraindications for thrombolysis in 3 patients; 6 patients received the treatment. Of 106 CT scans, only 3% of full examinations had to be repeated. Mobile-CT mean radiation dose was only slightly higher compared to in-hospital CT, findings and image quality were similar. Low temperatures and uneven mountainous terrain were responsible for 4% of repeat CT scans. Good-quality US images were achieved and no hemodynamically significant stenosis was found but approximately 15min extracranial carotid examination was needed. Moreover, 2 US-guided cannulations were performed.
**Conclusion:** Mobile-CT and portable US onboard of h-MSU were safe, feasible, and effective modalities for neuroemergency patients and proved to be beneficial for time reduction and faster treatment decision-making in challenging geographical/weather conditions.
**Limitations:** Older age of mobile-CT/US, CT contrast agent only for study second-half, low patient number examined by US, and lack of Doppler US.
**Funding for this study:** None.
**Ethics committee - additional information:** Tomas Bata Hospital Zlin Ethics Committee (approval 2023-66)
**Author Disclosures:**
Anežka Chalánková: Nothing to disclose
Radim Líčeník: Nothing to disclose
David Pakizer: Nothing to disclose

**Optimisation in CT using tin filtration: A systematic Review**
*A. Bellizzi*, J. L. Portelli, P. Bezzina, G. Galea, F. Zarb; Msida/MT
*(bellizziandrea22@gmail.com)*

**Purpose or Learning Objective:** To identify which non-contrast CT examinations benefit from image quality and radiation dose optimisation using tin filtration (TF) and which optimisation strategy is best suited for this purpose.
**Methods or Background:** The review was registered in PROSPERO, and used PICO to create the research question, and exclusion/inclusion criteria. From the research question, MeSH search terms were obtained and inputted into five electronic databases: PUBMED, Scopus, CINAHL complete, Cochrane Central Register of Controlled Trials and Health & Medical Collection. Studies identified from the search were loaded into Covidence and reviewed by a team of 3 experts using PRISMA guidelines. The Joanna Briggs Institute (JBI) critical appraisal tool was used to evaluate the studies while data extraction was performed using a self-designed validated tool.
**Results or Findings:** From the retrieved 1479 studies, 410 were found to be duplicates leaving 1069 studies for title/abstract screening. Subsequently, 130 studies were included for full text-review, with a final 84 studies included for evaluation. TF was used to optimise scanning in 14 protocols. Scan parameters used in conjunction with TF as an optimisation strategy were: iterative reconstruction (IR) level, tube voltage (kV), pitch, rotation time and reference mAs. Use of TF achieved a significant dose reduction ranging from 17-95% in all protocols.
**Conclusion:** TF is an efficient dose reduction technique in non-contrast CT examinations, but has limitations meriting consideration in terms of objective image quality. These limitations could potentially be solved by varying the IR level, however more studies are needed for this to be confirmed.
**Limitations:** Six studies could not be retrieved. A meta-analysis could not be conducted due to the heterogeneity of the studies. Paediatrics and CT protocols using IVCM were excluded.
**Funding for this study:** No funding was received for this study.
**Ethics committee - additional information:** None required - systematic review.
**Author Disclosures:**
Gabriel Galea: Nothing to disclose
Jonathan Loui Portelli: Nothing to disclose
Francis Zarb: Nothing to disclose
Andrea Bellizzi: Nothing to disclose
Paul Bezzina: Nothing to disclose

**The use of Ultra-Low Dose Computed Tomography in the diagnosis of Suspected Physical Abuse in paediatric patients - A phantom study**
E. K. Mahon, A. A. Mohammed, A. England, R. Young, *N. Moore*, G. A. Curran, M. F. Mcentee; Cork/IE
*(niamh.moore@ucc.ie)*

**Purpose or Learning Objective:** Suspected physical abuse (SPA) poses a global threat to children, particularly those under the age of two. Current practices utilise conventional radiography skeletal surveys (SSs) to help with the diagnosis and management of SPA. The recent exploration of ultra-low dose CT (ULDCT) could improve diagnostic accuracy of SPA whilst minimising radiation exposure and thus is the focus of this study.
**Methods or Background:** CT datasets were acquired on a paediatric phantom using ULDCT (DLP=1.49 mGycm2) and standard-dose (DLP=22.92 mGycm2) CT protocols. Participants (radiographers and radiologists) subjectively scored the image quality (IQ) of both protocols using a 5-point Likert scale. Mann-Whitney U tests assessed significant differences in IQ between protocols. Participants also estimated radiation dose differences and confidence in diagnosing SPA using the CT datasets provided.
**Results or Findings:** Responses from 46 participants were included. Data were categorised into four anatomical areas; head/neck, thorax, abdomen/pelvis and extremities. IQ scores were consistently higher for STD when compared to ULDCT (H&N 2.7 vs 2.1; Thorax 2.8 vs. 2.2; Abdo/Pelvis 2.8 vs. 2.0 and Extremities 2.9 vs. 2.2; $p<0.05$). Importantly, the ULDCT protocol scored highest in "Borderline acceptable" for all regions. Interestingly, 45 (97.8%) participants underestimated the dose difference (1.49 versus 22.92 mGycm2) between the two protocols. The ULDCT protocol scored a total of 38% (88/230) confidence for the diagnosis of SPA, whereas the STD protocol scored a total of 70% (161/230) confidence.
**Conclusion:** Despite the IQ difference between protocols, ULDCT results are promising. Results may suggest that with adjustments to the ULD protocol 'optimisation' CT could contribute further SPA diagnosis but further research, including clinical studies, are needed.
**Limitations:** Phantom-based study.
**Funding for this study:** None.
**Ethics committee - additional information:** Medical School Social Research Ethics Committee - University College Cork
**Author Disclosures:**
Mark F. Mcentee: Nothing to disclose
Niamh Moore: Nothing to disclose
Rena Young: Nothing to disclose
Ahmed Abdulahad Mohammed: Nothing to disclose
Gráinne Alison Curran: Nothing to disclose
Andrew England: Advisory Board: RoClub Research/Grant Support: GE Healthcare, Organon Pharma Ltd. Board Member: EFRS
Eimear Kate Mahon: Nothing to disclose

**Deep-learning based image reconstruction in body CT imaging: What is the real gain? A quantitative study**
*D. Delarbre*[1], E. Maturana[1], M. Scheffler[1], J. L. Navarro Quirante[1], D. Racine[2]; [1]Geneva/CH, [2]Lausanne/CH
*(david.delarbre@hug.ch)*

**Purpose or Learning Objective:** Compare the performance between deep learning-based Advanced intelligent Clear-IQ Engine (AICE), iterative Adaptive Iterative Dose Reduction (AIDR-3D) and filtered back projection (FBP) computed tomography (CT) image reconstruction algorithms in terms of image texture, low-contrast lesion detectability, and dose reduction potential.
**Methods or Background:** An abdominal anthropomorphic phantom was scanned at five computed tomography dose index (CTDIvol) level settings:

10.3, 6.4, 3.3, 2.4, and 1.9 mGy. Images were reconstructed using AICE, kernel “Body Sharp”, 1mm slice thickness, then AIDR-3D, kernel “FC08”, 1mm and 2mm slice thicknesses, and classic FBP including quantum denoising system (QDS+) reconstruction. Noise and contrast-dependent spatial resolution were assessed through noise power spectra (NPS) and target transfer functions (TTF). Texture similarity of these algorithms was evaluated using peak frequency difference (PFD) and root mean square deviation (RMSD). Lesion detectability was quantified using a non-prewhitening (NPW) observer model with eye filter. The area under the curve (AUC) and receiver operating characteristic (ROC) served as the figure of merit (FOM). Dose reduction potential for AICE 1 mm, compared to AIDR-3D with 2mm slice thickness, was calculated to achieve equivalent AUC values.
**Results or Findings:** At higher dose levels, AIDR-3D better preserved FBP-like noise texture. At lower doses, this difference diminished. The PFD for AIDR-3D ranged from -0.05 to -0.14, while for AICE it ranged from -0.13 to -0.17. RMSD values followed a similar trend. AICE consistently achieved higher AUC values than AIDR-3D with 1mm slice thickness, with an increasing difference as dose decreased. AICE especially demonstrated a radiation dose reduction potential of up to 45% compared to AIDR-3D with 2mm slice thickness.
**Conclusion:** AICE provides equivalent low-contrast lesion detectability at significantly reduced radiation doses compared to AIDR-3D, without adversely affecting noise texture.
**Limitations:** Constant kV, phantom morpho-type M
**Funding for this study:** 0
**Ethics committee - additional information:** None
**Author Disclosures:**
Damien Racine: Nothing to disclose
Jose Luis Navarro Quirante: Nothing to disclose
David Delarbre: Nothing to disclose
Enrique Maturana: Nothing to disclose
Max Scheffler: Nothing to disclose

**The effect of simulated reduced temporal resolution and motion artefacts on CT-derived cardiac left ventricular ejection fraction**
*M. W. Kusk*[1], O. Gerke[2], S. Hess[2], S. J. Foley[3]; [1]Esbjerg/DK, [2]Odense C/DK, [3]Dublin/IE
*(martin.weber.kusk@rsyd.dk)*

**Purpose or Learning Objective:** To test whether mid-range CT-scanners, with low temporal resolution (TR) can reliably measure left ventricular ejection fraction, (LVEF), compared to high-end cardiac scanners.
**Methods or Background:** 77 low-dose functional CT datasets, from a 3rd generation DSCT scanner, with 66 msec (TR) , reconstructed at 5% intervals of the entire cardiac cycle were used Cardiac MRI served as gold standard for classifying patients with potential heart failure (LVEF below 50%). Reduced TR dataset were simulated by temporal averaging between adjacent phases, using a MATLAB script. Furthermore, in 25 artefact-free datasets, we inserted simulated discontinuity artefacts of varying magnitude and location. LVEF was measured on a clinical workstation using standard (ST) and Blood Volume (BV) modes. Absolute LVEF was compared between original and simulated images with Bland-Altman plots and t-tests, while correlation between effective TR was examined wiht Spearman rank-correlation.
**Results or Findings:** For BV-mode, LVEF was not significantly different between original and reduced TR images, (p=0.88) but significantly lower in ST-mode by 2.8% (p<0.01.) However, no patients were reclassified according to the 50% threshold. There was significant negative correlation between LVEF and effective TR in the ST, but not in BV mode. Motion artefacts increased the widths of 95% limits of agreement to 5.6% in ST mode vs 2.8% in BV mode.
**Conclusion:** Halving effective TR did not affect LVEF in the BV-mode . This mode also provided lower dispersion in the presence of motion artafacts, making it the recommended measurement method. Mid-range scanners appear suitable for LVEF measurement, e.g. in conjunction with standard Chest-Abdomen-Pelvis scans.
**Limitations:** The main limitation is the lack of a true reference standard with superior temporal, and similar spatial resolution, thus making the effect of the two factors hard to disentangle.
**Funding for this study:** The study was funded by: The Esbjerg Fund, Research Fund of Danish Radiographers Association, Karola Jørgensen Fund
**Ethics committee - additional information:** Regional
**Author Disclosures:**
Martin Weber Kusk: Nothing to disclose
Søren Hess: Nothing to disclose
Oke Gerke: Nothing to disclose
Shane J Foley: Nothing to disclose

**Patient-tailored contrast optimization in coronary CT angiography on Photon Counting CT**
*T. Busselot*, P. Giordano, W. Coudyzer, H. Bosmans, S. Dymarkowski; Leuven/BE
*(t.busselot@gmail.com)*

**Purpose or Learning Objective:** Standardized injection protocols are often integrated in coronary CTA (cCTA), yet failing to account for patient variability. This study aims to introduce an evidence based injection protocol, achieving a target enhancement (expressed in terms of HU) in the overall patient population.
**Methods or Background:** In a first retrospective study, 162 cCTA patients, scanned on a photon-counting CT, were included. Injection parameters, demographic data and coronary enhancement were retrospectively collected and HU enhancement was measured in the aorta ascendens and proximal RCA using circular ROIs. Using the principle that iodine concentrations and HU correlate linearly, ideal iodine delivery rates (IDR) that would have provided a HU target of 500 in 55keV mono-energetic images were calculated from the HU of the original scans. Linear regression analysis was performed with the different demographic parameters and their ideal IDR, to obtain an evidence based IDR. Secondly, 62 patients were prospectively recruited in an IRB-approved study and injected with newly proposed IDRs, wherefrom volume was calculated. To obtain lowest volumes iodine-saline dilution ratios were used. A minimal total volume of 60ml and fixed injection duration of 17s was defined to ensure complete coronary enhancement. Coronary enhancement was measured in the same ROIs as retrospectively.
**Results or Findings:** Ideal IDRs correlated best with bodyweight adjusted for Deurenberg's fat formula (Pearson r=0.69), with IDR=0.0116x+0.4556 Mean coronary enhancement was 530±99HU (median: 539 and IQR:462 – 599), agreeing with the target HU of 500. Mean injected iodine volume was 51ml (median:51ml and IQR:45–57ml). All low iodine volumes could be administered under the same duration and injection rates (from 3.5–3.8ml/s).
**Conclusion:** A new method for injection protocol optimization, based on linear regression and integrating iodine-saline dilutions, was introduced and achieved the preset target HU enhancement.
**Limitations:** Mono-centric study.
**Funding for this study:** AI-POD project.
HORIZON Action Grant Budget-Based. Grant number 101080302.
**Ethics committee - additional information:** UZ/KU Leuven ethics, internal number S58042
**Author Disclosures:**
Walter Coudyzer: Nothing to disclose
Hilde Bosmans: Nothing to disclose
Tim Busselot: Nothing to disclose
Pierpaolo Giordano: Nothing to disclose
Steven Dymarkowski: Nothing to disclose

**13:00-14:30** **Research Stage 1**

### Research Presentation Session: Breast

### RPS 402

### Advances in breast imaging: innovations shaping the future of cancer care

**Moderator**
R. Woitek; Vienna/AT
*(ramona.woitek@dp-uni.ac.at)*

**18F-fluoroestradiol hybrid imaging in clinical management of breast carcinoma**
*J. Ferda*, E. Ferdova, T. Barakova, S. Vokurka; Plzen/CZ
*(ferda@fnplzen.cz)*

**Purpose or Learning Objective:** 18F-fluoroestradiol is a novel radiopharmaceutical useful in breast carcinoma, the indications in clinical scenarios are under development. The purpose of the study is to assess the clinical impact of the imaging of the breast carcinoma with estrogen-positive receptors (ER+) using 18F-fluoroestradiol (18F-FES) PET/CT or PET/MRI according to its impact to the treatment decision making. The study is concerned in the different preference of PET/CT and PET/MRI in the staging and restaging.
**Methods or Background:** 40 patients with estrogen positive breast carcinoma underwent the hybrid imaging after intravenous application of 18F-FES, in 25 cases it was used PET/CT, in 15 cases PE/MRI. The radiopharmaceutical was

Wednesday injected in the activity of 2,5 MBA/kg and the imaging was performed the imaging. In 10 patient PET/MRI was used as restaging method, PET/MRI was performed in the 5 cases of the staging before surgery with targeted full diagnostic MRI imaging of the breast in prone position, followed by the trunk imaging in supine position, in five to seven steps. All PET/MRI were performed with the gadolinium contrast material, the imaging included brain imaging in T1 STARVIBE. PET/CT was performed using the continuous PET acquisition after CT with the administration of the iodinated contrast material, in 5 cases was performed in staging, in 20 cases in rest-aging
**Results or Findings:** The most important information was detection of ER+ metastases when 18F-FDG-PET was negative (12x) - including brain and liver metastases, the persistent ER+ of the metastases (7x), staging of the disease (10x), the loss of the ER (4x) and the negative finding for metastases (2), no added information was found in 5 examinations.
**Conclusion:** 18F-FES-PET provided the important clinical information to treatment strategy, PET/MRI improves the imaging of brain and liver.
**Limitations:** Small sample
**Funding for this study:** No
**Ethics committee - additional information:** according to the Helsinky declaration
**Author Disclosures:**
Eva Ferdova: Nothing to disclose
Tana Barakova: Nothing to disclose
Samuel Vokurka: Nothing to disclose
Jirí Ferda: Nothing to disclose

**Conspicuity as new CEDM descriptor: likelihood of Malignancy and Relationship With Breast Tumor Receptor Status**

L. Corradini, *D. Ballerini*, A. Bonanomi, E. D'Ascoli, G. Della Pepa, C. De Berardinis, E. Ancona, C. Depretto, G. P. Scaperrotta; Milan/IT
*(Daniela.Ballerini@istitutotumori.mi.it)*

**Purpose or Learning Objective:** Lesion conspicuity, defined as the "degree of enhancement" relative to the background, was the focus of this retrospective monocentric study, which aimed to explore its correlation with malignancy of lesions, histology, receptor profile and grading in breast cancer patients.
**Methods or Background:** Two breast radiologist and one radiology resident evaluated all CEDM performed in our oncological center from January 2023 to April 2024, assigning degrees of conspicuity to breast lesions, and evaluating a possible correlation with Ki-67 values (≤ 20% or > 20%), HER-2 status, estrogen (ER) and progesterone (PGR) receptor positivity, molecular subtype, and histological grade. Statistical analysis employed the Cramer's V test.
**Results or Findings:** Out of 352 patients included (median age=54, IQR=18), 100 were excluded due to chemotherapy controls and 53 due to B3 lesion. In the 199 remaining patients we observed a moderate to strong association between conspicuity and ER expression (V=0.534) and Ki-67 value (V=0.36). A moderate association was found between conspicuity and PGR expression (V=0.31). No significant correlation was noted between conspicuity and histological grade (V=0.184) or HER2 status (V=0.2).
**Conclusion:** Conspicuity, a recently incorporated descriptor in CEDM BI-RADS lexicon, was validated by our findings, which are in line with the initial evidence in the literature.
**Limitations:** Retrospective monocentric study with limited number of patients. Conspicuity is a subjective descriptor, potentially introducing variability in the data and affecting the findings.
**Funding for this study:** None
**Ethics committee - additional information:** None
**Author Disclosures:**
Gianmarco Della Pepa: Nothing to disclose
Claudia De Berardinis: Nothing to disclose
Elisa D'Ascoli: Nothing to disclose
Alice Bonanomi: Nothing to disclose
Daniela Ballerini: Nothing to disclose
Eleonora Ancona: Nothing to disclose
Gianfranco Paride Scaperrotta: Nothing to disclose
Catherine Depretto: Nothing to disclose
Lisa Corradini: Nothing to disclose

**A novel metabolic MRI method for malignant breast tumors detection**

M. Rivlin[1], *R. Sivan Hoffmann*[2], V. Hadar[2], S. Sukhotnik[2], N. E. Weisenberg[2], O. Shmain-Naydenov[2], M. Zaiss[3], S. Weinmüller[3], G. Navon[1]; [1]Tel Aviv/IL, [2]Kfar Saba/IL, [3]Erlangen/DE
*(rotemsivan3@gmail.com)*

**Purpose or Learning Objective:** Molecular imaging with 18F-fluorodeoxyglucose positron emission tomography (18FDG-PET) is a powerful and well-established tool in breast cancer management, as increased glucose uptake is a known cancer hallmark.However, it carries the risk of repeated radiation exposure. We have recently discovered that glucosamine (GlcN), a non-toxic, biocompatible glucose-based material can be detected using a unique MRI contrast mechanism termed chemical exchange saturation transfer (CEST). CEST has emerged as an attractive molecular imaging approach for providing valuable metabolic information. Our goal is to develop an innovative molecular imaging modality based on CEST-MRI of GlcN to visualize and measure breast tumors while also distinguishing between benign and malignant tumors.
**Methods or Background:** Breast cancer patients and control group were scanned using the CEST-MRI pulse sequence on a 3T scanner (VIDA, Siemens, Germany) equipped with breast coil. The protocol included CEST scans before and two hours after drinking a solution of GlcN (184 mg/kg). The data were evaluated using magnetization transfer asymmetry ratio (MTRasym) and area under curve (AUC) analysis.
**Results or Findings:** GlcN treatment resulted in higher CEST values in tumor regions of interest (ROIs), with maximum net MTRasym signal (at 2 ppm) of 6.3±2.6% and averaged AUC (2-5 ppm) increase ratio of 3.3±2.1% (N=7). Yet, no significant GlcN CEST signal enhancement was detected in healthy volunteers (N=9). GlcN CEST signal values were highly correlated with the BI-RADS category.
**Conclusion:** These findings suggest that the GlcN CEST MRI technique can detect breast cancer while also providing molecular-level diagnostic tools for discriminating between benign and malignant breast tumors. These promissing results pave the way to a future metabolic imaging of additional diseases.
**Limitations:** Due to the size of the study group we were not able to clarify into sub-groups.
**Funding for this study:** Funding by the ISF No. 1689/18
**Ethics committee - additional information:** approval number MMC0201-21
**Author Disclosures:**
Olga Shmain-Naydenov: Nothing to disclose
Noemi Edith Weisenberg: Nothing to disclose
Moritz Zaiss: Nothing to disclose
Rotem Sivan Hoffmann: Nothing to disclose
Stephanie Sukhotnik: Nothing to disclose
Simon Weinmüller: Nothing to disclose
Vivian Hadar: Nothing to disclose
Michal Rivlin: Nothing to disclose
Gil Navon: Nothing to disclose

**Artificial intelligence in digital mammography and serial breast tomosynthesis for neoadjuvant breast cancer treatment response prediction**

*D. Förnvik*[1], S. Zackrisson[1], I. Skarping[2]; [1]Malmö/SE, [2]Lund/SE
*(daniel.fornvik@med.lu.se)*

**Purpose or Learning Objective:** To predict neoadjuvant chemotherapy (NACT) treatment response by applying artificial intelligence (AI) to digital mammography (DM) and serial breast tomosynthesis (DBT) images. Explainable AI (XAI) for enhanced clinical credibility is explored.
**Methods or Background:** NACT for early-stage breast cancer (BC) has recently become an attractive approach to patients who are eligible for chemotherapy. MRI is the imaging modality of choice for evaluating tumor response but not as readily available as DM and lately DBT. Nevertheless, predicting residual cancer, as assessed by radiologist, after NACT using imaging is challenging; thus, AI could be an alternative. Between 2005/2014 - 2019, 453 (DM) and 149 (DBT) patients, respectively, at Skane University Hospital, Sweden, comprised the cohorts. Two deep learning architectures (DM: ResNet24, DBT: backbone 3D ResNet) applied to images from both the cancer and contralateral healthy breasts acquired at three time points: pre-NACT (DM and DBT), mid-NACT (DBT) and post-NACT (DBT) were used to predict pathological complete response (pCR). For DBT, GradCAM was used to produce saliency maps to obtain insights into the model-based decisions.
**Results or Findings:** The DM and the DBT AI models predicted pCR as represented by the area under the ROC curve of 0.71 (95% CI: 0.53–0.90; p = 0.035) and 0.83 (95% CI: 0.63–1.00; p = 0.008), respectively. The spatial correlation of saliency maps for DBT volumes from the same patient but at different time points was high, likely indicating that the model focuses on the same areas during decision-making.
**Conclusion:** The DBT model demonstrates a high discriminative performance for predicting pCR/non-pCR, possibly outperforming radiologists' assessment.
**Limitations:** Availability of larger datasets and inclusion of clinicopathological variables would permit more comprehensive training of the models and more rigorous evaluation of their prediction performance for future patients.
**Funding for this study:** Swedish Breast Cancer Group (BRO), Allmänna Sjukhusets i Malmö Stiftelse för bekämpande av cancer, and the Governmental Funding of Clinical Research within the National Health Services.
**Ethics committee - additional information:** Regional Ethics Committee in Lund, Sweden (committee reference numbers: 2014/13, 2014/521, and 2016/521, and 2021-05637-02).

**Author Disclosures:**
Daniel Förnvik: Nothing to disclose
Ida Skarping: Nothing to disclose
Sophia Zackrisson: Nothing to disclose

**Image Quality and Diagnostic Values of Diffusion-Weighted Breast MRI: A Comparison of Single-Shot EPI with Deep Learning Reconstruction and Multi-Shot EPI with Simultaneous Multislice**

*H. S. Ahn*, S. H. Kim, M. J. Hong, H-S. Lee; Seoul/KR

**Purpose or Learning Objective:** To evaluate the image quality and diagnostic value of single-shot echo-planar imaging (ss-EPI) with deep learning reconstruction (DLR) versus simultaneous multi-slice echo-planar imaging (SMS rs-EPI) in breast MRI.
**Methods or Background:** This study included 77 cases of breast cancer from 74 patients who underwent preoperative breast MRI. All patients underwent breast MRI that included the ss-EPI sequence combined with post-processing using DLR, as well as the SMS rs-EPI sequence. Two radiologists independently assessed qualitative image quality factors and determined their preferences, while the cancer detection rate (CDR) was calculated. Quantitative analysis included measurements of apparent diffusion coefficient (ADC), signal-to-noise ratio (SNR), contrast-to-noise ratio (CNR), and lesion contrast, including phantom measurements.
**Results or Findings:** Regarding qualitative image quality parameters, ss-EPI with DLR demonstrated significantly higher scores in fat suppression and overall image quality as assessed by both radiologists, with comparable scores in artifact presence and lesion conspicuity to the SMS rs-EPI sequence. The CDR showed no significant difference between the two sequences. Both radiologists preferred ss-EPI with DLR (Reader 1: 78.4%, Reader 2: 89.2%). For quantitative parameters, ss-EPI with DLR exhibited significantly higher CNR ($p = 0.002$) and lesion contrast ($p < 0.001$), while ADC and SNR values were comparable. In phantom measurements, mean ADC was lower for ss-EPI with DLR (DLR: 1.08 ± 0.58 vs. SMS: 1.12 ± 0.59, $p = 0.007$), but SNR was not significantly different (DLR: 607.45 ± 346.1 and SMS: 630.03 ± 624.51, $p = 0.911$). The acquisition time was shorter for ss-EPI with DLR (2:06 min) compared to SMS rs-EPI (3:29 min).
**Conclusion:** ss-EPI with DLR provided superior image quality and greater reader preference compared to SMS rs-EPI.
**Limitations:** This is a retrospective study which performed at single center.
**Funding for this study:** None
**Ethics committee - additional information:** None
**Author Disclosures:**
Hyun-Soo Lee: Nothing to disclose
Min Ji Hong: Nothing to disclose
Sung Hun Kim: Nothing to disclose
Hye Shin Ahn: Nothing to disclose

**Dual Imaging Power: CT and Contrast-Enhanced Mammography (CEM) for Advanced Detection of Metastatic Breast Cancer**

*M. Balbino*[1], M. Montatore[2], F. Masino[3], F. A. Carpagnano[4], G. Capuano[2], G. Guglielmi[5]; [1]Triggiano/IT, [2]Barletta/IT, [3]Bari/IT, [4]Foggia/IT, [5]Andria/IT
*(marinabalbino93@gmail.com)*

**Purpose or Learning Objective:** This study, one of the first in Italy, aims to evaluate the efficacy of performing CT and CEM consecutively using the same contrast medium in a single imaging session. The goals include reducing the amount of contrast agent injected into oncological patients and enhancing the detection of metastases in various districts through CT, while also providing a precise diagnosis of breast extension and identifying additional foci within the breasts through CEM.
**Methods or Background:** A cohort of female patients with confirmed primary breast cancer and suspected metastatic disease were enrolled. Each patient underwent a CT scan followed immediately by a CEM using the same contrast medium. The CT was performed to identify visceral metastases, while the CEM targeted the detection of additional breast lesions and regional lymph node involvement. Both imaging modalities utilized iodine-based contrast agents, administered intravenously. The diagnostic outcomes were compared with those from conventional imaging techniques, including standard mammography, ultrasound, and MRI.
**Results or Findings:** The combined CT and CEM approach demonstrated a higher sensitivity and specificity in detecting metastatic sites compared to traditional imaging methods. In particular, CEM revealed additional lesions in the breast and regional lymph nodes that were not identified by CT alone. The concurrent use of the same contrast medium was found to be safe and well-tolerated, with no significant increase in adverse reactions. The integrated imaging protocol provided comprehensive anatomical and functional information, leading to more accurate staging and better-informed treatment decisions.
**Conclusion:** The integration of CT and CEM using the same contrast agent offers a promising advancement in the diagnostic imaging of metastatic breast cancer. This combined approach enhances the detection of metastatic lesions, providing a more comprehensive assessment of disease spread.
**Limitations:** No
**Funding for this study:** No
**Ethics committee - additional information:** No
**Author Disclosures:**
Marina Balbino: Nothing to disclose
Giuseppe Guglielmi: Nothing to disclose
Francesca Anna Carpagnano: Nothing to disclose
Manuela Montatore: Nothing to disclose
Federica Masino: Nothing to disclose
Giulia Capuano: Nothing to disclose

**Novel and robust approach to breast density prediction: utilising the Tree-Structured Parzen Estimator algorithm-driven transfer learning approach**

*M. Bobowicz*, M. Kosno, K. P. Brzozowski, M. Rygusik; Gdańsk/PL
*(maciej.bobowicz@gumed.edu.pl)*

**Purpose or Learning Objective:** The breast density visual assessment in mammography is a subjective process prone to errors but impacting diagnostic decisions. To overcome this problem, we developed a robust and reliable AI model that employs convolutional neural network-based transfer learning, specifically ResNet, DenseNet, and EfficientNet architectures, to predict breast density. Our research benefits from the Tree-structured Parzen Estimator (TPE) algorithm, an advanced tool for hyperparameter optimisation.
**Methods or Background:** A dataset of 2101 digital MLO mammography images performed at the Medical University of Gdansk from 2014 to 2022 was selected for analysis. The images were acquired using various devices from SIEMENS, GE HEALTHCARE, and HOLOGIC to ensure a high degree of image characteristics variability. The dataset was divided into 80% training and 20% validation sets. ResNet, DenseNet, and EfficientNet architectures were trained using the TPE algorithm. The assembly model comprises three five-fold cross-validated convolutional networks.
**Results or Findings:** An ensemble model resulted in good performance metrics: AUC-ROC (0.99), accuracy (0.91), F1-score (0.91), and recall values (0.90) for the test dataset. The TPE algorithm facilitates the development of high-performance models on a relatively small dataset, eliminating the need for image segmentation to extract the skin and pectoral muscle opacities, which is challenging to implement and often burdened with significant errors.
**Conclusion:** Our methodology enables the straightforward training of a robust model that can provide highly precise breast density predictions, reducing and automating the burden of required density reporting. Furthermore, our findings demonstrate the efficacy of advanced hyperparameter numerical optimisation methods in enhancing the efficiency of transfer deep learning models in the context of breast density prediction.
**Limitations:** The study's limitations are its relatively small dataset, single-centre design, and lack of external validation.
**Funding for this study:** Funding was provided by the European Union's Horizon 2020 research and innovation programme under grant agreement No 952103 (EuCanImage project) and was co-funded by the Digital Europe programme under grant agreement No 101100633 (EUCAIM project).
**Ethics committee - additional information:** This retrospective study uses fully anonymised data from the EuCanImage project under the global ethics committee agreement for MUG.
**Author Disclosures:**
Maciej Bobowicz: Nothing to disclose
Michał Kosno: Nothing to disclose
Marlena Rygusik: Nothing to disclose
Krystian Paweł Brzozowski: Nothing to disclose

**13:00-14:30** **Research Stage 2**

### Research Presentation Session: Genitourinary

### RPS 407

### What's new in prostate imaging: advances and emerging techniques

**Moderator**
P. A. Bonaffini; Bergamo/IT
*(pa.bonaffini@gmail.com)*

**Advancing prostate cancer imaging: a comparative analysis of MET-RADS-P and PCWG3 in the assessment of mCRPC**
*L. Russo*[1], S. Bottazzi[1], O. Longoria[2], G. Avesani[1], S. J. Withey[2], L. D'Erme[1], E. Sala[1], D-M. Koh[2], N. Tunariu[2]; [1]Rome/IT, [2]London/UK

**Purpose or Learning Objective:** Treatment response assessment in metastatic castration-resistant prostate cancer (mCRPC) is critical because the Prostate Cancer Working Group 3 (PCWG3) criteria have notable limitations. The METastasis Reporting and Data System for Prostate Cancer (MET-RADS-P) provides standardised guidelines using whole-body MRI (WBMRI). Our main aim was to compare MET-RADS-P and PCWG3 criteria for disease progression categorization in mCRPC, as well as the prognostic value of MET-RADS-P for progression-free survival (PFS) and overall survival (OS).
**Methods or Background:** A cohort of 201 mCRPC patients treated at The Royal Marsden Hospital between January 2013 and February 2024 was retrospectively included. All patients underwent WBMRI, CT and BS at each time point. CT and BS were interpreted according to PCWG3 and WBMRI according to MET-RADS-P. Concordance between MET-RADS-P and PCWG3 in disease progression categorization was assessed overall, in bone and soft-tissue only. PFS and OS were evaluated using Kaplan-Meier survival curves with log-rank test comparisons.
**Results or Findings:** Overall, 64.5% of time points (302/468) were concordant between MET-RADS-P and PCWG3 criteria, with MET-RADS-P detecting progression earlier in 31.8% (149/468). Discrepancies were more pronounced in bone metastases, where MET-RADS-P identified progression in 55.1% of cases classified as non-progressive disease by PCWG3. Progressing patients by MET-RADS-P had significantly worse PFS: median 2.7 months versus 4.2 months by PCWG3 ($p<0.001$). The median OS was 12.5 months for progressing patients by MET-RADS-P at 12-week assessment compared to 19.8 months for those stable or responding ($p<0.001$).
**Conclusion:** MET-RADS-P allowed for earlier progression detection compared with PCWG3, particularly in bone metastases, potentially permitting earlier therapeutic interventions. MET-RADS-P also demonstrated strong predictive value for PFS and OS, suggesting its potential role as an imaging biomarker in future clinical trials.
**Limitations:** Retrospective design and lack of cost and availability comparative analysis.
**Funding for this study:** This study represents independent research funded by the National Institute for Health and Care Research (NIHR) Biomedical Research Centre at The Royal Marsden NHS Foundation Trust and The Institute of Cancer Research, London, and by the Royal Marsden Cancer Charity, and Cancer Research UK (CRUK) National Cancer Imaging Trials Accelerator (NCITA) and Prostate Cancer UK. The views expressed are those of the author(s) and not necessarily those of the NIHR or the Department of Health and Social Care. This work uses data provided by patients and collected by the NHS as part of their care and support.
**Ethics committee - additional information:** The study was approved by the Institutional Ethics Committee (reference no. 21/LO/0605).
**Author Disclosures:**
Samuel Joseph Withey: Nothing to disclose
Giacomo Avesani: Nothing to disclose
Dow-Mu Koh: Nothing to disclose
Nina Tunariu: Nothing to disclose
Ossian Longoria: Nothing to disclose
Silvia Bottazzi: Nothing to disclose
Evis Sala: Nothing to disclose
Luca D'Erme: Nothing to disclose
Luca Russo: Nothing to disclose

**Analysis of biopsy strategy in young men with suspicious PSA in a prostate cancer screening setting – data from the PROBASE trial**
*M. Boschheidgen*[1], R. Al-Monajjed[1], J. P. Radtke[1], H-P. Schlemmer[2], G. Antoch[1], L. Schimmöller[1], P. Albers[1]; [1]Düsseldorf/DE, [2]Heidelberg/DE
*(matthias.boschheidgen@med.uni-duesseldorf.de)*

**Purpose or Learning Objective:** To analyze the performance of targeted (TB) and systematic (SB) MRI/US fusion-guided prostate biopsy within the prospective PROBASE prostate cancer (PC) screening trial.
**Methods or Background:** Men aged forty-five from the general population were invited to screening. Those with confirmed prostate-specific antigen (PSA) levels of 3 ng/ml or higher were offered an MRI and were referred to MRI/US-guided biopsy. Biopsies were performed in every participant unrespective of the MRI result. Targeted and systematic biopsies with software-based fusion techniques were offered. The primary endpoint of this analysis was the PC detection in either TB or SB.
**Results or Findings:** A total of 554 men (median age 50 (range 44-54), median PSA level 4.1 ng/ml) were analyzed who underwent an MRI followed by MRI/US-guided biopsy. Of 217 PC diagnosed, 198 (91%) and 140 (65%) were detected by SB and TB, respectively. 64 of 217 PC (29%) were low grade (ISUP 1). 40 significant tumors were found exclusively by SB (26%), while 9 significant tumors were only diagnosed with TB (6%). SB detected significantly more low-grade cancer compared to TB ($p<0.001$). Cancer detection rate was 20% for PIRADS 1-2, 26% for PIRADS 3, 59% for PIRADS 4, and 92% for PIRADS 5.
**Conclusion:** In young men and in the setting presented here, systematic biopsy in addition to targeted MRI/US guided fusion biopsy still appears to be justified to adequately detect PC. Most PC (71%) were clinically significant. Performing only TB in young men without SB faces the risk of missing a significant number of csPC even if it simultaneously diagnoses fewer low-grade carcinomas.
**Limitations:** MRI did not influence clinical decision-making; experience in MRI reading and the image quality differed widely at the time of initiation
**Funding for this study:** Deutsche Krebshilfe
**Ethics committee - additional information:** The study was approved by local ethics committee.
**Author Disclosures:**
Jan Philipp Radtke: Nothing to disclose
Peter Albers: Nothing to disclose
Rouvier Al-Monajjed: Nothing to disclose
Matthias Boschheidgen: Nothing to disclose
Lars Schimmöller: Nothing to disclose
Heinz-Peter Schlemmer: Nothing to disclose
Gerald Antoch: Nothing to disclose

**Is there an MRI phenotype for the cribiform pattern of Prostate Cancer?**
M. D. M. Palma, D. Freire Maia Vieira, T. A. Leite De Lima, J. Nather, F. Chahud, R. B. Reis, *V. F. Muglia*; Ribeirao Preto/BR
*(fmuglia@fmrp.usp.br)*

**Purpose or Learning Objective:** Cribriform pattern (CP) is a distinct histological feature present in various neoplasms, characterized by cohesive tumour cells surrounding circular spaces, creating a "Swiss cheese" appearance. In prostate cancer (PCa), CP is one of the four architectural subtypes of the Gleason 4 pattern and has been linked to worse outcomes compared to other morphologies. This study aimed to determine if CP presents distinct features on multiparametric magnetic resonance imaging (MRI).
**Methods or Background:** In this retrospective, single-centre study, we identified PCa cases with CP from 2016 to 2023, with MRI conducted within four months of histological diagnosis. Patients without CP but with equivalent Gleason grades and risk stratification were included as controls in a ratio of up to 1.5:1. Two radiologists, with over 5 and the other with 7 years of prostate imaging experience, evaluated lesion size, form, location, prostate volume, mean apparent diffusion coefficient (ADC) values, and post-contrast kinetic curves. Clinical staging, prostate-specific antigen (PSA), and PSA density (dPSA) were also reviewed.
**Results or Findings:** The study included 42 patients with CP and 72 without CP. No significant differences were found between the groups regarding PSA ($p=0.43$), dPSA ($p=0.37$), lesion size ($p=0.33$), location ($p=0.65$), mean ADC ($p=0.21$), or kinetic curve type ($p=0.75$). Significant differences were observed for age ($p<0.0001$), prostate volume ($p=0.001$), and PI-RADS category ($p=0.05$). In univariate logistic regression, age and PI-RADS score were independent predictors of CP presence, but only age remained significant in multivariate analysis ($p=0.0001$).
**Conclusion:** Cribrifrom pattern in PCa is more common in older men with larger prostates and higher PI-RADS scores. However, no specific morphological or functional MRI parameters were associated with the presence of this pattern.
**Limitations:** Single-centre, retrospective study.
**Funding for this study:** None

**Ethics committee - additional information:** Our Institutional Review Board approved the research with a waiver for informed consent due to the retrospective nature.
**Author Disclosures:**
Fernando Chahud: Nothing to disclose
David Freire Maia Vieira: Nothing to disclose
Julio Nather: Nothing to disclose
Matheus De Moraes Palma: Nothing to disclose
Valdair Francisco Muglia: Nothing to disclose
Rodolfo B. Reis: Nothing to disclose
Thalyne Aparecida Leite De Lima: Nothing to disclose

**The effect of prostate volume - does PSA density always work?**

*S. Durmaz*[1], S-C. J. Wu[2], K-L. Lee[2], A. Shakur[2], I. Caglič[2], T. Barrett[2]; [1]Istanbul/TR, [2]Cambridge/UK
*(drselahattindurmaz@gmail.com)*

**Purpose or Learning Objective:** To evaluate the impact of PSA density (PSAd) on the probability of detecting clinically significant prostate cancer (csPCa) across different prostate volume ranges and PI-RADS scores.
**Methods or Background:** 2097 patients undergoing multiparametric MRI (mpMRI) for suspected PCa were included. 738/2097 (35.2%) had PCa, and 566/2097 (27%) had csPCa (Gleason ≥3+4), patients were classified as negative after biopsy (n=299) or were diagnosed with clinically insignificant PCa (n=172) or having a negative mpMRI and completing at least one-year follow-up without developing PCa (n=1060). Single-variable logistic regression analyses were conducted to assess the impact of PSAd on the probability of csPCa within the different prostate volume ranges (<40 mL, 40-60 mL, 60-80 mL, >80 mL) and PI-RADS groups.
**Results or Findings:** The median age, PSA, PSAd, and prostate volume was 66 years (IQR: 61-72), 5.6 ng/mL (IQR:4.05-8.05), 0.10 ng/mL/mL (IQR:0.07-O.15), and 56 mL (IQR:39-80), respectively. Logistic regression at a PSAd of 0.15 ng/mL/mL showed the probability of csPCa decreased with increasing prostate volume: <40 mL (44%), 40-60 mL (38%), 60-80 mL (29%), and >80 mL (18%). At the same PSAd level, the probability of csPCa increased with increasing PI-RADS score: PIRADS 1-2 (3%), PI-RADS 3 (26%), PI-RADS 4 (63%), PI-RADS 5 (70%). Regardless of the PSAd level, the risk of csPCa in patients with PI-RADS 4-5 lesions was always >20%.
**Conclusion:** When using PSAd to assist in the decision to perform MRI, or to biopsy patients with PI-RADS 1-3 scores, caution should be exercised in those with larger volume prostates, as the lower PSAd can provide false reassurance.
**Limitations:** Retrospective design. All patients underwent mpMRI and prostate biopsy at a single tertiary referral center with extensive experience in prostate MRI and biopsy.
**Funding for this study:** No funding was received for this study.
**Ethics committee - additional information:** This is a retrospective study.
**Author Disclosures:**
Iztok Caglič: Nothing to disclose
Amreen Shakur: Nothing to disclose
Shun-Chin Jim Wu: Nothing to disclose
Selahattin Durmaz: Nothing to disclose
Kang-Lung Lee: Nothing to disclose
Tristan Barrett: Nothing to disclose

**A transformer-based deep learning model for early prediction of biochemical recurrence after radical prostatectomy using pretreatment mpMRI**

*F. Li*[1], L. Zhuo[1], L. Yue[1], L. Juan[1], L. Wang[1], R. Liu[1], F. Wang[2], Y. Xiang[3]; [1]Mianyang City/CN, [2]Luzhou/CN, [3]Leshan/CN
*(317064491@qq.com)*

**Purpose or Learning Objective:** The purpose of this study is to develop and verify a deep learning model using preoperative multi-parameter MRI images to predict BCR risk after radical prostatectomy.
**Methods or Background:** Patients after radical surgery at 4 centers between August 2013 and September 2021 were retrospectively included with the endpoint outcome of 3-year BCR (two consecutive specific antigen [PSA] levels > 0.2 ng/mL [0.2μg/L]). A transformer-based DL model was used to predict BCR after radical surgery using 3D tumor images, a clinical model was constructed by multivariate logistic regression, Kaplan-Meier plots were used for estimating recurrence-free survival, and finally, pre- and post-surgical Capra models, a clinical model, a multi-instance model, and a transformer model, Multimodal Combine model were compared to assess the performance of predicting BCR.
**Results or Findings:** A total of 582 patients (median age 70 years, (IQR 44-89 years) with a median follow-up of 43 months (IQR, 29-71 months) were randomized into a training group (n=249 ), an internal test set (n=107), an external test set 1 (n=189), and an external test set 2 (n=37).The AUC of the Transformer model in the 0.92 in the internal test set, 0.84 in the external test set 1, and 0.82 in the external test set 2, and the multimodal Combine model further improves the performance, respectively, with 0.94 (95% CI. 0.885 - 0.992), 0.94 (95% CI, 0.900 - 0.969), and 0.83 (95% CI, 0.693 - 0.965), and early recurrence-free survival and overall survival could be better risk-stratified and predicted using the Combine model.
**Conclusion:** A transformer-based DL model for predicting BCR after radical surgery was developed and internally and externally validated, and the joint model is better and expected to guide individualized treatment.
**Limitations:** Not applicable.
**Funding for this study:** No funding was provided for this study
**Ethics committee - additional information:** Ethics （2024）014-1
**Author Disclosures:**
Lu Wang: Nothing to disclose
Ruishan Liu: Nothing to disclose
Fan Li: Nothing to disclose
Fei Wang: Nothing to disclose
Liao Juan: Nothing to disclose
Ye Xiang: Nothing to disclose
Lv Yue: Nothing to disclose
Lihua Zhuo: Nothing to disclose

**Short MRI sequence suitable for re-identification of prostate lesion during in-bore biopsy?**

*C. Peter*, A. Schaudinn, C. Ehrengut, T. Franz, L-C. Horn, N. Linder, J-U. Stolzenburg, H. Busse, T. Denecke; Leipzig/DE
*(christian.peter1@web.de)*

**Purpose or Learning Objective:** To evaluate the image quality of a rapid intraprocedural balanced steady-state free precession (b-SSFP) sequence for re-identification of prostate lesions during transrectal in-bore biopsies in comparison with that of a T2-weighted
**Methods or Background:** In this retrospective study, 127 patients with 140 PI-RADS ≥ 3 (version 2.1) lesions based on multiparametric 3T MRI (mpMRI) underwent transrectal in-bore biopsies. b-SSFP images were acquired at 1.5T for interventional guidance. Two radiologists (R1: 11 years and R2: 2 years of mpMRI experience) independently rated the image quality of both b-SSFP (acquisition time: 11-15 seconds) and diagnostic T2-weighted TSE (3T, acquisition time: 4-5 minutes) sequences using a 4-point scale (3: good, 2: acceptable, 1: poor, 0: impossible for lesion identification). Recognition rates (RR) were calculated as the percentage of cases with sufficient image quality (scores of 3 or 2). Subgroup analyses were performed by zonal location (PZ/TZ), lesion size (</≥ 0.5 mL), and PI RADS score (3/4-5). Differences between readers and sequences were analysed using McNemar's test ($p < 0.05$).
**Results or Findings:** The RR for the T2-weighted reference sequence was 98% for both radiologists, with subgroups ranging from 94% to 99%. For b-SSFP, the RR was 87% for R1 and 81% for R2, with subgroups ranging from 75% (PI-RADS 3) to 92-93% (PI-RADS 4/5, large lesions). No significant differences were found between readers. RR differences between sequences were statistically significant, except for TZ and large lesions rated by R1.
**Conclusion:** b-SSFP showed only moderately lower RR than the very high RR of T2-weighted reference images, especially for the experienced reader R1. Given its much shorter acquisition time, b-SSFP of discernible lesions therefore has the potential to reduce biopsy times, particularly for large or (highly) suspicious lesions (PI-RADS 4-5).
**Limitations:** Retrospective; single-center.
**Funding for this study:** None
**Ethics committee - additional information:** Ethics committee was consulted, written informed consent was obtained from participating patients
**Author Disclosures:**
Nicolas Linder: Nothing to disclose
Timm Denecke: Nothing to disclose
Harald Busse: Nothing to disclose
Lars-Christian Horn: Nothing to disclose
Constantin Ehrengut: Nothing to disclose
Alexander Schaudinn: Nothing to disclose
Jens-Uwe Stolzenburg: Nothing to disclose
Toni Franz: Nothing to disclose
Christian Peter: Nothing to disclose

**Diagnostic assessment of early DWI changes after Single-Dose Ablative Radiation Therapy for localized prostate cancer**

*P. N. Franco*, C. R. G. L. O. M. Talei Franzesi, C. Maino, R. Corso, D. Ippolito; Monza/IT
*(francopaoloniccolo@gmail.com)*

**Purpose or Learning Objective:** To investigate the diagnostic value of diffusion-weighted (DWI) MRI early changes, 1 hour after treatment, in patients with organ-confined unfavourable prostate cancer (PCa) treated with Single-Dose Ablative Radiation Therapy (SDART), in comparison with biochemical markers.
**Methods or Background:** Twenty-four patients with intermediate unfavourable or high-risk localized PCa treated with SDART (21 Gy on the entire prostate with boost up to 24 Gy on the focal lesion) associated with hormone therapy were prospectively enrolled. Each patient was examined with a 3T scanner four times: (1) 1-2 weeks before RT (t0) for treatment planning; (2) 1 hour after treatment (t1); (3) 3 months after treatment (t2); (4) 2 years after treatment (t3). Regions of interest (ROIs) were plotted on apparent diffusion coefficient (ADC) maps and T2-HR sequences on lesions, benign peripheral zone, and the entire prostate gland. Patients' laboratory data (PSA and testosterone) was collected.
**Results or Findings:** ADC values significantly increased in neoplastic lesions at t1, t2 and t3 (+22%, +43% and +53%, respectively). Conversely, no significant changes were observed in the benign peripheral zone and the entire prostate gland. On T2 sequences, signal intensity progressively decreased in the benign peripheral zone (t1: -1%; t2: -33%; t3: -42%) and in the entire prostate gland (t1: -6%; t2: -24%; t3: -31%), while no significant changes were observed in lesions. All patients except one had a complete biochemical response.
**Conclusion:** The study findings showed high diagnostic value of DWI and a good correlation between early (t1) changes in ADC values after SDART and later (t2 and t3) tumour response (both biochemical and imaging) in patients with unfavourable PCa. Early DWI changes can represent a useful parameter to evaluate treatment response and predict patients' outcomes.
**Limitations:** Small sample size; associated hormone therapy.
**Funding for this study:** None
**Ethics committee - additional information:** The local ethics committee formally approved this study.
**Author Disclosures:**
Cesare Maino: Nothing to disclose
Cammillo Roberto Giovanni Leopoldo Oreste Massimiliano Talei Franzesi: Nothing to disclose
Rocco Corso: Nothing to disclose
Paolo Niccolò Franco: Nothing to disclose
Davide Ippolito: Nothing to disclose

**Maximal radial distance as a new parameter for predicting extraprostatic extension of prostate cancer on multiparametric magnetic resonance imaging: a histo-radiological study**

*F. Porões*, A. Nobile, L. Widmer, J. A. Vidal, J. Di Vincenzo, H. Najberg, J. M. M. Froehlich, C. Reischauer, H. Thoeny; Fribourg/CH
*(fabioporoes@gmail.com)*

**Purpose or Learning Objective:** We introduce a new parameter for predicting extraprostatic extension (EPE) on multiparametric magnetic resonance imaging (mpMRI): the maximal radial distance (maxRADD). It corresponds to the largest diameter of a prostate cancer focus (PCF) perpendicular to a contact with the prostate pseudocapsule. We compare accuracy and reproducibility of maxRADD with the previously proposed maximal capsular contact length (maxCCL) for predicting EPE.
**Methods or Background:** We retrospectively and consecutively included 81 patients undergoing prostate mpMRI between October 2018 and December 2020, followed by radical prostatectomy. One uropathologist with 8 years of experience collected for each PCF: location, maxCCL, maxRADD, and presence/absence of EPE. Four radiologists with 0, 2, 3, and 6 years of experience in prostate mpMRI determined maxRADD and maxCCL on mpMRI for each PCF twice in separate readings. Accuracy in predicting EPE was assessed using the area under the curve (AUC), with the pathologic findings as the gold standard. Inter-/intra-reader agreement were assessed using intraclass correlation coefficients (ICCs) and Cronbach's alpha.
**Results or Findings:** On histolpathology, there was no significant difference in the accuracy of predicting EPE between maxRADD and maxCCL (AUCmaxRADD = 0.92, AUCmaxCCL = 0.91, p = 0.28). Pearson correlation showed a strong correlation of both parameters determined on mpMRI with their histopathological counterparts (>0.7), with the exception of maxCCL assessed by the reader w/o experience in prostate mpMRI (0.54). On mpMRI, inter-reader agreement was significantly higher for maxRADD (ICCmaxRADD = 0.96, ICCmaxCCL = 0.94, p = 0.046) and intra-reader agreement was higher but did not reach significance (average alphamaxRADD = 0.95, average alphamaxCCL = 0.92, p = 0.31).
**Conclusion:** MaxRADD permits assessing EPE with good accuracy and shows higher reproducibility compared with maxCCL.
**Limitations:** No significant limitation.
**Funding for this study:** This study has received funding by the Swiss National Science Foundation (Grant/Award Number: 32003B_176229/1) and the HFR Research GRANT (2352).
**Ethics committee - additional information:** The study was approved by our institutional ethics committee (CER-VD). The ethics committee notification can be found under the project-ID 2020-01859.
**Author Disclosures:**
Harriet Thoeny: Advisory Board: Guerbet
Jana Di Vincenzo: Nothing to disclose
Hugo Najberg: Nothing to disclose
Johannes Malte Maria Froehlich: Consultant: Guerbet
Antoine Nobile: Nothing to disclose
Fabio Porões: Nothing to disclose
Lucien Widmer: Nothing to disclose
Julian Alexis Vidal: Nothing to disclose
Carolin Reischauer: Nothing to disclose

**Impact of Centrally Reviewed PI-QUAL v2 Scores on the Diagnostic Performance of Prostate MRI**

*G. Brembilla*, D. Cannoletta, M. Cosenza, F. Pellegrino, M. E. Porzi, L. Quarta, A. Stabile, A. Briganti, F. De Cobelli; Milan/IT

**Purpose or Learning Objective:** To assess the impact of image quality, defined by PI-QUAL v2 scores, on the diagnostic yield of prostate MRI in centrally reviewed scans.
**Methods or Background:** We retrospectively identified consecutive patients who underwent MRI-targeted and systematic biopsies at our Institution (January 2023 - June 2024), with MRI performed externally. All the external MRI scans were centrally reviewed by an experienced uro-radiologist, who assigned PI-QUAL v2 and PI-RADS v2.1 scores. We assessed the proportion of PI-RADS 3 lesions and the detection rate of clinically significant prostate cancer (csPCa), stratified by PI-QUAL v2 scores, before and after central revision. Histopathological results from the biopsies were used as the reference standard.
**Results or Findings:** A total of 151 consecutive patients were included in the analysis. 37/151 (24%) of the MRI scans were scored PI-QUAL 1, 72/151 (48%) PI-QUAL 2, and 42/151 (38%) PI-QUAL 3. Based on original reports, the overall proportion of PI-RADS 3 scans was 34/151 (23%). In PI-QUAL 1-2 vs 3 scans, the proportion of PI-RADS 3 in was 27% vs 12%, respectively; the csPCa detection rate was 48% vs 62%, respectively. The reclassification rate of PI-RADS scores at central review was 64/151 (42%), and was higher for PI-QUAL 1-2 scans (47%) than for PI-QUAL 3 scans (31%). After central revision, the overall proportion of PI-RADS 3 in PI-QUAL 1-2 vs 3 was 19% vs 5%, respectively; the detection rate of csPCa was 58% vs 78%, respectively.
**Conclusion:** Lower prostate MRI image quality, as defined by the PI-QUAL v2 scoring system, is associated with a higher proportion of equivocal scans (PI-RADS 3) and a reduced csPCa detection rate in centrally revised MRI scans.
**Limitations:** Small sample size, only one radiologist for review
**Funding for this study:** None
**Ethics committee - additional information:** IRB approved
**Author Disclosures:**
Maria Elena Porzi: Nothing to disclose
Donato Cannoletta: Nothing to disclose
Michele Cosenza: Nothing to disclose
Francesco Pellegrino: Nothing to disclose
Alberto Briganti: Nothing to disclose
Armando Stabile: Nothing to disclose
Leonardo Quarta: Nothing to disclose
Francesco De Cobelli: Nothing to disclose
Giorgio Brembilla: Nothing to disclose

**Prostate Volume Assessment on MRI: Comparison of full manual segmentation to PIRADS-based approximation in 2 planes and its influence on PSA-density**

*J. Uhlig*, L. Biggemann, C. Louizi, A. Uhlig; Göttingen/DE
*(johannes.uhlig@med.uni-goettingen.de)*

**Purpose or Learning Objective:** To evaluate differences in prostate volume quantification on MRI comparing full manual segmentation and PIRADS-based approximation in 2 planes.
**Methods or Background:** Patients imaged with 3T mpMRI (Siemens VIDA) for suspected prostate cancer between 2021-2023 were included. PSA measurements were obtained at the time of mpMRI or extracted from patients records up to 3 months prior. Manual segmentation of the prostate was performed on all axial T2w slices serving as reference standard. Prostate volume was approximated using 3 measurements on T2w sagittal and axial planes according to the PIRADSv2.1 recommendations. Prostate volumes from manual segmentation and approximation were compared and the influence on PSA-density quantified using different cut-off values.

**Results or Findings:** n=331 patients were included (mean age 67 ± 7 years) with a mean PSA value of 8.1 ± 5.7 ng/ml. Mean prostate volume using manual segmentation was 63.3cc (± 33cc), with n=142 patients having a volume of <=50cc, 51-100cc: n=148, 101-150cc: n=32, and >151cc: n=8. The mean absolute difference of prostate volume using approximation vs. segmentation was 9.1cc (±9.3cc, p=0.01). In general, smaller prostate volumes were overestimated, and larger volumes underestimated by approximation. Using a PSA-density cut-off <0.1 ng/ml/cc, the approximation method yielded an accuracy = 88%. Using a PSA-density cut-off <0.15 ng/ml/cc, the approximation method yielded an accuracy = 90%.
**Conclusion:** Using PIRADSv2.1-based approximation of prostate volume on mpMRI yields a statistically significant difference when compared to full manual segmentation. These differences have a relevant effect on PSA-density calculation with potential impact on downstream patient management.
**Limitations:** Patients were recruited in only one tertiary center and imaged on one MRI scanner, which could limit the generalizability of presented results.
**Funding for this study:** This study received no funding.
**Ethics committee - additional information:** Ethics committee of the University Medical Center Goettingen
**Author Disclosures:**
Annemarie Uhlig: Nothing to disclose
Johannes Uhlig: Nothing to disclose
Lorenz Biggemann: Nothing to disclose
Chiheb Louizi: Nothing to disclose

**MRI without contrast media injection for prostate cancer screening: results from Prostate Cancer Secondary Screening in Sapienza (PROSA)**
*E. Messina*, A. Borrelli, L. Laschena, S. Lucciola, M. Pecoraro, V. Panebianco; Rome/IT
*(emanuele.messina@uniroma1.it)*

**Purpose or Learning Objective:** PROSA is a randomized MRI-based screening protocol, investigating the role of MRI without contrast media injection (bi-parametric MRI, bpMRI) as secondary prevention test for prostate cancer (PCa) early diagnosis, comparing MRI with PSA-test. PROSA aims to investigate the efficiency of this screening protocol, both in terms of diagnostic accuracy, and cost-effectiveness.
**Methods or Background:** 590 men aged 49 to 69 years were enrolled and blindly randomized into two different arms: (A) Men underwent bpMRI regardless of their PSA values; (B) Men with increased PSA were directed to bpMRI, while those with normal PSA were not. Men screened positive on MRI were directed to MR-directed targeted biopsy. To evaluate the efficiency of the protocol we calculated the experimental event rate (EER), control event rate (CER), absolute risk reduction (ARR), number needed to treat (NNT). Health Technology Assessment analysis was implemented to evaluate the cost-effectiveness. The cost/effectiveness ratio is calculated as follows: Delta Costs/ Delta effectiveness = (CA–CB)/(EA-EB).
**Results or Findings:** 289 men were randomized on Arm A and among them 15 clinically significant PCa (csPCa) were detected; 291 men were randomized on Arm B, with 6 csPCa detected (p=0.04). On arm A, 8 men diagnosed with csPCa (53.3%) presented normal PSA levels. Considering the efficiency of the screening protocol, EER was 5.23%, CER 2.06%, ARR 3.17%, and NNT 31.6. Therefore 32 interventions (in this study MRIs) are needed to find one event (in this study one csPCa). The final cost/effectiveness ratio resulted to be € 3.562,61 for the diagnosis of one csPCa.
**Conclusion:** Prostate MRI without contrast media injection showed promising results compared to the use of PSA analysis alone as a screening tool, both in terms of efficiency and cost-effectiveness.
**Limitations:** Single center
**Funding for this study:** No
**Ethics committee - additional information:** CE Approved
**Author Disclosures:**
Sara Lucciola: Nothing to disclose
Valeria Panebianco: Nothing to disclose
Antonella Borrelli: Nothing to disclose
Emanuele Messina: Nothing to disclose
Martina Pecoraro: Nothing to disclose
Ludovica Laschena: Nothing to disclose

**13:00-14:30 Research Stage 3**

### Research Presentation Session: Neuro

### RPS 411

### Aging brain and neurodegeneration imaging

**Moderator**
F. Barkhof; Amsterdam/NL
*(f.barkhof@vumc.nl)*
**Author Disclosures:**
Frederik Barkhof: Advisory Board: Combinostics, Scottish Brain Sciences, Alzheimer Europe, Merck; Author: Clinical Neuroradiology - The ESNR textbook; Consultant: Roche, Celltrion, Rewind Therapeutics, Bracco; Founder: Queen Square Analytics; Grant Recipient: Roche, UK MS Society, Biogen, Merck, ADDI; Other: DSMB member Prothena, EISAI

**Connecting the Dots: Linking White Matter Hyperintensity Patterns to Longitudinal Cognitive Changes in Aging**
*M. M. Courtney*, R. A. Kenny, J. F. Meaney, C. De Looze; Dublin/IE
*(michaelc0001@hotmail.com)*

**Purpose or Learning Objective:** White matter hyperintensities (WMHs) are known to correlate with cognitive decline, stroke, and dementia. Previous research has explored the independent effects of white matter macrostructure, microstructure, and spatial distribution on cognitive function, yet a comprehensive analysis combining elements is limited. We provide a comprehensive WMH analysis to assess their association with cognitive decline over a six-year period.
**Methods or Background:** Data was obtained from The Irish Longitudinal Study on Ageing (TILDA), including MRI scans and cognitive performance scores from 497 community-dwelling older adults. WMHs were segmented using Lesion Prediction Algorithm, analysed using Explore DTI for diffusion metrics. Statistical Analysis performed in R-Studio. Linear mixed effect models used to assess relationship between lesion phenotypes and cognitive decline, adjusting for demographic and health-related variables.
**Results or Findings:** 11,933 WMHs analysed. Average 24 lesions per subject. Average lesion volume 263mm3. Average lesion FA 0.29, MD 1.10. K-means clustering identified 3 primary WMH phenotypes. Deep WMHs associated with older age and 2 or more cardiovascular risk factors (p<0.001 respectively). Higher volume lesions were associated with cardiovascular risk factors (p<0.001), smoking (p<0.01) and older age (p<0.001). High-volume, low FA lesions in both deep (p=0.5) and periventricular (p=0.04) white matter exhibited accelerated cognitive decline over six years. Increased number of periventricular lesions was associated with cognitive decline (p<0.01).
**Conclusion:** WMHs manifest diverse phenotypes associated with cognitive decline. High-volume, low FA lesions in both periventricular and deep white matter are predictive of cognitive deterioration. Identifying WMH phenotypes may inform early intervention strategies and improve patient outcomes by targeting individuals at higher risk of cognitive decline.
**Limitations:** Cross-sectional MRI data implies findings are associative and correlate with longitudinal findings, but correlation does not equal causation. Further investigation with serial MRI would provide more reliable data.
**Funding for this study:** The Irish Longitudinal Study on Ageing is funded by the Irish Department of Health, the Atlantic Philanthropies and Irish Life.
**Ethics committee - additional information:** This study was approved by the Trinity College Faculty of Health Sciences Research Ethics Committee, Dublin, Ireland. Protocols conformed with the Declaration of Helsinki. Signed informed consent was obtained from all respondents prior to participation. Additional ethics approval was received for the magnetic resonance imaging (MRI) sub-study from the St James's Hospital/Adelaide and Meath Hospital, Inc. National Children's Hospital, Tallaght (SJH/AMNCH) Research Ethic Committee, Dublin, Ireland. Those attending for MRI also completed an additional MRI-specific consent form. (De Looze et al)
**Author Disclosures:**
Michael Michael Courtney: Nothing to disclose
Céline De Looze: Nothing to disclose
Rose Anne Kenny: Nothing to disclose
James F Meaney: Nothing to disclose

**Normal Aging-Related Brain Morphological Connectivity Network Linked to Multiple Neurological Diseases**
*L. Yuna*; Beijing/CN
*(yunali1094@163.com)*

**Purpose or Learning Objective:** Disentangling the complex interaction between aging and various aging-related neurological diseases at the brain network level.
**Methods or Background:** We presented a unified analysis framework to identify aging-related morphological connectivity networks (MCNs) and determined their clinical relevance in various neurological diseases (including mild cognitive impairment, Alzheimer's disease, Parkinson’s disease, small vessel disease multiple sclerosis and multiple sclerosis). First, individual MCNs in the HC group were constructed and further decomposed into distinct subnetworks using linked independent component analysis. Aging-related subnetworks were defined as those significantly associated with age. The aging-related subnetworks were spatially correlated with disease-related MCN disruptions. Further, the regression coefficients of the aging-related subnetworks were calculated for each patient’s MCN using linear regression. The regression coefficients were then correlated with various clinical variables within each disease group to assess the clinical significance of the aging-related subnetworks. Finally, a series of annotated biological maps were utilized to advance the biological interpretation of the identified aging-related subnetworks.
**Results or Findings:** We first identified three aging-related subnetworks, including the perceptual-limbic subnetwork, attention-somatomotor subnetwork, and somatomotor-predominant subnetwork, that exhibited distinct aging trajectories. Normal aging interacted with various neurological diseases, exhibiting both transdiagnostic and diagnosis-specific patterns at the brain network level. The aging-related subnetworks were closely related to cognitive and physical performance in patients. Biological correlation analysis revealed that glucose metabolism and several neurotransmitters, such as cannabinoids and dopamine, played critical roles in aging-related subnetworks.
**Conclusion:** This study elucidated the network mechanisms underlying the complex interactions between aging and neurological diseases, offering insights that could improve clinical management and therapy development by evaluating aging effects.
**Limitations:** This study is limited by an uneven sample distribution, variability in disease durations, and the absence of longitudinal research
**Funding for this study:** We demonstrated how normal aging interacted with various neurological diseases at the brain network level, both transdiagnostically and diagnosis-specifically. The identified aging-related subnetworks might serve as imaging markers to distinguish normal aging effects from disease-specific mechanisms, thereby improving disease monitoring and management.
**Ethics committee - additional information:** Beijing Tiantan Hospital, Capital Medical University, Beijing, China; No. KY-2019-050-02
**Author Disclosures:**
Li Yuna: Nothing to disclose

**A comparative evaluation of four commercially available artificial intelligence software solutions for brain volumetry and lesion segmentation in dementia**
*G. Di Cerbo*, G. Saltarelli, A. Innocenzi, M. Cella, C. De Felici, F. Bruno, A. Splendiani, E. Di Cesare; L'Aquila/IT
*(giovannidicerbo96@gmail.com)*

**Purpose or Learning Objective:** The purpose of this study is to compare the operating features and analysis outputs of four different commercially available software for brain volumetric analysis.
**Methods or Background:** We analyzed consecutive brain MRI scan of 32 patients (25 males, aged between 50 and 90 years) evaluated in a singles Institution for cognitive decline. All MRI examinations were performed on 3T scanner (GE MR750w.), including a volumetric T1 GRE sequence (slice 1 mm, TR 8.5, frequency FoV 25.6, phase FoV 0.8). MRI data were analyzed through four different dedicated softwares (S1, S2, S3, S4) after quality check by an experienced neuroradiologist. Volumetric output data of brain segmentation and volume for frontal, temporal, parietal, occipital lobes, hippocampus, and lateral ventricles, were collected and compared.
**Results or Findings:** The results revealed no significant consensus among the four artificial intelligence software applications in measuring various brain areas. S1-S2 showed non statistically significant output values in all brain regions. S1-S3 showed statistically significant differences in frontal and parietal lobe, lateral ventricles and hippocampus. S1-S4 showed statistically significant differences in frontal parietal and occipital lobe, and lateral ventricles. S2-S3 showed statistically significant differences in temporal and occipital lobes. S3-S4 showed statistically significant differences in parietal and occipital lobes, hippocampus and lateral ventricles.
**Conclusion:** Although AI software are becoming increasingly popular in clinical practice, the findings indicate a low degree of concordance among the four applications evaluated in this study. Therefore, clinicians integrating these tools into routine practice should be aware of the limited result interchangeability across different software platforms and consider their use as complementary aids rather than substitutes for clinical expertise.
**Limitations:** Small sample size
**Funding for this study:** None
**Ethics committee - additional information:** Local IRB
**Author Disclosures:**
Alessandra Splendiani: Nothing to disclose
Gaspare Saltarelli: Nothing to disclose
Antonio Innocenzi: Nothing to disclose
Ernesto Di Cesare: Nothing to disclose
Giovanni Di Cerbo: Nothing to disclose
Marco Cella: Nothing to disclose
Claudia De Felici: Nothing to disclose
Federico Bruno: Nothing to disclose

**Glymphatic dysfunction mediates the impact of tau pathology on neurodegeneration in cognitively unimpaired individuals and prodromal Alzheimer’s Disease**
X. Xu, B. Zhang, *Z. Zhu*; Nanjing/CN

**Purpose or Learning Objective:** To elucidate the pathological mechanism of glymphatic system dysfunction are associated with regional tau deposition and tau-mediated neurodegeneration across the preclinical and prodromal stage of the AD continuum.
**Methods or Background:** Cognitively normal (CN) controls (n=94), individuals with mild cognitive impairment (MCI; n = 83), and those with significant memory concern (SMC; n =84) were included from the Alzheimer's Disease Neuroimaging Initiative. Tau pathology was measured by positron emission tomography, the glymphatic activity assessed by diffusion tensor image analysis along the perivascular space (DTI-ALPS), and neurodegeneration reflected by hippocampal volume. Mediation analysis was used to study the possible pathways.
**Results or Findings:** ALPS was significantly associated with tau and tau-mediated neurodegeneration, especially in parahippocampal gyrus. The relationship between glymphatic function and neurodegeneration was mediated by tau pathological deposition (indirect effect: 0.012, 95%CI [0.001—0.029]) rather than ALPS index mediated the relationship between tau and neurodegeneration (indirect effect: 0.212, 95%CI [-0.013,0.0002]). The relationship between glymphatic dysfunction and cognitive decline were fully mediated by tau deposition and neurodegeneration in preclinical AD.
**Conclusion:** Tau deposition in specific region may mediate the relationship of glymphatic dysfunction and neurodegeneration, which contribute to cognitive decline in the preclinic AD stage, facilitating the development of therapeutics targeting tau protein and glymphatic dysfunction in AD.
**Limitations:** Firstly, the cross-sectional design employed in this study limits us to explore the causal relationships or investigate longitudinal changes over time, future longitudinal studies could provide further. Secondly, the ALPS index is mainly used to measure the function of the subcortical glymphatic system. Future research should continue to use other methods, such as BOLD-CSF coupling measurements, to validate the function of the subcortical glymphatic system in AD.
**Funding for this study:** This work was supported by the National Science and Technology Innovation 2030 -- Major program of "Brain Science and Brain-Like Research" (2022ZD0211800); the National Natural Science Foundation of China (82271965, 81971596, 82001793); the Fundamental Research Funds for the Central Universities, Nanjing University (2020-021414380462); the Key Scientific Research Project of Jiangsu Health Committee (K2019025); Special Funded Project of Nanjing Drum Tower Hospital (No. RC2022-023), Development Plan (Social Development) Project of Jiangsu Province (No. BE2022679). China Postdoctoral Science Foundation (2023M741648). The National Natural Science Foundation of China (82302172); The funders had no role in the study design, data collection and analysis, decision to publish, or preparation of the manuscript.
**Ethics committee - additional information:** ADNI Ethics committee
**Author Disclosures:**
Bing Zhang: Nothing to disclose
Zhengyang Zhu: Nothing to disclose
Xinru Xu: Nothing to disclose

**The Impact of Temporal Muscle Thickness as an Indicator of Sarcopenia on Clinical Status in Parkinson's Disease**
B. Atalay, K. Erincik, M. B. Doğan, *M. Gezgin*, H. Yıldız, F. B. Ozdilek; Istanbul/TR
*(me.egezgin@hotmail.com)*

**Purpose or Learning Objective:** To assess the impact of temporal muscle thickness (TMT), as an indicator of sarcopenia on cognitive status and medication dosage in patients with Parkinson's disease (PD).

**Methods or Background:** A total of 54 patients with PD and 46 healthy controls were retrospectively analyzed. Brain MR images from both groups were reviewed by two radiologists who independently measured the right and left temporal muscle thickness using T1-weighted axial images. Clinical assessments included the Unified Parkinson's Disease Rating Scale (UPDRS), Mini-Mental State Examination (MMSE), Hoehn and Yahr Scale, L-dopa equivalent daily dose (LEDD), and disease duration, collected by a neurologist. Interobserver agreement was evaluated using the intraclass correlation coefficient. The relationship between TMT and clinical data was analyzed using Spearman's correlation.
**Results or Findings:** In the PD group, 33.3% of patients were female, compared to 50% in the control group. Interobserver agreement for TMT measurements was excellent. No significant difference in TMT was observed between the PD and control groups (p=0.16, p=0.34). A weak but statistically significant correlation was found between TMT, LEDD, and disease duration, while no correlation was found between TMT and UPDRS or Hoehn and Yahr scores. A weak but significant correlation was observed between TMT and MMSE scores.
**Conclusion:** Sarcopenia, characterized by muscle mass loss, shares contributing factors with Parkinson's disease. TMT, a reliable marker of muscle mass, can be measured on brain MRIs to assess sarcopenia risk in PD patients. Our findings suggest that TMT correlates with cognitive function and medication dosage in PD patients, making it a valuable tool for early sarcopenia detection and management in clinical settings.
**Limitations:** Lack of patient follow-up and inability to assess the impact of muscle mass increase on medication dosage.
**Funding for this study:** No funding was provided for this study.
**Ethics committee - additional information:** The ethics committee notification can be found under the number 2023/0912.
**Author Disclosures:**
Merve Gezgin: Nothing to disclose
Hüseyin Yıldız: Nothing to disclose
Kendal Erincik: Nothing to disclose
Fatma Betül Ozdilek: Nothing to disclose
Başak Atalay: Nothing to disclose
Mahmut Bilal Doğan: Nothing to disclose

**Can a Coronal Swallow Tail Cleft Sign Increase the Confidence of Visualization of the Nigrosome-1 Layer of the Substantia Nigra in Normal Subjects and Those with Parkinsonism?**
*S. Rajan*, J. S. Chatha, H. Mahajan; New Delhi/IN
*(drsriramrajan@gmail.com)*

**Purpose or Learning Objective:** To assess whether reconstruction of phase images from susceptibility-weighted imaging (SWI) in a coronal plane enhances the visualization confidence of the nigrosome-1 layer in the substantia nigra. The coronal Swallow Tail cleft sign may serve as a valuable indicator for this structure.
**Methods or Background:** A retrospective review was conducted on 433 consecutive MR brain scans acquired using a routine protocol for various indications. The axial phase images of SWI were analyzed in Phase 1 by two radiologists with 9 and 22 years of experience. They evaluated the visibility of the nigrosome-1 layer below the level of the red nucleus using a 5-point Likert scale (1 = very difficult to 5 = easily seen). In Phase 2, coronal reconstructed images of the phase SWI were assessed perpendicularly to the substantia nigra in posterior sections for the presence of the cleft sign, also scored with the 5-point Likert scale.
**Results or Findings:** In Phase 1, the Likert scores of 1 and 2 were similar (6% and 9%, respectively). There was a marginal decrease in score 3 (from 4.97% to 4.04%). Scores of 4 decreased significantly from 16.97% in Phase 1 to 7.96% in Phase 2, while the score of 5 increased substantially from 62.01% in Phase 1 to 72.97% in Phase 2. Statistical analysis demonstrated that the addition of the coronal Swallow Tail cleft sign significantly enhanced confidence in visualizing the nigrosome-1 layer ($p < 0.01$).
**Conclusion:** The coronal Swallow Tail cleft sign significantly improves the confidence of visualization of the nigrosome-1 layer in both normal subjects and those with Parkinsonism. This finding underscores the utility of coronal reconstructions in enhancing diagnostic accuracy in neuroimaging.
**Limitations:** Correlation with nuclear scans or clinical history was not done
**Funding for this study:** None
**Ethics committee - additional information:** Restrospective
**Author Disclosures:**
Jagneet Singh Chatha: Nothing to disclose
Sriram Rajan: Nothing to disclose
Harsh Mahajan: Nothing to disclose

**Machine learning approach effectively discriminates between Parkinson's disease and progressive supranuclear palsy: multi-level indices of rs-fMRI**
*W. Cheng*; Nanchang/CN
*(ndyfy04042@ncu.edu.cn)*

**Purpose or Learning Objective:** Parkinson's disease (PD) and progressive supranuclear palsy (PSP) present similar clinical symptoms, but their treatment options and clinical prognosis differ significantly. Therefore, we aimed to discriminate between PD and PSP based on multi-level indices of rs-fMRI via the machine learning approach.
**Methods or Background:** A total of 58 PD and 52 PSP patients were prospectively enrolled in this study. Participants were randomly allocated to a training set and a validation set in a 7:3 ratio. Various resting-state functional magnetic resonance imaging (rs-fMRI) indices were extracted, followed by a comprehensive feature screening for each index. We constructed fifteen distinct combinations of indices and selected four machine learning algorithms for model development. Subsequently, different validation templates were employed to assess the classification results and investigate the relationship between the most significant features and clinical assessment scales.
**Results or Findings:** The classification performance of logistic regression (LR) and support vector machine (SVM) models, based on multiple index combinations, was significantly superior to that of other machine learning models and combinations when utilizing automatic anatomical labeling (AAL) templates. This has been verified across different templates.
**Conclusion:** The utilization of multiple rs-fMRI indices significantly enhances the performance of machine learning models and can effectively achieve the automatic identification of PD and PSP at the individual level.
**Limitations:** Only the rs-fMRI index was used in this study, and DTI-related microstructure data was not included.
**Funding for this study:** This study was supported by the National Natural Science Foundation of China (82160331), Jiangxi Province Double Thousand Talent Plan (jxsq2023201039). This project is implemented by the Jiangxi Clinical Research Center for Medical Imaging (20223BCG74001), and Jiangxi Province Key Laboratory for Precision Pathology and Intelligent Diagnosis (2024SSY06281).
**Ethics committee - additional information:** This study was approved by the Medical Ethics Committee of the First Affiliated Hospital of Nanchang University (approval number: IIT2022124).
**Author Disclosures:**
Weiling Cheng: Nothing to disclose

**Exploring the Relationship Between Body Composition and Brain Morphology in Aging: A Focus on Thigh Muscle Mass and Subcutaneous Fat as Predictors of Cortical Thickness in Healthy Older Adults**
*M. Sarkinaite*[1], U. Lukoseviciute[1], N. Masiulis[1], S. Lukoševičius[1], O. Levin[2], R. Gleiznienė[1]; [1]Kaunas/LT, [2]Leuven/BE
*(milda.sarkinaite@gmail.com)*

**Purpose or Learning Objective:** Subcutaneous fat accumulation has been linked to adverse brain health outcomes. This study examines the relationship between thigh muscle mass, subcutaneous fat distribution, and brain structure in elderly adults. It explores how body composition affects cortical thickness in brain regions linked to cognitive function.
**Methods or Background:** Fifty-four healthy elderly individuals underwent imaging of the right thigh and brain using a 3T Siemens Avanto MRI system. Muscle and subcutaneous fat cross-sectional areas (CSA) were measured at 50% and 20% of thigh length, with the muscle-to-fat ratio calculated at the 50% mark. Cortical thickness was assessed through brain volumetric analysis using Freesurfer 7.4.1 software.
**Results or Findings:** Significant positive correlations ($r \geq 0.2$, $p \leq 0.05$) were found between the muscle-to-fat ratio and cortical thickness in the left cerebellum, cuneus, and transverse temporal cortex, in the right entorhinal cortex, inferior temporal cortex, postcentral gyrus, superior temporal cortex, and the banks of the superior temporal sulcus (BANKSSTS). Additionally, significant negative correlations ($r \leq -0.2$, $p \leq 0.05$) were observed between subcutaneous fat CSA at 50% of thigh length and cortical thickness in the left cuneus and entorhinal cortex, in the right BANKSSTS, postcentral gyrus, and superior temporal cortex. Additionally, subcutaneous fat CSA at 20% of thigh length was inversely correlated with cortical thickness in the left cuneus and right BANKSSTS ($r \leq -0.2$, $p \leq 0.05$).
**Conclusion:** Our findings demonstrate that increased thigh muscle mass correlates with greater cortical thickness in cognitive regions, while elevated subcutaneous fat is linked to reduced thickness. These results highlight the role of body composition in maintaining brain health in the elderly and underscore the importance of muscle mass in mitigating age-related cortical decline.
**Limitations:** With only 54 participants, the study's findings may lack generalizability.
**Funding for this study:** Supported by the Research Council of Lithuania (grant number P-MIP-22-217).

**Ethics committee - additional information:** Approved by the Kaunas region Medical Ethics Committee for Biomedical Research (No. BE-2-22).
**Author Disclosures:**
Milda Sarkinaite: Nothing to disclose
Nerijus Masiulis: Nothing to disclose
Saulius Lukoševičius: Nothing to disclose
Rymantė Gleiznienė: Nothing to disclose
Urte Lukoseviciute: Nothing to disclose
Oron Levin: Nothing to disclose

**Age-related hearing loss may be associated with small vessel disease. Peak skeletonized mean diffusivity and TBSS study**
B. Genç, *I. C. Koc*, K. Aslan; Samsun/TR

**Purpose or Learning Objective:** Peak skeletonized mean diffusivity (PSMD) is being proposed as a novel biomarker for small vessel disease. Tract-Based Spatial Statistics (TBSS) is a well-established DTI analysis method that enables the fully automated detection of microstructural changes in white matter. The aim of this study is to investigate white matter changes in patients with age-related hearing loss using both PSMD and TBSS.
**Methods or Background:** All individuals from the OpenNeuro hearing loss connectome dataset were included in the study (https://openneuro.org/datasets/ds005026). The dataset consisted of 52 hearing loss (HL) patients and 30 healthy controls. From the DTI images in the dataset, FA, MD, RD, and AD were obtained using FSL with preprocessing steps including TOPUP and eddy. The standard TBSS procedure was applied to investigate group differences in FA, MD, RD, and AD. The standard PSMD method with histogram analysis was used to calculate the difference between the 95th and 5th percentile MD values (https://www.psmd-marker.com/). A comparison between the groups was made.
**Results or Findings:** The TBSS analysis did not show any differences between the groups for any of the DTI parameters. The PSMD value in the HL group was 225,21±23,50 x 10^-6 mm²/s, while in the control group it was 214,53±24,01 x 10^-6 mm²/s, showing a statistically significant increase in the hearing loss group (p=0,039).
**Conclusion:** Our findings suggest that small vessel disease may underlie the pathophysiology in patients with age-related hearing loss. To our knowledge this is the first study to show the relationship between age-related hearing loss and small vessel disease.
**Limitations:** Since numerical data of hearing tests were not available, the correlation between PSMD data and hearing test could not be analyzed.
**Funding for this study:** No funding was received for this study.
**Ethics committee - additional information:** OpenNeuro permits the use of patient data under the CC0 license. Ethical approval has already been obtained by the "University of Salerno." Therefore, no separate ethical approval has been obtained from our institution.
**Author Disclosures:**
Bariş Genç: Nothing to disclose
Kerim Aslan: Nothing to disclose
Irem Ceren Koc: Nothing to disclose

**Glymphatic Dysfunction Correlate with Spatial Navigation Deficits in Subjective Cognitive Decline: Insights from 5.0T MRI and Plasma Biomarkers Analysis**
F. Chen[1], B. Zhang[1], Q. Chen[1], X. Fan[2], L. Zou[2], Y. Li[2], G. Cheng[2], *G. Danni*[1]; [1]Nanjing/CN, [2]Shenzhen/CN
*(gedniii@163.com)*

**Purpose or Learning Objective:** To assess the feasibility of analysis along the perivascular space (ALPS) using DTI obtained from 5.0 Tesla MR, assess glymphatic system function in SCD and its correlation with spatial navigation abilities and plasma biomarkers.
**Methods or Background:** Glymphatic dysfunction is implicated in cognitive impairment associated with AD. Spatial navigation impairments are among the earliest manifestations in individuals with SCD. However, the relationship between glymphatic function and spatial navigation remains poorly understood. Between May 2023 and January 2024, 62 SCD patients and 62 matched controls underwent high-resolution DTI on 5.0T MR scanner, spatial navigation behavioral tests, cognitive assessments, and Simoa plasma biomarker analyses. The ALPS index reflecting glymphatic activity was calculated by a ratio of the diffusivities along the x-axis in the projection and association neural fibers to the diffusivities perpendicular to them and compared according to the groups with use of multivariate analysis of variance. Inter-reproducibility of ALPS index among 5.0TMR and 3.0TMR scanners was evaluated using consistency interclass correlation coefficient. Pearson correlation analysis was used to assess the relationship between cognitive performance, spatial navigation performance, plasma biomarkers, and the ALPS index.
**Results or Findings:** The ALPS on 5.0TMR and ALPS on 3.0TMR showed strong consistency and correlation. SCD patients had significantly lower ALPS index on 5.0TMR and higher navigation errors compared to controls. The ALPS index was positively correlated with spatial navigation, cognitive performance, and memory performance, and negatively correlated with plasma pTau217 levels.
**Conclusion:** The glymphatic function is impaired in SCD at the preclinical AD stage, which may represent one of the physiological mechanisms leading to deficits in spatial navigation abilities. DTI-ALPS on 5.0T MR may serve as a sensitive neuroimaging biomarker for the preclinical stage of AD.
**Limitations:** Our study is a small-sample cross-sectional study.
**Funding for this study:** National Science and Technology Innovation 2030 -- Major program of "Brain Science and Brain-Like Research" (2022ZD0211800)
**Ethics committee - additional information:** The Research Ethics Committees of Nanjing Drum Tower Hospital, the Shenzhen Institute of Advanced Technology of the Chinese Academy of Sciences, and Peking University Shenzhen Hospital.
**Author Disclosures:**
Qian Chen: Nothing to disclose
Ye Li: Nothing to disclose
Xiang Fan: Nothing to disclose
Guanxun Cheng: Nothing to disclose
Bing Zhang: Nothing to disclose
Ge Danni: Nothing to disclose
Futao Chen: Nothing to disclose
Lixian Zou: Nothing to disclose

**Static and Dynamic Functional Connectivity Alternations of Medial and Lateral Entorhinal Cortex with Subjective Cognitive Decline**
*G. Danni*, Z. Bing; Nanjing/CN
*(gedniii@163.com)*

**Purpose or Learning Objective:** To investigate the static functional connectivity (sFC) and dynamic functional connectivity (dFC) of medial entorhinal cortex (MEC) and lateral entorhinal cortex (LEC) in individuals with subjective cognitive decline (SCD) and the associations with cognitive performance, spatial navigation and olfactory memory.
**Methods or Background:** Seventy-seven control subjects and 106 SCD individuals were enrolled, and neuropsychological evaluations, 2D computerized spatial navigation test, olfactory memory test and resting-state functional magnetic resonance imaging (rs-fMRI) were collected. Bilateral MEC and LEC were selected as seeds to investigate alternations of the volumes, sFC and dFC.
**Results or Findings:** Compared to control subjects, SCD individuals exhibited decreased sFC between bilateral LEC and visual network, between right LEC and left posterior cingulate gyrus and sensory motor network, and between right MEC and left hippocampus, visual network and sensory motor network. The dFC between right LEC and right triangular part of inferior frontal gyrus (IFGtriang) decreased, while dFC between left MEC and right putamen, and between right MEC and right middle temporal gyrus increased. In SCD group, volumes of bilateral MEC were positively correlated with spatial navigation ability, and sFC between bilateral LEC and visual network was positively correlated with olfactory memory. The dFC between right LEC and right IFGtriang was correlated positively with global cognitive performance. The combination of sFC and dFC as biomarkers to identify SCD showed an area under curve of 92.1%.
**Conclusion:** There were functional alternations of EC subregions in SCD individuals, and we demonstrated the association between LEC and spatial navigation, and MEC and olfactory memory. The combination of sFC and dFC may be a new neuroimaging biomarker for the early diagnosis of AD.
**Limitations:** The study lacked genetic and biomarker data. Also, we didn't have follow-up data to track pathological progression.
**Funding for this study:** National Science and Technology Innovation 2030_Major program of "Brain Science and Brain-Like Research" (No. 2022ZD0211800)
**Ethics committee - additional information:** Nanjing Drum Tower Hospital Ethics Committee
**Author Disclosures:**
Ge Danni: Author: Department of Radiology, the Affiliated Drum Tower Hospital of Nanjing University Medical School
Zhang Bing: Nothing to disclose

**Automated cerebral microhemorrhage detection on T2* GRE for Alzheimer's disease screening**
S. Van Eyndhoven, *R. Magalhaes*, R. Khan, T. V. Phan, A. Liseune, A. Brys, D. M. Sima, J. Verheyden, A. Ribbens; Leuven/BE
*(ricardo.magalhaes@icometrix.com)*

**Purpose or Learning Objective:** Develop a robust automated deep learning-based method for assessment of cerebral microhemorrhages on T2* gradient-echo (GRE) images.
**Methods or Background:** Hypointensities on GRE images can be indicative of cerebral microhemorrhages, and serve as exclusionary criteria for anti-amyloid

Wednesday therapies for Alzheimer's disease, as they are linked to increased risk of intracerebral hemorrhage. Automated detection tools could greatly aid radiologists in the challenging task of accurately quantifying these findings. A deep learning model was developed to detect microhemorrhages on cross-sectional GRE images. Training was done using 247 2D GRE images and accompanying manual microhemorrhage annotations from the EMERGE clinical trial (NCT02484547). Detection accuracy was evaluated on a stratified subset of cases (N=600) of the Alzheimer's Disease Neuroimaging Initiative, where up to 10 microhemorrhages were annotated on a 2D GRE sequence by experts. This is representative of the population that would be screened via MRI before administration of anti-amyloid treatment, for which assessment is more challenging relative to cases with a large number of microhemorrhages. For each case, we evaluated the F1 score, i.e., the harmonic mean between detection sensitivity and positive predictive value, and the absolute error between the number of microhemorrhages according to the expert ground truth and the automated count.
**Results or Findings:** The median F1 score of the trained model was 0.67, and its median absolute count error was 1 microhemorrhage.
**Conclusion:** Detection of microhemorrhages on 2D GRE images is a challenging task, which is gaining importance with the advent of novel anti-amyloid treatments that may lead to hemorrhagic side effects. Using a robust, validated AI tool, as described here, can assist radiologists in microhemorrhage detection, providing value especially in sparse MR images, though expert assessment remains vital.
**Limitations:** None.
**Funding for this study:** N/A
**Ethics committee - additional information:** N/A
**Author Disclosures:**
Arne Brys: Employee: icometrix
Ricardo Magalhaes: Employee: icometrix
Annemie Ribbens: Employee: icometrix Shareholder: icometrix
Rafay Khan: Employee: icometrix
Diana M. Sima: Employee: icometrix
Thanh Vân Phan: Employee: icometrix
Simon Van Eyndhoven: Employee: icometrix
Arno Liseune: Employee: icometrix
Jan Verheyden: Shareholder: icometrix Employee: icometrix

**13:00-14:30 Research Stage 4**

### Research Presentation Session: Abdominal and Gastrointestinal

### RPS 401

### Estimation of liver fat and stiffness with imaging

**Moderator**
J. M. Lee; Seoul/KR
*(jmlshy2000@gmail.com)*

**The Diagnostic Accuracy of Quantitative Ultrasound with Fat Fraction parameter for the Assessment of Hepatic Steatosis in Patients with Metabolic-Dysfunction Associated Fatty Liver Disease**
R. Cannella, A. A. Blandino, A. Tulone, S. Petta, *T. V. Bartolotta*; Palermo/IT
*(tv_bartolotta@yahoo.com)*

**Purpose or Learning Objective:** To investigate the performance of quantitative ultrasound with US Fat Fraction (USFF) in patients with metabolic dysfunction-associated steatotic liver disease (MASLD).
**Methods or Background:** This study included consecutive patients with MASLD who prospectively underwent MRI and quantitative ultrasound on the same day. MRIs were acquired on a 3T scanner and the fat fraction quantification was obtained with mDixonQuant sequence. Quantitative ultrasound consisted of Tissue Attenuation Imaging (TAI), Tissue Scatter-distribution Imaging (TSI), and US Fat Fraction (USFF) acquired by two operators (a radiologist and a radiology resident) to evaluate the inter-reader reliability. Spearman's rank-order correlation was calculated between USFF and MRI fat fraction. The diagnostic performance was investigated with the area under the receiver operating characteristics curve (AUC), sensitivity and specificity according to the optimal cutoff.
**Results or Findings:** Fifty-nine patients (40 males, median age of 60 years) were enrolled. Among them, 47 (79.7%) had grade≥1 steatosis and 23 (39.0%) had grade≥2 steatosis. There was a high positive correlation between USFF and MRI fat fraction (rho: 0.877, p<0.001). For the diagnosis of grade≥1 steatosis the AUCs were 0.959 (95%CI: 0.778, 0.999) for TAI, 0.847 (95%CI: 0.631, 0.963) for TSI, and 0.988 (95%CI: 0.825, 1.000) for USFF. An USFF >10.0 had a sensitivity 94.1% and a specificity of 100% for grade≥1 steatosis. For the diagnosis of grade≥2 steatosis, the AUCs were 0.958 (95%CI: 0.777, 0.999) for TAI, 0.858 (95%CI: 0.757, 0.916) for TSI, and 0.958 (95%CI: 0.777, 0.999) for USFF. An USFF >11.8 had a sensitivity 100% and a specificity of 83.3% for grade≥2 steatosis. The reproducibility of USFF was excellent (ICC of 0.98; 95%CI: 0.95, 0.98).
**Conclusion:** USFF provides an excellent performance and reproducibility for the quantification of hepatic steatosis in patients with MASLD.
**Limitations:** Lack of liver biopsy.
**Funding for this study:** Study supported by Samsung.
**Ethics committee - additional information:** All participants provided written informed consent.
**Author Disclosures:**
Adele Tulone: Nothing to disclose
Antonino Andrea Blandino: Nothing to disclose
Roberto Cannella: Research/Grant Support: Co-funding by the European Union - FESR or FSE, PON Research and Innovation 2014-2020 - DM 1062/2021; research collaboration with Siemens Healthineers. Other: Support for attending meetings from Bracco and Bayer
Salvatore Petta: Nothing to disclose
Tommaso Vincenzo Bartolotta: Nothing to disclose

**Multiparametric ultrasound evaluation of hepatic fibrosis and steatosis in patients treated with bempedoic acid: a comparative study on 100 patients**
*G. Daccordi*, P. Sacco, F. Lazzeretti, G. De Filippo, C. Tucci, M. A. Mazzei; Siena/IT
*(g.daccordi@student.unisi.it)*

**Purpose or Learning Objective:** This study evaluates the effectiveness of bempedoic acid in improving hepatic steatosis using multiparametric ultrasound and serum lipid levels, comparing results with a control group.
**Methods or Background:** Background: Hepatic steatosis is associated with dyslipidemia and liver dysfunction. Multiparametric ultrasound, using techniques like shear-wave elastography and attenuation imaging (ATI), offers a non-invasive assessment of liver fibrosis and steatosis. Bempedoic acid is a potential therapy to reduce liver fat and improve lipid profiles. Materials and methods: We enrolled 100 patients with hepatic steatosis: 50 received bempedoic acid therapy, and 50 served as controls. Ultrasound was performed using the Canon Aplio i800, evaluating fibrosis via shear-wave elastography, and steatosis using ATI and dispersion imaging. Two operators with different experience levels performed the exams at baseline and after treatment. Serum cholesterol, triglycerides, and liver enzymes were also monitored.
**Results or Findings:** Significant improvements in fibrosis, steatosis, and dispersion were observed in the treatment group ($p < 0.001$). No significant changes were found in the control group ($p > 0.05$), highlighting the utility of multiparametric ultrasound for follow-up. Good inter-operator agreement was noted for ATI (ICC = 0.96), with slight discordance in fibrosis evaluation (ICC = 0.88), likely due to experience differences.
**Conclusion:** Multiparametric ultrasound plays a fundamental role for the non-invasive follow-up of hepatic fibrosis and steatosis during therapy. This approach is a valuable tool for assessing patient response, aiding in informed clinical decisions.
**Limitations:** Ongoing single-center study
**Funding for this study:** Not addictional funding
**Ethics committee - additional information:** University of Siena
**Author Disclosures:**
Maria Antonietta Mazzei: Nothing to disclose
Cristina Tucci: Nothing to disclose
Francesco Lazzeretti: Nothing to disclose
Palmino Sacco: Nothing to disclose
Giorgia Daccordi: Nothing to disclose
Giovanna De Filippo: Nothing to disclose

**Functional Liver Imaging Score: A biomarker for prediction of acute-on-chronic liver failure**
*A. Kristic*, L. Balcar, A. Ba-Ssalamah, T. Reiberger, M. Mandorfer, N. Bastati-Huber, R. Ambros, L. Beer, S. Pötter-Lang; Vienna/AT

**Purpose or Learning Objective:** The Functional Liver Imaging Score (FLIS) derived from gadoxetic acid (GA)-enhanced MRI is a prognostic biomarker in patients with advanced chronic liver disease (ACLD). The aim of this study was to investigate whether FLIS, as well as quantitative imaging parameters, including the relative liver enhancement (RLE), relative enhancement ratio of the biliary system (REB), and liver-to-portal vein contrast ratio (LPC), can predict acute-on-chronic liver failure (ACLF; a syndrome defined by extrahepatic organ failure and high short-term mortality) in patients with an acute decompensation (AD) of cirrhosis, i.e., the main at-risk population.

**Methods or Background:** We included 322 ACLD patients with GA-MRI-derived, semi-quantitative FLIS, in whom the RLE, REB, and LPC were also computed by two independent radiologists. Patients were stratified into stable ACLD (compensated or non-hospitalized/electively hospitalized/non-liver-related hospitalized decompensated ACLD patients) and acutely decompensated (AD) patients (non-elective liver-related hospitalization). The predictive values of semi-quantitative FLIS and quantitative GA-MRI parameters for ACLF development were investigated by Cox regression analyses.
**Results or Findings:** The FLIS was lower in AD (vs. stable ACLD) patients. Furthermore, low FLIS was an independent risk factor for ACLF development/liver-related death in AD patients (adjusted hazard ratio [aHR]: 2.26; 95%CI: 1.08-4.71; P=0.03), as well as in clinically stable ACLD patients (aHR: 2.35; 95%CI: 1.21-4.55; P=0.01). Conversely, while RLE, REB, and LPC distinguished between AD and clinically stable ACLD patients (P<0.001), they failed to predict ACLF/liver-related death.
**Conclusion:** The FLIS is a simple prognostic imaging biomarker in AD patients in whom ACLF risk stratification is important to identify patients who may benefit from intensified monitoring or timely liver transplant evaluation.
**Limitations:** The retrospective design could have led to a selection bias; however, the study allowed for a long clinical follow-up and a high number of endpoints.
**Funding for this study:** No funding was received for this study.
**Ethics committee - additional information:** Received approval EK 2023/2017.
**Author Disclosures:**
Antonia Kristic: Nothing to disclose
Sarah Pötter-Lang: Nothing to disclose
Lorenz Balcar: Speaker: received speaker fees from Chiesi, and Gilead.
Raphael Ambros: Nothing to disclose
Mattias Mandorfer: Advisory Board: served as a speaker and/or consultant and/or advisory board member for AbbVie, Collective Acumen, Echosens, Gilead, Ipsen, Takeda, and W. L. Gore & Associates. Other: received travel support from AbbVie and Gilead. Consultant: served as a speaker and/or consultant and/or advisory board member for AbbVie, Collective Acumen, Echosens, Gilead, Ipsen, Takeda, and W. L. Gore & Associates. Grant Recipient: received grants from Echosens. Speaker: served as a speaker and/or consultant and/or advisory board member for AbbVie, Collective Acumen, Echosens, Gilead, Ipsen, Takeda, and W. L. Gore & Associates.
Lucian Beer: Speaker: received speaker fees from Takeda, and Lilly.
Ahmed Ba-Ssalamah: Speaker: received honoraria for lectures and a consultancy from Bayer without relation to the present article. Consultant: received honoraria for lectures and a consultancy from Bayer without relation to the present article.
Nina Bastati-Huber: Nothing to disclose
Thomas Reiberger: Speaker: speaking honoraria from AbbVie, Gilead, W. L. Gore & Associates, Intercept, Roche, and MSD. Grant Recipient: received grant support from AbbVie, Boehringer-Ingelheim, Gilead, Intercept, MSD, Myr Pharmaceuticals, Philips Healthcare, Pliant, Siemens, and W. L. Gore & Associates. Other: and travel support from AbbVie, Boehringer-Ingelheim, Gilead, and Roche. Consultant: consulting/advisory board fees from AbbVie, Bayer, Boehringer-Ingelheim, Gilead, Intercept, MSD, and Siemens; and travel support from AbbVie, Boehringer-Ingelheim, Gilead, and Roche. Advisory Board: consulting/advisory board fees from AbbVie, Bayer, Boehringer-Ingelheim, Gilead, Intercept, MSD, and Siemens.

### A compound model improves the accuracy of ultrasound-estimated fat-fraction

*P. N. Kaposi-Novák*, B. Zsély, Z. Zsombor, M. Himsel, V. Bérczi, G. Györi, P. Maurovich-Horvat, A. D. Rónaszéki; Budapest/HU

**Purpose or Learning Objective:** The ultrasound-estimated fat-fraction (UEFF) is new biomarker that can facilitate the diagnosis and follow-up of liver steatosis. Different models have been devised to calculate UEFF, but these have yet to be compared side-by-side.
**Methods or Background:** We retrospectively collected ultrasound parameters from sixty patients with various grades (S0-S3) of metabolic dysfunction-associated steatotic liver disease (MASLD), including attenuation coefficient (AC), backscatter-distribution coefficient (BSC), and liver capsule-to-skin distance (CSD). The training set had balanced distribution of steatosis grades (S0, S1, S3 - 16 cases each, S2 - 12 cases). Univariable and multivariable linear and exponential models were trained to predict the MRI proton density fat-fraction (PDFF) using repeated cross-validation. The models were tested on fifty cases for which the scanner's application calculated an ultrasound fat-fraction (USFF). We compared the R-squared (R2) and the one-way random effect intraclass correlation coefficients (ICC) among the models.
**Results or Findings:** In low-grade steatosis (≤ S1), the linear model using AC and BSC achieved the best fit (R2= 0.379, p<0.002), and USFF had the best agreement (ICC=0.538, p<0.003) with PDFF. In high-grade steatosis (≥ S2), the ASC and BSC-based multi-exponential model performed best (R2= 0.252, p<0.008, ICC=0.422, p<0.012). The fit could be improved by including CSD in the model (R2=0.263, p<0.007). The univariable nonlinear AC model resulted in a slightly weaker agreement in both low-grade (ICC=0.236, p<0.019) and high-grade (ICC=0.227, p<0.012) steatosis. A compound model using linear regression in low-grade or nonlinear regression in high-grade steatosis outperformed (R2= 0.6, p<0.001, ICC=0.849, p<0.001) other models in predicting the test cases.
**Conclusion:** The UEFF is a robust method to diagnose liver steatosis across all stages. The compound model substantially improves the fit and the agreement between UEFF and PDFF.
**Limitations:** Single center study.
**Funding for this study:** Pál Novák Kaposi was recipient of a research grant from Samsung Medison Ltd. (Contract number: SE4K/2023/195).
Zita Zsombor was recipent of a resarch scholarship from the New National Excellence Program (ÚNKP-23-3-I-SE-23) of the Hungarian Ministry of Culture and Innovation.
**Ethics committee - additional information:** Semmelweis University's Regional and Institutional Science and Research Ethics Committee (Protocol number: SE RKEB 140/2020, 16 July 2020, and SE RKEB 6/2023, 9 February 2023)
**Author Disclosures:**
Pál N. Kaposi-Novák: Research/Grant Support: Samsung Medison Ltd.
Aladár David Rónaszéki: Nothing to disclose
Pál Maurovich-Horvat: Nothing to disclose
Boglárka Zsély: Nothing to disclose
Marco Himsel: Nothing to disclose
Viktor Bérczi: Nothing to disclose
Zita Zsombor: Nothing to disclose
Gabriella Györi: Nothing to disclose

### Dual-Energy CT Liver Fat Quantification as Imaging Biomarker of Mortality and Morbidity in Intensive-Care Patients

*J. Erley*, J. Breckow, K. Roedl, A. Duoerkongjiang, G. De Heer, E. Tahir, J. Yamamura, G. Adam, I. Molwitz; Hamburg/DE

**Purpose or Learning Objective:** This study aimed to evaluate the association between liver fat content, assessed using dual-energy computed tomography (DECT) material decomposition, in immobilized intensive care unit (ICU) patients with in-hospital mortality, length of ICU stay, and indicators of morbidity (need for tracheotomy and renal replacement therapy).
**Methods or Background:** ICU patients who received a DECT upon ICU admission between November 2019 and December 2022 were retrospectively investigated. DECT liver fat fraction (DECT-FF) was determined by material decomposition for fat, liver tissue, and iodine by combining two regions of interest (ROI) in the right and one ROI in the left liver lobe (min. size 3.6 cm2). Cox proportional hazard models were employed, including DECT-FF, sex, age, body mass index, ICU scoring systems for disease prediction, reason for admission, pre-existing malignancies, chronic diseases, and inflammatory diseases.
**Results or Findings:** In total 76 patients were included (33 female, mean age 61±12 years, of which 59% died in the hospital. DECT-FF at CT1 was 3.3±5.4%. DECT-FF was associated with in-hospital mortality (hazard ratio 1.09 [95% confidence interval 1.03; 1.15], p=0.004), with the length of ICU-stay (odds ratio (OR) -4.28 [-6.64; -1.92], p=0.001) and with the need for a tracheotomy (OR: 0.90 [0.80; 0.99], p=0.039). No association was observed between DECT-FF and ICU scoring systems or renal replacement therapy.
**Conclusion:** A higher liver DECT-FF upon ICU admission was associated with in-hospital mortality and, probably as a consecutive effect/bias, with a decreased length of ICU stay and a lower likelihood of tracheotomy in surviving patients. The liver DECT-FF may serve as a predictive imaging biomarker of mortality in critically ill patients.
**Limitations:** The sample size and its heterogeneity.
**Funding for this study:** Not applicable.
**Ethics committee - additional information:** The study has been approved by the ethics committee of the medical association in Hamburg.
**Author Disclosures:**
Gerhard Adam: Nothing to disclose
Isabel Molwitz: Nothing to disclose
Alidan Duoerkongjiang: Nothing to disclose
Geraldine De Heer: Nothing to disclose
Kevin Roedl: Nothing to disclose
Jin Yamamura: Nothing to disclose
Jennifer Erley: Nothing to disclose
Julia Breckow: Nothing to disclose
Enver Tahir: Nothing to disclose

**Accuracy of estimates of liver fat content based on 2point-Dixon in comparison to multi-echo-Dixon sequences in a population-based cohort**
*M-N. Von Itter*[1], T. Nonnenmacher[2], T. Norajitra[2], S. Rospleszcz[1], J. Machann[3], F. Bamberg[1], J. Nattenmüller[1]; [1]Freiburg/DE, [2]Heidelberg/DE, [3]Tübingen/DE

**Purpose or Learning Objective:** The rising prevalence of metabolic dysfunction-associated steatotic liver disease (MASLD), formerly NAFLD, makes it a leading liver disease and a risk factor for steatohepatitis, cirrhosis, and hepatocellular carcinoma. As it is potentially reversible, early detection is key.
**Methods or Background:** A validated nnU-Net liver segmentation model processed 10,636 MRI scans from NAKO participants using 2-point- (2p) and multi-echo- (me) Dixon data. Samples with significant mask mismatches were excluded. The segmentation masks were used to calculate mean liver fat content (LFC) from 2p-Dixon data, which was then compared to the me-Dixon proton density fat fraction.
**Results or Findings:** The overall mean difference between the fat fraction from me-Dixon measurements and the 2p-Dixon fat estimation is -1.101±0.012%. 95% of the measured differences are within the range of -3.8% and 1.4%. Mean LFCs from 2p-Dixon data overestimate the fat content compared to me-Dixon, especially for higher fat deposition. Liver iron content did not lead to a systemic offset in our cohort, though only participants with mild to moderate liver iron content were part of our sample.
**Conclusion:** As the 2p-Dixon sequence is often acquired for various clinical questions beyond specific liver imaging, assessment of 2p-Dixon-based LFC can identify individuals with MASLD in possible screening programs or MRI studies for other reasons and guide them to the complementary acquisition of a me-Dixon sequence. This could benefit individuals by initiating preventive measures and reduce the socio-economic burden of MASLD caused by its complications.
**Limitations:** No histological confirmation was performed.
**Funding for this study:** Funding was received from the German Research Foundation (Deutsche Forschungsgemeinschaft, grant number: 428224476). The NAKO is funded by the Federal Ministry of Education and Research (BMBF) [project funding reference numbers: 01ER1301A/B/C and 01ER1511D], the federal states and the Helmholtz Association, with additional financial support by the participating universities and the institutes of the Leibniz association.
**Ethics committee - additional information:** The study received institutional review board approval and written informed consent was obtained from all participants.
**Author Disclosures:**
Tobias Nonnenmacher: Nothing to disclose
Susanne Rospleszcz: Nothing to disclose
Johanna Nattenmüller: Nothing to disclose
Jürgen Machann: Nothing to disclose
Fabian Bamberg: Nothing to disclose
Marc-Nicolas Von Itter: Nothing to disclose
Tobias Norajitra: Nothing to disclose

**Deep learning-based liver volume and fat fraction quantification from Dixon-MRI: Reference curves from over 66,000 individuals and their prognostic value**
*M. Jung*[1], L. Michel[2], M. Reisert[2], S. Jäck[2], S. Rospleszcz[2], M. T. Lu[1], F. Bamberg[2], V. Raghu[1], J. Weiß[2]; [1]Boston, MA/US, [2]Freiburg/DE
*(matthias.jung@uniklinik-freiburg.de)*

**Purpose or Learning Objective:** Steatotic liver disease (SLD) is a major public health concern with a global prevalence of 32.4% and an independent risk factor for cardiometabolic and liver disease. We used a deep learning framework to quantify liver volume and SLD from MRI in a large Western European population to calculate reference curves and investigate their prognostic value.
**Methods or Background:** We developed a deep-learning model that takes an MRI as input and outputs liver volume (L) and fat fraction (FF, %) using data from the UK Biobank (UKBB) and German National Cohort (NAKO). Established FF-thresholds were used to define mild(5%), moderate(15%), and severe(25%) SLD. We computed age-, sex-, and height-normalized reference curves and assessed the prognostic value of liver volume z-score (z<1; z=1-2; z>2) and SLD-categories for incident outcomes (diabetes; liver disease; all-cause mortality) in the UKBB (n=35,002). Cox regression assessed the association between volume z-score and SLD categories with outcomes after adjustment for age, sex, BMI, race, and cardiometabolic risk factors (serum glucose, Hb1Ac, lipid panel, prevalent hypertension, history of cancer, alcohol consumption, smoking status).
**Results or Findings:** Among 66,664 individuals from the general population (57.7±12.9 years; BMI: 26.2±4.5 kg/m2, 48.3% female), SLD was high with a prevalence of 80.4%. In the UKBB, multivariable-adjusted Cox regression showed that severe steatotic liver disease (SLD) was associated with an increased risk of incident diabetes (adjusted hazard ratio [aHR] 2.66) and liver disease (aHR 6.34) compared with no SLD. A liver volume z-score >2 was associated with higher all-cause mortality (aHR 2.25) compared with a z-score <1.
**Conclusion:** SLD and normalized liver volume categories predicted outcomes beyond traditional risk factors. We will release open-source reference curves to enhance clinical liver risk assessment and improve comparability in research.
**Limitations:** Predominantly white population.
**Funding for this study:** This project was conducted with data from the German National Cohort (NAKO) (www.nako.de). The NAKO is funded by the Federal Ministry of Education and Research (BMBF) [project funding reference numbers: 01ER1301A/B/C, 01ER1511D, and 01ER1801A/B/C/D], federal states of Germany, and the Helmholtz Association, the participating universities and the institutes of the Leibniz Association. This research has been conducted using the UK Biobank Resource under Application Number 80337. We thank all participants who took part in the NAKO and UKBB study and the staff of these research initiatives. MJ was funded by the Deutsche Forschungsgemeinschaft (DFG, German Research Foundation) - 518480401. VKR was funded by Norn Group Longevity Impetus Grant, NHLBI K01HL168231, and AHA Career Development Award 935176.
**Ethics committee - additional information:** Informed consent was obtained from all participants in the UK Biobank and the German National Cohort study. In addition, we received local IRB approval (IRB of the University of Freiburg: 23-1316-S1-retro and 24-1099-S1-retro).
**Author Disclosures:**
Susanne Rospleszcz: Nothing to disclose
Marco Reisert: Nothing to disclose
Jakob Weiß: Nothing to disclose
Lea Michel: Nothing to disclose
Matthias Jung: Nothing to disclose
Fabian Bamberg: Nothing to disclose
Saskia Jäck: Nothing to disclose
Vineet Raghu: Nothing to disclose
Michael T. Lu: Nothing to disclose

**Intra-individual quantitative crossover comparision of liver fat measurements between free-breathing radial GRE and conventional cartesian GRE breath-hold methods**
*T. B. Rodrigues*[1], M. D. Santana[1], V. Hérida[1], N. Almeida[1], T. Castela[1], R. C. Semelka[2], M. Ramalho[1]; [1]Lisbon/PT, [2]Chapel Hill, NC/US
*(teresabaratarodrigues@gmail.com)*

**Purpose or Learning Objective:** To evaluate and compare liver fat fraction measurements derived from a free-breathing radial sequence with those obtained from reference cartesian breath-hold techniques in an intra-individual fashion.
**Methods or Background:** The study included 40 subjects (19 males, 21 females; mean age 60.5 ± 13.2) who underwent MRI examinations.Three T1-weighted sequences were used: Cartesian 2D-FLASH dual gradient-echo(2D-GRE), Cartesian 3D-GRE with Dixon technique, and free-breathing 3D-GRE with radial data sampling(Radial 3D-GRE). Two independent readers measured the mean region of interest (ROI) values of the liver for in-phase (IP) and out-of-phase (OP) images for each sequence with an equal ROI and similar location. Quantitative liver fat fractions (FF) were calculated using ((SI(IP)−SI(OP))/(2 × SI(IP))×100. The liver FF were compared across sequences. The inter-reader agreement was assessed using the intraclass correlation coefficient (ICC).Pearson correlation and regression analyses examined relationships among different measurement techniques. P-value of <0.05 was considered significant.
**Results or Findings:** The study found strong correlations between liver FF measurements across 2D-GRE, 3D-GRE, and radial 3D-GRE sequences. Pearson correlation coefficients were 0.9788 for 2D-GRE vs.3D-GRE, 0.9506 for 3D-GRE vs.Radial 3D-GRE, and 0.9478 for 2D-GRE vs.Radial 3D-GRE (p<0.0001). Regression analyses confirmed strong agreement between methods. ICC was 0.9489 (95%CI=0.9279 to 0.9637).
**Conclusion:** Our findings underscore the promising potential of the new IP/OP free-breathing Radial 3D-GRE sequence as a reliable alternative to traditional breath-hold techniques for liver FF in patients who cannot hold their breath. The excellent correlation between Radial 3D-GRE and the cartesian methods should recommend its use for patients incapable of suspending breathing.
**Limitations:** Measurement values may be affected by variations in fat distribution within the liver and small sample size.These factors may impact accuracy and reproducibility and introduce selection bias. However, identical ROI and matched locations were used in all patients.
**Funding for this study:** No funding was received for this study

**Ethics committee - additional information:** Retrospective study
**Author Disclosures:**
Vasco Hérida: Nothing to disclose
Miguel Ramalho: Nothing to disclose
Tiago Castela: Nothing to disclose
Nuno Almeida: Nothing to disclose
Mariana Domingues Santana: Nothing to disclose
Richard C. Semelka: Nothing to disclose
Teresa Barata Rodrigues: Nothing to disclose

**Magnetic resonance elastography of the liver: are the results reliable?**
*V. Atamaniuk*, M. Obrzut, L. Hanczyk, M. Cholewa, B. Obrzut; Rzeszów/PL
*(vitaliyacera500@gmail.com)*

**Purpose or Learning Objective:** Magnetic resonance elastography (MRE) has become the gold standard for non-invasive assessment of liver stiffness, especially in patients with liver fibrosis, replacing liver biopsy. While guidelines for MRE protocols and interpretation have been standardized by the Quantitative Imaging Biomarkers Alliance (QIBA), the impact of vibration amplitude and slice thickness on hepatic stiffness measurements remains uncertain. This study aims to evaluate the reliability of MRE under varying vibration amplitudes and slice thicknesses.
**Methods or Background:** Twenty volunteers (10 men, 10 women), aged 18–68, underwent MRE on a 1.5 T whole-body scanner using a 2D GRE sequence. The Resoundant system delivered vibrations at 60 Hz with amplitudes of 25%, 50%, 75%, and 100%. Standard slice thickness was 10 mm, and an additional scan was performed at 75% amplitude with a 5 mm slice thickness. Regions of interest (ROIs) were manually drawn per QIBA guidelines to ensure consistent location for stiffness measurements across tested conditions. Statistical analyses included repeated measures ANOVA and intraclass correlation coefficients (ICC).
**Results or Findings:** The effects of both vibration amplitude ($p = 0.11$) and slice thickness ($p = 0.69$) on hepatic stiffness were not statistically significant. The ICC for different amplitudes was 0.92, and for slice thickness, it was 0.95, indicating excellent agreement across conditions.
**Conclusion:** MRE provides reliable liver stiffness measurements, with no significant influence from changes in vibration amplitude or slice thickness. Given the excellent agreement across varying imaging parameters, MRE can be considered a stable and reproducible method for assessing liver stiffness. Further multicentre studies with larger samples and 3D MRE may help confirm these findings and expand clinical applications of this technique.
**Limitations:** The study's limitations include the small sample size and the use of 2D MRE.
**Funding for this study:** No external funding was obtained for this study.
**Ethics committee - additional information:** Approved by the Regional Medical Chamber ethics committee (Resolution No 60/2022/B); informed consent was obtained from all participants.
**Author Disclosures:**
Marzanna Obrzut: Nothing to disclose
Marian Cholewa: Nothing to disclose
Lukasz Hanczyk: Nothing to disclose
Vitaliy Atamaniuk: Nothing to disclose
Bogdan Obrzut: Nothing to disclose

**Frequency and distribution of steatotic liver disease in the NAKO study – magnetic resonance imaging of 30,000 participants**
*M-N. Von Itter*[1], T. Nonnenmacher[2], E. Grune[1], J. Machann[3], J. Weiß[1], J. Nattenmüller[1], T. Norajitra[2], S. Rospleszcz[1], N. Consortium[1]; [1]Freiburg/DE, [2]Heidelberg/DE, [3]Tübingen/DE

**Purpose or Learning Objective:** Steatotic liver disease (SLD) and its subtype, metabolic dysfunction-associated steatotic liver disease (MASLD), are risk factors for cardiometabolic disease, liver cirrhosis, and hepatocellular carcinoma, and represent a major public health burden. Using magnetic resonance imaging in Germany's largest population-based study (NAKO), we describe the frequency of SLD and MASLD, and evaluate the distribution according to sex, age, BMI, geographic region, and socio-economic status.
**Methods or Background:** A validated nnU-Net liver segmentation model processed 29,842 MRI scans from NAKO participants (44.1% women) using T1-weighted 6-point Dixon data. The segmentation masks were used to calculate mean liver fat content (LFC). SLD was defined as LFC ≥ 5.56%, and MASLD according to established criteria.
**Results or Findings:** Overall frequency of SLD and MASLD was 37.6% and 31.8% in men, and 20.3% and 18.6% in women, respectively. Frequency increased with increasing BMI and age, with different patterns in men and women. Geographically, the highest frequency of SLD was found in Eastern Germany (40.6%). Frequency was higher in individuals with low socio-economic status, and this difference was more pronounced in women (frequency for high vs. low socio-economic status: 35.7% vs. 47.2% in men, and 17.1% vs. 37.9% in women).
**Conclusion:** Frequency of SLD and MASLD in Germany is high, with pronounced differences according to sex, age, BMI, geographic region, and socio-economic status. Our findings provide a robust basis to estimate the public health impact of these liver diseases in Germany.
**Limitations:** Ultrasound for diagnosis of SLD was not available.
**Funding for this study:** Funding was received from the German Research Foundation (Deutsche Forschungsgemeinschaft, grant number: 428224476). The NAKO is funded by the Federal Ministry of Education and Research (BMBF) [project funding reference numbers: 01ER1301A/B/C and 01ER1511D], the federal states and the Helmholtz Association, with additional financial support by the participating universities and the institutes of the Leibniz association.
**Ethics committee - additional information:** The study received institutional review board approval and written informed consent was obtained from all participants.
**Author Disclosures:**
Tobias Nonnenmacher: Nothing to disclose
Susanne Rospleszcz: Nothing to disclose
Johanna Nattenmüller: Nothing to disclose
Elena Grune: Nothing to disclose
Jakob Weiß: Nothing to disclose
Jürgen Machann: Nothing to disclose
Nako Consortium: Nothing to disclose
Marc-Nicolas Von Itter: Nothing to disclose
Tobias Norajitra: Nothing to disclose

**Assessment of using Multimodal Magnetic Resonance Imaging (MRI) for Noninvasive Evaluation of type 2 diabetic**
*M. W. Yang*, W. J. Shao; KunMing/CN
*(yangmengweid@163.com)*

**Purpose or Learning Objective:** To investigate the capability of intravoxel incoherent motion (IVIM), diffusion kurtosis imaging (DKI)and diffusion tensor imaging (DTI) to assess the renal function changes of type 2 diabetes.
**Methods or Background:** Prospectively included 46 patients diagnosed with T2MD, and divided them into three groups based on estimated glomerular filtration rate (eGFR) and the presence of diabetic nephropathy: the simple diabetes group (DM), the early diabetic nephropathy group (e-DKD), and the middle-to-late diabetic nephropathy group (m- DKD). At the same time, 33 volunteers（control group,CG） were recruited and underwent MRI examinations to collect images from various sequences. The true diffusion coefficient D, pseudo-diffusion coefficient D*, perfusion fraction f, mean diffusion kurtosis MK, mean diffusivity MD, fractional anisotropy FA, and apparent diffusion coefficient ADC of the renal cortex and medulla of the subjects were measured and statistically analyzed.
**Results or Findings:** In the e-DKD and m-DKD groups, ACR increased while eGFR decreased. The ACR of the e-DKD group significantly differed from the DM group and CG ($P<0.05$). IVIM values, DKI (cortical and medullary MD), and DTI (cortical and medullary ADC) values from all four groups showed a declining trend with disease progression, while cortical and medullary MK values from DKI showed an increasing trend. The cortical MK value of the DM group significantly differed from the CG ($P<0.05$). Medullary MK value effectively distinguished the CG and e-DKD groups (AUC=0.881, cutoff=0.593, sensitivity=76.9%, specificity=95.0%).
**Conclusion:** IVIM, DKI, DTI Sequences can be used to supplement renal dysfunction assessment. Different magnetic resonance parameters (IVIM, DKI, and DTI Sequences) identify different renal impairment changes in type 2 diabetic patients. Cortical MK value has higher diagnostic efficiency in the early detection of renal damage in DM patients.
**Limitations:** The sample size needs to be increased.Further multicenter studies are needed to supplement .
**Funding for this study:** The "SKY Imaging Research Fund" by the China International Medical Exchange Foundation.
**Ethics committee - additional information:** Chinese Ethics Review Number:2020143
**Author Disclosures:**
Wei Ju Shao: Nothing to disclose
Meng Wei Yang: Nothing to disclose

**Population-scale MRI body composition analysis: associations between single-slice and volumetric measurements of muscle and adipose tissue**
*M. Nowak*[1], L. M. Nunez[1], C. Hill[1], S. Marriage[1], R. Salvati[1], M. Pansini[2], H. B. Thomaides-Brears[1], M. Robson[1]; [1]Oxford/UK, [2]Lugano/CH

**Purpose or Learning Objective:** Accurate body composition tools are important for assessing adipose and muscle tissue in both clinical and research settings, including obesity management, sarcopenia, and weight loss trials. The use of whole-body MRI is limited by cost and processing demands, while single-slice MRI offers a more efficient alternative. This study evaluated the correlations between single-slice and volumetric assessments of visceral adipose tissue (VAT), subcutaneous adipose tissue (SAT), and muscle tissue, and their associations with cardiometabolic risk factors.
**Methods or Background:** We analyzed data from a subset of 67,509 individuals from the UK Biobank with water and fat MRI scans (mean age: 66, 51% male, BMI 26.7). A single axial slice at the L3 vertebra was used to measure VAT, SAT, and skeletal muscle via semi-automatic segmentations. These were compared with volumetric assessments of VAT, SAT, total lean tissue, and thigh fat-free muscle. Correlation coefficients were used to assess the relationship between the two methods, and their associations with cardiometabolic risk factors.
**Results or Findings:** Single-slice L3 measurements of SAT and VAT demonstrated very strong correlations with SAT (rho=0.94, p<0.001) and VAT volume (rho=0.97, p<0.001), independent of sex, age, BMI, waist circumference, diabetes status, and liver tissue characteristics (SAT: median rho 0.93, VAT: median rho 0.96, all p<0.001). Both body composition assessments showed similar correlations with cardiometabolic risk factors (all p<0.01 for HbA1c, triglycerides, high-density lipoprotein, systolic blood pressure, liver cT1, and liver fat content). Strong correlations were also observed between single-slice skeletal muscle and both total lean tissue (r=0.90, p<0.001) and thigh fat-free muscle volume (r=0.91, p<0.001).
**Conclusion:** Single-slice L3 measurements of VAT, SAT, and muscle CSA metrics show robust correlations with volumetric assessments across individuals with diverse cardiometabolic profiles, while exhibiting comparable associations with cardiometabolic risk factors.
**Limitations:** N/A
**Funding for this study:** Perspectum Ltd.
**Ethics committee - additional information:** In UK Biobank, ethical approval for data collection was received from the North-West Multi-centre Research Ethics Committee and the research was carried out in accordance with the Declaration of Helsinki of the World Medical Association.
**Author Disclosures:**
Luis Miguel Nunez: Nothing to disclose
Roberto Salvati: Nothing to disclose
Charles Hill: Nothing to disclose
Michele Pansini: Nothing to disclose
Magdalena Nowak: Nothing to disclose
Helena B Thomaides-Brears: Nothing to disclose
Matthew Robson: Nothing to disclose
Scott Marriage: Nothing to disclose

**13:00-14:30 Room G1**

### Research Presentation Session: Radiographers

### RPS 414

### AI-driven evolution: enhancing image quality, workflow, and professional identity for radiographers

**Moderators**
F. Doo; Baltimore, MD/US
*(fdoo@som.umaryland.edu)*
S. McFadden; Newtownabbey/UK
**Author Disclosures:**
Florence Doo: Equipment Support Recipients: Cloud credits from Amazon AWS, Microsoft Azure, Google Cloud; Grant Recipient: Funded in part by Association for Academic Radiology (AAR) Clinical Effectiveness in Radiology Research Academic Fund (CERRAF) in part by GE Healthcare; Research Grant/Support: Funded in part by the Johns Hopkins Mid-Atlantic Center for Cardiometabolic Health Equity (MACCHE), which is supported by National Institutes of Minority Health and Health Disparities (P50MD017438); Speaker: Honoraria from Eli Lilly

### R-AI-diographers: a European survey to explore the perceived impact of AI on professional identity, careers, and roles of radiographers

N. Stogiannos[1], *G. Walsh*[1], B. K. Ohene-Botwe[1], K. Mchugh[2], B. Potts[1], J. St John-Matthews[1], M. F. Mcentee[3], Y. Kyratsis[4], C. Malamateniou[1]; [1]London/UK, [2]Portsmouth/UK, [3]Cork/IE, [4]Rotterdam/NL
*(gemma.walsh@outlook.com)*

**Purpose or Learning Objective:** Artificial intelligence is changing radiographer clinical practice and roles. It is therefore vital to understand its impact on the careers, roles and professional identity of these professionals.
**Methods or Background:** A European-wide, EFRS-endorsed, cross-sectional, mixed methods online survey was designed on qualtrics. Snowball sampling was used to improve uptake. Survey questions explored radiographer perceptions for the short-term and long-term impact of AI implementation on their roles, responsibilities and professional identity. The study was translated in 8 languages. Responses were compared between different demographic groups including gender, age, education and country digital literacy level.
**Results or Findings:** 2206 valid responses were received from 37 different countries in Europe. 50.4% reported no AI education, and 26.6% were self-taught in AI. Over half (51.1%) thought patient-centered care skills will remain the same. 50.9% agreed radiographers will have more time to spend with patients thanks to AI. 57.8% agreed radiographers will have to work closer with other MIRT professionals in the future, for efficient AI implementation. Men appeared slightly more enthused about the development of technological skills and women about the honing of patient centered care skills, similar to previous studies. Radiographers were overall optimistic about the use of AI in healthcare, and optimism was higher in those countries with high digital literacy, better education levels and with more AI experience.
**Conclusion:** Radiographers were overall optimistic about the use of AI in healthcare and strongly believed that AI will advance patient-centred care. AI education currently lags for European radiographers, and this should be acutely addressed at the scale and pace required to keep up with current technological developments. Interprofessional collaboration was seen as essential for fostering mutual support among professionals.
**Limitations:** Snowball sampling can lead to selection-bias, but allows for many recruits.
**Funding for this study:** Funded by the College of Radiographers Industry Partnership Scheme (CoRIPS) [grant number: 2018].
**Ethics committee - additional information:** Ethics approval was obtained from City St George's, University of London School of Health and Psychological Sciences Ethics Committee (ETH2223-1346).
**Author Disclosures:**
Gemma Walsh: Nothing to disclose
Mark F. Mcentee: Nothing to disclose
Yiannis Kyratsis: Nothing to disclose
Benard Kwadwo Ohene-Botwe: Nothing to disclose
Nikolaos Stogiannos: Nothing to disclose
Janice St John-Matthews: Nothing to disclose
Ben Potts: Nothing to disclose
Christina Malamateniou: Nothing to disclose
Kevin Mchugh: Nothing to disclose

### An investigation into radiographers' perception of quality control auditing of radiographic practice and the potential role of Artificial Intelligence

*L. A. Rainford*, M. Mujaydia Alotaibi, J. Mcnulty, J. Potočnik; Dublin/IE
*(louise.rainford@ucd.ie)*

**Purpose or Learning Objective:** Quality assurance (QA) of radiographic technique is an essential part of radiation protection, traditionally performed through Reject Analysis. Digital imaging has increased the difficulty in completing radiographic technique auditing and staff shortages further compromise QA monitoring. This research aimed to seek radiography opinion on the use of Artificial Intelligence (AI) for QA.
**Methods or Background:** An online survey was developed (n=30 questions) to seek information related to QA monitoring of radiographic technique. Participant demographics, including area of employment and country of work, and professional and AI experience, were captured. Current QA auditing details were requested and participant confidence in these processes. Their opinion was requested on the potential challenges and benefits of AI use in QA monitoring. The survey was distributed to affiliate EFRS academic institutions to distribute to their clinical training sites and via Radiography social media.
**Results or Findings:** Good representation across all radiography professional grades was received from 125 participants (n=22 countries). 19.8% reported QA of radiographic images on at least a weekly basis, 18.8% stated monthly, whilst 60% reported it occurred far less frequently. 20% of responses stated staff were not individually reviewed. Only 26.8% were very confident in current QA processes, 48% were somewhat confident and the remainder not confident or unsure. 80% of participants indicated they perceived AI as having a role in QA, less than 10% demonstrated concern. Improved quality standards and skills were perceived as benefits however consideration of difficult patients was an identified challenge.
**Conclusion:** Poor confidence in current QA processes was identified and a lack of standardisation of practice. Radiographers identified AI as having the potential to support radiographic technique audits. Benefits and challenges were identified in open comments.
**Limitations:** Online survey: English language could have limited uptake
**Funding for this study:** Self funded
**Ethics committee - additional information:** University College Dublin, Human Research Ethics Committee – Sciences (HREC-LS) - LS-LR-24-141-Alotaibi-Rainford.

**Author Disclosures:**
Meshal Mujaydia Alotaibi: Nothing to disclose
Louise A. Rainford: Nothing to disclose
Jaka Potočnik: Nothing to disclose
Jonathan Mcnulty: Nothing to disclose

**A comparative study assessing the effectiveness of artificial intelligence and simulation education on reporting radiographer lung cancer detection**

E. Compton[1], S. Lightfoot[1], R. Shah[2], S. Ather[2], P. Taylor[1], *N. H. Woznitza*[1]; [1]London/UK, [2]Oxford/UK
*(nicholas.woznitza@nhs.net)*

**Purpose or Learning Objective:** Chest radiographs (CXRs) are a high-volume test, performed for a broad spectrum of reasons. Education has been shown to improve CXR reporting accuracy, in particular for less experienced reporters. Similarly, artificial intelligence (AI) as a clinical decision support tool provides novice readers with the most benefit. The aim of this study was to compare the impact of education (SIM) with AI in CXR reporting accuracy.
**Methods or Background:** A multi-reader, multi-case diagnostic accuracy study was conducted to determine the impact of SIM and AI on reporting radiographer (RR) CXR accuracy. 64 RR consented and completed bank 1 and were randomised stratified by years' experience (n=32,50% to AI). 43 RRs (24 AI, 19 education) completed both image banks (n=52 CXRs, 26 abnormal).
**Results or Findings:** Similar pre and post intervention accuracy was found. The AI cohort decreased sensitivity (74%-65%,p=0.015) but increased specificity (63%-77%,p<0.0001), the increase SIM sensitivity (69%-62%,p=0.115) and decrease in specificity (62%-68%,p=0.217) were not statistically significant. Standalone AI sensitivity and specificity were 54% and 77% respectively. For the AI arm, when the AI was correct specificity improved (67%-86%,p<0.001) with no significant difference in sensitivity (89%-86%,p=0.31), however when AI was incorrect there was a significant decrease in sensitivity (52%-32%,p<0.001) with no difference in specificity (both 57%,p=1). There were four CXRs that only had one (n=3) or three (n=1) pre-intervention correct decisions, suggesting the bank selected comprised of very challenging cases.
**Conclusion:** In a challenging CXR bank, both education and AI improved RR performance. As AI tools are adopted for CXR interpretation in clinical practice further work is required to ensure reporters are education in their use.
**Limitations:** The enhanced prevalence (50% abnormal) and single pathology (lung cancer) may limit transferability into clinical practice.
**Funding for this study:** This study was conducted as part of a clinical fellowship supported by NHS England (London).
**Ethics committee - additional information:** Canterbury Christ Church University ETH2223-0246 21st April 2023
**Author Disclosures:**
Paul Taylor: Nothing to disclose
Sam Lightfoot: Nothing to disclose
Emma Compton: Nothing to disclose
Ruchir Shah: Nothing to disclose
Sarim Ather: Founder: RAIQC
Nicholas Hans Woznitza: Consultant: InHealth Grant Recipient: SBRI Healthcare Employee: NHS London Consultant: SMR Health & Tech

**Radiographers' and students' perspectives on artificial intelligence - A cross-sectional online survey**

*M. R. V. Pedersen*[1], M. W. Kusk[2], S. Lysdahlgaard[2], H. Mork-Knudsen[3], C. Malamateniou[4], J. Jensen[5]; [1]Vejle/DK, [2]Esbjerg/DK, [3]Bergen/NO, [4]London/UK, [5]Odense/DK
*(Malene.Roland.Vils.Pedersen@rsyd.dk)*

**Purpose or Learning Objective:** The integration of artificial intelligence (AI) into radiography offers potential in enhancing workflow efficiency, image processing, patient positioning, and quality assurance.. This study aimed to investigate the perspectives and attitudes towards AI in radiography.
**Methods or Background:** An online survey including of 29 items was distributed via social media platforms to Nordic students and radiographers working in Denmark, Norway, Sweden, Iceland, Greenland, and the Faroe Islands. The survey included questions on demographics, specialization, educational background, place of work, and perspectives and knowledge on AI. The items were a mix of closed-type and scaled questions, with options for free-text responses when relevant.
**Results or Findings:** The survey received 586 responses from all Nordic countries. The mean age was 37.2 years with a standard deviation (SD) of ±12.1 years,. A total of 43% (n = 254) of the respondents had not received any AI training in clinical practice, while 13% (n = 76) had received AI training during their radiography undergraduate studies. Additionally, 77.9% (n = 412) expressed interest in pursuing AI education. The majority of respondents (82.8%, n = 485) were aware of the potential use of AI, and 39.1% (n = 204) had no reservations about AI.
**Conclusion:** Overall, radiographers have a positive attitude towards AI. However, there has been very limited training or education provided to radiographers, despite 82.8% reporting plans to implement AI in clinical practice. Generally, awareness of AI applications is high
**Limitations:** Limitations include language barriers as this survey was provided in English. Most Nordic radiographers speak, read, and write English very well. Yet, when it come to complex sentences in English there is a higher risk of skipping items, survey drop out, language misunderstanding or misinterpretation.
**Funding for this study:** No funding
**Ethics committee - additional information:** The study was approved by the Research Ethics Committee at the University of Southern Denmark (ID: 22-58485)
**Author Disclosures:**
Helene Mork-Knudsen: Nothing to disclose
Martin Weber Kusk: Nothing to disclose
Malene Roland Vils Pedersen: Nothing to disclose
Janni Jensen: Nothing to disclose
Simon Lysdahlgaard: Nothing to disclose
Christina Malamateniou: Nothing to disclose

**An analysis of the user interface preferences of imaging professionals for AI to support clinically relevant decision making**

*A. Gill*, S. L. Mcfadden, C. Rainey, L. Mclaughlin, J. Mcconnell, C. Hughes, R. Bond; Belfast/UK
*(avgill16@gmail.com)*

**Purpose or Learning Objective:** This study investigates the cognitive behaviour of imaging staff when interacting with Explanation User Interfaces (EUI). Data was gathered on user preferences of chest radiograph Artificial Intelligence (AI)-based EUIs.
**Methods or Background:** Human and machine interaction involves the EUI that clinicians use to link medical diagnosis or report. However, there is currently a lack of EUI standardisation within this field (Schalekamp et al, 2022). Building on an international questionnaire undertaken at ECR 2024, a multi-methods study was undertaken incorporating eye-tracking, Think-Aloud and a questionnaire at UKIO 2024. Diagnostic radiographers', radiologists', trainee radiologists' and student radiographers' identified visual preferences when reviewing four different types of chest radiograph AI EUIs i.e. 1) salience maps, 2) textual reports, 3) area of interest and 4)abnormality score EUIs. Participants reviewed the images whilst wearing eye-tracking software and voiced their thought processes i.e. the "Think-Aloud" method. The post study questionnaire asked the participants about their perceived level of confidence against the four different interfaces.
**Results or Findings:** 24 participants enabled understanding of which components of the chest radiograph EUI are focused on and subsequently preferred. Eye-tracking data relating to fixations and saccades statistically described patterns where maximal attention was directed in the interpretation process. Think-Aloud and post-study questionnaire data added further insights to participant EUI preferences. The analysis of the eye-tracking study remains ongoing, and completion is aimed for January 2025.
**Conclusion:** Understanding user preference for chest radiograph AI EUI supports appropriate user engagement with the information provided by the technology. This gives radiographers and radiologists the ability to explain this further to patients, positively impacting their understanding and subsequent care.
**Limitations:** Small sample size may have affected the wider generalisability of findings. Eye-tracking software capabilities
**Funding for this study:** PhD funded by Department for the Economy
**Ethics committee - additional information:** FCNUR-23-084 reference
**Author Disclosures:**
Jonathan Mcconnell: Nothing to disclose
Laura Mclaughlin: Nothing to disclose
Clare Rainey: Nothing to disclose
Ciara Hughes: Nothing to disclose
Raymond Bond: Nothing to disclose
Sonyia Lorraine Mcfadden: Nothing to disclose
Avneet Gill: Nothing to disclose

**Systematic review on advanced image post-processing and workflow optimization in cardiovascular MRI**

*V. Tambè*, M. Zanardo, C. Torrito, P. Della Cagnoletta, F. Secchi; Milan/IT
*(valentina.tambe@gmail.com)*

**Purpose or Learning Objective:** Cardiovascular magnetic resonance imaging (CMR) is a critical tool for diagnosing heart disease, but is hindered by long acquisition times and manual post-processing. This systematic review examines recent advancements in image post-processing and workflow optimization, focusing on the integration of artificial intelligence (AI).

**Methods or Background:** A systematic search was conducted using PubMed and EMBASE. Included studies involved the use of AI-based methods to optimize CMR workflows and post-processing. Eligible articles were those addressing any of the following: image reconstruction, segmentation, workflow automation, and clinical integration of AI tools. Studies without quantitative outcomes related to workflow efficiency or post-processing improvements were excluded.
**Results or Findings:** Out of 151 articles screened, 33 studies were included. Key findings included: automated segmentation reported in 15/33 (45%) studies; image reconstruction in 10/33 (30%); workflow automation 8/33 (24%); clinical efficiency in 7/33 (21%); quality control in 5/33 (15%). In the automated segmentation articles, results showed improvements in segmentation speed and accuracy with Dice similarity coefficients exceeding 0.90 in many studies, and reducing manual post-processing time by up to 66%. Other studies focused on reducing scan times and enhancing image quality, with AI-based methods reducing scan times by up to 40% while maintaining image quality. Articles showed significant improvements in reporting times (by up to 30%), while 5 articles presented data on AI-based quality control reducing rescans (by up to 20%).
**Conclusion:** AI integration into CMR has significantly improved workflow efficiency, reducing acquisition times and enhancing diagnostic accuracy. Automated segmentation, image reconstruction, and workflow automation have accelerated processes, reduced operator dependency, and minimized rescans.
**Limitations:** Further large-scale validation is needed to fully implement AI in CMR across diverse clinical settings.
**Funding for this study:** None
**Ethics committee - additional information:** Not Applicable
**Author Disclosures:**
Francesco Secchi: Nothing to disclose
Moreno Zanardo: Nothing to disclose
Valentina Tambè: Nothing to disclose
Paolo Della Cagnoletta: Nothing to disclose
Carmelo Torrito: Nothing to disclose

### Blended intensive program for innovative technologies and deep learning models (AI) in the radiographer's working environment

*C. Schneckenleitner*, C. Kamp, C. Vogl, A. Raith, G. Guevara; Vienna/AT
*(christian.schneckenleitner@fh-campuswien.ac.at)*

**Purpose or Learning Objective:** The working environment of radiographers is characterized by permanent technological innovations. We developed an international intensive program containing theory and hands-on training to introduce bachelor and masters' students to future technologies. This program is designed to prepare students for innovative technologies and expand their professional skills in areas such as deep learning models, Computer Assisted Surgery Simulation, 3D Printing, Optical Scanning and visualization utilizing mixed reality.
**Methods or Background:** During a supervised online phase, students learned how to create patient-specific 3D-printed models, CT-data segmentation for deep learning training with 3D-Slicer, computer-assisted surgical planning (CAS), how to acquire optical 3D-scans and mixed reality visualization of the respective 3D-models they created. All this content was worked on by the students in a follow-up international skills lab week at the University of Applied Sciences Vienna. Each skills lab block included 6h hours of hands-on training. The students generated deep learning models with the platform MONAI, created surgical plans with Materialise Mimics, scanned with optical 3D-scanners from ARTEC and created mixed reality visualizations.
**Results or Findings:** The results were uploaded to an online platform (Moodle) by the students and analyzed based on defined criteria. Results, 39 out of 42 students were able to create a segmentation for deep learning training according to the required criteria. 39 out of 42 successfully performed a CAS plan, 20 created a 3D-printable patient specific model and 42 of 42 were able to produce an optical 3D-scan of the face.
**Conclusion:** The results show that radiography students can produce results in adjacent technology areas and expand their competence in future technologies.
**Limitations:** The limited time can generate interest but not a specialization in the profession. Explicit training programs are needed to deepen radiographers' expertise in these areas.
**Funding for this study:** No funding.
**Ethics committee - additional information:** The results includes no patient specific information or clinical interventions.
**Author Disclosures:**
Christoph Kamp: Nothing to disclose
Christian Schneckenleitner: Nothing to disclose
Alexander Raith: Nothing to disclose
Godoberto Guevara: Nothing to disclose
Christoph Vogl: Nothing to disclose

### Evaluation of ChatGPT as support in image qualitative assessment for cardiac sonographers

*K. Tissir*[1], G. R. Bonfitto[1], A. Roletto[1], A. Signoroni[2]; [1]Milan/IT, [2]Brescia/IT
*(karimatissir@hotmail.it)*

**Purpose or Learning Objective:** The integration of Large Language Models (LLM) tools like ChatGPT in clinical settings is changing how healthcare professionals manage diagnoses and workflow. In cardiac clinics, the accurate and timely interpretation of images is crucial for effective diagnosis and monitoring of cardiac conditions. ChatGPT could be particularly beneficial for cardiac sonographers. This study aims to assess whether ChatGPT can effectively support cardiac sonographers in the qualitative evaluation of echocardiographic images.
**Methods or Background:** A database of 50 anonymized echocardiographic images was retrospectively analyzed, including 2-chamber, 4-chamber, and apical 3-chamber views. Three evaluators, a junior sonographer, a senior sonographer and ChatGPT-4o conducted the qualitative evaluation of the images by identifying scoring them on a 5-point Likert scale. The guidelines of the European Association of Echocardiography served as references.
**Results or Findings:** Junior sonographer correctly identified views in 84% of cases (n=42), while ChatGPT correctly identified 58% of cases (n=29).In comparison to senior sonographer, the junior sonographer overestimated 22% of the images (n=11), underestimated 36% (n=18), and agreed in 42% of the images (n=21). In contrast, ChatGPT overestimated 52% of the images (n=26), underestimated 18% (n=9), and agreed in 30% of the images (n=15).
**Conclusion:** ChatGPT-4o shows limitations in identifying echo cardiac views compared to other participants. In addition, ChatGPT is inclined to overestimate image quality. This can be explained by limited training of the LLM, mainly done with information from guidelines. As other studies in literature showed, more in-depth training could increase the performance of ChatGPT. LLM can assist cardiac sonographers in qualitative analysis of images and supporting anomaly evaluation, but concerns remain about its reliability and bias.
**Limitations:** The small sample of participants and cases limited the strength of the conclusions of this study.
**Funding for this study:** N/A
**Ethics committee - additional information:** University of Brescia
**Author Disclosures:**
Andrea Roletto: Nothing to disclose
Karima Tissir: Nothing to disclose
Alberto Signoroni: Nothing to disclose
Giuseppe Roberto Bonfitto: Nothing to disclose

### Navigating Artificial Intelligence (AI) Leadership: Radiographers' Readiness and Challenges in Europe

*G. Walsh*[1], Y. Kyratsis[2], A. Goodall[1], J. St John-Matthews[1], C. Malamateniou[1]; [1]London/UK, [2]Rotterdam/NL
*(gemma.walsh@outlook.com)*

**Purpose or Learning Objective:** This study offers unique insights into the preparedness of radiographers to pursue AI leadership roles within healthcare and potential barriers preventing radiographers excelling in the AI ecosystem.
**Methods or Background:** A European-wide, cross-sectional study utilising a mixed methods online survey. Snowball sampling allowed qualified radiographers, and radiography students, to answer the survey, irrespective of their current role. The survey explored the following areas of interest: a) general radiographer demographics, b) radiographers preparedness and confidence to lead the implementation of AI in healthcare, c) suggested day-to-day responsibilities of an AI-lead radiographer and d) motivations for considering AI leadership roles.
**Results or Findings:** There were 1733 valid responses from 37 European countries. The typical respondent was female (64%), a diagnostic radiographer (59.9%) with >20 years' experience (31.3%). A lack of education, training and resources led 72.3% of radiographers to feel the profession is not prepared to lead the implementation of AI in healthcare. Those that felt confident to lead in an AI-enabled work environment (50.06%), felt they already have the necessary experience and skills, but also admitted additional resources would be needed. The top two motivators to pursue an AI leadership role included championing change and the promise of appropriate training. Inferential Statistics ongoing as of October 2024.
**Conclusion:** Radiographers have a unique skill-set making them the ideal candidates for AI leadership roles within healthcare. Radiographers do not currently feel confident or prepared to undertake AI leadership roles with education, training and a lack of resources creating barriers for this. It is reassuring radiographers feel motivated to undertake AI leadership roles, however increased training and educational support are needed.
**Limitations:** Gives a snapshot view of radiographers perceptions.
Snowball sampling can lead to selection-bias, but allows for many recruits.
**Funding for this study:** This research has been funded by the AI special call of the College of Radiographers Industry Partnership Scheme (CORIPS) of the College of Radiographers (Reference Number 218).

**Ethics committee - additional information:** Ethics approval (ETH2223-1346) was granted by City, University of London.
**Author Disclosures:**
Gemma Walsh: Nothing to disclose
Yiannis Kyratsis Yiannis Kyratsis: Nothing to disclose
Janice St John-Matthews: Nothing to disclose
Amanda Goodall: Nothing to disclose
Christina Malamateniou: Nothing to disclose

**MRI deep learning models for assisted diagnosis of knee pathologies and injuries: A systematic review**
*K. M. Mead*, T. Cross, G. Roger, R. Sabharwal, S. Singh, N. Giannotti; Sydney/AU
*(keileymead13@gmail.com)*

**Purpose or Learning Objective:** Several studies have demonstrated that deep learning (DL) models can be effectively trained on MRI data to assist clinicians in identifying knee injuries and pathologies. This systematic review was conducted to explore the current landscape of existing DL models developed for detecting knee injuries and pathologies through magnetic resonance imaging (MRI) and to assess their potential clinical applications.
**Methods or Background:** Five databases were systematically searched using the following terms 'Knee AND 3D AND MRI AND Deep Learning'. The Covidence platform was used to screen publications based on title, abstract, and full text. Only original research articles focussing on the automatic detection of knee injuries and pathologies using DL models for MRI were included. The synthesis of results was performed by two independent reviewers.
**Results or Findings:** Fifty-four studies were included. The studies focused on anterior cruciate ligament injuries (n=19), osteoarthritis (n=9), meniscal injuries (n=13), general abnormal knee appearance (n=10), tibial plateau fractures (n=1) and synovial fluid detection (n=1). The following convolutional neural network (CNN) infrastructures were used: ResNet, VGG, DenseNet, and DarkNet. The averaged performance outcomes of the DL models demonstrated sensitivity, specificity, AUC-ROC, and accuracy of 87%, 90%, 92%, and 88%, respectively. The DL models for the detection of a specific injury or pathology outperformed those for general abnormality detection.
**Conclusion:** This systematic review underscores that fine-tuned DL models for knee pathologies and injuries can effectively support automatic diagnosis. Further large-scale validation and prospective studies are needed to confirm their clinical utility as assistive diagnostic tools.
**Limitations:** Inconsistent data reporting across the studies analysed resulted in variations in the reporting of DL model performance. Sub-group analyses were performed to minimise bias.
**Funding for this study:** No funding was received for this study.
**Ethics committee - additional information:** The study is retrospective. Ethics approval was deemed unnecessary by the Research Integrity and Ethics Committee at the University of Sydney, Australia.
**Author Disclosures:**
Keiley Michelle Mead: Nothing to disclose
Nicola Giannotti: Nothing to disclose
Sahaj Singh: Nothing to disclose
Tom Cross: Nothing to disclose
Rohan Sabharwal: Nothing to disclose
Greg Roger: Nothing to disclose

**An Innovative AI-Based Interactive Tool for Learning Chest X-Ray Anatomy**
*R. S. T. Ribeiro*, T. Coutaudier, L. Mourot, C. S. D. Reis, L. Raileanu; Lausanne/CH
*(ricardo.ribeiro@hesav.ch)*

**Purpose or Learning Objective:** To enhance the learning process of chest X-ray/(CXR) anatomy for medical imaging/(MI) students by integrating AI segmentation and classification tools into an educational web-application/(webapp).
**Methods or Background:** The webapp was designed as an interactive platform where students can identify CXR anatomy. The platform was implemented in Python using Flask to provide the web-interface, TorchXRayVision to classify and segment key regions (heart, lungs, clavicles, spine, scapula, trachea) with AI. PostgreSQL stored and managedCXR public datasets allowing students' practice. The user interface allows selection and outline regions of interest on the radiographs, that are compared to segmentations obtained with AI-algorithms. Feedback is provided through Dice coefficient that assess the accuracy of the user's segmentation compared to the AI-based reference. The app's system architecture is modular.
**Results or Findings:** The developed framework successfully integrates AI for fast and accurate segmentation of CXR. Its design allows users to upload their own radiographs or use others supplied by public datasets, interact with radiographs by selecting anatomical regions and receiving immediate feedback. This functionality aims to support autonomous learning and reduce the need for constant instructor supervision. The modular architecture ensures scalability, enabling the inclusion of more types of radiographic images and enhancing its potential for broader applications in medical imaging education.
**Conclusion:** The web application demonstrates a promising approach to improve MI education by providing an interactive and AI-powered learning tool. Its design is intended to be adaptable/accessible through any web-browser, with potential to expand into areas such as quality assessment. Further development of this framework is planned to test its impact on the MI students learning process.
**Limitations:** The app is limited to CXR and relies on AI segmentation performance.
**Funding for this study:** Not applicable
**Ethics committee - additional information:** Not applicable
**Author Disclosures:**
Claudia Sa Dos Reis: Nothing to disclose
Ricardo Silva Teresa Ribeiro: Nothing to disclose
Lucas Mourot: Nothing to disclose
Théo Coutaudier: Nothing to disclose
Laura Raileanu: Nothing to disclose

**15:00-16:00** **Research Stage 1**

### Research Presentation Session: Breast

### RPS 502

### MRI and contrast-enhanced mammography for treatment planning

**Moderator**
M. Dorrius; Groningen/NL
*(m.d.dorrius@umcg.nl)*
**Author Disclosures:**
Monique Dorrius: Research Grant/Support: KWF PPS Grant

**Radiomic Shape Features for Assessment of Early Therapy Response to Neoadjuvant Chemotherapy of Breast Cancer Patients - Preliminary Results of the PREDICTOME-Study**
*D. A. Resch*, O. Lafcı, P. Clauser, Z. Bago-Horvath, Y. Tan, G. Langs, T. Helbich; Vienna/AT
*(daphne.resch@gmail.com)*

**Purpose or Learning Objective:** To analyze the significance of radiomic shape features derived from dynamic contrast-enhanced (DCE) breast MRI in assessing early treatment response in breast cancer patients undergoing neoadjuvant chemotherapy (NAC).
**Methods or Background:** We report on the first 29 breast cancer patients of a prospective study, who underwent NAC and received multiparametric 18F-FDG PET/MRI imaging at baseline (T0) and after three weeks of NAC (T1). DCE-MRI derived radiomic shape features, including SHAPE_Volume (mL), SHAPE_Volume (vx), SHAPE_Sphericity, SHAPE_Surface($mm^2$) and SHAPE_Compacity were extracted using LIFEx. The agnostic shape features were compared to the treatment response assessment by two radiologists. All data were stratified by the pathological complete response status (pCR or non-pCR). Mean change (Δ) of all investigated features were calculated. Pearson Chi-Square Test, T-Test and Mann-Whitney U Test were applied.
**Results or Findings:** Twelve out of 29 (41%) patients had pCR and 17/29 (59%) had non-pCR. Radiomic shape features, including ΔSHAPE_Compacity and SHAPE_Volume at T1, were significantly associated with pCR (P = 0.015 and P = 0.04, respectively). Radiologist's response assessment (stable disease, partial response, disease progression) was not significantly associated with pathological outcomes (pCR vs. non-pCR) (Pearson Chi-Square: $\chi2$=3.727$\chi2$=3.727, P = 0.155). Similarly, the likelihood ratio test did not show significant results (P = 0.105).
**Conclusion:** While the radiologist's assessment did not significantly correlate with pathological outcomes, radiomic shape features, particularly ΔSHAPE_Compacity and SHAPE_Volume, demonstrated significant associations with pCR. These findings suggest that DCE derived radiomic shape features might be a promising tool for prediction of early NAC response in breast cancer patients and may challenge the validity of RECIST criteria at this stage.
**Limitations:** This study is limited by the small sample size. We will be able to report on more data in March 2025.
**Funding for this study:** This study is supported by the WWTF (Vienna Science and Technology Fund), grant number LS20-065.

**Ethics committee - additional information:** EK Nr. 510/2009
**Author Disclosures:**
Georg Langs: Nothing to disclose
Daphne Ariane Resch: Nothing to disclose
Thomas H. Helbich: Nothing to disclose
Oğuz Lafcı: Nothing to disclose
Zsuzsanna Bago-Horvath: Nothing to disclose
Yen Tan: Nothing to disclose
Paola Clauser: Nothing to disclose

**Reactive Cutaneous Capillary Endothelial Proliferation in Breast Tissue on MRI during Neoadjuvant Chemoimmunotherapy with Camrelizumab in TNBC**

*X. Ma*, Q. Xiao, Y. Huang, Y. Gu; Shanghai/CN
*(maxiaowen9397@163.com)*

**Purpose or Learning Objective:** To describe the Reactive cutaneous capillary endothelial proliferation (RCCEP) that occurs within the breast tissue of triple-negative breast cancer (TNBC) patients undergoing neoadjuvant chemoimmunotherapy with camrelizumab and to investigate the potential factors influencing its occurrence.
**Methods or Background:** We retrospectively collected 106 cases of TNBC patients from March 2021 to August 2023, including 60 cases who received neoadjuvant chemotherapy (NAC) and 46 cases who underwent neoadjuvant chemoimmunotherapy (NACI). We analyzed the clinical data, pathological characteristics, MRI at baseline and during each treatment cycle for all patients, then identified abnormal lesions after treatment and explored their influencing factors.
**Results or Findings:** Abnormal enhancement was observed in 30 patients (3 NAC vs. 27 NACI, P<0.001). In the NACI group, cases of abnormal enhancement included 3 cases of ring enhancement, 2 cases of linear enhancement, and 22 cases of mass enhancement, which typically appeared after the second cycle and rarely appeared after the fourth or sixth cycle. These lesions generally decreased in size with continued treatment. In the NACI group, the development of new enhancement lesions was correlated with younger age (P=0.007), premenopausal status (P=0.014), a lack of peritumoral edema on baseline MRI (P=0.007), and the presence of mass enhancements (P=0.012).
**Conclusion:** TNBC patients treated with camrelizumab frequently exhibit RCCEP in the breast tissue, primarily presenting as mass enhancements on MRI. These lesions often regress in size even without drug withdrawal, suggesting that recognizing this pattern can prevent unnecessary biopsies and help in adjusting treatment strategies accordingly.
**Limitations:** First, this study is retrospective and includes a small sample size; second, in future studies, it is necessary to extend the research period to explore the relationship between RCCEP in the breast and their prognosis.
**Funding for this study:** None
**Ethics committee - additional information:** Fudan University Shanghai Cancer Center
**Author Disclosures:**
Yan Huang: Nothing to disclose
Yajia Gu: Nothing to disclose
Qin Xiao: Nothing to disclose
Xiaowen Ma: Nothing to disclose

**Patient Perspective: Evaluating Imaging Preferences in Women Undergoing Contrast-Enhanced Breast MRI and Contrast-Enhanced Mammography (CEM)**

N. Caldarelli, *G. Della Pepa*, G. Irmici, E. D'Ascoli, C. De Berardinis, D. Ballerini, A. Bonanomi, C. Depretto, G. P. Scaperrotta; Milan/IT
*(gianmarco.dellapepa@gmail.com)*

**Purpose or Learning Objective:** The study evaluated patient preferences between contrast-enhanced MRI (MRI) and contrast-enhanced mammography (CEM) for breast cancer imaging. MRI has long been the gold standard for breast cancer staging and monitoring neoadjuvant chemotherapy, while CEM has gained popularity due to its lower cost, faster examination times, and accessibility, offering comparable diagnostic performance.
**Methods or Background:** The aim of this study was to understand patient preferences between these two modalities to support the broader implementation of CEM in clinical practice. The study included 152 patients who underwent both procedures within a six-month interval between 2018 and 2024. A Likert scale questionnaire was used to assess patient preferences focusing on three main aspects: breast positioning (compression for CEM and coil positioning for MRI), sensation during contrast injection, and overall comfort (exam duration, machine noise, and environmental factors).
**Results or Findings:** Results showed that 72.4% of patients preferred CEM, 26.3% preferred MRI, and 1.3% expressed no preference. CEM was significantly more comfortable (p<0.001), with higher median scores than MRI. The main reasons for preferring CEM included faster exam time (28%), lack of claustrophobia (17%), and absence of noise (15%). In terms of breast positioning, there was a slight preference for MRI (p=0.04). No significant differences were found in the sensation during contrast injection (p=0.07).
**Conclusion:** In conclusion, CEM was the preferred option for most patients, indicating its potential as an alternative to MRI in clinical settings. These findings support further exploration of CEM's role in breast cancer imaging.
**Limitations:** none. We have no limitations.
**Funding for this study:** none. We don't need any funding for this study.
**Ethics committee - additional information:** none. We don't have any Ethics committee.
**Author Disclosures:**
Nazarena Caldarelli: Nothing to disclose
Gianmarco Della Pepa: Nothing to disclose
Elisa D'Ascoli: Nothing to disclose
Alice Bonanomi: Nothing to disclose
Daniela Ballerini: Nothing to disclose
Gianfranco Paride Scaperrotta: Nothing to disclose
Claudia De Berardinis: Nothing to disclose
Catherine Depretto: Nothing to disclose
Giovanni Irmici: Nothing to disclose

**Non-invasive imaging of the tumor pH in breast cancer with CEST-MRI: A preclinical study**

*D. Prinz*[1], S. J. Bartsch[1], J. Friske[1], D. Laimer-Gruber[1], T. H. Helbich[1], K. Pinker-Domenig[2]; [1]Vienna/AT, [2]New York, NY/US
*(daniela.a.prinz@meduniwien.ac.at)*

**Purpose or Learning Objective:** Tumor acidosis is a key hallmark of breast cancer (BC). The increased glucose consumption triggers aerobic glycolysis, leading to the production of lactic acid which results in therapy resistance. Currently, there is no non-invasive tool available to image tumor pH in vivo. We attempted to image the extracellular pH (pHe) with acidoCEST using Iopamidol, and the intracellular pH (pHi) using the CEST-derived AACID (amine and amide concentration-independent detection) metric. Non-invasive imaging of tumor pH is of great interest because pH is one of the first biomarkers which changes during treatment.
**Methods or Background:** Female athymic nude mice were inoculated with BC cells of HER2+ (SKBR-3, n = 5) and triple-negative (MDA-MB-231, n = 4) molecular subtypes. MRI imaging was performed using a preclinical 9.4T MRI system. CEST images were acquired and ratiometric measurements were evaluated for the endogenous AACID from baseline images and the acidoCEST signal based on post-challenge images.
**Results or Findings:** Both acidoCEST and AACID produced reliable and stable signals. The lower pHe and the higher pHi could be visualized for HER2+ and triple-negative BC subtypes. Parametric maps of AACID and acidoCEST revealed differences in the pH gradients between BC subtypes, which correlated with tumor aggressiveness.
**Conclusion:** We conclude that the AACID-based measurement of pHi sufficiently quantifies the pH gradient between extracellular and intracellular compartments and may become a promising non-invasive contrast-free imaging method to monitor early treatment response in BC.
**Limitations:** Due to the preclinical nature of this study, the small number of mice used presents a limitation.
**Funding for this study:** This work was funded by the Vienna Science and Technology Fund (WWTF), grant no. LS19-018.
**Ethics committee - additional information:** This animal study was approved by Austrian Federal Ministry of Education, Science and Research [66.009/0284-WF/V/3b/2017; 2020-0.363.124; 2022-0.726.820] and the Intramural Committee for Animal Experimentation of the Medical University of Vienna.
**Author Disclosures:**
Silvester Julian Bartsch: Nothing to disclose
Daniela Laimer-Gruber: Nothing to disclose
Katja Pinker-Domenig: Nothing to disclose
Thomas H. Helbich: Nothing to disclose
Daniela Prinz: Nothing to disclose
Joachim Friske: Nothing to disclose

**CEM Background Parenchymal Enhancement: Exploring Its Clinical and Biological Correlations**

*C. De Berardinis*[1], C. Depretto[1], G. Della Pepa[1], E. D'Ascoli[1], G. Irmici[1], E. Ancona[1], R. Spiaggia[2], L. Corradini[2], G. P. Scaperrotta[1]; [1]Milan/IT, [2]Mussomeli/IT
*(cla.deberardinis@gmail.com)*

**Purpose or Learning Objective:** To assess the correlation between the degree of background parenchymal enhancement (BPE) on contrast-enhanced mammography (CEM) and breast density, menopausal state, receptor status, HER 2 expression and proliferation index (Ki-67) of malignant tumors.

**Methods or Background:** We retrospectively evaluated all patients who underwent CEM at our Institution from January 2023 to April 2024 for pre-operative staging and problem solving. BPE was classified as minimal, mild, moderate, or marked, in accordance with the BIRADS lexicon. We assessed the menopausal status and evaluated the receptor status and Ki-67 of each malignant lesion. Statistical analysis was performed using the Spearman's test to evaluate the correlation between density and BPE. Chi-square test was used to evaluate the correlation between BPE, menopausal status, receptor status, and Ki-67.
**Results or Findings:** A total of 194 patients were included. Spearman's test analysis demonstrated a weak correlation between BPE and breast density ($\rho$=0.353, p<0.001, CI:0.219-0.469). The Chi-Square test revealed a strong association between BPE and menopausal state, with lower BPE levels in postmenopausal patients compared to premenopausal patients ($X^2$=30.846, p<0.001). There was no statistically significant association between BPE and receptor status ($X^2$=14.494, p=0.270) and HER2+ status ($X^2$=1.648, p=0.649). There was suggestive but not statistically significant association ($X^2$=6.738, p=0.081) between BPE and Ki67.
**Conclusion:** A significant correlation was demonstrated between the level of BPE on CEM and menopausal status. Instead, the correlation with breast density proved to be weak, while no statistically significant correlation was found with tumor receptor status. BPE also appears to have a suggestive correlation with Ki-67% and consequently with tumor aggressiveness.
**Limitations:** This association needs more data and larger sample size to be confirmed but suggests how BPE might be related to breast cancer risk.
**Funding for this study:** None
**Ethics committee - additional information:** None
**Author Disclosures:**
Gianmarco Della Pepa: Nothing to disclose
Elisa D'Ascoli: Nothing to disclose
Claudia De Berardinis: Nothing to disclose
Eleonora Ancona: Nothing to disclose
Gianfranco Paride Scaperrotta: Nothing to disclose
Catherine Depretto: Nothing to disclose
Lisa Corradini: Nothing to disclose
Rossana Spiaggia: Nothing to disclose
Giovanni Irmici: Nothing to disclose

**Role of Breast MRI to identify patients with lesions of uncertain malignant potential (B3) who might avoid surgery: a systematic review and metanalysis**
*G. Vatteroni*[1], N. Pötsch[2], P. Clauser[2], P. A. Baltzer[2]; [1]Milan/IT, [2]Vienna/AT
*(giulia.vatteroni@gmail.com)*

**Purpose or Learning Objective:** This systematic review and meta-analysis investigates the added value of Contrast Enhanced Breast MRI (CE-MRI) to rule out malignancy in patients with lesions of uncertain malignant potential (B3) diagnosed at image guided biopsy.
**Methods or Background:** A systematic review and meta-analysis were conducted using predefined criteria. Eligible articles, published in English until August 2024, dealt with CE-MRI in the context of B3 lesions. Two reviewers extracted study characteristics, including true positives (TP), false positives (FP), true negatives (TN), and false negatives (FN). Sensitivity, specificity, negative likelihood ratio, and positive likelihood ratio were calculated using bivariate random effects. Fagan nomograms identified the maximum pretest probability at which post-test probabilities of a negative MRI aligned with the 2% malignancy rate benchmark for downgrading BI-RADS 4 to BI-RADS 3. $I^2$ statistics and meta-regression explored sources of heterogeneity. P-values <0.05 were considered significant.
**Results or Findings:** Seven studies investigating 479 patients with 493 high risk lesions undergoing CE-MRI were included. The average breast cancer prevalence was 17% (88/493). Pooled sensitivity was 91.3% (95%-CI: 82.8%-95.8%) and pooled specificity was 68.8% (95%-CI 50.3%-82.8%) using a random effects model. Overall, CE-MRI missed only 6/493 lesions, all small low-grade Ductal Carcinoma in Situ. Fagan nomograms showed that CE-MRI could rule out malignancy in B3 lesions diagnosed at image guided biopsy up to a pre-test probability of 13.1%.
**Conclusion:** CE-MRI in the assessment of B3 lesions could potentially identify patients who might avoid surgery, saving costs and time as well as reducing patient anxiety and morbidity. Breast cancer can be ruled out up to pre-test probabilities of 13.1%.
**Limitations:** n/a
**Funding for this study:** None
**Ethics committee - additional information:** Not applicable
**Author Disclosures:**
Pascal A.T. Baltzer: Nothing to disclose
Nina Pötsch: Nothing to disclose
Giulia Vatteroni: Nothing to disclose
Paola Clauser: Nothing to disclose

**Contrast-Enhanced Digital Mammography for the Diagnosis and Determination of Extent of Disease in Invasive Lobular Carcinoma: Our Experience and Literature Review**
*J. Lipin Margaret*, T. Friehmann, S. Tamir, G. Bachar, A. Grubstein; Petach Tikva/IL
*(margaret.lipinski@gmail.com)*

**Purpose or Learning Objective:** In contrast-enhanced imaging for the precise evaluation of Invasive lobular carcinoma (ILC), the aim was to further validate the use of CEM for evaluation of extent of disease in ILC cases, with Digital breast tomosynthesis (DBT) as an adjunct.
**Methods or Background:** A retrospective study was conducted in a tertiary medical center. All CEM examinations of ILC patients between 2017–2023 were re-read by two breast radiologists. The longest lesion diameter was correlated to pathology findings. For each case, readers provided commentary on the added value of DBT.
**Results or Findings:** Twenty-four CEM examinations were evaluated. The cohort were on average older than expected for ILC patients (74 vs. 63 years) and were unable to undergo MRI due to advanced age and comorbidities. Enhancing lesions, ranging from 4–75 mm, strongly correlated to pathology results, with statistical significance, for mass and non-mass lesions (r = 0.94, p < 0.001 and r = 0.99, p = 0.002, respectively). For most lesions (17/24, 71%), readers remarked that DBT allowed for improved characterization of lesion margins.
**Conclusion:** When compared to the pathology findings, ILC was accurately diagnosed and assessed using CEM. The addition of DBT was reported by the interpreting radiologists as a valuable adjunct for margin analysis.
**Limitations:** Sample size was small, a general issue among studies of ILC in CEM (e.g., 30 subjects in the study by Patel et al. in 2018, 31 in Amato et al. in 2019, and 38 in Costantini M et al. in 2022). The design was retrospective, and furthermore, since all patients underwent CEM due to contraindication to MR, introducing selection bias. No patient had extremely dense breasts or marked enhancement, conclusions regarding the accuracy of CEM in the most challenging breast types cannot be reached.
**Funding for this study:** None.
**Ethics committee - additional information:** Helsinki approval
**Author Disclosures:**
Tal Friehmann: Nothing to disclose
Johansson Lipin Margaret: Author: Rabin Medical Center, Mammography Unit
Ahuva Grubstein: Nothing to disclose
Gil Bachar: Nothing to disclose
Shlomit Tamir: Nothing to disclose

**Comparison of additional malignant lesions detection in Dense vs. Non-dense breasts with Magnetic Resonance Imaging (MRI) or Contrast-Enhanced Mammography (CEM) performed for loco-regional staging**
*P. Minichetti*, M. Lorenzon, S. Sanità, L. Nardone, L. Cereser, R. Girometti, C. Zuiani; Udine/IT
*(paola.minichetti@libero.it)*

**Purpose or Learning Objective:** To compare Additional Malignant Lesions (AML) detection of CEM or MRI (Breast Contrast-Imaging – BCI) performed for preoperative loco-regional staging of Breast Cancer (BC) in patients with Dense Breast (DB) or Non-DB (N-DB).
**Methods or Background:** We retrospectively included 290 patients (median age 62 years) with a biopsy-proven BC who underwent CEM (n=129) or 1.5T-MRI (n=161) at our Institute between January 2022 and December 2023. Staging was performed based on EUSOMA criteria or clinical requests. A radiology resident (with >3 years of experience in breast imaging) reviewed all reports written by one-of-four breast radiologists (with 5-25 years of experience). Extracted data included: density according to BI-RADS (C, D classified as DB; A, B as N-DB), background parenchymal enhancement, features of index lesions and AML (not identified before BCI, pathologically proven). The BCI detection rate of AML in DB and N-DB groups was assessed and compared using T-test. P-values<0.05 were considered statistically significant.
**Results or Findings:** In 201 DB patients, preoperative BCI detected 40 AML (19.9%). In 89 N-DB patients, BCI detected 10 AML (8.9%). The difference in the BCI AML detection rate between DB and N-DB groups was 11% (p=0.0202). In the DB group, the AML detection rate was found to be more than double that in the N-DB group. Specifically, among 71 DB patients, CEM detected 15 AML (21.1%) vs 6 AML in 58 N-DB patients (10.3%); among 130 DB patients, MRI detected 25 AML (19.2%) vs 4 AML in 31 N-DB patients (12.9%).
**Conclusion:** The usefulness of BCI performed for loco-regional staging is significantly higher in DB than in N-DB patients. The detection rate of AML is comparable between MRI and CEM in DB and N-DB patients.
**Limitations:** Small cohort, monocentric.
**Funding for this study:** Nothing.
**Ethics committee - additional information:** Institutional Review Board (IRB-DMED)

Author Disclosures:
Chiara Zuiani: Nothing to disclose
Silvia Sanità: Nothing to disclose
Michele Lorenzon: Nothing to disclose
Rossano Girometti: Nothing to disclose
Lorenzo Cereser: Nothing to disclose
Paola Minichetti: Nothing to disclose
Luigi Nardone: Nothing to disclose

**15:00-16:00** **Research Stage 2**

### Research Presentation Session: Genitourinary

### RPS 507

### Non-malignant pathology of the female pelvis: insights and imaging approaches

**Moderator**
C. Panico; Rome/IT
*(camilla.panico@guest.policlinicogemelli.it)*

### Diaphragmatic Endometriosis: Correlation with Pelvic Disease and Symptoms

*N. Bogveradze*, A. Santonocito, J. Heine, T. Helbich, P. A. Baltzer, R. Wenzl, P. Clauser; Vienna/AT
*(bogveradze.nino@gmail.com)*

**Purpose or Learning Objective:** Diaphragmatic endometriosis (DE) is rare, with limited data on its frequency and management. The deep Pelvic Endometriosis Index (dPEI) and #Enzian classification systems improve pelvis magnetic resonance imaging (MRI) accuracy for diagnosing endometriosis by stratifying disease severity. Our study aimed to assess the prevalence of DE and its correlation with disease severity and associated clinical symptoms.
**Methods or Background:** In this IRB-approved retrospective study, consecutive abdominal MRIs performed for endometriosis (2018-2022) were reviewed by three radiologists in consensus. Positive cases of diaphragmatic endometriosis were defined by consensus among readers or available histology. Pelvic disease severity was assessed using dPEI and #Enzian scores, classifying endometriosis as mild (score ≤2), moderate (scores 3-4), or severe (scores ≥5). Descriptive statistics were used to analyze the relationship between pelvic disease severity, diaphragmatic involvement, and symptoms.
**Results or Findings:** DE was detected in 16/108 patients (14.8%). Among 92 patients with clinical data, 7/92 (7.6%) showed upper abdominal symptoms, with DE confirmed in 4/7 (57.1%). No imaging correlates were identified for three symptomatic patients. Based on dPEI, 2/92 asymptomatic patients had moderate disease (2.2%), while symptomatic patients, 17/92 had mild (18.5%), 36/92 moderate (39.1%), and 37/92 severe (40.2%) disease. Among 16 DE patients by dPEI, 2/16 (12.5%) had mild, 7/16 (43.8%) moderate, and 7/16 (43.8%) severe disease. According to #Enzian, 2/92 asymptomatic patients had severe disease (2.2%), while in symptomatic patients, 8/92 had mild (8.7%), 11/92 moderate (12.0%), and 71/92 severe (77.2%). By #Enzian, of the 16 DE patients, none had mild, 2/16 (12.5%) had moderate, and 14/16 (87.5%) had severe disease.
**Conclusion:** DE detected on MRI was associated with symptoms in 25% of the cases. The presence of diaphragmatic endometriosis is associated with more severe pelvic disease in both scoring systems.
**Limitations:** Retrospective
**Funding for this study:** N/A
**Ethics committee - additional information:** Number: 2057/2017
**Author Disclosures:**
Pascal A.T. Baltzer: Nothing to disclose
Rene Wenzl: Nothing to disclose
Thomas H. Helbich: Nothing to disclose
Ambra Santonocito: Nothing to disclose
Nino Bogveradze: Nothing to disclose
Jana Heine: Nothing to disclose
Paola Clauser: Nothing to disclose

### Adenomyosis and Deep Infiltrating Endometriosis: The Role of Adenomyosis Dominant Side and Type in Implant Distribution

A. Durur Karakaya, *H. Özen Atalay*, V. Samadli, U. Kalkan; Istanbul/TR
*(handeozen15@gmail.com)*

**Purpose or Learning Objective:** The aims of this study are to evaluate the correlation between the types/dominant side of the adenomyosis and the location of deep infiltrating endometriosis (DIE) implants, as well as the relationship between the type of adenomyosis and the presence of endometrioma.
**Methods or Background:** We retrospectively evaluated 311 patients with adenomyosis on MRI examinations between January 2022 and September 2024. The poor quality MRI examination and patients without DIE were excluded from the study, and the final evaluation was performed with 50 patients with adenomyosis and accompanying DIE. Adenomyosis was classified into 3 based on the type as focal, diffuse, cystic; and based on the dominant side in the uterus as anterior, posterior, no dominance. DIE implants were also categorized based on the location as anterior, posterior, or involvement of both compartments. Additionally, the presence and size of endometrioma were evaluated. Statistical significance was examined by chi-square tests ($p < 0.05$).
**Results or Findings:** The median patient age was 39 years. A statistically significant correlation was found between adenomyosis dominant side and DIE implant location ($p = 0.019$). Adenomyosis with no dominant side or diffuse adenomyosis was not significantly associated with effecting of both compartments by DIE implants ($p = 0.275$ and $p = 0.102$, respectively). The correlation between adenomyosis type and endometrioma presence ($p = 0.390$) or size >40 mm ($p = 0.687$) was not statistically significant.
**Conclusion:** To our knowledge, this is the first study evaluating the correlation between the dominant side of adenomyosis and DIE implant location. Previous studies indicated the relationship between the adenomyosis dominant side's and symptom severity, pregnancy loss. Our findings emphasize the need for further investigation into this subject.
**Limitations:** The limitations are the small sample size and retrospective nature.
**Funding for this study:** Not applicable
**Ethics committee - additional information:** Koc University Biomedical Research Ethics Committee, Istanbul/Turkey
**Author Disclosures:**
Afak Durur Karakaya: Nothing to disclose
Hande Özen Atalay: Nothing to disclose
Vugar Samadli: Nothing to disclose
Uzeyir Kalkan: Nothing to disclose

### MRI for endometriosis: ESUR Consensus for indications, reporting and classifications

*I. Thomassin-Naggara*[1], M. Dolciami[2], L. Chamie[3], A. Guerra[4], N. Bharwani[5], S. Freeman[6], P. Rousset[7], L. Manganaro[2]; [1]Paris/FR, [2]Rome/IT, [3]São Paulo/BR, [4]Lisbon/PT, [5]Surbiton/UK, [6]Cambridge/UK, [7]Lyon/FR
*(isabellethomassin@gmail.com)*

**Purpose or Learning Objective:** The ESUR Research Committee appointed two chairs to supervise the development of the updated guidelines.
**Methods or Background:** These guidelines are recommendations developed by the European Society of Urogenital Radiology (ESUR). A targeted literature search was performed to discover recent evidence concerning the imaging of endometriosis. The guidelines were formulated after careful consideration of the available literature by a group of international experts. The panel included 20 experts from 10 different countries, including 14 European centers and one non-European institution. The methodology was based on DELPHI process. Each item was classified as follows: “RECOMMENDED” (if agreement ≥ 80%); “OPTIONAL” (if agreement ≥ 70 % but < 80 %); or “NOT RECOMMENDED” (if consensus was not reached, with < 70 % agreement). The survey was composed of three parts: 1) Indications, 2) MR protocol and lexicon 3) classification/reporting.
**Results or Findings:** Regarding indications, MRI is recommended when TVUS is inconclusive in diagnosing endometriosis or negative, in a symptomatic patient, before surgery or interventional procedure or after surgical treatment if symptoms persist. Regarding reporting, MR classification is recommended, especially radiological score (dPEI). “Patient centered care” is a key dimension of quality care. Good communication with patients, as well as among the healthcare team, has the potential to improve care coordination, enhance safety and outcomes, increase patient satisfaction, and reduce healthcare costs. Standardized MR report and drawing are crucial for improving communication with patients and surgeons.
**Conclusion:** In conclusion, the ESUR consensus on endometriosis emphasizes the importance of standardized reporting and MR classifications to enhance communication between radiologists and the multidisciplinary team, as well as between radiologists and their patients. This is crucial in managing a disease where optimized communication is essential for providing patient-centered and value-based care

**Limitations:** Consensus paper
**Funding for this study:** None
**Ethics committee - additional information:** Not applicable
**Author Disclosures:**
Miriam Dolciami: Nothing to disclose
Pascal Rousset: Consultant: ZIWIG
Adalgisa Guerra: Nothing to disclose
Susan Freeman: Nothing to disclose
Isabelle Thomassin-Naggara: Speaker: GE, Siemens, Guerbet, Hologic, Canon, Guebet, Bracco, GSD, Samsung, Fujifilm, Incepto, ICAD
Luciana Chamie: Nothing to disclose
Nishat Bharwani: Nothing to disclose
Lucia Manganaro: Nothing to disclose

**MRI for endometriosis: ESUR Consensus for protocol and lexicon**
*I. Thomassin-Naggara*[1], M. Dolciami[2], L. Chamie[3], A. Guerra[4], S. Freeman[5], N. Bharwani[6], P. Rousset[7], L. Manganaro[2]; [1]Paris/FR, [2]Rome/IT, [3]São Paulo/BR, [4]Lisbon/PT, [5]Cambridge/UK, [6]Surbiton/UK, [7]Lyon/FR
*(isabellethomassin@gmail.com)*

**Purpose or Learning Objective:** The ESUR Research Committee appointed two chairs to supervise the development of the updated guidelines
**Methods or Background:** The panel included 20 experts from 10 different countries
**Results or Findings:** Pre imaging fasting, the use of antiperistalsic agents, a moderately filled bladder and bowel preparation before MRI are highly recommended. Vaginal and rectal opacification should be considered as an option. MR protocol must include multiplanar T2W and T1W sequence and a sequence that visualizes the kidneys. Superficial endometriosis should be described on T1FS as high signal intensity foci on the peritoneal surface. Endometriomas or implants should be described regarding multiplicity, signal intensity, central or peripheral location and bilaterality. MR evaluation of deep pelvic endometriosis should be performed using a compartmental division defining two horizontal and vertical lines. A bladder nodule should be accurately described according to location measured and the distance to ureteric orifice provided. Uterosacral ligament (USL) of ≤3mm is normal. A USL must be considered as abnormal if a nodule or spiculation is visible in at least two planes or if a bright T1W spot is detected. A posterior vaginal wall or pouch of Douglas nodule should be described and measured. The term rectovaginal septum must be accurately used for sub peritoneal nodules. External adenomyosis should be described according to location and size. The description of a rectosigmoid nodule includes location, number of nodules, longitudinal extent, distance to the anal verge, and wall thickening. The lateral compartment includes the anterior distal round ligament, mediolateral parametrium and posterolateral parametrium. Abdominal wall nodules, ileocaecal junction and appendiceal nodules, as well as sigmoid nodules, must be systematically described.
**Conclusion:** This lecture will present the 10 ESUR statement regarding MR Endometriosis protocol and lexicon
**Limitations:** Consensus
**Funding for this study:** Nond
**Ethics committee - additional information:** Not applicable
**Author Disclosures:**
Miriam Dolciami: Nothing to disclose
Pascal Rousset: Consultant: ZIWIG
Adalgisa Guerra: Nothing to disclose
Susan Freeman: Nothing to disclose
Isabelle Thomassin-Naggara: Speaker: GE, Siemens, Guerbet, Hologic, Canon, Guebet, Bracco, GSD, Samsung, Fujifilm, Incepto, ICAD
Luciana Chamie: Nothing to disclose
Nishat Bharwani: Nothing to disclose
Lucia Manganaro: Nothing to disclose

**MRI features of Accessory Cavitated Uterine Mass (ACUM) and Cystic Adenomyosis**
*Y. Qi*, Z. Zhao, X. Gao, P. Zhang, H. Xue, Y. He, L. Zhu; Beijing/CN
*(qiyafei19910603@163.com)*

**Purpose or Learning Objective:** To assess MRI features in accessory cavitated uterine mass (ACUM) and cystic adenomyosis by evaluating lesion and uterine morphology.
**Methods or Background:** The study included 16 ACUM patients (mean age 25.8 years) and 14 cystic adenomyosis patients (mean age 35.5 years) with surgically and pathologically confirmed diagnoses. Pre-treatment pelvic MRI scans were performed, assessing lesion location, size, shape, cystic cavity, peripheral myometrial thickness, and uterine structure.
**Results or Findings:** Patients presented with symptoms like dysmenorrhea and abdominal pain. Lesions appeared as layered structures with T2 hypointensity peripherally and T1 hyperintensity centrally. ACUM lesions were typically single and located at the fundal lateral wall, while cystic adenomyosis lesions varied in location, 5 adenomyosis lesions were irregular. ACUM patients had lesions ranging from 22mm to 45mm, with a central cystic cavity diameter of 7mm to 36mm and peripheral myometrial thickness of 5mm to 10mm. Fifteen ACUM cases showed a concentric ring structure and the junctional zone was not significantly thickened. Cystic adenomyosis lesions ranging from 20mm to 64mm, with a cystic cavity diameter of 4mm to 60mm and peripheral myometrial thickness of 2mm to 34mm. 5 cases showing uneven peripheral myometrium, only 1 case showing a concentric ring structure. 8 cases showing thickening junctional zone and 8 cases showing adenomyosis or fibroid presence, and 7 patients with ovarian or tubal endometriosis.
**Conclusion:** MRI is valuable for diagnosing ACUM and cystic adenomyosis. ACUM patients are younger, with single, concentric lesions at the fundal lateral wall, rarely showing junctional zone thickening or adenomyosis-like changes. Cystic adenomyosis is suggested by lesions on the posterior wall, irregular shapes, large size, uneven shape, junctional zone thickening, deep endometriosis, and fibroids.
**Limitations:** ACUM and cystic adenomyosis are rare diseases, such the number of cases are limited.
**Funding for this study:** National High Level Hospital Clinical Research Funding, 2022-PUMCH-B-069
**Ethics committee - additional information:** Peking Union Medical College Hospital Ethics committee.
**Author Disclosures:**
Xin Gao: Nothing to disclose
Zichen Zhao: Nothing to disclose
Lan Zhu: Nothing to disclose
Huadan Xue: Nothing to disclose
Yonglan He: Nothing to disclose
Yafei Qi: Nothing to disclose
Peng Zhang: Nothing to disclose

**Comparison of pregnancy outcomes following hysterosalpingography (HSG) with either water soluble contrast media (WSCM) or oil soluble contrast media (OSCM) in routine clinical practice**
*A. P. Hemingway*, K. M. Wourms, S. Sudderuddin, E. Kashef; London/UK

**Purpose or Learning Objective:** Hysterosalpingography (HSG) represents the mainstay of the imaging evaluation of the patency of fallopian tubes in the investigation of subfertility. An apparent association of improved conception following HSG has been recognised since the early 1900's. An randomised controlled trial (RCT )published in 2017 established a significant difference between OSCM and WSCM with ongoing pregnancies in 39.7% following OSCM and 29.1% following WSCM .The purpose of this study was to determine if these results could be replicated in routine clinical practice.
**Methods or Background:** Imaging and medical records relating to 667 consecutive HSGs performed between January 2020 and December 2021 were reviewed. For both WSCM and OSCM data was analysed with respect to age, primary of secondary sub-fertility, normal or abnormal HSGs, number of pregnancies, mode of conception and pregnancy outcome.
**Results or Findings:** Records relating to 667 women were reviewed, 76 lost to follow-up. 591 records, 498 following WSCM, 93 following OSCM were analysed. A total of 354 conceptions were recorded, 291 (58.4%) following WSCM, 63 (67.7%) following OSCM. Spontaneous conceptions accounted for 160 of the WSCM (32.1%) pregnancies and 38 (40.9%) of the OSCM pregnancies. Time to conception was faster in the OSCM group and the pregnancy outcomes were similar in both groups. There were no observed differences in pregnancy rates following assisted reproduction in the two groups.
**Conclusion:** This retrospective study is in accordance with published RCTs and meta analyses that an HSG with WSCM or OSCM is advantageous in increasing the chances of spontaneous conception and that OSCM has a significant advantage over WSCM.
**Limitations:** This is a retrospective study. The OSCM group is smaller than the WSCM group. The study period and analysis is being extended to encompass HSGs undertaken in 2022 and 2023
**Funding for this study:** No external funding. HSGs standard of care. Retrospective data collection
**Ethics committee - additional information:** This retrospective study was approved IRAS Number 254621
**Author Disclosures:**
Katherine Marie Wourms: Nothing to disclose
Siham Sudderuddin: Nothing to disclose
Anne Patricia Hemingway: Nothing to disclose
Elika Kashef: Consultant: Rocket Medical

**Comparing Inter-reader Agreement Between dPEI and #Enzian Classifications: Which Is the Better One?**

*A. Santonocito*, N. Bogveradze, J. Heine, T. H. Helbich, P. A. Baltzer, R. Wenzl, P. Clauser; Vienna/AT
*(santonocitoambra@gmail.com)*

**Purpose or Learning Objective:** The deep pelvic endometriosis index (dPEI) and #Enzian classifications have been introduced to facilitate reporting endometriosis patients. While studies show good inter-reader agreement with #Enzian, limited data exist for dPEI. This study aimed to assess the inter-reader agreement of both classifications.
**Methods or Background:** In this retrospective, monocentric, IRB-approved study the pelvic MRIs performed in consecutive patients for suspected endometriosis from February 2018 to November 2023 were evaluated. Two readers (R1, dedicated radiologist, 5 years of experience; R2, general radiologist, 2 years of experience) independently assessed the presence of lesions using dPEI and #Enzian classifications. The extent of disease was then categorized as mild (score ≤2), moderate (3-4), or severe (≥5). Inter-reader agreement was evaluated by using Cohen's Kappa.
**Results or Findings:** A total of 108 patients (mean age 32.7 ±7.1 years; range 21-50 years old) were included in this study. Using dPEI, R1 classified 22 (20.4%) patients with mild, 43 (39.8%) moderate, and 43 (39.8%) severe disease; while R2 classified 28 (25.9%) patients with mild, 43 (39.8%) moderate, and 37 (34.3%) severe disease. The inter-reader agreement for dPEI was k=0.47 (p<0.001). Using #Enzian, R1 classified 10 (9.3%) patients as mild, 12 (11.1%) as moderate, and 86 (79.6%) as severe disease; while R2 classified 21 (19.4%) patients as mild, 34 (31.5%) moderate, and 53 (49.1%) severe disease. The inter-reader agreement for #Enzian was k=0.31 (p<0.001).
**Conclusion:** Inter-reader agreement was moderate for dPEI and fair for #Enzian. This evidence suggests that dPEI, as a radiologically based classification, could be easier to use by non-specialist radiologists. However, a dedicated training is essential to improve inter-reader agreement in the evaluation of endometriosis.
**Limitations:** Retrospective study; lacking of assessment of histology
**Funding for this study:** None
**Ethics committee - additional information:** Number: 2057/2017
**Author Disclosures:**
Pascal A.T. Baltzer: Nothing to disclose
Rene Wenzl: Nothing to disclose
Thomas H. Helbich: Nothing to disclose
Ambra Santonocito: Nothing to disclose
Nino Bogveradze: Nothing to disclose
Jana Heine: Nothing to disclose
Paola Clauser: Nothing to disclose

**15:00-16:00** **Research Stage 3**

### Research Presentation Session: Chest

### RPS 504

### Imaging of diffuse lung diseases: old and new

**Moderator**
M. Occhipinti; Florence/IT
*(mariaelena.occhipinti@gmail.com)*

**Imaging and Clinical Features of Interstitial Lung Abnormalities (ILA) that Predict Progression to Idiopathic Pulmonary Fibrosis (IPF)**

*T. Schnitzler*, J. H. Sohn; San Francisco, CA/US
*(tician.schnitzler@ucsf.edu)*

**Purpose or Learning Objective:** Interstitial lung abnormality (ILA) is often an incidental imaging finding, representing early or mild fibrosis. While most cases do not progress, some advance to idiopathic pulmonary fibrosis (IPF), leading to severe outcomes. Accurate risk stratification of ILA on non-contrast chest CT is crucial for guiding follow-up and early treatment. This study aims to improve stratification by identifying imaging and clinical features that predict progression from ILA to IPF.
**Methods or Background:** This retrospective case-control study included patients from a longitudinal ILD database: a low-risk ILA cohort (n = 525) and a high-risk ILA cohort (n = 221). Imaging features analyzed included subpleural fibrotic reticulation, cranial extent of fibrosis, anterior lung involvement, and emphysema severity. Clinical variables included age and gender. Statistical analyses were conducted using chi-square tests for categorical variables and independent t-tests for continuous variables.
**Results or Findings:** The high-risk ILA cohort had significantly higher rates of subpleural fibrotic changes (78% vs. 36%, p < 0.001), cranial extent of fibrosis (61% vs. 14%, p < 0.001), anterior lung involvement (86% vs. 37%, p < 0.001), and severe emphysema (48% vs. 39%, p < 0.001) compared to the low-risk cohort. The high-risk cohort was also older (mean age 72.64 years vs. 70.65 years, p = 0.020), with no significant gender difference.
**Conclusion:** This study identifies key imaging and clinical predictors of ILA progression to IPF, such as subpleural fibrotic changes, cranial extent of fibrosis, and older age. These findings could improve risk stratification, guiding timely monitoring and interventions to enhance patient outcomes. Future research should validate these findings in larger, multi-center cohorts.
**Limitations:** The main limitation is the retrospective single-center design.
**Funding for this study:** RSNA Research Fellow Grant 2024
Swiss Society for Radiology Research Grant 2023
Bangerter-Rhyner Foundation, Basel, Switzerland
**Ethics committee - additional information:** This study is IRB approved (17-22317).
**Author Disclosures:**
Tician Schnitzler: Nothing to disclose
Jae Ho Sohn: Nothing to disclose

**Identifying progressive pulmonary fibrosis on serial CT: An international multi-observer study**

*L. Sun*[1], M. A. Mestas Nuñez[2], J. Jacob[1], S. Piciucchi[3], L. Calandriello[4], A. Carvalho[5], R. E. Ledda[6], M. Chen[1], A. Devaraj[1]; [1]London/UK, [2]Barcelona/ES, [3]Forlì/IT, [4]Rome/IT, [5]Porto/PT, [6]Parma/IT
*(logan.sun@doctors.org.uk)*

**Purpose or Learning Objective:** To evaluate the performance and agreement of thoracic radiologists and interstitial lung disease (ILD) physicians in identifying progressive pulmonary fibrosis on serial CT scans in patients without idiopathic pulmonary fibrosis (IPF).
**Methods or Background:** 100 patients with various non-IPF fibrotic lung diseases (median age, 64 years [range, 36 to 85]; male, n=40) had serial CTs obtained 6 to 24 months apart, which were reviewed independently by 12 ILD physician and thoracic radiologist readers blinded to clinical data. CTs were reviewed side-by-side and categorised as one of two groups: Stable Disease or Progressive Fibrosis. Groups were compared using contemporary relative change in percentage predicted forced vital capacity (FVC), per reader and across the cohort, and analysed by Mann-Whitney U test and mixed-effects modelling. Interobserver agreement was assessed using intraclass correlation coefficient (ICC).
**Results or Findings:** Preliminary data are presented. Mean FVC change for all patients was -6.28% (SD, 14.9). For individual readers, there was a significant difference in median FVC decline between corresponding Progressive Fibrosis versus Stable Disease CT groups (range, -6.84% to -11.44%, p=<0.001–0.015). For the whole reader cohort, mean FVC decline was significantly greater in Progressive Fibrosis versus Stable Disease on CT (-10.70%, 95% CI [-11.89%, -9.50%] versus -1.47%, 95% CI [-2.78%, -0.15%]). Interobserver agreement was moderate (ICC = 0.501, 95% CI [0.420, 0.588]).
**Conclusion:** Among specialist thoracic radiologists and ILD physicians, visual evaluation of serial CT scans is a valuable method for determining progressive fibrosis in non-IPF fibrotic lung diseases, judged against contemporary FVC decline, though interobserver agreement remains moderate.
**Limitations:** Single-centre retrospective study
**Funding for this study:** Nil sought
**Ethics committee - additional information:** Prior IRAS approval for retrospective research within the Royal Brompton Hospital radiology department
**Author Disclosures:**
Sara Piciucchi: Nothing to disclose
Roberta Eufrasia Ledda: Nothing to disclose
Andre Carvalho: Nothing to disclose
Joseph Jacob: Nothing to disclose
Mitchell Chen: Nothing to disclose
Anand Devaraj: Nothing to disclose
Logan Sun: Nothing to disclose
Lucio Calandriello: Nothing to disclose
Marcos Alejandro Mestas Nuñez: Nothing to disclose

**Diagnostic Delay of Lung Cancer in Interstitial Lung Disease**
*T. Schnitzler*, J. H. Sohn; San Francisco, CA/US
*(tician.schnitzler@ucsf.edu)*

**Purpose or Learning Objective:** Interstitial lung disease (ILD) patients have an increased risk of lung cancer, but detection is challenging due to background fibrosis, leading to diagnostic delays. There is limited research on lung cancer in ILD, particularly regarding diagnostic delays. This study aims to analyze delayed lung cancer diagnoses in ILD patients, including tumor stage at diagnosis, growth rates, treatment regimens, and outcomes.
**Methods or Background:** This retrospective study included ILD patients with concomitant lung cancer (pathology proven or >50% radiologically suspected) from two referral centers. A thoracic radiologist re-reviewed chest CTs to determine the earliest visible and callable lesion time, when it was first deemed suspicious, and its growth rate. Tumor staging, treatment regimens, and outcomes were analyzed. Survival curves were generated using the Kaplan-Meier method, comparing median survival times between delayed and non-delayed cancer cases with the log-rank test.
**Results or Findings:** Seventy-seven cases of concurrent ILD and lung cancer were identified (53 pathology proven, 24 radiologically presumed). Delayed diagnoses occurred in 47% (36/77) of cases, with an average delay of 3.42 years. These delayed cases had a mean annual growth rate of 293% and a mean doubling time of 3.3 years. An additional 5% (4/61) were diagnosed post-lung transplant. The median survival time was 1269 days for early detection versus 867 days for delayed detection. However, the difference in survival was not statistically significant ($p = 0.80$).
**Conclusion:** This study found that 52% of lung cancer cases in ILD had delayed diagnoses, with an average delay of 3.42 years. Despite the delays, there was no significant difference in mortality between early and delayed detection cases.
**Limitations:** The main limitation is the retrospective study design.
**Funding for this study:** Swiss Society for Radiology Research Grant 2023 Bangerter-Rhyner Foundation, Basel, Switzerland
**Ethics committee - additional information:** This study was approved by the local IRB (17-22317)
**Author Disclosures:**
Tician Schnitzler: Nothing to disclose
Jae Ho Sohn: Nothing to disclose

**Radiological assessment of bronchial and arterial dimensions and mucus plug presence in 640 bronchiectasis patients: insights from the EMBARC registry**
*Y. Chen*[1], A. Pieters[1], E-R. Andrinopoulou[1], S. Aliberti[2], M. Loebinger[3], P. Ciet[1], J. Chalmers[4], H. A. W. M. Tiddens[1], On Belalf Of Embarc Study Group[4]; [1]Rotterdam/NL, [2]Humanitas Research Hospital, Milan/IT, [3]London/UK, [4]Dundee/UK
*(y.chen.1@erasmusmc.nl)*

**Purpose or Learning Objective:** Key features of bronchi in bronchiectasis disease are irreversible widening, wall thickening and mucus plugging. The bronchiectasis registry EMBARC lacks currently objective quantitative metrics for these features. The aim of our study was to analyse EMBARC chest CTs using an AI-based algorithm measuring bronchus and artery (BA) dimensions and ratios and counting mucus plugs (MP).
**Methods or Background:** 885 CTs from eight EMBARC centres were retrospectively collected for automatic analysis using LungQ (Thirona, The Netherlands), which segments the bronchial tree and identifies segmental (G0) and distal (G1,2,3…) generations. For each BA-pair, the following dimensions are computed: diameters of bronchial outer edge (Bout), inner edge (Bin), and artery (A), and wall thickness (Bwt) and the following BA-ratios: Bout/A, Bin/A, Bwt/A, and bronchial wall area/outer area (Bwa/Boa). Cut-offs for mild and severe bronchial widening are Bout/A>1.1 and >1.5, respectively and for thickening (Bwt/A>0.14). The MP analysis automatically segments the bronchial tree, detects the total number and volume of MP.
**Results or Findings:** 640 CTs were successfully analysed, identifying 141,978 BA-pairs from G0 until G29 (222 BA-pairs per CT). Bout/A>1.1 or >1.5 were observed in 73% and 39% of all BA-pairs, respectively. Bwt/A>0.14 was observed in 49% of all BA-pairs. The median(IQR) Bout/A, Bin/A, Bwt/A, and Bwa/Boa for G1-6 were 1.34(1.07, 1.72), 1.04(0.81, 1.35), 0.13(0.1, 0.2). MP were found in 83% of CTs, with a median number of 8 plugs and a median volume of 0.44mL per CT.
**Conclusion:** Our study demonstrates the capability of AI-based algorithms to measure BA-dimensions and detect mucus plugs on chest CT scans of bronchiectasis patients. Our findings show widespread but heterogeneous bronchial widening and thickening, along with the presence of mucus plugs, indicative of active infection and/or inflammation.
**Limitations:** Retrospective study
**Funding for this study:** Supported by the Innovative Medicines Initiative and The European Federation of Pharmaceutical Industries and Associations companies under the European Commission–funded Horizon 2020 Framework Program and by Inhaled Antibiotic for Bronchiectasis and Cystic Fibrosis (grant 115721). EMBARC3 is funded by the European Respiratory Society through the EMBARC3 clinical research collaboration. EMBARC3 is supported by project partners Armata, AstraZeneca, Boehringer Ingelheim, Chiesi, CSL Behring, Grifols, Insmed, Janssen, Lifearc, and Zambon. J.D.C. is supported by the GlaxoSmithKline/Asthma and Lung UK Chair of Respiratory Research.
**Ethics committee - additional information:** The study received central ethical approval from the Multicentre Research Ethics Committee in the UK on Jan 8, 2015 (14/SS/1101) and the study is sponsored by the University of Dundee, Dundee, UK.
**Author Disclosures:**
Stefano Aliberti: Grant Recipient: Stefano Aliberti has received grants or contracts fees from INSMED incorporated, CHIESI, Fisher&Paykel; and received consulting fees from GSK, McGRAW HILL, Zambon, AstraZeneca, CSL Behring GmbH, Moderna, Chiesi, MSD Italia, Pysioassist SAS, GlaxoSmithKline
Eleni-Rosalina Andrinopoulou: Nothing to disclose
James Chalmers: Grant Recipient: James D Chalmers has received research grants from AstraZeneca, Boehringer Ingelheim, GlaxoSmithKline, Gilead Sciences, Grifols, Novartis, Insmed and Trudell; and received consultancy or speaker fees from Antabio, AstraZeneca, Boehringer Ingelheim, Chiesi, GlaxoSmithKline, Insmed, Janssen, Novartis, Pfizer, Trudell and Zambon.
Pierluigi Ciet: Grant Recipient: Pierluigi Ciet has received grants or contracts fees from NOW-Dutch Research Councel and Horizon Pathfinder; and has received payment or honoraria for lectures, presentations etc from Chiesi and Vertex.
Michael Loebinger: Grant Recipient: Michael Loebinger has received consulting fees from Armata, 30T, AstraZeneca, Parion, Ismed, Chiesi, Zambon, Electromed, Recode and Boehringer Ingelheim; and received payment or honoraria for lectures, presentations etc from Ismed.
Harm A W M Tiddens: Employee: Harm AWM Tiddens has received research grants from Thirona as Chief Medical Officer and Emeritus Professor ErasmusMC Sophia; and has received payment or honoraria for lectures, presentations etc from Vertex.
Yuxin Chen: Nothing to disclose
On Belalf Of Embarc Study Group: Nothing to disclose
Angelina Pieters: Nothing to disclose

**Computed Tomography-Derived Quantitative Imaging Biomarkers enable the prediction of survival and disease severity in patients with Systemic Sclerosis**
*M. M. Sieren*[1], H. Graßhoff[1], L. Berkel[1], G. Riemekasten[1], F. Nensa[2], R. Hosch[2], J. Barkhausen[1], R. Klöckner[1], F. Wegner[1]; [1]Lübeck/DE, [2]Essen/DE
*(malte.sieren@uksh.de)*

**Purpose or Learning Objective:** Systemic Sclerosis (SSc) is a complex connective tissue disorder with variable disease progression and outcome. While chest CT imaging is recommended in all patients to evaluate interstitial lung disease, AI-driven body composition analysis (BCA) can further enhance radiological assessment by providing quantitative imaging biomarkers. This study aims to assess BCA's ability to predict survival, complications, and disease severity on chest CT.
**Methods or Background:** CT scans were obtained from a prospectively maintained cohort of 452 SSc patients, including 128 with at least one CT scan and 35 patients with up to three follow-up exams. The follow-up period averaged 36.5±4.5 months. An AI-based 3D BCA algorithm measured muscle volume, adipose tissue compartments, and bone mineral density. BCA Parameters were evaluated in relation to clinical, laboratory, and functional data on baseline and follow-up scans. Survival prediction was performed using regression analysis, comparing models based on BCA, BMI, and clinical parameters.
**Results or Findings:** The BCA model outperformed BMI and clinical models in predicting survival (BCA AUC=0.74, BMI AUC=0.49, clinical parameters AUC=0.53). Including longitudinal BCA data further improved the model's AUC to 0.82. Altered BCA parameters were linked to increased odds ratios [with 95% confidence interval] for complications like acral ulcers (1.7 [1.1-1.9]), interstitial lung disease (2.1 [1.4-4.4]), cardiac (2.0 [1.3-3.0]) and gastrointenstinal manifestations (1.6 [1.4-1.9], all $p<0.05$).
**Conclusion:** This study highlights that quantitative body composition biomarkers outperform established parameters in predicting survival and specific disease manifestations. These findings provide a blueprint how radiological assessment can transform from primarily qualitative assessment to including previously unavailable quantitative data leading to more personalized patient care, potentially improving outcomes for SSc patients.
**Limitations:** The study's single-center design and small sample size may limit generalizability, and variations in CT quality could affect AI-based BCA accuracy.

**Funding for this study:** None.
**Ethics committee - additional information:** Study/protocol number: AZ 22-289
**Author Disclosures:**
Lennart Berkel: Nothing to disclose
Franz Wegner: Nothing to disclose
Roman Klöckner: Nothing to disclose
Gabriela Riemekasten: Nothing to disclose
Jörg Barkhausen: Nothing to disclose
Malte Maria Sieren: Nothing to disclose
Hanna Graßhoff: Nothing to disclose
René Hosch: Nothing to disclose
Felix Nensa: Nothing to disclose

**Development of imaging protocol and radiomics-based nomogram for assessing lesion reversibility in connective tissue disease-associated interstitial lung disease**

Y. Zhang, *Y. Wang*, X. Yu, J. Wei, H. Wu; Shanghai/CN
*(yu.wang@philips.com)*

**Purpose or Learning Objective:** To develop an imaging protocol for assessing lesion reversibility and a radiomics-based nomogram for predicting lesion reversibility in connective tissue disease-associated interstitial lung disease (CTD-ILD).
**Methods or Background:** A retrospective study categorized CTD-ILD patients into training, internal and external validation cohorts. An imaging protocol of serial chest CT scans for assessing lesion reversibility was developed, classifying patients as completely reversible (CR) and non-CR groups based on CT lesion changes. Lesions were evaluated using morphological CT features and radiomics signatures at the lung-zone level. Visual, radiomics, and combined nomogram models were developed and compared through receiver operating characteristic (ROC) curve analysis.
**Results or Findings:** Among 153 patients with 575 affected lung zones, a five-feature radiomics signature significantly correlated with ILD lesion reversibility. The radiomics model showed robust discrimination, comparable to the visual model in the validation cohorts (internal: 0.77, 95% CI: [0.68, 0.86] versus 0.87, 95% CI: [0.81, 0.94], p=0.056; external: 0.73, 95% CI: [0.66, 0.79] versus 0.78, 95% CI: [0.72, 0.84], p=0.20), and inferior to the visual model in the training cohort (0.72, 95% CI: [0.66, 0.79] versus 0.82, 95% CI: [0.77, 0.87], p=0.02). The combined nomogram model outperformed the visual model alone in the training and external validation cohorts (0.86, 95% CI: [0.81, 0.91], p=0.03; 0.82, 95% CI: [0.77, 0.87]; p=0.048).
**Conclusion:** An imaging protocol was established for assessing lesion reversibility in CTD-ILD. The radiomics signature provided a quantitative approach to predict lesion reversibility. The combined nomogram improved the predictive accuracy beyond morphological features alone.
**Limitations:** First, clinical information were incomplete and not included in the predictive model. Future research incorporating more clinical information is needed. Second, the manual segmentation of lung zones might introduce bias across various scans.
**Funding for this study:** None
**Ethics committee - additional information:** Ethics approval (No. LY2023-019-B) was granted by the institutional review board (IRB) of Shanghai Jiaotong University, School of Medicine, Renji Hospital. The IRB waived informed consent requirement for this retrospective study.
**Author Disclosures:**
Yu Wang: Nothing to disclose
Jiaxu Wei: Nothing to disclose
Ying Zhang: Nothing to disclose
Xiao Yu: Nothing to disclose
Huawei Wu: Nothing to disclose

**Pleural Effusion as a Prognostic Indicator in COVID-19: A nationwide Multicenter Analysis**

*A. M. Bucher*[1], E. Frodl[1], F. G. Meinel[2], M. M. Sieren[3], M. A. Fink[4], M. S. May[5], M. S. Kim[6], T. Vogl[1], A. Surov[7]; [1]Frankfurt/DE, [2]Rostock/DE, [3]Lübeck/DE, [4]Heidelberg/DE, [5]Erlangen/DE, [6]Essen/DE, [7]Minden/DE

**Purpose or Learning Objective:** This study evaluates the prognostic significance of pleural effusion (PE) in COVID-19 patients across 13 German centers, part of the RACOON (Radiological Cooperative Network) project. We aimed to assess the relationship between PE and key clinical outcomes, in a large multicentre study.
**Methods or Background:** In this retrospective study, 1183 COVID-19 patients (29.3% women, 70.7% men) underwent chest CT to assess the presence, volume, and density of PE. We analyzed associations between PE and clinical outcomes including 30-day mortality, ICU admission, and mechanical ventilation. We used univariable and multivariable regression analyses, adjusting for confounders such as the COVID-19 CT severity score.
**Results or Findings:** PE was identified in 31.5% of patients. A significant correlation was found between PE and 30-day mortality (47.5% in non-survivors vs. 27.3% in survivors, p<0.001). PE presence independently predicted mortality with a hazard ratio (HR) of 2.22 (95% CI 1.65-2.99, p<0.001). However, PE volume and density were not significantly associated with mortality. ICU admission was necessary in 46.8% of patients, and 26.7% required mechanical ventilation. PE presence was also linked to ICU admission and ventilation but not its volume or density.
**Conclusion:** Pleural effusion is a significant independent predictor of 30-day mortality in COVID-19 patients, irrespective of its volume or density. These findings underscore the importance of including PE detection in routine CT assessments to enhance clinical decision-making and patient care.
**Limitations:** This retrospective study was limited to German tertiary care centers, which may not represent other settings.
**Funding for this study:** Funded by „NUM 2.0“ (FKZ: 01KX2121)
**Ethics committee - additional information:** IRB approval for this retrospective multi centre study was obtained (20-719).
**Author Disclosures:**
Eric Frodl: Nothing to disclose
Alexey Surov: Nothing to disclose
Thomas Vogl: Nothing to disclose
Moon Sung Kim: Nothing to disclose
Matthias Stefan May: Nothing to disclose
Matthias Alexander Fink: Nothing to disclose
Felix G. Meinel: Nothing to disclose
Malte Maria Sieren: Nothing to disclose
Andreas Michael Bucher: Nothing to disclose

**QIP Are Chest Radiographs Being Conducted in Accordance with the British Thoracic Society Recommendations for Adults Diagnosed with Community Acquired Pneumonia?**

*M. Mobini*, A. Nehvi, S. Buckingham, L. Mills; Stevenage/UK

**Purpose or Learning Objective:** This audit evaluates whether follow-up chest radiographs are being performed for adults diagnosed with Community-Acquired Pneumonia (CAP) according to British Thoracic Society (BTS) guidelines. A gap was identified when many patients did not receive a follow-up chest X-ray within six weeks, prompting an audit to identify barriers and gaps in care.
**Methods or Background:** CAP affects 0.5% to 1% of UK adults annually and carries a mortality risk of 5-14%. Follow-up X-rays are crucial to ensure the resolution of pneumonia and to exclude underlying conditions such as lung cancer. The first cycle retrospectively reviewed 50 adult patients diagnosed with CAP between November 2023 and March 2024. The second cycle repeated the review from March to July 2024, after implementing several interventions. Data were collected to assess compliance with follow-up X-ray recommendations, virtual Pneumonia clinic (VPC) referrals, and Casualty (CAS) alerts.
**Results or Findings:** In the first cycle, only 32.6% of patients received follow-up X-rays within six weeks, with 67.4% failing to comply. Among those discharged from the emergency department, 67.8% did not have a follow-up X-ray. Referrals to the Virtual Pneumonia Clinic (VPC) were low (23.2%), and only 5% of reports included a CAS alert. Following interventions aimed at raising awareness among doctors, improving documentation, enhancing patient education, and increasing CAS alerts, the second cycle showed significant improvement. Compliance with follow-up X-rays increased to 70%, with VPC referrals rising to 77% and CAS alerts reaching 73%. The compliance rate for follow-up X-rays among patients discharged from emergency care increased from 32.2% to 65%.
**Conclusion:** A marked improvement in adherence to BTS guidelines after targeted interventions, lead to more follow-up X-rays, VPC referrals, and CAS alerts.
**Limitations:** 1) Retrospective Design 2)Small sample size 3) Single-Centered Audit
**Funding for this study:** East and North Hertfordshire NHS Trust.
**Ethics committee - additional information:** The study was approved by the trust ethics committee and audit department
**Author Disclosures:**
Moein Mobini: Author: second author
Aabid Nehvi: Author: 1
Susan Buckingham: Consultant: 2 Author: 2
Lauren Mills: Author: 3

**15:00-16:00** **Research Stage 4**

### Research Presentation Session: Cardiac

### RPS 503

### Cardiac CT: plaques and beyond

**Moderator**
D. Suchá; Utrecht/NL
**Author Disclosures:**
Dominika Suchá: Research Grant/Support: Philips Healthcare Research Support received by the Department of Radiology, UMC Utrecht, NL

### CT coronary calcium scoring to detect obstructive coronary artery disease in primary care patients with non-typical chest pain

M. Y. Koopman[1], R. Willemsen[2], B. Kietselaer[3], P. M. A. Van Ooijen[1], J-W. Gratama[4], R. Braam[4], R. Van Bruggen[4], P. Van Der Harst[5], *R. Vliegenthart*[1]; [1]Groningen/NL, [2]Maastricht/NL, [3]Rochester, MN/US, [4]Apeldoorn/NL, [5]Utrecht/NL
*(r.vliegenthart@umcg.nl)*

**Purpose or Learning Objective:** Computed Tomography coronary calcium scoring (CT-CCS) has higher sensitivity for detection of obstructive coronary artery disease (OCAD) than exercise electrography (x-ECG), but its utility as an initial diagnostic test in primary care remains unclear. This pilot study compares CT-CCS results with x-ECG results in primary care and assesses patients' perspectives.
**Methods or Background:** Thirty-eight primary care offices participated in this study. After cluster randomisation, 19 offices referred patients with atypical angina pectoris or non-specific thoracic complaints for CT-CCS and 19 offices used x-ECG as the primary test (standard care). Clinical data were collected using electronic patient records, and patients' perspectives on the diagnostic test were assessed through a questionnaire. Outcome measures included CAD diagnosis, initiation of cardiovascular risk management (CVRM), and patient satisfaction.
**Results or Findings:** In total, 101 patients were included. In 25 patients undergoing X-ECG, one (4%) had a positive test result and received CVRM, but no patients were diagnosed with obstructive CAD. CT-CCS was performed in 76 patients. 17 CT-CCS patients (23%) had a positive test result (calcium score >100), and 14 (19%) received CVRM. Obstructive CAD was diagnosed in four CT-CCS patients (5.3%). Of CT-CCS patients, 31 (43%) perceived the test as 'very easy' compared to none of the x-ECG patients.
**Conclusion:** CT-CCS is a promising diagnostic tool in primary care for the detection of obstructive CAD, offering a more patient-friendly experience compared to x-ECG.
**Limitations:** Small cohort, especially in the x-ECG arm, and low OCAD rate. A few patients received the test result before completing the questionnaire. Baseline cardiovascular related risk factors were inconsistently reported in electronic patient records.
**Funding for this study:** Funding was received from the Dutch Heart Foundation (Hartstichting, grant number: CVON2017-14).
**Ethics committee - additional information:** The Medical Ethical Committee of the University Medical Center of Groningen approved CONCRETE (number 2018/404).
**Author Disclosures:**
Robert Willemsen: Nothing to disclose
Pim Van Der Harst: Nothing to disclose
Richard Braam: Nothing to disclose
Peter M.A. Van Ooijen: Nothing to disclose
Moniek Yvonne Koopman: Nothing to disclose
Rozemarijn Vliegenthart: Research/Grant Support: Siemens Healthineers
Bas Kietselaer: Nothing to disclose
Rykel Van Bruggen: Nothing to disclose
Jan-Willem Gratama: Nothing to disclose

### Thin-slice non-contrast CT detects prognostically relevant calcified plaques missed by conventional calcium scoring

*F. Yavuz*, F. Biavati, K. Schulze, S. Tsogias, B. Föllmer, A-M. Stantien, M. Bosserdt, M. Dewey; Berlin/DE

**Purpose or Learning Objective:** To evaluate whether thin-slice non-contrast CT (NCCT) can detect prognostically relevant coronary plaques missed by conventional 3.0-mm reconstructions.
**Methods or Background:** This study included 141 patients from the CAD-Man trial [NCT00844220] (mean age 60.77 ± 11.06 years, 55% female) with available thin-slice NCCT (0.5-mm). The Agatston method was used to detect calcified plaques. Sensitivity and specificity for the detection of calcified plaques were calculated using CT angiography (CTA) as the reference standard. Lesion- and patient-level statistics were calculated for plaque volume parameters. Prognostic relevance was assessed by evaluating plaque progression rates for plaques detected only on thin-slice reconstructions, using median 10-year follow-up data when available.
**Results or Findings:** In total 551 calcified plaques were detected. Thin-slice NCCT showed a higher sensitivity (91.83%; 506/551) for detecting coronary calcified plaques compared to 3.0-mm reconstruction (82.76%; 456/551), although standard reconstructions showed an overall per-patient increased mean calcified plaque volume (197.22 mm3 ± 330.05 mm3) compared to thin-slice NCCT (162.65 mm3 ± 284.1 mm3). Conversely, we observed a slightly lower specificity (97.23%; 492/506) for thin-slice NCCT compared to standard reconstructions (99.56%; 454/456). Coronary calcified plaques missed in standard reconstructions were smaller in volume (2.67 mm3 ± 1.47 mm3) compared to all detected plaques (20.68 mm3 ± 25.56 mm3). Missing calcified plaques on standard reconstructions would have led to the omission of 9 out of 141 patients (6.4%). Additionally, plaques only identified on thin-slice NCCT at baseline were clearly visible at follow-up, with an average 7.9-fold increase in volume.
**Conclusion:** Coronary calcified plaques detected exclusively on thin-slice NCCT reconstructions showed increased plaque progression rates compared to plaques detected in conventional calcium scoring.
**Limitations:** This study involved patients from a single-centre, and 10-year follow-up data were not available for all patients with missed plaques.
**Funding for this study:** This study was funded by a grant of the Heisenberg programme.
**Ethics committee - additional information:** The study was approved by ethics committee at Charité (EA1/124/23).
**Author Disclosures:**
Kenrick Schulze: Nothing to disclose
Ferhat Yavuz: Nothing to disclose
Anne-Marieke Stantien: Nothing to disclose
Marc Dewey: Board Member: M.D. is European Society of Radiology (ESR) Publications Chair (2022-2025); the opinions expressed in this presentation are the author's own and do not represent the view of ESR Grant Recipient: EU (EC-GA 603266 in HEALTH.2013.2.4.2-2) DFG (DE 1361/14-1, DE 1361/18-1, BIOQIC GRK 2260/1, Radiomics DE 1361/19-1 [428222922] and 20-1 [428223139] in SPP 2177/1), GUIDE-IT (DE 1361/24-1), Berlin University Alliance (GC_SC_PC 27), G-BA (01NVF23002), Berlin Institute of Health (Digital Health Accelerator) Patent Holder: Patent on fractal analysis of perfusion imaging (jointly with Florian Michallek, EPO 2022 EP3350773A1, and USPTO 2021 10,991,109, approved) Author: Cardiac CT (Springer Nature) Research/Grant Support: Siemens, General Electric, Philips, Canon Other: Hands-on cardiac CT courses (www.ct-kurs.de) Institutional research agreements: Siemens, General Electric, Philips, Canon. Patent on fractal analysis of perfusion imaging (jointly with Florian Michallek, EPO 2022 EP3350773A1, and USPTO 2021 10,991,109, approved) M.D. is European Society of Radiology (ESR) Publications Chair (2022-2025); the opinions expressed in this presentation are the author's own and do not represent the view of ESR
Sotirios Tsogias: Nothing to disclose
Federico Biavati: Nothing to disclose
Maria Bosserdt: Nothing to disclose
Bernhard Föllmer: Nothing to disclose

### Association of features derived from segment-level coronary artery calcium scoring with major adverse cardiovascular events: A multicentre study

*S. Tsogias*, B. Föllmer, M. Mohamed, F. Biavati, K. Schulze, M. Bosserdt, M. Dewey; Berlin/DE
*(sotirios.tsogias@charite.de)*

**Purpose or Learning Objective:** To investigate the association of segment-level coronary artery calcium (CAC) scoring derived features with major adverse cardiovascular events (MACE) compared to vessel-based and overall CAC scoring.
**Methods or Background:** This subanalysis of the multicentre DISCHARGE trial (NCT02400229) included a total of (N = 1446) patients (mean age 59.9 ± 10.2 years) who had received a calcium scoring CT and were followed up over a median timespan of 3.5 years. The definition of MACE included nonfatal stroke, nonfatal myocardial infarction and cardiovascular death. Associations with MACE were examined for proximal (LM and proximal segments of the LAD, LCX and RCA) versus non-proximal calcifications and the total number of segments containing calcifications out of 19 (0: No calcification; 1: Limited; 2-9: Moderate, ≥ 10: Extensive). CAC scores were obtained both manually and using deep learning-based scoring methods. Analysis was performed using Cox proportional hazards regression adjusting for age, sex, body-mass-index, diabetes, dyslipidemia, hypertension, family history, smoking status and Agatston categories (< 400; ≥ 400) with hazard ratios (HR) and 95% confidence intervals (CI).

Wednesday

**Results or Findings:** During follow-up a total of 31 MACE occurred. Proximal vessel calcifications were associated with higher risk for MACE (HR = 3.9, 95% CI [1.02, 14.5], p < .05). A moderate number of calcified segments [2-9 segments] was also associated with an increased risk for MACE (HR = 4.2, 95% CI [1.08, 16.1], p < .05).
**Conclusion:** Proximal vessel calcification as well as moderate segment calcification were associated with a higher risk for MACE.
**Limitations:** Due to the low number of MACE in this study population 2.1% (31 of 1446) the overall predictive value of the segment level CAC scoring may have been underrepresented.
**Funding for this study:** This work was funded by the German Research Foundation through the graduate program BIOQIC (GRK2260, project-ID: 289347353) and the DISCHARGE project (603266-2, HEALTH-2012.2.4.-2) funded by the FP7 Program of the European Commission.
**Ethics committee - additional information:** The study was approved by The German Federal Office for Radiation Protection and the local or national authorities at each trial site.
**Author Disclosures:**
Kenrick Schulze: Nothing to disclose
Mahmoud Mohamed: Nothing to disclose
Marc Dewey: Grant Recipient: EU (EC-GA 603266 in HEALTH.2013.2.4.2-2) DFG (DE 1361/14-1, DE 1361/18-1, BIOQIC GRK 2260/1, Radiomics DE 1361/19-1 [428222922] and 20-1 [428223139] in SPP 2177/1), GUIDE-IT (DE 1361/24-1), Berlin University Alliance (GC_SC_PC 27), Berlin Institute of Health (Digital Health Accelerator) Patent Holder: Patent on fractal analysis of perfusion imaging (jointly with Florian Michallek, EPO 2022 EP3350773A1, and USPTO 2021 10,991,109, approved) Other: Hands-on cardiac CT courses (www.ct-kurs.de) Other: Institutional research agreements: Siemens, General Electric, Philips, Canon. Other: M.D. is European Society of Radiology (ESR) Publications Chair (2022-2025); the opinions expressed in this presentation are the author's own and do not represent the view of ESR. Author: Cardiac CT (Springer Nature)
Sotirios Tsogias: Nothing to disclose
Federico Biavati: Nothing to disclose
Maria Bosserdt: Research/Grant Support: Received funding from EU-FP7 Framework Program (DISCHARGE EU FP EC-GA 603266)
Bernhard Föllmer: Nothing to disclose

**Prognostic value of semi-quantitative cCTA scores**
*E. Bruno*, A. Bettinelli, V. Morrone, A. Colombo, C. Gnasso, F. Pisu, D. Vignale, A. Palmisano, A. Esposito; Milan/IT
*(bruno.elisa@hsr.it)*

**Purpose or Learning Objective:** Coronary artery disease (CAD) is a global leading cause of morbidity and mortality, with complex pathogenesis. Coronary computed tomography angiography (cCTA) is a powerful non-invasive tool for diagnosing obstructive CAD. However, most patients have non-obstructive CAD, and risk stratification data are limited. Many cCTA-based risk scores were developed, however with low predictive value and reproducibility. This study aims to develop clinical-imaging models to predict major adverse cardiac events (MACEs) in patients undergoing cCTA for suspected CAD.
**Methods or Background:** Observational, single-center retrospective study including 4096 out of 10104 patients undergoing cCTA between 2016 and 2020. Patients with cardiovascular comorbidities or terminal cancer were excluded. Demographics, cardiovascular risk-factors, and medical history were collected via phone contact and medical records, to calculate known semiquantitative cCTA scores (CAD-RADS, Leiden risk score, Leaman risk score, SSS, SIS, Calcium score). Patients were compared after a minimum 4-year follow-up according to the occurrence of MACEs (cardiovascular death, nonfatal myocardial infarction, all-cause mortality, angina-related hospitalization, late coronary revascularization). Multivariable Cox regression models, adjusted for age and sex, were created using significant clinical variables and one cCTA score.
**Results or Findings:** Among 1933 patients enrolled (65% men, age:63.5±11.6 year-old), 353/1933(18%) had MACE. Patients with MACE had higher rates of hypertension, dyslipidemia, diabetes, and higher cCTA scores(all p<.001). All cCTA scores significantly predicted MACE occurrence in Kaplan-Meier survival analysis(p<.005). Six multivariable models including clinical features (diabetes, dyslipidemia, hypertension) and one cCTA score have been developed: in each model cCTA score was the strongest prognosticator of outcome, with CAD-RADS having the highest HR(2.996, 95%CI 2.374-3.781, p<.001), followed by CACS(2.103, 95%CI 1.646-2.687, p<.001).
**Conclusion:** CCTA scores area all predictors of outcome, in particular CAD-RADS, indicating the highest-grade coronary artery lesion, had the higher Hazard Ratio.
**Limitations:** No prospective data.
**Funding for this study:** None
**Ethics committee - additional information:** Approved by San Raffaele hospital ethics committee (124/2023)
**Author Disclosures:**
Davide Vignale: Nothing to disclose
Alberto Colombo: Nothing to disclose
Antonio Esposito: Nothing to disclose
Elisa Bruno: Nothing to disclose
Anna Palmisano: Nothing to disclose
Francesco Pisu: Nothing to disclose
Chiara Gnasso: Nothing to disclose
Vittorio Morrone: Nothing to disclose
Andrea Bettinelli: Nothing to disclose

**Myocardial delayed enhancement with first-generation dual-source photon-counting detector CT: an image quality comparison across available spectral acquisition modes**
*B. Longere*[1], R. Cusumano[1], C. V. Gkizas[1], A. Rodriguez Musso[1], F. Dubus[1], C. Croisille[2], C. Artaud[1], M. Haidar[1], F. A. Pontana[1]; [1]Lille/FR, [2]Bordeaux/FR
*(benjamin.longere@chu-lille.fr)*

**Purpose or Learning Objective:** To compare the image quality of myocardial delayed enhancement (CT-MDE) obtained by two different tube voltages and three distinct cardiac synchronization modes on a first-generation dual-source photon-counting detector CT (PCD-CT).
**Methods or Background:** Ninety patients (43 women) aged 63 years (54–73y) referred for cardiac CT with CT-MDE were enrolled. CT-MDE acquisition was performed 5min after injection of 90mL of iodine contrast medium (400mgI/mL). Tube voltage was set to either 120 or 140kVp. Current was automatically adjusted to a predetermined image quality level of 50. CT-MDE was acquired using helicoidal retrospective gating (R120; R140), sequential triggering (S120; S140) or prospective high-pitch gating (F120; F140). Triggering was set to an RR delay of 300ms. Signal-to-noise ratio (SNR), contrast-to-noise ratio (CNR) and subjective image quality were assessed on virtual monoenergetic images at 65keV (VMI65) and iodine maps.
**Results or Findings:** High-pitch acquisitions provided the lowest CT dose index (P<0.001) with no differences in body mass index between the 6 groups (P=0.09). No differences were observed in SNR across the six acquisition types on VMI65 (P=0.07) and iodine map (P=0.22). F120 demonstrated a CNR that was equivalent to or better than that of the other acquisitions (VMI65, P=0.01; iodine map, P=0.04). No difference was observed in artifact scores (VMI65, P=0.43; iodine maps, P=0.83). Global subjective image quality provided by F120-derived VMI65 was better than or equivalent to that of other series (P=0.04).
**Conclusion:** High-pitch acquisition at 120kVp provides lower radiation dose without compromising image quality. This acquisition mode should be recommended for the assessment of CT-MDE with PCD-CT.
**Limitations:** It was a single-center study with a limited sample size. The detectability of iodine enhancement of pathological finding was not assessed as it was considered to be outside the scope of this study.
**Funding for this study:** None
**Ethics committee - additional information:** IRB number: CRM-2408-417
**Author Disclosures:**
Benjamin Longere: Nothing to disclose
Raphael Cusumano: Nothing to disclose
Christos Vasileiou Gkizas: Nothing to disclose
François Dubus: Nothing to disclose
Mehdi Haidar: Nothing to disclose
Cedric Croisille: Employee: Siemens Healthineers
Camille Artaud: Nothing to disclose
Aimée Rodriguez Musso: Nothing to disclose
François Ascagne Pontana: Nothing to disclose

**Quantification of Extracellular Volume in Acute Myocarditis Using Dual-Source Photon-Counting Detector CT: A Comparative Analysis with CMR**
*C. V. Gkizas*[1], J. Limousin[1], W. Ben Mansoura[1], B. Longere[1], A. L. Rodriguez Musso[1], C. Croisille[2], F. A. Pontana[1]; [1]Lille/FR, [2]Bordeaux/FR
*(chgkizas@gmail.com)*

**Purpose or Learning Objective:** The aim of this study was to assess the feasibility and accuracy of myocardial late enhancement (LE) scanning for extracellular volume (ECV) quantification with dual-source photon-counting detector computed tomography (PCD-CT) in acute myocarditis.
**Methods or Background:** Patients with clinical suspicion of myocarditis who were referred for coronary CT angiography (CCTA) to exclude CAD were included in this retrospective study. The CCTA protocol using a first-generation PCD-CT, included a systematic LE acquisition. ECV was calculated from the iodine ratio of the myocardium and blood pool on the LE scan. A comprehensive CMR protocol was used as the reference method to confirm myocarditis according to the Lake Louise 2018 criteria. All subjects underwent CCTA and CMR within 24 hours.

**Results or Findings:** 32 patients were included (mean age 36 years; 13 females). The mean dose length product of the LE scan was 96± 32 mGy.cm. The mean global ECV between CCT and CMR did not show significant difference (29.4% ±4.5 vs 30.0 ±4.1, P=0.69). In patients diagnosed with myocarditis confirmed by CMR (n=25), the mean ECV-CT was notably elevated compared to individuals with normal CCT and CMR findings (31.6% ±3.6 vs 25.6% ±3.2, P<0.01). ECV-CT value showed a strong positive correlation with LGE mass (r =0.85; p < 0.001).
**Conclusion:** Calculation of ECV using iodine maps derived from LE cardiac CT images is both feasible and accurate at low radiation dose. PCD-CT offers a promising non-invasive imaging method in the context of acute myocarditis.
**Limitations:** Retrospective, single study
**Funding for this study:** None
**Ethics committee - additional information:** All subjects were informed and provided their consent.
**Author Disclosures:**
Benjamin Longere: Nothing to disclose
Christos Vasileiou Gkizas: Nothing to disclose
Cedric Croisille: Nothing to disclose
Jean Limousin: Nothing to disclose
Aimée Leilen Rodriguez Musso: Nothing to disclose
François Ascagne Pontana: Nothing to disclose
Wissem Ben Mansoura: Nothing to disclose

**16:30-17:30** **Research Stage 1**

### Research Presentation Session: Oncologic Imaging

### RPS 616

### Haematologic malignancies: multimodality imaging

**Moderator**
G. Cowell; Glasgow/UK
*(Gordon.Cowell@ggc.scot.nhs.uk)*

### Prospective assessment of 3T Whole-Body MRI and 18F-FDG PET-CT in diagnosing multiple myeloma and its influence on patient care

*A. Rossi*[1], D. Bezzi[1], D. Diano[1], A. Prochowski Iamurri[1], A. Cattabriga[2], E. Antognoni[1], G. Feliciani[1], P. Caroli[1], C. Cerchione[1]; [1]Meldola/IT, [2]Bologna/IT
*(alice.rossi@irst.emr.it)*

**Purpose or Learning Objective:** This study aims to compare the diagnostic efficacy of Whole Body-Magnetic Resonance Imaging (WB-MRI) and 18F-Fluorodeoxyglucose Positron Emission Tomography (PET-CT) in detecting bone marrow infiltration (BMI) in myeloma patients and assess their impact on patient management.
**Methods or Background:** We prospectively enrolled myeloma patients from October 2020 to January 2024. Within a month, patients underwent 3T WB-MRI (following MY-RADS criteria) and PET-CT to assess BMI, para, and extramedullary disease. Clinical and laboratory data were collected. Two haematologists determined treatment plans using International Myeloma Working Group (IMWG) criteria based on all findings, which were then used to evaluate imaging performance.
**Results or Findings:** The cohort included 137 patients (73 male; mean age, 66 years), with 39 having High Risk-Smoldering Multiple Myeloma (SMM) and 98 with Multiple Myeloma (MM). WB-MRI sensitivity and specificity for BMI in MM were 100% and 97%, respectively, while PET-CT showed 89% sensitivity and 97% specificity (p=0.02). In SMM, BMI-positive patients had higher paraprotein levels (p=0.01); in MM, they had higher paraprotein (p=0.007) and lower hemoglobin (p=0.002). Clinical management changed in 54% of cases based on combined imaging results, with WB-MRI consistent with management changes in 97% compared to 61% for PET-CT (p < 0.001).
**Conclusion:** WB-MRI and PET-CT play key roles in evaluating myeloma patients. WB-MRI demonstrated superior sensitivity in detecting BMI and had a greater influence on therapeutic decision-making.
**Limitations:** No limitations were identified
**Funding for this study:** This study was partly funded by the Italian Ministry of Health for Institutional Research (Ricerca Corrente) within the research line "Innovative therapies, phase I-III clinical trials, and therapeutic strategy trials based on preclinical models, onco-immunological mechanisms, and nano vectors.
**Ethics committee - additional information:** the study was approved by C.E.R.O.M comitato etico della Romagna (AccuMRI trial IRST code 100.15)
**Author Disclosures:**
Paola Caroli: Nothing to disclose
Davide Bezzi: Nothing to disclose
Danila Diano: Nothing to disclose
Claudio Cerchione: Nothing to disclose
Eleonora Antognoni: Nothing to disclose
Arrigo Cattabriga: Nothing to disclose
Alice Rossi: Nothing to disclose
Andrea Prochowski Iamurri: Nothing to disclose
Giacomo Feliciani: Nothing to disclose

### Prognostic value of maximum tumor spread (Dmax) in lymphoma patients treated with CD19-specific CAR-T cell therapy

*M. Winkelmann*, P. Achhammer, V. Blumenberg, K. Rejeski, G. Sheikh, M. Brendel, J. Ricke, M. Subklewe, W. G. Kunz; Munich/DE

**Purpose or Learning Objective:** CD19 specific CAR T-cell therapy (CART) is an effective treatment for relapsed or refractory (r/r) lymphoma. The maximum distance (Dmax) of lymphoma lesions holds potential as imaging biomarker in lymphoma treated with conventional therapies, but has not been studied in context of CART. We evaluated Dmax at baseline imaging as a prognostic tool for assessment of metabolic and overall response, progression-free survival (PFS) and overall survival (OS).
**Methods or Background:** Consecutive r/r lymphoma patients with (PET/)CT at baseline before CART were included. Dmax was measured in cm at BL. Patients were divided into three groups according to Dmax: low, intermediate and high. The sum of product diameters (SPD) according to Lugano criteria was used to represent tumor burden (TB). Overall response according to Lugano criteria and Deauville score were determined at follow-up imaging.
**Results or Findings:** 103 patients were included. Median baseline Dmax was 40.0 cm (IQR: 16.4 – 70.3 cm). Median TB was significantly higher in the intermediate and high risk group compared to the low risk group (p=0.005). Intermediate and high risk group showed significantly higher Ann Arbor stages (p<0.001). The survival analysis revealed a significantly (p=0.030) shorter PFS in the high-risk group compared to the other patients (91 vs 364 days), but no relevant differences in OS (p=0.151). In addition, no significant differences in Deauville score and ORR were detected.
**Conclusion:** Patients with high Dmax showed a shorter PFS, but no significant differences in OS. Dmax as an interval-scaled parameter represents a useful alternative to the Ann Arbor classification.
**Limitations:** Single center study, limited number of subjects. Few patients were excluded because of no measurable disease. Some patients had only CT without PET at FU, with a possible redistribution of Deauville score among Dmax-based groups.
**Funding for this study:** The work was supported by funding from the research program “Förderung für Forschung und Lehre (FöFoLe) project number 1147” of the Medical Faculty of Ludwig Maximilian University (LMU) Munich and the Bavarian Cancer Research Center (BZKF)
**Ethics committee - additional information:** All medical records and imaging studies underwent review with approval from the LMU Munich Institutional Review Board (LMU Ethics Committee, project number 19-817).
**Author Disclosures:**
Marion Subklewe: Speaker: Amgen; Astra Zeneca; BMS/Celgene; GSK; Incyte Biosciences; Janssen; Novartis; Pfizer; Seattle Genetics; Takeda Consultant: Aven Cell; CDR-Life; GSK; Incyte Biosciences; Janssen; Miltenyi Biotec; Molecular Partners Novartis; Pfizer; Takeda Research/Grant Support: Amgen; BMS/Celgene; Gilead; Incyte Biosciences; Janssen; Miltenyi Biotec; Morphosys; Novartis; Roche; Seattle Genetics; Takeda
Viktoria Blumenberg: Consultant: Kite/Gilead Research/Grant Support: Janssen; BMS/Celgene; Novartis; Takeda; Roche
Philipp Achhammer: Nothing to disclose
Michael Winkelmann: Nothing to disclose
Kai Rejeski: Research/Grant Support: Kite/Gilead; Novartis Consultant: Kite/Gilead; MBS/Celgene
Wolfgang Gerhard Kunz: Advisory Board: Bristol Myers Squibb; Boehringer Ingelheim; mintMedical; Need, Inc.
Gabriel Sheikh: Nothing to disclose
Matthias Brendel: Nothing to disclose
Jens Ricke: Nothing to disclose

### Early Whole-Body MRI as a Predictor of long-term Metabolic Response in Large B-Cell Lymphoma Patients following CAR T-Cell Therapy

*C. Neelsen*[1], C. Sachpekidis[1], J. M. E. Jende[1], R. Gnirs[1], F. Kurz[2], P. Dreger[1], A. Dimitrakopoulou-Strauss[1], H-P. Schlemmer[1]; [1]Heidelberg/DE, [2]Geneva/CH

**Purpose or Learning Objective:** To evaluate the utility of early whole-body MRI (wbMRI) for the prediction of long-term metabolic response in patients with large B-cell lymphoma (LBCL) following chimeric antigen receptor T-cell therapy (CARTT).

**Methods or Background:** In this prospective, IRB-approved study, we assessed 9 LBCL patients with target lesions identified on baseline wbMRI according to adapted Response Evaluation Criteria in Lymphoma (RECIL), who were fit to undergo wbMRI within two weeks (mean 11 days) following CARTT in an outpatient setting. Complete remission was defined as the absence of measurable disease and any lesions on diffusion-weighted imaging. Early wbMRI findings were compared with PET-CT results at 3-month follow-up, with metabolic responses classified according to the Lugano criteria.
**Results or Findings:** At the 3-month PET-CT follow-up 4 patients showed a complete metabolic response (CMR), 2 patients had a partial metabolic response (PMR) and 3 patients demonstrated progressive disease (PD). Of the 4 patients with CMR, 2 had already achieved complete remission on the early wbMRI, while the other 2 patients had a minor and partial response. The 2 patients with PMR exhibited minor responses on early wbMRI and finally of the 3 patients with PD, one had a partial and two had minor responses.
**Conclusion:** All patients demonstrated some degree of response on early wbMRI within two weeks after CARTT. Early complete remission appeared to be a predictor of long-term metabolic response. However, early wbMRI was inconclusive in patients with initial minor or partial responses, as these patients may still achieve complete metabolic remission or progress over time.
**Limitations:** The lymphodepleting chemotherapy administered prior to the CAR T-cell infusion complicates the differentiation of the specific effects of chemotherapy versus the CAR T-cell therapy itself.
**Funding for this study:** No funding was provided for this study.
**Ethics committee - additional information:** The study was approved by the institutional review board (S-950/2021) and written informed consent was obtained from all participants.
**Author Disclosures:**
Christos Sachpekidis: Nothing to disclose
Peter Dreger: Nothing to disclose
Regula Gnirs: Nothing to disclose
Christian Neelsen: Nothing to disclose
Johann Malte Enno Jende: Nothing to disclose
Felix Kurz: Nothing to disclose
Antonia Dimitrakopoulou-Strauss: Nothing to disclose
Heinz-Peter Schlemmer: Nothing to disclose

**Diagnostic value Whole-body Magnetic resonance imaging (WBMRI) short protocols can be useful in Multiple Myeloma patients**
*C. R. G. L. O. M. Talei Franzesi*, C. Maino, P. N. Franco, D. Ippolito, R. Corso; Milan/IT
*(ctfdoc@hotmail.com)*

**Purpose or Learning Objective:** To compare the effectiveness and accuracy of whole-body magnetic resonance imaging (WBMRI) short protocols for the overall assessment of bone marrow involvement in patients with multiple myeloma (MM), in comparison with standard whole-body MRI protocol
**Methods or Background:** Sixty-four patients with biopsy-proven MM, who underwent an WBMRI with full body coverage (from vertex to feet) were retrospectively enrolled. WBMRI images were independently evaluated, by two expert radiologists. After identifying the infiltration pattern (normal, focal, diffuse and combined), the whole skeleton was divided into six anatomic districts: skull, spine, sternum and ribs, upper limbs, pelvis and proximal two-third of femur, remaining parts of lower limbs, and patients were grouped according to number(< 5, 5-20, and > 20) and location of the lesions
**Results or Findings:** Most of patients showed a focal (59%) and combined (33%) infiltration patterns with lytic lesions predominantly distributed in the spine (82%) and pelvis (67%). Locations less frequently involved by focal bone lesions were skull and lower limbs (12%, respectively). Excluding both the anatomic regions mentioned before from the standard MRI protocol, a short MRI protocol with a shorter execution time (saving about 14 minutes) could be obtained, maintaining a good sensitivity (89.9%), specificity (66.7%) and diagnostic accuracy (AUROC=0.881; 95%CIs: 0.797-0.965)
**Conclusion:** MRI short protocols could be proposed as an effective and reliable approach to reduce the examination time, preserving a high diagnostic accuracy and can be more focused on the main involved districts
**Limitations:** None
**Funding for this study:** None
**Ethics committee - additional information:** None
**Author Disclosures:**
Cesare Maino: Nothing to disclose
Cammillo Roberto Giovanni Leopoldo Oreste Massimiliano Talei Franzesi: Nothing to disclose
Rocco Corso: Nothing to disclose
Paolo Niccolò Franco: Nothing to disclose
Davide Ippolito: Nothing to disclose

**Dual-Vessel Microcirculation Imaging in Differentiation of B cell and T cell subtype in intranodal Non-Hodgkin Lymphoma Using Super-Resolution Ultrasound: An Exploring Study**
*Y. Dong*; Shanghai/CN
*(dyj11584@rjh.com.cn)*

**Purpose or Learning Objective:** To explore the diagnostic performance of the super-resolution ultrasound (SRUS) imaging in dual-vessel systems, i.e., the microvascular system and the microlymphatic system, for predicting B cell and T cell subtypes in intranodal NHL.
**Methods or Background:** Forty-two patients with intranodal NHL were included in this prospective study. All patients underwent dual-vessel system SRUS imaging via intravenous and intra-lymph node routes. SRUS parameters such as vessel density, vessel ratio, vessel complexity level, diameter, distance, tortuosity, and flow velocity were measured for the microvascular and microlymphatic circulations. Principal component analysis (PCA) was utilized to interpret parameters, and a regression model was developed to predict NHL subtypes. The areas under the receiver operating characteristic curve (AUC), sensitivity, and specificity were calculated.
**Results or Findings:** Among the 42 patients, 35 were diagnosed with B cell NHL and 7 with T cell NHL. Sixty parameters from dual-vessel SRUS image analysis were obtained for each case. PCA extracted six principal components accounting for 93.1% of the total variance. The regression model utilizing these components to distinguish between B-cell and T-cell lymphomas achieved an AUC of 0.927 (95% CI: 0.841-1.000), a sensitivity of 85.7% (95% CI: 42.1%-99.6%), and a specificity of 94.3% (95% CI: 80.8%-99.3%).
**Conclusion:** Dual-vessel SRUS imaging, in conjunction with quantitative analysis, could effectively differentiate between B-cell and T-cell NHL, offering a non-invasive diagnostic alternative.
**Limitations:** First, due to the relatively low prevalence of lymphoma in the general population, the sample size is relatively small. Second, the methodology of microlymphatic SRUS imaging is confined primarily to intact lymph nodes. In cases of 'bulky mass' lymphomas, where there is extensive disruption of the lymph node architecture, as well as in NHL presenting within the trunk region, this approach might not be appropriate.
**Funding for this study:** No.
**Ethics committee - additional information:** Ruijin Hospital Clinical Research Center Shanghai Jiaotong University, School of Medicine, Ethic No. 20240116021828990.
**Author Disclosures:**
Yijie Dong: Nothing to disclose

**16:30-17:30** **Research Stage 2**

### Research Presentation Session: Imaging Informatics and Artificial Intelligence

### RPS 605

### Artificial intelligence in cardiovascular imaging

**Moderator**
T. Leiner; Rochester, MN/US
*(leiner.tim@mayo.edu)*
**Author Disclosures:**
Tim Leiner: Research Grant/Support: Philips Healthcare; Other: Editor-in-Chief, Journal of Cardiovascular Magnetic Resonance (JCMR)

**CT Deep learning AI quantified fibrosis predicts prognosis in Pulmonary Hypertension associated with Chronic Lung Disease**
*K. Dwivedi*, M. Sharkey, S. Alabed, A. Maiter, C. S. Johns, S. Rajaram, R. Condliffe, D. Kiely, A. J. Swift; Sheffield/UK
*(k.dwivedi@sheffield.ac.uk)*

**Purpose or Learning Objective:** Pulmonary Hypertension associated with Chronic Obstructive Pulmonary Disease (PH-COPD) is a heterogenous condition, with a spectrum of predominantly emphysema and some overlapping fibrosis. All patients undergo CT, but it is not used for prognostication. The study aim is to investigate the prognostic value of an AI model that quantifies the percentage of fibrosis on baseline CT, compared to radiological assessment.
**Methods or Background:** PH-COPD patients with baseline CT between 2001-19 were identified from the ASPIRE registry. A validated in-house PH specific deep-learning model was run and provided percentage of fibrosis by quantifying ground glass change, ground glass with reticulation, and honeycombing. Scans were scored as none/mild/moderate/severe fibrosis by sub-specialist radiologists. Cases with mean pulmonary arterial pressure ≥ 35 mmHg were classified as severe PH-COPD, and fibrosis was grouped with a threshold of 3%. Scaled cox regression and Kaplan Meier survival analysis was performed.
**Results or Findings:** 157 PH-CLD patients (113 severe PH-COPD) were included. AI quantified fibrosis % was a significant predictor of mortality (HR 1.46, p<0.001) .There was a significant difference (p=0.001) in survival between patients with more and less than 3% fibrosis. One and five-year survival was 84% and 35% respectively in those with <3% fibrosis and 63% and 18% respectively in those with ≥3% fibrosis. Radiologist scored mild (HR 2.05, p=0.36) and moderate (HR 2.82, p=0.045) fibrosis was a significant predictor, but not severe fibrosis. In severe PH-COPD, radiological scoring was not a significant predictor at any level, but AI fibrosis% was a significant predictor (HR 1.37, p<0.001).
**Conclusion:** CT Deep learning AI model quantified fibrosis is prognostic in predicting survival and treatment response in PH-COPD and provides additional value over radiological assessment in severe PH-COPD.
**Limitations:** Single registry analysis, but imaging from 21 hospitals.
**Funding for this study:** Research conducted during post funded by UK National Institute for Health and Care Research
**Ethics committee - additional information:** Ethical approval was granted by the Institutional Review Board and approved by the National Research Ethics Service (16/YH/0352).
**Author Disclosures:**
David Kiely: Consultant: Ferrer, MSD, Janssen, United Therapeutics, Acceleron
Andrew J. Swift: Consultant: Janssen pharamceuticals Grant Recipient: Wellcome Trust, National Institute for Health and Care Research and Janssen pharamceuticals
Ahmed Maiter: Nothing to disclose
Chris S. Johns: Nothing to disclose
Michael Sharkey: Grant Recipient: Wellcome Trust
Samer Alabed: Advisory Board: Royal College of Radiologists AI Working Group Grant Recipient: Wellcome Trust and National Institute for Health and Care Research
Smitha Rajaram: Nothing to disclose
Robin Condliffe: Consultant: Janssen pharamceuticals and MSD
Krit Dwivedi: Advisory Board: Royal College of Radiologists AI Working Group member Grant Recipient: Wellcome Trust and National Institute for Health and Care Research

### External Validation of a Deep Learning Cardiac Metal Artifact Reduction Algorithm (DL-C-MAR) to reduce Metal Artifacts of Transcatheter Aortic Valves in CT: a retrospective cohort and phantom study

*I. H. T. Khargi*[1], M. Selles[1], N. Huber[2], J. Browne[2], B. Kietselaer[2], T. Leiner[2], M. F. Boomsma[1]; [1]Zwolle/NL, [2]Rochester, MN/US
*(indirakhargi@gmail.com)*

**Purpose or Learning Objective:** To assess the performance of a novel deep learning-based cardiac metal artifact reduction algorithm (DL-C-MAR) in a retrospective comparison with unedited conventional computed tomography angiograms (CTAs) of transcatheter aortic valve implantation (TAVI) valves and phantom experiments.
**Methods or Background:** DL-C-MAR was trained using multiple simulated metal implants and artifacts in 1000 CTAs. Performance of DL-C-MAR was quantitatively and qualitatively investigated in 50 TAVI patients and compared to unedited conventional CTAs. To quantitatively assess image quality, noise, contrast-to-noise ratio (CNR), artifact index (AI), and artifact volume were calculated. Diameters of the valve struts were also measured. Images were qualitatively rated on overall image quality, extent of metal artifacts and valve leaflet definition by two readers on a four-point scale. Phantom experiments were conducted using four different size steel cylinders. Diameters of the cylinders were measured by two readers and compared to their conventional counterparts and the ground truth. All images were visually screened for presence of hallucinations.
**Results or Findings:** In the CTAs, DL-C-MAR resulted in a higher CNR (9.1±5.8 vs. 7.9±4.8), and lower noise (57.2±33.9 vs. 82.1±54.0), AI (53.0±36.0 vs. 75.7±55.9), and artifact volume (0.02±0.12mL vs. 0.06±0.42mL) compared to unedited conventional CTAs (all p<0.001). The strut diameter also decreased after DL-C-MAR (1.52±0.26mm vs. 2.05±0.46mm, p=0.005). Initial results from the qualitative analysis suggest increased valve leaflet definition and decreased metal artifact severity after DL-C-MAR. In the phantom scans, DL-C-MAR decreased cylinder diameter by 7-67% (p<0.001), bringing them closer to the ground truth. No hallucinations were observed.
**Conclusion:** DL-C-MAR increases image quality and reduces metal artifacts in CTAs after TAVI implantation and does not seem to hallucinate on clinical or phantom images.
**Limitations:** This study did not include impact on clinical decision-making outcomes.
**Funding for this study:** No funding was received for this study
**Ethics committee - additional information:** This study was reviewed and approved as exempt with waived informed consent. Reference no.: RPR - 2024-00000086
**Author Disclosures:**
Mark Selles: Nothing to disclose
Tim Leiner: Nothing to disclose
Nathan Huber: Employee: Philips Healthcare
Bas Kietselaer: Nothing to disclose
Indira Hélène Theodora Khargi: Nothing to disclose
Jacinta Browne: Nothing to disclose
Martijn Franklin Boomsma: Nothing to disclose

### Diagnostic confidence in coronary stent evaluation using coronary CT angiography. Comparison of Deep Learning Reconstruction, Hybrid Iterative Reconstruction and Model Based Iterative Reconstruction

*M. Finazzo*[1], M. M. Lagana[2], F. Graziano[3], F. Pinto[2], C. Duranti[1], F. Finazzo[1]; [1]Palermo/IT, [2]Milan/IT, [3]Monza/IT
*(mariofinazzo67@gmail.com)*

**Purpose or Learning Objective:** The assessment of coronary stents using Coronary CT Angiography (CCTA) can be challenging. Deep Learning Reconstruction (DLR) is an innovative CT image reconstruction method that reduces noise, enhancing image quality. This study aims to evaluate whether DLR improves diagnostic confidence in coronary stent evaluation using CCTA images, compared to hybrid iterative reconstruction (HIR) and model-based iterative reconstruction (MBIR).
**Methods or Background:** CCTA images of 20 patients with 35 stents were evaluated retrospectively using three reconstruction methods: HIR, MBIR, and DLR. All examinations were conducted using a 320-row whole-heart CT scanner. The diagnostic confidence of the images obtained with each reconstruction method was evaluated using a Likert score (1=non-diagnostic, 2=poor, 3=acceptable, 4=good, 5=excellent). Stents were divided into proximal and distal according to their location. Stents located in the proximal and intermediate segments of the coronary arteries were considered proximal; stents situated in the distal segments of the main coronary arteries and side branches were considered distal. A cumulative liked mixed model was created in Rstudio version 4.3.1 to examine the differences across reconstruction methods while accounting for the stent position, to explore its potential effect on diagnostic confidence. Post-hoc comparisons were conducted, and the p-values were adjusted using the Tukey method.
**Results or Findings:** The reconstruction method had a significant impact, irrespective of stent position. Likert scores were significantly higher for DLR images compared to those reconstructed using HIR and MBIR (p<0.001), with no significant difference between HIR and MBIR (p=0.957).
**Conclusion:** DLR provided the best diagnostic confidence and significantly enhanced the evaluation of coronary stents. As a further development, Super Resolution DLR, a new reconstruction algorithm, could improve spatial resolution, thereby increasing diagnostic confidence in coronary stents.
**Limitations:** Limited number of patients.
Qualitative analysis only
**Funding for this study:** No funding
**Ethics committee - additional information:** It's a non-pharmacological retrospective observational study which have been approved by the local ethics committee
**Author Disclosures:**
Maria Marcella Lagana: Investigator: Canon Medical Systems Investigator
Mario Finazzo: Nothing to disclose
Francesca Pinto: Investigator: Canon Medical Systems Clinical Application Specialist
Francesca Graziano: Nothing to disclose
Cristiana Duranti: Nothing to disclose
Francesca Finazzo: Nothing to disclose

### Comparing the performance of Large Language Models for automatic CAD-RADS 2.0 classification from cardiac-CT reports

*P. Arnold*, M. Russe, E. Kotter, M. T. Hagar; Freiburg/DE
*(philipp.arnold@uniklinik-freiburg.de)*

**Purpose or Learning Objective:** The Coronary Artery Disease-Reporting and Data System (CAD-RADS) 2.0 offers standardized guidelines for interpreting coronary artery disease in cardiac computed tomography (CT). Accurate and consistent CAD-RADS 2.0 scoring is crucial for comprehensive disease characterization and clinical decision-making. This study investigates the capability of large language models (LLMs) to autonomously generate CAD-RADS 2.0 scores from cardiac CT reports.
**Methods or Background:** A dataset of 200 synthetic cardiac CT reports was created to evaluate the performance of several state-of-the-art LLMs in generating CAD-RADS 2.0 scores via in-context learning. The tested models included GPT-3.5, GPT-4o, Mistral 7b, Mixtral 8x7b, LLama3 8b, LLama3 8b with a 64k context length, and LLama3 70b. The generated scores from each model were compared to the ground truth, which was provided by an independent committee of two board-certified cardiothoracic radiologists.
**Results or Findings:** The GPT-4o model and Llama3 70b achieved the highest accuracy in generating full CAD-RADS 2.0 scores including all modifiers, with a performance rate of 93% and 92.5% respectively, followed by Mixtral 8x7b with 78%. In contrast, less advanced LLMs, such as Mistral 7b and GPT-3.5 provided poor performance (16%). Llama3 8b demonstrated intermediate results, with an accuracy of 41.5%.
**Conclusion:** Advanced LLMs are capable of generating autonomously CAD-RADS 2.0 scores for cardiac CT reports with excellent accuracy, potentially enhancing both the efficiency and consistency of cardiac CT report evaluations. Open-source models not only deliver competitive accuracy but also present the benefit of local hosting, mitigating concerns around data privacy.
**Limitations:** To ensure data privacy and avoid ethical concerns, this study was conducted using synthetically generated cardiac CT reports. Even though these were deemed indistinguishable from real patient reports, further research is needed to validate LLM performance in real-world settings.
**Funding for this study:** Hans A. Krebs Medical Scientist Program (Uniklinikum Freiburg) German Research Foundation (DFG) - SFB 1597 - 499552394
**Ethics committee - additional information:** None
**Author Disclosures:**
Muhammad Taha Hagar: Nothing to disclose
Maximilian Russe: Nothing to disclose
Philipp Arnold: Nothing to disclose
Elmar Kotter: Nothing to disclose

**Multi-stage deep learning architecture for carotid artery segmentation and stenosis degree evaluation: a comparative study with DSA**
*Z. Zheng*, X. Cao, W. Liu; Shanghai/CN
*(23110860044@m.fudan.edu.cn)*

**Purpose or Learning Objective:** HR-MRI provided a non-invasive and radiation-free method for assessing atherosclerosis, with strong advantages for vessel wall visualization. However, efficient segmentation and stenosis degree evaluation remained a challenging dilemma that is both labor- and time-consuming and susceptible to interobserver variability. Thus, a multi-stage deep learning architecture was developed to address above issues.
**Methods or Background:** The method contained three modules: artery localization, automatic segmentation, and stenosis degree evaluation modules. The 422 scans were retrospectively collected from two tertiary hospitals between 2018 and 2023 with a training-validation set (372 patients, 545 lesions) and an independent test set (50 patients, 96 lesions). An external validation set (26 patients, 42 lesions) was collected prospectively between 2023 and 2024. Subsequently, the artery segmentation and stenosis degree evaluation were compared against the ground truth, which was established by consensus among three radiologists and derived from diagnostic results obtained via DSA.
**Results or Findings:** The results showed outstanding performance with high DSC, IOU, and low RVE, ASSD, and HD95. The concordance correlation coefficient (CCC) was 0.985(95% CI: 0.981-0.987), 0.979(95% CI: 0.963-0.984), and 0.963(95% CI: 0.944-0.992) for volumes of artery on all datasets. Stenosis degree was evaluated on the NASCET achieved Acc of 0.8750, 0.8571, AUC of 0.89, 0.80, Sens of 0.8611, 0.9333, and Spec of 0.9167, 0.6667 on the independent test and external validation sets, respectively.
**Conclusion:** The method achieved no less accuracy than manual segmentation by physicians and maintained a high consistency with the DSA diagnostic criteria. In addition, by shortening diagnostic time and minimizing inter-observer variability, it offered an efficient intelligent aid in clinical practice.
**Limitations:** The method performed in multi-stage may take up a large amount of computational resources and modifications to the architecture are required to optimize the inference speed.
**Funding for this study:** This work has received funding from the National Natural Science Foundation of China (82402393, 82102132, 8237071280), the Science and Technology Commission of Shanghai Municipality (20S31904300, 22TS1400900, 23S31904100, 22ZR1409500) and the Greater Bay Area Institute of Precision Medicine (Guangzhou) (KCH2310094).
**Ethics committee - additional information:** All patients or their guardians gave informed consent to use their anonymized MRI images and clinical data for research purposes. Since all data were obtained in the course of daily work, the Ethics Committee waived the need for informed consent.
**Author Disclosures:**
Zhiji Zheng: Nothing to disclose
Wanchen Liu: Nothing to disclose
Xin Cao: Nothing to disclose

**AI-driven joint segmentation of myocardium, scar, and microvascular obstruction in bright-blood late gadolinium enhancement cardiac magnetic resonance imaging**
*B. Durand*, V. De Villedon De Naide, T. Génisson, M. Stuber, A. Bustin, H. Cochet; Bordeaux/FR
*(baptisted55@gmail.com)*

**Purpose or Learning Objective:** develop and test an AI-driven deep learning model for joint segmentation of healthy myocardium, scar tissue, and microvascular obstruction (MVO) in cardiac MRI using bright-blood phase-sensitive inversion recovery (PSIR) imaging.
**Methods or Background:** Current methods for scar and MVO quantification in PSIR imaging are manual or semi-automated, time-consuming, and prone to errors and variability. Using a nnUNET architecture, the model was trained on 50 PSIR exams with suspected ischemic heart disease and evaluated on a test set of 20 cases. Data augmentations were applied, and manual segmentations by radiologists were used for comparison. To maximize performance, a joint segmentation approach was employed, and both magnitude and phase maps were used together.
**Results or Findings:** The AI model demonstrated excellent performance despite only 50 exams in training, in segmenting healthy myocardium (median Dice score 0.96) and good results for scar segmentation (median Dice score 0.75). MVO detection was successful in 2 out of 3 cases. Inference time was under 5 seconds per exam, and no false positives were identified outside the myocardium.
**Conclusion:** AI-driven approach showed robust segmentation of myocardium and scar tissue, with promising results in MVO detection. It could streamline clinical workflows for myocardial infarction assessment by reducing time-counsuming manual segmentations.
**Limitations:** The model was trained on a small dataset from post-ischemic patients, which limits its generalizability to other cardiac conditions such as hypertrophic cardiomyopathy or infiltrative diseases, where scarring patterns differ. We plan to expand the training population to improve the model's performance in non-ischemic cardiomyopathy.
**Funding for this study:** This research was supported by funding from the French National Research Agency under grant agreement ANR-22-CPJ2-0009-01, and from the European Research Council (ERC) grant "SMHEART" under the European Union’s Horizon 2020 research and innovation programme (grant agreement No101076351).
**Ethics committee - additional information:** The study was approved by the Biomedical Research Ethics Committee and all participants provided informed consent for participation.
**Author Disclosures:**
Victor De Villedon De Naide: Nothing to disclose
Aurelien Bustin: Nothing to disclose
Hubert Cochet: Nothing to disclose
Thaïs Génisson: Nothing to disclose
Baptiste Durand: Nothing to disclose
Matthias Stuber: Nothing to disclose

**16:30-17:30** **Research Stage 3**

### Research Presentation Session: Physics in Medical Imaging

### RPS 613

### MRI spinning for development and sustainability

**Moderator**
T. G. Maris; Iraklion/GR
*(tmaris@med.uoc.gr)*

**Revisiting TE selection for T2-weighted spin-echo MRI of the prostate**
*S. J. Riederer*[1], R. Pabi[1], E. Borisch[1], A. Froemming[1], A. Kawashima[2], N. Takahashi[1]; [1]Rochester, MN/US, [2]Phoenix, AZ/US
*(riederer@mayo.edu)*

**Purpose or Learning Objective:** To study if the long echo trains of fast-spin-echo (FSE) prostate T2-weighted imaging (T2-WI) cause the optimum echo time (TE) for distinguishing normal peripheral zone (PZ) vs. malignancy to deviate from the optimum TE chosen based on standard T2 decay.
**Methods or Background:** All work was done at 3 Tesla. Experiments were done using a standard (NIST) phantom containing ten vials with known T2 relaxation times. Vials 5 (T2=133.3 msec) and 6 (96.9 msec) were analyzed, having values closest to literature-taken T2 measurements in normal PZ (125 to 150 msec) and PZ malignancy and normal transition zone (TZ) (75 to 105 msec). The phantom was imaged using conventional spin-echo (TE 97, 113, 129, 153 msec; scan time 20 min for each) and a clinical T2-WI sequence (echo-train-length 21; TE-EFF 104, 114, 135, 145 msec; scan time 2:30 for each). 30 consecutive subjects with suspected prostate cancer were imaged using the clinical T2-WI sequence at both TE-EFF 100 and TE-EFF 150. Results were compared visually for relative contrast of PZ to TZ and any suspected lesions.
**Results or Findings:** Contrast-to-noise ratio (CNR) between Vials 5 and 6 for conventional spin-echo peaked at TE=125 msec, consistent with theory. However, CNR between the vials for the long-ETL T2-WI sequence was highest at TE-EFF 145 msec, 10% higher vs. 104 msec. In 18 of 30 patient studies the TE-EFF 150 series had superior contrast vs. TE-EFF 100, inferior in 2/30, and equivalent in 10/30.
**Conclusion:** Fast-spin-echo acquisition in prostate T2-WI artifactually prolongs the apparent T2 relaxation, causing the optimum echo times for distinguishing normal from malignant tissue to be higher than that predicted assuming tabulated T2 values. TE-EFF 150 consistently provides improved contrast vs. TE-EFF 100 msec.
**Limitations:** Limited number of subjects
**Funding for this study:** This work was funded by NIH.
**Ethics committee - additional information:** Informed consent was provided by all human subjects.
**Author Disclosures:**
Adam Froemming: Nothing to disclose
Stephen J. Riederer: Nothing to disclose
Eric Borisch: Nothing to disclose
Naoki Takahashi: Nothing to disclose
Ronard Pabi: Nothing to disclose
Akira Kawashima: Nothing to disclose

**Comparison Between Conventional and Compressed SENSE Sequences on MRI Brain in Paediatric Population**
*I. S. Shah*, P. C. P. Joshi, V. Jahanvi; Pune/IN
*(ishshah04@gmail.com)*

**Purpose or Learning Objective:** To compare the quality and image acquisition time between conventional and Compressed SENSE sequences in brain magnetic resonance imaging (MRI) in paediatric population.
**Methods or Background:** Thirty children (below the age of 18 years) undergoing MRI brain were included in this study. In addition to the routine sequences, one Compressed SENSE sequence was added. 2D - T1, T2, and FLAIR axial sequences were acquired for brain using conventional and Compressed SENSE techniques. One of each sequence was acquired in 10 patients undergoing an MRI brain study on a 3T MRI using coil 32 channel coil for adults and pediatric 8ch head coil for neonates. Two consultant radiologists (with 35 years and 5 years experience in radiology) independently scored the image quality using the 5-point Likert scale based on resolution, visualization of anatomical regions, grey-white matter differentiation, sharpness of the image and artefacts. The subjective criteria details for image quality as per the 5-point Likert scale were: non-diagnostic (1), poor (2), moderate (3), good (4) and excellent (5).
**Results or Findings:** The time reduction achieved with 2D T1 at 2 reduction factor were 60 seconds(24%), with 2D T2 at reduction factor of 2.2 66 seconds(47.83%) and with 2D FLAIR at reduction factor 2 66 seconds(40%). Inter-rater agreement for overall diagnostic confidence was rated higher for Compressed SENSE (k – 0.632) than conventional (k – 0.464). Nonsignificant statistical difference was found regarding image quality and image contrast ratio between both techniques.
**Conclusion:** Compressed SENSE has potential in reducing the image acquisition time without compromising the image quality and diagnostic confidence. Motion artefacts are also reduced with reduction in time with the use of Compressed SENSE sequence.
**Limitations:** A small sample size.
**Funding for this study:** None
**Ethics committee - additional information:** Institutional ethics committee approval was obtained
**Author Disclosures:**
Priscilla Col Priscilla Joshi: Nothing to disclose
Vandana Jahanvi: Nothing to disclose
Isha Sandip Shah: Nothing to disclose

**Comparison and optimization of deep learning enhanced 2D ATPw-CEST MRI at 1.5 Tesla and 3 Tesla: A clinically relevant phantom study**
*L. Wei*[1], A. Volk[1], S. Campana Tremblay[2], J. Poujol[3], S. Ammari[1], G. Garcia[1], C. Balleyguier[1], N. Lassau[1], F. Bidault[1]; [1]Villejuif/FR, [2]La Ciotat/FR, [3]Buc/FR
*(lecong.wei@gustaveroussy.fr)*

**Purpose or Learning Objective:** The purpose was to compare the CEST effect at 1.5T and 3T on high resolution 2D images provided by a deep-learning algorithm. In addition, the effect of saturation offset number reduction combined with B0 Mapping on MTRasym values was investigated, in order to minimize acquisition time for clinical use.
**Methods or Background:** The phantom consisted of 12 tubes filled with BSA at pH 7 for 3 different physiological concentrations, at 37 °C. Acquisitions were performed on 1.5T (GE Artist) and 3T (GE Signa Premier) MR scanners. CEST data were acquired with 2D SSFSE using the AIR Recon Deep Learning option (ARDL) for image reconstruction, CW saturation was used, with 61 offsets. SNR was compared with previously acquired data without ARDL. B0 correction was performed by using the chemical shifts of Z-spectra minima, and by using the 2D B0 Mapping GRE sequence. MTRasym was compared for different offset numbers ranging from 6 to 61 using B0 Map GRE.
**Results or Findings:** 2D CEST SSFSE using ARDL had better SNR compared to the sequence without ARDL at 1.5T and 3T. For the two B0 correction methods, MTRasym values were similar and increased with protein concentration at 1.5T and 3T. For $\Delta B0<0.3$ppm, MTRasym variations were small for all offset numbers (max 6%). For $\Delta B0>0.5$ppm, MTRasym were similar for 16 offsets or more.
**Conclusion:** This study provided a comparison of 2D CEST SSFSE at 1.5T and 3T on a phantom carried out during the same imaging session. The findings open up the prospect of high-resolution time efficient APTw-CEST clinical MRI at 1.5T.
**Limitations:** However, the results are preliminary, hence repeatability studies and proof of concept in patients will be considered.
**Funding for this study:** This material is based upon work supported by the ANRT with a CIFRE fellowship granted to Lecong Wei.
**Ethics committee - additional information:** Not applicable
**Author Disclosures:**
Gabriel Garcia: Nothing to disclose
François Bidault: Nothing to disclose
Corinne Balleyguier: Nothing to disclose
Lecong Wei: Employee: Olea Medical, La Ciotat, France Research/Grant Support: This material is based upon work supported by the ANRT with a CIFRE fellowship granted to Lecong Wei.
Samy Ammari: Nothing to disclose
Nathalie Lassau: Nothing to disclose
Sophie Campana Tremblay: Employee: Olea Medical
Julie Poujol: Employee: GE HealthCare
Andreas Volk: Nothing to disclose

**Can we measure extreme brain iron content with Quantitative Susceptibility Mapping?**
*C. Birkl*[1], M. Panzer[1], C. Kames[2], A. Rauscher[2], B. Glodny[1], E. R. R. Gizewski[1], H. Zoller[1]; [1]Innsbruck/AT, [2]Vancouver, BC/CA
*(christoph.birkl@i-med.ac.at)*

**Purpose or Learning Objective:** Aceruloplasminemia (ACP) is a rare autosomal recessive disorder characterized by progressive iron accumulation in multiple organs, including the brain, liver, and pancreas. Magnetic Resonance Imaging (MRI) is commonly used to detect iron overload, with Quantitative Susceptibility Mapping (QSM) emerging as a promising method for assessing brain iron levels. Despite its potential, QSM faces challenges such as susceptibility artifacts and a lack of standardization. This prospective study aimed to evaluate the performance of different QSM algorithms in measuring brain iron in patients with severe iron overload, compared to healthy controls.
**Methods or Background:** QSM images were acquired using a 3D multi-echo gradient echo sequence in three patients with ACP and three healthy controls. We evaluated six QSM algorithms: (I) Fast Nonlinear Susceptibility Inversion (FANSI), (II) Improved Sparse Linear Equation and Least-Squares (iLSQR), (III) Morphology-Enabled Dipole Inversion (MEDI), (IV) Streaking Artifact Reduction (STAR) QSM with Rapid Open-source Minimum Spanning Tree (ROMEO) phase unwrapping, (V) STAR QSM with Laplacian phase unwrapping, and (VI) Multi-Echo Rapid Two-Step (MERTS) QSM. Regional susceptibility values were analyzed in the caudate nucleus, putamen, globus pallidus, and thalamus.
**Results or Findings:** We observed significant variability in susceptibility values across the different algorithms for patients with ACP. Among the algorithms tested, only one showed consistently elevated susceptibility values in the globus pallidus of ACP patients compared to healthy controls. Many susceptibility maps showed signal dropouts in brain regions with extreme iron overload.

Wednesday

**Conclusion:** Our findings suggest that only a subset of QSM algorithms reliably reflect extreme brain iron deposition. Additionally, the study highlights that performing echo combination prior to phase unwrapping and background field removal may introduce artifacts, resulting in lower-than-expected susceptibility values due to signal dropouts.
**Limitations:** A limitation is the small sample size.
**Funding for this study:** No funding was received for this study.
**Ethics committee - additional information:** The study was approved by the local ethics committee (number 1270/2021)
**Author Disclosures:**
Alexander Rauscher: Nothing to disclose
Bernhard Glodny: Nothing to disclose
Elke Ruth R Gizewski: Nothing to disclose
Christoph Birkl: Nothing to disclose
Marlene Panzer: Nothing to disclose
Heinz Zoller: Nothing to disclose
Christian Kames: Nothing to disclose

**MRI signal intensity comparison of high relaxivity vs standard gadolinium-based contrast agents: Concentration-dependent effects across different MRI sequences and field strengths**

*L. Widmer*[1], S. Bhumiwat[2], F. Porões[1], J. M. M. Froehlich[3], H. Thoeny[1]; [1]Fribourg/CH, [2]Phatum Wan/TH, [3]Zurich/CH

**Purpose or Learning Objective:** Gadopiclenol (Elucirem™) is a recent high-relaxivity macrocyclic gadolinium-based contrast agent (GBCA), with limited data on its detailed concentration dynamics, essential for optimizing its use in diverse clinical scenarios. This study compared signal intensity (SI) curves from three GBCAs across various MRI sequences, field strengths, coils and concentrations.
**Methods or Background:** Signal intensity of gadopiclenol, gadoteric acid and gadobutrol vials were measured across 18 MRI sequences on 1.5T and 3T machines and 8 concentrations ranging from 0 to 25 mmol/L in an experimental in-vitro setting. Relationship between SI and concentrations were compared in SE, FSE, GRE and IR sequences.
**Results or Findings:** Concentration had no linear correlation with the SI. At the same concentrations, gadopiclenol produced higher maximal SI than the other two contrast agents in half of the sequences (50%, 9/18). In most sequences (56%, 10/18), gadopiclenol had a left-shifted curve maximum, reflecting higher SI at lower concentrations. Results of identification of curves patterns by sequence type are still pending.
**Conclusion:** Signal intensity curve analysis helps optimize imaging and injection parameters, though in-vivo application requires considering vessel and tissue distribution. These findings suggest using reduced dose of high-relaxivity agents compared to conventional GBCAs, supporting sustainable radiology.
**Limitations:** Experimental
**Funding for this study:** None
**Ethics committee - additional information:** None
**Author Disclosures:**
Harriet Thoeny: Nothing to disclose
Johannes Malte Maria Froehlich: Nothing to disclose
Siwat Bhumiwat: Nothing to disclose
Fabio Porões: Nothing to disclose
Lucien Widmer: Nothing to disclose

**Deep Learning-Based Spatial Resolution Improving Algorithm for MRI: Comparison of Capabilities for Scan Time Reduction and Image Quality Improvement with Conventional Protocol with and without ZIP**

*D. Takenaka*, H. Nagata, T. Ueda, M. Nomura, T. Yoshikawa, Y. Ozawa, Y. Ohno; Toyoake/JP
*(daisuke.takenaka.fr@fujita-hu.ac.jp)*

**Purpose or Learning Objective:** Deep learning reconstruction (DLR) and zero fill interpolation (ZIP) technique have been clinically applied on routine clinical MRIs. Recently, deep learning-based spatial resolution improving algorithm (Precise IQ Engine: PIQE) is developed to transform MR data from low-spatial resolution data to high-spatial resolution data. The purpose of this study was to directly compare utilities of PIQE for scan time reduction and image quality improvement of MRIs as compared with DLR with and without ZIP techniques.
**Methods or Background:** 28 consecutive patients suspected with 17 brain tumors, 6 spinal diseases and 5 musculoskeletal diseases were prospectively scanned with conventional MR (224-382×256-512matrix) and new MR protocols (160-192×192-416matrix). Then, both MR protocol data were reconstructed by DLR with and without ZIP technique or PIQE techniques (total five MR data sets). Each standard protocol was determined as conventional MR protocol reconstructed by DLR without ZIP technique. To compare scan time reduction and image quality improvement among all protocols, mean examination time and signal-to-noise ratios (SNRs) were compared between standard protocol and others by Dunnett's test. To evaluate qualitative image quality improvement, overall image quality, artifact and diagnostic confidence level were assessed by 5-point scales and compared between standard protocol and others by Steel's multiple comparison test.
**Results or Findings:** Mean examination times of new MR protocols were significantly shorter than that of conventional protocols ($p<0.05$), although SNRs had no significant differences. As compared with standard protocol, overall image quality and artifact were significantly improved by conventional protocol reconstructed by DLR with ZIP and new protocol with PIQE ($p<0.05$).
**Conclusion:** PIQE is equal to or more useful for reduce examination time and image quality improvements as with DLR with and without ZIP technique.
**Limitations:** LImited study number and no diagnostic performance evaluation
**Funding for this study:** Canon Medical Systems Corporation
**Ethics committee - additional information:** Fujita Health University Hospital
**Author Disclosures:**
Yoshiyuki Ozawa: Research/Grant Support: Grant-in-Aid for Scientific Research from the Japanese Ministry of Education, Culture, Sports, Science and Technology Research/Grant Support: Smoking Research Foundation
Masahiko Nomura: Nothing to disclose
Takahiro Ueda: Research/Grant Support: Grant-in-Aid for Scientific Research from the Japanese Ministry of Education, Culture, Sports, Science and Technology
Daisuke Takenaka: Nothing to disclose
Hiroyuki Nagata: Research/Grant Support: Grants-in-Aid for Scientific Research from the Japanese Ministry of Education, Culture, Sports, Science and Technology Research/Grant Support: Canon Medical Systems Corporation
Takeshi Yoshikawa: Nothing to disclose
Yoshiharu Ohno: Research/Grant Support: Canon Medical Systems Corporation Research/Grant Support: Smoking Research Foundation

**Power Grid Independent Low Field MRI**

*H-M. Klein*; Burbach/DE
*(mklein@greenscan-imaging.de)*

**Purpose or Learning Objective:** Purpose:
Develop a concept for grid independent, power saving MRI operation using a permanent magnet, solar energy, and a generator supported battery system.
**Methods or Background:** We installed a 0,4 T MRI system with an open design permanent magnet. Regenerative energy is produced with a 29,8 kWp solar array. To achieve grid independency, we installed an ´island solution´ using a 22 kWh LiFePO4 battery. For longer periods of power outage, and insufficient solar energy, a specially designed, direct current (DC), high voltage diesel generator is used. This generator simulates the power profile of a solar array, and is connected to the solar power converter, feeding the battery.
**Results or Findings:** Annual energy uptake of the MRI was 7.022 kWh in 2023. RIS and PACS components consumed 4.959 kWh. Heating and air conditioning consumed 12.500 kWh. Total energy consumption of the practice was 26.801 kWh. Total energy production was 30.930 kWh. Energy balance was positive with 4.129 kWh. Battery and DC generator can provide power grid independent operation. Without grid and solar energy, the practice has an energy consumption rate of max. 1,9 l gasoil/hour.
**Conclusion:** Grid independent, sustainable MRI operation is possible using permanent magnet technology, solar energy production, battery storage and a specially designed power generator.
**Limitations:** Only very few high quality low field MRI with permanent magnet technology are available in the market.
**Funding for this study:** None
**Ethics committee - additional information:** Not applicable
**Author Disclosures:**
Hans-Martin Klein: Nothing to disclose

**Reduced Energy Consumption with Accelerated MRI Using Deep-Learning Reconstruction: A Phantom Study**

*Y. Jung*, R. Alizadeh, M. Corwin, L. Hacein-Bey, A. M. Hernandez; Sacramento/US
*(yojung@ucdavis.edu)*

**Purpose or Learning Objective:** To quantify image quality, potential cost savings, and greenhouse gas emission reductions in accelerated brain and prostate MRI exams using deep learning reconstruction (DLR) and phantom imaging.
**Methods or Background:** A data logger and current transformer sensor were installed upstream of the power distribution unit on three 3T MRI platforms (GE, Siemens, United Imaging) to measure power consumption at 1-second intervals. The ACR phantoms were scanned using the T2 FLAIR sequence from routine brain MRI and the T2-weighted sequence from routine prostate MRI protocols. Phantom scanning was performed using three scan times with different acceleration factors, and images were reconstructed using conventional inverse Fourier transform (IFT) and DLR at three strength levels: Low, Medium, and High. Signal-to-noise ratio (SNR) and low-contrast detectability (LCD) measurements were taken using the ACR phantom. Total energy consumption was recorded for each acquisition.
**Results or Findings:** Total energy consumption decreased monotonically with reduced scan time across all systems and protocols. SNR and LCD, averaged across all scan times, were generally higher for DLR compared to IFT, and SNR increased with increasing DLR strength. Reducing the scan time by ~ 5 minutes with medium-strength DLR resulted in a ~65% reduction in energy consumption compared to a non-accelerated acquisition, while maintaining comparable SNR and LCD. Extrapolating these savings to all T2 FLAIR and T2-weighted sequences performed annually at our institution would result in estimated total savings of 15,944 USD and 74.1 MTCO2e, equivalent to 16.6 gasoline-powered passenger vehicles driven for one year.
**Conclusion:** DLR-accelerated MRI exams provide substantial reductions in cost and greenhouse gas emissions without compromising image quality in phantom imaging experiments.
**Limitations:** Patient data would be required to assess the actual impact of DLR on image quality and energy savings in clinical practice.
**Funding for this study:** RSNA Emerging Issues Environmental Impact and Sustainability grant
**Ethics committee - additional information:** None
**Author Disclosures:**
Ramsey Alizadeh: Nothing to disclose
Lotfi Hacein-Bey: Nothing to disclose
Youngkyoo Jung: Nothing to disclose
Michael Corwin: Nothing to disclose
Andrew M Hernandez: Nothing to disclose

**16:30-17:30** **Research Stage 4**

### Research Presentation Session: Interventional Radiology

### RPS 609

### Developments in vascular and neurovascular interventions

**Moderator**
V. Bérczi; Budapest/HU
*(berczi@hotmail.com)*

**Thrombolysis in basilar infarction (TIBI): A novel angiographic scale for evaluating mechanical thrombectomy in basilar artery occlusion**
*M. E. Chevasco Hanze*, A. Lopez Rueda, A. Nuñez, E. Ripoll, V. Cuba, S. Aixut, L. Aja, M. A. De Miquel Miquel, O. Chirife;
L'Hospitalet de Llobregat/ES

**Purpose or Learning Objective:** Acute Basilar Artery Occlusion (BAO) has the highest morbidity and mortality in posterior circulation strokes. Current reperfusion scores for mechanical thrombectomy (MT) are based on anterior circulation strokes (mTICI score). This study proposes a basilar artery-specific reperfusion score, based on digital subtraction angiography (DSA), to assess MT efficacy in acute BAO.
**Methods or Background:** A retrospective analysis was conducted on a prospective database of acute BAO patients treated with MT within 24 hours of symptom onset at a stroke center from January 2014 to December 2023. Informed consent was obtained, and institutional review board approval was granted. Clinical, procedural, and radiological data were collected. The Thrombolysis in Basilar Infarction (TIBI) score was developed by grading posterior circulation territories in DSA post-MT, using PC-ASPECTS as reference (deducting 1 point for each occipital lobe/cerebellar lobe/thalamus or 2 points for pons/mesencephalon). Successful recanalization was defined as TIBI≥8 and assessed by modified Rankin Scale at 90 days.
**Results or Findings:** Ninety-eight patients were included (median age 70, 56 men). Successful recanalization (mTICI2b/3) was achieved in 84.7% and TIBI≥8 in 71.4%. Good functional status (mRS≤3) was seen in 50% of patients, with better outcomes in mTICI 2b/3 and TIBI≥8 groups ($p < 0.001$ and $p = 0.002$). After adjusting for age, NIHSS socre and Glasgow Coma Sacle, TIBI≥8 was associated with good outcomes at 90 days (OR = 6.18; $p = 0.001$), and TIBI≥7 was also linked to good outcomes (OR = 9.45; $p < 0.001$).
**Conclusion:** The TIBI scale is a novel tool for evaluating MT efficacy in acute BAO. A TIBI≥8 should be the target for successful MT.
**Limitations:** unweighted TIBI (<25% were TIBI<7)
**Funding for this study:** No
**Ethics committee - additional information:** Observational study
**Author Disclosures:**
Antonio Lopez Rueda: Nothing to disclose
Sonia Aixut: Nothing to disclose
Victor Cuba: Nothing to disclose
Miguel Emilio Chevasco Hanze: Nothing to disclose
Enric Ripoll: Nothing to disclose
Oscar Chirife: Nothing to disclose
Ana Nuñez: Nothing to disclose
Maria Angeles De Miquel Miquel: Nothing to disclose
Lucía Aja: Nothing to disclose

**MTICI 2b-stopped or continued after first-pass: interim results of the Rossetti registry for M1 occlusion**
*J. I. García García*[1], O. Chirife[1], P. Vega Valdés[2], E. Gonzalez[3], F. Delgado[4], G. Dolz[1], A. López-Frías López-Jurado[5], F. Aparici Robles[6], A. Lopez Rueda[1]; [1]Barcelona/ES, [2]Oviedo/ES, [3]Barcaldo/ES, [4]Cordoba/ES, [5]Madrid/ES, [6]Valencia/ES
*(juanignaciogarciarx@gmail.com)*

**Purpose or Learning Objective:** The purpose of this study is to analyze the predictive factors of safety and efficacy of additional mechanical thrombectomy in patients with acute ischemic stroke due to M1 occlusion, who achieve mTICI2B recanalization after the first pass of endovascular treatment.
**Methods or Background:** We retrospectively analyzed patients with acute ischemic stroke due to M1 occlusion from the ROSSETTI registry who achieved mTICI 2b recanalization after the first pass of endovascular treatment. Patients were divided into two groups: those who stopped the procedure with a mTICI 2b result and those who continued treatment for a better angiographic outcome. Among those who continued, patients were further split into two subgroups: those with unchanged results (mTICI 2b) and those with improved results (mTICI 2c/3). Demographic, clinical data, procedure details, and outcomes were compared across groups.
**Results or Findings:** We included 300 patients with acute ischemic stroke M1 occlusion who achieved mTICI2b recanalization score after the first pass of endovascular treatment. 132 patients underwent no further passes (group 1), while 168 patients underwent additional passes, with 65 of them maintaining a final mTICI 2b score (group 2) and 103 achieving a final mTICI 2c-3 score (group 3). Group 3 exhibited a higher incidence of distal embolism to new territories compared to group 1 (7.8% vs. 0%; $p < 0.001$). No significant differences in clinical outcomes were observed between the groups and subgroups.
**Conclusion:** Patients who achieved an improved mTICI score after the first recanalization attempt (mTICI 2c/3) had a higher incidence of distal embolisms to new territories compared to patients with no further passes. No significant differences in mRS scores at 3 months were observed between groups and subgroups.
**Limitations:** Inherent limitations of retrospective designs.
**Funding for this study:** None.
**Ethics committee - additional information:** ROSSETTI registry creation was approved by an ethics committee.
**Author Disclosures:**
Guillem Dolz: Nothing to disclose
Pedro Vega Valdés: Nothing to disclose
Eva Gonzalez: Nothing to disclose
Fernando Aparici Robles: Nothing to disclose
Alfonso López-Frías López-Jurado: Nothing to disclose
Juan Ignacio García García: Nothing to disclose
Antonio Lopez Rueda: Nothing to disclose
Fernando Delgado: Nothing to disclose
Oscar Chirife: Nothing to disclose

**Comparison of DSA Morphology Parameters In Predicting Time To Recanalization of Internal Carotid Artery Saccular Aneurysms Treated With Primary Coiling: Does Neck Angle Matter?**
*H. Akkaya*, A. I. Soylu, F. Uzunkaya; Samsun/TR
*(dr.hsynakkaya@gmail.com)*

**Purpose or Learning Objective:** Coil embolization is the most commonly used method of endovascular treatment of narrow-neck saccular aneurysms. However, recanalization and subsequent aneurysm enlargement and rupture are common in aneurysms embolized only with coils. The aim of this study was to investigate which of the morphology findings during the treatment of internal carotid artery (ICA) aneurysms treated with primary coiling is more successful in predicting the time to recanalization.
**Methods or Background:** In this study, DSA images of 51 ICA aneurysms treated with coiling in our center between January 2016 and July 2024 were retrospectively analyzed. Dates of embolization and recanalization times were noted. The segment of the ICA in which the aneurysms were located, the height of the aneurysm, the diameter of the neck, the height/diameter ratios, whether there was a parent artery originating from the aneurysm, and the angle of the neck of the aneurysm were noted. The relationship between these parameters and recanalization times was examined.
**Results or Findings:** The mean age of the patients was 59.6±13.9 years. 24 (47.1 %) patients had recanalization in follow-up examinations. The mean duration of recanalization was 12.73±1.7 months. The aneurysm neck angle was found to be higher in patients with recanalization (p<0.001). A negative (inverse) moderate correlation was found between the time of recanalization and aneurysm size/neck diameter ratio: aneurysm neck angles (r=-0.425; r=-0.537, respectively).
**Conclusion:** The angle of the neck of the saccular aneurysm at the time of treatment is one of the angiographic morphology findings that are successful in predicting the time to recanalization.
**Limitations:** The study has some limitations. First of all, the study was single-centered and the number of patients was small. Another limitation is that only aneurysms localized in the internal carotid artery are evaluated.
**Funding for this study:** N/A
**Ethics committee - additional information:** Ondokuz Mayıs University Ethics committee approval was obtained for the study . The requirement for informed consent from the patients was waived due to the retrospective nature of the study.
**Author Disclosures:**
Ayşegül Idil Soylu: Nothing to disclose
Fatih Uzunkaya: Nothing to disclose
Hüseyin Akkaya: Nothing to disclose

**Radiation Dose Comparison in Endovascular Clot Retrieval: General Anaesthetic versus Conscious Sedation Approaches**
*F. Taylor*, K. Sehgal, D. Carrion, M. Masterson, M. K. Badawy, L-A. Slater; Melbourne/AU
*(fergustay@gmail.com)*

**Purpose or Learning Objective:** Endovascular clot retrieval (ECR) is a time-sensitive, adjunct treatment for large vessel occlusion (LVO) strokes. We present the results of this single high-volume stroke centre, to establish differences in radiation doses between ECR cases performed under general anaesthetic (GA) and conscious sedation (CS).
**Methods or Background:** All ECR cases in adult patients between October 2018 and June 2023 were included. Procedure records, patient characteristics, stroke outcome, and radiation dosimetry measures were collected and analysed retrospectively. Summative data, i.e. median and interquartile range for overall dosimetry measures were described overall and by subgroup according to anaesthetic strategy. ANOVA was used to compare radiation dose measures, clot location, stroke severity measures and endovascular clot retrieval times between anaesthetic strategy.
**Results or Findings:** Radiation doses were higher in patients receiving a GA; DAP 9,346 cGy.cm2, (4,996, 17,442) vs. 7,052 cGy.cm2 (4,529, 10,926), reference air kerma 627 mGy (325, 1,287) vs 507 (300, 789), total fluoroscopy time 34 minutes (19, 63) vs 31 (18, 47). This was associated with a greater pre-reperfusion morbidity (NIHSS of 13 (7, 19) vs 11 (6, 17), more complex thrombus location (more tandem, less M2, more ICA, more basilar clots) and delayed presentation to hospital (227 minutes (101, 452) vs. 125 (66, 281), p <0.001).
**Conclusion:** We have demonstrated higher radiation doses under GA versus CS cases and some of the factors that may contribute to this. These values serve as benchmark ranges for comparable centres performing ECR. Larger multicentre analysis is required to establish more generalisable dose reference levels.
**Limitations:** Single centre cohort. Affected by the COVID pandemic; departmental decision to preference GA at several points between March 2020-October 2021. TICI grading wasn't core lab adjudicated.
**Funding for this study:** This research did not receive any specific grant from funding agencies in the public, commercial, or not-for-profit sectors.
**Ethics committee - additional information:** All procedures performed in studies involving human participants were in accordance with the ethical standards of the institutional and/or national research committee and with the 1964 Helsinki declaration and its later amendments or comparable ethical standards. For this type of study formal consent is not required.
Ethics approval was obtained from the local Human Research Ethics Committee (HREC), reference no. QA/91812/MonH-2022-343316 and the need for informed consent was waived.
**Author Disclosures:**
Fergus Taylor: Nothing to disclose
Mohamed Khaldoun Badawy: Nothing to disclose
Daniel Carrion: Nothing to disclose
Kunal Sehgal: Nothing to disclose
Lee-Anne Slater: Nothing to disclose
Maeve Masterson: Nothing to disclose

**Long-term outcome results after endovascular abdominal aneurysm repair with Zenith stent-graft**
*J. Reymen*, H. Mufty, A. Laenen, S. Houthoofd, G. Maleux; Leuven/BE

**Purpose or Learning Objective:** To report the long-term outcome of patients presenting with an aortic or aortoiliac aneurysm treated with the Zenith Endoprosthesis.
**Methods or Background:** A retrospective analysis of the collected data of 209 consecutive patients who underwent endovascular aneurysm repair (EVAR) with the Zenith Endoprosthesis (Cook Medical) between January 1998 and December 2009 in an academic, tertiary care centre for aortic disease was performed. Patients' imaging and clinical follow-up was performed until December 2023 to ensure a follow-up time of at least 14 years. The primary end points were overall survival and reintervention-free survival. Secondary end points were endograft-related complications, endoleaks, and reinterventions.
**Results or Findings:** Overall survival at 2-, 5-, 10-, and 15-year follow-up was 89.00%, 71.29%, 39.71% and 18.18%, respectively, with n=2 (1%) aneurysm-related deaths. Freedom from type I and III endoleak at 5- and 15-years was 92.82% and 86.12%, respectively; type I (n=37 ; 17.7%) and type III (n=4 ; 2%) endoleaks occurred in the follow-up period until 8 years postoperatively. Reintervention-free survival was 83.08%, 75.62%, 68.66% and 66.17% at 2-, 5-, 10-, and 15-year follow-up, respectively. Reinterventions occurred meanly in the 0- to 8-year follow-up period.
**Conclusion:** Endovascular aneurysm repair using the Zenith Endoprosthesis is effective and durable on long-term follow-up, with acceptably low endograft-related complications and reinterventions. The number of adverse events and reinterventions is minimal after 8 years of follow-up.
**Limitations:** It is a single-centre retrospective study, susceptible to selection bias. The study also has a relatively small patient cohort ( n=209) because not all patients eligible for EVAR were treated with the Zenith Endoprosthesis. Finally, no comparison with other endograft devices was made.
**Funding for this study:** No funding was received for this study.
**Ethics committee - additional information:** The institutional ethics committee approved this retrospective analysis (MP024667).
**Author Disclosures:**
Annouschka Laenen: Nothing to disclose
Sabrina Houthoofd: Nothing to disclose
Geert Maleux: Nothing to disclose
Hozan Mufty: Nothing to disclose
Jessie Reymen: Nothing to disclose

**Assessing abdominal aortic aneurysm growth by using radiomics of different radii of perivascular adipose tissue after endovascular repair**
*R. Lv*, G. Hu, S. Zhang, Z. Zhang, Z. Wang; Beijing/CN
*(18763896097@163.com)*

**Purpose or Learning Objective:** To investigate the relationship between radiomic features of different radii of perivascular adipose tissue (PVAT) and abdominal aortic aneurysm (AAA) growth after endovascular aneurysm repair (EVAR).
**Methods or Background:** Patients with sub-renal AAA who underwent regular follow-up after EVAR from September 2014 to September 2024 were retrospectively collected. Two radiologists segmented the aneurysm and different radii of PVAT (PVAT1-7: 5mm, 7.5mm, 10mm, 12.5mm, 15mm, 17.5mm, 20mm) to evaluate the aneurysm volume changes during follow-up and calculate radiomic features of different PVAT regions. Univariable and multivariable logistic regression was performed to construct models to evaluate the growth of AAA based on the radiomic features of PVAT with different radii (5mm-20mm for models 1-7). Calculate the area under the curve (AUC), sensitivity, specificity, and accuracy of the models, and test the significance of the performance differences among the models.
**Results or Findings:** A total of 79 patients (67±7 years, 82% men) were enrolled in this study, 20 of whom had a growing aneurysm. The AUC for Models1-7 are 0.72, 0.77, 0.71, 0.70, 0.69, 0.71, and 0.75, respectively. The specificities for Models1-7 are 95%, 95%, 97%, 97%, 93%, 92%, and 97%, respectively. DeLong test and McNemar test: p > 0.05 (no statistical significance).
**Conclusion:** The models constructed using the radiomic features of PVAT with different radii after EVAR showed no significant differences in performance for evaluating AAA growth. The models achieved an average specificity of 95%, indicating their effectiveness in minimizing the misclassification of non-growing AAA cases as growth cases.
**Limitations:** The retrospective research highlights the need for prospective and long-term follow-up studies. There are also limitations in the development of imaging segmentation techniques, which still require manual intervention for three-dimensional aneurysm segmentation.
**Funding for this study:** This study has received funding by the National High Level Hospital Clinical Research Funding (2022-PUMCH-B-068).

**Ethics committee - additional information:** Institutional review board of Peking Union Medical College Hospital.
**Author Disclosures:**
Ge Hu: Nothing to disclose
Zhe Zhang: Nothing to disclose
Zhiwei Wang: Nothing to disclose
Shenbo Zhang: Nothing to disclose
Rui Lv: Nothing to disclose

**Towards Clinical Magnetic Particle Imaging: Safety Measurements of Medical Implants in an Extracorporeally-Perfused Human Cadaver Model**

*F. Wegner*[1], T. Friedrich[1], P. Elfers[1], F. Kleefeldt[2], D. Peter[2], P. Gruschwitz[2], T. Kampf[2], P. Vogel[2], V. Hartung[2]; [1]Lübeck/DE, [2]Würzburg/DE
*(franz.wegner@uksh.de)*

**Purpose or Learning Objective:** Magnetic Particle Imaging (MPI) is an emerging, tracer-based, 3D imaging modality on the way to clinical application. It offers high temporal resolution and operates without the use of ionizing radiation, making it particularly advantageous for cardiovascular imaging and real-time interventional monitoring. However, the potential heating of metallic medical devices within the magnetic fields of MPI scanners is a critical safety concern. This study aimed to assess the thermal behavior of commercially available medical implants during MPI-scans in a human cadaver model.
**Methods or Background:** A fiberoptic thermometer probe was introduced into the superficial femoral artery (SFA) of a human cadaver model via a 7 F sheath. A series of commercially available endovascular implants (including six stents, five coils, and one vascular plug) were then positioned sequentially within the SFA. The thermometer probe was retracted sequentially to ensure direct contact with each implant. Additionally, a hole was drilled in the femur, which contained a gamma nail, and the fiberoptic probe was externally inserted to establish contact with the nail. A custom-built human-sized MPI-scanner for interventional purpose was positioned around the cadavers' thigh, and an MPI-sequence consisting of 40 pulses (4 pulses per second) was applied, with the respective implant centrally located within the scanner. Throughout the MPI-sequence, the cadavers' thigh was perfused extracorporeally with a blood-equivalent fluid using an external flow pump.
**Results or Findings:** The gamma nail exhibited a temperature increase of 0.04 K during the MPI-sequence, while no detectable heating was observed in any of the endovascular devices tested.
**Conclusion:** Commonly used medical implants do not heat up significantly in a human-sized MPI-scanner under realistic conditions.
**Limitations:** Only a limited number of commercial devices was tested in this work.
**Funding for this study:** N/A
**Ethics committee - additional information:** Protocol Number 20220413 01
**Author Disclosures:**
Franz Wegner: Nothing to disclose
Viktor Hartung: Nothing to disclose
Florian Kleefeldt: Nothing to disclose
Philipp Gruschwitz: Nothing to disclose
Thomas Friedrich: Nothing to disclose
Dominik Peter: Nothing to disclose
Patrick Elfers: Nothing to disclose
Patrick Vogel: Nothing to disclose
Thomas Kampf: Nothing to disclose

**Electroporation with local or systemic bleomycin for the treatment of vascular malformations: early results of a prospective study**

*N. Papalexis*, G. Peta, M. Di Carlo, S. Quarchioni, L. Campanacci, M. Carta, M. Miceli, G. Facchini; Bologna/IT
*(nicolaspapalexis@gmail.com)*

**Purpose or Learning Objective:** Purpose: To evaluate the safety and efficacy of electrochemotherapy with bleomycin for the treatment of soft tissue vascular malformations.
**Methods or Background:** Materials and Methods: This study analyzes the early results of a prospective study "BESVAM", designed to prospectively evaluate the safety and efficacy of electrochemotherapy for vascular malformations. 18 patients were enrolled from February 2023 to July 2024. Bleomycin was injected intralesionally for low-flow vascular malformations or systematically for high-flow vascular malformations. The primary goal was pain control, measured in VAS score at 3,6, and 12 months follow-up. The secondary goal was the size reduction of the lesion and variations in the QLQ questionnaire.
**Results or Findings:** Results: Twelve patients received bleomycin intravenously and six patients intralesional. Baseline VAS scores averaged 6.9 (SD 2.1), decreasing to 3,1 (SD 3.2) at three months. Further reduction was observed at 6 and 12 months with scores of 2.1 (SD 2.0) and 1.6 (SD 2.0) respectively. Size was reduced from a mean of 155.1 cm3 (range 56.7 to 515.3 cm3) pre-treatment to a mean of 122.5 cm3 (range 42.2 to 438.9 cm3) at the 6-month follow-up ($p<0.05$). (12 months) Twelve patients discontinued pain relief therapy. Ten patients experienced skin discoloration at the site of insertion of the needles.
**Conclusion: Conclusion:** The preliminary results are promising, suggesting that electroporation with local or systemic bleomycin could be a safe and effective tool for the management of vascular malformations.
**Limitations:** Small sample size, lack of control group
**Funding for this study:** None
**Ethics committee - additional information:** Prospective study approved by the local ethcis committee of Emilia Romagna, Italy.
**Author Disclosures:**
Marco Miceli: Nothing to disclose
Michela Carta: Nothing to disclose
Laura Campanacci: Nothing to disclose
Giancarlo Facchini: Nothing to disclose
Giuliano Peta: Nothing to disclose
Simone Quarchioni: Nothing to disclose
Maddalena Di Carlo: Nothing to disclose
Nicolas Papalexis: Nothing to disclose

### Thursday, February 27

**08:00-09:30** **Research Stage 1**

### Research Presentation Session: Abdominal and Gastrointestinal

### RPS 701

### Imaging of the intestines with focus on Crohn's disease

**Moderator**
S. A. Taylor; London/UK
**Author Disclosures:**
Stuart A. Taylor: Advisory Board: aztrazeneca; Grant Recipient: takeda; Share Holder: Motilent

### Improvement of diagnostic performance in low-dose CT enterography: the impact of an artificial intelligence iterative reconstruction algorithm

R. Guo[1], W. Zhou[2], G. Zhang[2], *T. Wang*[2], P. Hu[1], Q. Liang[1], P. Rong[1]; [1]Changsha/CN, [2]Shanghai/CN
*(tiantian.wang@cri-united-imaging.com)*

**Purpose or Learning Objective:** To investigate the clinical value of artificial intelligence iterative reconstruction (AIIR) in optimizing image spatial resolution and diagnostic performance of low-dose CT enterography (CTE) for patients with Crohn's disease (CD), compared with the routine hybrid iterative reconstruction (HIR).
**Methods or Background:** Forty patients with suspected CD were prospectively enrolled to receive low-dose CTE (80kVp, ref 120mAs) with ilecolonoscopy-guided biopsy as the reference. Images were reconstructed using the AIIR and HIR. Diagnosis of CD was made on a per-segment level. The diagnostic confidence was scored with a five-point scale (1=insufficient, 5=definitely confirmed). Signal-to-noise ratio (SNR), contrast-to-noise ratio (CNR), and edge rise slope (ERS) of the bowel wall were measured. Diagnostic image quality, including conspicuity of bowel wall enhancement, thickness, luminal narrowing, comb signs, and creeping fat signs, was evaluated with a five-point scale (1=poor, 5=excellent).
**Results or Findings:** There were 77 bowel segments confirmed with CD, of which 57 were detected on HIR images and 71 on AIIR images. AIIR images showed 124.3% higher SNR, 136.3% higher CNR, 68.4% higher ERS, and more conspicuous diagnostic imaging features (all p<0.001), indicating higher image spatial resolution than HIR. With higher spatial resolution, AIIR images showed better diagnostic performance for CD detection than HIR images (sensitivity: 88.7% vs 71.2%; accuracy: 92.9% vs 87.9%; specificity: 94.5% vs 94.5%; false-positive-rate: 13.4% vs 16.2%; false-negative-rate: 4.5% vs 10.9%). The diagnostic confidence was significantly improved by AIIR (4.5±0.6 vs 3.4±0.6, p<0.001).
**Conclusion:** AIIR improved the image spatial resolution of low-dose CTE and thus delivered higher diagnostic confidence and better diagnostic performance than HIR. Low-dose CTE with AIIR provides excellent image quality and reliable CD detection, making it a feasible option for follow-up examinations for CD patients.
**Limitations:** A single-centre study.
**Funding for this study:** No funding was received for this study.
**Ethics committee - additional information:** The study received approval from the Institutional Review Board (R20230019).
**Author Disclosures:**
Pengfei Rong: Nothing to disclose
Tiantian Wang: Nothing to disclose
Wanhui Zhou: Nothing to disclose
Guozhi Zhang: Nothing to disclose
Rui Guo: Nothing to disclose
Qi Liang: Nothing to disclose
Pengzhi Hu: Nothing to disclose

### Comparison of Conventional vs. Abbreviated MR Enterography in Crohn's Disease: Assessment of Inter-Radiologist Agreement for Categorizing Disease Activity and Complications

*J. R. Rimola Gibert*[1], C. Saavedra[1], N. Capozzi[2], I. De Kock[3], A. R. Radmard[4], M. Scharitzer[5], M. C. Masamunt[1], I. Ordás[1], J. Dillman[6]; [1]Barcelona/ES, [2]Bologna/IT, [3]Ghent/BE, [4]Tehran/IR, [5]Vienna/AT, [6]Cincinnati, OH/US
*(jrimola@clinic.cat)*

**Purpose or Learning Objective:** Crohn's disease (CD) often requires multiple imaging evaluations. An abbreviated MR enterography (aMRE) protocol, without IV contrast, could improve patient experience and reduce costs. This study aims to compare inter-observer agreement for detecting CD activity and complications using conventional MRE protocol (cMRE) versus an abbreviated protocol (aMRE).
**Methods or Background:** Ten radiologists from six countries independently reviewed cMRE and aMRE exams from 80 CD patients, with imaging assessments separated by at least one month. The exams included both pre- and post-treatment images of CD patients. Interobserver agreement, Fleiss' Kappa statistics, and Gwet's concordance 1 (AC1), which corrects the prevalence dependence in categorizations, were calculated at the bowel segment and patient levels for the presence of different findings indicative of active inflammation and complications.
**Results or Findings:** Overall (n=80), the concordance for detecting disease activity was high and comparable between aMRE and cMRE: agreement was 0.83 (0.80-0.86) for aMRE vs. 0.84 (0.82-0.87) for cMRE; Kappa values were 0.41 (0.29-0.53) vs. 0.36 (0.23-0.49); and AC1 values were 0.76 (0.64-0.88) vs. 0.79 (0.69-0.89). Agreement for detecting active disease was similar across the small bowel and colon, as well as between pre-treatment (n=51) and post-treatment (n=29) MREs. For detecting strictures, agreement was 0.76 (0.73-0.78) for aMRE vs. 0.72 (0.68-0.75) for cMRE; Kappa values were 0.35 (0.24-0.46) vs. 0.34 (0.24-0.43); and AC1 values were 0.61 (0.47-0.74) vs. 0.50 (0.35-0.65). For penetrating complications, agreement was 0.81 (0.79-0.84) for aMRE vs. 0.85 (0.82-0.87) for cMRE; Kappa values were 0.47 (0.34-0.59) vs. 0.57 (0.45-0.69); and AC1 values were 0.71 (0.56-0.86) vs. 0.76 (0.63-0.90).
**Conclusion:** The interobserver agreement for detecting active CD and related complications using an aMRE protocol was comparable to that of the cMRE protocol that supports the adoption of abbreviated MRE protocols.
**Limitations:** None
**Funding for this study:** None
**Ethics committee - additional information:** Local ethics committee approved the study with the code HCB/2021/0629
**Author Disclosures:**
Maria Carme Masamunt: Nothing to disclose
Carolina Saavedra: Nothing to disclose
Ingrid Ordás: Nothing to disclose
Isabelle De Kock: Nothing to disclose
Nunzia Capozzi: Nothing to disclose
Jordi Rimola Rimola Gibert: Advisory Board: Janssen Consultant: AstraZeneca, Janssen Alimentiv, Clario, Lument, Origo Research/Grant Support: Abbvie
Martina Scharitzer: Nothing to disclose
Amir Reza Radmard: Nothing to disclose
Jonathan Dillman: Nothing to disclose

### Inflammatory Burden in Crohn’s Disease: Insights from PET/MR Enterography

*N. Bogveradze*, K. Kranz, T. Traub-Weidinger, C. Primas, A. Macher-Beer, W. Reinisch, T. Mang, M. Hacker, M. Scharitzer; Vienna/AT
*(bogveradze.nino@gmail.com)*

**Purpose or Learning Objective:** To evaluate the global and regional inflammatory burden in patients with Crohn’s Disease (CD) using FDG PET/MR enterography (PET/MRE), in correlation with histopathological findings and relevant biomarkers.
**Methods or Background:** Patients with CD undergoing PET/MRE and ileocolonoscopy were included in this retrospective study between 2016-2021. Eight intestinal segments were manually segmented, and the uncorrected total lesion glycolysis values (TLG) were summed to calculate global CD activity score (GCDAS). GCDAS and highest SUVmax/ patient were correlated with clinical biomarkers (fecal calprotectin [FC], serum C-reactive protein [CRP], peripheral blood leukocyte counts, Harvey-Bradshaw index [HBI]) to evaluate global inflammation. For assessing regional inflammation, SUVmax was correlated with histopathological disease activity. Comparisons were made using Spearman's coefficient and Wilcoxon-W tests.
**Results or Findings:** In 41 patients (mean age, 40 years ±14 (SD), 26 men), the highest segmental SUVmax correlated significantly with FC (r= 0.443 p < 0.004) and CRP (r = 0.645, p < 0.001). The GCDAS correlated significantly with CRP (r = 0.498, p = 0.01) but not with FC (p = 0.49), leukocyte counts (p=0.56) or HBI (p = 0.518). SUVmax values significantly correlated with grading of active inflammation in corresponding histopathological samples (r = 0.515, p < 0.001) and were higher in segments with severe histopathological inflammation (4.7, 95% CI: 3.8-5.6) compared to segments with moderate (3.3, 95% CI: 2.6-4.1) or without active inflammation (2.0, 95% CI: 1.8-2.0).
**Conclusion:** SUVmax was found to be a reliable biomarker for assessing global inflammatory burden and correlated well with histopathological segmental activity. Furthermore, SUVmax enabled grading of active inflammation. GCDAS showed good correlation with CRP, but not with FC and may therefore be less useful for quantifying global disease burden.
**Limitations:** Retrospective study design
**Funding for this study:** N/A
**Ethics committee - additional information:** Ek 1356/2023

**Author Disclosures:**
Kerstin Kranz: Nothing to disclose
Thomas Mang: Nothing to disclose
Tatjana Traub-Weidinger: Nothing to disclose
Christian Primas: Nothing to disclose
Andrea Macher-Beer: Nothing to disclose
Martina Scharitzer: Nothing to disclose
Nino Bogveradze: Nothing to disclose
Marcus Hacker: Nothing to disclose
Walter Reinisch: Nothing to disclose

**Multiparametric and multi-score MRI evaluation of pediatric Crohn's disease: characterization of the perianal fistulizing phenotype**
*A. Valenti*, F. Maccioni, L. Busato, L. Bottino, A. Longhi, C. Catalano; Rome/IT
*(alessandra.valenti@uniroma1.it)*

**Purpose or Learning Objective:** Perianal fistulas are severe complications of pediatric Crohn's disease (CD), due to a high risk of demolitive surgery. MRI is the gold standard for scoring intestinal and perianal CD. The purpose of this study was to stage severity and activity of both lesions using MRI scores in pediatric CD.
**Methods or Background:** A retrospective study was performed on 186 pediatric patients. Inclusion criteria were: proven CD, intestinal and perianal disease, complete MRI. Forty patients were finally included. MR Enterography (MRE) and high-resolution MRI (HRMRI) of the perianal region were used to analyze intestinal and perianal disease. The MEGS score was applied to assess the severity of intestinal disease, the Parks' classification and MAGNIFI-CD score to classify perianal disease and quantify its activity. Correlations between location and activity of intestinal and perianal lesions were investigated.
**Results or Findings:** Simple perianal fistulas (Parks A and B) were found in 82.5% of patients, whereas complex fistulas (Parks C-E or a combination of them) in 17.5 %. Jejunal, ileal and colonic lesions were found in 22,5%, 80% and 35%, variably associated. Considering colorectal lesions only, 70% were left-sided. Disease of the left colon was associated with a more extensive disease, > 25 cm in length ($p < 0.001$), and a more severe activity, MEGS grade 3. Correlations were found between left-sided colonic lesions and severe fistulas, and between MAGNIFI-CD and MEGS scores both grade 3 ($p<0.05$).
**Conclusion:** The perianal fistulizing phenotype in pediatric patients is correlated with the severity of intestinal inflammation and with left-sided colonic disease.
**Limitations:** We used two differente MRI scanners to obtoin our data.
**Funding for this study:** No funding.
**Ethics committee - additional information:** We have received the approvation of the ethics commitee of our Hospital
**Author Disclosures:**
Alessandro Longhi: Nothing to disclose
Alessandra Valenti: Nothing to disclose
Lorenza Bottino: Nothing to disclose
Carlo Catalano: Nothing to disclose
Ludovica Busato: Nothing to disclose
Francesca Maccioni: Nothing to disclose

**Magnetic Resonance Imaging biomarkers in the diagnosis of gastrointestinal acute Graft-versus-Host-Disease**
*L. Busato*, F. Maccioni, A. Valenti, L. Bottino, A. Iori, U. La Rocca, C. Catalano; Rome/IT
*(ludovica.busato@uniroma1.it)*

**Purpose or Learning Objective:** Acute gastrointestinal Graft-versus-Host disease (GI-a GVHD) is one of the most severe complications stem cell transplantation, occurring when the transplanted immune cells attack the host's intestinal tissues. Aim of this study was to evaluate the efficacy of MRI biomarkers in the diagnosis and staging of acute gastrointestinal GI-aGVHD, currently based on clinical and endoscopic criteria only.
**Methods or Background:** Thirty-five patients with clinical suspicion of GI-aGVHD were retrospectively and prospectively analysed, 21/35 retrospectively, 14/35 prospectively, both separately and in conjunction. In these patients we investigated 15 MRI biomarkers suggestive of bowel inflammation and GVHD severity. A diagnostic score was tested, based on the most relevant GVHD biomarkers: small bowel involvement, T2 and post-contrast bowel wall stratification, ascites , oedema of the retroperitoneal and declivous tissues.
**Results or Findings:** GI -GVHD was confirmed by biopsy in 13/35 patients (37%). Analysing the 6 key biomarkers (diagnostic score), MRI showed 88.2% and sensitivity 100%specificity. In addition to these siw biomarkers, wall stiffness and mesenteric oedema appeared useful for differentiating GVHD from non-GVHD patients. Most commonly affected segments were the proximal, middle and distal ileum (82.3%). Considering all the 15 MRI Biomarkers, MRI showed high predictive value on disease severity and mortality, superior to the clinical score.
**Conclusion:** MRI is a noninvasive and accurate tool for the diagnosis of GI-GVHD,which can provide crucial information and implement current clinical and endoscopic criteria. Limitations: The small number of patients, due to the low prevalence of this disease.
**Limitations:** The small number of patients, due to the low frequence of the disease.
**Funding for this study:** No funding
**Ethics committee - additional information:** Our study has been approved by the ethics committee of our hospital
**Author Disclosures:**
Alessandra Valenti: Nothing to disclose
Ursula La Rocca: Nothing to disclose
Annapaola Iori: Nothing to disclose
Lorenza Bottino: Nothing to disclose
Carlo Catalano: Nothing to disclose
Ludovica Busato: Nothing to disclose
Francesca Maccioni: Nothing to disclose

**Reevaluating MR-Enterography: Value or Overuse?**
*R. Martín-Márquez*[1], E. Gutiérrez Dorta[2], D. J. L. Ruiz[1], J. Mesa[1]; [1]Córdoba/ES, [2]Ourense/ES
*(rociomartinmarquez@gmail.com)*

**Purpose or Learning Objective:** To evaluate the profitability of Magnetic Resonance Enterography (MRE) in different clinical contexts, determining if it is both clinically and cost-effective, optimizing resources and improving patient care. To establish a protocol for performing MRE in various clinical scenarios.
**Methods or Background:** A retrospective study of 615 patients who underwent MRE at Reina Sofía Hospital (Córdoba, Spain) over one year. Patients were divided into two groups: patients with known inflammatory bowel disease (IBD) and those without IBD history. Variables included the reason for the examination, clinical unit, patient presentation, and findings from MRE, colonoscopy, and intestinal biopsy. A descriptive analysis was performed, and differences were assessed with chi-square or Student's t-tests ($p<0.05$). Diagnostic indices of MRE were compared to colonoscopy (sensitivity, specificity, positive predictive value (PPV), negative predictive value (NPV).
**Results or Findings:** A total of 242 (39.3%) patients had a history of IBD, while 373(60.7%) did not. Pathological findings were seen in 68.2% of IBD patients versus 23.3% without IBD ($p<0.05$). 9.1% of IBD patients with normal colonoscopy had pathological findings on MRE, compared to 10.4% without IBD ($p>0.05$). Among those with elevated fecal calprotectin, MRE detected more findings in IBD patients (86.4% vs. 18.8%; $p<0.05$). Diarrhea occurred in 8.7% of IBD patients and 37.3% of non-IBD patients, with MRE findings in 52.4% and 16.5%, respectively ($p<0.05$). Diagnostic indices of MRE: IBD group: Sensitivity 70.6%, Specificity 90.9%, PPV 92.3%, NPV 66.7%. Non-IBD group: S 43.2%, E 89.5%, PPV 70.7%, NPV 73%.
**Conclusion:** Our study highlights the importance of MRE in IBD patients and the need to optimize its use in non-IBD patients, especially with negative colonoscopies.
**Limitations:** The main limitations of this study are its retrospective design, which may lead to missing information, and the lack of long-term follow-up.
**Funding for this study:** No
**Ethics committee - additional information:** There is no additional information.
**Author Disclosures:**
Juan Mesa: Nothing to disclose
Eduardo Gutiérrez Dorta: Nothing to disclose
Rocío Martín-Márquez: Nothing to disclose
Daniel José López Ruiz: Nothing to disclose

**The diagnostic yield of non-contrast versus contrast-enhanced magnetic resonance enterography (MRE) for small bowel (SB) evaluation in undiagnosed patients: Experience from four centres in the UK**
*S. Martin*, J. Pancholi, S. Liong; Manchester/UK
*(sarahh.martinn93@gmail.com)*

**Purpose or Learning Objective:** This service evaluation compares the diagnostic yield and utilisation trends of non-contrast versus contrast-enhanced magnetic resonance enterography (MRE) for small bowel (SB) evaluation in a cohort of patients without a diagnosis of inflammatory bowel disease or clear gastrointestinal symptom aetiology.
**Methods or Background:** A retrospective review of all 1,012 MREs performed across four hospitals in Greater Manchester, United Kingdom, between 1 January and 31 December 2023, identified 208 undiagnosed patients. Among these, 92(44.2%) underwent non-contrast and 116(57.8%) underwent contrast-enhanced MRE. Diagnostic yield was assessed by reviewing MRE reports and correlating findings with colonoscopic histopathology results (within 6 weeks of MRE) and faecal calprotectin levels (FC, within 12 weeks of MRE).

**Results or Findings:** SB abnormalities were observed in a similar minority of patients in both the contrast (14/116 [12.1%]) and non-contrast (5/92 [5.4%], p=0.15) groups. Of 45 patients with colonoscopies completed within 6 weeks of MRE, 22 had terminal ileum (TI) biopsies available (non-contrast: n=8; contrast-enhanced: n=14). Compared with TI biopsies, contrast-enhanced MRE has sensitivity of 100% (95%CI 15.8-100%) and specificity of 83.3%(95%CI 51.5-97.9%), whereas non-contrast MRE has sensitivity of 100%(95%CI 2.5-100%) and specificity of 100%(95%CI 59-100%). Contrast-enhanced MRE has positive predictive value (PPV) of 50%(95%CI 22-78%) and accuracy of 85.7(95%CI 57.2-98.2%). Non-contrast MRE has PPV 100%(95%CI 2.5-100%) and accuracy of 100%(95%CI 63.1-100%). FC was available for 27 patients, but there was no clear relationship between FC levels and MRE results.
**Conclusion:** Contrast enhancement did not significantly alter the diagnostic yield of small bowel pathology in our cohort and is known to require longer acquisition and reporting times, impacting service capacity. Non-contrast MRE has high sensitivity and specificity for diagnosis of SB pathology.
**Limitations:** Retrospective design. Small number of patients with histopathology and FC available.
**Funding for this study:** None.
**Ethics committee - additional information:** Ethics approval was not required as this was an educational project and retrospective service evaluation.
**Author Disclosures:**
Jay Pancholi: Nothing to disclose
Sue Liong: Nothing to disclose
Sarah Martin: Nothing to disclose

**Ultrasound is more effective than MRI for monitoring the response to medical treatment in patients with active ileocolonic Crohn's disease – a prospective blinded multicenter study**
J. Brodersen[1], *S. R. Rafaelsen*[2], M. Agerbæk Jue[1], T. Knudsen[1], J. Keldsen[3], M. D. Jensen[1]; [1]Esbjerg/DK, [2]Vejle/DK, [3]Odense/DK
*(soeren.rafael.rafaelsen@rsyd.dk)*

**Purpose or Learning Objective:** The aim of this study was to evaluate intestinal ultrasound (IUS), magnetic resonance imaging enterocolonography (MREC), panenteric capsule endoscopy (PCE) and faecal calprotectin (FC) for determining response to medical treatment in patients with ileocolonic CD
**Methods or Background:** This prospective, blinded, multicentre study included patients with endoscopically active CD. Patients were scheduled for IC, MREC, IUS, PCE and FC before and 12 weeks after medical treatment. The vascularity within the affected bowel wall areas was assessed according to the Limberg score. The Simple Ultrasound Score for Crohn's Disease (SUS-CD) was used for activity assessment. A > 50% reduction of the Simple Endoscopic Score for Crohn's Disease (SES-CD) with IC defined treatment response as gold standard.
**Results or Findings:** From 2018 to 2024, 50 patients completed the pre- and post-treatment evaluation with IC, and endoscopic response was achieved in 25 (50.0%). PCE was omitted in 12 (24.0%) patients because of stricturing CD. All activity scores decreased in patients achieving endoscopic response: The Simple Ultrasound Score for Crohn's Disease 2.2 vs. 6.1 (P < 0.001), Magnetic Resonance Index of Activity 29.0 vs. 37.1 (P = 0.05), SES-CD with PCE 3.1 vs. 12.8 (P < 0.001) and FC 115.3 vs. 1339.9 mg/kg (P < 0.001). The sensitivity and specificity of IUS, MREC, PCE and FC was 80.0% (95% CI 56.3-94.3) / 77.8% (52.4-93.6), 65.2% (42.7-83.6) / 87.0% (66.4-97.2), 87.5% (61.7-98.4) / 86.7% (59.5-98.3) and 90.0% (68.3-98.8) / 86.4% (65.1-97.1), respectively.
**Conclusion:** IUS, PCE and FC are equally effective for determining endoscopic response in patients with active CD. MREC is insufficient for determining endoscopic response.
**Limitations:** First, the sample size is limited. Second, IC served as gold standard for treatment response, which may favour modalities assessing mucosal inflammation (PCE and FC).
**Funding for this study:** The study was initiated by the investigators without funding from medical imaging companies or the capsule endoscope manufacturer.
**Ethics committee - additional information:** The study was approved by the Local Ethics Committee of Southern Denmark (S-20170188). All patients gave informed consent before participation. The study was registered: NCT03435016.
**Author Disclosures:**
Mie Agerbæk Jue: Nothing to disclose
Michael D. Jensen: Nothing to disclose
Jacob Brodersen: Nothing to disclose
Jens Keldsen: Nothing to disclose
Torben Knudsen: Nothing to disclose
Sören R. Rafaelsen: Nothing to disclose

**Gut feels emotion: Psychological distress is associated with alterations of magnetic resonance enterography in patients with Crohn's disease**
*Y. Ke*, R. Zhang, H. Cai, Q. Zeng, S-T. Feng, Z. Peng, X. Li; Guangzhou/CN
*(keyq7077@163.com)*

**Purpose or Learning Objective:** Psychological distress may affect bowel disease activity in patients with Crohn's disease (CD). However, limited studies have investigated its correlation with trans-/peri-intestinal alterations in CD. Therefore, we aimed to investigate the relationship between psychological distress and intestinal abnormalities identified by magnetic resonance enterography (MRE), and to explore their underlying association using blood neurotransmitters.
**Methods or Background:** 105 CD patients and 46 healthy controls (HCs) were prospectively recruited. CD patients underwent MRE and provided blood samples for 19 serum neurotransmitters measurement. All participants completed State-Trait Anxiety Inventory (including STAI-Trait and State scores), Beck Depression Inventory (BDI), and Perceived Stress Scale (PSS) questionnaires to assess psychological distress. Correlation analysis, multivariable logistic regression, and causal mediation analyses were employed to investigate the relationship between psychological distress and MRE features.
**Results or Findings:** Psychological scores of CD patients, including STAI-Trait, PSS, and BDI scores, were significantly higher than HCs (all P<0.001). Among them, STAI-Trait score was significantly correlated with stricture (r=0.505), mural T2WI hyperintensity (r=0.466), perianal diseases (r=0.359), and perienteric effusion (r=0.340) (all P<0.05). Multivariable logistic regression analysis indicated that STAI-Trait score significantly influenced the odds of perienteric effusion (OR: 1.124; 95% CI: 1.007-1.255; P=0.036). In causal mediation analysis, a direct effect of STAI-Trait score on perienteric effusion (P=0.04) was observed; STAI-Trait score and tryptophan had a combined effect on perienteric effusion (P=0.06), approaching statistical significance. Negative correlation between tryptophan level and perienteric effusion (r=-0.220, P<0.05) was also found.
**Conclusion:** Psychological state is associated with MRE-detectable intestinal morphological changes, and neurotransmitters may serve as mediators in establishing this connection.
**Limitations:** This was a single-center study with small sample size. To enhance reliability and validity, future investigations should consider conducting multicenter studies with larger sample sizes.
**Funding for this study:** This study was financially supported by National Natural Science Foundation of China (82070680, 82270693, 82271958, 82471948, and 82072002).
**Ethics committee - additional information:** The study was approved by the institutional ethics review board of our hospital (No. [2021]215-2).
**Author Disclosures:**
Shi-Ting Feng: Nothing to disclose
Zhenpeng Peng: Nothing to disclose
Yaoqi Ke: Nothing to disclose
Qiaoling Zeng: Nothing to disclose
Ruonan Zhang: Nothing to disclose
Xuehua Li: Nothing to disclose
Huasong Cai: Nothing to disclose

**MRI neurophenotype reflecting brain-gut interactions to predict intestinal disease progression in patients with Crohn's disease**
*R. Zhang*, X. Shen, Y. Wang, J. Lin, L. Huang, W. He, S-T. Feng, X. Li; Guangzhou/CN
*(zhangrn25@mail2.sysu.edu.cn)*

**Purpose or Learning Objective:** There is considerable recent interest in the role of brain-gut axis in the pathogenesis and manifestations of Crohn's disease (CD). We developed a multimodal neuroimaging-based model to characterize the neurophenotype of CD patients and predict intestinal disease progression, using multi-omics data to demonstrate its validity.
**Methods or Background:** This prospective study enrolled 109 CD patients who underwent baseline tests (including multimodal neuroimaging, psychological scales, MR enterography, ileocolonoscopy) and fecal/blood samples collection within one week. The neurophenotype of patients with different intestinal inflammation levels was characterized using a radiomics model, developed from 13 out of 13,870 neuroimaging features. This neurophenotype in predicting disease progression during follow-up was evaluated using Kaplan-Meier curves and Cox regression analysis. Multi-omics data (including fecal microbiome, fecal/blood metabolomics, intestinal/blood-brain-barrier permeability, and blood neurotransmitter) were used to elucidate how this neurophenotype reflecting brain-gut interactions.
**Results or Findings:** The model enabled accurate characterization of neurophenotypes in patients with different intestinal inflammation levels in training and test cohorts (AUC=0.824-0.842, both P<0.05). Neurophenotype was the most important predictor of disease progression (HR=29.05, P=0.033), surpassing psychological traits (HR=0.95-1.09, all P>0.05). Multi-omics analysis revealed that elevated intestinal inflammation was correlated with increased intestinal permeability and specific gut microbiota (e.g., Enterococcus) and metabolites (e.g., caproic acid), which collectively contributed to high-risk neurophenotype (all P<0.05). High-risk neurophenotype subsequently associated with intestinal disease progression by establishing correlations with six blood neurotransmitters (e.g., tryptophan) (all P<0.05).
**Conclusion:** The neurophenotype varies among CD patients with different intestinal inflammation levels and can predict intestinal disease progression. Multi-omics data offer biological evidence to support its validity.
**Limitations:** This was a single-centre study, and the potential mechanisms underlying the brain-gut axis in our study have yet to be validated.
**Funding for this study:** This study was financially supported by National Natural Science Foundation of China (82070680, 82270693, 82271958, 82072002, 82170537, and 82222010), Guangdong Basic and Applied Basic Research Foundation (2023B1515020070 and 2023A1515011097), 2023 SKY Imaging Research Fund of the Chinese International Medical Foundation (Z-2014-07-2301), and National Key R&D Program of China (2023YFC2507300).
**Ethics committee - additional information:** The study was approved by the institutional ethics review board of The First Affiliated Hospital of Sun Yat-sen University (No. [2021]215-2)
**Author Disclosures:**
Shi-Ting Feng: Nothing to disclose
Li Huang: Nothing to disclose
Weitao He: Nothing to disclose
Ruonan Zhang: Nothing to disclose
Xuehua Li: Nothing to disclose
Yangdi Wang: Nothing to disclose
Xiaodi Shen: Nothing to disclose
Jinjiang Lin: Nothing to disclose

**First Findings from the BIPOCUS Study: Progress and Educational Impact on Ultrasound Training in the Practical Year**
*E. Höhne*[1], V. Schäfer[2], S. Petzinna[2], A. Wittek[2], J. Gotta[1], P. Reschke[1], F. Recker[2]; [1]Frankfurt/DE, [2]Bonn/DE

**Purpose or Learning Objective:** Point-of-care ultrasound (POCUS) is increasingly important in clinical settings, leading to a growing demand for comprehensive ultrasound training in medical education. This study marks the University of Bonn's first attempt to integrate ultrasound courses and handheld devices into the regular curriculum for final-year medical students and assess their utilization.
**Methods or Background:** Forty students in their practical year received a handheld ultrasound device for four months and were invited to participate in eight optional ultrasound courses, where they acquired and rated images using a developed rating system. At the end of the tertial, they could complete a voluntary survey on equipment usage.
**Results or Findings:** Participation in the optional ultrasound courses was positive, with the Introduction and FAST module attracting the most participants (29). Lung images received the highest average rating (18.82 out of possible 23 points, SD ± 4.30), while aorta and vena cava images scored lowest (16.62, SD ± 1.55). The overall mean score for all images was 17.47 (SD ± 2.74). Only 21 students responded to the survey, and 67% used the device independently four times or fewer during the tertial.
**Conclusion:** The study aimed to enhance improving students' ultrasound skills, but device usage was unexpectedly low, with most students using it only once a month or less. This raises concerns about resource justification, prompting future initiatives to focus on technical improvements, better login data access, and closer monitoring of usage and progress to emphasize practical ultrasound training in medical education.
**Limitations:** This study's limitations include a small sample size, single-institution focus, incomplete usage data, and low response rates, which hinder the generalizability and reliability of the findings regarding ultrasound device integration into student routines.
**Funding for this study:** None
**Ethics committee - additional information:** The local ethics committee of the Uuniversity of Bonn approved the study (253/23-EP) .
**Author Disclosures:**
Agnes Wittek: Nothing to disclose
Valentin Schäfer: Nothing to disclose
Florian Recker: Nothing to disclose
Philipp Reschke: Nothing to disclose
Jennifer Gotta: Nothing to disclose
Elena Höhne: Nothing to disclose
Simon Petzinna: Nothing to disclose

**08:00-09:30 Research Stage 2**

### Research Presentation Session: Cardiac

### RPS 703

### New techniques and clinical applications of CMR

**Moderator**
K.-F. Kreitner; Mainz/DE
*(Karl-Friedrich.Kreitner@unimedizin-mainz.de)*

**Flip angle mapping and "without-gadolinium" enhancement: A new approach to acute myocardial infarction in magnetic resonance imaging**
*G. Lucchi*, G. D. Aquaro, A. Marcucci, M. Lombardo, L. Faggioni, R. Lencioni, D. Cioni, E. Neri; Pisa/IT

**Purpose or Learning Objective:** To evaluate Flip Angle Mapping (FAM) as a non-contrast alternative to Late Gadolinium Enhancement (LGE) for assessing ischemic core in acute myocardial infarction using balanced Steady-State Free Precession (bSSFP) MRI sequences.
**Methods or Background:** This study included 11 patients suspected of Myocardial Infarction with Non-Obstructive Coronary Arteries (MINOCA) and eight healthy controls. Two bSSFP datasets were acquired with flip angles of 15° and 60°, respectively. Signal intensity differences were mapped and compared to LGE images. Pathological areas were manually segmented on both FAM and LGE images. A statistical analysis was conducted to evaluate the correlation and concordance of the two methods in estimating the pathological area.
**Results or Findings:** The diagnosis of MINOCA was confirmed in five patients, while four others were diagnosed with myocarditis and two with Takotsubo syndrome. Comparison between FAM and LGE images showed a complete overlap of pathological areas. Linear regression analysis revealed a strong positive correlation between the extent of LGE and FAM abnormalities (r=0.99; p<0.001). Bland-Altman analysis confirmed good agreement between the two methods (mean difference: -0.3%; 95% limits of agreement: -4.3 to 3.6%).
**Conclusion:** These findings suggest that FAM could potentially replace LGE for acute myocardial damage assessment, dispensing with contrast agents. If validated in larger studies, FAM could represent a significant advancement in non-invasive cardiac imaging.
**Limitations:** No limitations were identified.
**Funding for this study:** No funding was received for this study.
**Ethics committee - additional information:** The study is a methodological proof of principle.
**Author Disclosures:**
Giacomo Lucchi: Nothing to disclose
Emanuele Neri: Nothing to disclose
Alessandro Marcucci: Nothing to disclose
Giovanni Donato Aquaro: Nothing to disclose
Riccardo Lencioni: Nothing to disclose
Lorenzo Faggioni: Nothing to disclose
Marilena Lombardo: Nothing to disclose
Dania Cioni: Nothing to disclose

**One-shot black-blood late gadolinium enhancement imaging for rapid, motion-free, and diagnostically accurate scar imaging**
*V. De Villedon De Naide*[1], K. Narceau[1], B. Durand[1], T. Küstner[2], M. Villegas-Martinez[1], P. Jais[1], M. Stuber[3], H. Cochet[1], A. Bustin[1]; [1]Bordeaux/FR, [2]Tübingen/DE, [3]Lausanne/CH
*(victor.de-villedon@ihu-liryc.fr)*

**Purpose or Learning Objective:** Multi-shot black-blood LGE imaging is increasingly being used to assess myocardial scars and overcome poor scar-blood contrast, often observed with conventional bright-blood LGE imaging. However, this method is time-consuming, requires multiple breath-holds, and is prone to residual motion artifacts. Here, we introduce a one-shot black-blood LGE sequence combined with image denoising to provide rapid, motion-free, and diagnostically accurate scar imaging.
**Methods or Background:** The 2D black-blood ECG-triggered LGE sequence acquires multiple single-shot short-axis images per slice using a non-selective 180° inversion pulse, followed by a T1-rho preparation. A dummy heartbeat is added between shots, for magnetization-recovery. Single-shot images are averaged to enhance quality. The proposed one-shot sequence eliminates dummy heartbeats and employs a patch-based low-rank denoising algorithm (PROST) to achieve image quality comparable to multi-shot techniques. 19

patients with ischemic heart disease underwent 1.5T CMR (Siemens Area) using reference PSIR and five-shot black-blood LGE imaging 12min after gadolinium injection. One-shot images were retrospectively selected from multi-shot datasets and were PROST-denoised. A blinded radiologist graded diagnostic confidence, documented eventual residual motion artefact and extracted scar volume and signal intensities (blood, scar, remote myocardium) using Circle CVI42 for the three datasets.
**Results or Findings:** Acquisition times were in average 4min shorter for black-blood one-shot PROST compared to reference sequences. No statistically significant differences were observed between black-blood multi-shot and one-shot PROST in signal intensities or in scar detection, while scar volume agreement was excellent. Diagnostic confidence was rated good or excellent in 95% of black-blood multi-shot and 89% of one-shot PROST scans. No residual motion artefacts were found in black-blood one-shot PROST datasets.
**Conclusion:** Black-blood one-shot PROST provides rapid, motion-free, and diagnostically accurate scar imaging, offering a more efficient and patient-friendly solution.
**Limitations:** Prospecting testing is now warranted.
**Funding for this study:** This research was supported by funding from the French National Research Agency under grant agreement ANR-22-CPJ2-0009-01, and from the European Research Council (ERC) grant "SMHEART" under the European Union's Horizon 2020 research and innovation programme (grant agreement No101076351).
**Ethics committee - additional information:** The study was approved by the Biomedical Research Ethics Committee and all participants provided informed consent for participation.
**Author Disclosures:**
Victor De Villedon De Naide: Nothing to disclose
Aurelien Bustin: Nothing to disclose
Hubert Cochet: Nothing to disclose
Kalvin Narceau: Nothing to disclose
Manuel Villegas-Martinez: Nothing to disclose
Pierre Jais: Nothing to disclose
Baptiste Durand: Nothing to disclose
Matthias Stuber: Nothing to disclose
Thomas Küstner: Nothing to disclose

**Incremental Value of Multiparametric Cardiac MRI for Non-invasive Identification of Significant Acute Cardiac Allograft Rejection: a Prospective and Biopsy-proven Study**
*P. Zhou*, Z. Dong, S. Zhao; Beijing/CN
*(zhoupengyu996@163.com)*

**Purpose or Learning Objective:** Using endomyocardial biopsy as the reference standard, this study aimed to 1) evaluate the association between cardiac MRI (CMR) multiparameters and significant acute cardiac allograft rejection (SR), and 2) assess the incremental value of CMR multiparameters over conventional serum examinations for identifying SR in heart transplantation (HTx) recipients.
**Methods or Background:** HTx recipients with endomyocardial biopsy and healthy controls were prospectively recruited for CMR assessment. CMR feature tracking (CMR-FT) was performed to evaluate the left ventricular (LV) global strain in all three directions. The last serum examinations including N-terminal pro brain natriuretic peptide (NT-proBNP) before anti-rejection therapy were recorded. Participants were divided into 3 groups: control, SR (acute cellular rejection grade≥2R and/or antibody-mediated rejection [AMR] grade≥pAMR1), and NSR (non-SR).
**Results or Findings:** Finally, thirty controls (43.3±13.6 years, 26 male) and 51 HTx recipients comprising 23 SRs (48.6±12.6 years, 24 male) and 28 NSRs (42.7±14.9 years, 16 male) were enrolled for analysis. Compared with NSRs, SRs showed elevated NT-proBNP (7797.0±7527.6pg/ml vs 3334.6±5935.3pg/ml, p<.001), worse LV global longitudinal strain (GLS) (-9.7±3.1% vs -13.1±2.9%, p<.001), and increased native T1 (1384±80.1ms vs 1321±69.9ms, p<.001) and T2 values (50.9±2.7ms vs 45.7±4.3ms, p<.001). In multivariable analysis, LVGLS (OR=0.76, 95%CI, 0.59 to 0.98, p=.03) and T2 value (OR=1.35, 95%CI, 1.10 to 1.65, p=.01) were independently associated with SR after NT-proBNP adjustment. Furthermore, the likelihood ratio test showed LVGLS (p=.002) and T2 value (p<.001) had incremental value over NT-proBNP for identifying SR.
**Conclusion:** LV GLS and T2 value were independently associated with SR, providing incremental value for non-invasive identification of significant rejection in HTx recipients.
**Limitations:** Although a relatively small participant sample, this is a prospective and biopsy-proven study with comprehensive cardiac examinations, including T1 and T2 mapping of CMR.
**Funding for this study:** This study is supported by the National Key R&D Program of China (Nos. 2021YFF0501400 and 2021YFF0501404) and the Key Project of National Natural Science Foundation of China (No. 81930044).
**Ethics committee - additional information:** The ethics committee from Fuwai Hospital.
**Author Disclosures:**
Pengyu Zhou: Nothing to disclose
Zhixiang Dong: Nothing to disclose
Shihua Zhao: Nothing to disclose

**Multiparametric cardiac MRI for the detection of chimeric antigen receptor T-cell therapy associated myocardial changes**
*D. Kravchenko*[1], L. Bischoff[1], A. Isaak[1], T. Holderried[1], T. S. Emrich[2], A. Varga-Szemes[2], N. Mesropyan[1], D. Kütting[1], J. A. Luetkens[1]; [1]Bonn/DE, [2]Charleston, SC/US
*(dmitrij.kravchenko@live.ca)*

**Purpose or Learning Objective:** New chimeric antigen receptor (CAR)-T cell therapy has demonstrated advantages over traditional cancer therapies for treatment of highly refractory or relapsing hematological malignancies. Unfortunately, there is a paucity of data regarding cardiotoxic cardiac MRI (CMR) findings of therapy associated cytokine release syndrome (CRS).
**Methods or Background:** Consecutive patients were enrolled for CAR-T cell therapy and received a standard 1.5 T CMR examination consisting of functional cines, parametric mapping, late gadolinium enhancement (LGE), and featuring tracking strain, before therapy (baseline), during acute cytokine release syndrome (CRS; as determined by treating oncologist and lab parameters), and at 6-month follow-up (mean 184±20 days). Data was compared using RM-ANOVA with Tukey's posthoc test.
**Results or Findings:** 29 patients were available for analysis (mean age 60±15 years, 23 males [79%]). The most common malignancy was diffuse large B-cell lymphoma (13 [45%]). CRS was observed at a median time of one day (IQR 1-2 days) after CAR T-cell therapy and reached a median degree of 1 (IQR 1-2). One patient passed away due to non-cardiac related CRS. One patient developed therapy associated heart failure. No new instances of LGE were observed in any cases. There were no differences from baseline to CRS or to follow-up scans for left ventricular ejection fraction (61±5 vs 60±6 vs 59±7%, p=0.39), T1 relaxation times (969±18 vs 988±26 vs 972±22 ms, p=0.11), T2 relaxation times (53.3±2.4 vs 54.0±3.0 vs 52.9±1.8 ms, p=0.36), global longitudinal strain (-16.3±2.2 vs -15.6±2.4 vs -14.9±2.8, p=0.18), global circumferential strain (-12.5±2.9 vs -13.0±2.7 vs -12.2±1.8, p=0.51), or global radial strain (28.3±12.5 vs 29.2±8.1 vs 30.5±6.0, p=0.67).
**Conclusion:** CAR T-cell therapy related low degree CRS does not produce significant myocardial changes on multiparametric CMR from baseline to acute CRS or follow-up.
**Limitations:** Small study size.
**Funding for this study:** None
**Ethics committee - additional information:** Ethikkommission der Medizinischen Fakultät Bonn
Geb. 74, 4. OG
Venusberg-Campus 1
53127 Bonn
**Author Disclosures:**
Julian Alexander Luetkens: Nothing to disclose
Narine Mesropyan: Nothing to disclose
Alexander Isaak: Nothing to disclose
Leon Bischoff: Nothing to disclose
Dmitrij Kravchenko: Speaker: Philips
Daniel Kütting: Nothing to disclose
Tilman Stephan Emrich: Grant Recipient: Siemens
Tobias Holderried: Nothing to disclose
Akos Varga-Szemes: Grant Recipient: Siemens Consultant: Elucid

**Cardiovascular magnetic resonance–derived upper ventricular septal scar can predict the prognosis of left bundle branch area pacing**
*Y. Fan*, X. Zhu; Nan Jing/CN
*(13218017502@163.com)*

**Purpose or Learning Objective:** As a novel technique, left bundle branch pacing (LBBP) can achieve excellent resynchronization in patients with left bundle branch block (LBBB). The study is to use cardiovascular magnetic resonance (CMR) to evaluate myocardial scars of different segments to predict the prognosis of patients with LBBB.
**Methods or Background:** Consecutive patients with LBBB, left ventricular ejection(LVEF)≤35% and who underwent CMR examination and successful LBBP were retrospectively enrolled. The myocardial scar of different segments is assessed by CMR. LVEF response was defined as a 15% increase in LVEF assessed by echocardiography at 6 months.
**Results or Findings:** Among 68 patients who were included, and 52 patients patients showed a favorable LVEF response . The responders had lower global, septal scar burden by CMR (P<0.001). The scar burden of AHA 8 is independently associated with the prognosis of LBBP (AUC: 0.877 [95% CI:

0.782, 0.971]) and the linear equation was that ΔLVEF= -0.4161(scar burden) + 23.229 (r=-0.60,P＜0.001), indicating that each 1% increased in scar burden, LVEF decreased by 0.4161%. Moreover, the patterns of scar of AHA8 and the scar morphology are independent of the improvement of LVEF (P＞0.05).
**Conclusion:** The scar burden of AHA 8, as a common implantation area, can predict LVEF improvement. And we recommend to pay more attention to the extent of mycardial LGE rather than the patterns and morphology.
**Limitations:** The limitations are that firstly, it is a retrospective single-center investigation and it may lead to an inclusion bias in that some patients who did not undergo CMR before surgery were not included in the examination. Secondly, the study only investigated patients with low LVEF, and further confirmation is needed on the relationship between the prognosis of patients with high LVEF.
**Funding for this study:** Project supported by the Young Scientists Fund of the National Natural Science Foundation of China (No.82302163 )
**Ethics committee - additional information:** Given that the study was retrospective, the committee has been waived.
**Author Disclosures:**
Xiaomei Zhu: Nothing to disclose
Yin Fan: Nothing to disclose

### Wideband myocardial T2 mapping with implantable cardiac device: A preliminary evaluation in healthy volunteers at 1.5 T

*P. Gut*[1], D. Kim[2], H. Cochet[3], F. Sacher[3], P. Jais[3], M. Stuber[1], A. Bustin[4]; [1]Lausanne/CH, [2]Northwestern/US, [3]Pessac/FR, [4]Bordeaux/FR
*(paulinerose.gut@gmail.com)*

**Purpose or Learning Objective:** Myocardial T2 mapping allows assessment of myocardial inflammation and edema, but is impacted by artefacts related to implantable cardioverter defibrillators (ICDs), leading to image artifacts and inacurate T2 values. This study aimed to integrate a wideband T2 preparation into a T2 mapping sequence and evaluate its performance against conventional T2 mapping in healthy subjects with and without ICDs.
**Methods or Background:** Three short-axis slices covering the heart at the basal, mid-ventricular, and apical levels were acquired in eight healthy volunteers (2 females, age: 26±6y) at 1.5T (MAGNETOM Aera, Siemens) during end-expiration in mid-diastole using both conventional and wideband T2 mapping, with and without an ICD taped below the left clavicle (~10 cm from the heart). The T2 preparation module (duration=0, 27, 55ms) included two adiabatic hyperbolic secant refocusing pulses of 1.6 kHz (conventional) and 5.0 kHz (wideband). Common parameters included: resolution=1.4mmx1.4mm, slice thickness=8mm, FA=15°, GRAPPA x2, partial Fourier phase 6/8, TE/TR=2.09/3.95ms, readout bandwidth=1221Hz/pixel, FOV=360mmx287mm, and gradient recalled-echo (GRE) readout. T2 maps were reconstructed using a 2-parameter (M0 and T2) fitting model. Myocardial T2 values were manually extracted in 16 heart segments. Statistical analyses were performed using repeated measures ANOVA and Bonferroni correction.
**Results or Findings:** Without ICD, T2 values were not significantly different between conventional (mean: mean: 43.5, SD: 2.21) and wideband sequences (mean: 44.0, SD: 2.15) (P=0.111). With ICD, conventional T2 values significantly decreased (mean: 36.4, SD: 5.91) (P<0.01), especially in apical anterior, apical inferior, mid-ventricular anterior, and basal anterior segments. Wideband T2 values remained unchanged (mean: 42.7, SD: 1.91) (P=0.377).
**Conclusion:** Wideband T2 mapping effectively reduces ICD-related artifacts, providing more accurate myocardial T2 values than conventional T2 mapping.
**Limitations:** The study was conducted solely on healthy individuals. Validation in clinical populations is warrented.
**Funding for this study:** This research was supported by funding from the French National Research Agency under grant agreements Equipex MUSIC ANR-11-EQPX-0030, ANR-22-CPJ2-0009-01, ANR-21-CE17-0034-01, and Programme d'Investissements d'Avenir ANR-10-IAHU04-LIRYC, and from the European Research Council (ERC) under the European Union's Horizon 2020 research and innovation program (grant agreement 101076351).
**Ethics committee - additional information:** The study was approved by the Biomedical Research Ethics Committee and all participants provided informed consent for participation.
**Author Disclosures:**
Pauline Gut: Nothing to disclose
Aurelien Bustin: Nothing to disclose
Hubert Cochet: Nothing to disclose
Daniel Kim: Nothing to disclose
Pierre Jais: Nothing to disclose
Frederic Sacher: Nothing to disclose
Matthias Stuber: Nothing to disclose

### Left ventricular remodelling index to predict ventricular tachyarrhythmia in nonischemic dilated cardiomyopathy with ejection fraction <35%

*X. Jia*, S. Zhao; Beijing/CN
*(jiaxi981014@163.com)*

**Purpose or Learning Objective:** Based on current guidelines, only a few dilated cardiomyopathy (DCM) patients with left ventricular ejection fraction (LVEF) <35% receive appropriate implantable cardioverter-defibrillator therapy, leading to increased medical costs and patient complications. We explored the predictive value of LV remodeling index (LVRI) for ventricular tachyarrhythmia (VTA) in nonischemic DCM with LVEF <35%.
**Methods or Background:** In this retrospective single-center study, consecutive nonischemic DCM patients with LVEF <35% (n=271) who underwent cardiac magnetic resonance (CMR) imaging were followed up for VTA events, including sustained ventricular tachycardia, ventricular fibrillation/flutter, sudden cardiac death (SCD), and aborted SCD. The newly derived LVRI was defined as the cubic root of the LV end-diastolic volume divided by the maximal LV wall thickness. Competing risk regression analysis and Kaplan-Meier analysis were used to evaluate the association of LVRI with VTA.
**Results or Findings:** During a median follow-up of 71 months (interquartile range: 17–134 months), 35 (12.9%, mean age 46.7 years, 27 males) participants reached VTA events. The presence of late gadolinium enhancement (LGE) (62.9% vs. 60.2%, p=0.761) and LVEF (23.3±6 vs. 21.9±10.3, p=0. 197) were not significantly different between the patients with and without VTA events. Kaplan-Meier curve analysis showed that participants with LVRI ≥7.5 were more likely to experience VTA (p<0.0001). In the multiple competing risk analysis, when heart transplantation and heart failure-related death were counted as competing risks, LV mass index (hazard ratio [HR], 0.983; 95% confidence interval [CI]: 0.968-0.999; p=0.033) and LVRI ≥7.5 (HR, 2.496; 95% CI: 1.213-5.138; p=0.013) were observed as the independent predictors of VTA after adjusting for age, sex and left bundle branch block.
**Conclusion:** In the cohort of patients with nonischemic DCM with LVEF <35%, CMR-assessed LVRI ≥7.5 was an independent predictor of VTA events.
**Limitations:** Not applicable.
**Funding for this study:** Funding was received from the National Key R&D Program of China (No. 2021YFF0501400, 2021YFF0501404); Key Project of National Natural Science Foundation of China (No. 81930044).
**Ethics committee - additional information:** The study received institutional review board approval and written informed consent was obtained from all participants.
**Author Disclosures:**
Shihua Zhao: Nothing to disclose
Xi Jia: Nothing to disclose

### The Effect of Obesity on Cardiac Structure and Function: A Magnetic Resonance Study of the Hamburg City Health Cohort

*J. Erley*, D. G. Aydemir, K. Muellerleile, E. Cavus, G. Adam, M. Meyer, E. Tahir; Hamburg/DE

**Purpose or Learning Objective:** To analyze the effect of waist-to-hip ratio (WHR) and body mass index (BMI) on cardiac structure and function using magnetic resonance imaging (CMR).
**Methods or Background:** The Hamburg City Health Study (HCHS) is a population-based cohort study. Individuals between 45-74 years of age underwent 3T CMR. Subjects with cardiac diseases (e.g., coronary artery disease, myocardial infarction), and previous cardiac interventions were excluded. Linear regression models were conducted, adjusted for age and sex.
**Results or Findings:** 1671 subjects (41% female, mean age 64±8 years) were analyzed. Median WHR was 0.95 [interquartile range: 0.88; 1.01] and median BMI was 26.2kg/m² [23.8; 29.2]. Concerning BMI cut-off values, 44% of subjects were overweight (BMI 25-29.9kg/m²) and 20% obese (BMI ≥ 30kg/m²). According to the WHR, 81% of subjects were obese (WHR ≥0.85 in females and 0.90 in males). An increase in WHR was associated with a 5.2% [0.1-10.2] higher left ventricular (LV) ejection fraction (p=0.044), and a 43.6g [27.6; 59.5] higher LV end-diastolic mass (EDM) (p<0.001), but lower LV and right ventricular (RV) end-diastolic volumes (EDV) (LV: -37.3ml [-57.4; -17.3], p<0.001; RV: -34.0ml [-56.0; -12.1], p=0.002) and end-systolic volumes (LV: -18.7ml [-28.5; -8.8], p<0.001; RV: -16.0ml [-28.8;-3.1], p=0.015), leading to lower stroke volumes (SV) (LV: -18.5ml [-32.2;-4.7], p=0.008; RV: -18.8ml [-33.6;-4.1], p=0.013). An increase in BMI was associated with a 1.9g [0.2; 2.2] higher LVEDM (p<0.001), higher EDV (LV: 0.5ml [0.2; 0.9], p=0.002; RV: 0.4ml [0.1; 0.7], p=0.047) and a 0.4ml [0.2; 0.7] higher LVSV (p<0.001).
**Conclusion:** An increase in WHR is associated with a higher left ventricular mass and lower volumes as a sign of concentric remodeling, while an increase in BMI is associated with ventricular dilatation.
**Limitations:** Analyses are not adjusted for other cardiovascular risk factors.

**Funding for this study:** The HCHS is supported by the Innovative medicine initiative (IMI) under Grant No. 116074, by the Fondation Leducq under Grant Number 16 CVD 03, by the euCanSHare Grant Agreement No. 825903-euCanSHare H2020 and the DFG under project Grant TH1106/5-1; AA93/2-1. The DIFE provides the licence for the Food Frequency and Physical activity. Technical equipment is provided by SIEMENS according to a contract for 12 years as well as by the Schiller AG on a loan basis for 6 years and by Topcon on a loan basis from 2017 until 2022. The Hamburg City Health Study is additionally supported by an unrestricted Grant (2017–2022) by Bayer. Project-related analyses are supported by Amgen, Astra Zeneca, BASF, Deutsche Gesetzliche Unfallversicherung (DGUV), DKFZ, DZHK, Novartis, Seefried Stiftung and Unilever. The study is further supported by donations from the "Förderverein zur Förderung der HCHS e.V.", TEPE (2014) and Boston Scientific (2016). A current list of the supporters is online available on www.uke.de/hchs.
**Ethics committee - additional information:** The study was approved by the local ethics committee of the medical association in Hamburg.
**Author Disclosures:**
Gerhard Adam: Nothing to disclose
Ersin Cavus: Nothing to disclose
Mathias Meyer: Nothing to disclose
Jennifer Erley: Nothing to disclose
Kai Muellerleile: Nothing to disclose
Destina Gizem Aydemir: Nothing to disclose
Enver Tahir: Nothing to disclose

**Lipomatous hypertrophy of the atrial septum in cardiac magnetic resonance**
*A. Fortunati*, D. Fazzini, S. Papa, M. Alì, F. Darvizeh, F. Secchi; Milan/IT
*(alice.fortunati@unimi.it)*

**Purpose or Learning Objective:** Lipomatous hypertrophy of the atrial septum is a benign anomaly, consisting in a fatty infiltration of the interatrial septum with sparing of the fossa ovalis and typically associated with elderly. This retrospective study aim to define the prevalence of LHAS through cardiac magnetic resonance and its correlation with age or functional biventricular parameters.
**Methods or Background:** A retrospective analysis of 621 patients who underwent CMR from March 2020 to March 2022 was performed, with the following inclusion criteria: the presence of a 4-chamber sequence and the presence of volume analysis. All images were reviewed by a reader to evaluate the presence of LHAS. The atrial septum thickness was measured, and the functional biventricular parameters retrieved from the clinical report. The statistical analysis was conducted using the Spearman's correlation test.
**Results or Findings:** Among the 619 patients included in the study, 241 patients were found with LHAS and 150 showed lipomatous deposition of the upper half of the atrial septum with a mean thickness of the atrial septum of 3 mm. A significant negative correlation was found between the degree of LHAS and left ventricle end-diastolic volume ($r = -0.21$, $p<0.001$) and systolic volume ($r = -0.20$, $p<0.001$). A significant negative correlation was also recognized between the degree of LHAS and right ventricle end-diastolic volume ($r = -0.25$, $p<0.001$) and systolic volume ($r = -0.18$, $p<0.001$).
**Conclusion:** LHAS reached a 39% prevalence. Findings confirm a significant negative correlation between LHAS and biventricular end-diastolic volumes according to a consecutive reduction of atrial and ventricular volumes to offset the volumetric increase of atrial septum and a progressive increase of LHAS disease prevalence with age.
**Limitations:** The study has some limitations due to its monocentric nature and the relatively small sample size.
**Funding for this study:** None
**Ethics committee - additional information:** The local Ethics Committee approved this retrospective study.
**Author Disclosures:**
Francesco Secchi: Nothing to disclose
Sergio Papa: Nothing to disclose
Deborah Fazzini: Nothing to disclose
Marco Alì: Nothing to disclose
Alice Fortunati: Nothing to disclose
Fatemeh Darvizeh: Nothing to disclose

**Risk Stratification of Sudden Cardiac Death in Non-Ischemic Cardiomyopathy: Towards Arrhythmogenic Substrate Assessment in Cardiac MRI**
D. Zhou, M. Lu, *Y. Wang*; Beijing/CN

**Purpose or Learning Objective:** Magnetic resonance imaging (MRI)-derived arrhythmogenic substrate is indicative of sudden cardiac death (SCD) in patients with non-ischemic cardiomyopathy (NICM). A key issue that needs to be addressed is what extent of T1 mapping metric contributes to the prognosis for SCD over late gadolinium enhancement (LGE).
**Methods or Background:** A total of 837 NICM patients who underwent T1 mapping MRI were consecutively enrolled in this study. The primary endpoint is a composite of SCD-related events, including SCD, appropriate implantable cardioverter-defibrillator shock and resuscitated cardiac arrest.
**Results or Findings:** Over a median follow-up of 58.3 months, 78 patients reached the primary endpoint, and 198 patients reached the secondary endpoint. In the adjusted analysis, LGE ≥ 7.2%(HR: 4.748, $p < 0.001$), extracellular volume (ECV) fraction ≥ 31.8% (HR: 2.913, $p = 0.001$), and native T1 z-score ≥ 2.1 (HR: 1.686, $p = 0.035$) were associated with SCD-related events. Patients with LGE (-) and ECV ≥ 31.8% were at higher risk of experiencing SCD events compared to those with ECV < 31.8% and LGE between 0-7.2% or mid-wall/focal LGE. Patients stratified by LGE ≥ 7.2% exhibited a high risk of experiencing SCD-related events with an annual event rate of 4.65%, regardless of ECV. Patients with LVEF > 35%, LGE < 7.2%, and ECV < 31.8 exhibited an actual low risk of SCD with an annual event rate of 0.2%.
**Conclusion:** LGE ≥ 7.2% was strongly associated with high SCD risk, superior to LGE distribution and pattern. ECV serves a crucial role in differentiating patients at low to moderate risk, particularly those with negative LGE or focal/mid-wall LGE.
**Limitations:** This is a retrospective study.
**Funding for this study:** High-level research projects of the National Health Commission (2022-GSP-QZ-5)
**Ethics committee - additional information:** Fuwai Hospital
**Author Disclosures:**
Minjie Lu: Nothing to disclose
Yining Wang: Nothing to disclose
Di Zhou: Nothing to disclose

**One-click joint bright- and black-blood late gadolinium enhancement and T2 mapping for advanced myocardial imaging in the acute STEMI population**
*V. De Villedon De Naide*[1], E. Gerbaud[1], B. Durand[1], M. Villegas-Martinez[1], A. I. Schmid[2], P. Jais[1], M. Stuber[3], H. Cochet[1], A. Bustin[1]; [1]Bordeaux/FR, [2]Vienn/AT, [3]Lausanne/CH
*(victor.de-villedon@ihu-liryc.fr)*

**Purpose or Learning Objective:** CMR imaging enables post-infarction risk-stratification by identifying prognostic markers, such as infarct size (IS), the presence of microvascular obstruction (MVO), ejection fraction, area-at-risk (AAR) and myocardial salvage (MS). However, collecting these markers requires the use of several MRI sequences. Here, we propose a unified 'one-click' sequence jointly collecting scar, MVO, and MS information for seamless planning, fast acquisition, and enhanced image quantification and analysis in the acute STEMI population.
**Methods or Background:** The proposed 2D whole-heart SPOT-MAPPING acquisition is a single-shot breath-held sequence gathering black- and bright-blood LGE images, averaged for optimal measurement of IS and MVO. For the bright-blood shots, a T2 preparation module with increasing duration is used to generate a T2 map for myocardial tissue quantification (MS and AAR). Seven patients with acute STEMI underwent CMR (1.5T Siemens). Pre-contrast T2 maps and post-contrast PSIR, SPOT and SPOT-MAPPING images were collected in a random order 12 after injection of gadolinium. Left ventricular wall and scar contours were drawn by a radiologist using Circle CVI42. Prognostic markers were extracted according to literature, along with T2 values in remote and injured myocardium. Acquisition times were recorded.
**Results or Findings:** Acquisition times for PSIR, SPOT, T2 mapping and proposed SPOT-MAPPING were 10, 10, 13 and 10 heartbeats per slice, respectively. No significant differences were found between SPOT-MAPPING and PSIR for IS and between SPOT-MAPPING and T2 mapping for AAR, and T2 values. By imaging both IS and AAR in a co-registered fashion, SPOT-MAPPING enabled the measurement of the MS and the MVO.
**Conclusion:** SPOT-MAPPING enables easy planning, fast acquisition and enhanced image quantification and analysis for patients with acute STEMI.
**Limitations:** Further validation in a larger cohort is warranted, as SPOT-MAPPING clinical application is still in its early stages.

Thursday

**Funding for this study:** This research was supported by funding from the French National Research Agency under grant agreement ANR-22-CPJ2-0009-01, and from the European Research Council (ERC) grant "SMHEART" under the European Union's Horizon 2020 research and innovation programme (grant agreement No101076351).
**Ethics committee - additional information:** The study was approved by the Biomedical Research Ethics Committee and all participants provided informed consent for participation.
**Author Disclosures:**
Victor De Villedon De Naide: Nothing to disclose
Aurelien Bustin: Nothing to disclose
Hubert Cochet: Nothing to disclose
Albrecht Ingo Schmid: Nothing to disclose
Manuel Villegas-Martinez: Nothing to disclose
Pierre Jais: Nothing to disclose
Baptiste Durand: Nothing to disclose
Edouard Gerbaud: Nothing to disclose
Matthias Stuber: Nothing to disclose

**08:00-09:30** **Research Stage 3**

### Research Presentation Session: Imaging Informatics and Artificial Intelligence

### RPS 705

### Artificial intelligence in breast imaging

**Moderator**
J. Teuwen; Nijmegen/NL
*(j.teuwen@nki.nl)*

### Optimal utilization of an AI diagnostic software in a mammography screening program in Switzerland

*M. Blum*, A. Geissler, D. Ehlig, J. Vogel, J. Subelack, R. Morant; St.Gallen/CH
*(marcel.blum@unisg.ch)*

**Purpose or Learning Objective:** The goal of this study is to evaluate Profound AI® (pAI) in the screening process of the organized mammography screening program (MSP) "donna". We aim to identify the optimal utilization of pAI in the MSP regarding its effectiveness (sensitivity and specificity) and its influence on required resources.
**Methods or Background:** In this retrospective study, we analyse all mammographies from one screening round, i.e., the years of 2022 and 2023, of the MSP "donna" in the Swiss canton of St.Gallen (approximately 27,600 mammographies) using pAI by iCAD, which will assign each mammography a case and predictive risk score. We use optimization models, such as the receiver operating characteristics curve, to find the optimal threshold for case discussion in a consensus conference. We simulate multiple AI implementation scenarios within the MSP, including AI as a substitute for one of the two radiologists and AI as a preselection tool to identify mammographies for double reading.
**Results or Findings:** First results of this study are expected in early 2025 with anticipation to determine an optimal threshold when a mammography should be further discussed in a consensus conference. This threshold is expected to increase the effectiveness by increasing the breast cancer detection rate. In the simulated scenarios, we expect that the workload of radiologists can be reduced significantly, thus increasing the efficiency of the MSP, without loss of effectiveness.
**Conclusion:** Our study will contribute to identifying the optimal implementation of AI in the screening process of an MSP, optimize its effectiveness, i.e., increasing the cancer detection rate, and its efficiency, as well as initiate a discussion about the future of organized screening.
**Limitations:** This study's limitation lies in its retrospective design and the initial omission of interval carcinomas.
**Funding for this study:** This study is partly funded by the Cancer League of Eastern Switzerland.
**Ethics committee - additional information:** This study has been approved by the Ethics Committee of Eastern Switzerland (EKOS) under the project ID 2024-01310.
**Author Disclosures:**
Jonas Subelack: Nothing to disclose
Alexander Geissler: Nothing to disclose
David Ehlig: Nothing to disclose
Marcel Blum: Nothing to disclose
Justus Vogel: Nothing to disclose
Rudolf Morant: Nothing to disclose

### Artificial intelligence mammography interpretation systems are affected more by mammographic image quality issues than radiologists are

*S. D. Verboom*, J. M. D. S. Boita, M. Broeders, I. Sechopoulos; Nijmegen/NL
*(sarah.verboom@radboudumc.nl)*

**Purpose or Learning Objective:** To determine how common image quality issues in mammograms affect the performance of artificial intelligence (AI)-based mammography interpretation systems compared to expert breast radiologists.
**Methods or Background:** Five common image quality issues were simulated on 80 digital screening mammograms (40:20:20, cancer:benign:normal). Each issue was simulated at two levels: the lowest quality that was acceptable to radiologists, and a realistic quality that was not acceptable. Thirteen expert breast radiologists from five countries and two commercial AI systems assessed all mammograms and scored the mammograms with a probability of malignancy (PoM) and a recall decision. The AI recall decision was obtained by matching the specificity on standard quality images to that of the radiologists. The area under the receiver operating characteristics curve (AUC) and recall decisions of radiologists and AI for the two lower quality levels were compared to those for the standard quality images.
**Results or Findings:** The radiologists' original mean AUC of 0.76 (95%CI 0.68-0.84) was not affected by the lower image quality (p=0.77, 0.46). The AUCs of AI system A were 0.72 (0.60-0.83) on the original quality, 0.68 (0.55-0.80) (p=0.47) for the lower-acceptable quality, and 0.61 (0.49-0.74) (p=0.06) for the unacceptable quality. For system B, the AUC decreased from 0.95 (0.90-1.0) to 0.91 (0.84-0.96) (p=0.25) and to 0.87 (0.78-0.95) (p=0.02), respectively. Radiologists gave the same recall decision in 83% and 82% of the cases for each quality level. Meanwhile, system A gave the same recall decision in 75% (p=0.06) and 68% (p=0.001) of the cases and system B in 80% (p=0.47) and 78% (p=0.27) of the cases.
**Conclusion:** Image quality can affect AI performance and recall decision more than radiologists', even when radiologists' performance is not affected.
**Limitations:** Retrospective study with limited sample size.
**Funding for this study:** aiREAD financed by the Dutch Research Council (NWO), Dutch Cancer Society (KWF), Health Holland (HH).
**Ethics committee - additional information:** Approval of the etics committe was not applicable due to the retrospective nature of this study with anonymized data that was previously approved for retrospective use.
**Author Disclosures:**
Mireille Broeders: Speaker: Hologic and Siemens Healthcare Research/Grant Support: Hologic, Screenpoint Medical, Sectra Benelux, Volpara Healthcare, Lunit, and iCAD
Sarah Delaja Verboom: Nothing to disclose
Ioannis Sechopoulos: Research/Grant Support: Siemens Healthcare, Canon Medical Systems, ScreenPoint Medical, Sectra Benelux, Volpara Healthcare, Lunit Advisory Board: Koning Corp. Speaker: Canon, Siemens Healthcare
Joana Maria Dos Santos Boita: Employee: After completing this project employee at Canon Medical Systems Europe

### Evaluation of a Digital Breast Tomosynthesis Cancer Detection AI Algorithm Using the Personal Performance in Mammographic Screening Scheme (PERFORMS)

G. Partridge[1], P. Phillips[2], J. James[1], N. Sharma[3], K. Satchithananda[4], R. Butler[5], J. Lewin[5], M. Michell[4], *Y. Chen*[1]; [1]Nottingham/UK, [2]Lancaster/UK, [3]Leeds/UK, [4]London/UK, [5]New Haven, CT/US
*(yan.chen@nottingham.ac.uk)*

**Purpose or Learning Objective:** To compare the performance of a Digital Breast Tomosynthesis (DBT) Artificial Intelligence (AI) model as a standalone reader to that of a large cohort of breast imaging readers, using the Personal Performance in Mammographic Screening (PERFORMS) scheme. The performance of a subset of readers, assisted by the DBT AI during image interpretation, will also be reported.
**Methods or Background:** 75 challenging combined DBT and Synthetic 2D mammography (S2D) screening cases were collated into a PERFORMS test-set. Test-set images were analysed by a prototype server allowing batch-processing of a commercial AI model (Hologic Genius AI Detection [GAID] v2.0). The set was also distributed to 156 readers from 8 UK National Health Service (NHS) hospitals that use DBT in screening as part of the PROSPECTS trial, and to 6 readers from 1 US institution that employs DBT in routine screening. The AI performance will be benchmarked against the performance of this reader cohort. The US readers will additionally re-review the test-set

Thursday with AI-markup available for decision support, following a 6-8 week washout period. Performance with and without AI-support will be investigated and compared to the AI as a standalone reader.
**Results or Findings:** The AI model achieved an Area Under the Receiver Operating Characteristic Curve (AUC) of 0.935, and a sensitivity of 89.5% and specificity of 85.7% at the optimal threshold (=33). Human readers are currently undertaking the case review, but their data will be reported at the conference.
**Conclusion:** This international, Multiple Reader Multiple Case (MRMC) study enables the comparison of a very large cohort of breast imaging readers to a DBT AI model, as well as investigating the affect of reading DBT with AI-support.
**Limitations:** The test-set is enriched with malignant cases which may influence human reader decisions.
**Funding for this study:** Funding was acquired from Hologic Inc.
**Ethics committee - additional information:** This study is classed as a clinical audit for quality assurance for improvement of the breast screening programme. Ethics Reference No: 88-1223.
**Author Disclosures:**
Nisha Sharma: Nothing to disclose
Reni Butler: Nothing to disclose
Keshthra Satchithananda: Nothing to disclose
Michael Michell: Nothing to disclose
George Partridge: Nothing to disclose
Peter Phillips: Nothing to disclose
John Lewin: Nothing to disclose
Jonathan James: Nothing to disclose
Yan Chen: Nothing to disclose

### Evaluation of an AI System for Cancer Detection in Abbreviated Breast MRI

*K. Eppenhof*[1], A. Rodriguez Ruiz[1], W. B. Veldhuis[2], C. Van Gils[2], A. M. Rosanò[3], R. Yang[4], D. E. Lehrer[5], L. Çelik[6], R. Mann[1]; [1]Nijmegen/NL, [2]Utrecht/NL, [3]Sion/CH, [4]East Brunswick, NJ/US, [5]Buenos Aires/AR, [6]Istanbul/TR
*(koen.eppenhof@screenpointmed.com)*

**Purpose or Learning Objective:** To investigate the performance of an AI system for breast cancer detection in abbreviated DCE-MRI.
**Methods or Background:** A combination of high-risk screening and diagnostic DCE-MRI exams from five hospital groups and a public data set (Duke-Breast-Cancer-MRI) were acquired. Each MRI exam was processed by an AI system, which takes as input the pre-contrast and a single post-contrast T1 image (abbreviated breast MRI), detects suspicious regions, and outputs a malignancy score per breast between 1 and 10. Additionally, the AI system was evaluated on an enriched screening dataset from the DENSE trial.
**Results or Findings:** Area under the Receiver Operating Characteristic curve (AUROC) was computed for classifying exam malignancy for exams from four hospital groups located in Argentina (41 of 780 exams containing biopsy-proven cancer, AUROC 0.891 (95% CI=0.828-0.944)), Switzerland (98/3499, 0.863(0.824-0.896)), Turkey (33/164, 0.955(0.898-0.998)), and the US (153/1096, 0.904(0.877-0.929)). The consistency in AUROCs indicates robustness across populations, protocols, and scanners. Because Duke-Breast-Cancer-MRI exams all contain cancer, a breast-level analysis was done where breasts without cancer were used as the negative class (904/1808 breasts containing cancer). The AUROC (0.965(0.957-0.972)) is similar to an earlier published AI that used two post contrast images. For exams that had a BIRADS assessment, the agreement between the AI (score >= 9) and the radiologist interpretation (BIRADS 1 or 2 vs. 4 or 5) was found to be moderate (Cohen kappa=0.502(0.449-0.555)). The performance on screening-only data was measured in exams from the fifth hospital located in the Netherlands (66/2920 exams containing cancer, AUROC 0.812(0.753, 0.868)), and exams from the DENSE trial (83/517, AUROC 0.803(0.747-0.856)).
**Conclusion:** A first evaluation of an AI system for abbreviated DCE-MRI shows potential for decision support in detecting breast cancer.
**Limitations:** The study has a retrospective design.
**Funding for this study:** Not applicable
**Ethics committee - additional information:** Not applicable
**Author Disclosures:**
Carla Van Gils: Nothing to disclose
Alejandro Rodriguez Ruiz: Employee: ScreenPoint Medical
Koen Eppenhof: Employee: ScreenPoint Medical
Daniel Eduardo Lehrer: Nothing to disclose
Wouter B. Veldhuis: Nothing to disclose
Levent Çelik: Nothing to disclose
Roger Yang: Nothing to disclose
Anna Maria Rosanò: Nothing to disclose
Ritse Mann: Advisory Board: ScreenPoint Medical

### Validating the impact of real-world live use of AI as an additional reader in breast cancer screening (BCS)

*A. Ng*[1], E. Ambrozay[2], E. Szabó[2], B. Glocker[1], P. Kecskemethy[1]; [1]London/UK, [2]Budapest/HU
*(Annie.ng@deephealth.com)*

**Purpose or Learning Objective:** To validate that the measured impact of deploying AI as an additional reader is a result of the AI intervention and not purely the result of additional reading.
**Methods or Background:** Live-use of an AI-system as an additional reader (XR) to flag cases for additional review that it suggested to recall but the standard double reading (DR) decision was "no recall", has been demonstrated to result in a 0.8/1000 increase in cancer detection rate (CDR) and a 0.1% increase in positive predictive value when 6.0% of cases are additionally reviewed, compared to DR. To validate that the increased effectiveness in early cancer detection of the XR AI-workflow is not purely from additional reading, the maximum CDR increase opportunity due to third-human-reading a random 6.0% of cases was simulated, calculated as interval cancer rate (ICR) times the portion of cases to be third-human-read (6.0%), times the portion of human-detectable interval cancers (ICs) i.e. visible on priors. A range of ICR of 0.84-2.11/1000 was used (DOI:10.1038/s41523-017-0014-x). Studies have measured that 22% of ICs are human-detectable (DOI:10.1007/s00330-020-07130-y), however, a wider range of 22-100% was used. Third-human-reading was assumed to have an unrealistic 100% sensitivity among human-detectable ICs.
**Results or Findings:** For the lower and upper end of assumptions, respectively, the maximum CDR increase opportunity calculated for third-human-reading is 0.01 and 0.13/1000, which is 98.9% and 87.2% less than the XR AI-workflow, suggesting that the increased CDR impact of XR is 6-70 times more effective than third-human-reading without AI.
**Conclusion:** Simple simulations show that CDR improvements from third-human-reading a random set of cases would be marginal, validating that the substantial CDR increase demonstrated by XR is a direct effect from using AI to flag cases for additional review.
**Limitations:** Single AI assessed
**Funding for this study:** Kheiron Medical Technologies
**Ethics committee - additional information:** Not required
**Author Disclosures:**
Annie Ng: Nothing to disclose
Ben Glocker: Employee: Kheiron Medical Technologies Ltd
Eva Ambrozay: Nothing to disclose
Peter Kecskemethy: CEO: Kheiron Medical Technologies Ltd
Endre Szabó: Nothing to disclose

### Adding artificial intelligence (AI) case scoring in a breast screening programme to optimize reading workflow and workload: a retrospective study

*A. Nitrosi*, R. Vacondio, L. Verzellesi, M. Creola, M. Bertolini, P. Giorgi Rossi, V. Iotti, P. Pattacini, C. Campari; Reggio Emilia/IT
*(nitrosi.andrea@ausl.re.it)*

**Purpose or Learning Objective:** The objective of this study was to retrospectively evaluate a strategy to optimize reading workflow and readers' workload based on the iCAD Case Malignancy Score (CMS).
**Methods or Background:** We analyzed 122,216 2D mammography screening reading times (RT) corresponding to 61,108 exams including 244 proven tumours, consequentially acquired in Reggio Emilia Breast Screening Program (BSP) starting from January 2023 to June 2024 and elaborated by iCAD Inc. ProFound AI 2D system. ICAD Case Malignancy Scores (CMS) represents the relative confidence that a case is malignant on a scale of 0% to 100%. A pool of radiologists performs blinded double reading plus arbitration framed in work-shift. Packs are assigned to a reader respecting a numerical criterion of maximum readings per work-shift. We analyzed the correlations (Spearman) between the RT of individual readers (normalized on the personal median) with the CMS and the breast density (D). The analysis was repeated considering only the women recalled / not recalled / true positive (TP).
**Results or Findings:** A positive correlation was demonstrated between CMS and RT (R = 0.76) and slightly between D and RT (R = 0.52), overall and in recalled and non-recalled women separately. Using CMS, packs could be optimized based on individual reader characteristics to maximize the number of exams for each reader's pack with constant recall rate (and TP): first simulations show up to 14% increase in the number of exams read over 4 hours effective reading period.
**Conclusion:** This scenario would not undermine the reading screening workflow while ensuring resource optimization nor introduce any cognitive bias influencing the readers since each session would have similar expected recall and detection rate.
**Limitations:** Cases refer only to Reggio Emilia BSP, limiting this study.
**Funding for this study:** This study was partially supported by the Italian Ministry of Health - Ricerca Corrente

**Ethics committee - additional information:** Compliance with Ethical Standards Institutional Review Board approval was not required because it is a Clinical Audit about a technical development. This study was conducted in accordance with the routine quality assurance procedures established by the Local Health Authority for its screening programmes. The Reggio Emilia Cancer Registry, which routinely collects the screening history of each case of breast cancer, has been approved by the Provincial Ethics Committee.
**Author Disclosures:**
Laura Verzellesi: Nothing to disclose
Valentina Iotti: Speaker: Invited speaker
Cinzia Campari: Nothing to disclose
Marco Bertolini: Nothing to disclose
Pierpaolo Pattacini: Speaker: Invited speaker
Andrea Nitrosi: Speaker: Invited speaker
Rita Vacondio: Speaker: Invited speaker
Paolo Giorgi Rossi: Nothing to disclose
Martina Creola: Nothing to disclose

**Assessment of an AI-system in indicating breast laterality for screen-detected and interval cancers in breast screening in a large-scale retrospective study**
A. Ng[1], C. Oberije[1], G. Fox[1], R. Currie[2], A. Redman[3], A. Leaver[3], W. Teh[4], B. Glocker[1], *P. Kecskemethy*[1]; [1]London/UK, [2]Exeter/UK, [3]Gateshead/UK, [4]Harrow/UK
*(peter@kheironmed.com)*

**Purpose or Learning Objective:** Assess the utility of an AI-system in indicating breast laterality in breast screening.
**Methods or Background:** Employing a commercially available AI-system as an independent reader, utilising its case-wise recall suggestions, within double reading has previously been shown to maintain/improve screening performance, while providing substantial workload savings. This has been demonstrated in a large-scale retrospective clinical study (306,839 cases from 236,739 participants between 2017-2021), involving three Hologic sites across the UK's major genetic clusters (South-East/West/North), including more diverse ethnicities in London. To further assess the AI-system's utility in supporting follow-up investigations for recalls, its breast laterality recommendation for screen-detected cancers (SDCs) and interval cancers (ICs) were compared to pathology information.
**Results or Findings:** The study included 2592 SDCs and 379 ICs. The AI-system correctly recalled 2304 SDCs (88.9% sensitivity) and 152 ICs (40.1% IC detection rate). Among the correctly recalled SDCs, the AI-system: A) indicated pathology-agreeing laterality in 84.5% (83.5% unilateral/1.1% bilateral), B) recalled unilateral cases as bilateral in 13.9%, C) recalled one side in bilateral cancer cases in 0.4%, and D) indicated the opposite side not assessed in 1.2%. The respective results for ICs were: A) 58.8% (58.1% unilateral/0.7% bilateral), B) 18.4%, C) 2.2%, and D) 20.6%. For category D, it is unknown if an early abnormality could be present as the AI-indicated side was not assessed by biopsy nor by additional diagnostic imaging. The AI-system provides screening utility in scenarios A-C, which comprises 98.8% for SDCs, 79.4% for ICs, 88.1% for ICs diagnosed within 1 year, and 97.1% for false negative ICs (FNICs).
**Conclusion:** The AI-system's laterality detection demonstrated utility in almost all SDCs/FNICs, and the large majority of ICs, showing it can support the clinical workflow with laterality information for follow-up assessments.
**Limitations:** Single AI assessed
**Funding for this study:** NIHR AI in Health and Care Award
**Ethics committee - additional information:** UK HRA REC reference: 21/HRA/4830
**Author Disclosures:**
Cary Oberije: Employee: Kheiron Medical Technologies Ltd
Annie Ng: Employee: Kheiron Medical Technologies Ltd
William Teh: Nothing to disclose
Ben Glocker: Employee: Kheiron Medical Technologies Ltd
Alice Leaver: Nothing to disclose
Georgia Fox: Employee: Kheiron Medical Technologies Ltd
Alan Redman: Nothing to disclose
Peter Kecskemethy: CEO: Kheiron Medical Technologies Ltd
Rachael Currie: Nothing to disclose

**Repurposed AI-Based Mammography Interpretation in Diverse Clinical Scenarios**
*H. Ngo*[1], J. Neubauer[1], A. L. Palacios Acedo[2], M. Windfuhr-Blum[1], E. Kotter[1], F. Bamberg[1], J. Weiß[1]; [1]Freiburg/DE, [2]Marseille/FR
*(helen.ngo@uniklinik-freiburg.de)*

**Purpose or Learning Objective:** This study evaluates the diagnostic performance of an artificial intelligence (AI) tool originally developed for screening mammography, now repurposed for use in various clinical scenarios, including diagnostic mammograms in 1) asymptomatic women, 2) symptomatic women and 3) patients with a personal history of breast cancer (PHBC).
**Methods or Background:** A total of 601 women with were retrospectively included and categorized into three subgroups: diagnostic mammograms of 1) asymptomatic women (n = 423), 2) symptomatic women (palpable abnormality, suspicious sonography, n=66) and 3) patients with PHBC (n =112). The AI-tool provided continuous scores (1 to 100) for potential malignancy, with histopathological confirmation and/or follow-up ≥2 years as reference standard.
**Results or Findings:** The AI-tool showed high performance across all three cohorts, with areas under the curve (AUC) for diagnostic mammograms of 1) asymptomatic women: 0.75 (95% CI: 0.51-0.98), 2) symptomatic women: 0.92 (95% CI: 0.81-1.0), and 3) patients with PHBC: 0.71 (95% CI: 0.52-0.90). Excluding women with extremely dense breasts (ACR D) increased the AUC for diagnostic mammograms of 1) asymptomatic women to 0.79 (95% CI: 0.41-1.0), 2) symptomatic women: 0.92 (95% CI: 0.81-1.0), and 3) patients with PHBC: 0.73 (95% CI: 0.51-0.95). Using a threshold of the highest 10% AI-scores to binarize the continuous AI-output resulted in sensitivity 0.92 and specificity 0.50 for subgroup 1); 0.96 and 0.77 for 2) and 0.81 and 0.67 for 3), respectively.
**Conclusion:** Repurposed AI-tools can enhance malignancy detection across diverse patient groups, especially in less dense breasts. Optimizing thresholds for specified populations, such as asymptomatic and symptomatic cohorts, may further improve AI's diagnostic effectiveness.
**Limitations:** Varying breast densities, particularly extremely dense breast, can pose detection challenges, and the sample size of 601 may influence the generalizability of the findings.
**Funding for this study:** Unrestricted research grant from Lunit.
**Ethics committee - additional information:** Approved by local IRB.
**Author Disclosures:**
Helen Ngo: Research/Grant Support: Lunit
Jakob Neubauer: Research/Grant Support: Lunit
Jakob Weiß: Research/Grant Support: Lunit
Ana Luisa Palacios Acedo: Employee: Lunit Europe
Fabian Bamberg: Research/Grant Support: Lunit
Marisa Windfuhr-Blum: Research/Grant Support: Lunit
Elmar Kotter: Research/Grant Support: Lunit

**Patient perceptions towards the use of artificial intelligence (AI) in breast cancer imaging**
D. Velazquez-Pimentel, S. Khan, T. Falco, S. Hickman, S. Dani, *T. Suaris*; London/UK
*(tamarasuaris@hotmail.com)*

**Purpose or Learning Objective:** The aim of this study is to evaluate patient perceptions towards the use of artificial intelligence (AI) in breast cancer imaging
**Methods or Background:** Women presenting to a single breast cancer unit in East London were invited to participate in a prospective survey. Baseline knowledge and attitude towards technology in daily living and attitude towards the use of AI in mammography screening was measured using a 4-point Likert scale. Demographic data including age, ethnicity, education was collected.
**Results or Findings:** 944 responses were analysed. Of these, 90% (n=853/944) expressed a preference for combined computer-physician reading with more women expressing confidence in the accuracy of combined computer-physician (93%, n=882/944) reading over computer reading alone (54% n=513/944). Self-reported understanding of technology was associated with a higher level of concern. In patients with limited understanding 46% expressed concern with regards to the accuracy of computer read mammograms compared to 38% in patients with expert understanding. Level of concern was not significantly associated with age, ethnicity or education level ($p > 0.05$). Regardless of level of concern, the majority of respondents expressed a positive opinion on the impact computer read mammograms can have on improving both efficiency (85%, n=798/944) and pick up rate (84%, n=797/944).
**Conclusion:** Despite confidence in the ability of AI to improve efficiency and pick up rate there is a strong preference expressed by patients towards combined computer-physician read mammograms. This study demonstrates that this remains true regardless of age, ethnicity or level of education. Level of concern is associated with self-reported understanding of technology; targeted patient education programs may support implementation of AI workflow in breast screening programs.
**Limitations:** Survey responses are subject to bias.
**Funding for this study:** No funding was received for this study.
**Ethics committee - additional information:** Patient Survey - local research lead confirmed no formal ethics application necessary
**Author Disclosures:**
Tamara Suaris: Nothing to disclose
Thamiris Falco: Nothing to disclose
Sarah Khan: Nothing to disclose
Diana Velazquez-Pimentel: Nothing to disclose

Sarah Hickman: Other: -SEH have research collaborations with Vara, ScreenPoint, Lunit, Google, Volpara, iCAD, Therapixel, CureMetrix, Sunnybrook Research Institute, and Massachusetts Institute of Technology. -SEH is a Radiology AI: Trainee Editorial Board member.
Shefali Dani: Nothing to disclose

**ADMEDVOICE – The Pathway to Polish Language Automatic Structured Reporting in Breast Ultrasound using Voice Recognition and Large Language Models**

*M. Bobowicz*, D. Szplit, A. Dąbkowska, J. Bogdan, K. Gwozdziewicz, J. Omernik, B. Graff, A. Czyżewski, K. Narkiewicz; Gdansk/PL
*(maciej.bobowicz@gumed.edu.pl)*

**Purpose or Learning Objective:** Breast ultrasound (BUS) equipped with the ACR BI-RADS lexicon is a well-described diagnostic procedure with mandatory fields and a relatively closed vocabulary. This study aims to generate BUS-structured reports automatically using voice recognition and topic modelling in Polish.
**Methods or Background:** A dataset of 6269 BUS radiology reports from the University Clinical Center's Hospital Information System covering 2013-2023 was obtained. The reports were created by more than ten experienced breast radiologists and multiple residents. They covered various clinical scenarios, including diagnosis, treatment, and follow-up tests in breast cancer, benign disease, mutation carriers, and studies without pathology.
**Results or Findings:** From 6269 reports, 48721 text fragments were obtained, representing specific parts of the BUS report used as training data. We identified specific 'topics' relating to 'ontologies' in these fragments. Topics represented parts of the radiologist's report that could be structured into subsections: 1) reference letter information, 2) tissue composition, 3) pathology descriptors (masses and calcifications), 4) associated features, 5) axillary and intramammary lymph node descriptors, 6) other special cases, 7) conclusions, 8) recommendations, 9) final remarks. For automatic text recognition BERTOPIC was explored. As a next step, we invited 25 specialist radiologists, residents, medical students and other HCPs to record 3328 separate sentences for voice recognition algorithms training.
**Conclusion:** The presented research, which involved topic modelling, is a first step towards creating Polish language automatic structured BUS reporting using voice recognition and LLMs. The resulting database with voice samples at three quality levels will be released soon. It will allow AI training to reduce the radiology reporting burden with more natural voice commands being transferred to structured reports.
**Limitations:** The single-centre design, restriction to the Polish language, and lack of external validation.
**Funding for this study:** Funding for the ADMEDVOICE Project was provided by the Polish National Centre for Research and Development; Infostrateg IV action; grant number: INFOSTRATEG-IV/003/2022.
**Ethics committee - additional information:** The study was approved by the Bioethics Committee for Scientific Research of Medical University of Gdansk.
**Author Disclosures:**
Andrzej Czyżewski: Nothing to disclose
Beata Graff: Nothing to disclose
Maciej Bobowicz: Nothing to disclose
Krzysztof Narkiewicz: Nothing to disclose
Katarzyna Gwozdziewicz: Nothing to disclose
Anna Dąbkowska: Nothing to disclose
Dariusz Szplit: Nothing to disclose
Julia Bogdan: Nothing to disclose
Justyna Omernik: Nothing to disclose

**Contrastive Learning in Breast MRI: MLIP as the Base Foundation Model**

*N. Rasoolzadeh*[1], T. Zhang[2], R. Mann[1]; [1]Nijmegen/NL, [2]Amsterdam/NL
*(nika.rasoolzadeh@gmail.com)*

**Purpose or Learning Objective:** To explore the potential of utilizing a contrastive language image pretraining approach for 3D breast MRI images.
**Methods or Background:** A dataset of 15005 pairs of dynamic contrast-enhanced (DCE) and subtraction MRI images with corresponding radiological reports from the Netherlands Cancer Institute were used for training a model to find the most similar image-text pairs by contrasting positive pairs (similar) against negative pairs (dissimilar) samples. Full MRI images and complete Dutch reports were utilized. The image and text embeddings were obtained using a 3D ResNet50 architecture and RadioLOGIC as the image and text encoders, respectively. Two inference scenarios were tested: image retrieval by text queries and BI-RADS prediction. The area under the curve (AUC) was used to evaluate the model's performance. The developed Multimodal Breast MRI Language-Image Pretrained (MLIP) model was first used for the zero-shot BI-RADS prediction task and was later fine-tuned.
**Results or Findings:** The preliminary results show an AUC of 0.717 (95% CI: 0.604, 0.824) for BI-RADS 4/5 abnormal MRI images retrieval, 0.640 (95% CI: 0.538, 0.740) for dense breast retrieval, and 0.601 (95% CI: 0.505, 0.698) for low background parenchymal enhancement (BPE) retrieval. In the second inference, the performance of MLIP was compared to that of a fine-tuned model. The fine-tuned model demonstrated improved accuracy, with a reduction in the number of originally benign cases misclassified as malignant.
**Conclusion:** In this study, a multi-modal breast MRI pretrained model was developed. The preliminary results suggest MLIP can be adjusted to perform diagnostic tasks and radiology report generations, holding the potential to serve as a foundation model for breast MRI analysis.
**Limitations:** The model needs to be validated on larger datasets and across more downstream tasks.
**Funding for this study:** Funding was provided by the ODELIA project (from the European Union's Horizon Europe research and innovation programme under grant agreement, No 101057091)
**Ethics committee - additional information:** This study did not require formal ethics committee approval, as it exclusively used fully anonymized MRI images and reports. No identifiable personal data was collected or used in the analysis. All MRI data was anonymized prior to access, ensuring that no individual participants can be identified from the data
**Author Disclosures:**
Tianyu Zhang: Nothing to disclose
Nika Rasoolzadeh: Nothing to disclose
Ritse Mann: Nothing to disclose

**Generating virtual T2w-fat-saturated breast MRI acquisition using neural-networks**

*A. Liebert*[1], D. Hadler[1], C. M. Ehring[1], H. Schreiter[1], F. B. Laun[1], M. Uder[1], E. Wenkel[2], S. Ohlmeyer[1], S. Bickelhaupt[1]; [1]Erlangen/DE, [2]Munich/DE
*(andrzej.liebert@uk-erlangen.de)*

**Purpose or Learning Objective:** Multi-parametric breast MRI protocols typically include T2-weighted fat-saturated(T2w-FS) sequences, which are used for tissue characterization. However, their acquisition can significantly increase scan time. This study aims to evaluate, whether a 2D-U-Net neural-network can generate virtual T2w-FS images(VirtuT2) from other acquisitions of a routine multiparametric breast MRI protocol.
**Methods or Background:** This IRB-approved, retrospective study included n=914 breast MRI examinations performed between January 2017 and June 2020 at University Hospital Erlangen. The dataset was divided into training(n=665), validation(n=74), and test(n=175) sets. 2D-U-Net was trained on T1w, DWI, and DCE sequences to generate VirtuT2 . Quantitative metrics and a qualitative multi-reader assessment by two radiologists were used to evaluate the VirtuT2 images. For qualitative readings radiologist were asked to identify, whether an image is original T2w-FS or VirtuT2 image, evaluate the diagnostic image quality(DIQ) and wheter they can identify presence of edema around the mass-lesions.
**Results or Findings:** VirtuT2 images demonstrated high structural similarity(SSIM=0.87) and peak signal-to-noise ratio(PSNR=24.90) compared to original T2w-FS images. High level of the frequency error norm(HFEN=0.87) indicates strong blurring presence in the VirtuT2 images, which was also confirmed in qualitative reading. Radiologists correctly identified VirtuT2 images with 92.3% and 94.2% accuracy, respectively. No significant difference in DIQ was noted for one reader(p=0.21), while the other reported significantly lower DIQ for VirtuT2(p<=0.001). Moderate inter-reader agreement was observed for edema detection on T2w-FS images(ꞵ=0.43), decreasing to fair on VirtuT2 images(ꞵ=0.36).
**Conclusion:** Neural-networks can technically generate VirtuT2 images with high similarity to real T2w-FS images, using T1w, DWI and DCE acquisitions, however blurring remains a limitation. Future investigations with different architectures and using larger datasets are needed to improve clinical applicability.
**Limitations:** Limited dataset from a single site was used. Qualitative reading was performed on just n=52 cases.
**Funding for this study:** This project is funded by the Bavarian State Ministry of Science and the Arts in the framework of the bidt Graduate Center for Postdocs. L.B. is funded by the DFG Grant No: 518689644
**Ethics committee - additional information:** The study protocol was approved by the ethics committee of the Friedrich-Alexander Universität Erlangen-Nürnberg. The ethics comitee waived the need for informed consent.
**Author Disclosures:**
Sebastian Bickelhaupt: Research/Grant Support: Siemens Healthineers
Evelyn Wenkel: Nothing to disclose
Dominique Hadler: Nothing to disclose
Frederik Bernd Laun: Nothing to disclose
Michael Uder: Research/Grant Support: Siemens Healthineers
Hannes Schreiter: Nothing to disclose
Sabine Ohlmeyer: Nothing to disclose
Andrzej Liebert: Nothing to disclose
Chris Matthias Ehring: Nothing to disclose

**08:00-09:30** **Research Stage 4**

### Research Presentation Session: Neuro

### RPS 711

### Stroke and neurovascular imaging: inside and outside of the blood vessels

**Moderator**
E. Papadaki; Iraklion/GR
*(fpapada@otenet.gr)*

**Advancing Neurovascular Imaging with Ultra-High-Resolution Photon-Counting Detector CT: Optimization of Reconstruction Kernel and Quantum Iterative Reconstruction**

*A. Toth*, Y. (. Cho, E. Wilson, J. Crow, E. Bass, J. Joyce, M. G. Matheus, S. Tipnis, M. V. Spampinato; Charleston, SC/US
*(adrienntoth706@gmail.com)*

**Purpose or Learning Objective:** Our goal was to identify the optimal combination of dedicated neurovascular reconstruction kernels and quantum iterative reconstruction (QIR) levels for ultra-high-resolution (UHR) photon-counting detector (PCD)-CT angiography of the head and neck.
**Methods or Background:** 18 patients with intracranial aneurysms were prospectively included in this study. CT angiograms were obtained in UHR mode using a clinical dual-source PCD-CT scanner. Images were reconstructed with a slice thickness of 0.2 mm, utilizing six strength levels of a dedicated neurovascular kernel (Hv48/Hv56/Hv64/Hv72/Hv80/Hv89) and four levels of QIR (1-4). We assessed image noise, signal-to-noise ratio (SNR), contrast-to-noise ratio (CNR), and vessel sharpness for all reconstructions.
**Results or Findings:** With higher kernel sharpness and lower QIR, image noise continuously increased. The best performing reconstructions in terms of CNR were Hv48 and Hv72 in combination with QIR-4. Vessel sharpness improved with higher kernel levels, reaching a plateau with the Hv64 and Hv72 reconstructions, as observed in the small intracranial arteries (maximum ΔHU values of 260.59 and 255.11, respectively). Based on the results of the quantitative analysis, the kernels identified as the top performers and selected for further evaluation in the qualitative analysis were Hv56, Hv64, and Hv72.
**Conclusion:** Vessel sharpness increased with higher kernels levels, reaching a plateau at Hv64 and Hv72. Overall, Hv56, Hv64 and Hv72 were recognized as the best performing kernels based on the quantitative results. In the following steps, qualitative image quality evaluation will be conducted by three readers using a 5-point Likert scale. This evaluation will focus on the best-performing kernels in combination with the available QIR levels to assess overall image quality and diagnostic performance.
**Limitations:** The limitations of the study are the relatively small patient cohort and the fact that quantitative measurements were performed by a single observer.
**Funding for this study:** Funding was provided by Siemens Healthineers (research grant).
**Ethics committee - additional information:** The study was approved by the Institutional Review Board of the Medical University of South Carolina (Pro00123327)).
**Author Disclosures:**
Eric Bass: Nothing to disclose
Yongjoo (Jennifer) Cho: Nothing to disclose
John Crow: Nothing to disclose
Evan Wilson: Nothing to disclose
Adrienn Toth: Nothing to disclose
Maria G. Matheus: Nothing to disclose
Jennifer Joyce: Nothing to disclose
Sameer Tipnis: Nothing to disclose
M. Vittoria Spampinato: Research/Grant Support: Siemens Healthineers

**Performance of Dual-Layer Spectral Detector Non-contrast Computed Tomography in Identifying Early Ischemic Changes in Acute Ischemic Stroke Patients**

*Y. Wang*, H. Zhu, J. Wen, S. Ma, S. Yang; Beijing/CN
*(wangyujie619@126.com)*

**Purpose or Learning Objective:** The study aimed to evaluate the effectiveness of non-contrast dual-layer spectral computed tomography (DLCT) for detecting early ischemic changes in patients with acute ischemic stroke (AIS).
**Methods or Background:** NCCT is a common imaging technique for suspected AIS patients. This study involved 27 AIS patients who underwent both DLCT and MRI within 12 hours of symptom onset. A retrospective analysis was conducted on the imaging data, focusing on quantitative measurements from regions identified as acute infarction on diffusion-weighted MRI. Various parameters, including conventional CT values, virtual monoenergetic (monoE) CT values, and electron density relative to water (EDW) were compared between ischemic and normal brain. Statistical analyses, including the Mann-Whitney U test and ROC curve analysis, were performed to assess the diagnostic performance of these parameters. Z test was performed to compare the ROC curves of different parameters.
**Results or Findings:** This study analyzed 59 lesions in 27 patients to evaluate the diagnostic performance of various spectral parameters. The study found that EDW had the highest area under the curve (AUC) of 0.957, with an sensitivity of 95%, specificity of 85%. MonoE at 100 keV achieved an AUC of 0.955, with high sensitivity (95%) and negative predictive value (94%). In contrast, monoE at 40 keV showed the lowest performance, with an AUC of 0.701 and sensitivity of 66%. Conventional CT images had an AUC of 0.887, comparable to monoE at 70 keV (0.910). Statistically significant differences were noted between the AUCs of EDW/100 keV and conventional CT, while no significant difference was found between EDW and 100 keV.
**Conclusion:** Our findings indicated that EDW and monoE CT images obtained from DLCT can improve the detection of AIS compared to conventional non-contrast CT imaging.
**Limitations:** The sample size was small.
**Funding for this study:** None
**Ethics committee - additional information:** This retrospective study was approved by the local Ethics Committee of Civil Aviation General Hospital (2024-L-K-122).
**Author Disclosures:**
Jing Wen: Nothing to disclose
Haifeng Zhu: Nothing to disclose
Yujie Wang: Nothing to disclose
Shanrui Ma: Nothing to disclose
Shan Yang: Nothing to disclose

**DTI-derived Perivascular Space Diffusion Index could mirror the polarization of AQP4 following cerebral ischemia**

*X. Hao*, J. Tian, Z. Yao; Shanghai/CN
*(haoxiaozhu123@sohu.com)*

**Purpose or Learning Objective:** The purpose of this study was to investigate the dynamic changes of the perivascular space diffusion index (ALPS) and its correlation with aquaporin 4 (AQP4) polarization following cerebral ischemia in rats, using advanced diffusion tensor imaging (DTI) technique.
**Methods or Background:** Rats were divided into a normal group (n=5) and an ischemic group (n=25). The ischemic group underwent transient middle cerebral artery occlusion (tMCAO) and was further subdivided into five subgroups (n=5 each) based on the time post-ischemia (1, 3, 7, 14, and 28 days). Rats underwent MRI scans, including DTI, T2-weighted imaging (T2WI), and susceptibility-weighted imaging (SWI). Subsequently, immunofluorescence staining for AQP4 and glial fibrillary acidic protein (GFAP) was performed. The ALPS index was analyzed based on T2W, SWI, and fractional anisotropy (FA) obtained by DTI post-processing. And regions of interest were selected on the ipsilateral periventricular area, the ipsilateral corpus callosum/cingulate area and their mirror areas of the contralateral side. The AQP4 polarization was analyzed by GFAP/AQP4 in the non-glial scar area around the infarction in cortex and striatum.
**Results or Findings:** ALPS indexes were markedly reduced in ischemic rats, particularly on the affected side, with a notable drop on day 1, then rising at days 14 and 28. AQP4 polarization mirrored this trend, falling initially, then rising significantly by days 14 and 28. The ALPS index closely aligns with AQP4 index fluctuations.
**Conclusion:** The DTI-based ALPS index mirrors changes in AQP4 polarization after stroke, dropping sharply in the hyperacute phase and recovering in the early chronic phase, which could be a useful biomarker for glymphatic pathway function following stroke.
**Limitations:** This experiment requires high consistency of the lesions.
**Funding for this study:** National Natural Science Foundation of China (No. 81801660) and the grant of National Natural Science Foundation of China (No. 82272061)
**Ethics committee - additional information:** Institutional Animal Care and Use Committee of Fudan University
**Author Disclosures:**
Xiaozhu Hao: Nothing to disclose
Zhenwei Yao: Nothing to disclose
Jiaqi Tian: Nothing to disclose

**A novel model to quantify blood transit time in cerebral arteries using ASL-based 4D magnetic resonance angiography with example clinical application in moyamoya disease**

A. Bhogal[1], S. Uniken Venema[1], *P. T. Deckers*[1], K. Van De Ven[2], M. Versluis[2], K. Braun[1], B. Van Der Zwan[1], J. Siero[1]; [1]Utrecht/NL, [2]Best/NL
*(p.t.deckers-3@umcutrecht.nl)*

**Purpose or Learning Objective:** Angiography is critical for visualizing cerebral blood flow in intracranial steno-occlusive diseases. Current 4D magnetic resonance angiography (MRA) techniques primarily focus on macrovascular structures, yet few have quantified hemodynamic timing. This study introduces a novel model to estimate macrovascular arterial transit time (mATT) derived from arterial spin labeling (ASL)-based 4D-MRA. We provide examples of our method that visualize mATT differences throughout the brain of patients with intracranial steno-occlusive disease (moyamoya), as well as changes in mATT resulting from the cerebrovascular reactivity (CVR) response to acetazolamide (ACZ).
**Methods or Background:** The study population consisted of twelve patients with intracranial steno-occlusive disease, with a clinical indication to undergo hemodynamic imaging with an ACZ challenge to measure CVR. CVR is measured using multi-PLD ASL-MRI, acquired pre- and post-ACZ, and includes a four-dimensional dynamic MRA sequence using an ASL-scheme. The scan indications varied, but mostly involved cerebrovascular hemodynamic evaluation before or after neurosurgical intervention.
**Results or Findings:** We provide examples of our method that visualize mATT differences throughout the brain of patients with intracranial steno-occlusive disease (moyamoya), as well as changes in mATT in response to an ACZ injection. Furthermore, we present a method that projects sparse arterial signals into a 3D native brain-region atlas space and correlates regional mATT with other hemodynamic parameters of interest, such as tissue transit time and CVR.
**Conclusion:** Our approach offers a non-invasive, quantitative assessment of macrovascular dynamics, which enhances the understanding of large-vessel and tissue-level hemodynamics and augment monitoring of treatment outcomes in steno-occlusive disease patients. This can directly be used in stroke trial stratifications and peri-procedural treatment monitoring. Furthermore, it sets the stage for more in-depth investigations of the macrovascular contribution to brain hemodynamics.
**Limitations:** Pilot data in a small subgroup of patients (n=12).
**Funding for this study:** This work was supported by the W.M. De Hoop Foundation, the Janivo Foundation and Friends of UMC Utrecht & Wilhelmina Children's Hospital, and an NWO VIDI grant awarded to A.A.B. (VI.Vidi.223.085). JCWS is supported by the Brain Center Young Talent Fellowship 2019 of the University Medical Center Utrecht, The Netherlands.
**Ethics committee - additional information:** The Medical Ethics Review Committee of the University Medical Centre Utrecht declared that the Medical Research Involving Human Subjects Act (WMO) did not apply to the present research since all study measures were part of routine clinical practice. All patients or their legal representative (i.e., parent or guardian) provided written informed consent to use their data. Healthy subjects were acquired under a sequence development ethical protocol, which was approved by the Medical Ethics Review Committee of the University Medical Centre Utrecht. Informed consent was given by each healthy subject.
**Author Disclosures:**
Kees Braun: Nothing to disclose
Bart Van Der Zwan: Nothing to disclose
Alex Bhogal: Nothing to disclose
Simone Uniken Venema: Nothing to disclose
Maarten Versluis: Other: Works for Philips Healthcare and provided the advanced ASL patch used in this work. No financial support was provided.
Jeroen Siero: Nothing to disclose
Pieter Thomas Deckers: Nothing to disclose
Kim Van De Ven: Other: Works for Philips Healthcare and provided the advanced ASL patch used in this work. No financial support was provided.

**The use and pitfalls of hemodynamic MRI using multidelay arterial spin labelling for intracranial steno-occlusive disease in clinical practice: a single-center experience**

*S. Uniken Venema*, P. Deckers, J. W. Dankbaar, B. Van Der Worp, J. Hendrikse, B. Van Der Zwan, K. Braun, A. Bhogal, J. Siero; Utrecht/NL
*(s.m.unikenvenema@umcutrecht.nl)*

**Purpose or Learning Objective:** The primary objective is to describe a clinically feasible advanced neuroimaging protocol developed at an academic medical center that uses multi-delay arterial spin labeling (ASL) and blood oxygen level dependent (BOLD)-MRI with acetazolamide. This protocol is designed to assess cerebrovascular reactivity (CVR) in patients with intracranial steno-occlusive disease (e.g. moyamoya), while avoiding the limitations of PET scans.
**Methods or Background:** Image acquisition on 3-Tesla MRI involves acetazolamide-augmented multi-delay ASL and dynamic BOLD, in addition to structural sequences. Image processing is done using customized MATLAB-based toolboxes. ASL-CVR is calculated by subtracting pre-acetazolamide cerebral blood flow (CBF) from post-acetazolamide CBF and additional hemodynamic maps, such as arterial transit time (ATT), are generated. Imaging interpretation includes assessment of scan quality and success of the hemodynamic challenge.
**Results or Findings:** Since 2018, approximately 100 patients were scanned using this protocol. Multi-delay ASL enables a more accurate assessment of CBF and CVR compared to single-delay ASL in patients with prolonged ATT owing to their stenosis, and enables quantifying ATT simultaneously – a useful marker in itself. While CVR assessment is primarily done using ASL-derived CVR maps, BOLD-CVR provided useful complementary information in some cases. A typical patient with an intracranial stenosis experiencing ischemic symptoms will demonstrate lower baseline CBF, lower CVR and prolonged ATT in the affected hemisphere.
**Conclusion:** Our multi-delay ASL-based protocol demonstrates clinical feasibility and utility, allowing detailed cerebral hemodynamic evaluations of individual patients that is useful for clinical decision-making. This work serves as a practical guide for clinicians and MRI experts seeking to implement these advanced imaging methods in their institutions.
**Limitations:** Potential pitfalls in imaging acquisition and interpretation, including motion artefacts, inadequate labeling, the effects of anesthesia on CVR and the uncertainties of acetazolamide-augmented BOLD, must be carefully considered.
**Funding for this study:** This work was supported by the W.M. De Hoop Foundation, the Janivo Foundation and Friends of UMC Utrecht & Wilhelmina Children's Hospital.
**Ethics committee - additional information:** NedMec (study number 21-406)
**Author Disclosures:**
Kees Braun: Nothing to disclose
Jan Willem Dankbaar: Nothing to disclose
Bart Van Der Zwan: Nothing to disclose
Alex Bhogal: Nothing to disclose
Pieter Deckers: Nothing to disclose
Bart Van Der Worp: Board Member: Past president at the European Stroke Organization Advisory Board: Liva Nova and Bayer Grant Recipient: Dutch Heart Foundation, Stryker, and the European Union
Simone Uniken Venema: Nothing to disclose
Jeroen Siero: Nothing to disclose
Jeroen Hendrikse: Nothing to disclose

**Predictive Value of Venous Outflow in SAH (PreViOS)**

*H. Briody*, J. Henry, R. Bruen, P. Mchugh, P. Rohan, M. Javadpour, P. Nicholson; Dublin/IE
*(hayleybriody@rcsi.ie)*

**Purpose or Learning Objective:** Favorable cortical venous outflow (VO) is linked to better outcomes in acute ischemic stroke. It's role in aneurysmal subarachnoid hemorrhage (aSAH) remains unclear. This study investigates the association between VO profiles and functional outcomes in aSAH.
**Methods or Background:** Patients with aSAH referred to a tertiary neurosurgery center between 2016 and 2023 were included if presentation computed tomographic angiography (CTA) demonstrated satisfactory venous system opacification. VO was assessed using the cortical vein opacification score (COVES). The primary outcome was poor functional outcome (Glasgow Outcome Scale [GOS] 1-3) at 90 days. Associations between COVES and outcomes were assessed using univariable and multivariable (adjusted for World Federation of Neurosurgical Societies [WFNS] grade) binomial regression.
**Results or Findings:** Of 675 patients with aSAH and available CTA, 204 (30%) met inclusion criteria. The median age was 54.2 years (range 12-85). 182 (89%) had favorable VO. No significant association was found between unfavorable VO and poor 90-day functional outcome (RR 0.78, 95% CI 0.38-1.58, p=0.48), even after adjusting for WFNS grade (RR 1.04, 95% CI 0.38-2.83, p=0.94). Unfavorable VO was not associated with poor outcome at discharge, need for cerebrospinal fluid diversion, or shunt dependence.
**Conclusion:** The study failed to demonstrate a link between venous outflow (as measured by COVES) and outcomes in aSAH. This is an important negative finding. It suggests that, unlike in ischemic stroke, venous outflow might not be a major determinant of outcome in aSAH. Prospective studies are needed to definitively assess the role of VO in aSAH.
**Limitations:** This retrospective, single-center study may be subject to selection bias due to the inclusion criteria requiring adequate jugular bulb opacification on CTA. The impact of delayed cerebral ischemia, a major determinant of outcome in aSAH, was not specifically assessed.
**Funding for this study:** None
**Ethics committee - additional information:** Institutional review board

**Author Disclosures:**
Patrick Nicholson: Nothing to disclose
Mohsen Javadpour: Nothing to disclose
Paul Mchugh: Nothing to disclose
Richard Bruen: Nothing to disclose
Hayley Briody: Nothing to disclose
Pat Rohan: Nothing to disclose
Jack Henry: Nothing to disclose

**Enhanced Detection of Cerebral Lesions in Cerebral Amyloid Angiopathy Using 7T MRI: Insights into Cognitive Correlation and Clinical Implications**

*D. Botta*, A. Cusin, L. Sveikata, K-O. Loevblad, F. T. Kurz; Geneva/CH

**Purpose or Learning Objective:** The aim of this study is to evaluate the detection of cerebral microbleeds (CMBs) and cortical microinfarcts using 7 Tesla (7T) MRI in patients with cerebral amyloid angiopathy (CAA) and to correlate these findings with cognitive performance as measured by the Montreal Cognitive Assessment (MoCA).
**Methods or Background:** 19 patients with probable or possible CAA were scanned using both 7T and 3T MRI. Imaging protocols at 7T included susceptibility-weighted imaging (SWI) for CMB detection with an in-plane resolution of 0.15x0.15mm, and 3D FLAIR and T1 MP2RAGE sequences for cortical microinfarcts detection. Cognitive function was assessed using the MoCA score. Statistical analyses were conducted to assess correlations between lesion burden and MoCA scores.
**Results or Findings:** 7T MRI detected 379 CMBs compared to 179 at 3T (average: 18.37±34.18 CMBs at 7T vs 9.53±15.03 at 3T). Additionally, 7T identified 27 cortical microinfarcts across 5 patients, while none were detected at 3T. MoCA scores ranged from 9 to 30 with a mean of 23.0±4.82. Weak correlations were found between the number of CMBs and MoCA scores (7T: r =-0.28; 3T: r =-0.24).
**Conclusion:** 7T MRI at high-resolution is superior to 3T for detecting CMBs and cortical microinfarcts in CAA patients. However, the weak correlation between lesion burden and cognitive decline suggests that other factors may also contribute to cognitive impairment in these patients.
**Limitations:** Not applicable.
**Funding for this study:** No funding was provided for this study.
**Ethics committee - additional information:** Written informed consent was obtained from all participants, the study was approved by the institutional review board of Geneva University Hospitals.
**Author Disclosures:**
Professor Karl-Olof Loevblad: Nothing to disclose
Lukas Sveikata: Nothing to disclose
Alexandre Cusin: Nothing to disclose
Felix T Kurz: Nothing to disclose
Daniele Botta: Nothing to disclose

**Diagnostic Performance of Low-Dose Cerebral CTA Images Using Artificial Intelligence Iterative Reconstruction for Differentiating intracranial Aneurysms and Infundibula**

H. Chen[1], S. Xu[2], G. Zhang[2], *J. Wang*[1], X. Yin[1]; [1]Baoding/CN, [2]Shanghai/CN
*(jianing0218@163.com)*

**Purpose or Learning Objective:** Intracranial aneurysms and infundibula with similar morphology and anatomical location are difficult to distinguish using low-dose cerebral CT angiography (CTA). This study evaluated the diagnostic performance of cerebral low-dose CTA with artificial intelligence iterative reconstruction (AIIR) for differentiating intracranial aneurysms and infundibula.
**Methods or Background:** Sixty-four patients (38 male, mean age 62.2 ± 12.5 years) with suspected intracranial aneurysms were prospectively enrolled. Each patient underwent routine-dose (RD) and low-dose (LD) cerebral CTA. The RD protocol used 100kVp, ref. 180mAs, and hybrid iterative reconstruction (HIR), whereas the LD protocol used 100kVp, ref. 30mAs, and AIIR. Two radiologists, blinded to scan/reconstruction parameters, independently detected aneurysms and infundibula on low-dose images. The diagnostic reports of RD CTA served as references. Diagnostic performance in differentiating aneurysms and infundibula was assessed using receiver operating characteristic (ROC) analysis, calculating sensitivity, specificity, positive predictive value (PPV), negative predictive value (NPV), accuracy, and area under the curve (AUC) with 95% confidence interval (CI).
**Results or Findings:** A total of 64 lesions were identified, including 44 aneurysms and 20 infundibula. Reader 1 detected 62 out of 64 lesions (96.9%) on low-dose images, while Reader 2 detected 61 out of 64 (95.3%). Two infundibula with sizes of 1.5 mm and 2.4 mm were missed by both readers, whereas one aneurysm of 1.8 mm was missed by Reader 2. In differentiating aneurysms and infundibula, the sensitivity, specificity, PPV, NPV, diagnostic accuracy, and AUC for Reader 1 were 97.72%, 100%, 100%, 94.74%, 98.39%, and 0.989 (95% CI: 0.963–1.015), while for Reader 2 they were 97.67%, 100%, 100%, 94.74%, 98.36%, and 0.988 (95% CI: 0.962–1.015).
**Conclusion:** The AIIR shows the potential in reducing the radiation dose of the cerebral CTA when diagnosing intracranial aneurysms and infundibula.
**Limitations:** Not applicable.
**Funding for this study:** the Key Research and Development Program of Hebei Province (grant number 202330604010017)
**Ethics committee - additional information:** This study was approved by the local institutional review board.
**Author Disclosures:**
Guozhi Zhang: Employee: at United Imaging Healthcare
Shijie Xu: Nothing to disclose
Jianing Wang: Nothing to disclose
Haoyan Chen: Nothing to disclose
Xiaoping Yin: Nothing to disclose

**Temporal muscle trophicity as a prognostic factor for functional recovery in non-traumatic intracerebral hemorrhage**

*S. Nataf*[1], O. Curtinot[2], T. Maghfour[2], G. Boulouis[2], A. Aignatoaie[1], C. Ozsancak[1], M. Pasi[2], C. Cohen[1]; [1]Orleans/FR, [2]Tours/FR
*(snataf98@gmail.com)*

**Purpose or Learning Objective:** Intracerebral hemorrhage (ICH) is associated with poor outcome. Identifying patients with higher risk of disability is a key feature of optimal care. Recently, temporal muscle thickness (TMT) has been shown to predict ability after ischemic stroke. We explored the relationship between temporal muscle trophicity and the functional ability of non-traumatic ICH patients.
**Methods or Background:** Patients with acute non-traumatic ICH (2021-2022) from two university centers were retrospectively included. Imaging ICH parameters were retrieved (e.g. volume, location). Temporal trophicity was assessed through thickness (TMT), area (TMA) and density (TMD) on baseline brain computed tomography (CT). Good prognosis at 3-6 months was defined as a modified Rankin Scale (mRS)<3. Association between TMT, TMA and TMD and 3-6months mRS was evaluated using student T-test.
**Results or Findings:** Among 453 ICH patients, 216 with both CT and 3-6months mRS were analyzed (49% women, 70±15.9y). Mean hemorrhage volume was 47.3±46mm$^3$, mainly lobar (46%) and located in basal ganglia (41%). Mean TMT was 7.32±2.70mm in the good prognosis group, vs 6.37±2.13mm in the poor prognosis group (p=0.005). Mean TMA was 408±203mm$^2$ in the good prognosis group, vs 343±161mm$^2$ in the poor prognosis group (p=0.01). Prognosis was not significatively associated with TMD (p=0.051). 3-6months mRS was associated with hemorrhage volume (p=0.01).
**Conclusion:** To our knowledge, this is the first study to analyze and demonstrate that temporal muscle trophicity serves as a prognostic factor in intracerebral hemorrhage (ICH). Temporal muscle thickness (TMT) is a simple and practical indicator for assessing overall health and guiding patients toward targeted rehabilitation.
**Limitations:** Since many patients underwent only MRI, further research is needed to evaluate the association between temporal muscle trophicity and prognosis using this imaging modality.
**Funding for this study:** No funding was received for this study.
**Ethics committee - additional information:** This study was approved by a Comité de protection des personnes (CPP).
**Author Disclosures:**
Olivier Curtinot: Nothing to disclose
Marco Pasi: Nothing to disclose
Simon Nataf: Nothing to disclose
Grégoire Boulouis: Nothing to disclose
Andreea Aignatoaie: Nothing to disclose
Canan Ozsancak: Nothing to disclose
Clara Cohen: Nothing to disclose
Tasnym Maghfour: Nothing to disclose

**3D variable flip angle turbo spin echo black-blood MRI for diagnosing cerebral venous thrombosis: a systematic review and meta-analysis**

*A. Akhavi Milani*; Tabriz/IR
*(ali.akhavi.milani@gmail.com)*

**Purpose or Learning Objective:** To evaluate the performance of 3D variable flip angle turbo spin echo black-blood MRI (BB-MRI) in diagnosing cerebral venous thrombosis (CVT). The secondary objectives included, comparing BB-MRI with conventional MRI, MRV, MPRAGE, and SWI in diagnosing CVT, and assessing the utility of BB-MRI in estimating thrombus age.
**Methods or Background:** This study was registered in PROSPERO [ID: blinded]. The PubMed/MEDLINE, Web of Science, Scopus, and Embase databases were systematically searched and studies were selected based on predefined eligibility criteria. The risk of bias was assessed using the QUADAS-2 tool. Meta-analysis was performed to calculate pooled sensitivity, specificity, and AUC.

**Results or Findings:** Nine studies were included in the review. Two of them insufficiently reported the quantitative data; therefore, seven studies involving 176 CVT patients and 217 controls, encompassing 610 thrombosed and 3,279 normal cerebral venous segments were included in the meta-analysis. The studies demonstrated a high bias risk in the patient selection and reference standard domains. The pooled sensitivity and specificity of BB-MRI were 96% [95% CI: 92%–98%] and 96% [95% CI: 93%–98%] on a patient-based level, and 92% [95% CI: 87%–95%] and 98% [95% CI: 92%–99%] on a venous segment-based level, respectively. The AUC was 0.98 for patient-based data and 0.96 for venous segment-based data. For secondary objectives, a narrative summary indicated that BB-MRI outperforms conventional MRI, MRV, and MPRAGE. It also outperforms SWI in assessing cerebral cortical veins. Moreover, BB-MRI can prove beneficial in thrombus age estimation.
**Conclusion:** BB-MRI demonstrates significant potential in diagnosing CVT. Further comparative studies are required to specify its role in clinical decision-making for CVT.
**Limitations:** The small number of the retrieved studies.
**Funding for this study:** No funding was received for this study.
**Ethics committee - additional information:** This was a systematic review study for which ethics committee approval was waived.
**Author Disclosures:**
Ali Akhavi Milani: Nothing to disclose

**The probability of cerebral amyloid angiopathy according to the Simplified Edinburgh CT criteria in a large, unselected lobar intracerebral hemorrhage population**
*A. Hillal*[1], T. Ullberg[2], J. Wassélius[1]; [1]Lund/SE, [2]Malmö/SE
*(amirhilal13@gmail.com)*

**Purpose or Learning Objective:** Early identification of the underlying cause of intracerebral hemorrhage (ICH) is important for treatment and prognosis. This study aims to investigate the association of hematoma volume and other clinical parameters on the distribution of Cerebral Amyloid Angiopathy (CAA) probability according to the simplified Edinburgh CT criteria in a large, unselected intracerebral hemorrhage (ICH) population.
**Methods or Background:** Patients with spontaneous ICH residing in Skane county registered with clinical data in the Swedish Stroke Register 2016–2020 were included. Radiological parameters were evaluated using baseline non-contrast CT (NCCT) for categorization according to the simplified Edinburgh CT criteria by the presence of subarachnoid hemorrhage (SAH) and fingerlike-projections (FLP). Multivariable logistic regression analysis was used to determine factors associated with an increased (intermediate/high) CAA probability.
**Results or Findings:** Of 666 patients with lobar ICH, 190 (29%) had high CAA probability, 92 (14%) intermediate, and 384 (58%) low CAA probability. Patients with increased CAA probability more often presented with decreased level of consciousness, larger hematoma volumes, and had higher 90-day mortality. Female sex, age, and increasing baseline hematoma volume (Odds Ratio up to 30) were associated with increased odds of having an increased CAA probability.
**Conclusion:** We identified a strong association between baseline hematoma volume and an increased probability of CAA in lobar ICH patients on NCCT, indicating that large hematoma volumes per se may contribute to the occurrence of FLP and SAH, and act as a confounder for the Simplified Edinburgh CT Criteria. Validation against MRI is warranted.
**Limitations:** The lack of MRI studies to allow for the correlation between CT imaging characteristics and the gold imaging standard MRI Boston criteria
**Funding for this study:** ALF grants to Teresa Ullberg and Johan Wasselius, the Crafoord Foundation to JW, VINNOVA to Johan Wasselius, and by SUS Stiftelser & Fonder to Johan Wasselius.
**Ethics committee - additional information:** This study was approved, and individual informed consent was waived by the Swedish Ethical Review Authority (reference number 2020-06800).
**Author Disclosures:**
Teresa Ullberg: Nothing to disclose
Johan Wassélius: Nothing to disclose
Amir Hillal: Nothing to disclose

**Evaluation of a FLAIR Hyperintensity Algorithm for the prediction of DWI-FLAIR Mismatch in Acute Ischemic Stroke**
*C. M. Offersen*, J. Johansen, A. H. Brandt, T. C. Truelsen, A. Pai, S. Darkner, M. B. Bachmann Nielsen, J. F. Carlsen; Copenhagen/DK

**Purpose or Learning Objective:** Moderate inter-rater variability of Diffusion-Weighted Imaging (DWI) – Fluid-Attenuated Inversion Recovery (FLAIR) mismatch in wake-up stroke raises concerns about the potential exclusion of eligible patients for treatment with thrombolysis. A novel FLAIR algorithm has shown promising potential to perform this mismatch assessment but has only been evaluated on a small dataset. In the present study, we aimed to evaluate an updated version of the FLAIR algorithm for predicting the DWI-FLAIR mismatch in a large cohort of wake-up stroke patients.
**Methods or Background:** We conducted a single-centre, retrospective study. A consecutive cohort of patients suspected of wake-up stroke, who underwent MRI between 2019 and 2021 was included. Two radiologists and one resident, blinded to clinical data, manually assessed DWI-FLAIR mismatch according to the current clinically used binary categorisation. Cohens Kappa was calculated for the inter-rater agreement. The FLAIR algorithm depends on a DWI segmentation. We used a commercial DWI segmentation model and then tested the ability of the FLAIR algorithm on the identified ischemic lesions to predict manual mismatch. This was analysed with logistic regression test.
**Results or Findings:** The DWI model identified 495 patients with possible ischemic lesions. Manual radiological assessments found 365 of those patients to have actual ischemic lesions. Inter-rater agreement for binary DWI-FLAIR mismatch assessment was moderate ($\kappa = 0.461 \pm 0.028$SD). We found a high accuracy (82.3 ± 3.3SD). Low sensitivity (59.1 ± 9.1SD). Specificity (91.5 ± 4.2SD) and AUC (0.845 ± 0.03SD) were high in the automatic mismatch assessment.
**Conclusion:** The FLAIR algorithm predicted DWI-FLAIR mismatch status with a high AUC, which suggests the algorithm could provide a more standardized decision on mismatch, and reduce the inter-rater variability through an objective assessment to assist the radiologist.
**Limitations:** This was a single-centre, retrospective study.
**Funding for this study:** Innovation Fund Denmark
**Ethics committee - additional information:** Danish National Center for Ethics
**Author Disclosures:**
Thomas Clement Truelsen: Nothing to disclose
Cecilie Mørck Offersen: Grant Recipient: Received funding from IFD for part of the work focused on automated assessment in wake up stroke. This project was in collaboration with Cerebriu A/S.
Michael B Bachmann Nielsen: Nothing to disclose
Akshay Pai: Founder: Founder and CTO at Cerebriu A/S, a company that makes automated diagnostic software for radiology.
Jacob Johansen: Other: Earlier employment as an industrial PhD student at Cerebriu A/S
Andreas Hjelm Brandt: Nothing to disclose
Sune Darkner: Nothing to disclose
Jonathan Frederik Carlsen: Nothing to disclose

**10:00-11:00 — Research Stage 1**

### Research Presentation Session: Head and Neck

### RPS 808

### Imaging the skull base and face

**Moderator**
A. Bernaerts; Antwerp/BE
*(anja.bernaerts@zas.be)*

**Dual-layer spectral detector CT for differentiating middle ear cholesteatoma and chronic suppurative otitis media**
*S. Zhou*[1], L. Mei[1], H. Liu[1], X. M. Liu[2], J. Li[1]; [1]Changsha/CN, [2]Guangzhou/CN
*(zsy_amory1003@163.com)*

**Purpose or Learning Objective:** To compare the diagnostic performance of dual-layer spectral detector CT (DLCT) and high-resolution CT (HRCT) in differentiating middle ear cholesteatoma and chronic suppurative otitis media.
**Methods or Background:** This prospective, institutional review board-approved study included sixty-six patients who were preliminary diagnosed as cholesteatoma or otitis media, and received DLCT scanning before surgery. Thirty-three patients were finally diagnosed cholesteatoma based on intraoperative or pathological findings. Two blinded readers (Reader 1: one radiologist; Reader 2: one otologist) provided diagnoses and diagnostic confidence scores using a five-point scale, based on HRCT images and DLCT multi-parameter images, including virtual mono-energetic image at 40keV (VMI 40keV) and effective atomic number (Zeff). Diagnostic accuracy of HRCT and DLCT maps were compared using McNemar's test. Interobserver agreement was evaluated by Kappa statistic.
**Results or Findings:** HRCT and DLCT identified a total of 27/33 and 31/33 cholesteatomas by Reader 1, 19/33 and 30/33 cholesteatomas by Reader 2 respectively. The sensitivity, specificity, PPV, NPV, accuracy of HRCT and DECT by Reader 1 were 81.8, 75.8, 77.1, 68.2, 78.8% and 93.9, 69.7, 75.6, 92.0, 87.5%, by Reader 2 were 57.6, 90.9, 86.3, 68.2, 74.2% and 90.9, 75.8,

Thursday

78.9, 89.3, 83.3%, respectively. Compared to HRCT, the diagnostic sensitivity of DLCT increased for both readers, with a statistically significantly improvement in Reader 2 ($p < 0.05$). Diagnostic confidence scores of DLCT versus HRCT by Reader 1 and Reader 2 both improved significantly ($4.85 \pm 0.36$ vs $3.77 \pm 1.30$, $4.44 \pm 0.73$ vs $3.45 \pm 0.98$, respectively, all $p < 0.05$). Interobserver reproducibility was higher for diagnoses made with DLCT maps ($k = 0.717$) than for that made with HRCT images ($k = 0.495$).
**Conclusion:** Spectral CT improved diagnostic performance and interobserver reproducibility of determination of cholesteatoma versus otitis media.
**Limitations:** None
**Funding for this study:** None
**Ethics committee - additional information:** IRB of Xiangya Hospital
**Author Disclosures:**
Shuangyuan Zhou: Nothing to disclose
Xiao Min Liu: Nothing to disclose
Juan Li: Nothing to disclose
Lingyun Mei: Nothing to disclose
Heng Liu: Nothing to disclose

### Redefining Sinonasal Cancer response assessment to induction chemotherapy with tumor volumetry, results from two prospective multicentric trials: SINTART-1 and SINTART-2

P. Rondi[1], *E. Massoni*[1], A. Borghesi[1], P. Bossi[2], C. Resteghini[2], D. Farina[1], M. Ravanelli[1]; [1]Brescia/IT, [2]Milan/IT
*(e.massoni@unibs.it)*

**Purpose or Learning Objective:** Aim of this study is to identify the best response criteria in patients with sinonasal cancer undergoing induction chemotherapy (IC).
**Methods or Background:** Patients enrolled in SINTART-1 and SINTART-2 were included in this study. Unidimensional diameters (antero-posterior, AP; latero-lateral, LL; cranio-caudal, CC), maximum axial area (Amax) and volume (V) were performed on MRI by two radiologists. RECIST 1.1 assessment was included as a categorical variable. Variables were evaluated at baseline, after 1st chemotherapy cycle and at best response. Interobserver repeatability was analyzed. Stepwise univariable and multivariable Cox proportional-hazards regression models were used to correlate variables with Disease-Free Survival (DFS) and Overall Survival (OS).
**Results or Findings:** 60 patients were included in this study. Interobserver correlation at baseline and after 1st IC cycle was excellent for V (0.916 and 0.928 respectively), CC diameter (0.96 and 0,863) and AP diameter (0.846 and 0.796); good for LL diameter and moderate for Amax. RECIST 1.1 criteria after 1st IC cycle and at best response were not correlated with OS and DFS. Volume variation after 1st IC cycle and at best response was the variable most correlated with OS ($p<0.0001$and $p=0.002$) and DFS ($p<0.0001$and $p=0.005$). At the multivariable analysis V variation after 1st IC cycle and at best response resulted to be the only variable statistically associated with OS ($p<0.001$ and $p=0.0019$ respectively) and DFS ($p=0.0004$and $p=0.004$respectively).
**Conclusion:** Volume variation should be preferred to RECIST 1.1 and other measurements as objective radiological response in sinonasal cancer.
**Limitations:** The main limitation of this study is that despite considering two prospective studies the size of the cohort is small and this could reduce the statistical power.
**Funding for this study:** This study has received no funding.
**Ethics committee - additional information:** The ethics committee of participating centers have approved this study
**Author Disclosures:**
Elena Massoni: Nothing to disclose
Andrea Borghesi: Nothing to disclose
Paolo Bossi: Nothing to disclose
Davide Farina: Nothing to disclose
Paolo Rondi: Nothing to disclose
Marco Ravanelli: Nothing to disclose
Carlo Resteghini: Nothing to disclose

### Diagnostic accuracy of MRI for orbital and intracranial invasion of sinonasal malignancies: a systematic review and meta-analysis

*U. B. Abdullaeva*[1], B. Pape[2], J. Hirvonen[3]; [1]Tashkent/UZ, [2]Turku/FI, [3]Tampere/FI
*(umidasamira2@gmail.com)*

**Purpose or Learning Objective:** To review the diagnostic accuracy of MRI in detecting orbital and intracranial invasion of sinonasal malignancies using histopathological or surgical evidence as the reference standard.
**Methods or Background:** The systematic review protocol was pre-registered in the Prospective Register of Systematic Reviews (PROSPERO) under registration number CRD42024492090. A systematic search of the studies in English was conducted in PubMed and Embase, limited to articles published since 1990. We included studies that used preoperative MRI to detect intracranial and orbital invasion of sinonasal malignancies, using histological or surgical confirmation as the reference standard, and reported patient numbers in each class required for assessing diagnostic accuracy. The outcome measures were sensitivity, specificity, positive predictive value (PPV), and negative predictive value (NPV). Heterogeneity was assessed with the Higgins inconsistency test (I2).
**Results or Findings:** Seven original articles with 546 subjects were included in the review, six of these in the meta-analysis. Pooled overall accuracy for orbital invasion was higher at 0.88 (95% CI, 0.75-0.94) than for intracranial invasion - 0.80 (95% CI, 0.76-0.83). Meta-analytic estimates and their 95% confidence intervals were as follows for intracranial/orbital invasion: sensitivity 0.77 (0.69-0.83)/ 0.71 (0.40-0.90); specificity 0.79 (0.74-0.83)/0.91 (0.78-0.97); PPV 0.76 (0.64-0.85)/0.78 (0.61-0.88); and NPV 0.82 (0.72-0.89)/0.90 (0.63-0.98). Significant heterogeneity was observed in the Higgins inconsistency test (I2) for orbital invasion (84%, 83%, and 93% for sensitivity, specificity, and NPV, respectively).
**Conclusion:** MRI yielded moderate to high diagnostic accuracy for intracranial and orbital invasion, but there are limitations leading to false diagnoses. Loss of the hypointense zone on MRI predicts dural invasion. Infiltration of the extraconal fat beyond the periorbita is an MRI feature of orbital invasion.
**Limitations:** Limitations include a small number of predominantly retrospective studies, some with a small subset of patients.
**Funding for this study:** Funding was provided by the Sigrid Jusélius Foundation, grant number 240053.
**Ethics committee - additional information:** Since this is a systematic review, Institutional Review Board approval was not necessary.
**Author Disclosures:**
Bernd Pape: Nothing to disclose
Jussi Hirvonen: Nothing to disclose
Umida Bafoevna Abdullaeva: Nothing to disclose

### Advanced MRI Techniques for Evaluation of Sinonasal Masses: Exploring the Additive Utility

*M. Saini*, S. Manchanda, A. S. Bhalla, D. Kandasamy; New Delhi/IN

**Purpose or Learning Objective:** Sinonasal area is affected by a wide spectrum of benign and malignant tumours presenting with nonspecific symptoms and differentiation solely based on conventional magnetic resonance imaging has only limited specificity
To evaluate the role of newer imaging techniques like intravoxel incoherent motion (IVIM), diffusion kurtosis imaging (DKI) and dynamic contrast enhanced MRI (DCE-MRI) in differentiating benign and malignant sinonasal masses
**Methods or Background:** A prospective study was performed on 30 patients with sinonasal masses (18 malignant and 12 benign) who underwent routine MRI, DWI, IVIM and DCE MRI. Apparent diffusion coefficient (ADC) from diffusion weighted imaging, true diffusion coefficient (Dt), Pseudodiffusion coefficient (Dx), perfusion fraction (f) from IVIM, apparent kurtosis coefficient (Kapp) and apparent diffusion coefficient (Dapp) from DKI, semiquantitative and quantitative perfusion parameters from DCEMRI were measured and compared between two groups.
**Results or Findings:** The mean ADC, Dt and Dapp values were significantly lower in malignant sinonasal lesions than in benign sinonasal lesions with p values of 0.000, 0.015 and 0.030 respectively. The mean Kapp value was higher in malignant lesions than in benign lesions (p value of 0.001). There was no significant difference Dx, f and in semiquantitative and quantitative perfusion parameters.
**Conclusion:** The mean ADC derived from the DWI, Dt derived from IVIM, and Dapp & Kapp derived from the DKI can be used as a non-invasive method to differentiate benign and malignant sinonasal masses. Among these, ADC is the best parameter to differentiate, however there is no incremental role of DKI and IVIM over conventional DWI. The perfusion parameters showed no significant difference
**Limitations:** The sample size was small and a heterogeneous group of pathologies were included in the final analysis. In addition, the b values were set arbitrarily in the IVIM-DKI sequence.
**Funding for this study:** No funding was provided for this study
**Ethics committee - additional information:** Study was preapproved by the Institute Review Board (IRB) [Ref No: IECPG-487/25.08.2021].
**Author Disclosures:**
Manish Saini: Nothing to disclose
Ashu Seith Bhalla: Nothing to disclose
Smita Manchanda: Nothing to disclose
Devasenathipathy Kandasamy: Nothing to disclose

**Presence of bone exposure to the aerodigestive tract as an important imaging feature in patients with skull base osteomyelitis following prior irradiation for nasopharyngeal carcinoma**

*H. S. Leung*, K. K. F. Tsoi, Q-Y. H. Ai, A. D. King; Hong Kong/HK

**Purpose or Learning Objective:** Osteoradionecrosis is one the long-term complications associated with radiotherapy for nasopharyngeal carcinoma (NPC), and may result in the significant complications particularly skull base osteomyelitis (SBOM). A limited number of clinical case series have reported on SBOM and their predictors, imaging features and associations with other radiation-induced complications remain poorly understood. This study is to evaluate the imaging factors associated with SBOM in patients with previous irradiation for NPC.
**Methods or Background:** This is a retrospective matched case-control study, of patients with clinically proven SBOM and computer tomography (CT) scan performed at diagnosis selected as cases, while controls were selected within NPC post-RT patients and without SBOM, matched by initial staging and time from initial treatment. CT studies were reviewed for the presence of bone exposure to aerodigestive tract, bony sclerosis, bone loss and dehiscence and abscess formation; baseline demographics and clinical outcomes were analyzed by logistic regression and survival analyses.
**Results or Findings:** A total of 31 SBOM cases and 31 controls were analysed. Presence of bone exposure to aerodigestive tract is the only independent factor associated with SBOM (p<0.001 by McNemar’s test), while the degree of bone loss also shows borderline significance (p=0.052 by Wilcoxon sign rank test). Bone exposure to upper aerodigestive tract remains significant upon regression controlling for bone loss and staging of initial NPC. SBOM patients had worse survival with a higher incidence of other RT-related complications including carotid occlusion, blowout or RT-induced malignancy.
**Conclusion:** The presence of bone exposure to upper aerodigestive tract is an independent factor associated with SBOM, which could be helpful in early identification and treatment to avoid complications of SBOM which adversely affects survival.
**Limitations:** Recall bias from retrospective study.
**Funding for this study:** Nil
**Ethics committee - additional information:** This study has been approved by Joint CUHK-NTEC Clinical Research Ethics Committee; The Chinese University of Hong Kong (Reference number: CREC 2024.306)
**Author Disclosures:**
Kelvin Kam Fai Tsoi: Nothing to disclose
Ann Dorothy King: Nothing to disclose
Ho Sang Leung: Nothing to disclose
Qi-Yong Hemis Ai: Nothing to disclose

**Can HU analysis by used for jaw lesions differentiation?**

*C. Nadler*, Y. Pakanaev-Levi, H. Rushinek, N. Yavnai, Y. Zadik, I. Zeevi; Jerusalem/IL
*(Nadler@hadassah.org.il)*

**Purpose or Learning Objective:** Bone lesions demonstrated on Computed Tomography (CT) images may be differentiated by several methods including Hounsfield Unit (HU) analysis. However, the routine use of this method in pre-operative assessment of jaw lesions remains underexplored. We aimed to distinguish using HU analysis 3 types of jaw lesions.
**Methods or Background:** We retrospectively included pre-operative Multidetector CT (MDCT) scans of patients with unilocular hypodense jaw lesions, with histologically proven, non-inflame either odontogenic keratocyst OKC, central giant cell granuloma CGCG or unicystic ameloblastoma UA. Demographic data and anonymized DICOM files were retrieved. Two observers, blinded to the lesions’ diagnosis, measured three HU values for each lesion, in axial slices, on Philips IntelliSpace Portal software. Statistical analysis included intra and inter-observer reliability and validity evaluations as well as comparisons of mean HU values between the different lesions and between the same lesion in different jaws
**Results or Findings:** Our cohort included 30 jaw lesions (17 OKCs, 8 CGCGs and 5 UAs). Mean HU values for OKCs, CGCGs and UAs were 27.99±13.8, 70.68±46.3 and 31.38±7.4, respectively. Statistically significant difference was found between mean HU values of OKC, CGCG and UA (P=0.035). Following additional pooled analysis, mean HU values of CGCG was statistically higher than OKC and UA (<0.001). No statistically difference was found between HU values of different lesions in different jaws.
**Conclusion:** Pre-operative differentiation using HU analysis may be used to diagnose CGCG from OKC and UA. Future multi-center studies with additional types of lesions are needed to substantiate our results
**Limitations:** Our limitation included: small sample size, as is a result of stringent inclusion criteria and the transition of referrals from MDCT to CBCT and the fact that all cases were from a single medical center.
**Funding for this study:** None
**Ethics committee - additional information:** The study protocol was approved by the Institutional Review Board
**Author Disclosures:**
Yehuda Zadik: Nothing to disclose
Yehuda Pakanaev-Levi: Nothing to disclose
Itai Zeevi: Nothing to disclose
Nirit Yavnai: Nothing to disclose
Chen Nadler: Nothing to disclose
Heli Rushinek: Nothing to disclose

**Multiparametric magnetic resonance imaging in determining disease activity of thyroid-associated ophthalmopathy: Added value from dynamic contrast-enhanced and diffusion-weighted imaging**

*X-Y. Pu*, H. Hu, J. Zhou, L. Jinling, X-Q. Xu, F-Y. Wu; Nanjing/CN
*(pxyxxzy@163.com)*

**Purpose or Learning Objective:** To evaluate the performance of dynamic contrast-enhanced MRI (DCE-MRI) and diffusion-weighted imaging (DWI) in determining disease activity of thyroid-associated ophthalmopathy (TAO) and to establish their additional value for staging TAO compared to conventional T2-weighted imaging (T2WI).
**Methods or Background:** Seventy-two patients with TAO (48 active, 96 eyes; 24 inactive, 48 eyes) who underwent DCE, DWI and T2WI with fat suppression were prospectively enrolled. Simplified histogram parameters (mean, max, min) of DCE-MRI derived parameters (Ktrans, Kep, Ve), apparent diffusion coefficient (ADC) and signal intensity ratio (SIR) at extraocular muscles were calculated for each orbit and compared between the active and inactive groups. Multivariate analyses were used to identify independent predictors. Receiver operating characteristic curves analyses and DeLong tests were performed to evaluate and compare the performances of the identified significant imaging parameters and their combinations.
**Results or Findings:** Active TAO patients showed significantly higher mean and maximum Ve, higher minimum, mean and maximum ADC, higher minimum, mean and maximum SIR than inactive patients (P < 0.05). The mean Ve, mean ADC and mean SIR were found to be independent predictors for active TAO (all P < 0.05). Combination of mean Ve, mean ADC and mean SIR outperformed mean SIR alone in staging TAO (AUC, 0.839 vs 0.769, P = 0.016).
**Conclusion:** DCE-MRI and DWI could help to determine the disease activity of TAO. DCE-MRI-derived Ve and DWI-derived ADC values could provide added value to conventional T2WI-derived SIR in staging TAO
**Limitations:** First, the sample size was relatively small. More research should be done to increase the number of datasets to verify our findings. Second, only multiparametric MRI-derived simplified histogram metrics were analysed. Future studies integrating radiomics and machine learning have the potential to further improve staging performance.
**Funding for this study:** Jiangsu Province Hospital (the First Affiliated Hospital with Nanjing Medical University) Clinical Capacity Enhancement Project (JSPH-MC-2021-8 to Xiao-Quan Xu) , Jiangsu Province Capability Improvement Project through Science, Technology and Education (JSDW202243 to Fei-Yun Wu) and National Natural Science Foundation of China (NSFC) (81801659 to Hao Hu),
**Ethics committee - additional information:** This study was approved by the Institutional Review Board of the First Affiliated Hospital of Nanjing Medical University (IRB No.2021-SRFA-024)
**Author Disclosures:**
Fei-Yun Wu: Nothing to disclose
Jiang Zhou: Nothing to disclose
Lu Jinling: Nothing to disclose
Xiao-Quan Xu: Nothing to disclose
Hao Hu: Nothing to disclose
Xiong-Ying Pu: Nothing to disclose

**10:00-11:00** **Research Stage 2**

### Research Presentation Session: Musculoskeletal

### RPS 810

### Imaging in metabolic and inflammatory arthropathies

**Moderator**
T. Diekhoff; Berlin/DE
*(torsten.diekhoff@charite.de)*
**Author Disclosures:**
Torsten Diekhoff: Advisory Board: Eli Lilly, AbbVie, UCB; Speaker: Novartis, MSD, UCB, Janssen, Eli Lilly, Canon MS, Berlinflame, Bracco

**Evaluation of contrast-enhanced ultrasound for rheumatoid arthritis activity in patients who do not respond to second-line biologic therapy compared with superb microvascular imaging: first results**
*S. Lavalle*[1], A. Montana[2], Y. Dal Bosco[2], F. Aiello[3], R. Foti[2], G. Privitera[2], R. Foti[2], P. Romeo[2]; [1]Milan/IT, [2]Catania/IT, [3]Enna/IT
*(slavalle6@gmail.com)*

**Purpose or Learning Objective:** Detection of synovitis is essential for assessing rheumatoid arthritis (RA) activity and changing the therapy. This study aim to evaluate the level of agreement and correlation between DAS 28 (Disease activity score 28) and contrast-enhanced ultrasound (CEUS) and Superb microvascular imaging (SMI) in the classification of disease severity index in patients with RA who did not respond to second-line biologic therapy.
**Methods or Background:** SMI and CEUS were applied to 25 patients with active RA not respond to second-line biologic therapy. We evaluate the radiocarpal joint of both wrists. Differences in positive synovial vascularity (SV) and its semi-quantitative scale were observed, and the correlations of SMI and CEUS results with DAS-28.
**Results or Findings:** The results indicate that CEUS method shows high-moderate agreement with DAS 28 clinical method (Kappa = 0.406), 95% CI (0.1916, 0.5854), p = 0.00584, while SMI has weaker agreement (Kappa = 0.121) 95% CI (0.0098, 0.2466), p = 0.0846 (Kappa = 0.121). The correlation between CEUS and SMI is very strong (ρ = 0.828), CI 95% (0.6911, 0.9044), suggesting that the two radiological methods tend to produce very similar classifications, although CEUS is more in line with the gold standard. The Wilcoxon signed-rank test showed significant differences between DAS 28 and each of the two radiological methods, with CEUS appearing closer to the clinical method
**Conclusion:** Use of CEUS to detect vessels in the synovium and visualization of local SV is the method that most correlates with disease severity in relation to DAS 28 in patients with synovial arthritis who do not respond to second-line biologic therapy compared with SMI.
**Limitations:** Small sample size and the need for larger multicenter studies to confirm our findings.
**Funding for this study:** No funding
**Ethics committee - additional information:** Not applicable
**Author Disclosures:**
Placido Romeo: Nothing to disclose
Salvatore Lavalle: Nothing to disclose
Roberta Foti: Nothing to disclose
Fabio Aiello: Nothing to disclose
Rosario Foti: Nothing to disclose
Angelo Montana: Nothing to disclose
Giambattista Privitera: Nothing to disclose
Ylenia Dal Bosco: Nothing to disclose

**Role of imaging in inflammatory hand arthritis with diagnostic ambiguity: how complementary MRI findings in clinically established DIP arthritis may facilitate the specific diagnosis**
*Y. Yaraşir*, G. Ayan, H. Avci, L. Kılıç, Ü. Aydingöz, U. Kalyoncu, A. E. Yildiz; Ankara/TR
*(yasinyarasirmd@gmail.com)*

**Purpose or Learning Objective:** Ascertaining whether synovium or synovioentheseal complex (SEC) is predominantly involved and pattern of any degeneration would help radiologists in distinguishing inflammatory hand arthritis (IHA). We aimed to characterize the role of MRI in reaching specific diagnosis in IHA.
**Methods or Background:** Patients aged ≥18 years with suspicious IHA in at least one joint (but not treated other than with NSAIDs) were consecutively enrolled in this prospective study. 3T-MRI with a fine-tuned protocol was utilized, whereby differential diagnoses were made according to the predominant involvement of synovium or SEC, and/or specific degenerative findings. Physical examination, laboratory and imaging findings, treatment response, and already-established rheumatological classification criteria were used to reach final diagnosis.
**Results or Findings:** Of 80 patients initially enrolled, 57 (42 females; mean age, 54 [range, 28-79]) constituted the final group with eventual clinical diagnoses of 11 psoriatic arthritis (PsA), 14 rheumatoid arthritis (RA), 11 erosive osteoarthritis and/or calcium pyrophosphate dihydrate deposition disease, 21 arthritis with distal interphalangeal joint involvement (ADIPI) not otherwise classified into any group. MRI revealed no difference between PsA and ADIPI groups, except for nail-bed enthesitis. Comparison between PsA and RA disclosed that enthesitis (p=0.033) and periarticular soft tissue edema (p=0.042) were more frequent in PsA. When ADIPI and PsA groups were combined, enthesitis and periarticular soft tissue edema were more common than in other groups (p<0.001). Those with enthesitis were 24 times more likely to be in the PsA+ADIPI group than those without enthesitis (95% CI: 2.6–63.3). Accurate classification rate of the model was 83.7%, and area under the curve (AUC) value was 0.81.
**Conclusion:** SEC inflammation and periarticular edema on MRI are strong predictors of PsA, especially in patients with DIP arthritis who don't meet rheumatological classification criteria.
**Limitations:** Small sample size
**Funding for this study:** Funding was provided by Hacettepe University Scientific Research Projects Coordination Unit
**Ethics committee - additional information:** Our study was approved by Hacettepe University Clinical Studies Ethics Committee (2021/23-20)
**Author Disclosures:**
Umut Kalyoncu: Nothing to disclose
Gizem Ayan: Nothing to disclose
Levent Kılıç: Nothing to disclose
Adalet Elcin Yildiz: Nothing to disclose
Yasin Yaraşır: Nothing to disclose
Üstün Aydingöz: Nothing to disclose
Hanife Avci: Nothing to disclose

**Hemosiderin Quantification in Hemophilic Arthropathy of the Knee using Quantitative Magnetic Resonance Imaging**
*S. Sedaghat*[1], P. Leutz-Schmidt[1], J. Park[2], E. Fu[2], H. Jang[2]; [1]Heidelberg/DE, [2]Davis/US
*(samsedaghat1@gmail.com)*

**Purpose or Learning Objective:** This study aims to establish quantitative magnetic resonance imaging (qMRI) as a precise, noninvasive tool for evaluating hemosiderin deposition in hemophilic arthropathy (HA) of the knee.
**Methods or Background:** This prospective study included nine ex-vivo knee synovial tissues from HA patients and the same tissues from healthy controls. All tissues underwent standardized qMRI protocols using quantitative susceptibility mapping (QSM), based on ultrashort echo time MRI, which was optimized to detect and quantify hemosiderin deposits. Also, standard MRI sequences were employed. The HA tissues were processed histologically using Perl's Prussian Blue (PPB) staining to identify iron contents. Several regions of interest were drawn in each tissue. Using specialized algorithms, voxel-wise magnetic susceptibility was calculated to assess iron deposition within the knee tissues objectively.
**Results or Findings:** qMRI demonstrated high sensitivity in detecting and quantifying hemosiderin deposition, whereas conventional imaging showed no abnormalities. The estimated susceptibility values (ESVs) showed significant differences between HA and control samples. HA tissues presented a mean ESV of 0.48 ± 1.08 ppm and control tissues of 0.13 ± 0.12 ppm (p<0.05). A significant linear correlation was found between the iron level quantified by histology and the ESV estimated by QSM (R = 0.908, p < 0.01). There was a significant difference in the susceptibility in high load (HL) tissues compared to low load (LL) tissues (ESV = 5.57 ± 1.23 ppm for HL vs. 0.57 ± 0.85 ppm for LL, p<0.001).
**Conclusion:** This study establishes qMRI, particularly QSM, as a noninvasive and highly sensitive technique for quantifying hemosiderin in HA of the knee. By providing an objective measure of hemosiderin deposition, qMRI offers potential as a tool for early diagnosis and disease monitoring in patients with hemophilic arthropathy.
**Limitations:** Main limitation: ex-vivo study design.
**Funding for this study:** This study was funded by the National Institutes of Health (NIH R01AR078877) and the Deutsche Forschungsgemeinschaft (DFG SE 3272/1-1)
**Ethics committee - additional information:** The Institutional Review Board (IRB) of the University of California San Diego approved the study.

**Author Disclosures:**
Jinil Park: Nothing to disclose
Sam Sedaghat: Nothing to disclose
Hyungseok Jang: Nothing to disclose
Eddie Fu: Nothing to disclose
Patricia Leutz-Schmidt: Nothing to disclose

**Diabetes-related foot disease: the added value of zte**

M. Di Diego, D. Perla, A. Infante, A. M. Costantini, M. L. Angeli, C. Gullì, *G. Ferrara*; Rome/IT
*(giuseppe.ferrara07@icatt.it)*

**Purpose or Learning Objective:** The primary endpoint is to evaluate the additional diagnostic information obtained from ZTE sequences added to the standard MRI protocol (particularly compared to T1-weighted images) for improved assessment of bone structures in diabetes-related foot disease. Secondary endpoints include assessing accuracy compared to CT in the evaluation of bone alterations and investigating interobserver agreement between three musculoskeletal radiologists with different expertise.
**Methods or Background:** This retrospective single-center study analyzed 32 MRIs with ZTE sequences from 31 patients (22 males, 10 females; age range: 49-87 years) from March 2024 to September 2024. Inclusion criteria included patients >18 years old with a confirmed diagnosis of diabetes mellitus. In 11 cases, comparison between ZTE sequences and CT bone imaging was possible.
**Results or Findings:** ZTE sequences compared to standard MRI protocol (particularly to T1-weighted images) were superior in identifying soft tissue air (43% vs 31%), bone pneumatosis (19% vs 9%), bone erosions (77% vs 67%), bone exposure (20% vs 15%), bone sclerosis (49% vs 41%), periosteal reaction (19% vs 17%), and bone fragments (39% vs 25%). Compared to CT, ZTE demonstrated high sensitivity (75-100%) and specificity (92-100%) for all musculoskeletal alterations analyzed. Interobserver agreement between musculoskeletal radiologists was excellent (k-range 0.82).
**Conclusion:** ZTE sequences provided additional musculoskeletal information compared to T1-weighted MRI sequences, particularly for the morphological evaluation of bones affected by diabetes-related foot disease. This is crucial when hypointensity on T1-weighted images reduces the ability to visualize bone structures and their alterations. Moreover, the high sensitivity and specificity values compared to CT suggest that ZTE is a valid alternative. The interobserver agreement for the qualitative evaluation of ZTE sequences was excellent, indicating the ease of interpretation.
**Limitations:** Small sample size.
**Funding for this study:** None
**Ethics committee - additional information:** None
**Author Disclosures:**
Amato Infante: Nothing to disclose
Consolato Gullì: Nothing to disclose
Maria Luigia Angeli: Nothing to disclose
Daniele Perla: Nothing to disclose
Giuseppe Ferrara: Nothing to disclose
Mario Di Diego: Nothing to disclose
Alessandro Maria Costantini: Nothing to disclose

**The assessment of rheumatoid arthritis and other arthropathies through power Doppler and superb microvascular imaging: is there any difference?**

*P. Del Nido Recio*, J. D. Aquerreta, A. Paternain Nuin, M. R. López De La Torre Carretero, M. Jiménez Vázquez, C. Mbongo, C. Urtasun Iriarte, D. A. Zambrano, M. B. Barrio Piqueras; Pamplona/ES
*(pdelnidor@unav.es)*

**Purpose or Learning Objective:** This study aims to analyze whether the upgrade Doppler activity, when comparing SMI and PD, is significantly different in Rheumatoid Arthritis (RA), compared to other arthropathies.
**Methods or Background:** Between May 2023 and April 2024, we prospectively analyzed a cohort of 57 joints of 21 different patients. Most of them were previously diagnosed with RA and other arthropathies, such as Osteoarthritis, Psoriatic Arthritis, Gout, etc. PD and SMI imaging were obtained in all joints and the individual grades for Doppler Activity were registered for each joint with active synovitis, raging from 0 to 3, according to the EULAR - OMERACT US Score. Mann-Whitney U test was applied to calculate means in independent samples. Two-tailed p-values of <0.05 were considered statistically significant.
**Results or Findings:** 21 joints of 8 patients with RA and 36 joints of 13 patients with other arthropathies were studied. Metacarpophalangeal joints were the most frequently analyzed in the RA group (11), and interphalangeal joints were the most frequently analyzed in the other group (18). When comparing Doppler activity, the mean PD was significantly higher in the RA group compared to the other group (1.14 vs. 0.75, p = 0.043). When upgrading the Doppler activity with SMI, we did not find statistically significant differences (2.52 vs 2.69, p = 1.04). Nevertheless, when comparing the mean of the difference between SMI and PD, it was significantly lower in the RA group (1.38 vs. 1.94, p = 0.016).
**Conclusion:** Our results indicate that a higher upgrade between PD and SMI exams is more likely seen in arthropathies such as oligoarthritis, Psoriatic Arthritis or Gout, rather than in RA.
**Limitations:** The number of patients. Interobserver variability. The degree of arthritis and type of treatment.
**Funding for this study:** No funding was received for this study.
**Ethics committee - additional information:** The study received institutional review board approval and written informed consent was obtained from all participants
**Author Disclosures:**
Pablo Del Nido Recio: Nothing to disclose
Carmen Mbongo: Nothing to disclose
Cesar Urtasun Iriarte: Nothing to disclose
Jesús Dámaso Aquerreta: Nothing to disclose
Miguel Barrio Barrio Piqueras: Nothing to disclose
Manuel Rafael López De La Torre Carretero: Nothing to disclose
Daniel Alfonso Zambrano: Nothing to disclose
Alberto Paternain Nuin: Nothing to disclose
Marcos Jiménez Vázquez: Nothing to disclose

**Prevalence of diffuse idiopathic skeletal hyperostosis (DISH) according to recently established imaging criteria and coexistent pelvic enthesophytes**

*V. Yaman*, A. E. Yıldız, B. Fırlatan, H. Avcı, O. Karadağ, U. Kalyoncu, Ü. Aydingöz; Ankara/TR
*(drvedatyaman@gmail.com)*

**Purpose or Learning Objective:** Recent (2019) criteria that supplement classic DISH criteria were developed to diagnose this condition at an earlier stage. The aims of the present study were to investigate the prevalence of the early-stage (ES-DISH) as well as classic (late-stage) (LS-DISH) disease and to determine pelvic enthesophyte status in patients with ES-DISH and LS-DISH.
**Methods or Background:** 636 consecutive patients aged ≥18 years who underwent thorax CT during October 2023 in a tertiary medical center were retrospectively evaluated. CTs were scored according to 2019 DISH criteria by two independent observers. Pelvic (including parasacroiliac) enthesophytes in patients who also had a simultaneous abdomen CT were examined by a musculoskeletal radiologist blinded to patients' DISH status as well as clinical information, yielding a personal "pelvic enthesophyte load score" (PELS). Malignancy and metabolic syndrome data from patients were also analyzed.
**Results or Findings:** Prevalences of ES-DISH and LS-DISH were 16.7% and 15.4%, respectively. Intra- and interobserver agreement were "almost perfect" (ICC = 0.88; 95% CI, 0.77–0.99) and "substantial" (ICC = 0.73; 95% CI, 0.68–0.78), respectively. Mean ages (and age range) of "no DISH", ES-DISH, and LS-DISH subgroups were 55.1 (18–86), 65.5 (46–90), and 69.2 (48–96), respectively. DISH prevalence was similar in patients without and with cancer. When age was factored in, frequency of metabolic syndrome in DISH patients did not differ significantly compared to those without DISH. Mean±SD PELS were 6.2±3.5, 8.9±3.4 and 10.1±3.2 in "no DISH", ES-DISH and LS-DISH groups. Logistic regression analysis yielded an overall accuracy of 68% for PELS in predicting DISH status.
**Conclusion:** This study demonstrated that prevalence of DISH doubled when ES-DISH criteria were used. Pelvic enthesophytes are more profusely seen in ES- or LS-DISH than in patients without DISH.
**Limitations:** Retrospective study
**Funding for this study:** No funding was received for this study.
**Ethics committee - additional information:** This study was approved by Hacettepe University Clinical Studies Ethics Committee (SBA 24/166).
**Author Disclosures:**
Umut Kalyoncu: Nothing to disclose
Hanife Avcı: Nothing to disclose
Büşra Fırlatan: Nothing to disclose
Üstün Aydingöz: Nothing to disclose
Omer Karadağ: Nothing to disclose
Vedat Yaman: Nothing to disclose
Adalet Elçin Yıldız: Nothing to disclose

**10:00-11:00** **Research Stage 3**

### Research Presentation Session: Imaging Informatics and Artificial Intelligence

### RPS 805

### Artificial intelligence in neuroimaging

**Moderator**
M. M. Serra; Avignon/FR
*(mechyserra@gmail.com)*
**Author Disclosures:**
Maria Mercedes Serra: Employee: BC Platforms; Research Grant/Support: Sanofi; Share Holder: BC Platforms

### Impact of data quality variations caused by dose and image reconstruction on AI assessment of intracranial aneurysms

L. Gölz, A. Laudani, U. Genske, M. Scheel, G. Bohner, H-C. Bauknecht, S. Mutze, *P. Jahnke*; Berlin/DE
*(paul.jahnke@charite.de)*

**Purpose or Learning Objective:** To assess the performance of a commercial AI algorithm in detecting intracranial aneurysms when scan data quality variations occur due to changes in dose and image reconstruction.
**Methods or Background:** Consistency testing of AI performance was performed using a realistic head CT phantom designed for AI evaluation. The phantom simulated a patient with three intracranial aneurysms located in the anterior communicating artery (ACoA), middle cerebral artery (MCA), and basilar artery (BA). The phantom was repeatedly examined at 21 dose levels (0.47 to 20.09 mGy) using iterative reconstruction and filtered back projection. Aneurysm labeling by an FDA-approved and CE-marked AI solution was analyzed. In addition, five neuroradiologists rated aneurysm visiblity in all examinations.
**Results or Findings:** AI detection rates varied by aneurysm type, with detection rates of 74.6% for the ACoA, 92.9% for the MCA, and 2.4% for the BA aneurysm across all examinations. The AI response was inconsistent at doses below 8 mGy with iterative reconstruction and at doses below 7 mGy and above 14 mGy with filtered back projection. In contrast, readers consistently reported 100% visibility for all aneurysms at doses above 2 mGy regardless of image reconstruction.
**Conclusion:** AI approved for managing intracranial aneurysms shows performance issues due to variations in data quality and requires different data quality standards than neuroradiologists.
**Limitations:** This prospective study was limited to a single AI application, a single scanner system, and three intracranial aneurysms.
**Funding for this study:** This work has not received any funding.
**Ethics committee - additional information:** Ethics committee of the Charité
**Author Disclosures:**
Hans-Christian Bauknecht: Nothing to disclose
Paul Jahnke: Shareholder: PhantomX GmbH Employee: PhantomX GmbH
Ulrich Genske: Nothing to disclose
Georg Bohner: Nothing to disclose
Leonie Gölz: Nothing to disclose
Angelo Laudani: Nothing to disclose
Michael Scheel: Shareholder: PhantomX GmbH
Sven Mutze: Nothing to disclose

### Improving diagnostic precision: a deep learning system for differentiating multiple sclerosis from small vessel disease using standard non-enhanced brain MRI scans

K. Firouznia, *M. Arab Ahmadi*, H. Hashemi, M. Boroomand-Saboor, R. Ghavami Modegh, M. Akhlaghpasand, H. Dashti, M. Gity, M. Mohammadzadeh; Tehran/IR
*(mehran_arabahmadi@yahoo.com)*

**Purpose or Learning Objective:** The diagnosis of Multiple Sclerosis (MS) primarily depends on clinical evaluation, bolstered by magnetic resonance imaging (MRI) interpreted by skilled radiologists.However, the typical imaging characteristics of MS can resemble those of other central nervous system disorders.One such condition is Cerebral SVD, which can complicate the radiologist's ability to make a diagnosis.This differential diagnosis can be particularly challenging in the early stages of the disease.The objective of this study is to create and assess a Computer-Aided Diagnosis (CAD) system utilizing brain MRI images to differentiate between MS and SVD.
**Methods or Background:** Brain MRI scans were obtained from a 3 Tesla scanner for patients diagnosed with MS during acute attacks and silent phases, alongside individuals diagnosed with SVD based on cardiovascular risk factors. MRI sequences included FLAIR,T1, and T2. An expert neuroradiologist identified white matter lesions, which were segmented using artificial intelligence software. The dataset was divided into 80% for training, 10% for validation, and 10% for testing. A neuroradiologist then evaluated the AI results against established clinical and imaging criteria.
**Results or Findings:** The study included 80 MS patients with 265 lesions compared to 67 SVD patients with 218 lesions. The AI tool achieved a sensitivity of 78.57% and specificity of 93.33% (P-value $< 0.05$). It also demonstrated a positive predictive value (PPV) of 91.67%, a negative predictive value (NPV) of 82.35%, balanced accuracy of 85.95%, and an area under the curve (AUC) of 78.71.
**Conclusion:** The findings suggest that artificial intelligence can effectively differentiate MRI images of MS from those of SVD using routine sequences.Implementing AI in distinguishing between MS and SVD lesions could enhance diagnostic accuracy and improve patient management in clinical practice.
**Limitations:** Sample volume and one center study listed as some of the limitations.
**Funding for this study:** None
**Ethics committee - additional information:** This study was approved by an institutional ethics committee.
**Author Disclosures:**
Masoumeh Gity: Nothing to disclose
Kavous Firouznia: Nothing to disclose
Mohammadhosein Akhlaghpasand: Nothing to disclose
Hassan Hashemi: Nothing to disclose
Melika Boroomand-Saboor: Nothing to disclose
Maryam Mohammadzadeh: Nothing to disclose
Rassa Ghavami Modegh: Nothing to disclose
Hamed Dashti: Nothing to disclose
Mehran Arab Ahmadi: Nothing to disclose

### Previously proposed radiomics features for ruptured intracranial aneurysm classification: Overview, auto-segmentation, and external validation

*D. Zhu*, Y. Yang; Wenzhou/CN
*(zhudongqin@wmu.edu.cn)*

**Purpose or Learning Objective:** To automatically segment and extract radiomics features of intracranial aneurysms (IAs), validate existing radiomics predictors for ruptured IAs, and construct machine learning (ML) and deep learning (DL) models for classifying ruptured IAs.
**Methods or Background:** In this retrospective study, we used data from the MIRACLE Cohort, registered with the Chinese Clinical Trial Registry (ChiCTR2400084601). IAs were segmented automatically using the DGIS method. We systematically reviewed studies reporting radiomics predictors for ruptured IAs and externally validated those predictors. We developed five ML and DL models for classifying ruptured IAs, employing the SHapley Additive exPlanations (SHAP) method to enhance model interpretability.
**Results or Findings:** The study included 632 patients with 668 aneurysms, divided into training (n=593) and external testing (n=75) datasets. The DGIS method achieved great segmentation accuracy with Dice coefficients of 0.98 and 0.75 in the source and target domains, respectively. When comparing radiomics features derived from manual and automatic segmentations, the original_shape_VoxelVolume, MeshVolume, and SurfaceArea showed the highest stability (with all ICC of >0.9). Upon external validation of radiomics predictors from 12 studies, the AUCs ranged from 0.59 to 0.71 in the training dataset and 0.48 to 0.65 in the external testing dataset. The original_shape_ Elongation feature emerged as the most frequently utilized predictor. The Gradient Boosting and DRE models performed well in classifying ruptured IAs, with AUCs reaching 0.995 and 0.95 in the training dataset, and 0.85 and 0.80 in the external testing dataset, respectively.
**Conclusion:** This study presents a comprehensive workflow for automatic IAs rupture risk analysis and an overview of existing radiomics predictors. After external validation, certain original shape features demonstrated significant stability, utility, and predictive power. The ML and DL models offer a promising tool for risk stratification of IAs.
**Limitations:** Not applicable.
**Funding for this study:** This study was supported by the Wenzhou Major Program of Science and Technology Innovation (Grant No. ZY2020012) and Key Laboratory of Novel Nuclide Technologies on Precision Diagnosis and Treatment & clinical Transformation of Wenzhou City (Grant No. 2023HZSY0012).
**Ethics committee - additional information:** Ethics Committee of the First Affiliated Hospital of Wenzhou Medical University
**Author Disclosures:**
Yunjun Yang: Nothing to disclose
Dongqin Zhu: Nothing to disclose

**Diagnostic Performance of Neural Network Algorithms in Skull Fractures Detection in CT Scans: A Systematic Review and Meta-Analysis**

*R. Hajibeygi*[1], G. Sharifi[1], M. Fathi[1], A. Bahrami[2], R. Eshraghi[2], I. Dixe De Oliveira Santo[3], A. Mirjafari[4], J. Chan[4], L. Tu[3]; [1]Tehran/IR, [2]Kashan/IR, [3]New Haven, CT/US, [4]Los Angeles, CA/US
*(Ramtin.beygi75@gmail.com)*

**Purpose or Learning Objective:** The potential intricacy of skull fractures as well as the complexity of underlying anatomy poses diagnostic hurdles for radiologists evaluating CT scans. The necessity for automated diagnostic tools has been brought to light by the shortage of radiologists and the growing demand for rapid and accurate fracture diagnosis. Convolutional Neural Networks (CNNs) are a potential new class of medical imaging technologies that use deep learning (DL) to improve diagnosis accuracy. The objective of this systematic review and meta-analysis is to assess how well CNN models diagnose skull fractures on CT images.
**Methods or Background:** PubMed, Scopus, and Web of Science were searched for studies before February 2024 that used CNN models to detect skull fractures on CT scans. Meta-analyses were conducted for area under the receiver operating characteristic curve (AUC), sensitivity, specificity, and accuracy. Egger's and Begg's tests were used to assess publication bias.
**Results or Findings:** Meta-analysis was performed for 11 studies with 20798 patients. Pooled average AUC for implementing pre-training for transfer learning in CNN models within their training model's architecture was 0.96 ± 0.02. The pooled averages of the studies' sensitivity and specificity were 1.0 and 0.93, respectively. The accuracy was obtained 0.92 ± 0.04. Studies showed heterogeneity, which was explained by differences in model topologies, training models, and validation techniques. There was no significant publication bias detected.
**Conclusion:** CNN models perform well in identifying skull fractures on CT scans. The results suggest that CNNs have the potential to improve diagnostic accuracy in the imaging of acute skull trauma. To further enhance these models' practical applicability, future studies could concentrate on the utility of DL models in prospective clinical trials.
**Limitations:** One of the limitations is lack of homogeneity in CT image quality across studies.
**Funding for this study:** None
**Ethics committee - additional information:** None
**Author Disclosures:**
Reza Eshraghi: Nothing to disclose
Mobina Fathi: Nothing to disclose
Janine Chan: Nothing to disclose
Guive Sharifi: Nothing to disclose
Ashkan Bahrami: Nothing to disclose
Irene Dixe De Oliveira Santo: Nothing to disclose
Arshia Mirjafari: Nothing to disclose
Ramtin Hajibeygi: Nothing to disclose
Long Tu: Nothing to disclose

**AI as a second reader in post-traumatic head CT at Oktoberfest 2024: A prospective performance monitoring study**

*M. B. Steinberger*, M. Bock, A. S. Duque, B. F. Hoppe, J. P. Rudolph, Y. Dikhtyar, P. Reidler, W. Flatz, D. Hinzmann, V. Bogner-Flatz, J. Ricke, C. C. Cyran; Munich/DE
*(maria.steinberger@med.uni-muenchen.de)*

**Purpose or Learning Objective:** To prospectively assess the impact of an AI algorithm on radiologists' diagnostic confidence in detecting intracranial haemorrhage (ICH) in post-traumatic head CT at Oktoberfest 2024.
**Methods or Background:** A mobile CT scanner (Somatom go.Top, Siemens Healthineers) was operated on-site for triaging patients with mild to moderate traumatic head injuries. This prospective study included n=219 patients who underwent head CT. Instant AI analysis was provided via auto-routing to a fully PACS-integrated, GDPR-compliant clinical AI platform. Initially, one of 15 board-certified radiologists, alternating in shifts, read the head CT unassisted, rating ICH likelihood on a 5-point Likert scale (-2, "very low"; 2, "very high"). After submitting this evaluation, algorithm results were made available for reassessment of ICH likelihood. Performance monitoring of the AI tool was implemented in PACS (Visage Imaging) via Fast Healthcare Interoperability Resources (FHIR) pop-up forms.
**Results or Findings:** AI support was utilised in 66% (146/222 scans) of the readings, varying significantly between readers (43%-100%). At a probability threshold of 0.1, the AI tool correctly identified 6 out of 7 ICH, 139 true negatives, no false positives (sens=0.857, spec=1.000, acc=0.993, ppv=1.000, npv=0.993). AI assistance increased radiologists' confidence in ruling out ICH in 19 cases (-1 to -2) and confirming ICH in two cases (1 to 2). In two borderline cases, AI aided in excluding ICH (0 to -1). Overall, diagnostic confidence was significantly higher with AI support ($p<0.001$).
**Conclusion:** AI assistance significantly improved diagnostic confidence of radiologists reading trauma head CT at Oktoberfest 2024, serving as a virtual second reader in this emergency setting. PACS-integrated FHIR forms set a framework for seamless monitoring of AI performance and its impact on diagnostic workflow.
**Limitations:** In 40 cases, no AI analysis was performed due to incorrect specifications or failed auto-routing.
**Funding for this study:** No funding was provided for this study.
**Ethics committee - additional information:** The ethics committee notification can be found under the number UID 24-0813. Written informed consent was obtained from all participants and the study was registered in the German Clinical Trial Register (DRKS00034969).
**Author Disclosures:**
Jan Philipp Rudolph: Nothing to disclose
Boj Friedrich Hoppe: Nothing to disclose
Yevgeniy Dikhtyar: Nothing to disclose
Clemens C. Cyran: Nothing to disclose
Wilhelm Flatz: Nothing to disclose
Dominik Hinzmann: Nothing to disclose
Matthias Bock: Nothing to disclose
Paul Reidler: Nothing to disclose
Maria Barbara Steinberger: Nothing to disclose
Veronika Bogner-Flatz: Nothing to disclose
Anna Sophie Duque: Nothing to disclose
Jens Ricke: Nothing to disclose

**Impact of Defacing Procedures on Brain Age Gap Estimation**

*V. L. Ivan*[1], J. Caspers[1], M. Vach[1], D. M. Hedderich[2], D. Weiß[1], C. Rubbert[1]; [1]Düsseldorf/DE, [2]Munich/DE

**Purpose or Learning Objective:** Removal of facial features from MRI brain scans ("Defacing") is mandatory from data privacy perspective. We investigated the impact of defacing on Brain Age Gap Estimation (BrainAGE), an imaging biomarker used in various research areas such as atypical aging.
**Methods or Background:** A total of 364 Alzheimer's disease (AD) patients and 717 cognitively normal (CN) participants were analyzed including unaccelerated (AD:n=290; CN:n=386) and accelerated 3DT1 imaging (AD:n=203;; CN:n=500). BrainAGE was computed after defacing using either afni_refacer, fsl_deface, mri_deface, mri_reface, PyDeface, or spm_deface and without defacing. For BrainAGE, gray matter features were extracted using CAT12 for SPM12. BrainAGE was calculated as predicted age minus chronological age. A subset of participants (AD:n=74, CN:n=84) had within-session repeat imaging available and were processed without defacing, serving as a benchmark for BrainAGE differences. Mean absolute error (MAE), and mean squared error (MSE) were calculated. Outliers due to defacing were identified using Grubbs's tests.
**Results or Findings:** Benchmark analysis found MAE of 1.15 and MSE of 2.25 for BrainAGE differences between initial and repeat scans without defacing in CN, and an MAE of 1.43 and MSE of 3.29 for AD. Among defacing methods, PyDeface exhibited the best performance with an overall MAE of 0.33 and MSE of 0.27, showing a mean BrainAGE difference of 0.08±0.52. PyDeface also had the fewest outliers (n=99) based on the benchmark criteria. Grubbs's test identified 23 outliers after PyDeface, with 11 found after mri_reface and 20 after spm_deface.
**Conclusion:** Defacing can be employed for data privacy without significantly affecting the reliability of BrainAGE as an imaging biomarker. PyDeface is recommended.
**Limitations:** BrainAGE may be affected by defacing, however, in most approaches this influence is lesser than the variability observed in BrainAGE in repeat non-defaced imaging.
**Funding for this study:** No
**Ethics committee - additional information:** No
**Author Disclosures:**
Marius Vach: Nothing to disclose
Julian Caspers: Nothing to disclose
Christian Rubbert: Nothing to disclose
Daniel Weiß: Nothing to disclose
Vivien Lorena Ivan: Nothing to disclose
Dennis M Hedderich: Nothing to disclose

**Identification of depression subtypes in Parkinson's disease patients via structural MRI whole-brain radiomics: an unsupervised machine learning study**

*Z. Shu*; Zhejiang, Hangzhou/CN
*(cooljuty@hotmail.com)*

**Purpose or Learning Objective:** Unsupervised machine learning methods based on whole-brain radiomic analysis were used to identify subtypes of depression that occur during the progression of Parkinson's disease (PD).
**Methods or Background:** Data from 272 PD patients in the PPMI database were used, among which 81 experienced depression in Parkinson's Disease (DPD) during a 5-year follow-up period. Quantitative radiomic features were extracted from the whole-brain magnetic resonance structural images of each

Thursday patient, and principal component analysis (PCA) was used for feature dimensionality reduction. All of the cases were classified into different subtypes by unsupervised cluster analysis (UCA). The high-risk subtypes were selected through comparative analysis. The high-risk subtype data were divided into training subgroups and testing subgroups at a 7:3 ratio. On the basis of the clinical characteristics of the training subgroups, multiple logistic regression analysis was performed to confirm the risk factors for DPD subtypes. The DPD subtypes were subsequently identified on the basis of the risk factors. A prediction model was constructed via decision trees, and the diagnostic accuracy of the model was evaluated via receiver operating characteristic (ROC) curves.
**Results or Findings:** Logistic regression analysis based on high-risk subtype groups revealed that rem, updrs1_score, updrs2_score, and ptau were independent predictors of DPD. The prediction model based on high-risk subgroups had AUC values of 0.853 and 0.81 in the training and testing subgroups, sensitivities of 0.765 and 0.786, and specificities of 0.771 and 0.815, respectively. The AUC, sensitivity, and specificity in the non-high-risk subgroup were 0.859, 0.654, and 0.852, respectively.
**Conclusion:** An UCA based on MRI structural imaging features can identify high-risk subtypes of DPD, and the constructed model can also predict the progression of DPD well.
**Limitations:** This study was designed as a retrospective analysis.
**Funding for this study:** The work was supported by the Natural Science Foundation of Zhejiang Province of China (LGF22H090021)
**Ethics committee - additional information:** The case data used in this study came from the Parkinson's Progression Markers Initiative (PPMI) (http://www.PPMI-info.org) database, and data collection was approved by institutional review board; For ethical review information on the data, please refer to the website.
**Author Disclosures:**
Zhenyu Shu: Nothing to disclose

**A Machine-Learning Model Based on US Radiomics to Classify Benign and Malignant Thyroid Nodules**
A. Guerrisi[1], *V. Dolcetti*[1], L. Miseo[1], A. Valenti[1], F. Elia[1], G. Del Gaudio[1], F. Raponi[2], E. David[2], V. Cantisani[1]; [1]Rome/IT, [2]Catania/IT
*(vincenzodolcetti@gmail.com)*

**Purpose or Learning Objective:** The aim of this work was to develop a machine learning model based on thyroid ultrasound images in order to classify nodules into benign and malignant classes. Ultrasound and fine needle biopsy are the most reliable diagnostic methods to date, but they have some limitations. Radiomics and machine learning could be useful to improve diagnosis while reducing invasive procedures.
**Methods or Background:** Ultrasound images from 142 subjects were collected: 40 patients belonged to "malignant" and 102 to "benign" class, according to histological diagnosis (fine-needle aspiration). Those images were used to train, cross-validate and internal test three different machine learning models, using the “Trace for Research” software. A robust radiomic approach was applied, and the models (random forests, SVM and k-NN classifiers) were evaluated. Finally, the best model was externally tested on an additional cohort of 21 patients.
**Results or Findings:** The best model (ensemble of random forest) showed ROC-AUC (%) of 85 (majority vote), 83.7** (mean) [80.2-87.2], accuracy (%) of 83, 81.2** [77.1-85.2], sensitivity (%) of 70, 67.5** [64.3-70.7], specificity (%) of 88, 86.5** [82-91], PPV (%) of 70, 66.5** [57.9-75.1], and NPV (%) of 88, 87.1** [85.5-88.8] (*p<0.05, **p<0.005) in the internal test cohort. This model was then externally tested, achieving an Accuracy of 90.5%, a sensitivity of 100%, a specificity of 86.7%, a PPV of 75% and an NPV of 100%.
**Conclusion:** The best model could successfully identify all the malignant nodes and the consistent majority of benign in external testing cohort. Further investigations could be conducted by testing the model with images of nodules from different centers.
**Limitations:** Additional external tests should be performed, with images from different ultrasound machines and different healthcare centers to increase variability of target population.
**Funding for this study:** This research was supported by Italian Ministry of Health
**Ethics committee - additional information:** This study was performed in line with the principles 417 of the Declaration of Helsinki. Approval was granted by the Ethics Committee of IRCCS 418 IFO-Fondazione GB Bietti (Date: 23/01/2023, N: 1820/23)

**Author Disclosures:**
Antonino Guerrisi: Nothing to disclose
Giovanni Del Gaudio: Nothing to disclose
Alessandro Valenti: Nothing to disclose
Fulvia Elia: Nothing to disclose
Vincenzo Dolcetti: Nothing to disclose
Flavia Raponi: Nothing to disclose
Ludovica Miseo: Nothing to disclose
Vito Cantisani: Nothing to disclose
Emanuele David: Nothing to disclose

**10:00-11:00** **Research Stage 4**

### Research Presentation Session: Abdominal and Gastrointestinal

### RPS 801

### Advances in liver imaging

**Moderator**
J.-I. Choi; Seoul/KR
*(dumkycji@gmail.com)*
**Author Disclosures:**
Joon-Il Choi: Grant Recipient: Guerbet Korea, Siemens Healthineers, Samsong Medison, Bracco Korea; Speaker: Bayer Heathcare

**Multiparametric spectral imaging for characterization of small hypoattenuating liver lesions**
*N. Abou Zeid*, C. Nelles, Z. Gurbanova, N. Große Hokamp, T. Persigehl, S. Lennartz; Cologne/DE

**Purpose or Learning Objective:** To investigate the diagnostic utility of spectral reconstructions for determining cystic nature of small, hypoattenuating liver lesions.
**Methods or Background:** Patients with portal venous phase dual-layer dual-energy CT (dlDECT) who were diagnosed with hypoattenuating liver lesions smaller than one centimeter that were verified as cysts in corresponding MRI examinations were retrospectively included. ROI-based measurements were conducted by two raters in conventional images (CI), virtual unenhanced images (VUE) and iodine images. CT-based determination of cystic nature of the lesions was conducted using a HU attenuation threshold of less than 20 HU or an iodine concentration threshold of less than 0.5 mg/dl, the latter of which has been reported as the scanner-specific lower limit of iodine detection. Accuracy for determining cystic nature was compared between CI, VUE and iodine images.
**Results or Findings:** 77 patients with 287 small liver cysts were included. Mean attenuation in CI for small liver cysts was 20.8 ± 24.4 HU, and 11.1 ± 16.8 HU in VUE images. Mean iodine concentration was 0.46 ± 0.57 mg/dl. Using the 20 HU threshold in CI resulted in an accuracy of 60.3 % (173/287), whereas the corresponding accuracy using the same threshold in VUE images was 76.7 % (220/287). Accuracy solely based on the iodine threshold was 57.5 % (165/287). Combining the VUE and iodine threshold resulted in an accuracy of 92.7 % (266/287) for determining cystic nature of the lesions in dlDECT.
**Conclusion:** Combining quantitative VUE and iodine measurements using established thresholds facilitated correctly diagnosing 92.7% of small hypoattenuating liver lesions as cysts, compared to an accuracy of 60.3 % when using HU measurements in conventional images. This approach may help reducing correlative imaging and thereby accelerating staging of cancer patients.
**Limitations:** Retrospective, mono-centric study
**Funding for this study:** None to report
**Ethics committee - additional information:** IRB waiver due to retrospective nature of the study.
**Author Disclosures:**
Simon Lennartz: Speaker: Amboss GmbH Author: Amboss GmbH
Nils Große Hokamp: Research/Grant Support: Philips Speaker: Philips Speaker: Amboss GmbH
Christian Nelles: Nothing to disclose
Nour Abou Zeid: Nothing to disclose
Zuleykha Gurbanova: Nothing to disclose
Thorsten Persigehl: Nothing to disclose

**Could MRI-radiomics predict Liver Metastasis Recurrence and Overall Survival after surgery or ablation treatment?**

*T. Russo*, A. Belardo, A. Della Corte, D. Santangelo, F. Calabrese, M. M. Vincenzi, M. Mori, C. Fiorino, F. De Cobelli; Milan/IT
*(t.russo.1994@gmail.com)*

**Purpose or Learning Objective:** To investigate the potentials of MRI radiomics to predict recurrence (R), hepatic recurrence (IR), and overall survival (OS) in a cohort of patients with colorectal liver metastases (CLM) who underwent microwave ablation (MWA) performed alone or in combination with surgical resection.
**Methods or Background:** 121 CLM patients with pre-operative Gadoxetic acid-MRI treated at our Institute between October 2015 and December 2022 were analyzed. One observer manually segmented the largest CLM on T2 scans. The abdominal aorta at the level of second lumbar vertebrae was used for the z-score normalization of the lesion. Cox multivariate analysis was run to establish a few-features radiomic model (RAD-T2), to predict recurrences and death. A bootstrap-based methodology for robust feature selection, including redundancy filtering, was optimized to select the best combination of two, three, four features. Correction of the models for optimism was then performed by internal bootstrap-based validation.
**Results or Findings:** For R, IR and OS the median follow-ups were respectively 12, 13 and 23 months; the number of events were 80, 68 and 34. After corrections for optimism, the resulting best RAD-T2 models were based on the combination of 2-3 features; they were able to predict R with C-index=0.65 (p=0.0002), IR (C-index=0.64, p=0.0029) and OS (C-index=0.71, p=0.0046). As an example, based on the best cut-off value of the RAD-T2 index, OS at 2 year was 58% and 88% when the cohort was stratified accordingly.
**Conclusion:** T2-MRI-based radiomic evaluation of CLMs is feasible and potentially useful for outcome prediction.
**Limitations:** The limited number of patients and the retrospective nature of the study.
**Funding for this study:** The limited number of patients and the retrospective nature of the study.
**Ethics committee - additional information:** All procedures were carried out in accordance with the Declaration of Helsinki (1964) and its later amendments.
**Author Disclosures:**
Monica Maria Vincenzi: Nothing to disclose
Angelo Della Corte: Nothing to disclose
Claudio Fiorino: Nothing to disclose
Domenico Santangelo: Nothing to disclose
Alfonso Belardo: Nothing to disclose
Martina Mori: Nothing to disclose
Tommaso Russo: Nothing to disclose
Francesca Calabrese: Nothing to disclose
Francesco De Cobelli: Nothing to disclose

**Intraindividual Comparison of Half-dose Gadopiclenol and Standard Dose of Gadobenate Dimeglumine for Abdominal MRI**

*A. Del Gaudio*[1], K. Kalisz[1], D. Kruse[1], F. Ria[1], D. De Santis[2], L. Lofino[1], D. Marin[1]; [1]Durham, NC/US, [2]Rome/IT
*(antonella.delgaudio@uniroma1.it)*

**Purpose or Learning Objective:** To intraindividually compare image quality and lesion conspicuity of abdominal MRI using gadopiclenol at 0.05 mmol/kg and gadobenate dimeglumine (Gd-BOPTA) at 0.1 mmol/kg.
**Methods or Background:** From September 2023 to March 2024, consecutive patients who had undergone two clinically indicated abdominal MRIs within 12 months using gadopiclenol and Gd-BOPTA on the same scanner were retrospectively enrolled. One independent radiologist manually measured the signal intensity of abdominal organs, arterial and venous vessels, and abdominal lesions (liver, pancreas, and kidneys) on unenhanced, late arterial, venous, and equilibrium phases. SNR, CNR, and magnitude of contrast enhancement (ΔE) were calculated for all organs and vessels on each contrast-enhanced phase. Percentage enhancement (%E) was calculated for all lesions on contrast-enhanced phases. Subjective image quality was assessed using a 5-point Likert scale, including: organs' and vessels' enhancement, liver-to-vessels contrast, and overall image quality. Lesion characteristics were also evaluated. Statistical analysis employed paired t- and Wilcoxon tests.
**Results or Findings:** One hundred subjects (64 years ± 14; 55 men) and 21 abdominal lesions were included. Compared to Gd-BOPTA, gadopiclenol yielded significantly higher CNR and SNR for pancreas, porta, and kidney in the late arterial phase ($p \leq .040$). No significant differences in CNR and SNR were observed between gadopiclenol and Gd-BOPTA across all organs in the portal venous and equilibrium phases. Gadopiclenol showed significantly higher pancreatic ΔE in all contras-enhanced phases ($p \leq .049$) compared to Gd-BOPTA. The %E of abdominal lesions was comparable between gadopiclenol and Gd-BOPTA for all contrast-enhanced phases ($p \geq .100$). No significant differences were observed in readers' perception of image quality and lesions' characteristics.
**Conclusion:** Gadopiclenol at 0.05 mmol/kg yields similar image quality and improved pancreatic enhancement compared to Gd-BOPTA at 0.1 mmol/kg.
**Limitations:** Retrospective study design
**Funding for this study:** None
**Ethics committee - additional information:** Written informed consent was waived and Institutional Review Board approval was obtained
**Author Disclosures:**
Danielle Kruse: Nothing to disclose
Ludovica Lofino: Nothing to disclose
Domenico De Santis: Nothing to disclose
Daniele Marin: Nothing to disclose
Antonella Del Gaudio: Other: Bracco research fellowship
Francesco Ria: Nothing to disclose
Kevin Kalisz: Nothing to disclose

**Preclinical profile of a new macrocyclic MRI liver agent**

*J. Lohrke*, T. Brumby, S. Herbert, T. Frenzel, G. Jost, M. Berger, H. Pietsch; Berlin/DE
*(jessica.lohrke@bayer.com)*

**Purpose or Learning Objective:** The established liver-specific gadolinium-based magnetic resonance imaging (MRI) contrast agents, gadoxetate disodium and gadobenate dimeglumine are based on linear, DTPA like ligands. In the present study a new early preclinical macrocyclic liver-specific candidate will be presented.
**Methods or Background:** The MRI efficiency (r1-relaxivity) of the candidate BAY 3393081 was determined at 37°C 1.41 T in human plasma. The kinetic inertness of the complex stability was investigated using an established zinc transmetallation assay. The in vitro liver cell uptake was assessed in rat hepatocytes and human transfected organic anion transporter protein 1B1 or 1B3 embryonic kidney cells. Pharmacokinetic parameters were evaluated in rodent (rats) and non-rodent (dogs) species by analyzing the gadolinium (Gd) concentrations in plasma over time. The in vivo liver elimination was examined in bile-duct-cannulated rats and the bile was analyzed using inductively coupled plasma mass spectroscopy. Contrast-enhanced liver MRI was performed in mice, pigs and a VX2 tumor model in rabbits.
**Results or Findings:** The relaxivity of BAY3393081 was determined with 8.7±0.3 L/(mmol·s) in human plasma at 1.41 T. The kinetic inertness of the complex stability was comparable to marketed macrocyclic GBCAs. The in vitro cell uptake results revealed that BAY3393081 is specifically taken up by rat and human OATPs. BAY 3393081 showed a high plasma clearance in rat and dog. In rats ~80% of the injected dose were eliminated in an unchanged form via the bile. Strong signal enhancement of liver parenchyma was demonstrated in rats, rabbits and pigs.
**Conclusion:** The preclinical candidate BAY3393081 showed the high kinetic inertness of macrocyclic GBCAs and a strong liver parenchyma enhancement in the MRI.
**Limitations:** Limited transferability of preclinical data to human liver elimination due to significant interspecies variability in hepatobiliary transporter expression.
**Funding for this study:** Funding for this preclinical study was provided by Bayer AG.
**Ethics committee - additional information:** All animal experiments were approved by LaGeSo.
**Author Disclosures:**
Simon Herbert: Employee: Bayer AG
Markus Berger: Employee: Bayer AG
Gregor Jost: Employee: Bayer AG
Thomas Frenzel: Employee: Bayer AG
Jessica Lohrke: Employee: Bayer AG
Thomas Brumby: Employee: Bayer AG
Hubertus Pietsch: Employee: Bayer AG

**Non-invasive diagnosis of chronic liver disease and portal hypertension using intravoxel incoherent motion imaging and magnetic resonance elastography**

*D. Catucci*, S. U. Von Däniken, V. Obmann, A. Berzigotti, L. Ebner, J. T. Heverhagen, A. Christe, P. Vermathen, A. T. Huber; Bern/CH

**Purpose or Learning Objective:** This study aimed to analyse the performance of intravoxel incoherent motion (IVIM) imaging parameters (tissue-diffusivity D, perfusion-fraction PF and pseudo-diffusion-coefficient D*) and liver stiffness (LS) measured by magnetic resonance elastography (MRE) to screen for chronic liver disease (CLD) and clinically significant portal hypertension (CSPH) on liver MRI examinations.
**Methods or Background:** This prospective study included 103 patients without CLD (noCLD-group, n=103) and 82 patients with biopsy-proven CLD who underwent liver MRI examinations including MRE and IVIM imaging

Thursday between 03/2016 and 11/2023. Patients with CLD were subdivided based on their liver fibrosis degree: early CLD (F0-F1; eCLD-group, n=21), intermediate CLD (F2; iCLD-group, n=19), advanced CLD (F3-F4; aCLD-group, n=20) and aCLD with CSPH according to the BAVENO VII consensus (aCLDPH-group; n=22). IVIM imaging parameters (D, PF and D*) of the liver as well as LS were measured in all patients. Statistical analysis included the Kruskal-Wallis test for group comparison and receiver operating characteristic (ROC) curve analysis with multiple logistic regression analysis for group differentiation.
**Results or Findings:** D, PF, D* and LS differed significantly between all groups (p<0.001). The overall best parameter for detecting CLD and CSPH was LS with a cut-off value of >3.2 kPa (sensitivity 67%/specificity 96%) respectively >3.8 kPa (sensitivity 95%/specificity 88%), both with p<0.001. For CLD-detection, a combination of D <99 x 10-5 mm2/sec and LS >3.2 kPa increased the AUC from 0.89 with LS to 0.95. For CSPH-detection, a combination of D* <434 x 10-5 mm2/sec and LS >3.8 kPa increased the specificity (90%) with a slightly lower sensitivity (91%, p<0.001).
**Conclusion:** IVIM imaging parameters (D, PF and D*) as well as LS measured by MRE allow non-invasive screening for CLD and CSPH.
**Limitations:** This was a single center study and should be externally validated.
**Funding for this study:** This study received funding by the Swiss National Science Foundation (SNF), grant number 188591.
**Ethics committee - additional information:** This study was approved by the cantonal ethics committee of Bern (Kantonale Ethikkommission Bern).
**Author Disclosures:**
Sandro Urs Von Däniken: Nothing to disclose
Johannes T. Heverhagen: Nothing to disclose
Damiano Catucci: Nothing to disclose
Verena Obmann: Nothing to disclose
Peter Vermathen: Nothing to disclose
Lukas Ebner: Nothing to disclose
Andreas Christe: Nothing to disclose
Adrian Thomas Huber: Nothing to disclose
Annalisa Berzigotti: Nothing to disclose

**Evaluation of Artificial Intelligence supported Third Harmonic B-mode in gallbladder ultrasound**
*P. Spiesecke*, T. Fischer; Berlin/DE

**Purpose or Learning Objective:** Superharmonic imaging is a useful B-mode ultrasound technology increasing spatial resolution. Currently, there is a novel technology available which produces B-mode images by a combination of different harmonics up to third harmonic using an Artificial Intelligence (AI) driven algorithm. The aim of the present study is the first evaluation of this technology.
**Methods or Background:** For this prospective study, overall 52 healthy test persons and patients were included. Standard and novel B-mode images of the gallbladder were captured in each subject – in each case by recording several combinations of additional parameters such as Dynamic Range and Speckle Reduction. For this purpose, a premium ultrasound system was used (Canon Aplio i800 including third-harmonic imaging). The images were manually segmented manually and were subjected to computer-aided analysis to analyze artifacts and edge sharpness. Additionally, Radiologists in different stages of training and subspecialisation rated the images by means of different parameters on a Likert scale.
**Results or Findings:** N = 26 data sets each with and without gallbladder pathology were included. In gallbladder B-mode ultrasound, ratings of the Third Harmonic Imaging-derived images showed a significant reduction of artifacts in the gallbladder lumen as well as a higher sharpness of interfaces. Subjective analysis revealed higher image quality in Third Harmonic Imaging compared to standard B-mode.
**Conclusion:** Our results suggest, that the AI-driven Third Harmonic Imaging can be a useful tool to increase the sharpness of interfaces and reduce the artifacts in gallbladder B-mode ultrasound.
**Limitations:** The present study is a single center study which first evaluates this novel AI-driven Third Harmonics Imaging B-mode ultrasound technology. Further studies are necessary to evaluate this technology more detailed.
**Funding for this study:** None.
**Ethics committee - additional information:** Local ethics committee.
**Author Disclosures:**
Paul Spiesecke: Nothing to disclose
Thomas Fischer: Nothing to disclose

**12:30-13:30** **Research Stage 1**

### Research Presentation Session: Physics in Medical Imaging

### RPS 913

### Striving for lower radiation dose and better image quality

**Moderator**
D. Kostova-Lefterova; Sofia/BG
*(dessi.zvkl@gmail.com)*

**Results from a decade (2012-2021) of periodical patient dose surveys for CT in Belgium**
*A. S. L. Dedulle*, T. Vanaudenhove, K. Van Slambrouck, A. Fremout; Brussels/BE
*(andedulle@gmail.com)*

**Purpose or Learning Objective:** In Belgium, diagnostic reference levels (DRLs) are established based on periodical patient dose surveys carried out by the regulatory body. This study evaluates trends in doses from CT scans, using data from these surveys.
**Methods or Background:** From 2012 to 2021, 10 periodical patient dose surveys were conducted in Belgium, covering CT equipment nationwide, as participation in the surveys is mandatory. Anonymous patient dose data were collected for 10 types of CT examinations (abdomen, chest angiography, coronary angiography, colon, cervical spine, lumbar spine, skull, sinus, thorax, thorax-abdomen). For each type of examination, dose data (CTDIvol, DLP) were registered for minimal 30 adult patients per CT device. The typical dose value was calculated (median) for each type of examination and each CT device. DRLs for each type of examination along with other statistical parameters were derived from the distribution of these values.
**Results or Findings:** The participation rate exceeded 85% across all 10 surveys. Between 2012 and 2021, the 75th percentile of the typical DLP-values for complete examinations showed a decrease between 22% and 63%, depending on the type of examination. Additionally, the data spread narrowed between 8% and 65%, and the 95th percentiles decreased between 16% and 62%. The DRL for CTDIvol per acquisition reduced between 31% and 71%, while the 95th percentile of the typical CTDIvol-values decreased between 38% and 71%. For most examination types, the largest decrease in DRL was obtained during the first five periodical surveys.
**Conclusion:** Over the 10-year period, patient doses from CT scans in Belgium substantially decreased. This is reflected in both lower DRLs and a reduced spread in dose data. This decrease is likely the result of improved protocols and the introduction of advanced CT technology.
**Limitations:** No limitations were identified.
**Funding for this study:** No funding was received.
**Ethics committee - additional information:** Not applicable.
**Author Disclosures:**
An Saskia Luc Dedulle: Nothing to disclose
Thibault Vanaudenhove: Nothing to disclose
Katrien Van Slambrouck: Nothing to disclose
An Fremout: Nothing to disclose

**Accounting for imaging dose in Hodgkin's lymphoma patients undergoing PBS proton therapy and photon VMAT: a SINFONIA study**
*M. Azizi*[1], M. Romero-Expósito[2], I. Múñoz[2], A. Gkavonatsiou[2], O. Norrlid[2], C. Goldkuhl[3], D. Molin[2], I. Toma-Dasu[1], A. Dasu[2]; [1]Stockholm/SE, [2]Uppsala/SE, [3]Gothenburg/SE
*(mona.azizi@fysik.su.se)*

**Purpose or Learning Objective:** This project aimed to fill a knowledge gap on the magnitude of secondary doses including the out-of-field and the imaging doses contributing to the risks from photon and proton radiotherapy.
**Methods or Background:** A framework was developed for determining and integrating the imaging and therapy doses for individual determination of total organ doses. VirtualDose software [1] was used for dose determinations from individual CT scans, while Monte Carlo simulations were used for CBCT dose determinations. Synthetic whole body CTs from the individual planning CTs were generated using IS2aR-software [2]. Neutron doses in proton radiotherapy were calculated using MCNP. Out-of-field doses in photon therapy patients were determined with Periphocal3D [3].

**Results or Findings:** To our knowledge, this was the first systematic assessment of total dose administered to patients throughout the course of the radiotherapy, encompassing clinically relevant frequency of use of the imaging procedures. The numbers of kV-CBCT ranged from 3 to 17 and 3 to 11 CTs, respectively in photon and proton plans. Imaging doses contribute 60-570 mSv for photon and 6-200 mSv for proton treatments over the entire treatment course for organs close to the target. Distant organs like the stomach, bladder, and liver showed a 13.5% increase in imaging dose relative to the photon treatment dose, while PBS indicates a 400% increase (though with lower absolute doses), indicating its greater relative impact.
**Conclusion:** Radiation burden in high precision radiotherapy depending on the imaging protocols will have to be taken into account in epidemiological studies on the incidence of second cancers in future patient cohorts. References: [1] A. Ding et al., Phys Med Biol; 2015. [2] I. S. Muñoz-Hernández et al., Phys. Medica, 2023. [3] B. Sánchez-Nieto, et al., Front. Oncol., 2022.
**Limitations:** No limitation was identified.
**Funding for this study:** This project has received funding from Euratom's research and innovation programme 2019-20 under grant agreement no. 945196.
**Ethics committee - additional information:** The study is retrospective.
**Author Disclosures:**
Mona Azizi: Nothing to disclose
Alexandru Dasu: Nothing to disclose
Maite Romero-Expósito: Nothing to disclose
Ola Norrlid: Nothing to disclose
Angeliki Gkavonatsiou: Nothing to disclose
Iuliana Toma-Dasu: Nothing to disclose
Christina Goldkuhl: Nothing to disclose
Daniel Molin: Nothing to disclose
Isidora Múñoz: Nothing to disclose

**Fetal radiation dose from iodine-125 seeds in pregnant breast cancer patients**
*J. Pluim*[1], J. Van De Kamer[2], E. Heeling[2], I. Ploeg[2], D. Hulsen[1]; [1]'s-Hertogenbosch/NL, [2]Amsterdam/NL

**Purpose or Learning Objective:** The treatment of breast cancer during pregnancy (PrBC) requires careful consideration of consequences for both maternal and fetal health. In non-pregnant patients, the use of radioactive iodine-125 (125I)-seeds is standard practice for localising non-palpable breast tumors before breast-conserving surgery. However, the use of 125I-seeds in pregnant patients has been avoided due to concerns about fetal radiation exposure.
**Methods or Background:** This study developed a mathematical model to estimate the fetal absorbed dose based on several factors: the radioactivity of the 125I-seed, the duration of implantation, and the distance between the 125I-seed and fetus as a function of maternal anatomy, gestational age, and fetal development. Three scenarios, representing a range of maternal and fetal anatomy, were evaluated, including a worst-case scenario from a radiation safety perspective.
**Results or Findings:** The results show that the fetal absorbed dose varies across the three scenarios, with ranges of 0–1.6 mGy, 0.0–1.0 mGy, and 0.0–0.4 mGy, depending on when the 125I-seed was implanted and when it was removed. These dose ranges are similar to conventional diagnostic x-ray scans. The maximum calculated absorbed dose (1.6 mGy) is unlikely to be reached in practice and is well below the 100 mGy threshold associated with possible fetal malformations. The associated cancer risk increase (0.016%) is minimal.
**Conclusion:** The use of 125I-seeds as localisation method of breast tumors in pregnant patients results in low fetal radiation doses and should not be avoided due to dose concerns.
**Limitations:** No limitations were identified.
**Funding for this study:** No funding was received for this study.
**Ethics committee - additional information:** Not applicable
**Author Disclosures:**
Eva Heeling: Nothing to disclose
Iris Ploeg: Nothing to disclose
Dennis Hulsen: Nothing to disclose
Jip Pluim: Nothing to disclose
Jeroen Van De Kamer: Nothing to disclose

**Implementing novel optimization strategies in x-ray interventional cardiology imaging for paediatric examinations with a simulation framework**
R. Massera, *N. W. Marshall*, H. Bosmans; Leuven/BE
*(nicholas.marshall@uzleuven.be)*

**Purpose or Learning Objective:** To apply novel optimization strategies in the search for optimal x-ray technique factors in paediatric interventional cardiology examinations.
**Methods or Background:** A simulation framework previously developed for adult interventional radiology examinations was adapted to use paediatric phantoms. The optimization framework implemented the Monte Carlo (MC) code PENELOPE(2018)/penEasy(2020) for dose and image quality (IQ) calculations, combined with a ray-tracing routine to calculate the attenuation through the patient and table. A figure of merit (FOM) defined as $SDNRw^2(u)$/Dose was used. SDNRw(u) is a signal-difference-to-noise ratio weighted for the impact of geometric blurring from the focal spot and from object motion. This was evaluated for the task of detecting a 0.36 mm diameter iron guidewire. Dosimetric quantities comprised incident air kerma (AK) at the reference point, used to approximate skin dose, and the effective dose (Deff), used to estimate stochastic risk. To calculate Deff and SDNRw(u), ICRP female paediatric phantoms of 1- and 5-year-old were used. The tube voltage, spectral copper filtration and x-ray focus that yielded the highest FOM value for a particular dose quantity were found, taking into consideration x-ray tube loading limitations.
**Results or Findings:** For the 1-year-old and 5-year-old phantoms, optimal FOM values were achieved at respectively 65kV/0.7 mm Cu/micro focus and 64kV/0.5 mm Cu/small focus, when AK was the cost function. Using effective dose as the cost function gave optimal factors of 59kV/0.5 mm Cu/micro focus and 60kV/0.3 mm Cu, for the 1-year-old and 5-year-old cases, respectively.
**Conclusion:** The framework was successfully adapted to work with paediatric phantoms. Optimization based on effective dose selected lower tube voltages and copper spectral filtration thicknesses compared to a typical optimization using incident air kerma.
**Limitations:** A limited number of phantoms were used in the simulations.
**Funding for this study:** This study is the result of a research agreement with Siemens Healthineers.
**Ethics committee - additional information:** Na
**Author Disclosures:**
Nicholas William Marshall: Nothing to disclose
Rodrigo Massera: Nothing to disclose
Hilde Bosmans: Other: Research agreement with Siemens Healthineers

**Image Quality in lung cancer screening LDCT: comparing the NELSON trial to current conventional and photon-counting thoracic CT**
*K. Torfs*[1], D. Petrov[1], L. D'Hondt[2], M. Lefere[3], K. Bacher[2], A. Snoeckx[4], W. De Wever[1], H. Bosmans[1]; [1]Leuven/BE, [2]Gent/BE, [3]Bonheiden/BE, [4]Zandhoven/BE
*(kwinten.torfs@uzleuven.be)*

**Purpose or Learning Objective:** Current guidelines for lung-cancer-screening (LCS) with low-dose chest CT (LDCT) are focused on dose, without specifying image quality (IQ) targets. This study compares noise and resolution in patient scans between the NELSON LCS trial, an ultra-low-dose (ULDCT) LCS study and current clinical standard-dose (SDCT) and LDCT on both energy-integrating (EIDCT) and photon-counting CT (PCCT).
**Methods or Background:** IQ was measured in 54 patient scans (24-26cm water-equivalent-diameter, sharp, 1mm slice-thickness reconstructions) of 6 protocols: LDCT-NELSON (Siemens Sensation 16), SDCT-EIDCT, LDCT-EIDCT and ULDCT-EIDCT (Siemens SOMATOM Force) and LDCT-PCCT and SDCT-PCCT (Siemens Naeotom Alpha). Noise was computed per scan by averaging global-noise-levels (GNL) for soft tissue (0-170HU) from 50 equidistant slices. Resolution was quantified using AUC of the digital modulation-transfer-function (MTF) measured from the patient skin-air-interface. Protocol averages were presented as: [kVp|reconstruction kernel|CTDIvol(mGy)|GNL-soft(HU)|MTF-AUC(mm-1)]. To assess standardized-condition-protocols, patient-specific influence of pixel-size and dose was removed by predicting GNL at 1.6mGy CTDIvol and measuring presampled-MTF.
**Results or Findings:** The results can be summarized as follows: LDCT-NELSON [120|B50|1.6±0.2mGy|155±8HU|0.52±0.06mm-1] ULDCT-EIDCT [Sn100|Br64-IR3|0.16mGy|151±7HU|0.49±0.04mm-1] SDCT-EIDCT [120|Br54|5.8±1.6mGy|70±4HU|0.49±0.06mm-1] SDCT-PCCT [120|Bl56|4.8±0.6mGy|127±9HU|0.86±0.05mm-1] LDCT-PCCT [Sn100|Bl56-IR1|1.08±0.15mGy|129±3HU|0.71±0.06mm-1] Compared to LDCT-NELSON scans, noise was significantly lower (p<0.001) in SDCT-EIDCT, SDCT-PCCT and LDCT-PCCT and the AUC-MTF significantly sharper (p<0.001) in SDCT-PCCT and LDCT-PCCT. ULDCT had similar noise and resolution properties as LDCT-NELSON, at a mean dose of only 0.16mGy versus 1.6mGy. However, for standardized-conditions, LDCT-PCCT, SDCT-EIDCT and ULDCT-EIDCT protocols were inherently less noisy (p<0.01) than NELSON, with SDCT-PCCT, LDCT-EIDCT and LDCT-PCCT being significantly sharper (p<0.001).
**Conclusion:** We have proposed a method to compare IQ of successful historical LCS scans to current state-of-the-art candidates with a dose – image quality evaluation from patient CT scans. Taking the NELSON setting as minimal reference, there are several candidate (ultra)LDCT protocols, with the LDCT on PCCT outperforming.
**Limitations:** Limited no. cases
**Funding for this study:** This work was performed with a grant from Kom op Tegen Kanker (G0B1922N), a Flemish NGO active in the fight against cancer

**Ethics committee - additional information:** Study approved under internal reference number S68527
**Author Disclosures:**
Mathieu Lefere: Nothing to disclose
Klaus Bacher: Nothing to disclose
Kwinten Torfs: Nothing to disclose
Louise D'Hondt: Nothing to disclose
Annemiek Snoeckx: Nothing to disclose
Hilde Bosmans: Nothing to disclose
Dimitar Petrov: Nothing to disclose
Walter De Wever: Nothing to disclose

**Combining rapid kVp-switching and photon-counting detectors for high-resolution spectral CT imaging at ultra-low doses**
*O. Sandvold*[1], R. Proksa[1], A. Perkins[2], P. Noël[1]; [1]Philadelphia, PA/US, [2]Cleveland, OH/US

**Purpose or Learning Objective:** This work presents a CT acquisition paradigm utilizing sparse spectral imaging to deliver both high spatial resolution and spectral imaging, specifically designed for pediatric imaging.
**Methods or Background:** Combining spectral imaging with high spatial resolution at ultra-low doses is challenging with current technology. In our method, most of the scan is captured using single low tube voltage with the detector operating in non-spectral, high-resolution mode by combining x-ray photons across energy bins (excluding electronic noise). During sparse intervals, the system switches to rapid kVp mode, leveraging the detector's spectral capabilities. Data is continuously acquired and combined to generate both high-resolution and spectral images. A Monte Carlo simulation demonstrated this pediatric imaging protocol, using 70 kVp with intermittent 110 kVp pulses for spectral data. The detector pixel size was set to 0.5x0.5 $mm^2$, with an additional sampling protocol investigated using 1x1 $mm^2$ pixels. The simulated phantom represented a 150 mm pediatric patient. Spectral SNR in monoenergetic images was estimated using the Cramér-Rao Lower Bound, and the area under the monoenergetic curve (AUMC) was calculated as the total SNR over 35–120 keV.
**Results or Findings:** The sparse spectral protocol improved AUMC spectral SNR by 220% compared to a constant 100 kVp photon-counting scan using the same dose level and pixel size. Binning pixels to measure 1x1 $mm^2$, the sparse spectral performance was 475% the 100 kVp reference scan AUMC. At 62% of the 100 kVp dose, the sparse protocol AUMC was 170% greater than reference AUMC.
**Conclusion:** Pediatric spectral CT faces three main challenges: achieving high spatial resolution, obtaining low-noise spectral data, and minimizing radiation dose. Our proposed acquisition method combines multiple technologies to address these challenges. Future clinical translation promises improved pediatric care with minimal radiation exposure.
**Limitations:** None
**Funding for this study:** None
**Ethics committee - additional information:** Not applicable
**Author Disclosures:**
Olivia Sandvold: Nothing to disclose
Peter Noël: Nothing to disclose
Roland Proksa: Nothing to disclose
Amy Perkins: Employee: Philips Healthcare

**A Machine Learning-based method for predicting normalized glandular dose coefficients and associated uncertainty in digital mammography and digital breast tomosynthesis**
*A. Sarno*[1], R. Massera[2], G. Paternò[3], P. Cardarelli[3], N. W. Marshall[2], H. Bosmans[2], K. Bliznakova[4]; [1]Milan/IT, [2]Leuven/BE, [3]Ferrara/IT, [4]Varna/BG
*(antonio.sarno@unimi.it)*

**Purpose or Learning Objective:** To investigate the use of a machine learning algorithm and patient-derived digital breast phantoms for predicting normalized glandular dose (DgN) coefficients and factors that influence the DgN uncertainty in digital mammography (DM) and digital breast tomosynthesis (DBT).
**Methods or Background:** Monte Carlo dosimetry calculations were performed for a set of 126 anatomically realistic digital breast phantoms to establish the ground truth DgN. The DgN was then predicted using a linear regression with an Automatic Relevance Determination Regression algorithm from 5 anatomical breast features: compressed breast thickness, glandular fraction, total glandular volume, center of mass and standard deviation of the glandular tissue distribution in the cranio-caudal direction. An algorithm for data imputation was explored to account for the cases where the latter two features are not available. The regression algorithm was validated using 5-fold Cross Validation.
**Results or Findings:** With the use of all 5 selected anatomical features, average difference between predicted DgN and the ground truth was 1%, with 50% of cases differing from the ground truth by less than 3%; estimated uncertainty on the DgN values was 9%. Uncertainty on DgN coefficients increased to 17% when the features related to the glandular distribution were excluded; however, this had only a minor impact on the prediction accuracy. The data imputation algorithm reduced the uncertainty on the predicted values, but could not match the prediction performance obtained by using all the available anatomical features.
**Conclusion:** The proposed methodology predicts the normalized glandular dose in DM and DBT with an error of 1%, on average, and with an estimated uncertainty of only 9%. 50% of the predicted DgN coefficients differed by less than 3% from the ground truth.
**Limitations:** Limited to single DM/DBT geometry
**Funding for this study:** None
**Ethics committee - additional information:** Not applicable
**Author Disclosures:**
Nicholas William Marshall: Nothing to disclose
Gianfranco Paternò: Nothing to disclose
Kristina Bliznakova: Nothing to disclose
Rodrigo Massera: Nothing to disclose
Hilde Bosmans: Nothing to disclose
Antonio Sarno: Nothing to disclose
Paolo Cardarelli: Nothing to disclose

**12:30-13:30** **Research Stage 2**

### Research Presentation Session: Paediatric

### RPS 912
### Insights into foetal imaging

**Moderator**
M. Rebollo Polo; Barcelona/ES
*(monica.rebollo@sjd.es)*

**Validation of fetal brain 3D slice-to-volume registration (SVR) in detecting the cause of antenatal ventriculomegaly confirmed by neonatal scan**
*W. H. E. Hamed*, G. Kendall, L. Dyet, L. Srinivasan, D. Peebles, A. David, M. Sokolska, K. P. Baruteau; London/UK
*(weaam.hamed@nhs.net)*

**Purpose or Learning Objective:** Validates 3DSVR for detecting anatomical and structural pathologies in fetal MRI and assesses quality improvement in a cohort of antenatal ventriculomegaly confirmed by neonatal MRI.
**Methods or Background:** Detecting subtle anatomical abnormalities in fetal brain MRI is challenging due to motion artefacts and the limited spatial resolution of 2D slices. Recently, slice-to-volume reconstruction (SVR) software has been developed to realign multiple 2D stacks into a high-resolution 3D volume (3DSVR), enabling better visualisation through multiplanar reconstruction. However, clinical validation of 3D-SVR is limited due to lack of ground truth data. A retrospective cross-sectional study was conducted on pregnancies with ventriculomegaly. Inclusion criteria included fetal and neonatal MRI performed with standard protocols and 3DSVR. The median gestational age at fetal MRI was 28weeks (range 21-33w), and at neonatal MRI, 1week (range 1d-4w). Ventriculomegaly causes were assessed on fetal 2DT2w-HASTE and 3DSVR and confirmed with 2DT2w-TSE on neonatal scans. Eleven brain structures were scored on a 3-point visibility scale, and image quality was rated based on signal-to-noise ratio (SNR) and motion artefacts. Statistical analysis was performed using the Wilcoxon signed-rank test.
**Results or Findings:** Of 20 subjects, eight had aqueduct stenosis identified on neonatal MRI. This was confirmed in 3/8 on 2D and 8/8 on 3DSVR. Fetal 3DSVR improved visibility scores in six of eleven structures, with significant differences in PLIC (0.65vs1.85, $p<0.001$), Sylvian aqueduct (1.05vs1.9, $p<0.001$), olfactory bulbs (0.9vs1.7, $p<0.01$), and grey-white matter contrast (0.9vs1.9, $p<0.01$). SNR improved in 35% of scans, and motion artefacts were reduced in 25%.
**Conclusion:** 3DSVR provides improved diagnoses of aqueduct stenosis, as evidenced by comparison with neonatal ground-truth scans. This is achieved by improving the visibility and overall quality of fetal brain MRI. Future work will validate 3DSVR in other pathologies.
**Limitations:** Not applicable.
**Funding for this study:** No funding was provided for this study.
**Ethics committee - additional information:** The study is retrospective.

**Author Disclosures:**
Magdalena Sokolska: Nothing to disclose
Donald Peebles: Nothing to disclose
Giles Kendall: Nothing to disclose
Kelly Pegoretti Baruteau: Nothing to disclose
Leigh Dyet: Nothing to disclose
Weaam Hamed Elsayed Hamed: Nothing to disclose
Latha Srinivasan: Nothing to disclose
Anna David: Nothing to disclose

**Quantification of pathological fetal brain development through segmentation: a novel generative AI approach for synthetic pathological data generation**

*M. Kaandorp*[1], H. Asma-Ull[2], H. G. Kim[2], D. Agbelese[1], K. Payette[1], A. Jakab[1]; [1]Zurich/CH, [2]Seoul/KR
*(Misha.Kaandorp@kispi.uzh.ch)*

**Purpose or Learning Objective:** Fetal MRI is increasingly used for the quantification of the developing brain through segmentation of various anatomical structures. One of the challenges is that current segmentation algorithms perform poorly in cases with ventriculomegaly due to limited high-quality annotations. Additionally, privacy concerns often restrict data sharing. We aimed to overcome these challenges through the generation of realistic synthetic pathological MRIs from manipulated healthy label images using generative AI.
**Methods or Background:** We trained a stable diffusion model for 3D brain MRI synthesis (Med-DDPM) using 727 fetal and preterm neonatal MRI-label image pairs. We generated 47 synthetic ventriculomegaly label images (S47-ventriculomegaly) from 33 healthy fetal MRIs (R33-healthy) by dilating ventricular labels. Combining label images from R33-healthy and S47-ventriculomegaly, we generated 80 synthetic MRIs (S80-ventriculomegaly) using Med-DDPM and visually assessed their quality. To evaluate segmentation performance, we trained two nnUNet models on S80-ventriculomegaly or R33-healthy MRI-label pairs and measured performance using Dice score on 40 test cases from FeTA2021 Challenge and 26 spina bifida severe ventriculomegaly cases (SPINABIFIDA). Performance was also assessed for 80 healthy and pathological MRI-label pairs (R80) and their synthetic equivalent (S80).
**Results or Findings:** Med-DDPM generated diverse, high-quality synthetic fetal MRIs. In segmentation tasks, S80-ventriculomegaly outperformed R33-healthy, achieving higher Dice scores in FeTA2021 (0.773 vs. 0.751) and SPINABIFIDA (0.759 vs. 0.722), especially for ventricles (0.780 vs. 0.672). S80 also surpassed R80 in SPINABIFIDA (0.822 vs. 0.815), with comparable performance in FeTA2021 (0.766 vs. 0.773).
**Conclusion:** This study demonstrates that generating realistic pathological fetal MRIs by manipulating labels from normally developing subjects can enhance data augmentation and data anonymization in prenatal imaging. This is an important step towards addressing privacy concerns while improving segmentation performance.
**Limitations:** Our method could be expanded to include more pathologies, increasing clinical applicability.
**Funding for this study:** Funding was provided by SNF grant: IZKSZ3_218590
**Ethics committee - additional information:** The study has been approved by the ethics committee of Zurich Children's Hospital, decision number: 2022-01157)
**Author Disclosures:**
Hyun Gi Kim: Nothing to disclose
Andras Jakab: Nothing to disclose
Hosna Asma-Ull: Nothing to disclose
Kelly Payette: Nothing to disclose
Damola Agbelese: Nothing to disclose
Misha Kaandorp: Nothing to disclose

**Feasability of fetal cardiac MRI in the prenatal evaluation of congenital heart defects in comparison to US**

*G. Biechele*[1], B. Stos[2], D. Laux[2], S. Stöcklein[1], D. Grevent[2], L. J. Salomon[2]; [1]Munich/DE, [2]Paris/FR
*(gloria.biechele@med.uni-muenchen.de)*

**Purpose or Learning Objective:** Congenital heart defects (CHD) are common severe birth defects, resulting in a clinical need for precise prenatal assessment. Magnetic Resonance Imaging (MRI) of the fetal heart recently became feasible with the advent of novel gating techniques , but its feasibility has not yet been systematically evaluated in pregnancies with fetal CHD.
**Methods or Background:** This study yet evaluated 42 singleton pregnancies, consisting of 25 fetuses (60%) with diagnosis of CHD and 17 healthy control fetuses (40%). Diagnosis of CHD was refined by a cardiopediatrician. All MRI images were reviewed by a single blinded operator. Image quality of cardiac sequences was rated from 0 (not usable) to 5 (high image quality in multiple orientations). Cardiac anatomy was evaluated and correlation between MRI findings and US diagnosis was assessed.
**Results or Findings:** Fetal cardiac MRI was feasible in 40 patients (image quality score >0) due to extensive fetal movement in n=2 cases. Among, in n=13 (32.5%), n=21 (52.5%) and in n=6 (15.0%) cases, image quality was rated high (image quality score 5), medium (image quality score 3-4) and poor (image quality score 1-2), respectively. Among the 25 fetuses with CHD, fetal cardiac MRI was capable to fully confirm US-diagnosis in n=19 cases (76.0%) and to partly confirm US-diagnosis in n=6 (24.0%) cases, mostly due to incomplete recording of structures of interest. There was a substantial agreement between MRI and US (κ-value 0.70 with an accuracy of 0.85).
**Conclusion:** Our preliminary study suggests a similar diagnostic performance of MRI to US.
**Limitations:** With improvements in sequences, gating techniques and operator experience, MRI examination might soon become an essential part of the prenatal management of CHD, not only to identify associated abnormalities but also to reinforce the assessment of the heart itself.
**Funding for this study:** DFG Walter-Benjamin-Stipendium (BI 2563/1-1, G.B.)
**Ethics committee - additional information:** Local ethic commitee number NCT 04142606
**Author Disclosures:**
Laurent J. Salomon: Nothing to disclose
Sophia Stöcklein: Nothing to disclose
David Grevent: Nothing to disclose
Bertrand Stos: Nothing to disclose
Daniela Laux: Nothing to disclose
Gloria Biechele: Equipment Support Recipient: Company Northh Medical for the lend of a cardiac gating device during the period of a fellowship abroad.

**Thoracic findings at early gestation fetal post-mortem micro-CT**

*I. C. Simcock*[1], A. Lamouroux[2], S. C. Shelmerdine[1], C. Hutchinson[1], N. Sebire[1], O. Arthurs[1]; [1]London/UK, [2]Nimes/FR
*(ian.simcock@gosh.nhs.uk)*

**Purpose or Learning Objective:** To identify the range and frequency of thoracic diagnoses at less-invasive autopsy, following a post-mortem micro-CT investigation for early gestation pregnancy loss.
**Methods or Background:** Micro-CT provides high-resolution imaging for early gestation fetuses (<300g), typically following miscarriage or termination of pregnancy, allowing parents a less invasive autopsy option. We retrospectively analysed micro-CT diagnoses made on an unselected population of over 1190 early gestation fetuses between 2017 and 2024 at our tertiary referral institution.
**Results or Findings:** Thoracic abnormalities on micro-CT were identified in (147/1190; 12.3%), comprising 148 cardiac and 79 non-cardiac individual abnormalities. The commonest cardiac abnormalities were septal defects (50/147; 34.0%), aortic abnormalities e.g. coarctation (23/147; 15.6%), congenital heart disease particularly tetralogy of Fallot (16/147; 10.9%) and ventricular abnormalities e.g. hypoplastic left heart (11/147; 7.5%). Most common non-cardiac chest abnormalities were abnormal fluid accumulation (41/147; 27.9%), which included pleural effusion (34/147; 23.1%), hydrops (4/147; 2.7%), and pericardial effusion (3/147; 2%). A thoracic wall defect was observed in (18/147; 12.7%) of cases.
**Conclusion:** A range of thoracic abnormalities were made by micro-CT in our cohort, commonly from cardiac causes. This information is useful for parents regarding the likelihood of congenital abnormalities in subsequent pregnancies and provides an alternative to conventional invasive autopsy.
**Limitations:** Single centre data from a large specialist centre. Not all parents consented to invasive autopsy, so some diagnoses could not be histologically confirmed.
**Funding for this study:** ICS was funded by a National Institute for Health Research (NIHR) Clinical Doctoral Research Fellowship (ICA-CDRF-2017-03-53), Development and Skills Enhancement Award (NIHR302390) and a Research for Patient Benefit Award (NIHR206174), OJA was funded by a NIHR Career Development Fellowship (NIHR-CDF-2017-10-037) and SCS is supported by a NIHR Advanced Fellowship Award (NIHR-301322), and the work is funded by the Great Ormond Street Hospital Children’s Charity. AL was funded by two mobility funding from Nimes and Montpellier university hospital All research at Great Ormond Street Hospital NHS Foundation Trust and UCL Great Ormond Street Institute of Child Health is made possible by the NIHR Great Ormond Street Hospital Biomedical Research Centre. The views expressed are those of the author(s) and not necessarily those of the NHS, the NIHR or the Department of Health & Social Care.

**Ethics committee - additional information:** We obtained ethical approval from the UK NHS Health Research Authority (HRA) Ethics Committee (IRAS ID: 131395).
**Author Disclosures:**
Ian C. Simcock: Nothing to disclose
Neil Sebire: Nothing to disclose
Audrey Lamouroux: Nothing to disclose
Ciaran Hutchinson: Nothing to disclose
Susan Cheng Shelmerdine: Nothing to disclose
Owen Arthurs: Nothing to disclose

**Optimizing Outcomes in PPROM: Ultrasound-Based Fetal Lung Maturity Analysis**
*A. Verma*, A. Malik; New Delhi/IN
*(vermaanimesh53@gmail.com)*

**Purpose or Learning Objective:** To evaluate fetal lung maturity using multiparametric ultrasound in pregnant women with preterm premature rupture of membranes (PPROM), and to determine the accuracy of ultrasound parameters in predicting neonatal respiratory distress in this population. This study highlights the role of multiparametric ultrasound in predicting neonatal respiratory distress.
**Methods or Background:** We conducted an 18 month observational prospective cohort study on 81 women with singleton pregnancies under 37 weeks gestation, complicated by PPROM with fetuses between the 10th and 90th weight percentiles. Fetal biometric measurements included biparietal diameter (BPD), head circumference (HC), abdominal circumference (AC), and femur length (FL). We also assessed distal femoral and proximal tibial epiphyseal ossification centers, placental maturity (Grannum system), and fetal main pulmonary artery (MPA) Doppler flow measuring resistive index (RI), pulsatility index (PI), acceleration time, and ejection time. These parameters were correlated with neonatal respiratory distress to predict fetal lung maturity in PPROM cases.
**Results or Findings:** BPD demonstrated highest diagnostic accuracy (81.48%) for predicting neonatal respiratory distress syndrome (RDS). The appearance of proximal tibial epiphyses showed remarkable sensitivity (92.31%) in predicting RDS. Among the Doppler parameters, fetal MPA resistive index (RI) showed the highest diagnostic accuracy (88.89%). Notably, combined fetal main pulmonary artery indices yielded the highest diagnostic accuracy (95.06%), followed by combined fetal biometry (91.36%).
**Conclusion:** Fetal lung maturity, a key determinant for neonatal respiratory distress can be assessed by grayscale ultrasound and Doppler parameters. Multiparametric ultrasonographic assessment is a promising tool in prediction of neonatal RDS in women with PPROM.
**Limitations:** The study's generalizability is constrained by its limited sample size. Additionally, assessment of the fetal main pulmonary artery via ultrasonography is subject to inter-observer variability and may be affected by inherent artifacts in ultrasound and Doppler imaging techniques.
**Funding for this study:** The cost of this study was covered by VMMC and Safdarjung Hospital, which provided essential resources and infrastructure to ensure its completion.
**Ethics committee - additional information:** This study was approved by Institutional Ethics Committee, VMMC and Safdarjung Hospital, New Delhi (06/2022/CC-265)
**Author Disclosures:**
Amita Malik: Nothing to disclose
Animesh Verma: Nothing to disclose

**Prenatal Prediction of Fetal Lung Maturity Using 3D Lung Volume, Lung-to-Liver Intensity Ratio Tissue Histogram and Pulmonary Artery Doppler Indices**
A. Omar, A. Mohamed Tharwat, M. Aboelnasr, H. Abo-Ali Hamza, *S. A. Hassanein*, W. Gaber Eldamaty; Shebin El Kom/EG
*(shaimaahamid@hotmail.com)*

**Purpose or Learning Objective:** Fetal lung maturity assessment is the most critical factor for identifying the optimal delivery time. A non-invasive sonographic technique is necessary to evaluate fetal lung development. we aimed to predict maturity of fetal lung utilizing 3D lung volume ultrasound, lung to liver intensity ratio, and pulmonary artery doppler indices measurement
**Methods or Background:** A prospective observational study was conducted on 200 pregnant females with gestational age of 32 to 40 weeks age underwent 3D ultrasound (3DUS) for determination the fetal lung volume (FLV) and fetal lung-to-liver intensity ratio (FLLIR) (tissue histogram) with doppler examination of the main pulmonary artery (MPA) for the following parameters; acceleration-time to ejection-time ratio (At/Et), pulsatility index (PI) and resistive index (RI) during a period of one week from delivery and comparing the results to the neonatal outcome.
**Results or Findings:** Of 200 fetuses investigated; 113 cases (56.5%) were found to have respiratory distress syndrome. The MPA RI and PI were significantly greater in fetuses with respiratory distress syndrome comparing with those without (2.6± 0.3 and 0.9±0.05 vs. 1.9±0.3 and 0.8±0.2, respectively with p-value < 0.001 for both). MPA At/Et was significantly lesser for fetuses with RDS than fetuses without RDS (0.2±0.1 vs. 0.3±0.1 respectively, p-value less than 0.001). FLLIR was significantly lesser in RDS +ve group comparing with RDS -ve group (0.9±0.2 versus 1.3±0.3 respectively, p-value < 0.001) and FLV was significantly smaller in fetuses with respiratory distress syndrome comparing with those with no (31.5±2.5 vs. 38.1±2.8; p-value < 0.001).
**Conclusion:** The utilization of main pulmonary artery Doppler indices, together with mean fetal lung volume and FLLIR for assessing fetal lung is a quick non-invasive accurate technique for estimation of neonatal lung maturity and respiratory distress syndrome
**Limitations:** A uni-center study
**Funding for this study:** No funding was present.
**Ethics committee - additional information:** Menoufia faculty of medicine research ethics committee under code no: (4/2022OBSG35
**Author Disclosures:**
Amal Omar: Nothing to disclose
Mohamed Aboelnasr: Nothing to disclose
Ahmed Mohamed Tharwat: Nothing to disclose
Shaimaa Abdelhamid Hassanein: Nothing to disclose
Wael Gaber Eldamaty: Nothing to disclose
Haytham Abo-Ali Hamza: Nothing to disclose

**Intrauterine blood transfusion causes dose- and time-dependent signal alterations in the liver and the spleen on fetal magnetic resonance imaging**
M. Schwarz, V. Schmidbauer, *N. M. Nowak*, P. Kienast, D. Bettelheim, J. Binder, T. Reiberger, D. Prayer, G. Kasprian; Vienna/AT

**Purpose or Learning Objective:** This study aimed to investigate the effects of IUTs on MRI findings in the fetal liver and spleen.
**Methods or Background:** Intrauterine transfusions (IUTs) are a life-saving treatment for fetal anemia. However, with each transfusion iron bypasses uptake regulation through the placenta and accumulates in fetal organs. Unlike other imaging modalities, fetal magnetic resonance imaging (MRI) is capable of non-invasively assessing fetal liver disease and/or organ iron overload. This study aimed to investigate the effects of IUTs on MRI findings in the fetal liver and spleen. For this retrospective study, we included eight fetuses undergoing IUT and prenatal MRI from 2014 to 2023. The fetuses were gestational age-matched with a cohort that received fetal MRI for other indications, but no IUTs. Signal intensity (SI) and volumetric analyses of the liver and the spleen were performed.
**Results or Findings:** Fetuses receiving transfusions had significantly larger volumes of both liver (p=0.003) and spleen (p=0.029). T1 SI inversely correlated with the number of IUTs (Pearson's r=-0.43, p = 0.099). This effect regressed over time (r=0.69, p=0.057). T2 SI did not correlate significantly with transfusion frequency but showed a strong positive correlation with the number of days between IUT and MRI (r=0.91, p=0.002). For splenic SI measures, similar effects were observed regarding T1 SI reduction per received transfusion (r=-0.36, p=0.167) and recovery of T2 SI after IUT (r=0.88, p=0.004).
**Conclusion:** This is the first study to report the effects of IUTs on MRI data of fetal livers and spleens. We observed considerable dose- and time-dependent SI alterations of the liver and spleen following IUT. Furthermore, fetal hepatosplenomegaly can be expected following IUT.
**Limitations:** Two fetuses have had parvovirus B19 infection, which may cause hepato-/splenomegaly, but may also mandate the need for IUT.
**Funding for this study:** Medical University of Vienna
**Ethics committee - additional information:** In concordance with the principles of the Declaration of Helsinki, approved by the ethics committee of the Medical University of Vienna.
**Author Disclosures:**
Victor Schmidbauer: Nothing to disclose
Nikolaus Michael Nowak: Nothing to disclose
Julia Binder: Nothing to disclose
Michael Schwarz: Nothing to disclose
Dieter Bettelheim: Nothing to disclose
Daniela Prayer: Nothing to disclose
Thomas Reiberger: Nothing to disclose
Patric Kienast: Nothing to disclose
Gregor Kasprian: Nothing to disclose

**Improving image quality and decreasing SAR with high dielectric constant pad in 3.0T fetal MRI**

*Z. Zhu*, C. Yan, Z. Lin, B. Zhang; Nanjing/CN
*(161230042@smail.nju.edu.cn)*

**Purpose or Learning Objective:** To assess the potential of high dielectric constant (HDC) pad in increasing image quality and decreasing specific absorption rate (SAR) in 3.0T fetal MRI.
**Methods or Background:** This prospective single-center observational study included 168 pregnant participants taking 3.0T fetal MRI scanning with and without HDC pad between 1 May 2021 and 31 November 2023. Quantitative Image-quality analysis included signal-to-noise ratio (SNR) and contrast-to-noise ratio (CNR). Qualitative analysis was performed by three radiologists with four-point scale to evaluate overall image quality, dielectric artifact and diagnostic confidence. Whole-body total SAR was also compared. Correlation between image quality variable changes and participant clinical characteristics was evaluated using spearman correlation.
**Results or Findings:** 128 participants (mean gestational age 30.25±3.53 weeks, range 22-37 weeks) undertook balanced steady state free precession (bSSFP) sequence and 40 participants (mean gestational age 30.38±3.50 weeks, range 23-37 weeks) undertook single-shot fast spin-echo (SSFSE) sequence. With HDC pad, SNR and CNR was significantly higher (41.45% increase in SNR, 54.05% increase in CNR on bSSFP, $p<0.001$; 258.76% increase in SNR, 459.55% increase in CNR on SSFSE, $p<0.001$). Overall qualitative image quality, dielectric artifact and diagnostic confidence improved significantly ($p<0.001$). Adding HDC pad significantly reduced Whole-body total SAR (32.60% on bSSFP, $p<0.001$; 15.40% on SSFSE, $p=0.005$). There was no significant correlation between image quality variable changes and participant clinical characteristics ($p>0.05$).
**Conclusion:** In a clinical setting, adding HDC pad can increase overall quantitative and qualitative image quality while reducing dielectric artifact and SAR.
**Limitations:** Due to technical limitations, we could not conduct further analysis to compare the radiofrequency power deposited on uterus, whole fetal body and fetal brain separately. We only conducted experiments on magnetic resonance machines produced by United Imaging and didn't include machines from other manufactures.
**Funding for this study:** National Science and Technology Innovation 2030 (2022ZD0211800)
**Ethics committee - additional information:** Ethic committe of Nanjing Drum Tower Hospital
**Author Disclosures:**
Bing Zhang: Nothing to disclose
Zhengyang Zhu: Nothing to disclose
Zengping Lin: Nothing to disclose
Chenchen Yan: Nothing to disclose

| 12:30-13:30 | Research Stage 3 |
|---|---|

### Research Presentation Session: Hybrid, Molecular and Translational Imaging

### RPS 906

### Exploring the frontiers in hybrid and molecular imaging

**Moderator**
M. Naik; London/UK
*(m.naik@nhs.net)*

**Low-dose Fibronectin-targeted Gd-based contrast agent Enables Early and Accurate Assessment of Chemotherapy Response in Pancreatic Cancer**

*W. Zhang*, X. Liang, Y. Du, J. Tian, N. Hong; Beijing/CN

**Purpose or Learning Objective:** Albumin-bound paclitaxel and gemcitabine (AG) chemotherapy is a mainstay in treatment of pancreatic ductal adenocarcinoma (PDAC), unfortunately not all patients respond to this treatment. Clinical imaging techniques cannot precisely evaluate and predict the response to AG therapies over several weeks. A strong fibrotic reaction is a hallmark of drug-resistance while depletion of fibrosis is a positive response to AG. Extradomain-B fibronectin (EDB-FN) is an important element of fibrosis in PDAC. Here, we prepared EDB-FN targeted Gd-based contrast agent (EDB-Gd) to perform molecular MRI for early, noninvasive and quantitative assessment of treatment response in PDAC.
**Methods or Background:** BxPC-DR was pre-treated with AG to establish acquired drug resistance. Subcutaneous and orthotopic models with BxPC-DR or BxPC were established. Mice were intravenously injected with EDB-Gd or Gd-DOTA. The ratio of T1 value reduction (T1d%) were compared quantitatively. For chemotherapy monitoring, MRI was performed before and after AG treatments. Histological analyses were used for validation.
**Results or Findings:** Molecular MRI with EDB-FN could specifically detect and quantify fibrogenesis in PDAC xenografts at a low dose 0.05mmol/kg, which is half of clinical dosage of Gd. The optimal imaging time point was 30min after injection of EDB-Gd. In addition, the targeted probe generated more robust contrast-enhanced and longer retention compared to traditional Gd-DOTA. For chemotherapy montoring, T1d% were significantly increased 2.5-fold in drug-resistance xenografts group in fibrotic tumor areas compared to AG-sensitive group ($p < 0.05$). Comparing the T1d% before and 5 days after AG predicted treatment response.
**Conclusion:** This study indicates EDB-FN-targeted molecular MRI possesses clinical applications in accurate assessment and prediction of AG chemotherapy.
**Limitations:** Correlations between the observed fibrotic changes on MRI with pathologic markers of treatment response and even survival and prognosis are needed in the future.
**Funding for this study:** This study was funded by the Beijing Natural Science Foundation (Grant No. 7244524, 7212207) and National Natural Science Foundation of China (Grant Nos. 62027901, 82272111, 92159303, 82071896, 81871422, 81871514, and 81227901).
**Ethics committee - additional information:** All experimental studies were approved by the Ethics Committee of the Peking University People's Hospital (2024PHE048).
**Author Disclosures:**
Jie Tian: Nothing to disclose
Yang Du: Nothing to disclose
Nan Hong: Nothing to disclose
Wenjia Zhang: Nothing to disclose
Xiaolong Liang: Nothing to disclose

**Investigating the Relationship of Endothelin Receptor Expression and Tumor Hypoxia by Optoacoustic Tomography**

*A. Helfen*, M. Mallik, M. Stölting, E. Hoffmann, C. Höltke; Münster/DE
*(anne.helfen@ukmuenster.de)*

**Purpose or Learning Objective:** A significant prognostic factor of tumor malignancy is the formation of a hypoxic supportive tumor microenvironment (TME). The endothelin (ET)M signaling network is linked to tumor hypoxia through stabilized hypoxia-inducible factor 1 in a feedback loop. Here, we examined the interrelation of both cellular signaling systems by multispectral optoacoustic tomography (MSOT).
**Methods or Background:** Murine syngeneic 4T1 breast tumors were examined in vivo using MSOT depicting deoxygenated (Hb) and oxygenated (HbO2) hemoglobin content to detect hypoxic regions over one week. An exogenous fluorescent endothelin-A receptor (ETAR) probe served for evaluating ETAR expression status. Therapeutic interventions (anti-angiogenic and macrophage depletion) were evaluated concerning Hb/HbO2 ratio and ETAR expression changes. Treatment response to Bevacizumab, Clodronate and Sorafenib was detected over one week.
**Results or Findings:** MSOT was capable of delineating and quantifying hypoxia within tumor lesions. 4T1 tumors were highly hypoxic compared to healthy tissue, represented by oxygen saturation (sO2) of 0.33 vs. 0.79, respectively. Baseline data of the ETAR probe showed an initial rise in signal intensity from day 0 to day 8, corresponding to tumor growth. Therapeutic interventions showed that the ETAR signal intensity could be significantly reversed, while all applied therapies did not lead to significant tumor growth reduction. However, Sorafenib and Bevacizumab led to a significant increase in sO2 values. MSOT data were supported by subsequent immunohistochemistry.
**Conclusion:** Tumor hypoxia within syngeneic murine breast cancer can be visualized non-invasively by MSOT evaluating hemoglobin (oxygenated and deoxygenated ratios) as well as ETAR expression. Furthermore, MSOT was able to depict therapeutic effects already in the early course of treatment representing a potential imaging biomarker.
**Limitations:** Limited penetration depth of MSOT is significantly improved compared to optical imaging, but with regard to translation still mainly suitable for superficial tissues.
**Funding for this study:** Financial support from the German Research Foundation (DFG, SF656 A04), from the medical faculty of the University of Münster (IMF: I-HÖ111709) and from the Joachim Herz Stiftung is gratefully acknowledged.
**Ethics committee - additional information:** All animal experiments described in this study were approved by the responsible authorities ("Landesamt für Natur, Umwelt und Verbraucherschutz NRW", Germany, Protocol No. 84-02.04.2017.A011).

**Author Disclosures:**
Miriam Stölting: Nothing to disclose
Carsten Höltke: Nothing to disclose
Anne Helfen: Nothing to disclose
Emily Hoffmann: Nothing to disclose
Moushami Mallik: Nothing to disclose

### Theranostic Innovation: SPCCT-Triggered Photodynamic X-ray Therapy (XPDT) in a Murine Breast Cancer Model

*P. Akl*, A. Carret, A. Houmeau, A. Gautheron, I. Goddard, J-B. Langlois, B. Montcel, F. Lerouge, P. C. Douek; Lyon/FR
*(piaakl@gmail.com)*

**Purpose or Learning Objective:** Photodynamic therapy (PDT) uses light to activate photosensitizers (PS) but is limited by shallow penetration. X-ray PDT (XPDT) overcomes this by using X-rays for deeper activation. Spectral Photon-Counting Computed Tomography (SPCCT) enhances imaging with K-Edge capabilities. This study aims to optimize XPDT in vitro and in vivo using SPCCT and gadolinium nanoparticles (GdNp) as contrast and therapeutic agents.
**Methods or Background:** GdNp doped with terbium were synthesized, functionalized for biocompatibily with different coatings (PEG and Silica) and the photosensitizer, rose bengal (RB) and irradiated at different concentrations (0.02-1 M) in vitro using SPCCT with different X-ray dose parameters (80-140 KVp and 10-300 mAs). Luminescence emission was recorded using optical fiber immersed in the GdNp solution. 63 athymic nude mice with MDA-MB-231 cell-derived breast cancer xenograft models were irradiated using SPCCT(120kVp and 300mAs) 60 axial acquisitions/ 1 sec, meandose 30mGy, 1800mGy in total ,24 hours after intra tumoral injection. Safety, biodistribution, and therapeutic effects were assessed through mice examination every 3 days for tumor volume to assess tumor growth delay .Biodistribution was monitored using SPCCT imaging.
**Results or Findings:** Fluorescence emission increased with X-ray dose and concentration of GdNp. SPCCT imaging revealed specific distribution of GdNp, with luminescence emission proportional to the administered dose in vitro and in vivo. The silica + RB nanoparticle group showed a slight improvement in efficacy compared to controls, with delayed tumor growth. Biodistribution studies indicated relatively different patterns of intratumoral and peritumoral localization, and heterogenous distribution.
**Conclusion:** This technology shows potential for customizing and enhancing the efficacy of XPDT, modulate luminescence intensity by adjusting X-ray dose parameters and nanoparticle concentration. Silica + RB showed a slight improvement in efficacy compared to controls.
**Limitations:** Though further studies are required to confirm significance.
**Funding for this study:** EU Horizon 2020 grant agreement 899549
**Ethics committee - additional information:** ethical comittee IRB APAFIS#44558.
**Author Disclosures:**
Isabelle Goddard: Nothing to disclose
Jean-Baptiste Langlois: Nothing to disclose
Angele Houmeau: Nothing to disclose
Frederic Lerouge: Nothing to disclose
Philippe Charles Douek: Nothing to disclose
Bruno Montcel: Nothing to disclose
Alison Carret: Nothing to disclose
Arthur Gautheron: Nothing to disclose
Pia Akl: Nothing to disclose

### [18F]FDG-PET/CT imaging biomarkers for time point-matched response characterization of experimental melanomas to anti-PD-L1/anti-CTLA-4 immunotherapy

*M. J. Antons*, S. Kloiber-Langhorst, H. Hirner-Eppeneder, F. Herr, S. Ziegler, M. Brendel, J. Ricke, M. Heimer, C. C. Cyran; Munich/DE
*(melissa.antons@med.uni-muenchen.de)*

**Purpose or Learning Objective:** Three-time point [18F]FDG-PET/CT imaging allows for in vivo monitoring of a combined anti-PD-L1/anti-CTLA-4 immunotherapy in a murine melanoma model validated by time point-matched multiparametric immunohistochemical reference standard
**Methods or Background:** Melanoma cells (B16-F10) were injected subcutaneously into the abdominal flank of C57BL/6 mice (n=40). Following a baseline scan after day 7, the therapy group received 5 injections (i.p.) of anti-PD-L1 and anti-CTLA-4 antibodies on days 7, 9, 11, 13 and 15. The control group received sham treatment. Follow-up scans were performed on day 13 and 19. Tumor allografts were harvested for time point-matched immunohistochemistry (CD8, Ki-67, TUNEL) to validate PET/CT parameters (MTV, SUVmax) as imaging biomarkers of early therapy response.
**Results or Findings:** At follow-up 1 (FU-1), the therapy group exhibited significantly lower MTV compared to the control group (p=0.0037). By follow-up 2 (FU-2), both MTV and SUVmax were significantly lower in the therapy group versus the control group (MTV: p=0.0078; SUVmax: p=0.00034). Ex vivo analysis revealed significant anti-tumor effects in the therapy group, with a significantly higher apoptosis rate at FU-1 (p= 0.012) and FU-2 (p= 0.001). Moreover, the therapy group demonstrated a significant increase in CD8-positive T-cells at FU-2 (p=0.0027), while tumor cell proliferation was significantly lower at both follow-up time points (FU-1: p=0.012; FU-2: p=0.012).
**Conclusion:** Multi-time point [18F]FDG-PET/CT allowed for the early non-invasive monitoring of a combined immunotherapy with anti-PD-L1/anti-CTLA-4 in experimental melanomas, validated by multiparametric immunohistochemistry. The significantly lower tumor glucose metabolism was paralleled by significant pro-immunogenic, pro-apoptotic and anti-proliferative effects of the combined immunotherapy.
**Limitations:** The allograft model of melanoma may have limited translational relevance to human tumor pathophysiology. Secondly, the observation period was relatively short, and no clinical endpoints such as overall survival of the animals were determined.
**Funding for this study:** None
**Ethics committee - additional information:** All animal experiments were performed in accordance with the guidelines for the use of living animals in scientific studies and the animal study was officially approved (ROB-55.2-2532.Vet_02-19-32).
**Author Disclosures:**
Clemens C. Cyran: Nothing to disclose
Maurice Heimer: Nothing to disclose
Felix Herr: Nothing to disclose
Sibylle Ziegler: Nothing to disclose
Melissa J. Antons: Nothing to disclose
Heidrun Hirner-Eppeneder: Nothing to disclose
Sandra Kloiber-Langhorst: Nothing to disclose
Matthias Brendel: Nothing to disclose
Jens Ricke: Nothing to disclose

### Identification of intratumoral clusters in breast cancer xenograft tumors by simultaneous multiparametric [18F]FMISO-PET/MRI

*S. J. Bartsch*[1], J. Friske[1], M. Hacker[1], D. Laimer-Gruber[1], D. Prinz[1], T. Wanek[1], T. H. Helbich[1], K. Pinker-Domenig[2]; [1]Vienna/AT, [2]New York, NY/US

**Purpose or Learning Objective:** Hypoxia is a driver of breast cancer (BC) progression, inducing more aggressive phenotypes and intratumoral neovascularization. The quantification of hypoxia and neovascularization with simultaneous multiparametric [18F]FMISO-PET/MRI would benefit the characterization of the hypoxic tumor microenvironment in BCs. We aim to combine simultaneous multiparametric [18F]FMISO-PET/MRI biomarkers and use them for the identification of intratumoral clusters for a holistic assessment of hypoxia and neovascularization in BCs.
**Methods or Background:** Female athymic nude mice (n = 32) were inoculated with luminal A, HER2+ or triple negative BC cells. PET/MRI was performed on a Bruker 94/30USR system, combined with a Bruker PET-insert. Hypoxia was evaluated using [18F]FMISO-PET, and by hyperoxic blood oxygen level dependent (BOLD) MRI. Neovascularization was assessed via dynamic contrast enhanced MRI, and non-contrast-enhanced intravoxel incoherent motion MRI. Intratumoral clusters were identified based on a hierarchical cluster analysis using R (version 4.2.3).
**Results or Findings:** The cluster analysis of [18F]FMISO-PET/MRI revealed distinct clusters in all BC subtypes. Clusters corresponding to hypoxia showed elevated SUVbw values and the lowest ktrans and ve, indicating dense tissue and limited vessel permeability, along with little change in R2* following hyperoxic BOLD-MRI. The least hypoxic cluster had the lowest SUVbw values and the highest ktrans and ve of all clusters and high change in R2*. Hypoxic clusters were more prevalent in HER2+ and triple negative BCs than in luminal A BCs, while less hypoxic clusters were most common in luminal A BCs.
**Conclusion:** Simultaneous multiparametric [18F]FMISO-PET/MRI provides a holistic perspective on hypoxia and neovascularization in three BC molecular subtypes. These insights enable a non-invasive characterization of BC, and may be used for assessing treatment response.
**Limitations:** The use of intratumoral cluster identification for the assessment of treatment response has to be confirmed in upcoming studies.
**Funding for this study:** This work was funded by the Vienna Science and Technology Fund (WWTF), project number LS19-018.
**Ethics committee - additional information:** This animal study was approved by Austrian Federal Ministry of Education, Science and Research [66.009/0284-WF/V/3b/2017; 2020-0.363.124; 2022-0.726.820] and the Intramural Committee for Animal Experimentation of the Medical University of Vienna.

**Author Disclosures:**
Silvester Julian Bartsch: Nothing to disclose
Daniela Laimer-Gruber: Nothing to disclose
Katja Pinker-Domenig: Nothing to disclose
Thomas H. Helbich: Nothing to disclose
Daniela Prinz: Nothing to disclose
Marcus Hacker: Nothing to disclose
Joachim Friske: Nothing to disclose
Thomas Wanek: Nothing to disclose

**Comparative Radiomics and Feature Consistency: In Vivo CT vs. Ex Vivo Micro-CT in Classifying Lung Cancer Subtypes**

*L. Brizzi*, L. Preda, C. Bortolotto, S. Megalizzi, D. Malerba, F. Checchin; Pavia/IT
*(md.brizzileonardo@gmail.com)*

**Purpose or Learning Objective:** The study aimed to compare radiomic features between conventional in vivo CT and micro-CT of ex vivo lung tumor blocks following lobectomy. This analysis included 60 patients with lung cancer, comprising 30 adenocarcinomas (ADK) and 30 squamous cell carcinomas (SCC). The goal was to assess the variation and significance of features to improve predictive accuracy in distinguishing between tumor types.
**Methods or Background:** The dataset comprised 107 radiomic features, compliant with IBSI standards, extracted using Pyradiomics software. Statistical analyses, including a percentage variation calculation and t-tests, were performed to evaluate the correlation and consistency of features between in vivo CT and ex vivo micro-CT. The analysis focused on features with potential utility in discriminating between ADK and SCC tumors.
**Results or Findings:** Out of the 107 radiomic features, 82 showed less than 10% variation between the two imaging modalities, with 46 features exhibiting a variation of less than 1%. Shape features and approximately 90% of GLCM features demonstrated strong consistency. T-tests revealed that 21 radiomic features had a p-value < 0.05, indicating statistical significance. These features included various shape characteristics and first-order statistics that are crucial for tumor classification.
**Conclusion:** The findings indicate a strong correlation between the radiomic features extracted from in vivo CT and micro-CT, particularly in shape and GLCM features. The identified significant features offer promising potential for improving predictive models for lung cancer classification between ADK and SCC.
**Limitations:** The study was limited to a specific subset of lung tumors (ADK and SCC), and the generalizability to other lung cancer subtypes remains uncertain. Additionally, technical variations in CT acquisition parameters might affect feature extraction consistency. Further validation with larger datasets is necessary.
**Funding for this study:** Founding are provided by Research Foundings on AI of the IRCCS Policlinico San Matteo
**Ethics committee - additional information:** Protocol 25657/2024
**Author Disclosures:**
Leonardo Brizzi: Nothing to disclose
Silvia Megalizzi: Nothing to disclose
Davide Malerba: Nothing to disclose
Filippo Checchin: Nothing to disclose
Lorenzo Preda: Nothing to disclose
Chandra Bortolotto: Nothing to disclose

**12:30-13:30 Research Stage 4**

### Research Presentation Session: Chest

### RPS 904

### Imaging of pulmonary embolism and pulmonary hypertension

**Moderator**
N. J. Screaton; Cambridge/UK
*(n.screaton@nhs.net)*

**Radiomics parameters of epicardial adipose tissue predict mortality in acute pulmonary embolism**

*H-J. Meyer*[1], S. Zimmermann[1], J. Borggrefe[2], A. Surov[2]; [1]Leipzig/DE, [2]Minden/DE

**Purpose or Learning Objective:** Accurate prediction of short-term mortality in acute pulmonary embolism (APE) is very important. The aim of the present study was to analyze the prognostic role of radiomics values of epicardial adipose tissue (EAT) in APE.
**Methods or Background:** Overall, 508 patients were included into the study, 209 female (42.1%), mean age, 64.7 ± 14.8 years. 4.6%and 12.4% died (7- and 30-day mortality, respectively). For external validation, a cohort of 186 patients was further analysed. 20.2% and 27.7% died (7- and 30-day mortality, respectively). CTPA was performed at admission for every patient before any previous treatment on multi-slice CT scanners. A trained radiologist, blinded to patient outcomes, semiautomatically segmented the EAT on a dedicated workstation using ImageJ software. Extraction of radiomic features was applied using the pyradiomics library. Patients were randomly assigned to a training and a validation cohort with a ratio of 7:3. We characterized two models (30-day and 7-day mortality).
**Results or Findings:** We fitted the characterized models to a validation cohort (n = 169) in order to test accuracy of our models. We observed an AUC of 0.776 (CI 0.671-0.881) and an AUC of 0.724 (CI 0.628-0.820) for the prediction of 30-day mortality and 7-day mortality, respectively. The overall percentage of correct prediction in this regard was 88% and 79% in the validation cohorts. Lastly, the AUC in an independent external validation cohort was 0.721 (CI 0.633-0.808) and 0.750 (CI 0.657-0.842), respectively.
**Conclusion:** Radiomics parameters of EAT are strongly associated with mortality in patients with APE.
**Limitations:** It is a retrospective study, which should be evaluated in a prospective multi center analysis.
**Funding for this study:** None
**Ethics committee - additional information:** Nr. 145/21, Ethics Committee, Otto-von-Guericke University of Magdeburg, Magdeburg, Germany)
**Author Disclosures:**
Alexey Surov: Nothing to disclose
Silke Zimmermann: Nothing to disclose
Hans-Jonas Meyer: Nothing to disclose
Jan Borggrefe: Nothing to disclose

**Feasibility Study on the Use of 6ml Iodine Contrast Agent in Pulmonary Artery CT**

*H. Shang*, Y. Gao, D. D. Tian, K. Li, X. Zhang, P. Cao; Xi'an/CN
*(727537956@qq.com)*

**Purpose or Learning Objective:** To assess the viability of using a low dose of iodine contrast medium (CM) and slow injection rate in single-energy 40keV CT imaging for pulmonary artery angiography (CTA).
**Methods or Background:** Seventy patients, clinically suspected of pulmonary embolism and treated between January and September 2024, were randomly assigned to either an experimental group (35 patients) or a control group (35 patients). The experimental group underwent imaging at 100kV with 6ml CM at an injection rate of 2.5ml/s, triggered at a threshold of 60HU. The control group was imaged at 120kV with 35-40ml CM at an injection rate of 3.5ml/s, triggered at 80HU. All scans targeted the pulmonary artery trunk and utilized the non-ionic, water-soluble iodine contrast agent iomeprol (400mg/ml). Both groups were scanned under free breathing conditions. Image quality was subjectively rated on a 5-point scale, and objectively assessed based on pulmonary vascular enhancement, CT values, signal-to-noise ratio (SNR), contrast-to-noise ratio (CNR), and radiation dose metrics including dose-length product (DLP) and volumetric CT dose index (CTDIvol).

**Results or Findings:** The CT values of the main pulmonary artery showed no significant difference between groups (P>0.05). However, the experimental group demonstrated significantly higher CT values in the right upper, middle, interlobar, and lower lobe arteries, as well as the left upper and lower lobe arteries (P < 0.05). Although the experimental group exhibited higher noise, SNR, and CNR, these differences were not statistically significant (P>0.05). The experimental group also had significantly lower CTDIvol and DLP values compared to the control group (P<0.05). Subjective image quality evaluations revealed no significant difference between the two groups (P>0.05).
**Conclusion:** CTA of the pulmonary artery at 40keV with 6ml CM and an injection rate of 2.5ml/s can provide diagnostic image quality.
**Limitations:** Not applicable
**Funding for this study:** Not applicable
**Ethics committee - additional information:** Not applicable
**Author Disclosures:**
Pan Cao: Nothing to disclose
Dong Dong Tian: Nothing to disclose
Xiaoyue Zhang: Nothing to disclose
Kai Li: Nothing to disclose
Haitao Shang: Nothing to disclose
Yanjun Gao: Nothing to disclose

### Double-low protocol CTPA for Pulmonary Embolism detection: Enabling artificial intelligence with deep learning-reconstruction based Images

*L. Shen*, J. Lu, C. Zhou, Z. Bi, X. Ye, M. Zeng, W. Mingliang; Shanghai/CN
*(809797357@qq.com)*

**Purpose or Learning Objective:** To assess the effectiveness of artificial intelligence software (AI) in detecting pulmonary embolism (PE) using low-dose CT pulmonary angiography (CTPA) enhanced by deep learning reconstruction (DLR) and contrast-enhancement boost (CE-boost) technique.
**Methods or Background:** This prospective two-center study included 180 patients who underwent CTPA for suspected PE. Patients were randomly divided into two groups: the routine CTPA group with 50 mL contrast medium (CM) was reconstructed using HIR, and the low-dose CTPA group with 25 mL CM was reconstructed using DLR. The CE-boost was additionally performed to generate DLR-boost images in the low-dose group. Signal-to-noise ratio (SNR) and contrast-to-noise ratio (CNR) of pulmonary arteries were quantitatively assessed. For qualitative image quality assessment, two experienced radiologists independently rated CT images (5, best; 1, worst). A subset of 46 randomly selected patients in each group (1:1 ratio) were evaluated by the AI software (Discover PE, uAI) for the presence of PE. Reference standard was established by expert consensus. The diagnostic accuracy (sensitivity and specificity) of the AI interpretations were compared between methods by bootstrapping.
**Results or Findings:** DLR-boost images produced lower noise, higher SNR and CNR, and superior subjective image quality compared to HIR images in the routine group (p < 0.05). For detecting PE, compared to HIR images, DLR-boost images showed comparable sensitivity (97.67% vs. 93.02%, p < 0.001), and equivalent specificity of (both 100.00%, p > 0.05). The effective dose of the double-low group and the routine group was 1.19 ± 0.45 mSv and 2.69 ± 0.49 mSv, respectively.
**Conclusion:** DLR-boost significantly enhances CTPA image quality at reduced radiation and contrast doses. AI software achieves diagnostic performance comparable to traditional reconstruction methods, supporting its use in clinical practice.
**Limitations:** N/A
**Funding for this study:** This study has received funding by Shanghai Anticancer Association EYAS PROJECT (Grant NO. SACA CY22C15).
**Ethics committee - additional information:** This study was approved by the Ethics Committee of Shanghai Geriatric Medical Center(B2024-009).
**Author Disclosures:**
Xiaodan Ye: Nothing to disclose
Leilei Shen: Nothing to disclose
Zhenghong Bi: Nothing to disclose
Wang Mingliang: Nothing to disclose
Chun Zhou: Nothing to disclose
Mengsu Zeng: Nothing to disclose
Jinjuan Lu: Nothing to disclose

### AI-driven pulmonary vascular analysis with computed tomography in patients with chronic thromboembolic pulmonary disease

*A. Cisarri*, A. Valentini, E. M. Bassi, I. Fiorina, A. D'Onorio De Meo, G. Rodolico, M. Zacchino, K. Ellena, L. Preda; Pavia/IT
*(andrea.ciso94@gmail.com)*

**Purpose or Learning Objective:** Chronic thromboembolic pulmonary disease (CTEPD) is a rare condition in which patients may or may not develop pulmonary hypertension. Although both groups are treated in the same way, through pulmonary thromboendarterectomy (PEA), the pathogenic mechanisms by which some patients develop pulmonary hypertension while others do not have yet to be clarified. The study aims to investigate vascular changes using AI-driven quantification of pre- and post-operative CT scans in patients undergoing PEA.
**Methods or Background:** The study analyzed 35 patients with chronic thromboembolic disease, divided into three groups based on preoperative mean pulmonary arterial pressure (mPAP), from 2017-2022. Pre- and post-operative CT scans of 22 of these patients were analyzed with AI-software to quantify vascular morphology, including vessel number, diameter, and blood volume at various pleural depths. Hemodynamic parameters such as mPAP and pulmonary vascular resistance (PVR) were also assessed.
**Results or Findings:** No significant differences in vascular quantification parameters were observed between patient groups pre-operatively. Post-treatment analysis showed a reduction in the number of small vessels (p=0.0065) and blood volume of small vessels (BV5) at 24 mm depth (p=0.036), with an increase in mean vessel diameter (p=0.0005). A significant correlation was found between BV5 reduction and PVR improvement (p=0.01).
**Conclusion:** CT quantification revealed significant post-operative vascular changes in CTEPD patients, especially in the medium-caliber vasculature. The findings suggest that PVR reduction is primarily linked to the reperfusion of larger vessels, with limited improvement in smaller vessel volumes. Future studies should explore these parameters as potential biomarkers for diagnosis and prognosis in CTEPD.
**Limitations:** The small sample size and limited postoperative follow-up restrict the study's ability to generalize findings. Further prospective studies with larger cohorts are needed.
**Funding for this study:** No funding
**Ethics committee - additional information:** Approved
**Author Disclosures:**
Giuseppe Rodolico: Nothing to disclose
Emilio Maria Bassi: Nothing to disclose
Ilaria Fiorina: Nothing to disclose
Alessandro D'Onorio De Meo: Nothing to disclose
Andrea Cisarri: Nothing to disclose
Michela Zacchino: Nothing to disclose
Lorenzo Preda: Nothing to disclose
Katia Ellena: Nothing to disclose
Adele Valentini: Nothing to disclose

### Automated Quantified CT analysis of Morphological Differences in Chronic Thromboembolic Pulmonary Disease and chronic thromboembolic pulmonary hypertension

*W. Xu*, L. Xi, A. Liu, M. Liu, S. Zhao; Beijing/CN
*(wenqingxu2021@163.com)*

**Purpose or Learning Objective:** We aim to study the new morphological markers of Chronic Thromboembolic Pulmonary Disease (CTEPD) and chronic thromboembolic pulmonary hypertension (CTEPH) on computed tomography pulmonary angiography (CTPA).
**Methods or Background:** We retrospectively enrolled CTEPH, CTEPD patients, and control group from January 2019 to October 2023 in our hospital. The morphological metrics including pulmonary blood volume, tortuosity, and fractal dimension (FD) on CTPA were automatically quantified on an Artificial Intelligence workstation. We compared these metrics among three groups and assessed their correlation with hemodynamics.
**Results or Findings:** A total of 190 participants (97 men, 56.2±10.9 years old) including 116 CTEPH patients ,54 CTEPD patients and 20 control enrolled in this study. The pulmonary artery tortuosity in the control group, CTEPD group, and CTEPH group showed a gradually increased progressively (1.07 [1.06–1.10] vs. 1.10 [1.07–1.14] vs. 1.14 [1.10–1.18], P<0.01). There was a positive correlation between pulmonary artery tortuosity and mean pulmonary artery pressure (r=0.47, P<0.01), pulmonary vascular resistance (r=0.44, P<0.01). Additionally, the volume of small and medium-sized pulmonary arteries was significantly higher in CTEPD patients compared to those with CTEPH (P<0.01). FD among three groups was comparable(p>0.05).
**Conclusion:** Pulmonary arterial tortuosity on CTPA is a crucial imaging biomarker for distinguishing between CTEPH and CTEPD. The preservation of normal volumes in the small and medium-sized pulmonary arteries observed in CTEPD patients implies that this feature could be a key determinant in maintaining normal resting pulmonary artery pressure.
**Limitations:** First, it was a single-center investigation with a small cohort of CTEPD patients and controls, and the non-normal distribution limits the generalizability of our findings. Larger studies with more diverse populations are needed to validate our results. Secondly, there is a need for further refinement in the precision of pulmonary vessel segmentation.
**Funding for this study:** This work was supported by This study is supported by the National Natural Science Foundation of China (82272081), Chinese Academy of Medical Sciences Innovation Fund for Medical Sciences (2021-I2M-1-049, 2022-I2M-C&T-B-109). All authors have nothing to disclose.

**Ethics committee - additional information:** This single-center study was approved by the hospital's Ethics Committee (2022-KY-048) and was performed in accordance with the Declaration of Helsinki.
**Author Disclosures:**
Min Liu: Nothing to disclose
Wenqing Xu: Nothing to disclose
Linfeng Xi: Nothing to disclose
Anqi Liu: Nothing to disclose
Shihua Zhao: Nothing to disclose

**Comparison of dual energy CT and V/Q SPECT in diagnosis of chronic thromboembolic pulmonary hypertension**
E. Pershina, D. Shchekochikhin, *A. Oganesyan*; Moscow/RU
*(talilen@mail.ru)*

**Purpose or Learning Objective:** To assess diagnostic value of DECT versus V/Q SPECT for CTEPH detection in PAH patients in Moscow Pulmonary Hypertension Center.
**Methods or Background:** DECT with the calculation of iodine maps and V/Q SPECT were performed in 29 patients (f/m - 9/13; age 65 ±10) with PAH and risk factors for CTEPH. All patients underwent right heart catheterization for PAH confirmation. CTA analysis included the number and level of vessel occlusions together with the presence and size of lung perfusion defects on iodine maps and CT signs of right heart failure.
**Results or Findings:** 21 of 29 patients (72,4%) demonstrated CTA features of possible CTEPH such as intraluminal defects, enlarged pulmonary trunk, pulmonary mosaic patten. Iodine maps revealed perfusion defects of 19 patients (86%). Two patients (7%) had intraluminal irregular defects without perfusion defects by DECT. V/Q SPECT determined perfusion defects in 20 patients (69%). There was one case (3, 4%) with mismatch between iodine maps and V/Q SPECT. However, it was explained by the enlarged pressure on the right heart chambers due to the cava-caval (cava superior – cava inferior) venous anastomosis and hepatic veins abnormalities. Sensitivity for perfusion defects by DECT was 95% and specificity 100%. Furthermore, the mean radiation dose was significantly lower for DECT vs. V/Q-SPECT ($p = 0.006$). Spreading of perfusion defect had strong correlation with severity of right heart failure ($r=0.2$, $p<0.05$).
**Conclusion:** Pulmonary CTA with iodine mapping improves the detection of CTEPH. Iodine maps demonstrate high sensitivity and specificity in comparison with V/Q SPECT. According to our preliminary data DECT could be potentially implemented in future diagnostic PH algorithms at least on par with V/Q-SPECT. Further research is needed.
**Limitations:** Pregnant
Older 18 years
GFR>30 ml/min
Iodine allergic reaction
**Funding for this study:** No funding
**Ethics committee - additional information:** The study was approved by local ethical committee.
**Author Disclosures:**
Ekaterina Pershina: Nothing to disclose
Anait Oganesyan: Other: Analysis datas Author: Has wrote study Speaker: presents study
Dmitry Shchekochikhin: Nothing to disclose

**Development of a CT based prognostic predictive model in chronic thromboembolic pulmonary hypertension (CTEPH)**
*S. Gowda*, R. Kothari, V. Raj; Bengaluru/IN
*(srjgwd@gmail.com)*

**Purpose or Learning Objective:** Chronic thromboembolic pulmonary hypertension (CTEPH) is a severe complication of pulmonary embolism characterized by persistent pulmonary arterial hypertension due to unresolved thromboembolic obstructions. This study explores the development and validation of a CT based prognostic predictive model designed to simulate hemodynamic parameters and guide management in CTEPH patients.
**Methods or Background:** We retrospectively analysed the data of 1100 patients diagnosed with CTEPH. Integrated data consisted of clinical history, echocardiogram reports, right heart catheterisation reports if any, CT pulmonary angiogram studies and proposed management strategies. CT clot burden score, CT parameter score and Perfusion defect score was obtained in all the patients. Patients were contacted to review the clinical outcomes.
**Results or Findings:** Parameter scoring was seen to be useful in predicting hemodynamic parameters and outcomes. A cut-off of 3 is close to normal hemodynamic status (sPAP, accuracy of 76%), while a cut-off of 5 represents higher than normal values of mPAP. A score of ≥7 indicated increased risk of mortality.
**Conclusion:** The CT based prognostic predictive model represents a valuable tool in the management of CTEPH by integrating detailed imaging with clinical factors. We suggest this model can be a standard component of CTEPH patient evaluation and management especially when right heart catheterisation is contraindicated or not possible. Future studies should focus on prospective validation and integration into clinical practice to further refine its utility and impact in the management. It can be used to avoid right heart catheterisation and ventilation-perfusion scans in selected patients who meet the cut-off values, thereby reducing additional cost burden to the patients.
**Limitations:** Single centre study, which may also have an in-built case selection bias. Small sample size to ascertain likelihood of mortality
Needs prospective validation to integrate into clinical practice
**Funding for this study:** No funding was provided for this study.
**Ethics committee - additional information:** The study received institutional review board approval.
**Author Disclosures:**
Richa Kothari: Nothing to disclose
Vimal Raj: Nothing to disclose
Suraj Gowda: Nothing to disclose

**Performance of chest contrast-enhanced CT in pulmonary hypertension clinical grouping: a dual-center, expert-blinded analysis**
*L. Nardone*[1], L. Cereser[1], G. Agati[1], P. Ciolli[2], T. Nadarević[3], C. Cicciò[4], A. Borghesi[2], R. Girometti[1], C. Zuiani[1]; [1]Udine/IT, [2]Brescia/IT, [3]Rijeka/HR, [4]Negrar/IT
*(luiginardone1996@gmail.com)*

**Purpose or Learning Objective:** This study aimed to evaluate the diagnostic accuracy of contrast-enhanced CT imaging in classifying patients with pulmonary hypertension (PH) across international guidelines-derived clinical groups I-V. The analysis focused on quantifying the utility of CT in a blinded setting, comparing the results with those from the PH multidisciplinary team meeting (PH-MDTM).
**Methods or Background:** We retrospectively included 172 contrast-enhanced CT studies from patients with PH performed in two tertiary referral centers. Three chest-devoted radiologists, blinded to the clinical data, independently reviewed all the CTs, assigning probability percentages for each of the five PH groups. A consensus grouping hypothesis was reached by averaging the probabilities across readers, and this was compared with the PH-MDTM grouping. Accuracy and Cohen's Kappa (k)-derived inter-reader agreement values with 95% confidence intervals (95% CI) were calculated. The readers' discriminatory power between individual PH groups was evaluated through areas under the receiver operating characteristic curve (AUC) analyses.
**Results or Findings:** The expert-blinded consensus diagnosis agreed with the PH-MDTM in 124/172 cases (accuracy, 72%; k, 0.62; 95%CI, 0.50-0.70). When including the second most probable group, the readers correctly grouped 148/172 cases (accuracy, 86%; k, 0.81; 95%CI, 0.74-0.88). Discriminatory power analysis for individual groups demonstrated good readers' performance, with AUC values ranging from 0.79-0.90 depending on the group.
**Conclusion:** This study highlights the value of contrast-enhanced CT in classifying PH according to clinical groups, with expert readers achieving substantial-to-almost perfect agreement with the PH-MDTM grouping and good discriminatory power for individual PH groups. The results suggest that CT imaging can serve as a reliable tool in the clinical work-up of PH patients, supporting its integration into multidisciplinary evaluations.
**Limitations:** Retrospective design, low number of group V PH cases.
**Funding for this study:** No funding was received for this study.
**Ethics committee - additional information:** The ethics committee notification can be found under the number IRB 250/2023.
**Author Disclosures:**
Andrea Borghesi: Nothing to disclose
Chiara Zuiani: Nothing to disclose
Rossano Girometti: Nothing to disclose
Giorgio Agati: Nothing to disclose
Pietro Ciolli: Nothing to disclose
Tin Nadarević: Nothing to disclose
Lorenzo Cereser: Nothing to disclose
Luigi Nardone: Nothing to disclose
Carmelo Cicciò: Nothing to disclose

**14:00-15:30** **Research Stage 1**

### Research Presentation Session: Genitourinary

### RPS 1007

### Imaging of renal and bladder malignancies: advances in diagnosis and characterisation

**Moderator**
N. Cowan; Portsmouth/UK

**Enhanced U-Net for Precise Auto-Segmentation of Bladders and Tumors in CT Urography Imaging**
*L. Chen*, L. Mao, X. Li, X. Zhang, X. Bai, G. Zhang, H. Xue, Z. Jin, H. Sun; Beijing/CN
*(li.cherish@outlook.com)*

**Purpose or Learning Objective:** This study developed a deep learning model for bladder and tumor segmentation from CT Urography images (CTU), integral to a system aiding bladder cancer management.
**Methods or Background:** A 381-case dataset from two centers, approved by the Institutional Review Board, was utilized. It comprised 280 training cases (275 with masses, 5 normal), 56 internal validation cases (54 with masses, 2 normal), and 45 external validation cases (44 with masses, 1 normal). The set included nephrographic phase CTU scans with 0.625mm and 1mm slice thicknesses for patients with pathologically confirmed lesions. A reference standard with manual contours was provided by an experienced radiologist and reviewed by a senior one. The nnU-Net framework trained a U-Net-based segmentation model using an ensemble prediction in a five-fold cross-validation and test-time augmentation. Performance was assessed using DSC, 95% HD, and MSD on the testing set.
**Results or Findings:** Results show that our approach achieves superb segmentation accuracy. In the internal validation set, the U-Net-based model showed strong performance with a DSC of 97.9%, 95% HD of 0.48mm, and MSD of 3.43mm for bladder segmentation. It excelled in the external set with a DSC of 98.4%, 95% HD of 0.34mm, and MSD of 2.52mm. For tumor segmentation, the internal set results were a DSC of 76.6%, 95% HD of 3.70mm, and MSD of 19.15mm, while the external set showed a slight decrease to a DSC of 74.4%, 95% HD of 3.73mm, and MSD of 22.08mm.
**Conclusion:** Though the tumor segmentation was less precise than bladder segmentation, the U-Net-based model still provided satisfactory accuracy for both, excelling in bladder delineation. This model proves valuable for detecting bladder cancer and evaluating treatment efficacy.
**Limitations:** The limited sample size of the external validation cohort limited the generalizability.
**Funding for this study:** This work was supported by National High-Level Hospital Clinical Research Funding(2022-PUMCH-A-035), National High-Level Hospital Clinical Research Funding(2022-PUMCH-B-069), National High-Level Hospital Clinical Research Funding(2022-PUMCH-A-033), the Natural Science Foundation of China (Grant No.81901742), the Beijing natural Science Foundation (Grant No. L232133), and the CAMS Innovation Fund for Medical Sciences (2022-12M-C&T-B-019).
**Ethics committee - additional information:** The Pecking Union College Hospital Institutional Review Board approval was obtained (ethical approval number: I-22PJ887).
**Author Disclosures:**
Xiuli Li: Nothing to disclose
Li Mao: Nothing to disclose
Gumuyang Zhang: Nothing to disclose
Hao Sun: Nothing to disclose
Xin Bai: Nothing to disclose
Zhengyu Jin: Nothing to disclose
Li Chen: Nothing to disclose
Xiaoxiao Zhang: Nothing to disclose
Huadan Xue: Nothing to disclose

**Radiogenomics of renal cell carcinoma: using MRI tumor features to predict mir-15a expression**
*Y. Mytsyk*[1], P. Kowal[2], Y. Kobilnyk[3], I. Dutka[1], I. Komnatska[1], A. Górecki[4]; [1]Lviv/UA, [2]Wroclaw/PL, [3]Przemyśl/PL, [4]Przeworsk/PL
*(mytsyk.yulian@gmail.com)*

**Purpose or Learning Objective:** The aim of this study was to evaluate the effectiveness of MRI tumor parameters in predicting tissue expression of miR-15a in renal cell carcinoma (RCC) patients.
**Methods or Background:** The study involved 64 patients with histologically confirmed conventional RCC, where miR-15a expression was measured, and preoperative contrast-enhanced MRI (1.5 T) was performed. MiR-15a expression was determined using reverse transcription and real-time PCR. A polynomial regression model assessed associations between miR-15a expression and radiological tumor parameters, with accuracy evaluated by the Fisher method (adjusted $R^2$).
**Results or Findings:** It was found that radiological features of the cystic component, exophytic growth, necrosis, macroscopic fat, and nodular contrast enhancement of the tumor were observed in 29.69%, 23.44%, 32.81%, 20.31%, and 37.5% of patients, respectively. The mean levels of miR-15a expression in the presence of these features were 0.35±1.02 U, 0.34±1.09 U, 4.01±3.42 U, 0.29±0.87 U, and 2.91±3.24 U, respectively. In the absence of these features, the mean expression of miR-15a was 2.01±2.93 U, 1.88±2.85 U, 0.82±1.85 U, 1.83±2.83 U, and 0.68±1.72 U, respectively ($p<0.05$). The highest miR-15a expression levels were observed with necrosis, and the lowest with macroscopic fat ($p<0.05$). Tumor size strongly correlated with miR-15a expression ($r=0.724$; $p<0.001$). Tumor size alone predicted miR-15a expression with an adjusted $R^2$ of 0.8281, and combining tumor size with other radiological features predicted 85% of miR-15a expression ($R^2=0.8336$; $p<0.001$). The study developed a predictive formula for miR-15a expression based on RCC radiological features.
**Conclusion:** The findings suggest that MRI parameters can accurately predict miR-15a expression, which holds diagnostic and prognostic value in RCC.
**Limitations:** The main limitation was the inclusion of only conventional RCC.
**Funding for this study:** No funding.
**Ethics committee - additional information:** The study was approved by the Ethics Committee of the Danylo Halytsky Lviv National Medical University (protocol No. 5 dated May 25, 2021). The work was conducted in accordance with accepted standards for conducting research in the field of biology and medicine, based on the guidelines of the World Health Organization, the International Council of Medical Scientific Societies, the International Code of Medical Ethics (1983), the Helsinki Declaration adopted by the General Assembly of the World Medical Association, the Convention on Human Rights and Biomedicine of the Council of Europe (1997), and the requirements and standards of ICH GCP (2002). In each specific case, patients or responsible individuals provided written consent for the surgical intervention.
**Author Disclosures:**
Yulian Mytsyk: Nothing to disclose
Iryna Komnatska: Nothing to disclose
Yuriy Kobilnyk: Nothing to disclose
Andrzej Górecki: Nothing to disclose
Paweł Kowal: Nothing to disclose
Ihor Dutka: Nothing to disclose

**An Artificial Intelligence Framework Based On Contrast-Enhanced CT For Preoperative Predicting WHO/ISUP Nuclear Grade Of Clear Cell Renal Cell Carcinoma: A Multicenter Study**
*J. Han*[1], T. Liu[2], J. Li[1], Y. Zhang[3]; [1]Hohhot/CN, [2]Guangzhou/CN, [3]Zhuhai/CN
*(handaile@163.com)*

**Purpose or Learning Objective:** To determine whether the artificial intelligence integrated model based on automatic segmentation of CT images can provide a robust prediction of clear cell renal cell carcinoma (ccRCC) ISUP/WHO grade.
**Methods or Background:** Pretreatment CT scans were retrospectively acquired in patients with surgically proven ccRCC at multiple centers from January 2017 to September 2023.The proposed framework comprised five modules, including a 3D tumor segmentation model by 3D-UNet, a deep learning feature extraction module, a radiomic feature extraction module, a clinical-radiological feature screening module, and a fully-connected classification module that combines features from different sources to classify low-grade (I and II) and high-grade (III and IV) ccRCC. The Grad-CAM method and SHAP method are used to analyze the interpretability of the artificial intelligence model.

**Results or Findings:** The training data set was comprised of 335 patients from three centers, and 110 and 84 patients were included in the two external test data sets. The average Dice coefficient of the 3D-UNet automatic segmentation network in the test sets was 0.86 and 0.82. Synchronous distant metastasis, Planned nephrectomy type, and tumor long axis as independent predictors of high-grade ccRCC. In the test sets, the AUC and accuracy of integrated model were 0.85-0.92, 78-85%, respectively, which were exceeded those of clinical-radiological feature model (0.85 vs0.75 [P = 0.039], 0.93 vs 0.76 [P = 0.043], 78% vs 65% [P < 0.001], 85% vs 71% [P = 0.035].
**Conclusion:** An integrated model based on clinical features, radiological features, radiomics features, and deep learning features provided reliable prediction of WHO/ISUP grade for ccRCC, which outperformed the clinical-radiological feature model.
**Limitations:** This is a retrospective study and we only included ccRCC with a pathological diagnosis of WHO/ISUP grading after nephrectomy , with some selection bias.
**Funding for this study:** This work was supported in part by the National Natural Science Foundation of China under Grant Nos. 81801809, 82371917, 81830052, 81971691, 12126610, 62371476; in part by the Basic and Applied Basic Research Foundation of Guangdong Province under Grant Nos. 2020A1515010572, and in part by the Zhuhai Basic and Applied Basic Research Foundation under Grant Nos. ZH22017003200001PWC.
**Ethics committee - additional information:** This study was approved by the institutional review boards of the Fifth Hospital of Sun Yat- sen University.
**Author Disclosures:**
Tao Liu: Nothing to disclose
Yaqin Zhang: Nothing to disclose
Junlin Li: Nothing to disclose
Jiayue Han: Nothing to disclose

**Prevalence of Venous Extension in Malignant Adrenal Neoplasia: Identification of a Novel Imaging Sign**
L. Melges, C. Torres, F. Chahud, D. F. Maia, L. Colli, J. Elias, C. A. Fernandes Molina, M. Castro, *V. F. Muglia*; Ribeirao Preto/BR
*(fmuglia@fmrp.usp.br)*

**Purpose or Learning Objective:** Adrenal vein involvement is a common feature of adrenocortical carcinomas, but its prevalence in metastatic adrenal lesions remains unknown. Our goal was to assess the prevalence of adrenal vein involvement in primary and metastatic adrenal lesions and to determine if morphological changes in tumor shape precede venous extension.
**Methods or Background:** This retrospective, single-center observational study evaluated 102 patients: 28 adrenal cortical carcinoma (ACC) patients; and 74 non-ACC cancer patients that presented adrenal metastasis (82 metastatic adrenal lesions). Two readers reviewed cross-sectional imaging to assess tumor size, laterality, venous invasion, and the presence of the "edge sign." Surgical and histopathological confirmation was the reference standard for ACCs, while for metastases, sequential imaging or PET-CT results showing hypermetabolism were used when histopathology was unavailable.
**Results or Findings:** Of the 28 ACC patients, 82.1% were female, with balanced laterality. Metastases primarily originated from the lung (24.4%), colorectal (13.4%), and breast (12.2%) cancers and had a left-side dominance (61.7%). Venous extension was present in 14.6% of metastases and 21.4% of ACCs. The "edge sign" was more frequently observed in metastatic lesions (26.8%) than in ACCs (17.8%). Interobserver agreement was almost perfect for venous extension ($\kappa = 0.9256$) and substantial for the edge sign ($\kappa = 0.7844$).
**Conclusion:** Venous extension was less prevalent in metastatic adrenal lesions compared to ACCs. The edge sign may precede venous extension, especially in metastatic cases. Although these findings could impact clinical evaluation, prospective multicenter studies are needed to confirm the clinical significance of the edge sign.
**Limitations:** Retrospective, single-center study.
**Funding for this study:** FAEPA - Foundation for the development of learning, assistance and research of Ribeirao Preto School of Medicine Hospital.
**Ethics committee - additional information:** The study was approved by the Ethics and Research Committee of the Clinical Hospital at Ribeirao Preto Medical School under number CAAE 78221024.2.0000.5440, with a waiver from the Informed Consent Form
**Author Disclosures:**
Jorge Elias: Nothing to disclose
Margaret Castro: Nothing to disclose
David Freire Maia: Nothing to disclose
Fernando Chahud: Nothing to disclose
Lais Melges: Nothing to disclose
Carlos Augusto Fernandes Molina: Nothing to disclose
Leandro Colli: Grant Recipient: Bristol Myers Squibb - Immunotherapy for Renal Cell Carcinnoma
Valdair Francisco Muglia: Nothing to disclose
Cecilia Torres: Nothing to disclose

**IRM K01 study: Diagnostic Value of Multiparametric MRI for Small Solid Renal Tumors**
*E. Jambon*[1], N. Grenier[1], C. Marcelin[1], A. Crombé[1], G. Margue[1], J-C. Bernhard[1], F. H. Cornelis[2]; [1]Bordeaux/FR, [2]New York, NY/US
*(eva.fourage@gmail.com)*

**Purpose or Learning Objective:** Small renal tumors are increasingly being detected incidentally, posing diagnostic challenges. Up to 23% of small renal tumors result in non-contributive biopsies. This study evaluates the diagnostic value of multiparametric MRI (mpMRI) in the characterization of small solid renal tumors. The aim was to assess the diagnostic accuracy of mpMRI in differentiating malignant from benign small solid renal tumors in patients with suspected malignancy but no evident signs of metastasis.
**Methods or Background:** This is a prospective multicentric French study. A cohort of 387 patients in 17 centers with non-hereditary, solid renal masses between 1.5 and 4 cm in diameter was enrolled between November 2018 and May 2022. MRI protocols included T1w, T2w, diffusion-weighted imaging, and dynamic contrast-enhanced sequences. Radiologists performed blinded readings with a centralized review in case of discordance. The primary endpoint is the negative predictive value (NPV) of a dichotomized Likert scale score, targeting a 98% NPV.
**Results or Findings:** The study found a 45% NPV for mpMRI, falling short of the expected 98% due to difficulties in distinguishing clear cell renal cell carcinoma (ccRCC) from oncocytomas, which constituted 80% of the benign tumors in the cohort. Despite this, mpMRI influenced clinical management decisions, increasing "surveillance without biopsy" by 25% and reducing biopsies by 42%. However, it also led to a 25% increase in partial/total nephrectomies.
**Conclusion:** While mpMRI showed limited ability to accurately distinguish certain benign lesions from malignant tumors under predefined criteria, it proved effective in identifying malignant cases. This led to a shift in clinical management favoring surgical interventions over biopsies. The study highlights the need for more objective imaging criteria and further research into quantitative measures and radiomic analysis for better tumor characterization.
**Limitations:** Predefined criteria
**Funding for this study:** No funding
**Ethics committee - additional information:** PHRC-K
Approval from the French Ethics Committee (CPP)
**Author Disclosures:**
Jean-Christophe Bernhard: Nothing to disclose
Gaelle Margue: Nothing to disclose
François H Cornelis: Nothing to disclose
Eva Jambon: Nothing to disclose
Amandine Crombé: Nothing to disclose
Clément Marcelin: Nothing to disclose
Nicolas Grenier: Nothing to disclose

**ADC measurement may improve the diagnostic performance of bi-parametric bladder MRI in predicting detrusor muscle invasion of bladder cancer**
*M. N. Tasdemir*, U. Eryürük, S. Aslan; Giresun/TR
*(mervetsdmr@gmail.com)*

**Purpose or Learning Objective:** To assess the diagnostic performance of a modified biparametric VIRADS (mbp-VIRADS), derived from a combination of ADC measurements and biparametric MRI (bp-MRI), in predicting detrusor muscle invasion in bladder cancer (BC).
**Methods or Background:** Patients with histopathologically confirmed BC between June 2020 and May 2024 were analyzed retrospectively. Two image sets, biparametric MRI (set 1) and multiparametric (mp) MRI (set 2), were formed. Tumors were categorized using both the bp-VIRADS and mp-VIRADS systems. The optimal ADC value to differentiate muscle-invasive bladder cancer (MIBC) from non-muscle-invasive bladder cancer (NMIBC) was determined using a receiver operating characteristic (ROC) curve. To assess the mbp-VIRADS category, for cases with a bp-VIRADS score of 2-4, scores were upgraded for those below the ADC cut-off value and downgraded for those above it.
**Results or Findings:** A total of 182 patients with BC met the study criteria. Of these patients, 146 had NMIBC and 36 had MIBC. Comparing VIRADS categories with MIBC detection, AUC of the ROC analysis was 0.896, 0.940, and 0.941 for the bp-MRI, mbp-MRI, and mp-MRI protocols, respectively. The sensitivity for bp-VIRADS, mbp-VIRADS, and mp-VIRADS scores (with a cutoff ≥4) were 78%, 88%, and 90%, specificity were 91%, 95%, and 93%; and overall accuracy were 88%, 92%, and 93%, respectively. Using bp-MRI, there were 12 false-positive and 9 false-negative cases for predicting muscle invasion. With mp-MRI, false positives decreased to 9, and false negatives to 4. When using mbp-MRI, false positives further decreased to 6, with 5 false negatives.

**Conclusion:** By combining ADC measurements with bp- MRI features, the diagnostic performance of bp-MRI in predicting muscle invasion of bladder cancer can be significantly improved, approaching that of mp- MRI, while reducing the false-positive and false-negative rates.
**Limitations:** This was a retrospective study,
**Funding for this study:** None
**Ethics committee - additional information:** The study was approved by the local ethics commitee.
**Author Disclosures:**
Uluhan Eryürük: Nothing to disclose
Merve Nur Tasdemir: Nothing to disclose
Serdar Aslan: Nothing to disclose

**Evaluating VI-RADS Score Performance in the Post-TURBT setting: Exploring the Need for Modification**

*A. Dehghanpour*, M. Pecoraro, L. Laschena, M. Bicchetti, C. Catalano, V. Panebianco; Rome/IT
*(ad48ad@yahoo.com)*

**Purpose or Learning Objective:** The aim of this study was to assess the diagnostic accuracy of the VI-RADS score and its individual MRI categories, structural category (T2W), diffusion category (DWI), and contrast-enhanced category (DCE), in patients who underwent diagnostic transurethral resection of bladder tumor (TURBT). Additionally, we correlated the diagnostic accuracy of VI-RADS and its individual sequences with the time interval between TURBT and MRI, to suggest the optimal timing for MRI after TURBT. We also calculated the inter-reader agreement in scoring VI-RADS after TURBT.
**Methods or Background:** This retrospective single-center study included 150 patients who underwent mpMRI after TURBT at varying intervals. Four experienced readers in bladder MRI, independently and blinded to clinicopathological information, evaluated the scans, providing both VI-RADS scores and local staging. Each evaluation was performed twice: once with DWI as the dominant sequence and once with DCE as the dominant sequence. The only exclusion criterion was prior systemic therapy. Histopathological results from therapeutic TURBT or radical cystectomy were used as the reference standard.
**Results or Findings:** The AUC for VI-RADS in detecting muscle-invasive bladder cancer was 0.88 (95% CI 0.84-0.92) for the most experienced reader. On a per-sequence analysis, DWI showed the highest AUC (0.83 [95% CI 0.78-0.87]), followed by DCE (0.68 [95% CI 0.63-0.74]). The diagnostic accuracy of VI-RADS improved when the time between TURBT and MRI exceeded 2 weeks and became optimal after 4 weeks, regardless of whether DWI or DCE was the dominant sequence.
**Conclusion:** The earliest acceptable timing for MRI after TURBT is at least 2 weeks, with the optimal timing being after 4 weeks. In scoring VI-RADS after TURBT, DWI should be considered the dominant sequence, due to its high sensitivity and specificity.
**Limitations:** Retrospective design and readers being from the same center.
**Funding for this study:** None.
**Ethics committee - additional information:** Institutional Ethical Committee.
**Author Disclosures:**
Marco Bicchetti: Nothing to disclose
Valeria Panebianco: Nothing to disclose
Ailin Dehghanpour: Nothing to disclose
Martina Pecoraro: Nothing to disclose
Ludovica Laschena: Nothing to disclose
Carlo Catalano: Nothing to disclose

**Uncertainty-Aware Interactive Deep Learning System for Predicting Pathological T3a Upstaging in Renal Cell Carcinoma Using CT Images**

*E. Yuan*[1], Q. Zhou[2], Y. Chen[1], C. He[1], L. Ye[1], J. Yao[1], B. Song[1]; [1]Chengdu/CN, [2]Shanghai/CN

**Purpose or Learning Objective:** To develop and validate a deep learning system for predicting pathological T3a upstaging in renal cell carcinoma while integrating prediction uncertainty to facilitate more reliable clinical decision-making.
**Methods or Background:** In this retrospective study, we collected pathology-confirmed RCC patients who underwent surgical resection from three tertiary academic medical centers. The data in first center were split into training and testing datasets. Three DenseNet-121 models were trained to predict the overall T3a invasion, the inner invasion, and the outer invasion. The uncertainty was quantified by ensemble-based and voting-based methods. For uncertain cases, manual interpretation was performed to obtain the final prediction. The performance of the pure model and uncertainty-aware interactive system were evaluated and compared on the testing dataset and two external datasets using area under the ROC curve (AUC).
**Results or Findings:** The data of 1329 patients (975:235:119) were collected and analyzed. The DL system performed worse in the uncertain group compared to the certain group of testing dataset (AUC 0.73 (95% CI: 0.61, 0.85) vs 0.81 (95% CI: 0.71, 0.90)), the external_1 dataset (AUC 0.50 (95% CI: 0.33, 0.68) vs 0.95 (95% CI: 0.90, 0.99)), and the external_2 dataset (AUC 0.82 (95% CI: 0.62, 1.00) vs 0.94 (95% CI: 0.89, 0.99)). The net reclassification index for the DL system were 0.11, 0.18, and 0.13 in testing, external_1, and external_2 datasets.
**Conclusion:** The uncertainty-aware interactive deep learning system effectively predicts pathological T3a upstaging in renal cell carcinoma, with manual interpretation improving performance in uncertain cases. This approach enhances diagnostic reliability, demonstrating potential for improved clinical decision-making across multiple datasets.
**Limitations:** The sample size in external validation datasets were limited.
**Funding for this study:** None
**Ethics committee - additional information:** The written consent was waived for the retrospective design.
**Author Disclosures:**
Jin Yao: Nothing to disclose
Qing Zhou: Nothing to disclose
Chunlei He: Nothing to disclose
Lei Ye: Nothing to disclose
Enyu Yuan: Nothing to disclose
Bin Song: Nothing to disclose
Yuntian Chen: Nothing to disclose

**Enhancing Diagnostic Accuracy in Renal Tumor Identification: Impact of Structured Training on the Clear Cell Likelihood Score**

*M. Cosenza*, G. Brembilla, G. Imperiale, A. Larcher, U. Capitanio, F. Montorsi, F. De Cobelli; Milan/IT
*(Cosenza.michele@hsr.it)*

**Purpose or Learning Objective:** This study aimed to evaluate the improvement in diagnostic performance of radiologists in identifying clear cell and papillary renal tumors using the Clear Cell Likelihood Score (CCLS) before and after a structured training program.
**Methods or Background:** This monocentric study analyzed 60 MRI scans, including 28 cases of clear cell carcinoma and 16 cases of papillary carcinoma, all confirmed by histopathological examination. Five radiologists evaluated the scans twice: the first assessment was based solely on their prior knowledge, using a cutoff of ≥4 for likelihood of clear cell carcinoma; the second assessment followed a training session that introduced the CCLS, where a score of ≥4 was assigned for clear cell tumors and ≤1 for papillary tumors. A washout period of 4 weeks was implemented between assessments.
**Results or Findings:** Clear cell carcinoma overall sensitivity improved from 56% (95%CI: 47-64) pre-training to 86% (95%CI: 79-91) post-training, with a corresponding AUC for the ROC curve enhancing from 0.81 (95%CI: 0.76-0.86) to 0.85 (95%CI:0.81-0.90). Papillary carcinoma sensitivity increased from 52% (95%CI:41-64) pre-training to 65% (95% CI: 54-75) post-training, with specificity rising from 90% (95%CI:85-93) to 95% (95%CI:91-97). The AUC for the ROC curve for papillary carcinoma rose significantly from 0.79 (95%CI:73-85) to 0.89 (95%CI: 85-94). Additionally, the agreement improved for clear cell tumors, with a K of Conger increasing from 0.293 (95%CI: 0.181-0.406) to 0.594 (95%CI:0.469-0.718), and for papillary tumors, from 0.360 (95%CI:0.211-0.51) to 0.489 (95%CI:0.35-0.628).
**Conclusion:** Structured training and the application of the CCLS significantly enhance the diagnostic accuracy of radiologists in identifying clear cell and papillary renal tumors on MRI, underscoring the importance of targeted education in improving radiological interpretations.
**Limitations:** Limitations include monocentric design, small sample size, limited radiologist cohort, and no longitudinal follow-up, impacting generalizability and sustainability.
**Funding for this study:** None
**Ethics committee - additional information:** IRB approved
**Author Disclosures:**
Francesco Montorsi: Nothing to disclose
Michele Cosenza: Nothing to disclose
Giulio Imperiale: Nothing to disclose
Alessandro Larcher: Nothing to disclose
Francesco De Cobelli: Nothing to disclose
Giorgio Brembilla: Nothing to disclose
Umberto Capitanio: Nothing to disclose

**Differentiating solid from friable tumor thrombus in renal cell carcinoma using MRI ADC volumetric analysis**

P. Kowal[1], *Y. Mytsyk*[2], K. Ratajczyk[1], W. Bursiewicz[1], M. Trzciniecki[1], K. Marek-Bukowiec[1], J. Rogala[1]; [1]Wrocław/PL, [2]Lviv/UA
*(mytsyk.yulian@gmail.com)*

**Purpose or Learning Objective:** This study aimed to evaluate the utility of first-order radiomic features derived from MRI apparent diffusion coefficient (ADC) maps using volumetric analysis in distinguishing solid from friable thrombus in patients with renal cell carcinoma (RCC).

**Methods or Background:** A cohort of 27 patients with conventional histologic subtype of RCC and tumor thrombus in the renal vein or inferior vena cava (IVC) was included. All patients underwent surgical intervention, comprising nephrectomy and thrombectomy, and received preoperative abdominal MRI with diffusion-weighted imaging sequences at b-values of 50, 200, 800 s/mm². The ADC map was used for volumetric analysis, calculating various radiomic first-order features across the thrombus volume, including ADC mean, median, range, 10th percentile, 90th percentile, interquartile range, entropy, kurtosis, skewness, uniformity, and variance. Tumor thrombi were histologically classified as solid or friable, and associations between the radiomic features and thrombus consistency were analyzed.
**Results or Findings:** Solid and friable tumor thrombi were identified in 51.9% and 48.1% of patients, respectively. Inverse association noted between RCC thrombus cellularity and skewness (r=-0.799, p<0.001). No significant differences were observed in the mean values of range, 90th percentile, interquartile range, kurtosis, uniformity, and variance between groups. For distinguishing solid from friable thrombus, the ADC mean, median, and entropy showed equal sensitivity (93%) and specificity (69%), with entropy yielding the highest area under the curve (AUC) at 0.808. Skewness demonstrated a sensitivity of 86% and specificity of 92%, with an AUC of 0.931.
**Conclusion:** In RCC patients with tumor thrombus in the renal vein or IVC, volumetric analysis of first-order radiomic features using ADC mapping facilitates accurate differentiation between solid and friable thrombus variants.
**Limitations:** The primary limitation of this study is that only conventional histologic subtype of RCC was included in the analysis.
**Funding for this study:** No funding.
**Ethics committee - additional information:** This study was approved by the Local Bioethical Committee in the Research and Development Center, Regional Specialist Hospital in Wroclaw (no. KB/12/2021). All procedures conducted followed the ethical guidelines set by the institutional and/or national research committee, adhering to the principles outlined in the 1964 Helsinki Declaration and its subsequent revisions, or equivalent ethical standards. The written informed consent for enrolment in the study was signed by all patients.
**Author Disclosures:**
Yulian Mytsyk: Nothing to disclose
Krzysztof Ratajczyk: Nothing to disclose
Maciej Trzciniecki: Nothing to disclose
Wiktor Bursiewicz: Nothing to disclose
Joanna Rogala: Nothing to disclose
Paweł Kowal: Nothing to disclose
Karolina Marek-Bukowiec: Nothing to disclose

**Does the Bosniak 2019 classification really provide an objective assessment among the radiologists**
Ş. Evrimler, *E. Cigdem Karatayli*; Ankara/TR
*(elifcigdem95@hotmail.com)*

**Purpose or Learning Objective:** The Bosniak Classification system was revised in 2019 to reduce subjectivity and prevent unnecessary nephron loss. In this plot study, we aimed to evaluate the inter-reader agreement in Bosniak classification among radiologists with different experience levels.
**Methods or Background:** Out of 320 patients imaged between 01.2022 and 04.2024 in our hospital, 12 patients were randomly selected among those with pathology results, 22 patients were randomly selected among those without pathology results, a total of 34 patients were selected. Eight residents, two radiology specialists, and one abdominal radiologist evaluated abdominal CT and MRI scans of these patients. All participants were trained on the 2019 Bosniak classification prior to the assessment. They assessed each criteria (septation presence, nodularity characteristics, contrast enhancement,etc.) separately and ultimately identified the Bosniak type. The abdominal radiologist's classifications served as the reference for Kappa analysis. Participants also completed a questionnaire regarding difficulties in evaluating the classification and their use of objective values.
**Results or Findings:** Agreement with the reference increased with radiology training duration (min kappa: 0.47, max kappa: 0.79, p<0.01). Substantial agreement was noted between specialists and the reference (kappa: 0.68-0.72, p<0.01). Agreement was better for non-measurable parameters (e.g., presence of septation) compared to measurable ones (e.g., thickness of septation). Survey results indicated the most challenging parameter was septation thickness, with 100% of participants uncertain between categories 2 vs. 2F and 2F vs. 3. While 53% used objective measurements during assessments, 81% relied on them as a guide rather than exclusively.
**Conclusion:** Moderate-substantial agreement was found among radiologists, improving with experience. Thickness of septations was particularly confusing, especially between certain categories.
**Limitations:** The study's small sample size suggests further research with larger, diverse groups is needed.
**Funding for this study:** No funding was provided for this study.
**Ethics committee - additional information:** AEŞH-EK1-2024-54 numbered file, Ethical Committee, Etlik City Hospital, Ankara

**Author Disclosures:**
Elif Cigdem Karatayli: Nothing to disclose
Şehnaz Evrimler: Nothing to disclose

**14:00-15:30** **Research Stage 2**

### Research Presentation Session: Oncologic Imaging

### RPS 1016

### Radiologic advances in hepatobiliary and pancreatic cancer

**Moderator**
S. De Vuysere; Leuven/BE
*(sofie.devuysere@gmail.com)*

**3D Fractal Dimension Analysis of CT Imaging for Microvascular Invasion prediction in Hepatocellular Carcinoma**
B. Song, *F. Che*; Chengdu/CN
*(chefeng2020@163.com)*

**Purpose or Learning Objective:** This study aimed to assess the potential role of 3-dimensional (3D) fractal dimension (FD) derived from contrast-enhanced computed tomography (CT) images in predicting microvascular invasion (MVI) in hepatocellular carcinoma (HCC) patients.
**Methods or Background:** This retrospective study included 655 patients with surgically confirmed HCC from two medical centers (training set: 406 patients; internal test set: 170 patients; external test set: 79 patients). Box-counting algorithms were employed to compute the 3D FD values from portal venous phase images. Univariable and multivariable logistic regression analyses were used to determine independent clinical and imaging predictors. Diagnostic performance of the model was assessed using receiver operating characteristic (ROC) curve analysis. Recurrence-free survival (RFS) and overall survival (OS) curves were generated via Kaplan-Meier method and compared using the log-rank test.
**Results or Findings:** Patients with MVI-positive HCC exhibited significantly higher FD values compared to those with MVI-negative HCC (P< 0.01). The combined model, integrating alpha-fetoprotein level, tumor size, number, and FD, demonstrated superior diagnostic performance for MVI prediction compared to the clinical model alone, with AUCs of 0.80 (95% CI: 0.75, 0.88) and 0.75 (95% CI: 0.67, 0.83) for the internal test set, and 0.83 (95% CI: 0.72, 0.92) and 0.74 (95% CI: 0.61, 0.85) for the external test set. Patients predicted to have high-risk MVI showed worse RFS and OS outcomes than those predicted to have low-risk MVI (all P<0.05).
**Conclusion:** The 3D FD value significantly differed between MVI-positive and MVI-negative HCC patients. Integration of FD into the clinical model enhances MVI prediction accuracy and may identify patients at high risk.
**Limitations:** Firstly, the retrospective nature of our study introduces inherent limitations. Secondly, our study only analyzed 3D fractal features on the PVP phase.
**Funding for this study:** None
**Ethics committee - additional information:** Institutional Review Board approval was obtained by West China Hospital and Henan Provincial People's Hospital
**Author Disclosures:**
Bin Song: Nothing to disclose
Feng Che: Nothing to disclose

**The impact of pretreatment body composition on conversion surgery feasibility and survival in pancreatic cancer patients undergoing neoadjuvant therapy**
*H. Y. Chen*, B-B. Chen; Taipei/TW

**Purpose or Learning Objective:** This study aims to evaluate the impact of pretreatment body composition, assessed via CT or MRI, on the likelihood of achieving conversion surgery and overall survival (OS) in pancreatic cancer patients undergoing neoadjuvant therapy (NAT).
**Methods or Background:** A retrospective analysis was conducted on 154 patients with pancreatic cancer receiving NAT before planned conversion surgery between May, 2018 and February, 2024. Clinical and laboratory data, including carcinoembryonic antigen (CEA) and carbohydrate antigen 19-9 (CA19-9), were collected. Body composition parameters such as skeletal muscle volume (SM), skeletal muscle index, subcutaneous adipose tissue (SAT), and visceral adipose tissue (VAT) at the L3 vertebral level were automatically calculated using the TotalSegmentator software. Independent predictors of conversion surgery were identified using multivariable logistic regression, and Kaplan-Meier curves and Cox regression models assessed the impact of these variables on OS.
**Results or Findings:** Patients who underwent conversion surgery group had significantly smaller tumor sizes (P=0.033), lower rates of vascular invasion (P=0.042), lower log-transformed CA19-9 levels (P=0.002), higher SM (P=0.049), and higher VAT (P=0.048). Multivariate analysis revealed that log-transformed CA19-9 (P=0.002) and higher SM (P=0.049) were independent predictors of conversion surgery. Conversion surgery (P<0.001) and higher SAT (P=0.001) were associated with better OS. SAT was identified as an independent predictor of OS (P=0.04) after adjusting for age, conversion surgery status, and TNM stage in the multivariate model.
**Conclusion:** Pretreatment body composition, as evaluated through imaging, may be associated with the feasibility of conversion surgery and overall survival in patients with pancreatic cancer receiving NAT. Specifically, higher skeletal muscle volume and SAT appear to be favorable prognostic factors, highlighting the importance of body composition in clinical decision-making and patient outcomes.
**Limitations:** The limitations of the study are single center study and relatively small patient group.
**Funding for this study:** No funding was received for this study
**Ethics committee - additional information:** Research Ethics Committee of National Taiwan University Hospital
**Author Disclosures:**
Bang-Bin Chen: Nothing to disclose
Hsin Yu Chen: Nothing to disclose

### Iodine quantification and LI-RADS classification of hepatocellular carcinoma lesions in contrast-enhanced spectral CT studies

*A. Celestino*, P. Marra, A. Barbaro, C. Gargiulo, R. Muglia, G. Muscogiuri, P. A. Bonaffini, S. Sironi; Bergamo/IT
*(a.celestino1@campus.unimib.it)*

**Purpose or Learning Objective:** The LI-RADS classification is universally employed for hepatocellular carcinoma (HCC) risk stratification of liver nodules in cirrhosis, but it relies on a qualitative evaluation. This study aims to investigate the potential role of material density (MD) parameters in the iodine maps of Spectral Computed Tomography (SCT), to discriminate between LI-RADS categories in cirrhotic patients, therefore increasing the radiologists' confidence in LI-RADS class allocation.
**Methods or Background:** Dual-energy SCT scans of cirrhotic patients with nodules between March 2022 and September 2023 were retrospectively reviewed. All the images were reviewed by trained radiologists to classify nodules as LI-RADS 3, 4 or 5 by consensus. MD maps were generated in the hepatic arterial phase (HAP), portal venous (PVP) and equilibrium phase (EP). Iodine concentration density (ICD) of nodules (ICDnodule) and non-nodular liver parenchyma (ICDliver) were measured to calculate lesion-to-non-nodular liver ICD ratio (LNR), their differences (ΔICD) and ratios (rLNR). Results were correlated with LI-RADS categories.
**Results or Findings:** 69 patients were included and 79 DECT exams were examined. 197 nodules (size 24.67 ± 23.11 mm, mean ± SD) were categorised under different LI-RADS classes, as follows: 44 as LI-RADS 3 (22.3%), 14 as LI-RADS 4 (7.1%), and 139 as LI-RADS 5 (70.6%). Arterial LNR, arterial ICDnodule, ΔICD and rLNR between HAP and PVP could discriminate between LI-RADS 3 and LI-RADS 4+5 nodules (p < 0.001). All the calculated MD parameters showed similar, or slightly higher diagnostic accuracy rates (all AUCs = 70-73%) compared to those previously reported by non-spectral CT (up to 70%).
**Conclusion:** MD parameters of liver nodules measured in SCT scans are viable diagnostic tools that may increase the radiologist's confidence in LI-RADS class allocation in cirrhotic patients.
**Limitations:** The lack of an adequate number of LI-RADS 4 nodules
**Funding for this study:** None
**Ethics committee - additional information:** None
**Author Disclosures:**
Antonio Celestino: Nothing to disclose
Sandro Sironi: Nothing to disclose
Paolo Marra: Nothing to disclose
Pietro Andrea Bonaffini: Nothing to disclose
Alessandro Barbaro: Nothing to disclose
Riccardo Muglia: Nothing to disclose
Carlotta Gargiulo: Nothing to disclose
Giuseppe Muscogiuri: Nothing to disclose

### Simple Cystic Lesions of the Pancreas: Image Quality and Diagnostic Accuracy of Photon-Counting Detector Computed Tomography

*S. Rau*[1], T. Stein[1], A. Rau[1], C. Wilpert[1], F. B. Pallasch[1], B. Bogner[1], S. Faby[2], J. Weiß[1]; [1]Freiburg/DE, [2]Forchheim/DE
*(stephan.rau@uniklinik-freiburg.de)*

**Purpose or Learning Objective:** To evaluate image quality and diagnostic accuracy of photon-counting detector (PCD) computed tomography (CT) for the detection of PCLs compared to conventional energy-integrating detector (EID) CT with MRI serving as reference standard.
**Methods or Background:** In this prospective study, we included consecutive patients who underwent clinically indicated contrast-enhanced PCD-CT of the abdomen and for whom an additional abdominal EID-CT was available. Multiparametric MRI served as the reference standard. CT images were assessed for the presence of PCLs by three radiologists independently in a blinded reading. In addition, image quality, lesion conspicuity, and diagnostic confidence were rated on a 5-point Likert scale (5=excellent). The coefficient-of-variation (CV) and the density difference (in Hounsfield units [HU]) between PCLs and visually normal pancreatic parenchyma were calculated as quantitative imaging measures. Radiation dose was assessed using CTDIvol [mGy].
**Results or Findings:** Among 106 included patients (age 62.7±12.6 years; 45 [42.5%] male), 46 had MRI-confirmed cystic lesions (mean size 8.7±7.4mm; range: 2-45 mm). Diagnostic accuracy for PCLs was significantly higher for PCD-CT vs. EID-CT (area under the curve: 0.81 vs. 0.74; p=0.002; sensitivity: 76.8% vs. 59.4%; specificity 84.4% vs. 88.3%, respectively). Image quality, lesion conspicuity and diagnostic confidence were rated superior for PCD-CT vs. EID-CT (all p<0.001). Quantitative analyses revealed a significantly lower CV (0.19 vs. 0.24; p=0.002) and a higher density difference (94.1 HU vs. 76.6 HU p<0.001) between PCLs and visually normal pancreatic parenchyma at lower radiation doses (7.13 vs. 8.68 mGy; p<0.001) for PCD-CT vs. EID-CT.
**Conclusion:** PCD-CT provided significantly higher diagnostic accuracy and superior image quality for the detection of PCLs compared to conventional EID-CT at lower radiation dose.
**Limitations:** No long-term follow-up and/or histopathological correlation of the detected PCLs were omitted.
**Funding for this study:** None
**Ethics committee - additional information:** The local Institutional Review Board (Ethics Committee of the University Medical Center Freiburg, case number 21-2469) approved this prospective study and written informed consent was obtained from all patients prior to study inclusion.
**Author Disclosures:**
Caroline Wilpert: Nothing to disclose
Jakob Weiß: Nothing to disclose
Alexander Rau: Nothing to disclose
Balazs Bogner: Nothing to disclose
Fabian Bernhard Pallasch: Nothing to disclose
Sebastian Faby: Employee: Siemens Healthineers
Thomas Stein: Nothing to disclose
Stephan Rau: Nothing to disclose

### Prognostic Value of RECIST, mRECIST, and LI-RADS TRA Early Response in Predicting Survival in Hepatocellular Carcinoma Treated with Selective Internal Radiation Therapy

M. Dupuis, A. Dupont, S. Pizza, V. Vilgrain, A. Bando Delaunay, R. Lebtahi, M. Bouattour, M. Ronot, *J. Gregory*; Clichy/FR
*(jules.gregory@aphp.fr)*

**Purpose or Learning Objective:** This study evaluates the prognostic value of tumor response at three months on CT, assessed by RECIST, mRECIST, and LI-RADS Treatment Response Algorithm (LR-TRA) in patients with hepatocellular carcinoma (HCC) treated with selective internal radiation therapy (SIRT).
**Methods or Background:** A retrospective analysis was conducted on 102 HCC patients treated with SIRT between 2018 and 2020. RECIST, mRECIST, and LR-TRA were assessed at 3 months post-SIRT. Overall survival (OS) and progression-free survival (PFS) were assessed using Kaplan-Meier analysis and Cox proportional hazards models.
**Results or Findings:** Median age was 71 years, most patients (90%) had advanced-stage tumors (BCLC-C). After a median follow-up of 32.0 months (95% CI: 16.8-60.9), 60/102 patients died (59%), and 90/102 patients showed tumor progression (88%). Median OS was 20.4 months (95% CI: 15.4-33.0), and median PFS was 14.5 months (95% CI: 6.5-24.5); 1-year OS and PFS rates were 65.6% and 50.7%. Multivariable analysis revealed that early response according to RECIST 1.1 (HR 1.66, p=0.30), mRECIST (HR 1.40, p=0.215), and LR-TRA (HR 0.67, p=0.30) were not predictors of OS. Disease progression evaluated by RECIST (HR 2.55, p<0.001) and mRECIST (HR 2.53, p<0.001), bilirubin levels (HR 1.03, p<0.001) and prothrombin time (HR 0.98, p=0.005) were predictors of OS. For PFS, neither RECIST nor mRECIST response, disease progression, nor LR-TRA viability were predictors.

**Conclusion:** In this advanced-stage HCC population, early response assessed by RECIST, mRECIST, and LR-TRA criteria did not predict OS or PFS after SIRT. However, early disease progression and liver function indicators were prognostic factors for OS.
**Limitations:** Several limitations exist in this single-center, retrospective study, including a small sample size, which may reduce the generalizability of the findings. Additionally, tumor heterogeneity, a key prognostic-factor for poor treatment response and shorter PFS, was not considered.
**Funding for this study:** None
**Ethics committee - additional information:** This single-center retrospective clinical study was approved by the local Institutional Review Board (IRB 00006477), and informed consent was waived due to its retrospective nature.
**Author Disclosures:**
Axelle Dupont: Nothing to disclose
Michel Dupuis: Nothing to disclose
Mohamed Bouattour: Nothing to disclose
Jules Gregory: Nothing to disclose
Maxime Ronot: Nothing to disclose
Silvia Pizza: Nothing to disclose
Rachida Lebtahi: Nothing to disclose
Valérie Vilgrain: Nothing to disclose
Aurélie Bando Delaunay: Nothing to disclose

### Contrast-enhanced Ultrasound using Perfluorobutane for Diagnosing Small HCC (≤20mm) in High-risk Patients: Comparison with MRI LI-RADS Version 2018

*J. Zhou*, Y. Li, L. Lii; Guangzhou/CN
*(zhoujh@sysucc.org.cn)*

**Purpose or Learning Objective:** The sensitivity of Contrast-enhanced ultrasound (CEUS) for diagnosing hepatocellular carcinoma (HCC) is lower than MRI, especially in small liver nodules measuring 20mm or less. This study aimed to compare the diagnostic performance between CEUS with perfluorobutane (P-CEUS) and MRI Liver Imaging Reporting and Data System (LI-RADS) version 2018 (v2018) for small liver nodules in high-risk patients.
**Methods or Background:** This multi-center retrospective study included high-risk patients with newly detected liver nodules measuring 20mm or less from March 2020 to November 2023. Patients underwent CEUS with perfluorobutance (P-CEUS) and concurrent MRI at intervals of no more than 1 month. The reference standard was pathological confirmation or 24-month imaging follow-up (only for benign lesions). The diagnostic performance of CEUS LI-RADS v2017 for P-CEUS, modified criteria for P-CEUS, and MRI LI-RADS v2018 was calculated and compared. For the modified criteria for P-CEUS, LR-4 observations measuring 10 mm or larger with nonrim APHE and no washout were reclassified as LR-5 if aslo showing a Kupffer defect; LR-M observations measuring 10 mm or larger with nonrim APHE and early washout were reclassified as LR-5 if aslo showing a mild Kupffer defect.
**Results or Findings:** A total of 367 participants (301 males; mean age, 54±11 years) with 401 observations (mean diameter, 15±4 mm) were included. Using LR-5 for HCC daignosis, MRI LI-RADS v2018 had higher sensitivity (73% vs 57%, P<0.001) with no significant lower specificity (90% vs 94%, P=0.18) compared with CEUS LI-RADS v2017. The modified criteria for P-CEUS and MRI LI-RADS v2018 showed no significant difference (P>0.05) in sensitivity (70% vs 73%) and specificity (92% vs 90%).
**Conclusion:** CEUS using perfluorobutane with modified criteria demonstrated a diagnostic performance comparable to MRI in HCC diagnosis for small liver nodules (≤20mm).
**Limitations:** Retrospective study
**Funding for this study:** This study was not supported by any funding.
**Ethics committee - additional information:** Approved
**Author Disclosures:**
Lingling Lii: Nothing to disclose
Jianhua Zhou: Nothing to disclose
Yu Li: Nothing to disclose

### Evaluation of dual-layer spectral CT compared with conventional CT for the diagnosis of hepatocellular carcinoma

*M. Youssef Francis*, T. Broussaud, M. Wagner, J. Benzimra, N. Brillat-Savarin, O. Lucidarme; Paris/FR
*(marieyousseffrancis@hotmail.com)*

**Purpose or Learning Objective:** To investigate whether the use of dual-layer spectral CT (DLSCT) improves the diagnostic accuracy of hepatocellular carcinomas (HCCs) compared with conventional CT.
**Methods or Background:** 127 patients were included in this retrospective study. All patients underwent multiphase DLSCT (IQon, Philips Healthcare) for the initial diagnosis of HCC. Arterial phase hyperenhancement (APHE) and portal and delayed washout of each lesion were qualitatively assessed by two radiologists independently using three-point Likert scales and the lesion-to-liver contrast-to-noise ratio (LLCNR) was quantified using ROI in conventional (CIs) and 40keV virtual monoenergetic images (VMIs). For qualitative analysis, interobserver agreement was assessed using the kappa statistic. For quantitative analysis, LLCNRs were compared using Wilcoxon and Scheirer-Ray-Hare tests.
**Results or Findings:** 163 HCCs were independently analyzed. The APHE was considered significantly higher for both observers in VMIs compared to CIs: mean 2.36 vs 1.32 p<0.001. The washout was considered significantly better seen in VMIs compared to CIs in the delayed phase: mean 1.99 vs 1.33, p<0.001; but not in the portal phase: mean 1.74 vs 1.36, NS. Interobserver agreement was good for APHE and washout in both CIs and VMIs. The LLCNR was significantly higher in the arterial phase (6.18vs2.05;p<0.001) and significantly lower in the delayed phase (-2.02 vs -1.10; p<0.001) in 40 Kev-VMIs compared to CIs. As in the subjective analysis, LLCNR was not significantly different in the portal phase (-1.89 vs -1.52;NS).
**Conclusion:** Multiphasic DLCT with 40 keV VMIs increases the visibility of both APHE and washout in the delayed phase of focal liver lesions compared to CIs, leading to the final diagnosis of a higher number of HCCs.
**Limitations:** This is a single-center, retrospective study. DLSCT was not compared with MRI, which is currently the gold standard in the detection of HCC.
**Funding for this study:** No funding was received for this study.
**Ethics committee - additional information:** This study was IRB approved (number CRM-2304-339).
**Author Disclosures:**
Olivier Lucidarme: Nothing to disclose
Marie Youssef Francis: Nothing to disclose
Thomas Broussaud: Nothing to disclose
Mathilde Wagner: Nothing to disclose
Nina Brillat-Savarin: Nothing to disclose
Julie Benzimra: Nothing to disclose

### Bone Mineral Density as a Prognostic Indicator for Overall Survival in Hepatocellular Carcinoma Patients with Portal Venous Tumor Thrombus

L. Müller[1], R. Kloeckner[2], L. Heim[1], F. Stoehr[1], F. Hahn[1], T. Bäuerle[1], A. Weinmann[1], D. Pinto Dos Santos[3], *A. Mähringer-Kunz*[1]; [1]Mainz/DE, [2]Lübeck/DE, [3]Frankfurt/DE
*(aline.maehringer.kunz@googlemail.com)*

**Purpose or Learning Objective:** Low bone mineral density (BMD) has recently emerged as a risk factor in hepatocellular carcinoma (HCC). However, its role in patients with HCC complicated by portal vein tumor thrombosis (PVTT) is unclear. This study explores the potential of BMD as a prognostic indicator within this subgroup, which is characterized by an exceptionally poor prognosis.
**Methods or Background:** This retrospective study included 462 patients with HCC and PVTT diagnosis at our tertiary care center between January 2005 and December 2020. BMD was measured by mean Hounsfield units (HUs) at the midvertebral core of the first lumbar vertebra in computed tomography using the established cut-off of 160 HU. Analysis was performed at two points in time: initial HCC diagnosis and PVTT onset. We analyzed the impact of BMD on median overall survival (OS) and conducted multivariate analysis with established survival predictors.
**Results or Findings:** Median BMD was 136 HU (IQR, 113–160 HU) at HCC diagnosis and 134 HU (IQR, 109–159 HU) at PVTT diagnosis. At initial HCC diagnosis, patients with BMD ≥ 160 HU had a median OS of 10.4 months, compared to 5.5 months in those with BMD < 160 HU (p < 0.001). At PVTT diagnosis, those with higher BMD had a median OS of 8.5 months, versus 4.7 months in patients with lower BMD (p < 0.001). BMD remained an independent prognostic factor in multivariate analysis, alongside growth type and ALBI grade.
**Conclusion:** BMD serves as an independent prognostic marker for survival in patients with HCC and PVTT. Incorporating BMD into existing classification and scoring systems could enhance the accuracy of survival predictions and inform clinical decision-making processes.
**Limitations:** The primary limitation is the retrospective study design, necessitating validation of these findings in a prospective framework.
**Funding for this study:** None
**Ethics committee - additional information:** The study was approved by the responsible ethical body: Ethics committee of the Medical Association of Rhineland Palatinate, Mainz, Germany (Permit number: 15913).

**Author Disclosures:**
Daniel Pinto Dos Santos: Other: Chair of the ESR eHealth and Informatics Subcommittee
Lorena Heim: Nothing to disclose
Fabian Stoehr: Grant Recipient: Research grant, clinician scientist program, Else Kröner-Fresenius-Foundation
Aline Mähringer-Kunz: Nothing to disclose
Felix Hahn: Nothing to disclose
Roman Kloeckner: Speaker: Astra Zeneca, Boston Scientific, , BTG, EISAI, Guerbet, Ipsen, Siemens, and SIRTEX Other: Chair of the ESR Audit and Standards Subcommittee Advisory Board: Boston Scientific, Bristol-Myers Squibb, Guerbet, MSD Sharp & Dohme, Roche, and SIRTEX
Lukas Müller: Grant Recipient: Research grant, clinician scientist program, Else Kröner-Fresenius-Foundation
Tobias Bäuerle: Nothing to disclose
Arndt Weinmann: Nothing to disclose

**Solid pancreatic neoplasms: bridging radiology and cytopathology for accurate diagnosis**
*D. J. A. D. C. E. Aragão*, S. Santos, J. Nobre, L. M. Cabral; Lisboa/PT
*(diogoaragao97@gmail.com)*

**Purpose or Learning Objective:** To retrospectively evaluate a cohort of patients with solid pancreatic lesions who underwent endoscopic ultrasound-guided fine-needle aspiration (EUS-FNA). To review the radiologic features of common solid pancreatic neoplasms and correlate them with cytopathological findings.
**Methods or Background:** A retrospective review was performed at our institution, encompassing 100 EUS-FNA procedures during 18 consecutive months. Patients with solid pancreatic lesions or lesions with a solid component (n=48) were included in the analysis. Clinical data, imaging studies, and cytopathology reports were collected. The most illustrative cases were selected to demonstrate the radiologic and cytopathologic characteristics of these neoplasms.
**Results or Findings:** The most frequently diagnosed solid pancreatic neoplasm was adenocarcinoma (median age: 70 years; 61% male; 71% located in the pancreatic head), typically presenting as a hypovascular mass. Neuroendocrine tumors, often hypervascular, were the second most common neoplasm. Rare cases included one case of pancreatic lymphoma and one case of metastatic disease involving the pancreas.
**Conclusion:** As expected, pancreatic adenocarcinoma, particularly in elderly males and localized in the pancreatic head, was the most commonly diagnosed neoplasm. EUS-FNA combined with cytopathologic analysis, along with multimodality imaging, remains essential in the accurate diagnosis, staging, and management of solid pancreatic neoplasms. The integration of these diagnostic tools ensures a comprehensive multidisciplinary approach, leading to more effective patient care and treatment planning.
**Limitations:** No limitations were identified.
**Funding for this study:** No funding was received for this study.
**Ethics committee - additional information:** This study is educational.
**Author Disclosures:**
Sofia Santos: Nothing to disclose
Luís Monteiro Cabral: Nothing to disclose
João Nobre: Nothing to disclose
Diogo José Afonso Da Cruz E Aragão: Nothing to disclose

**Improving Diagnostic Confidence in Assessing Pancreatic Tissue: Prospective Evaluation of Mechanical Elastography**
*V. Koch*[1], M. Cimprich[1], L. D. Grünewald[1], C. Booz[1], T. Vogl[1], O. Darwish[2], J. Gotta[1], S. Mahmoudi[1], R. Sinkus[3]; [1]Frankfurt/DE, [2]London/UK, [3]Paris/FR

**Purpose or Learning Objective:** This study aimed to evaluate 2D/3D magnetic resonance imaging (MRE) utilizing the gravitational transducer concept compared to the current acoustic product solution (2D-MRE Resoundant) to further characterize pancreatic carcinoma and its potential to provide imaging biomarkers for outcome prediction.
**Methods or Background:** In this prospective study, 40 patients with confirmed pancreatic cancer undergoing MRI of the upper abdomen were enrolled between June 2023 and September 2024. Furthermore, 15 healthy volunteers were included as a healthy reference standard. All participants underwent two examinations using a 40Hz mechanical vibration frequency (Aera 1.5T, Siemens Healthineers, Germany): initially with the acoustic MRE (Resoundant, 2D-MRE, SE-EPI sequence, 11s breath hold [BH]), and subsequently with the gravitational MRE (2D-MRE and 3D-MRE, GRE sequence, TE=9.53ms [in-phase], and fractional motion encoding at 30mT/m, 14s BH). Two experienced readers independently conducted data analysis. Additionally, superimposed analytic plane waves with known properties at various amplitudes and temporal noise levels were employed to investigate biases in stiffness reconstruction (2D/3D) and suggest Quality Indices for 2D/3D.
**Results or Findings:** Significant differences were observed in stiffness values, shear wave speed, and phase angle between healthy volunteers and patients with pancreatic cancer using both MRE approaches (p<.05). Additionally, patients who showed clinical response to chemotherapy exhibited differences in stiffness (p<.05). However, the Bland-Altman plots exhibited a notable bias, with 2D-MRE tending to overestimate stiffness values. 3D-MRE provided several imaging biomarkers that correlated with disease progression and response to therapy. Proposed Quality Indices enabled the identification of pixels exhibiting a deviation exceeding 10% from actual stiffness values in 3D-MRE.
**Conclusion:** Gravitational MRE proves to be an accurate technique for noninvasively characterizing pancreatic tissue. In particular, 3D MRE can provide pertinent functional imaging markers, advancing functional abdominal imaging.
**Limitations:** Single-center study.
**Funding for this study:** This study has been funded by the Doktor Robert Pfleger Foundation.
**Ethics committee - additional information:** The institutional ethical review board approved this prospective study. Written informed consent was obtained from all participants.
**Author Disclosures:**
Christian Booz: Nothing to disclose
Omar Darwish: Nothing to disclose
Thomas Vogl: Nothing to disclose
Vitali Koch: Nothing to disclose
Scherwin Mahmoudi: Nothing to disclose
Ralph Sinkus: Nothing to disclose
Leon David Grünewald: Nothing to disclose
Jennifer Gotta: Nothing to disclose
Marina Cimprich: Nothing to disclose

**Imaging features, management and outcomes of Solitary Necrotic Nodule of the Liver: a case series and review of literature**
L. Asmundo, L. Giaccardi, A. Soro, C. Buonomenna, R. Vigorito, F. G. Greco, A. Casale, *M. Vaiani*; Milan/IT
*(marta.vaiani@gmail.com)*

**Purpose or Learning Objective:** Solitary Necrotic Nodule of the Liver (SNNL) is a rare, benign hepatic lesion frequently misdiagnosed as malignant, leading to unnecessary invasive procedures. This study presents a case series of patients diagnosed with SNNL, focusing on their imaging characteristics, clinical management and outcomes.
**Methods or Background:** This retrospective case series analyzed data from patients diagnosed with SNNL at a tertiary referral center. Collected data included demographics, imaging studies, and follow-up outcomes. A radiologist with 20 years of experience reviewed all imaging studies. The reference standard was histological examination or follow-up imaging
**Results or Findings:** Among 13 patients (54% female; median age 54 years), MRI was the preferred imaging modality, with 3 to 5 scans performed over a median follow-up of 25 months (range 22-70). SNNL typically presented as a necrotic core surrounded by a fibrotic capsule, often with a nodular, elongated, or C-shaped appearance that mimicked biliary dilatation. T1-weighted images showed isointensity in 53.8% of cases, with hypointensity of the core in 84.6%. T2-weighted images revealed isointensity (38.5%) or hypointensity (61.5%) of the core. Nodules remain hypovascular after contrast media injection. Diffusion-weighted imaging displayed no restricted diffusion. During follow-up, most nodules (92.3%) showed a reduction in size, with increased calcification on CT (from 38.5% to 69.2%).
**Conclusion:** SNNL presents diagnostic challenges due to its resemblance to aggressive hepatic lesions, particularly cholangiocarcinoma and metastases. However, careful interpretation of MRI and CT findings can prevent unnecessary invasive procedures.
**Limitations:** The small sample size and single-center design limit the generalizability of the findings. While only a few cases had histological confirmation (30%), long-term imaging follow-up supports the benign nature of SNNL, reducing the need for biopsies and surgeries.
**Funding for this study:** none. The authors did not receive support from any organization
**Ethics committee - additional information:** Local ethic committee approved the study protocol
**Author Disclosures:**
Francesca Gabriella Greco: Nothing to disclose
Luca Giaccardi: Nothing to disclose
Ciriaco Buonomenna: Nothing to disclose
Raffaella Vigorito: Nothing to disclose
Alberto Soro: Nothing to disclose
Luigi Asmundo: Nothing to disclose
Marta Vaiani: Nothing to disclose
Alessandra Casale: Nothing to disclose

14:00-15:30 Research Stage 3

### Research Presentation Session: Interventional Radiology

### RPS 1009

### Advances in musculoskeletal and soft tissue interventions

**Moderator**
M. Szmygin; Lublin/PL
*(mszmygin@gmail.com)*

**Efficacy and safety of image-guided bone biopsies: Insights gained from the German Society for Interventional Radiology and Minimally Invasive Therapy (DeGIR) registry data 2018-2022**

*S. Zensen*[1], F. Behr[1], M. Opitz[1], D. Bos[1], M. Holtkamp[1], L. Salhöfer[1], J. Haubold[1], M. Moche[2], B. M. Schaarschmidt[1]; [1]Essen/DE, [2]Leipzig/DE
*(sebastian.zensen@uk-essen.de)*

**Purpose or Learning Objective:** Image-guided bone biopsies are critical for the diagnosis of a wide range of bone lesions, yet there is limited large-scale data on the technical success, diagnostic yield, and safety of these procedures. This study aims to evaluate the efficacy and safety of image-guided bone biopsies using data from the German Society for Interventional Radiology and Minimally Invasive Therapy (DeGIR, Deutsche Gesellschaft für Interventionelle Radiologie und minimal-invasive Therapie) registry.
**Methods or Background:** In this retrospective observational study, 17,397 bone biopsies from 214 centers between 2018 and 2022 were analyzed. Technical success was defined as the visually successful placement of the biopsy needle within the target lesion. Complications were classified according to the Society of Interventional Radiology (SIR) guidelines.
**Results or Findings:** About one-third of biopsies were performed as outpatient procedures (34%,5,924/17,397). Most biopsies were conducted under local anesthesia (86.6%,15,072/17,397). CT-guidance was used in the majority (68.7%,11,952/17,397). The technical success rate was 98.9% (17,201/17,397), with histological representativeness of 93.2% (10,316/11,073). Outpatient biopsies had a slightly higher technical success rate (99.32%,5,884/5,924) than inpatient biopsies (98.63%,11,316/11,473), though histological representativeness was lower (91.06%,1,284/1,410 vs. 93.48%,9,031/9,661, $p=0.001$). The overall complication rate was low at 0.62% (108/17,397), with major complications in 23.1% (25/108) of cases. Patients with abnormal coagulation parameters had higher complication rates. Sub-/solid lesions had higher histological representativeness compared to necrotic-cystic lesions (94.01%,7,846/8,346 vs. 90.32%,1,558/1,725, $p<0.0001$).
**Conclusion:** Image-guided bone biopsies are highly effective and safe, even in outpatient settings. The data support their continued use as a minimally invasive diagnostic tool, with low complication rates and high diagnostic accuracy.
**Limitations:** This study is limited by the nature of registry data, which is subject to reporting biases and lacks external validation. Incomplete data from some centers also restricted the scope of certain analyses.
**Funding for this study:** This research received no specific grant from any funding agency in the public, commercial, or not-for-profit sectors.
**Ethics committee - additional information:** Ethical approval for this retrospective registry study was granted by the ethics committee of the University of Duisburg-Essen, Germany (22-10893-BO).
**Author Disclosures:**
Denise Bos: Nothing to disclose
Benedikt Michael Schaarschmidt: Nothing to disclose
Johannes Haubold: Nothing to disclose
Sebastian Zensen: Nothing to disclose
Luca Salhöfer: Nothing to disclose
Florian Behr: Nothing to disclose
Michael Moche: Nothing to disclose
Marcel Opitz: Nothing to disclose
Mathias Holtkamp: Nothing to disclose

**Early results of a prospective study on palliative arterial embolization for bone metastases: the EMBONEMET study**

*N. Papalexis*, G. Peta, S. Quarchioni, M. Di Carlo, L. Campanacci, M. Carta, M. Miceli, G. Facchini; Bologna/IT
*(nicolaspapalexis@gmail.com)*

**Purpose or Learning Objective:** To evaluate the clinical and radiological effect of arterial embolization using N-butyl cyanoacrylate (NBCA) as palliation for bone metastases.
**Methods or Background:** This study analyzes the early results of a prospective study "EMBONEMET", designed to prospectively evaluate the safety and efficacy of palliative arterial embolization for bone metastases. Thirty-three patients were enrolled from June 2023 to June 2024. The primary goal was pain control, measured in VAS score at 3, 6 and 12 months follow-up. The secondary goal was the size reduction of the lesion. Data on technical success were also collected. All embolization-related complications were evaluated according to the CIRSE classification system for complications.
**Results or Findings:** The average follow-up was 9 months (range 2 to 12 months). Baseline VAS scores averaged 5,3 (SD 3,19), decreasing to 3.56 (SD 3,1) at three months. Progressive reduction was observed at 6 and 12 months with scores of 3,52 (SD 3,33), and 2,8 (SD 2,7) respectively. Metastatic tumor size was reduced from a mean of 196.8 cm3 (range 39.5 to 486.4 cm3) pre-embolization to a mean of 179.4 cm3 (range 38.2 to 458.6 cm3) at the 6-month follow-up ($p<0.05$). Twenty-nine patients experienced post embolization-related pain, that resolved within 15 days. Three patients experienced sensory loss of the lower leg, paresthesia and pain in the femoral catheter access point.
**Conclusion:** The preliminary results are promising, suggesting that arterial embolization could be a safe and effective tool for pain management and disease control in metastatic bone disease.
**Limitations:** Small sample size, lack of control group
**Funding for this study:** None
**Ethics committee - additional information:** Prospective study approved by the local ethics committee of Emilia Romagna, Italy.
**Author Disclosures:**
Marco Miceli: Nothing to disclose
Michela Carta: Nothing to disclose
Laura Campanacci: Nothing to disclose
Giancarlo Facchini: Nothing to disclose
Giuliano Peta: Nothing to disclose
Simone Quarchioni: Nothing to disclose
Maddalena Di Carlo: Nothing to disclose
Nicolas Papalexis: Nothing to disclose

**Percutaneous cryoablation of progressing extra-abdominal desmoid tumours**

*A. Vanzulli*, L. V. Sciacqua, L. Saggiante, T. Cascella, C. Colombo, E. Palassini, C. Morosi, S. Stacchiotti, A. Gronchi; Milan/IT
*(andrea.vanzulli@unimi.it)*

**Purpose or Learning Objective:** To evaluate the safety and efficacy of percutaneous cryoablation for the treatment of extra-abdominal desmoid tumours (DT) progressing after active surveillance/first-line treatments or threatening to life/function/quality-of-life.
**Methods or Background:** We retrospectively evaluated baseline and post-treatment MR and CT imaging of 27 consecutive procedures performed at our Institution between May 2021 and July 2024. Treatment response was assessed both with standard and modified (m-) RECIST 1.1 (employing also T2WI and DWI to define tumour viability), with the underlying premise that dimensional reduction alone does not adequately capture tissue viability and may incompletely depict responses to local treatments. Three different timepoints were considered: approximately 40 days after treatment, 5-10 months after treatment and 11-16 months after treatment.
**Results or Findings:** The study cohort comprised 25 patients (21 females and 4 males; median age at treatment: 36 years; age range 13-59 years) affected by extra-abdominal treatment eligible for local ablative therapies. Tumour locations included the head & neck (3), the thoracic wall (4), the abdominal wall (19) and the lower extremities (1), with an average maximum diameter of 81,2 mm (range 32-162,3 mm). At approximately 40 days after treatment, mRECIST responses were: 2/19 (10,5%) Stable Disease (SD), 6/19 (31,6%) Partial Response (PR) and 11/19 (57,9%) Complete Response (CR). At 5-10 months after treatment, mRECIST responses were: 9/18 (50%) SD, 2/18 (11,1%) PR and 7/18 (38,9%) CR. At 11-16 months after treatment, mRECIST responses were: 6/16 (37,5%) SD, 4/16 (25%) PR and 6/16 (37,5%) CR. At 11-16 months after treatment, standard RECIST responses were: 1/16 (6,3%) Progressive Disease (PD), 14/16 (87,5%) SD and 1/16 (6,3%) PR.
**Conclusion:** Percutaneous cryoablation represents a feasible treatment for extra-abdominal DT requiring treatment. mRECIST 1.1 outperform standard RECIST1.1 in this clinical scenario.
**Limitations:** Retrospective

Thursday

Funding for this study: Nothing to disclose
**Ethics committee - additional information:** Not applicable
**Author Disclosures:**
Lorenzo Saggiante: Nothing to disclose
Lucilla Violetta Sciacqua: Nothing to disclose
Elena Palassini: Nothing to disclose
Chiara Colombo: Nothing to disclose
Alessandro Gronchi: Nothing to disclose
Andrea Vanzulli: Nothing to disclose
Tommaso Cascella: Nothing to disclose
Carlo Morosi: Nothing to disclose
Silvia Stacchiotti: Nothing to disclose

**Transarterial Embolization for Adhesive Capsulitis: outcome assessment using MRI**
*B. Wang*[1], K-W. Liang[2], H. Y. Lin[2]; [1]Tainan/TW, [2]Taichung/TW
*(wangbow1227@gmail.com)*

**Purpose or Learning Objective:** To assess the efficacy of transarterial embolization (TAE) for adhesive capsulitis (AC) by evaluating clinical outcomes and changes in inflammatory status using magnetic resonance imaging (MRI).
**Methods or Background:** Patients diagnosed with AC, undergoing TAE, and with baseline and 3-month contrast-enhanced MRI evaluations, were included. MRI results were analyzed to assess periarticular capsule/ligament inflammation. Clinical assessments included pain scores using the Numeric Rating Scale (NRS) and functional scores using the Quick Disabilities of the Arm, Shoulder, and Hand (Quick DASH) questionnaire.
**Results or Findings:** Twenty-five patients with AC were included. Significant reductions in average NRS pain scores, and significant improvements in Quick DASH scores and ROM, including anterior flexion and abduction, were observed at 1, 3, and 6 months after TAE (all $P < 0.001$). MRI analyses revealed that TAE significantly decreased the grade of axillary recess (AR) capsule enhancement, the grade of rotator interval (RI) capsule T2 signal intensity, and the grade of RI capsule enhancement (all $P \leq 0.004$).
**Conclusion:** TAE is a promising and safe therapeutic approach for AC, improving pain alleviation and functional recovery. The observed MRI findings suggest that the mechanism of TAE for AC may involve the reduction of inflammation and the elimination of angiogenesis.
**Limitations:** First, it was a single-arm study without a control group, meaning that clinical outcomes such as pain and functional scores were subjective and could be influenced by a placebo effect, necessitating cautious interpretation. Second, the study involved a relatively small number of patients. Third, the classification of hypersignality and enhancement intensity is arbitrary. Finally, different MRI machines with varying magnetic field strengths (1.5T and 3T) were used across patient examinations, introducing potential discrepancies in image interpretation.
**Funding for this study:** Nil
**Ethics committee - additional information:** Institutional Review Board of National Cheng Kung University Hospital: IRB No: B-ER-113-038
**Author Disclosures:**
Bow Wang: Nothing to disclose
Keng-Wei Liang: Nothing to disclose
Hsuan Yin Lin: Nothing to disclose

**A novel treatment for persistent symptoms following spinal surgery: percutaneous ct-guided trans-facetal screw fixation**
*K. Desalos*, V. Sala, P-A. Ranc, L. J. Pavan, T. Vivarrat-Perrin, N. Amoretti; Nice/FR
*(kevindesalos@gmail.com)*

**Purpose or Learning Objective:** Segmental spinal instability after laminectomy and adjacent segment disease (ASD) following arthrodesis often requires repeated surgical interventions, usually by complex surgical procedures such as surgical arthrodesis under general anesthesia which can be often demanding in patients with associated comorbidities. Trans-facetal fixation (TFF) under local anesthesia and CT guidance is a minimally invasive technique which involves placement of percutaneous screws through the posterior facet joints to improve spinal stability. Our retrospective study is aimed at evaluating the efficacy of pain reduction and improvement of daily activities following CT guided TFF, in patients with symptoms related to focal instability secondary to laminectomy or ASD.
**Methods or Background:** TFF were performed in 43 symptomatic patients with previous history of spinal surgery (laminectomy and/or surgical arthrodesis) at Nice University Hospital between 2017 and 2024. The pre and postoperative pain and disability levels were collected prospectively, using the visual analogue scale (VAS) and the Oswestry Disability Index (ODI), at 6-months and 1-year. Long term outcomes were assessed by telephone consultations.
**Results or Findings:** There was a mean decrease of VAS by 3.4 points at 6 months and by 3. 6 points at 1 year. Mean decrease of ODI was 17.1 points at 6 months (47.7 +/- 13.4 versus 30.6 +/- 17.5, P=0.0009)). All the screws were satisfactorily positioned and the pain tolerance under local anesthesia was very good. The average duration of the procedure was 46 minutes, without any significant complications.
**Conclusion:** TFF under CT guidance and local anesthesia resulted in a significant pain reduction and improvement in daily activities, without any complications. TFF under CT guidance and local anesthesia is a safe and effective alternative for symptomatic instability after previous surgical laminectomy and/or arthrodesis.
**Limitations:** Small cohort
Monocentric study
**Funding for this study:** CHU Nice
**Ethics committee - additional information:** Not applicable
**Author Disclosures:**
Luca Jacopo Pavan: Nothing to disclose
Kevin Desalos: Nothing to disclose
Thomas Vivarrat-Perrin: Nothing to disclose
Nicolas Amoretti: Nothing to disclose
Paul-Alexis Ranc: Nothing to disclose
Vincent Sala: Nothing to disclose

**Genicular Artery Embolization for the Treatment of Symptomatic Knee-OA using resorbable particles: a pilot study of 66 patients**
*F. N. N. Fleckenstein*, B. Gebauer, F. Collettini; Berlin/DE
*(florian.fleckenstein@charite.de)*

**Purpose or Learning Objective:** Genicular artery embolization (GAE) is an innovative, minimally invasive therapy for patients with symptomatic knee osteoarthritis (OA) that is unresponsive to conservative treatments. Despite its potential, there is ongoing debate regarding the optimal embolic material for this procedure. This study assesses both the safety and efficacy of GAE using a novel, resorbable particle in treating symptomatic knee OA.
**Methods or Background:** A single-center study was conducted at our institution. Participants were aged between 40 and 90 years, all presenting with moderate to severe knee OA (Kellgren-Lawrence grades 2 to 4) and a history of failed conservative treatments. Baseline pain was measured using the visual analog scale (VAS), and symptoms were assessed using the Knee Injury and Osteoarthritis Outcome Score (KOOS). After femoral arterial access was achieved with a 4 Fr sheath, embolization was performed using Nexsphere-F particles (100-300 µm). Target vessels were identified through digital subtraction angiography, correlating with patients' pain locations. Adverse events and symptom improvements were evaluated at 6 weeks, 3 months, and 6 months following the procedure.
**Results or Findings:** 66 patients were enrolled, with a median age of 68y. OA severity was grade 2 (14%), 3 (44%), and 4 (42%). Technical success was 100%. Skin discoloration and mild knee pain, were reported in 9% of cases. No major complications occurred. At the 6-month, the KOOS quality-of-life index showed an 89% improvement, while VAS score indicated an 82% reduction in pain, median baseline 52/100 and 8/10, respectively.
**Conclusion:** This study demonstrates that using resorbable particles for GAE is both safe and effective in alleviating symptoms associated with OA that do not respond to conservative treatments.
**Limitations:** This is a single-center study without a control group, yet the size of the cohort is considerably large.
**Funding for this study:** No
**Ethics committee - additional information:** Ethics approved
**Author Disclosures:**
Bernhard Gebauer: Nothing to disclose
Federico Collettini: Nothing to disclose
Florian Nima Nima Fleckenstein: Nothing to disclose

**Results of US-guided Hyaluronic Acid in patients with ankle osteoarthritis and osteochondral lesions of the talus**
*M. De Albert De Delas-Vigo*, E. A. Vargas Meouchi, I. Benegas, A. Sallent, G. Duarri, Y. Lara Taranchenko, S. Roche, M. Veintemillas, J. Alonso; Barcelona/ES
*(tisodealbert@gmail.com)*

**Purpose or Learning Objective:** Describe the effects regarding pain relief and possible complications of hyaluronic acid (HA) injection in patients with ankle OA and OLT.
**Methods or Background:** Observational study of patients with ankle OA and OLT that had an ultrasound guided HA injection by the radiology department between January 1st 2020 and December 1st 2023 in our center and a minimum follow-up of 6 months. Patients' visual analog scale (VAS) at baseline, 3 months and 6 months after injection were recorded. Exclusion criteria were administration of another medication (except mepivacaine) or biological therapy and incomplete data collection during follow-up.

**Results or Findings:** 137 patients were referred to the radiology department for an ultrasound guided injection. Fifty-eight patients (63 ankles) that received HA injection for ankle OA and OLT were identified. Thirty-seven (42 ankles) were included. Baseline VAS score was 7.98 ± 1.37; 5.76 ± 2.14, and 6.64 ± 2.07 at 3 and 6 months post injection respectively ($p<0.05$). Patients reported a mean of 7.9 ± 7.81 months with some pain relief. Patients mean age was 59.6 (range 32-83) and mean follow-up was 18.8 ± 12.1 months. Eleven (26.2%) patients received a second HA injection and 7 (16.6%) underwent surgery during follow-up. No complications were recorded in this series.
**Conclusion:** HA injections is a safe treatment that may provide a temporary pain improvement in patients with ankle OA and OLT.
**Limitations:** Retrospective studio
**Funding for this study:** None
**Ethics committee - additional information:** Prospective clinical study
**Author Disclosures:**
Yuri Lara Taranchenko: Nothing to disclose
Sarai Roche: Nothing to disclose
Matias De Albert De Delas-Vigo: Nothing to disclose
Gemma Duarri: Nothing to disclose
Maite Veintemillas: Nothing to disclose
Jaime Alonso: Nothing to disclose
Enrique A. Vargas Meouchi: Nothing to disclose
Iker Benegas: Nothing to disclose
Andrea Sallent: Nothing to disclose

**Percutaneous Hydrodissection Technique For Anterior Cervical Approach of the spine**
I. Ben Rejeb[1], J. Lavigne[1], S. Grange[2], J-B. Noel[3], *J-B. Pialat*[1], N. Stacoffe[1]; [1]Pierre-Bénite/FR, [2]Saint-Étienne/FR, [3]Lyon/FR
*(jean-baptiste.pialat@chu-lyon.fr)*

**Purpose or Learning Objective:** Hydrodissection aims to reduce the risks associated with traditional open surgery by enabling minimally invasive access to the cervical spine. To evaluate the feasibility, effectiveness, and safety of using hydrodissection in percutaneous anterior cervical spine procedures.
**Methods or Background:** A retrospective analysis was conducted on 32 hydrodissection procedures (30 patients) performed between 2020 and 2024 in three medical centers. The patient cohort included individuals with advanced oncological conditions, benign tumors, trauma, and infections. The hydrodissection technique involved gradual injection of a mixture of normal saline and contrast medium under CT guidance, displacing the jugulo-carotid structures and creating a safe space for interventional procedures such as odontoid osteosynthesis, cementoplasty, biopsy, and thermoablation.
**Results or Findings:** The procedure was successfully completed in all 30 patients. The right-sided approach was predominantly used to avoid esophageal injury. The mean volume of dissection fluid used was around 250 ml, with continuous hydrodissection being essential to maintain the created space. A few patient with multiple level procedure reach to 500 ml. The extubation following the procedure was shortly delayed to avoid any compression risk. No perioperative or postoperative complications were reported.
**Conclusion:** The use of hydrodissection in anterior cervical spine procedures offers a safe and effective alternative to open surgery, particularly for high-risk patients. This technique minimizes neurovascular complications and ensures safe procedural access, with no recorded complications in this short serie.
**Limitations:** Further studies with larger cohorts are warranted to standardize the technique and confirm its safety and efficacy.
**Funding for this study:** None
**Ethics committee - additional information:** Actually evaluated by a comittee to be approved for multiple center retrospective inclusion
**Author Disclosures:**
Sylvain Grange: Nothing to disclose
Nicolas Stacoffe: Nothing to disclose
Ilyess Ben Rejeb: Nothing to disclose
Joris Lavigne: Nothing to disclose
Jean-Baptiste Pialat: Nothing to disclose
Jean-Baptiste Noel: Nothing to disclose

**Patient reported outcomes and return to work after CT-guided percutaneous lumbar discectomy: a prospective study**
*P-A. Ranc*, N. Amoretti; Nice/FR
*(passi_ranc@msn.com)*

**Purpose or Learning Objective:** To evaluate the efficacy percutaneous lumbar discectomy (PLD) under computed tomography (CT) guidance on pain, functional capacities and to estimate the speed of recovery by assessing the time before return to work.
**Methods or Background:** Patients treated by PLD were prospectively included between December 2019 and April 2021. Data collected consists in pain, duration of the symptoms, analgesia intakes, time of absence from work and the Oswestry Disability Index (ODI). Patients were followed-up during six months. Duration of hospitalization and time before return to work were reported. The Fisher test was used to compare nominal variables, the Kruskal-Wallis test for ordinal variables, and the Student test to compare quantitative continuous variables.
**Results or Findings:** A total of 87 patients were evaluated (median age, 56; interquartile range [IQR], 42.5-66). The median ODI decreased from 44 (IQR, 33-53) to 7 (IQR, 2-16.5) at six months ($p<0,001$). The median visual analog scale (VAS) decreased from 8 (IQR, 8-9) to 2 (IQR, 0-3) within six months ($p<0,001$). In 96,5% of cases, patients were discharged on the day of the procedure, and 3,5% the following day. No severe adverse events were reported according to the society of interventional radiology (SIR) classification system. Out of the 57 patients at work, 50 were able to return to work during the follow-up with a median time of 8 days (IQR, 0-20).
**Conclusion:** Symptomatic lumbar disc herniations can be successfully treated by PLD, resulting in significant improvement of symptoms, functional capacities, and a fast return to work.
**Limitations:** The limitations of the study are the lack of a control group, so no real comparison among patients treated with surgical methods. Full follow-up was not obtained for 20 patients who had to be excluded from the analysis.
**Funding for this study:** No funding was received for this study.
**Ethics committee - additional information:** This study received approval from the institutional review board and reference on the clinicaltrials.gov database.
**Author Disclosures:**
Nicolas Amoretti: Author: Last Author
Paul-Alexis Ranc: Author: First Author

**Immediate imaging findings after positioning of a new percutaneous interspinous process spacer**
*L. J. Pavan*, P-A. Ranc, T. Vivarrat-Perrin, K. Desalos, V. Kilani, E. Dien, N. Amoretti; Nice/FR
*(lucajpavan@gmail.com)*

**Purpose or Learning Objective:** To evaluate the immediate changing in spine imaging after positioning of a percutaneous interspinous process spacer (IPS) for symptomatic degenerative lumbar spinal stenosis (DLSS).
**Methods or Background:** All patients treated in our Centre from January 2021 to December 2023 with a new percutaneous IPS (Lobster®-Diametros Medical®) were retrospectively reviewed. Patients underwent this procedure for treating a symptomatic DLSS nonresponding to lumbar epidural injection. All procedures were performed with combined CT-scan and fluoroscopy guidance under general anesthesia. For each patient neural foramina area on sagittal plane, as well as zygapophyseal articular space on axial plane, were independently measured by two operators on the treated level of preoperative and postoperative CT-scans.
**Results or Findings:** Thirty-four consecutive patients were treated in the considered period, and all were retrospectively included in the study (mean age 79.2 ± 8.3 years [72-92], 27 males, 17 females). Mean neural foramina area increased from 73 ± 19 to 93 ± 24 mm2 (+ 27%; $p < 0.01$). Mean facet joint articular space increased from 2,2 ± 0,9 to 3,1 ± 1,1 mm (+ 40%; $p < 0.01$). Inter-observer reliability was very good (Cronbach's alpha = 0.9). No procedural complication was reported.
**Conclusion:** These imaging changes may explain the clinical effects of IPS. The widening of foramina may be related to a decrease pression on the middle column and the posterior portion of the intervertebral disk, with reduction of its protrusion. The widening of the zygapophyseal space is linked to a stretching of flavum ligaments with a consequent reduction of its bulging into the spinal canal. Both factors will play a major role in reducing clinical symptoms of DLSS.
**Limitations:** The retrospective design of the study.
**Funding for this study:** None
**Ethics committee - additional information:** Not neede since observational and retrospective
**Author Disclosures:**
Victor Kilani: Nothing to disclose
Luca Jacopo Pavan: Nothing to disclose
Kevin Desalos: Nothing to disclose
Emmanuel Dien: Nothing to disclose
Thomas Vivarrat-Perrin: Nothing to disclose
Nicolas Amoretti: Nothing to disclose
Paul-Alexis Ranc: Nothing to disclose

**Evaluation of the therapeutic value of conventional lymphography for the treatment of inguinal lymphatic fistulas after lymphadenectomy**

*C. Wolfram*, T. J. Vogl, K. Eichler, T. Gruber-Rouh; Frankfurt/DE
*(Christian.Wolfram@unimedizin-ffm.de)*

**Purpose or Learning Objective:** There is critics that advancements in technology, particularly in cross-sectional imaging techniques such as computed tomography (CT) and magnetic resonance imaging (MRI) are better than conventional lymphography. The aim of the present study is to prove that a lipiodol-based conservative lymphography seals persistent lymphatic fistulas, providing a safe and effective alternative to conservative therapies and surgical interventions.
**Methods or Background:** A group of 39 patients who underwent lymphadenectomy resulting into inguinal lymphatic fistulas between 2003 and 2023 was selected. Participants were eligible if they had persistent lymphatic leakage after inguinal lymphadenectomy and were unresponsive to conservative treatment. Of these 39 patients, four could not be statistically evaluated in our retrospective study due to various technical problems. The remaining 35 patients were evaluated. Lipiodol lymphography was performed using transpedal lymphatic vessel cannulation. Data were collected through clinical records (RIS/PACS) and imaging follow-ups. Statistical analyses included the Wilcoxon–Mann–Whitney test using BiAS software. Success was defined as the complete occlusion of lymphatic leakage, and data on complications and additional interventions were collected.
**Results or Findings:** Therapeutic success was achieved in 22 patients (62.86%), with a mean resolution time of 7.13 days. For four patients there was no data on further course, while 13 required additional interventions (three surgical, six radiotherapy). Statistical analysis showed no significant correlation between the volume of administered iodized oil and therapeutic success (p = 0.51), nor lymphatic drainage volume (p = 0.69).
**Conclusion:** Our results highlight that conventional lymphography is a successful therapy. These findings could inform future studies aiming to refine patient selection criteria and optimize treatment protocols for complex lymphatic conditions.
**Limitations:** Not applicable.
**Funding for this study:** No funding was provided for this study.
**Ethics committee - additional information:** The ethics committee notification can be found under the number UID 2023-1444.
**Author Disclosures:**
Christian Wolfram: Nothing to disclose
Kathrin Eichler: Nothing to disclose
Thomas J. Vogl: Nothing to disclose
Tatjana Gruber-Rouh: Nothing to disclose

**14:00-15:30 Research Stage 4**

### Research Presentation Session: Breast

### RPS 1002

### Exploring the role of artificial intelligence in breast imaging

**Moderator**
I. Thomassin-Naggara; Paris/FR
*(isabelle.thomassin@aphp.fr)*

**A 10-year image-derived AI risk model for use in primary prevention of breast cancer**

*M. Eriksson*[1], K. Czene[1], C. Scott[2], P. Hall[1], C. Vachon[2]; [1]Stockholm/SE, [2]Rochester, MN/US
*(mikael.eriksson@ki.se)*

**Purpose or Learning Objective:** Image-derived artificial intelligence (AI) risk models have shown significant potential in enhancing breast cancer (BC) screening through short-term risk assessment. A long-term image-derived AI risk model for primary prevention has yet to be developed and externally validated.
**Methods or Background:** This study utilized a case-cohort approach, including women aged 35-94 recruited between 2009-2017 from population-based screenings in Olmsted County, Minnesota (U.S.), and the KARMA cohort in Sweden. Median follow-up was 10 years, with BCs diagnosed before 5/2022. An image-derived AI risk model, initially developed in a Swedish population, was validated independently in the Olmsted/KARMA cohorts. At study entry, 10-year absolute risks were estimated. Time-dependent discriminatory performance (AUC(t)) and expected-to-observed event ratios (E/O) were calculated.
**Results or Findings:** The combined Olmsted/KARMA cohorts included 8,721 women, with a mean age of 54.4 years (±10.6) in the subcohort and 1,633 incident BC cases with a mean age of 57.0 years (±10.6). The AI-derived 10-year average risks were 3.83% and 3.14%, with E/O ratios of 0.99 (95%CI 0.94-1.05) in Olmsted and 0.99 (95%CI 0.91-1.08) in KARMA. The 10-year AUC(t) values were 0.70 (95%CI 0.68-0.73) for Olmsted and 0.73 (95%CI 0.69-0.77) for KARMA. Using the U.S. Preventive Services Task Force (USPSTF) guidelines, 41% of cases in KARMA were identified as high-risk, compared to 15% with Tyrer-Cuzick-v8 and 5.1% with BCSC-v3 (p<0.01). Under the National Institute for Health and Care Excellence (NICE) guidelines, these figures were 31%, 7.4%, and 0.2%, respectively.
**Conclusion:** The 10-year image-derived AI risk model demonstrated strong predictive performance in both U.S. and Swedish case-cohorts, outperforming traditional clinical risk models in KARMA. This AI model holds significant potential for clinical application in primary prevention, targeting up to 40% of BCs.
**Limitations:** The study population was mainly White women.
**Funding for this study:** Swedish Research Council
**Ethics committee - additional information:** Mayo Clinic and Olmsted Medical Center Institutional review board and the Swedish Ethical Review Authority
**Author Disclosures:**
Mikael Eriksson: Patent Holder: Patent on "system and method for assessing breast cancer risk using imagery" with a licence to iCAD medical, Nashua, NH, U.S. Patent on "compositions and methods for monitoring the treatment of breast disorders" with a licence to Atossa Therapeutics, Seattle, WA, U.S.
Celine Vachon: Nothing to disclose
Christopher Scott: Nothing to disclose
Kamila Czene: Patent Holder: Patent on "system and method for assessing breast cancer risk using imagery" with a licence to iCAD medical, Nashua, NH, U.S. Patent on "compositions and methods for monitoring the treatment of breast disorders" with a licence to Atossa Therapeutics, Seattle, WA, U.S.
Per Hall: Patent Holder: Patent on "system and method for assessing breast cancer risk using imagery" with a licence to iCAD medical, Nashua, NH, U.S. Patent on "compositions and methods for monitoring the treatment of breast disorders" with a licence to Atossa Therapeutics, Seattle, WA, U.S.

**Cracking the Code: Predicting Pathogenic Mutations in Breast Cancer with Ultrasound Radiomics**

*R. M. Pintican*, N. Antone; Cluj-Napoca/RO
*(roxana.pintican@gmail.com)*

**Purpose or Learning Objective:** To evaluate the potential of US-based in the prediction of pathogenic mutational status of breast cancer patients, relevant to prophylactic mastectomy recommendations.
**Methods or Background:** This retrospective study included 73 breast cancer patients tested with multigene panel tests including all seven pathogenic mutations (BRCA1, BRCA2, TP53, PTEN, CDH1, PALB2, and STK11 mutations). US images were acquired prior to any treatment and tumoral and peritumoral areas were used to extract radiomics data. The study population was divided into testing and validation group, each with pathogenic- and non-pathogenic mutation population. Radiomics features were analyzed using machine learning models, alone and in combination with clinical features ( ki67%).
**Results or Findings:** We observed significant differences in radiomics features between pathogenic- and non-pathogenic mutation driven tumors. Using a three-step feature selection process we develop the prediction models (The Mann-Whitney U test, Spearman Correlation and LASSO Regression); the Rad-score 1 ( tumor) achieved an accuracy of 78.6% in identifying pathogenic mutation carriers, while Rad-score 2 (tumor+peritumoral) increased the model's accuracy to 85%. The Rad-Clin 1 and Rad-Clin 2 achieved 83% and 95% acuracy in predicting mutational status. On validation cohort we obtained the following AUCs: Rad-score 1 = 66%; Rad-score 2 = 91%; Rad-Clin 1 = 58%; Rad-clin 2 = 83%.
**Conclusion:** Radiomics models based on US images of breast tumors may provide a promising alternative for predicting pathogenic mutation status in BC patients. The highest accuracy was reached when we combined radiomics data extracted from the tumor and peritumoral area. This approach could reduce dependence on costly genetic testing and expedite the diagnostic process.
**Limitations:** Small sample size
Unicentric study
**Funding for this study:** No funding
**Ethics committee - additional information:** Retrospective study - the informed consent was waived.
**Author Disclosures:**
Roxana Maria Pintican: Nothing to disclose
Nicoleta Antone: Nothing to disclose

**Do we still need to double read the most suspicious screening mammograms when using AI for decision support? A sub-analysis from the AITIC breast cancer screening prospective trial**

*E. Elías Cabot*[1], A. Rodriguez Ruiz[2], J. L. Raya Povedano[1], S. Romero Martin[1], M. Álvarez Benito[1]; [1]Cordoba/ES, [2]Nijmegen/NL
*(eeliascabot@gmail.com)*

**Purpose or Learning Objective:** To evaluate the differences between single and double reading of the most suspicious mammograms after the introduction of AI in breast cancer screening.
**Methods or Background:** This was a sub-analysis of the AITIC paired prospective trial in the breast cancer screening program in Cordoba, Spain. In this trial, between March 2022 and January 2024, 31,301 women (age 50-71) were included and imaged with either DM or DBT based on equipment availability. Two reading strategies were independently applied to each exam: Double blind and non-consensual reading of all exams (standard strategy) and AI-based triaging (AI strategy), where an AI system (Transpara v1.7, ScreenPoint Medical) evaluated the cancer risk of all exams. Cases identified by AI as Low risk were automatically assessed as negative, while exams with Intermediate or Elevated risk were double read with concurrent AI-support. For the latter group, cancer detection (CDR) and false positive rates (FPR) were compared between single and double reading. P values using McNemar and binomial confidence intervals (CI) were computed.
**Results or Findings:** The AI strategy, double reading only 36% of the total screening mammograms, resulted in 228 screen-detected cancers (CDR=7.3/1000, CI: 6.4-8.2/1000) and 1,723 recalls (FPR=4.8%, CI: 4.5-5.0%). Should these exams have been single read with AI support, there would have been 190 screen-detected cancers (CDR = 6.0/1000, CI: 5.2-7.0/1000), and 1,082 recalls (FPR = 2.9%, CI: 2.7-3.0%), a -17% (P<0.05) and -42% reduction (P<0.05) with respect to double reading. The standard strategy resulted in 1,501 recalls (FPR=4.2%, CI: 4.0-4.4%) and 198 cancers (CDR=6.3/1000, CI: 5.5-7.2/1000).
**Conclusion:** After introduction of AI for triage and decision support in screening, increased cancer detection rates were achieved in comparison to standard of care by still double reading a subgroup of the most suspicious exams.
**Limitations:** Single-site.
**Funding for this study:** None.
**Ethics committee - additional information:** Local IRB approval.
**Author Disclosures:**
Alejandro Rodriguez Ruiz: Employee: ScreenPoint Medical
Marina Álvarez Benito: Nothing to disclose
Sara Romero Martin: Nothing to disclose
Esperanza Elías Cabot: Nothing to disclose
Jose Luis Raya Povedano: Nothing to disclose

**Benefits and risks of AI use for reviewing negative screening mammograms**

*C. De Wolf*[1], K. Brändle[2], J-L. Bulliard[2]; [1]Geneva/CH, [2]Lausanne/CH
*(c.dewolf@adsan.org)*

**Purpose or Learning Objective:** Introduction: Breast cancer remains a global health concern, with artificial intelligence (AI) offering promising advancements in improving screening accuracy. Traditional methods, requiring high-volume readings, often lead to fatigue and reading errors. AI addresses these limitations by providing fatigue-free, reproducible results. This study assesses the benefits and costs of AI in detecting high-risk lesions in mammograms initially classified as negative by radiologists.
**Methods or Background:** Methods: Risk scores (Transpara® version 1.7.3) were calculated for 54'300 mammograms from a public Swiss screening program (2018–2021). Data included screen detected (n=321) and interval cancers (n=94), lesion location, and double-blind radiologist readings. We included risk score thresholds considered as elevated risk (61 to 90). Key outcomes included additional workload (additional mammograms in consensus conference), avoided false-negative interval cancers (FN-IC, n=39), and increased false-positive (FP) rates. Multivariable logistic regression was used to predict the rise in FP cases across thresholds.
**Results or Findings:** Results: The FN-IC rate reduction ranged from 8.3% (threshold 90) to 31.3% (threshold 61), with an additional workload of 2 to 67 extra mammograms per 1,000 participants. Avoiding one FN-IC case required 28 to 242 extra readings, resulting in 12 to 86 additional false positives (FP). FP rates rose by 2.1% to 59.3%, with the workload increasing by a third for every 5-point threshold drop up to 75. With an AI threshold set to 85, the false positive rate increased by 2.3‰ (from 40.5‰ to 42.8‰) and the workload would increase by 6 mammograms /1000 participations.
**Conclusion:** AI assistance may enhance mammography sensitivity. However, this comes with a relatively high cost in terms of FP results and additional readings. Therefore, determination of the critical threshold must be context-specific to achieve optimal benefit – risk ratio.
**Limitations:** Retrospective design.
**Funding for this study:** No external funding
**Ethics committee - additional information:** All women signed an informed consent that their anonymized screening data could be used for quality assurance purposes.
**Author Disclosures:**
Jean-Luc Bulliard: Nothing to disclose
Karen Brändle: Nothing to disclose
Christophorus De Wolf: Nothing to disclose

**Patient perceptions and attitudes towards the use of AI in the symptomatic breast unit**

*S. Singh*, R. P. Crean, H. Briody, R. Bruen, N. Hambly, M. Bambrick, D. Duke, M. Mullooly, N. Healy; Dublin/IE
*(snehasingh2412@gmail.com)*

**Purpose or Learning Objective:** Artificial intelligence (AI) has been evaluated in a number of breast screening settings with favourable results. While there are limited studies looking at patient attitudes to AI in breast screening none have examined perceptions of AI in the symptomatic setting. The aim of this study was to determine attitudes towards AI among patients attending the symptomatic breast unit.
**Methods or Background:** An anonymous 15 question, voluntary questionnaire was given to all patients attending the symptomatic breast clinic imaging department of Beaumont Hospital from 01/07/2024 to 30/09/2024. Results were collated in a password protected Excel database and descriptive statistics performed. Likert responses were numericised so that mean of 1 denotes strong agreement and 5 denotes strong disagreement.
**Results or Findings:** Of the 1500 patients who were surveyed, most were aged 40–59 years (62.1%). Almost one-quarter had either a personal (364/1500) or family history of breast cancer (360/1500). 62% (927/1500) had some or strong interest in AI. Regarding the use of AI in healthcare, 46% agreed it was a good idea, 8% disagreed and 46% were indifferent. There was support for AI assisting radiologists in reading mammograms (Mean (M)=2.43,95% CI:2.39-2.48) but disapproval of AI being the sole reader (M=3.82,95% CI:3.77-3.87). Respondents strongly preferred human radiologists over AI for reading mammograms, even if AI were more efficient (M=1.95,95% CI:1.90-1.99) or more accurate (M=2.17, 95% CI:2.13-2.22). 75% of patients would blame both the AI developer and the human radiologist for an incorrect result. All results were statistically significant (p<0.001).
**Conclusion:** Respondents hold favourable views towards the use of AI in healthcare. They welcome use of AI as an adjunct for radiologists but disagree with AI being the only reader of their mammogram.
**Limitations:** N/A
**Funding for this study:** RCSI seed funding
**Ethics committee - additional information:** Approval has been obtained from the hospital audit committee (CA2024/126). Formal ethical approval was not deemed necessary as this is an anonymised, voluntary study.
**Author Disclosures:**
Niamh Hambly: Nothing to disclose
Maeve Mullooly: Nothing to disclose
Deirdre Duke: Nothing to disclose
Marie Bambrick: Nothing to disclose
Richard Bruen: Nothing to disclose
Hayley Briody: Nothing to disclose
Rory Peter Crean: Nothing to disclose
Nuala Healy: Nothing to disclose
Sneha Singh: Nothing to disclose

**The effect of an artificial intelligence decision support system on radiologists' screening mammography performance and visual search patterns**

*J. Gommers*, S. D. Verboom, M. Broeders, I. Sechopoulos; Nijmegen/NL
*(jessie.gommers@radboudumc.nl)*

**Purpose or Learning Objective:** To investigate the effect of using a commercial artificial intelligence (AI) decision support system on the diagnostic performance and visual search patterns of radiologists interpreting screening mammograms.
**Methods or Background:** A multi-reader, multi-case study was performed with 12 Dutch screening radiologists interpreting 150 screening mammography examinations (75 normal, 75 malignant). Radiologists read the examinations without and with AI support while an eye tracker recorded their eye movements. AI classified the examinations as low (maximum region scores:<40), intermediate (40-59), medium-high (60-79), or very-high risk (80-100). Radiologists provided a probability of malignancy score (0-100) and recall decision for each examination. The performance under the two reading conditions was compared using the area under the receiver operating characteristics curve (AUC), sensitivity, and specificity through mixed-model analysis of variance. Reading time and eye tracking outcomes were compared by bootstrap resampling (n=20,000).

**Results or Findings:** The average AUC increased significantly from 0.93 without AI support to 0.97 with AI support (P<.001). There was no evidence of a significant change in sensitivity (81.7% vs 87.2%, P=.06) or specificity (89.0% vs 91.1%, P=.46), although sensitivity tended to increase for AI-classified high-risk examinations (medium-high: 54.9% vs 61.8%, very-high: 89.5% vs 95.6%). Overall reading time did not change significantly (29.4 vs 30.8 seconds, P=.32), but decreased for AI-classified low-risk examinations (25.1 vs 20.1 seconds, P<.001). When using AI, radiologists covered less of the breast area with fixations (11.1% vs 9.5%, P=.005), while spending more time fixating in lesion areas (4.0 vs 5.1 seconds, P<.001).
**Conclusion:** Reading with an AI decision support system increased radiologists' screening performance and allowed them to focus more on lesion-specific areas without increasing overall reading time, indicating a more efficient search.
**Limitations:** Enriched case set and one AI system only.
**Funding for this study:** aiREAD financed by KWF Dutch Cancer Society and the Dutch Research Council (NWO) Domain Applied and Engineering Sciences (AES), as part of their joint strategic research program Technology for Oncology II. The collaboration project is co-funded by the PPP Allowance made available by Health-Holland, Top Sector Life Sciences & Health, to stimulate public-private partnerships.
**Ethics committee - additional information:** The need for ethical approval for this retrospective multi-reader multi-case study was waived by the Research Ethics Committee of Radboud University Medical Center (registration number 2021–13186).
**Author Disclosures:**
Mireille Broeders: Research/Grant Support: Screenpoint Medical, Sectra Benelux, Hologic, Volpara Solutions, Lunit, iCAD Speaker: Siemens Healthcare, Hologic
Sarah Delaja Verboom: Nothing to disclose
Jessie Gommers: Nothing to disclose
Ioannis Sechopoulos: Research/Grant Support: Siemens Healthcare, Canon Medical, ScreenPoint Medical, Sectra Benelux, Volpara Solutions, Lunit, iCAD Speaker: Siemens Healthcare, Canon Medical

### Mammographic features of false positive AI markings on screening mammograms from BreastScreen Norway

*M. A. Martiniussen*[1], M. B. Bergan[2], J. Gjesvik[2], M. Undrum Kristiansen[1], S. Hofvind[2]; [1]Graalum/NO, [2]Oslo/NO
*(maritkarl@gmail.com)*

**Purpose or Learning Objective:** False positive AI markings are an expected challenge when implementing artificial intelligence (AI) in mammographic screening and might contribute to an unsustainable increase in the workload for the radiologists. The aim of this study was to gain knowledge about false positive AI markings from two AI systems on screening mammograms.
**Methods or Background:** In this retrospective study, 129 385 screening examinations from BreastScreen Norway, performed at Ostfold Hospital Trust, 2008-2018, were run through two AI systems. System A was Lunit INSIGHT MMG version 1.1.7.2, and system B was a non-commercial system, developed by the Norwegian Computing Center and the Cancer Registry of Norway. Each model provided a score on a scale from 0-100, and marked the most suspicious areas. Higher score indicated higher risk of cancer. Two radiologists performed a consensus-based informed review of examinations among those with the 5% highest AI score from both systems, interpreted negative at index screening and without cancer diagnosed at index and two consecutive screening rounds. Mammographic features corresponding to the AI markings were classified according to the Breast Imaging Reporting and Data System (BI-RADS). The results were analyzed using descriptive statistics.
**Results or Findings:** Among the examinations that met the inclusion criteria (n=252), 120 examinations from 120 women were randomly selected for review. The mammographic feature corresponding to the AI markings was calcifications for 71.7% (86/120) for system A and 67.5% (81/120) for system B, a mass for 12.5% (15/120) for system A and 14.2% (17/120) for system B, while asymmetry accounted for 10.8% (13/120) for system A and 11.7% (14/120) for system B.
**Conclusion:** Calcifications was the main mammographic feature in screening mammograms with high AI score without diagnosed cancer.
**Limitations:** No limitations were identified.
**Funding for this study:** The South-Eastern Norway Regional Health Authority Ostfold Hospital Trust
**Ethics committee - additional information:** The study was approved by the Regional Committees for Medical and Health Research Ethics (#13294, #11022)
**Author Disclosures:**
Jonas Gjesvik: Nothing to disclose
Marie Burns Bergan: Nothing to disclose
Marit Almenning Martiniussen: Nothing to disclose
Solveig Hofvind: Nothing to disclose
Merete Undrum Kristiansen: Nothing to disclose

### Re-attendance in BreastScreen Norway after a false positive screening result

*M. Larsen*, N. Moshina, J. Gjesvik, S. Sagstad, Å. S. Holen, M. B. Bergan, T. E. Nilsen, S. Hofvind; Oslo/NO
*(maln@kreftregisteret.no)*

**Purpose or Learning Objective:** Higher risk of breast cancer after a false positive versus a negative screening result has been reported. We aimed to compare re-attendance for women with a false positive versus negative screening result using more than 25 years of screening data.
**Methods or Background:** BreastScreen Norway invites women aged 50-69 to biennial screening. The study sample included 3 990 388 screening examinations from 921 309 women where an invitation to the subsequent screening round was available (eligible for re-attendance). Attendance in the subsequent screening round was analysed using mixed logistic regression with age at screening and screening history as covariates and screening outcome as exposure. Predicted probabilities (re-attendance) and 95% confidence intervals (CI) were calculated using average marginal effects.
**Results or Findings:** Having a false positive result after the prevalent screening examination resulted in a re-attendance rate of 88.3%. For women with a negative result, re-attendance was 90.3% after the prevalent examination. Having a false positive or negative result in the 9th screening round, gave a re-attendance rate of 89.0% and 91.1%, respectively. Predicted re-attendance rate was 88.9% (95% CI: 88.9-89.0) after a false positive result and 88.1% (95% CI 88.0-88.3%) after a negative result. Using negative result, false positive without invasive procedure or false positive with invasive procedure as exposure variable, the predicted probabilities of re-attendance were 88.9% (95% CI: 88.9-89.0), 88.4% (95% CI: 88.2%-88.6%) and 87.6% (95% CI: 87.3%-88.0%), respectively.
**Conclusion:** Despite small differences in re-attendance after a false positive versus negative screening result, we consider the difference clinically important. Women should be informed about the importance of re-attending the screening programme after a false positive result.
**Limitations:** We do not have patient reported data on reasons for non-attendance.
**Funding for this study:** No funding was received for this study.
**Ethics committee - additional information:** Programme quality assurance is covered by the Cancer Registry Regulations.
**Author Disclosures:**
Nataliia Moshina: Nothing to disclose
Jonas Gjesvik: Nothing to disclose
Marie Burns Bergan: Nothing to disclose
Åsne Sørlien Holen: Nothing to disclose
Silje Sagstad: Nothing to disclose
Tom Erik Nilsen: Nothing to disclose
Solveig Hofvind: Nothing to disclose
Marthe Larsen: Nothing to disclose

### The application of artificial intelligence to enhance the identification of previously missed non-palpable breast carcinomas

S. A. Mansour, R. M. Kamal, S. Hussien, M. Emara, Y. Kassab, S. Taha, M. M. Gomaa, *Y. M. Nada*; Cairo/EG
*(dr.yasmin.nada.nl@gmail.com)*

**Purpose or Learning Objective:** To investigate the impact of artificial intelligence (AI) on digital mammograms in increasing the chance of detection of missed breast cancer, study the early morphology indictors detected by AI and overlooked by the radiologist and correlate with the missed cancer pathological types.
**Methods or Background:** Screening and diagnostic mammograms (done in 2020-2023) presenting breast carcinomas (n = 1998) were analyzed in concordance with prior one-year-ago (2019-2022) assumed negative or benign) mammograms. Present mammograms were reviewed for the mammographic descriptors: asymmetry, distortion, mass, and microcalcifications. The AI analyzed mammograms and presented abnormalities by overlaying color hue and scoring percentage for the degree of suspicion of malignancy.
**Results or Findings:** Artificial intelligence detected 555 (54%) lesions in the prior mammograms, and in present mammograms (year 2020-2023) targeted 904 (88%) carcinomas. The descriptor proportion of asymmetry was the common presentation of missed breast carcinoma (n=356/555, 64.1%) in the prior mammograms and the AI highest detection rate presented by distortion (100%) followed by grouped microcalcifiactions (80%). AI performance to predict malignancy in previously assigned negative or benign mammograms showed a sensitivity of 73.4%, a specificity of 89%, and an accuracy of 78.4%.
**Conclusion:** Reading mammograms with artificial intelligence enhanced the detection of early cancerous changes. AI detection rate is not correlated with certain pathological types of breast cancer. Close follow-up is required for AI abnormality scoring of low values to minimize the potential for missed breast carcinoma.

**Limitations:** The study is being limited by the retrospective study design; and that it was a two institutional-based study, so multiple institutional-based studies are recommended.
**Funding for this study:** The study has no source of funding
**Ethics committee - additional information:** The study has been ethically approved by the research center of the affiliated institute
**Author Disclosures:**
Sahar Abdelkhalek Mansour: Nothing to disclose
Samar Hussien: Nothing to disclose
Mohammed Mohamed Gomaa: Nothing to disclose
Yasmin Mohamed Nada: Nothing to disclose
Youmna Kassab: Nothing to disclose
Mostafa Emara: Nothing to disclose
Sherif Taha: Nothing to disclose
Rasha Mohamed Kamal: Nothing to disclose

**AI-assisted Breast Mass Classification in Digital Breast Tomosynthesis (DBT): Applicability and insights from a single academic centre**
*G. Cura Curà*[1], G. Bartoli[2], M. Costa[2], E. Regini[2], E. Puglisi[2], F. Piccione[2], F. Schettini[2], M. Durando[2], P. Fonio[2]; [1]Vercelli/IT, [2]Torino/IT
*(gaiacuracura@gmail.com)*

**Purpose or Learning Objective:** In previous research, we trained a deep-learning model to classify benign and malignant masses identified on DBT images (convolutional neural network: efficientNetB0; dataset: 448 masses, size < 6 cm, 221 malignant, 227 benign; accuracy 94%, sensitivity 95.6%, specificity 91.7%). The aim of this study is to evaluate its applicability on breast mass lesions diagnostic work-up in clinical practice.
**Methods or Background:** In this single-centre multireader study, we prospectively collected DBT images from patients with biopsy-proven breast masses (size < 6 cm). For each case, masses were manually delineated with orthogonal axes on the best focused slice in both DBT standard views. A preliminary set of 64 DBTs (46 benign, 18 malignant) was reviewed by three independent dedicated breast radiologists with different experience, then assessed with the AI model. The software provides the benign/malignant classification combined with a prediction confidence score. The response of the software was compared to biopsy results, focusing on BI-RADS classification, error rates, inter-reader agreement, and reading time.
**Results or Findings:** In 7% of cases, there was inter-reader disagreement on the software prediction. The model correctly classified 84% of masses, confirming the 92.8% of lesions categorized as BI-RADS 3 as benign. Software-assisted reading did not modify the reading time compared to conventional methods.
**Conclusion:** In these preliminary results, the highest agreement between radiologists and the AI model was observed with BI-RADS 3 lesions, highlighting the benefit of software-assisted characterization of benign masses. However, variability in inter-reader agreement on the software's predictions limits its reliability in real practice. Further investigations and model refinement are necessary to improve the model robustness.
**Limitations:** Small sample size and single-centre study
**Funding for this study:** No funding was provided for this study
**Ethics committee - additional information:** Non applicable
**Author Disclosures:**
Manuela Durando: Nothing to disclose
Gaia Cura Curà: Nothing to disclose
Matilde Costa: Nothing to disclose
Francesca Schettini: Nothing to disclose
Eugenia Puglisi: Nothing to disclose
Elisa Regini: Nothing to disclose
Federica Piccione: Nothing to disclose
Germana Bartoli: Nothing to disclose
Paolo Fonio: Nothing to disclose

**Multi-site validation of an image-based AI breast cancer risk model for mammography to drive personalized screening after a negative screening**
*A. D. Lauritzen*[1], A. Rodriguez-Ruiz[2], N. Karssemeijer[2], C. De Wolf[3], R. Mann[2], M. Nielsen[1], I. Vejborg[4], M. Lillholm[1]; [1]Copenhagen/DK, [2]Nijmegen/NL, [3]Geneva/CH, [4]Gentofte/DK
*(al@di.ku.dk)*

**Purpose or Learning Objective:** To validate the performance of an image-based AI breast cancer risk model to stratify women attending screening after a negative screening.
**Methods or Background:** Exams from women attending two European screening programs (Denmark and Switzerland) and from a public U.S. database (EMBED) were consecutively sampled. All exams were screen-negative (cancer-free for 180 days) and had follow-up information of between two and six years. Mammography exams were processed by an AI breast cancer risk model (Transpara Risk, ScreenPoint Medical, trial version for research). The risk model computes three image biomarkers (suspicious findings, volumetric breast density, breast texture), and combined with age, it generates a five-year breast cancer risk score per exam. All exams were fully independent from the development of the risk model. Risk model AUCs were computed for each cohort along with sensitivity for women with the highest 10% risk and breast density, respectively.
**Results or Findings:** In total, 98,084 exams were included (31,349, 17,445, and 49,290 from Switzerland, US, and Denmark, respectively) with 1,336 breast cancers diagnosed within 5 years from screening. Images were acquired with machines from four manufacturers (Hologic, Siemens, GE, Philips). The AUCs of the AI risk model were 0.73 (95% CI: 0.69-0.76), 0.74 (95% CI: 0.69-0.79) and 0.74 (95% CI: 0.73-0.76) for Switzerland, US, and Denmark, respectively. When simulating using risk to offer supplemental imaging to 10% of women, after a negative screening, sensitivity was 37% (95% CI: 34%-39%), in comparison to 15% (95% CI: 13-17%) when using density alone.
**Conclusion:** An image-based AI breast cancer risk model shows high accuracy and robustness to stratify women attending screening according to risk and could support personalized screening with higher sensitivity than breast density.
**Limitations:** The retrospective study design is a limitation of this study.
**Funding for this study:** Supported in part by Eurostars (grant E9714 IBSCREEN)
**Ethics committee - additional information:** The Danish Patient Safety Authority and Danish Data Protection Agency approved this retrospective study and the use of relevant Danish data, and waived the need for informed consent (ref. 3–3013–2118, addendum 2019/2023).
**Author Disclosures:**
Andreas David Lauritzen: Nothing to disclose
Alejandro Rodriguez-Ruiz: Employee: ScreenPoint Medical
Mads Nielsen: Nothing to disclose
Martin Lillholm: Nothing to disclose
Nico Karssemeijer: Employee: ScreenPoint Medical
Chris De Wolf: Nothing to disclose
Ilse Vejborg: Nothing to disclose
Ritse Mann: Nothing to disclose

**Replacing one radiologist with AI for independent double reading in mammographic screening**
*M. B. Bergan*, M. Larsen, J. Gjesvik, N. Moshina, S. Sagstad, T. Hovda, H. W. Koch, M. A. Martiniussen, S. Hofvind; Oslo/NO
*(mbbe@kreftregisteret.no)*

**Purpose or Learning Objective:** The aim of this study was to explore how replacing one radiologist with artificial intelligence (AI) for independent double reading in mammographic screening would affect cancer detection.
**Methods or Background:** This study sample consisted of 1,027,430 screening examinations, including 5786 screen-detected cancers, that were independently interpreted by two radiologists in BreastScreen Norway, 2004-2018. The radiologists scored each breast from 1, negative for abnormality, to 5, high suspicion of malignancy, and score ≥2 was considered positive. All examinations were processed by the AI system Lunit INSIGHT MMG version 1.1.7.2, assigning a continuous malignancy score from 0, no risk, to 100, very high risk. Cancer detection was presented for the combination of one radiologist and AI at various AI thresholds for positive examinations.
**Results or Findings:** Of all screen-detected cancers, 86.9% (5028/5786) were classified as positive (score ≥2) by one radiologist. When defining 10% of the examinations with the highest AI score as positive by AI, 79.9% (4622/5786) of the screen-detected cancers and 7.5% (134/1783) of the interval cancers would be detected. When 5% with the highest AI scores were considered positive, 75.5% (4348/5786) of the screen-detected and 5.7% (102/1783) of the interval cancers would be detected. In a scenario where 1% of the examinations were classified as positive by AI, 58.2% (3369/5786) of the screen-detected and 2.4% (42/1783) of the interval cancers would be detected.
**Conclusion:** At an AI threshold of 5%, replacing one of the radiologists with AI in independent double reading of screening mammograms will reduce the reading volume by 50% at the cost of missing 24.5% of screen-detected cancers, but with the possibility of detecting 5.7% of the interval cancers.
**Limitations:** We assume that all cancers classified as positive by the radiologist and AI were detected.
**Funding for this study:** Funding was provided by the Norwegian Cancer Society (Pink Ribbon)
**Ethics committee - additional information:** The study was approved by the Regional Committees for Medical and Health Research Ethics (#2018/2574).

**Author Disclosures:**
Nataliia Moshina: Nothing to disclose
Jonas Gjesvik: Nothing to disclose
Marie Burns Bergan: Nothing to disclose
Henrik Wethe Koch: Nothing to disclose
Tone Hovda: Nothing to disclose
Marit Almenning Martiniussen: Nothing to disclose
Silje Sagstad: Nothing to disclose
Solveig Hofvind: Nothing to disclose
Marthe Larsen: Nothing to disclose

**16:00-17:30** **Research Stage 1**

### Research Presentation Session: Cardiac

### RPS 1103

### Exploring cardiac imaging through quantitative MRI

**Moderator**
N. Fink; Munich/DE

**Improved and automated detection of papillary muscle infarction using joint bright- and black-blood LGE MRI**
*T. Richard*, V. Nogues, T. Boulle, V. De Villedon De Naide, K. Narceau, B. Durand, S. Sridi, H. Cochet, A. Bustin; Bordeaux/FR
*(theo.richard@ihu-liryc.fr)*

**Purpose or Learning Objective:** Papillary muscle infarction (PMI) has been linked to significantly increased mortality, and is a source of ventricular arrhythmias and mitral regurgitation. Bright-blood LGE (PSIR) imaging is the clinical gold standard for myocardial fibrosis characterization. However, the limited contrast at the blood-scar interface makes PMI visualization often challenging. Black-blood LGE imaging overcomes this limitation by improving scar-to-blood contrast. Here, we aim to develop a novel co-registered joint bright- and black-blood LGE technology (SPOT) that could improve visual PMI detection (visu-PMI) , while allowing an automated PMI detection algorithm (auto-PMI).
**Methods or Background:** Short-axis 2D whole-heart PSIR and SPOT images were collected on a 1.5T Siemens Aera system under breath-hold 12min post gadolinium injection. Auto-PMI included image acquisition, statistics-based slice selection, left ventricular endocardium segmentation, blood pool preprocessing, and fibrosis detection. 198 patients participated to the study and were divided into an optimization dataset for auto-PMI parameters selection, and a testing dataset to evaluate visu-PMI and auto-PMI performances. Two radiologists assessed PMI on PSIR and SPOT images. A consensus reading was used as reference standard. Number of patients with PMI were compared. Sensitivity and accuracy of both sequences and auto-PMI were measured. Inter- and intra-observer reproducibility were reported.
**Results or Findings:** Radiologists detected significantly more PMI with SPOT (average increase: 30%). SPOT outperformed average PSIR sensitivity (93% vs. 75%) and accuracy (93% vs. 86%). Average inter- and intra- reproducibility increased with SPOT (79% vs. 74%, 97% vs. 88%). Auto-PMI outperformed PSIR sensitivity (87%), while the accuracy equaled the SPOT average (86%).
**Conclusion:** Co-registered joint bright- and black-blood SPOT imaging allows for improved PMI detection and opens a new door for automated PMI assessment.
**Limitations:** Further validation in larger cohort is warranted. Visu- and auto-PMI reliability depends mostly on contrast selection.
**Funding for this study:** This research was supported by funding from the French National Research Agency under grant agreement ANR-22-CPJ2-0009-01, and from the European Research Council (ERC) grant "SMHEART" under the European Union’s Horizon 2020 research and innovation programme (grant agreement No101076351).
**Ethics committee - additional information:** The study was approved by the Biomedical Research Ethics Committee and all participants provided informed consent for participation.
**Author Disclosures:**
Victor De Villedon De Naide: Nothing to disclose
Soumaya Sridi: Nothing to disclose
Aurelien Bustin: Nothing to disclose
Hubert Cochet: Nothing to disclose
Kalvin Narceau: Nothing to disclose
Thibault Boulle: Nothing to disclose
Victor Nogues: Nothing to disclose
Théo Richard: Nothing to disclose
Baptiste Durand: Nothing to disclose

**Optimizing Static B1+ Shimming for Cardiac MRI at 7 Tesla: Impact on Image Quality and Myocardial Strain**
*A. A. Peters*, K. Fischer, M. Hundertmark, C. Schaub, G. Bonanno, S. Schmitter, D. Günsch, C. Gräni, B. Jung; Bern/CH

**Purpose or Learning Objective:** The purpose of this work was to: 1) establish an efficient workflow for additional 7T-specific static B1+ shimming procedure as short as possible, 2) investigate whether a single set of B1+ shim values calculated at the beginning of the exam provides reliable image quality over a region of interest (ROI), 3) investigate whether spatial resolution or field strength has an influence on volumetric and myocardial strain parameters.
**Methods or Background:** Ten healthy volunteers underwent cine imaging at 7T and 3T on the same day. The B1+ shimming process at 7T used relative B1+ maps to minimize the coefficient of variation (CV) within a ROI covering the heart, with the constraint to maximize excitation homogeneity. Image quality was assessed by two experienced readers using a 4-point Likert scale, and quantitative measures such as left and right ventricular volumes and strain parameters were evaluated.
**Results or Findings:** Results showed that B1+ shimming significantly improved homogeneity, reducing the CV from 61.5% to 23.3%, while increasing transmit efficiency. Image quality at 7T exhibited more inhomogeneities compared to 3T, but these did not impact the clinical assessment of myocardial function. Quantitative analysis revealed higher myocardial mass and smaller ventricular volumes at 7T, though these differences were minimal and insignificant regarding clincial assessment. Strain parameters were comparable between 3T and 7T.
**Conclusion:** In conclusion, this study demonstrates that a fast and efficient workflow for B1+ shimming at 7T can achieve diagnostic image quality and accurate functional analysis comparable to 3T MRI.
**Limitations:** - Small sample size
- Small number of readers
**Funding for this study:** Not applicable
**Ethics committee - additional information:** Not applicable
**Author Disclosures:**
Moritz Hundertmark: Nothing to disclose
Alan Arthur Peters: Nothing to disclose
Kady Fischer: Nothing to disclose
Christoph Gräni: Nothing to disclose
Sebastian Schmitter: Nothing to disclose
Gabriele Bonanno: Nothing to disclose
Christof Schaub: Nothing to disclose
Dominik Günsch: Nothing to disclose
Bernd Jung: Nothing to disclose

**Changes of myocardial extracellular volume fraction measurements in acute versus chronic disease in a large animal infarct model**
*M. C. Halfmann*[1], L. Van Der Meulen[2], M. W. Smulders[2], H. M. J. M. Nies[2], F. Prizen[2], C. Mihl[2], A. Varga-Szemes[3], R. J. Holtackers[2], T. S. Emrich[1]; [1]Mainz/DE, [2]Maastricht/NL, [3]Charleston, SC/US

**Purpose or Learning Objective:** Cardiac MRI derived myocardial extracellular volume fraction (ECV) is a reproducible imaging biomarker for myocardial fibrosis. However, well-controlled evidence on the influence of the timing of the scan in relation to the contrast injection is scarce. Therefore, this study aimed to compare ECV measurements at different time points after contrast injection in a large animal infarct model.
**Methods or Background:** Cardiac MRI was performed at 1.5T while the animals were ventilated and under general anesthesia. Hematocrit levels were drawn directly prior to the scan. Native short-axis T1-maps of the left ventricle were acquired. 7 and 30 minutes following an iv-administration of 0.2 mg/kg gadobutrol, post-contrast T1-maps at identical slice locations were acquired. ECV was calculated for both global and separately for the infarcted and remote myocardium. Results were compared using Pearson’s correlation and paired sample Student’s t-tests.

**Results or Findings:** A total of 13 Yorkshire pigs with balloon catheter-induced myocardial infarction were included in this prospective study. However, 7 animals died before the MRI due to severe arrhythmias and two animals did not undergo the scan due to instability. Thus, MRI was successful in four pigs. Median time between infarction and cardiac MRI was 8 days [IQR 8–8]. While there was a strong correlation between measurements at both time points (r=0.94), ECV was significantly higher at 30 vs. 7 minutes (32.2±5.0% vs. 27.8±4.2%,P=.015). This was confirmed when assessing infarcted (56.9±11.4% vs. 43.7±9.1%,P=.018) and remote myocardium (28.4±2.8% vs. 25.5±3.2%,P=.010) separately.
**Conclusion:** Myocardial ECV by cardiac MRI significantly increases with increasing time after contrast injection in a large animal infarct model. A similar effect was observed in regions with only remote myocardium and only infarcted myocardium.
**Limitations:** The limitation of the study is the limited number of animals.
**Funding for this study:** No funding was received for this study.
**Ethics committee - additional information:** Animal handling complied with the Dutch Law on Animal Experimentation and the European Directive on the Protection of Animals used for Scientific Purposes (2010/63/EU). This study was approved by the Experimental Animal Committee of Maastricht University (DEC2016-002).
**Author Disclosures:**
Casper Mihl: Nothing to disclose
Fritz Prizen: Nothing to disclose
Robert J. Holtackers: Nothing to disclose
Tilman Stephan Emrich: Advisory Board: Siemens Healthineers Speaker: Siemens Healthineers
Moritz Christian Halfmann: Nothing to disclose
Lara Van Der Meulen: Nothing to disclose
Martin W. Smulders: Nothing to disclose
Akos Varga-Szemes: Nothing to disclose
Hedwig M. J. M. Nies: Nothing to disclose

### CMR e' as a novel diagnostic biomarker of Asymptomatic Left Ventricular Diastolic Dysfunction (ALVDD)

*N. Mcveigh*[1], D. T. Ryan[1], F. Ryan[2], M. Ferre[2], J. Mccambridge[2], M. Ledwidge[2], K. Mcdonald[2], J. Dodd[1]; [1]Dublin 4/IE, [2]Dublin/IE

**Purpose or Learning Objective:** Evaluate a novel cardiac MRI biomarker of diastolic dysfunction, CMRe', in pre-clinical patients at risk of heart failure(HF).
**Methods or Background:** 236patients from the PARABLE trial (NCT04687111) underwent CMR. Mitral annular relaxation velocity(CMRe') was measured at four mitral annular anchor points and compared with feature tracking analysis of radial, circumferential and longitudinal diastolic strain rate and velocity as the gold-standard. Comparison were made with a control group of 25 age/gender-matched subjects. Comparisons were made with independent t-test, diagnostic accuracy was performed with receiver operator curve analysis and predictors of diastolic dysfunction were analysed using logistic regression.
**Results or Findings:** LAVimax, LVEDVi, LVESVi and cardiac mass all demonstrated significant increases between patient and control groups (p<0.001 for all). Peak diastolic longitudinal velocity was the only significant feature tracking variable that differed between groups (p<0.001). LAVimax did not correlate with any measured feature tracking parameter when adjusted for clinical, left ventricle and left atrial parameters. In similar multivariate analysis, CMRe' correlated with diastolic radial, circumferential and longitudinal strains rates, as well as radial and longitudinal diastolic velocity measurements (p<0.001). It also correlated with echo e' (r=0.195,p=0.0069), LV mass (r=-.18,p=0.008), LAVimax (r=-.18,p=0.008) and BNP (r=-0.30,p<0.0001). LAVimax and total CMR e' both exhibited high accuracy as independent predictors of diastolic dysfunction (AUC:0.89, 0.76,p<0.001 for both). Combined model (LAVImax and CMR e' total) predicted diastolic dysfunction with an AUC = 0.99. LAVimax, CMR e' and peak diastolic longitudinal velocity were independent predictors of diastolic dysfunction (p<0.001 for all), adjusted for clinical and standard CMR parameters.
**Conclusion:** CMRe' is a precise imaging biomarker for ALVDD. Integrating LAVimax and CMRe' holds promise in optimizing CMR methodologies for identifying patients at risk of diastolic dysfunction.
**Limitations:** Lack of BNP and Echo markers for the control group.
**Funding for this study:** This trial was supported by the Health Research Board of the Government of Ireland, the European Commission Framework Programme 7, the Heartbeat Trust CLG, and Novartis (the manufacturer of sacubitril/valsartan). Under the terms of the grant from Novartis, the study was an investigator-led, Heartbeat Trust–sponsored clinical trial.
**Ethics committee - additional information:** SVUH Ethics Committee
**Author Disclosures:**
Jonathan Dodd: Nothing to disclose
Kenneth Mcdonald: Nothing to disclose
Fiona Ryan: Nothing to disclose
David Thomas Ryan: Nothing to disclose
Maria Ferre: Nothing to disclose
Niall Mcveigh: Nothing to disclose
Joe Mccambridge: Nothing to disclose
Mark Ledwidge: Nothing to disclose

### Right Ventricular Function Predicts Outcome in Heart Failure with Preserved Ejection Fraction: Strain Analysis Derived from MR Feature-Tracking

*L. Zhu*, J. He, S. Zhao, M. Lu; Beijing/CN

**Purpose or Learning Objective:** To evaluate the association between right ventricular (RV) strain parameters derived from cardiac magnetic resonance feature tracking (CMR-FT) and adverse outcomes in patients with heart failure with preserved ejection fraction (HFpEF).
**Methods or Background:** Patients with HFpEF who underwent CMR examination from January 2010 to December 2018 were retrospectively enrolled. The primary endpoint was all-cause mortality.
**Results or Findings:** A total of 1019 consecutive patients with HFpEF (age 56.9 ± 12.3 years; 70% male) were enrolled in this study. During a median follow-up of 7.8 years, 103 (10.1%) patients reached the primary endpoint. In multivariable Cox regression analysis, both RV global longitudinal and circumferential strain were independent predictors of the primary endpoint (HRadj per 1% increase, 1.07 [95% CI: 1.02, 1.12; P = .005] and 1.13 [95% CI: 1.05, 1.21; P < .001], respectively). The full model based on clinical, conventional imaging, and RV strain variables for the primary endpoint improved the model discrimination (C-index = 0.794) compared with the baseline model based solely on clinical variables (C-index = 0.716) and the model incorporating clinical and conventional imaging variables (C-index = 0.760). In receiver operating characteristic analysis for the primary endpoint, the addition of CMR-specific variables including late gadolinium enhancement and FT RV strain yielded an improved area under the curve for the baseline models (all P < .001).
**Conclusion:** RV global longitudinal and circumferential strain derived from CMR-FT were independent predictors of adverse clinical outcomes in patients with HFpEF, providing incremental prognostic value over traditional clinical and CMR-derived risk markers.
**Limitations:** This was a single-center, retrospective study. Echocardiographic parameters, including E/e', were excluded from the Cox regression analysis due to missing values exceeding 50%.
**Funding for this study:** The Beijing Natural Science Foundation (grant no. 7242110)
**Ethics committee - additional information:** This study was approved by the institution ethics review board of Fuwai Hospital.
**Author Disclosures:**
Minjie Lu: Nothing to disclose
Leyi Zhu: Nothing to disclose
Shihua Zhao: Nothing to disclose
Jian He: Nothing to disclose

### Non-compaction Cardiomyopathy and Diastolic dysfunction

*S. S. D. Dereli Bulut*, S. N. Emir; Istanbul/TR
*(ssanembulut@gmail.com)*

**Purpose or Learning Objective:** Non-compaction cardiomyopathy (NCCMP) is an uncommon disorder marked by increased trabeculation of the ventricular wall and the existence of non-compacted myocardial regions. These anatomical alterations may hinder the ventricle's relaxing capacity. Following the preliminary evaluation by echocardiogram (TTE), cardiac magnetic resonance imaging (CMR) is conducted for an in-depth assessment. A non-compacted to compacted myocardium ratio (N/C) exceeding 2.3 substantiates the diagnosis. This study aims to assess individuals with NCCMP who underwent CMR for signs of diastolic dysfunction (DD) and to explore the correlation between disease severity and DD.
**Methods or Background:** This retrospective, single-center study comprised 82 patients initially diagnosed with NCCMP based on TTE data. Cardiac Magnetic Resonance imaging was conducted utilising a 1.5 Tesla MRI scanner (Avanto, Siemens). Morphological and functional evaluations including left ventricular (LV) and right ventricular (RV) volume quantifications, cardiac output (CO), ejection fraction (EF), LV mass, peak ejection rate (PER), and peak filling rate (PFR). Correlation analysis was performed among these metrics.

**Results or Findings:** A substantial relationship was seen between the elevated N/C ratio and both PER and PFR ($p<0.05$; $r=0.31$, $r=0.21$, respectively). A positive association was noted between the N/C ratio and variations in LV mass assessed during the average and end-diastolic phases ($r=0.35$, $p<0.05$). No significant link was seen between the N/C ratio and LV EF ($p>0.05$), and no additional significant relationships were detected.
**Conclusion:** The structural alterations in NCCMP may hinder ventricular relaxation, adversely impacting diastolic function, which can be accurately identified using CMR. Timely identification of DD is essential for enhancing patients' quality of life.
**Limitations:** The patient population was small, the study was planned retrospectively
**Funding for this study:** The authors declared that this study has received no financial support.
**Ethics committee - additional information:** This study was approved by the Ethics Committee of the University Hospital
**Author Disclosures:**
Sevde Nur Emir: Nothing to disclose
Safiye Sanem Dereli Dereli Bulut: Nothing to disclose

### Impact of Formalin Fixation on Biventricular Parameters in Diffusion Tensor CMR: Insights from a Miniature-Swine Model

*L. Zhu*, J. Xu, H. Zhang, C. Cui, P. Sun, S. Zhao, M. Lu; Beijing/CN

**Purpose or Learning Objective:** To examine the impact of formalin fixation on biventricular parameters derived from diffusion tensor cardiac magnetic resonance (CMR) in a miniature-swine model, using histological findings as the reference standard.
**Methods or Background:** High-resolution ex-vivo diffusion tensor CMR data of one healthy miniature-swine were acquired at baseline, and at 5- and 9-days after the fixation with 10% neutral buffered formalin. Diffusion tensor CMR parameters were estimated using the AHA 16-segment model for the left ventricular (LV) wall, and an 8-segment model for the right ventricular (RV) wall. Histology with hematoxylin and eosin staining was performed at 10 days following formalin fixation to assess helix angles (HA) and HA gradients.
**Results or Findings:** Diffusion tensor CMR data at baseline, and 5- and 9-days following formalin fixation were head-to-head analyzed. Subepicardial HAs became much more negative after fixation in both LV and RV walls, and endocardial HAs showed a positive increase, which led to a significant elevation in HA gradients. In the LV wall, mean diffusivity values were slightly reduced during the first 5 days of fixation, followed by a marked decrease over the subsequent 4 days; while in the RV wall, these values also reduced during the first 5 days of fixation but did not change significantly over the next 4 days. HAs derived from diffusion tensor CMR exhibited excellent consistencies with those assessed with histology, among which baseline HAs yielded the highest interclass correlation coefficient of 0.953.
**Conclusion:** Formalin fixation had an impact on both fiber orientations and diffusion properties derived from diffusion tensor CMR, and baseline fiber orientations assessed before fixation showed the best consistency with histology findings.
**Limitations:** This study requires larger sample sizes to enhance the robustness of the findings.
**Funding for this study:** The Beijing Natural Science Foundation (grant no. 7242110)
**Ethics committee - additional information:** Ethics approval was obtained from the Ethics Committee for Animal Study of Fuwai Hospital.
**Author Disclosures:**
Minjie Lu: Nothing to disclose
Leyi Zhu: Nothing to disclose
Peng Sun: Nothing to disclose
Shihua Zhao: Nothing to disclose
Jing Xu: Nothing to disclose
Huaying Zhang: Nothing to disclose
Chen Cui: Nothing to disclose

### Automated myocardial scar segmentation on joint bright- and black-blood late gadolinium enhancement images

*T. Génisson*[1], V. De Villedon De Naide[1], B. Durand[1], K. Narceau[1], J-D. Maes[1], P. Gut[2], H. Cochet[1], M. Stuber[2], A. Bustin[1]; [1]Bordeaux/FR, [2]Lausanne/CH
*(thais.genisson@ihu-liryc.fr)*

**Purpose or Learning Objective:** Bright-blood sequences are used to retrieve heart anatomy information, while black-blood late gadolinium enhancement has shown promise for scar detection. However myocardial scar assessment, crucial for accurate patient prognostic, is time-consuming, operator-dependent and labor-intensive. Here, we propose an artificial intelligence-based method for automated scar segmentation on joint bright- and black-blood LGE (SPOT) images.
**Methods or Background:** A cohort of 70 patients (21% female, age range 28-81yo) with known or suspected ischemic heart disease was divided into a training (70%), validation (10%) and testing (20%) set. Breath-held short-axis 2D whole-heart single-shot co-registered bright- and black-blood SPOT, and reference phase-sensitive inversion recovery (PSIR) images were collected on a 1.5T (Siemens Aera) 12min post-contrast injection. An experienced radiologist manually performed scar segmentation on SPOT and PSIR images. A transformer-based model automatically segmented left ventricular wall on SPOT bright-blood images and the contours were propagated onto black-blood images. Then, a U-net automatically segmented the scar within these contours. Scar segmentation accuracy was assessed. Another experienced radiologist graded the scar segmentation clinical quality (Likert scale: 0=redo; 1=major adjustments; 2=minor adjustments; 3=no adjustments needed). Concordance between scar size assessed with manual PSIR and automated SPOT processing was evaluated. Scar segmentation times were recorded.
**Results or Findings:** Scar segmentation was automatically achieved on SPOT in 0.14s per slice, reaching a global Dice of 76.1%. Scar segmentations were rated 3 in 62%, 1 or 2 in 33% and 0 in only 5% of the cases. No significant differences between scar sizes were found when comparing with manual PSIR processing ($P<0.05$).
**Conclusion:** The proposed method allows for fast, accurate and automated scar segmentation on SPOT images, achieving clinical quality needed to better help guide therapy.
**Limitations:** Validation in larger cohort is warranted.
**Funding for this study:** This research was supported by funding from the French National Research Agency under grant agreement ANR-22-CPJ2-0009-01, and from the European Research Council (ERC) grant "SMHEART" under the European Union’s Horizon 2020 research and innovation programme (grant agreement No101076351).
**Ethics committee - additional information:** The study was approved by the Biomedical Research Ethics Committee and all participants provided informed consent for participation.
**Author Disclosures:**
Victor De Villedon De Naide: Nothing to disclose
Pauline Gut: Nothing to disclose
Aurelien Bustin: Nothing to disclose
Hubert Cochet: Nothing to disclose
Jean-David Maes: Nothing to disclose
Thaïs Génisson: Nothing to disclose
Kalvin Narceau: Nothing to disclose
Baptiste Durand: Nothing to disclose
Matthias Stuber: Nothing to disclose

### Impact of Reduced Spatial Resolution on Cardiac Strain Using Deformable Registration and Feature-Tracking: A Pilot Comparison

*C. G. Glessgen*[1], T. Chitiboi[2], J. Wetzl[2], J-P. Vallee[1]; [1]Geneva/CH, [2]Erlangen/DE

**Purpose or Learning Objective:** Accelerated cardiac MRI cine sequences are essential for real-time imaging and for patients unable to hold their breath but come with reduced spatial resolution. A comparison of strain measurements from high-resolution (HR) and low-resolution (LR) cine data is presented, using two strain methods: deformable registration analysis (DRA) and feature-tracking (FT).
**Methods or Background:** Twenty patients with normal cardiac MRI findings who underwent standard SSFP cines (HR; 0.8×0.8mm2) and compressed-sensing accelerated single-shot SSFP cines (LR; 2.4×2.4mm2) at 1.5 T were retrospectively analyzed. Breath-hold short-axis images were processed using DRA (TrufiStrain v2.4, Siemens Healthineers) and FT (cvi42 v6.1, Circle CVI). Global radial (GRS) and circumferential strain (GCS) were calculated for each image/software pair. Correlations between HR and LR images and between software were assessed using Pearson's R coefficient; concordance was evaluated using Bland-Altman analysis.
**Results or Findings:** GCS correlation between HR and LR was stronger for DRA ($r = 0.93$, $p < 0.05$) than for FT ($r = 0.68$, $p < 0.05$). GRS correlations were similar for DRA and FT ($r = 0.63$ and $r = 0.65$, respectively, $p < 0.05$). Bland-Altman analysis showed a mean GCS difference between HR and LR of 0.02 (LoA: -3.14 to 3.19) for DRA and -4.35 (LoA: -7.00 to -1.69) for FT; for GRS, a mean difference of 18.37 (LoA: 0.60 to 36.15) for DRA and 13.81 (LoA: 6.23 to 21.38) for FT.
**Conclusion:** Cardiac strain measurements from LR and HR cines are well correlated but exhibit significant limits of agreement, which should be considered in clinical practice. DRA showed superior correlation and narrower limits of agreement for GCS, while FT demonstrated narrower but significant limits for GRS.
**Limitations:** This pilot work is limited by its sample size and the absence of pathological data.
**Funding for this study:** None
**Ethics committee - additional information:** Waiver for informed consent (ID: 01574)

**Author Disclosures:**
Jean-Paul Vallee: Nothing to disclose
Carl Guillaume Glessgen: Nothing to disclose
Teodora Chitiboi: Employee: Siemens Healthineers
Jens Wetzl: Employee: Siemens Healthineers

**Precision prediction of heart failure events in patients with NDLVC using multi-parametric cardiovascular magnetic resonance**
*M. Jiang*, M. Lu; Beijing/CN
*(jiangmd1302@163.com)*

**Purpose or Learning Objective:** To assess whether left ventricular (LV) global longitudinal strain (GLS), derived from cardiovascular magnetic resonance (CMR), is associated with (i) major heart failure (HF) events, and (ii) sudden cardiac death (SCD) in patients with non-dilated left ventricular cardiomyopathy (NDLVC).
**Methods or Background:** We conducted a retrospective observational cohort study of patients with NDLVC assessed by CMR, including feature-tracking to assess LV GLS and late gadolinium enhancement (LGE). Long-term adjudicated follow-up included (i) HF hospitalization, heart transplantation or HF death, and (ii) SCD or aborted SCD (aSCD).
**Results or Findings:** Of 386 patients with NDLVC (mean age 45 years, 258 men [66.8%], median LVEF 49% [46–54]) followed up for a median 6.2 years, 68 patients (17.6%) experienced HF events and 15 (3.9%) experienced SCD or aSCD. Following adjustment in a multivariable model, the presence of LGE and LV GLS predicted the HF events (HR 1.95; 95% CI 1.17-3.27; p=0.011 vs. per % HR 1.14, CI 1.07–1.22, p<0.001). However, LV GLS was not associated with SCD/aSCD, whereas LGE presence still was (unadjusted HR 5.36, 95% CI 1.20–23.99, p=0.028). LVEF was neither associated with HF events nor SCD/aSCD.
**Conclusion:** Multi-parametric CMR has utility for precision prognostic stratification of patients with NDLVC. LV GLS stratifies risk of progressive HF, while LGE stratifies both HF and SCD risk.
**Limitations:** T1 mapping and extracellular volume calculation, which were more sensitive in detecting subtle myocardial alteration and fibrosis, were not not performed systematically in patients. Additional investigations are required to assess the potential diagnostic and prognostic significance of mapping techniques within this particular context.
**Funding for this study:** None
**Ethics committee - additional information:** The Institutional Review Board approved this study, and written informed consent was waived due to the retrospective nature of the study.
**Author Disclosures:**
Minjie Lu: Nothing to disclose
Mengdi Jiang: Nothing to disclose

**16:00-17:30** **Research Stage 2**

### Research Presentation Session: Emergency Imaging

### RPS 1117

### Emergency radiology: new technologies and workload challenges

**Moderator**
A. Platon; Geneva/CH
*(alexandra.platon@hcuge.ch)*

**Non-invasive bullet characterization by material decomposition in Photon counting CT**
*B. M. Schaarschmidt*[1], J. Hegmanns[1], J. Wulff[1], V. Haase[2], S. Faby[2], C. Bäumer[1], S. Zensen[1], J. Haubold[1], B. Hartung[1]; [1]Essen/DE, [2]Forchheim/DE

**Purpose or Learning Objective:** Gunshot deaths are a worldwide health concern. Especially in patients with lodged bullets or forensic analyses, image-based bullet characterization is of major interest. Therefore, the present study investigated bullet differentiation from different materials using photon counting computed tomography (PCCT).
**Methods or Background:** Using a new research scan mode, six lead and three brass bullets were analyzed on a NAEOTOM Alpha PCCT system (Siemens Healthineers, Forchheim, Germany). For each scan, a set of four images was reconstructed using four different energy thresholds of the detector (20, 55, 72, and 90 keV). Two independent readers placed three circular regions of interest (ROI) on the 20 keV threshold images on an OsiriX Workstation (Pixmeo SARL, Bernex, Switzerland). These ROIs were then automatically duplicated to the other threshold images. Dual energy indices (DEIs) were computed for the energy threshold pairs of 20/90 keV, 55/90 keV, and 72/90keV based on the measured HUmean and HUmax values.
**Results or Findings:** DEIs of lead and brass projectiles differed significantly, most notably for the 20/90 keV DEI HUmean (Qr40): lead: -0.085±0.021, brass: 0.024±0.048, p<0.001; HUmax (Qr40): lead: -0.093±0.011, brass: 0.023±0.057, p<0.001). For the 55/90 keV and 72/90 keV DEIs, differences between the two projectile materials decreased, but remained statistically significant.
**Conclusion:** In the DEIs derived from different energy threshold images obtained by PCCT, significant differences could be observed between lead and brass bullets. Therefore, PCCT might be a potential technique for bullet material analysis in both clinical and forensic imaging.
**Limitations:** Phantom study only.
**Funding for this study:** The study was performed at Siemens Healthineers facilities in Forchheim, Germany, with support from Viktor Haase and Sebastian Faby (employees of Siemens Healthineers AG).
**Ethics committee - additional information:** As no human or animal subjects were investigated in this study, no approval by the local ethics committee was necessary.
**Author Disclosures:**
Benno Hartung: Nothing to disclose
Viktor Haase: Employee: Siemens Healthineers AG
Benedikt Michael Schaarschmidt: Nothing to disclose
Johannes Haubold: Nothing to disclose
Sebastian Zensen: Nothing to disclose
Christian Bäumer: Nothing to disclose
Jan Hegmanns: Nothing to disclose
Jörg Wulff: Nothing to disclose
Sebastian Faby: Employee: Siemens Healthineers AG

**Virtual monoenergetic images from photon-counting detector CT in thoracic trauma: Improved discriminability of severe lung injury and atelectasis at low-keV energy levels**
H-L. Kaatsch, *B. V. Becker*, D. Dillinger, J. Piechotka, C. Schreyer, R. Schwab, D. Overhoff, S. Waldeck; Koblenz/DE

**Purpose or Learning Objective:** The aim of this study was to assess the usability of virtual monoenergetic images (VMIs) derived from photon-counting detector CT (PCD-CT) for discriminability of severe lung injury and atelectasis after thoracic trauma.
**Methods or Background:** We retrospectively selected 20 polytraumatized patients, who underwent contrast-enhanced whole-body PCD-CT and showed simultaneous presence of trauma-associated atelectasis and pulmonary injury. VMIs were reconstructed from 40 to 120 keV at 10 keV increments. Quantitative image analysis was performed based on density measurements and the calculation of injury-to-atelectasis contrast-to-noise ratio (CNR)). Three radiologists rated subjective discriminability, noise perception and overall image quality by means of a 5-point Likert scale.
**Results or Findings:** CT values for severe lung injury and atelectasis differed significantly at each keV level (p < 0.001) with a gradual decrease for atelectasis from 342 ± 97 HU at 40 keV to 69 ± 15 HU at 120 keV and a near constant behavior for severe lung injury from 42 ± 49 HU at 40 keV to 44 ± 22 at 120 keV. In line with this, the highest injury-to-atelectasis CNR was achieved at 40 keV (3.97) with a continuous decline down to 120 keV (1.21). CNR reached no significant differences between 40 and 50 keV as well as 110 and 120 keV (p >0.05), whereas all other pairings were significantly different (p<0.05). The best ratings for subjective discriminability were reported for VMIs at 40 keV, whereas VMIs at 60-70 keV provided the optimal noise perception and overall image quality.
**Conclusion:** Low-keV VMIs at 40-50 keV from PCD-CT considerably improved the discriminability of severe lung injury and atelectasis after thoracic trauma.
**Limitations:** Lack of a validated reference standard for lung injury vs. atelectasis, retrospective study design and small sample size.
**Funding for this study:** No funding was received for this study.
**Ethics committee - additional information:** This study is a retrospective single-centre analysis that has been approved by the local ethics committee of the chamber of physicians Rhineland-Palatinate in Mainz, Germany (number 2022-16314).
**Author Disclosures:**
Joel Piechotka: Nothing to disclose
Daniel Overhoff: Nothing to disclose
Hanns-Leonhard Kaatsch: Nothing to disclose
Robert Schwab: Nothing to disclose
Benjamin Valentin Becker: Nothing to disclose
Christof Schreyer: Nothing to disclose
Stephan Waldeck: Nothing to disclose
Daniel Dillinger: Nothing to disclose

**Body composition parameters in initial CT imaging of mechanically ventilated trauma patients: Single-centre observational study**
*H-J. Meyer*, T. Dermendzhiev, T. Denecke, M. Struck; Leipzig/DE
*(jonas90.meyer@web.de)*

**Purpose or Learning Objective:** Body composition parameters provide relevant prognostic significance in critical care cohorts and cancer populations. Published results regarding polytrauma patients are inconclusive to date. The goal of this study was to analyse the role of body composition parameters in severely injured trauma patients.
**Methods or Background:** All consecutive patients requiring emergency tracheal intubation and mechanical ventilation before initial computed tomography (CT) at a level-1 trauma centre over a 12-year period (2008-2019) were reanalysed. The analysis included CT-derived body composition parameters based upon whole-body trauma CT as prognostic variables for 30-day mortality, intensive care unit length of stay (ICU LOS) and mechanical ventilation duration.
**Results or Findings:** Four hundred seventy-two patients (75% male) with a median age of 49 years, median injury severity score of 26 and 30-day mortality rate of 22% (104 patients) met the inclusion criteria and were analysed. Regarding body composition parameters, 231 patients (49%) had visceral obesity, 75 patients had sarcopenia (16%) and 35 patients had sarcopenic obesity (7.4%). After adjustment for statistically significant univariable predictors age, body mass index, sarcopenic obesity, visceral obesity, American Society of Anesthesiologists classification ≥3, injury severity score and Glasgow Coma Scale ≤ 8 points, the Cox proportional hazard model identified sarcopenia as significant prognostic factor of 30-day mortality (hazard ratio 2.84; 95% confidence interval 1.38-5.85; P = 0.004), which was confirmed in Kaplan-Meier survival analysis (log-rank P = 0.006).
**Conclusion:** In a multivariable analysis of mechanically ventilated trauma patients, CT-defined sarcopenia was significantly associated with 30-day mortality whereas no associations of body composition parameters with ICU LOS and duration of mechanical ventilation were observed.
**Limitations:** It is a single-center study with a retrospective design.
**Funding for this study:** None
**Ethics committee - additional information:** Approval by the ethics committee at the Medical Faculty, Leipzig University, Leipzig, Germany (IRB00001750, project ID 441/15ek, September 14, 2020)
**Author Disclosures:**
Timm Denecke: Nothing to disclose
Manuel Struck: Nothing to disclose
Tihomir Dermendzhiev: Nothing to disclose
Hans-Jonas Meyer: Nothing to disclose

**AI Denoising Enhances Image Quality and Diagnostic Accuracy While Reducing Radiation Exposure in Prospective LDCT Scans for Acute Abdomen**
*A. S. Brendlin*, U. Schmid, S. Afat; Tübingen/DE

**Purpose or Learning Objective:** To identify the optimal low-dose CT protocol that minimizes radiation exposure while preserving high image quality and diagnostic accuracy in patients presenting with acute abdomen.
**Methods or Background:** A prospective, randomized study was conducted with 180 patients assigned to one of three CT protocols. Each protocol included high-dose (HD) and low-dose (LD) scans reconstructed using Iterative Reconstruction (IR2) and AI Denoising (AID). Subjective image quality was assessed by radiologists evaluating diagnostic confidence, contrast, and sharpness. Objective image quality metrics, including noise and contrast-to-noise ratio (CNR), were measured. Diagnostic accuracy was evaluated through sensitivity and specificity calculations for detecting abdominal pathologies.
**Results or Findings:** Protocol 2 LD achieved the lowest Size-Specific Dose Estimate (SSDE) at 4.83 mGy, significantly reducing radiation dose compared to Protocols 1 and 3 (P < 0.001). AID significantly enhanced subjective image quality in LD scans across all protocols, with Protocols 2 and 3 achieving ratings comparable to HD scans with IR2. Objective assessments showed that AID substantially reduced image noise and increased CNR in LD scans, with Protocol 2 LD exhibiting the highest CNR. In outcome analysis, both Protocols 1 and 2 demonstrated 100% sensitivity and specificity in LD scans with AID, eliminating false negatives and matching the diagnostic performance of HD scans. Protocol 3 maintained high diagnostic accuracy across all doses and reconstruction methods.
**Conclusion:** Protocol 2 with AID emerges as the most effective low-dose CT strategy, offering significant radiation dose reduction while maintaining superior image quality and diagnostic accuracy.
**Limitations:** - Single-Center Study with Limited Sample Size - Potential Observer Bias in Subjective Assessments - Lack of Long-Term Outcome Data - Equipment and Protocol Specificity
**Funding for this study:** None
**Ethics committee - additional information:** University Hospital Tuebingen
**Author Disclosures:**
Andreas Stefan Brendlin: Nothing to disclose
Saif Afat: Nothing to disclose
Ulrich Schmid: Nothing to disclose

**Can perfusion-derived cerebral CT angiography replace routine cerebral CT angiography by using artificial intelligence iterative reconstruction for acute ischemic stroke patients?**
J. Xie[1], *T. Wang*[2], G. Zhang[2], J. Huang[1], M. Wang[1], Y. Lin[1]; [1]Taizhou/CN, [2]Shanghai/CN
*(tiantian.wang@cri-united-imaging.com)*

**Purpose or Learning Objective:** To investigate the feasibility and realiability of replacing routine cerebral CT angiography (CTA) with CT perfusion (CTP)-derived cerebral CTA by using artificial intelligence iterative reconstruction (AIIR) for acute ischemic stroke (AIS) patients.
**Methods or Background:** Forty-nine patients (33-93 y, male: 35) with AIS undergoing a cerebral CTP and a routine CTA were prospectively collected. CTA images derived from CTP at the arterial phase (100kVp/150mAs) were reconstructed with hybrid iterative (Group A1) and AIIR (Group A2), whereas routine CTA images (100kVp/ref. 200mAs) were obtained with hybrid iterative reconstruction (Group B). Two radiologists independently located the responsible vessels, with digital subtraction angiography (DSA) as reference standard. They further graded the image noise, sharpness of the vascular edge, small vessel visibility, and overall diagnosability using a five-point Likert scale (1: poor, 5: excellent). Objective parameters, including the SNR and CNR of the internal carotid artery, the middle cerebral artery, and the basilar artery, were also calculated.
**Results or Findings:** The diagnosis of responsible vessels was consistent by both radiologists, where the diagnostic accuracy of Groups A2 and B were comparable (47/49, 95.92%) and higher than that in Group A1 (43/49, 87.76%). The inter-observer agreement was excellent ($\kappa = 0.84$).All subjective scores were significantly higher in Group A2 than those in Groups A1 and B (all $p < 0.017$), especially for the small vessel visibility ($4.9 \pm 0.2$ vs.$2.8\pm 0.4$ vs. $4.2 \pm 0.5$). AIIR significantly reduced noise, leading to a significantly higher SNR and CNR for all arteries in Group A2 than those in Groups A1 and B (all $p < 0.017$).
**Conclusion:** Perfusion-derived cerebral CTA by use of the AIIR achieves comparable diagnostic image quality to that of the routine cerebral CTA for AIS patients.
**Limitations:** Not applicable
**Funding for this study:** No funding was received for this study.
**Ethics committee - additional information:** Not applicable
**Author Disclosures:**
Jicheng Xie: Nothing to disclose
Tiantian Wang: Nothing to disclose
Guozhi Zhang: Nothing to disclose
Minke Wang: Nothing to disclose
Jinbiao Huang: Nothing to disclose
Youyou Lin: Nothing to disclose

**Real life performance of a commercially available AI for post-traumatic intracranial haemorrhage detection on CT-scans: a supportive tool**
*L. Mabit*, G. Herpe; Poitiers/FR
*(leo.mabit@outlook.fr)*

**Purpose or Learning Objective:** Investigate the real-world performance of qER.AI, an artificial intelligence-based CT haemorrhage detection tool, in a post-traumatic population.
**Methods or Background:** Retrospective monocentric observationnal study of a dataset of consecutively acquired head CT scans at the emergency radiology unit to explore a brain trauma. AI performance was compared to groundtruth determined by expert consensus. A subset of nighshift cases with radiological report of junior resident was compared to AI results and groundtruth.
**Results or Findings:** 682 head CT scan were analyzed. AI demonstrated a sensitivity of 88.8% and specificity of 92.1% overall, with a positive predictive value of 65.4% and a negative predictive value of 98%. AI's performance was comparable to junior residents in detecting ICH, with the latter showing a sensitivity of 85.7% and a high specificity of 99.3%. Interestingly, the AI detected two out of three ICH cases missed by junior residents. When AI and residents performances were combined, the sensitivity improved to 95.2%, and the overall accuracy reached 98.8%.
**Conclusion:** This study shows a better performance of AI and radiologist resident associated than each one alone. These results are encouraging to rethink the radiological workflow and the future of triage of this large population of brain traumatised patients in emergency unit.

**Limitations:** The limitations of the study are exclusion of some CTs due to logistics issues (potential selection bias), and a low number of positive ICH cases in the nightshift subset.
**Funding for this study:** No funding was received for this study.
**Ethics committee - additional information:** This study was reviewed and approved by the Institutional Review Board of CERIM (CRM-2401-400).
**Author Disclosures:**
Guillaume Herpe: Nothing to disclose
Léo Mabit: Nothing to disclose

**Increasing workload in emergency radiology: A decade-long trend analysis using Large Language Models**
*M. T. Meyer*, E. M. Merkle, T. Heye; Basel/CH

**Purpose or Learning Objective:** This study aims to identify trends explaining the significant rise in CT examinations ordered by the emergency department (ED) of a tertiary hospital in the last decade (2014 to 2023) using large language models (LLM).
**Methods or Background:** Radiology Information System data were extracted for CT scans performed on ED patients between 2014-2023. The open-source Llama 3.1 405B LLM was used to classify each report into YES (expected pathology confirmed), NO (no relevant pathology), and OTHER (unexpected but relevant pathology found). Trends were analyzed by body-region, focusing on patients older than 65 years, as this group showed the highest increase in imaging.
**Results or Findings:** Over the past decade, the number of CT scans increased 2.2 times for ED patients, with a more pronounced rise for patients over 65 years (2.4). Preliminary results show that the LLM achieved an accuracy of 84% compared to a manual review of 500 randomly sampled reports. Scans of abdomen/pelvis (YES in 49-60%) and thorax/abdomen/pelvis (YES in 54-63%) remained stable with minor annual fluctuation. CT scans of the skull in polytrauma patients showed steady increase in NO findings from 43% (2016) to 59% (2022). CT of the skull showed a gradual increase in NO findings from 57% (2015) to 66% (2022). CT of the thorax remained relatively stable (YES 41-46% from 2014-2019), with notable peaks during the COVID-pandemic (52% in 2021).
**Conclusion:** Diagnostic yield across different organ groups varies greatly with high proportions of NO findings in skull CTs (typically to rule-out bleeding). Over time, unremarkable CT scans, particularly of the skull, have increased, while trends for other body regions (e.g., thorax/abdomen/pelvis), were less distinct.
**Limitations:** The results are preliminary. Accuracy of the LLM is limited, which might lead to over-/underestimation of trends.
**Funding for this study:** None
**Ethics committee - additional information:** The study is retrospective and General Consent is available.
**Author Disclosures:**
Tobias Heye: Nothing to disclose
Elmar M. Merkle: Nothing to disclose
Manfred Tobias Meyer: Nothing to disclose

**Increasing On-Call Workload for Radiology Trainees: A Five-Year Analysis in a Tertiary Referral Centre**
*P. Rohan*, H. Briody, C. Mccarthy, M. M. Morrin; Dublin/IE
*(pat_rohan@outlook.com)*

**Purpose or Learning Objective:** The demand for acute diagnostic radiology services during on-call hours continues to increase, placing strain on radiology trainees. This study aims to assess the on-call workload for Radiology Specialist Registrars (SpRs) over a five-year period at a model 4 tertiary referral centre in Ireland.
**Methods or Background:** A retrospective review was conducted, analysing the volume of computed tomography (CT) scans performed during on-call hours (weekdays 17:00-08:00, weekends, and public holidays) across three months—January, July, and November—from 2019 to 2023. Data were categorized by study type, focusing on Emergency Department (ED) requests and key scan types, including non-contrast brain CT (NCB), CT abdomen/pelvis (CTAP), CT thorax/abdomen/pelvis (CT TAP), and cerebral stroke protocol ("FAST"). Statistical analysis included percentage changes and paired t-tests.
**Results or Findings:** Between 2019 and 2023, there was a 25% increase in the total number of on-call CTs performed ($p = 0.098$), with a significant 46% rise in ED-related studies ($p < 0.05$). Significant increases were observed in NCBs (38%, $p < 0.05$) and CT TAPs (220%, $p < 0.05$). The number of CTs performed after midnight increased by 82% ($p = 0.055$), while FAST protocol CTs rose by 41% ($p = 0.056$), reflecting the growing incidence of stroke in Ireland.
**Conclusion:** The increasing on-call workload for Radiology SpRs, particularly driven by ED requests and the rising demand for stroke imaging, underscores the need for careful planning and resource allocation to manage future demand. Addressing trainee workflows is essential to sustaining radiology services while maintaining high standards of care.
**Limitations:** This retrospective study is limited to on-call CT workload. Other modalities like ultrasound, MRI, and X-rays, as well as communication burdens with clinical teams, were not assessed and should be explored in future studies.
**Funding for this study:** No funding was received for this study.
**Ethics committee - additional information:** Ethics committee approval not required.
**Author Disclosures:**
Cathal Mccarthy: Nothing to disclose
Martina M Morrin: Nothing to disclose
Hayley Briody: Nothing to disclose
Pat Rohan: Nothing to disclose

**Trends in CT examination utilization in the emergency department during and after the COVID-19 pandemic**
*F. Kempter*, D. Jäschke, J. Vosshenrich, B. Ceresa, T. Heye; Basel/CH

**Purpose or Learning Objective:** To investigate the impact of COVID-19 pandemic-related measures on trends and volume in CT examinations requested in the emergency department.
**Methods or Background:** CT examinations of the head, chest, and/or abdomen-pelvis (n=161,008), and chest radiographs (n=113,240) performed at our tertiary care hospital between 01/2014- 12/2023 were retrospectively analyzed. CT examinations (head, chest, abdomen, dual- region and polytrauma) and chest radiographs requested by the emergency department during (03/2020-03/2022) and after the COVID-19 pandemic (04/2022-12/2023) were compared to a pre-pandemic control period (02/2018-02/2020). Analyses included CT examinations per emergency department visit, and prediction models based on pre-pandemic trends and inpatient data. A regular expressions text search algorithm determined the most common clinical questions.
**Results or Findings:** The usage of dual-region and chest CT examinations were higher during (+116,4% and +115.8%, respectively; $p<0.001$) and after the COVID-19 pandemic (+88,4% and +70.7%, respectively; $p<0.001$), compared to the control period. Chest radiograph usage decreased (-54.1% and -36.4%, respectively; $p<0.001$). The post-pandemic overall CT examination rate per emergency department visit increased by 4.7%. The prediction model underestimated ($p<0.001$) the growth (dual-region CT: 22.3%, chest CT: 26.7%, chest radiographs: -30.4%), and the rise ($p<0.001$) was higher compared to inpatient data (dual-region CT: 54.8%, chest CT: 52.0%, CR: -32.3%). Post- pandemic, the number of clinical questions to rule out “pulmonary infiltrates”, “abdominal pain” and “infection focus” increased up to 235.7% compared to the control period.
**Conclusion:** Following the COVID-19 pandemic, chest CT and dual-region CT usage in the emergency department experienced a disproportionate and sustained surge compared to pre-pandemic growth.
**Limitations:** Single-center, retrospective design limits generalizability. CT as standard imaging for suspected COVID-19 pneumonia (2020–2022) may explain the shift. Only clinical questions, not diagnoses, were analyzed. Despite a large sample size, data mislabeling is possible.
**Funding for this study:** Not applicable.
**Ethics committee - additional information:** The local ethics committee of northwestern and central Switzerland (EKNZ, project ID 2022-01016) approved this study.
**Author Disclosures:**
Tobias Heye: Nothing to disclose
Dominik Jäschke: Nothing to disclose
Felix Kempter: Nothing to disclose
Benjamin Ceresa: Nothing to disclose
Jan Vosshenrich: Nothing to disclose

**An 18-year Retrospective Analysis of Urgent Inpatient and Emergency CT Reporting at a UK Local General Hospital**
*A. Gmati*, Z. Foster, M. Mobley; Warwick/UK
*(aimen.gmati@outlook.com)*

**Purpose or Learning Objective:** Radiology has transformed how acute medicine is practiced, with growing imaging demands placing significant strain on radiology departments in the UK. This study retrospectively examines 18 years of urgent inpatient and emergency CT reporting at South Warwickshire University NHS Foundation Trust (SWFT) to assess trends in workload, staffing, and operational changes over this period.

**Methods or Background:** Data were extracted from the Radiology Information System (RIS) at SWFT, encompassing approximately 140,000 urgent scans performed since 2007. These were analysed to show changes over time such as seasonal variability and peaks in workload throughout the week, as well as the overall trend for increased imaging.
**Results or Findings:** Urgent CT workload at SWFT has significantly increased over the study period, reflecting national trends. This timescale covers a period of rapid growth and operational change at SWFT, such as the introduction of weekend shifts and the implementation of outsourced teleradiology for out-of-hours reporting. Typical daily reporting numbers have increased from 12 scans per day in 2007 to over 50 in 2024. Staffing has not kept pace, with WTE consultant numbers dropping from 12 in 2019 to 9.5 in 2023. Despite outsourcing, many departments continue to struggle with growing diagnostic demands.
**Conclusion:** Across the UK, CT usage surged from 1 million scans in 1997 to over 6.7 million in 2021. Rising CT demand at SWFT reflects broader national trends, with increasing pressure on radiology services. The UK's Royal College of Radiologists predicts a shortage of 1,669 radiologists by 2025, highlighting a national critical staffing issue. Operational changes such as weekend working may provide temporary relief but place increasing demands on the same pool of radiologists. Systemic reforms and increased investment are essential to meet future diagnostic demands.
**Limitations:** N/A
**Funding for this study:** N/A
**Ethics committee - additional information:** N/A
**Author Disclosures:**
Zoe Foster: Nothing to disclose
Mark Mobley: Nothing to disclose
Aimen Gmati: Nothing to disclose

**Is population aging behind the increasing workload in emergency radiology?**

*J. Sarnecki*, M. T. Meyer, E. M. Merkle, T. Heye; Basel/CH
*(jedrzej.sarnecki@gmail.com)*

**Purpose or Learning Objective:** To investigate the trends in imaging in emergency patients over the last decade.
**Methods or Background:** 190'028 emergency CT examinations from 1/1/2015 to 31/12/2023 of patients aged 18-100 years, performed within 24h of the request, were included. The examination time was stratified into dayshift (8-17:30h), late-shift (17:30-22h), night-shift (22-8h) and weekend-dayshift (Saturday/Sunday 8-17:30h). The patients were divided into two age groups (18-64 versus 65-100 yrs.) and binned using 5-year increments. The percentage increase in imaging numbers was calculated based on 2015.
**Results or Findings:** A sharp overall increase in CT imaging numbers was observed in 2020 (18-64 yrs.: 127.0% vs. 65-100 yrs.: 155.3%), peaking in 2022 (18-64 yrs.: 182.4% vs. 65-100 yrs.: 235.6%). The mean age in the 18-64 yrs. group increased from 44.8 (2015) to maximally 46.3 (2022), but did not increase for 65-100 yrs. (79.2 vs. 79.3 yrs.). The day-shift imaging numbers showed a linear steady increase, whereas the night-shift examinations showed the strongest increase, peaking in 2023 with 267.5% (18-64 yrs) and 403.0% (65-100 yrs) followed by changes in weekend and late-shift CT numbers (18-64 yrs.: 119.8%;127.1% vs. 65-100 yrs.: 173.1%; 139.0%). Between 2020-2023 for the younger age group, age-bins 55-59 (199.5-260.9%) and 60-64 (140.8-200.6%) showed the largest relative increase compared to 2015 for late-, night- and weekend-dayshift CTs combined (all age bins 18-64yrs: 30-112.2%). In the 65-100 yrs. group, the largest relative increase was seen for patients 95-100 years old (318.2-360.0%) compared to other 5-year age bins (145.7-276.8%). Chest, Chest-Abdomen-Pelvis and Polytrauma CT showed the greatest increase.
**Conclusion:** The results support that a change in imaging was triggered and sustained by the COVID-19 pandemic with a disproportionate increase in CT imaging volume overall and especially for older patients during non-day-shifts.
**Limitations:** Retrospective, single-center study.
**Funding for this study:** No funding.
**Ethics committee - additional information:** Not applicable
**Author Disclosures:**
Tobias Heye: Nothing to disclose
Elmar M. Merkle: Nothing to disclose
Jędrzej Sarnecki: Nothing to disclose
Manfred Tobias Meyer: Nothing to disclose

**The increase and inappropriate requests for cranial CT scans in emergency departments contribute to overuse and decrease test accuracy**

*A. Martínez López*, G. Pagán Vicente, E. Otón González, H. Ortiz Mayoral, E. C. Cotillo Ramos, M. S. Canales, B. Molina-Lozano, J. Plasencia Martínez, I. Santiago Suárez; Murcia/ES
*(amabandrea6@gmail.com)*

**Purpose or Learning Objective:** The number of non-traumatic urgent cranial computed tomography (NT-UCCT) is exponentially increasing but limited research has been conducted on the quality of clinical justification. We aimed (1) to assess how clinical information in the electronic NT-UCCT request agreed with that provided in the emergency department discharge summary and (2) to analyze the potential effect of those discrepancies on the NT-UCCT overload.
**Methods or Background:** Patients undergoing NT-UCCT in 2017-2021 were randomly selected for this retrospective study. Signs and symptoms (S/S) in electronic request and emergency department discharge summary, acute and relevant computed tomography (CT) findings, and final diagnosis at emergency department discharge summary were collected. Concordance between both and their association with CT findings and final diagnosis were analyzed.
**Results or Findings:** We recruited 156 patients: 80 men; mean age, 55. Acute, relevant CT findings were detected in 28 cases (17.9%). The final diagnosis was neurological disease 46 (29.5%), non-neurological disease 58 (37.2%), and no definitive diagnosis 51 (32.7%). Full agreement between the electronic request and emergency department discharge summary occurred in 36 patients (23.1%). Motor deficit was the most frequent false positive S/S (18; 11.54%), having low positive predictive value (30.30%; 95%CI 15.59-48.71%) and worst association with acute relevant CT findings than when true positive (OR 2.54; 95%CI 0.04-6.21 vs. OR 6.26, 95%CI 2.21-17.78). Nausea/vomiting was the third most common false negative S/S (13; 10.26%) and reduced the likelihood of acute relevant CT findings (OR 0.126; 95%CI 0.016-0.971; p = 0.020).
**Conclusion:** Discrepancies between electronic request and emergency department discharge summary were observed in >75% of patients, leading to unnecessary NT-UCCT tests.
**Limitations:** Differences in how clinical information is recorded, the variability among physicians, results not applicable to large hospitals, small sample size limited detailed analysis.
**Funding for this study:** No
**Ethics committee - additional information:** The study was approved by the ethics committee.
**Author Disclosures:**
Estefania Corina Cotillo Ramos: Author: Author
Elena Otón González: Author: Author
Isabel Santiago Suárez: Author: Author
Marta Sánchez Canales: Author: Author
Juana Plasencia Martínez: Advisory Board: Author
Gonzalo Pagán Vicente: Author: Author
Belén Molina-Lozano: Author: Author
Andrea Martínez López: Author: Author
Herminia Ortiz Mayoral: Author: Author

**16:00-17:30** **Research Stage 3**

### Research Presentation Session: Imaging Informatics and Artificial Intelligence

### RPS 1105

### Artificial intelligence and planet radiology: the green machine

**Moderator**
R. Mirón Mombiela; Herlev/DK
*(mirona@ufm.edu)*

**How do radiology department carbon footprints contribute to climate change?**

*S. D. Jagadeesha*[1], R. Botchu[2]; [1]Mysuru/IN, [2]Birmingham/UK
*(sushdj98@gmail.com)*

**Purpose or Learning Objective:** The purpose of this study is to investigate paper usage in the radiology department of a single hospital institution over the last three years to forecast paper usage up to 2050.

Thursday

**Methods or Background:** This retrospective study was performed in the radiology department of our tertiary orthopedic hospital. The study included forms used for diagnostic and interventional procedures in various departmental modalities. Diagnostic procedures require one to three forms and interventional procedures require three forms each. Based on the established ratio that 1.2 trees are cut for every 10,000 papers used, the study calculated the number of trees cut annually over the past three years and projected paper usage and tree loss until 2050
**Results or Findings:** Paper usage was distributed between diagnostic and interventional procedures, with 67% used in diagnostics and 33% in interventions. The corresponding number of trees cut during this period amounted to 53.7 trees, with 47.4 trees for diagnostic procedures and 6.4 trees for interventional procedures. A total of 57.8 trees for diagnostic procedures and 11.7 trees for interventional procedures were forecasted to be cut annually from 2024 to 2050, cumulatively being 1227 trees by the year 2050.
**Conclusion:** Our individual department had a significant contribution from paper usage in the carbon footprint of the department. Adoption of digitalized appointment, prescribing and patient records is important in reducing this and achieving NHS net-zero targets.
**Limitations:** The use of paper for forms, there are other significant sources of paper consumption within the department. For example, extensive paper packaging used for interventional consumables, and tissue paper used for various applications, such as covering ultrasound and CT couches, are also contributing to the overall paper usage in the radiology department. This has been excluded in the study.
**Funding for this study:** Not applicable
**Ethics committee - additional information:** This has obtained ethical committee clearance from the Hospital.
**Author Disclosures:**
Rajesh Botchu: Nothing to disclose
Sushmitha Devihalli Jagadeesha: Nothing to disclose

**AI-driven green gains: Enhancing efficiency with environmental benefits in Imaging**
*P. Strouhal*[1], N. Khan[2], A. Heathcote[1], M. Darwish[3], S. Persichini[3], B. Miles[1], M. Trumann[4], I. Farid[3]; [1]Warwick/UK, [2]Dubai/AE, [3]Chalfont St Giles/UK, [4]Freiburg/DE
*(pstrouhal@alliance.co.uk)*

**Purpose or Learning Objective:** Alliance Medical Ltd (AML) provides diagnostic imaging for 800,000 NHS patients annually via networked facilities. Growing concerns over operational and energy efficiencies in 2022 prompted AML's implementation of GE HealthCare's Imaging360 solution. 18 months on, we showcase how such Artifical Intelligence (AI) driven solutions are pivotal in refining patient flows, scheduling, staffing, energy usage and logistics management within imaging services.
**Methods or Background:** Integration comprised 6 separate Imaging360 components utilising data from various sources, including HL7, DICOM, Business Intelligence software and CSV extracts; mobile and static CT and MRI scanners were incorporated from multiple sites across England (with PET-CT scanners now being onboarded).
**Results or Findings:** Using predictive analytics, AML reduced missed appointments from 17% to 3% per week, improving resource utilisation. Optimising protocols and schedules done on-cloud allowed reduced senior staff travel (approx. 380-480 km/month) and time (37.5hr/week) to manage scanner protocols; and significantly increased scanner efficiency: - MRI: Throughput rose from 21 to 27 scans per day (+33%), with kWh/patient reduced from 15.5 to 11.8. This saved 3.7 kWh per exam —enough to power 45 average households annually. One MRI site increased throughput by 43%, achieving 410 exams per month increase. - CT: scanner throughput improved by 256 scans per month average, cutting idle time; and reducing protocol variability for CT chest, abdomen, pelvis from 47 to 15 standardised protocols, with related radiation doses lowered from 500 to 350 mGy.cm. Increased throughput was achieved with no extra staff or equipment.
**Conclusion:** Integrating AI into radiology workflows allows transformative changes not only of operational efficiencies and cost savings, but improved sustainable practice. Going forward, further eco-friendly innovations could enhance both performance and sustainability across the healthcare imaging sector.
**Limitations:** Imaging360 optimised for GE HealthCare scanners
**Funding for this study:** GE HealthCare supporting implementation of AI platform
**Ethics committee - additional information:** N/A
**Author Disclosures:**
Naeem Khan: Employee: GE HealthCare
Peter Strouhal: Board Member: Alliance Medical
Brad Miles: Employee: Alliance Medical
Stefano Persichini: Employee: GE HealthCare
Mariam Darwish: Employee: GE HealthCare
Imran Farid: Employee: GE HealthCare
Markus Trumann: Employee: GE HealthCare
Ann Heathcote: Employee: Alliance Medical

**Balancing Sustainability and Performance: Evaluating Energy Use, Carbon Footprint and Task Performance of Locally run Large Language Models for Radiology Report Simplification**
*A. Gupta*, R. Dheeka, R. Kumar, A. Rastogi, H. Malhotra, K. Rangarajan; New Delhi/IN
*(amit.aiims2014@gmail.com)*

**Purpose or Learning Objective:** To investigate tradeoffs between performance and energy use when using different locally-run large language models (LLMs) and prompts for patient-centric simplification of radiology reports.
**Methods or Background:** This study evaluated three different open-source LLMs (Meta's Llama 3.1-8B, Microsoft's Phi-3.5-Mini and Mistral-7B) using five different prompts to simplify 50 computed tomography report impressions, collected from our tertiary-care oncology centre. Models were run on a local workstation with graphic processing unit. Energy use (in watt-hours) and carbon emissions (in grams) for each inference, were measured using an open-source tool (CodeCarbon). Readability of original and generated simplified reports was quantitatively assessed using an average score of four readability indices. LLM performance for simplification task was measured as difference in readability scores between original reports and LLM-generated reports. Energy efficiency ratios (performance per watt-hour) and carbon footprint (performance per gram of emissions) were calculated for each model-prompt combination.
**Results or Findings:** Llama-prompt 5 (multi-shot learning) demonstrated the highest task performance (7.36), best energy efficiency ratio (31.89/Wh), and least carbon footprint (44.70/g). Phi-prompt 5 achieved high simplification (6.14) and energy efficiency (25.87/Wh). For Mistral, prompt 1 (no context) was optimal (2.15/Wh and 3.01/g), but performance (1.16) lagged behind Llama and Phi. Friedman test revealed significant differences among readability scores ($p < 0.001$), with post-hoc Wilcoxon tests showing significant improvements for Llama and Phi over the original and Mistral, and Llama outperforming Phi (adjusted $p < 0.0033$).
**Conclusion:** Different LLM-prompt combinations showed variability in energy use, carbon emissions, and simplification task performance. These results highlight the importance of LLM-prompt combination selection for medical applications, balancing sustainability and performance.
**Limitations:** Development of test prompts has inherent potential for subjectivity. Apart from prompt engineering, we did not use other accuracy improving techniques like retrieval augmented generation.
**Funding for this study:** None
**Ethics committee - additional information:** Study approved by the Institute Ethics Committee All India Institute of Medical Sciences, New Delhi (Ref. No. - IEC-343/15.06.2023)
**Author Disclosures:**
Krithika Rangarajan: Nothing to disclose
Rohit Kumar: Nothing to disclose
Ashish Rastogi: Nothing to disclose
Amit Gupta: Nothing to disclose
Hema Malhotra: Nothing to disclose
Rahul Dheeka: Nothing to disclose

**Greenhouse gas emissions due to long-term data storage of reformatted CT series and strategies for mitigation**
Y. Jia[1], M. Deng[1], *R. Burger*[1], S. L. Sheard[1], K. Hanneman[2], M. Drucker Iarovich[2], R. Illing[1], A. G. Rockall[1]; [1]London/UK, [2]Toronto, ON/CA
*(r.burger@nhs.net)*

**Purpose or Learning Objective:** Image data storage and associated greenhouse gas (GHG) emissions is accelerating, yet strategies to minimise this are limited. Reducing the average file size of CT studies by reducing the number of reformats stored could help reduce emissions. This study aims to estimate GHG emissions associated with storage of CT reformats by modelling measurements from endometrial cancer baseline staging CT. Secondary aims were to model the findings comparing cloud storage emissions and assess the hypothetical GHG mitigation impact of a data retention policy

**Methods or Background:** Baseline staging CT chest, abdomen, and pelvis (CT-CAP) in 183 endometrial cancer patients in a UK cancer centre between 2013-2016 were analysed (Cohort A). The number of stored multiplanar reformats, maximum intensity projections images and lung reconstructions were recorded. The file size of each reformat was noted for 30 studies (Cohort B). Comparison was made with an external dataset of 100 baseline CT-CAP from Canada between 2018-2023 (Cohort C). Mitigation of GHG emissions was projected for different storagescenarios.
**Results or Findings:** Reformatted series were present in cohort A (97%, 179/183), cohort B (97%, 29/30) and cohort C (100% ,100/100). Of the total file size of cohort B (25590mb), 65% (16685mb) was reformats and/or duplicate series. On-premise storage of all reformats for cumulative new UK endometrial cancer cases from 2020-2040 would produce 349 metric tonnes CO2 equivalent (MTCO2e). Over 20 years, projected reductions in MTCO2e were 69%(107/349) for storing only acquired axial slices, 80%(70/349) for switching to cloud storage, and 36%(222/349) for implemented a data retention policy.
**Conclusion:** A significant number of studies contained unnecessary reformats, increasing average file size. A strategy to revise CT data storage protocols can substantially lower radiology GHG emissions, without compromising patient care.
**Limitations:** Small selective patient dataset.
**Funding for this study:** None
**Ethics committee - additional information:** Institutional approval was obtained for a quality improvement project.
**Author Disclosures:**
Kate Hanneman: Nothing to disclose
Yifan Jia: Nothing to disclose
Michael Deng: Nothing to disclose
Rebecca Burger: Nothing to disclose
Moran Drucker Iarovich: Nothing to disclose
Rowland Illing: Nothing to disclose
Sarah Lucy Sheard: Nothing to disclose
Andrea Grace Rockall: Nothing to disclose

**Ultrasound's Hidden Environmental Cost: Linens and Disposables**
C. L. Thiel[1], J. Leschied[2], D. Carver[2], *J. R. Scheel*[2], R. Omary[2], M. Vigil-Garcia, Phd[3], P. Gehrels[3], C. Meijer[3], O. Struk[3]; [1]Madison, WI/US, [2]Nashville, TN/US, [3]Amsterdam/NL
*(john.scheel@vumc.org)*

**Purpose or Learning Objective:** To understand the sources of environmental impact of ultrasound imaging in a US-based, adult diagnostic radiology service.
**Methods or Background:** A life cycle assessment (LCA) was used to evaluate the environmental impact of 2 ultrasound machines and their surrounding resource needs, including production, use and disposal of other capital equipment, linens, disposable supplies, pharmaceuticals, and data storage. A sensitivity analysis was performed to assess the impact of low-carbon electricity sources.
**Results or Findings:** Contrary to expectations, linens and disposable supplies emerged as the major contributors to ultrasound's greenhouse gas (GHG) emissions, each accounting for approximately 30% of its total impact. Energy use from the ultrasound units themselves was comparatively lower, at 7%, along with the production of the ultrasound units (7%), and the production and use of workstations (11%). The study also noted that ultrasound equipment spent 30-45% of the time in non-scanning mode. The sensitivity analysis showed the use of photovoltaics as an electricity source would reduce US’s GHGs by 9%; however, it would not shift the major sources of GHGs away from linens and disposable supplies.
**Conclusion:** Reducing linen use, adopting reusable alternatives for disposable supplies, and encouraging textile and supply manufacturers and laundering facilities to develop lower carbon alternatives are essential for improving the sustainability of ultrasound practices.
**Limitations:** The study, while providing valuable insights into the environmental impact of US, has limitations due to its single-center focus; exclusion of mammography, nuclear medicine, and interventional radiology; a one-month data collection period; and some data and modeling limitations.
**Funding for this study:** No external funding was received for this study. Philips and VUMC independently contributed to this study through in-kind labor.
**Ethics committee - additional information:** This study was deemed non-human subjects research.
**Author Disclosures:**
Marta Vigil-Garcia, Phd: Employee: Philips
Cecilia Meijer: Employee: Philips
Reed Omary: Nothing to disclose
Pien Gehrels: Employee: Philips
Jessiva Leschied: Nothing to disclose
Olesya Struk: Employee: Philips
Diana Carver: Nothing to disclose
John R. Scheel: Nothing to disclose
Cassandra L Thiel: Consultant: Philips

**CT's Carbon Footprint: Energy and Disposables**
S. Pruthi[1], C. L. Thiel[2], D. Carver[1], J. R. Scheel[1], R. Omary[1], M. Vigil-Garcia[3], P. Gehrels[3], C. Meijer[3], *O. Struk*[3]; [1]Nashville, TN/US, [2]Madison, WI/US, [3]Amsterdam/NL
*(olesya.struk@philips.com)*

**Purpose or Learning Objective:** To understand the sources of environmental impact of CT scanning within a US-based, adult diagnostic radiology setting.
**Methods or Background:** A life cycle assessment (LCA) was conducted, evaluating the production, use, and end-of-life of CT scanners, including energy consumption, production and use of other capital equipment, disposable supplies, linens, pharmaceuticals, and data storage. A sensitivity analysis was performed assessing the impact of a low-carbon electricity source.
**Results or Findings:** Energy use and disposable supplies were identified as major contributors to CT's greenhouse gas (GHG) emissions, accounting for 42% and 20%, respectively. The production of CTs contributed 17% to GHG emissions. Furthermore, the study revealed a 50% difference in GHG emissions between CT scanners of the same model, suggesting opportunities for optimization. CT scanners were also found to spend between 44-72% of the time in a non-scanning mode. Sensitivity analysis showed that using low-carbon electricity could significantly decrease CT's energy emissions, shifting the major sources of emissions to the production of CT imaging equipment and disposable supplies.
**Conclusion:** Optimizing energy use, minimizing disposable supplies, and ensuring efficient equipment utilization are crucial for reducing CT's environmental impact.
**Limitations:** The study, while providing valuable insights into the environmental impact of CT, has limitations due to its single-center focus; exclusion of mammography, nuclear medicine, and interventional radiology; a one-month data collection period; and some data and modeling limitations.
**Funding for this study:** No external funding was received for this study. Philips and VUMC independently contributed to this study through in-kind labor.
**Ethics committee - additional information:** This study was deemed non-human subjects research.
**Author Disclosures:**
Sumit Pruthi: Nothing to disclose
Marta Vigil-Garcia: Employee: Philips
Cecilia Meijer: Employee: Philips
Reed Omary: Nothing to disclose
Pien Gehrels: Employee: Philips
Olesya Struk: Employee: Philips
Diana Carver: Nothing to disclose
John R. Scheel: Nothing to disclose
Cassandra L Thiel: Consultant: Philips

**The Environmental Cost of MRI: A Life Cycle Assessment**
D. Carver[1], C. L. Thiel[2], J. R. Scheel[1], *R. Omary*[1], M. Vigil-Garcia[3], P. Gehrels[3], S. Thornander[3], C. Meijer[3], O. Struk[3]; [1]Nashville, TN/US, [2]Madison, WI/US, [3]Amsterdam/NL
*(reed.omary@vumc.org)*

**Purpose or Learning Objective:** To understand the sources of environmental impact of MRI within a US based diagnostic radiology department.
**Methods or Background:** A life cycle assessment (LCA) was conducted that evaluated the production, use, and end-of-life of 3 MRI scanners in an adult diagnostic radiology department. Other model inputs included the production and energy use of other capital equipment, disposable supplies, linens, pharmaceuticals, and data storage. A sensitivity analysis assessed the impact of using a low-carbon electricity source.
**Results or Findings:** Energy consumption emerged as the dominant source of MRI's greenhouse gas (GHG) emissions, representing 79% of its total impact. Notably, the 3T MRI demonstrated 1.4 times higher energy use and 1.9 times higher production emissions compared to the 1.5T. Additionally, MRI scanners were found to be in low-power or ready-to-scan mode for 72-75% of the time, indicating potential for energy optimization. Sensitivity analysis revealed that decarbonizing the electricity grid could lead to an 87% reduction in energy-related GHG emissions from MRI. In this scenario, the production of imaging equipment itself would become the largest contributor to MRI's GHG emissions.
**Conclusion:** Improving energy efficiency through measures such as optimizing scan protocols, developing automation of scanner efficiency modes, and transitioning to renewable energy sources are crucial steps in reducing MRI's environmental footprint. If changing the grid is not possible, other opportunities include reducing scan times via AI (e.g. Smart speed) or optimized scheduling.
**Limitations:** The study, while providing valuable insights into the environmental impact of MRI, has limitations due to its: single-center focus; exclusion of mammography, nuclear medicine, and interventional radiology; one-month data collection period; and some data and modeling limitations.

Shifting to a low-carbon electricity grid highlights the additional need to address emissions associated with the production of MRI equipment itself.
**Funding for this study:** No external funding was received for this study. Philips and VUMC independently contributed to this study through in-kind labor.
**Ethics committee - additional information:** This study was deemed non-human subjects research.
**Author Disclosures:**
Marta Vigil-Garcia: Employee: Philips
Cecilia Meijer: Employee: Philips
Reed Omary: Nothing to disclose
Pien Gehrels: Employee: Philips
Olesya Struk: Employee: Philips
Diana Carver: Nothing to disclose
John R. Scheel: Nothing to disclose
Cassandra L. Thiel: Consultant: Philips
Sophie Thornander: Employee: Philips

**AI-Powered MRI: Time, Energy, and Emission Savings for a Greener Future**
*T. Polidori*, M. Zerunian, D. De Santis, F. Pucciarelli, B. Masci, A. Del Gaudio, F. Fanelli, D. Caruso, A. Laghi; Rome/IT
*(tiziano.polidori13@gmail.com)*

**Purpose or Learning Objective:** The study aimed to assess the energy and greenhouse-gas (GHG) emission savings feasible using artificial intelligence (AI) in multi-district MRI-protocols, including lumbar-spine-MRI, cardiac-MRI, and upper abdomen-MRI. We evaluated the impact of AI-algorithms applied on MRI acquisition on scan time reduction, energy consumption, and CO2 emissions per patient, providing insights into the potential benefits of AI in routine clinical practice.
**Methods or Background:** This retrospective study analyzed 148 patients, including 45 upper abdomen-MRI, 53 cardiac-MRI, and 50 lumbar spine-MRI.MRI scans were acquired both without and with AI assistance applied to specific 2D and 3D sequences.The Air Recon-DL (GE Healthcare) was used for T2 and DWI sequences in upper abdomen-MRI, as well as T1, T2, and STIR sequences in lumbar spine-MRI. The Sonic-DL (GE Healthcare) was applied to SSFP sequences specifically for cardiac-MRI.The outcomes measured were time savings per patient, reduced energy consumption (kW/h), and the corresponding reduction in CO2-equivalent emissions.
**Results or Findings:** The application of AI across all three districts studied resulted in significant time savings per patient compared to non-AI protocol ($p<0.01$): 5’11’’ (58%) for upper abdomen-MRI, 1’30’’ (52%) for cardiac-MRI, and 6’ (50%) for lumbar spine-MRI.These time reductions corresponded to significant energy savings of 1.39kW/h, 0.40kW/h, and 1.68kW/h per patient ($p<0.05$), respectively. The equivalent reduction in CO2 emissions was 0.57kg for upper abdomen-MRI, 0.16kg for cardiac-MRI, and0.69 kg for lumbar spine-MRI ($p<0.05$).
**Conclusion:** The implementation of AI in MRI protocols significantly reduces scan time, energy consumption, and GHG emissions, highlighting its potential for enhancing the sustainability of medical imaging practices.Integrating AI into routine clinical protocols could offer considerable environmental benefits, contributing to the reduction of the healthcare sector’s carbon footprint.
**Limitations:** Limitations include a small patient cohort and the use of a single vendor for MRI-protocols.
**Funding for this study:** No-funding was received for this study.
**Ethics committee - additional information:** No
**Author Disclosures:**
Damiano Caruso: Nothing to disclose
Benedetta Masci: Nothing to disclose
Francesco Pucciarelli: Nothing to disclose
Marta Zerunian: Nothing to disclose
Federica Fanelli: Nothing to disclose
Domenico De Santis: Nothing to disclose
Tiziano Polidori: Nothing to disclose
Andrea Laghi: Nothing to disclose
Antonella Del Gaudio: Nothing to disclose

**Automated scout-image based estimation of contrast agent dosing: a deep learning approach**
*R. T. Schirrmeister*, P. S. Friemel, M. Reisert, F. Bamberg, J. Weiß, A. Rau; Freiburg/DE
*(robin.schirrmeister@uniklinik-freiburg.de)*

**Purpose or Learning Objective:** To develop and test a deep learning algorithm for approximation of contrast agent dosage based on CT scout images.
**Methods or Background:** We prospectively enrolled 817 patients undergoing clinically indicated CT imaging, predominantly of the chest and/or abdomen. Patient weight was collected 1) manually and 2) self-reported prior to the examination by study staff. Based on the scout images, we developed an EfficientNet convolutional neural network pipeline to estimate the optimal contrast agent dose based on patient weight and provide a browser-based user interface as a versatile open-source tool to account for different contrast agent compounds We additionally analyzed the body-weight-informative CT features using a weight-conditional variational autoencoder.
**Results or Findings:** The training cohort consisted of 218 chest, 51 abdominal, 511 whole-body, and 37 CT scans of various other anatomical regions. Self-reported patient weight was statistically significantly lower than manual measurements (75.02 kg vs.76.92 kg; $p < 10^{-5}$, Wilcoxon signed-rank test). Our pipeline predicted patient weight with a mean absolute error of 4.74 ± 0.14 kg in 5-fold cross-validation and is publicly available at https://nora-imaging.org/ct-scout-weight/. Interpretability analysis revealed that both larger anatomical shape and higher overall Hounsfield units were predictive of body weight.
**Conclusion:** Our open-source deep learning pipeline allows for automatic estimation of accurate contrast agent dosing based on scout images in routine CT imaging studies. This approach has the potential to streamline contrast agent dosing workflows, improve efficiency, and enhance patient safety by providing quick and accurate weight estimates without additional measurements or reliance on potentially outdated records.
**Limitations:** The model's performance may vary depending on patient positioning and scout image quality and the approach requires validation on larger patient cohorts and other clinical centers.
**Funding for this study:** Funded by an unrestricted research grant from Siemens Healthineers.
**Ethics committee - additional information:** The study was approved by an ethics committee. Written informed consent was obtained from each participant.
**Author Disclosures:**
Paul Simeon Friemel: Nothing to disclose
Marco Reisert: Nothing to disclose
Jakob Weiß: Nothing to disclose
Alexander Rau: Advisory Board: Bayer
Robin Tibor Schirrmeister: Nothing to disclose
Fabian Bamberg: Nothing to disclose

**Cross-Modality Image Conversion from non-contrast Cardiac Magnetic Resonance to contrast-enhanced Computed Tomography Angiography using Diffusion Models**
*E. Almar Munoz*, C. G. Colintenorio, C. Kremser, M. Haltmeier, A. Mayr; Innsbruck/AT
*(enrique.almar@i-med.ac.at)*

**Purpose or Learning Objective:** Transcatheter Aortic Valve Implantation (TAVI) is the preferred treatment for patients with severe aortic stenosis at high to intermediate surgical risk. The gold-standard preoperative imaging modality is contrast-enhanced CTA; however, non-contrast CMR is an alternative for patients with contraindications to contrast agents despite its limitations in detecting calcifications. We propose diffusion models to improve CMR-to-CTA conversion, facilitating comprehensive TAVI planning and predicting valve calcifications without contrast.
**Methods or Background:** Our pipeline integrates Denoising Diffusion Probabilistic Models (DDPMs) and Stochastic Differential Equation (SDE) models. This pipeline was evaluated using an in-house dataset consisting of 39 paired CTA and CMR scans. The image pairs were aligned using rigid registration techniques. To improve the registration process, we utilized aorta segmentation masks obtained using nnUNet for CMR scans and TotalSegmentator for CTA scans.
**Results or Findings:** Regarding the aorta segmentation, we obtained Dice values of 0.987±0.006 for CMR and 0.980±0.005 for CTA. The Dice Score obtained in the rigid registration was above 0.87. Regarding the image conversion, our results demonstrate that the overall synthesized CTA images exhibit high fidelity to their real counterparts, validated by metrics including the Structural Similarity Index Measure (SSIM) and Peak Signal-to-Noise Ratio (PSNR), both exceeding 0.80 and 22, respectively. Focusing on the valve's calcifications, some are accurately converted into CTA-calcified regions but are not always consistent or repeatable.
**Conclusion:** This study highlights the potential of diffusion models in medical imaging, offering a promising solution for patients unable to receive contrast agents, thereby improving the safety and efficacy of TAVI planning.
**Limitations:** Firstly, the model encounters difficulties in replicating small details in the CTA, including calcifications. Secondly, the diffusion models applied are very sensitive to image training; both data modalities must present low noise levels or artifacts.
**Funding for this study:** Fund provided by FWF-DOC-110
**Ethics committee - additional information:** Nothing to declare

**Author Disclosures:**
Markus Haltmeier: Nothing to disclose
Enrique Almar Munoz: Nothing to disclose
Agnes Mayr: Nothing to disclose
Christian Kremser: Nothing to disclose
Carmen Guadalupe Colintenorio: Nothing to disclose

**Diffusion Model for Non-contrast MR to Aid Diagnosis of Focal Liver Lesions: A Multi-Center Study**
*S. Dong*, Z. Shen, F. Yan, R. Li; Shanghai/CN
*(sjdong@sjtu.edu.cn)*

**Purpose or Learning Objective:** To develop a diffusion model for generating virtual dynamic contrast-enhanced MRI (DCE-MRI) images from non-contrast T1-weighted scans and assess its efficacy in FLL diagnosis.
**Methods or Background:** Gadolinium-based contrast agents (GBCAs) in DCE-MRI are crucial for characterizing focal liver lesions (FLLs), but their use increases risks for patients with renal impairment and adds to imaging costs. Virtual contrast-enhanced images from non-contrast T1-weighted scans could reduce these risks and streamline diagnostics. FLLs ≥1 cm, identified through DCE-MRI, were included, with lesion types such as HCC, ICC, liver metastases, cysts, hemangiomas, and FNH. A diffusion model was trained on non-contrast T1-weighted and corresponding multiphase DCE-MRI images (arterial, portal venous, and delayed phases). Training occurred at Center 1 (2018–2023) with a 3:1 split for training and internal testing. External validation used data from three other centers (2018–2024). A diagnostic model for FLLs was also trained on synthetic DCE-MRI images. Normalized mean absolute error (NMAE), peak signal-to-noise ratio (PSNR), and structural similarity index measure (SSIM) were used for evaluation. Three radiologists scored image quality on a three-point scale. The human machine comparison was conducted with six radiologists in different experience.
**Results or Findings:** The study included 1187 patients in the training set (mean age, 51 ±12), with 395 internal (52 ±16) and 347, 271, and 219 external patients (57 ±11, 56 ±12, 58 ±11). The model showed strong similarity between virtual and real DCE-MRI images, with NMAE 0.021–0.038, PSNR 28.9–31.8 dB, and SSIM 0.881–0.927. Diagnostic accuracy was 93% for the internal and 91% for external sets, outperforming three junior radiologists (P < .001) and matching three senior radiologists (P = .19).
**Conclusion:** The diffusion model provides a safe, cost-effective alternative to traditional DCE-MRI, maintaining high diagnostic accuracy for FLLs.
**Limitations:** None reported.
**Funding for this study:** None
**Ethics committee - additional information:** None
**Author Disclosures:**
Ruokun Li: Nothing to disclose
Shunjie Dong: Nothing to disclose
Fuhua Yan: Nothing to disclose
Zhehan Shen: Nothing to disclose

**16:00-17:30** **Research Stage 4**

### Research Presentation Session: Neuro

### RPS 1111

### Neuro interventions and beyond

**Moderator**
D. Ozretić; Zagreb/HR
*(david.ozretic@ck.t-com.hr)*

**Cerebral metabolic rate of oxygen on admission MRI may predict infarct growth in hyperacute ischemic stroke patients treated successfully with thrombectomy: a retrospective observational study**
*A. Bani Sadr*, J. Fournel, M. Hermier, N. Nighoghossian, Y. Berthezene; Lyon/FR
*(apbanisadr@gmail.com)*

**Purpose or Learning Objective:** Despite successful thrombectomy, most stroke patients experience infarct growth which negatively affect functional outcomes. Advances in oxygen metabolism mapping on admission dynamic-susceptibility contrast MRI have shown promise in assessing the viability of diffusion-weighted imaging (DWI) lesions. We aimed to assess the utility of oxygen metabolism mapping at the voxel scale in determining the fate of diffusion-mismatch regions following successful thrombectomy.
**Methods or Background:** We conducted a retrospective analysis of the HIBISCUS-STROKE cohort (NCT: 03149705), a single-center, observational study enrolling patients treated with thrombectomy between 2016 and 2022. Admission DSC-MRI was used to generate time-to-maximum (Tmax), cerebral blood volume (CBV), cerebral metabolic rate of oxygen (CMRO2), and oxygen extraction fraction (OEF) maps. In patients with successful reperfusion (modified Thrombolysis in Cerebral Infarction [mTICI] score ≥2B), Tmax ≥6s voxels excluding DWI abnormalities were analyzed on day 6 T2-fluid attenuated inversion recovery MRI. Semi-quantitative measurements of CMRO2, CBV, and OEF were extracted from regions identified as either necrotic or salvaged at follow-up.
**Results or Findings:** Among the 321 patients enrolled, 134 (41.7%) met inclusion criteria (median age 71.0 years; 58.2% male; median NIHSS score 15.0). In the training cohort, ROC analysis identified optimal thresholds for predicting necrosis: CBV (0.99), CMRO2 (0.64), and OEF (1.59). In the validation cohort, CMRO2 achieved an area under the curve (AUC) of 73.6% (95% confidence interval [CI]: 65.0–82.1), significantly outperforming CBV (AUC: 63.5%, 95% CI: 53.8–73.1; P=0.003) and OEF (AUC: 55.0%, 95% CI: 45.2–64.7; P=0.0005). Multivariable logistic regression revealed that CMRO2 <0.64 was independently associated with necrosis in diffusion-perfusion mismatch regions (OR: 6.0, 95% CI: 3.2–11.6, P<0.0001).
**Conclusion:** In acute stroke patients achieving successful thrombectomy, a CMRO2 < 0.64 in regions of diffusion-perfusion mismatch.
**Limitations:** DSC-MRI dervied oxygen metabolism mapping is not yet validated against PET
**Funding for this study:** This work was supported by the RHU MARVELOUS (ANR-16-RHUS-0009) of Université de Lyon, within the program “Investissements d'Avenir” operated by the French National Research Agency.
**Ethics committee - additional information:** The local ethics committee (Institutional Review Board No: 00009118) approved this study, and all participants or their relatives gave provided informed consent.
**Author Disclosures:**
Yves Berthezene: Nothing to disclose
Marc Hermier: Nothing to disclose
Norbert Nighoghossian: Nothing to disclose
Julien Fournel: Nothing to disclose
Alexandre Bani Sadr: Nothing to disclose

**Why we fail: Factors leading to unsuccessful mechanical thrombectomies**
*R. Bruen*[1], H. Briody[1], S. Singh[1], N. Healy[2], M. T. Crockett[1], J. Müller[1], S. O' Reilly[1], J. Thornton[1], P. Nicholson[1]; [1]Dublin 9/IE, [2]Dublin/IE
*(richardbruen93@gmail.com)*

**Purpose or Learning Objective:** Mechanical thrombectomy (MT) has revolutionized acute ischemic stroke care, demonstrating superior functional outcomes compared to intravenous t-PA alone. A substantial minority of patients fail to achieve successful recanalization. This study analyses the causes of unsuccessful MT in a large tertiary referral centre over an 11-year period.
**Methods or Background:** We retrospectively reviewed a prospective stroke registry at our institution, identifying all patients with acute ischemic stroke who underwent MT between January 2012-2023. Unsuccessful MT was defined as a post-interventional modified Thrombolysis in Cerebral Infarction (mTICI) score < 2b. We collected demographic data, NIHSS, ASPECTS, occlusion location, pre-MT alteplase administration and specific reasons for MT failure, categorizing them into three groups: Target not reached, Target reached but recanalization failed, and non-technical reasons.
**Results or Findings:** Of 2620 MT procedures performed, 259 (9.9%) were unsuccessful. The median patient age was 75 years (IQR 65-83), 48% (n=124) were female and the median NIHSS score was 16 (IQR 10-19). Occlusions were located in the anterior circulation in 98% of cases, with a median ASPECTS of 9. In Category 1 (n=48, 19%), non-reaching of the target was attributed to cervical artery tortuosity (n=23, 48%), challenging aortic arch anatomy (n=7, 14.6%) and inability to traverse a cervical occlusion (n=18, 37.5%). Category 2 failures were the most common (n=146, 56%) and were caused by unsuccessful microcatheter advancement beyond the occlusion (n=8, 5.4%), stent retriever and aspiration catheter failed recanalization (n=39, 26.7%), and spontaneous/iatrogenic re-occlusion (n=99, 67.8%). Category 3 (non-technical failures; n=62, 24%) were less common and were mainly due to patient neurological decline.
**Conclusion:** Unsuccessful mechanical thrombectomy was encountered in 9.9% of cases in our cohort. The most common reason was spontaneous/iatrogenic re-occlusion.
**Limitations:** Study was performed in a single site tertiary referral centre.
**Funding for this study:** None
**Ethics committee - additional information:** Retrospective data. No ethics required.

**Author Disclosures:**
Patrick Nicholson: Nothing to disclose
Richard Bruen: Nothing to disclose
John Thornton: Nothing to disclose
Hayley Briody: Nothing to disclose
Matthew Thomas Crockett: Nothing to disclose
Sean O' Reilly: Nothing to disclose
Nuala Healy: Nothing to disclose
Sneha Singh: Nothing to disclose
Jennifer Müller: Nothing to disclose

**Comparison of Endovascular Thrombectomy Outcomes Between In-Hospital and Out-of-Hospital Stroke**

*S. Singh*, P. Rohan, C. Leneghan, R. Bruen, M. T. Crockett, A. O'Hare, S. Power, J. Thornton, P. Nicholson; Dublin/IE
*(snehasingh2412@gmail.com)*

**Purpose or Learning Objective:** To compare patient characteristics and outcomes following endovascular thrombectomy (EVT) for ischemic stroke between patients experiencing in-hospital stroke (IHS) and out-of-hospital stroke (OHS).
**Methods or Background:** A single-center, retrospective observational cohort study was conducted using data from the institutional EVT database at Beaumont Hospital. Patients were categorized as IHS or OHS, and their baseline characteristics and outcomes were compared.
**Results or Findings:** Of 2619 patients undergoing EVT, 383 (14.6%) experienced IHS (median age 72 years, 57% male) and 2235 (85.4%) experienced OHS (median age 72 years, 54% male). OHS patients had higher pre-stroke modified Rankin Scale (mRS) scores (p<0.0001) and were significantly more likely to receive intravenous thrombolysis (p<0.0001). IHS patients had higher median National Institutes of Health Stroke Scale (NIHSS) scores at day 1 (11 vs. 8, p=0.14) and day 5 (6 vs. 4, p=0.0586), although these differences were not statistically significant. IHS patients also had higher mRS scores at day 30 (p<0.0001) and day 90 (p<0.0001). OHS patients tended to have better Thrombolysis in Cerebral Infarction (TICI) scores post-revascularization (p=0.0535). Treatment was faster in the IHS group: onset to groin puncture (210 vs. 308 minutes, p<0.0001), onset to first reperfusion (243 vs. 341 minutes, p<0.0001). There was no significant difference in groin puncture to first reperfusion time (23 vs. 21 minutes, p=0.1367). IHS patients had a longer median EVT procedure time (37 vs. 32 minutes, p=0.0363).
**Conclusion:** Despite shorter time intervals to intervention, patients with IHS experienced worse functional outcomes after EVT compared to patients with OHS.
**Limitations:** N/A
**Funding for this study:** N/A
**Ethics committee - additional information:** Approved by the local ethics committee at Beaumont Hospital, Dublin, Ireland
**Author Disclosures:**
Patrick Nicholson: Nothing to disclose
Richard Bruen: Nothing to disclose
John Thornton: Nothing to disclose
Alan O'Hare: Nothing to disclose
Matthew Thomas Crockett: Nothing to disclose
Sarah Power: Nothing to disclose
Caoimhe Leneghan: Nothing to disclose
Sneha Singh: Nothing to disclose
Pat Rohan: Nothing to disclose

**How predictive is CT angiography source image ASPECTS (CTA-SI ASPECTS) score on initial CT for futile mechanical thrombectomy? An ongoing study**

*A. Tsaoulia*, M. Mantatzis, L. Kougias, A. Stoforiadi, P. K. Prassopoulos; Thessaloniki/GR
*(kat_tsaoulia@yahoo.gr)*

**Purpose or Learning Objective:** The standard of care for patients with large vessel occlusion (LVO) is mechanical thrombectomy, with or without preceding thrombolysis. Patients with a large infarction core have a higher risk of reperfusion edema or hemorrhage. Alberta stroke program early computed tomography (ASPECTS) score is widely used to evaluate the extent of acute ischemic stroke at the middle cerebral artery territory. An extention of it is CTA-SI ASPECTS (CTAsp), which shows the collateral circulation and discriminates hypoperfused areas. However, it is not established whether this hypoperfusion depicts core or penumbra and if CTAsp is more accurate for patient selection. We aim to find a possible correlation between CTAsp and infarct core
**Methods or Background:** We analyzed the initial CT/CTAs of seven patients with LVO that had complete first pass recanalization and the CTAsp scores were calculated. All CT/CTAs were performed on a 16-slice SIEMENS Emotion-16. A non-contrast CT scan was obtained after thrombectomy, 24 and 48 hours later. These scans were performed on a 128-slice GE OPTIMA. Ten ROIS were applied in each hemisphere in areas defined by ASPECTS, to find density differences.
**Results or Findings:** The patients were divided into two groups based on CTAsp scores; 3 patients had a score of 6-7 and 4 with a score of 8-10. In two patients a discrepancy between ASPECTS and CTAsp was found. The final infarct core was associated with CTAsp in 6/7 patients, while in one, the hypodense area in CTAsp was normalized in follow up.
**Conclusion:** CTAsp proves to be helpful for attempting to predict which patients will have a good outcome after endovascular treatment.
**Limitations:** Small number of cases-preliminary results
**Funding for this study:** None
**Ethics committee - additional information:** The study is based on an imaging score from emergency obtained CT scans.
**Author Disclosures:**
Michalis Mantatzis: Nothing to disclose
Panos K. Prassopoulos: Nothing to disclose
Aikaterini Tsaoulia: Nothing to disclose
Anatoli Stoforiadi: Nothing to disclose
Leonidas Kougias: Nothing to disclose

**Comparison of antithrombogenic coated and uncoated flow-diverters in ruptured and unruptured cerebral aneurysms**

*D. Weiß*, M. Vach, V. L. Ivan, S. Muhammad, B. Hofmann, M. Neyazi, B. Turowski, M. Kaschner; Düsseldorf/DE
*(DanielArvid.Weiss@med.uni-duesseldorf.de)*

**Purpose or Learning Objective:** Flow-diversion has become a key treatment option for complex intracranial aneurysms. Recent advancements include coated flow-diverters (FD), designed to potentially reduce the need for dual antiplatelet therapy thereby removing the associated secondary risks, while maintaining patency and low complication rates. Comparing coated and uncoated FDs may offer insights into long-term outcomes and treatment optimization.
**Methods or Background:** In this retrospective single-center study, we investigated the data of 21 consecutive patients with cerebral aneurysms, treated between 2021 and 2023 with the coated Derivo 2heal Embolization Device (D2H) and the uncoated Derivo Embolization Device (DED) (both Acandis, Pforzheim, Germany). We described the procedure and analyzed clinical and radiological data, along with long-term outcomes after 18 months of follow-up.
**Results or Findings:** Nine patients (42.9%) had incidental, while 12 (57.1%) had symptomatic aneurysms, including ten with WFNS IV subarachnoid hemorrhages. Aneurysm locations included mostly the internal carotid (n=9) and the vertebral artery (n=7). All FDs were successfully deployed: 11 patients received the coated device and 10 the uncoated device. After 18 months, 73.3% patients had favorable outcomes (mRS 0-2). One coated FD occluded asymptomatically after six months, and one uncoated FD occluded immediately but could be recanalized.
**Conclusion:** We observed favorable occlusion rates for both coated and uncoated FDs. The role of dual antiplatelet therapy remains debated. Large multicenter studies are essential to evaluate the patency of coated compared to uncoated FDs and determine whether they can reduce thrombogenicity, potentially allowing for less or no antiplatelet therapy in emergencies.
**Limitations:** Unequal distribution of emergency and elective treatments. Limited by both the number of patients. The study does not address whether monotherapy would be sufficient for drug-coated stents.
**Funding for this study:** None
**Ethics committee - additional information:** Local ethics committee of medical faculty
**Author Disclosures:**
Marius Kaschner: Nothing to disclose
Marius Vach: Nothing to disclose
Milad Neyazi: Nothing to disclose
Sajjad Muhammad: Nothing to disclose
Daniel Weiß: Nothing to disclose
Björn Hofmann: Nothing to disclose
Vivien Lorena Ivan: Nothing to disclose
Bernd Turowski: Nothing to disclose

**Evaluation the Effect of CT Black Blood Technique in Post-treatment Follow-up of Intracranial Aneurysms Treated with Flow-diverting Stents**

*D. Xie*, Z. Lai, H. Ma, R. Xu, J. Wu, J. Zhao; Guangzhou/CN
*(xiedx7@mail.sysu.edu.cn)*

**Purpose or Learning Objective:** To assess the feasibility and effect of CT black blood technique in the post-treatment follow-up of intracranial aneurysms treated with flow-diverting stents.
**Methods or Background:** A retrospective analysis was conducted on 18 patients who underwent treatment with flow-diverting stents for intracranial aneurysms and subsequently underwent digital subtraction angiography (DSA)

follow-up. All patients underwent CT angiography (CTA) examination before DSA. The contrast-enhancement boost technique was employed to process CTA images to obtain CT black blood images. Two radiologists independently rated the image quality and diagnostic confidence for stent-related stenosis on both conventional CTA and CT black blood images using a 4-point scale. The performance of conventional CTA and CT black blood in diagnosing stent-related stenosis was compared, and the examination time and radiation dose of CTA and DSA were recorded and compared.
**Results or Findings:** Subjective ratings of image quality and diagnostic confidence for stent-related stenosis were significantly higher for CT black blood images compared to conventional CTA images (3.94±0.23 vs. 3.06±0.62 and 3.89±0.31 vs. 2.83±0.6, respectively; all $p<0.01$). DSA detected 2 cases of distal stenosis and 5 cases of overall stenosis within the stent. Compared to conventional CTA (sensitivity: 100%, specificity: 36.4%, accuracy: 61.1%), CT black blood demonstrated significantly improved performance in detecting stent-related stenosis, with diagnostic sensitivity, specificity, and accuracy all reaching 100%, and perfect inter-observer agreement (k=1.0). Regarding radiation dose, the average radiation dose for conventional CTA was (67.85±8.31) mGy, whereas the radiation dose required for DSA significantly increased to (516.81±193.83) mGy ($p<0.001$). The examination times for CT and DSA were (3.53±0.74) minutes and (9.57±8.26) minutes, respectively($p<0.01$).
**Conclusion:** The CT black blood technique shows potential as the preferred method for post-treatment follow-up of intracranial aneurysms treated with flow-diverting stents.
**Limitations:** Sample size was relatively small.
**Funding for this study:** None
**Ethics committee - additional information:** None
**Author Disclosures:**
Jiale Wu: Author: author
Dingxiang Xie: Nothing to disclose
Rulin Xu: Nothing to disclose
Zhiman Lai: Nothing to disclose
Hui Ma: Nothing to disclose
Jing Zhao: Author: author

**Impact of Automatically Assessed Collateral Circulation and Infarct Core on Functional Outcome in Acute Ischemic Stroke Patients treated with Endovascular Thrombectomy**
*I. Požar*[1], F. F. Bajrović[2], L. Umek[2], K. Šurlan Popović[2]; [1]Izola/SI, [2]Ljubljana/SI

**Purpose or Learning Objective:** This study aimed to evaluate the predictive value of automatically assessed collateral circulation (CC) and infarct core for functional outcome in acute ischemic stroke (AIS) patients treated with endovascular thrombectomy (EVT).
**Methods or Background:** We conducted a retrospective cohort study of 208 patients with anterior large vessel occlusion treated with EVT. Two AI-powered software were used to automatically assess CC and infarct core. Comparative analyses included patient demographics, clinical and imaging data, and functional outcome. Univariate and multivariable logistic regression analyses were conducted to predict the 90-day functional outcome. A favorable outcome was defined as a modified Rankin scale (mRS) score≤2.
**Results or Findings:** Among the 208 patients, 114 (54.8%) were women and 94 were men, with a mean age of 71.4±13.3 years. Patients with higher collateral score (CS) exhibited lower infarct core volumes ($p<0.001$) and better mRS score at 90 days (p=0.008). Among patients with a favorable outcome, the mean infarct core volume was lower compared to those with poor outcomes (5 mL vs. 8.6 mL, p=0.003). In univariate logistic regression, both infarct core (OR 0.94, p=0.005) and CC (OR 1.84, p=0.014) were predictors of favorable outcome. However, in multivariable models, only infarct core remained a significant independent predictor [AORs of 0.95 (p=0.021) and 0.96 (p=0.039)].
**Conclusion:** Automatically assessed infarct core is a robust predictor of functional outcome in AIS patients post-EVT, while CC's predictive value diminishes when adjusted for infarct core. These findings support the integration of AI-powered evaluations in clinical settings to improve prognosis and treatment strategies for AIS.
**Limitations:** Our study's limitations include using a 40-slice CT scanner, which affects data acquisition speed, particularly in perfusion; non-blinded assessments potentially introducing bias; and reliance on a single CS evaluation, which could lead to inaccurate data.
**Funding for this study:** No funding was received for this study.
**Ethics committee - additional information:** The study was approved by the National Medical Ethics Committee of the Republic of Slovenia (No. 0120-377/2019/4).
**Author Disclosures:**
Lan Umek: Nothing to disclose
Fajko F. Bajrović: Nothing to disclose
Katarina Šurlan Popović: Nothing to disclose
Ingrid Požar: Nothing to disclose

**Higher Relative Brain Age of stroke patients treated with mechanical thrombectomy is associated with poor outcomes**
*M. Guettier*, H. Biegalski, R. Lopes, J. Dumont, W. Gorwood, H. Henon, N. Bricout, G. Kuchcinski, M. Bretzner; Lille/FR
*(melanie.gttr@hotmail.com)*

**Purpose or Learning Objective:** Relative brain age (RBA) is a novel MRI-derived biomarker that quantifies brain health relative to other patients within a cohort. It has previously been associated with poorer outcomes in untreated ischemic stroke patients. However, its impact on poststroke outcomes in patients treated with mechanical thrombectomy (MT) remains unclear.We investigated the clinical determinants of RBA and its association with poststroke outcomes in patients treated with MT
**Methods or Background:** We conducted a retrospective analysis of clinical and imaging data from stroke patients admitted to Lille University Hospital for anterior circulation MT between 2015 and 2020. Admission axial T2 FLAIR MRI images were used to obtain brain parcellation data. A modified brain age prediction pipeline was applied to estimate brain age and derive RBA. Linear regression was used to identify cardiovascular risk factors associated with higher RBA, while logistic regression was employed to assess the impact of RBA on post-stroke outcomes.
**Results or Findings:** A total of 1,296 patients were included, with a mean age of 70 years; 54% were women. Patients with a history of diabetes mellitus and smoking had significantly higher RBA, indicating older-appearing brains (p=0.001 and p=0.010, respectively). Univariate analysis showed that patients with higher RBA, reflecting poorer brain health, were less likely to achieve favorable functional outcomes after stroke (p=0.027). In multivariate analysis, several factors, including age, RBA, admission NIHSS score, intravenous thrombolysis, successful MT, glycemia, time from imaging to MT, and MT duration, were significantly associated with post-stroke outcomes (adjusted odds ratios: 0.48, 0.80, 0.48, 2.04, 5.72, 0.75, 0.81, and 0.69, respectively).
**Conclusion:** Our study highlights the influence of smoking and diabetes on brain aging and the detrimental effects of poor brain health on post-stroke outcomes, building on decades of clinical knowledge.
**Limitations:** Retrospective data
**Funding for this study:** This study has been funded by the ESR/EIBIR 2022 Seed Grant.
**Ethics committee - additional information:** The ethical committee (Comité de protection des personnes Nord-Ouest IV) classified the study as observational on March 9, 2010, and the committee protecting personal information of the patient approved the study by December 21, 2010 (n°10.677). Anonymized data supporting the findings of this study are available from the corresponding author upon reasonable request.
**Author Disclosures:**
William Gorwood: Nothing to disclose
Julien Dumont: Nothing to disclose
Martin Bretzner: Nothing to disclose
Grégory Kuchcinski: Nothing to disclose
Mélanie Guettier: Nothing to disclose
Hugo Biegalski: Nothing to disclose
Hilde Henon: Nothing to disclose
Nicolas Bricout: Nothing to disclose
Renaud Lopes: Nothing to disclose

**Silent Brain Infarcts Post-Interventional Cardiac Catheterization: Insights from High-Resolution DW-MRI in a Randomized Study**
*N. Tan*, X. Zhou; Kunming/CN
*(1041023400@qq.com)*

**Purpose or Learning Objective:** To analyze the incidence and anatomic distribution of acute cerebral embolism and identify associated risk factors in patients undergoing interventional cardiac catheterization.
**Methods or Background:** We conducted a prospective study of patients from two cohorts between January 2023 and April 2024. Diffusion-weighted magnetic resonance imaging (DW-MRI) was used to detect silent brain infarcts (SBIs) preoperatively and within 48 hours postoperatively. For transcatheter aortic valve implantation (TAVI) patients, a cerebral embolic protection device (CEPD, model CEP016F) was randomly employed to minimize the risk of small emboli entering the cerebral vasculature. In atrial fibrillation ablation procedures, some patients underwent Vein of Marshall Ethanol Infusion (VOMEt) to decrease the recurrence of atrial fibrillation and prevent the formation of new embolic events. Independent risk factors were identified through multivariate logistic regression analysis.

**Results or Findings:** Silent brain infarcts were detected in 34 of 48 patients (70.8%) within 48 hours postoperatively. Among the patients who used CEPDs (n = 12), 7 (56.8%) found SBIs. Patients who used CEPDs showed a trend toward smaller infarct volumes ( 5.32 $cm^3$ vs. 8.11 $cm^3$; P = 0.07). In patients who underwent VOMEt procedures (n = 20), 13 (65.0%) developed SBIs; however, neither the incidence nor the volume of SBIs showed significant differences compared to those who did not undergo VOMEt. Multivariate logistic regression analysis identified operation time as an independent positive predictor of SBIs (odds ratios, 6.190 and 13.564; both P < .001).
**Conclusion:** Silent brain infarcts were detected in 70.8% of patients undergoing interventional cardiac catheterization, predominantly affecting the parietal lobes. These findings highlight the importance of procedural optimization to reduce cerebral embolic risk.
**Limitations:** The study was with a relatively small simple size, and conducted at a single center.
**Funding for this study:** No
**Ethics committee - additional information:** Kunming Yan'an Hospital Ethics Committee
**Author Disclosures:**
Na Tan: Nothing to disclose
Xinyan Zhou: Nothing to disclose

**Prognostic Value of CT Contrast Staining after Endovascular Therapy in Basilar Artery Occlusion Stroke**
P. Reidler, O. Öcal, J. Ricke, D. Puhr-Westerheide, *M. P. Fabritius*; Munich/DE

**Purpose or Learning Objective:** Contrast staining (CS) signifies prolonged tissue absorption of iodinated contrast media following endovascular therapy (EVT) for large vessel occlusion stroke, indicating blood-brain barrier disruption. With EVT becoming the standard for treating basilar artery occlusion (BAO) stroke, our study aimed to determine the prognostic significance of post-interventional CS in BAO stroke cases.
**Methods or Background:** We included BAO patients who received postinterventional noncontrast CT within 24h after EVT. Expert radiologists confirmed the presence of CS on CT and its volume was quantified. Functional outcomes were assessed on the modified Rankin (mRS) scale at 90 days and unfavorable outcome was defined as mRS ≥4. A multivariable LASSO-penalized logistic regression analysis was used to determine association of CS and other clinical and imaging parameters with functional outcome.
**Results or Findings:** 42 patients fulfilled the inclusion criteria (15 female, 35,7%). CS on postinterventional CT was present in 18 patients (42.9%) with a median [interquartile range / IQR] volume of 7.9 mL [3.7-14.6]. Patients with CS had a worse outcome with higher mRS after 90 days (median [IQR]: 6 [4-6] vs. 2 [1-4], p<0.001). Multivariable LASSO analysis revealed significant and strongest association of CS with clinical outcome.
**Conclusion:** CS on postinterventional CT after EVT for BAO is an independent predictor of unfavorable functional outcome, outperforming other pre- and post-interventional imaging parameters.
**Limitations:** Retrospective, small sample size
**Funding for this study:** None
**Ethics committee - additional information:** LMU Munich
**Author Disclosures:**
Matthias Philipp Fabritius: Nothing to disclose
Daniel Puhr-Westerheide: Nothing to disclose
Osman Öcal: Nothing to disclose
Paul Reidler: Nothing to disclose
Jens Ricke: Nothing to disclose

**EVT in young adults with stroke: Outcomes and procedural considerations**
*C. Leneghan*[1], D. Leneghan[2], P. Nicholson[1], S. Singh[1], J. Thornton[1], M. T. Crockett[1], A. O'Hare[1], J. Müller[1], P. Fearon[1]; [1]Dublin/IE, [2]Lucerne/CH
*(leneghac@tcd.ie)*

**Purpose or Learning Objective:** This study compares clinical presentations, outcomes, and procedural aspects of endovascular thrombectomy (EVT) in young adults (18-49 years) versus older adults (≥50 years) with acute ischemic stroke due to intracranial artery occlusion (IAO).
**Methods or Background:** We analysed data from a prospectively maintained registry of patients treated with EVT in a large tertiary referral centre between 2012 and 2022. Young (18-49) and older (≥50) patients were compared regarding baseline characteristics, 30- and 90-day modified Rankin Scale (mRS), 24-hour and 5-day NIHSS, reperfusion success (mTICI), post-EVT ASPECTS, and 90-day mortality.
**Results or Findings:** Of 2201 patients, 243 (11%) were young adults. Young patients presented with lower median pre-treatment ASPECTS (8 vs. 9, p=0.0008). Older patients had higher NIHSS at 24 hours (OR 1.63, 95% CI 1.07-2.46), but no difference was observed at 5 days. Functional dependence (mRS ≥3) was more frequent in older patients at 30 (OR 1.92, 95% CI 1.45-2.53) and 90 days (OR 2.22, 95% CI 1.66-2.97). Mortality at 90 days was lower in younger patients (OR 0.45, 95% CI 0.28-0.70).
**Conclusion:** Younger patients undergoing EVT for IAO have lower initial ASPECTS but demonstrate faster neurological recovery and improved functional outcomes compared to older patients. This suggests that aggressive EVT is warranted even in younger patients with lower ASPECTS.
**Limitations:** Retrospective design, single-centre study.
**Funding for this study:** No specific funding was received for this study.
**Ethics committee - additional information:** The study was approved by the local clinical audit committee.
**Author Disclosures:**
Patricia Fearon: Nothing to disclose
Patrick Nicholson: Nothing to disclose
John Thornton: Nothing to disclose
Darren Leneghan: Nothing to disclose
Alan O'Hare: Nothing to disclose
Matthew Thomas Crockett: Nothing to disclose
Caoimhe Leneghan: Nothing to disclose
Sneha Singh: Nothing to disclose
Jennifer Müller: Nothing to disclose

**Could be venous MT safe and effective for dural sinus thrombosis?**
*M. T. Contaldo*, A. Cervo, A. Macera, C. Rollo, A. Vitiello, G. Pero, G. Schwarz, M. Sessa, M. Piano; Milan/IT
*(maria.contaldo@unimi.it)*

**Purpose or Learning Objective:** Cerebral venous thrombosis (CVT) is a rare cause of stroke, that tends to affect young people and the role of endovascular treatment (EVT) remains debated. This study aims to evaluate the efficacy and safety of mechanical thrombectomy (MT) performed at our center for dural sinus thrombosis.
**Methods or Background:** Within a retrospective observational analysis conducted over a 6-year period, data from 62 patients referred to the stroke unit for CVT were analyzed. Among them, 32 patients, classified with severe CVT, underwent EVT. We assessed safety by examining intraprocedural and periprocedural adverse events (asymptomatic, mild, or severe). Clinical outcomes were evaluated at baseline and discharge. Efficacy was determined by analyzing the recanalization rate (complete, partial, or absent) at the end of the procedure.
**Results or Findings:** A total of 32 patients received EVT, accounting for 36 procedures. EVT with MT was performed within 6 hours from onset in 21 out of 32 cases. In most cases, MT was performed as primary treatment, alongside best medical therapy. An intravenous bolus of heparin was administered in the angio-suite to patients who had not yet received anticoagulants or thrombolytics. Successful recanalization (complete or partial without cortical venous drainage delay) was achieved in 91.3% of procedures. CVT recurrence occurred in 3 out of 36 procedures. Mortality rate was 3.1%.
**Conclusion:** This is one of the largest series of patients treated with MT in combination with best medical therapy, underscoring the favorable safety and efficacy profile of EVT. MT could be a first-line option for multiple dural sinus thrombosis, rapid deterioration, venous hypertension, or anticoagulant failure.
**Limitations:** This study is a single center experience with no control-group and a retrospective design, with a heterogeneous case-by-case patient selection.
**Funding for this study:** No funding was received for this study.
**Ethics committee - additional information:** Not applicable
**Author Disclosures:**
Amedeo Cervo: Nothing to disclose
Maria Teresa Contaldo: Nothing to disclose
Antonio Macera: Nothing to disclose
Alessio Vitiello: Nothing to disclose
Maria Sessa: Nothing to disclose
Guglielmo Pero: Nothing to disclose
Mariangela Piano: Nothing to disclose
Ghil Schwarz: Nothing to disclose
Claudia Rollo: Nothing to disclose

### Friday, February 28

08:00-09:00 **Research Stage 1**

### Research Presentation Session: Oncologic Imaging

### RPS 1216

### New perspectives in breast and gynaecological cancer

**Moderator**
G. Ivanac; Zagreb/HR
*(gordana.augustan@gmail.com)*

### Prognostic role of Whole-body MRI (WB-MRI) in patients with metastatic breast cancer receiving systemic anti-cancer therapy

*C. Pizzi*[1], C. Sattin[1], F. Arnone[1], D. Berloco[1], P. Hoxha[1], P. Summers[1], R. Maggioni[1], A. R. R. Padhani[2], G. Petralia[1]; [1]Milan/IT, [2]Northwood/UK
*(caterina.pizzi@unimi.it)*

**Purpose or Learning Objective:** To investigate the potential of the response assessment category (RAC) from MET-RADS-P guidelines as prognostic biomarker in metastatic breast cancer (MBC) patients.
**Methods or Background:** We enrolled MBC patients who underwent whole-body MRI at baseline and at each time point (every 12 weeks disease until progression) after systemic anti-cancer therapy (SACT). We correlated the maximum RAC at time point 1 (TP1) with overall survival (OS). Patients were divided in two groups: those with a maximum RAC 1-2 (highly likely or likely to be responding, respectively) and those with a maximum RAC 3-4-5 (stable disease, likely or highly likely to be progressing) at TP1. Survival curves were depicted in Kaplan-Meier plots and compared via a log-rank test and hazard ratio (HR) using Cox regression model, with point comparisons of three-year survival and median survival duration, using R.
**Results or Findings:** Out of 45 MBC patients enrolled, a higher OS was observed in patients with a maximum RAC 1-2 (N=18) than in those with a maximum RAC 3-4-5 (N=27) at TP1 (log-rank test p=0.007). Because more than 50% of the maximum RAC 1-2 patients are still living, the median survival duration could not be determined, median survival in the maximum RAC 3-4-5 group was 36 months (upper limit of 95%CI not available). The HR for the maximum RAC 3-4-5 patients was 2.28 (95%CI 1.24 – 3.33). Three years OS was 88.9% for RAC1-2 vs 42.6% for RAC 3-4-5; for a difference of 46.2% (95%CI 12.7%-79.8%, p=0.0068).
**Conclusion:** Our observations support the potential of RAC after TP1 as a prognostic biomarker in MBC patients undergoing SACT.
**Limitations:** Retrospective and monocentric study.
**Funding for this study:** No funding was received for this study.
**Ethics committee - additional information:** Not applicable
**Author Disclosures:**
Roberta Maggioni: Nothing to disclose
Francesca Arnone: Nothing to disclose
Giuseppe Petralia: Nothing to disclose
Caterina Pizzi: Nothing to disclose
Paolo Hoxha: Nothing to disclose
Paul Summers: Nothing to disclose
Anwar R. R Padhani: Nothing to disclose
Caterina Sattin: Nothing to disclose
Donatello Berloco: Nothing to disclose

### Dual-energy CT machine learning model to characterize lymph nodes in patients with breast cancer

*P. Morrone*, C. Zampieri, C. Esposito, E. Barone, I. Capitoni, F. Gentili, G. Bagnacci, S. Guerrini, M. A. Mazzei; Siena/IT
*(p.morrone1@student.unisi.it)*

**Purpose or Learning Objective:** To identify a machine learning (ML) model with morphological and dual-energy (DE) data, to characterize lymph node's (LN) status during breast cancer (BC) staging.
**Methods or Background:** From a cohort of 636 patients who undergone whole-body DE-CT and subsequent surgery with axillary lymphadenectomy between April 2015 to July 2023, 117 patients were included. Exclusion criteria: previous ipsilateral breast or axillary surgery, or chemo-radiotherapy; poor quality CT; lack of anatomopathological data. For the morphological analysis, the main diameter of the neoplasm and location, long and short axis and morphological features (fat hilum, cortical area status, extranodal extension-ENE) of the ipsilateral axillary LNs were recorded. For quantitative analysis regions of interest (ROIs) were placed on the neoplasm and axillary LNs encompassing an area of post-contrast enhancement as large and homogeneous as possible. An attempt was made to place the ROIs on the entire LN excluding the fat hilum and surrounding structures, setting a HU displaying threshold to suppress negative HU values. For each ROI, mean attenuation value at 40 and 70keV, iodine concentration (IC), water concentration (WC) and effective-Z value were recorded.
**Results or Findings:** 116 BC and 375 LNs were analyzed, 180 pathological and 195 non-pathological. On univariate analysis the attenuation (HU) at 40 and 70keV, slope, IC, WC, long and short LNs axis showed statistically significant differences between histologically proven pathological and non-pathological LNs (p<0.001). There were statistically significant differences (p<0.001) according to the cortical status and ENE. The logistic regression-based ML model included IC, short axis, fat hilum, cortical status and ENE; the ROC curve showed an AUC of 0.881, demonstrating good model accuracy.
**Conclusion:** The ML model provides a good discriminatory ability to differentiate pathological from non-pathological axillary LNs in patients with BC.
**Limitations:** Not applicable
**Funding for this study:** No funding was received for this study.
**Ethics committee - additional information:** Waived from our etical committe due to the retrospective nature of this study.
**Author Disclosures:**
Chiara Esposito: Nothing to disclose
Maria Antonietta Mazzei: Nothing to disclose
Iacopo Capitoni: Nothing to disclose
Paola Morrone: Nothing to disclose
Francesco Gentili: Nothing to disclose
Cecilia Zampieri: Nothing to disclose
Elisa Barone: Nothing to disclose
Susanna Guerrini: Nothing to disclose
Giulio Bagnacci: Nothing to disclose

### Associations between ADC histogram analysis values and tumor-micro milieu in uterine cervical cancer

*H-J. Meyer*[1], A-K. Höhn[1], A. Surov[2]; [1]Leipzig/DE, [2]Minden/DE
*(jonas90.meyer@web.de)*

**Purpose or Learning Objective:** The complex interactions of the tumor micromilieu could be reflected by diffusion-weighted imaging (DWI) derived from the magnetic resonance imaging (MRI). The present study investigated the association between apparent diffusion coefficient (ADC) values and histopathological features in uterine cervical cancer.
**Methods or Background:** This retrospective study used the prebiopsy MRI to analyze histogram ADC-parameters. The biopsy specimens were stained for Ki 67, E-cadherine, vimentin and tumor-infiltrating lymphocytes (TIL, all CD45 positive cells). Tumor-stroma ratio (TSR) was calculated on routine H&E specimen. Spearman's correlation analysis and receiver-operating characteristics curves were used as statistical analyses.
**Results or Findings:** The patient sample comprised 70 female patients (age range 32-79 years; mean age 55.4 years) with squamous cell cervical carcinoma. The interreader agreement was high ranging from intraclass coefficient (ICC)=0.71 for entropy to ICC=0.96 for ADCmedian. Several ADC-histogram parameters correlated strongly with the TSR. The highest correlation coefficient achieved p10 (r=-0.81, p<0.0001). ADCmean can predict tumors with high TSR, AUC: 0.91, sensitivity: 0.91 (95%CI 0.77;0.96), specificity: 0.91 (95%CI 0.78;0.97). Also, several ADC-histogram parameters correlated slightly with the proliferation index Ki 67. No associations were found with TIL, E-Cadherin and vimentin. In well and moderately differentiated cancers, ADC histogram values showed stronger correlations with Ki 67 and TSR than in poorly differentiated tumors.
**Conclusion:** ADC values are strongly associated with tumor-stroma ratio. ADC mean can be used for prediction of tumors with high TSR. Associations between histopathology and ADC values depend on tumor differentiation. ADC values show only weak associations with Ki 67 and none with TIL, vimentin and E-Cadherin.
**Limitations:** First, it is a retrospective study with known inherent bias. Second, the patient sample is comprised from a single center.
**Funding for this study:** None
**Ethics committee - additional information:** Ethics commitee University of Leipzig (Ethical code: 012/13–28012013)
**Author Disclosures:**
Alexey Surov: Nothing to disclose
Anne-Kathrin Höhn: Nothing to disclose
Hans-Jonas Meyer: Nothing to disclose

**Low-dose pre-operative CT of ovarian tumor with artificial intelligence iterative reconstruction for diagnosing peritoneal invasion**

*X. Cai*[1], J. Han[2], G. Zhang[2], F. Yang[1], Y. Wang[1], J. Liu[1], R. Li[1]; [1]Shijiazhuang/CN, [2]Shanghai/CN

**Purpose or Learning Objective:** To test the feasibility of low-dose abdominopelvic CT with an artificial intelligence iterative reconstruction (AIIR) for diagnosing peritoneal invasion in pre-operative imaging of ovarian tumor.
**Methods or Background:** In this prospective study, 88 patients with pathology-confirmed ovarian tumors were enrolled, where the routine-dose CT scan at portal venous phase (120 kV/ref. 200 mAs) was followed immediately with a low-dose scan (120 kV/ref. 40 mAs). Images at routine dose were reconstructed with hybrid iterative reconstruction (HIR) and images at low dose were reconstructed with AIIR. Two radiologists independently diagnosed the peritoneal invasion using a 5-point confidence scale (1: definitely absent, 5: definitely present). In case of disagreement, the consensus was obtained through a third radiologist. The diagnostic performance was assessed using receiver operating characteristic (ROC) analysis with pathological results serving as the reference. The inter-observer agreement was assessed by Cohen's Kappa test.
**Results or Findings:** The 88 patients consisted of 37 patients with benign/borderline ovarian tumors and 51 patients with ovarian carcinomas. The effective dose of low-dose CT at portal venous phase was 79.8% lower than that of routine-dose scan (2.64 ± 0.46 mSv vs. 13.04 ± 2.25 mSv, $p < 0.001$). In diagnosing peritoneal invasion, the area under the ROC curve (AUC) of low-dose AIIR and routine-dose HIR images was 0.961 and 0.960, respectively ($p = 0.734$). The sensitivity, specificity, and accuracy were 86.1%, 92.3%, and 89.8%, respectively, for low-dose AIIR images, and 86.1%, 90.4%, and 88.6%, respectively, for routine-dose HIR images. The interobserver agreement was good for diagnosing peritoneal invasion ($\kappa = 0.694$).
**Conclusion:** In low-dose pre-operative CT of ovarian tumor with 80% dose reduction, AIIR delivers similar diagnostic accuracy for peritoneal invasion as compared to routine abdominopelvic CT.
**Limitations:** Not applicable.
**Funding for this study:** No funding was received for this study.
**Ethics committee - additional information:** The ethics committee notification can be found under the number 2024KS138.
**Author Disclosures:**
Jing Liu: Nothing to disclose
Ruxun Li: Nothing to disclose
Guozhi Zhang: Nothing to disclose
Yaning Wang: Nothing to disclose
Xiaojia Cai: Nothing to disclose
Fan Yang: Nothing to disclose
Jintao Han: Nothing to disclose

**Developing a machine learning model for the differentiation of uterine leiomyosarcoma from leiomyomas using clinical and MRI radiomics features**

*K. Shapriya*, A. Jackson, X. Li, S. Das, N. Bharwani, A. G. Rockall; London/UK
*(kavita.shapriya@nhs.net)*

**Purpose or Learning Objective:** Preoperative differentiation between leiomyosarcoma (LMS) and atypical benign leiomyoma (LM) is diagnostically challenging. This study aims to develop and validate a machine learning (ML) model using MRI-based clinical and radiomic features to distinguish LMS from LM.
**Methods or Background:** This retrospective study included 214 patients with atypical myometrial lesions who underwent surgery between 2013 and 2023. All subjects had preoperative full blood count (FBC) and MR imaging. Among 214 cases, 193 were LM and 21 were LMS. T2-weighted sagittal MRI sequences were manually segmented then optimized using nonuniformity correction method to ensure image stability. 4114 radiomic features were extracted using TexLab (version 2) and IBSI compliant MATLAB™ software. These radiomic features and 11 clinical variables (including age and FBC) were incorporated into several ML models, with the dependent variable being LMS (binary). Data was split 70:30 into training and testing sets. To address data imbalance, an ensemble classification model was employed with unequal classification costs, penalizing the misclassification of LMS. The model's performance was evaluated using area under the curve (AUC), sensitivity, specificity, accuracy, F1 score, and confusion matrix.
**Results or Findings:** The final ensemble model included four clinical and six radiomic features. The test dataset demonstrated a sensitivity, specificity, accuracy and AUC of 1, 0.8, 0.84 and 0.90, and F1 score of 0.50.
**Conclusion:** This study presents a promising ML model for preoperative differentiation of LMS from LM, achieving high accuracy (84%). As sarcoma subjects are uncommon, the ML model was developed to take data imbalance into account. High sensitivity was achieved, but with some loss of specificity. Future research will focus on validating this model using larger datasets to enhance its reliability and clinical application.
**Limitations:** None
**Funding for this study:** No funding
**Ethics committee - additional information:** HRA and Health and Care Research Wales (HCRW)
Reference: 20/HRA/4925
**Author Disclosures:**
Xingfeng Li: Nothing to disclose
Kavita Shapriya: Nothing to disclose
Andrea Grace Rockall: Nothing to disclose
Saranya Das: Nothing to disclose
Alastair Jackson: Nothing to disclose
Nishat Bharwani: Nothing to disclose

**08:00-09:00** **Research Stage 2**

### Research Presentation Session: Musculoskeletal

### RPS 1210

### Imaging of body composition

**Moderator**
G. Guglielmi; Foggia/IT
*(giuseppe.guglielmi@unifg.it)*

**Clinical validation of a deep learning based automated HUAC analysis for improved sarcopenia assessment**

V. K. Venugopal[1], V. Rengan[2], *S. Ingole*[1]; [1]New Delhi/IN, [2]Chennai/IN
*(sarang.ingole@carpl.ai)*

**Purpose or Learning Objective:** To assess the validity and reliability of an automated sarcopenia estimation approach using a deep-learning based ensemble psoas segmentation and Hounsfield Unit Average Calculation (HUAC) model in comparison to traditional manual measurements.
**Methods or Background:** This study retrospectively analyzed 149 CT scans, comparing sarcopenia assessments between manual HUAC measurements and those derived from an automated TransUNet-based system. The AI model combined convolutional neural networks with Transformer blocks to enhance feature extraction and contextual understanding of muscle tissue, crucial for precise sarcopenia evaluation. The HUAC was calculated by measuring the area and mean Hounsfield Units (HU) of the left and right psoas muscles at the L3 vertebra level. Statistical analysis included mean, standard deviation, correlation, paired t-tests, Bland-Altman plots, and advanced validation metrics such as Intersection over Union (IoU) and Dice coefficient to evaluate the model's segmentation accuracy.
**Results or Findings:** The AI model produced a mean HUAC of 19.66, slightly higher than the 18.03 from manual assessments, with corresponding standard deviations of 4.27 and 4.54, respectively. The correlation coefficient of 0.78 indicated strong agreement between the two methods. The model achieved an IoU of 90% and a Dice coefficient of 0.90, demonstrating high precision in muscle segmentation. The systematic bias observed (mean difference of -1.63 HUAC) highlights areas for further calibration of the AI model.
**Conclusion:** The integration of AI in sarcopenia assessment through HUAC calculations offers a promising alternative to manual measurements, providing speed, reproducibility, and precision. Despite some variance, the AI method aligns closely with traditional approaches, suggesting that with further refinement, it could become a standard tool in clinical settings.
**Limitations:** Small sample set
**Funding for this study:** Nil
**Ethics committee - additional information:** IRB Waiver
**Author Disclosures:**
Sarang Ingole: Nothing to disclose
Vasantha Kumar Venugopal: Consultant: Carpl.ai
Vinayak Rengan: Founder: Curium life technologies

**Qualitative and quantitative CT evaluation of abdominal fat and muscle tissue in patients with ankylosing spondylitis and investigation of their possible effects on biological agent treatment response**

N. Kaştan, *I. Erdem Toslak*, S. Bakırcı, A. Yavuz; Antalya/TR
*(driclalerdem@yahoo.com)*

**Purpose or Learning Objective:** Ankylosing spondylitis (AS) is a chronic inflammatory disease affecting the axial skeleton. Inflammatory cytokines like TNFalpha and interleukins increase in AS, along with adipose tissue and muscle catabolism. Sarcopenia in AS correlates with higher inflammation, greater disease activity, and reduced muscle performance. We hypothesize that the sarcopenia index and quantitative measures of muscle and fatty tissues may relate to the response to biological agent treatment
**Methods or Background:** This retrospective study involved 62 adults diagnosed with AS who underwent CT at the L2 vertebra level before any treatment and received biological agent therapy. CT measurements included visceral and subcutaneous abdominal adipose tissue cross-sectional area (VAT cm², SAT cm²), total abdominal muscle area (TAMA), psoas muscle volume (PsoA), sarcopenia index (SMI), visceral and subcutaneous abdominal adipose tissue attenuation (VAT HU, SAT HU), and psoas muscle attenuation (Pso HU). BASDAI score changes were assessed at the first post-treatment visit to evaluate disease activity. Comparisons and correlations were performed between SMI, adipose and muscle tissue measurements, and clinical parameters.
**Results or Findings:** TAMA, PsoA, and SMI values were significantly higher in patients with full recovery compared to those with partial recovery (p<0.05). However, VAT and SAT (cm²), VAT and SAT (HU), and Pso HU did not significantly affect recovery (p>0.05). This study demonstrates that SMI, TAMA, and psoas muscle volume can serve as prognostic markers for biological treatment response in AS patients.
**Conclusion:** Our study is an example of opportunistic-quantitative imaging methods and it has been shown that SMI value, TAMA and psoas muscle volume value can be used as prognostic markers in response to biological treatment in AS patients.
**Limitations:** Retrospective design and small sample size were the limitations of our study.
**Funding for this study:** No external funding.
**Ethics committee - additional information:** Local IRB
**Author Disclosures:**
Iclal Erdem Toslak: Nothing to disclose
Alpaslan Yavuz: Nothing to disclose
Nazmi Kaştan: Nothing to disclose
Sibel Bakırcı: Nothing to disclose

### Opportunistic Osteoporosis Assessment from Routine CT - Effect of Intravenous Contrast Agents on Absolute Values, T-Scores, and Derived Classifications in Single- and Dual-Energy CT

L. D. Grünewald, V. Koch, S. Mahmoudi, J-E. Scholtz, *J. Gotta*, S. Martin, C. Booz, I. Yel, T. Vogl; Frankfurt/DE

**Purpose or Learning Objective:** To evaluate the impact of intravenous contrast agents on osteoporosis assessment via routine CT in arterial and venous phases and identify mitigation strategies using dual-energy CT (DECT).
**Methods or Background:** 288 patients (154 men, 134 women; median age 62 years) who underwent abdominal DECT scans in non-contrast, late-arterial, and portal venous phases between January 2018 and December 2023 were retrospectively analyzed. Trabecular HU values were measured in all phases, including 90kV and 150kV DECT series, using automatic segmentation. T-scores were calculated to classify patients as osteoporotic, osteopenic, or normal. Changes in HU values, T-scores, and classifications due to contrast were compared to non-contrast images, with effects quantified using Cohen's d.
**Results or Findings:** Median trabecular HU at L1 was 147 (IQR 116–185). Contrast in late arterial and portal venous phases increased HU values by +14.4 (+11.2%) and +25.7 (+20.7%), respectively. Using 150kV DECT reduced these changes to -20.5 (-12.2%) for arterial and -23.15 (-12.6%) for venous phases. Cohen's d was lowest for normal arterial phase (+0.55) and highest for 90kV arterial phase (+1.9). Based on T-scores, 120 patients were classified as healthy, 108 as osteopenic, and 60 as osteoporotic. The lowest number of reclassifications occurred in arterial (n=92) and venous (n=104) phases. For arterial phase, 44 patients shifted from osteoporosis to osteopenia; for venous phase, 52 shifted similarly. High-kV acquisition reduced these reclassifications (n=24 arterial, n=32 venous) but increased shifts from healthy to osteopenia.
**Conclusion:** Intravenous contrast significantly affects HU-based osteoporosis assessment, leading to reclassifications, especially from osteopenia to healthy. Using 150kV DECT can partially reduce these reclassifications, though it may incorrectly shift healthy cases toward osteopenia.
**Limitations:** Modifying kV settings is not immediately possible without dedicated equipment
**Funding for this study:** No funding was received for this study
**Ethics committee - additional information:** Consent waived due to retrospective nature
**Author Disclosures:**
Simon Martin: Nothing to disclose
Christian Booz: Nothing to disclose
Ibrahim Yel: Nothing to disclose
Thomas Vogl: Nothing to disclose
Jan-Erik Scholtz: Nothing to disclose
Vitali Koch: Nothing to disclose
Scherwin Mahmoudi: Nothing to disclose
Leon David Grünewald: Nothing to disclose
Jennifer Gotta: Nothing to disclose

### Femoral osteoporosis prediction model using autosegmentation and machine learning analysis with PyRadiomics on abdomen-pelvic computed tomography (CT)

*H. Ha*[1], H. Lim[2], M. Park[1]; [1]Anyang-Si/KR, [2]Seoul/KR
*(ha.hongil@gmail.com)*

**Purpose or Learning Objective:** This study aimed to assess the diagnostic performance of osteoporosis prediction by the combination of autosegmentation of the proximal femur and machine learning analysis with a reference standard of dual-energy X-ray absorptiometry (DXA)
**Methods or Background:** Abdomen-pelvic CT scans were retrospectively analyzed from 1,122 patients who received both DXA and abdomen-pelvic computed tomography (APCT) scan from January 2018 to December 2020. The study cohort consisted of a training cohort and a temporal validation cohort. The left proximal femur was automatically segmented, and a prediction model was built by machine-learning analysis using a random forest (RF) analysis and 854 PyRadiomics features. The technical success rate of autosegmentation, diagnostic test, area under the receiver operator characteristics curve (AUC), and precision recall curve (AUC-PR) analysis were used to analyze the training and validation cohorts.
**Results or Findings:** The osteoporosis prevalence of the training and validation cohorts was 24.5%, and 10.3%, respectively. The technical success rate of autosegmentation of the proximal femur was 99.7%. In the diagnostic test, the training and validation cohorts showed 78.4% vs. 63.3% sensitivity, 89.4% vs. 98.1% specificity. The prediction performance to identify osteoporosis within the groups used for training and validation cohort was high and the AUC and AUC-PR to forecast the occurrence of osteoporosis within the training and validation cohorts were 90.8% [95% confidence interval (CI), 88.4–93.2%] vs. 78.0% (95% CI, 76.0–79.9%) and 94.6% (95% CI, 89.3–99.8%) vs. 88.8% (95% CI, 86.2–91.5%), respectively.
**Conclusion:** The osteoporosis prediction model using autosegmentation of proximal femur and machine-learning analysis with PyRadiomics features on APCT showed excellent diagnostic feasibility and technical success.
**Limitations:** The limitation of this study was that there was an imbalance in the sex ratio of osteoporosis patients and that this was a single-center study.
**Funding for this study:** This work was supported by the Central Medical Service (CMS) Research Fund. The specific grant number was not assigned by the company or funder (Central Medical Service Company, Ltd., Seoul, Korea).
**Ethics committee - additional information:** The study was approved by the institutional review board of Hallym University Sacred Heart Hospital (No. HALLYM 2020-12-015), and the need for informed consent was waived due to the nature of the retrospective analysis.
**Author Disclosures:**
Hongil Ha: Nothing to disclose
Minsu Park: Nothing to disclose
Hyunkyung Lim: Nothing to disclose

### Radiomic Analysis of Thigh Fat Fraction Maps to Identify Patterns in Neuromuscular Disorders

*G. Vignati*, M. Moscatelli, R. Fabrizio, R. Pascuzzo, C. Foschini, F. Doniselli, D. Aquino, F. Mazzi, L. M. Sconfienza; Milan/IT
*(giacomo.vignati@unimi.it)*

**Purpose or Learning Objective:** To analyze radiomics features extracted from thigh fat fraction (FF) maps in order to identify common patterns in neuromuscular disorders across different patients.
**Methods or Background:** Radiomics features of the classes "shape", "first-order", and "gray-level co-occurrence matrix" (GLCM) were extracted from the thigh FF maps of all patients for each of the 13 VOIs using PyRadiomics, with a fixed bin size of 32. A preliminary feature selection step was necessary due to the large number of features extracted (n=1305) relative to the limited number of patients (n=25). After feature selection, the sparse K-means clustering algorithm was applied: it is a clustering approach that identifies relevant features while performing clustering. Finally, Uniform Manifold Approximation and Projection (UMAP) was used to visualize the selected features and statistical analyses were done with R (version 4.3.1) using the caret, sparcl, and umap packages.

**Results or Findings:** The sparse K-means algorithm identified two clusters of 14 and 11 patients, respectively, based on 60 selected radiomic features from 8 muscles. Clinical diagnosis of patients affected by neuromuscolar disorders is compared with cluster assignment and distinctive features were observed between genetic and inflammatory/autoimmune myopathies.
**Conclusion:** This study successfully utilized radiomics features from thigh fat fraction (FF) maps to identify distinct patterns in neuromuscolar disorders across different patients suggesting that radiomic analysis could be a valuable tool for understanding and classifying muscle disorders in clinical settings.
**Limitations:** Limitations of this study are the small sample size (only 25 patients), the large number of initial radiomic features (n=1305) and the feature selection process, which could introduce bias. The study also relied on manually segmented regions of interest (VOIs), which may introduce variability in the analysis
**Funding for this study:** None
**Ethics committee - additional information:** None
**Author Disclosures:**
Domenico Aquino: Nothing to disclose
Marco Moscatelli: Nothing to disclose
Fabio Doniselli: Nothing to disclose
Federica Mazzi: Nothing to disclose
Giacomo Vignati: Nothing to disclose
Luca Maria Sconfienza: Nothing to disclose
Chiara Foschini: Nothing to disclose
Riccardo Pascuzzo: Nothing to disclose
Renato Fabrizio: Nothing to disclose

**Multiparametric MRI at 3 and 7 T for characterization of skeletal muscle pathology in patients with filamin-C, desmin and LBD3 mutations**
*C. S. Mathy*, L. V. Gast, T. Gerhalter, M. Türk, T. Bäuerle, A. Doerfler, M. Uder, A. M. Nagel, R. Schröder; Erlangen/DE

**Purpose or Learning Objective:** To characterize patterns of skeletal muscle changes in myofibrillar myopathy (MFM), a group of rare neuromuscular diseases with desmin-positive aggregates and myofibrillar degeneration, using multiparametric MRI.
**Methods or Background:** Less affected lower leg of nine patients with genetically confirmed MFM due to FLNC (n=5), DES (n=2) and LDB3 (n=2) mutations (50.9±8.6 years, 6m, 3f) and 10 healthy controls (50.0±11.0 years, 6m, 4f) were examined. 1H-MRI at 3 T included T1-weighted and T2-weigthed STIR for qualitative assessment of fatty replacement/edema, Dixon-type sequence for proton-density fat fraction (PDFF) quantification and diffusion-tensor imaging (DTI) for characterization of (micro-)structural changes. 39K/23Na-MRI acquisition-weighted Stack-of-Stars sequences at 7 T allowed after partial-volume and relaxation correction quantification of apparent tissue sodium/potassium concentrations (aTSC/aTPC).
**Results or Findings:** Muscular fatty replacement and edema-like alterations were highly variant intermuscular and interindividual. 35/63 of elevated muscle compartments of patients with MFM were highly fatty replaced (PDFF >50%). Calculated apparent diffusion coefficients (ADCs) from DTI were reduced in gastrocnemius lateralis (GL), peroneus (PER) and extensor digitorum longus (EDL) muscles (p = 0.04 – 0.03) when excluding highly fatty replaced muscles, but simulations showed that this behavior could primarily be attributed to increasing PDFF. Fat-corrected aTSC were increased in all muscle regions (mean all muscles: 55.6±16.3 mM vs 23.2±5.5 mM, p <0.001), aTPC decreased in all regions but GL and PER (mean all muscles: 75.4±13.3 mM vs 108.9±9.9 mM, p <0.001).
**Conclusion:** Alterations of 39K/23Na ion homeostasis that go beyond changes caused by fatty-replacement in irreversible disease stages could be proved in patients with MFM. This could form the basis for a novel biomarker for determining early disease extent and disease response to therapies. (Micro-)structural changes were indistinguishable from mere fatty replacement changes.
**Limitations:** Low number of participants (MFM prevalence <1:100.000).
**Funding for this study:** C.S.M. and T.B. were founded by the Deutsche Forschungsgemeinschaft (DFG, German Research Foundation) – 493624887 (Clinician Scientist Program NOTICE). Funding by the DFG is gratefully acknowledged (project 500888779 / RU5534 MR biosignatures at UHF).
**Ethics committee - additional information:** Approved by local ethic comittee of Friedrich-Alexander University Erlangen-Nuremberg
**Author Disclosures:**
Tobias Bäuerle: Nothing to disclose
Teresa Gerhalter: Nothing to disclose
Claudius Sebastian Mathy: Nothing to disclose
Matthias Türk: Nothing to disclose
Michael Uder: Nothing to disclose
Rolf Schröder: Nothing to disclose
Lena V. Gast: Employee: Siemens Healthineers
Armin M. Nagel: Nothing to disclose
Arnd Doerfler: Nothing to disclose

**The Top 100 Most Cited Articles on Musculoskeletal Radiology: A Bibliometric Analysis**
*L. Moore*; Dublin/IE
*(lucymooreart@gmail.com)*

**Purpose or Learning Objective:** To identify and characterize the most influential publications relating to the musculoskeletal system and radiology. The number of citations an article receives is reflective of its impact in the scientific community.
**Methods or Background:** The top 100 most cited articles were identified using the Web of Science database. Data pertaining to the year of publication, publishing journal, journal impact factor, authorship, country of origin and institution were collected.
**Results or Findings:** The number of citations per article for the top 100 list ranged from 149 to 709 (median 208; mean 240). The average number of citations per year, per article, ranged from 5 to 60 (median 12, mean 26). The United States was the most common country of origin (n=74). The Journal with the greatest number of articles was Radiology (n=34). The University of California contributed the most articles (n=11).
**Conclusion:** This study presents a detailed analysis of the top 100 most-cited articles published in musculoskeletal radiology. It provides clinicians and researchers with insight into the current influential research papers in this field and the characteristics of those studies. It also highlights research trends and areas that may benefit from further research.
**Limitations:** The use of citation count is a source of bias; the more time that has passed since the publication of an article, the more likely it is to be cited over time. In order to mitigate this source of bias, the average citation count per year was also used. Some articles may have been inadvertently excluded as a result of search criteria used. In addition, using journal IF from one particular year (2024) does not allow for temporal fluctuations in IF. Furthermore, the potential bias of self-citation was not accounted for in this study.
**Funding for this study:** None.
**Ethics committee - additional information:** This article does not require ethics committee approval.
**Author Disclosures:**
Lucia Moore: Nothing to disclose

**08:00-09:00** **Research Stage 3**

### Research Presentation Session: Physics in Medical Imaging

### RPS 1213

### Evolution of CT: a key to its sustainability

**Moderator**
N. Saltybaeva; Lucerne/CH
*(natalia.saltybaeva@luks.ch)*

**Validation of a novel CBCT reconstruction algorithm for treatment planning and IGRT in neoadjuvant radiotherapy of locally advanced rectal cancer patients**
*M. C. Daniotti*[1], S. Trivellato[2], L. De Sanctis[1], V. Pisoni[2], J. Stancanello[3], J. Mason[3], R. Pellegrini[3], S. Arcangeli[2], E. De Ponti[2]; [1]Milan/IT, [2]Monza/IT, [3]Stockholm/SE
*(martina.daniotti@gmail.com)*

**Purpose or Learning Objective:** A new CBCT reconstruction algorithm based on poli-energetic quantitative (Polyquant) method empowered with a convolutional neural network scatter correction has been recently proposed. This study aimed to validate the use of the Polyquant CBCTs (pCBCTs) for image-guided radiotherapy (IGRT) and planning for locally advanced rectal cancer (LARC).
**Methods or Background:** Translational shifts obtained on all axes with the registration of pCBCTs to CT were compared to the clinical version of Elekta XVI CBCT ones and statistical significance was investigated with the t-test and ANOVA-test. pCBCTs were calibrated with a population-based curve (pop-CC) elaborated coupling pCBCT gray levels to the CT relative electron density (RED) for ten pelvic patients. pop-CC was validated by comparing dose calculations on pCBCT and bulk density pseudo-CT using 1%/3mm local gamma-analysis. Five LARC patients treated on Elekta VersaHD were selected. The RED difference between CT and first session-pCBCT were assessed on a voxel-to-voxel basis, on a contour basis, and on a dosimetric basis using 1%/3mm local gamma-analysis to compare pCBCT and pseudo-CT calculations.

**Results or Findings:** The translational differences between IGRT results of CBCT and pCBCT were always <1mm and not statistically significant. The pop-CC pCBCT calibration resulted in dose calculations comparable with the pseudo-CT ones, with gamma passing rates > 95%. For LARC patients, voxel-to-voxel and structure-based analysis showed no relevant RED discrepancies between pCBCT and CT. Residual RED differences resulted dosimetrically negligible compared with dose distributions calculated on pseudo-CT, with gamma passing rates > 95%.
**Conclusion:** The optimized pCBCTs were successfully RED calibrated and validated for IGRT and planning for LARC radiotherapy. The results suggest that pCBCTs could be exploited in the clinical workflow for adaptive radiotherapy in LARC patients,
**Limitations:** Further investigation for their extended use might still be necessary.
**Funding for this study:** No funding
**Ethics committee - additional information:** Not applicable
**Author Disclosures:**
Jonathan Mason: Employee: Elekta AB
Sara Trivellato: Nothing to disclose
Martina Camilla Daniotti: Nothing to disclose
Stefano Arcangeli: Nothing to disclose
Elena De Ponti: Nothing to disclose
Valerio Pisoni: Nothing to disclose
Joseph Stancanello: Employee: Elekta AB
Lorenzo De Sanctis: Nothing to disclose
Roberto Pellegrini: Employee: Elekta AB

**The impact of detector coverage on motion artefacts in photon-counting CT imaging using a motion phantom**
*E. Verelst*[1], G. Van Gompel[1], D. Crotty[2], H. Linder[3], P. D. Deak[4], J. De Mey[1], N. Buls[1]; [1]Brussels/BE, [2]Cork/IE, [3]Stockholm/SE, [4]Münsingen/CH

**Purpose or Learning Objective:** Reducing motion-induced artefacts is an increasingly important aspect of ultra high-resolution (UHR) photon-counting CT (PCCT). Using a motion-controlled phantom, this study evaluates the benefit of using wider detector coverage in PCCT to reduce motion artefacts.
**Methods or Background:** A tissue-simulating cuboid phantom, 80-mm in length, was mounted onto a custom-built motion-controlled phantom. A 3-mm diameter stent (Superia, Nano-Therapeutics, India) was inserted into a commensurate hole inside the phantom. Programmed to simulate pulsatile motion, helical images were acquired during motion by a prototype Silicon-based PCCT (Si-PCCT) using 40- and 80-mm detector coverage, representing table speeds of 128.6-mm/s and 257.1-mm/s, respectively. Motion-induced image artefacts were evaluated by measuring the volumetric error relative to the nominal phantom volume. A 5-point Likert rating evaluated stent appearance for both detector coverages against a reference static image (1-no similarity, 5-similar). Differences were assessed using a paired sample t-test and Wilcoxon signed-rank test, respectively. P-values < 0.05 indicated statistical significance.
**Results or Findings:** Relative to the nominal phantom volume (1571-mm3), the 80-mm detector coverage statistically significantly reduced volumetric error (mean 81-mm3, SD 18-mm3) compared to the error generated with 40-mm coverage (254-mm3, 48-mm3), p=0.006. Stent appearance under wider detector coverage was likewise assessed to better match the reference image, with average Likert scores for 40 mm and 80mm of 1 [1–1.5] and 4 [3.5–3.75], respectively, p=0.043.
**Conclusion:** In high-resolution PCCT imaging, to combat motion-induced artefacts, it is important to combine wide-detector UHR CT acquisitions with higher table speeds. This study demonstrates the potential of using a prototype wide-coverage Si-PCCT system with fast tables speeds to reduce such artefacts. While this study specifically evaluated pulsatile motion, an 80-mm detector coverage may reduce additional body motion-induced artefacts, such as peristalsis.
**Limitations:** This is an ex-vivo phantom study.
**Funding for this study:** Flemish Research Foundation (FWO), personal grant, nr: 1SH1Z24N.
**Ethics committee - additional information:** Ethical approval was not required for this study
**Author Disclosures:**
Johan De Mey: Nothing to disclose
Emma Verelst: Nothing to disclose
Dominic Crotty: Employee: GE Healthcare
Gert Van Gompel: Nothing to disclose
Paul D. Deak: Employee: GE Healthcare
Nico Buls: Nothing to disclose
Hugo Linder: Employee: GE Healthcare

**3D-printed Anthropomorphic Head Phantom Featuring White and Gray Matter Structures for Evaluating CT Imaging**
K. Mei[1], L. Roshkovan[1], S. Sharma[2], S. Ross[2], J. Woo[1], S. S. Halliburton[3], *L. Liu*[1], R. Thompson[3], P. Noël[1]; [1]Philadelphia, PA/US, [2]Vernon Hills, IL/US, [3]Mayfield Village, OH/US

**Purpose or Learning Objective:** To develop a 3D-printed, patient-specific brain phantom for assessing performance of non-contrast CT head imaging.
**Methods or Background:** Unenhanced T1 MRI scan (best available gold standard) of a healthy brain (21y/o,F) was retrospectively collected and converted into CT Hounsfield unit image to generate a realistic phantom using PixelPrint technique. The brain phantom was created as a 30 mm thickness section including both left and right hemispheres (approximately 157 x 120 mm) at 1:1 scale. Additionally, a separate skull phantom was printed from the same patient images using calcium-doped filament. The brain phantom, with surrounding skull phantom, was scanned with CT at 120 kVp and 24.4 mGy. Images were reconstructed with and without iterative denoising at 0.5 mm pixel spacing. Attenuation values were measured in gray and white matter.
**Results or Findings:** Gray and white matter were clearly distinguishable in an appropriate CT examination window, with and without denoising. Line profile was plotted along the center of the phantom. Realistic CT values of approximately 45 HU for gray matter and 25 HU for white matter were observed. Maximum density observed in the skull reached approximately 750 HU, which was limited by the density of the 3D-printing filament used. Image noise, estimated by standard deviation, ranged between 3 and 4.5 HU across both sets of denoised images.
**Conclusion:** The PixelPrint 3D-printed brain phantom successfully depicts realistic tissue attenuationfor white and gray matter, demonstrating potential as a valuable tool for evaluating CT head imaging performance.
**Limitations:** This study converts MR images to CT numbers and simulates non-contrast CT scans.
**Funding for this study:** This work was partly supported by Canon Medical Systems Corporation (Otawara, Japan).
**Ethics committee - additional information:** University of Pennsylvania
**Author Disclosures:**
Steven Ross: Employee: Canon Medical Research USA
Kai Mei: Nothing to disclose
Peter Noël: Nothing to disclose
Richard Thompson: Employee: Canon Healthcare USA
Leening Liu: Nothing to disclose
Sandra Simon Halliburton: Employee: Canon Healthcare USA
Leonid Roshkovan: Nothing to disclose
Shobhit Sharma: Employee: Canon Medical Research USA
John Woo: Nothing to disclose

**Towards functional lung color K-edge imaging enabled by spectral photon-counting CT in combination with dedicated contrast agents: a phantom study**
*A. J. Gutwinska*[1], D. Rosario[2], A. Pang[2], C. A. Hernandez-Fajardo[1], R. Coulibaly[1], A. Robert[1], S. Rit[1], D. P. Cormode[2], S. A. Si-Mohamed[3]; [1]Lyon/FR, [2]Philadelphia, PA/US, [3]Bron/FR
*(agnieszka.gutwinska@creatis.insa-lyon.fr)*

**Purpose or Learning Objective:** To evaluate the image quality of color K-edge imaging for contrast agents based on 8 different elements using a clinical prototype spectral photon-counting CT (SPCCT).
**Methods or Background:** A SPCCT with a field-of-view of 500mm was used (Philips; Israel). An anthropomorphic thoracic phantom (QRM GmbH) with twelve 1.5mL K-edge solutions (gadolinium-Gd, holmium-Ho, ytterbium-Yb, hafnium-Hf, tantalum-Ta, tungsten-W, gold-Au, bismuth-Bi) ranging from 0 to 2mg/mL was scanned at 120kVp and 50/75/150mAs. Five acquisitions per agent were performed using dedicated energy thresholds. Conventional images in Hounsfield units and color K-edge images in mg/mL were reconstructed with isotropic voxels of 0.7mm3. Noise, mean relative error (MRE) between prepared and measured concentrations, signal-to-noise ratio (SNR) were measured on color K-edge images. Contrast-to-noise ratio (CNR) on conventional and color K-edge images were measured and compared.
**Results or Findings:** Mean noise ranged from 0.04 to 0.13mg/mL among all samples with a lowest value for Gd at 75mAs (0.04±0.01 mg/mL) and highest for Ta at 50mAs (0.13±0.03mg/mL). Overall MRE was 29.7% with higher accuracy for Yb (e.g., 7.0% at 150mAs), and lower for Ta (55.7% at 150mAs). SNR increased as function of concentrations with a factor per mg of 16.7, 14.6, 10.9, 9.5, 9.3, 9.3, 8.8, 4.7 for Gd, Yb, Ho, Hf, Au, W, Ta and Bi, respectively, at 75mAs. CNR in color K-edge images increased as function of concentrations, and were higher in comparison to CNR in conventional images (e.g., 1075%, 957%, 677%, 658%, 601%, 590%, 536%, 411%, for Gd, Au, W, Ta, Yb, Ho, Bi and Hf, respectively, at 75mAs).
**Conclusion:** Image quality of color K-edge imaging in an anthropomorphic thoracic phantom demonstrated high performances for 8 color K-edge agents using SPCCT whilst outperforming sensitivity in comparison to conventional imaging.

Friday

**Limitations:** Phantom study.
**Funding for this study:** The ERC starting Grant "KOLOR SPCCT Imaging" (N°101118079).
**Ethics committee - additional information:** Not applicable.
**Author Disclosures:**
Antoine Robert: Nothing to disclose
Salim Aymeric Si-Mohamed: Nothing to disclose
David Peter Cormode: Nothing to disclose
Christian Alejandro Hernandez-Fajardo: Nothing to disclose
Simon Rit: Nothing to disclose
Agnieszka Joanna Gutwinska: Nothing to disclose
Derick Rosario: Nothing to disclose
Ramata Coulibaly: Nothing to disclose
Amanda Pang: Nothing to disclose

### Task-based image quality evaluation of ultra-high resolution color K-edge imaging enabled by spectral photon-counting CT: a phantom study

*R. Coulibaly*[1], A. Robert[1], A. Houmeau[1], M. N. Antonuccio[2], P. C. Douek[1], S. Rit[1], J. Greffier[3], S. A. Si-Mohamed[1]; [1]Lyon/FR, [2]Paris/FR, [3]Nimes/FR
*(ramata.coulibaly@creatis.insa-lyon.fr)*

**Purpose or Learning Objective:** To evaluate the image quality of color K-edge imaging with a gadolinium agent using spectral photon-counting CT (SPCCT) in a phantom with a mixture of contrast agents.
**Methods or Background:** A clinical prototype SPCCT system (FOV 500mm, Philips; Israel) was used to scan custom-made cylindrical phantom of 27cm (Color iQCT). Three inserts of the phantom were filled up with agents as follows: iodine only, mixture of iodine and gadolinium, gadolinium only. Two configurations were considered, one with 0.5mg/mL of each contrast agent and another with 2mg/mL. For each configuration, two series of nine helical scans (120kVp) were acquired at 75mAs and 150mAs. Spectral K-edge images of gadolinium were obtained by doing material decomposition using 3 basis (water/iodine/gadolinium), using an iterative reconstruction algorithm at 3 levels (iDose 0, 6, 11) were compared between inserts with gadolinium using iQMetrix-CT software.
**Results or Findings:** Despite the presence of iodine, color K-edge imaging enable specific differentiation of the gadolinium, showing a concentration difference of 1.1% between inserts of gadolinium only and mixture (150mAs, i11, 2mg/mL). NPS peak was observed at the same spatial frequency in all configurations (i.e., 0.053±0.018 $mm^{-1}$), whereas the noise magnitude decreased when the dose and the iDose4 levels increased (-19.65±0.01% between iDose0 and iDose11). TTF values at 50% (f50) were similar between inserts with gadolinium only and the mixture, regardless of the dose or iDose levels (e.g., 0.213±0.090 $mm^{-1}$ vs 0.237±0.061 $mm^{-1}$, at 150mAs, i11, 0.5mg/mL). f50 values were improved with increasing dose, iDose levels, and with higher concentrations.
**Conclusion:** Color K-edge imaging of a gadolinium contrast agent using SPCCT demonstrated high spatial resolution and low noise magnitude at low concentrations, even in the presence of iodine.
**Limitations:** Phantom study.
**Funding for this study:** ERC starting Grant "KOLOR SPCCT Imaging" (N°101118079).
**Ethics committee - additional information:** No ethics was required for this phantom study.
**Author Disclosures:**
Antoine Robert: Nothing to disclose
Salim Aymeric Si-Mohamed: Nothing to disclose
Angele Houmeau: Nothing to disclose
Philippe Charles Douek: Nothing to disclose
Simon Rit: Nothing to disclose
Joel Greffier: Nothing to disclose
Ramata Coulibaly: Nothing to disclose
Maria Nicole Antonuccio: Employee: Philips

### X-ray phase contrast imaging, moving beyond traditional X-ray imaging methods: a first pilot in intra-operative specimen imaging

*G. Havariyoun*; London/UK
*(g.havariyoun@nhs.net)*

**Purpose or Learning Objective:** Several surgical procedures benefit from the ability to image resected tissue samples in real time, e.g. to ensure no margin involvement. Micro-CT or tomosynthesis have great potential, but suffer from limited soft-tissue sensitivity of X-rays. X-ray phase contrast imaging (XPCI) provides soft tissue sensitivity and increased contrast through exploitation of phase effects. This work is presented on behalf of the UCL AXIm team.
**Methods or Background:** XPCI was initially restricted to specialized facilities such as synchrotrons. Our group has developed a method that has enabled creation of a pre-commercial prototype compatible with surgical and radiology workflows. This has been used to image >100 breast tissue samples from breast conserving surgery both in vitro and in real time. Images were compared to standard specimen radiography and histopathology. The system also allows higher resolution (~10 micrometre) imaging in slower scans for e.g. digital histology.
**Results or Findings:** System optimization (which also included size reduction) led to clinically acceptable scan times, which were verified by trialling the system in a real intra-operative context. XPCI imaging resulted to sensitivity and specificity values of 83% (95% CI 69-92%) and 83% (95% CI 70-92%), respectively. Standard specimen radiography resulted to sensitivity and specificity values of 32% (95% CI 20-49%) and 86% (95% CI 73-93%), respectively.
**Conclusion:** XPCI has a specificity comparable to standard specimen radiography but a significantly higher sensitivity. This would lead to significant reduction in re-excision rates and in turn a reduction in patient stress, surgical times, healthcare costs and improved cosmetic outcomes.
**Limitations:** Comparisons with standard specimen radiography were made as this is the most commonly used tool in the clinical setting. Comparison with more advanced techniques will be made in the future.
**Funding for this study:** This work is funded by the Wellcome Trust (Grant 200137/Z/15/Z). Alessandro Olivo (AXIm lead) is funded by the Royal Academy of Engineering under their “Chairs in Emerging Technologies” scheme (CiET1819/2/78).
**Ethics committee - additional information:** The Breast Cancer Now Tissue Bank (Approval No. 15/EE/0192) provided the ethically approved samples, the authors thank the patients who have generously consented to donate their tissues which have been utilised in this work.
**Author Disclosures:**
Glafkos Havariyoun: Nothing to disclose

### A workflow to harmonize CT abdomen protocols beyond dose equalization

*J. Vignero*, B. Miseur, J. Binst, H. Bosmans; Leuven/BE
*(janne.vignero@uzleuven.be)*

**Purpose or Learning Objective:** A radiologist had raised concerns about excessive noise in CT abdomen images of scanner A, while another scanner (B) of the same model, using similar protocol settings, produced images of acceptable quality. This discrepancy led to a study aimed at harmonizing protocol settings to ensure consistent image quality across all patient sizes, moving beyond standard dose equalization methods. Currently, protocol adjustments—based on dose level, scan task, and tube current modulation strength—are often made intuitively.
**Methods or Background:** Clinical CT scan data, collected through a dose monitoring platform (DOSE, Qaelum), was analysed using water equivalent diameter (WED), global noise level (GNL), kVp and CTDI. Three phantoms with ellipsoid cross-sections (WED: 22, 33 and 43cm) and iodine/calcium inserts were scanned to map protocol settings to scan parameters (kVp, mAs, CTDI) and GNL for each scanner and phantom. The reconstruction settings were kept fixed.
**Results or Findings:** Initially, scanner A and B produced similar radiation doses for the same WED groups, but scanner A had on average a 13% higher GNL, with a maximum of 18% difference for the smallest WED group. The radiologist could define GNL upper limits for each kVp. Using the phantom scan maps, protocol settings were chosen to achieve the desired image quality. Post-optimization, 87% of scans met the GNL criteria, compared to 70% before. To achieve this, radiation doses were increased with 17%.
**Conclusion:** Identical scanner models and protocol settings do not guarantee consistent image quality. Quality measures should be included in optimization efforts. We present a procedure using new phantoms and dedicated metrics to improve protocol harmonization.
**Limitations:** The pipeline has only be verified on two scanners.
**Funding for this study:** In part funded by the i-Violin project that is co-funded under the EU4Health Programme 2021-2027, grant agreement no. 101056832.
**Ethics committee - additional information:** Retrospective, technical study
**Author Disclosures:**
Hilde Bosmans: Shareholder: Qaelum
Janne Vignero: Nothing to disclose
Joke Binst: Nothing to disclose
Bram Miseur: Nothing to disclose

**Impact of Acquisition Parameters on Quantitative Imaging Using Rapid kVp-Switching Spectral CT**

*O. Sandvold*[1], A. Perkins[2], H. Daerr[3], T. Koehler[3], R. Proksa[1], R. Manjeshwar[2], P. Noël[1]; [1]Philadelphia, PA/US, [2]Cleveland, OH/US, [3]Hamburg/DE

**Purpose or Learning Objective:** To investigate the quantitative effects of varying the ratio of high and low kVp tube voltage durations on spectral CT results with a rapid kVp-switching X-ray tube.
**Methods or Background:** Rapid kVp-switching offers excellent spectral separation, but experimental research on acquisition parameters is limited. This study addresses the gap. A rapid kVp-switching X-ray tube (Philips Healthcare) on a spectral CT bench system was operated at 500 mAs alternating between 140 and 80 kVp. The total integration period (IP) containing high and low kVp IPs was 1 ms. We varied the ratio of 140 kVp duration to total IP time from 0.15 to 0.85 and correspondingly adjusted the low kVp IP time. A 3D-printed plastic phantom (20 cm diameter) was rotated at 1 Hz and contained four tissue-mimicking inserts: iodine (2.0, 5.0 mg/ml), iodine 4.0 mg/ml + human equivalent (HE) blood, and HE blood (Sun Nuclear). We performed reconstruction and two-material decomposition without applying denoising. The noise and contrast-to-noise ratio (CNR) of the known concentration inserts were measured in photoelectric material images. Dose was estimated from the reference detector.
**Results or Findings:** Dose comparisons showed 0.15, 0.25, 0.33, and 0.67 ratio scans used 47%, 51%, 63%, and 91% of the highest dose scan (0.85 ratio). Measured noise values appeared to follow a quadratic trend as the ratio increased, with 0.33 containing the lowest average noise. The 0.33 ratio image dose normalized CNR was approximately 1.24x, 1.14x, 1.11x, and 1.68x greater than the dose normalized CNR in 0.15, 0.25, 0.67, and 0.85 ratio images respectively.
**Conclusion:** Dose normalized CNR depends strongly on the ratio between high and low kV durations. This ratio should be considered for optimizing the spectral acquisition.
**Limitations:** None
**Funding for this study:** None
**Ethics committee - additional information:** Not applicable
**Author Disclosures:**
Heiner Daerr: Employee: Philips Innovative Technologies
Thomas Koehler: Employee: Philips Innovative Technologies
Olivia Sandvold: Nothing to disclose
Peter Noël: Nothing to disclose
Roland Proksa: Nothing to disclose
Ravindra Manjeshwar: Employee: Philips Healthcare
Amy Perkins: Employee: Philips Healthcare

**08:00-09:00** **Research Stage 4**

### Research Presentation Session: Breast

### RPS 1202

### Improving the clinical impact of contrast-enhanced mammography

**Moderator**
R. Alcántara; Barcelona/ES
**Author Disclosures:**
Rodrigo Alcántara: Advisory Board: GE Healthcare; Research Grant/Support: GE Healthcare; Speaker: GE Healthcare, BD, Bayer

**CEM for the assessment of screening recalls: diagnostic performance at three-year follow-up**

*S. Marziali*[1], A. Cozzi[1], M. Fanizza[1], V. Magni[1], L. Menicagli[1], A. Benedek[1], G. Di Giulio[2], F. Sardanelli[1]; [1]Milan/IT, [2]Pavia/IT
*(Sara.Marziali@unimi.it)*

**Purpose or Learning Objective:** To evaluate the diagnostic performance of contrast-enhanced mammography (CEM) for the assessment of screening recalls.
**Methods or Background:** Recalled women were prospectively enrolled at two centres to undergo CEM alongside standard assessment (SA) through additional views, tomosynthesis, and/or ultrasound between January 2019 and July 2021. Exclusion criteria were symptoms, implants, allergy to contrast agents, renal failure, and pregnancy. SA and CEM were independently evaluated by one of six radiologists, who recommended biopsy or ≥3-year follow-up. Diagnostic performance of CEM (low-energy plus recombined images) was calculated considering histopathology for lesions biopsied and/or surgically removed as well as ≥3-year follow-up for both breasts.
**Results or Findings:** Between January 2019 and July 2021, 220 women were enrolled, 207 of them (median age 56.6 years) with 225 suspicious findings, 135 of them referred for biopsy (4 by rCEM alone, 2/4 being one DCIS and one invasive carcinoma). During ≥3-year follow-up, 4 interval cancers were reported: one mucinous invasive carcinoma and one DCIS at the site of previous assessment with biopsy; one DCIS in a different quadrant of the same breast; and one invasive carcinoma NST at the contralateral breast (found at the third year after CEM). No woman was lost at follow-up. The overall CEM performance was: sensitivity 80/84 (95.2%, 95% CI 88.3−98.7%); □p□□ifi□ity 108/123 (87.8%, 95% CI 80.7−90.3%9).
**Conclusion:** The role of CEM in the assessment of recalls is confirmed at over 3-year follow-up in terms of both sensitivity and specificity. The two cases of interval cancers at the site of previous biopsy highlight the need of radiologic-pathologic correlation.
**Limitations:** Limited sample size.
**Funding for this study:** GE Healthcare
**Ethics committee - additional information:** San Raffaele Hospital, Milan, Italy
**Author Disclosures:**
Andrea Cozzi: Nothing to disclose
Sara Marziali: Nothing to disclose
Francesco Sardanelli: Speaker: Bayer AG, Siemens Healthineers Advisory Board: Bayer AG, Bracco imaging, GE healthcare Research/Grant Support: Bayer AG, Bracco imaging, GE healthcare,
Marianna Fanizza: Nothing to disclose
Giuseppe Di Giulio: Nothing to disclose
Veronica Magni: Nothing to disclose
Adrienn Benedek: Nothing to disclose
Laura Menicagli: Nothing to disclose

**COntrast enhanced Mammography in women with previous Breast cancer Operated with conserving surgery (COMBO TRIAL): interim results of a prospective intraindividual study**

*G. Vatteroni*[1], N. Turri[1], F. Fici[1], M. Filippini[2], N. Basla[3], G. Pinna[1], G. Pruneddu[1], R. M. Trimboli[1], D. Bernardi[1]; [1]Milan/IT, [2]Brescia/IT, [3]Pavia/IT
*(giulia.vatteroni@gmail.com)*

**Purpose or Learning Objective:** To present interim results from the 'COMBO TRIAL, a prospective intraindividual study evaluating the performance of Contrast-Enhanced Mammography(CEM) vs Digital Mammography(DM) for the surveillance of women with a personal history of breast cancer(BC).
**Methods or Background:** Between January 2023 and April 2024, women who underwent breast-conserving surgery for BC were invited to undergo CEM for routine surveillance. Exclusion criteria included: suspicious symptoms of BC, allergy to iodinated contrast agents, renal failure, breast implants. For each patient, one reader reported CEM while a second reader, independent and blinded, evaluated only LE images equivalent to DM. The reference standard was 1-year follow-up for negative cases and biopsy/surgery for BI-RADS 4/5. Cancer Detection(CD) rate for both DM and CEM and incremental CD rate for CEM, sensitivity, specificity, PPV, NPV and accuracy were calculated. We evaluated differences in diagnostic performance between DM and CEM using McNemar test($p<0.05$ significant).
**Results or Findings:** Overall, 600 women were included in the analysis with a recall rate of 9.8%. Among them, 14 cases of BC were detected: 8 (5 DCIS +3 invasive) were identified by both DM and CEM, while CEM detected 6 additional cases (1 DCIS+5 invasive). Three cases were missed by both DM and CEM but subsequently detected by US, resulting in a global recurrence rate of 2.8%. The CD rate for CEM was 23 per 1000, compared to 13 per 1000 for DM, indicating an incremental CD for CEM of 10 per 1000 (p=0.014). Compared to DM, CEM demonstrated significantly higher sensitivity(82.4% vs. 47.1%), slightly lower specificity(96.4% vs. 97.6%), slightly higher PPV(40.0% vs. 36.4%), slightly higher NPV(99.5% vs. 98.4%), and similar accuracy(96.0% vs. 96.2%).
**Conclusion:** Implementation of CEM in BC surveillance was associated with a significant increased detection of invasive cancers.
**Limitations:** n/a
**Funding for this study:** this study received research support by Siemens Helthineers
**Ethics committee - additional information:** The ethics commitee approved this study
**Author Disclosures:**
Rubina Manuela Trimboli: Nothing to disclose
Federica Fici: Nothing to disclose
Giulia Pruneddu: Nothing to disclose
Daniela Bernardi: Nothing to disclose
Nicolò Turri: Nothing to disclose
Giulia Vatteroni: Nothing to disclose
Marco Filippini: Nothing to disclose
Nicoletta Basla: Nothing to disclose
Giulia Pinna: Nothing to disclose

**Evaluation of lesion conspicuity on contrast-enhanced mammography improves the performance in the assessment of malignancy**

*M. Conti*, R. Rella, S. Palma, S. Amodeo, N. Di Cataldo, D. Moretti, M. Costantini, O. Tommasini, P. Belli; Rome/IT
*(conti.marco87@gmail.com)*

**Purpose or Learning Objective:** Aim of the study is to assess the performance of Lesion Conspicuity (LC) in Contrast Enhanced Mammography (CEM) in the prediction of malignancy.
**Methods or Background:** This is an observational retrospective study involving 153 women (median age, 44.1 years, IQR: 36-52) who underwent CEM and subsequent histological assessment at Policlinico Universitario A. Gemelli IRCCS (April 2021-October 2023). Two radiologists (with 2 and 7 years of experience in breast imaging, independently) evaluated low-energy (LE) images and LC (categorizing it as absent, low, moderate or high) and assigned a BIRADS category of suspicion to the lesion basing on both. Diagnostic performance of LE images and LC interpretation together was calculated using histological results of the biopsy as gold standard. Subgroup analyses based on mammographic breast density, background parenchymal enhancement (BPE) on CEM and type of lesions were also performed.
**Results or Findings:** The interpretation of LE images together with the value of LC showed a sensitivity (SE) of 96.8% (95%CI: 92.1%-99.1%) and a specificity (SP) of 66.7% (95%CI: 46.0%-83.5%) vs. a SE=90.7% (95%CI: 82.5%-95.9%) and a SP=52.6% (95%CI: 28.8%-75.5%) for LE images evaluation alone. Diagnostic performance of LE images + LC evaluation was better than LE images alone both in dense (SE=96.4% and SP=89.8% vs. SE=93.3% and SP=60.0%) and adipose (SE=97.3% and SP=66.7% vs. SE=89.3% and SP=44.4%) breasts. Diagnostic performance of LE images + LC evaluation is better than LE images alone both in minimal/mild and moderate/marked BPE and in all types of lesions.
**Conclusion:** The evaluation of LE images joint with the value of LC demonstrated a better performance than LE images alone in predicting the malignancy of lesions.
**Limitations:** Limitations of the present study include its retrospective and monocentric design.
**Funding for this study:** No funding was received for this study.
**Ethics committee - additional information:** The study was approved by the local Institutional Review Board (ID: 6476)
**Author Disclosures:**
Nicola Di Cataldo: Nothing to disclose
Rossella Rella: Nothing to disclose
Oscar Tommasini: Nothing to disclose
Simone Palma: Nothing to disclose
Marco Conti: Nothing to disclose
Melania Costantini: Nothing to disclose
Silvia Amodeo: Nothing to disclose
Delia Moretti: Nothing to disclose
Paolo Belli: Nothing to disclose

**Diagnostic Accuracy of Contrast-Enhanced Mammography (CEM) in Preoperative Staging of Breast Tumors: A Comparative Study with Histology and Mammography**

*M. Balbino*[1], F. Masino[2], M. Montatore[3], S. Suriano[4], F. A. Carpagnano[4], G. Capuano[3], G. Guglielmi[5]; [1]Triggiano/IT, [2]Bari/IT, [3]Barletta/IT, [4]Foggia/IT, [5]Andria/IT
*(marinabalbino93@gmail.com)*

**Purpose or Learning Objective:** To evaluate the diagnostic accuracy of CEM in detecting and measuring breast tumors, comparing it with histological findings.
**Methods or Background:** This study evaluated the diagnostic accuracy of Contrast-Enhanced Spectral Mammography (CEM) in 69 breast cancer patients treated at the “Santa Maria” Hospital (Bari, Italy) from January 2018 to September 2023. Patients ranged from 33 to 86 years old, with an average age of 55.8 years. All underwent CEM and subsequent biopsy or cytology for confirmation. Exclusion criteria included deep lesions, post-biopsy hematomas, or renal insufficiency. CEM was performed using a GE Healthcare system with two post-injection projections: Cranio-Caudal and Medio-Lateral-Oblique. Three experienced radiologists analyzed the scans, comparing lesion sizes from mammography, CEM, and histology.
**Results or Findings:** Results showed that CEM underestimated lesion size in 28 cases, while mammography did so in 21. CEM identified 92.3% of multifocal masses, compared to 58.3% with mammography. In histology, 49.27% of patients had mass-like neoformations, with 33.3% showing homogeneous contrast enhancement. High contrast enhancement correlated with higher Ki67 proliferation indices (50-65%). The majority of neoplasms were Infiltrating Ductal Carcinomas. Statistical analysis revealed that CEM was more accurate than mammography, with the Wilcoxon signed-rank test showing no significant difference between CEM and histological measurements (p=0.9928). CEM showed high diagnostic accuracy in preoperative staging, supporting its use over MRI, particularly due to its lower cost, faster acquisition time, and better patient tolerance. The study concludes CEM is a valuable, cost-effective alternative to MRI for assessing tumors before surgery, particularly in preoperative staging and the identification of multifocal lesions.
**Conclusion:** CEM demonstrated high accuracy in assessing breast tumor size and extent, proving to be a valid alternative to MRI due to its lower cost, faster acquisition time, and better patient tolerability.
**Limitations:** No
**Funding for this study:** No
**Ethics committee - additional information:** No
**Author Disclosures:**
Marina Balbino: Nothing to disclose
Giuseppe Guglielmi: Nothing to disclose
Francesca Anna Carpagnano: Nothing to disclose
Silvia Suriano: Nothing to disclose
Manuela Montatore: Nothing to disclose
Federica Masino: Nothing to disclose
Giulia Capuano: Nothing to disclose

Friday

**Optimizing microcalcification assessment: the role of contrast-enhanced mammography on reducing unnecessary biopsies**

*A. Santonocito*, T. H. Helbich, P. Clauser, P. A. Baltzer; Vienna/AT
*(santonocitoambra@gmail.com)*

**Purpose or Learning Objective:** Microcalcifications are frequently observed in screening mammography, with malignancy rates ranging from 6% to 82%. Their characterization and work-up are a major challenge for the radiologist. This study aimed to assess the role of contrast-enhanced mammography (CEM) in managing microcalcifications.
**Methods or Background:** This retrospective, single-centre, IRB-approved, study included consecutive patients underwent CEM for BI-RADS 4 microcalcifications between October 2018 and September 2024. Patients without a standard of reference were excluded. The standard of reference was histology from biopsy or surgery, or a one-year follow-up for non-suspicious cases. Morphology, distribution, density, and density heterogeneity of microcalcifications were assessed on LE images; enhancement type and lesion conspicuity were assessed on RC images according to the CEM lexicon. Microcalcification and enhancement characteristics were analysed through Spearman correlation and chi-square tests to evaluate associations with histological outcomes.
**Results or Findings:** A total of 210 lesions in 197 patients (mean age 55 ±11 years old) were analysed. Of these, 124 (63%) were benign and 72 (37%) were malignant. Lesion conspicuity correlated with morphology (r=0.429), pleomorphism (r=0.514), distribution (r=0.235), and density heterogeneity (r=0.204), while no correlation was found with density (r=-0.111). Chi-square test showed significant differences between benign and malignant calcifications for lesion type (p<0.001), morphology (p<0.001), grade of pleomorphism (p<0.001), distribution (p<0.001), density (p<0.001), enhancement type (p<0.001), lesion conspicuity (p<0.001) and grade of density heterogeneity (p=0.02). No significant differences were found for size (p>0.05).
**Conclusion:** Our findings suggest that CEM may help differentiate between benign and malignant microcalcifications, potentially reducing unnecessary biopsies.
**Limitations:** Retrospective study; small cohort
**Funding for this study:** None
**Ethics committee - additional information:** Number: 1391/2022
**Author Disclosures:**
Pascal A.T. Baltzer: Nothing to disclose
Thomas H. Helbich: Nothing to disclose
Ambra Santonocito: Nothing to disclose
Paola Clauser: Nothing to disclose

**Contrast Enhanced Mammography (CEM) in the management of locally advanced breast cancer receiving neoadjuvant therapy (NAT)**

*S. Vidali*, F. Di Naro, D. De Benedetto, G. Bicchierai, E. Vanzi, C. Bellini, C. Boeri, V. Miele, J. Nori; Florence/IT
*(sofia.vidali@yahoo.it)*

**Purpose or Learning Objective:** Among contrast-enhanced mammography (CEM)'s indication, is the evaluation of locally advanced breast cancer(LABC)'s response to neoadjuvant therapy (NAT). CEM has potential not only in the assessment of radiologic complete response (rCR) but also in predicting tumour response based on biological features and enhancement patterns.
**Methods or Background:** We retrospectively analysed post-NAT CEMs of 141 patients diagnosed with LABC between 2016-2024 and correlated enhancement patterns (rCR, residual enhancement, RE) with diagnostic biopsy biologic features and post-surgical pathology data (pCR, residual disease RD).

**Results or Findings:** CEM showed RE in 92 patients (68 with RD and 24 with pCR) was negative in 49 (17 with RD and 32 with pCR), with resulting mean sensitivity of 80% and specificity of 57%. Reclassifying DCISs as RD instead of pCR, specificity raised to 65%: these performance data are adequate and comparable to those reached by CE-MRI, the gold standard. Based on biological subtypes, distinct enhancement patterns resulted coherent with the expected therapeutic responses: luminal A tumours (46 patients) were less responsive to NAT (11% pCR, 5 patients, 4 of them with rCR, 80%), while HER2+ (37 patients) and triple negative(TN)BCs (20 patients), the more aggressive forms were more likely to achieve pCR and rCR: respectively 59% pCR (22 patients) in the HER2+ group of which 64% (14 patients) with rCR, 50% pCR in the TNBCs (10 patients) of which 60% (6 patients) with rCR. Luminal B lesions (38 patients) achieved pCR in 45% of cases (17 patients) of which 13 with rCR (76%).
**Conclusion:** CEM is adequate and reliable in assessing disease response to NAT: enhancement patterns demonstrated correlation with biological features, forecasting CEM's potential as a prognostic and management tool for increasing conservative therapies and diagnostic follow-up.
**Limitations:** Sample's numerosity and heterogeneity.
**Funding for this study:** No funding was received for this study.
**Ethics committee - additional information:** Regione Toscana, Comitato Etico Area Vasta Centro, reference number: SPE_16.251
**Author Disclosures:**
Giulia Bicchierai: Nothing to disclose
Cecilia Boeri: Nothing to disclose
Jacopo Nori: Nothing to disclose
Sofia Vidali: Nothing to disclose
Vittorio Miele: Nothing to disclose
Chiara Bellini: Nothing to disclose
Ermanno Vanzi: Nothing to disclose
Diego De Benedetto: Nothing to disclose
Federica Di Naro: Nothing to disclose

**Contrast Enhanced Mammography Screening in Women with Dense Breasts**
*J. M. Net*, J. Spoont, S. Stamler, C. Pluguez-Turull, N. Brofman, A. Hamedi-Sangsari, M. Yepes; Miami, FL/US
*(josenet@me.com)*

**Purpose or Learning Objective:** To determine outcomes of screening Contrast Enhanced Mammography (CEM) in women with BI-RADS density 3 or 4, more specifically to determine outcomes of CEM in this population who did not undergo screening breast ultrasound.
**Methods or Background:** HIPAA compliant and IRB approved retrospective study evaluated all medical records of patients who underwent CEM at our institution between 8/2019-8/2024. Reports were reviewed and scored for breast density, additional work up (additional imaging, US, MRI), final BI-RADS, biopsy results and whether target represented CEM finding, if high risk lesion or malignant case was scored based on final surgical pathology. Final results were tallied to include overall CDR, PPV3, and interval cancer rate.
**Results or Findings:** 1215 CEM studies were performed between 2020-2024, 1079 were performed for screening of which 740 were assigned BIRADS density 3 or 4. 87 patients (11.7%) were referred for additional work up including US in 75, and MRI in 12. Of these, 46 were referred for biopsy which confirmed malignancy in 16 patients translating into a CDR of 21.6/1000 and PPV3 of 35.5%. Of the remaining 653 patients (88.2%) without additional work up, 46 underwent annual MRI within 12 months of CEM confirming the only 2 interval cancers in this study (0.3%), one stage 0 DCIS/linear 1.3 cm NME on staggered 6 month MRI and the other a 1.2 cm TNBC (T1Nitc) on staggered 6 month MRI - neither was seen in retrospect.
**Conclusion:** The low interval cancer rate combined with high CDR suggest that CEM screening in women with dense breasts can potentially replace supplemental breast US for adjunct screening.
**Limitations:** Limitations include modest sample size, retrospective design, and inclusion of cases from a single institution which limits generalizability.
**Funding for this study:** None
**Ethics committee - additional information:** IRB approved study.
**Author Disclosures:**
Antoine Hamedi-Sangsari: Nothing to disclose
Jose Miguel Net: Nothing to disclose
Monica Yepes: Nothing to disclose
Nicole Brofman: Nothing to disclose
Sarah Stamler: Nothing to disclose
Cedric Pluguez-Turull: Nothing to disclose
Jamie Spoont: Nothing to disclose

**09:30-11:00** **Research Stage 1**

### Research Presentation Session: Vascular

### RPS 1315

### Imaging of the aorta, pulmonary, and coronary arteries

**Moderator**
M. Cejna; Feldkirch/AT
*(manfred.cejna@lkhf.at)*

**Low-energy virtual monochromatic CT with deep-learning image reconstruction to improve detection of endoleak**
*T. Higashigawa*[1], Y. Ichikawa[1], K. Nakajima[2], T. Kobayashi[1], K. Domae[1], A. Yamazaki[1], N. Kato[1], H. Sakuma[1]; [1]Tsu/JP, [2]Ise/JP

**Purpose or Learning Objective:** To evaluate the diagnostic performance of low-energy virtual monochromatic CT imaging (VMI) combined with deep-learning image reconstruction (DLIR) for the detection of endoleaks.
**Methods or Background:** A cohort of 71 patients after endovascular aortic repair who underwent dynamic contrast-enhanced CT between March 2022 and August 2023 were studied. Raw data were reconstructed using three different methods: 70-keV VMI using conventional hybrid iterative reconstruction (HIR [ASiR-V50%]), and 40- and 70-keV VMI using DLIR (TrueFidelity-H). Contrast-to-noise ratio (CNR) of the endoleaks on venous phase CT was calculated. Three observers assessed the presence or absence of endoleak on a 5-point scale, taking into account the confidence level: score-1, endoleaks are definitely not present; score-2, probably not present; score-3, may be present; score-4, probably present; score-5, definitely present. A score of 3 or higher was considered positive for endoleak.
**Results or Findings:** Endoleaks were observed in 41 (58%) of 71 subjects. The CNRs of endoleaks were significantly higher in 40-keV DLIR (17.1±9.8) compared to 70-keV HIR (6.4±3.8; P<0.001) and 70-keV DLIR (10.2±6.2; P<0.001). ROC analysis for endoleak detection showed that AUC for 40-keV DLIR (0.92-0.99) was the largest for all observers (70-keV DLIR, 0.91-0.97; 70-keV HIR, 0.88-0.96). The percentage of patients with endoleaks who were correctly identified with a confidence level of ≥ score-4 in 40-keV VMI with DLIR was significantly higher compared to those in 70-keV VMI with HIR in one observer (Observer1, 85%(35/41) vs 73%(30/41), P=0.02; Observer2, 85%(35/41) vs 78%(32/41), P=0.20; Observer3, 98%(40/41) vs 90%(37/41), P=0.10, respectively).
**Conclusion:** The 40-keV VMI combined with DLIR reconstruction method improves the CNR of endoleaks and may help to correctly identify endoleaks with higher confidence compared to 70-keV VMI with HIR.
**Limitations:** The limitations of the study is the relatively small study population.
**Funding for this study:** No funding was received for this study.
**Ethics committee - additional information:** The study was approved by institutional review board (approval number; H2019-207).
**Author Disclosures:**
Kensuke Domae: Nothing to disclose
Takatoshi Higashigawa: Nothing to disclose
Hajime Sakuma: Nothing to disclose
Yasutaka Ichikawa: Nothing to disclose
Noriyuki Kato: Nothing to disclose
Tatsuhiro Kobayashi: Nothing to disclose
Akio Yamazaki: Nothing to disclose
Ken Nakajima: Nothing to disclose

**Image Quality Improvement of Ultra-low Dose CT Pulmonary Angiography Using Deep Learning Reconstruction Algorithm: Two-center Prospective Study**
*J. Lu*, L. Shen, Z. Zhao, Z. Bi, M. Zeng, M. M. Wang; Shanghai/CN
*(lujinjuan1016@163.com)*

**Purpose or Learning Objective:** To investigate the effects of deep learning reconstruction (DLR) on the image quality in ultra-low dose CT pulmonary angiography (CTPA), compared to hybrid iterative reconstruction (HIR) at routine dose.
**Methods or Background:** This study prospectively included 130 patients with suspected pulmonary embolism (PE) who underwent CTPA examination in two hospitals from April to July 2024. The noise index of routine dose (RD) group and ultra-low dose (ULD) group was set to 10 and 20, respectively. The CT images of RD group were reconstructed using HIR, while ULD group were reconstructed with HIR and DLR. Pulmonary CT value, signal-to-noise ratio

(SNR), and contrast-to-noise ratio (CNR) were assessed as quantitative criteria of image quality. Two senior radiologists independently evaluated the overall image noise, pulmonary artery visibility, and diagnostic confidence based on a 5-point Likert scale (5, best; 1, worst). The Mann-Whitney U test and the Wilcoxon signed rank test was used for statistical analysis.
**Results or Findings:** No statistically significant difference was found in the clinical data between the two groups (p>0.05). The ULD-DLR group exhibited higher SNRs and CNRs in all seven pulmonary arteries compared to the RD-HIR group (both p<0.05). The overall image noise and diagnostic confidence of the ULD-DLR images were significantly better than that in the RD-HIR images and ULD-HIR images (both p<0.001). The effective dose in the RD group and ULD group were 2.84±0.49mSv and 0.70±0.21mSv, respectively, representing a reduction of approximately 75% in the ULD group (p<0.001).
**Conclusion:** DLR can significantly reduce the radiation dose of CTPA examination without compromising the diagnosis of PE. Even at ultra-low radiation dose, its image quality is still better than HIR at routine dose.
**Limitations:** Not applicable.
**Funding for this study:** No.
**Ethics committee - additional information:** Shanghai Geriatrics Medical Center Ethics Committee (B2024-009)
**Author Disclosures:**
Mengsu Zeng: Nothing to disclose
Zicheng Zhao: Nothing to disclose
Mingliang Mingliang Wang: Nothing to disclose
Leilei Shen: Nothing to disclose
Zhenghong Bi: Nothing to disclose
Jinjuan Lu: Nothing to disclose

### Patient tailored contrast volume for preoperative CT angiography of the aorta: a prospective study based on patient heart rate and body surface area

*M. Dewilde*, W. Coudyzer, A. Laenen, H. Bosmans, G. Maleux; Leuven/BE
*(miloud.dewilde@gmail.com)*

**Purpose or Learning Objective:** To prospectively compare aortic image quality by adapting contrast volume and kiloVoltage (kV) in patients referred for preoperative aortic computed tomography (aCT).
**Methods or Background:** Eighty prospectively included patients were assigned into 3 groups: 50% of the contrast dose calculated on body surface area (BSA) and heart rate (HR) (group 1, n=56); 50% of the contrast dose calculated on BSA and HR and additional kV reduction (group 2, n=11); 50% of contrast dose calculated on BSA and HR and additional contrast dilution 80% contrast & 20% NaCl (group 3, n=13). Quantitative (measurement of Hounsfield units) analysis at different anatomical aortic levels and qualitative image analysis by 2 radiologists using a visual score (1 = inadequate; 5 = excellent) was performed.
**Results or Findings:** Mean contrast dose injected was 46.1 ml, 28.3 ml and 35.0 ml for group 1, 2 and 3 respectively, with a significant difference between group 1 and 2 (P=<0.001) and between group 1 and 3 (P=<0.001); no difference between group 2 and 3 (P=0.072). Mean qualitative scores were 4.35/5, 2.82/5 and 3.46/5 for group 1, 2 and 3 respectively. No patient needed repeat imaging for inadequate aortic CT-imaging. Interobserver agreement was moderate for group 1 and 3 (0.577 and 0.576 resp.) and fair for group 2 (0.282) with consistent difference in scoring.
**Conclusion:** Meaningful contrast dose reduction in preoperative aCT while maintaining diagnostic efficacy is feasible through utilization of a contrast injection algorithm incorporating patient’s HR and BSA, coupled with adjusting kV values.
**Limitations:** Limited number of patients (n=80). Only patients with a pre operative angio CT were included.
**Funding for this study:** No funding was received for this study.
**Ethics committee - additional information:** The institutional ethics committee approved this study (S58042).
**Author Disclosures:**
Walter Coudyzer: Nothing to disclose
Annouschka Laenen: Nothing to disclose
Hilde Bosmans: Nothing to disclose
Miloud Dewilde: Nothing to disclose
Geert Maleux: Nothing to disclose

### AI-based reconstruction algorithm applied to low-kV and low contrast medium volume CT for TAVI planning compared with low dose CT with Model-Based algorithm: image quality and radiation dose exposure

*C. R. G. L. O. M. Talei Franzesi*, D. Ippolito, C. Maino, P. N. Franco, D. G. Gandola, R. Corso; Milan/IT
*(ctfdoc@hotmail.com)*

**Purpose or Learning Objective:** To evaluate image quality and radiation dose reduction of deep learning reconstruction algorithm in CT angiography (CTA) studies performed for TAVI planning, compared with low dose CTA reconstructed with hybrid iterative algorithm
**Methods or Background:** Fifty six patients candidates for TAVI were enrolled in this study and 26 patients (study-group) were examined with 128 MDCT scanner, with 80 kV, automated mAs dose-modulation and 50 mL of contrast media (CM), combined with a new deep learning reconstruction algorithm (Precise Image); while a control group of 32 patients were evaluated with 256 MDCT (100 KV; automated mAs; 50 mL of CM) reconstructed with hybrid iterative reconstruction algorithm (iDose4). Subjective (using a 4-point Likert scale) and objective image quality (vascular enhancement, SNR and CNR in different aortic levels and in the iliac arteries) were evaluated and the radiation dose exposure of both groups (CTDIvol and DLP) was calculated
**Results or Findings:** Study group with deep learning algorithm demonstrated significantly higher mean attenuation values (p<.05) in all the measurements compared to the control group with model based algorithm (aortic root 621HU vs 314 HU; external iliac arteries 537HU vs 335HU). Mean DLP and CTDI of study group was significantly lower than in control group (DLP: 395 mGy*cm vs 1600 mGy*cm, p<0.001; CTDI: 8.03 mGy vs 23.5 mGy, p<0.001), with an overall radiation dose reduction of about 75%. Furthermore, study group showed a significant decrease of image noise with an increase of image quality
**Conclusion:** Deep learning based CT reconstruction algorithm combined with low Kv setting allows to significantly reduce radiation dose exposure and increase the image quality in CTA protocol for TAVI planning, in comparison with low dose CTA reconstructed with hybrid iterative algorithm
**Limitations:** None
**Funding for this study:** None
**Ethics committee - additional information:** None
**Author Disclosures:**
Davide Giacomo Gandola: Nothing to disclose
Cesare Maino: Nothing to disclose
Cammillo Roberto Giovanni Leopoldo Oreste Massimiliano Talei Franzesi: Nothing to disclose
Rocco Corso: Nothing to disclose
Paolo Niccolò Franco: Nothing to disclose
Davide Ippolito: Nothing to disclose

### Determining elasticity of the thoracic aorta in patients with giant cell arteritis using non-contrast-enhanced magnetic resonance imaging at 1.5 T

*M. Both*, C. Jochum, J. H. Schirmer, E. A. Strathmann, P. Langguth, S. Sandra Freitag-Wolf, C. Von Der Burchard, O. Jansen, M. Salehi Ravesh; Kiel/DE
*(mboth@rad.uni-kiel.de)*

**Purpose or Learning Objective:** The application of imaging techniques for early detection of thoracic aortic aneurysms in patients with giant cell arteritis (GCA), including the identification and monitoring of subgroups at high risk for this condition, is still the subject of debate. We investigated whether aortic stiffness could be quantified based on MRI and used as a potential biomarker for post-inflammatory damage.
**Methods or Background:** Ten GCA patients in clinical remission and 36 healthy volunteers (HVs) were examined using non-contrast-enhanced cine-balanced steady-state free precession (bSSFP) MRI technique to determine the distensibility and diameter of the ascending (AAo), descending (DAo), and arch (AArch) segments of the thoracic aorta. In addition, changes in aortic diameters during follow-up in GCA patients and the impact of demographic and clinical characteristics on the aortic elasticity were investigated.
**Results or Findings:** Distensibility was significantly higher in the AArch (p=0.039) and in the DAo (p=0.004) than in the AAo in HVs, but not in GCA patients. Aortic distensibility was significantly lower in patients than in HVs in the AArch (0.89 vs. 2.15, p=0.035). Age was an additional predictor of aortic stiffening in the AAo (p=0.029) and DAo (p=0.001) of HVs. In patients with GCA, the diameter increased at an above-average rate in all aortic segments (AAo 1.04 mm/year, AArch 1.12 mm/year, DAo 0.95 mm/year) compared to baseline MRI.
**Conclusion:** The bSSFP MRI technique revealed functional and structural differences in the thoracic aorta of patients with GCA as a potential marker for weakness of the thoracic aortic wall.

**Limitations:** The small size of our patient group is the main limitation due to its single-center design. Another limitation relates to the fact that most of our study patients presented with predominantly cranial symptoms, some without proof of aortitis on MRI.
**Funding for this study:** None
**Ethics committee - additional information:** The Ethics Committee at the Faculty of Medicine of Kiel University approved this study (No. D577/18).
**Author Disclosures:**
Claus Von Der Burchard: Nothing to disclose
Mona Salehi Ravesh: Nothing to disclose
Patrick Langguth: Nothing to disclose
Marcus Both: Nothing to disclose
Chiara Jochum: Nothing to disclose
Jan Henrik Schirmer: Nothing to disclose
Sandra Sandra Freitag-Wolf: Nothing to disclose
Olav Jansen: Nothing to disclose
Eike Andreas Strathmann: Nothing to disclose

**Accelerating Coronay CT Angiographies via Improved Patient Preparation**
*A. M. C. Boehner*, B. Salam, A. Jacob, A. Isaak, C. C. Pieper, D. Kütting; Bonn/DE
*(boehner.amc@gmail.com)*

**Purpose or Learning Objective:** Coronary Computed Tomography Angiography (CCTA) often requires extensive preparation, contributing to prolonged in-room time. This study aims to assess the impact of pre-examination preparation, including the administration of IV beta-blockers and ECG lead placement outside the examination room, on reducing in-room time for coronary CT scans.
**Methods or Background:** A prospective study with 139 patients was conducted, comparing standard in-room preparation (control cohort) with receiving preparation outside (intervention cohort) and mostly omitted preparation, scanned via a spiral acquisition protocol (spiral cohort). Patients' heart rates were regulated in the intervention group before entering the examination room. Key measures included: patient entering the examination-room, installation of patient monitoring, heart rate adjustment, first scan, heart rate during scanning and image quality.
**Results or Findings:** The intervention cohort demonstrated significantly (P<0.0001) reduced in-room time compared to the control cohort (984±347s vs. 704±308s). The spiral cohort performed best and displayed the lowest variability (583±103s, P<0.0001). The heart rates of the spiral cohort was highest with 69±21bpm (P<0.04), but the intervention cohort did not differ from the control cohort (60±7bpm vs. 59±6bpm, P=0.88). The rate of non-diagnostic segments remained low across all groups (control: 3.5%, intervention: 1.5%, spiral: 2.0%).
**Conclusion:** Pre-examination preparation outside of the examination room, including installation of patient monitoring and administration of IV beta-blockers, significantly reduces in-room time for CCTA without compromising image quality. Alternatively, a spiral image acquisition protocol allows for the omission of most preparatory steps. Both approaches offer a feasible strategy to streamline workflow and enhance efficiency in cardiac imaging departments.
**Limitations:** The limitation regarding the spiral cohort is the need to preselect patients with a coronary-calcium-score <400. Some radiological departments may lack the premises to implement our approach.
**Funding for this study:** The study was conducted in collaboration with Siemens Healthineers.
**Ethics committee - additional information:** Administrational
**Author Disclosures:**
Alexander Isaak: Nothing to disclose
Alexander Marc Christian Boehner: Nothing to disclose
Alice Jacob: Nothing to disclose
Daniel Kütting: Nothing to disclose
Claus Christian Pieper: Nothing to disclose
Babak Salam: Nothing to disclose

**Optimizing HU Thresholds for Accurate Calcium Scoring in Contrast-Enhanced CT: Robust Alternatives to the Agatston Score**
*L. D. Grünewald*, V. Koch, S. Mahmoudi, J. Gotta, P. Reschke, J-E. Scholtz, S. Martin, C. Booz, T. Vogl; Frankfurt/DE

**Purpose or Learning Objective:** To approximate the Agatston score in contrast-enhanced CTs using a volumetric approach without distortions from contrast agents.
**Methods or Background:** The aorta of 1276 patients (886 men, 390 women; median age 67 years; interquartile range 57-76) without prior surgical interventions who underwent contrast-enhanced multi-phase CT between January 2018 and December 2023 were retrospectively analyzed. For all patients, the Agatston score was derived from unenhanced CT scans for the thoracic and abdominal aorta. The number and volume of plaques were assessed for the thoracic and abdominal aorta in arterial and venous contrast phases using thresholds ranging from 100 to 1000 to assess the influence of contrast agents. Correlations with the Agatston score were calculated, and linear regression was used to identify the optimal threshold.
**Results or Findings:** Median aortic enhancement was 46 HU (unenhanced), 323 HU (arterial), and 120 HU (venous). In venous phases, a threshold of 300 HU yielded the highest correlations with the Agatston score (thoracic: r=0.91; abdominal: r=0.93; p<0.001). In arterial phases, a threshold of 900 HU provided the best correlation (thoracic: r=0.72; abdominal: r=0.65; p<0.001). Linear regression confirmed these thresholds, but showed only moderate predictive power ($R^2$=0.66 for venous, $R^2$=0.52 for arterial phases). Dynamic thresholding resulted in poor correlation (r=0.26; p<0.001) and low predictive value ($R^2$=0.07).
**Conclusion:** Plaque volume assessment using optimized HU thresholds can reliably approximate the Agatston score in contrast-enhanced CTs, offering a robust assessment without contrast-induced distortion. This approach is particularly valuable when non-contrast images are unavailable, such as in staging or pre-TAVR evaluations.
**Limitations:** Patient collective with high plaque burden.
**Funding for this study:** No funding was received for this study.
**Ethics committee - additional information:** Waiver due to retrospective nature
**Author Disclosures:**
Simon Martin: Nothing to disclose
Christian Booz: Nothing to disclose
Thomas Vogl: Nothing to disclose
Jan-Erik Scholtz: Nothing to disclose
Vitali Koch: Nothing to disclose
Scherwin Mahmoudi: Nothing to disclose
Philipp Reschke: Nothing to disclose
Leon David Grünewald: Nothing to disclose
Jennifer Gotta: Nothing to disclose

**Performances of spectral CT for the detection and characterization of communications between the true and the false lumen in aortic dissections**
A. Janin-Manificat[1], M. Sigovan[1], L. Boussel[1], A. Millon[1], P. C. Douek[1], *S. Boccalini*[2]; [1]Lyon/FR, [2]Villeurbanne/FR
*(sara.boccalini@yahoo.com)*

**Purpose or Learning Objective:** To assess the performance of conventional CT (conv-CT) and spectral CT (spectral-CT) for the detection and characterization of communications between the true (TL) and false lumen (FL) in aortic dissections, using 4D-flow MRI as the reference.
**Methods or Background:** 18 patients with type A and B aortic dissection who underwent 4D-flow MRI, conv-CT and spectral-CT were included. For each patient, the exams closest in time, without any surgical or endovascular intervention in-between were retrieved and subjectively analysed by two observers, independently for conv-CT and in consensus for MRI and spectral-CT. Communications between the two lumens were identified as: intimal tears on conv-CT; focal alterations in velocities corresponding to jet flows on 4D-flow; both intimal tears and focal changes of contrast concentration corresponding to jet flows on spectral-CT. The number, size, and location of communications were noted. Additionally, the direction of the flow was assessed for spectral-CT and MRI.
**Results or Findings:** Of the 176 communications detected with 4D-flow, spectral-CT allowed visualisation of 122 (69%) compared to 58 (33%) for Obs1 and 38 (22%) for Obs2 for conv-CT, yielding an accuracy twice as high (63% vs. 29-30%). On spectral CT, in only 45 cases (26%) the size of the communications could be assessed, in all other cases only jet flows were detected without visible intimal tears. The flow was unidirectional TL-FL in 2 cases for both MRI and spectral-CT and bidirectional in 5 and 3 cases for the two modalities. In all other cases the flow was in the direction TL-FL.
**Conclusion:** Spectral-CT outperformed conv-CT for the detection of communications between TL and FL in aortic dissections. Spectral-CT allows direct visualization of flow jets, and their direction, through intimal tears.
**Limitations:** Low number of patients; time in-between different exams
**Funding for this study:** No
**Ethics committee - additional information:** Approved
**Author Disclosures:**
Antoine Janin-Manificat: Nothing to disclose
Philippe Charles Douek: Speaker: Philips
Loïc Boussel: Speaker: Philips
Sara Boccalini: Speaker: Philips
Monica Sigovan: Nothing to disclose
Antoine Millon: Nothing to disclose

**Comparison of artificial intelligence and inexperienced physicians in pulmonary embolism detection at deep learning reconstruction-based ultra-low radiation dose CT pulmonary angiography**
*J. Lu*, L. Shen, Z. Zhao, Z. Bi, M. Zeng, M. M. Wang; Shanghai/CN
*(lujinjuan1016@163.com)*

**Purpose or Learning Objective:** To assess the performance of artificial intelligence (AI) software and inexperienced physicians in diagnosing pulmonary embolism (PE) at deep learning reconstruction-based ultra-low dose (ULD) CT pulmonary angiography (CTPA).
**Methods or Background:** This prospective two-center study contained 210 patients with suspected pulmonary embolism (PE) who underwent CTPA examination, randomizing into two groups with equal proportion of patients. Images in the routine-dose (RD) group were reconstructed using hybrid iterative reconstruction (HIR, AIDR 3D, FC08), while ULD images were reconstructed using HIR and deep learning reconstruction (DLR, AiCE), respectively. A subset of 74 participants (1:1 PE to non-PE ratio) was randomly selected and evaluated by two inexperienced physicians and AI software (Discover PE, uAI). Reference standard was established by expert consensus. The diagnostic accuracy (sensitivity and specificity) of the AI or reader interpretations were compared between methods by bootstrapping.
**Results or Findings:** There was no statistically significant difference in the patient demographics between two groups. ULD-DLR images exhibited significantly higher objective and subjective image quality compared to both RD-HIR and ULD-HIR images. The AI software exhibited near-perfect accuracy in both ULD-HIR and ULD-DLR sets (sensitivity: 97.30 %, specificity: 100 %). In comparison, two physicians showed a mean sensitivity of 75.68% and specificity of 93.75% in ULD-HIR sets, and a mean sensitivity of 94.59% and specificity of 100.00% in ULD-DLR sets. Inter-observer agreement was moderate for HIR ($\kappa = 0.75$) and good for DLR ($\kappa = 0.81$). The effective dose of ULD group was significantly lower than the RD group (2.74±0.47 mSv vs. 0.73±0.25 mSv, $p<0.001$).
**Conclusion:** DLR can significantly reduce the radiation dose of CTPA examination without compromising the diagnosis of pulmonary embolism even at ultra-low radiation dose. AI software outperforms inexperienced physicians in interpreting ULD images.
**Limitations:** Not applicable.
**Funding for this study:** No.
**Ethics committee - additional information:** Shanghai Geriatrics Medical Center Ethics Committee (B2024-009)
**Author Disclosures:**
Mengsu Zeng: Nothing to disclose
Zicheng Zhao: Nothing to disclose
Mingliang Mingliang Wang: Nothing to disclose
Leilei Shen: Nothing to disclose
Zhenghong Bi: Nothing to disclose
Jinjuan Lu: Nothing to disclose

**09:30-11:00** **Research Stage 2**

### Research Presentation Session: Interventional Radiology

### RPS 1309

### Interventions in malignant liver disease

**Moderator**
L. Novosel; Zagreb/HR
*(novosel_luka@hotmail.com)*

**Oncologic Ablation in Germany: 2018-2023 data from the German Society of Interventional Radiology Registry**
*J. Uhlig*[1], L. Biggemann[1], J. Nadjiri[2], T. Kroencke[3]; [1]Göttingen/DE, [2]Munich/DE, [3]Augsburg/DE
*(johannes.uhlig@med.uni-goettingen.de)*

**Purpose or Learning Objective:** To assess the current utilization, technical approaches and complications of oncologic ablation in Germany.
**Methods or Background:** The German Society of Interventional Radiology ("DeGIR") registry was queried for patients receiving ablation treatments between 2018-2023. Patient demographics, indications, and technical ablation parameters were descriptively assessed.
**Results or Findings:** N=9157 patients receiving oncologic ablation were included (34.3% female; median age 67yo). Between 2018-2023, annual ablation number remained approximately constant at 1000 cases/year. Ablations were performed in the liver (71.4%), kidney (11.3%), musculoskeletal system (7.3%) and lung (5.4%), mainly with curative intent (64%) or for symptomatic treatment / palliation (30.6%). N=7371 patients were imaged with CT before ablation (80.5%), 4176 with MRI (45.6%), and 173 with PET (1.9%; not mutually exclusive). Ablation guidance was achieved using CT (89.6%), ultrasound (5.7%), MRI (2.8%), cone-beam CT or fluoroscopy (0.9%, each). Ablation procedures were mainly performed under general anesthesia (74.6%) or analgosedation (14.5%). Microwave ablation was performed in most cases (69.4%), followed by radiofrequency (23.6%) and cryoablation (2.8%), often combined with tract ablation (51.2%). Only 48 procedures (0.5%) were preemptively terminated, mainly due to anatomical difficulties (n=16) or uncooperative patients (n=11). During or within the first 24h after ablation, 4.8% of patients experienced any complications, the majority being low-grade. Another 45 patients (0.5%) experienced delayed complications 24h or later after ablation, mainly infections/abscesses (n=21).
**Conclusion:** Oncologic ablations are routinely performed in Germany with low procedural complication rates, mostly using CT-guided microwave or radiofrequency ablation for hepatic or renal tumors.
**Limitations:** Since participation in the DeGIR registry is not mandatory, there could be selection bias of included cases and participating sites, limiting the generalizability of results.
**Funding for this study:** Not applicable.
**Ethics committee - additional information:** Not applicable - retrospective anonymised registry data.
**Author Disclosures:**
Thomas Kroencke: Nothing to disclose
Johannes Uhlig: Nothing to disclose
Lorenz Biggemann: Nothing to disclose
Jonathan Nadjiri: Nothing to disclose

**Enhancing Neoadjuvant Immunotherapy Efficacy through Partial Cryoablation in a Hepatocellular Carcinoma Mouse Model**
*T. Kao*[1], E. Meister[1], J. Santana[2], J. Israel[2], A. Shewarega[2], J. Tefera[2], D. C. Madoff[2], L. J. Savic[1], J. Chapiro[2]; [1]Berlin/DE, [2]New Haven, CT/US
*(Tabea.kao@charite.de)*

**Purpose or Learning Objective:** Hepatocellular carcinoma (HCC) exhibits an immunosuppressive microenvironment which can be aggravated by incomplete tumor ablation. Immune checkpoint inhibitors (ICIs) such as anti-PD-1 are guideline-approved therapies for advanced HCC. Combining ablation with ICIs could potentially strengthen anti-cancer immunity, but supporting evidence is limited. We aim to evaluate the effect of neoadjuvant systemic anti-PD-1 on the local immune response in residual tumors following partial cryoablation in a TIB-75 murine HCC model.
**Methods or Background:** Forty-eight male and female BALB/c mice aged 6-12 weeks underwent orthotopic inoculation of TIB-75 cells to induce a solitary HCC lesion. After 7 days, mice were randomized into 4 treatment groups: (a) control, (b) anti-PD-1, (c) partial cryoablation, and (d) anti-PD-1 followed by partial cryoablation. The percentage of positively stained T-cell subsets and tumor-associated macrophages within the tumor was assessed in paraffinized liver tissue samples using immunohistochemistry (CD3+, CD4+, CD8+, CD68+, CD206+, FOXP3+) and quantified on digitized slides. Treatment groups were compared using unpaired Mann-Whitney U and Kruskal-Wallis test with Dunn correction.
**Results or Findings:** Mice treated with anti-PD-1 (n=12, group b) showed greater tumoral infiltration of CD3+, CD4+ and CD8+ T-cells than control (CD3+: mean 21.4% vs. 6.7%; P=<0.0001, CD4+: mean 21.3% vs. 6.0%; P=<0.0001, CD8+: mean 7.5% vs. 3.8%; P=0.005). Partial cryoablation alone (n=12) had greater infiltration of CD206+ M2-like macrophages than control (mean 32.4% vs. 14.6%; P=0.007). Anti-PD-1 combined with partial cryoablation (n=12) showed significantly more infiltration of CD3+ T-cells (mean 13.7% vs. 6.1%; P=0.002) and fewer CD206+ M2-like macrophages (mean 26.1% vs. 32.4%; P=0.3474) than partial cryoablation alone (n=12).
**Conclusion:** Immune evasion following partial cryoablation can be counteracted with neoadjuvant anti-PD-1, suggesting effective combination therapy to treat both early-stage and advanced-stage HCC.
**Limitations:** The model was inoculated in healthy, non-cirrhotic mouse liver.
**Funding for this study:** NIH grant 2R01CA206180
**Ethics committee - additional information:** All experimental procedures were approved by the Yale University Institutional Animal Care and Use Committee (IACUC protocol number: 2022-20262).
**Author Disclosures:**
Joshua Israel: Nothing to disclose
Julius Chapiro: Nothing to disclose
Jonathan Tefera: Nothing to disclose
Jessica Santana: Nothing to disclose
Annabella Shewarega: Nothing to disclose
David Craig Madoff: Nothing to disclose
Ellen Meister: Nothing to disclose
Tabea Kao: Nothing to disclose
Lynn Jeanette Savic: Nothing to disclose

**MRI-based risk stratification for viable Hepatocellular Carcinomas post-Transarterial Chemoembolization: Correlation with pathological outcomes and prognostic implications**
*W. Wang*, Y-C. Wang; Nanjing/CN
*(weilang_wang@163.com)*

**Purpose or Learning Objective:** Accurate risk stratification of viable hepatocellular carcinomas (HCC) following transarterial chemoembolization (TACE) is essential for the development of individualized treatment strategies and enhancing the accuracy of prognosis predictions.
**Methods or Background:** This multi-center, retrospective study includes HCC patients who received TACE as their initial and sole treatment from February 2015 to October 2022 as training set (203 viable tumors). Additionally, a dataset from a multicenter clinical trial (NCT03113955) was subject to secondary analysis as test set (102 viable tumors). The final pathological validation set consists of a separate center, including individuals who had liver resection post-first TACE (120 viable tumors). All participants in both the training and test cohorts underwent contrast-enhanced MRI scans at baseline, and at one and six months after TACE. In the training set, univariate and multivariate logistic regression analysis was performed to identify clinical, laboratory and imaging variables to include in the predictive model.
**Results or Findings:** The predictive model incorporated five key imaging features: Mild-moderate T2 hyperintensity, T2-weighted peritumoral hyperintensity, Diffusion restriction, Irregular shape, and Heterogeneity. The model achieved areas under the curve (AUCs) of 0.85 (95% confidence interval [CI] 0.79 to 0.90) for the training cohort and 0.88 (95% CI 0.81 to 0.95) for the external test cohort. The risk model effectively distinguished high-risk from low-risk groups in the test cohort, with significant differences in progression-free survival (PFS) (P = 0.004) and two-year overall survival (OS) (P = 0.028). In the pathology cohort, the model correlated with microvascular invasion (MVI) grades (P = 0.003) and liver capsule invasion (P = 0.007).
**Conclusion:** This risk model based on imaging features for viable HCCs post-TACE exhibits robust predictive power for tumor viability at six months and for long-term survival outcomes.
**Limitations:** Not applicable
**Funding for this study:** This study has received funding by National Natural Science Foundation of China (NSFC, No. 82271978, 92359304, 82330060) and Zhongda Hospital Affiliated to Southeast University, Jiangsu Province High-Level Hospital Pairing Assistance Construction Funds (No. zdyyxy09).
**Ethics committee - additional information:** This multicenter, retrospective study was reviewed and approved by IEC for clinical research of the Zhongda Hospital, Southeast University, approval number [2022ZDSYLL410-P01], and conducted following the ethical principles outlined in the Helsinki Declaration of 1964 and its subsequent amendments, or other ethical standards with equivalent requirements. All patients and their families signed informed consent forms prior to surgery.
**Author Disclosures:**
Yuan-Cheng Wang: Nothing to disclose
Weilang Wang: Nothing to disclose

**Transarterial Embolization Alone Versus Drug-Eluting Beads Chemoembolization for HepatocellularCarcinoma (RAD-18-TAcE): a Randomized Clinical Trial**
*M. Taninokuchi Tomassoni*[1], M. Renzulli[1], S. Zanella[1], A. Doriguzzi Breatta[2], P. Marra[3], F. De Cobelli[4], C. Mosconi[1]; [1]Bologna/IT, [2]Turin/IT, [3]Bergamo/IT, [4]Milan/IT
*(makoto.taninokuchi@studio.unibo.it)*

**Purpose or Learning Objective:** This randomized clinical trial aims to compare transarterial embolization (TAE)and drug-eluted beads transarterial chemoembolization (DEB-TACE) in the treatment of hepatocellular carcinoma (HCC).
**Methods or Background:** Patients diagnosed with unresectable HCC were randomly assigned to either theTAE or DEB-TACE group. The primary endpoint was time to progression (TTP), and secondary endpoints included overall survival, cost-effectiveness, tumor response rates, and adverse events.
**Results or Findings:** A total of 111 patients were enrolled, with 56 in the TAE group and55 in the DEB-TACE group. Baseline characteristics were balanced between the two groups. The primary endpoint analysis showed that TAE was not different from DEB-TACE in termsof TTP (average of 12.13 and 10.87 months respectively, p=0.432). Overall survival, tumorresponse rates, and adverse events were also similar between the two groups. The cost-effectiveness ratio of DEB-TACE vs. TAE was evaluated considering that, with equaleffectiveness of the two treatments highlighted by the previous points, there being nostatistically significant difference in terms of days of hospitalization between DEB-TACEand TAE (average of 4.62 days and 5.20 days respectively, p=0.638).
**Conclusion:** In this randomized clinical trial, TAE showed comparable outcomes to DEB-TACE in the treatment of unresectable hepatocellular carcinoma. These findings suggest that TAE could be considered as an alternative for treating HCC with no differences in terms of safety and efficacy.
**Limitations:** The relatively small sample size and short-term follow-up period may limit the generalizability of the findings and the ability to detect subtle differences in outcomes between TAE and DEB-TACE.
**Funding for this study:** This study was funded by the Italian Ministry of Health.
**Ethics committee - additional information:** The trial was conducted in accordance with ethical standards and received approval from the institutional review boards. The study was registered at www.clinicaltrials.gov (NCT04803019).
**Author Disclosures:**
Andrea Doriguzzi Breatta: Nothing to disclose
Paolo Marra: Nothing to disclose
Matteo Renzulli: Nothing to disclose
Cristina Mosconi: Nothing to disclose
Sara Zanella: Nothing to disclose
Makoto Taninokuchi Tomassoni: Nothing to disclose
Francesco De Cobelli: Nothing to disclose

**MR guided catheter-based radiotherapy/brachytherapy of liver tumours – first experience and feasibility**
*M. P. Fabritius*, A. Haghpanah, O. Dietrich, D. Puhr-Westerheide, V. F. Schmidt, S. Corradini, J. Ricke, O. Öcal, M. Seidensticker; Munich/DE

**Purpose or Learning Objective:** To show feasibility and safety of MR guided catheter-based radiotherapy/brachytherapy of primary or secondary liver tumours
**Methods or Background:** Between June 2023 and April 2024, 27 patients with 54 liver lesions were treated within a prospecitve single-center trial on MR-guided catheter-based radiotherapy (MR BRIGHT trial). Treatments were performed under conscious sedation and local anesthesia using a 1.5T MRI system (Magnetom Solafit, Siemens) with a 15 cm loop coil. Gadoxetic acid (0.1 mmol/kg) was administered for contrast enhancement, followed by insertion of an 18G coaxial needle and navigation to the lesion via real-time gradient-echo fluoroscopy sequences (iMRI UI Extension, Research Software Package). The needle was exchanged for a 6F hydrophilic angiography sheath with a brachytherapy catheter. 3D T1-weighted sequences were sent to the radiation department for brachytherapy with an IR192 high-dose-rate (HDR) afterloading unit. Target doses ranged from 15 to 25 Gy, depending on tumor type (HCC, CRC, GIST, NET, and other metastases). Catheters were removed after BT, and the radiation tract sealed with gelatin sponge.
**Results or Findings:** The average lesion diameter was 13 ± 6 mm, whereas the average clinical target volume (CTV) was 3.0 ± 2.9 cm3. The average room time was 74 ± 35 minutes, the average time for catheter placement was 19 ± 11 minutes. The mean dose administered per lesion (D100) was 18.9 ± 3.6 Gy. Complications during and after BT were generally rare with only 2 patients (7.4 %.) having a minor bleeding without need for blood transfusion or intervention.
**Conclusion:** Overall, MR-guided catheter-based radiotherapy for liver tumours is feasible and safe, particularly for small lesions. With low complication rates and precise dosimetry achieved through advanced imaging, this approach holds promise for effective tumour management.
**Limitations:** n/a
**Funding for this study:** None
**Ethics committee - additional information:** LMU Munich
**Author Disclosures:**
Stefanie Corradini: Nothing to disclose
Olaf Dietrich: Nothing to disclose
Matthias Philipp Fabritius: Nothing to disclose
Alireza Haghpanah: Nothing to disclose
Vanessa Franziska Schmidt: Nothing to disclose
Max Seidensticker: Nothing to disclose
Daniel Puhr-Westerheide: Nothing to disclose
Osman Öcal: Nothing to disclose
Jens Ricke: Nothing to disclose

**Evaluation of Pain and Satisfaction in Patients with Liver Tumor treated with CT guided High Dose Rate Brachytherapy under Analgosedation– Preliminary Results**
*M. Z. Erforth*, L. K. Segger, U. Fehrenbach, F. Collettini, B. Gebauer, T. A. Auer; Berlin/DE
*(mo-zelda.erforth@charite.de)*

**Purpose or Learning Objective:** To evaluate feasibility for CT guided high dose rate (HDR) brachytherapy under analgosedation performed by interventional radiologists in patients with liver tumors.
**Methods or Background:** In this prospective single-center study (EA/122/23), 97 patients who received CT-guided HDR brachytherapy along with analgosedation using fentanyl and midazolam were enrolled and 77 were included in the final analysis. At the outset, a personality profile (from the EORTC catalog) related to their pain experience was also recorded for each patient. Structured questionnaires were employed to assess the patients' pain levels and satisfaction both at and after the intervention. Three months later, the patients were recontacted, and a follow-up survey was conducted. The results were recorded by means of a numeric analog scale and presented as categorical variables.
**Results or Findings:** First, pain was measured (0: no pain; to 10: maximum pain) 1. at catheter placement; 2. at the radiation; 3. after the radiation (catheter removal). At catheter placement, 75.5% (58/77) rated the pain as low (0-2), 18.0% (14/77) as moderate (3-6), and 6.5% (5/77) as severe (7-10). At the radiation and afterwards pain levels were rated as low in 83.0% (64/77) and 79.0% (61/77), as moderate in 11.5% (9/77) and 17.0% (13/77) and as severe in 5.5% (4/77) and 4.0% (3/77), respectively. Second, patient satisfaction was measured (1: completely dissatisfied; to 10: completely satisfied). In 1.3% (1/77) the lowest score was recorded while in 98.7% (76/77) a score ≥7 was recorded.
**Conclusion:** CT guided HDR brachytherapy under analgosedation is feasible and can be performed by interventional radiologists themselves without general anesthesia.
**Limitations:** Limitations include the short observation period and the small cohort of patients with a heterogeneous clinical history, neoplasm histology, and location, as well as inhomogeneity regarding previous treatments.
**Funding for this study:** None
**Ethics committee - additional information:** Institutional Review Board approved prospective study (EA1/122/23).
**Author Disclosures:**
Laura Katharina Segger: Nothing to disclose
Mo Zelda Erforth: Nothing to disclose
Uli Fehrenbach: Nothing to disclose
Bernhard Gebauer: Nothing to disclose
Timo Alexander Auer: Nothing to disclose
Federico Collettini: Nothing to disclose

**Dual-phase Cone-Beam CT (DP-CBCT) role as imaging navigation guidance in HCC lesions treatment with trans-arterial chemoembolization (TACE): a single centre experience**
*N. Rossini*[1], C. Floridi[1], M. Macchini[1], L. M. Cacioppa[1], A. Felicioli[1], C. Mincarelli[2], R. Candelari[1], A. Giovagnoni[1]; [1]Ancona/IT, [2]Macerata/IT
*(nicolorossini44@gmail.com)*

**Purpose or Learning Objective:** To evaluate how intra-procedural DP-CBCT navigation guidance influences TACE success rate in terms of residual disease in follow-up imaging.
**Methods or Background:** This retrospective analysis includes all patients with HCC treated with TACE (cTACE or DEB-TACE) in our centre between January 2017 and January 2024 with at least 1 month of imaging (CT or MRI) follow-up available. All patients had a recent baseline CT or MRI before TACE. Patients were divided in two groups, the first one included patients with DP-CBCT performed intra-procedurally during TACE (DP-CBCT group), the second included patients with no CBCT performed during treatment (no-DP-CBCT group). The two groups were similar in vascular anatomy, lesions number, morphology and localization. Response to treatment was evaluated in imaging follow-up with mRECIST criteria. The two groups were compared for treatment response after TACE in terms of residual disease in follow-up imaging.
**Results or Findings:** 152 patients were included in the study (M:F 112:40). 82 patients were included in the DP-CBCT group whereas 70 in the no-DP-CBCT group. Residual disease was of 26.9% in DP-CBCT group and of 63.0% in no-DP-CBCT group. A significant difference in terms of residual disease was observed between the two groups (p<0.05). Significant lower cases of residual disease were present in DP-CBCT group.
**Conclusion:** DP-CBCT imaging navigation guidance improves significantly success rate in TACE, ensuring a better visualization of HCC lesions feeding vessels and a consequent higher possibility of complete treatment of the nodules without residual disease.
**Limitations:** The main limitation of this study is the brief follow up imaging that should be extended in future studies.
**Funding for this study:** This research received no external funding.
**Ethics committee - additional information:** All the procedures performed in studies involving human participants were in accordance with the ethical standards of the institutional and/or national research committee and with the 1964 Declaration of Helsinki and its later amendments or comparable ethical standards. This study obtained the approval of the Internal Review Board (IRB) of University Politecnica Delle Marche.
**Author Disclosures:**
Cinzia Mincarelli: Nothing to disclose
Alessandro Felicioli: Nothing to disclose
Chiara Floridi: Nothing to disclose
Laura Maria Cacioppa: Nothing to disclose
Marco Macchini: Nothing to disclose
Roberto Candelari: Nothing to disclose
Andrea Giovagnoni: Nothing to disclose
Nicolo' Rossini: Nothing to disclose

**Identifying Key Predictors of Mortality and Liver Decompensation in Hepatocellular Carcinoma Patients Treated with Yttrium-90 Radioembolization**
M. Arabi, H. Alghamdi, *A. A. F. Almesned*, O. Alanazi, M. Alghamdi, M. Bukhaytan, M. Alkhalaf, M. Almaimoni, N. Alagrafy; Riyadh/SA
*(azizmesned@gmail.com)*

**Purpose or Learning Objective:** This study aimed to identify the predictors of mortality and liver decompensation in patients with HCC treated with Y-90 radioembolization.
**Methods or Background:** A retrospective analysis of 140 patients with HCC who underwent Y-90 radioembolization was conducted. Kaplan–Meier and multivariate Cox regression analyses were performed to identify the significant predictors of mortality.
**Results or Findings:** The cohort comprised 69.3% males with a mean age of 71.3 ±11.9 years. Most patients (73.6%) had Child-Pugh class A cirrhosis and 34.3% had BCLC stage B disease. Among the 140 patients, 57.1% died after treatment and liver decompensation was recorded in 39.2%. The median survival was significantly longer in those without liver decompensation (3.2 vs 0.7 years, p<0.001). Multivariate analysis revealed that male sex (adjusted odds ratio [aOR] 5.889, p=0.009), cirrhosis (aOR 6.82, p=0.047), and international normalized ratio (INR) (aOR 316.664, p=0.013) were independent predictors of liver decompensation. Cox regression analysis revealed several significant predictors of mortality. Ascites (HR 2.012, 95% CI, 1.122–3.61; p=0.019), portal vein invasion (HR 1.695, 95% CI, 1.057–2.718; p=0.029), and diabetes mellitus (HR 1.823, 95% CI, 1.017–3.265; p=0.044) were associated with increased mortality risk. Conversely, non-multifocal HCC (HR 0.593, 95% CI, 0.369–0.955; p=0.031), treatment of the liver lobe other than the right lobe (HR, 0.482; 95% CI 0.236–0.986, p=0.046), and age ≥60 years (HR 0.288, 95% CI, 0.139–0.597; p=0.001) were associated with a reduced risk of mortality.
**Conclusion:** This study identified the key predictors of mortality in patients with HCC undergoing Y-90 radioembolization, potentially improving patient selection and management strategies.
**Limitations:** While this study provides valuable insights, several limitations should be acknowledged. The retrospective nature of the study introduces potential biases in patient selection and data collection. The lack of post-infusion dosimetry limits the precision of dose-response analyses.
**Funding for this study:** The study was not supported by funding.
**Ethics committee - additional information:** The study was approved by the institutional review board, and the need for informed consent was waived. This study was conducted in accordance with the 2010 guidelines of the Declaration of Helsinki.
**Author Disclosures:**
Omar Alanazi: Nothing to disclose
Muath Almaimoni: Nothing to disclose
Mohammed Bukhaytan: Nothing to disclose
Mohammed Alkhalaf: Nothing to disclose
Meshari Alghamdi: Nothing to disclose
Hamdan Alghamdi: Nothing to disclose
Abdulaziz Abdullah F Almesned: Nothing to disclose
Nawaf Alagrafy: Nothing to disclose
Mohammad Arabi: Nothing to disclose

**Application of cross-modality image registration system for localising intraoperative colorectal cancer liver metastases during ablation**
*X. Wu*; Hangzhou/CN
*(Wuxia1981@zju.edu.cn)*

**Purpose or Learning Objective:** Localisation of target tumours under CT guidance can be challenging due to insufficient soft tissue resolution and metal artifacts. This study aims to validate the accuracy of the automatic image registration system (AIRS) in localising target lesions throughout the CT-guided CRLM ablation procedure, thereby exploring a novel guidance method for interventional procedures.
**Methods or Background:** This retrospective, single-center study included patients with CRLM who underwent CT-guided liver ablation between January 2021 and August 2023. Three experienced physicians collectively annotated the visibility and lesion centre positions of CRLMs on both the preprocedural contrast-enhanced MRI and intraoperative CT image series, which served as the ground truth. The AIRS and two junior physicians delineated the lesion centre positions in the same CT sequences. The localisation errors of the AIRS and junior physicians were analysed using the non-parametric Kruskal–Wallis test for one-way analysis.
**Results or Findings:** One hundred and twenty consecutive patients with 224 CRLMs treated across 128 sessions were enrolled. There were 128 pairs of MR-pCT (pre-procedural CT) multi-modal registrations and 1,008 pairs of pCT-iCT (intra-procedural) mono-modal registrations. AIRS demonstrated superior localisation error than the physician group in localising lesions suboptimal visible on pCT (5.94±2.61 mm vs 8.04±5.32 mm, p=0.006), lesions excellently visible on iCT (5.14±2.65 mm vs 6.15±3.84 mm, p=0.01) and lesions suboptimal visible on iCT (6.13±2.80 mm vs 8.94±4.60 mm, p＜0.001).
**Conclusion:** Compared with less experienced physicians, an AIRS can quickly and accurately locate target lesions in CT-guided colorectal cancer liver metastasis ablation procedures, especially for lesions with poor visibility, thus paving the way for a new navigation method in colorectal cancer liver metastasis ablations.
**Limitations:** This was a retrospective investigation with a relatively small sample size, which may have restricted the generalisability of our findings.
**Funding for this study:** None
**Ethics committee - additional information:** This single-centre retrospective study was approved by the local ethics committee and was exempted from informed consent.
**Author Disclosures:**
Xia Wu: Nothing to disclose

**Deep Learning-Based Reconstruction and Superresolution for MR-guided Thermoablation**
*M. T. Winkelmann*[1], J. Kuebler[1], S. Gassenmaier[1], D. Nickel[2], K. Nikolaou[1], S. Afat[1], R. Hoffmann[1]; [1]Tuebingen/DE, [2]Erlangen/DE
*(moritz.winkelmann@med.uni-tuebingen.de)*

**Purpose or Learning Objective:** This study explores the impact of deep learning-enhanced image generation for T1-weighted volume-interpolated breath-hold examinations (DL-VIBE) on image quality and procedural parameters during MR-guided thermoablation of liver malignancies, compared to standard VIBE images (SD-VIBE).
**Methods or Background:** 34 consecutive patients (mean age: 65.4 ± 11.5 years, women: n=13) with liver malignancies underwent MR-guided microwave ablation using a 1.5 T MR scanner. Intraprocedural VIBE sequences (SD-VIBE) were used to monitor needle position and assess the ablation zone. The raw T1-weighted VIBE data were retrospectively processed with a deep learning algorithm (DL-VIBE) to reduce noise and improve sharpness. Two interventional radiologists independently evaluated the image sets in a blinded manner, comparing DL-VIBE with unprocessed SD-VIBE images. Criteria assessed included diagnostic confidence, image quality, noise, artifacts, and sharpness. Interrater agreement was analyzed, and noise maps were created to evaluate signal-to-noise ratio improvements.
**Results or Findings:** DL-VIBE significantly improved overall image quality, reduced noise and artifacts, and enhanced the sharpness of liver contours and portal vein branches compared to SD-VIBE (P<0.001). Additionally, DL-VIBE improved imaging of the interventional path, needle tip detectability, and diagnostic confidence in needle positioning and the ablation zone (P<0.001), with high interrater agreement ($\kappa$ = 0.86). Quantitative noise maps demonstrated a higher signal-to-noise ratio, and the reconstruction process took approximately 4 seconds, reducing breath-hold time by 2 seconds.
**Conclusion:** DL-VIBE significantly enhances image quality and diagnostic confidence during MR-guided thermal ablation procedures, offering time savings and potential improvements in patient outcomes.
**Limitations:** - Small number of patients. - Sequences were retrospectively processed, not used during actual interventions. - Image quality assessments were retrospective and may differ from real-time evaluations. - Time savings are theoretical due to the retrospective study design, with uncertainty about feasibility during actual interventions.
**Funding for this study:** No funding was received for this study
**Ethics committee - additional information:** This retrospective study was approved by the institutional review board (Eberhard Karls University of Tübingen, project number: 055/2017BO2)
**Author Disclosures:**
Konstantin Nikolaou: Nothing to disclose
Moritz T. Winkelmann: Nothing to disclose
Saif Afat: Nothing to disclose
Rüdiger Hoffmann: Nothing to disclose
Dominik Nickel: Nothing to disclose
Jens Kuebler: Nothing to disclose
Sebastian Gassenmaier: Nothing to disclose

**Machine learning using MR imaging radiomics and clinical features can predict the response of large hepatocellular carcinoma to transarterial radioembolization**
O. Sarioğlu, A. Canturk, *R. C. Yarol*, H. Gulmez, E. Derebek, A. Gülcü; Izmir/TR
*(raifyarol@gmail.com)*

**Purpose or Learning Objective:** To evaluate the potential of machine learning-based models for predicting the response of large hepatocellular carcinoma to transarterial radioembolization
**Methods or Background:** A total of 49 patients (38 responder and 11 non-responder) were included in the study. Laboratory results and clinical conditions were collected. Treatment response was assessed according to mRECIST criteria from the 3-month follow-up MR examinations. Complete or partial response was categorized as the responder group, while stable or progressive disease was classified as the non-responder group. Radiomics features were extracted from contrast-enhanced T1-weighted images (CE-T1) and T2-weighted images (T2WI). 141 radiomics features were obtained from each lesion. Classification learning models were used to create prediction models for TARE response. 5-fold cross-validation technique was utilized to identify the prediction rates of treatment response.
**Results or Findings:** Number of radiomics features demonstrated statistically significant differences between the groups are 9 and 12 on T2W and CE-T1 images, respectively. The model based on radiomics features obtained from CE-T1 images demonstrated an accuracy rate of %79.6 to predict response with an AUC of 0.92. The sensitivity and specificity rates were %79 and %100, respectively. The accuracy and AUC rates of the model using radiomics features extracted from T2W images were %79.6 and 0.77, respectively. Sensitivity and specificity rates of the model were %80 and %67, respectively. When only clinical and laboratory parameters were used, the model showed an accuracy rate of %77.6 and an AUC of 0.65. The sensitivity and specificity values of the clinical and laboratory model were %79 and %50, respectively. Another model using both clinical and CE-T1 radiomics features showed an accuracy rate of %73.5
**Conclusion:** Machine learning-based radiomics models based on MRI can predict the response of large hepatocellular carcinoma to transarterial radioembolization
**Limitations:** None
**Funding for this study:** None
**Ethics committee - additional information:** Ethics committee approval obtained
**Author Disclosures:**
Aytaç Gülcü: Nothing to disclose
Ali Canturk: Nothing to disclose
Raif Can Yarol: Nothing to disclose
Orkun Sarioğlu: Nothing to disclose
Hakan Gulmez: Nothing to disclose
Erkan Derebek: Nothing to disclose

**09:30-11:00** **Research Stage 3**

### Research Presentation Session: Cardiac

### RPS 1303

### The evolving impact of artificial intelligence (AI) in cardiac imaging

**Moderator**
A. Isaak; Bonn/DE

**Formulation of a predictive model for total cardiac volume (TCV) estimation: Optimizing donor-recipient size matching and outcomes**
*S. Gowda*, V. Raj, R. Kothari; Bengaluru/IN
*(srjgwd@gmail.com)*

**Purpose or Learning Objective:** Accurate donor heart size measurement is crucial for successful heart transplantation (HT). Traditional weight-based donor-to-recipient (D-R) size matching in paediatric HT has poor correlation with cardiac size and significantly restricts the donor pool. The aim of the study is to develop a novel predictive model to accurately calculate Total Cardiac Volume (TCV) tailored to the Indian population, aiming to expand the donor pool and reduce size mismatches.
**Methods or Background:** This multi-centre study incorporated paediatric and young adults (ages 0-30) with normal CT chest angiograms. TCV was predicted using common variables such as weight, height, gender and cardiac width on chest radiograph (CXR) with CT derived TCV (3D segmentation) as the gold standard. Three predictive models were analysed, and subjects were split into training and testing data. Model A- weight only Model B- weight, height, gender and age Model C- Model B plus horizontal cardiac width from CXR.
**Results or Findings:** Model C showed highest accuracy in predicting TCV with an $R^2$ of 0.94 for training data and 0.91 for testing data, with mean absolute percentage error (MAPE) of 3%. Model A was weakest with an $R^2$ of 0.82 for training data, 0.68 for testing data, and a MAPE of 6.3%.
**Conclusion:** TCV can be accurately predicted using readily available donor metrics. The proposed D-R TCV matching model can significantly expand the donor pool and improve size matching in paediatric heart transplantation in India.

**Limitations:** Single centre study, which may also have an in-built case selection bias.
**Funding for this study:** No funding was provided for this study.
**Ethics committee - additional information:** Owing to the retrospective nature of the study, ethical committee approval was waived off by the institutional ethics committee.
**Author Disclosures:**
Richa Kothari: Nothing to disclose
Vimal Raj: Nothing to disclose
Suraj Gowda: Nothing to disclose

### Prospective Comparison of Automated vs. Human-Guided Cardiac MRI Planning

*C. G. Glessgen*[1], L. A. Crowe[1], J. Wetzl[2], M. Schmidt[2], S. S. Yoon[2], J-P. Vallee[1], J-F. Deux[1]; [1]Geneva/CH, [2]Erlangen/DE

**Purpose or Learning Objective:** Cardiac MRI (CMR) is demanding due to the number of planning steps and parameters requiring continuous monitoring. The high mistake risk can impact procedure quality, scan times, and data homogeneity. The impact of an AI-based automated CMR planning software on procedure errors and scan times compared to human-guided examinations is evaluated.
**Methods or Background:** Consecutive patients undergoing non-stress CMR were prospectively enrolled into two acquisition modes: manual or automated utilizing prototype software (Siemens Healthineers, Erlangen, Germany). Patients with pacemakers or targeted indications were excluded. All underwent the same CMR protocol with contrast administration, in breath-hold (BH) or free breathing (FB). Supervising radiologists recorded procedure errors (plane prescription, forgotten views, incorrect propagation of a cardiac plane, field-of-view mismanagement). Scan times and Dead Phase (non-acquisition portion) were computed from scanner logs. Most data were non-normally distributed and compared using nonparametric tests.
**Results or Findings:** Eighty-two patients (mean age, 51.6 years; 56 male) were included. Forty-four patients underwent automated CMR and 38 manual CMR. The rate of procedure errors per CMR was lower (p=0.01) in automated (0.45) than in manual (1.13). The ratio of error-free examinations was higher (p=0.03) in automated (31/44; 70.5%) than in manual (17/38; 44.7%). Automated studies were shorter than manual studies in FB (30.3 vs. 36.5 minutes, p<.001) but had similar durations in BH (42.0 vs. 43.5 minutes, p=0.42). Dead Phase was lower in automated studies for both FB and BH strategies (p<.001).
**Conclusion:** AI-based automation performed cardiac MRI studies at a clinical level with fewer planning errors and improved efficiency compared to human planning.
**Limitations:** No reproductibility analysis of plane adjustments between radiologists was performed. Radiologists could not realistically be blinded to the study arm as automatic acquisitions were performed with almost no visible human interaction.
**Funding for this study:** None
**Ethics committee - additional information:** All patients gave informed consent
**Author Disclosures:**
Lindsey A. Crowe: Nothing to disclose
Jean-Paul Vallee: Nothing to disclose
Carl Guillaume Glessgen: Nothing to disclose
Seung Su Yoon: Employee: Siemens Healthineers
Michaela Schmidt: Employee: Siemens Healthineers
Jean-François Deux: Nothing to disclose
Jens Wetzl: Employee: Siemens Healthineers

### Predicting Mortality After Transcatheter Aortic Valve Replacement Using AI- Based Fully Automated Left Atrioventricular Coupling Index

*E. Zsarnóczay*[1], A. Varga-Szemes[2], U. J. Schoepf[2], S. Rapaka[3], N. Fink[2], M. Vecsey-Nagy[2], P. Sharma[3], P. Maurovich-Horvat[1], T. S. Emrich[2]; [1]Budapest/HU, [2]Charleston, SC/US, [3]Princeton, NJ/US

**Purpose or Learning Objective:** To determine whether artificial intelligence (AI)–based fully automated assessment of left atrioventricular coupling index (LACI) can provide incremental value above other traditional risk factors for predicting mortality among patients with severe aortic stenosis (AS) undergoing coronary CT angiography (CCTA) before transcatheter aortic valve replacement (TAVR).
**Methods or Background:** This retrospective study evaluated patients with severe AS who underwent CCTA examination before TAVR between 2014 and 2019. An AI-prototype software fully automatically calculated left atrial (LA) and left ventricular (LV) end-diastolic volumes and LACI was defined as the ratio between them. Clinical parameters, the Society of Thoracic Surgeons Predicted Risk of Mortality (STS-PROM) risk score, and all-cause mortality after TAVR were recorded. Uni- and multivariate Cox proportional hazard methods were used to identify the predictors of mortality in models adjusting for relevant significant parameters, STS-PROM score, and patients with preserved LV ejection fraction (EF).
**Results or Findings:** A total of 656 patients (77 years [IQR, 71-84 years]; 387 [59.0%] male) were included. The all-cause mortality rate was 21.6% over a median follow-up time of 24 (10–40) months. When adjusting for clinical confounders, LACI≥43.7% was found to independently predict mortality (adjusted HR, 1.52, [95CI: 1.03,2.22]; p=0.032). After adjusting for the STS-PROM score in a separate model, LACI ≥43.7% remained an independent prognostic parameter (adjusted HR, 1.47, [95CI: 1.03,2.08]; p=0.031). In a sub-analysis of patients with preserved LVEF, LACI remained a significant predictor (adjusted HR, 1.72 [95CI: 1.02,2.89]; p=0.042).
**Conclusion:** AI-based fully automated assessment of LACI can be used independently to predict mortality in patients undergoing TAVR, including those with preserved LVEF.
**Limitations:** This study was performed in a single-center and single-vendor setting, using an AI-powered software prototype, selection bias may exist because only patients with available outcomes data were included.
**Funding for this study:** Not applicable.
**Ethics committee - additional information:** Not applicable.
**Author Disclosures:**
Emese Zsarnóczay: Nothing to disclose
Puneet Sharma: Employee: Siemens Healthineers
Pál Maurovich-Horvat: Nothing to disclose
Milán Vecsey-Nagy: Nothing to disclose
Uwe Joseph Schoepf: Research/Grant Support: Bayer, Bracco, Elucid Bioimaging, Guerbet, HeartFlow
Saikiran Rapaka: Employee: Siemens Healthineers
Tilman Stephan Emrich: Consultant: Siemens Medical Solutions USA Inc
Nicola Fink: Nothing to disclose
Akos Varga-Szemes: Research/Grant Support: Siemens

### Deep Learning Denoising Algorithm for Improved Assessment of Coronary Arteries in Transcatheter Aortic Valve Implantation CT Imaging

*L. R. M. Lanzafame*[1], T. D'Angelo[1], A. Othman[2], C. Booz[3]; [1]Messina/IT, [2]Mainz/DE, [3]Frankfurt/DE
*(ludovicalanzafame@gmail.com)*

**Purpose or Learning Objective:** This study aimed to evaluate the impact of a deep learning-based denoising (DLD) technique on image quality and diagnostic accuracy for the assessment of coronary arteries in pre-procedural transcatheter aortic valve implantation (TAVI) CT planning.
**Methods or Background:** A retrospective analysis was conducted on 200 patients with severe aortic stenosis who underwent CT scans for TAVI planning between October 2022 and April 2024. Conventional images were reconstructed, and denoised images were generated using DLD model. Objective image quality was assessed by measuring the mean Hounsfield unit (HU) and standard deviation (SD) in the aortic root, coronary arteries, and subcutaneous fat to calculate noise, signal-to-noise ratio (SNR), and contrast-to-noise ratio (CNR). Two independent readers subjectively evaluated sharpness, noise, vascular contrast, and overall image quality using a 5-point Likert scale. Diagnostic performance was compared between original and denoised images by assessing accuracy, sensitivity, specificity, positive predictive value (PPV), and negative predictive value (NPV), using invasive coronary angiography as the reference standard.
**Results or Findings:** Denoised images demonstrated significantly improved SNR (37.5 ± 12.8 vs. 12.3 ± 4.1) and CNR (45.3 ± 15.4 vs. 14.7 ± 4.4), along with reduced noise levels (16.9 ± 7.9 vs. 47.9 ± 11.6 HU) (all p<0.001). Subjective evaluations also favored denoised images in terms of sharpness, noise reduction, contrast, and overall quality (all p<0.001). DLD reconstructions revealed higher diagnostic performance, showing a on a per-segment basis sensitivity of 95.9%, specificity of 94.3%, PPV of 86.5%, NPV of 98.4%, and accuracy of 94.8%.
**Conclusion:** The DLD algorithm significantly improves image quality and diagnostic accuracy in pre-TAVI CT imaging for coronary artery evaluation.
**Limitations:** The retrospective design prevented evaluation of image quality at reduced radiation doses. Furthermore, the results are specific to our acquisition protocol.
**Funding for this study:** This research did not receive external funding.
**Ethics committee - additional information:** The study was approved by the Ethics Committee of Johannes Gutenberg University of Mainz (Ref. Nr. 2022-16477_1)
**Author Disclosures:**
Christian Booz: Speaker: Siemens Healthineers
Ludovica Rosa Maria Lanzafame: Nothing to disclose
Tommaso D'Angelo: Speaker: Philips Speaker: Bracco
Ahmed Othman: Nothing to disclose

**A recommendation: test-retest reliability of radiomic features in myocardial T1 and T2 mapping**

*M. Manzke*[1], F. C. Laqua[2], B. Böttcher[1], A-C. Klemenz[1], M-A. Weber[1], B. Baeßler[2], F. G. Meinel[1]; [1]Rostock/DE, [2]Würzburg/DE
*(Mathias.Manzke@med.uni-rostock.de)*

**Purpose or Learning Objective:** To investigate the reproducibility of radiomic features in myocardial native T1 and T2 mapping.
**Methods or Background:** Cardiac MRI T1 maps from 50 healthy volunteers (29 women and 21 men, mean age 39.4 ± 13.7 years) underwent two identical cardiac MRI examinations at 1.5T. The protocol included native T1 and T2 mapping in both short-axis and long-axis orientation. For T1 mapping, we investigated standard (1.9 x 1.9 mm) and high (1.4 x 1.4 mm) spatial resolution. After manual segmentation of the left ventricular myocardium, 100 radiomic features from seven feature classes were extracted and analyzed. Test–retest repeatability of radiomic features was assessed using the intraclass correlation coefficient (ICC) and classified as poor (ICC <0.50), moderate (0.50–0.75), good (0.75–0.90) and excellent (>0.90).
**Results or Findings:** For T1 maps acquired in short-axis orientation at standard resolution, repeatability was excellent for 6 features, good for 29 features, moderate for 19 features and poor for 46 features. We identified 15 features from 6 classes which showed good to excellent reproducibility for T1 mapping in all resolutions and all orientations. For short-axis T2 maps, repeatability was excellent for 6 features, good for 25 features, moderate for 23 features and poor for 46 features. 12 features from 5 classes were found to have good to excellent repeatability in T2 mapping independent of slice orientation.
**Conclusion:** We have identified a subset of radiomic features with good to excellent repeatability independent of slice orientation and spatial resolution. We recommend using these features for further radiomics research in myocardial T1 and T2 mapping.
**Limitations:** This study was limited to healthy volunteers. The reproducibility of radiomic features in patients with diffuse or focal myocardial disease cannot be directly concluded.
**Funding for this study:** The study was in part funded by the Federal Ministry of Education and Research (BMBF) through the Network University Medicine „NUM 2.0“ (grant number 01KX2121).
**Ethics committee - additional information:** This study was approved by the institutional review board and written informed consent was obtained from all volunteers prior to enrollment.
**Author Disclosures:**
Fabian Christopher Laqua: Nothing to disclose
Benjamin Böttcher: Nothing to disclose
Mathias Manzke: Nothing to disclose
Bettina Baeßler: Author: This study has been supported by the Deutsche Forschungsgemeinschaft (DFG, German Research Foundation) within the Priority Programme SPP 2177 Radiomics (BA 6438/4–2) and by the Federal Ministry of Education and Research (BMBF; “SWAG” project). BB is founder and CEO of Lernrad GmbH and has received speaker fees by Bayer Vital GmbH.
Ann-Christin Klemenz: Nothing to disclose
Felix G. Meinel: Author: Unrelated to this work, Dr. Meinel has received institutional research support from GE Healthcare and speaker’s honoraria from GE Healthcare, Circle Cardiovascular Imaging and Bayer Vital.
Marc-André Weber: Nothing to disclose

**Reproducibility of an AI-assisted plane positioning tool for cardiac MRI**

*B. Böttcher*[1], K. K. Deyerberg[1], A-C. Klemenz[1], L-M. Watzke[1], M. Gorodezky[2], M. Manzke[1], M-A. Weber[1], F. G. Meinel[1]; [1]Rostock/DE, [2]Munich/DE
*(benjamin.boettcher@gmx.net)*

**Purpose or Learning Objective:** Plane positioning in cardiac magnetic resonance imaging (cMRI) is crucial for diagnostic image quality and comparability of cardiac functional parameters in follow-up exams. Manual planning is influenced by user’s training making it susceptible for inter-reader variability and errors. This prospective cohort study aims to investigate the reproducibility of an artificial intelligence-based planning approach against state-of-the-art manual plane prescription.
**Methods or Background:** 25 healthy participants (mean age 41.5, range: 23-65 years, mean BMI 25.2 kg/m²) underwent two identical cMRI exams on a 1.5T scanner (Signa Artist, GE HealthCare). Short axis, 2-, 3- and 4-chamber planes (FOV: 34x34cm2, matrix size: 200x224, slice thickness: 8mm) were acquired using an AI-based planning tool (TeslaFlow prototype, GE HealthCare) and manual planning. Short axis left ventricular volumetric analysis (end-diastolic volume (EDV), end-systolic volume (ESV), stroke volume (SV) and ejection fraction (EF)) were performed using an established post-processing software (cvi42, Circle Cardiovascular Imaging). The Wilcoxon matched-pairs signed rank test with a significance level of p≤0.05 was used to compare the first to the second exam for both the manual and automated planning.
**Results or Findings:** Volumetric parameters calculated on manual and AI-assisted planned images showed following median of differences between both scans: EDV -5.0ml (p=0.220), -2.8ml (p=0.474); ESV 0.1ml (p=0.560), 2.0ml (p=0.367); SV -3.6ml (p=0.096), -4.2ml (p=0.043) and EF -1.2% (p=0.329), -2.8% (p=0.045), respectively. The only statistically significant differences were observed in SV and EF for AI-based planning, though the deviation is not clinically relevant.
**Conclusion:** AI-based planning for cMRI showed high reproducibility without clinically relevant variability between follow-up scans. This novel technique can simplify and accelerate cMRI maintaining high diagnostic quality.
**Limitations:** This study was conducted on a cohort of healthy individuals at a single MRI scanner, provided by a single vendor.
**Funding for this study:** None.
**Ethics committee - additional information:** The study was designed as a prospective, single-center cohort study and approved by the responsible institutional review board of the Medical University Center of Rostock.
**Author Disclosures:**
Benjamin Böttcher: Nothing to disclose
Margarita Gorodezky: Employee: GE HealthCare
Mathias Manzke: Nothing to disclose
Ann-Christin Klemenz: Nothing to disclose
Felix G. Meinel: Nothing to disclose
Marc-André Weber: Nothing to disclose
Lena-Maria Watzke: Nothing to disclose
Karolin Kristina Deyerberg: Nothing to disclose

**Accelerated Deep Learning-Based Function Assessment in Cardiovascular Magnetic Resonance**

*F. Fanelli*, D. De Santis, L. Pugliese, G. G. Bona, C. Santangeli, T. Polidori, G. Tremamunno, D. Caruso, A. Laghi; Rome/IT
*(f.fanelli@uniroma1.it)*

**Purpose or Learning Objective:** Cardiovascular magnetic resonance (CMR) is the reference standard for the assessment of cardiac function, achieved through conventional balanced steady-state free precession (bSSFP) cine sequences, which represent a considerable part of the CMR exams, contributing to patient discomfort. The aim of our study was to evaluate diagnostic accuracy and image quality of deep-learning(DL)cine sequences for LV and RV parameters compared to bSSFP cine sequences in CMR.
**Methods or Background:** From January to April 2024, patients with clinically indicated CMR were prospectively included. LV and RV were segmented from short-axis bSSFP and DL cine sequences. LV and RV end-diastolic volume, end-systolic volume, stroke volume, ejection fraction, and LV end-diastolic mass were calculated. The acquisition time of both sequences was registered. Results were compared with paired-samples t-test or Wilcoxon signed-rank test. Agreement between DL cine and bSSFP was assessed using Bland-Altman plots. Image quality was graded by two readers based on blood-to-myocardium contrast, endocardial edge definition, and motion artifacts, using a 5-point Likert scale (1= insufficient quality; 5= excellent quality).
**Results or Findings:** Sixty-two patients were included (mean age: 47±17 years, 41 men). No significant differences between DL cine and bSSFP were found for all LV and RV parameters (P≥ .176). DL cine was significantly faster (1.35 ±.55 m vs 2.83 ± .79 m; P< .001). The agreement between DL cine and bSSFP was strong, with near-zero bias and good limits of agreement. Overall image quality was comparable (median: 5, IQR: 4-5; P= .330), while endocardial edge definition of DL cine (median: 4, IQR: 4-5) was lower than bSSFP (median: 5, IQR: 4-5; P= .002).
**Conclusion:** DL cine allows fast and accurate quantification of LV and RV parameters and comparable image quality with conventional bSSFP.
**Limitations:** Not applicable
**Funding for this study:** No funding was provided for this study.
**Ethics committee - additional information:** This study has been approved by local Ethics committee .
**Author Disclosures:**
Luca Pugliese: Nothing to disclose
Damiano Caruso: Nothing to disclose
Curzio Santangeli: Nothing to disclose
Federica Fanelli: Nothing to disclose
Domenico De Santis: Nothing to disclose
Giuseppe Tremamunno: Nothing to disclose
Tiziano Polidori: Nothing to disclose
Andrea Laghi: Nothing to disclose
Giovanna Grazia Bona: Nothing to disclose

**Super-Resolution Deep Learning Reconstruction to Improve the Accuracy of CT Fractional Flow Reserve: Comparison to Model-based Iterative Reconstruction**

*N. Tomizawa*, Y. Nozaki, R. Fan, Y. Kawaguchi,, K. Takamura, F. Shinichiro, K. Kumamaru, T. Minamino, S. Aoki; Bunkyo-Ku/JP
*(tomizawa-tky@umin.ac.jp)*

**Purpose or Learning Objective:** The purpose of this study was to compare the diagnostic performance of CT fractional flow reserve (CT-FFR) using model-based iterative reconstruction (MBIR) and super-resolution deep learning reconstruction (SR-DLR) to detect functionally significant stenosis as assessed by invasive FFR.
**Methods or Background:** This single-center retrospective study included 79 patients (mean age, 70 years ± 11 [SD]; 57 men) who underwent coronary CT angiography showing intermediate stenosis (30%–70% stenosis) and subsequent invasive FFR between February 2022 and March 2024. Vessels with heavy calcification were not excluded from the analysis. Computational fluid dynamics was used to calculate the CT-FFR using MBIR and SR-DLR images. Per-vessel diagnostic performance to detect FFR ≤0.80 in coronary angiography was compared by analyzing receiver operating characteristic (ROC) curves.
**Results or Findings:** Of the 98 vessels evaluated, 46 vessels (47%) had functionally significant stenosis. The median (interquartile range) calcium score was 462 (134–932). CT-FFR values calculated using both MBIR (mean difference: −0.088; 95% CI: −0.129, −0.048; p <0.001) □□d SR-DLR (mean difference: −0.026; 95% CI: −0.050, −0.002; p = 0.03) w□r□ u□d□r□□tim□t□d compared to invasive FFR. The area under the ROC curve to diagnose functionally significant stenosis was higher for SR-DLR (0.88; 95% CI: 0.80, 0.95) than for MBIR (0.76; 95% CI: 0.67, 0.86; p = 0.003). CT-FFR calculated using SR-DLR had improved diagnostic accuracy (88% vs. 70%, p <0.001) and specificity (87% vs. 63%, p <0.001) over MBIR, but had similar sensitivity (89% vs. 78%, p = 0.06).
**Conclusion:** SR-DLR images improved the diagnostic performance of CT-FFR over MBIR images in detecting functionally significant stenosis as assessed by invasive FFR.
**Limitations:** This study is retrospective and used a single CT vendor. Multi-vendor multi-center study is necessary to confirm the findings.
**Funding for this study:** None
**Ethics committee - additional information:** Approved by the Ethics Committee of Juntendo University on May 2, 2024 (No. E23-0040-H02)
**Author Disclosures:**
Fujimoto Shinichiro: Nothing to disclose
Yuko Kawaguchi,: Nothing to disclose
Shigeki Aoki: Nothing to disclose
Kazuhisa Takamura: Nothing to disclose
Tohru Minamino: Nothing to disclose
Yui Nozaki: Nothing to disclose
Nobuo Tomizawa: Nothing to disclose
Kanako Kumamaru: Nothing to disclose
Ruiheng Fan: Nothing to disclose

**Evaluating the Feasibility of a Customised GPT-4 Model for Extracting CAD-RADS Classification from Coronary CT Angiography Reports**

*V. Vingiani*, B. Proner, N. Cortellini, R. Valletta, T. Gorgatti, A. Posteraro, V. Corato, M. Bonatti; Bolzano/IT
*(vincenzovingiani@gmail.com)*

**Purpose or Learning Objective:** This study assessed the feasibility of a customized GPT-4 model in categorising cardiac radiological reports using the Coronary Artery Disease Reporting and Data System (CAD-RADS) classification.
**Methods or Background:** A customised GPT-4 model was developed using the CAD-RADS 2.0-2022 guidelines, provided as a PDF, and fine-tuned on 30 clinical scenarios. The model was tested on 118 anonymized Coronary CT Angiography (CCTA) reports. Data included patient metrics and report details (e.g., length, conclusions). The reports were also reviewed by a radiologist with 9 years of experience, who categorised them according to the CAD-RADS classification. The time required for manual assessment was recorded. The GPT-4 model's performance was evaluated using Cohen's kappa for agreement and the Wilcoxon test for comparing processing times between the model and the radiologist.
**Results or Findings:** The mean patient age was 59.6 years (±10.9), with 41% women. Reports were authored by 10 radiologists, with 88% in Italian and 12% in German. The median report length was 1,456 characters, with conclusions in 76% of reports. The GPT-4 model showed substantial agreement with the radiologist, achieving a Cohen's kappa of 0.79 (95% CI: 0.68 - 0.89). It significantly reduced processing time, averaging 16 seconds per report compared to 57 seconds for the radiologist (P < 0.0001).
**Conclusion:** These findings suggest that a customized GPT-4 model is a promising tool for autonomously categorising radiological findings using the CAD-RADS classification when the original report does not include it, thereby offering a time-efficient alternative. Implementing such a system could assist clinicians and cardiologists in consistently interpreting reports by providing CAD-RADS classifications when they are not explicitly reported.
**Limitations:** The GPT model was fine-tuned using only 30 scenarios. A larger dataset could be used to improve the model's performance.
**Funding for this study:** None
**Ethics committee - additional information:** This study was conducted in accordance with the principles of the Declaration of Helsinki
**Author Disclosures:**
Andrea Posteraro: Nothing to disclose
Matteo Bonatti: Nothing to disclose
Valentina Corato: Nothing to disclose
Tommaso Gorgatti: Nothing to disclose
Bernardo Proner: Nothing to disclose
Riccardo Valletta: Nothing to disclose
Nino Cortellini: Nothing to disclose
Vincenzo Vingiani: Nothing to disclose

**DLR-based Motion Correction of Coronary CTA: Preliminary Evaluation**

F. Tatsugami[1], A. Streiff[2], T. Higaki[1], A. Labani[2], W. Fukumoto[1], S. El Ghannudi[2], K. Haioun[3], K. Awai[1], *M. Ohana*[2]; [1]Hiroshima/JP, [2]Strasbourg/FR, [3]Tokyo/JP
*(mickael.ohana@gmail.com)*

**Purpose or Learning Objective:** DLR-based Motion Correction (MC-DLR) for Coronary CTA has the potential to reduce/eliminate kinetic artifacts in CCTA more effectively than traditional algorithms, but its clinical impact is still unknown. We aim to evaluate the effect of MC-DLR on coronary luminal and stenosis assessment in a varied CCTA cohort.
**Methods or Background:** Sixty CCTA (20 with HR<60bpm, 20 with HR=60-75, 20 with HR>75) with various degrees of stenosis (50% CAD-RADS 1 & 2, 50% CAD-RADS 3 & 4) were retrospectively selected from 2 tertiary centers. All scans were acquired on 4th/5th-gen wide-area detector CT within 1 heartbeat. Best phase for each included CCTA was reconstructed without and with MC-DLR, using Super Resolution DLR with 1024 matrix-size. MC-DLR subdivides the data required for volume reconstruction into smaller time sections to estimate coronary artery motion. Four radiologists with varying levels of expertise independently and randomly reviewed all 120 datasets to: (A) grade the luminal/wall image quality using a 3-level scale, for the 9 coronary artery segments, and (B) assess CAD-RADS. Statistical analysis used descriptive and Bayesian approaches.
**Results or Findings:** For each reader and for the pooled analysis, overall luminal/wall image quality score was significantly better with MC-DLR than without (p<0.05). Per segment, the positive effect was more consistent on the RCA (improvement in 62% of cases) than on the LAD (44%) and the Cx (21%). Effect of MC-DLR was non-existent in cases with absent/minimal coronary kinetic artifacts. Non-significant changes in intra/inter-reader variability were noted in CAD-RADS 3/4.
**Conclusion:** MC-DLR significantly enhances coronary artery luminal and wall image quality in cases with moderate or severe kinetic artifacts, suggesting a potential clinical role in refining stenosis assessment when above >50%.
**Limitations:** Quantitative analysis with attenuation profile curves was not performed.
**Funding for this study:** No funding was received for this study.
**Ethics committee - additional information:** IRB from Strasbourg University Hospital
**Author Disclosures:**
Wataru Fukumoto: Nothing to disclose
Aissam Labani: Nothing to disclose
Soraya El Ghannudi: Nothing to disclose
Fuminari Tatsugami: Nothing to disclose
Toru Higaki: Nothing to disclose
Kazuo Awai: Nothing to disclose
Amandine Streiff: Nothing to disclose
Mickaël Ohana: Consultant: Boehringer Ingelheim Consultant: Canon Medical Systems Europe
Karim Haioun: Employee: Canon Medical Systems Japan

**Performance of AI-based automated coronary artery calcium density quantification on CT**

Y. J. Suh[1], C. Kim[2], W-S. Yoo[1], J. Y. Kim[3], S. Chang[1], C. H. Park[4], *S. J. Hong*[5], D. H. Yang[1], H. S. Yong[1]; [1]Seoul/KR, [2]Ansan/KR, [3]Daegu/KR, [4]Cheonan/KR, [5]Guri/KR

**Purpose or Learning Objective:** Coronary artery calcium (CAC) density on electrocardiogram (ECG)-gated CT has been suggested as an inverse prognostic marker for prediction of future adverse cardiovascular events. We aimed to evaluate the performance of artificial intelligence (AI)-based automated CAC density quantification on ECG-gated calcium scoring CT

(CSCT) and non-ECG-gated low-dose chest CT (LDCT), using multi-institutional datasets.
**Methods or Background:** A total of 1,540 pairs of CSCT-LDCT scans from a multicenter database were retrospectively included. AI-based automated CAC quantification was conducted on the CSCT and LDCT. For cases with CAC score>0, mean and peak CAC density was calculated from the labeled CAC. For peak CAC density factors, a value of 1 to 4 was assigned based on the measured peak density attenuation (1: 130-199HU; 2: 200-299HU; 3: 300-399HU; 4: >400HU). The reliability and agreement of the mean CAC density and peak CAC density categories obtained from the automated scoring were analyzed compared to manual measurement, using the intraclass correlation coefficient (ICC), Bland-Altman analysis, and weighted kappa (κ) statistics, respectively.
**Results or Findings:** A total of 808 CSCT scans and 579 LDCT scans were positive for CAC. Automated mean density measurement demonstrated excellent ICCs on CSCT and LDCT (0.988 [95% CI, 0.986-0.989] vs. 0.956 [95% CI, 0.948-0.962]). Mean bias with 95% limits of agreement for the mean density was 0.7 ± 22.4 on CSCT and 0.6 ± 27.4 on LDCT. In terms of peak density category, automated measurement on CSCT and LDCT exhibited excellent reliability with manual measurement (weighted κ 0.980 [95% CI, 0.969-0.991) and 0.964 [95% CI, 0.945-0.982]).
**Conclusion:** AI-based automated CAC quantification can provide accurate and reliable measurement of CAC density on CSCT and LDCT across multi-institutional datasets.
**Limitations:** Prognostic value of AI-based CAC density should be further investigated.
**Funding for this study:** The Researcher Supporting Program funded by Korean Society of Cardiovascular Imaging (KOSCI) and the National Research Foundation of Korea (NRF) grant funded by the Korea government (MSIT)(No. 2021R1A2C4002195)
**Ethics committee - additional information:** Approval numbers: KC22RIDI0156, 2021-12-027, 2022GR0064, 2021AS0371, 2022-01-001, 2021-0303, 2021-12-029, and 4-2021-1589
**Author Disclosures:**
Dong Hyun Yang: Nothing to disclose
Young Joo Suh: Nothing to disclose
Suyon Chang: Nothing to disclose
Chan Ho Park: Nothing to disclose
Jin Young Kim: Nothing to disclose
Su Jin Hong: Nothing to disclose
Cherry Kim: Nothing to disclose
Hwan Seok Yong: Nothing to disclose
Won-Seok Yoo: Nothing to disclose

**09:30-11:00 Research Stage 4**

### Research Presentation Session: Neuro

### RPS 1311

### Decoding the mind: sculpting neuroimaging with technology

**Moderator**
A. Krainik; Grenoble/FR
*(akrainik@gmail.com)*
**Author Disclosures:**
Alexandre Krainik: Advisory Board: Geodaisics

### Clinical Evaluation of 3D Motion-Correction via Scout Accelerated Motion Estimation and Reduction (SAMER) framework versus Conventional T1-Weighted MRI at 1.5 T in Brain Imaging

*L. Leukert*, A. Kronfeld, R. Paul, M. A. Brockmann, S. Altmann, A. Othman; Mainz/DE
*(laura@s-leukert.com)*

**Purpose or Learning Objective:** To evaluate the presence of motion artifacts in 1.5 T T1-weighted MRI scans using 3D motion correction via the Scout Accelerated Motion Estimation and Reduction (SAMER) framework versus conventional image reconstruction.
**Methods or Background:** MRI long scan times often cause motion artifacts, reducing image quality. SAMER uses an ultrafast pre-scan and repeated acquisition of a minimal number of additional k-space encoding lines, enabling feasible computation times. A preliminary study (14 volunteers) assessed SAMER's effect on induced motion at 3T. The main study (82 patients) compared conventional resonstruction (Non-Moco) and SAMER (SAMER Moco) motion correction using 3D T1-weighted imaging at 1.5T. Radiologists evaluated images with a 5-point Likert scale.
**Results or Findings:** In the preliminary study, SAMER Moco showed significant improvements over Non-Moco across all imaging parameters ($p < 0.001$), with 52.4% and 66.7% of cases rated as excellent or good for artifact freedom and image quality, compared to 21.4% for Non-Moco. The main study underlined these findings. SAMER Moco demonstrated superior image quality and outperformed Non-Moco, particularly in diagnostic confidence and overall image quality ($p < 0.0001$). Diagnostic confidence was rated excellent or good in 93.8% of SAMER Moco cases versus 72.0% for Non-Moco. Similarly, 84.6% of SAMER Moco cases had excellent or good image quality (56.8% for Non-Moco). Odds ratios favoured SAMER Moco (5.444 and 5.807, respectively, $p < 0.0001$). Multi-reader agreement was excellent across all parameters.
**Conclusion:** The use of SAMER in T1-weighted imaging is feasible in clinical practice and significantly enhances the reliability of 1.5 T brain MRI by successfully mitigating motion artifacts.
**Limitations:** This study's limitations include its single-centre design, reliance on a single 3D MR sequence, and inclusion of both contrast-enhanced and non-contrast scans. SAMER may also be affected by patient-induced k-space gaps.
**Funding for this study:** This research received no funding from any public, commercial, or not-for-profit sources. The authors declare that they have no competing interests that are relevant for the content of this article.
**Ethics committee - additional information:** This single-center prospective study was approved by our institution's local ethics committee, and written informed consent was obtained (approval number 2021-15811). Our study was conducted in accordance with the Declaration of Helsinki and its amendments.
**Author Disclosures:**
Roman Paul: Nothing to disclose
Laura Leukert: Nothing to disclose
Andrea Kronfeld: Nothing to disclose
Marc A Brockmann: Nothing to disclose
Ahmed Othman: Nothing to disclose
Sebastian Altmann: Nothing to disclose

### Comparison of Photon-Counting CT and Conventional CT for Determining Rotational Orientation of Directional DBS Electrodes: A Phantom Study

*D. Fedders*[1], A. Hellerbach[2], M. Eichner[2], C. Panknin[3], S. Faby[3], J. Wirths[2], V. Visser-Vandewalle[2], H. Treuer[2], S. Hunsche[2]; [1]Chemnitz/DE, [2]Cologne/DE, [3]Forchheim/DE
*(dieter.fedders@gmail.com)*

**Purpose or Learning Objective:** Accurate determination of the rotational orientation of directional deep brain stimulation (DBS) electrodes is crucial for optimizing therapeutic outcomes in functional neurosurgery. Conventional CT methods, relying on artifact analysis, face precision limitations, especially at certain angles. Photon-counting detector CT (PCD-CT), with its superior resolution, offers a potential alternative. This study compares the efficacy of PCD-CT against conventional CT-based artifact analysis in determining DBS electrode orientation.
**Methods or Background:** A phantom study was conducted using directional leads from Boston Scientific, Medtronic, and Abbott embedded in cylindrical phantoms. The phantoms were scanned with PCD-CT for direct orientation detection and conventional CT for stripe artifact analysis. Scans covered varying polar angles to assess accuracy and consistency. Key metrics included orientation accuracy and dependency on lead position relative to the CT gantry.
**Results or Findings:** PCD-CT demonstrated high accuracy across all tested angles, independent of lead alignment. In contrast, conventional CT showed reduced precision, particularly at extreme angles where artifact detection was unreliable. PCD-CT enabled consistent, precise assessments of segmented contacts, enhancing postoperative DBS programming.
**Conclusion:** PCD-CT offers a robust solution for determining the rotational orientation of DBS electrodes, overcoming limitations of conventional artifact-based methods. This supports more accurate electrode positioning and programming, potentially improving functional neurosurgery outcomes.
**Limitations:** The phantom-based design may not replicate clinical complexity, limiting generalizability. The study only evaluated specific directional DBS leads, so results may not apply to other types. Additionally, PCD-CT's limited availability could hinder immediate clinical application.
**Funding for this study:** None beside scanning time at the research facility from Siemens
**Ethics committee - additional information:** Phantom study

**Author Disclosures:**
Harald Treuer: Nothing to disclose
Jochen Wirths: Nothing to disclose
Veerle Visser-Vandewalle: Nothing to disclose
Dieter Fedders: Nothing to disclose
Christoph Panknin: Other: Computed Tomography, Siemens Healthineers AG, Forchheim/DE
Sebastian Faby: Other: Computed Tomography, Siemens Healthineers AGC
Alexandra Hellerbach: Nothing to disclose
Markus Eichner: Nothing to disclose
Stefan Hunsche: Nothing to disclose

**A Multimodal MRI-Based Machine Learning Framework for Classifying Cognitive Impairment in Cerebral Small Vessel Disease**
*G. Lin*, W. Chen, M. Chen, J. Ji; Lishui/CN

**Purpose or Learning Objective:** This study aims to propose a multimodal magnetic resonance imaging (MRI)-based machine learning framework to effectively classify mild cognitive impairment (MCI) and no cognitive impairment (NCI) in patients with cerebral small vessel disease (CSVD).
**Methods or Background:** We enrolled 223 patients with CSVD, categorized into NCI (n = 121) and MCI (n = 102) groups based on neurocognitive assessments. Multimodal MRI data, including T1-weighted, resting-state functional MRI, and diffusion tensor images, were collected. Image preprocessing, feature extraction, and feature selection methods were applied to obtain MRI features from the three modalities. The AutoGluon platform was utilized for model development, and traditional machine learning algorithms were applied for comparison. The models were validated using a validation cohort of 97 patients with CSVD, and their performance was assessed via receiver operating characteristic curve (ROC) analysis.
**Results or Findings:** The AutoGluon model to distinguish MCI from NCI based on multimodal MRI features demonstrated a high area under the ROC curve (AUC), accuracy, sensitivity, specificity, and F1-score in the testing set (0.894, 85.65%, 84.31%, 86.78%, and 84.31%, respectively) and validation cohort (0.846, 79.38%, 81.82%, 77.36%, and 78.26%, respectively). Other models built using traditional machine learning algorithms had AUCs of 0.661–0.732, and their prediction accuracies were significantly lower than that of the AutoGluon model (P < 0.001).
**Conclusion:** Our study provides a multimodal MRI-based machine learning framework, utilizing the AutoGluon platform, that outperforms traditional algorithms in classifying MCI and NCI in patients with CSVD, offering a promising tool for the early prediction of MCI in CSVD.
**Limitations:** As a retrospective study, it is susceptible to selection bias, which may limit its generalizability.
**Funding for this study:** This study is supported by Zhejiang Public Welfare Research Program (LGF20H220002, LGF19H180010), and Zhejiang Provincial Healthcare Program (2024KY562)
**Ethics committee - additional information:** This study was approved by the Ethics Committee of the Fifth Affiliated Hospital of Wenzhou Medical University (approval number: 2024-266)
**Author Disclosures:**
Minjiang Chen: Nothing to disclose
Jiansong Ji: Nothing to disclose
Weiyue Chen: Nothing to disclose
Guihan Lin: Nothing to disclose

**Detection of intracranial hemorrhage using ultralow-dose brain computed tomography with deep learning reconstruction versus conventional-dose computed tomography**
*C. Otgonbaatar*[1], H. Kim[2], P-H. Jeon[2], S. H. Jeon[2], S. Cha[2], J-K. Ryu[1], H. Shim[1], S. M. Ko[2], J. Kim[2]; [1]Seoul/KR, [2]Wonju-si/KR
*(chukarad@gmail.com)*

**Purpose or Learning Objective:** This study aimed to evaluate the diagnostic performance, image quality, and radiation dose among ultralow-dose protocol with deep learning reconstruction (DLR), ultralow-dose computed tomography (CT) with iterative reconstruction (IR), and conventional-dose protocols for detecting intracranial hemorrhage.
**Methods or Background:** This retrospective study enrolled 93 patients. All patients underwent follow-up noncontrast CT with ultralow-dose setting after initial conventional-dose CT within 5 days. A conventional-dose CT was obtained using 123–188 mA and IR. Ultralow-dose CT was obtained using 50 mA with IR and DLR. Qualitative assessments and quantitative assessments (image noise, differentiation between gray and white matter, and artifact) were conducted. The diagnostic performance for detecting intracranial hemorrhage using ultralow-dose CT with IR and ultralow-dose CT with DLR was assessed.
**Results or Findings:** An approximately 84.0% reduction in median volume CT dose index was found in the ultralow-dose CT protocol (5.6 mGy) compared with conventional-dose CT (35.02 mGy; IQR: 33.09–37.36). Ultralow-dose CT with DLR significantly (p < 0.001) improved image noise, SNR, and CNR compared with ultralow-dose CT with IR and conventional-dose CT. Ultralow-dose CT with DLR resulted in higher sensitivity (99.3% vs. 98.6%) and specificity (97.5% vs. 97.5%) for detecting intracranial hemorrhage than ultralow-dose CT with IR.
**Conclusion:** Ultralow-dose CT with DLR is an acceptable technique that provides higher image quality and diagnostic performance with a reduction in radiation dose of approximately 87.7% compared with conventional-dose CT.
**Limitations:** We did not investigate the effect of a tube current of <50 mA on the diagnostic performance of intracranial hemorrhage and image quality. Further validation is required to investigate a lower effective dose of <0.21 mSv. Additionally, all results were limited to one scanner, and acquisition parameters may require adjustment for different CT vendors.
**Funding for this study:** None
**Ethics committee - additional information:** No
**Author Disclosures:**
Sung Min Ko: Nothing to disclose
Pil-Hyun Jeon: Nothing to disclose
Jinwoo Kim: Nothing to disclose
Sungjin Cha: Nothing to disclose
Jae-Kyun Ryu: Nothing to disclose
Hyunjung Kim: Nothing to disclose
Sang Hyeon Jeon: Nothing to disclose
Hackjoon Shim: Nothing to disclose
Chuluunbaatar Otgonbaatar: Nothing to disclose

**Correlation of diffusion tensor imaging findings in cerebral sensorimotor regions with neurophysiological deficits in patients after spinal cord injury**
A. Zimny[1], *W. N. Machaj*[1], P. Podgórski[1], W. Fortuna[1], J. Huber[2], B. Bobek-Billewicz[3], P. Tabakow[1]; [1]Wrocław/PL, [2]Poznań/PL, [3]Gliwice/PL

**Purpose or Learning Objective:** The aim of the study is to examine the correlation between DTI findings, clinical motor and sensory deficits, and motor evoked potential (MEP) parameters. This will provide insights into the potential of DTI metrics as biomarkers for predicting functional recovery and guide therapeutic interventions in patients with chronic spinal cord injury (SCI).
**Methods or Background:** A total of 29 patients with SCI (both paraplegic p-SCI and tetraplegic t-SCI), matched by sex and age to 29 healthy controls, were neurologically and neurophysiologically evaluated, including MEPs recorded from upper and lower limb muscles. Diffusion tensor imaging (DTI) was performed using a 3 Tesla MRI scanner and processed using Human Motor Area (HMAT) and Sensorimotor Area Tract (SMATT) templates.
**Results or Findings:** No significant DTI differences were found between p-SCI and t-SCI or p-SCI and healthy controls. However, patients with t-SCI had lower fractional anisotropy (FA) in primary motor (M1) and sensorimotor (S1) tracts, pre-supplementary motor area (pre-SMA) tracts, M1 and S1 cortices, and left pre-SMA cortex compared to controls. In t-SCI patients, higher motor scores correlated with increased FA in ventral premotor area (PMv) tracts and cortices, and higher sensory scores with higher FA in S1 tracts. MEP amplitudes from rectus femoris also positively correlated with FA in motor tracts, M1, PMd, PMv, and SMA cortices.
**Conclusion:** DTI findings reveal distant degeneration in the sensorimotor cortex and supraspinal tracts in chronic SCI, which correlates with clinical motor and sensory scores, as well as MEP parameters from rectus femoris muscles in t-SCI patients. DTI metrics can serve as potential biomarkers to predict motor and sensory recovery in patients with SCI and to guide and track therapeutic interventions.
**Limitations:** Cross-sectional design and the small sample size.
**Funding for this study:** Grant NCBiR ERA-NET-NEURON/13/2018, Wroclaw Medical University grant SB.
**Ethics committee - additional information:** The study was performed in accordance with the Declaration of Helsinki and was approved by the Bioethics Committee of the Wroclaw Medical University.
**Author Disclosures:**
Juliusz Huber: Author: Nothing
Anna Zimny: Author: Nothing
Paweł Tabakow: Author: Nothing
Weronika Natalia Machaj: Author: Nothing
Przemysław Podgórski: Author: Nothing
Barbara Bobek-Billewicz: Author: Nothing
Wojciech Fortuna: Author: nothing

**MR Neurography at 3T and 7T for the assessment of proximal nerve damage in polyneuropathies**

*J. M. E. Jende*[1], C. Mooshage[1], K. Zhang[1], T. Platt[1], C. Neelsen[1], M. Bendszus[1], H-P. Schlemmer[1], M. Ladd[1], F. Kurz[2]; [1]Heidelberg/DE, [2]Geneva/CH

**Purpose or Learning Objective:** Disorders of the peripheral nervous system such as polyneuropathies pose a huge challenge to the global healthcare system. The exact pathophysiology underlying most polyneuropathies remains poorly understood. Previous studies on 3T MR neurography (MRN) have found that the maximum of fascicular nerve damage in various polyneuropathies is located at the level of the sciatic nerve although clinical symptoms usually occur further distally.
**Methods or Background:** To understand the clinical impact and physiological background of fascicular sciatic nerve lesions, 10 patients with distal symmetric polyneuropathy and 10 healthy controls matched for age and BMI underwent T2-weighted, high resolution MRN of the right thigh at 3T and 7T. At 7T, the maximum fascicular diameter and the average number of nerve fascicles per slice were measured. At 3T, additional diffusion-weighted and T2-relaxometry sequences were acquired and the sciatic nerve's fractional anisotropy (FA) and T2 relaxation times (T2R) were calculated.
**Results or Findings:** At 7T, the sciatic nerve's maximum fascicular diameter in patients with polyneuropathies was larger compared to controls (1.41mm±0.16 vs. 1.03mm±0.07; p=0.049mm). In patients with polyneuropathies, the fascicular diameter was negatively correlated with the number of nerve fascicles (r=-0.72;p=0.018) and the FA (r=-0.78;p=0.017mm). Positive correlations were found between the fascicular diameter and T2R (r=0.76;p=0.019).
**Conclusion:** The results indicate that polyneuropathies cause a fusion of nerve fascicles that results in a reduced number of nerve fascicles and a larger fascicular diameter that is associated with a structural and functional decline represented by a decrease in nerve FA and an increase in T2R. This study is the first to show that the fascicular diameter of the sciatic nerve is directly related to changes in FA, T2R and clinical neuropathy status. The causes of fascicular fusion remain to be determined.
**Limitations:** Cohort Size
**Funding for this study:** Else Kröner Fresenius Foundation (EKFS)
**Ethics committee - additional information:** This study was appoved by the local ethics committee of Heidelberg University Hospital.
**Author Disclosures:**
Tanja Platt: Nothing to disclose
Ke Zhang: Nothing to disclose
Christian Neelsen: Nothing to disclose
Johann Malte Enno Jende: Nothing to disclose
Mark Ladd: Nothing to disclose
Felix Kurz: Nothing to disclose
Martin Bendszus: Nothing to disclose
Christoph Mooshage: Nothing to disclose
Heinz-Peter Schlemmer: Nothing to disclose

**Deep-learning-reconstructed 3D MR neurography of extraforaminal cranial and spinal nerves**

F. Ensle[1], *F. Zecca*[2], B. J. Kerber[1], M. Lohezic[1], J. Kroschke[1], K. Pawlus[1], R. Guggenberger[3]; [1]Zurich/CH, [2]Cagliari/IT, [3]Winterthur/CH
*(fabio.zecca92@gmail.com)*

**Purpose or Learning Objective:** To assess and compare DESS and post-contrast STIR sequences in deep-learning(DL)-reconstructed 3D MR neurography of the extraforaminal cranial and spinal nerves.
**Methods or Background:** Eighteen consecutive exams of 18 patients with unclear cephalgia undergoing head-and-neck MRI at 1.5T were retrospectively included (mean age: 51 ± 14 years, 11 female). 3D DESS and post-contrast 3D STIR sequences were reconstructed with a prototype DL algorithm. Two blinded readers qualitatively evaluated visualization of the inferior alveolar (IAN), lingual (LN), facial (FN), hypoglossal (HN), greater occipital (GON), lesser occipital (LON) and greater auricular (GAN) nerves, as well as overall image quality, vascular suppression and artifacts. Apparent signal-to-noise ratio (aSNR) and contrast-to-noise ratios (aCNR) were measured. Qualitative ratings were compared between sequences using Wilcoxon signed-rank test, quantitative analysis with paired sample Student's t-testing.
**Results or Findings:** DESS demonstrated significantly improved visualization of the LON and GAN and proximal GON ($p < 0.015$). Post-contrast STIR showed significantly enhanced visualization of the LN, HN and distal IAN ($p < 0.001$). The FN, proximal IAN and distal GON did not demonstrate significant differences in visualization between DESS and post-contrast STIR ($p > 0.08$). With regard to overall image quality and artifacts, there was also no significant difference between sequences. Post-contrast STIR achieved superior vascular suppression, reaching statistical signifi-cance for one reader ($p = 0.039$). Quantitatively, there was no significant difference between sequences ($p > 0.05$).
**Conclusion:** Our findings suggest that 3D DESS generally provides improved visualization of spinal nerves (GON, LON, GAN), while post-contrast 3D STIR facilitates enhanced delineation of extraforaminal cranial nerves (IAN, LN, HN). 3D DESS and post-contrast 3D STIR could each add value to head-neck MRN protocols, depending on the main clinical area of interest.
**Limitations:** Retrospective study. Limited sample size.
**Funding for this study:** Not applicable.
**Ethics committee - additional information:** This study was approved by the institutional review board.
**Author Disclosures:**
Maelene Lohezic: Employee: GE HealthCare
Fabio Zecca: Nothing to disclose
Falko Ensle: Nothing to disclose
Roman Guggenberger: Nothing to disclose
Karolina Pawlus: Nothing to disclose
Bjarne Jonas Kerber: Nothing to disclose
Jonas Kroschke: Nothing to disclose

**Enhancing Imaging Efficiency in Advanced Diffusion Imaging Using Denoising and Post-Processing Techniques**

*V. Sedlák*, K. Vambersky, A. Kavková, K. Sichova, D. Netuka, T. Belsan, M. Majovsky; Prague/CZ

**Purpose or Learning Objective:** The objective of this study is to demonstrate how the application of advanced denoising techniques, such as MP-PCA and P2S, along with post-processing methods for enhancing angular resolution, can significantly reduce imaging times in advanced diffusion imaging. This approach aims to preserve or improve image quality while reducing the acquisition time burden in clinical and research settings.
**Methods or Background:** We acquired two sets of advanced diffusion MRI data from patients with glial brain tumors: one using a full-length acquisition and the other using a fast acquisition protocol. The full-length dataset was processed directly, while the fast protocol data was enhanced with denoising algorithms (e.g. MP-PCA, P2S) and angular super-resolution reconstruction techniques. We evaluated data quality by assessing signal-to-noise ratio (SNR), angular resolution, and diagnostic accuracy for predicting glioma grade and IDH mutation status.
**Results or Findings:** Data from 100 patients with glial brain tumors were processed and analyzed. The shortened protocol data, when enhanced by MP-PCA and P2S denoising, provided a significant improvement in SNR, aligning closely with or even superseding the quality of the full-length acquisition. Angular super-resolution reconstruction techniques further enhanced the angular resolution without extending scan time. The diagnostic accuracy for predicting glioma grade and IDH mutation status was comparable between the full-length and processed shortened datasets, demonstrating that these post-processing methods can preserve clinical diagnostic value while reducing scan duration
**Conclusion:** Denoising techniques and angular resolution enhancement significantly improve the quality of shortened diffusion MRI acquisitions, maintaining diagnostic accuracy for glioma grading and IDH status while reducing scan time.
**Limitations:** Results are based on 100 glioma patients and require validation in broader populations. The computational demands may limit immediate clinical application, and performance may vary with MRI hardware and sequence parameters.
**Funding for this study:** This study was supported by the Grant Agency of Charles University, grant number GAUK 222623
**Ethics committee - additional information:** Approved by the Ethics committee of the Military. University Hospital Prague
**Author Disclosures:**
Martin Majovsky: Nothing to disclose
Vojtěch Sedlák: Nothing to disclose
Anna Kavková: Nothing to disclose
David Netuka: Nothing to disclose
Kamil Vambersky: Nothing to disclose
Kristyna Sichova: Nothing to disclose
Tomas Belsan: Nothing to disclose

**DTI-ALPS Mapping: A Novel Method that Can Comprehensively Reflect the DTI-ALPS pattern**

X. Fan, *G. Cheng*, X. Zhang, N. Zhang; Shenzhen/CN

**Purpose or Learning Objective:** Our objectives are to depict the whole white matter DTI-ALPS changes pattern in cognitive impairment and provide an intuitive and comprehensive method to assess the activity of the glymphatic system.
**Methods or Background:** The glymphatic system is increasingly recognized as a critical factor in the pathogenesis of dementia. We creatively propose a novel method to analyze the whole white matter diffusion tensor image analysis along the perivascular space (DTI-ALPS). We included 304

participants from the Shenzhen Multimodal Aging Research (STAR) Cohort recruited in Peking University Shenzhen Hospital, including 182 cognitively unimpaired (CU) participants, 93 participants with mild cognitive impairment, and 29 patients with dementia. All participants underwent 3.0T MRI scans with DTI sequences. We calculated the DTI-ALPS values using the conventional method and depicted the whole white matter DTI-ALPS mapping with the novel method we developed. We performed the analysis of variance (ANOVA) to find the differences in ALPS values among three groups and used post-hoc tests to find intergroup differences in the conventional method and DTI-ALPS mapping, respectively.
**Results or Findings:** We revealed the whole brain ALPS pattern using the DTI-ALPS mapping. Over 13 out of 30 regions showed significant differences ($p < 0.05$) in the inter-group analysis, which provided additional information beyond the significant differences based on the conventional ROI-based ALPS.
**Conclusion:** In conclusion, the DTI-ALPS mapping provides a robust, intuitive, and comprehensive way to evaluate the changing pattern of the glymphatic system, overcoming the limitations introduced by conventional ROI-based DTI-ALPS calculating methods.
**Limitations:** Future studies should integrate additional statistics such as minimum, maximum, and standard deviation into ALPS-mapping analyses for a comprehensive understanding of the DTI-ALPS mapping.
**Funding for this study:** This study is principally supported by the Shenzhen Science and Technology Program (KCXFZ 20211020163408012).
**Ethics committee - additional information:** the Ethics Committee of Peking University Hospital
**Author Disclosures:**
Xiang Fan: Nothing to disclose
Xiqian Zhang: Nothing to disclose
Guanxun Cheng: Nothing to disclose
Na Zhang: Nothing to disclose

**HDD-Net: Hippocampus Dual Decoder Network for automated segmentation of hippocampus from computed tomographic scans**
*S. J. Ahn*, W. J. Son, J. Y. Lee, H. Lee; Seoul/KR
*(aahng77@yuhs.ac)*

**Purpose or Learning Objective:** Changes of brain hippocampal volumes are closely associated with the development of Alzheimer's disease. In this work, we develop a new deep learning (DL) network model for volumetric hippocampal segmentation from computed tomography (CT) head images, a task that has been challenged due to the modality's limited brain contrast.
**Methods or Background:** HDD-Net: The proposed network model is characterized by four major elements – 1) an encoder , 2) two parallel decoders (namely, seg-decoder and edge-decoder), 3) a feature cross module (FCM) fusing features from the two decoders and 4) a cross loss computing differences between outputs. Datasets and preprocessing: 150 pairs of MRI-CT volumetric head images collected at Gangnam Severance Hospital were used for model training (N=120) and internal validation (N=30), while 47 pairs were selected from Seoul St. Mary's Hospital database for external validation. Ground-truth hippocampal labels were generated from T1-weighted MR images using FreeSurfer, and then were processed by a Gaussian high-pass filter leading to reference edge maps. Each pair of MR-CT images were coregistered using SPM12. Training and Evaluation: The DL model was trained with a cost function combining segmentation, edge, and cross losses. Its performance was evaluated by calculating Dice coefficient and intersection-over-union (IOU). The performance our model was compared with that of conventional U-Net model.
**Results or Findings:** Dice and IOU of our model is higher than those of U-net for internal validation set (DICE : 0.840 vs. 0.822, IOU: 0.726 vs. 0.699) and external validation (DICE : 0.784 vs. 0.751, IOU: 0.650 vs. 0.613)
**Conclusion:** Results suggest feasibility of DL-based automated hippocampal segmentation from CT scans and its improved performance via edge decoding.
**Limitations:** The applicability of the model could be enhanced by training it on patients with dementia
**Funding for this study:** None
**Ethics committee - additional information:** Gangnam severance hospital IRB
**Author Disclosures:**
Sung Jun Ahn: Nothing to disclose
Hyunyeol Lee: Nothing to disclose
Ji Young Lee: Nothing to disclose
Won Jun Son: Nothing to disclose

**Initial experience with 60kVp craniocervical CT angiography: achieving 0.2 millisievert while maintaining diagnostic performance via artificial intelligence iterative reconstruction**
Y. Han[1], L. Peng[2], T. Meng[2], Q. Sun[1], G. Zhang[2], *T. Wang*[2], X. Wang[1]; [1]Jinan/CN, [2]Shanghai/CN
*(tiantian.wang@cri-united-imaging.com)*

**Purpose or Learning Objective:** To describe the initial experience and evaluate the clinical feasibility of 60kVp craniocervical CT angiography (CTA) with artificial intelligence iterative reconstruction (AIIR).
**Methods or Background:** Sixty consecutive patients scheduled for craniocervical CTA were prospectively enrolled and underwent a 60kVp (350 ref. mAs) and a followed 120kVp (120 ref. mAs) craniocervical CTA, in a 5-minute interval, using two separate contrast medium (CM) injections. The 120kVp scans were reconstructed with hybrid iterative reconstruction (HIR), while the 60kVp scans were reconstructed with HIR and AIIR. Two radiologists diagnosed the stenosis, the intracranial aneurysm (IA), and the vascular anatomic variant (VAV) in consensus using a 5-point confidence scale (1=definitely absent, 5=definitely present) on a per-patient basis, which was used for a receiver operating characteristic analysis, and evaluated the vessel visibility (1=blur, 5=clear). Image noise on the common carotid artery (CCA) was measured. The diagnostic performance and image quality of 60kVp scans were evaluated, using 120kVp scans as the reference standard.
**Results or Findings:** The mean effective dose and CM dosage was 0.18 ± 0.04 mSv and 30.58 ± 5.15 ml, respectively, for 60kVp acquisition, corresponding to an 83.02% and 38.46% reduction as compared to 120kVp acquisition. Under 60kVp acquisition, AIIR outperformed HIR in diagnosing all three abnormal manifestations, showing higher AUC (stenosis: 0.98 vs 0.57; IA: 0.92 vs 0.62; VAV: 0.92 vs 0.79; all $p<0.05$). No significant difference in vessel visibility was found between 60kVp AIIR and reference images (4.32±0.97 vs 4.42±0.72, $p=0.412$), while AIIR images showed lower image noise (10.14±4.64 HU vs 14.68±5.23 HU; $p<0.05$).
**Conclusion:** The 60kVp craniocervical CTA with AIIR has the potential for profound dose reduction without compromising the image quality and the diagnostic performance.
**Limitations:** N/A
**Funding for this study:** N/A
**Ethics committee - additional information:** This study was approved by the local IRB
**Author Disclosures:**
Qizhong Sun: Nothing to disclose
Tiantian Wang: Employee: at United Imaging Healthcare
Yicheng Han: Nothing to disclose
Guozhi Zhang: Employee: at United Imaging Healthcare
Liying Peng: Nothing to disclose
Ximing Wang: Nothing to disclose
Ting Meng: Nothing to disclose

**12:30-13:30 Research Stage 1**

### Research Presentation Session: Imaging Informatics and Artificial Intelligence

### RPS 1405

### Artificial intelligence in musculoskeletal imaging

**Moderator**
S. Gitto; Milan/IT
**Author Disclosures:**
Salvatore Gitto: Other: Abiogen Pharma, Biolive Alpinion, Bracco Imaging

**Development and validation of deep learning model for screening low bone mineral density using chest radiographs: A multicentre, multinational study**
*J. Song*, M. Kim, G. Lee, J. Jeong, S. J. Bae, J-M. Koh, N. Kim; Seoul/KR
*(jmsong@promedius.ai)*

**Purpose or Learning Objective:** This study aimed to develop and validate a deep learning model for screening of patients with low bone mineral density (BMD) using chest radiographs (CXRs).
**Methods or Background:** We retrospectively collected CXR data paired with DXA results from patients aged 50 and above from five different resources. Each patient's BMD was classified using a T-score threshold of -1.0, with scores of -1.0 or above defined as 'normal' and those below as 'low BMD'. Of the 57,589 CXRs from Hospital A, 55,600 were utilized for training, and 1,989 were used for internal validation. For external validation, 3338, 938, and 295 CXRs were collected from B, C hospitals and D platform, respectively, representing diverse patient demographics and clinical backgrounds. A deep learning model was developed to perform binary classification of patients' BMD as either normal or low, based on their CXRs.
**Results or Findings:** In the A dataset, the model yielded an AUC of 0.95 and demonstrated sensitivity of 0.97, specificity of 0.65, and F1 score of 0.90. In the datasets B, C, and D, the model achieved AUCs of 0.91, 0.89, and 0.82. The model's sensitivity was 0.87, 0.88, and 0.64; specificity was 0.77, 0.71, and 0.85; and F1 score was 0.80, 0.88, and 0.76, respectively.
**Conclusion:** The proposed low BMD screening system demonstrated performance exceeding an AUC of 0.8 in all external datasets, highlighting the robustness of the system. Notably, the system showed promising performance even on the D dataset, which comprised individuals of completely different racial backgrounds. This suggests the potential to promptly identify patients with low BMD from CXRs, the most widely used imaging modality globally.
**Limitations:** First, this is a retrospective study. Second, a substantial proportion of the dataset comprises a single national population.
**Funding for this study:** No funding was provided for this study.
**Ethics committee - additional information:** Ethics approval was obtained from the Ethics Committee of Asan Medical Center (No. 2019-1226), the Public institutional Bioethics Committee (No.2024-0256-001) and the Ethics Committee of Korea VHS Medical Center (No.2022-10-003-001).
**Author Disclosures:**
Jeongmin Song: Employee: Promedius Inc.
Namkug Kim: Shareholder: Promedius
Minjee Kim: Employee: Promedius Inc.
Jung-Min Koh: Advisory Board: Promedius
Sung Jin Bae: Nothing to disclose
Gaeun Lee: Employee: Promedius Inc.
Jinhoon Jeong: Employee: Promedius Inc.

### Evaluating the Impact of Artificial Intelligence on Fracture Detection: A Multinational Randomized Crossover Study on diagnostic thinking efficacy

*B. J. Van Der Zwart*[1], H. C. Ruitenbeek[1], M. Boesen[2], M. W. Brejnebol[2], G. Gunes[3], K-G. A. Hermann[4], K. Ziegeler[4], E. Oei[1], J. J. Visser[1]; [1]Rotterdam/NL, [2]Copenhagen/DK, [3]Ortaca/TR, [4]Berlin/DE
*(basvanderzwart1@hotmail.com)*

**Purpose or Learning Objective:** To assess the impact of AI assistance on fracture detection on conventional radiographs by conducting a multi-country, multicenter randomised crossover study.
**Methods or Background:** Radiography data from 1,500 consecutive adult cases with suspected posttraumatic fractures were gathered along with relevant clinical information, with 500 cases from each of three European sites. All cases were read by senior and junior radiologists and orthopedic surgeons both without and with AI assistance in two sessions separated by at least four weeks. A reference standard was established by expert radiologists with the help of clinical data and follow-up imaging. The mean change in diagnostic accuracy was measured both per case (sensitivity and specificity) and per fracture (sensitivity).
**Results or Findings:** Sensitivity at the case-level increased with the AI assistance for all reader groups with +0.074 for senior radiologists, +0.181 for junior radiologists, +0.095 for both senior and junior orthopedic surgeons. The specificity was negatively impacted with the AI assistance for the radiologists with changes of -0.010 for senior and -0.027 for junior radiologists, respectively. The specificity increased for orthopaedic surgeons with +0.016 and +0.024 for senior and junior surgeons, respectively. The changes in sensitivity per fracture with the AI assistance were +0.089 and +0.168 for senior and junior radiologists and +0.082 and 0.111 for senior and junior surgeons, respectively.
**Conclusion:** Our study demonstrates that AI assistance enhances fracture detection on conventional radiographs, yielding improved patient-wise sensitivity across participating centers. The changes in specificity was positive for orthopedic surgeons, but negative for radiologists.
**Limitations:** The study was conducted in a simulated setting, potentially impacting reader performance. Additionally, the Hawthorne effect may have influenced reader behaviour, as participants were aware they were being observed.
**Funding for this study:** This project has received funding from the European Union's Horizon 2020 Research and Innovation Programme under grant agreement no. 954221. The results presented in this work reflect only the views of the authors. The Commission is not responsible for any use that may be made of the information it contains.
**Ethics committee - additional information:** This study was approved by the institutional review board of the Erasmus MC, Rotterdam (Study ID: MEC-2021-0430) and the need to obtain informed consent was waived by the institutional review boards of Charité Universitätsmedizin–Berlin (no. EA4/079/22) and the Danish Patient Safety Authority.
**Author Disclosures:**
Huibert C. Ruitenbeek: Nothing to disclose
Edwin Oei: Nothing to disclose
Mikael Boesen: Advisory Board: Radiobotics
Katharina Ziegeler: Nothing to disclose
Gözde Gunes: Employee: Radiobotics
Bastiaan Johannes Van Der Zwart: Nothing to disclose
Mathias Willadsen Brejnebol: Nothing to disclose
Kay-Geert A. Hermann: Nothing to disclose
Jacob Johannes Visser: Nothing to disclose

### Post implementation validation - False Positives and Negatives in AI Fracture Detection in Clinical Workflow: A Deep Dive

*R. Sivanandan*[1], J. Vardal[2]; [1]Sandvika/NO, [2]Drammen/NO
*(drprabu@gmail.com)*

**Purpose or Learning Objective:** This study evaluates post-implementation monitoring of an AI fracture detection algorithm, focusing on false positives and negatives, their clinical implications, and mitigation strategies. It also assesses the algorithm's impact on workflow and patient outcomes and further possibility of research studies.
**Methods or Background:** The algorithm was implemented sequentially across 5 hospitals in Vestre Viken Health Trust, Norway, with a follow-up validation conducted at the primary hospital using 1284 cases. AI results were negative (60.8%), positive (37.9%) and doubtful (1.3%). Our new workflow after AI implementation allowed AI-negative patients to be discharged, while AI-positive cases were referred to clinicians. Radiographers initially reviewed AI results and guided patients flow, while radiologists reporting all cases. Orthopedic residents and consultants subsequently examined these images with AI results.
**Results or Findings:** Validation revealed 86% true negatives, 2% false negatives, 7% true positives, 4% false positives, and 4% doubtful cases. These results were consistent with preliminary external validation. Patients with AI-positive had false positives (5.6%) were primarily attributed to old fractures, skin folds and heterotopic calcifications, with minimal clinical significance. Among the patient sent home with AI-negative result, false negatives (2.3%) mainly included minimal knee effusion in adults, benign bone lesions, and small avulsion fractures requiring conservative treatment. Only one patient with an avulsion fracture and drop finger was erroneously discharged and recalled for clinical re-evaluation.
**Conclusion:** The validation results aligned with pre-implementation findings, with false positives and negatives having minimal clinical impact. This study highlights the potential for further research that is planned to evaluate the necessity of radiologist reporting for AI-positive cases, given that clinicians already review these images.
**Limitations:** Few patients' clinical results were not analyzed due to restricted access to patient journal. Few patients status after imaging were missing.
**Funding for this study:** No funding
**Ethics committee - additional information:** Information was collected as per the Ethics
**Author Disclosures:**
Jonas Vardal: Nothing to disclose
Ramprabananth Sivanandan: Nothing to disclose

### Assessing the Generalisability of a Paediatric Wrist Fracture Detection AI Model Using a Novel Dataset

*C. Pauling*, O. Arthurs, B. Kanber, S. C. Shelmerdine, E. Allan, E. Ashworth; London/UK
*(cato.pauling.22@ucl.ac.uk)*

**Purpose or Learning Objective:** The purpose of this study was to assess the generalisability of an artificial intelligence (AI) model, trained on open-source data, for the detection of fractures and other abnormalities in paediatric wrist radiographs using a novel, external, and multi-centric dataset.
**Methods or Background:** A novel retrospective case dataset was curated from two paediatric trauma centres in London, England. The dataset comprises 865 images with a mean patient age of 10.4 ± 3.5 [standard deviation] years. Ground truth annotations for the external test dataset were established by consensus opinion of at least two paediatric radiologists. To imitate real-world prospective data, no pre-processing was applied to the external data and only invalid scans were excluded. A YOLOv7-X model was trained on GRAZPEDWRI-DX, an open-source paediatric wrist trauma radiograph dataset. After achieving an optimal performance on the test split of data, the model was used to perform inference on the novel external data and the performance metrics were compared.
**Results or Findings:** The sensitivity of the model for the detection of fractures was 89.0% on the test split of the open-source data. When evaluating on the novel external data, the sensitivity decreased by 32.6%. The reduction in the performance of the model across all detection classes was less severe, with a

Friday change to mean Average Position (mAP) of -0.139mAP@0.5 (-0.067 mAP@[0.5:0.95]).
**Conclusion:** The model failed to adequately generalise to an external dataset evidenced by a notable decline in fracture detection sensitivity. It is of critical importance to ensure that AI models intended for use in a prospective clinical setting are externally validated. Additionally, data quality and pre-processing procedures can significantly impact model performance.
**Limitations:** The open-source training dataset contains annotations for additional pathologies which are not included in the external test dataset.
**Funding for this study:** CP is funded by the Great Ormond Street Hospital Children's Charity (GOSHCC) (Award Number: VS0618). OJA is funded by an NIHR Career Development Fellowship (NIHR-CDF-2017-10-037). SCS is funded by an NIHR Advanced Fellowship Award (NIHR-301322).
**Ethics committee - additional information:** Ethical approval was provided by the National Health Service (NHS) Health Research Authority (HRA) (IRAS ID: 274278, REC reference 22/PR/0334)
**Author Disclosures:**
Emily Ashworth: Nothing to disclose
Cato Pauling: Nothing to disclose
Susan Cheng Shelmerdine: Nothing to disclose
Owen Arthurs: Nothing to disclose
Baris Kanber: Nothing to disclose
Emma Allan: Nothing to disclose

### AI in radiological imaging of soft-tissue and bone tumours: a systematic review evaluating against CLAIM and FUTURE-AI guidelines

D. J. Spaanderman[1], M. Marzetti[2], X. Wan[1], A. Scarsbrook[2], E. Oei[1], D. Grünhagen[1], *S. Klein*[1], M. P. A. Starmans[1]; [1]Rotterdam/NL, [2]Leeds/UK
*(s.klein@erasmusmc.nl)*

**Purpose or Learning Objective:** Soft-tissue and bone tumours (STBT) are rare, diagnostically challenging lesions with variable clinical behaviours and treatment approaches. This systematic review aims to provide an overview of Artificial Intelligence (AI) methods using radiological imaging for diagnosis and prognosis of STBT, highlighting challenges in clinical translation, and evaluating study alignment with the Checklist for AI in Medical Imaging (CLAIM) and the FUTURE-AI international consensus guidelines for trustworthy and deployable AI to promote clinical translation of AI methods.
**Methods or Background:** The systematic review identified literature from several bibliographic databases, covering papers published before 17/07/2024. Original research published in peer-reviewed journals, focused on radiology-based AI for diagnosis or prognosis of primary STBT was included. Exclusion criteria were animal, cadaveric, or laboratory studies, and non-English papers. Abstracts were screened by two of three independent reviewers to determine eligibility. Included papers were assessed against the two guidelines by one of three independent reviewers. (PROSPERO Registration: CRD42023467970)
**Results or Findings:** The search identified 15,015 abstracts, and 325 articles were included for evaluation. Studies performed moderately on CLAIM, averaging a score of 28·9±7·5 out of 53, but poorly on FUTURE-AI, averaging 5·1±2·1 out of 30.
**Conclusion:** Imaging-AI tools for STBT remain at the proof-of-concept stage, indicating significant room for improvement. Future efforts by AI developers should focus on design (define unmet clinical need, intended clinical setting and integration), development (build on previous work, training with data reflecting real-world usage, explainability), evaluation (addressing biases, evaluating using best practices), and data reproducibility and availability. Following these recommendations could improve clinical translation of AI methods.
**Limitations:** Limitations include single-reviewer scoring due to the high volumn of literature included and assessment against as-of-yet unpublished, FUTURE-AI guidelines. However, FUTURE-AI were developed by a large group of international medical AI experts.
**Funding for this study:** Hanarth Fonds, ICAI Lab, NIHR, EuCanImage
**Ethics committee - additional information:** Not applicable to the systematic review.
**Author Disclosures:**
Stefan Klein: Grant Recipient: Payment to institution. Stefan Klein is scientific director of the ICAI lab "Trustworthy AI for MRI", a public-private research program partially funded by General Electric Healthcare.
Edwin Oei: Grant Recipient: Payment to institution. Edwin Oei co-leads a project embedded in the ICAI lab "Trustworthy AI for MRI", a public-private research program partially funded by General Electric Healthcare.
Andrew Scarsbrook: Nothing to disclose
Douwe Jan Spaanderman: Nothing to disclose
Matthew Marzetti: Nothing to disclose
Martijn Pieter Anton Starmans: Nothing to disclose
Dirk Grünhagen: Nothing to disclose
Xinyi Wan: Nothing to disclose

### Multi-Center External Validation of an Automated Method Segmenting and Differentiating Atypical Lipomatous Tumors from Lipomas Using Radiomics and Deep-Learning on MRI

D. J. Spaanderman[1], S. Hakkesteegt[1], D. Hanff[1], C. Messiou[2], L. Nardo[3], D. Grünhagen[1], C. Verhoef[1], M. P. A. Starmans[1], *S. Klein*[1]; [1]Rotterdam/NL, [2]London/UK, [3]Sacramento, CA/US
*(s.klein@erasmusmc.nl)*

**Purpose or Learning Objective:** Differentiating between lipomas and atypical lipomatous tumors (ALTs) on imaging is challenging, often requiring biopsies. This study aimed to externally and prospectively validate a radiomics model to distinguish between lipomas and ALTs using MRI across three large, multi-center cohorts. Additionally, the model was extended with automatic and minimally interactive segmentation methods to improve clinical applicability.
**Methods or Background:** Three cohorts were analyzed: two for external validation (US data from 2008–2018 and UK data from 2011–2017), and one for prospective validation (Netherlands, 2020–2021). Patient data, including MDM2 amplification status and MRI scans, were collected. An automatic segmentation method was developed for T1-weighted MRI scans, with interactive segmentation applied in case of poor quality. Radiomics model performance was compared with that of two radiologists.
**Results or Findings:** The cohorts included 150 (54% ALT), 208 (37% ALT), and 86 patients (28% ALT) from the US, UK, and Netherlands, respectively. Automatic segmentation succeeded in 78% of cases, while 22% required interactive segmentation, with only 3% needing manual adjustments. External validation yielded AUCs of 0.74 (95% CI: 0.66, 0.82) (US) and 0.86 (0.80, 0.92) (UK), and prospective validation achieved an AUC of 0.89 (0.83, 0.96) (Netherlands). The radiomics model performed similarly to radiologists in all cohorts.
**Conclusion:** The radiomics model, combined with automated and minimally interactive segmentation methods, effectively differentiated between lipomas and ALTs, matching the performance of expert radiologists and potentially reducing the need for biopsies.
**Limitations:** First, the segmentation workflow was performed by a single clinician, hence the potential impact of different users on radiomics performance was not assessed. Second, MDM2 amplification status, determined by core needle biopsy or resected specimens, may include false negatives, affecting the accuracy of the ground truth.
**Funding for this study:** This research was supported by an unrestricted grant of Stichting Hanarth Fonds, The Netherlands. MPAS and SK acknowledge funding from the research project EuCanImage (European Union's Horizon 2020 research and innovation programme under grant agreement Nr. 95210). This study was supported by the National Institute for Health Research (NIHR) Biomedical Research Centre at The Royal Marsden NHS Foundation Trust and The Institute of Cancer Research, London, and by The Royal Marsden Cancer Charity. The work was also supported by the In Vivo Translational Imaging Shared Resources with funds from NCI P30CA093373. The views expressed are those of the author(s) and not necessarily those of the NIHR or the Department of Health and Social Care.
**Ethics committee - additional information:** The study protocol was approved by the local medical ethics review committee (MEC-2020-0175), and performed in accordance with national and international legislation. Informed consent was required and obtained exclusively from participants in the prospective study cohort. For the training and external validation cohorts, approval by the local medical ethics review committee and the waiver of informed consent were previously reported.
**Author Disclosures:**
Stefan Klein: Nothing to disclose
Christina Messiou: Nothing to disclose
Douwe Jan Spaanderman: Nothing to disclose
Martijn Pieter Anton Starmans: Nothing to disclose
Lorenzo Nardo: Nothing to disclose
Dirk Grünhagen: Nothing to disclose
Stefanie Hakkesteegt: Nothing to disclose
Cornelis Verhoef: Nothing to disclose
David Hanff: Nothing to disclose

### Radiopsy, quantitative wb-mri adc and fat fraction sequences for discrimination of smoldering multiple myeloma and multiple myeloma: a prospective observational study

*G. Feliciani*[1], A. Rossi[1], C. Cerchione[1], E. Loi[1], E. Antognoni[1], A. Cattabriga[2], M. Marchesini[1], D. Barone[1], A. Sarnelli[1]; [1]Meldola/IT, [2]Bologna/IT
*(giacomo.feliciani@gmail.com)*

**Purpose or Learning Objective:** To distinguish between Multiple Myeloma and High-Risk Smouldering Myeloma at staging using image-based biomarkers obtained from Whole Body-MRI (WB-MRI) Apparent Diffusion Coefficient (ADC) and Fat Fraction (FF) sequences.
**Methods or Background:** From January 2021 to March 2024, we enrolled consecutive myeloma patients at staging into an prospective trial and divided them into Smouldering Multiple Myeloma (SMM) and Multiple Myeloma (MM).

Friday

All patients underwent WB-MRI. We use the term "Radiopsy" to indicate the quantification and modelling of image characteristics nearby the biopsy site to predict patient status. A radiologist placed a cylindrical VOI nearby the biopsy site and 5 more identical VOIs on distant sites such the pelvis bone and on D11 and L5 vertebrae. LASSO was used to select the most predictive features and build logistic regression models, which were then validated using the test set. ROC curves were used as metrics for models' performance assessment.
**Results or Findings:** The study included 102 patients (46 males, mean age 63 ± 12 [SD]) with 60 diagnosed with MM and 42 with SMM. 144 quantitative features were extracted from the VOI at the biopsy site WB-MRI ADC and FF sequences for each patient. Radiopsy model showed a median AUC of 0.80 (0.75-0.90) in the training phase and a median AUC of 0.70 (0.55-0.80) in the test phase. The best predictive model had an AUC of 0.95 and 0.75 in the training and test phase, respectively. The models used to predict patient status at biopsy site were also predictive in distant VOIs.
**Conclusion:** Conclusions: Radiopsy models can distinguish between MM and SMM with good performance nearby the biopsy site. Radiopsy can be used to predict disease invasion on distant sites where biopsy is not possible or not feasable
**Limitations:** Single center
**Funding for this study:** None
**Ethics committee - additional information:** protocol name: AccuMRI code: IRST 100.15
**Author Disclosures:**
Domenico Barone: Nothing to disclose
Claudio Cerchione: Nothing to disclose
Anna Sarnelli: Nothing to disclose
Eleonora Antognoni: Nothing to disclose
Arrigo Cattabriga: Nothing to disclose
Alice Rossi: Nothing to disclose
Emiliano Loi: Nothing to disclose
Matteo Marchesini: Nothing to disclose
Giacomo Feliciani: Nothing to disclose

### Feasibility of generating sagittal radiographs from coronal images using deep learning in adolescent idiopathic scoliosis

*M. E. Pellegrino*[1], T. Bassani[1], A. Cina[2], F. Galbusera[2], A. Cazzato[1], D. Albano[1], L. M. Sconfienza[1]; [1]Milan/IT, [2]Zurich/CH
*(maria.pellegrino@unimi.it)*

**Purpose or Learning Objective:** Minimizing radiation exposure is crucial in clinical monitoring of adolescent idiopathic scoliosis (AIS). Generative adversarial networks (GANs) have gained prominence in medical imaging due to their ability to learn complex patterns and generate high-quality synthetic images by transforming one type of image into another. This study explores GANs to generate synthetic sagittal radiographs from coronal views in AIS patients.
**Methods or Background:** A retrospective dataset of 3,935 AIS patients with mild-to-moderate scoliosis (Cobb angle <45°) was analyzed. The subjects underwent radiographic spine and pelvis examination using the EOS system, which acquires coronal and sagittal images simultaneously. The dataset was split into training (85%, n=3,356) and validation (15%, n=579). A pix2pix-based GAN model was trained to generate sagittal images from coronal views, targeting real sagittal views. To evaluate accuracy, 100 subjects from the validation set were randomly selected for manual measurement of lumbar lordosis (LL), sacral slope (SS), pelvic incidence (PI), and sagittal vertical axis (SVA) by two radiologists in both synthetic and real images.
**Results or Findings:** Of the 100 synthetic images, 69 were deemed assessable. Intraclass correlation coefficient ranged from 0.93 to 0.99 for measurements in real images and from 0.83 to 0.88 for synthetic images. Correlations between parameters in real and synthetic images (mean values between raters) were 0.52 (LL), 0.17 (SS), 0.18 (PI), 0.74 (SVA). Errors in parameters showed minimal correlation with Cobb angle. The mean±SD absolute errors were 7±7° (LL), 9±7° (SS), 9±8° (PI), 1.1±0.8 cm (SVA).
**Conclusion:** While the model generates sagittal images consistent with reference images, their quality is not sufficient for clinical parameter assessment, except for promising results in SVA, which describes lateral plumb line alignment.
**Limitations:** The quality of sagittal images is insufficient for assessing clinical parameters, except for SVA.
**Funding for this study:** Italian Ministry of Health.
**Ethics committee - additional information:** Not applicable.
**Author Disclosures:**
Andrea Cazzato: Nothing to disclose
Andrea Cina: Nothing to disclose
Luca Maria Sconfienza: Nothing to disclose
Fabio Galbusera: Nothing to disclose
Maria Elena Pellegrino: Nothing to disclose
Tito Bassani: Nothing to disclose
Domenico Albano: Nothing to disclose

**12:30-13:30 Research Stage 2**

### Research Presentation Session: Abdominal and Gastrointestinal

### RPS 1401

### Insights into gastroesophageal disease

**Moderator**
G. Bagnacci; Siena/IT
*(giulio.bagnacci@unisi.it)*

### Predicting Efficacy of Neoadjuvant Therapy in Esophageal squamous cell carcinoma Using Spectral CT Parameters

*J. Qu*, W. Li, F. Chu; Zhengzhou/CN
*(qjryq@126.com)*

**Purpose or Learning Objective:** To explore the differences in spectral CT parameters among patients with esophageal squamous cell carcinoma (ESCC), categorized by different tumor regression grade (TRG) groups.
**Methods or Background:** This prospective study analyzed 31 patients with ESCC who underwent neoadjuvant therapy. All patients received energy-spectrum CT scans within 7 days before surgery, and the following parameters were quantified: 40KeV, 70KeV, and 100KeV CT values in the arterial (AP) and venous phase (VP), iodine concentration (IC) in the normal esophageal wall, lesion and aorta, and Z Effective values. Finally, we assessed the differences in parameters among the different TRG groups (0, 1, 2, 3).
**Results or Findings:** In this study, a total of 19 participants were analyzed. 6 (31.58%) were in the TRG0 group, 3 (15.79%) in the TRG1 group, 6 (31.58%) in the TRG2 group, and 4 (21.05%) in the TRG3 group. Significant differences were observed among the four groups for arterial phase K40–70 keV (F=4.86, P=0.015) and K70–100 keV (F=4.78, P=0.016). However, no significant differences were found for NICD, NICratio, venous phase K40–70 keV and venous phase K70–100 keV (P>0.05).
**Conclusion:** Arterial phase parameters differ significantly among TRG groups, indicating potential in predicting tumor regression, while NICD, NICratio, and venous phase parameters do not.
**Limitations:** One limitation of this study is the small sample size, which may affect the generalizability of the findings. Additionally, the statistical methods employed were relatively simple, which may not fully capture the complexity of the data. Future research should involve larger sample sizes and develop models to further evaluate the significant parameters, enhancing the accuracy and reliability of the results.
**Funding for this study:** This study has received funding by the Projects of the General Program of the National Natural Science Foundation of China (No.82271979), Henan Province Medical Science and Technology Tackling Program Joint Construction Project (No. LHGJ20230096)，Henan Province Central Plains Talent Program (Nurturing talent Series)（No.20240220）
**Ethics committee - additional information:** The Research Ethics Committee of the Affiliated Cancer Hospital of Zhengzhou University (Henan Cancer Hospital) has approved this study (NCT03635619). Consent to participate Written informed consent was obtained from all subjects (patients) in this study.
**Author Disclosures:**
Wenshi Li: Nothing to disclose
Jinrong Qu: Nothing to disclose
Funing Chu: Nothing to disclose

### Diagnostic accuracy and reliability of CT-based Node-RADS for esophageal cancer

*J. Leonhardi*, B. Schnarkowski, T. Denecke, H-J. Meyer; Leipzig/DE
*(jakob.leonhardi@medizin.uni-leipzig.de)*

**Purpose or Learning Objective:** Staging in patients with oesophageal cancer is of high importance for treatment decisions. Regarding correct nodal staging, CT imaging was reported with low accuracy. Our observational retrospective study tried to elucidate the potential diagnostic benefit of the Node-RADS classification to discriminate benign from malignant lymph nodes.
**Methods or Background:** 126 patients (n= 15 females, 11.9%) with a mean age of 62.1 ± 10.4 years were included. Patients were surgically resected and lymph nodes were analyzed histopathologically. CT scans were acquired within one month before surgery. N = 182 lymph nodes were independently scored by two radiologists, following the Node-RADS classification. This lymph node scoring system ranges from 1 to 5, reflecting probability of malignancy (1–very low to 5–very high).
**Results or Findings:** 54 patients were nodal positive (42.9 %), whereas 72 patients were nodal negative (57.1 %). N1 stage was found in n= 28 cases

(22.2 %), N2 in n = 20 cases (15.9 %) and N3 stage in n = 6 cases (4.8 %). The tumors were squamous cell carcinomas (36 cases), adenocarcinomas (88 cases) and mixed adenoneuroendocrine carcinomas (2 cases). Inter-reader agreement reached k = 0.65 (p<0.001) for the overall Node-RADS scoring. Malignancy rates for Node-RADS-scores, ranging from score 1 - 5, were as following: 30 %, 14%, 81%, 90.1%, 86.5%. Total Node-RADS score showed statistically significant differences between N0 and N1-3 stage (N0: 2.68 ± 1.31 versus N1-3: 3.54 ± 1.11, p<0.001). ROC curve analysis yielded an AUC of 0.69, a threshold of 2 resulted in a sensitivity of 0.77 and a specificity of 0.55.
**Conclusion:** Node-RADS scores were associated with malignancy of lymph nodes and might help to improve staging in oesophagus carcinomas.
**Limitations:** For potential clinical translation there is need for external validation.
**Funding for this study:** This study did not receive funding of any kind.
**Ethics committee - additional information:** No. of the approval: 106-16-14032016
**Author Disclosures:**
Timm Denecke: Nothing to disclose
Hans-Jonas Meyer: Nothing to disclose
Jakob Leonhardi: Nothing to disclose
Benedikt Schnarkowski: Nothing to disclose

**The value of deep learning image reconstruction algorithm in improving dual-energy CT image quality of gastric cancer**
*T. Bei*, J. Li; Zhengzhou/CN
*(beitianxia@163.com)*

**Purpose or Learning Objective:** To investigate the value of deep learning image reconstruction algorithm (DLIR) in improving image quality of the dual-energy CT scans of gastric cancer by comparing with the adaptive statistical iterative reconstruction Veo (ASiR-V) algorithm.
**Methods or Background:** The original DECT images of 80 patients with surgical pathology confirmed gastric cancer between February 2023 and July 2023 were retrospectively collected. The virtual mono-energy images (VMI) and iodine images at three-phase including arterial phase, venous and delayed phase, were reconstructed using ASiR-V50% and DLIR of three strengths (DLIR-L/M/H). Objective evaluation indicators for assessing image quality included noise (SD), signal-to-noise ratio of gastric cancer lesions (SNR lesion) and muscle signal-to-noise ratio (SNR muscle), contrast-to-noise ratio of lesions (CNR lesion), and normalized iodine concentration (NIC). Subjective evaluation indicators included image noise and image sharpness. One-way analysis of variance and Kruskal-Wallis test were performed to compare the differences of image quality among different reconstruction images.
**Results or Findings:** Compared with ASiR-V50%, DLIR-M and DLIR-H significantly reduced the SD value of the images (P<0.05). The SNR lesion and SNR muscle of DLIR-M and DLIR-H were significantly higher than those of ASiR-V50% (P<0.05). The CNR lesion of DLIR-H were significantly higher than those of ASiR-V50% and DLIR-L (P<0.01). But compared with ASiR-V50%, there were no statistically significant difference in the NIC values of the iodine images between the groups (P>0.05). Subjective scoring results showed that the DLIR-H images displayed the lowest noise, and the highest image sharpness, significantly higher than ASIR-V50% (P<0.05).
**Conclusion:** Compared with ASiR-V50%, DLIR significantly reduced the noise of DECT images of gastric cancer and improved the image quality. Among the three deep learning reconstruction algorithms , DLIR-H had the best noise reduction performance, image noise and image sharpness.
**Limitations:** Not applicable.
**Funding for this study:** National Natural Science Foundation of China (82202146)
**Ethics committee - additional information:** This is a retrospective study
**Author Disclosures:**
Jing Li: Nothing to disclose
Tianxia Bei: Nothing to disclose

**Photon-Counting CT: Can Virtual Non-Contrast Match True Non-Contrast Imaging?**
*G. Marras*, F. Pisu, A. Palmisano, A. Esposito; Milan/IT
*(marras.gloria@hsr.it)*

**Purpose or Learning Objective:** Virtual Non-Contrast (VNC) has emerged as a potential alternative to True Non-Contrast (TNC) imaging, offering dose reduction while maintaining good tissue contrast. Photon-Counting CT (PCCT) for improved spectral separation should improve material decomposition providing accurate VNC. The aim of the present study is to evaluate the diagnostic reliability of VNC compared to TNC in PCCT images.
**Methods or Background:** This retrospective study included consecutive patients submitted to Contrast-Enhanced CT examination acquired with PCCT Naeotom Alpha (Iopromide or Iopamidol 370, 60 ml) from June to September 2024 for clinical indications with the availability of TNC. VNC were obtained from arterial (VNCa) and venous phases (VNCv) (Qr40, Q3). Using PACS tool, TNC, VNCa and VNCv were aligned and for the extraction of Hounsfield Unit, Regions of interest (ROI) were drawn in right liver lobe, spleen, kidney, pancreas, aorta, omental and subcutaneous fat, paravertebral muscle, vertebral cortical and spongious bone. Co-registration and minor adjustments were made to account for breathing variations. Attenuation values were then compared using Tukey's HSD or Dunn's test.
**Results or Findings:** A total of 38 studies have been analyzed. Significant differences in mean HU values between TNC and both VNC were observed for most tissues (p<.01), except the spleen. Highest attenuation differences were found for subcutaneous/omental fat and bone cortical and spongious with HU difference higher than 15HU in more than 50% of cases for fat and 100% for bone. No significant HU differences were found between VNCa and VNCv, except in the spleen.
**Conclusion:** VNC obtained from PCCT provide different attenuation values compared to TNC. Caution should be exercised in routine clinical practice.
**Limitations:** Higher sample size is required to confirm the present data.
**Funding for this study:** None
**Ethics committee - additional information:** Aimomics
**Author Disclosures:**
Antonio Esposito: Nothing to disclose
Anna Palmisano: Nothing to disclose
Gloria Marras: Nothing to disclose
Francesco Pisu: Nothing to disclose

**Sarcopenia effects on surgical outcomes and survival after gastrectomy (SESGa study)**
V. Sbacco, F. Puccetti, D. Palumbo, *G. J. Ortu*, A. Campisi, E. Mazza, F. De Cobelli, U. Elmore, R. Rosati; Milan/IT
*(gabrielejacopoo@gmail.com)*

**Purpose or Learning Objective:** Sarcopenia, defined as a progressive and generalized loss of muscle mass, strength, and function, is linked to increased morbidity and mortality in several nonmalignant conditions. Recently, it has emerged as an important factor in oncology, correlating with higher chemotherapy toxicity. However, its impact on survival and surgical outcomes in cancer patients remains unclear. This study evaluates the effects of sarcopenia on the response to neoadjuvant chemotherapy (NACT), overall survival, and postoperative outcomes in gastric cancer patients.
**Methods or Background:** A retrospective analysis was conducted on a prospective database of patients treated between September 2017 and December 2023. All patients received NACT with the FLOT regimen followed by total or subtotal gastrectomy (open/laparoscopic). Body composition parameters, including skeletal muscle area (SM), visceral (VAT), and subcutaneous adipose tissue (SAT), were measured from CT images before and after NACT using a semi-automated segmentation software (Sliceomatic 5.0). Sarcopenia was defined by the skeletal muscle index (SMI) adjusted for gender.
**Results or Findings:** The study included 101 patients, predominantly male (60.4%), with distal adenocarcinoma (71.3%). Post-NACT sarcopenia was present in 65 patients (64.4%), while visceral obesity was found in 33 (32.7%). NACT-induced sarcopenia had no significant effect on postoperative outcomes, except for a higher incidence of wound complications (p=0.012). Longitudinal changes in body composition showed a correlation between sarcopenia and a poorer tumor regression grade (TRG).
**Conclusion:** There is a strong association between NACT administration and the onset of sarcopenia (p = 0,001) , which, however, had minimal impact on survival and clinical outcomes in our center. This underscores the importance of completing the full neoadjuvant FLOT regimen.
**Limitations:** Although our goal is to expand it, our cohort of patients is currently quite limited. Further exploration of sarcopenia in gastric cancer is warranted.
**Funding for this study:** Unfunded study.
**Ethics committee - additional information:** 28/Int/2015
**Author Disclosures:**
Francesco Puccetti: Nothing to disclose
Diego Palumbo: Nothing to disclose
Gabriele Jacopo Ortu: Nothing to disclose
Elena Mazza: Nothing to disclose
Valentina Sbacco: Nothing to disclose
Antonino Campisi: Nothing to disclose
Riccardo Rosati: Nothing to disclose
Francesco De Cobelli: Nothing to disclose
Ugo Elmore: Nothing to disclose

**12:30-13:30** **Research Stage 3**

### Research Presentation Session: Cardiac

### RPS 1403

### Valves and pulmonary hypertension: the role of cardiac CT and MRI

**Moderator**
R. P. J. Budde; Rotterdam/NL
*(r.budde@erasmusmc.nl)*
**Author Disclosures:**
Ricardo P.J. Budde: Advisory Board: Bayer, payment to Erasmus MC; Board Member: ESCR; Research Grant/Support: Siemens, Heartflow, Bayer, Bracco all payements to Erasmus MC; Speaker: Bayer

### Mitral valve annulus assessment: a comparison between 3D-TOE, CCT and surgical ring

*E. Moliterno*, L. Giarletta, A. Pasquini, M. Massetti, L. Natale, R. Marano; Rome/IT
*(eleonora.moliterno@gmail.com)*

**Purpose or Learning Objective:** To compare the size assessment of the mitral annulus (MA) obtained by preoperative CCT with the intraoperative 3D-TOE in patients undergoing surgical mitral valve repair (MVr) and assess their agreement with the MA measured intraoperatively by the surgeon.
**Methods or Background:** 55 patients with severe primary mitral regurgitation and candidates to MVr by the Carpentier technique with annuloplasty underwent pre-surgery CCT to exclude coronary artery disease (CAD). We compared CCT-derived MA sizing on short-axis view according to the D-shape MV segmentation model obtaining inter-trigonal distance, septal-to-lateral and inter-commissural distances, with those obtained by 3D-reconstruction of intra-operative TOE and the surgical implanted ring sizes, using intraclass correlation.
**Results or Findings:** Good agreement resulted between the inter-trigonal distance measured by CCT and the surgical ring (ICC 0.89 [CI 0.330-0.985; p < 0.05]) and between inter-commissural distance obtained by 3D-TOE and surgical ring (ICC 0.81 [CI 0.458-0.936; p < 0.05]), while the inter-trigonal distance measured by 3D-TOE showed a moderate agreement (ICC 0.63 [CI 0.056-0.852; p < 0.05]). Excellent agreement resulted between the inter-trigonal distance assessed by intraoperative 3D-TOE and CCT (ICC 0.95 [CI 0.755-0.989; p<0.05]).
**Conclusion:** Our study shows the good accuracy of the pre-procedural CCT-based MA size assessment in comparison with the intraoperative 3D-TOE and the conventional surgical sizing, proposing CCT as a complementary non-invasive imaging technique in predicting eligibility and surgical ring sizing in patients candidate to MVr.
**Limitations:** The major study limitations are the relatively small sample size and the monocentric design.
**Funding for this study:** None
**Ethics committee - additional information:** No additional information
**Author Disclosures:**
Massimo Massetti: Nothing to disclose
Eleonora Moliterno: Nothing to disclose
Luigi Natale: Nothing to disclose
Annalisa Pasquini: Nothing to disclose
Riccardo Marano: Nothing to disclose
Lorenzo Giarletta: Nothing to disclose

### CT-based planning of transcatheter pulmonary valve implantation in patients operated for Tetralogy of Fallot and Double Outlet Right Ventricle

*P. Marakhouskay*, P. Chernoglaz, K. Marakhouski, K. Drozdovski; Minsk/BY
*(pmarakh@gmail.com)*

**Purpose or Learning Objective:** The aim of the study was to quantify the method of planning transcatheter pulmonary valve implantation (TPVI) in patients who have undergone surgery for congenital heart defects(CHD) by measuring in both ECG and non-ECG gated CTA sizes of pulmonary artery(PA).
**Methods or Background:** 22 patients with CHD as Tetralogy of Fallot and Double outlet Right Ventricle(DORV), operated in infancy, were included into study and divided into two groups: study group, in which direct intravascular balloon sizing of PA was performed, and control group in which TPVI was rejected for excessive artery size without performing angiography. CTA of all cases were retrospectively analyzed and 4 sizing zones were measured: RVOT, supravalvular zone, middle-segment of and bifurcation zone of PA. After that calculations to find appropriate patient -specific sizing methods by comparing parameters with an actual stent size were made (MedCalc Software Ltd, Ostend, Belgium; https://www.medcalc.org;2022).
**Results or Findings:** Analysis showed no significant correlation between diastolic RVOT sizing with any other measuring points or an actual stent size (P=0,0004). Only supravalvular zone was accepted as an independent residual in regression analysis (F-ratio 22, P=0,0004). After implementing a regression equation to calculate appropriate size of PA stent on the control group, all the stents exceeded 30 mm (TPVI exclusion point). Mann-Whitney U-Test showed that the significant difference between an actual stent size and calculated size for study group (U 91,00,P = 0,5678) and no significant difference between calculated sizes of two groups (U 0,50 P = 0,0004).
**Conclusion:** Our study showed that any cardiac CT-based preTPVI landing zone sizing in diastolic phase can be based solely on measuring supravalvular zone of PA (r= 0,7992, F-ratio 22, P=0,0004), especially as an exclusion criteria (U 0,50 P = 0,0004).
**Limitations:** None
**Funding for this study:** No funding
**Ethics committee - additional information:** Approved by the Ethics Committee of the NACPS No. 11 22.03.24.
**Author Disclosures:**
Kirill Marakhouski: Nothing to disclose
Palina Marakhouskay: Nothing to disclose
Konstantin Drozdovski: Nothing to disclose
Pavel Chernoglaz: Nothing to disclose

### Lung Perfusion Changes Following Pulmonary Valve Replacement in Repaired Tetralogy of Fallot Patients: A Time-Resolved MR Angiography Study

A. F. F. Tekin, *B. Tütüncüoğlu*, T. Banaz, Ö. Altun, Y. C. Kartal, S. Ozkök; Istanbul/TR
*(berk.tutun97@gmail.com)*

**Purpose or Learning Objective:** Tetralogy of Fallot (TOF) is a congenital heart disease surgically repaired in early life.Guidelines recommend pulmonary valve replacement (PVR) for these patients. However, the effects of PVR on lung perfusion are not yet fully understood.In our study, we aimed to evaluate the changes in lung perfusion following PVR in patients with surgically corrected TOF.
**Methods or Background:** The study included 10 patients with surgically corrected TOF (M/F: 6/4, mean age: 16 years, median: 11-19 years).Lung perfusion was assessed using 4D time-resolved magnetic resonance angiography (MRA) before and 1 year after pulmonary valve replacement (PVR) .Gadolinium-based contrast was administered and images were analyzed with Philips Intellispace workstation.For measurement, ROI 's were manually placed in the main pulmonary artery before bifurcation, the aortic arch, upper, middle, and lower lobes of the right and left lungs.Changes in both ventricular parameters, pulmonary insufficiency ratio, and perfusion were assessed before and after PVR. Statistical analyses were performed using SPSS .Data were reported as medians and the Wilcoxon-sum rank test was used.
**Results or Findings:** Following PVR, a reduction in pulmonary regurgitation fraction and right ventricular end-diastolic volume was observed, while no changes were seen in right ventricular ejection fraction and left ventricular volume. After PVR, a difference was noted in right and left lung perfusion but no differences were observed between lung zones.
**Conclusion:** Lung perfusion assessment is a non-invasive, radiation-free imaging technique for the diagnosis and monitoring of various respiratory diseases.Improved pulmonary artery flow volumes, reduced perfusion heterogeneity, and enhanced perfusion dynamics following PVR reflect a positive impact on pulmonary hemodynamics.MRA-perfusion imaging can be a valuable tool for evaluating perfusion changes in this patient population.
**Limitations:** The planning of retrospective studies using a larger number of patient groups is necessary.
**Funding for this study:** No funding was received for this study.
**Ethics committee - additional information:** This study was approved by Istanbul Medeniyet University Göztepe Training and Research Hospital Clinical Trials Ethics Commitee
**Author Disclosures:**
Serçin Ozkök: Nothing to disclose
Berk Tütüncüoğlu: Nothing to disclose
Ali Fuat Fuat Tekin: Nothing to disclose
Yiğit Can Kartal: Nothing to disclose
Tuba Banaz: Nothing to disclose
Ömer Altun: Nothing to disclose

**Role of CT-derived Extracellular Volume Fraction for Predicting Response to Percutaneous Aortic Valve Replacement**

*C. Gnasso*, D. Vignale, A. Palmisano, S. Barbieri, E. Agricola, A. Esposito; Milan/IT
*(gnasso.chiara@hsr.it)*

**Purpose or Learning Objective:** Aortic stenosis (AS) causes myocardial microstructural modifications: the development of interstitial fibrosis, in particular, negatively impacts patients’ prognosis. Echocardiography, the gold standard for AS phenotypization, can’t investigate microstructural changes. CT -routinely performed for pre-procedural TAVI planning- can be implemented with delayed scans to assess myocardial fibrosis as ECV quantification. The prognostic role of ECV has already been demonstrated. The study aims to investigate the capacity of ECV to improve risk stratification on top of echocardiographic classification (low-flow low-gradient AS -LF-LG- vs high-gradient AS -HG-).
**Methods or Background:** Prospective, single-center study (IRCCS OSR Milan), enrolling consecutive patients undergoing CT for TAVI planning (Oct2020-Mar2023); clinical, echocardiographic, and laboratoristic data were collected. Patients were categorized as LF-LG or HG. The CT protocol included a low-dose delayed scan (5 mins after iodixanol 320 administration, 85-110 mL based on patient’s BMI). ECV was calculated with the formula: (1-hematocrit)x(ΔHUmyocardium/ΔHUblood), with ΔHU being the HU-difference in pre and post-contrast scans. The composite endpoint (death; heart failure hospitalization) was collected after a 1-year follow-up.
**Results or Findings:** The final cohort consisted of 415 patients (82 years [78-85]); 87 reached the endpoint. At Cox multivariable analysis including clinical, echo, and CT data, ECV (using the cut-off of 29% derived from Youden index) resulted an independent prognosticator of the endpoint, along with sex, hypercholesterolemia, diabetes and transvalvular gradient, with HR of 1.828 (95%CI1.151-2.903, p=0.0106), significantly higher in the LF-LG group (31.9(27.6-35.6) vs 28 (25.4;31), p<0.001). At Kaplan-Meier analysis, adding ECV to echo classification led to an improvement in risk stratification: HG-AS patients with higher ECV had a worse prognosis even compared to LF-LG-AS with low ECV (log-rank<.0001).
**Conclusion:** CT-ECV improves risk stratification in AS patients on top of echocardiographic evaluation.
**Limitations:** Lack of MR as reference standard
**Funding for this study:** None
**Ethics committee - additional information:** All patients signed informed consent.
protocol number: CTMyoC 112/INT/2019
**Author Disclosures:**
Davide Vignale: Nothing to disclose
Antonio Esposito: Nothing to disclose
Anna Palmisano: Nothing to disclose
Simone Barbieri: Nothing to disclose
Eustachio Agricola: Nothing to disclose
Chiara Gnasso: Nothing to disclose

**Relationship between myocardial strain and extracellular volume: Exploratory study in patients with severe aortic stenosis undergoing photon-counting detector CT**

*C. Lisi*[1], V. Mergen[2], L. J. Moser[2], K. Klambauer[2], H. Alkadhi[2], M. Eberhard[2]; [1]Milan/IT, [2]Zurich/CH
*(costanza.lisi@hotmail.it)*

**Purpose or Learning Objective:** Diffuse myocardial fibrosis and altered deformation are relevant prognostic factors in aortic stenosis (AS) patients. The aim of this exploratory study was to investigate the relationship between myocardial strain, and myocardial extracellular volume (ECV) in patients with severe AS with photon-counting detector (PCD)-CT.
**Methods or Background:** We retrospectively included 77 patients with severe AS undergoing PCD-CT imaging for transcatheter aortic valve replacement (TAVR) planning between January 2022 and May 2024 with a protocol including a non-contrast cardiac scan, an ECG-gated helical coronary CT angiography (CCTA), and a cardiac late enhancement scan. Myocardial strain was assessed with feature tracking from CCTA and ECV was calculated from spectral cardiac late enhancement scans.
**Results or Findings:** Patients with cardiac amyloidosis (n=4) exhibited significantly higher median mid-myocardial ECV (48.2% versus 25.5%, p=0.048) but no significant differences in strain values (p>0.05). Patients with prior myocardial infarction (n=6) had reduced median global longitudinal strain values (-9.1% versus -21.7%, p<0.001) but no significant differences in global mid-myocardial ECV (p>0.05). Significant correlations were identified between global longitudinal, circumferential, and radial strains, and CT-derived left ventricular ejection fraction (EF) (all, p<0.001). Patients with low-flow, low-gradient AS and reduced EF exhibited lower median global longitudinal strain (GLS) values compared with those with high-gradient AS (-15.2% versus -25.8, p<0.001). In these patients, the baso-apical mid-myocardial ECV gradient correlated with GLS values (R=0.33, p<0.05).
**Conclusion:** In patients undergoing PCD-CT for TAVR planning, ECV and GLS may enable to detect patients with cardiac amyloidosis and patients with reduced myocardial contractility after myocardial infarction. Patients with low-flow, low-gradient AS and reduced EF showed lower median GLS correlating with basal LV fibrosis.
**Limitations:** Retrospective design, monocentric design, small sample number
**Funding for this study:** No funding
**Ethics committee - additional information:** No additional information
**Author Disclosures:**
Costanza Lisi: Nothing to disclose
Victor Mergen: Nothing to disclose
Matthias Eberhard: Nothing to disclose
Lukas Jakob Moser: Nothing to disclose
Konstantin Klambauer: Nothing to disclose
Hatem Alkadhi: Nothing to disclose

**Cardiac magnetic resonance 4d flow in mitral annuloplasty: impact on left ventricular flow dynamics and functional correlations with different types of devices**

*G. C. Pambianchi*, G. Cundari, L. Marchitelli, C. Catalano, N. Galea; Rome/IT
*(giacomo.pambianchi@gmail.com)*

**Purpose or Learning Objective:** To evaluate functional and fluidodynamic modifications after MAP with different types of annuloplasty prosthetics using CMR 4D-flow techniques and left atrial (LA) strain feature-tracking
**Methods or Background:** We enrolled 12 patients treated with MAP (7 semi-flexible and 5 flexible incomplete rings), and had them undergo CMR at least 12 months after the procedure. The protocol included cineMR and 4D-Flow sequences with a whole heart coverage. We quantified TotalFlowVolume (TFV), PeakVelocity (Vmax), and WallShearStress (WSS) at LV inflow. We analyzed LA volumes and strain. MAP were compared with 30 age- and sex-matched healthy controls (10 studied with 4D-flow)
**Results or Findings:** MAP patients had lower EF (49.3±4.2% vs62.34±6.3%; p<0.021), reduced TFV (52.3±7.8ml vs69.6±9.7ml; p=0.039), and increased Vmax (159.7±21.3cm/s vs125.1±35.3 cm/s; p=0.002) compared to controls; the WSS resulted comparable between the groups (0.23±0.11 vs0.21±0.44; p=0.354). As for the flow patterns analysis, intraventricular and beneath the mitral valve flow, there was similar vortex formation among the two groups. MAP patients had decreased Reservoir (20.6±20.1% vs22.9±2.5%; p=0.033), lower Conduit (9.1±3.48% vs12.7±1.8%; p = 0.005), and increased Booster Pump strain (12.4±1.8% vs8.9±2.3%; p=0.001) compared to controls. The time between the surgery and CMR was inversely correlated with TVF (r: -0.95; p=0.04) but didn't affect the WSS.
**Conclusion:** Mitral annuloplasty with leaflets preservation did not considerably alter intraventricular flow patterns compared to healthy controls; while the prosthetic ring causes slight stenosis, WSS isn't significantly increased. Atrial function was preserved in MAP but still reduced if compared to healthy controls. Detailed evaluation of hemodynamic changes post-mitral repair and potential impact on the choice of annuloplasty devices and techniques to optimize long-term outcomes.
**Limitations:** The study had a numerically limited sample, patients didn't have pre-annuloplasty CMR,. We didn't analyze TKE and viscous energy loss.
**Funding for this study:** Not applicable
**Ethics committee - additional information:** The study was submitted to local ethics committee
**Author Disclosures:**
Giacomo Carlo Pambianchi: Nothing to disclose
Giulia Cundari: Nothing to disclose
Livia Marchitelli: Nothing to disclose
Nicola Galea: Nothing to disclose
Carlo Catalano: Nothing to disclose

**The non-invasive right heart catheter: Hemodynamic classification of pulmonary hypertension using 4D flow MRI**

*G. Reiter*, G. Kovacs, C. Reiter, H. Olschewski, M. Fuchsjäger, U. Reiter; Graz/AT
*(gert.reiter@siemens-healthineers.com)*

**Purpose or Learning Objective:** Mean pulmonary arterial pressure (mPAP), pulmonary arterial wedge pressure (PAWP), and pulmonary vascular resistance (PVR) are the hemodynamic parameters measured during right heart catheterization (RHC) for diagnosis and classification of pulmonary hypertension (PH). This study aimed to assess the accuracy of 4D flow MRI in predicting these parameters non-invasively.
**Methods or Background:** 103 patients with known or suspected PH (PH/non-PH, 77/26) prospectively underwent both RHC and whole-heart 4D flow MRI at 3T. From 4D flow data, the duration of vortical blood flow along the main pulmonary artery (t_vortex), the left atrial acceleration factor (acc) and the cardiac output (CO_MR) were determined to derive estimates for mPAP via mPAP_MR = 16 + 0.63·t_vortex, PAWP via PAWP_MR = −6.2 + 10.1·[illegible], [illegible]d

PVR via PVR_MR = (mPAP_MR – PAWP_MR)/CO_MR. Relationships between invasive and 4D flow MRI-derived parameters were analyzed by correlation analysis and t-test. The performance of 4D flow MRI-derived parameters to predict the hemodynamic classification of PH was assessed by receiver operating characteristic curve analysis.
**Results or Findings:** The area under the curve (AUC) for predicting PH (mPAP > 20 mmHg) using mPAP_MR was 0.96. In PH patients, mPAP and mPAP_MR correlated strongly (r = 0.94) and showed no significant bias (0.6 ± 4.5 mmHg, p=0.24). Within PH patients, the AUCs for predicting post-capillary PH (PAWP > 15 mmHg) from PAWP_MR and PVR > 2 WU from PVR_MR were 1.00 and 0.95, respectively. PAWP and PAWP_MR correlated strongly (r = 0.94) and demonstrated no significant bias (0.4 ± 1.6 mmHg, p=0.05). PVR and PVR_MR correlated strongly (r = 0.85) but demonstrated a significant bias (0.8 ± 2.2 WU, p<0.01).
**Conclusion:** 4D flow MRI allows accurate non-invasive diagnosis and hemodynamic classification of PH.
**Limitations:** Single-center study
**Funding for this study:** None
**Ethics committee - additional information:** Medical University of Graz, Austria
**Author Disclosures:**
Horst Olschewski: Nothing to disclose
Ursula Reiter: Nothing to disclose
Gert Reiter: Employee: Research and Development Siemens Healthineers
Michael Fuchsjäger: Nothing to disclose
Clemens Reiter: Nothing to disclose
Gabor Kovacs: Nothing to disclose

### Feasibility of pulmonary arterial pulse wave velocity assessment from 4D flow MRI

*C. Reiter*, G. Reiter, G. Kovacs, D. Scherr, A. Schmidt, H. Olschewski, M. Fuchsjäger, U. Reiter; Graz/AT
*(Clemens.Reiter@medunigraz.at)*

**Purpose or Learning Objective:** To assess the feasibility of measuring pulmonary arterial pulse wave velocity (PWV) – a potential prognostic marker in pulmonary hypertension (PH) – from 4D flow MRI.
**Methods or Background:** Thirty-one healthy subjects (15 females; age, 60 ± 10 years) and 10 patients with PH (6 females; age, 66 ± 11 years; mean pulmonary arterial pressure, 46 ± 11 mmHg) were prospectively recruited for 4D flow MRI at 3T. PWV was calculated using the transit-time approach (cvi42). Centerline segmentation of the pulmonary artery was performed twice, from the main pulmonary artery once to the left and once to the right pulmonary artery; PWV was measured in the main pulmonary artery as well as the entire segmented vessels. The pulmonary artery cross-section area was obtained from a multiplanar-reconstructed plane through the center of the main pulmonary artery. Results were analyzed using t-tests and correlation analysis.
**Results or Findings:** In healthy subjects, the main pulmonary arterial PWV was 2.4 ± 0.2 m/s with no significant difference between the main-to-right (2.7 ± 0.3 m/s) and main-to-left pulmonary PWV (2.7 ± 0.3 m/s, p=0.583). Moreover, no significant sex differences were observed (p=0.430). In PH subjects the PWV was higher than in controls in the main (6.1 ± 1.8 m/s, p<0.001), the main-to-right (7.7 ± 3.0 m/s, p<0.001) and the main-to-left pulmonary artery (7.1 ± 2.5 m/s, p<0.001). Main, main-to-right and main-to-left pulmonary artery PWV correlated significantly with the average pulmonary artery cross-section area (r = 0.78, 0.61 and 0.59, respectively; p<0.001 in all cases).
**Conclusion:** Pulmonary arterial PWV assessment from 4D flow MRI is feasible. Normal ranges align with values reported from 2D flow measurements, show small variations in healthy subjects, and differ compared PWV in patients with PH.
**Limitations:** Small sample size.
**Funding for this study:** OeNB Anniversary Fund 17934
**Ethics committee - additional information:** Approval was obtained from the Ethics committee of the Medical University of Graz, Austria.
**Author Disclosures:**
Horst Olschewski: Nothing to disclose
Ursula Reiter: Nothing to disclose
Gert Reiter: Employee: Research & Developement Siemens Healthineers
Michael Fuchsjäger: Nothing to disclose
Daniel Scherr: Nothing to disclose
Clemens Reiter: Nothing to disclose
Albrecht Schmidt: Nothing to disclose
Gabor Kovacs: Nothing to disclose

**12:30-13:30** **Research Stage 4**

### Research Presentation Session: Neuro

### RPS 1411

### Charting the brain's next frontier: glymphatic system imaging

**Moderator**
E. T. Tali; Ankara/TR
*(turgut.tali@gmail.com)*

### Association of Sleep Quality and Memory Performance With Glymphatic Function in Medical Interns and Residents

*H. Wu*; Chongqing/CN
*(cefradine1005@gmail.com)*

**Purpose or Learning Objective:** Poor sleep quality is prevalent among medical interns and residents, often characterized by insufficient sleep .Our objective is to examine the associations between glymphatic function, sleep quality, and neuropsychological performance within this population.
**Methods or Background:** 30 medical Interns and 15 residents on 2-week Internal Medicine and Radiology rotations were included . Sleep profile was accessed using questionnaires and polysomnography. Wechsler Memory Scale (WMS) was used for evaluation of the memory performance. The MRI for each subject was scheduled at least 72 hours after the completion of the night shift. Diffusion tensor imaging analysis along the perivascular space (DTI-ALPS) index was used to evaluate glymphatic function.
**Results or Findings:** Multivariate linear regression model for interns group determined that intern year (unstandardized β=-0.0045 [SE 0.0001]; p < 0.001), and the N3 sleep duration, index (unstandardized β=0.0071 [SE=0.0004]; p = 0.0013) were independently associated with DTI-ALPS. Increased DTI-ALPS was linked to enhanced memory scores (unstandardized β = −0.77 [SE = 0.23]; p = 0.014). Interns group had higher percentages of N1 and N2 sleep and a modestly higher amount of REM sleep than residents group. The higher percentages of N1, N2, and REM sleep were offset by a lower percentage of N3 sleep in interns. There was no significant difference of memory scales between these two groups.
**Conclusion:** Interns tend to have poorer sleep structure compared to residents, likely due to their more frequent extended overnight shifts, whereas residents rarely or never work such extended shifts. This study identified associations between DTI-ALPS and memory performance, suggesting the potential of DTI-ALPS as a biomarker for evaluating the neuropsychological status of young doctors.
**Limitations:** Our study does not include longitudinal data on temporal changes in DTI-ALPS between night shifts for individual participants.
**Funding for this study:** None
**Ethics committee - additional information:** The authors report no disclosures relevant to the Abstract.
**Author Disclosures:**
Hao Wu: Nothing to disclose

### Small vessel disease and glymphatic changes in hospitalized and non-hospitalized COVID-19 patients: A study on peak skeletonised mean diffusivity and DTI-ALPS

B. Genç, *A. Özçağlayan*, M. S. Buruk, L. Incesu, K. Aslan; Samsun/TR
*(ozcaglayan.ali@gmail.com)*

**Purpose or Learning Objective:** COVID-19 has also been associated with the development of dementia, cortical atrophy, and cognitive impairments such as brain fog, which are challenging or often impossible to detect using conventional MRI. Peak width of skeletonized mean diffusivity (PSMD), a recently developed quantitative marker, has been proposed as a sensitive biomarker for small vessel disease. The aim of this study is to investigate changes in PSMD, associated with small vessel disease, and DTI-ALPS parameters, related to the glymphatic system, in COVID-19 patients.
**Methods or Background:** Clinical, demographic data, and MRI images were obtained from the "neuroCOVID MRI dWIand fMRI with reversal learning" dataset available on OpenNeuro (https://openneuro.org/datasets/ds005364/versions/1.0.0 . After denoising and eddy current corrections, DTI-ALPS and PSMD index measurements were performed similarly to the previous literature. The values of the hospitalized and non-hospitalized COVID groups were compared separately with the control group.

**Results or Findings:** The PSMD index was significantly higher in the hospitalized COVID-positive group (326 x$10^{-6}$ mm²/s) compared to the control group (298 x$10^{-6}$ mm²/s, p=0.028), while there was no significant difference between the non-hospitalized COVID-positive group (301 x$10^{-6}$ mm²/s) and the control group (p=0.953). no significant differences were observed in left or right DTI-ALPS values between the hospitalized, non-hospitalized COVID-positive groups, and the control group.
**Conclusion:** Our study shows that hospitalized COVID-19 patients exhibit an increase in PSMD index, indicative of small vessel disease, while no such risk exists in non-hospitalized COVID-19 patients. Additionally, contrary to initial concerns, COVID-19 does not appear to cause glymphatic dysfunction.
**Limitations:** The use of the DTI-ALPS method to measure glymphatic function remains controversial. Perivascular spaces account for only 1% of cerebral tissue, making it difficult for this method to differentiate diffusion within perivascular spaces from diffusion along other axes.
**Funding for this study:** No funding was received for this study.
**Ethics committee - additional information:** We did not apply for ethics approval as the data utilized in this study were obtained from the OpenNeuro database.
**Author Disclosures:**
Mehmet Seyfi Buruk: Nothing to disclose
Bariş Genç: Nothing to disclose
Lütfi Incesu: Nothing to disclose
Kerim Aslan: Nothing to disclose
Ali Özçağlayan: Nothing to disclose

### Changes in cerebrospinal and vitreous fluid density after iodine contrast administration in acute ischemic stroke patients: potential role of the glymphatic system?

V. Khasminsky, J. Naftali, *G. Danieli*, E. Uriel; Tel Aviv/IL
*(guydanieli87@gmail.com)*

**Purpose or Learning Objective:** The aim of our study was to measure the changes in density of the ventricular CSF and vitreous fluid of the eyes in AIS patients who underwent emergent diagnostic CT work-up and endovascular thrombectomy. Our assumption was that iodine contrast medium leaked into infarcted brain parenchyma would be cleared by the glymphatic system into CSF and would be detected there by CT.
**Methods or Background:** A cohort of 119 subjects with the diagnosis of AIS who underwent head NCCT, CT-angiography, CT-perfusion, endovascular thrombectomy, and another follow-up head NCCT in up to 36 hours was selected. The density of CSF in Hounsfield units (HU) was measured in the frontal horns of lateral ventricles, in the third ventricle and in the vitreous body of both eyes on the admission head NCCT and compared to the same measurements on the follow-up head NCCT.
**Results or Findings:** Small but statistically significant increases in HU were observed across various locations on the follow-up CT. The average HU in the frontal and third ventricles combined rose from 3.89±1.49 to 5.01±2.24, a difference of 1.12±2.14 (P < 0.001). The average HU in both eyeballs rose from 5.93±2.75 to 7.14±4.18, a difference of 1.2±4.57 (P = 0.003). In the subgroup with ASPECT score below 8 we found a difference of 1.68±2.14 (P < 0.001) in the ventricles and 3.16±5.29 (P < 0.011) in the vitreous.
**Conclusion:** Our findings can be explained in the framework of glymphatic system theory by the clearence of leaked iodine via the bulk flow of CSF in the perivenular space to the lateral ventricles. The density increase in the vitreous fluid may be due to secondarily increased permeability of blood-retinal barrier or connections between intra-cranial and intra-ocular glymphatic systems.
**Limitations:** Not applicable.
**Funding for this study:** No funding was provided for this study.
**Ethics committee - additional information:** Uid: Rmc-0825-23
**Author Disclosures:**
Guy Danieli: Nothing to disclose
Vadim Khasminsky: Nothing to disclose
Jonathan Naftali: Nothing to disclose
Eitan Uriel: Nothing to disclose

### Diffusion tensor imaging along the perivascular space (DTI-ALPS) for the glymphatic system evaluation in idiopathic and GBA-associated Parkinson’s disease

L. De Carolis, *R. Pascuzzo*, D. Aquino, S. A. Della Seta, R. Eleopra, F. Doniselli, R. Cilia; Milan/IT
*(riccardo.pascuzzo@istituto-besta.it)*

**Purpose or Learning Objective:** Glymphatic system dysfunction has been associated with neurodegeneration in Parkinson's disease (PD). However, its role in PD patients carrying GBA mutations (GBA-PD), a key genetic risk factor for PD, remains unclear. This study aimed to compare glymphatic function in idiopathic PD (iPD) and GBA-PD using Diffusion Tensor Imaging Along the Perivascular Spaces (DTI-ALPS) and investigate correlations with motor and non-motor symptoms.
**Methods or Background:** Brain DTI sequences (32 directions, b=1000 s/mm2) acquired with a 3T MRI scanner were retrospectively collected from 40 PD (20 iPD, 20 GBA-PD) and 40 age- and sex-matched patients with essential tremor (ET, serving as controls). DTI-ALPS indeces were computed for both hemispheres using Taoka’s method. Severity of motor and non-motor symptoms of PD patients were assessed by Hoehn and Yahr (H&Y) and Unified Parkinson’s Disease Rating scales (UPDRS). DTI-ALPS values were compared between groups of patients using Mann-Whitney test and were correlated with clinical variables using Spearman’s (rho) correlation coefficient.
**Results or Findings:** DTI-ALPS values were similar between iPD and GBA-PD patients. However, DTI-ALPS values in the hemisphere contralateral to the less affected side (DTI-ALPS-ctrl) were significantly higher (p<0.001) in PD patients with H&Y stage <2 (1.42±0.07) compared to others (PD with H&Y stage ≥2: 1.26±0.18; ET: 1.27±0.19). In GBA-PD patients, DTI-ALPS-ctrl values were positively correlated with visual memory (rho=0.726, p<0.001) but negatively correlated with sleep disturbances (rho=-0.576, p=0.008), while in iPD they were negatively correlated with age at onset (rho=-0.591, p=0.006), worse response to dopaminergic medication (rho=-0.588, p=0.007), and total UPDRS score (rho=-0.591, p=0.006).
**Conclusion:** Glymphatic dysfunction is linked to disease severity in PD and may play distinct roles in the pathology of iPD and GBA-PD.
**Limitations:** The study limitations are the relatively small sample size and the absence of healthy controls.
**Funding for this study:** Funding was provided by the Italian Ministry of Health
**Ethics committee - additional information:** The study is retrospective
**Author Disclosures:**
Sara Adriana Della Seta: Nothing to disclose
Domenico Aquino: Nothing to disclose
Fabio Doniselli: Nothing to disclose
Roberto Eleopra: Nothing to disclose
Roberto Cilia: Nothing to disclose
Lanfranco De Carolis: Nothing to disclose
Riccardo Pascuzzo: Nothing to disclose

### Diffusion-Tensor MRI Study of the relationship between glymphatic system asymmetry and onset lateralization in Parkinson's disease

*Z. Li*; Nanjing/CN
*(1600372734@qq.com)*

**Purpose or Learning Objective:** Parkinson's disease (PD) is commonly characterized by asymmetric motor symptoms. Previous studies have implicated abnormal α-synuclein aggregation as a key factor in PD pathogenesis and suggested that glymphatic system dysfunction may be responsible for the disease progression. However, the relationship between glymphatic system asymmetry and the side of motor symptom onset in PD remains unclear.
**Methods or Background:** 27 left-onset PD (LPD) patients, 36 right-onset PD (RPD) patients, and 49 age- and sex-matched healthy controls (HCs) were included in this study. The bilateral hemispheric ALPS indices were used to evaluate glymphatic function. Asymmetry of the glymphatic system was assessed by the asymmetry index (AI). Partial correlation analysis was conducted to examine the relationship between impaired glymphatic system function and motor deficits.
**Results or Findings:** Compared to HCs, RPD patients exhibited a significant reduction in the left ALPS index, while both left and right ALPS indices were significantly reduced in LPD patients. In both LPD patients and HCs, the right ALPS index was lower than the left, suggesting a natural leftward asymmetry. However, this asymmetry was diminished in RPD patients, as indicated by a reduced AI. Moreover, in RPD patients, the Unified Parkinson's Disease Rating Scale Part III score showed a negative correlation with the left ALPS index, and with AI.
**Conclusion:** This study demonstrated PD patients with different onset sides have different patterns of glymphatic system function. The glymphatic system asymmetry may provide new insights into the mechanism underlying the lateralized onset of PD.
**Limitations:** Firstly, being a retrospective cohort study conducted at a single center, it involved a relatively small sample size. Secondly, further research is needed to investigate the potential therapeutic implications of targeting the glymphatic system in PD
**Funding for this study:** National Natural Science Foundation of China (81671258) Nanjing Medical University-Qilu Clinical Research Fund Project (2024KF0254)
**Ethics committee - additional information:** This study was approved by the Ethics Committee of the First Affiliated Hospital of Nanjing Medical University, and the requirement for written informed consent was achieved. IRB number: No. 2014-SRFA-097.
**Author Disclosures:**
Zihan Li: Nothing to disclose

**Glymphatic System Evaluation in Essential Tremor and Parkinson’s Disease: an MRI Study Using Diffusion Tensor Imaging Analysis Along Peri-vascular Spaces (DTI – ALPS)**
*A. Innocenzi*, M. Cella, G. Saltarelli, P. Badini, F. Pistoia, A. Catalucci, F. Bruno, E. Di Cesare, A. Splendiani; L'Aquila/IT

**Purpose or Learning Objective:** This study aims to assess the presence of glymphatic system alterations in patients with Essential tremor (ET) and Parkinson’s disease (PD) through the analysis of diffusion tensors along perivascular spaces (DTI-ALPS index).
**Methods or Background:** We retrospectively evaluated 35 patients (19 PD, 16 TE, 28 males, mean age 67 years) who were eligible for Vim thalamotomy using MRgFUS (2018-2022) and 17 healthy controls (13 female, mean age 39 years). All MR images were obtained using a 3.0 Tesla MRI and a 32-channel head coil. DTI images were analyzed using open-source software. The ALPS indices were compared between the ET and PD patient populations, respectively. Both these populations were compared to healthy controls.
**Results or Findings:** PD and ET groups were matched for disease duration, age, gender and cognitive score. The ALPS index in PD patients showed a mean of 1.33, with no statistically significant differences compared to ET patients (1.31, p-value = 0.31), both reduced compared to healthy controls (1.62; p-value < 0.001). We observed a more intense tremor in ET patients rather than in PD patients (FTM 34,14 VS 28,64; p-value = 0.041). There was a significant correlation between the ALPS index and tremor intensity in ET patients (R = -0.76; p-value < 0.001). We did not find statistically significant correlations between ALPS –index and MoCA score in PD and TE groups.
**Conclusion:** There were no differences observed in the MRI indices of the glymphatic system between ET and PD patients, and both are reduced with respect to healthy subjects. Our results suggest the need for further studies to better define the role of the ALPS index as a marker of disease progression and to evaluate the possibility of neuro-degenerative pathophysiology in ET.
**Limitations:** Small sample size.
**Funding for this study:** This research received no external funding.
**Ethics committee - additional information:** Our study received approval from the Internal Review Board of the University of L'Aquila (protocol code 21 January 2020 n. 01/2020), and all participating patients provided signed informed consent to take part in the study.
**Author Disclosures:**
Alessandra Splendiani: Nothing to disclose
Gaspare Saltarelli: Nothing to disclose
Antonio Innocenzi: Nothing to disclose
Ernesto Di Cesare: Nothing to disclose
Francesca Pistoia: Nothing to disclose
Marco Cella: Nothing to disclose
Alessia Catalucci: Nothing to disclose
Federico Bruno: Nothing to disclose
Pierfrancesco Badini: Nothing to disclose

**Glymphatic system evaluation in idiopathic intracranial hypertension-can ALPS index solve all issues of the disease?**
*R. Dahiya*, S. Rohilla; Rohtak/IN

**Purpose or Learning Objective:** idiopathic intracranial hypertension (IIH) is a common headache disorder in young and middle age females. A few theories postulate impaired cerebral glymphatic clearance in IIH, however there is a paucity of methods to quantify glymphatic activity in human brains. The purpose of this study was to use diffusion-tensor imaging to evaluate the glymphatic clearance of IIH patients and how it may relate to various clinical parameters.
**Methods or Background:** This observational cross-sectional study was conducted on 25 patients clinically diagnosed with IIH. DTI was used to separately evaluate the diffusivity in lateral association and projection fibers, with the degree of diffusivity used as a surrogate for glymphatic function (diffusion tensor image analysis along the perivascular space). Glymphatic clearance was correlated with several clinical metrics, including lumbar puncture opening pressure and Frisen papilledema grade and combined conduit score for transverse sinus stenosis.
**Results or Findings:** At an ALPS Index cut off of 1.64, ROC curve analysis showed sensitivity of 100%, specificity of 84.6%, PPV of 83.3%, NPV of 100%, DA of 91.3% and PLR of 6.5 with AUC of 0.973 demonstrating excellent diagnostic performance in differentiating severe from mild IIH. Also ALPS index showed significant negative correlation with BMI(P=0.012), CSF pressure(p=0.032), papilledema(p=0.001) indicating increasing ALPS index(i.e.worsened glymphatic clearance) with increasing severity of disease. CSF pressure and papilledema showed positive correlation(p=0.008).
**Conclusion:** Our results show that patients with IIH possess impaired glymphatic clearance, which is directly related to the extent of clinical severity and ALPS Index can be used for clinical diagnosis as a non invasive investigation.
**Limitations:** Since we did not record response to treatment on most recent follow up and its correlation with ALPS, we could assess whether ALPS can be used to guide treatment for IIH
**Funding for this study:** Not provided
**Ethics committee - additional information:** The ethics committee notification can be found under the
number BREC/22/231.
**Author Disclosures:**
Seema Rohilla: Nothing to disclose
Ravi Dahiya: Nothing to disclose

**Evaluation of the glymphatic system in patients with intracranial tumours with blood-brain barrier disruption**
*L-P. Schmill*, S. Seehafer, S. Aludin, S. Peters, O. Jansen, N. Larsen; Kiel/DE
*(lars-patrick.schmill@uksh.de)*

**Purpose or Learning Objective:** After decades of doubt, it has finally been proven that the human brain has its own glial-lymphatic system. Its role in various diseases has been studied and discussed. Although its exact function is still unclear, perivascular spaces and fluid transport by glial cells are thought to be the main components, and drainage appears to occur via the perivenous spaces and meningeal lymphatic vessels. These have been delineated by MRI for the first time in recent years, but there is still a lack of established methods to assess their functionality. The aim of this study was to investigate whether intracranial tumours have increased drainage via adjacent meningeal lymphatic vessels.
**Methods or Background:** MRI scans were performed on patients with glioblastomas and cerebral metastases of different primary tumours. The protocol included a fluid-attenuated inversion recovery sequence and a contrast-enhanced T1-weighted black blood sequence, which can be used to delineate meningeal lymphatic vessels. This study looked specifically at the contrast enhancement of these vessels and compared the signal intensities of lymphatics near the intracranial mass with those contralateral to it.
**Results or Findings:** Results from the first 5 out of 30 patients show increased visual contrast enhancement in the meningeal lymphatics adjacent to the tumour compared to the contralateral side. These results indicate an increased lymphatic drainage of intracranial masses in addition to their venous drainage.
**Conclusion:** The current aim is to gain new insights into the glymphatic system and its relationship with the pathogenesis of brain diseases. This study has already provided evidence for increased glymphatic drainage in intracranial tumours. However, the exact drainage areas and which sequences that can be used for optimal detection of lymphatic vessels are still unclear.
**Limitations:** Not applicable
**Funding for this study:** No funding was provided for this study.
**Ethics committee - additional information:** Ethic approval was obtained from the IRB of the Medical Faculty of the Christian-Albrechts-University Kiel.
**Author Disclosures:**
Sönke Peters: Nothing to disclose
Lars-Patrick Schmill: Nothing to disclose
Schekeb Aludin: Nothing to disclose
Svea Seehafer: Nothing to disclose
Olav Jansen: Nothing to disclose
Naomi Larsen: Nothing to disclose

14:00-15:30 Research Stage 1

### Research Presentation Session: Emergency Imaging

### RPS 1517

### Novel approaches in emergency imaging strategies

**Moderator**
A. Ugarte; San Sebastian/ES
*(augarte8689@gmail.com)*

**Risk factors and prognostic significance of inferior vena cava volume defined by initial polytrauma CT-imaging**

*H-J. Meyer*, V. Sotikova, T. Denecke, M. Struck; Leipzig/DE
*(jonas90.meyer@web.de)*

**Purpose or Learning Objective:** The role of the inferior vena cava (IVC) volume measurement in trauma patients is not yet fully understood. The aim of the present study was to identify associations between the IVC volume and red blood cell (RBC) transfusion and massive transfusion (≥10 RBC) within 24 hours after admission, as well as 24-hour and 30-day mortality in trauma patients.
**Methods or Background:** A retrospective analysis was conducted on all consecutive trauma patients who required emergency tracheal intubation and mechanical ventilation before initial whole-body CT imaging at a level-1 trauma center over a 12-year period. The IVC volume was determined in the initial trauma CT scan.
**Results or Findings:** A total of 438 patients (75.3% male) with a median age of 50 years, and a median injury severity score (ISS) of 26 points were included in the analysis. Median IVC volume was 36.25 cm3, and RBC transfusion and massive transfusion were performed in 197 and 90 patients, respectively. The 24-hour and 30-day mortality rates were 7.3% and 23.3%, respectively. IVC volume was found to be independently associated with the necessity of RBC transfusion and 24-hour mortality (HR 0.98, 95% CI 0.96–0.99, p =0.01 and HR 0.96, 95% CI 0.93–0.99, p =0.025, respectively), while associations with massive transfusion and 30-day mortality were not statistically significant in multivariable analyses.
**Conclusion:** The initial IVC volume may serve as a predictor of the general need for RBC transfusion, although it does not reach the prognostic threshold for massive transfusion. The association with 24-hour mortality rather than 30-day mortality suggests the possibility of its diagnostic efficacy in short-term outcomes.
**Limitations:** First, it is a single center retrospective study. Second, there might be bias induced by infusion therapy before the CT.
**Funding for this study:** None
**Ethics committee - additional information:** Ethics committee at the Medical Faculty of Leipzig University, Leipzig, Germany (IRB00001750, project ID 441/15ek, September 14, 2020)
**Author Disclosures:**
Timm Denecke: Nothing to disclose
Manuel Struck: Nothing to disclose
Hans-Jonas Meyer: Nothing to disclose
Veronika Sotikova: Nothing to disclose

**Unmasking the bleeding: post-traumatic CT-scan angiography of pelvic ring with and without pelvic belt**

*L. J. Pavan*, N. Ouamrane, G. Paesani, P-A. Ranc, K. Desalos, T. Vivarrat-Perrin, M-E. Amoretti, N. Amoretti; Nice/FR
*(lucajpavan@gmail.com)*

**Purpose or Learning Objective:** Patients who suffered a high energy trauma with pelvic ring fracture usually come to whole-body CT-room with a pelvic binder that improves outcome but may mask an active bleeding during CT contrast injection. Aim of study was to evaluate the importance of removing pelvic binder in the early imaging of pelvic ring fracture.
**Methods or Background:** All consecutive post-traumatic whole-body CT-scans performed in our emergency department from January 2022 to December 2023 were reviewed. Patients with pelvic ring fracture and a 3-phase CT evaluation (non-contrast, late arterial and portal venous) were included. CT-scan were directly performed without pelvic binder for hemodynamically stable patients. For hemodynamically unstable patients a first acquisition was performed with a tighten pelvic binder, and if no bleeding was found a second 2-phase contrast injection was realized after loosening the pelvic binder to reveal any possible hidden bleeding.
**Results or Findings:** Out of 847 whole-body CT scan performed in the considered period, a total of 149 patients (87 men, 62 women, mean age 43,6±17 years) with a pelvic ring fracture (78 Tile-A, 42 Tile-B, 29 Tile-C) were included. Seven patients were hemodynamically unstable, requiring a first CT examination with tighten pelvic binder and a second acquisition with loosen pelvic binder. Of these, 2 patients (2/7, 29%) showed a pelvic active bleeding only with loosen pelvic binder.
Of the 142 hemodynamically stable patients who directly underwent CT without binder, 10 presented an active bleeding.
**Conclusion:** Pelvic binders are a useful tool but may interfere with CT imaging, masking an active pelvic bleed. An acquisition with loosen pelvic binder should always be performed in order to avoid false negative examination.
**Limitations:** The retrospective design of the study.
**Funding for this study:** None
**Ethics committee - additional information:** None since observational and retrospective
**Author Disclosures:**
Luca Jacopo Pavan: Nothing to disclose
Kevin Desalos: Nothing to disclose
Nadine Ouamrane: Nothing to disclose
Thomas Vivarrat-Perrin: Nothing to disclose
Gaelle Paesani: Nothing to disclose
Nicolas Amoretti: Nothing to disclose
Paul-Alexis Ranc: Nothing to disclose
Marie-Eve Amoretti: Nothing to disclose

**Single-pass split-bolus abdominal computed tomography (CT) versus conventional biphasic CT in abdominal trauma patients**

*S. Gautam*, R. Gupta, A. Sharma; New Delhi/IN
*(shubhamgautam78866@gmail.com)*

**Purpose or Learning Objective:** To compare the image quality in single-pass split-bolus abdominal computed tomography (CT) and conventional biphasic CT in abdominal trauma patients.
**Methods or Background:** 66 consecutive patients of abdominal trauma referred for CT were randomized into two groups: the study group (n = 33) scanned using the split-bolus technique; and the control group (n = 33) scanned using the conventional biphasic technique. CT image quality was analyzed subjectively by two observers based on a 5-point Likert scale. The images were also analyzed quantitatively for attenuation values achieved by region of interest (ROI) placements in major arteries, veins and solid organs. In addition, radiation dose in terms of Dose Length Product (DLP) was compared in the two groups.
**Results or Findings:** The image quality in both groups ranged from good to excellent in most cases. There was no statistically significant difference in subjective image quality in both the groups as assessed by Likert score. Attenuation values in solid organs and major venous structures were significantly higher in the split-bolus group (p <0.001). Arterial attenuation values were significantly higher in the control group (p <0.001) but diagnostic levels were achieved in all patients. There was a reduction of 31.1% in DLP in the split-bolus group.
**Conclusion:** Split-bolus technique offers comparable image quality and higher solid organ and venous enhancement than conventional biphasic protocol at a reduced radiation dose.
**Limitations:** The sample size was limited, excluding pediatric patients. The split-bolus protocol used a fixed 120 ml contrast dose, unlike the weight-based regime in dual-phase CT. Most patients were young males, so the average contrast in the dual-phase group was similar. Although whole-body CT is increasingly used in trauma centers, we applied both protocols only for abdominal scans.
**Funding for this study:** None
**Ethics committee - additional information:** Abstract is approved by institutional ethics committee
**Author Disclosures:**
Shubham Gautam: Author: 1st author
Anuradha Sharma: Author: 2nd author
Rohini Gupta: Author: Corresponding author

**Contrast Timing Pulmonary CT-Angiography: fixed trigger delay in the ascending aorta vs. pulmonary trunk**

*G. G. De Almeida*, O. Krzystek, J. Heimer, T. Niemann, A. Euler; Baden/CH

**Purpose or Learning Objective:** CT angiography (CTA) is the gold standard for diagnosing pulmonary artery embolism (PE). Optimal scan timing is crucial for homogeneous enhancement of the pulmonary arteries (PA). Clinical differentiation between PE and aortic dissection is not always clear. This study tested whether bolus tracking in the ascending aorta provides sufficient diagnostic enhancement of the PA, whilst improving enhancement in the thoracic aorta in patients with suspected PE, with the objective of allowing additional diagnostic information within the same exam.
**Methods or Background:** Retrospective image analysis of 200 patients scanned for PE between 03.2024 and 07.2024 was conducted. Patients were imaged using a third-generation dual-source CT with application of 70 mL of iodinated contrast medium and bolus tracking triggering in either the pulmonary trunk (A) or the ascending aorta (B). A fixed trigger delay of 7 seconds and automatic tube voltage selection were applied. CT attenuation and contrast-to-noise ratio (CNR) were measured at the pulmonary trunk, main pulmonary arteries, segmental superior lobe arteries, ascending, and descending aorta. A mixed-effects model and post-hoc tests were applied.
**Results or Findings:** No significant difference in CNR was found when comparing both techniques for the pulmonary tree (all p>.05). Mean CNR at the pulmonary trunk, main pulmonary artery, and segmental pulmonary arteries were 13.8, 12.9, 12.5 for group A and 13.9, 13.6, 13.6 for group B, respectively. In the ascending and descending aorta, CNR was significantly higher in group B when compared to group A (13.1 vs. 8.8 and 11.8 vs. 6.9; both p<.001).
**Conclusion:** Bolus tracking in the ascending aorta showed similar contrast enhancement of the pulmonary tree compared to triggering in the pulmonary trunk while improving enhancement in the thoracic aorta, allowing simultaneous evaluation of both regions.
**Limitations:** Single center, rectrospective study.
**Funding for this study:** No dedicated funding
**Ethics committee - additional information:** Rectrospective study with anonymised patient data.
**Author Disclosures:**
Jakob Heimer: Nothing to disclose
Oezlem Krzystek: Nothing to disclose
Tilo Niemann: Nothing to disclose
Gonçalo Garcia De Almeida: Nothing to disclose
Andre Euler: Nothing to disclose

### Contrast-Enhanced Ultrasound for Post-Traumatic Spleen Lesion Assessment: A Radiation-Free Diagnostic Alternative

*N. Finardi*, F. Cicchetti, E. Xhepa, C. Lanza, A. M. Ierardi, G. Carrafiello; Milan/IT

**Purpose or Learning Objective:** Spleen injuries are common in abdominal trauma. While FAST and contrast-enhanced CT are commonly used to detect parenchymal or vascular spleen lesions, contrast-enhanced ultrasound (CEUS) is emerging as a radiation-sparing alternative. CEUS can detect lesions within minutes using microbubble agents, providing a fast and safe method for monitoring damage. This study aims to develop a CEUS protocol for monitoring spleen trauma cases managed non-operatively.
**Methods or Background:** A prospective, single-center observational cohort study was conducted at the Radiology Department of Fondazione IRCCS Ca' Granda Ospedale Maggiore Policlinico in Milan. The study involves 28 patients who presented to the emergency department with abdominal trauma and were diagnosed with parenchymal or vascular spleen injuries managed non-operatively. Initial assessments included contrast-enhanced CT and subsequent follow-up evaluations using contrast-enhanced ultrasound (CEUS) at 48 hours, 12-15 days, 30 days, and 60 days post-trauma to monitor lesion progression and healing.
**Results or Findings:** The primary endpoint was to evaluate the diagnostic accuracy of CEUS in detecting complications following non-operative management of splenic trauma. Among 28 patients, 25 (89.3%) underwent embolization, with CEUS at 48 hours revealing splenic infarcts in 10 (35.7%). No increased free fluid, vascular lesions, or hematoma progression cases were observed in 22 patients (78.6%).
**Conclusion:** CEUS proved to be a reliable, radiation-free tool for early detection of complications in non-operatively managed splenic trauma. Its high diagnostic accuracy, particularly at 48 hours, supports its use as a valuable alternative to CT in follow-up care.
**Limitations:** Small population and short follow-up.
**Funding for this study:** No funding was received for this study.
**Ethics committee - additional information:** Pending approval by the ethics committee.
**Author Disclosures:**
Anna Maria Ierardi: Nothing to disclose
Niccolò Finardi: Nothing to disclose
Francesco Cicchetti: Nothing to disclose
Edon Xhepa: Nothing to disclose
Carolina Lanza: Nothing to disclose
Gianpaolo Carrafiello: Nothing to disclose

### Multiplanar reconstruction of MDCT-images of the midface for CT-exophthalmometry: performance and reproducibility between different post-processing programs

*U. G. Müller-Lisse*, K. Donij, S. G. Priglinger, S. Otto, C. R. Hintschich; Munich/DE
*(ullrich.mueller-lisse@med.uni-muenchen.de)*

**Purpose or Learning Objective:** Measuring ocular protrusion (OP) on MDCT-images is particularly useful in cranio-facial trauma. We step-wisely performed multi-planar reconstruction of MDCT-images (MPR) for CT-exophthalmometry and investigated if results differ between physicians and between different MPR-programs.
**Methods or Background:** One dentist and one radiologist independently reformatted MDCT-images from primary multidetector-row-CT reconstructions and measured inter-frontozygomatic base-length and OP, applying two commercial and one freely available post-processing programs for MPR, respectively, in fifteen consecutive patients with cranio-facial trauma (five female, age 24-88 years), with ethics-committee approval. Ease of patient-and-exam selection, 3D-reconstruction, alignment of orbits, fine adjustment, and measurements were rated 1-10 for each MPR-program. Wilcoxon-matched-pairs-signed-ranks-tests and two-tailed Student-T-tests for paired data compared MPR-steps and distance-measurements, respectively (significance-level, p<0.05).
**Results or Findings:** All MPR-programs allowed physician-generated MPR-reformatting and measurements of base-length and OP, although with different levels of ease (range, 5-10). Results of distance measurements varied by 0.2-0.4 mm, correlated highly (Pearson-r=0.9411-0.9956) and did not differ significantly between different MPR-programs and different observers with few exceptions.
**Conclusion:** CT-exophthalmometry results appear highly reproducible and stable between different MPR-programs when different physicians independently reformatted CT-images and measured base-length and ocular protrusion.
**Limitations:** The study is limited to three exemplary post-processing programs, two independent observers with different training background and a small number of consecutive patients. However, it demonstrates that the principles of observer-performed multiplanr reconstruction of MDCT-images and measurements relating to CT-exophthalmometry transfer between different post-processing programs and Physicians with different subspecialty training.
**Funding for this study:** No funding has been obtained for this study.
**Ethics committee - additional information:** Ethics committee of the Medical Faculty, LMU Ludwig-Maximilians-Universität München, Vote No. 20-633 KB
**Author Disclosures:**
Kathleen Donij: Nothing to disclose
Siegfried Georg Priglinger: Nothing to disclose
Christoph Rudolf Hintschich: Nothing to disclose
Ullrich G. Müller-Lisse: Nothing to disclose
Sven Otto: Nothing to disclose

### Analyzing the Prevalence of Injury and Violence in Transgender Females Using Radiology Reports

*R. Chopra*, K. Patel, B. Rosner, O-P. Hamnvik, B. Khurana; Boston, MA/US
*(rrchopra@bwh.harvard.edu)*

**Purpose or Learning Objective:** Given the high risk of violence with significant underreporting among transgender and gender diverse patients, this study aims to investigate the prevalence and disparities in injuries and potential violence between transgender female and cisgender female patients by analyzing radiology reports.
**Methods or Background:** We utilized our hospital's Research Patient Data Registry to identify 263 transgender female patients and 525 age, race, and ethnicity-matched cisgender women. Adjusted incidence rate ratios (aIRR) and Odds ratios were calculated to compare imaging and injury patterns. Two radiologists blinded to the study's purpose assessed the likelihood of intimate partner violence (IPV) based on radiology reports. EMRs were reviewed for violence documentation in all patients with radiologically evident injuries.
**Results or Findings:** In our cohort, 25.4% (67/263) of cases sustained 141 injuries, compared to 14.7% (77/525) of controls who sustained 98 injuries. Injury rates were higher in cases (aIRR: 3.3 [2.5-4.3] P<0.0001), particularly for cranial (7.8 [2.1-29.1] P<0.0001), facial (36.4 [8.6-153.8] P<0.0001), and thoracic injuries (4.9 [1.4-17] P=0.01), with 78.9% of facial fractures (15/19) involving the midface. The percentage of imaging studies in emergency departments was significantly higher in the cases than in the controls (OR = 5.3 [3.3, 8.3]) (P<0.0001). Radiologists suspected IPV in 12 cases and 1 control, with 75% of cases confirming violence and 50% reporting IPV. A higher number of cases with radiologically evident injuries reported experiencing IPV (OR 6.5; [2.7-15.9]; P<0.0001) compared to controls.

**Conclusion:** Transgender females experience significantly higher injury rates, particularly to the head, face, and chest, with frequent presentations to emergency departments, indicating an elevated risk of violence and gaps in preventive care. By recognizing these patterns, radiologists can help identify at-risk patients and facilitate timely IPV screening and support.
**Limitations:** Retrospective single-institution, self-reporting by patient.
**Funding for this study:** National Institute of Biomedical Imaging and Engineering (NIBIB), National Institute of Health
**Ethics committee - additional information:** Approved by Mass General Brigham IRB
**Author Disclosures:**
Krishna Patel: Nothing to disclose
Bharti Khurana: Nothing to disclose
Bernard Rosner: Nothing to disclose
Rohan Chopra: Nothing to disclose
Ole-Petter Hamnvik: Nothing to disclose

### Abbreviated MRI in traumatic injury of spine

*C. Loberg*, L. Küsters, A. Gisevius, D. Roggenland, E. Yilmaz, T. Schildhauer, C. Kruppa, M. Aach; Bochum/DE
*(Christina.Loberg@bergmannsheil.de)*

**Purpose or Learning Objective:** Traumatic ligamentous injury is common in trauma of spine and can be overlooked easily. Patients outcome is based on fast diagnosis and surgery. MRI is the standard of care in evaluation of ligamentous injury. We invested if an abbreviated protocol (AP) consisting only of one T2wSTIR acquisition is suitable for detection of ligamentous injury.
**Methods or Background:** A cohort of 100 patients with underlying spinal trauma who underwent MRI were selected. Full MRI protocol (FP) comprised T1w, T2w, T2wSTIR sequences at 1.5T. For abbreviated MRI protocol (AP) we chose a rapid protocol that ensures the acquisition of maximum of contrast and maximum spatial-resolution images based on T2wSTIR acquisition. Two radiologist with 5 years and 16 years of experience reviewed the images to characterize ligaments, fracture, spinal instability and traumatic disc herniation.
**Results or Findings:** MRI acquisition for FP was 23 minutes versus 4.12 for the AP. Average time to read the single T2wSTIR and complete FP was 3.4 minutes versus 14.3 minutes. 42 ligamentous injuries were detected. Specificity and positive predictive value (PPV) of AP versus FDP were equivalent (96.5% to 94.2% and 23.9% v 23.2%).
**Conclusion:** An MRI acquisition of 4.20 minutes and expert radiologist reading time of 3.40 minutes are sufficient to confirm ligamentous injury of spine. With a reading time < 4 minutes diagnostic accuracy was equivalent to that of the FDP.
**Limitations:** This was a retrospective single center study.
**Funding for this study:** There was no funding
**Ethics committee - additional information:** Ethic Comittee Ruhr University Hospital Bochum 178/ 24
**Author Disclosures:**
Emre Yilmaz: Nothing to disclose
Astrid Gisevius: Nothing to disclose
Thomas Schildhauer: Nothing to disclose
Mirko Aach: Nothing to disclose
Leonie Küsters: Nothing to disclose
Christina Loberg: Nothing to disclose
Daniela Roggenland: Nothing to disclose
Christiane Kruppa: Nothing to disclose

### Diagnostic accuracy and time efficiency of a novel deep learning algorithm for the assessment of intracranial hemorrhage

*C. Booz*[1], T. Vogl[1], V. Koch[1], L. D. Gruenewald[1], A-I. Nica[1], T. D'Angelo[2], M. Dimitrova[1], G. M. Bucolo[1], I. Yel[1]; [1]Frankfurt/DE, [2]Messina/IT

**Purpose or Learning Objective:** To evaluate diagnostic accuracy and time efficiency of a deep learning-based pipeline using a Dense U-net architecture for the assessment of intracranial hemorrhage (ICH) in unenhanced head CT scans.
**Methods or Background:** This retrospective study included 1004 CT scans of 1004 patients (mean age, 71 ± 11 years; 496 men and 508 women) who had undergone an unenhanced head CT scan for the assessment of ICH. All CT scans were analyzed by the algorithm and a board-certified radiologist independently for the presence of ICH. In case of ICH presence, ICH had to be defined as intraparenchymal hemorrhage (IPH), intraventricular hemorrhage (IVH), subarachnoid hemorrhage (SAH), subdural hemorrhage (SDH) and epidural hemorrhage (EDH). Additionally, the time until first temporary diagnosis of ICH was measured. Three experienced board-certified radiologists analyzed the CT scans in consensus reading sessions to establish the standard of reference for hemorrhage presence and classification.
**Results or Findings:** The reference standard revealed a total of 1108 different ICH presences (IPH, n=344; IVH, n=52; SAH, n=326; SDH, n=356; EDH, n=30). The algorithm showed a high diagnostic accuracy for the assessment of ICH with a sensitivity of 92%, specificity of 95% and an accuracy of 93%. Concerning the most frequently present different ICH types in this study, the sensitivity was 92%, 93% and 93% (IPH, SAH and SDH, respectively), and the specificity was 95%, 96% and 95% (IPH, SAH and SDH, respectively). Regarding analysis time, the algorithm was significantly faster compared to the temporary report of the assigned radiologist (16 ± 3 s vs 273 ± 11 s, $p < 0.001$).
**Conclusion:** A novel deep learning algorithm provides high diagnostic accuracy combined with time efficiency for the identification and classification of ICH in unenhanced CT scans.
**Limitations:** Single-center retrospective study
**Funding for this study:** No funding was received.
**Ethics committee - additional information:** The local IRB approved this study.
**Author Disclosures:**
Christian Booz: Speaker: Siemens Healthineers
Leon D. Gruenewald: Nothing to disclose
Ibrahim Yel: Speaker: Siemens Healthineers
Mirela Dimitrova: Nothing to disclose
Thomas Vogl: Nothing to disclose
Vitali Koch: Nothing to disclose
Andreea-Ioana Nica: Nothing to disclose
Tommaso D'Angelo: Speaker: Bracco Speaker: Philips
Giuseppe Mauro Bucolo: Nothing to disclose

### Beyond ASL: SWI as the Superior Diagnostic Tool for Status Epilepticus When Conventional MRI Falls Short

*S. K. Kondapavuluri*, S. K. Patan, R. P. Yadav; Vijayawada/IN

**Purpose or Learning Objective:** This study evaluates the diagnostic utility of susceptibility-weighted imaging (SWI) in status epilepticus (SE), focusing on its role in identifying cerebral perfusion and oxygenation changes, particularly when conventional MRI sequences (diffusion, T2, FLAIR) appear normal. SWI's superior resolution for detecting subtle venous and metabolic changes makes it a better alternative to arterial spin labelling (ASL) in certain clinical settings. The study aims to demonstrate SWI's value as both a practical and superior tool in perfusion imaging.
**Methods or Background:** This observational study involved 50 patients with confirmed SE who underwent MRI within 6 hours of seizure onset. The imaging protocol included diffusion-weighted imaging (DWI), fluid-attenuated inversion recovery (FLAIR), SWI, and ASL. SWI patterns of venous oxygenation were compared with ASL perfusion maps to assess concordance in detecting hyper- and hypo-perfused regions, particularly in cases where conventional MRI sequences were unremarkable.
**Results or Findings:** SWI revealed distinct patterns of cerebral venous changes, categorised into four groups: Group 1 (20 patients) exhibited generalised hyperperfusion on ASL with globally diminished cortical veins on SWI, indicating global hyperoxygenation. Group 2 (13 patients) showed focal hyperperfusion with focally diminished cortical veins, reflecting focal hyperoxygenation. Group 3 (10 patients) displayed focal hyperperfusion with focally prominent cortical veins due to focal deoxygenation. Group 4 (7 patients) demonstrated generalised hyperperfusion with globally prominent veins, indicating global deoxygenation.
**Conclusion:** SWI is a valuable tool for detecting oxygenation changes in SE, even when conventional MRI sequences appear normal. Its superior resolution and availability make it a better alternative for detecting subtle venous and metabolic changes, supporting its broader adoption as a complementary or standalone tool in epilepsy management.
**Limitations:** Not applicable
**Funding for this study:** No funding was provided for this study.
**Ethics committee - additional information:** Ethics approval for this observational study was obtained from the relevant ethics committee. Informed consent was secured from all participants, ensuring compliance with ethical standards for research involving human subjects. The study adhered to the principles outlined in the Declaration of Helsinki.
**Author Disclosures:**
Sushen Kumar Kondapavuluri: Nothing to disclose
Sharuq Khan Patan: Nothing to disclose
Ratan Pal Yadav: Nothing to disclose

### Prognostic Indicators of Conservative Treatment Failure in Adhesive Small Bowel Obstruction: Insights from CT Imaging

*A. Ammirabile*, E. Desiato, A. M. A. Lucia, S. Giudici, M. Francone, D. Del Fabbro, E. Lanza; Milan/IT
*(angela.ammirabile@humanitas.it)*

**Purpose or Learning Objective:** This study aimed to identify the CT imaging features associated with the failure of conservative management using oral water-soluble contrast medium in patients presenting to the Emergency Room with Adhesive Small Bowel Obstruction (ASBO).

**Methods or Background:** This retrospective single-center study included all consecutive patients admitted to the ER from February 2019 to February 2023 with ASBO, who underwent contrast-enhanced CT at diagnosis and received conservative treatment. The assessed CT findings were type and location of the transition zone, ASBO severity, presence of fat notch sign, beak sign, small bowel feces sign, peritoneal free fluid, and pneumatosis intestinalis. Univariable and multivariable logistic regression analyses were performed to evaluate the association between these radiological parameters and treatment outcomes.
**Results or Findings:** Among the 106 patients included (median age 74.5 years), conservative management succeeded in 59 cases (55.7%), while 47 patients (44.3%) required surgery after initial non-operative treatment failure. Failure was more common in patients with previous ASBO episodes (p = 0.03), female gender (p = 0.04), and was associated with a longer hospital stay (p < 0.001). At multivariable analysis, the fat notch sign (OR = 2.95; p = 0.04) and the beak sign (OR = 3.42; p = 0.04) were significantly associated with conservative treatment failure.
**Conclusion:** Two CT features - the fat notch sign and the beak sign - were significantly correlated with the failure of conservative management in ASBO. These findings highlight the importance of an early identification of patients who may benefit from undelayed surgical intervention.
**Limitations:** The limitations of the study are the retrospective, single-center design and the evaluation of a limited number of CT signs and laboratory values.
**Funding for this study:** No funding was received for this study.
**Ethics committee - additional information:** The study is retrospective.
**Author Disclosures:**
Elena Desiato: Nothing to disclose
Angela Ammirabile: Nothing to disclose
Ada Maria Antonella Lucia: Nothing to disclose
Ezio Lanza: Nothing to disclose
Daniele Del Fabbro: Nothing to disclose
Marco Francone: Nothing to disclose
Simone Giudici: Nothing to disclose

**14:00-15:30** **Research Stage 2**

### Research Presentation Session: Imaging Informatics and Artificial Intelligence

### RPS 1505

### Generative AI in radiology

**Moderator**
C. Blüthgen; Zurich/CH

### Precision, Non-classifications, and Misclassifications of General and Medical Large Language Models in Liver Lesions Classification using LI-RADS from Unstructured Radiology Reports

J. Lu[1], F. F-Y. Tang[1], J. Ng[1], C. Chan[2], H. M. Cheng[1], P. L. H. Yu[1], W. K. W. Seto[1], *W. H. K. Chiu*[1]; [1]Hong Kong/HK, [2]Hampshire/UK
*(Keith.chiu@ha.org.hk)*

**Purpose or Learning Objective:** Large Language Models (LLM) are powerful tools for data extraction and summarization. However, scant evidence exists as to whether a medial-specific LLM is necessary to perform radiology tasks. This study evaluates the performance between a general and a medical LLM in extracting and categorizing liver lesions from radiology reports according to the LI-RADS.
**Methods or Background:** A total of 273 anonymized unstructured Computed Tomography (CT) reports, written by 115 radiologists from 5 institutions containing 599 liver observations were retrospectively collected. These reports were fed into GPT-4 and MedLM to assign LI-RADS categories for each observation using zero-shot prompts (GPT4sp and MedLMsp) and instructions post-prompt engineering (GPT4pe and MedLMpe). Ground truths and quality of the CT reports were derived by 2 board-certified radiologists.
**Results or Findings:** At lesion level, the accuracies for correctly classifying malignant lesions (LR-4/5/M) were 0.584, 0.634, 0.668, and 0.84 for GPT-4sp, MedLMsp, GPT-4pe, and MedLMpe respectively with MedLM outperforming GPT-4 using both simple prompts (p=0.023) and prompt engineering (p<0.001). At patient level, the accuracies were 0.762, 0.744, 0.791, and 0.883, respectively, with prompt engineering outperforming simple prompts in MedLM (p < 0.001). Prompt engineering improved performance by reducing non-classification in both MedLM (11.5% vs 33.7%, p<0.001) and GPT-4 (29.4% vs 38.4% p<0.001). The quality of the CT reports of the 31 misclassified/non-classified patients on MedLMpe were considered average (median LIKERT score 3/5) with a Fleiss' κ value 0.563 (95%CI 0.356 - 0.770).
**Conclusion:** While general LLM exhibits potential in text-based medical tasks, our findings suggest that medical LLM yields superior performance.
**Limitations:** Limitations include a small sample size, lack of prompt engineering exploration, and only one of general and medical LLM used.
**Funding for this study:** None
**Ethics committee - additional information:** The ethics committee notification can be found under Ref: KC/KE-23-0083/ER-3.
**Author Disclosures:**
Philip L. H. Yu: Nothing to disclose
Ho Ming Cheng: Nothing to disclose
Wan Hang Keith Chiu: Nothing to disclose
Chelsea Chan: Nothing to disclose
Justin Ng: Nothing to disclose
Wai Kay Walter Seto: Nothing to disclose
Fanny Fong-Yi Tang: Nothing to disclose
Jianliang Lu: Nothing to disclose

### Evaluating the Performance of LLaMA 3.1 in Classifying Mammography Reports Based on BIRADS Scores

*A. Kumar*, V. K. Venugopal; New Delhi/IN
*(mitugarg75@gmail.com)*

**Purpose or Learning Objective:** This study aimed to evaluate the performance of the LLaMA 3.1 large language model (LLM) in classifying mammography reports based on the Breast Imaging-Reporting and Data System (BIRADS) classification without fine-tuning the model.
**Methods or Background:** A total of 930 mammography reports, covering a range of BIRADS classifications from 0 to 6, were processed using the LLaMA 3.1 open-source LLM (8B version). The model was prompted using a five-shot prompting technique. The classification accuracy of the algorithm was analyzed, and errors in classification were recorded. Among the 930 reports, 8 instances of errors were identified, with 4 cases where BIRADS 2 was incorrectly classified as BIRADS 4 by the model.
**Results or Findings:** The LLaMA 3.1 model demonstrated a classification accuracy of 921 correct classifications out of 930 reports, yielding an overall accuracy rate of 98.99%. Despite the model's strong performance, errors were present, particularly in the misclassification of lower BI-RADS scores, with some benign reports (BIRADS 2) being classified at a higher risk level (BIRADS 4)
**Conclusion:** LLaMA 3.1, even without fine-tuning, shows significant potential for accurately classifying mammography reports based on BIRADS scoring. This indicates that large language models could serve as valuable tools in medical imaging analysis, offering high accuracy with minimal adjustments.
**Limitations:** The study is limited by the occurrence of misclassification in a small number of cases, particularly in distinguishing between benign and higher-risk categories. Further studies with larger datasets and fine-tuning may be needed to improve reliability.
**Funding for this study:** The study didn't receive any funding
**Ethics committee - additional information:** Anonymized data was used
**Author Disclosures:**
Amit Kumar: Nothing to disclose
Vasantha Kumar Venugopal: Nothing to disclose

### Evaluating local open-source large language models for data extraction from unstructured reports on mechanical thrombectomy in patients with ischemic stroke

*A. Meddeb*[1], A. Othman[2], N. F. Grauhan[2], M. Scheel[3], J. Nawabi[3]; [1]Reims/FR, [2]Mainz/DE, [3]Berlin/DE
*(aymenmeddeb@gmail.com)*

**Purpose or Learning Objective:** To assess the effectiveness of open-source Large Language Models (LLMs) in extracting clinical data from unstructured mechanical thrombectomy reports in patients with ischemic stroke caused by a vessel occlusion.
**Methods or Background:** We deployed local open-source LLMs to extract data points from free-text procedural reports in patients who underwent mechanical thrombectomy between September 2020 and June 2023 in our institution. The external dataset was obtained from a second university hospital and comprised consecutive cases treated between September 2023 and March 2024. Ground truth labeling was facilitated by a human-in-the-loop (HITL) approach, with time metrics recorded for both automated and manual data extractions. We tested three models—Mixtral, Qwen, and BioMistral—assessing their performance on precision, recall, and F1 score across 15 clinical categories such as National Institute of Health Stroke Scale (NIHSS) scores, occluded vessels, and medication details.
**Results or Findings:** The study included 1000 consecutive reports from our primary institution and 50 reports from a secondary institution. Mixtral showed the highest precision, achieving 0.99 for first series time extraction and 0.69 for occluded vessel identification within the internal dataset. In the external dataset, precision ranged from 1.00 for NIHSS scores to 0.70 for occluded vessels. The HITL approach yielded an average time savings of 65.6% per case, with variations from 45.95% to 79.56%.
**Conclusion:** LLMs showed high performance in automated clinical data extraction from medical reports. Incorporating HITL annotations enhances precision and also ensures the reliability of the extracted data. This methodology presents a scalable privacy-preserving option that can significantly support clinical documentation and research endeavors.
**Limitations:** Variability in the quality and consistency of the input data, such as differences in terminology, formatting, or detail level in the reports, can affect the performance of the models
**Funding for this study:** None
**Ethics committee - additional information:** This retrospective study was approved by the ethics committee of the Charité University Hospital in Berlin (No. EA4/062/20).The requirement for informed consent was waived due to the retrospective design of the study.
**Author Disclosures:**
Nils F. Grauhan: Nothing to disclose
Aymen Meddeb: Research/Grant Support: Berlin Institute of Health
Jawed Nawabi: Nothing to disclose
Michael Scheel: Nothing to disclose
Ahmed Othman: Nothing to disclose

**Large language models in healthcare: DRAGON performance benchmark for clinical NLP**
*J. S. Bosma*[1], K. Dercksen[1], M. De Rooij[1], F. Ciompi[1], A. Hering[1], J. Geerdink[2], H. E. Huisman[1]; [1]Nijmegen/NL, [2]Almelo/NL
*(Joeran.Bosma@radboudumc.nl)*

**Purpose or Learning Objective:** Artificial Intelligence (AI) requires large-scale annotated datasets to train clinical algorithms to perform at an expert level. Natural Language Processing (NLP) shows great potential to annotate large volumes of data from clinical routine and facilitate the training of these algorithms. This study aims to introduce a benchmark for clinical NLP algorithms, including Large Language Models (LLMs), to assess the ability of algorithms to extract information from medical reports.
**Methods or Background:** The DRAGON (Diagnostic Report Analysis: General Optimization of NLP) challenge has three objectives. First, it provides a unique and publicly available cloud-based benchmark for clinical NLP that spans 28 clinically relevant tasks. 28,824 annotated medical reports from five Dutch care centers from multiple imaging modalities (MRI, CT, X-ray, histopathology) and conditions spanning the entire body (lungs, pancreas, prostate, skin, etc.) are used. The tasks are designed to facilitate automated dataset curation and include predicting diagnoses, extracting lesion sizes, identifying protected health information, and more. Second, we release foundational LLMs pretrained using four million clinical reports from a sixth Dutch care center. Third, we investigate three pretraining strategies across five architectures by evaluating LLMs using the DRAGON benchmark.
**Results or Findings:** Results showed the superiority of domain-specific pretraining (benchmark score of 0.770, 95% CI 0.755-0.785) and mixed-domain pretraining (0.756, 95% CI 0.739-0.773), compared to general-domain pretraining (0.734, 95% CI 0.717-0.752, $p<0.005$). The best model achieved excellent or good performance for 18/28 tasks and poor or moderate performance for 10/28 tasks.
**Conclusion:** The DRAGON benchmark showed that NLP is ready to facilitate data curation in some settings, enabling high-quality, low-cost, and large-scale annotation, and uncovered where innovations are needed to improve clinical NLP.
**Limitations:** Half of the tasks were sourced from a single academic tertiary care center (14/28, 50%).
**Funding for this study:** Funding was provided by Health~Holland (LSHM20103), European Union HORIZON-HLTH-2022: COMFORT (101079894), European Union HORIZON-2020: ProCAncer-I project (952159), European Union HORIZON-2020: PANCAIM project (101016851), and NWO-VIDI grant (number 18388). The collaboration project is co-funded by PPP Allowance awarded by Health~Holland, Top Sector Life Sciences \& Health, to stimulate public-private partnerships. Views and opinions expressed are however those of the author(s) only and do not necessarily reflect those of the European Union or European Health and Digital Executive Agency (HADEA). Neither the European Union nor the granting authority can be held responsible for them.
**Ethics committee - additional information:** Retrospective use of anonymous patient data was approved by institutional or regional review boards at each contributing center (identifiers: CMO 2016-3045; IRBd22-159; A21-0349 2; A20-0777), and was conducted in accordance with the principles of the Declaration of Helsinki. Informed consent was waived.
**Author Disclosures:**
Joeran Sander Bosma: Nothing to disclose
Henkjan En Huisman: Nothing to disclose
Francesco Ciompi: Nothing to disclose
Alessa Hering: Nothing to disclose
Koen Dercksen: Nothing to disclose
Maarten De Rooij: Nothing to disclose
Jeroen Geerdink: Nothing to disclose

**Implementing Local Large Language Models and using Clinical Data Warehouse for Clinical Summarization and Decision Support**
*M. Segeroth*, M. Bach, J. Wasserthal, J. Cyriac, M. Pradella, H-C. Breit, B. Stieltjes, E. M. Merkle, S. Yang; Basel/CH
*(martin.segeroth@gmail.com)*

**Purpose or Learning Objective:** Recent advances in Large Language Models (LLMs) have improved medical text summarization and decision support but raised data privacy concerns. We aim to integrate local LLMs into clinical workflows for testing with real-world patient data.
**Methods or Background:** Within our institutional healthcare network, a clinical data warehouse (CDWH) serves as a central hub for querying all patient records and parameters, while ensuring data privacy. Exemplary parameters like temporal evolution of chemotherapies, dates and outcomes of resections, findings from previous imaging examinations, etc. were extracted for oncology patients. The collected data were fed via a prompt into local LLMs. We utilized privateGPT and Ollama as the primary platform, allowing integration of clinical treatment guidelines. Regarding LLMs we tested Llama3-70B and the German-language SauerkrautLM Mixtral 8X7B Instruct which both ran on a Nvidia A100 GPU with 80 GB memory. A set of anonymized data was processed with cloud-based ChatGPT-4 and Claude-3 for comparison.
**Results or Findings:** Using the privateGPT platform both tested LLMs ran on a single GPU with maximally 65 GB of memory usage. Both LLMs created text summaries within 15 seconds and provided decision support in under 5 seconds per request. For all brain cancer cases the local LLMs provided a correct and reasonable summary of medical history. In decision-making for a prostate tumor board, the decision accuracy amounted to 7 out of 10 test cases. For anonymized data, accuracy between the local LLMs and both ChatGPT-4 and Claude-3 was 8 out of 10 test cases.
**Conclusion:** Integration of local LLMs into clinical workflow or research task is possible. Local LLMs were able to summarize medical history or clinical data for tumor boards, preserving local data privacy policies.
**Limitations:** Only two local LLMs were evaluated on sophisticated datasets.
**Funding for this study:** None
**Ethics committee - additional information:** None
**Author Disclosures:**
Bram Stieltjes: Nothing to disclose
Jacob Wasserthal: Nothing to disclose
Michael Bach: Nothing to disclose
Hanns-Christian Breit: Nothing to disclose
Maurice Pradella: Nothing to disclose
Joshy Cyriac: Nothing to disclose
Martin Segeroth: Nothing to disclose
Elmar M. Merkle: Nothing to disclose
Shan Yang: Nothing to disclose

**Training and Evaluation of Sentence Transformer Model for Retrieval Augmented Generation on Radiology Reports**
*K. Arzideh*, H. Schäfer, A. Idrissi-Yaghir, C. S. Schmidt, J. Haubold, R. Hosch, F. Nensa; Essen/DE
*(kamyar.arzideh@uk-essen.de)*

**Purpose or Learning Objective:** In many medical settings physicians often have to sift through unstructured documents to find important information. This manual process is time-consuming and can lead to missed details. Retrieval Augmented Generation (RAG) can help physicians to quickly locate relevant information. By using Sentence Transformer models fine-tuned for retrieval tasks, similarity search between input query and document passages can be performed to find relevant context. However, most publicly available models are not specifically fine-tuned for the radiology domain and are therefore very limited in finding clinically relevant information.
**Methods or Background:** Document chunks from 400,000 German clinical notes including radiology reports and doctoral notes, were provided as input to the SauerkrautLM-SOLAR-Instruct Large Language Model. The model was prompted to generate clinically related questions and answers based on these chunks. The model generated 11 million clinically related question-answer pairs to fine-tune a multilingual-e5-large model. For evaluation, 1,717 question-answer pairs were generated from 215 radiology reports. A radiologist filtered out unrelated or incorrect pairs for a realistic evaluation. The fine-tuned model was then integrated into a RAG system, and its answers were compared to those from a non-fine-tuned model using the same dataset.

**Results or Findings:** Fine-tuning the model resulted in improved performance metrics. The BLEURT score increased from 0.551 to 0.563, indicating enhanced alignment with human judgment. Similarly, the BERTScore F1 rose from 0.750 to 0.756.
**Conclusion:** By using LLM to generate synthetic questions out of real world documents and fine-tuning sentence transformer models on these question and document pairs, information retrieval performance can improve as indicated by automated evaluation metrics.
**Limitations:** The evaluation was only carried out for documents in German. Fine-tuning on documents written in other languages and from other hospital sites could lead to a broader applicability.
**Funding for this study:** None
**Ethics committee - additional information:** This study was approved by the Ethics Committee of the Medical Faculty of the University of Duisburg-Essen (approval number 23-11557-BO). Due to the study's retrospective nature, the requirement of written informed consent was waived by the Ethics Committee of the Medical Faculty of the University of Duisburg-Essen. All methods were carried out in accordance with relevant guidelines and regulations.
**Author Disclosures:**
Johannes Haubold: Nothing to disclose
Henning Schäfer: Nothing to disclose
Cynthia Sabrina Schmidt: Nothing to disclose
Kamyar Arzideh: Nothing to disclose
René Hosch: Nothing to disclose
Ahmad Idrissi-Yaghir: Nothing to disclose
Felix Nensa: Nothing to disclose

**Large Language Models for Simplified Interventional Radiology Reports: A Comparative Analysis**
*E. Can*[1], W. Uller[1], K. Vogt[1], F. Busch[2], N. Bayerl[3], A. Kader[2], M. R. Makowski[2], K. K. Bressem[2], L. C. Adams[2]; [1]Freiburg/DE, [2]Munich/DE, [3]Erlangen/DE
*(elif.can@uniklinik-freiburg.de)*

**Purpose or Learning Objective:** To quantitatively and qualitatively evaluate and compare the performance of leading large language models (LLMs), including proprietary models (GPT-4, GPT-3.5 Turbo, Claude-3-Opus, and Gemini Ultra) and open-source models (Mistral-7b and Mistral-8x7b), in simplifying 109 interventional radiology reports.
**Methods or Background:** Qualitative performance was assessed using a five-point Likert scale for accuracy, completeness, clarity, clinical relevance, naturalness, error rates, including trust-breaking and post-therapy misconduct errors. Quantitative readability was assessed using Flesch Reading Ease (FRE), Flesch-Kincaid Grade Level (FKGL), SMOG Index, and Dale-Chall Readability Score (DCRS). Paired t-tests and Bonferroni-corrected p-values were used for analysis.
**Results or Findings:** Qualitative evaluation showed no significant differences between GPT-4 and Claude-3-Opus for any metrics (all Bonferroni-corrected p-values: $p=1$), while they outperformed other models across five qualitative metrics ($p < 0.001$). GPT-4 had the fewest content and trust-breaking errors, with Claude-3-Opus second. All models exhibited some trust-breaking and post-therapy misconduct errors, with GPT-4-Turbo and GPT-3.5-Turbo with few-shot prompting showing the lowest error rates, and Mistral-7B and Mistral-8x7B the highest. Quantitatively, GPT-4 surpassed Claude-3-Opus in readability metrics (all $p < 0.001$), with a median FRE score of 69.01 (IQR: 64.88-73.14) versus 59.74 (IQR: 55.47-64.01) for Claude-3-Opus. GPT-4 also outperformed GPT-3.5-Turbo and Gemini Ultra (both $p < 0.001$). Inter-rater reliability was strong ($\kappa$ = 0.77-0.84).
**Conclusion:** GPT-4 and Claude-3-Opus demonstrated superior performance in generating simplified IR reports, but the presence of errors across all models, including trust-breaking errors, highlights the need for further refinement and validation before clinical implementation.
**Limitations:** This study was based on predefined metrics, which, while comprehensive, may not capture all aspects of patient understanding and engagement. Future research should include real-world data, a broader range of medical documents, and consider patient feedback to more accurately assess the clinical utility of these models.
**Funding for this study:** This study did not receive any specific funding from public, commercial, or not-for-profit sectors.
**Ethics committee - additional information:** Since the reports did not include any real patient data, institutional review board approval was not required. This ensures that the study adhered to ethical standards by avoiding the use of real patient information and thereby eliminating the need for formal ethical approval processes typically required for studies involving human subjects.

**Author Disclosures:**
Keno K. Bressem: Nothing to disclose
Elif Can: Nothing to disclose
Marcus R. Makowski: Nothing to disclose
Lisa C. Adams: Nothing to disclose
Katharina Vogt: Nothing to disclose
Nadine Bayerl: Nothing to disclose
Avan Kader: Nothing to disclose
Felix Busch: Nothing to disclose
Wibke Uller: Nothing to disclose

**Automated Radiology Controlling - Using Large Language Models for Prediction of Radiological Services based on Radiological Reports**
*K. Arzideh*, A. Idrissi-Yaghir, H. Schäfer, K. A. Borys, J. Haubold, F. Nensa, R. Hosch; Essen/DE
*(kamyar.arzideh@uk-essen.de)*

**Purpose or Learning Objective:** In hospitals worldwide, controlling of radiological services is a manual process. In Germany, the so-called "Gebührenordnung für Ärzte" (GOÄ) regulates the billing of private medical or dental services, i.e. services outside the public health insurance scheme. GOÄ numbers can be used to indicate which private related clinical interventions were performed during treatment. These numbers are documented by going through radiological reports and picking out relevant information, which is time-consuming and error-prone.
**Methods or Background:** In order to automate billing of radiological services, a Large Language Model (LLM) was fine-tuned to generate GOÄ digits out of radiology reports. In total, 1,000,000 radiology reports and GOÄ digit pairs were split into 80 % training and 20 % test dataset. Training was performed on a Phi-3-small-8k-instruct model. For evaluation, the test dataset was compared against the numbers generated by the model.
**Results or Findings:** The fine-tuned LLM achieved an accuracy of 75 % calculated for the generation of GOÄ numbers. These generated GOÄ codes were identical to the ground truth. 83 % of the predicted codes were present in the ground truth, but may not have been a complete match.
**Conclusion:** LLM are capable of automatically extracting relevant controlling codings based on radiology reports only. Therefore, LLMs could be used as an enhanced method for the automation of controlling tasks in radiology.
**Limitations:** The LLM needs human feedback and manual correction in order to achieve human-like results. The radiology reports used in this study were also written in German language. The use of datasets in other languages and from other hospitals could enable broader generalizability.
**Funding for this study:** None
**Ethics committee - additional information:** This study adhered to all guidelines defined by the approving institutional review board of the investigating hospital. The Institutional Review Board waived written informed consent due to the study's retrospective nature. Complete anonymization of all data was performed before inclusion in the study.
**Author Disclosures:**
Katarzyna Anna Borys: Nothing to disclose
Johannes Haubold: Nothing to disclose
Henning Schäfer: Nothing to disclose
Kamyar Arzideh: Nothing to disclose
René Hosch: Nothing to disclose
Ahmad Idrissi-Yaghir: Nothing to disclose
Felix Nensa: Nothing to disclose

**Insights and Challenges in Implementing Vision Transformers for Thorax Radiography**
*S. Hyska*, A. Wollek, T. Lasser, M. Ingrisch, B. O. T. Sabel; Munich/DE

**Purpose or Learning Objective:** This study aimed to evaluate the performance of a Vision Transformer (ViT)-based AI model, trained on publicly available chest radiography datasets, when applied to real-world data from our clinic. The model's performance in detecting pleural effusion, pneumothorax, cardiomegaly, and consolidation was examined, along with potential confounders.
**Methods or Background:** The AI model, pre-trained on ImageNet and fine-tuned on >700,000 public chest X-rays (CXR), was tested on an internal dataset of 113 CXR, including 23 pneumothorax, 29 cardiomegaly, 31 consolidation, 52 pleural effusion cases, and 29 normal CXR. The model's performance was assessed through ROC-curves, AUC, Youden Coefficient, and sensitivity/specificity metrics. Logistic regression, odds ratios, and Fisher's test were used to analyse confounding factors.

**Results or Findings:** The model correctly identified all normal CXRs. For pleural effusion, sensitivity was 96.2% and specificity 98.4%, indicating strong performance. For pneumothorax, sensitivity was only 26.1% with 96.7% specificity. Pneumothorax size and presence of thoracic tubes were significant confounders. Cardiomegaly was detected with 55.2% sensitivity and 96.4% specificity, whereas concomitant pleural effusions, obscuring the heart contours, act as a potential confounder. Consolidation was detected with 45.2% sensitivity and 91.5% specificity, and higher density consolidations were more easily identified.
**Conclusion:** This study emphasizes the challenges AI models face when integrated into clinical practice, demonstrating the importance of carefully and clinically assessing model performance on real-world-data, especially in the context of confounding factors. While our ViT model showed strong performance for pleural effusion and normal findings, its detection of pneumothorax, cardiomegaly, and consolidation was limited. Known confounders, e. g. pneumothorax size and presence of thoracic tubes, were confirmed, and new ones, such as pleural effusion in cardiomegaly and density of consolidations, were identified.
**Limitations:** The exploratory nature and limited number of CXR were key limitations.
**Funding for this study:** This work was funded in part by the German federal ministry of health's program for digital innovations for the improvement of patient- centered care in healthcare [grant agreement no. 2520DAT920].
**Ethics committee - additional information:** Approval by an ethics committee is present.
**Author Disclosures:**
Bastian Oliver Theodor Sabel: Nothing to disclose
Sardi Hyska: Nothing to disclose
Alessandro Wollek: Nothing to disclose
Michael Ingrisch: Nothing to disclose
Tobias Lasser: Nothing to disclose

**14:00-15:30** **Research Stage 3**

### Research Presentation Session: Paediatric

### RPS 1512

### Body imaging in children: from head to toe

**Moderator**
L. B. Laborie; Bergen/NO
*(lene.bjerke.laborie@helse-bergen.no)*
**Author Disclosures:**
Lene Bjerke Laborie: Other: Involved in development of AI-algorithms for paediatric hip radiographs with software company Visiana (DK). No financial benefits.

### Automatic Identification and Classification of Pediatric Glomerulonephritis on Ultrasound Images Based on Deep Learning and Radiomics

*J. Kou*, Y. Tang; Chongqing/CN
*(koujun@my.swjtu.edu.cn)*

**Purpose or Learning Objective:** Glomerulonephritis (GN) includes a diverse range of kidney diseases that often exhibit subclinical manifestations in children. While renal biopsy is the gold standard, its invasiveness, susceptibility to sampling errors, and time requirements impede rapid diagnosis. This study aimed to create a noninvasive diagnostic model for childhood GN by integrating deep learning and radiomics techniques using renal ultrasound images.
**Methods or Background:** A total of 469 renal ultrasound images were selected from children undergoing ultrasound-guided biopsy and split into training and validation sets at an 8:2 ratio to train a U-Net model for kidney segmentation. Radiomic features were extracted from the segmented regions and categorized by GN types: IgA nephropathy (127 cases), minimal change disease (83 cases), and Henoch-Schönlein purpura nephritis (103 cases). These categories were also split into training and validation sets at an 8:2 ratio. ANOVA was used for feature selection in the training set, followed by LASSO regression for dimensionality reduction, yielding 37 features. A random forest algorithm was then used to develop a GN classification model, which was evaluated using the validation set.
**Results or Findings:** The segmentation model demonstrated excellent performance, achieving 95.19% accuracy on the validation set. Thirty-seven selected features were used to build a strong classification model, which showed high accuracy and predictive power across GN categories, with AUC values between 0.91 and 0.98.
**Conclusion:** The combination of deep learning and radiomics using renal ultrasound images shows great potential for classifying childhood GN subtypes, offering a noninvasive method to enhance diagnostic efficiency and patient outcomes.
**Limitations:** Firstly, the relatively limited data sources may have introduced some regional bias to our findings. Furthermore, we focused only on the pathological subtypes of three GN, which inevitably limited the scope of the model.
**Funding for this study:** 0
**Ethics committee - additional information:** the Ethics Committee of Children's Hospital at Chongqing Medical University.
**Author Disclosures:**
Jun Kou: Nothing to disclose
Yi Tang: Nothing to disclose

### Biomarkers of Primary Sclerosing Cholangitis detected with delayed gadolinium-enhanced Magnetic Resonance Imaging in pediatric patients

*F. Maccioni*, V. Cardinale, E. Damato, L. Busato, S. Veraldi, A. Valenti, C. Catalano; Rome/IT
*(francesca.maccioni@uniroma1.it)*

**Purpose or Learning Objective:** Primary sclerosing cholangitis (PSC) is a severe liver disease frequently associated with inflammatory bowel disease (IBD) with a late diagnosis, mostly based on biliary changes at MRCP. To identify specific biomarkers for PSC using delayed phases of gadolinium enhancement to detect ductal fibrosis, such as intestinal fibrosis in Crohn's disease.
**Methods or Background:** A prospective study based on the association of MRCP and gadolinium-enhanced MRI, including delayed (7 minutes) phases, was performed in 3 groups of pediatric patients, one with PSC and IBD, one with IBD only, and one of controls. Three radiologists blindly and independently analyzed: a) intra and extrahepatic bile ducts strictures (IHBDs, EHBDs) at MRCP; b) gallbladder volume; c) gallbladder wall gadolinium-enhancement; d) IHBDs-EHBDs gadolinium-enhancement.
**Results or Findings:** We included 39 patients, 12 with PSC and IBD (31%) , 16 with IBD only (41%) and 11 controls (28%). At MRCP, IHBDs strictures were detected in 82% PSC-IBD patients ($p<0.001$). Delayed enhancement of gallbladder wall was observed in 100% of PSC-IBD patients (100% sensitivity, 90% specificity, ($p<0.001$); delayed enhancement of the extrahepatic biliary ducts in 89% (89% sensitivity, 100% specificity, ($p<0.001$); and delayed enhancement of the intrahepatic biliary duct in 55,6% (56% sensitivity, 100% specificity).
**Conclusion:** Delayed gadolinium-enhancement of the gallbladder wall and extrahepatic bile duct showed remarkable sensitivity and specificity for PSC. These biomarkers may potentially increase MRI diagnostic accuracy in high-risk IBD patients.
**Limitations:** The main limitation is the small number of patients.
**Funding for this study:** No funding
**Ethics committee - additional information:** The study was approved by the ethics commitee of our hospital.
**Author Disclosures:**
Vincenzo Cardinale: Nothing to disclose
Alessandra Valenti: Nothing to disclose
Silvio Veraldi: Nothing to disclose
Elio Damato: Nothing to disclose
Carlo Catalano: Nothing to disclose
Ludovica Busato: Nothing to disclose
Francesca Maccioni: Nothing to disclose

### A nomogram model based on Combi-Elastography for preoperative differential diagnosis of biliary atresia

*J. Chen*, F. Xu, Y. Gao, M. Yu, Y. Tang; Chongqing/CN
*(cjy419103@163.com)*

**Purpose or Learning Objective:** This study constructs a nomogram prediction model based on combi-elastography indexes and laboratory indicators for the differential diagnosis of biliary atresia (BA) and other cholestatic liver diseases (non-BA), with a view to recognizing BA at an early stage and carrying out treatment in time.
**Methods or Background:** A total of 111 children aged < 180 d with cholestatic hepatitis are included in the study, 75 in the BA group and 36 in the non-BA group. Conventional ultrasound, combi-elastography, and laboratory tests are performed on each patient before pathologic biopsy. The variables are selected through logistics regression to construct a nomogram model, and the effectiveness of the model is evaluated.
**Results or Findings:** Multifactorial logistic regression analysis shows that gamma-glutamyl transferase (GGT) , total bilirubin (TBIL) , and liver fibrosis-related F index (FI) of the combi-elastography index could be used as independent predictors to differentiate BA from other causes of cholestasis. A nomogram model of these three indexes is constructed which shows better performance, with an area under the operating characteristic curve (AUC) of 0.887 (p < 0.001), sensitivity of 83.3%, and specificity of 81.3%. The internal validation of the model is performed using 1,000 bootstrap resamples and Decision curve analysis indicates that this model had a better diagnostic efficacy and accuracy.
**Conclusion:** The nomogram model based on combi-elastography indexes and laboratory indicators has certain value in differentiating BA from other cholestatic liver diseases.
**Limitations:** First, our sample size was small and it was a single-center study.Futher, we can expand the sample size to verify the validity of the nomogram model and provide more valuable insights for clinicians. Second, additional indicators can be included in the future, such as MMP-7 to improve the diagnostic efficacy of the nomogram model.
**Funding for this study:** Chongging Municipal Science and Health Joint Medical Research Project(2024MSXM050)
**Ethics committee - additional information:** This study was approved by the Institutional Review Board of the Children's Hospital of Chongqing Medical University (ethical approval number 2024-216), and all examinations and surveys were conducted after obtaining consent from the parents.
**Author Disclosures:**
Fenglin Xu: Nothing to disclose
Yi Tang: Nothing to disclose
Jingyu Chen: Nothing to disclose
Yang Gao: Nothing to disclose
Mingzhu Yu: Nothing to disclose

**Ultrasound spleen stiffness as a marker of portal vein anastomotic stenosis following paediatric liver transplantation: correlation with transhepatic portal venography**
*D. C. Missud*, S. Le Cam, I. Mannes, M. Duché, S. Franchi-Abella;
Le Kremlin-Bicêtre/FR
*(david.missud@aphp.fr)*

**Purpose or Learning Objective:** Spleen stiffness measurement (SSM) is known as a biomarker of significant portal hypertension in chronic liver disease, but has been poorly studied in the context of liver transplantation (LT). SSM may be particularly interesting to evaluate portal vein anastomotic stenosis, a common and serious complication of LT.
**Methods or Background:** Retrospective study including all portal vein stenoses assessed with transhepatic portal venography (TPV) among paediatric liver recipients between 2015 and 2024. Diagnostic performance of SSM for the diagnosis of portal vein stenosis prior to TPV was evaluated.
**Results or Findings:** 36 children who underwent 58 TPV (stenosis group) were included, with a median age at TPV of 3.0 years. Findings were compared to those of 58 randomly selected paediatric liver recipients without portal vein complication (control group). In the stenosis group, there were 17 mild, 20 moderate, and 21 severe stenoses. SSM was significantly increased in the moderate and severe groups vs no or mild stenosis groups (p < 0.005). The Area Under the Curve was 0.96 for significant vs no to mild stenosis. A SSM cut-off of 33.7 kPa led to a sensitivity of 0.976 and a specificity of 0.840 for the diagnosis of significant stenosis.
**Conclusion:** SSM correlates very well with transhepatic portal venography when there is a suspicion of significant portal vein anastomotic stenosis following LT in paediatric patients. Further studies may demonstrate that SSM is a good biomarker of portal hypertension following LT in paediatric patients, regardless of the aetiology.
**Limitations:** This is a retrospective study which doesn't have the strength of a prospective clilnical trial. This is a series of only 58 TPVs, but it is nonetheless one the largest series ever published regarding paediatric liver recipients with portal vein complications.
**Funding for this study:** No external funding
**Ethics committee - additional information:** This is a usual care clinical retrospective study, under review by the local ethics committee.
**Author Disclosures:**
Inès Mannes: Nothing to disclose
David Charles Missud: Nothing to disclose
Mathieu Duché: Nothing to disclose
Solène Le Cam: Nothing to disclose
Stéphanie Franchi-Abella: Nothing to disclose

**Evaluation of the Validity of Image-Defined Risk Factors (IDRFs) in Abdominal Neuroblastoma**
*Z. Can Beyoğlu*, N. G. Akyel, E. Arslantaş, T. Banaz, M. Söyleyici, E. Ayaz, S. Akpınar Tekgündüz; Istanbul/TR
*(zekicanbeyoglu@gmail.com)*

**Purpose or Learning Objective:** The presence of image-defined risk factors (IDRFs) in neuroblastoma plays a crucial role in clinical decision-making, particularly when choosing between primary tumor resection and neoadjuvant chemotherapy. This study aims to evaluate how the presence of IDRFs influences the surgical outcomes of patients with abdominal neuroblastoma, specifically focusing on complete tumor resection and the likelihood of recurrence within one year.
**Methods or Background:** This retrospective study involved 60 patients diagnosed with abdominal neuroblastoma. Of these, 30 patients had tumors with IDRFs present, while the remaining 30 patients had no IDRFs identified. The recurrence rates and surgical outcomes were analyzed over a one-year follow-up period. Factors such as tumor residuals and overall recurrence rates were examined to assess the impact of IDRFs on surgical success and prognosis.
**Results or Findings:** A total of 60 patients were included in the analysis. Among the 30 patients with IDRFs, 9 patients showed no signs of recurrence within one year, while 12 patients experienced tumor recurrence, and 9 had residual tumor tissue post-surgery. In the group of 30 patients without IDRFs, 22 patients did not experience recurrence, 5 patients had recurrences, and 3 had residual tumor tissue after surgery. These findings suggest that the presence of IDRFs significantly impacts the likelihood of recurrence and surgical success.
**Conclusion:** The study found that the presence of IDRFs in abdominal neuroblastoma patients is an important predictor of recurrence. Patients with IDRFs had a lower non-recurrence rate (30%) compared to those without IDRFs (73%). Therefore, IDRF presence can be a key factor in determining surgical approach and prognosis.
**Limitations:** The study is limited by the small sample size and variability in imaging techniques used during follow-up, which could affect the consistency of the results.
**Funding for this study:** No funding was received for this study.
**Ethics committee - additional information:** This study was approved by the Ethics Committee of Başakşehir Çam and Sakura City Hospital, Istanbul/Turkey
**Author Disclosures:**
Merve Söyleyici: Nothing to disclose
Sibel Akpınar Tekgündüz: Nothing to disclose
Esra Arslantaş: Nothing to disclose
Nazli Gülsüm Akyel: Nothing to disclose
Tuba Banaz: Nothing to disclose
Zeki Can Beyoğlu: Nothing to disclose
Ercan Ayaz: Nothing to disclose

**Validation of a New Scoring System for Residual Tumor Assessment After Surgery in Pediatric Neuroblastoma: Preliminary Results**
*J. F. Schäfer*[1], J. Spogis[1], J. Fuchs[1], B. Hero[2], T. Simon[2], A. Eggert[3], M. Müller[4], S. Warmann[3], B. Timmermann[5]; [1]Tübingen/DE, [2]Cologne/DE, [3]Berlin/DE, [4]Heidelberg/DE, [5]Essen/DE
*(juergen.schaefer@med.uni-tuebingen.de)*

**Purpose or Learning Objective:** The SIOPEN HR-NBL2 protocol for the treatment of high-risk neuroblastoma (NB) is a multinational trial with radiation randomization for patients with macroscopic residual tumors after induction chemotherapy and tumor resection. However, precise definitions of residual tumor on cross-sectional imaging are not available yet. This study aims to validate a newly developed scoring system for assessing residual tumors proposed by the German Neuroblastoma registry.
**Methods or Background:** Patients treated according to the GPOH NB guidelines and irradiated at the West German Proton Therapy Center Essen were retrospectively included if pre-/post-operative and current MRI were available at the time of radiotherapy. The score is based on MRI (tumor size and diffusion restriction), mIBG uptake, and the surgical report, assigning a lesion score from 1 to 3 for each point in time. Anonymized imaging data were uploaded to a browser-based imaging platform (mRay, Germany) for multi-reader, multi-institutional evaluation.
**Results or Findings:** A total of 15 patients (mean age 5.1y; SD 2.1y) with 45 MRIs were assessed by two experienced readers (R1/R2). The mean preoperative tumor volume was 100 ml (range 3–344 ml). Image-defined risk factors were identified in all patients except one. Based on the surgical reports, complete macroscopic resection was achieved in 9 patients, while imaging analysis by R1 and R2 confirmed complete resection in 4 cases. On a lesion-based analysis, surgery identified 6 residual lesions in 6 patients, while R1 and R2 identified 25 lesions in 11 patients (mean size: 13 mm, range: 4–32 mm). The inter-reader agreement for MRI scoring was excellent (ICC 0.87; 95% CI: 072–0.94).

**Conclusion:** Regarding MRI findings, the newly developed scoring system for residual tumors in pediatric neuroblastoma is feasible. Further validation through multi-institutional, platform-based readings is planned.
**Limitations:** Preliminary data
**Funding for this study:** No Funding.
**Ethics committee - additional information:** University of Essen, Germany
**Author Disclosures:**
Angelika Eggert: Nothing to disclose
Beate Timmermann: Nothing to disclose
Thortsen Simon: Nothing to disclose
Jörg Fuchs: Nothing to disclose
Jürgen F Schäfer: Nothing to disclose
Barbara Hero: Nothing to disclose
Steven Warmann: Nothing to disclose
Michael Müller: CEO: providing the research platform
Jakob Spogis: Nothing to disclose

**Imaging predictors of rupture in pediatric solid tumors**
*G. G. Koodaly*, V. Smriti, A. D. Baheti, S. Kulkarni, N. Shetty, K. B. Gala, M. Ramadwar, S. Quereshi, G. Chinnaswamy; Navi Mumbai/IN
*(ggkoodaly@gmail.com)*

**Purpose or Learning Objective:** Tumor rupture poses as a life-threatening complication in pediatric solid tumors, and requires a high-risk protocol management. This study aims to identify the potential imaging predictors for tumor rupture.
**Methods or Background:** The clinical data of children with pediatric solid tumor rupture at our institution from January 2021 to June 2024 were reviewed retrospectively.
**Results or Findings:** Total of 22 cases, which comprised of 14 hepatoblastoma, 6 Wilms' tumor, 1 neuroblastoma, and 1 Ewing sarcoma were analyzed. Patients were aged between 2 to 10 years (median 4.5 years). 17 patients were treatment naïve and 5 on chemotherapy. Patients commonly presented with abdominal pain, distension, nausea, vomiting, and signs of shock due to significant drop in hemoglobin. Imaging via ultrasound and CT scans revealed tumors, with a notable correlation between tumor rupture and factors such as high PRETEXT scores, tumor sizes over 10 cm, a greater percentage of necrotic component, intratumoral bleed and hyperdense ascitic fluid (>25 Hounsfield units). Among the hepatoblastoma cases, 71% had PRETEXT III and above. At diagnosis, 64% of the patients had hemoglobin levels ≤ 8 g/L, with 18% at ≤ 6 g/L. Seven patients required angioembolization, 2 underwent surgery and rest were treated conservatively. Unfortunately, one patient developed tumor lysis syndrome and 3 succumbed to tumor rupture.
**Conclusion:** This study identifies the imaging predictors of tumor rupture in pediatric solid tumors and associated risk factors, such as chemotherapy, larger size of tumor and other high-risk factors.
**Limitations:** Retrospective study. Its prevalence in cohort is not known.
**Funding for this study:** No funding was received for this study.
**Ethics committee - additional information:** Retrospective study
**Author Disclosures:**
Genesis Giddo Koodaly: Nothing to disclose
Suyash Kulkarni: Nothing to disclose
Sajid Quereshi: Nothing to disclose
Mukta Ramadwar: Nothing to disclose
Nitin Shetty: Nothing to disclose
Kunal Bharat Gala: Nothing to disclose
Akshay Dwarka Baheti: Nothing to disclose
Vasundhara Smriti: Nothing to disclose
Girish Chinnaswamy: Nothing to disclose

**The Risk of Pediatric and Adolescent Hematologic Malignancies Associated with Medical Imaging (RIC)**
*R. Smith-Bindman*[1], S. Albers[2], M. Kwan[3], W. Bolch[4], E. Bowles[5], C. Stewart[1], R. Greenlee[6], J. Pole[7], D. L. Miglioretti[2]; [1]San Francisco, CA/US, [2]Davis, CA/US, [3]Oakland, CA/US, [4]Gainesville, FL/US, [5]Seattle, WA/US, [6]Marshfield, WI/US, [7]Brisbane/AU
*(rebecca.smith-bindman@ucsf.edu)*

**Purpose or Learning Objective:** Risks of hematologic malignancies associated with medical imaging ionizing radiation exposure have not been evaluated in the U.S. or Canada.
**Methods or Background:** This retrospective cohort study followed 3,724,622 children born at one of 6 U.S. healthcare systems or in Ontario, Canada from 1/1/1996 to 4/30/2016 from birth until the earliest of a cancer diagnosis, death, emigration from Ontario, 6 months after disenrollment from healthcare system, age 21, or 12/31/2017. Active bone marrow radiation doses from medical imaging examinations were estimated. Hazards ratios (HR) and relative risks (RR) of hematologic malignancies associated with cumulative radiation exposure were estimated.
**Results or Findings:** A total of 2,961 hematologic malignancies were diagnosed during 35,735,719 person-years of follow up, including lymphoid malignancies (n=2,349, 79.3% of malignancies), myeloid or acute leukemia (myeloid, n=460, 15.5%); and histiocytic and dendritic cell malignancies (H&D, n=129, 5.1%). Malignancy risk increased with cumulative dose (p<0.0001); e.g., risk was 1.70 times higher among children with a cumulative dose of 15 to < 20 mGy vs. <1mGy (95%CI=1.27-2.28). The risk of malignancy was 3.7 times higher (95%CI=2.82-4.71) for children with a cumulative exposure of 100 mGy vs. no exposure and was significantly elevated for cancer subtypes. RRs decreased with increasing time since exposure and increase with age at exposure and attained age. We estimate 27 excess hematologic malignancies by age 21 per 10,000 children with a cumulative exposure of 30 mGy or higher vs. <1 mGy, equivalent to average dose of approximately 2 head CTs.
**Conclusion:** Children and adolescents who undergo radiation-based medical imaging are at a small, but significant increased risk of hematologic malignancy.
**Limitations:** While reverse causation is a potential limitation, analyses of clinical indications confirmed symptoms related to hematologic malignancy were rare in included studies.
**Funding for this study:** US National Institutes of Health, National Cancer Institute R01CA185687, R50CA211115
**Ethics committee - additional information:** The requirement for individual informed consent was waived for the study
**Author Disclosures:**
Erin Bowles: Nothing to disclose
Marilyn Kwan: Nothing to disclose
Jason Pole: Nothing to disclose
Rebecca Smith-Bindman: Nothing to disclose
Carly Stewart: Nothing to disclose
Diana L Miglioretti: Nothing to disclose
Wesley Bolch: Nothing to disclose
Susan Albers: Nothing to disclose
Robert Greenlee: Nothing to disclose

**Ultra-low dose CT for non-accidental injury**
A. A. Mohammed, M. F. Mcentee, A. England, N. Moore, E. K. Mahon, M. Maher, *R. Young*; Cork/IE

**Purpose or Learning Objective:** The aim of this study is to compare two whole-body CT protocols for SPA and to assess whether the ULD protocol can provide diagnostic images of sufficient quality compared to the standard dose (STD).
**Methods or Background:** In this cross-sectional study, two sets of images of a newborn whole-body anthropomorphic phantom were acquired using different protocols, one with STD and the other with ULD protocol. The effective dose (ED) of both protocols was calculated using the Monte Carlo dose simulation approach. The image quality arising from both protocols was then assessed at the ECR 2024 Congress using a four-section questionnaire. The questionnaire included demographic information, a comparison of the visualization of different bony anatomical structures, and confidence in diagnosis using either protocol. The Wilcoxon signed-rank test was used to evaluate the significant differences between STD and ULD image quality scores. VGC analyser was used for image quality rating and comparison.
**Results or Findings:** 46 participants were included in this study. For all body parts, STD showed significantly higher image quality than ULD (AUCVGC=0.75). 76% of the participants were confident to use the STD protocol for SPA diagnosis, whereas, 41% were confident to use the ULD protocol. The percentage effective dose difference between protocols was 93.5%(STD=0.56 mSv vs. ULD=0.04mSv) and most of the participants underestimated the dose reduction.
**Conclusion:** This study successfully compared the STD and ULD whole-body CT in phantom and shows ULD CT is a promising technique which may compete with digital radiography for SPA diagnosis.
**Limitations:** The limitations of the study are: 1-the use of phantom involves no patient movement experienced, and no pathologies. 2-sampling bias was due to the observers as only people attending the ECR 2024 could participate. 3-only compares two whole-body CT protocols.
**Funding for this study:** Taif University
**Ethics committee - additional information:** The ethical approval provided by the University College Cork
**Author Disclosures:**
Mark F. Mcentee: Nothing to disclose
Niamh Moore: Nothing to disclose
Rena Young: Nothing to disclose
Ahmed Abdulahad Mohammed: Nothing to disclose
Andrew England: Nothing to disclose
Eimear Kate Mahon: Nothing to disclose
Michael Maher: Nothing to disclose

**Deep Learning-based Detection of Pediatric Bone Tumors Using X-ray Imaging**

S. Consalvo, A. Curto Vilalta, *A. W. Marka*, S. Breden, B. Schlossmacher, C. Eisfeld, D. Rückert, R. Von Eisenhart-Rothe, F. Hinterwimmer; Munich/DE
*(alexander.marka@gmail.com)*

**Purpose or Learning Objective:** To address the challenge in musculoskeletal radiology of early detection of bone tumours in children with x-ray imaging. While machine learning (ML) has shown proficiency in differentiating tumour entities, a critical gap remains in initial tumour detection, particularly for non-oncology-trained professionals and general practitioners. Paediatric tumours are often incidentally discovered, underscoring the need for more sophisticated tools.
**Methods or Background:** This retrospective study utilized X-ray data from a diverse cohort of paediatric patients from our local musculoskeletal tumour database. The dataset comprised 817 images (567 pathological and 250 healthy) from 511 patients, including ten benign, intermediate and malignant tumour entities. We employed the ResNet18 architecture for classification, supported by cross-validation techniques and excessive data augmentation strategies. Our methodology focused on enhancing the ML system's ability to generalize across various clinical scenarios.
**Results or Findings:** The ML model demonstrated high performance with an accuracy of 96.39%, a sensitivity of 96.0%, and a specificity of 96.0% into “tumour” and “no tumour”. The variance in cross-validation splits was 0.05, 0.10, and 0.13, respectively, indicating stable results across different test sets. These metrics reflect the model's reliability and potential effectiveness in clinical settings.
**Conclusion:** Current ML applications in orthopaedic oncology are progressing yet remain insufficiently performant for widespread clinical use. However, our findings underscore the potential of ML tools in aiding both young professionals and general practitioners. Additionally, future advancements should focus on multimodal approaches that incorporate not only X-ray data but also MRI and, crucially, clinical data. Integrating these diverse data sources will enhance the performance and applicability of ML in diagnosing and managing paediatric bone tumours, offering a more holistic and effective approach to patient care.
**Limitations:** 90% Monocentric Data.
**Funding for this study:** Nemetschek Innovation Foundation and Bavarian Ministry of Science and the Arts
**Ethics committee - additional information:** Klinikum rechts der Isar, Technical University of Munich.
**Author Disclosures:**
Carolin Eisfeld: Nothing to disclose
Anna Curto Vilalta: Nothing to disclose
Sebastian Breden: Nothing to disclose
Alexander Wolfgang Marka: Nothing to disclose
Florian Hinterwimmer: Nothing to disclose
Sarah Consalvo: Nothing to disclose
Daniel Rückert: Nothing to disclose
Rüdiger Von Eisenhart-Rothe: Nothing to disclose
Benjamin Schlossmacher: Nothing to disclose

**14:00-15:30 Research Stage 4**

### Research Presentation Session: Neuro

### RPS 1511

### Insights into brain tumours: from visible to invisible and back again

**Moderator**
C. Eraslan; Izmir/TR
*(eraslancenk@gmail.com)*

**Improved Brain Tumor visualization with 3T Stack-of-Stars Echo Unbalanced T1 Relaxation-Enhanced Steady-State MRI – A Two Center Clinical Study**

*A. Toth*[1], R. Edelman[2], J. A. Chetta[1], J. Joyce[1], M. V. Spampinato[1], R. Zi[3], K. T. Block[3], A. Varga-Szemes[1]; [1]Charleston, SC/US, [2]Evanston, IL/US, [3]New York, NY/US
*(adrienntoth706@gmail.com)*

**Purpose or Learning Objective:** The novel stack-of-stars echo unbalanced T1 relaxation-enhanced steady-state (SOS echo-uT1RESS) sequence aims to provide improved motion robustness and enhanced dark blood contrast, and to improve the visualization of small metastases and low enhancing lesions. This study compared the image quality and diagnostic utility of SOS echo-uT1RESS with the widely used magnetization-prepared rapid acquisition gradient-echo (MPRAGE) sequence in brain tumor imaging.
**Methods or Background:** This two-center prospective study involved 25 adults with known brain tumors (n= 5 intra-axial primary brain tumors; n= 11 intra-axial brain metastases; n= 9 extra-axial brain tumors). Each participant underwent 3T contrast enhanced MRI of the brain with both standard MPRAGE and prototype SOS echo-uT1RESS sequences. Contrast-to-noise ratio (CNR) and tumor-to-brain contrast were quantitatively analyzed. Image quality, lesion conspicuity, and image artifacts were scored on a 4-point Likert scale. Diagnostic performance and assessment of the vascular and dural involvement were compared side-by-side by 2 readers.
**Results or Findings:** There was no significant difference in CNR between MPRAGE and SOS echo-uT1RESS (27.0 ± 19.2 vs. 26.5 ± 14.9, respectively; $p = 0.84$). SOS echo-uT1RESS demonstrated a 1.6-fold improvement in tumor-to-brain contrast compared with MPRAGE (0.7 ± 0.4 vs. 0.4 ± 0.3, respectively; $p < 0.001$). Image quality and artifacts were similar for both sequences, while SOS echo-uT1RESS showed improved lesion conspicuity, diagnostic performance and enhanced detection of vascular and dural invasion.
**Conclusion:** SOS echo-uT1RESS showed promising results for post-contrast evaluation of brain tumors on 3T MRI. This technique enhanced lesions visibility, achieving approximately 1.6-fold improvement in tumor-to-brain contrast compared to MPRAGE. It offered superior diagnostic performance and improved detection of vascular and dural involvement.
**Limitations:** The limitations of the study are the relatively small patient cohort and that quantitative measurements were performed by a single observer.
**Funding for this study:** Funding was provided by NIH HHS United States (1R01CA263091 and 1R21CA273280).
**Ethics committee - additional information:** The study was approved by the Institutional Review Board (Pro00128013)).
**Author Disclosures:**
Kai Tobias Block: Nothing to disclose
Robert Edelman: Nothing to disclose
Ruoxun Zi: Nothing to disclose
Adrienn Toth: Nothing to disclose
Jennifer Joyce: Nothing to disclose
Justin A Chetta: Nothing to disclose
Akos Varga-Szemes: Nothing to disclose
M. Vittoria Spampinato: Nothing to disclose

**Multiparametric MRI-based radiomics with interpretable machine learning for predicting progesterone receptor expression in meningioma: A multicenter study**

*G. Lin*, W. Chen, J. Ji; Lishui/CN

**Purpose or Learning Objective:** This study aimed to develop and validate an interpretable machine learning-based prediction model for preoperatively predicting progesterone receptor (PR) expression in meningioma patients using multiparametric magnetic resonance imaging (MRI).
**Methods or Background:** The study retrospectively enrolled 739 patients with pathologically confirmed meningioma from three medical centers, dividing them into four cohorts: training (n = 294), internal test (n = 126), external test 1 (n = 217), and external test 2 (n = 102). Radiomics characteristics were derived from T2-weighted and contrast-enhanced T1-weighted MRI images, followed by feature selection. A machine learning-based combined model was developed by incorporating radiomics scores (rad-scores) from the optimal radiomics model along with clinical predictors. The Shapley additive explanation (SHAP) method was employed to visually represent the process of making predictions. The prognostic value of the model was evaluated using Kaplan-Meier survival analysis.
**Results or Findings:** Among the 739 patients, 299 (40.5%) had negative PR expression confirmed by pathology. Twelve radiomics features derived from multiparametric MRI were selected to build the radiomics model. Tumor location and enhancement pattern were identified as key clinical predictors and were combined with rad-scores to create a combined model utilizing the extreme gradient boosting (XGBoost) algorithm. The combined model demonstrated strong accuracy and robustness, with area under the curve values of 0.907, 0.827, 0.846, and 0.807 across training, internal test, external test 1, and external test 2 cohorts, respectively. The survival analysis indicated that the combined model was able to effectively categorize patients based on recurrence outcomes.
**Conclusion:** The XGBoost combined model, utilizing multiparametric MRI, shows promise for predicting PR expression in meningioma patients. The SHAP visualization enhances the model’s clinical applicability.
**Limitations:** As a retrospective study, it is susceptible to information selection bias.
**Funding for this study:** This work was supported by the Key Project of Joint Construction by Provincial and Ministerial Authorities (Grant No.WKJ-ZJ-2452 to Minjiang Chen), Medical and Health General Project of Zhejiang Province (Grant No. 2023KY425 to Guihan Lin, Grant No. 2024KY562 to Shuiwei Xia), and Medical and Health Youth Innovation Project of Zhejiang Province (Grant No. 2023RC115 to Weiyue Chen).
**Ethics committee - additional information:** All procedures performed in studies involving human participants were in accordance with the ethical standards of the institutional and/or national research committee and with the 1964 Helsinki declaration and its later amendments or comparable ethical standards. This study was approved by the Institutional Review Board and Human Ethics Committee of the Fifth Affiliated Hospital of Wenzhou Medical University (2024-336), the Sixth Affiliated Hospital of Wenzhou Medical University, and the Third Affiliated Hospital of Wenzhou Medical University, with the requirement for patient informed consent being waived due to its retrospective nature. All patients' information was anonymized prior to the analysis.
**Author Disclosures:**
Jiansong Ji: Nothing to disclose
Weiyue Chen: Nothing to disclose
Guihan Lin: Nothing to disclose

**Histogram Analysis in Predicting the Intracranial Meningioma Grading Based on Amide Proton Transfer-Weighted Imaging**
*Y. H. Lee*, B-H. Kim, M. Kim, S-D. Kim; Ansan-Si/KR
*(younghen@korea.ac.kr)*

**Purpose or Learning Objective:** To determine whether amide proton transfer-weighted (APTW) histogram analysis is useful for predicting the grade of meningioma
**Methods or Background:** We retrospectively enrolled a total of 48 patients (M:F=16:32, mean age: 60.0 years; grade 1:grade 2/3=36:12) with pathologically proven intracranial meningioma who underwent mDIXON 3D-APT sequence of the fast spin echo method in addition to conventional 3T MR protocols prior to surgical resection. From the representative APTW images of the tumor registered with gadolinium-enhanced T1 images, the following parameters of each histogram were obtained: every 5 intervals from 5th to 95th percentile, mean, median, maximum, minimum, standard deviation, kurtosis and skewness. The diagnostic performance of each APTW histogram parameter for differentiating grade 2/3 from grade 1 intracranial meningioma were evaluated by drawing the receiver operating characteristic (ROC) curves and calculating the cut-off values
**Results or Findings:** Among all histogram parameters, only maximum, standard deviation and 75th,80th,85th,90th, and 95th percentiles for APTW signal in grade 2/3 were significantly higher than those of grade 1 ($p<0.05$). According to the ROC curve comparison analysis, the areas under the curves of 75th,80th,90th,95th percentile, maximum and standard deviation to discriminate grade 2/3 from grade 1 were 0.706,0.713,0.722,0.734, 0.738, 0.722, and 0.718, respectively.
**Conclusion:** Histogram analysis of APTW imaging can be used in clinical practice for grading of intracranial meningiomas
**Limitations:** 1.ROI-dependency, 2.small number of participants
**Funding for this study:** None
**Ethics committee - additional information:** None
**Author Disclosures:**
Young Hen Lee: Nothing to disclose
Baek-Hyun Kim: Nothing to disclose
Sang-Dae Kim: Nothing to disclose
Myungji Kim: Nothing to disclose

**Prognostic utility of intratumoral susceptibility signals in adult diffuse gliomas: a radiopathological study**
*J. I. Tudela Martínez*, V. Vázquez Sáez; Murcia/ES

**Purpose or Learning Objective:** Intratumoral susceptibility signals (ITSS) are promising radiological markers for assessing diffuse gliomas. This study evaluates the relationship between ITSS grading and key radiological and histopathological prognostic factors in adult diffuse gliomas.
**Methods or Background:** Between January 1st, 2022, and April 30th, 2024, we selected 99 patients diagnosed with adult diffuse glioma who met the following criteria: age over 18 years, MRI scans allowing ITSS quantification and confirmed pathological diagnosis with available molecular testing. Radiological variables included tumor volume, subventricular zone involvement and relative cerebral blood volume (rCBV) on MRI perfusion. Histopathological features examined were WHO-2021 grade, Ki-67 index, mitotic count, necrosis, microvascular proliferation, and key prognostic mutations (IDH, p53, ATRX, and CDKN2A/B). Spearman's correlation and chi-square tests were used for quantitative and qualitative variables, respectively. Multiple logistic regression models were developed to predict WHO tumor grade, categorized as low (1-2) or high (3-4), based on ITSS grade, tumor volume, and rCBV.
**Results or Findings:** ITSS grades 0-1 were more common in oligodendrogliomas and astrocytomas, while grades 2-3 were linked to glioblastomas ($p<0,001$). ITSS grade positively correlated with rCBV, tumor volume, WHO grade, mitotic count, and Ki-67 index ($p<0,001$). Higher ITSS grades also showed increased necrosis and microvascular proliferation ($p<0,001$). IDH mutations and 1p/19q co-deletions were more prevalent in grades 0-1 ($p<0,001$ and $p=0,001$, respectively), while CDKN2A/B alterations correlated with grades 2-3 ($p=0,02$). Regression models showed AUCs of 0,937 and 0,960 for ITSS combined with rCBV and tumor volume, respectively ($p=0,000$).
**Conclusion:** ITSS represent valuable biomarkers for assesing diffuse gliomas, offering diagnostic and prognostic insights that can guide clinical decision-making. Additionally, combining ITSS with MRI-rCBV and tumor volume enhances predictive capacity of these radiological parameters.
**Limitations:** ITSS grading remains semi-quantitative; further studies should focus on fully quantifying ITSS data.
**Funding for this study:** None
**Ethics committee - additional information:** We consulted with the ethics and research committee regarding the need for approval for the study. They confirmed that, due to its observational nature, such approval is not required.
**Author Disclosures:**
Victoria Vázquez Sáez: Nothing to disclose
Jose Ignacio Tudela Martínez: Nothing to disclose

**Relationship between Whole-tumor MRI-based Fractal Analysis and Molecular Features in IDH-wildtype Glioblastoma**
*B. Zhang*, J. Zhou; Lanzhou/CN

**Purpose or Learning Objective:** Molecular mechanisms and specific genes involved in the growth of Glioblastoma (GBM) are important factor in deciding the treatment strategy. In this study, we aimed to non-invasively explore the relationship between whole-tumor MRI-based fractal features and molecular features of GBM.
**Methods or Background:** The clinical and imaging data of 104 patients with IDH-wildtype GBM at our hospital between November 2018 and June 2024 were retrospectively analyzed. The molecular features of GBM were collected by molecular sequencing and immunohistochemical method, including MGMT promoter methylation, 1p/19q-codeleted, TERT promoter mutation, Ki67, and P53 status. The volume of interest of whole tumor was manually segmented slice-by-slice using ITK-SNAP software. Fractal features of whole-tumor in contrast-enhanced T1-weighted imaging were extracted using Image J software. As many as 24 fractal features (fractal dimensions and lacunarity) were generated within each volume of interest. Correlation analyses were performed using Spearman correlation analysis. Logistic regression was used to build prediction models.
**Results or Findings:** The L1 and L4 were positively correlated with 1p/19q-codeleted (correlation coefcient: 0.213 and 0.212). The L5 was positively correlated with TERT promoter mutation (correlation coefcient: 0.326). The L1, L4, and L9 were negatively correlated with TERT promoter mutation (correlation coefcient: -0.251, -0.310, -0.196, respectively). The L1, L3, and L4 were positively correlated with Ki-67 proliferation index (correlation coefcient: 0.226, 0.200, 0.241, respectively). MGMT promoter methylation and P53 had no correlation with fractal features. The AUC of fractal features predicting 1p/19q-codeleted was 0.677 and predicting TERT promoter mutation was 0.755.
**Conclusion:** The fractal features were correlated with 1p/19q-codeleted, TERT promoter mutation, and Ki67 status in IDH-wildtype GBM. Fractal features can be used as non-invasive quantitative parameters to predict the molecular features of GBM.
**Limitations:** Not
**Funding for this study:** This study was supported by the National Natural Science Foundation of China (grant no. 82071872 and 82371914), the Science and Technology Program of Gansu Province (grant no. 21YF5FA123 and 21JR11RA105), and the China International Medical Foundation (grant no. Z-2014-07-2101).
**Ethics committee - additional information:** This study was approved by the Medical Ethics Committee of the Second Hospital of Lanzhou University (approval number : 2020A-070) and informed consent was waived.
**Author Disclosures:**
Junlin Zhou: Nothing to disclose
Bin Zhang: Nothing to disclose

**T1 Curves in the evaluation of radionecrosis or disease recurrence**
*C. Monopoli*, A. Romano, G. De Rosa, A. Romano, G. Moltoni, A. M. Ascolese, G. Capriotti, A. Bozzao; Rome/IT
*(cristiana.monopoli@uniroma1.it)*

**Purpose or Learning Objective:** Radiation treatment of brain metastases creates diagnostic doubts in the differential diagnosis between disease progression or radionecrosis induced by radiosurgery. MRI with the administration of contrast medium doesn't offer the possibility of reliably distinguishing the two pathological entities. The aim of our study is to verify the presence of radionecrosis or disease recurrence through the evaluation of T1 enhancement curves.

**Methods or Background:** 40 brain metastases undergone to radiosurgery were evaluated (32 from lung, 4 from breast, 2 from melanoma and 2 colorectal). All patients underwent MRI examination with dynamic T1 acquisitions with contrast medium. For each lesions the T1 enhancement curve was extracted by positioning a region of interest corresponding to the solid component, excluding the necrotic areas. All patients underwent a PET-DOPA study and the result of the examination was used as the gold standard to distinguish radionecrosis from disease progression. The PET investigations identifies three stages of the disease; radionecrosis (rSUV<1.6); mixed picture (rSUV between 1.6 and 1.9); disease progression (rSUV>1.9)
**Results or Findings:** 4 types of T1 enhancement curves have been identified (A-D). Curve A showed constant growth over time; curve B showed faster growth in its initial portion and constant growth over time; curve C showed rapid initial growth and a final plateau; Curve D showed rapid growth and rapid final washout. Of the 40 lesions, 13 showed uptake compatible with radionecrosis, 12 with a mixed picture and 15 with disease progression. Curves A and B corresponded to radionecrosis or mixed in 90% of cases, curves C and D corresponded to a progression of the disease in 95% of cases.
**Conclusion:** T1 enhancement curves allows to distinguish a condition of radionecrosis from a progression of the disease.
**Limitations:** Small enrolled population
**Funding for this study:** Not funding received
**Ethics committee - additional information:** Not applicable.
**Author Disclosures:**
Cristiana Monopoli: Nothing to disclose
Anna Maria Ascolese: Nothing to disclose
Andrea Romano: Nothing to disclose
Gabriella Capriotti: Nothing to disclose
Alessandro Bozzao: Nothing to disclose
Allegra Romano: Nothing to disclose
Giulia Moltoni: Nothing to disclose
Giulia De Rosa: Nothing to disclose

**PET/MRI in brain primary and secondary tumors treated with radiochemotherapy: a radiomic-based analysis of brain Perfusion MRI and 11C-Methionine PET images acquired by a integrated hybrid system**
*E. Masiello*, M. Barbera, F. Fallanca, S. Paola, A. Castellano, A. Falini, N. E. Anzalone; Milan/IT
*(e.masiello@studenti.unisr.it)*

**Purpose or Learning Objective:** Perfusion-weighted MRI (PWI) and 11C-methionine PET (MET-PET) provide valuable hemodynamic and metabolic insights for assessing brain tumors. This study aimed to investigate the diagnostic role of PWI and MET-PET, using radiomic analysis, in distinguishing progression (PD), pseudoprogression (PsP), and radionecrosis (RN) in patients with brain tumors treated with radiotherapy (RT) or radiochemotherapy.
**Methods or Background:** Patients with primary and secondary brain neoplasms who developed post-treatment lesions of at least 1 cm within the radiation field were retrospectively enrolled. All patients underwent simultaneous PET/MRI examinations according to a standardized protocol. Radiomics features were extracted from the 3D-segmentation of parametric maps, including relative cerebral blood volume (rCBV) from DSC, plasma volume (Vp) and vascular permeability (Ktrans) from DCE, relative cerebral blood flow (CBF) based on pseudo-Continuous Arterial Spin Labeling (pCASL), and Standardized Uptake Value (SUV) from MET-PET. For each lesion, imaging data were compared with outcomes based on RANO criteria or histological examination.
**Results or Findings:** A semi-automatic 3D segmentation of 52 lesions (23 PD and 29 RN) was performed using PMod software (v. 3.7) to extract 263 radiomic features. After feature selection, CBF Maximum gray level and SUV Maximum gray level were the most correlated ($p < 0.001$). In terms of accuracy, the highest area under the curve (AUC) for PET features was SUV Entropy, while Vp Mean showed the highest AUC for PWI features. SUV Entropy achieved the highest sensitivity for detecting PD (95.65%), while rCBV Entropy demonstrated the highest specificity (79.31%). PET and PWI parameters exhibited similar overall accuracy, ranging from 71.15% to 76.92%.
**Conclusion:** Radiomics features from MET-PET and PWI demonstrate strong potential in accurately distinguishing PD and PsP from RN. Combining both modalities using radiomics enhances overall diagnostic accuracy.
**Limitations:** Small and heterogeneous population
**Funding for this study:** No funding was received for this study.
**Ethics committee - additional information:** The study is retrospective.
**Author Disclosures:**
Andrea Falini: Nothing to disclose
Nicoletta Emanuela Anzalone: Nothing to disclose
Edoardo Masiello: Nothing to disclose
Scifo Paola: Nothing to disclose
Antonella Castellano: Nothing to disclose
Maurizio Barbera: Nothing to disclose
Federico Fallanca: Nothing to disclose

**Multiparametric MRI-based clinical radiomics model for predicting TERTp genotype and overall survival in oligodendrogliomas**
*J. Zhao*[1], X. Ke[1], T. Gan[1], W. Hu[1], C. Xue[2], S. Li[3], Q. Zhou[1], J. Zhou[1]; [1]Lanzhou/CN, [2]Qingdao/CN, [3]Chengdu/CN
*(gtjlovezj@163.com)*

**Purpose or Learning Objective:** To test the hypothesis that combining features from multiparametric MRI with clinically relevant prognostic risk factors provides a more accurate prediction of TERTp genotype and overall survival(OS)in patients with oligodendrogliomas(OGS).
**Methods or Background:** Preoperative multiparametric MRI sequences (T1WI, T2WI, and CE-T1-3D) from 135 patients with OGS (grades 2 and 3) were collected and randomly divided into training (n = 95) and validation (n = 40) sets. Radiomics features were extracted, and the least absolute shrinkage and selection operator regression was used to select the most relevant features. Clinical relevant features identified through univariate and multivariate logistic regression analyses were incorporated to establish a clinical radiomics model. This model was used to develop a predictive TERTp genotype nomogram. Kaplan–Meier curves were used to assess OS differences between TERTp groups; the log-rank test determined significance.
**Results or Findings:** The T2WI-based clinical radiomics model demonstrated superior performance in predicting the TERTp genotype, with mean area under the receiver operating characteristic curve (AUC) values of 0.90 (95% CI: 0.88, 0.92; $P = 0.0002$) in the training set and 0.83 (95% CI: 0.82, 0.85; $P < 0.0001$) in the validation set. The one year, two year and three year survival probability prediction models achieved AUC values of 0.85, 0.80, and 0.79, respectively.
**Conclusion:** The multiparametric MRI-based clinical radiomics model provides the most accurate prediction of the TERTp genotype. Combined with clinical relevant prognostic risk factors, the prognostic model offers precise prediction of OS in patients with OGS.
**Limitations:** The limitation of this study was three-dimensional tumor segmentation was manually performed, future research should explore automated and efficient segmentation methods to reduce workload and simplify clinical application.
**Funding for this study:** Funding were provided by National Natural Science Foundation of China (82071872, 82371914), Science and Technology Program Funding Project of Gansu Province ( 21JR7RA404).
**Ethics committee - additional information:** The ethics committee notification can be found under the number 2021A-348.
**Author Disclosures:**
Shenglin Li: Nothing to disclose
Wanjun Hu: Nothing to disclose
Jun Zhao: Nothing to disclose
Junlin Zhou: Nothing to disclose
Caiqiang Xue: Nothing to disclose
Qing Zhou: Nothing to disclose
Xiaoai Ke: Nothing to disclose
Tiejun Gan: Nothing to disclose

**Intraoperative and postoperative MRI detection of ischemia in brain tumor surgery: a retrospective study on predictive factors and ischemic evolution**
*M. R. López De La Torre Carretero*, C. Mbongo, P. Corral Alonso, D. A. Zambrano, Á. R. Cabrera Abud, C. D. Solano, J. M. Rodríguez Ortega, M. Macías de la Corte Hidalgo, M. Calvo Imirizaldu; Pamplona/ES
*(mltorrec@unav.es)*

**Purpose or Learning Objective:** Intraoperative magnetic resonance imaging (iMRI) is an increasingly valuable tool in neurosurgical oncology, particularly for guiding tumour resection by real-time detection of residual tumour and margins, but also acute ischemic complications using diffusion weighted imaging (DWI). However, some studies suggest iMRI may underestimate ischemia compared to early postoperative MRI (epMRI), highlighting the importance of thorough postoperative evaluation. Our study aimed to compare iMRI´s ability to detect ischemia against epMRI and late postoperative MRI (lpMRI), and to identify predictive factors for postoperative ischemia in patients undergoing brain tumour resection.
**Methods or Background:** This retrospective study included 106 patients undergoing brain tumor resection at our centre. iMRI, epMRI (5-7 days post-surgery), and lpMRI (30 days post-surgery) were performed. Two radiologists analysed imaging to detect intraoperative ischemia (IOI), early postoperative ischemia (EPI), and late postoperative ischemia (LPI) using (DWI) and quantifying ischaemic volume on apparent diffusion coefficient (ADC) maps. Variables such as age, sex and tumour histology were recorded. Statistical analysis was conducted (StataNow 18.5) using McNemar tests, Pearson's chi-squared test, and multivariate logistic regression.
**Results or Findings:** McNemar test revealed that iMRI tends to underestimate ischaemia compared to epMRI ($p= 0.0039$). Only tumour type was a significant predictor of EPI ($p=0.041$), with glioblastoma patients having lower probabilities of EPI compared to other tumour types ($p=0.041$). EPI and

LPI were significantly associated (p <0.001), indicating ischaemia detected in epMRI didn't progress in lpMRI.
**Conclusion:** Our results suggest iMRI may underestimate the ischemia compared to epMRI. Glioblastoma was associated with a lower risk of ischemia, highlighting the importance of personalized surgical approach. The strong relationship between EPI and LPI emphasizes the need for close follow-up to monitor late complications.
**Limitations:** Retrospective study.
Clinical outcomes could be useful (further studies)
**Funding for this study:** No funding was provided for this study
**Ethics committee - additional information:** Not applicable
**Author Disclosures:**
Carmen Mbongo: Nothing to disclose
José Marlon Rodríguez Ortega: Nothing to disclose
Paula Corral Alonso: Nothing to disclose
Marta Macías de la Corte Hidalgo: Nothing to disclose
Manuel Rafael López De La Torre Carretero: Nothing to disclose
Marta Calvo Imirizaldu: Nothing to disclose
Daniel Alfonso Zambrano: Nothing to disclose
Álvaro Rafael Cabrera Abud: Nothing to disclose
Carlos Delgado Solano: Nothing to disclose

**Supratentorial Changes in Patients with Cerebellopontine Angle Tumors: A Comprehensive Morphologic Analysis**
*A. Çolakoğlu*, B. Genç, K. Aslan, L. Incesu; Samsun/TR
*(ardacolakoglu@hotmail.com)*

**Purpose or Learning Objective:** Neuroplasticity that develops in intracranial tumors may guide the management of post-surgical or radiotherapy treatment. To our knowledge, there is no study investigating morphometric changes in the brain of patients with cerebellopontine angle tumors. Our aim in this study is to investigate supratentorial morphometric changes in patients with cerebellopontine angle tumors.
**Methods or Background:** The study included 29 patients with cerebellopontine angle tumors who had not yet received any treatment, and 53 age- and sex-matched healthy controls. Voxel-based morphometry and surface-based morphometry analyses were performed using CAT12, running under SPM12, to examine gray matter volume changes and cortical thickness changes in these patients. A general linear model was used for statistical analysis, and a p-value<0.05 with family-wise error (FWE) correction was considered statistically significant.
**Results or Findings:** Our VBM results showed an increase in gray matter volume in the thalamus, ventral diencephalon, cingulate gyrus, precuneus, cuneus, superior parietal lobe, and parahippocampal gyrus in these patients (p<0.05 FWE). Our SBM results revealed an increase in cortical thickness in the right superior parietal and paracentral gyri in patients with cerebellopontine angle tumors (p<0.05 FWE).
**Conclusion:** Our study is the first to demonstrate an increase in gray matter volume and cortical thickness in the supratentorial region of patients with cerebellopontine angle tumors. These findings may be associated with neuroplastic changes in these patients.
**Limitations:** The limitations of the study are its single-center design, its retrospective nature, and the absence of neurocognitive tests.
**Funding for this study:** No funding was received for this study.
**Ethics committee - additional information:** The study is approved by clinical studies ethics committee of Ondokuz Mayıs University. The reference number is 2024090641.
**Author Disclosures:**
Bariş Genç: Nothing to disclose
Lütfi Incesu: Nothing to disclose
Arda Çolakoğlu: Nothing to disclose
Kerim Aslan: Nothing to disclose

**16:00-17:30 Research Stage 1**

### Research Presentation Session: Chest

### RPS 1604

### Lung cancer screening and nodule assessment

**Moderator**
D. Shaham; Jerusalem/IL
*(dshaham@hadassah.org.il)*
**Author Disclosures:**
Dorith Shaham: Consultant: AstraZeneca

**Automated triage to a virtual lung nodule clinic using Veye Lung Nodule volumetry could provide a cost saving compared to standard care**
*G. Dixon*[1], V. Dattani[1], M. Jordan[2], J. Rodrigues[1]; [1]Bath/UK, [2]Coventry/UK
*(gilesdixon@nhs.net)*

**Purpose or Learning Objective:** Pulmonary nodules are frequently identified on non-screening CT chest scans. The management of pulmonary nodules in the UK is governed by the British Thoracic Society (BTS) Guidance 2015. Artificial intelligence can provide automated nodule identification and volumetry. The clinical and cost effectiveness of incorporating automated nodule detection into patient pathways are currently unclear.
**Methods or Background:** All CT scans including the thorax over one week in a single UK centre during March 2022 were identified and their original reports reviewed. CT scans were categorised as to whether the original report gave a BTS recommendation or not. Currently, patients without a BTS recommendation are assumed to undergo a full lung MDT discussion to determine the recommendation. A virtual nodule clinic was modelled whereby all patients with >1 nodule of >80mm3 identified using Veye Lung Nodules who did not receive a BTS recommendation in the original CT report were referred to a virtual nodule clinic. The cost of this approach was compared to current practice.
**Results or Findings:** 80/357 scans had > 1 pulmonary nodule >80mm3 identified using Veye Lung Nodules. 51/80 patients did not receive a BTS Guideline based recommendation in the original radiologist report. A potential cost saving of £9.48 per case to review these cases in a virtual lung nodule clinic was calculated. This represents a cost saving of £483.48 during the study period (one week) and a potential annual cost saving of between £25,141 and £36,854.
**Conclusion:** The study identified a potential cost saving of automated triage of lung nodules into a virtual lung nodule clinic.
**Limitations:** Further work is ongoing to model longitudinal costs of this cohort of patients.
**Funding for this study:** No specific funding was used for this study
**Ethics committee - additional information:** N/A
**Author Disclosures:**
Giles Dixon: Speaker: Aidence
Vruti Dattani: Nothing to disclose
Mary Jordan: Nothing to disclose
Jonathan Rodrigues: Other: Aidence Speaker: Aidence

**SOLACE - CT acquisition protocols in lung cancer screening across Europe**
*M. F. G. Konrad*[1], E. Nischwitz[1], V. Palm[1], O. Von Stackelberg[1], A. Baca-Stera[2], K. Błasińska[2], M. Adamek[3], J. Chorostowska-Wynimko[2], H-U. Kauczor[1]; [1]Heidelberg/DE, [2]Warsaw/PL, [3]Gdansk/PL
*(mathis.konrad@med.uni-heidelberg.de)*

**Purpose or Learning Objective:** Exploration of the current status of institutional and technical factors of CT acquisition protocols applied in Europe that may affect the radiation exposure of screening participants in lung cancer screening procedures.
**Methods or Background:** To achieve an overview of the applied CT acquisition protocols in lung cancer screening across Europe, data were acquired from leading investigators responsible for the definition of CT acquisition protocols in the screening centres of the SOLACE consortium. Data regarding institutional and technical factors of CT acquisition protocols were collected through a baseline survey. All data embody the current status between June and October 2024.
**Results or Findings:** Survey responses of 16 screening centres from 10 European countries (Croatia, Czechia, Estonia, France, Greece, Hungary, Ireland, Italy, Poland and Spain) were received. In relation to institutional factors, the CT acquisition protocols are established and modified by personnel of multiple professional roles (radiologists, radiographers, medical physicists, manufacturer personnel). In eight of 16 (50 %) institutions the protocol is established by a multiprofessional team. CT protocols are mostly modifiable (88 %). Regarding technical factors, the number of detector rows in the z-direction ranges from 16 to 128. In 13 of 16 centres (81 %) automatic exposure control is applied. The applied reconstructed slice thickness and increment lies in the range of 0.625 - 1.25 mm.
**Conclusion:** Lung cancer screening in Europe within the SOLACE consortium is widely implemented reaching the technical limits of currently operated devices. The definition of CT acquisition protocols as a team effort and the possible modification of protocols are subjects of imaginable improvements.
**Limitations:** The brief survey data stems only from institutions with research focus.
**Funding for this study:** This project is co-funded under the EU4Health Programme 2021–2027 under grant agreement no. 101101187
**Ethics committee - additional information:** Not a patient study
**Author Disclosures:**
Joanna Chorostowska-Wynimko: Nothing to disclose
Alicja Baca-Stera: Nothing to disclose
Katarzyna Błasińska: Nothing to disclose
Hans-Ulrich Kauczor: Nothing to disclose
Oyunbileg Von Stackelberg: Nothing to disclose
Emily Nischwitz: Nothing to disclose
Viktoria Palm: Nothing to disclose
Mathis Franz Georg Konrad: Other: This contribution has been prepared on behalf of the SOLACE consortium
Mariusz Adamek: Nothing to disclose

**Variations in participant positioning, scan direction and scanogram angle influence organ-specific radiations doses in routine low-dose chest CT for lung cancer screening**
*L. D'Hondt*[1], C. Haentjens[1], A. Snoeckx[2], K. Bacher[1]; [1]Ghent/BE, [2]Antwerp/BE
*(louisdho.dhondt@ugent.be)*

**Purpose or Learning Objective:** In lung cancer screening (LCS), non-ideal positioning, changes in scan direction or scanogram angles are likely to occur due to the high turnover of participants and large volume of scans performed. Additionally, since we are dealing with healthy individuals, careful management of radiation dose is crucial. This study aims to systematically simulate how participant positioning and scanning parameters affect organ-specific radiation doses.
**Methods or Background:** Using the Alderson Rando phantom, we performed CT scans under varying conditions on two scanners (GE Revolution, Siemens SOMATOM Definition Flash) to establish automatic tube current modulation (ATCM) variations. ImpactMC Monte Carlo software simulated low-dose scans for 32 patient-specific voxel models, calculating percentage dose differences to lungs, heart, thyroid, liver, and female breasts. Deviations included lateral/vertical mispositioning (2cm increments), scan direction changes (craniocaudally, caudocranially), and varying scanogram angles (anteroposterior, posteroanterior, lateral, combined).
**Results or Findings:** Vertical deviations caused linear increase in organ doses when positioned closer to the tube during scanogram, with the GE scanner showing a 12-24% increase per 2cm deviations. For the Siemens scanner this linear increase was less pronounced (3-9% increase per 2cm deviation). Lateral mispositioning increased organ doses by <10% for both scanners. Caudocranial scanning only increased thyroid doses by 15.8% for GE, while Siemens in fact showed a 19.7% decrease in thyroid dose and significant increases in lung (18.7%), heart (28.7%), liver (32.6%), and breast (27.9%) doses. For both scanners, the highest doses occurred with a posteroanterior scanogram. Siemens showed 72% dose reduction with dual scanogram, while GE showed minimally 34% reduction with an anteroposterior scanogram.
**Conclusion:** Non-ideal positioning and varying CT parameters can significantly affect organ doses, potentially underestimating the anticipated organ-specific doses and related radiation-induced cancer risk in LCS.
**Limitations:** Limitations are use of a phantom.
**Funding for this study:** Funding was provided by the FWO “Kom op tegen Kanker”-project for lung cancer screening research in Belgium. (Project number: G0B1922N).
**Ethics committee - additional information:** Not applicable
**Author Disclosures:**
Klaus Bacher: Nothing to disclose
Louise D'Hondt: Nothing to disclose
Annemiek Snoeckx: Nothing to disclose
Claudia Haentjens: Nothing to disclose

**Pilot National Lung Cancer Screening in Poland – summary of the northern macroregion results - continuation of the Gdansk experience**
*J. Bidzinska*, J. Rymarowicz, K. Kobyłecka, K. Dziadziuszko, E. Szurowska, W. Rzyman; Gdansk/PL
*(jbidzinska@gumed.edu.pl)*

**Purpose or Learning Objective:** The aim of he study was to evaluate the results of the Pilot National Lung Cancer Screening program in the northern macroregion of Poland.
**Methods or Background:** After primary prevention, lung cancer screening is the strategy to reduce lung cancer-related mortality. A total of 3068 asymptomatic volunteers between 50 and 74 years of age with a smoking history of at least 20 pack years underwent screening with the use of low-dose computed tomography of the chest. The radiological nodule management protocol was designed for a 2-year follow-up. It was created specifically for the national program and implemented nationwide. In the northern macroregion, LDCT was performed in 4 radiological centers. All radiologists were trained. The program included an anti-smoking intervention.
**Results or Findings:** At the time of submitting this abstract, 59 (1.92%) participants of the Program were diagnosed with lung cancer. In this group, 43 participants had resectable lung cancer, which is 73% of cases, including 22 women and 21 men. Few participants undergo diagnostic workups. In addition, we assessed (or the analysis is ongoing) the lung cancer screening adherence rate, number and type of lung nodules, presence and severity of emphysema, CAC, incidental findings, and effectiveness of smoking cessation intervention.
**Conclusion:** Lung cancer screening with low-dose computed tomography of the chest is an effective tool for reducing lung cancer-related mortality. LDCT is an effective tool for the identification of comorbidities. It should be implemented as a National Lung Cancer Screening Strategy.
**Limitations:** Quality control in one of the sites revealed that the radiologist should be re-trained periodically.
**Funding for this study:** The program was financed by the Ministry of Health. The lung cancer prevention program was implemented in accordance with the competition regulations No. POWR.05.01.00-IP.00-010/19 Operational Program Knowledge Education and Development 2014-2020 Priority axis V Support for the health area Measure 5.1 Prevention programs.
**Ethics committee - additional information:** The study was approved by the Institutional Review Board of the Medical University of Gdańsk (NKBBN/72/2020). During realisation of the program we asked all apriticipants for the consent to use of data in the future analyses and studies. Only 8 participanst did not consent.
**Author Disclosures:**
Katarzyna Dziadziuszko: Nothing to disclose
Katarzyna Kobyłecka: Nothing to disclose
Julia Rymarowicz: Nothing to disclose
Witold Rzyman: Nothing to disclose
Edyta Szurowska: Nothing to disclose
Joanna Bidzinska: Nothing to disclose

**Implications of 2D single slice vs. 3D whole-chest body composition phenotyping on outcome prediction: insights from the National Lung Screening Trial**
*J. Jahn*, F. B. Pallasch, M. Jung, M. Reisert, F. Bamberg, J. Weiß; Freiburg/DE
*(johannes.jahn94@gmx.de)*

**Purpose or Learning Objective:** Body composition (BC) is linked to outcomes in cardiovascular (CV) disease and cancer. BC is typically estimated from a 3rd lumbar slice, but the role of standardized chest heights and 3D volumes is unclear. Here, we used a fully automated deep learning network 1) to investigate the correlation between 2D single-slice areas and 3D whole-chest volumes and 2) to explore their association with mortality in heavy smokers.
**Methods or Background:** Using baseline data from the National Lung Screening Trial, BC was estimated on chest CT as skeletal muscle (SM), intramuscular (IMAT), and subcutaneous adipose tissue (SAT). Correlations between 2D thoracic vertebra slices and 3D chest volumes were explored. Associations between BC measures and all-cause mortality were assessed for both approaches, secondarily for CV and lung cancer mortality. Kaplan-Meier curves (categories <15%; 15-85%; >85%) and Cox regression models adjusted for demographics and CV risk factors were used.
**Results or Findings:** Among 23,361 individuals (mean age 61.4±5, 41.6% female), 1,616 (6.9%) all-cause deaths occurred over a median follow-up of 6.5 years. The highest correlation between 2D and 3D volumes was at T4 vertebra. For 3D volumes, lower SM, lower SAT, and higher IMAT were associated with higher mortality in Kaplan-Meier curves (p<0.01). Associations between these groups remained robust after multivariable adjustment (adjusted hazard ratio (aHR): 1.51, 95% CI 1.33-1.71, p<0.001; aHR: 1.21, 95% CI: 1.04-1.40, p<0.05; aHR: 1.68, 95% CI: 1.36-1.78, p<0.001, respectively). Largely similar patterns were found for 2D measures, CV, and lung cancer mortality.

**Conclusion:** BC measures independently predict mortality in heavy smokers beyond traditional clinical risk factors. 2D measures at T4 and 3D BC showed similar results, offering interchangeable use for identifying high-risk individuals in lung cancer screening, potentially enhancing personalized prevention.
**Limitations:** No limitations were identified.
**Funding for this study:** No funding was received for this study.
**Ethics committee - additional information:** All National Lung Screening Trial participants provided written informed consent for the original trial and secondary use of the data was approved by the local IRB.
**Author Disclosures:**
Marco Reisert: Nothing to disclose
Jakob Weiß: Nothing to disclose
Matthias Jung: Nothing to disclose
Fabian Bamberg: Speaker: Bayer Healthcare Research/Grant Support: Siemens Healthineers Consultant: Bayer Healthcare Speaker: Siemens Healthineers Research/Grant Support: Bayer Healthcare
Fabian Bernhard Pallasch: Nothing to disclose
Johannes Jahn: Nothing to disclose

**Photon-Counting Detector CT Provides Superior Subsolid Nodule Characterization Compared to Same-Day Energy-Integrating Detector CT**
*J. Wang*, L. Song, W. Song; Beijing/CN
*(981891153@qq.com)*

**Purpose or Learning Objective:** To investigate the performance of photon-counting detector (PCD) CT compared to conventional energy-integrating detector (EID) CT in identifying subsolid nodule (SSN) characteristics.
**Methods or Background:** Participants with SSNs who underwent same-day EID CT and PCD CT between October 2023 and April 2024 were prospectively included. The 1.0 mm EID CT images and, subsequently, 1.0, 0.4, and 0.2 mm PCD CT images were reviewed to assess image noise and subjective image quality on a 5-point Likert scale. SSN characteristics, including lobulation, spiculation, pleural retraction, air cavities, intra-nodular vessel sign, internal vascular changes, and heterogeneous solid components, were evaluated.
**Results or Findings:** Forty-eight participants (mean age: 56 ± 11 years; 16 males) with 89 SSNs were included. PCD CT significantly reduced radiation dose when using matched scans (1.79 ± 0.39 vs. 2.17 ± 0.57 mSv, $P < 0.001$). Compared to 1.0 mm EID CT, 1.0 mm PCD CT images exhibited significantly lower objective image noise and higher subjective image quality (all $P < 0.001$). Compared to EID CT, PCD CT demonstrated enhanced visualization of subtle characteristics, except for lobulation, with a 0.4 mm section thickness offering a favourable balance between ultra-high resolution and perceived image quality for radiologists.
**Conclusion:** PCD CT facilitated radiation dose reduction and outperformed conventional EID CT in terms of image quality and visualization of SSN characteristics.
**Limitations:** First, the primary focus was on SSNs with various imaging characteristics suggestive of malignancy; consequently, SSNs < 6 mm were not included in the study. Second, quantitative SSN analysis was not performed because the study aimed to explore the qualitative value of PCD CT in assessing SSN characteristics. Third, evaluation of nodules on a mediastinal window was not performed in this study. Fourth, not all SSNs yielded histopathologic results.
**Funding for this study:** This work has received funding by the National Natural Science Foundation of China (NSFC No. 82171934) and the National High Level Hospital Clinical Research Funding (2022-PUMCH-B-069).
**Ethics committee - additional information:** The Institutional Review Board of our hospital approved this prospective study [I-23PJ1459].
**Author Disclosures:**
Wei Song: Nothing to disclose
Lan Song: Nothing to disclose
Jinhua Wang: Nothing to disclose

**Diagnostic Outcomes of Korean Lung-RADS Category 2b Nodules in Lung Cancer Screening: A Single Tertiary Hospital Experience**
*Y. Kim*, T. Ha, S. You, J. Sun; Suwon/KR
*(dbsk93@gmail.com)*

**Purpose or Learning Objective:** To evaluate the clinical outcomes of Korean Lung-RADS category 2b nodules in lung cancer screening and to compare the diagnostic performance between Korean Lung-RADS and Lung-RADS.
**Methods or Background:** This retrospective study included 2,908 participants who underwent low-dose chest CT scans at a single tertiary hospital between September 2019 and January 2024, as part of the Korean national lung cancer screening program. The modified Korean Lung-RADS added category 2b for nodules sized as category 3 or 4 but likely benign, aiming to reduce false positives. All CT scans were interpreted using the Korean Lung-RADS categorization (version 1.0 until 2021, version 1.1 from 2022, and version 2022 from 2024). To assess interobserver agreement, three radiologists independently reassessed each 2b nodule, indicating whether they would classify it as category 2b. Diagnostic performance comparisons were conducted using McNemar and permutation tests, while interobserver agreement was analyzed using multirater Fleiss κ statistics.
**Results or Findings:** 1,270 participants with confirmed diagnoses were finally included (mean age, 61.5 ± 5.2 years; 1,243 men). Lung cancer was identified in 28 participants (2.2%). Category 2b nodules were found in 24 participants (1.9%), with one diagnosed as lung cancer (4.2%) and the others benign (95.8%). Interobserver agreement was fair with a Fleiss kappa value of 0.36 (95% CI 0.17-0.79). In terms of diagnostic performance, Korean Lung-RADS showed significantly higher specificity (92.5%, 1,149 of 1,242) compared to Lung-RADS (90.7%, 1,126 of 1,242; $p < 0.001$), while sensitivity was comparable between Korean Lung-RADS (89.36%, 25 of 28) and Lung-RADS (92.8%, 26 of 28; $p = 1$).
**Conclusion:** Korean Lung-RADS category 2b helped reduce the false-positive rate in lung cancer screening. However, significant variability was observed among radiologists in classifying category 2b nodules.
**Limitations:** Retrospective study with small sample size.
**Funding for this study:** No
**Ethics committee - additional information:** This retrospective study was approved by the institutional review board, which waived the requirement for written informed consent (AJOUIRB-DB-2024-399).
**Author Disclosures:**
Youna Kim: Nothing to disclose
Joosung Sun: Nothing to disclose
Seulgi You: Nothing to disclose
Taeyang Ha: Nothing to disclose

**Safety of CT-guided core needle biopsy in patients with interstitial lung abnormalities (ILAs)**
*M. Balbi*[1], S. Capelli[2], A. Caroli[2], N. C. Culasso[1], R. Senkeev[1], D. Morbidelli[1], G. Reboli[1], L. Righi[3], A. Veltri[1]; [1]Turin/IT, [2]Ranica/IT, [3]Orbassano/IT
*(balbi.m@libero.it)*

**Purpose or Learning Objective:** To evaluate the safety of CT-guided core needle biopsy (CNB) in patients with interstitial lung abnormalities (ILAs).
**Methods or Background:** Consecutive CT-guided pulmonary biopsies performed at the San Luigi Gonzaga Hospital (Orbassano, Italy) from February 2010 to December 2023 (n=3251) were retrospectively reviewed to identify patients with ILAs who underwent CNB for the assessment of a pulmonary lesion (n=73, case group). A control group of 73 CNB patients with no evidence of ILAs, matched for age, sex, emphysema severity, and lesion depth and dimensions, was selected to compare the complication rates. Logistic regression was performed to identify risk factors for complications within the case group, as well as to compare complication rates between the case and control groups.
**Results or Findings:** Complications occurred in 21/73 cases (29%) and 24/73 controls (33%), including 2 major complications in cases (3%) and 3 in controls (4%). Considering the overall occurrence of complications, there were no statistically significant differences between cases and controls, as assessed by Fisher's exact test (p=0.72) and conditional logistic regression adjusted for matched variables (p=0.72). Among patient, procedural, and lesion-related data, three variables were found to be risk factors for complications in the case group based on univariate, multivariate, and stepwise logistic regression with bidirectional elimination, guided by the Akaike information criterion (AIC): longer time of the needle within the lung (odds ratio (OR), 1.17; 95% confidence interval (CI), 0.72–13.02; $p = 0.126$), needle traversal of ILAs (OR, 7.04; 95% CI, 2.07–26.28; $p = 0.002$), and multiple pleural passes (OR, 8.06; 95% CI, 1.26–70.46; $p = 0.035$).
**Conclusion:** CT-guided CNB in patients with ILAs showed safety comparable to patients without ILAs. However, crossing ILAs during the procedure may increase the complication risk.
**Limitations:** -Retrospective, single-center study -Limited number of cases -Lack of inter-reader agreement
**Funding for this study:** The study did not receive any funding.
**Ethics committee - additional information:** The institutional review board (Comitato Etico Territoriale Interaziendale of the AOU Città della Salute e della Scienza di Torino) approved this retrospective observational case-control study and waived the requirement for written informed consent.
**Author Disclosures:**
Serena Capelli: Nothing to disclose
Luisella Righi: Nothing to disclose
Andrea Veltri: Nothing to disclose
Rouslan Senkeev: Nothing to disclose
Noemi Cristina Culasso: Nothing to disclose
Davide Morbidelli: Nothing to disclose
Anna Caroli: Nothing to disclose
Maurizio Balbi: Nothing to disclose
Giulia Reboli: Nothing to disclose

**Deep Learning-Based Image Domain Reconstruction Enhances Image Quality and Pulmonary Nodule Detection in Ultralow-Dose CT with Adaptive Statistical Iterative Reconstruction-V**

*K. Ye*[1], B. Pan[2], J. Li[3], Z. Pan[2], H. Yuan[1], N-J. Gong[1]; [1]Beijing/CN, [2]Shagnhai/CN, [3]Fujian/CN
*(yekaibysy@bjmu.edu.cn)*

**Purpose or Learning Objective:** To evaluate the image quality and lung nodule detectability of ultralow-dose CT (ULDCT) with adaptive statistical iterative reconstruction-V (ASiR-V) post-processed using a deep learning image reconstruction (DLIR)-based image domain compared to low-dose CT (LDCT) and ULDCT without DLIR.
**Methods or Background:** A total of 210 patients undergoing lung cancer screening underwent LDCT (mean ± SD, 0.81 ± 0.28 mSv) and ULDCT (0.17 ± 0.03 mSv) scans. ULDCT images were reconstructed with ASiR-V (ULDCT-ASiR-V) and post-processed using DLIR (ULDCT-DLIR). The quality of the three CT images was analyzed. Three radiologists detected and measured pulmonary nodules on all CT images, with LDCT results serving as references. Nodule conspicuity was assessed using a five-point Likert scale, followed by further statistical analyses.
**Results or Findings:** A total of 463 nodules were detected using LDCT. The image noise of ULDCT-DLIR decreased by 60% compared to that of ULDCT-ASiR-V and was lower than that of LDCT (p <0.001). The subjective image quality scores for ULDCT-DLIR (4.4[4,1, 4.6]) were also higher than those for ULDCT-ASiR-V (3.6[3.1, 3.9]) (p <0.001). The overall nodule detection rates for ULDCT-ASiR-V and ULDCT-DLIR were 82.1% (380/463) and 87.0% (403/463), respectively (p <0.001). The percentage difference between diameters >1 mm was 2.9% (ULDCT-ASiR-V vs. LDCT) and 0.5% (ULDCT-DLIR vs. LDCT) (p =0.009). Scores of nodule imaging sharpness on ULDCT-DLIR (4.0 ± 0.68) were significantly higher than those on ULDCT-ASiR-V (3.2 ± 0.50) (p <0.001).
**Conclusion:** DLIR-based image domain improves image quality, nodule detection rate, nodule imaging sharpness, and nodule measurement accuracy of ASiR-V on ULDCT.
**Limitations:** Only ASiR-V algorithm was evaluated, and more different IR algorithms should be included in the future.
**Funding for this study:** With improved image quality and a higher nodule detection rate, deep learning-based image domain reconstruction is beneficial in facilitating the use of ultralow-dose CT in lung cancer screening.
**Ethics committee - additional information:** Peking university third hospital review board
**Author Disclosures:**
Huishu Yuan: Nothing to disclose
Kai Ye: Nothing to disclose
Boyang Pan: Nothing to disclose
Jie Li: Nothing to disclose
Zhenglin Pan: Nothing to disclose
Nan-Jie Gong: Nothing to disclose

**16:00-17:30** **Research Stage 2**

### Research Presentation Session: Musculoskeletal

### RPS 1610

### Imaging of the peripheral joints: shoulder to foot

**Moderator**
A. Serfaty; Cabo Frio, Rio de Janeiro/BR
*(alineserfaty@gmail.com)*

**Relationship Between Coraco-Glenoid Ligament Variants and SLAP Lesions: Insights from a 3-T Arthro-MRI Study**

*M. Curti*, A. Cozzi, V. Chianca, S. Rizzo, F. Del Grande; Lugano/CH
*(curti.marco.33@gmail.com)*

**Purpose or Learning Objective:** The insertion site of the coraco-glenoid ligament (CGL) of the shoulder seems to be involved in superior labrum anterior and posterior (SLAP) lesions. Anatomical and arthro-magnetic resonance imaging (MRA) studies have reported five CGL variants. This study aimed to evaluate inter-reader reliability in the identification of CGL presence and variants on 3-T MRA, also investigating potential associations between CGL variants and SLAP lesions.
**Methods or Background:** In this retrospective cohort study, three board-certified musculoskeletal radiologists (20, 10, and 5 years of experience) evaluated 1136 consecutive MRA examinations, 577 (50.2%) of patients with SLAP lesions. Inter-reader reliability in assessing CGL presence and variants was evaluated with Fleiss' κ. After 1:1 patient matching according to age and sex, the association between the presence of SLAP lesions and CGL variants was evaluated with binary logistic regression, calculating odds ratios (ORs) and their 95% confidence intervals (CI).
**Results or Findings:** "Almost perfect" reliability was found for the identification of CGL variants (κ 0.832, 95% CI 0.813–0.851), the most frequent being type I (393/1136 patients, 34.6%), and type II (320/1136 patients, 28.2%). 1:1 matching resulted in two groups of 427 patients with and without SLAP lesions (256 males in each group; median age 52 years, IQR 44–59 years). Among these 854 matched patients, compared to the type I CGL variant, all other CGL variants had high ORs for the presence of SLAP lesions, ranging from OR 4.3 (95% CI 2.5–7.5) of type III to OR 39.9 (95% CI 24.5–65.0) for type II.
**Conclusion:** 3-T MRA grants high reliability in identifying CGL presence and variants. In an age and sex matched comparison, the type II CGL variant was strongly associated with the presence of SLAP lesions.
**Limitations:** Single-center study
**Funding for this study:** None
**Ethics committee - additional information:** Local etichs commitee
**Author Disclosures:**
Andrea Cozzi: Nothing to disclose
Vito Chianca: Nothing to disclose
Stefania Rizzo: Nothing to disclose
Filippo Del Grande: Nothing to disclose
Marco Curti: Nothing to disclose

**CT Findings and the Time-course of Myositis Ossificans in Short Rotator Muscles**

*A. Fujii*, M. Katsumata, C. Sato, S. Tsukahara, T. Wada, M. Yamamoto, H. Kondo, H. Oba, A. Yamamoto; Tokyo, Japan/JP

**Purpose or Learning Objective:** Non-traumatic myositis ossificans (MO) in the pelvic region in patients with neurological disorders are classically reported on radiographs. However, CT findings have been rarely reported and the time-course has not been proven. The purpose is to investigate the patients' background, CT findings and the time-course of MO involving the short external rotator muscles (SERMs) of the hip.
**Methods or Background:** Of patients hospitalized for acute cerebrovascular disease between 2001 and 2023 in our hospital, 1,469 CT scans were performed for 436 patients. Swelling, calcification and ossification of SERMs, evaluated for each hip by two radiologists, were judged as calcification when the average density of the lesion was above 100HU, and as ossification when bone marrow fat was confirmed. Medical charts were reviewed for their clinical course and neurological findings.
**Results or Findings:** SERMs swelling was found in 22 cases, 29 hips (age 61.0±3.0, 9 males, 13 females). Follow-up CT scans were examined in 6 (6/436, 1.4%) (aged 35–72, mean 54.7±12.7, 2 male, 4 female). All showed ipsilateral limb paralysis due to cerebral hemorrhage. Swelling was detected in 8 hips of 6 patients, 4 unilaterally (right:left, 3:1) and 2 bilaterally. Follow-up CT revealed calcification in all swelling lesions in SERMs . The post-onset time-course in 6 hips was 13–36 days for swelling, 23–53 days for calcification, and 49–360 days for ossification. In 2 hips, calcification disappeared on follow-up CT.
**Conclusion:** CT imaging illustrated the drastic changes in the maturation process of SERMs-MO. Understanding the time-course of SERMs-MO is essential in the management, and frequency of follow-up CT for distinguishing neoplasms and infectious conditions.
**Limitations:** This was a retrospective study of a few patients. The follow-up durations varied between patients because examinations of the lesions were not always performed.
**Funding for this study:** No funding was received for this study.
**Ethics committee - additional information:** The study was approved by Teikyo University Medical Research Ethics Committee(23-030).
**Author Disclosures:**
Hiroshi Oba: Nothing to disclose
Satoshi Tsukahara: Nothing to disclose
Megumi Katsumata: Nothing to disclose
Akio Fujii: Nothing to disclose
Asako Yamamoto: Nothing to disclose
Chiaki Sato: Nothing to disclose
Masayoshi Yamamoto: Nothing to disclose
Hiroshi Kondo: Nothing to disclose
Takeshi Wada: Nothing to disclose

**Predicting the Surgical Requirement for Bone Substitutes in Distal Radius Fractures: Evaluation of Dual-Energy-Derived Metrics**

L. D. Grünewald, V. Koch, *S. Mahmoudi*, S. Martin, J-E. Scholtz, I. Yel, C. Booz, T. Vogl; Frankfurt/DE
*(scherwin.mahmoudi@gmail.com)*

**Purpose or Learning Objective:** Distal radius fractures (DRF) are commonly diagnosed in emergency departments. Depending on fracture characteristics and patient factors, bone substitutes may be needed, a decision usually made during surgery. However, preoperative preparations, such as obtaining informed consent, are necessary. This study aimed to evaluate metrics from routine CT scans as surrogates for bone texture to predict bone substitute use in surgery.
**Methods or Background:** Distal radius scans of patients who underwent dual-energy CT (DECT) between 01/2016 and 08/2021 were retrospectively analyzed. Cortical HU, trabecular HU, cortical thickness, and DECT-based bone mineral density (BMD) were measured. Patient records and follow-up images were reviewed to determine bone substitute use during surgery. Receiver-operating characteristic (ROC) analysis identified AUC values for BMD, HU values, and cortical thickness, while logistic regression assessed their association with bone substitute use.
**Results or Findings:** A total of 263 patients (median age, 52 years; 132 women; 192 fractures) were included. ROC analysis showed a higher AUC for DECT-derived BMD compared to cortical HU, trabecular HU, and cortical thickness (0.87 vs. 0.62, 0.52, and 0.60, respectively; $P < .001$). Logistic regression confirmed a significant association between lower DECT-derived BMD and the use of bone substitutes (Odds Ratio, 0.94; $P = .02$), while cortical HU, trabecular HU, and cortical thickness were not significantly associated ($P > .05$).
**Conclusion:** Routine CT scans of the distal radius can predict the use of bone substitutes in DRF surgical management, facilitating preoperative planning. DECT-derived BMD offers superior predictive performance compared to cortical HU, trabecular HU, and cortical thickness.
**Limitations:** Preselection bias, as patients received Xrays before CT.
**Funding for this study:** No funding was received for this study
**Ethics committee - additional information:** Consent waived due to the retrospective nature of the study
**Author Disclosures:**
Simon Martin: Nothing to disclose
Christian Booz: Nothing to disclose
Ibrahim Yel: Nothing to disclose
Thomas Vogl: Nothing to disclose
Jan-Erik Scholtz: Nothing to disclose
Vitali Koch: Nothing to disclose
Scherwin Mahmoudi: Nothing to disclose
Leon David Grünewald: Nothing to disclose

**Evaluation of findings in recalcitrant tennis elbow, to rule out SMILE lesion (Symptomatic Micro-Instability In Lateral Elbow) - a retrospective study:**

*S. Rajan*, J. S. Chatha, H. Mahajan; New Delhi/IN
*(drsriramrajan@gmail.com)*

**Purpose or Learning Objective:** Purpose: Recalcitrant lateral epicondylitis, often presents diagnostic challenges, particularly in ruling out symptomatic micro-instability in the lateral elbow (SMILE lesion). This study aims to evaluate the radiological findings in a retrospective cohort, focusing on defining MRI findings with arthroscopically confirmed SMILE lesions.
**Methods or Background:** Methods: A total of 313 consecutive elbow MRIs referred for tennis elbow of which 7 arthroscopically proven cases of SMILE lesions were identified as test cases, and key imaging findings were documented as a checklist. The remaining 306 cases were randomized and anonymized and analyzed by two experienced radiologists (9 and 22 years of experience). On MR, key findings included lateral ulnar collateral ligament (LUCL) tear, partial tear of the extensor carpi radialis brevis (ECRB), widened ulnotrochlear joint or radiocapitellar posterior shift . Statistical significance of findings was assessed using Chi-square tests for categorical variables and Fisher's exact test for small sample sizes.
**Results or Findings:** Results: Of the 306 evaluated cases, 122 (25.8%) showed partial LUCL tears, 12 (3.9%) had complete LUCL tears, 268 cases (87.5%). had deep surface partial tears of ECRB. Statistical analysis revealed a significant association between the presence of LUCL tears (partial and complete) and abnormal radiocapitellar alignment ($p < 0.01$). Abnormal ulnotrochlear alignment was significantly associated with the presence of ECRB tears ($p < 0.05$). Intra-articular abnormalities included posterior radial synovial plica in 22 cases (7.18%), synovitis in 3 cases (0.98%), and chondral lesions in the radial head in 39.78% of cases, with minimal occurrence of lateral capitellar lesions (0.65%).
**Conclusion:** Conclusions: The study underscores a significant association between MRI findings such as LUCL tears, ECRB injuries, and joint misalignments.
**Limitations:** Arthroscopic proof was not available in the cases. MR arthrography could possibly have helped.
**Funding for this study:** None
**Ethics committee - additional information:** Retrospective study using HIPAA guidelines
**Author Disclosures:**
Jagneet Singh Chatha: Nothing to disclose
Sriram Rajan: Nothing to disclose
Harsh Mahajan: Nothing to disclose

**Detecting and differentiating bone marrow edema in the knee using dual-energy CT with water-calcium and water-hydroxyapatite material decomposition**

W. Xiong[1], J. Han[2], G. Zhang[2], *T. Wang*[2], M. Cheng[1], L. Hu[1], W. Li[1], Z. Ding[1], X. He[1]; [1]Nanchang/CN, [2]Shanghai/CN
*(tiantian.wang@cri-united-imaging.com)*

**Purpose or Learning Objective:** To evaluate the diagnostic performance of dual-energy CT (DECT) with water-calcium and water-hydroxyapatite (HAP) material decomposition for quantitatively detecting and differentiating bone marrow edema (BME) in knee.
**Methods or Background:** This retrospective study included 26 patients who underwent DECT and magnetic resonance imaging (MRI) on the same day for the knee. The DECT images were post-processed to generate water-calcium and water-HAP material decomposed images, on which two readers independently measured the water mass density of edema zone and contralateral normal bone marrow by drawing ellipse regions of interest. The MRI was served as the reference for presence and extent of BME. The edema extent was graded as mild or severe by visual assessment. The diagnostic performance of DECT was evaluated using the receiver operating characteristic (ROC) analysis.
**Results or Findings:** Thirty-two bones were confirmed with BME at MRI, caused by fractures (n=16), arthritis (n=10) and bruises (n=6). The mean water mass density of severe BME, mild BME and normal bone marrow was 1073.9 ± 15.2, 1052.9 ± 13.0, and 1012.5 ± 22.4 mg/cm3 ($p < 0.001$), respectively, on water-calcium images; and 1012.9 ± 15.6, 979.7 ± 23.8, and 959.1 ± 12.8 mg/cm3 ($p < 0.001$), respectively, on water-HAP images. In detecting the presence of BME, the area under the ROC curve (AUC) of water-calcium and water-HAP images was 0.976 and 0.885 ($p = 0.001$), respectively, and the accuracy was 89.1% and 79.7%. In differentiating severe and mild BME, the AUC of the two decomposed images was 0.836 and 0.896 ($p = 0.318$), respectively, with accuracy of 70.3% and 87.5%.
**Conclusion:** Dual-energy CT with water-calcium decomposition showed superior diagnostic performance for quantitatively detecting BME in knee, while water-HAP decomposition showed better performance in differentiating severe and mild BME.
**Limitations:** Not applicable.
**Funding for this study:** No funding was received for this study.
**Ethics committee - additional information:** This study was a retrospective study.
**Author Disclosures:**
Wenfeng Li: Nothing to disclose
Zhichao Ding: Nothing to disclose
Tiantian Wang: Employee: Central Research Institute, United Imaging Healthcare
Xi He: Nothing to disclose
Guozhi Zhang: Nothing to disclose
Wei Xiong: Nothing to disclose
Leiming Hu: Nothing to disclose
Jintao Han: Nothing to disclose
Maochu Cheng: Nothing to disclose

**Synthetic Multiplanar Reformation Generates Quantitatively Comparable Knee MR Images to Standard-Of-Care Acquisitions**

T. C. Arnold, L. Wang, L. Yao, A. Shankaranarayanan, *S. Pasumarthi Venkata*; Menlo Park, CA/US
*(srivathsa@subtlemedical.com)*

**Purpose or Learning Objective:** Knee MRI protocols acquire multiple acquisition planes for three main 2D sequences: T1w, T2w, and PD. However, protocol sequence number is limited by time constraints, meaning some contrast and orientation combinations are not available. Here, we develop a deep-learning-based method to synthesize 2D sequences using complementary contrast and acquisition plane information from two input images, and quantitatively compare synthesized and ground truth acquisitions
**Methods or Background:** We retrospectively analyzed data from 50 knee MRI participants. Each contained three 2D sequences: axial T2 fat-sat, coronal T2 fat-sat, and coronal PD. The coronal T2 fat-sat served as our target sequence and the remaining sequences were our network inputs. All images were coregistered to the target image. Patients were divided into training (N=34),

Friday validation (N=8), and testing (N=8) sets. Quantitative metrics (PSNR, SSIM, and NMI) measured the similarity between the synthesized coronal T2 and acquired images.
**Results or Findings:** The algorithm demonstrated excellent quantitative performance when comparing acquired and synthesized coronal T2 fat-sat images across all three metrics (SSIM: 0.86±0.13, PSNR: 29.98±5.93 dB, and NMI: 1.33±0.10). We also compared the synthesized images to a coronal reformat of the acquired axial T2 fat-sat (SSIM: 0.68±0.22, PSNR: 23.33±5.21 dB, and NMI: 1.21±0.10) to illustrate the significant benefit of our algorithm over simple image reformation (paired t-test, SSIM: p<0.005, PSNR & NMI: p<0.001). Relative to simple reformats, the synthesized image showed a 26% SSIM increase, 29% PSNR increase, and 10% NMI increase. Qualitatively, pathological presentation in the synthesized image was similar to acquired sequences.
**Conclusion:** This study presents a deep-learning-based method to synthesize high quality multiplanar reformations in knee MRI. Synthesized images exhibited strong quantitative similarity to acquired images.
**Limitations:** In future work, these methods should undergo qualitative review by radiologists.
**Funding for this study:** This study was funded by Subtle Medical.
**Ethics committee - additional information:** All data was retrospective and anonymized and therefore not considered human subjects research.
**Author Disclosures:**
Srivathsa Pasumarthi Venkata: Employee: Subtle Medical Inc.
Lanhong Yao: Employee: Subtle Medical
Long Wang: Employee: Subtle Medical
Thomas Campbell Arnold: Employee: Subtle Medical
Ajit Shankaranarayanan: Employee: Subtle Medical

**A three-dimensional magnetic resonance imaging-based scoring system to evaluate continuity, thickness and shape of the injured anterior cruciate ligament**
*N. Giannotti*[1], A. Liu[2], H. Gauffin[2], S. Filbay[3], J. Kvist[2], M. Englund[4]; [1]Sydney/AU, [2]Linköping/SE, [3]Melbourne/AU, [4]Lund/SE
*(nicola.giannotti@sydney.edu.au)*

**Purpose or Learning Objective:** Anterior cruciate ligament (ACL) injuries are common, and ACL reconstruction (ACLR) is considered vital for restoring knee stability. However, recent evidence evaluating non-surgical approaches to the injured ACL challenges these notions. To date, no studies have used three-dimensional magnetic resonance imaging (3D-MRI) to systematically assess morphological changes of the injured ACL at different time-points in non-reconstructed patients. The aim of this study was to develop and investigate the reliability of a semi-quantitative scoring system to assess morphological changes of the injured ACL using 3D-MRI.
**Methods or Background:** Baseline and 4 follow-ups within 2 years 3T 3D-MRI scans were acquired with an isotropic proton-density fat-saturated sequence. Scans were censored from further analysis after ACLR. 3D-MRI data were reconstructed on oblique coronal parallel to ACL, sagittal, and axial planes. Unblinded for time sequence, two readers independently scored the MRI data from all available time-points. The intra-rater reliability was measured six months after the initial evaluation. Weighted kappa (κw) was used to calculate inter- and intra-reader reliability.
**Results or Findings:** 129 patients (46% female; 25 SD [7] years) with non-surgically treated ACL injury from the NACOX study were included. The scoring system developed assessed the following ACL 3D-MRI features: overall structure, fiber continuity, thickness, and shape. The injury location was scored at baseline. 2,430 gradings were collected in total. The averaged inter-reader reliability (κw) was 0.738, 0.673, 0.710 and 0.539 for ligament structure, fiber continuity, thickness and shape, respectively. The intra-rater reliability (κw) ranged from 0.409 to 0.873.
**Conclusion:** We introduce a novel 3D-MRI-based semi-quantitative scoring system to enable assessment of the acutely injured ACL and its morphological changes over time with acceptable inter-reader reliability. Future validation vs findings from knee arthroscopy will be required.
**Limitations:** No limitations were identified.
**Funding for this study:** The NACOX study received funding from the Swedish Research Council, Medical Faculty of Linköping University, Swedish National Centre for Research in Sports, Medical Research Council of Southeast Sweden (FORSS), ALF Region Östergötland
**Ethics committee - additional information:** Ethical approval for the study was granted by the Swedish Ethical Review Authority, Dnr: 2016/44/31.
**Author Disclosures:**
Håkan Gauffin: Nothing to disclose
Nicola Giannotti: Nothing to disclose
Joanna Kvist: Nothing to disclose
Stephanie Filbay: Nothing to disclose
Martin Englund: Nothing to disclose
Angie Liu: Nothing to disclose

**The posterior cruciate ligament angle in the setting of deficient anterior cruciate ligament deficient knees: the effect of gender, age, time from injury and tibial slope**
*M. V. Bausano*[1], F. Di Maria[2], R. D'Ambrosi[1], L. M. Sconfienza[1], S. Fusco[1], E. Abermann[3], C. Fink[3]; [1]Milan/IT, [2]Catania/IT, [3]Innsbruck/AT
*(mariavittoriabausano@gmail.com)*

**Purpose or Learning Objective:** The aim was to assess the posterior cruciate ligament (PCL) angle in anterior cruciate ligament deficient knees and correlate it with anatomic and demographic factors.
**Methods or Background:** Patients included were initially noted to have an ACL tear clinically as confirmed by MRI.For each patient were evaluated:PCL angle (PCLA), medial tibial slope (MTS), lateral tibial slope (LTS), medial anterior tibial translation (MATT) and lateral anterior tibial translation (LATT). Age, sex, and time from injury to MRI were manually recorded.Age groups were predefined dichotomizing age at its mean value while for time interval from injury to MRI the cut-off of 90 days was selected to differentiate between chronic and acute lesions. Differences by groups were assessed with t-test or a Wilcoxon-Mann Whitney test, according to score distribution. Spearman rank correlations were also estimated to explore correlation among collected variables.
**Results or Findings:** A total of 193 patients were included in the study of which 91 females and 102 males with a mean age of 30.27±12.54.The mean time from injury to MRI was 14.18±55.77 days.On overall population, mean PCLA resulted 128.72±10.33°, mean MTS 3.57±2.33, mean LTS 6.07±3.52, mean MATT and LATT were respectively 4.76±2.02 and 7.01 ±2.48 mm. In 190 cases PCLA angle was≥to 105° and only in 3 was inferior.PCLA negatively correlated with medial and lateral anterior tibial translation(p<0.05).Female showed a higher PCLA compared to male (130.55±10.23 vs 127.08±10.20; p=0.019).Patients with chronic ACL injury showed a lower value of PCLA compared to patients with acute injury(p=0.032) and a superior grade of LATT(p=0.015).
**Conclusion:** In the setting of ACL lesions, PCLA has normal value in acute injury and decrease over the time. PCLA is negatively correlated with anterior tibial translation and female have higher PCLA compared to male.
**Limitations:** First, the diagnosis was made exclusively by MRI analysis, second the data excluded partial ACL injuries.
**Funding for this study:** N/A
**Ethics committee - additional information:** After Institutional Review Board approval (ACL-L2104), two of the authors reviewed the MR images of all patients with a clinical diagnosis of acute ACL injury. The present study was conducted following the Strengthening the Reporting of Observational Studies in Epidemiology (STROBE) statement [3]. All procedures were conducted in accordance with the standards highlighted in the 1964 Helsinki Declaration and its later amendments.
**Author Disclosures:**
Elisabeth Abermann: Nothing to disclose
Fabrizio Di Maria: Nothing to disclose
Stefano Fusco: Nothing to disclose
Luca Maria Sconfienza: Nothing to disclose
Riccardo D'Ambrosi: Nothing to disclose
Maria Vittoria Bausano: Nothing to disclose
Christian Fink: Nothing to disclose

**Diminished Meniscal Height as an Indicator of Synovitis on Magnetic Resonance Imaging of the Knee**
S. N. Yılmazer Zorlu, *N. A. Ahmady*, K. B. Karaca, Z. Akkaya; Ankara/TR
*(nesarahmad.ahmadi123@gmail.com)*

**Purpose or Learning Objective:** To investigate the relationship between meniscal height and adjacent synovitis on magnetic resonance images (MRI) of the knee, based on the hypothesis that, in contrast to simple effusion, hypertrophic synovial tissue will cause compressive distortion on neighboring meniscus.
**Methods or Background:** Contrast-enhanced knee MRIs from patients (≥18years), acquired during 2014-2024, at a single university hospital were included in this cross-sectional, retrospective study. Patients with history of knee surgery, acute trauma, local and systemic malignancy were excluded. On post-contrast fat-suppressed(fs)-T1-weighted images, 2 independent observers assessed the presence of synovitis (synovial enhancement ≥2mm) adjacent to the medial meniscus body-posterior horn (MMB, MMPH) and lateral meniscus body-posterior horn (LMB, LMPH). Blinded to the synovitis status, meniscal heights were measured on coronal and sagittal fs-fluid sensitive images where menisci were intact and showed < grade 2 degeneration. The relationship between meniscal height and synovitis was evaluated using linear regression models adjusted for age, gender and body mass index (BMI). Intra-class correlations (ICC) were used to test the reproducibility of meniscal height measurements across 10 randomly selected patients.
**Results or Findings:** A total of 129 patients (85 women, mean age=46.6±15years, mean BMI=24.6±5.1 kg/m2) (119 patients on 1.5T; 10 patients on 3.0T systems; ranges of slice thickness=3.5-6mm, gap=0.3-1mm,

Friday

FOV=12-16 cm) were included. Mean heights of MMB, MMPH, LMB, LMPH were 5.5±1mm, 5.3±1.1mm, 5.9±0.9mm, 5.6+0.8mm, respectively. MMB, MMPH and LMPH were significantly thinner adjacent to synovitis regions(β=-0.9, [95%CI= -1.34, -0.44], p<0.001; β=-1.26, [95%CI=-1.79, -0.73], p<0.001; β=-0.7, [95%CI= -1.12, -0.29], p=0.001, respectively). ICC ranged between 0.96 - 0.99 (p<0.001), indicating excellent inter-rater agreement.
**Conclusion:** Diminished meniscal height adjacent to high-signal areas on fluid-sensitive images may help distinguish effusion from synovitis on clinical knee MRIs.
**Limitations:** The BMI values were not available in 25 patients.
**Funding for this study:** None
**Ethics committee - additional information:** Ethics Committee Approval Number: I03-251-24
**Author Disclosures:**
Kazım Burak Karaca: Nothing to disclose
Sezer Nil Yılmazer Zorlu: Nothing to disclose
Nesar Ahmad Ahmady: Nothing to disclose
Zehra Akkaya: Nothing to disclose

**The precision of a novel method for automated CT-based radio-stereometric analysis in evaluating tibial implant migration**

*M. Acke*[1], B. Keelson[1], L. H. W. Engseth[2], G. Van Gompel[1], F-D. Ohrn[3], J. De Mey[1], A. Schulz[2], S. M. Röhrl[2], N. Buls[1]; [1]Brussels/BE, [2]Oslo/NO, [3]Kristiansund/NO
*(manou.acke@gmail.com)*

**Purpose or Learning Objective:** The current gold-standard for implant migration analysis is radiostereometric analysis (RSA), which is a time-consuming and resource demanding method. We aim to evaluate the precision of a novel non-invasive method using automated CT-based radio-stereometric analysis (CT-RSA) for tibial implant migration analysis in a porcine model.
**Methods or Background:** A porcine knee cadaver with a tibial implant was examined with marker-based RSA and computed tomography (CT). RSA acquisitions (133kV, 6.3mAs) and CT images (120kV, 100mAs, 0.625mm, 0.5s, FOV 200×200mm) were acquired in seven different positions (P1,…,P7) of the cadaveric knee. To obtain enough statistical power, the 7 positions were compared to each other, resulting in 21 double examinations (P1_P2,…,P6_P7) for each method. Post-processing of the CT data was performed by a novel in-house built automatic image processing pipeline using SimpleITK. The reference standard was zero implant motion. Maximum-Total-Point-Motion (MTPM) was calculated for RSA to identify the implant point with maximum translation. Similarly, from the CT-RSA data we calculated Total-Translation (TT) for 6 non-invasive, virtual landmarks on the implant, including the centre-of-mass and 5 peripheral landmarks. In addition, we computed a virtual implant mesh from the CT data, including its displacement. For RSA and CT-RSA, displacement differences towards the reference-standard were assessed using a two-sample t-test.
**Results or Findings:** The precision for MTPM using marker-based RSA was 0.45mm (95%CI 0.19–0.70mm). TT calculation for virtual landmarks using CT-RSA was more precise than RSA (p<0.001) with 0.15mm (95%CI 0.12–0.18mm). The mesh data allowed to identify the point of maximum translation with a displacement of 0.16mm (95%CI 0.13–0.19mm).
**Conclusion:** Compared to RSA, the novel automated CT-RSA shows improved precision in analysing tibial implant migration for a porcine cadaver.
**Limitations:** This is a porcine phantom-study, which limits generalizability and results may differ in humans.
**Funding for this study:** Funding is provided by the UZBrussels, Belgium and CIRRO, Norway.
**Ethics committee - additional information:** The study was approved by the local research committee at Oslo University Hospital on December 13, 2021. No consent was necessary since exams were performed on a porcine cadaver phantom.
**Author Disclosures:**
Stephan Maximilian Röhrl: Nothing to disclose
Manou Acke: Nothing to disclose
Johan De Mey: Nothing to disclose
Lars Harald William Engseth: Nothing to disclose
Gert Van Gompel: Nothing to disclose
Anselm Schulz: Nothing to disclose
Nico Buls: Nothing to disclose
Frank-David Ohrn: Nothing to disclose
Benyameen Keelson: Nothing to disclose

**How Cone-Beam CT affects Clinical Management in Foot Trauma**

*E. Mcdermott*, D. P. Moloney, S. Murphy, B. Gibney, P. J. Macmahon, E. Kavanagh; Dublin/IE
*(edwardmcdermott@gmail.com)*

**Purpose or Learning Objective:** Foot trauma is a common presentation to the emergency department. In our institution Cone Beam Computed Tomography (CBCT) is routinely utilized in patients with suspected fractures with negative radiographs. Previous studies have investigated the role of CBCT in the acute trauma setting for wrist and ankle injuries, but no large study has been performed for foot trauma. Our study focuses on how CBCT can improve the diagnosis of foot fractures in a trauma setting and, with the input of orthopaedic surgery, how it can alter the patient's treatment plan.
**Methods or Background:** Patients with foot trauma who underwent CBCT between 2019 and 2023 were reviewed. In cases of discordance between CBCT and X-rays, images were assessed with an orthopaedic surgeon. Patients were classified into three treatment options based on X-rays and CBCT: No Treatment, Immobilisation, and Surgery.
**Results or Findings:** 204 trauma patients had foot X-rays and CBCT. In 102 cases, CBCT identified 164 additional fractures, leading to treatment changes in 62 cases (p < 0.01). 42 changed from 'No Treatment' to 'Immobilisation,' 3 from 'No Treatment' to Surgery,' and 3 from 'Immobilisation' to 'Surgery.' 12 were downgraded from 'Immobilisation' to 'No Treatment,' and 2 from 'Surgery' to 'Immobilisation'.
**Conclusion:** Our findings show that a large number of foot trauma goes un-diagnosed on radiographs. The involvement of orthopaedic surgery in reviewing the radiographs and CBCT to assess how treatment plans may change is a novel approach. We found that a significant number of patients would have their type of treatment altered with the use of CBCT. Our findings show the significant role CBCT can have when integrated into trauma care.
**Limitations:** Only a single centre was used for data collection. Reporting of initial radiographs by non-musculoskeletal specialised radiologists.
**Funding for this study:** None.
**Ethics committee - additional information:** Project approved by the Mater Misericordiae University Hospital's Clinical Audit and Effectiveness Committee.
**Author Disclosures:**
Peter Joseph Macmahon: Nothing to disclose
Darren Patrick Moloney: Nothing to disclose
Brian Gibney: Nothing to disclose
Edward Mcdermott: Nothing to disclose
Eoin Kavanagh: Nothing to disclose
Sophie Murphy: Nothing to disclose

**16:00-17:30** **Research Stage 3**

### Research Presentation Session: Imaging Informatics and Artificial Intelligence

### RPS 1605

### Artificial intelligence: real world results and large European projects

**Moderator**
L. Martí-Bonmatí; Valencia/ES
*(marti_lui@gva.es)*

**Assessing the effectiveness of artificial intelligence (AI) in prioritising CT Head interpretation: a stepped-wedge cluster-randomised trial (ACCEPT-AI)**

*K. Nash*[1], K. Vimalesvaran[2], R. Dharmadhikari[3], M. Hall[4], A. Novak[1], S. Ather[1], D. J. Lowe[4], H. Shuaib[2], Accept-Ai Investigators[2]; [1]Oxford/UK, [2]London/UK, [3]Northumberland/UK, [4]Glasgow/UK
*(katrinanash649@outlook.com)*

**Purpose or Learning Objective:** To evaluate the effectiveness of artificial intelligence (AI) in prioritisation of non-contrast head computed tomography scan (NCCT). We evaluated; 1) whether there was a reduction in report turnaround time (TAT) of prioritised NCCT, 2) the accuracy of the AI algorithm, and 3) the technical performance of the algorithm.
**Methods or Background:** This large-scale multi-centre trial has been conducted across three emergency departments between November 2023 to July 2024. Individuals above the age of 18 who presented to the emergency department and underwent NCCT were included. Data collected included demographic data, time from acquisition to report of CT scan, referral, and discharge, and death within 28 days. Findings were categorised into prioritised findings (intracranial haemorrhage, mass effect, fracture), non-prioritised findings (atrophy and infarct), and no prioritised findings. The study was conducted in 3 stages; pre-implementation, implementation and post-implementation. Baseline data was collected during the pre-implementation phase, whilst the implementation phase enabled training and integration of the AI tool into radiology workflow at each trust. During the post-implementation phase, AI results were visible to radiologists. Radiology reports were coded to assess AI accuracy, with discrepant cases sent for ground truthing by two independent radiologists.
**Results or Findings:** 7,500 scans from the pre-implementation phase, and 8,453 scans from the post-implementation phase have been included. The median TAT (minutes) for prioritised scans in the pre-implementation was 34 (inter-quartile range 20-55) and 35 in the post-implementation phase (22-59).
**Conclusion:** We have successfully conducted a prospective trial of AI implementation across multiple centres in the United Kingdom. Initial results do not show a difference in TAT for NCCT reports after implementation of AI.
**Limitations:** TAT has been calculated during preliminary analysis of a larger dataset, therefore full results will be available for presentation.
**Funding for this study:** This study has been awarded funding from NHSx AI Award (Award reference: AI_Award02354).
**Ethics committee - additional information:** The study protocol was approved by the Research Ethics Committee (REC) of East Midlands (Leicester Central), in May 2023 (REC 23/EM/0108) and was conducted in accordance with the principles of Good Clinical Practice.
**Author Disclosures:**
Haris Shuaib: Nothing to disclose
Rahul Dharmadhikari: Nothing to disclose
Accept-Ai Investigators: Nothing to disclose
Katrina Nash: Nothing to disclose
Alex Novak: Nothing to disclose
Sarim Ather: Shareholder: Share holder RAIQC Ltd
Kavitha Vimalesvaran: Nothing to disclose
Mark Hall: Nothing to disclose
David J. Lowe: Nothing to disclose

### ONCOPILOT: A Promptable CT Foundation Model For Solid Tumor Evaluation

*L. Machado*[1], H. Philippe[2], E. Ferreres[2], J. Khlaut[2], J. Gregory[1], M. Ronot[1], D. Tordjman[2], P. Manceron[2], P. Herent[2]; [1]Clichy/FR, [2]Paris/FR
*(dr.machado.leo@gmail.com)*

**Purpose or Learning Objective:** Carcinogenesis leads to tumors with diverse shapes and behaviors. Although RECIST 1.1 remains the standard for evaluation, its reliance on linear measurements and high inter-reader variability often result in misclassification. Volumetric biomarkers, such as total tumor burden, provide more information but need automated segmentation. Traditional segmentation models struggle with complex lesions, have a narrow focus, and lack interactivity. Foundation models with transformer architecture address these issues through zero-shot learning and visual prompts (point-click, bounding box). We developed ONCOPILOT, a foundation model that enhances RECIST 1.1 measurements, enabling volumetric analysis while integrating seamlessly into radiology workflows.
**Methods or Background:** ONCOPILOT was trained on 7,500 CT scans, including normal anatomy and oncological cases. Its segmentation performance was assessed against nnUnet, a state-of-the-art baseline, using the DICE coefficient. The evaluation also included comparisons with radiologists for RECIST 1.1 long-axis measurements, annotation speed, and inter-reader variability.
**Results or Findings:** ONCOPILOT outperformed state-of-the-art models, achieving a mean DICE of 0.78 post-editing versus 0.70 for the baseline. Its RECIST error (7.4%, 1.1 mm) was not significantly different to radiologists' (8.6%, 1.3 mm). ONCOPILOT-assisted measurements were quicker than manual (17.2 vs. 20.6 seconds, $p < .05$), with reduced inter-reader variability (1.7 vs. 2.4 mm, $p < .05$).
**Conclusion:** ONCOPILOT is among the first foundation model applications in radiology, acting as an interactive AI assistant for oncological evaluation. It improves RECIST reproducibility, facilitates access to volumetric biomarkers, and seamlessly integrates into radiology workflows, reducing inter-reader variability and measurement time. This approach offers substantial potential to advance oncology research and enhance clinical care.
**Limitations:** ONCOPILOT showed reduced performance on small tumors, particularly lung lesions, which were overrepresented in the test set. Future iterations should use a more balanced dataset and cover a broader range of tumor types.
**Funding for this study:** This work was granted access to the HPC resources of IDRIS under the allocation 2024-AD011013489R2 made by GENCI.
**Ethics committee - additional information:** The data used is anonymous and publically available
**Author Disclosures:**
Julien Khlaut: Employee: Raidium
Jules Gregory: Nothing to disclose
Pierre Manceron: Founder: Raidium
Maxime Ronot: Nothing to disclose
Léo Machado: Employee: Raidium
Paul Herent: Founder: Raidium
Elodie Ferreres: Employee: Raidium
Hélène Philippe: Employee: Raidium
Daniel Tordjman: Employee: Raidium

### Evaluating artificial intelligence for lung cancer detection on chest radiographs: multi-vendor comparison of diagnostic accuracy in a real-world UK population

*A. Maiter*, P. Metherall, J. Taylor, S. Matthews, K. Hocking, E. Burton, E. Anderson, A. Swift, C. S. Johns; Sheffield/UK

**Purpose or Learning Objective:** Automated detection of lung cancer on chest radiographs by AI could streamline diagnostic pathways. This study evaluated the diagnostic accuracy of commercially available software from seven vendors using a large dataset of radiographs from a real-world UK population.
**Methods or Background:** Consecutive chest radiographs obtained at our tertiary UK centre were retrospectively identified. Chest radiographs requested from primary care for adult patients, regardless of indication, were eligible for inclusion. Software from the seven vendors evaluated each radiograph independently. The radiologist report for each radiograph was also interrogated. Diagnostic accuracy metrics were determined by comparing the software outputs and radiologist reports against the diagnosis of lung cancer by multidisciplinary team decision.
**Results or Findings:** 5,722 chest radiographs were included from 5,592 patients (median age 59 years, 54% female, 79% white, 1.6% prevalence of lung cancer). The software yielded the following (mean±SD): sensitivity 46±9%, specificity 95±4%, positive predictive value (PPV) 15±5%, negative predictive value (NPV) 99±0%, accuracy 70±4% and false positive per image rate (FPPI) 0.05±0.04. Radiologist reports yielded the following: sensitivity 66%, specificity 98%, PPV 36%, NPV 99%, accuracy 82% and FPPI 0.02.
**Conclusion:** All software demonstrated high specificity and NPV comparable to radiologists, and could add value by helping to exclude lung cancer. However, all software also showed lower sensitivity and PPV than radiologists, suggesting that they could increase the number of false negative and false positive results. While AI has potential to improve the efficiency of “straight to CT” and other diagnostic pathways for suspected lung cancer, the risks of diagnostic errors require careful consideration.
**Limitations:** This study used a retrospective dataset from a single centre. Further testing of software performance in multi-centre patient cohorts and evaluation of downstream impacts are essential prior to clinical deployment.
**Funding for this study:** This study was funded by the NHS South Yorkshire Integrated Care System.
**Ethics committee - additional information:** This study received local research ethics committee approval (23/EM/0186). The need for dedicated patient consent was waived.
**Author Disclosures:**
Suzanne Matthews: Nothing to disclose
Jonathan Taylor: Nothing to disclose
Katie Hocking: Nothing to disclose
Emily Burton: Nothing to disclose
Andrew Swift: Nothing to disclose
Ahmed Maiter: Other: Bayer
Chris S Johns: Nothing to disclose
Elizabeth Anderson: Nothing to disclose
Peter Metherall: Nothing to disclose

### Evaluation of AI-assisted Diagnosis of Clinically Significant Prostate Cancer on MRI at Scale: Preliminary Findings from the PI-CAI Consortium

*J. J. Twilt*[1], A. Saha[1], J. S. Bosma[1], D. Yakar[2], M. Elschot[3], J. Veltman[4], J. Fütterer[1], H. E. Huisman[1], M. De Rooij[1]; [1]Nijmegen/NL, [2]Groningen/NL, [3]Trondheim/NO, [4]Almelo/NL
*(jasper.twilt@radboudumc.nl)*

**Purpose or Learning Objective:** To assess whether utilizing an internationally validated prostate artificial intelligence (AI) system enhances the accuracy of prostate MRI evaluations in diagnosing clinically significant prostate cancer (csPCa; Gleason Grade ≥2), compared to non-assisted assessments, in a comprehensive international reader study.
**Methods or Background:** In this retrospective study, imaging and a prostate AI system developed and benchmarked on 10,207 examinations through an international confirmatory study (PI-CAI) were used. A total of 780 biparametric prostate MRI examinations (2015-2021) from men suspected of csPCa, with diagnostic-sufficient image quality and no prior csPCa findings or treatment, were included. Reference was established through histopathology and ≥3

years of follow-up. The AI was calibrated to generate patient-level csPCa suspicion scores (0-10) and associated lesion-detection maps using 420 examinations from three Dutch centers. The remaining 360 examinations (three Dutch, one Norwegian center) were used for outcome analysis. Sixty-one readers (53 centers, 17 countries) provided PI-RADS 3-5 annotations and patient-level csPCa suspicion scores (0-100) with and without AI assistance in two phases, separated by a 4-week washout period. Multi-reader, multi-case analysis compared diagnostic outcomes at a per-patient level.
**Results or Findings:** Preliminary analysis of 22 readers (1-13 years of prostate MRI experience) demonstrated that AI assistance improved csPCa diagnosis, with AUROCs of 0.919 (95% CI: 0.896-0.942) compared to 0.870 (95% CI: 0.831-0.908) without assistance. Sensitivity and specificity at PI-RADS ≥3 improved from 94.1% to 96.5% and 46.6% to 49.1%, respectively.
**Conclusion:** AI assistance improves csPCa diagnosis and holds promise for improving clinical outcomes. Further research is needed to confirm generalizability and assess workflow efficiency.
**Limitations:** Preliminary findings limit the sample size and subgroup analysis considering reader expertise. Reuse of data may introduce generalization bias, and AI was not used to guide histologic verification.
**Funding for this study:** EU Horizon 2020: ProCAncer-I (grant number 952159), Health~Holland (grant number LSHM20103).
**Ethics committee - additional information:** Retrospective use of anonymous patient data was approved by institutional or regional review boards at each contributing center (identifiers: REK 2017/576; CMO 2016-3045; IRB 2018-597; ZGT23-37), and was conducted in accordance with the principles of the Declaration of Helsinki. Informed consent was waived.
**Author Disclosures:**
Jurgen Fütterer: Grant Recipient: Siemens Healthineers
Joeran Sander Bosma: Nothing to disclose
Henkjan En Huisman: Grant Recipient: Siemens Healthineers Grant Recipient: Canon
Anindo Saha: Speaker: Guerbet
Matthijs Elschot: Nothing to disclose
Derya Yakar: Nothing to disclose
Jeroen Veltman: Nothing to disclose
Maarten De Rooij: Nothing to disclose
Jasper Jonathan Twilt: Nothing to disclose

**The PANORAMA study results: Pancreatic Cancer Diagnosis - Radiologists meet AI**

*M. Schuurmans*, N. Alves, P. Vendittelli, G. Litjens, J. J. Hermans, H. E. Huisman; Nijmegen/NL
*(megan.schuurmans@radboudumc.nl)*

**Purpose or Learning Objective:** The PANORAMA study transparently evaluates radiologists and AI in detecting pancreatic ductal adenocarcinoma (PDAC) using contrast-enhanced computed tomography (CECT).
**Methods or Background:** This retrospective study includes 3338 abdominal CECTs of patients without prior history of treatment or positive histopathology findings of PDAC acquired between 2006 and 2021 from 5 centers (Netherlands, Norway, and Sweden). Of these, 2238 cases (676 PDAC) are publicly available to develop and train AI algorithms, and 100 and 1000 cases were sequestered for AI tuning and testing, respectively. The test set comprises data from two external centres not present in the other cohorts. A subset of 400 testing cases is used for the PANORAMA reader study. Both AI and radiologists indicate PDAC likelihood and localization of lesions. Additionally, radiologists provide a newly introduced PANC-RADS score to assess the urgency of expert referrals. AI is openly developed and evaluated using common metrics through the Grand Challenge platform. Patient-level performance and lesion localization performance are assessed using the area under the receiver operating characteristic curve (AUROC) and average precision (AP), respectively.
**Results or Findings:** The PANORAMA reader study results will be presented for the first time at ECR 2025. Currently, 57 radiologists (39 centers,13 countries, 2-30 years of experience, median: 9 years) participate in the study. The baseline AI algorithm (nnU-Net with cross-entropy loss) achieves 0.9776 AUROC and 0.7226 AP in the tuning set. There are currently 165 registered AI challenge participants across 11 teams.
**Conclusion:** Transparently benchmarked AI can enable early PDAC detection at the expert-radiologist level.
**Limitations:** While histopathology and follow-up were considered as the reference standard for the sequestered testing set, we could not guarantee this level of evidence for all cases in the public training set.
**Funding for this study:** This project has received funding from the European Union's Horizon 2020 research and innovation program under grant agreement No 101016851, project PANCAIM.
**Ethics committee - additional information:** Writen ethics committee approval was obtained from every center participating in the study.
**Author Disclosures:**
Natalia Alves: Nothing to disclose
Henkjan En Huisman: Nothing to disclose
Megan Schuurmans: Nothing to disclose
Pierpaolo Vendittelli: Nothing to disclose
John J Hermans: Nothing to disclose
Geert Litjens: Nothing to disclose

**Development and Implementation of a Web-Based Platform for Clinical Validation of Artificial Intelligence Models in Five Cancer Types: An Initiative of the European CHAIMELEON Project**

*A. Galiana-Bordera*, J. Aquerreta-Escribano, P. M. Martínez Gironés, P. Lozano, G. Ribas, P. Jimenez, L. Cerda Alberich, I. Blanquer, L. Marti-Bonmati; Valencia/ES
*(adrian_galiana@iislafe.es)*

**Purpose or Learning Objective:** This study evaluates AI-based predictive oncology models for prostate, lung, breast, and colorectal cancers through the creation of an innovative web-platform. The goal is to bridge the gap between AI development and clinical implementation, advancing AI adoption in healthcare as part of the European CHAIMELEON project.
**Methods or Background:** We developed a web-platform with a microservices architecture, including a REST API, ORTHANC PACS for image management, and Keycloak security. The frontend is a custom-built application serving as a control panel for users. It allows clinicians to open a patient-specific DICOM viewer alongside clinical information, AI predictions, and validation buttons. This interface facilitates a three-stage case review: standard evaluation, AI-aided assessment, and final comparison with ground truth. AI models, developed through an open challenge using anonymized data, were integrated into the platform.
**Results or Findings:** The platform demonstrates efficiency, customizability, and scalability, enhancing oncological study reproducibility. It creates an environment where medical images, clinical data, and AI predictions coexist, adapting to various clinical and research settings. Over 70 clinicians are using the system to evaluate more than 2000 patients, with 60% reviewed in less than a month. This rapid adoption highlights the platform's user-friendly design and potential for improving clinical workflow.
**Conclusion:** This platform represents a significant advancement in validating and adopting AI models in oncology. It provides a foundation for integrating AI into clinical environments, benefiting both clinicians and patients. The study highlights the importance of user-friendly interfaces and structured evaluation processes in bridging the gap between AI development and clinical application.
**Limitations:** The study's retrospective nature limits long-term outcome assessment. Further research is needed on implementation across diverse hospital infrastructures, the learning curve for clinicians, and ethical and regulatory aspects of AI integration in clinical practice.
**Funding for this study:** This particular study did not receive direct funding. However, it was developed as part of the CHAIMELEON project, which received funding from the European Union's Horizon 2020 research and innovation program under Grant Agreement No. 952172.
**Ethics committee - additional information:** Under the CHAIMELEON project, all necessary ethical committee approvals have been obtained for the use of medical images and the development of artificial intelligence models. These approvals cover the collection, anonymization, and utilization of patient data for research purposes. Furthermore, the web platform developed for evaluating these AI models has been registered and approved for use in clinical settings. This ensures compliance with data protection regulations and ethical standards in medical research. The project adheres to strict protocols for data handling and user access, maintaining patient privacy and data integrity throughout the study.
**Author Disclosures:**
Pau Lozano: Nothing to disclose
Ignacio Blanquer: Nothing to disclose
Luis Marti-Bonmati: Nothing to disclose
Paula Jimenez: Nothing to disclose
Javier Aquerreta-Escribano: Nothing to disclose
Gloria Ribas: Nothing to disclose
Pedro Miguel Martínez Gironés: Nothing to disclose
Leonor Cerda Alberich: Nothing to disclose
Adrian Galiana-Bordera: Nothing to disclose

**Hospital patients' attitudes towards AI worldwide: Results from the COMFORT study in 74 hospitals and 43 countries**

*F. Busch*[1], L. Hoffmann[2], L. Xu[2], L. Zhang[3], L. Saba[4], M. R. Makowski[1], H. Aerts[5], L. C. Adams[1], K. Bressem[1]; [1]Munich/DE, [2]Berlin/DE, [3]Nanjing/CN, [4]Cagliari/IT, [5]Boston, MA/US
*(felix.busch@tum.de)*

**Purpose or Learning Objective:** Too often, we see healthcare technology implementations that focus only on the clinician's point of view and undervalue the patient's perspective. Given the exponential rise in artificial intelligence (AI) applications in healthcare, this international, multicentre, cross-sectional study aimed to assess hospital patients' attitudes towards AI in healthcare worldwide.
**Methods or Background:** The present COMFORT study, involving 74 network hospitals in 43 countries, employed a quantitative 26-item instrument available in 26 languages on-site between February and November 2023.
**Results or Findings:** 13806 patients from Europe (41.7%, n=5764/13806), Asia (25.2%, n=3473/13806), North America (16.5%, n=2284/13806), South America (9.7%, n=1336/13806), Africa (5.3%, n=728/13806) and Oceania (1.6%, n=221/13806) were included. Overall, 57.6% of respondents were positive about the use of AI in healthcare. Significant differences in attitudes were observed based on demographic characteristics, health status and technological literacy. Female participants and those in poorer health had less positive attitudes towards the use of AI in medicine. Conversely, higher levels of AI knowledge and frequent use of technological devices were associated with more positive attitudes. Notably, less than half of the participants expressed positive attitudes to all items related to trust in AI, with the lowest level of trust being in the accuracy of AI in providing information about treatment response. Patients showed a strong preference for explainable AI and clinician-led decision-making, even if this meant a slight compromise in accuracy.
**Conclusion:** This large-scale, multinational study provides a comprehensive perspective on patient attitudes towards AI in healthcare across six continents. The findings suggest the need for tailored AI implementation strategies that account for patient demographics, health status, and preferences for explainable AI and physician oversight. All study data has been made publicly available to encourage replication and further research.
**Limitations:** Non-probability sampling
**Funding for this study:** This research is funded by the European Union (101079894).
**Ethics committee - additional information:** Ethical approval was obtained from Charité – Universitätsmedizin Berlin (EA4/213/22), which served as the lead institution.
**Author Disclosures:**
Lena Hoffmann: Nothing to disclose
Marcus R. Makowski: Nothing to disclose
Lisa C. Adams: Nothing to disclose
Hugo Aerts: Nothing to disclose
Lina Xu: Nothing to disclose
Longjiang Zhang: Nothing to disclose
Felix Busch: Nothing to disclose
Keno Bressem: Nothing to disclose
Luca Saba: Nothing to disclose

**Implementing Artificial Intelligence with a Multi-AI Platform across 15 centres: Experience and Strategy from an International Healthcare Organization**

D. Penha[1], A. Juhos[1], D. Tálos[1], L. Rosa[2], M. Santos[2], E. Dias[2], R. Paroczai[1], R. Barone[1], *A. Roncacci*[1]; [1]Amsterdam/NL, [2]Lisboa/PT

**Purpose or Learning Objective:** Implementing artificial intelligence (AI) in radiology is challenging due to the variety of AI tools and the complexity of IT infrastructure and workflows. This presentation details a qualitative case study examining the implementation of six AI solutions using a multi-AI platform across 15 centers.
**Methods or Background:** A longitudinal qualitative case study was conducted in Portugal, in 15 radiology centers over two years (May to October 2024), focusing on the implementation of different AI tools (Veye Lung Nodules, Icobrain DM, Transpara, Keros/Polaris, qXR, and ARVA) using a multi-AI platform (Incepto). Data collected included 833 days of work observations, 86 meeting observations, and from Incepto dashboard.
**Results or Findings:** The multi-country healthcare organization's AI team managed the process adhering to a standard operating procedure covering initiation, planning, implementation, and clinical/operational phases. AI deployment started with Icobrain DM (11 centers) and Veye Lung Nodules (12 centers), processing 1,699 exams and 49,278 respectively. Transpara was deployed across three centers, resulting in 5,791 studies over 19 months. Keros/Polaris in two centers, with 706 studies over 18 months. ARVA and qXR were not implemented due to clinical decision. Overall, the AI implementation was successful, with over 56,000 studies processed by four tools across 15 centers. The study identified advantages of the multi-AI platform, including workflow, cost-effectiveness, and improved patient care. However, disadvantages such as complexity, integration challenges, and potential vendor lock-in were also noted.
**Conclusion:** This case study provides valuable insights for healthcare organizations considering AI implementation via multi-AI platform over stand-alone AI tools.
**Limitations:** The implementation of a multi-AI platform in Radiology lacks a clear blueprint to follow, creating the need to define new procedures and metrics. These procedures suited our needs of but may not apply to other radiology institutions.
**Funding for this study:** Not applicable
**Ethics committee - additional information:** Ethics committe and data/ legal department involved and with full approval of the whole project
**Author Disclosures:**
Daniel Tálos: Nothing to disclose
Alessandro Roncacci: Nothing to disclose
Luis Rosa: Nothing to disclose
Arpad Juhos: Nothing to disclose
Robert Paroczai: Nothing to disclose
Diana Penha: Nothing to disclose
Rocco Barone: Nothing to disclose
Miguel Santos: Nothing to disclose
Eduardo Dias: Nothing to disclose

**From theory to practice: Re-identification Challenge to test imaging data anonymization effectiveness**

*R. Catalán Flores*[1], I. Gómez-Rico[1], P. Jimenez[1], J. Gomes Carvalho[2], S. Mazzetti[3], R. Martínez Martínez[1], M. França[2], D. Regge[3], L. Marti-Bonmati[1]; [1]Valencia/ES, [2]Porto/PT, [3]Torino/IT
*(rocio_catalan@iislafe.es)*

**Purpose or Learning Objective:** DICOM image de-identification is an effective measure to protect patient privacy and ensure compliance with the GDPR. However, no standardized methods guarantee the irreversible anonymization of DICOM images or provide evidence on the robustness of these procedures. Organizations such as NEMA propose de-identification profiles for DICOM metadata, but the risk to data protection is assumed by the entity responsible for de-identification, as the dangers of using these profiles cannot be accurately measured. Given this context, the Re-identification Challenge serves as a technical audit to assess the robustness of DICOM image de-identification methods. The objectives of this project are to validate the robustness of these de-identification methods and to gain insight into organizing a challenge within the context of a European project.
**Methods or Background:** The Re-identification Challenge consisted of a single phase in which the selected 68 participants were tasked to re-identify 38 pseudonymized DICOM studies from multiple European hospitals (including Spain, Portugal and Italy), modalities and anatomical regions. They were pseudonymized locally using the de-identification profiles of ChAImeleon and ProCancer-I European AI4HI projects.
**Results or Findings:** Despite 74% of the participants delivered result, none succeeded in re-identifying the studies. Based on participants' reports of their attempts, vulnerabilities were discovered that allowed them to narrow the population by obtaining their geographical region.
**Conclusion:** This challenge is a groundbreaking initiative that may pave the way for robust evaluations of de-identification methods and may culminate in the standardization of de-identification profiles in the field of radiological imaging.
**Limitations:** This challenge involved a limited sample of DICOM studies to ensure GDPR compliance and protect patients’ privacy. Participant selection was controlled through specific projects to prevent unauthorized access and reduce the risk of data breaches, marking a pioneering effort in de-identification research.
**Funding for this study:** This challenge is part of the ChAImeleon project, specifically of Work Package 10, titled 'Repository Sustainability'. ChAImeleon has received funding from the European Union's Horizon 2020 research and innovation programme under grant agreement No 952172.
**Ethics committee - additional information:** Ethical considerations have been paramount throughout the Challenge's development, garnering approval from the Ethics Committee of the involved data providers institutions. Rigorous measures ensure privacy, security, and data legitimacy: patient consent forms were obtained for the medical studies; legal experts conducted a comprehensive data protection impact assessment; and the platform hosting the studies adheres to stringent security protocols and privacy policies.
**Author Disclosures:**
João Gomes Carvalho: Nothing to disclose
Luis Marti-Bonmati: Nothing to disclose
Ricard Martínez Martínez: Nothing to disclose
Daniele Regge: Nothing to disclose
Rocío Catalán Flores: Nothing to disclose
Paula Jimenez: Nothing to disclose
Manuela França: Nothing to disclose
Simone Mazzetti: Nothing to disclose
Ignacio Gómez-Rico: Nothing to disclose

**Establishing an applied framework to establish the trustworthiness of an international secure data environment for AI in CT imaging (AICT Consortium)**

*J. Kellas*[1], S. Van Wortswinkel[2], E. Casany Pujol[3], R. Lee[1], E. R. Ranschaert[4]; [1]Oxford/UK, [2]Borgerhout/BE, [3]Barcelona/ES, [4]Ghent/BE
*(john.kellas@nds.ox.ac.uk)*

**Purpose or Learning Objective:** The AICT consortium, funded by Horizon Europe (NetZeroAICT), consists of international clinical sites across 3 continents and proposes a comprehensive trustworthiness framework by systematically integrating ethical, legal, sustainability and stakeholder engagement elements throughout research and development. The goal is to ensure acceptability and trust in our research, innovation pipeline and AI-driven radiology applications.
**Methods or Background:** The NetZero AICT trustworthiness framework is based on the European Commission's Trustworthy AI model, including foundational elements—Lawfulness, Ethics, and Robustness—structured with key pillars: Human Agency, Technical Robustness, Privacy, Transparency, Diversity, Societal Wellbeing, and Accountability. The project adheres to GDPR compliance, local applicable privacy laws, and the EU AI Act, and integrates ethics and privacy by design, broad and deep public involvement, sustainability, innovation management, clinical validation, and regulatory compliance.
**Results or Findings:** This is an interim report (year 1 of Horizon Europe program). Here, we showcase the 'ethics by design' approach and an applied model of patient and public patient involvement and engagement. A public advisory group (PAG) has been formed with current membership from 4 countries and diverse backgrounds) with member representation on project leadership groups (adopting and adapting a tiered model implemented by large-scale UK health data infrastructure projects (including OpenSAFELY). Feedback from the Public Advisory Group and the project team strongly supported the view that integrating ethics by design from the concept stage through to deployment has improved public confidence and ensured compliance with ethical and legal standards.
**Conclusion:** By incorporating ethics by design, legal compliance, sustainability considerations and stakeholder engagement, the (NetZero) AICT consortium established a reliable framework for radiology AI, promoting fairness and trustworthiness among users and stakeholders.
**Limitations:** This framework applies to healthcare imaging AI.
**Funding for this study:** Horizon Europe and UK Research Innovation
**Ethics committee - additional information:** HRA number 22/HRA/2302
**Author Disclosures:**
Regent Lee: Shareholder: AI Sentia
John Kellas: Consultant: This Equals
Steven Van Wortswinkel: Employee: Ziekenhuis aan de Stroom
Erik R. Ranschaert: Nothing to disclose
Ernest Casany Pujol: Employee: CMRAD

**Time impact of AI-Assisted knee MRI reading in a real-world multi-center study within a radiology network**

*B. Rizk*[1], P. Cordelle[2], B. Dufour[1], N. Heracleous[1], C. Thouly[1], P. Zille[2], F. Zanca[3]; [1]Sion/CH, [2]Poitiers/FR, [3]Leuven/BE
*(benrizk@gmx.net)*

**Purpose or Learning Objective:** We explored the impact on reading time of KEROS, a knee-MRI multifaceted AI algorithm, across three distinct interpretation workflows.
**Methods or Background:** Clinical routine data was gathered from ten different centers during the daily workflow of eight radiologists, including four musculoskeletal subspecialists (MSKs) and four general knowledge radiologists (GENs), two of whom were junior (0 to 1 year of experience in private practice). We use a standardized report relying on voice recognition without secretariat formatting assistance. Data collection was performed in three phases: Phase 1, generated reports without AI assistance; Phase 2, KEROS AI diagnosis was available before their interpretation; Phase 3, AI findings were pre-filling and auto-integrated into the structured report. Reporting time was measured from report opening to validation, using the radiology information system (RIS). The Kruskal-Wallis test ($p<0.01$) assessed significant differences, and time differences were calculated using weighted means.
**Results or Findings:** In Phase 1, 431 exams were read with an average reading time of 10.8±9.5 to 26.3±9.3 minutes. In Phase 2 had 429 exams, with reading times between 12.8±8.2 and 27.5±9.2 minutes. Finally, Phase 3 included 425 exams with times ranging from 9.2±6.4 to 21.6±5.6 minutes. Cases were nearly equally read by MSKs and GENs. Compared to Phase 1, Phase 3 showed an average time reduction of 2.1 minutes ($p<0.01$) (13%), primarily driven by GENs, who saved up to 3.1 minutes (17%) ($p<0.01$).
**Conclusion:** We observe the average 13.4% ($p<0.01$) time reduction after implementing KEROS and pre-filled reporting (Phase 3 vs. Phase 1). Generalists, the primary users of AI-assisted knee MRI readings, see an average 17.4% ($p<0.01$) decrease in their reading time. In clinical practice, AI-assisted knee MRI reporting saves time for general radiologists, who benefit the most from AI guidance.
**Limitations:** No limitations
**Funding for this study:** No funding
**Ethics committee - additional information:** We acquired the green light by the ethical committee in Switzerland for our research structure including data registry and patient general consent for research. For the specific study presented here, only timing of radiologist reporting data was used and not patient data.
**Author Disclosures:**
Pascal Zille: Nothing to disclose
Natalie Heracleous: Nothing to disclose
Cyril Thouly: Nothing to disclose
Philippine Cordelle: Nothing to disclose
Benoît Dufour: Nothing to disclose
Benoît Rizk: Nothing to disclose
Federica Zanca: Nothing to disclose

**PARROT: A Collaborative Polyglottal Annotated Radiology Reports Database for Open Testing of Large Language Models**

*B. Le Guellec*[1], K. Bressem[2]; [1]Lille/FR, [2]Munich/DE
*(bastien.le.guellec@gmail.com)*

**Purpose or Learning Objective:** To create a database of annotated radiology reports in diverse languages on which to test Large Language Models (LLMs).
**Methods or Background:** Large Language Models (LLMs) represent one of the most important advancements in artificial intelligence in recent years. In the field of medicine, they hold the potential to tranform how physicians interact with and interpret medical data. However, the current research and applications of LLMs in medicine are predominantly focused on English-language datasets. This narrow focus raises significant concerns about the ability of LLMs to generalize across the thousands of other languages. As a result of difficulties accessing high-quality data from low-resources languages, patients may be excluded from the benefits of AI-driven advancements in healthcare. To address this critical gap, we have launched the Polyglottal Annotated Reports for Open Testing (PARROT) project. PARROT seeks to gather fictional medical reports from diverse linguistic and cultural backgrounds, manually annotated by experts for ICD-10 codes and make them freely accessible to the global research community. PARROT is a completely open-source initiative, inviting radiologists and medical professionals from around the world to contribute.
**Results or Findings:** 2648 annotated radiology reports from 75 radiologists from 20 countries in 13 languages have been collected. Most prevalent languages were Polish (808 reports), French (480 reports) and Italian (285 reports). Contributions from the Global South included Ivory Coast, Mexico, Madagascar, Togo, Gabon, Argentina, Algeria, Turkey and China.
**Conclusion:** The to the collaborative effort of 75 radiologists from 20 countries, PARROT is the largest multilingual open database of radiology reports to date.
**Limitations:** Annotation for this first iteration of PARROT is limited to ICD-10 codes. Most reports originated from European countries and European languages.
**Funding for this study:** No specific funding
**Ethics committee - additional information:** None required
**Author Disclosures:**
Keno Bressem: Nothing to disclose
Bastien Le Guellec: Nothing to disclose

16:00-17:30 Research Stage 4

### Research Presentation Session: Breast

### RPS 1602

### Interventional radiology in breast cancer care

**Moderator**

I. Allajbeu; Cambridge/UK
*(Ia359@cam.ac.uk;)*

**Detection and characterization of cryoimmunologic response induced by ultrasound-guided cryoablation on early breast cancer: evaluation of circulating markers**

F. Galati, *M. Pasculli*, C. Napoletano, V. Rizzo, R. Maroncelli, F. Cicciarelli, M. Nuti, C. Catalano, F. Pediconi; Rome/IT
*(marcella1.pasculli@gmail.com)*

**Purpose or Learning Objective:** Cryoablation is a minimally-invasive procedure that uses cooling to induce necrosis of the targeted tissue. All other minimally invasive techniques use hyperthermia, which melts cell membranes and causes protein denaturation. In contrast, cryoablation leaves tumor proteins and tumor-associated antigens intact, with the potential to stimulate an anti-tumor immune response. Thus, the purpose of this prospective pilot study was to characterize the immune response induced by tumor cryoablation in blood samples from early breast cancer (BC) patients.
**Methods or Background:** We enrolled patients with early-stage BC, scheduled for breast surgery, not eligible for neo-adjuvant therapy, and with a cryo-feasible cancer location. Blood samples to assess immune response were taken before (T0) and one week after cryoablation (T1), and before (T2) and one week after (T3) surgery. Analysis of T cell subsets (CD3, CD8, CD4, CD137, and Tregs) and the inflammatory/damage molecule HMGB1 were performed by flow cytometry/ELISA. Circulating cytokines were also analyzed using the Luminex analysis.
**Results or Findings:** From July 2022 to January 2023, ten patients underwent cryoablation. Analysis of circulating markers of cryo-immunological response revealed a progressive release of HGBM1 after cryoablation (T0-T1, p=0.04) until surgical resection of the primary tumor (T0-T3, p=0.02). Cryoablation followed by surgery also induced a significant decrease in CD137 T cell subsets (total and CD4; p<0.01), and IL4 (T0-T3, p<0.05). Finally, a significant decrease in proliferative Treg cell subsets (Ki67+Tregs; p<0.05) was observed.
**Conclusion:** In our pilot study, cryoablation induced the release of HMGB1, which acts to activate the primary phases of the immune response, and the decrease of the immunosuppressive Treg subset and the pro-tumoral cytokine IL-4, probably released by CD4CD137 T cells.
**Limitations:** Although the limited number of patients, cryoablation was a valuable method to enhance the anti-tumor response.
**Funding for this study:** The study has received funding from the Seed Grant funding programme of the European Society of Radiology (ESR) in collaboration with the European Institute for Biomedical Imaging Research (EIBIR) kindly supported by an unrestricted, non-exclusive grant from GE Healthcare.
**Ethics committee - additional information:** The study obtained the approval of the Institutional Review Board of “Sapienza” University of Rome (Ref.6528, approved 24.11.2021).
**Author Disclosures:**
Roberto Maroncelli: Nothing to disclose
Veronica Rizzo: Nothing to disclose
Francesca Galati: Nothing to disclose
Marcella Pasculli: Nothing to disclose
Federica Cicciarelli: Nothing to disclose
Marianna Nuti: Nothing to disclose
Federica Pediconi: Nothing to disclose
Carlo Catalano: Nothing to disclose
Chiara Napoletano: Nothing to disclose

**A convenient model based on mammography and magnetic resonance imaging for preoperative differentiation of breast phyllodes tumors and fibroadenomas**

*X. Ma*; Shanghai/CN
*(maxiaowen9397@163.com)*

**Purpose or Learning Objective:** To establish a fusion model based on mammography (MG) and magnetic resonance imaging (MRI) for the preoperative differentiation of breast phyllodes tumors (PTs) and fibroadenomas (FAs).
**Methods or Background:** The clinical data, MG images, and MR images of patients with breast FAs treated in our institution from October 2019 to December 2020, as well as patients with PTs treated from January 2011 to December 2020, were retrospectively collected. Univariate and multivariate logistic regression analyses were conducted to select independent factors and to construct a diagnostic model to differentiate PTs and FAs. The diagnostic performance of the model was evaluated using the receiver operating characteristic (ROC) curve, calibration curve, and decision curve analysis (DCA).
**Results or Findings:** A total of 147 patients with FAs and 138 patients with PTs were included in this study. The results of the multivariate logistic regression analysis showed that patient age, maximum diameter of mass, density on MG images, lobulation on MR images, and time-intensity curve (TIC) were independent factors contributing to the differential diagnosis. Finally, the fusion model showed satisfactory discrimination (area under the curve (AUC) 0.90, 95% CI: 0.86-0.94) and calibration. DCA indicated good clinical benefit, as indicated by most values being within threshold probabilities.
**Conclusion:** MG and MRI findings help differentiate between FAs and breast PTs preoperatively. The multimodal fusion model was clinically efficacious and beneficial and thus useful for accurate clinical diagnosis and treatment.
**Limitations:** Our study is a retrospective and single-centre study, so it is necessary to validate the accuracy of the research results through multicentre studies.
**Funding for this study:** None
**Ethics committee - additional information:** Fudan University Shanghai Cancer Center
**Author Disclosures:**
Xiaowen Ma: Nothing to disclose

**Correlation of radiological and pathological tumor sizes in early-stage breast cancer based on molecular subtypes and accompanying in situ carcinoma: A retrospective multicenter study**

D. E. Tekcan Sanli[1], *G. Esen*[2]; [1]Gaziantep/TR, [2]Istanbul/TR
*(gulesenicten@gmail.com)*

**Purpose or Learning Objective:** To compare the accuracy of radiological tumor sizes obtained by mammography (MMG), ultrasonography (US) and magnetic resonance imaging (MRI) with pathological sizes and to determine whether tumor size measurements change based on molecular subtypes and the presence of accompanying ductal carcinoma in situ (DCIS).
**Methods or Background:** A total of 559 breast cancer patients diagnosed in 11 university hospitals in Turkey between 2010 and 2022, underwent preoperative MMG, USG, and MRI, and did not receive neoadjuvant chemotherapy (NAC) were included in the study. Tumors were divided into histopathological (in-situ/invasive/ in-situ+invasive (mixed)) and molecular (Luminal A/B/HER2+/triple-) subgroups. Tumor sizes were measured on each modality retrospectively and compared with the pathologic sizes reported in the postoperative pathology reports. Comparison was performed based on histological type (invasive/ in situ/ mixed), and molecular subtypes.
**Results or Findings:** The highest agreement in invasive tumors was obtained with MRI (MRI:0.831, US:0.769, MMG:0.650). In the presence of DCIS, the agreement was strong with MRI (r:0.770), moderate with MMG and US (r:0.517, r:0.593, respectively). In mixed tumors, agreement was strong with MRI (r:0.817), moderate with US (r:0.656), and low with MMG (r:0.499). Based on molecular subtypes, highest correlation was obtained with US and MRI in HER-2 (+) tumors (r:0.754, r:0.715, respectively), and with MRI in other subtypes (Luminal A-B-triple (-)) with MRI (r:0.856-0.815-0.858; respectively). There was no statistically significant difference in terms of other criteria.
**Conclusion:** This multicenter study shows that MRI is the most reliable method in preoperative determination of tumor size for both invasive and in-situ tumors and all molecular subtypes.
**Limitations:** In this retrospective study, the number of patients was sufficient but unbalanced when divided into subgroups. The presence of invasive lobular carcinoma and axillary lymph nodes were not taken into account separately.
**Funding for this study:** No
**Ethics committee - additional information:** Ethics committee approval is obtained from Acıbadem University. (No:2021-21/29)
**Author Disclosures:**
Gul Esen: Nothing to disclose
Deniz Esin Tekcan Sanli: Nothing to disclose

**Breast abnormalities identified on cross sectional imaging represent a small but significant subgroup of referrals to symptomatic breast service**
*C. O'Brien*, M. R. Common, N. Hambly, N. Ní Mhuircheartaigh, M. Bambrick, D. Duke, N. Healy, E. Downey; Dublin/IE

**Purpose or Learning Objective:** Our retrospective study examines patients referred to a symptomatic breast centre with breast abnormality identified on cross-sectional imaging with special reference to frequency and outcomes.
**Methods or Background:** All patients presenting to our symptomatic breast centre over a 29-month period (Dec. 21-May 24) were evaluated. Patients referred following detection of breast abnormality on cross-sectional imaging were identified. Results of subsequent breast imaging, image-guided biopsies and histopathology were analysed.
**Results or Findings:** 13,336 consecutive referrals over a 29-month period were reviewed. 179 female/ 3 male patients, mean age 56.3 years (range 26 - 89years), were referred with breast abnormality on cross-sectional imaging. 158/182 (86.8%) were identified on CT, 9/182 (4,9%) on MRI, 14/182 (7.7%) on nuclear medicine, 1/182 (0.5%) on ultrasound. Clinical breast examination was normal in most patients. All patients underwent breast imaging. 75/ 182 (41.2%) patients underwent image guided biopsy. Histopathology results of biopsies were benign in 26/75 (34.7 %), indeterminate (B3) in 5/74 (6.8%) and malignant in 44/75 (58.6%). 2/5 patients with B3 histopathology underwent surgical excision yielding final benign pathology, while 3/5 opted for lesion surveillance. Overall cancer detection rate was 24.2% (44/ 182).
**Conclusion:** While breast abnormalities detected on cross sectional imaging represent a small subgroup of referrals the cancer detection rate in this cohort is significant. With normal clinical examinations these patients are at risk of delays along the traditional referral pathways based on clinical suspicion of malignancy. Direct referral of these patients to breast radiology ensures prioritisation based on lesion appearance on cross-sectional imaging.
**Limitations:** Only cross-sectional breast abnormalities of patients referred to the symptomatic breast service were reviewed.
**Funding for this study:** Nil
**Ethics committee - additional information:** Approval obtained from the Quality and Safety Directorate team at our institution.
**Author Disclosures:**
Niamh Hambly: Nothing to disclose
Deirdre Duke: Nothing to disclose
Marie Bambrick: Nothing to disclose
Matthew R Common: Nothing to disclose
Eithne Downey: Nothing to disclose
Conor O'Brien: Nothing to disclose
Neasa Ní Mhuircheartaigh: Nothing to disclose
Nuala Healy: Nothing to disclose

**Mammography-based radiomic analysis in triple negative ductal invasive breast cancer. Comparison between BRCA and not BRCA mutated patients: Preliminary results**
*G. Sessa*, C. Beretta, C. Bozzola, F. Mogavero, C. Parlato, L. Nocetti, G. Ligabue, P. Torricelli, A. Pecchi; Modena/IT

**Purpose or Learning Objective:** This study aims to evaluate the applicability of radiomics analysis to mammographic images of patients diagnosed with triple negative breast cancer (TNBC) in order to identify radiomics features that can differentiate the mutational status of BRCA genes.
**Methods or Background:** This retrospective study included patients histologically diagnosed with TNBC who performed a mammographic examination between 2010 and 2021. Mammographic images were reviewed and for each patient the tumor lesions were manually segmented in the mammographic projection where they were better demarcable; a further elliptical ROI (region of interest) of standard size (100 mm2) was drawn in the most homogeneous area of the controlateral healthy gland using the analogue mammographic projection of the same date or, if not available, of the corresponding bilateral mammographic investigation closer to the time of diagnosis. Features from each ROI were extracted with Pyradiomics-3D.
**Results or Findings:** The population included 50 patients and 51 lesions (12 BRCA+ patients and 13 lesions therein, 38 BRCAwildtype patients and 38 lesions therein). The lesions included 37 nodules, 3 pathologic microcalcifications and 11 lesions appearing as nodules with contextual microcalcifications. The segmentation was carried out on the following projections: 24 LCC, 24 RCC, 21 LMLO, 2 LML, 21 RMLO, 2RML. The first preliminary analysis demonstrated the feasibility of the radiomics study in the population examined. Based on a previous study conducted on DCE-MRI of the same target population, we expect to be able to identify differences in radiomic patterns which represent the fenotipic expression of mutations occurring on a genetic level.
**Conclusion:** This study demonstrated the feasability of radiomics analysis on diagnostic mammograms of TNBC patients to build a predictive model able to discriminate between carriers and non carriers of BRCA gene mutations.
**Limitations:** Small population
**Funding for this study:** No funding received for this study
**Ethics committee - additional information:** The research was approved by the Area Vasta Emilia Nord Est Ethical Committee (463/2020/OSS/AOUMO) SIRER ID 236 - EMENDAMENTO SOSTANZIALE 1.0 (prot. AOU 0008681/22 del 23/03/2022) and informed consent was obtained from all subjects.
**Author Disclosures:**
Giulia Sessa: Nothing to disclose
Pietro Torricelli: Nothing to disclose
Annarita Pecchi: Nothing to disclose
Luca Nocetti: Nothing to disclose
Guido Ligabue: Nothing to disclose
Chiara Bozzola: Nothing to disclose
Cecilia Beretta: Nothing to disclose
Francesca Mogavero: Nothing to disclose
Chiara Parlato: Nothing to disclose

**Can the new combined CB SCORE reduce the number of breast false positive biopsies? Results from a monocentric study**
*A. Portaluri*[1], F. M. Arico[1], X. Wang[2], T. Zhang[2], C. Sofia[1], E. Condorelli[1], F. Catanzariti[1], M. A. Marino[1]; [1]Messina/IT, [2]Amsterdam/NL
*(antonio_3_ap@libero.it)*

**Purpose or Learning Objective:** To evaluate the performance of a new combined score, CEUS-BI-RADS (CB) score, in differentiating between benign and malignant lesions and in reducing the number of unnecessary breast biopsies.
**Methods or Background:** 331 women with a new breast lesion scheduled for US-guided biopsy (sonographically assesed as BI-RADS 4 a-c and 5) were enrolled. For each lesion, CEUS examination was performed before the biopsy. 2 single reader (1 high-experienced breast radiologist and 1 trainee) independently assessed all CEUS studies with qualitative analysis, in terms of time and intensity of enhancement, enhancement pattern and size increase after contrast administration, assigning them a CEUS score from 0 to 3. Moreover a score from 1 to 4 was assigned for each BI-RADS category sonographically assesed as BI-RADS 4a, 4b, 4c and 5. Finally a combined score (CB score) was obtained. Descriptive statistics, area under the curve (AUC), receiver operating characteristic (ROC) analysis, sensitivity and specificity were used to investigate the diagnostic performance of the combined approach. Inter-reader reliability was measured using Cohen's kappa statistics.
**Results or Findings:** 294 lesions have been found. CB score showed the highest diagnostic performance compared to the CEUS score alone (average AUCs = 0.935 vs 0. 890, $p < 0.0001$), the highest sensitivity (87.9% vs 91.3%) and specificity (75.2% vs 80.7%) . Moderate ($\kappa$= 0. 45) agreement was found between the two readers. Finally, CB score would have obviated up to 40% of unnecessary biopsies.
**Conclusion:** CB score allowed to improve the diagnostic performance of CEUS alone for lesions assesment, reducing the rate of false positives biopsies for both experienced and unexperienced reader.
**Limitations:** Deeper studies on the application of this score need to be carried out to improve the identification of small tumours that have minimal blood supply.
**Funding for this study:** None
**Ethics committee - additional information:** This is a single-center, retrospective study. Ethics committee approval was waived as all Contrast-Enhanced Ultrasound (CEUS) examinations were conducted as part of routine clinical care, following established protocols at our institution. These examinations were clinically indicated prior to biopsy, ensuring that no additional procedures were performed outside the Standard of Care (SOC).
**Author Disclosures:**
Carmelo Sofia: Nothing to disclose
Elvira Condorelli: Nothing to disclose
Maria Adele Marino: Nothing to disclose
Francesca Catanzariti: Nothing to disclose
Xin Wang: Nothing to disclose
Antonio Portaluri: Nothing to disclose
Tianyu Zhang: Nothing to disclose
Francesco Marcello Arico: Nothing to disclose

**Non-surgical treatment of breast cancer: a comparison of outcomes between Cryoablation with hormonal therapy versus Cryoablation alone and hormonal therapy alone in patients not eligible for surgery**
*S. E. Baldi Giorgi*, F. Di Naro, G. Migliaro, F. Pugliese, T. Amadori, S. Vidali, G. Bicchierai, V. Miele, J. Nori; Florence/IT
*(sofiabaldi97@gmail.com)*

**Purpose or Learning Objective:** This study aims to evaluate the most effective non-surgical treatment for breast cancer in surgery-ineligible patients, comparing ultrasound-guided Cryoablation combined with hormonal therapy (HT) versus Cryoablation alone and hormonal therapy alone.

**Methods or Background:** A cohort of 64 patients (mean age 83.4 years) not-suitable for surgery due to comorbidities and/or advanced age was enrolled, with a total of 73 biopsy-confirmed malignant breast lesions. All the lesions were invasive ductal carcinomas (mean size 14.8 mm), hormone-positive and HER2-negative, with no ultrasound-visible lymph node involvement. Patients were divided into three groups: 36 lesions were treated with Cryoablation and HT, 19 with Cryoablation only and 18 with HT only. A locoregional staging was performed at baseline with contrast-enhanced-mammography (CEM) and ultrasound, followed by CEM and ultrasound follow-up at 12 months post-treatment. Only patients completing the follow-up were included. Lesion size was compared at baseline and 12 months after-treatment. Fisher's exact test was used for group comparison.
**Results or Findings:** Of the 73 lesions, 47 completed the 12-months follow-up: 20 in the Cryoablation-with-HT group, 9 in the Cryoablation-only group, and 18 in the HT-only group. Tumor size reduction was greatest in the Cryoablation-with-HT group (94%, mean reduction of 15.4 mm), followed by Cryoablation-only (82%, mean reduction of 9.7 mm), and HT-only (43%, mean reduction of 4.6 mm). Tumors with absent or low CEM-enhancement, suggesting residual-disease reduction, were most frequent in the Cryoablation-with-HT group (80%), followed by Cryoablation-only (77.8%), and HT-only (38.9%). Fisher's test revealed a significant difference between the Cryoablation-with-HT and the HT-only groups (p<0.0015), expressing the added value of Cryoablation.
**Conclusion:** Cryoablation with hormonal-therapy significantly reduces tumor size and residual disease more effectively than therapy alone, making it a promising option for patients not-eligible for surgery.
**Limitations:** No limitations were identified.
**Funding for this study:** No funding was received for this study.
**Ethics committee - additional information:** The study was approved by Comitato Etico Regione Toscana - Pediatrico (reference number: 165/2024).
**Author Disclosures:**
Giulia Bicchierai: Nothing to disclose
Francesca Pugliese: Nothing to disclose
Jacopo Nori: Nothing to disclose
Sofia Vidali: Nothing to disclose
Vittorio Miele: Nothing to disclose
Sofia Elisabetta Baldi Giorgi: Nothing to disclose
Tommaso Amadori: Nothing to disclose
Giuliano Migliaro: Nothing to disclose
Federica Di Naro: Nothing to disclose

**Vacuum-Assisted Biopsy in The Era of Low-Risk Ductal Carcinoma in Situ (LR-DCIS) Active Surveillance: Real World Data and Implications**
*H. L. Couto*[1], C. N. Valadares[2], B. F. De Paula Ricardo[1], A. N. Soares[1], P. H. Toppa[1], B. A. Coelho[3], C. C. Pessoa[4], N. Sharma[5], E. Carvalho Pessoa[4]; [1]Belo Horizonte/BR, [2]São Paulo/BR, [3]Montes Claros/BR, [4]Botucatu/BR, [5]Leeds/UK
*(enriquecouto@hotmail.com)*

**Purpose or Learning Objective:** Evaluate vacuum assisted biopsy (VAB) as diagnostic test of LR-DCIS in the context of real-world clinical practice.
**Methods or Background:** Database analysis of 116 cancers [both invasive breast cancers (IC) and ductal carcinoma in situ (DCIS)] diagnosed by VAB submitted to standard surgical treatment with complete histological data from VAB and surgery from 04/13/2017 to 11/28/2020. The VAB results were matched to the surgical pathology, considered the gold standard. The pathological diagnoses were grouped into malignancies requiring immediate surgical treatment [DCIS with high risk (HR-DCIS) of progression to IC or IC] versus those eligible to active surveillance (LR-DCIS). HR-DCIS/IC were considered positive while LR-DCIS negative results. VAB sensitivity, specificity, positive predictive value (PPV), negative predictive value (NPV), and accuracy were obtained.
**Results or Findings:** Median age was 56 (20-91); final median invasive tumor size (T) of 6mm (0,8 – 25) and 8mm (2 – 65) for DCIS; 65.52% were US-guided (70/116) and 44.48% (46/116) stereotactic guided; 42.24% (49/116) were only masses, 26,72% (31/116) masses associated with calcifications and 31.03% (36/116) calcifications. Out of 116 malignancy cases diagnosed by VAB, 15 (12.9%) resulted in LR- DCIS in the biopsy, 10 (8.6%) confirmed LR-DCIS in surgery, and 5 (4.3%) were upgraded to HR-DCIS/IC in surgery. VAB showed 95.28% sensitivity and 100% specificity. The positive predictive value (PPV) was 100%, and the negative predictive value (NPV) was 66.67%. Of the 5 false negatives (FN) LR-DCIS upgraded in surgery: 3 were HR-DCIS and 2 IC (pT1a-bpN0-luminal).
**Conclusion:** VAB, based in conventional pathology and immunohistochemistry, had an elevated FNR LR-DCIS in real world practice and, if applied, VAB LR-DCIS upgraded cases could be treated by either hormone or radiation therapy isolated or combined counterbalanced by slight reduction of overtreatment.
**Limitations:** Retrospective data base
**Funding for this study:** None
**Ethics committee - additional information:** The study was approved by the Ethics Committee of Santa Casa of Belo Horizonte under the number 25761019.8.0000.5138, and all methods were carried out in accordance with national guidelines
**Author Disclosures:**
Nisha Sharma: Nothing to disclose
Bernardo F. De Paula Ricardo: Nothing to disclose
Carla Carvalho Pessoa: Nothing to disclose
Bertha Andrade Coelho: Nothing to disclose
Carolina Nazareth Valadares: Nothing to disclose
Henrique Lima Couto: Nothing to disclose
Eduardo Carvalho Pessoa: Nothing to disclose
Paola H. Toppa: Nothing to disclose
Aleida N. Soares: Nothing to disclose

**Does touch imprint cytology prepared from core needle biopsy specimens in breast lesions provide an immediate diagnosis?**
Ş. Kökten, H. Kılın, *N. Voyvoda*; Istanbul/TR
*(nuraykad@gmail.com)*

**Purpose or Learning Objective:** The touch imprint method is used during the frozen study of sentinel lymph node samples in breast cancer patients and provides a rapid response with high accuracy and ensures the correct management of patients during surgery. Similarly, imprint preparations made from core biopsies can also be helpful in reaching rapid and accurate results. The aim of this study was to determine the diagnostic value and accuracy of the imprint method.
**Methods or Background:** Between January 2024 and March 2024, patients who were referred to the breast imaging center of our hospital due to a mass in the breast and planned for US-guided core needle biopsy were included in the study. Touch imprint and core biopsy specimens were retrospectively evaluated at different times by the same pathologist. Pathological findings in touch imprint evaluation were classified using the guideline.
**Results or Findings:** 201 lesions of 178 patients with an average age of 48.05 (min: 16-max 82) were evaluated. Of the 201 lesions, 186 were breast lesions and 15 were axillary lymph nodes. Seven of 186 breast lesions were excluded from the evaluation because touch imprint was defined as insufficient. The sensitivity of touch imprint was calculated as 87.50%, specificity as 89.16%, ppv: 54.66%, npv as 97.95% and accuracy as 88.94%. If we exclude the lymphoma patient, the sensitivity of imprint for lymph node was calculated as 100%, specificity as 83.3%, ppv: 47.27%, npv as 100% and accuracy as 85.5%. When not excluded, the sensitivity of imprint cytology was 88.89%, specificity as 83.33%, ppv: 44.35%, npv as 98.05% and accuracy as 84.06%.
**Conclusion:** Imprint cytology prepared from core biopsies of breast lesions can provide highly accurate and rapid diagnosis. Thus, treatment can be started without delay.
**Limitations:** No limitations were identified.
**Funding for this study:** No funding was received for this study.
**Ethics committee - additional information:** Local ethics committee approval (decision no: 2024/010.99/3/19) was obtained before the study.
**Author Disclosures:**
Şermin Kökten: Nothing to disclose
Nuray Voyvoda: Nothing to disclose
Halil Kılın: Nothing to disclose

**Differential efficacy of Cryoablation in breast cancer subtypes: ultrasound-guided scar biopsy evaluation year post-treatment**
*F. Pugliese*, F. Di Naro, G. Migliaro, S. E. Baldi Giorgi, T. Amadori, D. De Benedetto, C. Bellini, V. Miele, J. Nori; Florence/IT
*(francescapugliese28@gmail.com)*

**Purpose or Learning Objective:** To assess the effectiveness of cryoablation (CR) in different subtypes of breast tumors, a cohort of 39 biopsy-proven B5 lesions underwent ultrasound-guided scar biopsy evaluation one year post-treatment.
**Methods or Background:** From 2022-2023, the B5 lesions comprised 35 invasive ductal carcinoma (IDC), 2 IDC-associated DCIS, and 2 invasive lobular carcinomas. All patients were deemed inoperable for advanced age and comorbidities, leading to their enrollment in an annual follow-up and ultrasound-guided scar biopsy. The study population was stratified into three subgroups: molecular subtype, dimensional cut-off, and growth index.
**Results or Findings:** These lesions were hormone-responsive, with 19 classified as Luminal A and 20 as Luminal B. The dimensional cutoff ranged to 2.5 cm, with ice ball dimensions tailored to encompass a one-centimeter margin around the lesions. Data analysis revealed notable differences in the efficacy of cryoablation among the various subgroups. When considering Luminal A lesions with ki67>20% the complete ablation rate was 84.2% and for Luminal B it was 90.0%. Conversely, tumors with Ki67 expression ≤20% exhibited higher complete ablation rates, with Luminal A reaching 100% and Luminal B at 84.6%. Additionally, lesions ≤10 mm in size exhibited a higher complete ablation rate of 100% compared to lesions >10 mm, which showed an 80.0% success rate. Histologically, CR was ineffective in achieving complete ablation in DCIS cases, presenting a rate of 0%, but other subtypes demonstrated a higher complete ablation rate at 91.9%. However, none of these differences were statistically significant.
**Conclusion:** Cryoablation emerges as a promising primary treatment option for breast cancer is a safe and effective nonsurgical alternative to breast-conserving surgery in select patients with unifocal IDC low grade, hormone receptor-positive, and ≤10 mm size
**Limitations:** No limitations were identified.
**Funding for this study:** No funding was received for this study
**Ethics committee - additional information:** The study is retrospective
**Author Disclosures:**
Francesca Pugliese: Nothing to disclose
Diego De Benedetto: Nothing to disclose
Jacopo Nori: Nothing to disclose
Vittorio Miele: Nothing to disclose
Sofia Elisabetta Baldi Giorgi: Nothing to disclose
Tommaso Amadori: Nothing to disclose
Chiara Bellini: Nothing to disclose
Giuliano Migliaro: Nothing to disclose
Federica Di Naro: Nothing to disclose

### Saturday, March 1

**08:00-09:00** **Research Stage 1**

### Research Presentation Session: Genitourinary

### RPS 1707

### Advances in imaging techniques for the genitourinary tract

**Moderator**
A. Shinagare; Boston, MA/US

**Synthesising and adapting multiple data sources to design environmentally sustainable and clinically effective imaging pathways for visible haematuria**

J. B. John[1], K. O'Flynn[2], *S. Lambracos*[3], B. Abdelqader[4], S. Nalagatla[4], S. Khadouri[5], T. W. R. Briggs[3], W. K. Gray[3], J. Mcgrath[1]; [1]Exeter/UK, [2]Salford/UK, [3]London/UK, [4]Glasgow/UK, [5]Leeds/UK
*(simon.lambracos@gmail.com)*

**Purpose or Learning Objective:** To design a risk-stratified imaging pathway that reduces greenhouse gas (GHG) emissions using diagnostic performance evidence for ultrasound and computerised tomography urogram (CTU) in detecting upper tract urothelial cancer (UTUC).
**Methods or Background:** An audit of 15 UK hospitals' first-line imaging for visible haematuria (VH) and non-visible haematuria (NVH), and use of one-stop cystoscopy and imaging, was performed. Urology referral data from the IDENTIFY study (N = 10,896) were linked to national Hospital Episode Statistics data to estimate absolute numbers of patients receiving ultrasound or CTU first-line across England annually.
**Results or Findings:** Ultrasound was the first-line imaging choice for VH and NVH in 53% and 93% of hospitals respectively; other hospitals used CTU as first-line. One-stop assessment was performed in 44% of audited cases. An estimated 127,701 ultrasound and 77,880 CTU were performed across England annually, including an estimated 20% and 2% of additional CTU for patients with VH and NVH respectively receiving ultrasound first due to persistent VH. Informed by these data, a risk-stratified imaging pathway for patients with haematuria was developed. The pathway comprised: one-stop clinic ultrasound first-line for all, additional CTU for high-risk cases (VH + age>65 + smoking history), patient-initiated follow up direct to CTU for persistent VH if not performed initially. We estimate that this pathway could results in 191,097 ultrasounds, 51,320 CTU and 73% of assessments completed with one-stop assessment, leading to an estimated 269 tonnes CO2e reduction in net greenhouse gas emissions across England for one year. Around 0.1% of patients would have UTUC missed with first-line ultrasound on this pathway.
**Conclusion:** Adoption of this evidence-based pathway will reduce GHG emissions whilst delivering greater risk-stratified imaging use, improving equity of healthcare access and reducing unwarranted use of CTU.
**Limitations:** N/A
**Funding for this study:** Not applicable
**Ethics committee - additional information:** Not applicable
**Author Disclosures:**
Joseph B John: Nothing to disclose
John Mcgrath: Nothing to disclose
Sinan Khadouri: Nothing to disclose
Bushra Abdelqader: Nothing to disclose
Sarika Nalagatla: Nothing to disclose
Simon Lambracos: Nothing to disclose
Tim W R Briggs: Nothing to disclose
Kieran O'Flynn: Nothing to disclose
William K Gray: Nothing to disclose

**Study on Optimization of Image Quality of Computed Tomography Urography Using Dual-layer Detector Spectral CT Virtual Monoenergetic Imaging Technique Combined with Contrast Split Bolus Protocol**

*F. Zhao*, K. Li; Xi'an/CN
*(553785313@qq.com)*

**Purpose or Learning Objective:** To investigate the optimal keV of dual-detector spectral CT virtual monoenergetic imaging (VMI) in computed tomography urography (CTU).
**Methods or Background:** Hematuria patients who underwent dual-layer detector spectral CTU were collected. In the experimental group, 25mL of contrast bolus was injected first, and then 20mL of contrast bolus was injected 15 minutes later, and cortico-excretory phase CT scan was performed. In control group, non-contrast phase CT scan was performed first, then 100mL of contrast bolus was injected, and cortical, medullary and excretory phases CT scans were performed later. The experimental group was reconstructed into 40keV, 50keV, 60keV, 70keV VMI images and mixed energy images, and the excretory phase in control group was reconstructed into mixed energy image. The differences of CT value, standard deviation (SD), signal-to-noise ratio (SNR), contrast-to-noise ratio (CNR), subjective score of the image quality were compared.
**Results or Findings:** There was no significant difference in SNR and CNR between the mixed energy images of the control group and the VMI 50keV images of the experimental group. In the score of image display effect, the VMI 50keV image of the experimental group had the highest score. In the score of filling degree of contrast bolus, VMI 40keV images in the experimental group had the highest score.The effective dose in the experimental group was about 19.96% of that in the control group, and the amount of contrast bolus in the experimental group was about 45% of that in the control group.
**Conclusion:** The dual-layer detector spectral CT VMI technique used in CTU can improve the image quality while reducing the amount of contrast bolus and radiation dose, and VMI 40～50keV is the best energy level for image display.
**Limitations:** A single-center study with a small number of cases
**Funding for this study:** This study was funded by the hospital by RMB 10,000
**Ethics committee - additional information:** This study was approved by the Ethics Committee
**Author Disclosures:**
Fanhui Zhao: Nothing to disclose
Kai Li: Nothing to disclose

**Prospective evaluation of high-resolution diffusion-weighted imaging accelerated by deep-learning reconstruction in multiparametric MRI of the prostate**

*S. Ursprung*[1], J. Herrmann[1], E. Weiland[2], D. Nickel[2], A. Lingg[1], S. Afat[1], S. Gassenmaier[1]; [1]Tübingen/DE, [2]Erlangen/DE

**Purpose or Learning Objective:** Prostate MRI is a gatekeeper for more invasive investigations in prostate cancer diagnosis. This study investigates the potential of deep-learning reconstruction of high-resolution (HR) diffusion-weighted imaging (DWI) to improve image quality and lesion detectability.
**Methods or Background:** Prospective study comparing multiparametric MRI according to PI-RADS 2.1 specifications with standard (DWI-Std) and DWI-HR (4-fold higher in-plane resolution) on a 3T MRI-system. Two radiologists (7yr experience) compared image quality of DWI-Std/DWI-HR qualitatively and quantitatively using the Prostate Imaging Quality Scoring System (PI-QUALv2), a 5-point Likert-scale assessing sharpness, noise, artefacts, overall impression and diagnostic confidence, and contrast-to-noise ratio (CNR) of prostatic lesions.
**Results or Findings:** 91 patients consented (17 exclusions for prostatectomy, 2 radiotherapy, 3 incomplete imaging); 69 patients were included. Average acquisition time for DWI-Std/DWI-HR was 04:30min/05:33min. DWI-DL showed higher sharpness at all b-values and on ADC maps (p<0.001). This came at the cost of higher noise on b1000 and ADC (p<0.001), resulting in comparable lesion detectability (p=0.28) and PI-RADS scoring (p=1). Readers favoured DWI-HR in 47% and DWI-Std in 33%. The PI-QUAL sub-score of DWI-Std/DWI-HR was similar (p=0.37); only the reduced SNR/Contrast in DWI-HR approached significance (p=0.054). PI-QUAL was optimal in 67%/61%, acceptable in 28%/33% and inadequate in 6%/6% when including DWI-Std/DWI-HR. The CNR of PI-RADS 3-5 lesions in the PZ was significantly higher at b1000/on ADC maps from DWI-Std (median 10.8 vs. 10.3, p=0.002 at b1000, 10.1 vs. 7.7, p=0.0002 for ADC). The CNR between PZ/TZ was higher in DWI-Std (median 3.0 vs. 2.2; p=0.04).
**Conclusion:** We prospectively evaluated high-resolution DWI with DL-reconstruction, showing improved sharpness at slightly reduced CNR and maintained diagnostic performance in prostate MRI.
**Limitations:** This study was conducted on scanners of a single vendor. Confirmatory histology was available for PI-RADS 3+ lesions only.
**Funding for this study:** This study received no funding.
**Ethics committee - additional information:** Tubingen University Hospital IRB
**Author Disclosures:**
Judith Herrmann: Nothing to disclose
Elisabeth Weiland: Employee: Siemens Healthineers
Saif Afat: Speaker: Siemens Healthineers Research/Grant Support: Siemens Healthineers
Dominik Nickel: Employee: Siemens Healthineers
Andreas Lingg: Nothing to disclose
Stephan Ursprung: Nothing to disclose
Sebastian Gassenmaier: Nothing to disclose

**Ultra-Fast Biparametric MRI in Prostate Cancer Assessment: Diagnostic Performance and Image Quality Compared to Conventional Multiparametric MRI**

*A-M. Pausch*, V. Filleböck, C. Elsner, N. Rupp, D. Eberli, A. M. Hötker; Zurich/CH

**Purpose or Learning Objective:** To compare the diagnostic performance and image quality of a deep-learning-assisted ultra-fast biparametric MRI (bpMRI) with the conventional multiparametric MRI (mpMRI) for the diagnosis of clinically significant prostate cancer (csPCa).
**Methods or Background:** This IRB-approved prospective single-center study enrolled 123 biopsy-naïve patients undergoing conventional mpMRI and additionally ultra-fast bpMRI at 3T between 06/2023-02/2024. Two radiologists (R1: 4 years and R2: 3 years of experience) independently assigned PI-RADS scores (PI-RADS v2.1) and assessed image quality (mPI-QUAL score) in two blinded study readouts. Weighted Cohen's Kappa (κ) was calculated to evaluate inter-reader agreement. Diagnostic performance was analyzed using clinical data and histopathological results from clinically indicated biopsies.
**Results or Findings:** Inter-reader agreement was good for both mpMRI (κ = 0.83) and ultra-fast bpMRI (κ = 0.87). Both readers demonstrated high sensitivity (≥94%/≥91%, R1/R2) and NPV (≥96%/≥95%) for csPCa detection using both protocols. The more experienced reader mostly showed notably higher specificity (≥77%/≥53%), PPV (≥62%/≥45%), and diagnostic accuracy (≥82%/≥65%) compared to the less experienced reader. There was no significant difference in the diagnostic performance of correctly identifying csPCa between both protocols (p>0.05). The ultra-fast bpMRI protocol had significantly better image quality ratings (p<0.001) and achieved an 80% reduction in scan time compared to mpMRI.
**Conclusion:** Deep-learning-assisted ultra-fast bpMRI protocols offer a promising alternative to conventional mpMRI for diagnosing csPCa in biopsy-naïve patients with comparable inter-reader agreement and diagnostic performance at superior image quality. However, reader experience remains essential for diagnostic performance.
**Limitations:** The single-center design and the exclusion of some patients with a PI-RADS ≥ 3 who did not undergo biopsy may limit the generalizability of our findings.
**Funding for this study:** Holcim Stiftung zur Förderung der wissenschaftlichen Fortbildung
**Ethics committee - additional information:** Cantonal Ethics Commission Zurich
**Author Disclosures:**
Niels Rupp: Nothing to disclose
Clara Elsner: Nothing to disclose
Daniel Eberli: Nothing to disclose
Vivien Filleböck: Nothing to disclose
Antonia-Maria Pausch: Nothing to disclose
Andreas M. Hötker: Nothing to disclose

**Increased dose of iodine contrast media does not increase the odds of contrast-associated acute kidney injury**

*F. B. Berglund*, P. Liss, R. Frithiof; Uppsala/SE
*(felix.berglund@uu.se)*

**Purpose or Learning Objective:** The aim of this study is to investigate if any of the two components of the g-I/eGFR ratio is independently associated with the development of contrast-associated acute kidney injury (CA-AKI).
**Methods or Background:** All patients admitted to the intensive care units (ICUs) of a tertiary hospital from January 2013 to February 2020 were retrospectively identified. Those who underwent iodine contrast media (ICM)-enhanced CT exams were included in this nested case-control study. CA-AKI was defined and staged based on the creatinine and urine output criteria set forth by the Kidney Disease Improving Global Outcomes guidelines. The two components of the ratio, the dose of ICM (measured in grams of iodine) and renal function estimated by plasma creatinine, were analyzed separately in relation to the odds of developing CA-AKI.
**Results or Findings:** Among the 214 patients included in the analysis PC-AKI occurred in 42 of the patients (19.6%). Median age was 61.5 years (IQR 40-73) and 59.3% were of male sex. Renal function at the day of the CT-scan differed between those developing PC-AKI (eGFR 56.9, IQR 35-87) and those that did not (eGFR 81.6, IQR 58-96). However, the dose of ICM was not associated with PC-AKI development (OR 1.31 (IQR 0.49-3.47), p=0.827).
**Conclusion:** In this case-control study, renal function at the day of the examination but not the administered dose of iodine contrast media was associated with PC-AKI. This suggest that including injected amount of iodine contrast media as a variable to clinically predict risk of PC-AKI is futile.
**Limitations:** This is a single center case-control study where only 42 critically ill patients developed CA-AKI. This reduces the generalizability of the study as well as its power.
**Funding for this study:** The study was supported with funding from ALF from Uppsala University Hospital, and the Swedish Research Council (2014-02569 and 2014-07606). Funding bodies had no role in the design of the study, data collection, interpretation, or in the writing of the manuscript.
**Ethics committee - additional information:** This study was approved by the Swedish Ethical Review Authority (Dnr 2017/168 with amendment 2020-00135). Declaration of Helsinki and its subsequent revisions were observed.
**Author Disclosures:**
Robert Frithiof: Nothing to disclose
Per Liss: Nothing to disclose
Felix Björn Berglund: Nothing to disclose

**Modified in-plane resolution while maintaining high image quality T2-weighted prostate MRI**

*S. J. Riederer*[1], E. Borisch[1], A. Froemming[1], A. Kawashima[2], N. Takahashi[1]; [1]Rochester, MN/US, [2]Phoenix, AZ/US
*(riederer@mayo.edu)*

**Purpose or Learning Objective:** To determine if an axial T2-weighted spin-echo (T2-WI) sequence with modified in-plane spatial resolution could provide non-inferior performance and reduced acquisition time vs. a standard PI-RADSv2.1-compatible sequence.
**Methods or Background:** Both sequences used 3 mm slice thickness, 16 cm FOV, acceleration R=1.5, TR>3000, TE 150. The PI-RADS-compatible sequence used 400×230 in-plane sampling, 0.4 mm × 0.7 mm resolution (0.280 mm2 pixel area). The modified sequence used 320×280 in-plane sampling, 0.5 mm × 0.57 mm resolution (0.285 mm2 pixel area). Although the increased phase sampling of the modified sequence (280 vs. 230) required more repetitions, the reduced frequency sampling (320 vs. 400) allowed lower bandwidth and reduced averaging. The two sequences were both applied to 62 consecutive subjects identified for clinical prostate MRI. The number of slices was patient-specific but identical for the two sequences. Results were blindly reviewed by three experienced radiologists. Each series was assessed individually for Image Quality (IQ) using a 0-3 scale. For each subject the two series were also compared for overall preference on a five-point (-2, -1, 0, +1, +2) scale.
**Results or Findings:** Scan time depended on slice count (29 to 45). Scan time range for the PI-RADS-compatible sequence was 2:56-5:04 (median 3:54) and for the modified sequence 2:16-3:56 (median 3:01). Scan time reduction using the modified sequence was 37 to 80 sec (median 53 sec). 51/62=82.2% of the PI-RADS-compatible series and 56/62=90.3% of the modified sequence were evaluated as diagnostically interpretable (IQ=2,3). Reviewer-averaged scores showed a significant preference for the modified sequence (p<0.001).
**Conclusion:** Although not adherent to PI-RADSv2.1 guidelines, acquisition with essentially identical in-plane pixel area (0.28 mm2) allows 53 sec (23%) reduction in acquisition time and significantly improved reviewer preference vs. a PI-RADS-adherent sequence.
**Limitations:** Limited number of subjects
**Funding for this study:** This work was supported by NIH.
**Ethics committee - additional information:** Approved by Institutional Review Board (IRB)
**Author Disclosures:**
Adam Froemming: Nothing to disclose
Stephen J. Riederer: Nothing to disclose
Eric Borisch: Nothing to disclose
Naoki Takahashi: Nothing to disclose
Akira Kawashima: Nothing to disclose

**CEST Imaging vs. DWI with and without CEST Imaging: Capability for Distinguishing Malignant from Benign Prostatic Areas**

*T. Ueda*, H. Nagata, M. Nomura, T. Yoshikawa, D. Takenaka, Y. Ozawa, Y. Ohno; Toyoake/JP
*(yohno@fujita-hu.ac.jp)*

**Purpose or Learning Objective:** 3D Chemical exchange saturation transfer (CEST) imaging is recently developed to obtain CEST information within entire tumor. The purpose of this study was to compare the capability for distinguishing malignant from benign prostatic areas among 3D CEST imaging, diffusion weighted imaging (DWI) and combined both information.
**Methods or Background:** Fifty-two suspected prostatic cancer patients underwent DWI at b value as 0 and 1500 s/mm2 and 3D CEST imaging, surgical treatments and pathological examinations. According to the pathological results, 154 areas were determined as malignant prostatic areas, and 154 out of 470 areas were computationally selected as benign prostatic areas. On each 3D CEST imaging, magnetization transfer ratio asymmetry (MTRasym) at 3.5 ppm map was generated. Then, 308 ROIs were placed over malignant or benign areas on each map, and MTRasym and ADC values were determined. Each index was compared between malignant and benign areas by Student's t-test. ROC analysis was performed to compare diagnostic performance among MTRasym, ADC and combined discriminators. Finally, sensitivity, specificity and accuracy were compared among all methods by McNemar's test.

**Results or Findings:** MTRasym and ADC of malignant area had significant differences with those of benign area (MTRasym: p<0.0001, ADC: p<0.0001). Area under the curves (AUC) of combined discriminators (AUC=0.86) was significantly better than that of MTRasym (AUC=0.81, p=0.001) and ADC (AUC=0.76, p<0.0001). Specificity (SP) and accuracy (AC) of combined discriminators (SP: 72.1%, AC: 78.6%) were significantly higher than those of MTRasym (SP: 60.4%, p<0.0001; AC: 73.1%, p<0.0001) and ADC (SP: 64.2%, p<0.0001; AC: 74.0%, p<0.0001).
**Conclusion:** 3D CEST imaging is considered at least as valuable as DWI and can improve capability for differentiation of malignant from benign prostatic areas with DWI.
**Limitations:** LImited study population
**Funding for this study:** Research grants from Canon Medical Systems
**Ethics committee - additional information:** Fujita Health University Hospital
**Author Disclosures:**
Yoshiyuki Ozawa: Research/Grant Support: Smoking Research Foundation Research/Grant Support: Grant-in-Aid for Scientific Research from the Japanese Ministry of Education, Culture, Sports, Science and Technology
Masahiko Nomura: Nothing to disclose
Takahiro Ueda: Research/Grant Support: Grant-in-Aid for Scientific Research from the Japanese Ministry of Education, Culture, Sports, Science and Technology
Daisuke Takenaka: Nothing to disclose
Hiroyuki Nagata: Research/Grant Support: Grants-in-Aid for Scientific Research from the Japanese Ministry of Education, Culture, Sports, Science and Technology Research/Grant Support: Canon Medical Systems Corporation
Takeshi Yoshikawa: Nothing to disclose
Yoshiharu Ohno: Research/Grant Support: Smoking Research Foundation Research/Grant Support: Canon Medical Systems Corporation

**The comparison between virtual non-contrast and true non-contrast imaging of adrenal masses on photon-counting CT**
*X. Bai*[1], G. Zhang[1], X. Zhang[1], J. Zhang[1], L. Chen[1], Q. Peng[1], Z. Lin[2], H. Sun[1], Z. Jin[1]; [1]Beijing/CN, [2]Shanghai/CN

**Purpose or Learning Objective:** To investigate the differences in CT attenuation and radiomics features between virtual non-contrast (VNC) and true non-contrast (TNC) of adrenal masses on photon-counting CT (PCCT).
**Methods or Background:** Patients with adrenal masses who underwent unenhanced and portal-venous-phase PCCT were included. Image reconstructions of portal-venous phase were performed, including Conventional VNC (VNCconv) and PureCalcium VNC (VNCpc) algorithms. For two dimensional (2D) measurements, we measured CT attenuation of adrenal mass at the largest slice on TNC, VNCconv, and VNCpc images, respectively. Semiautomatic segmentations of adrenal masses were performed to extract three-dimensional (3D) CT attenuation and radiomics features on TNC and VNC. The paired t-test, Bland–Altman plots and intraclass correlation efficient (ICC) were used for statistical analyses.
**Results or Findings:** The study consisted of 54 patients (27 female, mean age 45.3 years) with 68 adrenal lesions. CT attenuation on VNCconv and VNCpc was higher than that on TNC (Mean differences, 2D: 8.03 HU for VNCconv and 5.76 HU for VNCpc, 3D: 8.75 HU for VNCconv and 6.89 HU for VNCpc). The proportion of lipid-rich adenomas (TNC < 10 HU) correctly classified by VNCconv and VNCpc was 26.1% (6/23) and 39.1% (9/23), respectively. TNC, VNCconv, and VNCpc attenuation did not differ significantly between 2D and 3D measurements (all P ＞ 0.05). The ICCs of first-order features, shape features and texture features between TNC and VNCconv were 0.625, 0.820 and 0.591, respectively.
**Conclusion:** The VNC algorithms of PCCT overestimated CT attenuation. CT attenuation at the largest slice can replace 3D attenuation. VNC and TNC have excellent agreement on shape features and average agreement on first-order features and texture features.
**Limitations:** The limitations of the study are the study sample is relatively small and the retrospective study may have a selection bias.
**Funding for this study:** Funding was provided by the National High-Level Hospital Clinical Research Funding (2022-PUMCH-B-069, 2022-PUMCH-A-033 and 2022-PUMCH-A-035), CAMS Innovation Fund for Medical Sciences (2022-I2M-C&T-B-019) and Beijing Municipal Natural Science Foundation (L232133).
**Ethics committee - additional information:** The study was approved by the Institutional Review Board (No. 23PJ1487).
**Author Disclosures:**
Jiahui Zhang: Nothing to disclose
Gumuyang Zhang: Nothing to disclose
Hao Sun: Nothing to disclose
Xin Bai: Nothing to disclose
Qianyu Peng: Nothing to disclose
Zijing Lin: Nothing to disclose
Li Chen: Nothing to disclose
Zhengyu Jin: Nothing to disclose
Xiaoxiao Zhang: Nothing to disclose

**08:00-09:00** **Research Stage 2**

### Research Presentation Session: Cardiac

### RPS 1703

### Cardiac imaging: interactions with other organs and systemic diseases

**Moderator**
P. Krumm; Tübingen/DE
**Author Disclosures:**
Patrick Krumm: Research Grant/Support: Spimed AI, Siemens Healthcare; Speaker: Bayer Healthcare, Siemens Healthineers, Bracco

**Association between pericoronary fat attenuation index, fractional flow reserve and brain white matter hyperintensity: a case control study**
*J. Qin*, Y. Xu; Nanjing, Jiangsu/CN

**Purpose or Learning Objective:** To explore the association between coronary computed tomography angiography (CCTA)- derived pericoronary fat attenuation index (pFAI), fractional flow reserve (CT-FFR) and degree of white matter hyperintensities (WMH).
**Methods or Background:** Clinical, CCTA and brain magnetic resonance imaging (MRI) data of 561 participants were retrospectively analyzed. WMH were assessed in periventricular (PVWMH) and deep (DWMH) locations, and a total Fazekas score was calculated by summing the scores for PVWMH and DWMH. The study cohort was classified into mild WMH group (score 0-2) and moderate-to-severe WMH group (score 3-6). Coronary artery disease (CAD) was defined as one or more coronary arteries with diameter stenosis of ≥50%. The threshold value of CT-FFR was set as 0.80. Clinical data, pFAI, CT-FFR and other coronary parameters were compared between two groups, and independent variables associated with moderate-to-severe WMH were identified using multiple logistic regression analysis.
**Results or Findings:** Compared with patients with mild WMH, those with moderate-to-severe WMH showed larger volume of plaque (total, calcified, noncalcified palque), higher plaque burden, longer plaque length, higher Agatston Score, higher value of pFAI and higher proportion of CT-FFR≤ 0.80, higher proportion of patients with CAD and aortic ulcers (all p<0.05). Multiple logistic regression indicated that age [odds ratio (OR), 1.028; p=0.025], CAD (OR, 5.282; p=0.004), plaque burden (OR, 4.101; p=0.004) , noncalcified plaque burden (OR, 2.850; p<0.001) and pFAI (OR, 1.109; p<0.001) were independently associated with moderate-to-severe WMH.
**Conclusion:** Besides the well-known factors including age, CAD and plaque burden, pFAI was also found to be associated with moderate-to-severe WMH.
**Limitations:** As a cross-sectional study, the progression of WMH was not analyzed. Future research exploring the progression of CCTA- derived parameters including CT-FFR and pFAI and the progression of WMH would be more valuable.
**Funding for this study:** This work was supported by the grants from the Natural Scientific Foundation of China (Grant Nos. 82302163 for Yunfei Wang) and Young Scholars Fostering Fund fo the First Affiliated Hospital of Nanjing Medical University (Grant Nos. PY2022036 for Yunfei Wang).
**Ethics committee - additional information:** The requirement of written informed consent was waived due to the retrospective nature.
**Author Disclosures:**
Jie Qin: Nothing to disclose
Yi Xu: Nothing to disclose

**Quantitative T1 mapping for the evaluation of the iron overload in hereditary hemochromatosis**
*G. Prencipe*, I. Notarangelo, P. Mangano, L. Marinelli, L. Macarini, G. Guglielmi, M. Gravina; Foggia/IT
*(gianluca.prencipe.12@gmail.com)*

**Purpose or Learning Objective:** The aim of this study was to evaluate the efficacy of cardiac T1 mapping MRI sequences in assessing myocardial iron overload in patients with Hereditary Hemochromatosis, in comparison to the more commonly employed T2* sequences.
**Methods or Background:** A total of 28 cardiac MRI scans, conducted between 2019 and 2023, were analyzed. All patients had Hereditary Hemochromatosis and elevated serum ferritin levels. The MRI scans were performed using a Philips Achieva dStream 1.5T scanner with cardiac gating and included T2* and native T1 mapping sequences, without the use of contrast agents. Quantitative T2* analysis was conducted by placing regions of interest (ROIs) in the interventricular septum (IVS), and these results were compared with the corresponding native T1 mapping findings.

**Results or Findings:** Out of the 28 patients, 25 had T2* values over 20 msec, indicating no significant iron deposition. The remaining three patients exhibited T2* values between 15 and 20 msec, suggesting mild iron overload. None of the patients demonstrated T2* values below 15 msec. In the three patients with mild iron overload, T1 mapping showed correspondingly low values, consistent with their T2* measurements. However, 5 of the 25 patients with normal T2* values had T1 values below the expected range (980-1080 msec for the our scanner).
**Conclusion:** Native T1 values were reduced in patients with myocardial iron accumulation and correlated well with T2* measurements. T1 mapping provides the added benefit of detecting early-stage iron overload that might be missed by T2* alone, making it a valuable tool for the early diagnosis of iron deposition and for monitoring the effectiveness of chelation therapy
**Limitations:** The study is retrospective. Single center study. Few patients with low T2*.
**Funding for this study:** No funding was received for this study.
**Ethics committee - additional information:** The study is retrospective
**Author Disclosures:**
Luca Marinelli: Nothing to disclose
Giuseppe Guglielmi: Nothing to disclose
Ilenia Notarangelo: Nothing to disclose
Matteo Gravina: Nothing to disclose
Gianluca Prencipe: Nothing to disclose
Paola Mangano: Nothing to disclose
Luca Macarini: Nothing to disclose

**Sex-Specific Prognostic Value of Opportunistic Epicardial Adipose Tissue Quantification on Lung Cancer Screening Chest CT**
*E. Norton*, J. M. Brendel, I. Hadzic, T. Mayrhofer, I. L. Langenbach, M. C. Langenbach, M. T. Lu, H. Aerts, B. Foldyna; Boston, MA/US
*(eanorton@mgh.harvard.edu)*

**Purpose or Learning Objective:** To evaluate the prognostic value of epicardial adipose tissue (EAT) volume and density between women and men (who often present with different body fat distribution) eligible for lung cancer screening, a group with an unmet need for better risk stratification.
**Methods or Background:** Using a validated deep-learning algorithm, we measured EAT volume (indexed to body-surface-area; $cm^3/m^2$) and density (HU) on baseline non-contrast, low-dose chest CTs from the National Lung Screening Trial. Associations with 12-year all-cause and cardiovascular mortality were assessed using multivariable Cox regression models, stratified by sex, and adjusted for age, race/ethnicity, smoking status (current vs. former), pack-years, history of heart disease or stroke, diabetes mellitus, hypertension, educational status, and body-mass-index.
**Results or Findings:** Among 24,090 participants, 9,886 were women (41%; 61±5 years), and 14,204 were men (59%; 62±5 years). Women presented lower EAT volumes and higher densities than men (65.7±23.2$cm^3/m^2$ and -77.2±5.1HU vs. 73.5±25.0$cm^3/m^2$ and -78.0±5.2HU; p<0.001 for sex differences). A 10 $cm^3/m^2$ EAT volume increase revealed similar prognostic values in women and men (all-cause mortality: aHR:1.10 [95%-CI: 1.07–1.13] in women vs. aHR:1.11 [95%CI: 1.08–1.13] in men; cardiovascular mortality: aHR:1.13 [95%-CI: 1.06–1.20] in women vs. aHR:1.16 [95%-CI: 1.11–1.21] in men). EAT density demonstrated a stronger association with all-cause mortality in men than in women (aHR:1.41 [95%-CI: 1.28–1.56] in men vs. aHR:1.28 [95%-CI: 1.10–1.47] in women; p=0.010 for sex as interaction term). The associations were similar with cardiovascular death (aHR:1.76 [95%-CI: 1.29–2.41] in women vs. aHR:1.78 [95%-CI: 1.45–2.19] in men).
**Conclusion:** Opportunistic EAT volume and density quantification may improve risk stratification in women and men eligible for lung cancer screening, with EAT density being a stronger predictor of all-cause death in men than women.
**Limitations:** Exclusively heavy smokers were included, precluding comparison with non-smokers and lower-risk smokers.
**Funding for this study:** Original data collection for NLST was funded by NIH.
**Ethics committee - additional information:** Insight IRB protocol #: 2017P002400
**Author Disclosures:**
Jan Michael Brendel: Nothing to disclose
Isabel Luisa Langenbach: Nothing to disclose
Emilia Norton: Nothing to disclose
Hugo Aerts: Nothing to disclose
Ibrahim Hadzic: Nothing to disclose
Thomas Mayrhofer: Nothing to disclose
Borek Foldyna: Nothing to disclose
Marcel Christian Langenbach: Nothing to disclose
Michael T. Lu: Nothing to disclose

**Cardiovascular Magnetic Resonance Features in Cirrhotic Patients with Transjugular Intrahepatic Portosystemic Shunt**
*J. Arenja*, D. G. Aydemir, L. Naimi, I. Molwitz, M. Sterneck, G. Adam, P. Bannas, E. Tahir, J. Erley; Hamburg/DE

**Purpose or Learning Objective:** A transjugular intrahepatic portosystemic shunt (TIPS) results in blood flow from the splanchnic to the venous circulation. We aimed to evaluate if a TIPS impacts cardiovascular magnetic resonance (CMR) features in patients with liver cirrhosis.
**Methods or Background:** In this retrospective, monocentric study, 60 patients with liver cirrhosis received a CMR exam (3T, Ingenia, Philips or Siemens). Left ventricular (LV) mass, volumes (indexed to the body mass index [BMI]), and ejection fraction (EF) were analyzed. Strain was measured by feature tracking using Cvi42 (Circle Vascular Imaging). For statistics, mixed linear models were conducted.
**Results or Findings:** 30 patients with TIPS (40% females, age 56±11 years, BMI 26.8±5.2 kg/m2, hepatic venous pressure gradient (HVPG) pre-TIPS 27.9±6.7 and post-TIPS 11.2±4.1 mmHg) were compared to 30 matched patients without TIPS (47% females, age 53±14 years, BMI 26.1±5.1 kg/m2). Patients with TIPS showed a -14.8% attenuated LV global radial strain [95% confidence interval (CI): -28.1 to -1.5%] compared to patients without TIPS (p = 0.037). LV mass, volumes, and longitudinal/circumferential strain were not significantly different between the groups. In patients with TIPS, LV end-diastolic mass (regression coefficient = 1.5 ml/m2 [95%-CI: 0.1 to 3%], p = 0.040) and LV global longitudinal strain (0.6% [95%-CI: 0.1 to 1.2%], p = 0.035) were associated with HPVG post-TIPS.
**Conclusion:** Cirrhotic patients with TIPS show attenuated LV radial strain compared to patients without TIPS, possibly reflecting subclinical LV dysfunction due to TIPS-induced increase in preload. A higher post-TIPS HPVG is associated with increased LV end-diastolic mass and attenuated LV global longitudinal strain, indicating LV hypertrophy and subclinical dysfunction with persistent portal hypertension.
**Limitations:** This is a retrospective study with a small sample size, warranting validation in a larger cohort.
**Funding for this study:** Not applicable.
**Ethics committee - additional information:** The study was approved by the local ethics committee of the medical association in Hamburg.
**Author Disclosures:**
Gerhard Adam: Nothing to disclose
Lieda Naimi: Nothing to disclose
Isabel Molwitz: Nothing to disclose
Martina Sterneck: Nothing to disclose
Peter Bannas: Nothing to disclose
Jennifer Erley: Nothing to disclose
Jennis Arenja: Nothing to disclose
Destina Gizem Aydemir: Nothing to disclose
Enver Tahir: Nothing to disclose

**Arrhythmic Burden, Myocardial Markers, and Long-term Survival in Distinct Cardiac Magnetic Resonance Subsets of Systemic Sclerosis**
*E. Moliterno*[1], L. Giarletta[1], G. Rovere[1], S. L. Bosello[1], G. De Luca[2], A. Tonutti[3], M. A. D'Agostino[1], L. Natale[1], R. Marano[1]; [1]Rome/IT, [2]Milan/IT, [3]Rozzano/IT
*(eleonora.moliterno@gmail.com)*

**Purpose or Learning Objective:** Cardiac involvement in Systemic Sclerosis (SSc) is widely recognized as heterogeneous and, when clinically evident, it is associated with a poor prognosis. Recently, 5 cardiac magnetic resonance (CMR) phenotypes in SSc have been identified (Knight DS et al. European Heart Journal 2023). These phenotypes do not align with the existing clinical subgroup classifications or autoantibody statuses, yet each has a distinct 5-year prognosis. Our objective is to test the long-term prognostic significance of this classification system in an external cohort and to compare ECG Holter monitor parameters, NT-proBNP and troponin T levels and 10-year survival outcomes across these groups.
**Methods or Background:** CMR assessments were conducted in 3 Italian tertiary centers on 143 consecutive SSc patients who presented symptoms of dyspnea, palpitations or chest pain. Based on the CMR findings, patients were categorized into 5 distinct groups: dilated right hearts with right ventricular failure (RVF); biventricular failure with dilatation and dysfunction (BVF), normal function with average cavity (NF-AC), small cavity (NF-SC), and large cavity (NF-LC).
**Results or Findings:** The distributions for NF-AC, NF-SC, NF-LC, BVF, and RVF were 46.2%, 22.4%, 14.0%, 14.0%, and 3.5%, respectively. Proportions of male patients and pulmonary function tests showed statistically significant differences across the subsets. Troponin T and NT-proBNP values were similar across all subsets. The NF-LC and RVF groups exhibited Left Bundle Branch Block (LBB) and ventricular ectopic beats (VEB) more frequently compared to other groups. There was a variation in 10-year survival rates across the groups, with patients in the RVF, NF-LC, and BVF categories showing poorer prognosis.

**Conclusion:** This data confirms the prognostic value of the proposed CMR subsets in an another European SSc cohort, highlighting that subsets with poorer prognosis are associated with a higher arrhythmic burden.
**Limitations:** No
**Funding for this study:** None
**Ethics committee - additional information:** No additional information
**Author Disclosures:**
Silvia Laura Bosello: Nothing to disclose
Giacomo De Luca: Nothing to disclose
Maria Antonietta D'Agostino: Nothing to disclose
Eleonora Moliterno: Nothing to disclose
Giuseppe Rovere: Nothing to disclose
Antonio Tonutti: Nothing to disclose
Luigi Natale: Nothing to disclose
Riccardo Marano: Nothing to disclose
Lorenzo Giarletta: Nothing to disclose

**Evaluating the Relationship Between Systemic Inflammation Index (SII), Systemic Inflammation Response Index (SIRI), and Coronary Calcium (Ca) Scoring in Atherosclerotic Cardiovascular Disease (ASCVD)**
I. T. Rakıcı, *K. F. Kaldırımoğlu*, A. S. Mahmutoğlu; Istanbul/TR
*(kemal.kaldirimoglu@gmail.com)*

**Purpose or Learning Objective:** Atherosclerosis is the main cause of coronary artery disease, with inflammation as a key factor. Studies link SII (SII = PlateletxNeutrophil/ Lymphocyte) and SIRI (SIRI = NeutrophilxMonocyte/ Lymphocyte) to inflamatory diseases and cardiovascular outcomes. This study examines the relationship between these markers, Ca scoring, and ASCVD.
**Methods or Background:** Images of 460 patients who underwent coronary CTA and calcium scoring were analyzed and divided into five groups: normal calcium score, calcium score 1-100, calcium score >100, calcium score of 0 with hypodense plaques, and calcium score of 0 with myocardial bridging. The SIRI and SII were calculated to assess significant differences and establish cutoff values.
**Results or Findings:** In the group with a calcium score greater than 100, the SIRI value was significantly higher than in all other groups, indicating an inflammatory process in ASCVD. A notable difference in SIRI values was observed between calcium scores of 1-100 and above 100, suggesting a correlation between higher scores and elevated inflammatory markers. In cases with a calcium score of 0 and myocardial bridging, no significant differences in inflammatory markers were found, suggesting no increase in the inflammatory process. The SII value showed no significant differences ($p>0.05$) among the other groups, except that it was significantly higher ($p<0.05$) in the calcium score > 100 and 1-100 groups compared to the calcium score 0 group with myocardial bridging. No significant differences in SIRI and SII values were observed in cases with a calcium score of 0 and hypodense plaques, indicating SIRI and SII values were insufficient to detect inflammation in these cases.
**Conclusion:** SIRI effectively discriminates between calcium score levels, whereas SII is not a reliable indicator. Future research is needed to determine clinically relevant cutoff values in this area.
**Limitations:** No limitations were identified.
**Funding for this study:** No funding was received for this study.
**Ethics committee - additional information:** The study was approved by the University of Health Sciences Istanbul Training and Research Hospital Clinical Research Ethics Committee (Decision number: 91).
**Author Disclosures:**
Abdullah Soydan Mahmutoğlu: Nothing to disclose
Ibrahim Taşkın Rakıcı: Nothing to disclose
Kemal Furkan Kaldırımoğlu: Nothing to disclose

**Myocardial strain trajectories by cardiac magnetic resonance and its relationship with myocardial fibrosis in Duchenne muscular dystrophy associated cardiomyopathy**
*H. Xu*, X. Ting; Cheng du/CN
*(xuhuayan2022.09_@scu.edu.cn)*

**Purpose or Learning Objective:** This study aims to explore the relationship between Myocardial strain trajectories and myocardial fibrosis by cardiac magnetic resonance (CMR) in Duchenne Muscular Dystrophy (DMD).
**Methods or Background:** DMD is a severe, X-linked genetic disorder characterized by progressive degeneration of skeletal muscle due to mutations in the dystrophin gene. And cardiomyopathy is becoming the leading cause of death. Cardiac magnetic resonance (CMR) and late gadolinium enhancement (LGE) are important tools in recognizing myocardial involvement, myocardial strain assessed by CMR feature tracking imaging (FT) may demonstrate early functional changes. Trajectories is a new method used to explore the dynamic changes of disease progression. Dynamic changes of myocardial strain using trajectories and myocardial tissue characteristics has not been investigated. We obtained myocardial strain parameters of more than 2 times by CMR FT method on 110 DMD patients. Global circumferential strain data was used to construct trajectory model. Late gadolinium enhancement progression was measured. Group-based trajectory modeling (GBTM) was performed to detect the trajectories of FT. Data t tests or one-way ANOVA adjusting for multiple comparisons.
**Results or Findings:** Three circumferential strain FT trajectory groups were identified as: up-down FT trajectory group(N=14), FT trajectory group(N=32), the steady FT trajectory group (N= 64). The occurrence rate of adverse cardiac event in the three trajectory analysis groups were significantly different(P=0.03).
**Conclusion:** This study is the first to analyze the myocardial strain trajectory of DMD patients. It provides further evidence of the correlation between FT and progression of myocardial fibrosis.
**Limitations:** The relatively small number of patients assigned to the 3 groups may result in insufficient statistical power.
**Funding for this study:** National funding of china
**Ethics committee - additional information:** Ethics committee of west china second university hospital
**Author Disclosures:**
Xu Ting: Nothing to disclose
Huayan Xu: Nothing to disclose

**08:00-09:00** **Research Stage 3**

### Research Presentation Session: Neuro

### RPS 1711

### Foetal and paediatric neuroimaging: unveiling the youthful brain

**Moderator**
D. Zlatareva; Sofia/BG
*(dorazlat@yahoo.com)*

**Even Low Levels of Prenatal Alcohol Exposure Can Induce Structural Alterations in Fetal Temporal Gyrification**
*P. Kienast*, M. Stuempflen, J. Tischer, A. Taymourtash, G. Langs, D. Prayer, G. Kasprian; Vienna/AT
*(patric.kienast@meduniwien.ac.at)*

**Purpose or Learning Objective:** Around 10-20% of women in Europe consume alcohol during pregnancy, leading to negative neurodevelopmental outcomes such as fetal alcohol spectrum disorders (FASD). Although the harmful impact of alcohol on neuronal development during pregnancy is well-established, studies on its effects during the prenatal phase remain limited. This study evaluates prenatal changes in fetal gyrification resulting from maternal alcohol consumption using automated surface measurements from fetal MRI data.
**Methods or Background:** This study involved 500 fetal MRI examinations where mothers were asked about their alcohol consumption habits using tools like PRAMS and TACE. The brains were denoised, motion-corrected, and segmented for 3D reconstruction. Gyrification indices were calculated, and asymmetry of the cerebral cortex was quantified. Neurotypical alcohol-affected cases were compared with healthy control cases.
**Results or Findings:** The study finally included 22 alcohol-exposed fetuses (mean gestational-age [GW] 27.61 ± 3.94 weeks) and 22 non-alcohol-exposed control fetuses (mean GW 27.57 ± 3.94 weeks), matched in a 1:1 ratio. Of the mothers in the alcohol-exposed group, 17 consumed only small amounts of alcohol (<14 grams per week). The typical asymmetry observed in the temporal lobes of the fetal brain was significantly diminished (p=0.48, 95% CI -2.24 to -0.01) in fetuses exposed to alcohol.
**Conclusion:** Alcohol consumption during pregnancy impacts the development of the fetal brain, with alterations observable in temporal brain asymmetry through fetal MRI. These findings align with studies linking prenatal brain asymmetry to language development in childhood, which is frequently impaired in children diagnosed with FASD. These structural changes are found even in cases of small alcohol consumption.
**Limitations:** A potential limitation of the study is the relatively small sample size, which may limit the ability to detect more subtle effects of low-level alcohol exposure on fetal brain development.
**Funding for this study:** The sponsor is the Medical University of Vienna.
**Ethics committee - additional information:** This study was approved by the local IRB.

**Author Disclosures:**
Georg Langs: Nothing to disclose
Athena Taymourtash: Nothing to disclose
Marlene Stuempflen: Nothing to disclose
Johannes Tischer: Nothing to disclose
Daniela Prayer: Nothing to disclose
Patric Kienast: Nothing to disclose
Gregor Kasprian: Nothing to disclose

**Intracranial Volumes of Preterm Born Infants and Age-matched Fetuses: A Comparison of In Utero versus Ex Utero Conditions**
*G. Abaci*, G. Biechele, S. Schläger, F. Obereisenbuchner, K. Förster, A. Flemmer, J. Ricke, A. Hilgendorff, S. Stöcklein; Munich/DE
*(gizem.abaci@med.uni-muenchen.de)*

**Purpose or Learning Objective:** The aim of our study was to compare intracranial volume (ICV), brain volume (BV), and the volumes of the inner cerebral fluid (iCSF) and outer cerebral fluid spaces (oCSF) of preterm brorn infants with age-matched fetuses and to assess the impact of postnatal therapies including ventilation.
**Methods or Background:** Preterm born infants and fetuses were matched for gestational age (± 4 days), ranging from 32 to 39 weeks' gestation (GW). 26 preterm infants and 26 age-matched fetuses without reported brain pathology underwent MRI. Anatomical T2-weighted brain images were analyzed by manual segmentation.
**Results or Findings:** Preterm infants were characterized by reduced ICV (p= 0.005), BV (p= 0.007) and oCSF volumes (p= 0.074) when compared to age-matched fetuses undergoing prenatal imaging, whereas ICV-corrected BV (BV/ICV, p= 0.385), oCSF (oCSF/ICV, p= 0.568) and total CSF (total CSF/ICV, p= 0.274) did not differ significantly between the two groups. When considering postnatal therapies, more days of noninvasive positive pressure ventilation were associated with higher corrected values for oCSF (oCSF/ICV, p= 0.0392), and total CSF (total CSF/ICV, p= 0.0282) as well as lower corrected values for BV (BV/ICV, p= 0.0282).
**Conclusion:** Preterm infants showed reduced ICV, BV and oCSF volumes compared to age-matched fetuses in utero. CSF volumes in preterm infants were impacted by postnatal therapy, potentially indiciating that intrathoracic pressure might influence venous return and CSF reabsorption into the venous system. In conclusion, brain and CSF volumes are impacted by prematurity and associated therapeutic strategies. Potential mechanisms underlying these efffects and their implications for ex utero versus in utero brain development need to be further investigated.
**Limitations:** Retrospective design
**Funding for this study:** None
**Ethics committee - additional information:** Lmu 207-33
**Author Disclosures:**
Anne Hilgendorff: Nothing to disclose
Andreas Flemmer: Nothing to disclose
Gizem Abaci: Nothing to disclose
Sarah Schläger: Nothing to disclose
Kai Förster: Nothing to disclose
Sophia Stöcklein: Nothing to disclose
Florian Obereisenbuchner: Nothing to disclose
Gloria Biechele: Nothing to disclose
Jens Ricke: Nothing to disclose

**Machine Learning Analysis in Diffusion Kurtosis Imaging for Discriminating Pediatric Posterior Fossa Tumors**
*I. P. Voicu*, C. D'Orazio, E. Piccirilli, F. Dotta, P. Toma, G. S. Colafati; Rome/IT
*(paul.voicu@hotmail.it)*

**Purpose or Learning Objective:** Differentiating pediatric posterior fossa (PF) tumors medulloblastoma (MB), ependymoma (EP) and pilocytic astrocytoma (PA) remains relevant, because of prognostic implications. Diffusion kurtosis imaging (DKI) has not been investigated for pediatric PF tumors. Whole-tumor based (VOI) segmentations may improve repeatability compared to conventional region-of-interest (ROI) approaches. Our purpose was to compare repeatability between ROI and VOI measurements and assess DKI accuracy in discriminating among pediatric PF tumors with machine learning (ML) techniques.
**Methods or Background:** We retrospectively analyzed 34 children (M, F, mean age 7.48 years) with PF tumors who underwent preoperative MRI on a 3 Tesla magnet, including DKI. For each patient, two neuroradiologists segmented the whole solid tumor, the ROI of area of maximum tumor diameter and a small 5 mm ROI. The automated analysis pipeline included inter-observer variability (coefficient of variation- COV), and machine learning (ML) analyses. We estimated DKI accuracy with MANOVA analysis. We applied SMOTE to balance the dataset and performed a Random Forest (RF) machine learning classification analysis based on all DKI metrics from the SMOTE dataset ( 70/30 for the training and testing cohort).
**Results or Findings:** Tumor histology included medulloblastoma (15), pilocytic astrocytoma (14) and ependymoma (5). VOI-based measurements presented lower variability than ROI-based measurements. DKI-derived metrics discriminated accurately between PF tumors. SMOTE generated a balanced dataset with 45 instances (34 original and 11 synthetic, 10 EP and 1 PA). ML analysis yielded accuracy of 0.928, correctly predicting all but one lesion in the testing set.
**Conclusion:** VOI-based measurements presented improved repeatability compared to ROI-based measurements. ML techniques based on DKI-derived metrics are useful for discrimination of pediatric PF tumors.
**Limitations:** The study was approved by Institutional Review Board (IRB) of Bambino Gesù Children's Hos-pital (RAP-2024-0001). T
**Funding for this study:** None
**Ethics committee - additional information:** The study was approved by Institutional Review Board (IRB) of Bambino Gesù Children's Hos-pital (RAP-2024-0001).
**Author Disclosures:**
Claudia D'Orazio: Nothing to disclose
Paolo Toma: Nothing to disclose
Francesco Dotta: Nothing to disclose
Ioan Paul Voicu: Nothing to disclose
Giovanna Stefania Colafati: Nothing to disclose
Eleonora Piccirilli: Nothing to disclose

**Ultra-low dose computed tomography as an alternative to radiographic shunt series in the diagnosis of mechanical ventriculoperitoneal shunt complications – an ex vivo phantom study**
*B. Yildirim*, R. J. Serger, S. Zensen, H. Styczen, M. Schüßler, M. Forsting, C. Deuschl, M. Opitz, D. Bos; Essen/DE
*(berk.yildirim@uk-essen.de)*

**Purpose or Learning Objective:** The standard modality for the diagnosis of ventriculoperitoneal (VP) shunt failure is the radiographic shunt series (RSS). However, ultra-low dose computed tomography (ULD-CT) offers lower radiation exposure compared to RSS. The aim of this study was to compare the radiation doses of RSS and ULD-CT on photon-counting CT (PCCT) in the diagnosis of mechanical shunt failure in human phantom models and to demonstrate the diagnostic performance of ULD-CT.
**Methods or Background:** VP shunts with different mechanical complications were placed on human phantom models corresponding to ages of 1, 5, 10 and 30 years. RSS and ULD-CT on PCCT scans based on topograms with tube currents ranging from 10 and 55 mAs (Sn100 kV) were performed on each phantom. Effective doses of RSS in pediatric phantoms were estimated by using the conversion factors of Seidenbusch (2006, 2008 and 2009). Effective doses of ULD-CT were estimated according to ICRP 103.
**Results or Findings:** ULD-CT demonstrated lower effective doses compared to RSS for phantoms representing ages of 5, 10 and 30 years, while successfully detecting mechanical VP shunt complications in all cases. However, higher effective doses were assessed for ULD-CT scans of the 1-year phantom in comparison to RSS. The effective doses for radiographic RSS and ULD-CT (using the lowest dose by utilizing 10 mAs topograms), respectively, were as follows: 1 year: 0.056 vs. 0.104 mSv; 5 year: 0.186 vs. 0.092 mSv; 10 year: 0.240 vs. 0.082 mSv; 30 year: 0.641 vs. 0.050 mSv.
**Conclusion:** ULD-CT is a potentially superior alternative to radiographic shunt series for the detection of mechanical VP shunt complications in patients aged 5 years and above, which is particularly relevant in children due to reduction of the radiation risk.
**Limitations:** Phantom study.
**Funding for this study:** No funding was received for this study.
**Ethics committee - additional information:** Ethics committee approval was not required.
**Author Disclosures:**
Michael Forsting: Nothing to disclose
Maximilian Schüßler: Nothing to disclose
Hanna Styczen: Nothing to disclose
Denise Bos: Nothing to disclose
Cornelius Deuschl: Nothing to disclose
Sebastian Zensen: Nothing to disclose
Raya Juliane Serger: Nothing to disclose
Berk Yildirim: Nothing to disclose
Marcel Opitz: Nothing to disclose

**Living in a gold mining community: Assessing 3rd trimester estimated foetal weight, prevalence of multimorbidity across body systems, and a 6-year trend in birth outcomes**

*A. D. Piersson*[1], E. K. Effah[2], B. Brusah[3], S. T. Quartei[4], H. Mumuni[5], A. Akanchimayoro[3], L. Jones[6], K. Dzefi-Tettey[4], M. R. Asamani[7]; [1]York/UK, [2]Obuasi/GH, [3]Cape Coast/GH, [4]Accra/GH, [5]Tamale/GH, [6]Virginia, VA/US, [7]Michigan, MI/US
*(a.piersson@yorksj.ac.uk)*

**Purpose or Learning Objective:** In this study, we assessed the following – 3rd trimester estimated foetal weight (EFW), prevalence of multimorbidity across body systems, and a 6-year trend in birth outcomes in a mining community, exposed to Galamsey, a term used to define illegal small-scale and artisanal gold mining.
**Methods or Background:** We compared 3rd trimester estimated foetal weight on ultrasound systems between pregnant women (≥ 18 years old) with singleton pregnancies living in a mining (n = 181) and non-mining communities (n = 260). Then, we compared health problems across body systems in another two groups (mining, n = 507; non-mining, n = 127). Further, we analyzed a 6-year trend in birth outcomes in the mining community located in Obuasi, Ashanti Region in Ghana.
**Results or Findings:** Overall, women living in the mining area showed significantly higher 3rd-trimester EFW than those living in a non-mining community (mining: 2.95+/-0.66 kg; non-mining: 2.72+/-0.04 kg; p = 0.0002). Women living in the mining community showed a higher prevalence of morbidities in 8 body regions/conditions than those living in the non-mining community. A sub-analysis of the prevalence of the 8 body regions/conditions among women in the two communities showed significant difference (p = 0.016). A 6-year trend in birth outcomes showed increasing rates of stillbirth varying between 1.69% and 2.90%, a relatively low record of congenital anomalies, and a relatively high record of newborn complications.
**Conclusion:** Our preliminary findings suggest a potential link between maternal exposure to mining-related environmental factors and adverse perinatal outcomes, including increased foetal growth, a higher prevalence of maternal morbidities, rising stillbirth rates, and increased newborn complications. Further study is warranted to investigate the underlying mechanisms and long-term health implications for both mother and child.
**Limitations:** Our study did not provide quantitative maternal blood or environmental measurements of heavy metal exposures.
**Funding for this study:** None
**Ethics committee - additional information:** Ethical Clearance - UCCIRB/EXT/2022/30
**Author Disclosures:**
Lashell Jones: Nothing to disclose
Klenam Dzefi-Tettey: Nothing to disclose
Benjamin Brusah: Nothing to disclose
Albert Dayor Piersson: Nothing to disclose
Augustine Akanchimayoro: Nothing to disclose
Emmanuel Kofi Effah: Nothing to disclose
Mercedes Rowe Asamani: Nothing to disclose
Hanifatu Mumuni: Nothing to disclose
Sarah Teiko Quartei: Nothing to disclose

**08:00-09:00** **Research Stage 4**

### Research Presentation Session: Imaging Informatics and Artificial Intelligence

### RPS 1705

### Human and machine factors in artificial intelligence

**Moderator**
E. Kotter; Freiburg/DE
*(elmar.kotter@uniklinik-freiburg.de)*
**Author Disclosures:**
Elmar Kotter: Advisory Board: contextflow; Author: thieme, springer; Share Holder: Siemens, Bayer; Speaker: siemens healthineers, bayer

Saturday

**Visual acuity among participants of the European Congress of Radiology 2024: should visual assessment be recommended for radiologists?**

*T. Van Nijnatten*[1], M. Smidt[1], J. E. Wildberger[1], M. Fuchsjäger[2], F. J. Gilbert[3], F. Pediconi[4], R. G. H. Beets-Tan[5], F. Van Den Biggelaar[1], C. Catalano[4]; [1]Maastricht/NL, [2]Graz/AT, [3]Cambridge/UK, [4]Rome/IT, [5]Amsterdam/NL
*(Thiemovn@gmail.com)*

**Purpose or Learning Objective:** Currently there is no recommendation regarding visual assessment for radiologists. The aim was to evaluate visual acuity among participants of the European Congress of Radiology (ECR) 2024.
**Methods or Background:** Participants of ECR 2024 organized by the European Society of Radiology (February 28th-March 3rd 2024; Vienna, Austria) were asked to participate in an on-site visual assessment. Medical ethical approval was obtained. Each participant signed written informed consent. The assessment consisted of vision chart reading (Sloan ETDRS Vision Chart) at 66 cm. Afterwards, auto-refraction was performed to determine refractive error. Finally, participants re-read a different vision chart, using on-site glasses to correct for refractive error. A LogMAR score of 0.0 (i.e. Snellen equivalent 1.0/100%) was considered an adequate visual acuity for radiology reporting.
**Results or Findings:** 321 participants completed on-site visual assessment (41% (132/321) male and 59% (189/321) female). Reported professions were 114 consultant or board certified radiologists (36%), 121 radiology residents (38%), 24 PhD students in radiology (7%), 37 medical students (12%), 11 radiographers (3%), 3 medical physicists (1%) and 11 others (3%). Mean age was 30 years (range: 18-69). Of the 57% (182/321) participants who wore glasses/contact lenses, 171 (94%) wore glasses/contact lenses during image interpretation tasks. Among all participants, 24 participants (7.5%) did not achieve logMAR score of 0.0 when reading the first vision chart. After auto-refraction measurements, 11 out of these 24 participants improved to a logMAR score of at least 0.0 using on-site glasses to correct for the refractive error.
**Conclusion:** A considerable percentage of radiologists has accurate visual acuity at a radiology reporting distance of 66 cm. Yet, 7.5% of the participants of the on-site visual assessment did not achieve an adequate vision score. Visual assessment could be considered among radiologists.
**Limitations:** N/a
**Funding for this study:** N/a
**Ethics committee - additional information:** Metc 2023-0249.
**Author Disclosures:**
Marjolein Smidt: Nothing to disclose
Thiemo Van Nijnatten: Nothing to disclose
Michael Fuchsjäger: Nothing to disclose
Fiona J. Gilbert: Nothing to disclose
Joachim E. Wildberger: Nothing to disclose
Regina G. H. Beets-Tan: Nothing to disclose
Frank Van Den Biggelaar: Nothing to disclose
Federica Pediconi: Nothing to disclose
Carlo Catalano: Nothing to disclose

**Exploring how AI influences human gaze behaviour during mammography reading**

A. Taib[1], G. Partridge[1], P. Phillips[2], J. James[1], *Y. Chen*[1]; [1]Nottingham/UK, [2]Lancaster/UK
*(yan.chen@nottingham.ac.uk)*

**Purpose or Learning Objective:** Most studies assess artificial intelligence's (AI) diagnostic performance in mammography, but few examine its impact on human reader behaviour and decision-making. The aim was to investigate the influence of AI prompts on human performance, visual search patterns, reader confidence and look for any interaction with readers individual personality traits when reading standard 2D screening mammograms.
**Methods or Background:** In this paired reader study, eight readers working in the UK breast screening programme evaluated a set of 60 anonymised mammograms with and without AI (Lunit Insight MMG). Cases with false negative and false positive AI prompts were incorporated into the test set containing a mix of normal, benign and malignant cases. Readers initially assessed the mammograms without AI while their visual search behaviour was monitored using eye-tracking equipment (SmartEyePro). After a six-week washout period, readers reviewed the same cases with the addition of AI with eye tracking. For each read, clinical opinion was recorded using a scale (1-normal, 2-benign, 3-indeterminate, 4-suspicious, 5-malignant) and entered onto the Personal Performance in Mammographic Screening (PERFORMS) website. Each reader completed a specially designed psychological questionnaire.
**Results or Findings:** A paired analysis at breast level, using pathological data as the 'ground-truth', determined how correct and incorrect AI prompts influenced diagnostic accuracy, gaze behaviour and reader confidence. The effect of different reader personality traits was also correlated with these outcomes.
**Conclusion:** There is little evidence exploring how AI influences a reader's visual search patterns during mammography interpretation. This pilot study provides an insight into changes in reader behaviour when using AI and will help guide further studies and recommendations on how radiologists should interact with AI when interpreting screening mammography.
**Limitations:** The limited sample of human readers may lead to type two error.
**Funding for this study:** By Lunit.
**Ethics committee - additional information:** The study was approved by the institutional review board.
**Author Disclosures:**
George Partridge: Nothing to disclose
Peter Phillips: Nothing to disclose
Adnan Taib: Nothing to disclose
Jonathan James: Nothing to disclose
Yan Chen: Nothing to disclose

**Narrow AI as Double Edged Sword: effects of using AI for fracture detection on distributing attention among focal and peripheral tasks**

*F. Mol*[1], D. Pourhassan Gilkalaye[1], M. H. Rezazade Mehrizi[1], W. Grootjans[2]; [1]Amsterdam/NL, [2]Leiden/NL
*(f.p.j.mol@gmail.com)*

**Purpose or Learning Objective:** Exploring the impact of using AI on distributing attention among focal tasks (diagnosis) versus peripheral tasks (detection of incidental findings) in shoulder radiographs by radiographers.
**Methods or Background:** 17 radiographers evaluated 255 shoulder radiographs from 15 outpatient trauma patients, with fracture detection as primary task. To assess impact of AI assistance, 170 cases were analysed using commercially available AI software for fracture detection (Gleamer). Both fracture (204) and non-fracture (51) cases were included. Additionally, 102 cases had incidental findings (e.g., pulmonary nodules, bone cysts, rotator cuff calcifications). Eye-tracking (Tobii 5) and mouse-tracking (in-house) software were used to measure attention distribution. Data are presented as mean ± standard deviation, and statistical differences were assessed using Wilcoxon signed-rank test, with significance defined as $p<0.05$.
**Results or Findings:** Participants spent more time on cases with AI assistance (n=170), averaging 47±28.24 seconds, compared to 33 ± 28.24 seconds without AI ($p < .001$). Mouse clicks were 3.85 ± 10.24 without AI, and 12.09 ± 10.24 with AI ($p < .001$). Eye-tracking data indicated greater attention to peripheral tasks with AI assistance ($p = 0.024$), while fracture detection was higher without AI ($p = 0.038$). Radiographers mentioned 31% of incidental findings.
**Conclusion:** Use of narrow AI tools can increase sensitivity towards peripheral tasks, possibly resulting from enhanced cognitive availability by delegating the focal task to AI. Similarly, measured decrease in fracture detection with AI indicates decrease in attention towards the focal task. This positions narrow AI as a "double-edged sword": while automation can free up cognitive resources, it can lead to over-reliance and reduced attention to focal tasks.
**Limitations:** Real-world clinical environment may not be simulated completely in experimental setting. Eye and mouse tracking may not capture all attention distribution aspects.
**Funding for this study:** N.a.
**Ethics committee - additional information:** N.a.
**Author Disclosures:**
Dorsa Pourhassan Gilkalaye: Nothing to disclose
Ferdinand Mol: Nothing to disclose
Willem Grootjans: Nothing to disclose
Mohammad Hosein Rezazade Mehrizi: Nothing to disclose

**Colour map recommendations for MR relaxometry**

*B. D. Wichtmann*[1], M. Fuderer[2], N. Desouza[3], F. Crameri[4], V. Gulani[5], N. Sollmann[6], S. Weingärtner[7], S. Mandija[2], X. Golay[3]; [1]Bonn/DE, [2]Utrecht/NL, [3]London/UK, [4]Bern/CH, [5]Ann Arbor, MI/US, [6]Ulm/DE, [7]Delft/NL
*(barbara.wichtmann@ukbonn.de)*

**Purpose or Learning Objective:** Quantitative imaging data may be colour coded and represented as a colour-map. However, commonly used schemes (e.g. rainbow, jet) lack perceptual uniformity, have the brightest colour mid-range and are not usable by colour-blind individuals. Furthermore, lack of standardization of colour-maps, makes comparisons across studies and institutions difficult and misleading. This work describes recently published recommendations for standardisation of MR relaxometry colour-maps (Fuderer, MRM 2024) in order to promote their adoption and drive the process for other biomarkers.
**Methods or Background:** Recommendations were generated in 4 Delphi rounds. A multidisciplinary committee devised questions on key colour-map features, including the colour choice for T1/T2 maps, even colour gradient contrast, high overall colour and lightness contrast, intuitive and constant gradient magnitude, and recognizability. Questions were circulated to the ISMRM quantitative imaging group and European subspecialist society representatives. Respondents received feedback after each round to aid consensus. Responses on a 9-point Likert scale were summarised to Agree, Neutral, Disagree categories. 75% consensus was the threshold for items reaching recommendation. The proposed colour maps were based on previous proposals (Griswold, ISMRM 2018) but modified for perceptual linearity and readability by colour-blind people.
**Results or Findings:** 58 experts responded to Round 1; 48 (45% medical, 47% physicists) completed all 4 rounds. There was consensus that the logarithm-processed Lipari colour-map for T1 and the logarithm-processed Navia colour-map for T2 were suitable. Colour bars were deemed mandatory as was a specific value indicating "invalidity". There was no consensus on whether to fix ranges by anatomy.
**Conclusion:** The logarithm-processed Lipari colour map for displaying T1 and R1 values and the logarithm-processed Navia colour-map for displaying T2, R2, T2* and R2* are recommended for use in scientific reports.
**Limitations:** Future work will focus on range recommendations.
**Funding for this study:** No funding was provided for this study.
**Ethics committee - additional information:** No patient-sensitive data were processed in this study.
**Author Disclosures:**
Nandita Desouza: Nothing to disclose
Vikas Gulani: Grant Recipient: I get research support from Siemens Healthineers. I have intellectual property licensed by Siemens Healthineers.
Fabio Crameri: Nothing to disclose
Miha Fuderer: Grant Recipient: NWO grant number 17986, which has partly been funded by the company Philips
Sebastian Weingärtner: Nothing to disclose
Barbara Daria Wichtmann: Speaker: I have given scientific presentations for Philips GmbH, Lilly Deutschland, and Bender group/b.e.imaging GmbH on unrelated topics for which I received monetary compensation.
Stefano Mandija: Nothing to disclose
Xavier Golay: Consultant: Bioxydyn Founder: Gold Standard Phantoms: Founder, shareholder and employee
Nico Sollmann: Nothing to disclose

**Evaluating the Impact of Explainable AI on Anchoring and Automation Biases in Mammography Interpretation**

*F. Pesapane*, L. Nicosia, S. Carriero, L. Mariano, A. C. Bozzini, A. Latronico, L. Meneghetti, F. Abbate, E. Cassano; Milan/IT
*(Filippopesapane@gmail.com)*

**Purpose or Learning Objective:** This study investigates how AI support influences diagnostic biases (anchoring and automation) among radiologists with varying experience levels in breast imaging. It evaluates whether explainable AI (XAI), using a heatmap, reduces these biases and improves diagnostic accuracy.
**Methods or Background:** Six radiologists (2 low experience: 0-5 years, 2 medium: 5-10 years, 2 high: >10 years) participated. Each assessed 200 mammograms across two phases: (1) AI BIRADS score presented before diagnosis (anchoring phase), and (2) AI score presented after an independent diagnosis (automation phase). A crossover design was used, with a 30% AI

Saturday error rate. Two AI conditions were tested: standard (score only) and explainable (score with heatmap). Diagnostic changes, accuracy, and bias frequency were recorded, with subgroup analyses based on experience.
**Results or Findings:** In the anchoring phase, radiologists altered their diagnoses in 180/400 cases (45%) when AI was incorrect; XAI reduced this to 100/400 (25%). In the automation phase, 220/400 correct diagnoses (55%) changed after AI input; XAI reduced this to 120/400 (30%). Low-experience radiologists showed higher susceptibility, particularly in automation (260/400, 65% change rate). XAI improved accuracy in this group by 80/400 cases (20%). Experienced radiologists demonstrated minimal bias reduction with XAI, indicating experience as a moderating factor.
**Conclusion:** XAI reduces anchoring and automation biases, especially for less experienced radiologists when AI errors are present. Tailored AI solutions with explainability are crucial for unbiased decision-making in breast imaging.
**Limitations:** This study involved a small sample size, which may limit generalizability. Future studies should expand the sample and explore the long-term impact of XAI on diagnostic confidence.
**Funding for this study:** N/A
**Ethics committee - additional information:** Code of approval: UID 4810
**Author Disclosures:**
Luciano Mariano: Nothing to disclose
Luca Nicosia: Nothing to disclose
Antuono Latronico: Nothing to disclose
Anna Carla Bozzini: Nothing to disclose
Serena Carriero: Nothing to disclose
Francesca Abbate: Nothing to disclose
Filippo Pesapane: Nothing to disclose
Enrico Cassano: Nothing to disclose
Lorenza Meneghetti: Nothing to disclose

**The diagnostic performance of an AI model in prostate cancer detection decreased significantly in reduced scan-quality of biparametric MRIs, while radiologists' performances did not decrease**
*E. H. P. Pooch*[1], G. Agrotis[1], A. Dehghanpour[2], R. G. H. Beets-Tan[1], T. Janssen[1], I. G. Schoots[1]; [1]Amsterdam/NL, [2]Rome/IT

**Purpose or Learning Objective:** To assess the diagnostic performance of artificial intelligence (AI) model and radiologists in detecting Grade Group (GG) ≧2 disease in prostate cancer suspected men, on diagnostic biparametric MRI (bpMRI) scans, considering variations in scan quality as assessed by PI-QUAL scores.
**Methods or Background:** A nnU-Net GG≧2 cancer segmentation model used 1500 bpMRI scans for training (cohort PI-CAI) and 89 scans for external validation (cohort PROMIS). The external cohort analysis included PI-RADSv2.1 assessment by two readers (R), while one assigned PI-QUALv1 (MRI-quality) scores. The outcome measurement was GG≧2 cancer, based on biopsies. MRI-positive scans were defined as PI-RADS 3-5 scores. The model's and radiologists' diagnostic performance (AUCs) were compared.
**Results or Findings:** Overall, the trained model (AUC=0.888) achieved an AUC of 0.652(0.525-0.760) during external validation. At reduced scan quality (PI-QUAL 1-3), the model's AUC dropped to 0.552(0.350-0.747), while at high-quality scans (PI-QUAL 4-5), the model's AUC improved to 0.720(0.556-0.855). In contrast, the AUCs of R1 and R2 were 0.733(0.631-0.829) and 0.711(0.614-0.803), respectively, showing a significant difference to the AI model in the reduced-quality group ($p<0.04$), but not significant in the high-quality group ($p>0.99$). The readers' AUCs did not drop at reduced scan quality (AUCs 0.723(0.576-0.875) and 0.727(0.576-0.862) and did not improve at high-quality scans (0.743(0.616-0.848) and 0.695(0.562-0.812)), respectively.
**Conclusion:** The diagnostic performance of the AI model differed significantly between reduced- and high-quality scans. In contrast, radiologists maintained consistent diagnostic accuracy. To ensure optimal performance, consistently high-quality MRI scans are required for a successful implementation of AI in clinical practice.
**Limitations:** Limited sample size and only one radiologist provided PI-QUAL scores, which may limit the findings' generalizability.
**Funding for this study:** No funding was received for this study.
**Ethics committee - additional information:** The study was made using public data.
**Author Disclosures:**
Georgios Agrotis: Nothing to disclose
Ailin Dehghanpour: Nothing to disclose
Eduardo H. P. Pooch: Nothing to disclose
Tomas Janssen: Nothing to disclose
Regina G. H. Beets-Tan: Nothing to disclose
Ivo Gerardus Schoots: Nothing to disclose

**Variability of classification labels is an important barrier to effective comparison of artificial intelligence software between vendors**
*A. Maiter*, E. Hesketh, P. Metherall, J. Taylor, S. Alabed, K. Dwivedi, W. Tindale, A. Swift, C. S. Johns; Sheffield/UK

**Purpose or Learning Objective:** Comparing the performance of AI software between different vendors is important for guiding procurement and deployment decisions. This requires consistency in how software outputs are presented. We assessed the number, nature and terminology of classification labels provided by commercially available software from seven vendors for the evaluation of chest radiographs.
**Methods or Background:** The classification labels provided by the software from each vendor were appraised qualitatively and using descriptive statistics. Synonymous labels were reconciled by merging. Labels were categorised according to their intended purpose. Where relevant, label terminology was compared with the 2024 and 2008 Fleischner Society Glossary of Terms for Thoracic Imaging.
**Results or Findings:** The median number of labels per vendor was 17 (IQR 7 to 100). Most labels were for the detection of pathology (median 88%, IQR 74% to 94%); these varied from non-specific signs (e.g. 'bronchovascular markings') to specific diagnoses (e.g. 'sarcoidosis'). In some cases, individual vendors provided multiple labels with overlapping meanings (e.g. 'consolidation', 'air bronchogram', 'air space opacification' and 'alveolar pattern opacity'). Fewer labels were for the detection of devices (median 12%, IQR 0% to 23%); these also ranged from non-specific (e.g. 'catheter') to more specific with a decision on adequacy (e.g. 'suboptimal nasogastric tube'). The median concordance of terminology with the Fleischner Society Glossary was 58% (IQR 50% to 72%).
**Conclusion:** We identified considerable variability in the labels provided by software, including inconsistent adherence to established terminology. This represents a barrier to effective comparison of performance between vendors and potentially limits the clinical utility of software outputs. Our study highlights the need for better harmonisation of output labels across the AI field.
**Limitations:** The interpretation of classification labels can be subjective and may differ between assessors.
**Funding for this study:** This study was funded by the NHS South Yorkshire Integrated Care System.
**Ethics committee - additional information:** This study did not involve patient data, and no ethics committee approval was required.
**Author Disclosures:**
Jonathan Taylor: Nothing to disclose
Andrew Swift: Nothing to disclose
Ahmed Maiter: Other: Bayer
Chris S Johns: Nothing to disclose
Samer Alabed: Nothing to disclose
Peter Metherall: Nothing to disclose
Eleanor Hesketh: Nothing to disclose
Krit Dwivedi: Nothing to disclose
Wendy Tindale: Nothing to disclose

**Rethinking Radiology Reports: The Perspective of Referring Physicians**
*P. Reschke*, L. D. Gruenewald, V. Koch, E. Höhne, T. J. Vogl, J. Gotta; Frankfurt/DE

**Purpose or Learning Objective:** High report quality and completeness are essential for efficient patient management. However, the clarity and comprehensiveness of radiology reports are often a point of contention among referring physicians. This study aims to assess referring physicians' perspectives on the quality and utility of radiology reports in clinical practice.
**Methods or Background:** A prospective, anonymous online survey was conducted from June 2023 to June 2024, targeting practicing physicians in Germany, including internists, general practitioners, surgeons.
**Results or Findings:** A total of 149 participants were included: 40% internists, 35,8% general practitioners, 24,2% surgeons. The average satisfaction score for radiology report completeness was 34.4 (±42.3) on a scale from -100 to +100. The primary reasons for incomplete reports were a lack of clinical context (33.3%), missing prior imaging (18.6%), inappropriate imaging techniques (13.8%) and unclear clinical questions (11,6%). Nearly half of the respondents (48.9%) preferred concise reports, while 35.7% opted for medium-length, and only 15.4% favored detailed reports. A majority of participants preferred semi- or fully structured reporting formats (92.5%), with free-text being rarely chosen (7.5%), showing no significant differences across specialties ($p=0.08$). Most participants (84.1%) found imaging in interdisciplinary case conferences valuable for understanding reports, with 35.9% rating them as "very helpful."
**Conclusion:** Referring physicians strongly prefer structured reporting and concise radiology reports. Integrating imaging into interdisciplinary meetings can further improve report comprehension.

**Limitations:** As the survey was conducted exclusively in Germany, the results may not be directly applicable to other healthcare systems or international settings, where clinical practices and communication standards may differ. The study focused on a limited number of specialties (internists, general practitioners, surgeons), potentially overlooking the perspectives of other key stakeholders such as neurologists, oncologists, or emergency medicine physicians.
**Funding for this study:** None.
**Ethics committee - additional information:** Not applicable
**Author Disclosures:**
Leon D. Gruenewald: Nothing to disclose
Vitali Koch: Nothing to disclose
Philipp Reschke: Nothing to disclose
Thomas J. Vogl: Nothing to disclose
Jennifer Gotta: Nothing to disclose
Elena Höhne: Nothing to disclose

**08:00-09:00** **Room G1**

### Research Presentation Session: Radiographers

### RPS 1714

### Advancements in MR: safety, patient care, and technological innovation

**Moderators**
S. B. Grover; Greater Noida/IN
*(shabnamgrover@yahoo.com)*
C. Tsiotsios; Limassol/CY
*(crtsiotsios@gmail.com)*

### MRI safety education across Europe: perspectives on teaching and assessment

J. Mcnulty[1], A. Cradock[1], A. Mcgee[1], *A. De Bock*[2], J. L. Portelli[3], A. England[4]; [1]Dublin/IE, [2]Brussels/BE, [3]Msida/MT, [4]Utrecht/NL

**Purpose or Learning Objective:** Little has been published on the details of curricular content for radiographers in MRI safety despite the EFRS MRSO role descriptor benchmark document being published in 2021. This survey aimed to capture the current status quo in terms of MRI safety education for radiographers across Europe in terms of content, pedagogical and assessment approaches, challenges, and opportunities.
**Methods or Background:** An online survey of educational institutions (EIs) involved in the delivery of MRI safety education to radiographers was designed. The survey was distributed via the EFRS to all member EIs. Additionally, it was shared with all member national societies and to committee and working group members for onward distribution to EIs.
**Results or Findings:** Responses were received from 69 EIs. 58 EIs confirmed they deliver MRI safety education to radiographers (23 to undergraduate (UG) medical imaging (MI) students, 23 to UG combined MI and radiotherapy (RT) students, 5 to UG RT students, and 22 to Masters students. The most common MRI safety teaching methods for postgraduates (PGs) were in-person lectures (70.8%), small group discussions (61.9%), review of MRI safety scenarios (61.9%), and clinical placements (61.9%). The MRI safety contact hours for PGs averaged just 6 (range: 0-30 hours). For PGs, at the 'advanced level', the most infrequently taught topics were 'Digital data safety' (27.8%) and 'Special MRI systems' (33.3%). 65.2% of EIs were either 'very satisfied' or 'satisfied' with their PG MRI safety education provision.
**Conclusion:** Significant heterogeneity in content and levels of delivery of MRI safety education for radiographers was evident and highlights the potential for a common MRSO curriculum.
**Limitations:** This was a convenience sample of EIs across Europe accessed through the EFRS network and thus may not be representative of the population as a whole.
**Funding for this study:** The ECSO-MRI Project is co-funded by the Erasmus+ Programme of the European Union (KA220: 2022-1-BE02-KA220-HED-000085873).
**Ethics committee - additional information:** Due to the nature of this study and the target population, the University College Dublin Human Research Ethics Committee – Sciences granted a formal Ethics Exemption (Reference: LS-LR-24-203-McNulty).
**Author Disclosures:**
Jonathan Loui Portelli: Nothing to disclose
Andrea Cradock: Nothing to disclose
Anke De Bock: Nothing to disclose
Jonathan Mcnulty: Nothing to disclose
Allison Mcgee: Nothing to disclose
Andrew England: Nothing to disclose

### Vetting MR referrals, are radiographers as good as radiologists?

*E. Kjelle*, I. Ø. Brandsæter, J. Porthun, B. M. Hofmann; Gjøvik/NO
*(elin.kjelle@usn.no)*

**Purpose or Learning Objective:** This study compares radiographers' and radiologists' vetting of justification for MRI referrals for low back pain and headaches.
**Methods or Background:** 360 lower back MR referrals and 353 brain MR referrals in adult patients were collected from private imaging centers in Norway. Four experienced radiologists and four experienced radiographers vetted the referrals using the Choosing Wisely recommendations for patients with low back pain and uncomplicated headaches. The assessors had three alternatives: 1)Justified, 2)Unjustified, and 3)Need more information. Data was analyzed using descriptive statistics, chi-square test to compare groups, and Gwen's AC2 for inter-rater agreement analysis. Significant level was $p<0.05$
**Results or Findings:** On average, in brain MR, the radiographers rated 52% of the referrals as justified, compared to 53% among the radiologists. The unjustified rate was 32% among radiographers and 41% among radiologists. In the category need more information, the rate was 15% for radiographers and 6% for radiologists. The difference was statistically significant, $p<0.001$. The interrater agreement was moderate 0.45 (95% CI:0.38-0.52) among the radiographers and good 0.71 (95% CI:0.68-0.75) among the radiologists. In lower back MRI, the justified rate was 65% for radiographers and 59% among radiologists. The unjustified rate was lower among the radiographers (18%) than among radiologists (26%). While the need more information rate was higher among radiographers. The difference was statistically significant, $p<0.001$. The interrater variability was moderate in both groups, with 0.60 (95% CI:0.53-0.66) among the radiographers and 0.56 (95% CI:0.49-0.62) among the radiologists.
**Conclusion:** Radiographers need more referral vetting training to avoid performing unjustified imaging. In this study, radiographers often required more information or rated a referral as justified compared to radiologists.
**Limitations:** The general clinical practices and experience could influence the assessors' vetting; thus, these results are not necessarily generalizable.
**Funding for this study:** This study was funded by the Norwegian Research Council (Project number 302503).
**Ethics committee - additional information:** Regional Committees for Medical and Health Research Ethics ref.no. 378396
**Author Disclosures:**
Ingrid Øfsti Brandsæter: Nothing to disclose
Bjørn Morten Hofmann: Nothing to disclose
Elin Kjelle: Nothing to disclose
Jan Porthun: Nothing to disclose

### Assessing MRI referrals' appropriateness for low back pain post a radiology-initiated intervention

*C. C. Chilanga*[1], M. Heggelund[1], E. Kjelle[2]; [1]Drammen/NO, [2]Gjøvik/NO
*(catherine.chilanga@usn.no)*

**Purpose or Learning Objective:** To evaluate a pilot intervention to reduce low-value Magnetic Resonance Imaging (MRI) referrals for Low Back Pain (LBP).
**Methods or Background:** The study evaluated MRI referrals for LBP before and after an intervention involving information campaigns and return letters to clinicians whose referrals were declined. Four radiologists and two radiographers assessed the referrals based on quality and justification. Justification was classified as justified, unjustified, or requiring more information. A point system rated quality on an 8-point scale, with scores above 5.5 marked as "good" and below 2.5 as "poor." Pre- and post-intervention variations were analysed using mixed model in Stata (Release 18). A p-value <.05 was considered significant.
**Results or Findings:** A total n= 300 referrals (150 pre- and post-intervention) were assessed. Post-intervention, rated justified referrals increased from 63% to 68%, while unjustified referrals decreased from 19% to 17%. Those needing more information decreased from 19% to 16%. Poor-quality referrals decreased to 4% post intervention. Mixed model analysis estimated justified referrals at 61% (95% CI: 55.8–65.5) pre-intervention and increased to 66% (95% CI: 61.5–70.9) post-intervention. Unjustified referrals fell from 20% (95% CI: 15.9–24.3) to 17% (95% CI: 13.5–21.4), Rated good quality referrals increased from 7% (95% CI: 4.6–9.0) to 8% (95% CI: 5.5–10.7), poor-quality referrals decreased from 30% (95% CI: 25.4–35.3) to 27% (95% CI: 22.3–31.7). Variations were not statistically significant.

**Conclusion:** The intervention is ongoing and needs further evaluation. However, providing reasons for declined referrals can serve as an educational tool for clinicians and contribute to the reduction of low value MRI for LBP in radiology departments.
**Limitations:** Adherence to referrers' confidentiality during data sampling prevented confirmation of whether all post-intervention referrals originated from clinicians who received return letters; however, region-wide campaigns likely mitigated this issue.
**Funding for this study:** The Research council of Norway (Project number 302503)
**Ethics committee - additional information:** Regional Committees for Medical and Health Research Ethics (REK) reference number 378396 and Norwegian Agency for Shared Services in Education and Research (SIKT) reference 261461.
**Author Disclosures:**
Mina Heggelund: Nothing to disclose
Elin Kjelle: Nothing to disclose
Catherine Chilute Chilanga: Nothing to disclose

### White matter hyperintensities and silent brain infarcts in aortic valve repair: the PEARL Study

*M. Bono*[1], M. Zanardo[2], V. Bari[2], B. Cairo[2], A. Porta[2], F. Sardanelli[2], P. Vitali[2]; [1]Varese/IT, [2]Milan/IT
*(martinabono93@tiscali.it)*

**Purpose or Learning Objective:** We assessed the burden of white matter hyperintensities (WMH), a biomarker of chronic cerebrovascular disease, and quantified ischemic lesions using diffusion-weighted magnetic resonance imaging (DW-MRI) in patients undergoing surgical aortic valve replacement (SAVR) or transcatheter aortic valve implantation (TAVI). Our goal was to investigate whether a relationship exists between chronic cerebrovascular disease and acute ischemia in aortic valve repair patients.
**Methods or Background:** This prospective study involved brain MRI scans at 1.5-T performed within seven days following SAVR or TAVI. The semi-quantitative Fazekas scale was used to classify WMH severity into low, intermediate, and high categories of chronic cerebrovascular disease. WMHs and DWI-positive ischemic lesions were quantified using automatic segmentation by Quantib® ND and semi-automatic segmentation adjusted by a neuroradiologist and a trained radiographer.
**Results or Findings:** A total of 55 patients were included in the study, of whom 47 underwent SAVR (62±15 years) and 8 TAVI (83±3 years). The mean lesion count for SAVR patients was 2.11 ± 7.07, while the mean lesion count for TAVI patients was 7.25 ± 6.88(p = 0.081).An analysis of WMH using the Fazekas scale indicated that SAVR group had mean Fazekas score of 1.30 ± 0.69, while TAVI group 2.25 ± 0.71 (p = 0.053).
**Conclusion:** While TAVI patients tended to have a higher burden of chronic cerebrovascular disease as indicated by the Fazekas score, the lack of significant differences in ischemic lesion counts between SAVR and TAVI suggests that both procedures pose similar acute ischemic risks. The WMH burden may enable risk-stratification of patients who undergo TAVI/SAVR and identify those that would benefit most from the adoption of neuroprotective devices.
**Limitations:** Low number of TAVI patients enrolled.
**Funding for this study:** Ricerca Finalizzata Code: RF-2016-02361069.
**Ethics committee - additional information:** Ethics committee Lombardia
**Author Disclosures:**
Paolo Vitali: Nothing to disclose
Vlasta Bari: Nothing to disclose
Francesco Sardanelli: Nothing to disclose
Martina Bono: Nothing to disclose
Alberto Porta: Nothing to disclose
Moreno Zanardo: Nothing to disclose
Beatrice Cairo: Nothing to disclose

### Preliminary results on white matter hyperintensities and lesion counts in patients undergoing cardiac surgery with cardiopulmonary bypass: the PASCAL study

A. Nocita[1], M. Zanardo[2], V. Bari[2], B. Cairo[2], P. Singh[2], A. Porta[2], F. Sardanelli[2], P. Vitali[2], *M. Bono*[1]; [1]Varese/IT, [2]Milan/IT
*(martinabono93@tiscali.it)*

**Purpose or Learning Objective:** This preliminary study investigates changes in white matter hyperintensities (WMH) and lesion counts in patients undergoing cardiac surgery with cardiopulmonary bypass (CPB). The primary objective was to assess the differences in WMH volumes and lesion counts pre- and post-surgery, measured through brain magnetic resonance imaging (MRI) Fluid-Attenuated Inversion Recovery (FLAIR) 3D sequence.
**Methods or Background:** The first fifteen patients of the PASCAL study were included. Two brain MRI scans were acquired before the surgical intervention with cardiopulmonary bypass (pre), and after the surgical intervention (post) but within 10 days. Pre-operative and post-operative WMH volumes and lesion counts were evaluated using automatic segmentation by Quantib® ND and semi-automatic segmentation adjusted by a neuroradiologist and a trained radiographer. A t-test was conducted for statistical analysis, while reproducibility was evaluated using a Bland-Altman plot.
**Results or Findings:** The mean pre-surgical WMH volume was 0.49 $cm^3$, which increased slightly to 0.57 $cm^3$ post-surgery. A paired t-test showed a statistically significant difference between pre- and post-surgical volumes (p = 0.044). The lesion count analysis showed a mean pre-operative count of 13, increasing to 14 post-surgery. However, this difference was not statistically significant (p = 0.09). The Bland-Altman analysis for WMH volume demonstrated a mean difference of 0.04 $cm^3$ with limits of agreement ranging from -0.15 to 0.23 $cm^3$. For lesion count, the mean difference was 1 with limits of agreement from -5 to 7, indicating moderate reproducibility between the two methods.
**Conclusion:** The results suggest that while WMH volumes may increase after CPB surgery, the number of lesions does not significantly change, although the sample size limits the generalisability of these findings. Definitive results will be obtained once the estimated sample size is reached.
**Limitations:** Preliminary results.
**Funding for this study:** Ricerca Finalizzata Code: GR-2021-12372037.
**Ethics committee - additional information:** Ethics Committee Lombardia 1, code: 06/INT/2023
**Author Disclosures:**
Paolo Vitali: Nothing to disclose
Alba Nocita: Nothing to disclose
Vlasta Bari: Nothing to disclose
Pavandeep Singh: Nothing to disclose
Francesco Sardanelli: Nothing to disclose
Martina Bono: Nothing to disclose
Alberto Porta: Nothing to disclose
Moreno Zanardo: Nothing to disclose
Beatrice Cairo: Nothing to disclose

### Hardware as a Predictor of Anxiety in Patients Undergoing Magnetic Resonance Imaging Examinations

*D. A. A. Costa*, A. Grilo, E. Carolino, M. C. P. Ribeiro; Lisbon/PT
*(diogo2242@gmail.com)*

**Purpose or Learning Objective:** The objective of this study was to assess which type of Magnetic Resonance (MRI) examination, the coil used and the patient position (to scan the brain or the knee), most influence patient's anxiety levels.
**Methods or Background:** Hundred patients underwent MRI scan using a 1,5T Magneton Symphony by Siemens Healtineers. Fifty of them performed brain and the others knee scan. Before and after the MRI scan, the STAI Inventories Form Y-1 (state anxiety) and the Form Y-2 (trait anxiety) was applied. The physiological measurements of Blood Pressure and Heart Rate were collected at the beginning and at the end of scan.
**Results or Findings:** In brain studies, the initial mean anxiety levels ($\bar{x}$=37.28 ± 12.446) are higher than those collected after examination ($\bar{x}$=33.72 ± 13.389). In the knee examinations the initial mean anxiety values ($\bar{x}$=31.96 ± 10.681) are higher compared to the final values ($\bar{x}$=29.42 ± 8.094). When comparing brain and knee studies, the first shown higher initial anxiety levels ($\bar{x}$=37.28 ± 12.446) than patients who undergo knee scan ($\bar{x}$=31.96 ± 10.681). The mean values of Maximum BP ($\bar{x}$=130.06 ± 3.808) and Final HR ($\bar{x}$=125.10 ± 1.851), collected after brain studies, are higher than the values collected in knee ($\bar{x}$=88.24 ± 2.720) and ($\bar{x}$=81.12 ± 1.943).
**Conclusion:** Through Multiple Linear Regression (Stepwise method), the examination type and the patient's assessment before MRI are predictors of anxiety, as their probability of occurrence is low. Higher and significant differences were found at the beginning of MRI scan. The appliance of different transceiver coils, reduces the surrounding space, contributing to increasing the levels of state anxiety.
**Limitations:** Lack information about other pathologies. Higher periods of questions and evaluation
**Funding for this study:** Not Applied
**Ethics committee - additional information:** Approval by Ethical Commission of School of Health Technology with the reference CE-ESTeSL-Nº.107-2022
**Author Disclosures:**
Ana Grilo: Nothing to disclose
Elisabete Carolino: Nothing to disclose
Diogo André Arrais Costa: Nothing to disclose
Margarida Carmo Pinto Ribeiro: Nothing to disclose

**Educational background in MRI Safety of healthcare professionals working in MRI departments: Insights from the ECSO-MRI Project**

J. Scheurleer[1], A. V. Diepen[1], *A. De Bock*[2], H. Bijwaard[1]; [1]Haarlem/NL, [2]Brussels/BE

**Purpose or Learning Objective:** This study aimed to assess the educational needs and preferences regarding MRI safety training among healthcare professionals working in MRI departments. Findings aimed to support the development of a standardised European MRI Safety Officer (MRSO) curriculum as part of the funded ECSO-MRI project.
**Methods or Background:** A survey among radiographers, radiologists, and other healthcare professionals across European MRI departments assessed educational backgrounds, MRI safety responsibilities, and training preferences, with 313 responses analysed. The study received local ethics committee approval and confirmed European data security requirements.
**Results or Findings:** Of the respondents, 71.6% had clinical experience in MRI, but 49.5% did not regularly update their MRI safety knowledge. Informal peer-to-peer training was the most commonly used learning method (62.9%). Hands-on training was the most preferred approach (89.5%). Additionally, 70% of respondents considered the development of Standard Operating Procedures (SoP) an advanced MRI safety topic. Furthermore, 40% highlighted the need for formal basic training for all radiographers working with MRI, while 63% indicated a need for formal advanced training for key users of MRI. Only 31% reported following local MRI safety guidelines, and 18.5% adhered to international standards, underscoring the need for more consistent safety protocols.
**Conclusion:** Significant gaps in MRI safety education, particularly in advanced training, were identified. The ECSO-MRI project aims to address these by developing a standardized curriculum in collaboration with European institutions.
**Limitations:** The reliance on self-reported data may introduce bias in assessing actual MRI safety practices and training needs.
**Funding for this study:** This study was funded by the European Union’s Erasmus+ program.
**Ethics committee - additional information:** Ethics Committee of University College Dublin Research Ethics Research Ethics Reference Number is: LS-LR-24-220-McNulty
**Author Disclosures:**
Anja Van Diepen: Nothing to disclose
Anke De Bock: Nothing to disclose
Jelle Scheurleer: Nothing to disclose
Harmen Bijwaard: Nothing to disclose

**Radiographers’ Knowledge and Attitudes differences towards Cardiac Implant Patients between radiographers in Magnetic Resonance Imaging**

C. Maloney, A. England, N. Moore, R. Young, G. A. Curran, *M. F. Mcentee*; Cork/IE
*(mark.mcentee@ucc.ie)*

**Purpose or Learning Objective:** Radiographers in magnetic resonance imaging (MRI) are at the forefront of patient safety, and patients with cardiac implantable electronic devices (CIED) have been referred for MRI imaging more frequently in recent years. A rise in MR-conditional devices has resulted in CIED patients accessing MRI, and previously patients with CIEDs were classed as absolute contraindications. However, the magnetic fields from the MRI scanner can interfere with CIEDs causing damage to the device itself and/or the patient. This study evaluates CIED specific knowledge, confidence levels and attitudes amongst radiographers in MRI towards cardiac implant patients.
**Methods or Background:** A quantitative, online survey was conducted amongst qualified radiographers in MRI internationally to evaluate participants MRI qualification status, what CIED specific knowledge is held by radiographers, confidence levels amongst radiographers scanning CIED patients, and determine common attitudes amongst radiographers towards implant patients who present for an MRI scan.
**Results or Findings:** 90 responses were recorded and overall, and 58% of participants held a postgraduate degree in MRI while 42% did not. 73% of radiographers highlighted they had opportunities to update their MRI safety in the work setting, while only 36% had opportunities to update their cardiac implant safety knowledge. Overall, radiographers concluded that their MRI and CIED safety knowledge was sufficient and possessed adequate knowledge of the CIED and magnetic field relationship. However, evident disagreement on confidence statements on explaining MR field impacts on CIED functioning, having insufficient knowledge on device workings to confidently scan and conflicting safety results indicate that this area requires addressing.
**Conclusion:** Inclusion of specific CIED safety training for MRI radiographers should be recommended as educational topics to equip radiographers with the knowledge, skills and confidence to competently provide safe patient care.
**Limitations:** Survey distribution was in English.
**Funding for this study:** None
**Ethics committee - additional information:** Medical School Social Research Ethics Committee - University College Cork
**Author Disclosures:**
Mark F. Mcentee: Nothing to disclose
Chloe Maloney: Nothing to disclose
Niamh Moore: Nothing to disclose
Rena Young: Nothing to disclose
Gráinne Alison Curran: Nothing to disclose
Andrew England: Nothing to disclose

**09:30-11:00** **Research Stage 1**

### Research Presentation Session: Interventional Radiology

### RPS 1809

### What's new in genitourinary and female health interventions?

**Moderator**
V. Kostadinova; Ljubljana/SI

**P-RENAL: a new scoring system for the prediction of complications and recurrence in kidney malignancies percutaneous ablation**

*G. Ferrillo*, D. Poretti, N. Buffi, P. Casale, M. Francone, V. Pedicini; Milan/IT
*(giuseppe.ferrillo@humanitas.it)*

**Purpose or Learning Objective:** There is a lack of internationally validated scores for the assessment of outcomes in percutaneous ablation of kidney tumours. We designed the P-RENAL score and compared it to the surgical standard score (RENAL).
**Methods or Background:** We performed a retrospective analysis of 146 patients treated with RFA (N=90) or MWA (N=56) between 01/01/2016 and 31/03/2021 (minimum follow up of 6 months). RENALand p-RENAL were calculated in both populations. Primary outcomes were recurrence (presence of vital tumour >1 cm in the treated area) and complications (according to the CIRSE complications score). P-RENAL is derived from RENAL, it consists of 6 parameters, each assigned a value from 1 to 3: radius (<2 cm, 2-3cm, >3cm), exophytic area and nearness to the collecting system (identic to RENAL score), location (1 interpolar mass; 2 lower polar; 3 apical tumours), side (1 posterior lateral; 2 medial posterior; 3 medial anterior), proximity of external structure(>3 cm from an high risk structureas ileal or colic wall,renal artery, spleen or liver); 2 from 3 to 1 cm; 3 points if <1 cm.
**Results or Findings:** P-RENAL showed higher sensitivity than RENAL score in predicting recurrence after ablation of kidney tumours expecially with MWA (AUC 0.86). There was no association of the RENAL score with complications, while we observed an independent positive correlation between p-RENAL and the presence of grade > 2 complications.
**Conclusion:** P-RENAL has higher sensitivity than RENAL in detection of recurrence and complications in percutaneous ablation.
**Limitations:** Retrospective monocentric study.
**Funding for this study:** None
**Ethics committee - additional information:** Retrospective study board review.
**Author Disclosures:**
Vittorio Pedicini: Nothing to disclose
Nicolò Buffi: Nothing to disclose
Marco Francone: Nothing to disclose
Dario Poretti: Nothing to disclose
Paolo Casale: Nothing to disclose
Giuseppe Ferrillo: Nothing to disclose

**Thulium laser enucleation of the prostate versus prostatic artery embolization for big prostates above 80gms: a multicentre study**

*N. M. A. Attia*[1], M. A. El Hamid[2], M. Abd El Wahab[2], A. Salah[2]; [1]Assiut/EG, [2]Cairo/EG
*(nohamohamedali@yahoo.com)*

**Purpose or Learning Objective:** To compare the postoperative and functional outcomes of thulium laser enucleation of the prostate (ThuLEP) and prostatic artery embolization (PAE) for the treatment of large-volume benign prostate hyperplasia (BPH) > 80 ml.

**Methods or Background:** We performed a retrospective, multicentric study of 120 patients consecutively treated with THuLEP (60 patients) or PAE (60 patients) for symptomatic large BPH >80gms between March 2022 and March 2023. These two groups were compared by treatment response, postoperative recovery period and complication rate at baseline, 1, 3 and 6 months.
**Results or Findings:** Intraoperative complications were significantly higher in the THULEP group (13.5% vs 0%, p=0.006). There was no statistically significant difference in the postoperative complications between the two groups (10% vs 8.4%, p=0.287). There was significant delay of catheter removal in the PAE group with subsequent delay in hospital stay (25% vs 5%, p=0.002). International Prostate Symptom Score (4.4±0.9SD vs 10.7±1.5SD, p<0.001) and PSA (1.4±0.6SD vs 4.3±5.1SD, p<0.001) were significantly lower in the THuLEP versus PAE group after 6 months. Postvoid residual (PVR) was higher in the PAE group from baseline till 6months (12.5±3.9SD vs 20.35±4.8SD, p<0.001). Maximum urinary flow rate (Qmax) was significantly higher in the THULEP group from baseline (37.93 ±4SD vs 27.3±8.1SD, p<0.001). Erectile dysfunction was significantly lower in the PAE group after 3 months.
**Conclusion:** Both ThuLEP and PAE relieve lower urinary tract symptoms with high efficacy and safety, however ThuLEP was superior to PAE in improving the functional outcomes in the first 6months while PAE had lower intraoperative complications and erectile dysfunction.
**Limitations:** The quality of life was not assessed being a retrospective study. The sample size was relatively small. Longer follow-up time is needed to compare the durability of the symptomatic improvement from each procedure.
**Funding for this study:** None
**Ethics committee - additional information:** This study was approved by the Committee of Medical Ethics of the Faculty of Medicine Assiut University with IRB no: 04-2024-300429
**Author Disclosures:**
Mahmoud A. El Hamid: Nothing to disclose
Noha Mohamed Ali Attia: Nothing to disclose
Ahmed Salah: Nothing to disclose
Mohamed Abd El Wahab: Nothing to disclose

### A decade of varicocele embolizations: Success and recurrence

*M. Ni Mhiochain De Grae*[1], M. Alkhattab[1], A. Alkadhimi[2], M. Springael[2], G. O' Sullivan[1]; [1]Galway/IE, [2]Dublin/IE
*(meadhbhnimhioch24@rcsi.ie)*

**Purpose or Learning Objective:** Varicoceles are vascular lesions of the pampiniform plexus, affecting 10-20% of the population and found in 40% of men with primary infertility and 80% with secondary infertility. (1-4) Pain occurs in 2-10% of cases. (1,5) Treatment options include conservative management, percutaneous embolization, or surgery. (6) Percutaneous embolization, introduced in the late 1970s, has a technical failure rate of around 13% and recurrence rates ranging from 0.6% to 45%. (7,8) This study evaluates success and recurrence rates of percutaneous varicocele embolizations at Galway University Hospital and Galway Clinic between 2009-2022, with a minimum follow-up of 18 months.
**Methods or Background:** Data collected included patient age, procedure date, access site, side of occurrence, previous interventions, treatment method, need for re-intervention, and recurrence rates. Technical success was defined as successful access to the gonadal vein and insertion of coil/sclerosant. Clinical success was assessed through follow-up consultations and ultrasound when available.
**Results or Findings:** The technical success rate was 94.7%, with 98.1% of successful embolizations achieving clinical success. Of 225 patients, 3.12% had prior failed surgeries, all treated successfully with IR, and 0.89% required surgical intervention. Telephone follow-ups (42.7% response rate) revealed a recurrence rate of 10.7% with an average follow-up of 8.03 years. The complication rate was 1.78%. Fertility outcomes showed 51.35% of patients had successful pregnancies, while 6.9% experienced ongoing fertility issues.
**Conclusion:** Our study of 225 patients is the largest to date, showing higher success rates (technical 94.7%, clinical 98.1%) and a 10.7% recurrence rate with long-term follow-up.
**Limitations:** This is a single centre retrospective review. 42.7% response to telephone consultation follow up.
**Funding for this study:** No funding.
**Ethics committee - additional information:** Local ethics approval in Galway University Hospital.
**Author Disclosures:**
Maia Springael: Nothing to disclose
Amor Alkadhimi: Nothing to disclose
Meadhbh Ni Mhiochain De Grae: Nothing to disclose
Gerry O' Sullivan: Nothing to disclose
Maha Alkhattab: Nothing to disclose

### The Clinical Study of Magnetic Resonance-Guided High-Intensity Focused Ultrasound (MRgFUS) Treatment for Adenomyosis

*Q. Zhang*; Shanghai/CN
*(q_zhang18@fudan.edu.cn)*

**Purpose or Learning Objective:** Objective: To investigate the safety and efficacy of Magnetic Resonance-Guided High-Intensity Focused Ultrasound (MRgFUS) treatment on adenomyosis.
**Methods or Background:** After receiving approval from local Ethics Committee, 62 patients with adenomyosis underwent MRgFUS treatment from October 2018 to March 2021. The 62 patients with adenomyosis were evaluated for adverse reactions during and after MRgFUS treatment. The lesion volume was measured before and after treatment using T2-weighted MRI images. Non-perfused volume (NPV) was calculated using T1-weighted contrast-enhanced images, then the ablation rate was calculated by dividing NPV by the lesion volume. Visual analogue scale (VAS) was used to score dysmenorrhea in adenomyosis patients before and after treatment.
**Results or Findings:** All 62 patients successfully underwent MRgFUS treatment without severe adverse reactions, achieving an average ablation rate of 69.12%±17.64%. The average lesion volume before ablation was 83.72±84.02 $cm^3$. At 3 months post-ablation, the average lesion volume was 62.66±66.31 $cm^3$, at 6 months it was 56.09±66.67 $cm^3$, and at 12 months it was 68.53±87.04 $cm^3$. There were statistically significant differences in lesion volume (p<0.05) before and after ablation treatment. The preoperative VAS score for the 62 patients was 6.68±2.13. At 3 months post-treatment, the average dysmenorrhea score was 3.69±1.95, at 6 months it was 2.72±1.40, and at 12 months it was 3.38±1.45. Post-treatment dysmenorrhea scores showed statistically significant decrease compared to pre-treatment (p<0.05).
**Conclusion:** MRgFUS is a safe, feasible and effective technique in treatment for adenomyosis.
**Limitations:** Serological examinations were not performed in patients with adenomyosis to assess changes in serologic markers after treatment in this study.
**Funding for this study:** Not applicable
**Ethics committee - additional information:** Ethics Committee of Huashan Hospital affiliated to Fudan University
**Author Disclosures:**
Qi Zhang: Nothing to disclose

### Treatment of cesarean scar pregnancy with systemic Methotrexate followed by intra-arterial Methotrexate injection and uterine arteries embolization: experience from a tertiary center

*C. Intrieri*, C. Lanza, S. A. Angileri, J. Tintori, C. Ercolino, A. M. Ierardi, G. Carrafiello, V. Chiarpenello; Milan/IT
*(cristinaintrieri95@gmail.com)*

**Purpose or Learning Objective:** The aim of this retrospective study is to evaluate the efficacy and the safety of the treatment of cesarean scar pregnancy (CSP) based on a single or cyclic systemic methotrexate (MTX) injection followed by intra-arterial MTX injection and uterine arteries embolization (UAE).
**Methods or Background:** A pregnancy's ectopic implantation on a uterine scar tissue following a previous cesarean section is defined as CSP. Because of its possible complication in uterine rupture, massive hemorrhage, placenta accreta spectrum, fetal and maternal death, CSP is considered a life-threatening condition. Therefore, treatment is fundamental.
The cooperating group of Radiologists and Gynecologists at our institution evaluated all patients affected by CSP from 2013 to 2023 (n=21) treated with systemic MTX injection followed by intra-arterial MTX injection and UAE with gelatin sponge. The inclusion criteria were: laboratory tests (levels of beta-human chorionic gonadotropin and beta-HCG over the normal threshold), ultrasound diagnosis of CSP, gestational age ≤8 weeks, stable hemodynamic, at least one previous pregnancy and at least one delivery through cesarean section. Exclusion criteria were: contraindications to MTX administration, the presence of abdominal hemorrhage and significant shock.
**Results or Findings:** Technical success of UAC and clinical success (reduction of the the sac size, lack of active vaginal re-bleeding and declining beta-HCG values) was achieved in all procedures (100%). No peri-procedural complications were recorded.
**Conclusion:** In our experience, the use of systemic MTX and intra-arterial injection of MTX plus UAC proved to be effective and safe to achieve CSP termination. The use of gelatin sponge after intra-arterial MTX administration proved to be effective also in preventing massive bleeding in case of successive uterine curettage.
**Limitations:** Controlled randomized trial with a larger sample is needed to assess the efficacy and the safety of treatment.
**Funding for this study:** No funding was received for this study.

**Ethics committee - additional information:** All subjects gave their consent for inclusion in the present study. The study was conducted in accordance with the World Medical Association (WMA) Declaration of Helsinki [WMA].
**Author Disclosures:**
Vittoria Chiarpenello: Nothing to disclose
Salvatore Alessio Angileri: Nothing to disclose
Anna Maria Ierardi: Nothing to disclose
Carolina Ercolino: Nothing to disclose
Jacopo Tintori: Nothing to disclose
Cristina Intrieri: Nothing to disclose
Carolina Lanza: Nothing to disclose
Gianpaolo Carrafiello: Nothing to disclose

**What is the safe observation period following image-guided renal biopsies?**
*F. Taylor*, K. Sehgal, M. Van Wees, K. Li, D. W. De Boo, L-A. Slater; Melbourne/AU
*(fergustay@gmail.com)*

**Purpose or Learning Objective:** To investigate the timing, type, and severity of complications following percutaneous, image-guided renal biopsy and to determine if the current observation period of 4 hours can be safely reduced.
**Methods or Background:** Consecutive image-guided percutaneous targeted and non-targeted renal biopsies performed between 2017-2022 in adult patients by radiology medical staff were included. The PACS imaging system and electronic medical records (EMR) were accessed to obtain relevant patient information and procedural reports. Retrospective analysis of the type and timing of complications was performed against patient demographics and biopsy-related variables. Probabilities were calculated for a range of observation periods to assess the proportion of complications identified by shortening the observation period from 4 hours.
**Results or Findings:** 332 percutaneous renal biopsies were included and 44 (13%) complications identified within the 4-hour observation. 29 complications were post-operative bleeding, of which 2 developed macroscopic haematuria, 25 peri-nephric haematoma and 2 had hemodynamic instability with either haematuria or peri-nephric hematoma. 64% of all complications occurred within the first hour, 86% occurring within 2 hours. Of the 6 complications occurring after 2 hours, 2 were post-operative bleeding in non-targeted renal biopsies requiring admission, the other 4 did not require additional observation/procedures.
**Conclusion:** The vast majority of complications after targeted and non-targeted renal biopsies tend to occur within the first 2 hours of observation. Complications that occurred after 2-hour observation were often pain related, something that can be overcome with a standardised post-operative analgesic regime. It may be possible to safely reduce observation times following image-guided targeted renal biopsies.
**Limitations:** Single institute dataset, including targeted and non-targeted biopsies. Biopsies were performed by Radiologists with varied experience and supervision, not ascertained during retrospective data collection. The low number of complications limits our analysis of associated factors.
**Funding for this study:** This research did not receive any specific grant from funding agencies in the public, commercial, or not-for-profit sectors. This study was not supported by any funding.
**Ethics committee - additional information:** All procedures performed in studies involving human participants were in accordance with the ethical standards of the institutional and/or national research committee and with the 1964 Helsinki declaration and its later amendments or comparable ethical standards. For this type of study formal consent is not required.
Ethical approval was obtained from the local Human Research Ethics Committee (HREC), reference number: RES-23-0000-015Q and the need for informed consent was waived.
**Author Disclosures:**
Fergus Taylor: Nothing to disclose
Matthew Van Wees: Nothing to disclose
Kunal Sehgal: Nothing to disclose
Diederick Willem De Boo: Nothing to disclose
Kenny Li: Nothing to disclose
Lee-Anne Slater: Nothing to disclose

**Prostate artery embolization (PAE): are there predictors indicating the technical success rate?**
*T. Lauenstein*, K. Scherschel, J. Boddenberg, F. Verfürth, N. Ziayee; Düsseldorf/DE
*(thomas.lauenstein@evk-duesseldorf.de)*

**Purpose or Learning Objective:** To assess if factors including patients' age, prior history of cardiovascular disease or prostate size have an impact on the technical success rate of PAE.
**Methods or Background:** Data of 163 consecutive patients undergoing PAE was analyzed. Technical success rate was defined as full when arteries of both prostate lobes could be probed, as partial (one lobe) or as absent . These findings were correlated with patients' age (range 53-93 years; mean 72 years), prior history of cardiovascular disease (present in 88 patients) and size of the prostate gland (range 36-265ml; mean 84ml).
**Results or Findings:** Bilateral PAE could be achieved in 125 patients. In 29 patients only one lobe was embolized and PAE failed to be successful in 9 patients. Patient's age failed to show a statistically significant difference between these groups (71.4±8.4 years for successful vs. 72.4±9.2 years for unsuccessful procedures, P=0.5576, unpaired ttest), as well as prior history of cardiovascular disease (52% for successful vs. 60% for unsuccessful procedures, P=0.4576. Fishers ttest). In contrast, prostate size was significantly higher in patients with successful bilobar PAE: 88±40ml for successful vs. 68±39ml for unsuccessful procedures (Mann -Whitney-test; p=0.0021). A consecutive ROC analysis revealed a prostate size of 79ml as a cut-off value for the success rate of bilobar PAE.
**Conclusion:** Prostate size was found to be the only predictor for a successful completion of PAE. Patients with volumes of the prostate gland <79ml should be informed to have a higher risk of technical therapy failure.
**Limitations:** Limitations of this study are related to the retrospective nature of data analysis.
**Funding for this study:** Not applicable
**Ethics committee - additional information:** Not applicable
**Author Disclosures:**
Frank Verfürth: Nothing to disclose
Thomas Lauenstein: Nothing to disclose
Naim Ziayee: Nothing to disclose
Katharina Scherschel: Nothing to disclose
Jörg Boddenberg: Nothing to disclose

**First intermediate term report of safety and outcomes of radiofrequency ablation of Renal tumours using an automated energy delivery-controlled system**
*G. Ferrillo*, E. Baldassarre, T. Sirugo, E. Lanza, S. Romano, N. Buffi, P. Casale, M. Francone, V. Pedicini; Milan/IT
*(giuseppe.ferrillo@humanitas.it)*

**Purpose or Learning Objective:** To determine the outcomes (Primary efficacy and safety) of RF ablation of hystologically proven renal cell neoplasms using a new single tip internally cooled RF probe.
**Methods or Background:** After ethical commettee approval we performed a retrospective analiysis of data reguarding the first patients (N= 85)treated with a new RF system whose electrode has an active tip (1-5 cm) that erogates high energies (400-2500mAs) with a pulsing algorithm optimized efficiency. This allows fast ablation times, comparable to MW, with a higher safety due to lower peak temperature reached (< 100 °C). Minimum follow up was 6 months, on average 12.3 months The outcomes were clinical outcome (success, partial success or failure) and safety. Tecnichal success was defined as complete ablation evaluated at any follow up CT-scan; partial success if any area of suspicious recurrence > 1 cm was identified anytime during follow up.
Safety was assessed according to the CIRSE complications classification
**Results or Findings:** The primary success rate was 91%; secondary success rate of 100 % (7 patient retreated). Reguarding safety there was one case of a CIRSE >2 complication (grade 3, bleeding needing embolization), and a total of 16% of CIRSE grade < 2 complications (N = 14). There was no mortality. Interestingly, tumours were divided by location and there was a high percentage of central (N= 10) or endophytic lesions (N = 29), with superimposable outcomes of the peripheral lesions.
**Conclusion:** RFA performed with this new modality resulted safe and effective and it is associated with short procedural times. The automatic energy-delivery controlled system might overcome limitations due to long procedural times and heat-sink effect in RFA. Future studies are needed to confirm these results on larger populations
**Limitations:** The study is etrospective and monocentric
**Funding for this study:** None
**Ethics committee - additional information:** Regional ethical committee
**Author Disclosures:**
Vittorio Pedicini: Nothing to disclose
Silvio Romano: Nothing to disclose
Tiziano Sirugo: Nothing to disclose
Nicolò Buffi: Nothing to disclose
Eleonora Baldassarre: Nothing to disclose
Ezio Lanza: Nothing to disclose
Marco Francone: Nothing to disclose
Paolo Casale: Nothing to disclose
Giuseppe Ferrillo: Nothing to disclose

**Predicting local tumour control in renal cell carcinoma after cryoablation by 3D volumetric image features**

*C-Y. Huang*, J-A. Hong, N-W. Chang, C-C. Li, C-A. Liu, S-H. Shen;
Taipei City/TW
*(cy.jeffrey.huang@gmail.com)*

**Purpose or Learning Objective:** Cryoablation has been shown to be a safe and effective treatment for renal cell carcinoma (RCC); however, there is currently no consensus on the predictors for local tumour control. The aim of this study was to implement automatic segmentation of renal structures on pretreatment CT images, extract relevant image features, and identify potential predictors for local tumour control in RCC after cryoablation.
**Methods or Background:** A total of 124 patients with RCC managed with cryoablation were included. Pretreatment abdominal CT in nephrographic phase were obtained and segmented into masks of the kidney, tumour, cyst, and renal sinus using deep learning segmentation models and post-processing. Relevant image features were extracted, and logistic regression was utilized to assess the correlation between these image features and response to cryoablation.
**Results or Findings:** Among the 124 patients included, failure of local tumour control occurred in 15 (12.1%). Tumour volume (P = .005) and the contact area between the tumour and the renal sinus (P = .001) were significantly associated with local tumour control according to univariable logistic regression. A multivariable logistic regression demonstrated that higher tumour volume (P=.051, OR=1.038, 95% CI=1.000-1.079) and a larger contact area between the tumour and the renal sinus (P=.005, OR=1.316, 95% CI=1.088-1.591) were associated with failure of local tumour control. The area under the ROC curve to predict local tumour control after cryoablation was 0.76.
**Conclusion:** This study demonstrated that volumetric image features, including tumour volume and the contact area between the tumour and the renal sinus, significantly increase the risk of failure of local tumour control in RCC after cryoablation.
**Limitations:** This study was subject to the inherent shortcomings of its retrospective design. Additionally, it was conducted at a single centre, so external validation is warranted.
**Funding for this study:** No funding was received for this study.
**Ethics committee - additional information:** The study was approved by the Institutional Review Board of Taipei Veterans General Hospital (2023-09-014CC).
**Author Disclosures:**
Chien-An Liu: Nothing to disclose
Chih-Ying Huang: Nothing to disclose
Shu-Huei Shen: Nothing to disclose
Chih-Chien Li: Nothing to disclose
Nai-Wen Chang: Nothing to disclose
Jia-An Hong: Nothing to disclose

**Efficacy of point of care cortical assay testing during adrenal venous sampling**

*A. Tinney*, K. Lau, J. Dorey; Clayton/AU

**Purpose or Learning Objective:** The aim of this single centre retrospective study was to evaluate the QCA false negative prevalence during AVS.
**Methods or Background:** Primary aldosteronism is the most common cause of secondary hypertension. Adrenal venous sampling (AVS) is the gold standard for subtyping primary aldosteronism and guiding treatment. Quick cortisol assay (QCA) has proven to be a useful tool to confirm correct catheter position within the adrenal vein, increasing procedure success rates. All consecutive AVS procedures on adult patients at our institution from July 2022 until March 2024 utilising intraoperative QCA were included. AVS without QCA were excluded. QCA analysis was performed in accordance with manufacturer recommendations. Correct catheter position was confirmed with dedicated angiographic techniques, and with formal biochemical analysis.
**Results or Findings:** 158 successful and 7 unsuccessful procedures were included. No QCA false positive result was found in unsuccessful AVS. 37 (23.4%) procedures produced a false negative result on QCA analysis despite correct catheter. 23/37 (62.2%) of these procedures had a bilateral false negative QCA, and 14/37 (37.8%) had an asymmetrical unilateral false negative QCA result. There was no significant difference in patient demographics across cohorts. Of the false negative QCA tests, 10 individual test kits (3.2%) provided a negative result despite laboratory cortisol results returning a value above the manufacturer reference value of 828nmol/L.
**Conclusion:** QCA has been shown to be a useful tool intra-operatively in increasing AVS sampling success rates. Our results demonstrate that false negative QCA results not infrequently occur and proceduralists must remain vigilant. Detailed patient history, dedicated angiographic techniques and correlation with preoperative CT remain vital when interpreting QCA results. Ongoing research is required to assess patient factors which may contribute to false negative results.
**Limitations:** Retrospective data availability.
**Funding for this study:** N/A
**Ethics committee - additional information:** Local institute low risk ethics approval.
**Author Disclosures:**
James Dorey: Nothing to disclose
Adrian Tinney: Nothing to disclose
Ken Lau: Nothing to disclose

**09:30-11:00** **Research Stage 2**

### Research Presentation Session: Abdominal and Gastrointestinal

### RPS 1801

### Innovative imaging in colorectal cancer and pelvic floor disorders

**Moderator**
S. Rafaelsen; Vejle/DK

**MRI evaluation of nodal status after neoadjuvant therapy in rectal cancer with node-by-node pathological comparison**

*Q-Y. Li*, X-Y. Yan, D. Yang, Z. Guan, R-J. Sun, Q. Lu, L. X. Ting, X. Zhang, Y-S. Sun; Beijing/CN
*(631213971@qq.com)*

**Purpose or Learning Objective:** To validate the performance of the ESGAR criteria of nodal status after neoadjuvant therapy (NAT) in rectal cancer and to investigate how morphological features and changes before and after NAT can help with node-by-node pathological comparison.
**Methods or Background:** Rectal cancer patients who received radical surgery after NAT and had complete pre- and post-NAT MRI were consecutively and prospectively enrolled. For the nodes that achieved node-by-node matched between MRI and pathology, their SADs were measured, and morphological features (i.e. shape, internal structure, and border) were determined on pre- and post-NAT axial T2WI.
**Results or Findings:** 207 patients were included and 612 nodes achieved matched, including 471 (77.0%) benign and 141 (23.0%) metastatic nodes. On the post-NAT MRI, the ESGAR criteria, i.e. SAD≥5mm, yield an AUC, sensitivity, and specificity in determining nodal status of 0.67, 52.5%, and 82.4%, respectively. All morphological features differed between benign and metastatic nodes, with AUCs ranging from 0.55 to 0.65. The prediction model combined of the only morphological independent predictor, internal structure, and SAD didn’t result in an improved diagnostic performance compared to SAD alone (P=.64). As the changes before and after NAT, there were differences in the size reduction rate and the change in internal structure, but not in shape and border. The AUC of the only independent predictor, size reduction rate, was only 0.58.
**Conclusion:** SAD of 5mm is a feasible criterion for determining nodal status on post-NAT MRI and its diagnostic performance could not be improved by morphological features or changes before and after NAT, which suggests the limited efficacy of conventional features and the urgent need for novel features in the future.
**Limitations:** The limitation is the lack of histopathological gold standards for every visible node on MRI.
**Funding for this study:** Funding was received from the National Natural Science Foundation of China (82271955) and Capital's Funds for Health Improvement and Research (2024-1-1022).
**Ethics committee - additional information:** This study was approved by the institutional review board (No. 2019KT76).
**Author Disclosures:**
Li Xiao Ting: Nothing to disclose
Xin-Yue Yan: Nothing to disclose
Rui-Jia Sun: Nothing to disclose
Ding Yang: Nothing to disclose
Ying-Shi Sun: Nothing to disclose
Zhen Guan: Nothing to disclose
Qing-Yang Li: Nothing to disclose
Xiaoyan Zhang: Nothing to disclose
Qiaoyuan Lu: Nothing to disclose

**A method of matching nodes between MRI and pathology in rectal cancer patients**

*Q-Y. Li*, X-Y. Yan, D. Yang, Z. Guan, R-J. Sun, Q. Lu, L. X. Ting, X. Zhang, Y-S. Sun; Beijing/CN
*(631213971@qq.com)*

**Purpose or Learning Objective:** To develop a method that enables node-by-node matching between preoperative MRI and postoperative pathology in rectal cancer patients, thereby providing reliable node-based ground-truth labels for further radiological studies.
**Methods or Background:** This methodological study prospectively enrolled 535 patients (59 ± 11 years; 326 males) with rectal cancer between 2021 and 2023. Target nodes were defined as nodal structures with a short-axis diameter (SAD) of ≥ 3 mm in the mesorectum or around the superior rectal artery on MRI. With relative location to the tumor, rectal wall and mesorectal fascia, each target node was localized in three directions. Combining the coordinates of each node, a 3D node map centered on the tumor and including all target nodes was constructed for each patient and used as a bridge enabling node-by-node matching between MRI and pathology.
**Results or Findings:** 3,038 target nodes were detected on preoperative MRI, of which 2,220 (73.1%) achieved matched between MRI and pathology.
Of the 1,707 matched benign nodes, 1,321 (77.4%), 378 (22.1%), and 8 (0.5%) had SADs of <5 mm, 5-9 mm and >9 mm, respectively. Whereas of the 513 matched metastatic nodes, 224 (43.7%), 254 (49.5%), and 35 (6.8%) had SADs of <5 mm, 5-9 mm and >9 mm, respectively. Patients with lower matching rates tended to have higher T-stages and more target nodes on MRI, whereas other factors, e.g. the BMI, therapeutic regimen, tumor location, and time interval between MRI and pathological examination did not show significant effect on the matching accuracy.
**Conclusion:** A matching method between MRI and pathology was developed to label numerous nodes with precise statuses in rectal cancer patients, which contributes to future radiological studies.
**Limitations:** The limitation is the small proportion of metastatic nodes compared to benign ones.
**Funding for this study:** Funding was received from the National Natural Science Foundation of China (82271955) and Capital's Funds for Health Improvement and Research (2024-1-1022).
**Ethics committee - additional information:** This study was approved by the institutional review board (No. 2019KT76).
**Author Disclosures:**
Li Xiao Ting: Nothing to disclose
Xin-Yue Yan: Nothing to disclose
Rui-Jia Sun: Nothing to disclose
Ding Yang: Nothing to disclose
Ying-Shi Sun: Nothing to disclose
Zhen Guan: Nothing to disclose
Qing-Yang Li: Nothing to disclose
Xiaoyan Zhang: Nothing to disclose
Qiaoyuan Lu: Nothing to disclose

**Prognostic impact of MRI-detected risk factors in total neoadjuvant therapy for locally advanced rectal cancer**

*G. F. Cicala*, S. Parisi, F. De Cobelli, V. Burgio, M. Ronzoni, R. Rosati, U. Elmore, P. Passoni; Milan/IT
*(cicala.giuseppe@hsr.it)*

**Purpose or Learning Objective:** In Locally Advanced Rectal Cancer (LARC), Total Neoadjuvant Therapy (TNT) has shown to be a valid therapeutic option leading to a significant reduction in recurrence, distant metastases, and an improvement in disease-free survival. Magnetic resonance imaging (MRI) plays a crucial role in the detection of extramural venous invasion (EMVI), tumor deposits (TDs), mesorectal fascia invasion (MFI) and helps identify patients who may benefit from a TNT.
**Methods or Background:** A retrospective analysis was performed on prospectively collected data involving 109 patients who received TNT between 2009 and 2022. MRI scans were conducted both before and after TNT, with a focus on EMVI, TDs, MFI. Following TNT, viable EMVI and TDs were evaluated using a standardized five-point Likert scale.
**Results or Findings:** Among the 109 patients, 95 patients met the inclusion criteria, 64.2% were male, with a median age of 60.3 years. Positive EMVI scores were observed in 47.4% of cases, while TDs and MFI in 15.7% and 24.7%, respectively. Positive EMVI, TDs, MFI, and Likert scores significantly correlated with reduced time to progression (TTP) and poorer overall survival (OS). In multivariate analysis, the presence of TDs (HR 10.28, p=0.002) and a Likert score of 4 (HR 16.44, p=0.003) were strong independent predictors of shorter TTP.
**Conclusion:** This study confirms the impact of MRI-detected risk factors as significant predictors of prognosis, supporting the role of a standardized five-point Likert scale in the stratification of patients for personalized treatment.
**Limitations:** Treatment changes after TNT can lead to false negatives, necessitating a multidisciplinary evaluation for patients. Larger cohorts are needed to improve the Likert scale's reliability.
**Funding for this study:** No funding was received for this study.
**Ethics committee - additional information:** Pending confirmation from the ethics committee
**Author Disclosures:**
Giuseppe Franco Cicala: Nothing to disclose
Valentina Burgio: Nothing to disclose
Sergio Parisi: Nothing to disclose
Monica Ronzoni: Nothing to disclose
Riccardo Rosati: Nothing to disclose
Francesco De Cobelli: Nothing to disclose
Paolo Passoni: Nothing to disclose
Ugo Elmore: Nothing to disclose

**Evaluation of diffusion-weighted imaging in predicting response in locally advanced rectal cancer**

*P. N. Franco*, C. Maino, C. R. G. L. O. M. Talei Franzesi, R. Corso, D. Ippolito; Monza/IT
*(francopaoloniccolo@gmail.com)*

**Purpose or Learning Objective:** To assess the performance of Diffusion-Weighted Imaging (DWI) and apparent diffusion coefficient (ADC) values in predicting response to neoadjuvant chemoradiation therapy (CRT) in patients with locally advanced rectal cancer (LARC).
**Methods or Background:** Ninety-four patients with MRI pre- and post-neoadjuvant treatment were retrospectively enrolled. Three regions of interest (ROIs) were manually drawn on three different tumor slices for every DWI sequence. ROIs were automatically copied to the corresponding ADC maps and the system derived three different ADC values (mean, maximum, and minimum), and the standard deviation (SD). Only mean ADC values were considered. After surgical intervention, pTNM and Mandard tumor-regression-grade (TRG) were obtained. Patients with TRG 1-2 were classified as responders while patients with TRG 3-5 were classified as non-responders.
**Results or Findings:** No correlation was found between pre-ADC values and TRG classes, while post-ADC and ΔADC values showed a significant correlation with TRG classes (r= -0.285, p=0.002 and r= -0.290, p=0.019, respectively). Post-ADC values were statistically different between responders and non-responders (p=0.019). When considering the relation between overall survival (OS) and ADC values, pre-ADC showed a negative correlation with OS (r= -0.381, p=0.001) while a positive correlation was found between ΔADC values and OS (r= 0.323, p=0.013). According to ΔADC values, the mean OS time between responders and non-responders showed a significant difference (p=0.030). A statistical difference was found between TRG classes and OS (p=0.038) and by dividing patients into responders and non-responders (p=0.019).
**Conclusion:** The pre-ADC and ΔADC values could be used as useful predictors for patients' prognosis. Post-ADC values, due to their relationship with TRG classes, could be a useful tool to predict response.
**Limitations:** No correlation between imaging and surgical specimens; the mean interval among CRT, restaging MRI, and surgery was variable among patients.
**Funding for this study:** None
**Ethics committee - additional information:** Upon reviewing the protocol, the local ethical committee deemed formal approval unnecessary, owing to the retrospective, observational, and anonymous nature of this study.
**Author Disclosures:**
Cesare Maino: Nothing to disclose
Cammillo Roberto Giovanni Leopoldo Oreste Massimiliano Talei Franzesi: Nothing to disclose
Rocco Corso: Nothing to disclose
Paolo Niccolò Franco: Nothing to disclose
Davide Ippolito: Nothing to disclose

**Efficacy of interventional transarterial treatment in locally recurrent or unresectable colorectal carcinoma: Therapy response and survival**

*T. J. Vogl*, A-I. Nica, C. Booz, L. S. Alizadeh, I. Yel, T. Biciusca, A. Gökduman, T. Gruber-Rouh, H. Adwan; Frankfurt/DE

**Purpose or Learning Objective:** To evaluate the efficacy of transarterial chemoperfusion (TACP) and transarterial chemoembolization (TACE) as palliative and symptomatic treatment options for unresectable colorectal carcinoma (CRC) regarding local tumor response and survival.
**Methods or Background:** Between January 2000 and October 2023, 318 TACP and 80 TACE procedures were performed in 67 patients with locally recurrent or unresectable CRC. Forty-eight patients were treated with TACP (mean 6.2 procedures/patient, range 2-22), 14 with TACE (mean 4.6 procedures/patient, range 2-11) and 5 patients received a combination of both therapies (mean 4.5 procedures/patient, range 2-13). Local tumor response was retrospectively evaluated using the RECIST criteria and overall survival (OS) and progression-free survival (PFS) were calculated using the Kaplan-Meier estimator.
**Results or Findings:** 49 (73.13%) of the 67 patients had stable disease (SD), 15 (22.39%) progressive disease (PD) and 3 patients (4.48%) partial response (PR). Median OS was 16.17 months, median PFS was 11.25 months. There was no statistically significant difference in OS (P=0.598) and PFS (P=0.847) between patients either receiving TACP or TACE or both treatments. One year after the first procedure, 27 (40.3%) patients were still alive. Nine patients (13.4%) were still alive after 2 years and six patients (8.9%) were still alive after 3 years. No major complications were reported.
**Conclusion: CONCLUSION:** TACP and TACE are minimally invasive procedures that offer a treatment option for patients with locally recurrent or unresectable CRC, potentially preventing tumor progression and improving quality of life. However, their benefits in the treatment of CRC warrant further investigation.
**Limitations:** Retrospective, single-center study Varying follow-up intervals Short follow-up period
**Funding for this study:** No funding
**Ethics committee - additional information:** Approval of the ethics committee of the Johann Wolfgang Goethe University, Frankfurt
**Author Disclosures:**
Christian Booz: Nothing to disclose
Ibrahim Yel: Nothing to disclose
Teodora Biciusca: Nothing to disclose
Andreea-Ioana Nica: Nothing to disclose
Thomas J. Vogl: Nothing to disclose
Aynur Gökduman: Nothing to disclose
Tatjana Gruber-Rouh: Nothing to disclose
Hamzah Adwan: Nothing to disclose
Leona Soraja Alizadeh: Nothing to disclose

**Opportunistic Colorectal Cancer Screening in Computed Tomography: Exploration of the Colon-Liver Axis with Machine Learning**
*S. Grosu*[1], A. Hinterberger[2], A. E. Sint[1], J. Ricke[1], M. Ingrisch[1], P. Wesp[1]; [1]Munich/DE, [2]Heidelberg/DE

**Purpose or Learning Objective:** Colorectal cancer (CRC) detection in non-dedicated computed tomography (CT) examinations without bowel preparation is challenging. Current research suggests that liver diseases are associated with an increased risk of colorectal cancer. The aim of this study was to identify patients with CRC using machine learning (ML)-based opportunistic analysis of the liver in non-dedicated routine clinical CT scans.
**Methods or Background:** Patients 18 years or older with histologically proven CRC or negative colonoscopy that underwent contrast-enhanced CT of the abdomen for various indications within 5 years or less to colonoscopy were included retrospectively. Patients were randomly divided into a training set (75%) and a test set (25%), stratifying for age and sex. Deep learning-based automated liver segmentation on CT images was performed. Standardized Radiomic image features were extracted from the liver segmentations. A random forest ML algorithm was trained on the training set to differentiate between patients with histologically confirmed CRC or negative colonoscopy (non-CRC). Algorithm performance was evaluated on the test set using ROC-AUC, sensitivity and specificity.
**Results or Findings:** The training set comprised 809 CT scans from 809 patients (mean age = 62.2 years; 42% female), 66 (8 %) with CRC. The test set comprised 270 CT scans from 270 patients (mean age = 62.2 years; 44% female), 23 (9 %) with CRC. The ROC-AUC for random forest-based differentiation between CRC and non-CRC patients was 0.62, with a sensitivity of 74% at a specificity of 50%.
**Conclusion:** Our results indicate that opportunistic analysis of routine clinical CT images of the liver might have the potential to detect patients with an increased risk for CRC without additional radiation exposure or examinations.
**Limitations:** Further refinement of the presented model is needed to further increase its diagnostic performance.
**Funding for this study:** No funding was received for this study.
**Ethics committee - additional information:** Ethics committe of the LMU University Hospital, LMU Munich, Munich, Germany
**Author Disclosures:**
Alena Elisabeth Sint: Nothing to disclose
Sergio Grosu: Nothing to disclose
Michael Ingrisch: Nothing to disclose
Philipp Wesp: Nothing to disclose
Anna Hinterberger: Nothing to disclose
Jens Ricke: Nothing to disclose

**Prognostic value of lateral lymph node metastasis in pretreatment MRI for rectal cancer in patients with neoadjuvant CRT and surgical resection without LLND: A systemic review and meta-analysis**
*T. Lee*[1], N. Horvat[2], M. J. Gollub[2], J. Garcia-Aguilar[2], T. H. Kim[2]; [1]Seoul/KR, [2]New York, NY/US
*(lee8720@gmail.com)*

**Purpose or Learning Objective:** To systematically review and meta-analyze the prognostic significance of lateral lymph node metastasis (LLNM) on pretreatment MRI in patients with rectal cancer who undergo neoadjuvant chemoradiation followed by curative surgical resection without lateral lymph node dissection (LLND).
**Methods or Background:** We searched the MEDLINE and EMBASE databases until September 27, 2023, utilizing the following search terms: (rectal OR rectum OR colorectal) AND (lateral OR sidewall) AND (lymph OR node). The QUIPS tool was employed to evaluate methodological quality. We pooled the association between LLNM on pretreatment MRI and outcomes such as local recurrence, distant metastasis, disease-free survival, and overall survival using hazard ratio (HR) and odds ratio (OR) based on random effects model.
**Results or Findings:** We included 9 studies, encompassing 3180 patients. LLNM on pretreatment MRI revealed a significant association with increased local recurrence rates (HR: 4.11; 95 % CI: [1.87, 9.02]) and elevated risks for both disease-free (HR: 1.70; 95 % CI: [1.42, 2.03]) and overall survival (HR: 1.76; 95 % CI: [1.44, 2.15]). As for distant metastasis, our analysis indicated a potential trend towards increased rates, though this did not reach statistical significance (HR: 1.67; 95 % CI: [0.85, 3.27]).
**Conclusion:** Our findings underscore the relationship between LLNM and increased local recurrence and compromised disease-free and overall survival. This emphasizes the potential limitations of relying solely on neoadjuvant chemoradiation and highlights the potential need to intensify treatment in select patients.
**Limitations:** Firstly, we included a relatively small number of studies. Second, a notable heterogeneity was observed in the criteria used to define LLNM on MRI across different studies, which probably led to heterogenous proportion of LLNM-positive patients among included studies.
**Funding for this study:** The National Cancer Institute Cancer Center Core Grant P30 CA008748
**Ethics committee - additional information:** This study was a systematic review and therefore exempt from requiring approval from our institutional review board.
**Author Disclosures:**
Taehee Lee: Nothing to disclose
Julio Garcia-Aguilar: Nothing to disclose
Marc Jeffrey Gollub: Nothing to disclose
Natally Horvat: Nothing to disclose
Tae Hyung Kim: Nothing to disclose

**Open magnetic field and MRI defecography**
*G. Sterlicchio*, I. Carbone, M. Rengo, C. L. Saletti, D. Bellini; Latina/IT
*(giuseppe.sterlicchio@uniroma1.it)*

**Purpose or Learning Objective:** To investigate the diagnostic value and image quality of Open MRI scanner (0.5T) on the evaluation of pelvic floor disfunctions.
**Methods or Background:** Twenty one patients (20 women and 1 man) underwent MRI defecography using both Open MRI scanner (0.5 T) and high filed MRI scanner (1.5 T). For both scanners, the same MRI protocol has been adopted, including morphological sequences and dynamic sequences during rest and defecation. Contrast-to-noise ratio (CNR), and signal to noise ration (SNR) were calculated and compared among the different data sets. Qualitative assessment of image quality was performed by 3 readers using 5 points Likert scale.
**Results or Findings:** SNR was significantly lower using 0.5T compared to 1.5T (mean value 33.1 vs 95.2 for T2 sequences and 44.8 vs. 55.6 for dynamic sequences; $P<0.05$). CNR was significantly lower using 0.5T compared to 1.5T (mean value 37.1 vs 57.6 for T2 sequences and 27.1 vs. 51.3 for dynamic sequences; $P<0.05$). However, the readers' image quality scores showed that open MRI scanner is not inferior to High magnetic filed. Diagnostic accuracy was the same for both scanners.
**Conclusion:** Despite the low values of all quantitative quality metrics on 0.5T compared to i.5T, readers perception of image quality is the same between Open MRI scanner and High filed MRI scanner. Low magnetic field does not affect diagnostic accuracy.
**Limitations:** Small sample size.
**Funding for this study:** None

**Ethics committee - additional information:** Yes
**Author Disclosures:**
Iacopo Carbone: Nothing to disclose
Marco Rengo: Nothing to disclose
Giuseppe Sterlicchio: Nothing to disclose
Carlos Leon Saletti: Nothing to disclose
Davide Bellini: Nothing to disclose

**Assessment of puborectalis (PRM) and pubococcygeus (PCM) muscles thickness by RMI defecography: A promising adjuvant radiological parameter for the identification of spastic pelvic floor syndrome(SPFS)**
*M. Mandolini*, V. Carrozzo, A. Calculli, R. Faletti, A. Ferraris; Torino/IT
*(matilde.mandolini@unito.it)*

**Purpose or Learning Objective:** To investigate the association between radiological suspicion of SPFS and the thickness ratio R(PRM/PCM) in patients with pelvic floor disorders.
**Methods or Background:** The retrospective study involved 109 women (age 17-86 ys, BMI 16-35.5 kg/m2, past pregnancies 76%) who underwent RMI Defecography between November 2021 and September 2024 at our institution. The ESGAR-ESUR recommended protocol was complemented by measurements of PRM and PCM branches thickness made bilaterally at half the length of each bundle and averaged to obtain the ratio R (PRM/PCM). Continuous independent variables were compared with Mann-Whitney's Test when independent; Wilcoxon's Test and Bland-Altman plot when correlated; dichotomic variables were studied with Fisher'sTest. PropensityScoreMatching (PSM) was used to reduce the effect of confounding covariates on the outcome.The ReceivingOperatingCurve (ROC) was used to estimate the performance of R with the Area Under The Curve (AUC).
**Results or Findings:** The 23 (21%) patients satisfying the radiological criteria for SPFS recognized by scientific literature formed the Study Group.PSM extracted from the remaining 84 patients, a Control Group of 23 with baseline comparable ($p>0.77$, Standardized Mean Difference <0.20) to that of the Study patients. The thickness ratio R was 3.2(3.0-3.5) for the former vs 2.5(2.2-2.75) for the latter ($p<0.0001$). The ROC curve assessed a good diagnostic ability of R for SPSF, (AUC=0.94) with threshold R≥3.0 (sensitivity 0.87, specificity 0.96, PPV=0.95, NPV=0.88).The measurements made by two readers showed good intra- and inter-operator agreement.
**Conclusion:** The performance of the PRM/PCM thickness ratio is presently being explored over a larger population; if the positive results will be confirmed, this indicator may become an additional tool for radiological identification of SPFS.
**Limitations:** The main limitations of the study are the absence of gold standard method for SPFS diagnosis and the small number of our Study Group.
**Funding for this study:** No funding was provided for this study.
**Ethics committee - additional information:** Being a retrospective preliminary study based on the use of anonymous data, it has not yet been subjected to scrutiny by the ethics committee.
**Author Disclosures:**
Matilde Mandolini: Nothing to disclose
Annarita Calculli: Nothing to disclose
Valentina Carrozzo: Nothing to disclose
Riccardo Faletti: Nothing to disclose
Andrea Ferraris: Nothing to disclose

**09:30-11:00 Research Stage 3**

### Research Presentation Session: Neuro

### RPS 1811

### Brain in function

**Moderator**
N. Bargalló; Barcelona/ES
*(BARGALLO@clinic.cat)*

**Constructing normative human brain atlases of R1, R2, proton density, and myelin volume fraction using synthetic quantitative MRI**
*H. M. H. Sbaihat*, A. K. Roenneke, D. Müller, T. Ladopoulos, R. Schneider, B. Krieger, B. Bellenberg, C. Lukas; Bochum/DE
*(hasansbaihat@gmail.com)*

**Purpose or Learning Objective:** Quantitative MRI (qMRI) provides valuable insights into tissue-specific MR properties, extending the diagnostic capabilities of conventional MRI. We aimed to construct normative multimodal human brain atlases to serve as references for tissue alterations in neurological disorders.
**Methods or Background:** Fifty-eight healthy controls (HC) underwent qMRI of the brain at 1.5T using the QRAPMASTER sequence, resulting in parameter maps for Myelin Volume Fraction (MVF, %), Proton Density (PD, %), and Relaxation Rates R1 and R2 ($s^{-1}$). We constructed four high-resolution ($1mm^3$) atlases in standard space after bias-field correction, interpolation, normalization, and smoothing. These atlases allow visual and quantitative comparisons with individual datasets and will be made available to the research community. Additionally, quantitative data were extracted from 26 white matter regions of interest (ROIs). The atlases and extracted data were validated using z-score maps of three healthy controls and three multiple sclerosis (MS) patients. Group differences across the ROIs between the qMRI atlases and the testing subjects were assessed using t-tests.
**Results or Findings:** The resulting atlases demonstrated high anatomical accuracy, resolution, and comprehensive brain coverage. The ROI values for R1, R2, PD, and MVF were consistent with the published literature. The z-score maps, particularly for R1 and MVF, accurately reflected individual lesion patterns and diffuse tissue changes in each MS patient. T-test results for R1, R2, PD, and MVF confirmed the alignment between the HC atlases and the HC testing group, while significant differences were observed with the MS testing subjects at a p-value of 0.01.
**Conclusion:** We successfully generated high-resolution, multi-modal qMRI atlases of the human brain, providing normative baselines for R1, R2, PD, and MVF. The validation process underscores their potential for assessing microstructural brain alterations in individual patients with neurodegenerative diseases.
**Limitations:** Not applicable.
**Funding for this study:** No funding was provided for this study.
**Ethics committee - additional information:** The ethics committee of the Medical Faculty of the Ruhr-University Bochum, Germany (Approval Np. 20-7054-BR).
Area of Interest: CNS, Neuroradiology brain.
**Author Disclosures:**
Ruth Schneider: Nothing to disclose
Theodoros Ladopoulos: Nothing to disclose
Barbara Bellenberg: Nothing to disclose
Britta Krieger: Nothing to disclose
Hasan M H Sbaihat: Nothing to disclose
Anna Katharina Roenneke: Nothing to disclose
Carsten Lukas: Nothing to disclose
Dajana Müller: Nothing to disclose

**How does hippocampal volume in mesial temporal sclerosis affect brain networks during a functional MRI memory task?**
*S. B. Rosa*[1], B. Direito[2], F. Sales[2], D. J. Pereira[2]; [1]Lisbon/PT, [2]Coimbra/PT
*(saradbrosa@gmail.com)*

**Purpose or Learning Objective:** Mesial Temporal Sclerosis (MTS) is the most frequent histopathological abnormality in drug-resistant temporal lobe epilepsy. Anterior temporal lobectomy is a possible treatment, but postoperative memory deficits may follow. MTS patients activate extra-temporal regions during memory-related tasks, possibly as part of compensatory networks. Further research is required to understand these networks and how they may impact surgical outcomes. We aimed to understand how hippocampal atrophy affects connectivity during a memory fMRI task in MTS patients.

**Methods or Background:** We retrospectively included drug-resistant MTS patients admitted to CLHU (2019-2023) for surgical evaluation. Patients underwent MRI, including 3D T1WI and event-related memory fMRI. Hippocampal volume ratio was automatically calculated using volBrain. CONN toolbox was used to assess the effect of hippocampal volume on seed-to-voxel functional connectivity of the right and left hippocampus in patients with right and left MTS, respectively, while performing a verbal memory task.
**Results or Findings:** Thirteen patients were included (eight with lMTS, five with rMTS). Hippocampal volume ratio was 0.1047±0.0406 for lMTS and 0.1144±0.0331 for rMTS patients (mean ± standard deviation). Seed-to-voxel analysis showed a positive association between hippocampal volume and connectivity with contralateral hippocampus and parahippocampal gyri in both groups. In lMTS, hippocampal volume was negatively associated with connectivity to right superior parietal lobule and supramarginal gyrus ($p<0.05$ cluster-size p-FDR corrected). No significant negative associations were found in rMTS.
**Conclusion:** As expected, greater hippocampal atrophy corresponded to reduced connectivity with the parahippocampal gyri and contralateral mesial temporal regions. Surprisingly, lMTS patients with greater atrophy showed higher connectivity to right parietal areas, a compensatory network not previously described in this condition, to our knowledge.
**Limitations:** Small sample size, applying a resting-state fMRI analysis method to task-based fMRI, assessing right hippocampal connectivity with a left hippocampal task.
**Funding for this study:** No funding.
**Ethics committee - additional information:** All patients signed written consent for data usage and all data was anonymized.
**Author Disclosures:**
Francisco Sales: Nothing to disclose
Sara Botelho Rosa: Grant Recipient: ESR-EIBIR seed grant
Daniela Jardim Pereira: Nothing to disclose
Bruno Direito: Nothing to disclose

**Unveiling the Brain's Response to Valenced Sounds: Neural Correlates of Auditory Emotion**
*F. Aldhafeeri*; Hafar al-Batin/SA
*(aldhafeeri8@hotmail.com)*

**Purpose or Learning Objective:** Complex neural pathways that integrate auditory processing with emotional evaluation play a crucial role in the perception of emotional sounds. This study aimed to identify the neural circuits that differentially encode positive and negative valence during the implicit processing of emotional stimuli.
**Methods or Background:** A block-design fMRI experiment was conducted with thirty healthy participants. The study measured blood oxygen level dependent (BOLD) signal changes in response to pleasant and unpleasant sounds from the International Affective Digitized Sounds (IADS), with each condition compared against a neutral baseline
**Results or Findings:** Significant activation (pFDRcorrected <0.05) was found in the medial prefrontal cortex (mPFC), ventral anterior cingulate cortex (vACC), and temporal lobe when contrasting pleasant sounds with neutral conditions. In response to unpleasant sounds, significant activation (pFDRcorrected <0.05) was observed in the amygdala, nucleus accumbens, parahippocampal gyri, temporal lobe, visual cortex, PFC, insula, anterior cingulate gyrus, and cerebellum, compared to the neutral condition
**Conclusion:** The neural correlates of pleasant and unpleasant stimuli involve a complex interplay between brain regions that regulate emotional responses. The auditory cortex, amygdala, and nucleus accumbens are key components of this process, with distinct activation patterns depending on the emotional valence of the auditory stimuli. Understanding these neural mechanisms enhances our insight into how sound influences emotional experiences and may guide the development of therapeutic interventions for auditory-related emotional disorders
**Limitations:** The study was conducted with a relatively small sample size of thirty participants. While this is typical for many fMRI studies, the limited number of participants may affect the generalizability of the findings. A larger sample size could provide more robust and reliable results.
**Funding for this study:** None
**Ethics committee - additional information:** Local Research Ethics Committee
**Author Disclosures:**
Faten Aldhafeeri: Nothing to disclose

**Amygdala multimodal reorganization as an indicator of affective dysfunction in tinnitus patients**
*Q. Chen*; Beijing/CN
*(chenqian8319@163.com)*

**Purpose or Learning Objective:** This study aimed to systematically investigate structural and functional alterations in amygdala subregions using multimodal MRI in patients with tinnitus with or without affective dysfunction.
**Methods or Background:** Sixty patients with persistent tinnitus and 40 healthy controls (HCs) were recruited. Based on a questionnaire assessment, 26 and 34 patients were categorized into the tinnitus patients with affective dysfunction (TPAD) and tinnitus patients without affective dysfunction (TPWAD) groups, respectively. MRI-based measurements of gray matter volume, fractional anisotropy (FA), fractional amplitude of low-frequency fluctuations (fALFF), regional homogeneity (ReHo), degree centrality (DC), and functional connectivity (FC) were conducted within 14 amygdala subregions for intergroup comparisons. Associations between the MRI properties and clinical characteristics were estimated via partial correlation analyses.
**Results or Findings:** Compared with HCs, the patients exhibited significant structural and functional changes, with more pronounced WMI changes in the TPAD group, predominantly within the left auxiliary basal or basomedial nucleus (AB/BM), right central nucleus, right lateral nuclei (dorsal portion), and left lateral nuclei (ventral portion containing basolateral portions). Moreover, the TPAD group exhibited decreased FC between the left AB/BM and left middle occipital gyrus and right superior frontal gyrus (SFG), left basal nucleus and right SFG, and right lateral nuclei (intermediate portion) and right SFG. In combination, these amygdalar alterations exhibited a sensitivity of 65.4% and specificity of 96.9% in predicting affective dysfunction in patients with tinnitus.
**Conclusion:** Although similar structural and functional amygdala remodeling were observed in the TPAD and TPWAD groups, the changes were more pronounced in the TPAD group. These changes mainly involved alterations in functionality and white matter microstructure in various amygdala subregions; in combination, these changes could serve as an imaging-based predictor of emotional disorders in patients with tinnitus.
**Limitations:** This is a cross-section study
**Funding for this study:** None
**Ethics committee - additional information:** This study was approved by the Institutional Review Board of Beijing Friendship Hospital, Capital Medical University (No. 2017-P2-134-01).
**Author Disclosures:**
Qian Chen: Nothing to disclose

**The impact of diet on brain structural and functional networks**
*R. Rajiah*, Q. Aziz, P. Nachev, J. K. K. Ruffle; London/UK

**Purpose or Learning Objective:** To delineate the association between diet, brain structure and function in patients undergoing multi-modal MRI.
**Methods or Background:** Extensive evidence from lab-based and clinical studies support diet as an essential regulator of brain function. However accompanying neuroimaging evidence is scarce and small in scale. We studied 518 healthy participants from the Cambridge Centre for Aging and Neuroscience (CAM-Can) repository (261 male and 257 female, mean age 53 years). Participants were clustered via their self-reported dietary intakes using K-means clustering. From MRI, resting-state functional, white matter tractography, and grey matter volumetry networks conditional to dietary cluster were investigated using network-based statistics, with participant age and sex as nuisance covariates. Relevant clinical data comprising BMI and Hospital Anxiety and Depression Score (HADS) were compared using Welch's t-test.
**Results or Findings:** Four dietary intake patterns were identified: balanced, meat-predominant, vegetarian, and raw vegetable-predominant. Specific dietary types – balanced, meat-predominant and vegetarian – were associated with increased functional, grey matter or white matter connectivity between regions comprising the bilateral insula, frontomedial cortex (FMC) and bilateral anterior cingulate cortex (ACC) (p=<0.04). A balanced diet was associated with significantly increased functional, structural, and white matter connectivity between brain regions, including the orbitofrontal cortex (OFC), bilateral insula and FMC compared to all others (p=<0.04). A balanced diet was associated with a significantly reduced depression score (HADS-D), compared to a meat-predominant one (p=0.03). The meat-predominant diet cluster correlated with increased structural connectivity at the bilateral ACC and increased BMI (p=0.01), when compared to vegetarian diets (p=0.03).
**Conclusion:** In the largest study of its kind, we reveal the structural and functional brain associations of diet. Our findings imply physiological correlates for diet-induced brain changes for future research.
**Limitations:** We use self-reported dietary intake, limited by data availability.

**Funding for this study:** The Issac Shapiro grant supported RR. JKR was supported by the Medical Research Council (MR/X00046X/1). PN is supported by the Wellcome Trust (213038/Z/18/Z) and the UCLH NIHR Biomedical Research Centre.
**Ethics committee - additional information:** The Cam-CAN project was approved by Cambridgeshire Research Ethics Committee (reference: 10/H0308/50).
**Author Disclosures:**
Parashkev Nachev: Nothing to disclose
Qasim Aziz: Nothing to disclose
Rebekah Rajiah: Nothing to disclose
James Kelsey K Ruffle: Nothing to disclose

**Current European trends in the use of CTA as an ancillary test in the determination of brain death**
*H. Briody*, I. Alam, R. Bruen, P. Mchugh, P. Rohan, S. Looby; Dublin/IE
*(hayleybriody@rcsi.ie)*

**Purpose or Learning Objective:** To establish the current trends in European literature on the use of computed tomography angiography (CTA) as an ancillary test in the determination of brain death and to clarify the technical parameters and interpretative criteria currently in use.
**Methods or Background:** Brain death is primarily a clinical diagnosis made by confirming the absence of brainstem reflexes and the presence of apnoea in the setting of irreversible coma where confounding factors have been excluded. However, in specific circumstances where it is not possible to perform the required tests to satisfy the clinical criteria, ancillary testing may be required to support the diagnosis. The radiological study endorsed by most national guidelines remains four-vessel digital subtraction angiography although there is a move towards the less invasive, more accessible CTA.
**Results or Findings:** CTA is currently endorsed as an ancillary test for the determination of brain death in multiple European countries including France, Germany, Spain, Switzerland, Poland and the United Kingdom. The Polish and UK guidelines advise a three-phase study consisting of a non-contrast phase and post-contrast phases at 20 and 40 seconds post intravenous contrast injection. Opacification of the superficial temporal or facial artery is required to confirm correct contrast administration. Cessation of cerebral circulation is diagnosed when there is bilateral absence of contrast in the middle cerebral arteries and internal cerebral veins on the second post-contrast phase.
**Conclusion:** CTA is emerging as a feasible ancillary test for the diagnosis of brain death and is currently endorsed by a number of European guidelines. The most widely used technique involves a three-phase study with evaluation of intracranial vessels in four anatomical locations.
**Limitations:** This study is limited by a paucity of literature and lack of consensus guidelines on imaging in brain death.
**Funding for this study:** None.
**Ethics committee - additional information:** N/A
**Author Disclosures:**
Paul Mchugh: Nothing to disclose
Richard Bruen: Nothing to disclose
Imran Alam: Nothing to disclose
Seamus Looby: Nothing to disclose
Hayley Briody: Nothing to disclose
Pat Rohan: Nothing to disclose

**Speed-reading-induced changes in functional brain networks: A connectivity-based analysis**
*T. A. Walpola*, C. Yang, N. Dilhani, R. Iseki, T. Makino, T. N. Hoang, C. D. Kulathilake, I. Ichiro, A. Senoo; Tokyo/JP
*(thishuliwalpola@gmail.com)*

**Purpose or Learning Objective:** Speed-reading is a salient technique used among children to improve fast learning skills. The main aim of the present study is to assess the alterations of the brain's functional connectivity networks identified in a speed-reading-trained native Sinhalese cohort.
**Methods or Background:** A cohort of 18 healthy native Sinhala-speaking adults (>18yrs) who volunteered to undergo fMRI (scanner: 3.0 T SIGNA Premier) were selected ((male, 11: female, 7), Age (mean; Stdv: 31;4.0) and dominant hand: Right). Participants read a simple Sinhala novel silently inside the scanner. Three scans at one-month intervals each were done; the first two were the control scans, and the third was the training scan after the intervention of speed-reading training. Image acquisition; fMRI sequence using GRE EPI (TR: 1000(ms) TE: 30(ms), characters per task block: 320-350). Data analysis was performed using CONN toolbox v.22.a.
**Results or Findings:** The reading speed (mean (Stdv)) increased from 213 (87) to 712 (200) wpm in the training group. The seed-based connectivity (SBC) results showed that the language network has increased significant connectivity, with 57 voxels (32%) covering 20% of Right Heschl's gyrus and 68 (38%) voxels covering 15% of planum temporale (Significant increase threshold: $p<0.05$ cluster-size p-FDR corrected, voxel threshold: $p<0.001$ (p-uncorrected)). According to the generalized psychophysiological interactions (gPPI) results, the individual ROI analysis showed increased connectivity in the left posterior temporal gyrus with the bilateral visual lateral and occipital networks meanwhile a decreased connectivity with the visual medial network (p-FDR corrected <0.05).
**Conclusion:** The study concluded that the activation of Heschl's gyrus; crucial for sound perception in reading, and planum temporale; responsible for phonological processing highlights the importance of some auditory processing regions related to quick recognition and comprehension of text during speed reading.
**Limitations:** Eye-tracking is not performed
**Funding for this study:** None
**Ethics committee - additional information:** The Ethics have been approved by the Ethics Review Committee of Tokyo Metropolitan University, Tokyo, Japan Approval No-22022
**Author Disclosures:**
Niluka Dilhani: Nothing to disclose
Chutian Yang: Nothing to disclose
Tatsuya Makino: Nothing to disclose
Thishuli Anujaya Walpola: Nothing to disclose
Thanh Ngoc Hoang: Nothing to disclose
Rinako Iseki: Nothing to disclose
Atsushi Senoo: Nothing to disclose
Chathura Darshana Kulathilake: Nothing to disclose
Iso Ichiro: Nothing to disclose

**Illuminating Minds: The Transformative Role of Radiology in Mental Health Diagnosis**
R. Praveenkumar, *F. Abubacker Sulaiman*, J. Lydia; Chennai/IN
*(fasulaiman@gmail.com)*

**Purpose or Learning Objective:** This abstract examines the contributions of radiology to diagnosing and managing mental health disorders, emphasizing advanced imaging modalities' role in understanding neurobiological mechanisms.
**Methods or Background:** A systematic review of literature was conducted, focusing on the use of magnetic resonance imaging (MRI), computed tomography (CT), and positron emission tomography (PET) in evaluating conditions such as schizophrenia, major depressive disorder, anxiety disorders, and post-traumatic stress disorder (PTSD).
**Results or Findings:** Key findings illustrate the impact of radiology on mental health care: Structural Imaging: MRI and CT scans identify neuroanatomical changes in mental health disorders. For example, studies show reduced gray matter in the prefrontal cortex of patients with schizophrenia and altered hippocampal volumes in those with major depressive disorder. Functional Imaging: PET and functional MRI (fMRI) enhance understanding of neural circuitry. Research indicates hyperactivity in the amygdala of individuals with anxiety disorders and altered connectivity in the default mode network in patients with depression. Biomarker Discovery: Radiological imaging aids in identifying neurobiological biomarkers. Specific metabolic patterns in PET scans may predict treatment response in major depressive disorder, allowing for personalized therapy. Therapeutic Monitoring: Imaging techniques monitor treatment efficacy. In patients receiving transcranial magnetic stimulation (TMS) or electroconvulsive therapy (ECT), imaging provides real-time assessments of changes in brain activity.
**Conclusion:** Radiology significantly enhances the understanding and management of mental health disorders through advanced imaging techniques, improving diagnostic accuracy and facilitating personalized treatment strategies.
**Limitations:** The review highlights the need for standardized imaging protocols and further research to correlate neuroimaging findings with clinical outcomes
**Funding for this study:** Not applicable
**Ethics committee - additional information:** Not applicable
**Author Disclosures:**
Rathinamoorthy Praveenkumar: Nothing to disclose
Johnbosco Lydia: Nothing to disclose
Farook Abubacker Sulaiman: Nothing to disclose

**Altered Structural-Functional Coupling in Parkinson's Disease Patients with Depression: Insights from Multimodal Brain Network Analysis**
*M. Wang*[1], C. Tan[2], J. Hakumäki[1]; [1]Kuopio/FI, [2]Changsha/CN

**Purpose or Learning Objective:** This study integrates multimodal brain network data to explore alterations in structural-functional coupling across multi-scale brain networks in Parkinson's disease patients with depression (PDD). By identifying potential imaging biomarkers, this research aims to enhance sustainable diagnostic processes, improving healthcare efficiency through accurate and early detection of depression in Parkinson's disease.

Saturday

**Methods or Background:** A total of 106 consecutive, drug-naïve Parkinson's disease patients were prospectively enrolled and divided into PDD (n = 50, HAMD > 17, BDI > 10) and PDND (n = 56, HAMD < 7, BDI < 10) groups. All participants underwent diffusion tensor imaging (DTI) and resting-state MRI on a 3.0T scanner. Whole-brain functional and structural networks were constructed. Structural-functional coupling, intra- and inter-module connectivity, as well as topological parameters (e.g., clustering coefficient, local efficiency), were compared between the two groups. ROC curve analysis was performed to evaluate the diagnostic performance of these parameters in differentiating PDD from PDND.
**Results or Findings:** PDD patients exhibited significantly lower whole-brain structural-functional coupling compared to PDND patients (P = 0.017, TFCE-corrected). At the module level, PDD patients showed increased structural-functional coupling within the default mode network (P = 0.032, TFCE-corrected). Additionally, PDD patients had significantly lower clustering coefficients (P = 0.007) and reduced local efficiency (P = 0.021) across the brain's structural network. ROC analysis combining these metrics demonstrated a sensitivity of 65% and a specificity of 77.7% for distinguishing PDD from PDND.
**Conclusion:** Altered multi-scale brain network structural-functional coupling in PDD suggests potential imaging biomarkers for more sustainable and precise diagnosis. These findings may help reduce unnecessary interventions and optimise healthcare resources by providing a non-invasive tool for early detection of depression in Parkinson's disease.
**Limitations:** The limitation is the relatively small sample size.
**Funding for this study:** CSC funding No. 202306370155
**Ethics committee - additional information:** The study received institutional review board approval, and written informed consent was obtained from all participants.
**Author Disclosures:**
Changlian Tan: Nothing to disclose
Juhana Hakumäki: Nothing to disclose
Min Wang: Nothing to disclose

**Systematically altered connectome gradient in patient with Type2-diabetes mellitus: Potential effect on cognitive function**
*H. Ran*, K. Huang, T. Zhang; ZunYi/CN
*(haifengran_zmu@163.com)*

**Purpose or Learning Objective:** Type2-diabetes mellitus (T2DM) is known to affect brain networks and cognitive function. Connectome gradient studies have suggested a primary-to-transmodal gradient in functional brain network. However, whether this gradient structure is disrupted in patients with T2DM are still ambiguous. The aim of this study is to investigate connectome gradient alterations and its potential contribution to cognitive function in T2DM.
**Methods or Background:** We recruited resting-state functional magnetic resonance imaging (rs-fMRI) data of 45 participants (24 T2DM patients and 21 healthy controls) and Montreal Cognitive Assessment Scale (MoCA) and Mini-Mental State Examination (MMSE) were performed in patients with T2DM. We studied the related alterations in the principal and secondary connectome gradient between T2DM and healthy controls at the voxel and network levels. We further examined the associations between T2DM-related changes of connectome gradients and clinical variables. The RVR algorithm was employed to assess the predictive capacity of principal gradients in relation to cognitive function scores among patients diagnosed with T2DM
**Results or Findings:** Relative to the controls, T2DM patients extended gradient at different network-level and voxel-level. In the principal gradient, the left rolandic operculum, precuneus gradient score was negatively correlated with MMSE score and duration, respectively (r =-0.479, -0.481, P=0.028, 0.027), and the connectome gradient alterations in ventral attention network was negatively correlated with MMSE and MoCA (r=-0.484, -0.435, P=0.026, 0.049). Patients' principal gradient maps significantly predicted their MMSE (r = 0.465, P = 0.022).
**Conclusion:** We reported a systematically disrupted functional gradient in patients with T2DM and its negative correlation with cognitive function. These findings improve our comprehension the neurobiological mechanisms that underlie cognitive function and offer potential imaging biomarkers for the assessment of cognitive function in T2DM.
**Limitations:** The sample size of this study was relative small.
**Funding for this study:** This study was supported by Intelligent Medical Imaging Engineering Research Center of Guizhou Higher Education Institutions project (Grant No. Qianjiaoji [2023] 038).
**Ethics committee - additional information:** Ethics approval of this research was granted by the Ethic Committee of the Affiliated Hospital of Zunyi Medical University[KLL-2024-512]
**Author Disclosures:**
Tijiang Zhang: Nothing to disclose
Kexin Huang: Nothing to disclose
Haifeng Ran: Nothing to disclose

**Brain Structural Connectivity alteration and its role in Language processing in Post-stroke Aphasia**
*N-T. Hoang*, T. Hada, C. D. Kulathilake, T. A. Walpola, N. Dilhani, A. Senoo; Tokyo/JP
*(hnthanh@huemed-univ.edu.vn)*

**Purpose or Learning Objective:** Our main objectives are to evaluate the brain connectivity in post-stroke aphasia (PSA) and its correlation with language ability.
**Methods or Background:** Tractography of 20 PSA was reconstructed by using deterministic algorithms. Brodmann atlas was used to brain parcellation. Connectivity analysis with following network measures: density (den), clustering coefficient (Cc), transitivity (trans), characteristic path length (CPL), small worldness (Sw), global efficiency (G_eff), radius (r), diameter (d), assortativity coefficient (A_coeff), rich-club coefficient (club_coeff) were extracted. All statistical analysis were performed using SPSS version 27.
**Results or Findings:** There are significantly lower value of CPL (p = 0.046), A_coeff (p = 0.026), diameter (p = 0.031), and higher value of G_eff (p = 0.046) in the high-level repetition group (n = 6) compared to the low-level repetition group (n = 14). This findings indicate the brain networks of the high-level group are more efficient in transferring information. Additionally, the network connections in the high-level group are more random or diverse regarding node characteristics, whereas networks in the low-level group may display more organized connections between similar nodes. There are strong positive correlations between the speaking sentence repetition and d (r = 0.593, p = 0.006), Sw (r = 0.580, p = 0.007), G_eff (r = 0.566, p = 0.009), and strong negative correlation between the speaking sentence repetition and CPL (r = 0.571, p = 0.009).
**Conclusion:** The high-level repetition group transfers information across regions more efficiently than the low-level group. It indicates the potential for rehabilitation strategies and emphasizing the need for personalized approaches in aphasia treatment.
**Limitations:** This research have some limitations. Our small sample cannot represent for PSA population. Additionally, the lack of longitudinal research is insufficient to determine the impact of network connectivity on language recovery.
**Funding for this study:** Research grant was provided by the Tokyo Global Partner Scholarship Program
**Ethics committee - additional information:** This study is being approved by the ethics committee
**Author Disclosures:**
Niluka Dilhani: Nothing to disclose
Thishuli Anujaya Walpola: Nothing to disclose
Ngoc-Thanh Hoang: Nothing to disclose
Atsushi Senoo: Nothing to disclose
Chathura Darshana Kulathilake: Nothing to disclose
Takuya Hada: Nothing to disclose

**Exploring brain region changes following acute caffeine intake based on ASL and OEF**
*Z. Shu*; Zhejiang, Hangzhou/CN
*(cooljuty@hotmail.com)*

**Purpose or Learning Objective:** This study aims to employ Arterial Spin Labeling (ASL) and Oxygen Extraction Fraction (OEF) to assess the impact of caffeine consumption on local cerebral blood flow perfusion and brain oxygen metabolism.
**Methods or Background:** A prospective study was conducted involving 18 healthy young volunteers with no habitual caffeine consumption. ASL and OEF images were acquired both prior to and 90 minutes following the administration of 200 mg of caffeine. Voxel-based analysis was employed to quantify alterations in brain oxygen metabolism across various cerebral regions before and after caffeine consumption. Regional cerebral blood flow perfusion was assessed in areas exhibiting changes in brain oxygen metabolism, with a focus on evaluating the extent of perfusion variation subsequent to caffeine intake. Additionally, the correlation between OEF indices in regions with modified cerebral blood flow and shifts in Karolinska Sleepiness Scale (KSS) scores was analyzed.
**Results or Findings:** In regions exhibiting alterations in brain oxygen metabolism following caffeine consumption, significant reductions in cerebral blood flow perfusion were identified within the medial occipital-temporal areas of both hemispheres, the right anterior cingulate gyrus, the left postcentral gyrus, and the left cerebellum (P<0.05). Notably, only the left cerebellum demonstrated an increase in brain oxygen metabolism. A negative relationship between brain oxygen metabolism and cerebral blood flow perfusion in the left cerebellum after caffeine intake (r=-0.5, p<0.05). Furthermore, a negative correlation was observed between changes in brain oxygen metabolism and shifts in KSS scores before and after caffeine consumption (r=-0.738, p<0.05).
**Conclusion:** The increase in brain oxygen metabolism in the left cerebellum may compensate for the reduced cerebral blood flow perfusion following acute caffeine intake, which could be a key factor in reducing drowsiness.

**Limitations:** The research sample size only focuses on young people
**Funding for this study:** The work was supported by the National Natural Science Foundation of China (Grant No.82101983)
**Ethics committee - additional information:** Ethics Committee of Zhejiang Provincial People's Hospital
**Author Disclosures:**
Zhenyu Shu: Nothing to disclose

**09:30-11:00** **Research Stage 4**

### Research Presentation Session: Chest

### RPS 1804

### Technological advancements in chest imaging: MRI, photon counting CT and more

**Moderator**
P. Ciet; Rotterdam/NL
*(p.ciet@erasmusmc.nl)*
**Author Disclosures:**
Pierluigi Ciet: Advisory Board: European Medicine Agency (EMA); Board Member: European Society of Pediatric Radiology (ESPR) Research Committee, AI and Cardiothoracic Taskforces; Consultant: Siemens Healthineers, Vertex Pharmaceutical; Employee: Erasmus MC - Sophia Children's Hospital; Grant Recipient: Research Dutch Council, Horizon Pathfinder; Research Grant/Support: General Electrics; Speaker: ECR, ESPR, ERS, InSpIrEd.

### Characterization of Interstitial Lung Abnormalities and Prediction of Disease Progression with MRI

*D. Kütting*, J. A. Luetkens, D. Thomas, A. M. C. Boehner, T. Dell, A. Faron; Bonn/DE
*(daniel@kuetting.de)*

**Purpose or Learning Objective:** Interstitial lung abnormalities (ILA) impact survival and quality of life, yet predictive imaging markers for progression are lacking. MRI holds promise in enhancing ILA phenotyping, potentially enabling tailored follow-up strategies while reducing repetitive CT imaging and associated radiation exposure.
**Methods or Background:** Assessment of detectability and estimation of disease progression of ILA in a single center, lung cancer screening cohort (224 participants) receiving same day CT/MRI. Radiologists independently evaluated chest images for presence of ILA using standardized criteria. Follow-up exams were reviewed for disease progression. MRI sequences included T2-TSE MVXD, T2-STIR, and diffusion-weighted imaging (DWI). Statistical analyses evaluated the agreement between CT and MRI findings and MRI's diagnostic performance for ILA detection and progression prediction.
**Results or Findings:** Among the 224 participants (mean age 58.5 ± 5.7 years; 45% female), 26 exhibited ILA on baseline CT, with 65% categorized as subpleural fibrotic. Baseline CT findings served as the reference standard. MRI detected ILA in 30 cases, 20 of which were congruent with CT findings, yielding a sensitivity of 76.9% and a specificity of 94.9% (McNemar's test, p=0.3173). MRI detected ILA in 19/26 cases using T2-TSE MVXD, 20/26 using T2-STIR (7/20 with hyperintense signal), and 6/26 using DWI (3/6 with hyperintense signal). Seven subjects showed progressive disease on follow-up, with 6 of the subjects initially having a subpleural fibrotic pattern. Hyperintense signals in STIR and DWI sequences predicted progression, with hazard ratios of 6.79 and 5.43, respectively. The combination of hyperintense signals in STIR and DWI had a positive predictive value of 100%.
**Conclusion:** MRI reliably detected ILA and predicted disease progression, particularly in the fibrotic subtype. MRI offered valuable insights for ILA monitoring and phenotyping, potentially improving patient management and reducing radiation exposure.
**Limitations:** Limited amount of patients
**Funding for this study:** No funding
**Ethics committee - additional information:** University Hospital Bonn
**Author Disclosures:**
Julian Alexander Luetkens: Nothing to disclose
Tatjana Dell: Nothing to disclose
Anton Faron: Nothing to disclose
Daniel Thomas: Nothing to disclose
Alexander Marc Christian Boehner: Nothing to disclose
Daniel Kütting: Nothing to disclose

### Visualized quantitative evaluation of regional ventilation and perfusion in patients with COPD using MRI phase-resolved functional lung imaging (PREFUL)

Z. M. Xie, X. Gao, J. Gu, Z. Zhang, H. Yu, *L. Zhu*; Shanghai/CN

**Purpose or Learning Objective:** To evaluate the clinical value of phase-resolved functional lung imaging(PREFUL)in diagnosing and regional specificity assessment of ventilation and perfusion status of chronic obstructive pulmonary disease (COPD) patients of different severity.
**Methods or Background:** 100 healthy volunteers, 40 patients with COPD(18 as GOLD1, 10 as GOLD2, 7 as GOLD3, and 5 as GOLD4 )underwent MRI using 3D PREFUL under free breathing at 3.0 T(Free-breathing 1H MRI acquisition, no contrast agent administration). The PREFUL postprocessing method was used for the extraction of dynamic perfusion and ventilation parameters. Mean ventilation and perfusion maps, ventilation flow-volume loops(FVL) correlation, ventilation defect percentage(VDP), perfusion defect percentage (QDP), map of ventilation/perfusion defects (V/Q defects), and matched defect percentage on both perfusion and ventilation maps (VQM) were calculated.
**Results or Findings:** Compare to the homogenous ventilation and perfusion maps of healthy volunteers, COPD patients showed significant heterogeneity. The mean ventilation and perfusion percentage in COPD patients were significantly lower than the healthy volunteers (P<0.01), while the FVL is statistically higher in COPD patients (P<0.01). The ventilation map showed regional differences in visual agreement with emphysema on CT and all 3D PREFUL-derived ventilation parameters correlated with FEV1 and FEV1/FVC in the patients with COPD(all P<0.05). Besides, our data showed a trend of correlation between different GOLD grades and VDP (P<0.05) in the COPD patients, but no significant difference in QDP among groups. Compared to patients with low-grade COPD (GOLD 1-2), severe COPD (GOLD 3-4) had higher VQM which indicated a better consistency of regional defect in ventilation and perfusion maps(P<0.01).
**Conclusion:** MRI PREFUL plays a promising role in evaluating the severity of COPD and visually predicting regional ventilation and perfusion defect in 3D lung imaging.
**Limitations:** The study included a relatively small sample size of COPD patients
**Funding for this study:** This work was supported in part by the National Natural Science Foundation of China (8207070786), Young Scientists Fund of the National Natural Science Foundation of China (82302188), Shanghai Pujiang Program (22PJD069).
**Ethics committee - additional information:** Shanghai Chest Hospital ethics committee
**Author Disclosures:**
Zi Ming Xie: Nothing to disclose
Junfeng Gu: Nothing to disclose
Zhengqi Zhang: Nothing to disclose
Xiaokun Gao: Nothing to disclose
Hong Yu: Nothing to disclose
Lin Zhu: Nothing to disclose

### AI-Enhanced 3D Gradient Echo MRI: An Alternative for Lung Nodule Detection and Assessment

A. W. Marka[1], M. Steinhardt[1], M. Graf[1], L. Rahn[1], K. Weiss[2], M. R. Makowski[1], D. C. Karampinos[1], J. Gawlitza[1], *S. Ziegelmayer*[1]; [1]Munich/DE, [2]Hamburg/DE

**Purpose or Learning Objective:** Recent years have seen significant progress in pulmonary MR imaging for lung nodule detection through optimization and new sequences. This study evaluates the capabilities of a 3D gradient echo MRI sequence for detection and classification of pulmonary nodules, specifically in relation to the Lung CT Screening Reporting and Data System (Lung-RADS).
**Methods or Background:** In this prospective trial, patients with benign and malignant lung nodules admitted between December 2021 and July 2024 underwent low-dose chest CT and pulmonary MRI using a 3D gradient echo sequence, accelerated by parallel imaging, compressed sensing, and deep learning (CSAI). Three radiologists (4, 9, and 10 years of experience), blinded to clinical information, independently evaluated the MR images. Nodule detection, characterization (size, morphology), and Lung-RADS assessment were performed for all patients. To quantify interreader agreement, intraclass correlation coefficient (ICC) for nodule measurements and Cohen’s kappa for Lung-RADS classifications were calculated.
**Results or Findings:** A total of 75 patients (mean [SD] age, 65±12 years; 33 women [44%]) with 135 pulmonary nodules were included and analyzed. Nominal scan time was 3:53 min. The CSAI sequence achieved a detection rate of 96,3%, with 5 missed nodules all being ≤4mm. The mean nodule diameter for MRI deviated from CT by 0.1 mm (1.96 SD5.87mm; -1.96 SD-5.67mm). Nodule size for CT and MRI showed excellent inter-rater agreement (ICC-CT: 0.995, CI95: 0.993, 0.996; ICC-CSAI: 0.993, CI95: 0.991, 0.995). Lung-RADS category agreement between CT and MRI was almost perfect for

Reader 2 (k=0.86) and Reader 3 (k=0.90), while Reader 1 showed substantial agreement (k=0.69).
**Conclusion:** Pulmonary MRI with an accelerated 3D gradient echo sequence showed high detection rates for pulmonary nodules with comparable Lung-RADs scores and morphological assessments to CT.
**Limitations:** -Heterogeneous cohort
-No follow-up scans
**Funding for this study:** None
**Ethics committee - additional information:** It was approved by the local ethical review board (protocol number 692/21S).
**Author Disclosures:**
Markus Graf: Nothing to disclose
Marcus R. Makowski: Nothing to disclose
Alexander Wolfgang Marka: Nothing to disclose
Sebastian Ziegelmayer: Nothing to disclose
Kilian Weiss: Nothing to disclose
Leonie Rahn: Nothing to disclose
Dimitrios C. Karampinos: Nothing to disclose
Joshua Gawlitza: Nothing to disclose
Manuel Steinhardt: Nothing to disclose

**Conjugate Gradient and Deep Learning Reconstructions: Utility for Lung MRI with Ultra-Short TE to Reduce Acquisition Time with Keeping Image Quality and Nodule Detection Capability**
*Y. Ohno*, H. Nagata, T. Ueda, M. Nomura, T. Yoshikawa, D. Takenaka, Y. Ozawa; Toyoake/JP
*(yohno@fujita-hu.ac.jp)*

**Purpose or Learning Objective:** To determine capability of Conjugate gradient reconstruction (CG-recon) and deep learning reconstruction (DLR) for reducing acquisition time with keeping image quality and nodule detection performance on UTE-MRI.
**Methods or Background:** 35 patients with lung nodule underwent UTE-MRI obtained with CG-recon and grid-reconstruction (Grid-recon) by original (UTEoriginal), 1/2 (UTE1/2) and 1/4 (UTE1/4) sampling spoke numbers at 1.5T and 3T systems. Then, each UTE-MRI was reconstructed with and without DLR. Standard protocol in this study was UTEoriginal obtained by Grid-recon and reconstructed without DLR. In each patient, standard reference for nodule was determined by thin-section CT. To determine the influence of sampling spoke number reduction and reconstruction method differences, signal-to-noise ratios (SNRs) of lung and nodule, overall image quality and nodule presence probability were assessed by ROI measurements or 5-point scales. SNRs and overall image quality were compared between each UTE-MRI and standard protocol by Student's t-test or Wilcoxon's signed rank test. Then, ROC analysis was performed to compare nodule detection capability between each UTE-MRI and standard protocol.
**Results or Findings:** DLR was significantly improved SNRs of all UTEoriginals and UTE1/2 obtained by CG-recon as compared with standard protocol (p<0.05). Overall image qualities of each UTE1/4 and all UTE1/2s except that obtained by CG-recon and reconstructed with DLR were significantly lower than that of standard protocol (p<0.05). Area under the curve (Az) of standard protocol (Az=0.97) was significantly larger than that of all UTE1/4s (0.82<Az<0.92, p<0.0001) and UTE1/2 obtained by Grid-recon and reconstructed without DLR (Az=0.94, p=0.03), although it was significantly smaller than that of UTEoriginals obtained by CG-recon (Az=0.98, p<0.05).
**Conclusion:** CG-recon and DLR can reduce acquisition time without degradation of image quality and nodule detection on UTE-MRI.
**Limitations:** Limited study population and nodule numbers
**Funding for this study:** Canon Medical Systems Corporation
**Ethics committee - additional information:** Fujita Health University Hospital
**Author Disclosures:**
Yoshiyuki Ozawa: Research/Grant Support: Smoking Research Foundation Research/Grant Support: Grant-in-Aid for Scientific Research from the Japanese Ministry of Education, Culture, Sports, Science and Technology
Masahiko Nomura: Nothing to disclose
Takahiro Ueda: Research/Grant Support: Grant-in-Aid for Scientific Research from the Japanese Ministry of Education, Culture, Sports, Science and Technology
Daisuke Takenaka: Nothing to disclose
Hiroyuki Nagata: Research/Grant Support: Canon Medical Systems Corporation Research/Grant Support: Grants-in-Aid for Scientific Research from the Japanese Ministry of Education, Culture, Sports, Science and Technology
Takeshi Yoshikawa: Nothing to disclose
Yoshiharu Ohno: Research/Grant Support: Canon Medical Systems Corporation Research/Grant Support: Smoking Research Foundation

**Visual analysis of dynamic oxygen-enhanced MRI (OE-MRI): Comparison with V/Q SPECT and MR perfusion in chronic thromboembolic pulmonary hypertension**
*G. Agarwal*[1], D. Gopalan[1], M. Naik[1], N. Soneji[1], B. Statton[1], B. Ariff[1], M. Tibiletti[2], G. Parker[2], S. Copley[1]; [1]London/UK, [2]Manchester/UK
*(girija.agarwal@nhs.net)*

**Purpose or Learning Objective:** Chronic thromboembolic pulmonary hypertension (CTEPH) is an under-recognized condition associated with significant morbidity yet is surgically treatable. Oxygen-enhanced MRI (OE-MRI) is an emerging tool for quantifying and mapping regional gas delivery and uptake without the need for hyperpolarized gas. This study evaluates the accuracy of visual analysis of OE-MRI for diagnosing CTEPH compared to the current standard of care: V/Q SPECT and MR perfusion (MR-P).
**Methods or Background:** Prospective study conducted from 2018 to 2023. Participants with a clinical suspicion of CTEPH underwent T1-weighted dynamic OE-MRI on a 1.5T scanner, V/Q SPECT and MR-P. Four consultant radiologists (2 MRI, 2 NM specialists) scored relevant scans independently (positive, negative, or indeterminate) for CTEPH blinded to clinical data with differences resolved by consensus. The reference standard was the multidisciplinary team diagnosis of CTEPH.
**Results or Findings:** A total of 58 patients were included, with 49 undergoing both OE-MRI and V/Q SPECT, and 48 undergoing all three modalities. Studies considered indeterminate for CTEPH were excluded from sensitivity and specificity analyses (2/49 V/Q, 1/48 MR-P, and 1/49 OE-MRI). The sensitivity of OE-MRI, V/Q SPECT and MR-P were 0.932 (95% CI 0.78-0.981), 0.964 (95% CI 0.823-0.994) and 0.931 (95% CI 0.78-0.931) respectively. Specificities were 0.789 (95% CI 0.567-0.915), 0.947 (95% CI 0.754-0.991) and 0.833 (95% CI 0.608-0.942) respectively. There was no statistically significant difference between OE-MRI and V/Q or OE-MRI and MR-P using McNemar test (P > .05).
**Conclusion:** Visual analysis of OE-MRI maps is a valuable adjunct in diagnosing CTEPH with similar sensitivity but lower specificity than VQ SPECT and MR-P. Further analysis of quantitative data is required to fully assess the role of this technique.
**Limitations:** Limitations are a relatively small sample size (n=58) and only visual (not quantitative) analysis.
**Funding for this study:** Study funded by NIHR Imperial Biomedical Research Centre (BRC) grant
**Ethics committee - additional information:** Informed consent obtained from all patients for the extra oxygen-enhanced MRI sequence, the other investigations were standard of care.
**Author Disclosures:**
Deepa Gopalan: Nothing to disclose
Susan Copley: Nothing to disclose
Mitesh Naik: Nothing to disclose
Geoff Parker: Employee: Bioxydyn Ltd Board Member: Quantitative Imaging Ltd and Bioxydyn Ltd Shareholder: Quantitative Imaging Ltd and Bioxydyn Ltd
Ben Statton: Nothing to disclose
Girija Agarwal: Nothing to disclose
Marta Tibiletti: Board Member: Bioxydyn Limited Shareholder: Quantitative Imaging Ltd Employee: Bioxydyn Limited
Neil Soneji: Nothing to disclose
Ben Ariff: Nothing to disclose

**Early clinical experiences for chest imaging with a new photon counting CT system combining cadmium zinc telluride detectors and super-high resolution deep-learning image reconstruction**
S. S. Schalekamp, L. J. Oostveen, M. Simmelink, W-J. Van Der Woude, P. P. P. Van Der Tol, M. Prokop, *E. J. Smit*; Nijmegen/NL
*(ewoudsmit@gmail.com)*

**Purpose or Learning Objective:** To assess the image quality of chest scans acquired using a photon-counting CT (PCCT) scanner with cadmium-zinc-telluride (CZT) detectors and super-high resolution deep-learning image reconstruction (SHR-DLR).
**Methods or Background:** We analyzed the chest images from two consecutive cohorts of 18 and 25 patients who underwent imaging on a prototype PCCT scanner for various indications. Images were reconstructed using both normal resolution (NR: 0.62mm sections, 512-matrix, hybrid-iterative-reconstruction) and super-high resolution (SHR: 0.21mm sections, 1024-matrix, deep-learning-reconstruction) protocols. An experienced chest radiologist assessed image quality using a 5-point scale (poor to excellent) for overall quality, sharpness, detail visibility, noise, and artifacts. A homogeneous region in the left ventricle was used to measure image noise. The number of visible bronchial branching generations was quantified in three lung regions: the upper-right-lobe(1R), the upper-left-lobe(5L), and the right-lower-lobe(10R). Bronchus volumes were automatically calculated (first cohort only). Statistical significance was determined using a signed rank test (p<0.05).

**Results or Findings:** The SHR-DLR images were rated to have higher overall image quality (4.7 vs 3.6), image sharpness (4.8 vs 3.3), detail visibility (4.7 vs 3.6) compared to the NR images, while having lower perceived image noise (4.3 vs 3.3; all p<0.01). No image artifacts were observed with either protocol (both 4.0). Although measured noise levels were similar between SHR-DLR (33.2 HU) and NR (34.2 HU), SHR-DLR images demonstrated a finer noise texture. SHR-DLR enabled visualization of finer bronchial details, with 1.2 more peripheral branches visible on average (p<0.01). Automatic segmentation showed larger bronchus volumes in the SHR-DLR images (54.1 mL) compared to the NR images (47.2 mL;p<0.01).
**Conclusion:** PCCT with CZT detectors and SHR-DLR reconstruction provides excellent spatial resolution and superior visualization of the bronchial system while maintaining low image noise.
**Limitations:** No comparison to conventional CT.
**Funding for this study:** None
**Ethics committee - additional information:** Waived
**Author Disclosures:**
Pieternel P P Van Der Tol: Nothing to disclose
Steven S Schalekamp: Nothing to disclose
Luuk J. Oostveen: Nothing to disclose
Mathias Prokop: Research/Grant Support: Canon Medical Systems Speaker: Canon Medical Systems
Willem-Jan Van Der Woude: Nothing to disclose
Mirte Simmelink: Nothing to disclose
Ewoud J. Smit: Speaker: Canon Medical Systems

**Feasibility study on Photon-Counting CT-derived virtual non-contrast images substitute for true non-contrast images**
L. Lei, *Y. Zhou*; Zhengzhou City/CN
*(zyh921209zyh@163.com)*

**Purpose or Learning Objective:** To explore the feasibility of the virtual non-contrast images derived from Photon-Counting CT (PHCT) substitute for true non-contrast images.
**Methods or Background:** 40 patients underwent pre-and arterial-venous dual-phase post-contrast chest imagining on a PHCT and had previously undergone a chest CT with a standard energy-integrating detector system (EID-CT) scanner were retrospectively included in this study. The images were retrospectively analyzed. The arterial VNC images (VNC-A) and venous VNC images (VNC-V) were derived from raw datasets using dedicated software respectively. Two radiologists assessed image quality using a five-point Likert scale and performed measurements of vessels and lung parenchyma for signal-to-noise ratio (SNR), contrast-to-noise ratio (CNR), and in the case of solid lung masses-to-lung parenchyma contrast ratio.
**Results or Findings:** The image noise of all tissues among the four kinds of images had significant differences, images noise of VNC-A and VNC-V images were lower than TNC images and EID-CT images (P＜0.05), and VNC-V images had the highest SNR and CNR. Good equivalence between VNC and TNC images was observed in all relevant tissues with Bland-Altman analysis. Image quality subjective scoring of EID-CT, TNC, VNC-A, and VNC-V were 5.00(1.00), 5.00(1.00), 5.00(0.75), 5.00(1.00), respectively which had no significant differences (P=0.20).
**Conclusion:** The VNC image derived from PHCT enhanced image might be used as a substitute for the TNC image, and the image quality is higher than conventional EID-CT images.
**Limitations:** Not applicable
**Funding for this study:** Not applicable
**Ethics committee - additional information:** This study was approved by the Human Research Ethics Committee.
**Author Disclosures:**
Limin Lei: Nothing to disclose
Yuhan Zhou: Nothing to disclose

**Quantitative Lung Imaging using Ultra High-Resolution Spectral Capabilities of CZT-based Photon-Counting Detector CT: A Feasibility Study**
*S. Sharma*[1], S. Ross[1], T. Labno[1], R. Zhang[1], X. Zhan[1], R. Thompson[2], Z. Yu[1], A. Pourmorteza[3]; [1]Vernon Hills, IL/US, [2]Cleveland, OH/US, [3]Atlanta, GA/US
*(shsharma@mru.medical.canon)*

**Purpose or Learning Objective:** To evaluate ultra-high-resolution spectral capabilities of CZT-based photon-counting detector CT (PCD-CT) for quantitative lung imaging.
**Methods or Background:** A COPDGene2 phantom, with three reference foams (20-lb, 12-lb, and 4-lb with HU-120kVp of -703, -824, and -937) and airways (inner-diameter (ID): 2.5-6 mm, wall-thickness (WT): 0.4-1.5 mm), was scanned on a CZT-based PCD-CT (120 kVp, 0.4x0.5 mm focal-spot, and CTDIvol =12.8 mGy). Scans were reconstructed in normal-resolution (NR) and ultra-high-resolution (UHR) spectral modes (pixel size (PS): 0.125 mm, slice thickness (ST): 0.2 and 0.6 mm, respectively), with a lung kernel (FC52) and iterative denoising, followed by generation of 40-150 keV VMIs. For evaluation, the following were quantified: (1) HU bias between UHR and NR VMIs (ST=3.0 mm), (2) contrast (C) and contrast-to-noise ratios (CNRs) for ground-glass nodules (GGNs) and emphysema (ES) (ST=3.0 mm) (20-lb, 12-lb, and 4-lb foams were surrogates for GGN, normal lung, and ES), and (3) IDs and WTs in VMI with maximum CNR(GGN) and CNR(ES) (ST=0.6 mm).
**Results or Findings:** Bias between UHR-VMI and NR-VMI was found to be <5 HU for all materials. Noise was greater in UHR-VMI (8.2-22.6%) than NR-VMI. C(GGN) and C(ES) improved in UHR-VMI compared to non-spectral UHR images at <60 keV (max: 4.0 HU) and >60 keV (max: 3.5 HU), respectively. The 70 keV VMI was optimal for both CNR(GGN) and CNR(ES) due to minimal noise amplification. For airways, errors (mean±σ) (mm) in 70 keV UHR-VMI were lower: (UHR/NR) 0.06±0.09/0.15±0.19 (WT) and -0.18±0.17/-0.29±0.31 (ID), with highest improvements for smaller airways.
**Conclusion:** Spectral-UHR imaging with CZT-based PCD-CT offers diagnostically-relevant contrast improvements between diseased and normal lung over non-spectral images, with reduced measurement errors for airways.
**Limitations:** Study used a phantom approximating compositional similarity to normal and diseased lung instead of real patients.
**Funding for this study:** N/A
**Ethics committee - additional information:** N/A
**Author Disclosures:**
Zhou Yu: Employee: Canon Medical Research USA, Inc.
Steven Ross: Employee: Canon Medical Research USA, Inc.
Richard Thompson: Employee: Canon Healthcare USA, Inc.
Amir Pourmorteza: Nothing to disclose
Tom Labno: Employee: Canon Medical Research USA, Inc.
Ruoqiao Zhang: Employee: Canon Medical Research USA, Inc.
Xiaohui Zhan: Employee: Canon Medical Research USA, Inc.
Shobhit Sharma: Employee: Canon Medical Research USA, Inc.

**Ultra low dose Photon Counting CT versus low dose photon counting CT in patients with cystic fibrosis**
*L. G. Murkes*, M. Lidegran, M. Sund, P. Hillergren, S. Diaz; Stockholm/SE
*(lena.gordon-murkes@regionstockholm.se)*

**Purpose or Learning Objective:** To introduce ultra low dose photon counting CT (ULDPCCT) as the main diagnostic follow up method in patients with cystic fibrosis (CF) and thereby reduce radiation dose with preserved or improved diagnostic value.
**Methods or Background:** Patients with CF undergo lifelong yearly follow up alternating CT and chest radiography from an early age. CT is the method of choice and provides important information about the course of the disease, making low and ultralow dose methods imperative. This prospective study included 71 CF patients between 7 and 66 years of age. A specific study protocol was set up on a photon counting detector CT, Siemens Naeotom Alpha. All patients included underwent an inhaled and exhaled ULDPCCT and low dose PCCT (LDPCCT) examinations at their yearly follow up. Radiation doses were collected for each scan and patient. The median was also calculated. Images from all scans were assessed separately by two paediatric radiologists with different years of experience using a modified Bhalla scoring system. Interobserver agreement was calculated with Cohens' kappa coefficient. P-values of <0.05 were considered statistically significant.
**Results or Findings:** The effective dose median (IQR) was 0,11 mSv (0.1-0.13) for the ULDPCCT and 0,77 mSv (0.66-0.87) for the LDPCCT respectively. There was no statistically significant difference between the Bhalla scoring when comparing ULDPCCT versus LDPCCT scans with p-value 0,71 (0,37;1,04). Interobserver agreement was substantial (Kappa value 0.65 for ULDPCCT and 0.71 for LDPCCT)
**Conclusion:** A tailored ULDPCCT scan protocol might be used as yearly follow up diagnostic tool in CF patients with detailed diagnostic value and reduction of the radiation dose at approximately 1/7th of a regular LDCT, thereby reducing the accumulative dose contribution to the patient.
**Limitations:** Relatively limited amount of patients.
**Funding for this study:** Funding was provided by " Riksförbundet Cystisk Fibros " for the statistical analysis
**Ethics committee - additional information:** The study was approved by the swedish ethics commitee. According to the declaration of Helsinkii. Dnr 2023-01227-01
**Author Disclosures:**
Marika Lidegran: Nothing to disclose
Pierre Hillergren: Nothing to disclose
Lena Gordon Murkes: Nothing to disclose
Sandra Diaz: Nothing to disclose
Marie Sund: Nothing to disclose

**AI-based body composition analysis of COPD patients' CT scans – a multicentric study**

*B. K. Budai*[1], S. Hettinger[1], V. M. Wagner[1], V. Palm[1], R. Hosch[2], F. Nensa[2], O. Von Stackelberg[1], H-U. Kauczor[1], J. Biederer[1]; [1]Heidelberg/DE, [2]Essen/DE

**Purpose or Learning Objective:** This study focused on AI-based CT body composition analysis (BCA) as an alternative to bioelectrical impedance analysis (BIA) for identifying COPD patients at high risk of sarcopenia. We aimed to construct CT-based linear regression models for predicting the patients' fat mass (FM), fat-free mass (FFM), skeletal muscle mass (SMM), and total muscle mass (MM).
**Methods or Background:** A total of 571 COPD patients (349 males (61.1%), aged 65.5 ± 8.6y) from a prospective multicentric study (COSYCONET) underwent baseline chest CT scans and BIA. The AI-based BCA of inspiratory chest CTs was performed by the "Body and Organ Analysis" (BOA) algorithm. Volumes of muscles, bones, and fatty tissues were converted to mass in kg using standard human tissue densities. Linear regression with estimated thorax weight fitted to patient weight was used to extract residuals which combined with the respective CT-based measures, age, sex, weight, and height were used for predicting BIA-based results. The reliability of CT-predicted body composition measures was evaluated with intraclass correlation coefficients (ICC). The performance of the CT-based FFMI in identifying high-risk sarcopenia patients was assessed with ROC curve analysis.
**Results or Findings:** The CT-based estimated body composition measures correlated well with BIA with ICCs of 0.90 (FM), 0.94 (FFM), 0.92 (SMM), and 0.92 (MM). The CT-based FFMI achieved an ICC=0.88 and predicted high-risk sarcopenia patients with an AUC, accuracy, sensitivity, and specificity of 0.903, 88.3%, 88.7%, and 88.2%, respectively. Gwet's AC1 of 0.82 suggested excellent agreement between the two approaches.
**Conclusion:** AI-based body composition analysis of chest CT scans could be used to assess BIA-based body composition measures of COPD patients and to identify patients at high risk of sarcopenia.
**Limitations:** The limitation of the study is that no external validation was performed.
**Funding for this study:** Funding was provided by the German Federal Ministry of Education and Research (BMBF) (Projektträger: DLR e.V. Bonn, Funding ref. 01GI0884)
**Ethics committee - additional information:** This study was approved by the local ethics committee and the central ethics committee of the multicenter study.
**Author Disclosures:**
Jürgen Biederer: Nothing to disclose
Sophia Hettinger: Nothing to disclose
Hans-Ulrich Kauczor: Nothing to disclose
Oyunbileg Von Stackelberg: Nothing to disclose
Verena Maria Wagner: Nothing to disclose
Viktoria Palm: Nothing to disclose
René Hosch: Nothing to disclose
Bettina Katalin Budai: Nothing to disclose
Felix Nensa: Nothing to disclose

**Artificial intelligent based automated detection of chest x-ray abnormalities as a support for young radiologists**

*L. Giuliani*, G. M. Masci, N. Landini, P. Giuliani, V. Panebianco, C. Catalano; Rome/IT
*(lucagiuliani92@virgilio.it)*

**Purpose or Learning Objective:** To investigate whether AI represents an added value for chest X-ray (CXR) interpretation.
**Methods or Background:** A dataset of CXR performed between March 2023 and January 2024 were retrospectively selected from the institutional PACS by a senior thoracic radiologist. All CXR were evaluated by two young radiologists with 1 year of experience in chest imaging, who assessed the presence of several findings (consolidation, nodule, atelectasis, fibrosis, calcification, pneumothorax, cardiomegaly, pleural effusion, mediastinal enlargement, pneumoperitoneum). All examinations were then analyzed with an AI tool (Lunit INSIGHT CXR, Version 3.110) which assessed the same features. Finally, an additional AI-assisted evaluation was performed by the two radiologists. The ground truth was established by the radiologist in charge of image selection who classified the abnormalities as either visible or not visible on the radiograph.
**Results or Findings:** A total of 548 CXR examinations were selected. From the 500 CXR analyzed, a total of 876 findings were reported either from the radiologists or from the AI tool. The two radiologists showed a sensitivity/specificity of 80.6%/93.2% and 87.2%/95.3%, respectively. AI showed a sensitivity/specificity of 96.9%/64.3%. With AI assistance, the sensitivity of the two radiologists increased to 82.9% (+2.3%) and 89.3% (+2.1%), while specificity decreased to 87.2% (-6%) and 89.5% (-5.8%). The abnormalities for which the radiologists showed higher disagreement with AI were fibrosis and calcification (p<0.0001), whilst the abnormalities for which the radiologists more often changed interpretation after AI evaluation were nodules, calcification, and pneumothorax (p<0.0001).
**Conclusion:** AI does not show significant increase of the diagnostic performance compared to standard radiological evaluation of CXR. Particularly, AI slightly increases sensitivity but at cost of a significant decrease in specificity. Therefore, standard radiological interpretation still remains the gold standard for CXR.
**Limitations:** It was conducted retrospectively
**Funding for this study:** No funding was received for this study.
**Ethics committee - additional information:** Sapienza University, Rome, Rif.7226, Prot.0473/2024
**Author Disclosures:**
Valeria Panebianco: Nothing to disclose
Nicholas Landini: Nothing to disclose
Paolo Giuliani: Nothing to disclose
Giorgio Maria Masci: Nothing to disclose
Carlo Catalano: Nothing to disclose
Luca Giuliani: Nothing to disclose

**Dark-field Chest Radiography for Pneumothorax Assessment**

*F. T. Gassert*, H. Bast, T. Urban, M. Lochschmidt, L. Kaster, T. Koehler, M. R. Makowski, F. Pfeiffer, D. Pfeiffer; Munich/DE

**Purpose or Learning Objective:** Conventional imaging techniques have limitations in early detection of pneumothorax, particularly for small pneumothoraces. Therefore, the purpose of this study was to evaluate the potential of dark-field chest radiography in improving the detection and assessment of pneumothorax.
**Methods or Background:** This study included 100 participants, comprising 36 patients with clinically diagnosed pneumothorax and 64 healthy controls. All participants underwent dark-field X-ray chest radiography using a prototype system that simultaneously acquires attenuation-based and dark-field images. Sensitivity, specificity, accuracy, reading time, and diagnostic confidence were compared between attenuation-based radiographs and the combination of attenuation-based radiographs with dark-field images (dark-field overlays).
**Results or Findings:** Dark-field radiography increased sensitivity for pneumothorax detection from 84.2% (attenuation-based radiographs) to 87.4% (dark-field overlays; p = .26), while specificity remained constant (97.3% vs. 97.4%). The median reading time was significantly reduced from 30.8 seconds to 10.3 seconds (p < .001), and diagnostic confidence improved significantly across all readers (p < .001).
**Conclusion:** Dark-field chest radiography enhances the detection of pneumothorax, significantly reducing reading time and increasing diagnostic confidence without compromising specificity.
**Limitations:** Inclusion criteria and the imaging modality they were assessed on were different for pneumothorax patients and controls. While for pneumothorax patients, a conventional radiograph showing a pneumothorax was sufficient, controls had to show a normal CT scan to be included.
**Funding for this study:** We acknowledge financial support through the European Research Council (AdG 695045), the Center for Advanced Laser Applications (CALA), the Federal Ministry of Education and Research (BMBF) and the Free State of Bavaria under the Excellence Strategy of the Federal Government and the Länder, the German Research Foundation (GRK2274), as well as by the Technical University of Munich–Institute for Advanced Study. This work was carried out with the support of the Karlsruhe Nano Micro Facility (KNMF, KNMF, www.kit.edu/knmf), a Helmholtz Research Infrastructure at Karlsruhe Institute of Technology (KIT).
**Ethics committee - additional information:** Ethics committee of the Technical University of Munich
**Author Disclosures:**
Henriette Bast: Nothing to disclose
Florian Tilman Gassert: Nothing to disclose
Thomas Koehler: Employee: Philips
Marcus R. Makowski: Nothing to disclose
Theresa Urban: Nothing to disclose
Lennard Kaster: Nothing to disclose
Franz Pfeiffer: Nothing to disclose
Maximilian Lochschmidt: Nothing to disclose
Daniela Pfeiffer: Nothing to disclose

**12:30-13:30** **Research Stage 1**

### Research Presentation Session: Breast

### RPS 1902

### Introduction of artificial intelligence in breast screening

**Moderator**
S. Schiaffino; San Donato Milanese/IT
*(schiaffino.simone@gmail.com)*
**Author Disclosures:**
Simone Schiaffino: Board Member: European Journal of Radiology, Eurorad, European Radiology Experimental; Speaker: GE Healthcare

**Impact on quality performance indicators after implementing AI in a breast cancer screening program in Germany**

K. Hamm[1], *A. Rodriguez Ruiz*[2], T. Jordan[1], B. Vetter[1], C. Engel[3], C. Entrup[4], M. Engelke[5]; [1]Chemnitz/DE, [2]Nijmegen/NL, [3]Leipzig/DE, [4]Koblenz/DE, [5]Hamburg/DE

**Purpose or Learning Objective:** To evaluate breast cancer screening quality indicators after implementation of an AI system for support reading mammograms.
**Methods or Background:** Two prospective and consecutive collected cohorts of women attending breast cancer screening with mammography in a region of Germany where identified, just before and after implementation of an AI decision support system to aid radiologists reading mammograms (Transpara version 1.7, ScreenPoint Medical). Before AI implementation, all mammograms were double read without AI. Afterwards, mammograms were double read using AI as concurrent decision support. All mammograms were acquired with same devices (Siemens Mammomat Inspiration). A total of X radiologists assessed the exams in this screening program. Screening quality indicators (cancer detection rate, recall rate, false positive rate, PPV2) were compared in the cohorts of women before and after implementation of AI using multivariate logistic models adjusted for age, breast density, and interval from previous examination.
**Results or Findings:** 59.676 women attending screening before AI implementation (2020-2021) and 58.546 women after AI implementation (2022-2023) were included in the analysis. Average age was 60 years old in both cohorts. Average number of months between rounds was 838 days in the no-AI cohort and 815 days in the AI cohort. After implementing AI, cancer detection rate increased (349 screen-detected cancers, 6.0/1000 vs 286 screen-detected cancers, 4.8/1000, p=0.01), recall rate remained stable (2.5% vs 2.6%, p=0.29), false positive rate was reduced (1.9% vs 2.1%, p=0.002), and PPV2 increased (69%, 349/509 vs 60%, 286/477, p=0.009).
**Conclusion:** Implementing AI to support radiologists reading mammograms in a breast cancer screening program in Germany is safe and effective, improving cancer detection rates and reducing false positives.
**Limitations:** This prospective study has a non-paired non-randomized design.
**Funding for this study:** None.
**Ethics committee - additional information:** Approved by local ethics committee.
**Author Disclosures:**
Alejandro Rodriguez Ruiz: Employee: ScreenPoint Medical
Martin Engelke: Nothing to disclose
Klaus Hamm: Nothing to disclose
Torsten Jordan: Nothing to disclose
Bert Vetter: Nothing to disclose
Christoph Engel: Nothing to disclose
Christian Entrup: Nothing to disclose

**Breast Cancer Characteristics after the introduction of Artificial Intelligence-supported double-reading in a Mammography Screening Program: comparison of baseline and subsequent rounds**

*C. M. Weiss*, E. Di Gaetano, E. Cattarin, R. Cerniato, G. Soppelsa, I. Vinci; Treviso/IT
*(claudiamaria.weiss@aulss2.veneto.it)*

**Purpose or Learning Objective:** To analyse the prognostic factors of breast cancers (BCs) detected with artificial intelligence-supported double-reading (AI-DR) and attempt to determine the long-term impact of these changes, particularly on possible overdiagnosis.
**Methods or Background:** AI-DR was applied to all digital screening mammograms (DSM) from November 2021 to June 2024: 99320 in the AI-baseline-screen (AIBS) and 21237 in the AI-subsequent-screen (AISS). The collected data were compared by retrospective analysis to determine whether AIBS screen-detected BCs differed from AISS. We used the Z-test to compare the proportions of the data between AIBS and AISS.
**Results or Findings:** With a total of 1093 screen-detected BCs (AIBS: 944/99320; AISS: 149/21367), the study revealed a decrease (-26.6%) in the cancer detection rate (CDR) per 1000 in AISS compared to AIBS (6.97vs9.5). The recall rate (RR) was lower (-42.3%) in AISS than in AIBS (1.8%vs3.1%). No significant differences were found in the percentage of invasive BCs (AIBS82%vsAISS81.9%) and in situ BCs (AIBS18%vsAISS18.1%). Higher percentages of luminal BCs were observed in the AIBS than in AISS (91.1%vs82.6%), while in AISS, there were higher percentages of high-grade (AIBS25.3%vs AISS33.6%), HER2positive (AIBS 8.3%vsAISS12.4%) and triple-negative BCs (AIBS2.6%vsAISS 5%).
**Conclusion:** The reduction of RR and CDR in AISS aligns with the expectation of later screening focusing on disease onset or progression cases. In AISS, compared to AIBS, more aggressive BCs were detected, while less aggressive BCs were reduced. This might suggest an improved performance in the second round of screening, with a positive impact on the reduction of overdiagnosis.
**Limitations:** Data on interval and advanced cancers are lacking, which would allow for an analysis of long-term clinical outcomes.
**Funding for this study:** No funding
**Ethics committee - additional information:** Not requested
**Author Disclosures:**
Giorgia Soppelsa: Nothing to disclose
Eleonora Di Gaetano: Nothing to disclose
Elisa Cattarin: Nothing to disclose
Ivana Vinci: Nothing to disclose
Claudia Maria Weiss: Nothing to disclose
Roberta Cerniato: Nothing to disclose

**Multicenter Analysis of AI Assessed Mammography Technologist Positioning Variability Between Breast Screening Programs**

*G. Spear*[1], L. R. Margolies[2], J. Payne[3], S. E. E. Iles[3], J. Seely[4], N. Sharma[5], S. H. Heywang-Köbrunner[6], T. W. W. Vomweg[7], M. Abdolell[3]; [1]Chicago, IL/US, [2]New York, NY/US, [3]Halifax, NS/CA, [4]Ottawa, ON/CA, [5]Leeds/UK, [6]Munich/DE, [7]Koblenz/DE
*(ggspear21@gmail.com)*

**Purpose or Learning Objective:** Variability in mammographic positioning quality, both between breast screening programs (BSPs) and across different positioning errors, presents a challenge to establishing standardized mammography quality service delivery. Although high-quality mammography helps ensure diagnostic accuracy, and training can enhance image quality, there is a lack of supporting population-level empirical data. This study aims to quantitatively assess mammography technologists' positioning error rates across BSPs.
**Methods or Background:** The MAMMO.IQ study encompassed a total of 249,817 screening mammograms acquired between December 1, 2019, and February 28, 2021, from seven BSPs across North America and Europe. The positioning errors assessed included: exaggeration, portion cut off, posterior tissues missing, nipple not in profile, too high on IR, pectoralis shape/position, sagging, IMF missing/obscured, PNL difference, and compression. The Coefficient of Variation (CV) assessed variability in error rates, (1) between BSPs, and (2) between positioning errors. The within-BSS CV for each unmet positioning criterion was computed using rates for all technologists within a BSS.
**Results or Findings:** Images acquired by 310 technologists were analyzed. Over/under compression had the lowest variability (CV=16.44%) indicating consistent practices. Too High on IR exhibited the highest variability (CV=71.52%) reflecting a high level of inconsistencies. The MLO Inadequate Pectoralis Length had a CV of 50.45%, representing the median level of variability.
**Conclusion:** This study highlights variability in mammography technologists' positioning errors between and within BSPs. While some positioning criteria show consistent practices, others may benefit from improved standardization. Understanding inconsistencies in mammography service delivery helps identify opportunities to standardize positioning practices and reduce variability, leading to more equitable, high-quality care and fewer positioning errors.
**Limitations:** Missing data on technologist experience, staffing, and COVID-19 response measures limits the understanding of factors driving disparities.
**Funding for this study:** No funding was received for this study.
**Ethics committee - additional information:** Ethics approvals were obtained from participating BSPs (NSHA-REB#1026590).

**Author Disclosures:**
Nisha Sharma: Advisory Board: Densitas
Laurie R. Margolies: Nothing to disclose
Sylvia H. Heywang-Köbrunner: Nothing to disclose
Sian E. Elizabeth Iles: Nothing to disclose
Jean Seely: Nothing to disclose
Jennifer Payne: Other: Densitas
Toni Werner W Vomweg: Nothing to disclose
Georgia Spear: Nothing to disclose
Mohamed Abdolell: CEO: Densitas

**Characteristics of Breast Cancers before and after the introduction of Artificial Intelligence-supported double reading in a Mammography Screening Program**

*C. M. Weiss*, E. Di Gaetano, E. Cattarin, R. Cerniato, G. Soppelsa, I. Vinci; Treviso/IT
*(claudiamaria.weiss@aulss2.veneto.it)*

**Purpose or Learning Objective:** Screening supported by artificial intelligence (AI) increased cancer detection compared to screening without AI. However, it is still unclear whether the additional cancer detection improves outcomes or leads to overdiagnosis of breast cancers (BCs).
**Methods or Background:** From January 2019 to October 2021, 134259 women underwent digital screening mammography (DSM) with human-double-reading (HDR) and from November 2021 to June 2024, 131406 DMS with AI-supported HDR (AI-HDR) DM. The collected data (cancer detection rate [CDR], recall rate [RR] and tumour characteristics) were compared by retrospective analysis to determine whether the BCs detected by screening differed between HDR and AI-HDR. We used the Z-test to compare the proportions of the data between HDR and AI-HDR.
**Results or Findings:** With a total of 2044 screen-detected BCs (HDR:938/134259; AI-HDR:1106/131406), the study revealed a significant increase (+20.5%) in CDR per 1000 with AI-HDR compared to HDR (8.42vs6.99 per 1000, respectively). The RR was lower (-14.9%) with AI-HDR than with HDR (2.6%vs3.1%). The AI-HDR showed the following differences in BCs rates compared to HDR: lower for invasive BCs (82.1%vs84.1%), and higher for in situ BCs (17.9%vs15.9%); higher moderate-grade BCs (65.5%vs61.5%), and lower high-grade BCs (26.4%vs 27.9%); higher luminal BCs (88.2%vs83.2%), lower HER2positive (8.7%vs13.2%), and lower triple negatives (2.9%vs3.6%).
**Conclusion:** It can be concluded that the use of AI-HDR produced statistically significant differences in detecting various tumour subtypes compared to HDR. In particular, it seems to have increased the detection of less aggressive BCs and reduced unnecessary recalls.
**Limitations:** Data on interval and advanced cancers are lacking, which would allow for an analysis of long-term clinical outcomes.
**Funding for this study:** No fundings
**Ethics committee - additional information:** Not requested
**Author Disclosures:**
Giorgia Soppelsa: Nothing to disclose
Eleonora Di Gaetano: Nothing to disclose
Elisa Cattarin: Nothing to disclose
Ivana Vinci: Nothing to disclose
Claudia Maria Weiss: Nothing to disclose
Roberta Cerniato: Nothing to disclose

**Artificial intelligence as an initial reader for double reading in breast cancer screening: A prospective initial study of 32,822 mammograms of the Egyptian population**

S. A. Mansour, R. M. Kamal, M. M. Gomaa, E. Sweed, S. Hussien, E. Abdalla, *Y. M. Nada*, G. Mohamed, A. F. I. Moustafa; Cairo/EG
*(dr.yasmin.nada.nl@gmail.com)*

**Purpose or Learning Objective:** Although artificial intelligence (AI) has potential in the field of screening of breast cancer, there are still issues. It is vital to make sure AI doesn't overlook cancer or cause needless recalls. The aim of this work was to investigate the effectiveness of indulging AI in combination with one radiologist in the routine double reading of mammography for breast cancer screening.
**Methods or Background:** The study prospectively analyzed 32822 screening mammograms. Reading was performed in a blind-paired style by i) two radiologists and ii) one radiologist paired with AI. A heatmap and abnormality scoring percentage were provided by AI for abnormalities detected on mammograms. Negative mammograms and benign-looking lesions that were not biopsied were confirmed by a 2-year follow-up.
**Results or Findings:** Double reading by the radiologist and AI detected 1324 cancers (6.4%); on the other side, reading by two radiologists revealed 1293 cancers (6.2%) and presented a relative proportion of 1·02 (p<0·0001). At the recall stage, suspicion and biopsy recommendation were more presented by the AI plus one radiologist combination than by the two radiologists. The interpretation of the mammogram by AI plus only one radiologist showed a sensitivity of 94.03%, a specificity of 99.75%, a positive predictive value of 96.571%, a negative predictive value of 99.567%, and an accuracy of 99.369% (from 99.252% to 99.472%). The positive likelihood ratio was 387.260, negative likelihood ratio was 0.060, and AUC “area under the curve” was 0.969 (0.967 to 0.971).
**Conclusion:** AI could be used as an initial reader for the evaluation of screening mammography in routine workflow. Implementation of AI enhanced the opportunity to reduce false negative cases and supported the decision to recall or biopsy.
**Limitations:** The study is a single institute work.
**Funding for this study:** No source of funding.
**Ethics committee - additional information:** The study has been approved by the Baheya Charity Hospital research center.
**Author Disclosures:**
Sahar Abdelkhalek Mansour: Nothing to disclose
Samar Hussien: Nothing to disclose
Mohammed Mohamed Gomaa: Nothing to disclose
Engy Abdalla: Nothing to disclose
Enas Sweed: Nothing to disclose
Yasmin Mohamed Nada: Nothing to disclose
Ghada Mohamed: Nothing to disclose
Rasha Mohamed Kamal: Nothing to disclose
Amr Farouk Ibrahim Moustafa: Nothing to disclose

**Simulating single reading for high-risk examinations in the randomized controlled Mammography Screening with Artificial Intelligence trial (MASAI)**

*V. Josefsson*, D. Schmidt, H. Sartor, O. Hagberg, K. Lang; Malmö/SE
*(viktoria.josefsson@med.lu.se)*

**Purpose or Learning Objective:** To assess the value of double reading of high-risk examinations in the MASAI trial.
**Methods or Background:** In the randomised controlled MASAI trial AI supported screening was compared to standard double reading. AI was used to triage exams to single or double reading depending on malignancy risk and as detection support. Of the 53 048 participants in the intervention arm, 3800 exams were high risk and underwent double reading while the remaining exams underwent single reading. In this retrospective study, we assessed the relative performance in the intervention arm, comparing simulated single and factual double reading of high-risk exams and its effect on cancer detection, recalls, and false positives. Cancers solely detected by the second reader were described.
**Results or Findings:** The simulated single reading scenario resulted in 8.9% (308 vs. 338) fewer detected cancers and 5.9% fewer recalls (1045 vs. 1110) compared to the factual outcome in the intervention arm. Corresponding simulated vs. factual rates were 5.8/1000 vs. 6.4/1000 for cancer detection, 2.0% vs. 2.1% for recalls and 1.4% vs. 1.5% for false positive. Of the 30 cancers solely detected by the second reader, 24 (80.0%) were invasive and 21 (70.0%) were classified as T1. Of the invasive cancer, 23 (95.8%) were lymph-node negative and 8 (33.3%) non-luminal A, of which four were triple negative.
**Conclusion:** Double reading of high-risk exams improved cancer detection without unduly increasing false positives. The additional cancers detected were mostly small, lymph-node negative invasive cancers, including those of significant prognostic subtypes. These findings support the continued use of double reading for high-risk exams in AI-supported screening.
**Limitations:** Single-institution trial. Retrospective simulation.
**Funding for this study:** The Swedish Cancer Society
Regional Cancer Centers in Collaboration
Lund University ALF-funds
**Ethics committee - additional information:** The Swedish Ethical Review Authority
2020-04936
2023-026848-02
**Author Disclosures:**
Kristina Lang: Nothing to disclose
Viktoria Josefsson: Nothing to disclose
Hanna Sartor: Nothing to disclose
Oskar Hagberg: Nothing to disclose
David Schmidt: Nothing to disclose

**Using prior mammograms to improve specificity of an AI system for breast cancer detection: a large-scale retrospective multi-site validation**

*A. Rodriguez Ruiz*[1], S. Pires[1], R. Peeters[1], G. Rodriguez-Esteban[1], D. Sperber[1], C. De Wolf[2], J. L. Raya Povedano[3], S. Romero Martin[3], R. Mann[1]; [1]Nijmegen/NL, [2]Geneva/CH, [3]Cordoba/ES

**Purpose or Learning Objective:** To investigate how the use of prior mammograms impacts breast cancer detection performance of an AI system.
**Methods or Background:** Mammograms from women attending three European screening programs were collected based on availability of prior images and at least 2 years follow-up, including original radiologists assessments. Each case was analyzed by a breast cancer detection AI product (Transpara, ScreenPoint Medical, v2.1), resulting in two cancer risk scores: using as input the current mammogram alone and using prior mammograms. AI specificity was compared between using priors or not, matching the single radiologist sensitivity . Subsequently, the combination of a single radiologist and AI was modelled and compared to double human reading. P-values using McNemar and binomial confidence intervals were computed.
**Results or Findings:** 37,148 cases were included (20,300 from Switzerland, 916 from Spain 15,932 from The Netherlands), with 1,034 recalled cases (2.8%), 247 screen-detected cancers (6.6/1000), and 59 interval cancers (1.6/1000). 56% of cases had 1 prior mammogram, 44% had 2 or 3. Images were acquired with Hologic, Siemens, GE, Planmed and Philips machines. At the average sensitivity of a single radiologist (71.2%), AI specificity increased when using prior images, from 98.1% (98.0-98.2%) to 98.8% (98.7-98.9%), representing a 37% reduction in false positives (from 1.9% to 1.2%, P<0.001). Combining AI using priors with a radiologist achieved comparable sensitivity (83.0% vs 82.0%, P=0.66) and higher specificity (96.3%, 96.0-96.5%) than double human reading before consensus (95.4%, 95.2-95.6%), representing 20% fewer false positives (P<0.001). The improved specificity was higher for cases with breast density C/D (+0.8%, P<0.001) than for A/B (+0.3%, P=0.005).
**Conclusion:** A higher specificity was achieved by a breast cancer detection AI system using prior mammograms, potentially offering better aid to radiologists in breast cancer screening.
**Limitations:** Retrospective design.
**Funding for this study:** None
**Ethics committee - additional information:** Not applicable
**Author Disclosures:**
Santiago Pires: Employee: ScreenPoint Medical
Daan Sperber: Employee: ScreenPoint Medical
Alejandro Rodriguez Ruiz: Employee: ScreenPoint Medical
Ruud Peeters: Employee: ScreenPoint Medical
Sara Romero Martin: Nothing to disclose
Jose Luis Raya Povedano: Nothing to disclose
Gonzalo Rodriguez-Esteban: Employee: ScreenPoint Medical
Chris De Wolf: Nothing to disclose
Ritse Mann: Nothing to disclose

**12:30-13:30 Research Stage 2**

### Research Presentation Session: Vascular

### RPS 1915

### Carotid and intracranial artery imaging

**Moderator**
V. Silvestri; Seclin/FR
*(valentina.silvestri@ghsc.fr)*

**Sub-1-minute Relaxation-Enhanced Angiography without Contrast and Triggering of the Extracranial Arteries**

*J. P. Janssen*[1], K. Kaya[1], R. A. Terzis[1], J. Tristram[1], R. J. Gertz[1], L. Goertz[1], L. Pennig[1], C. H. Gietzen[1], K. Weiss[2]; [1]Cologne/DE, [2]Hamburg/DE
*(jan.janssen@uk-koeln.de)*

**Purpose or Learning Objective:** To evaluate the acceleration of a 3D isotropic flow-independent non-contrast MRA (REACT) of the neck using Compressed SENSE (CS) combined with deep learning-based reconstruction (CS-AI).
**Methods or Background:** Thirty-four volunteers received cervical REACT at 3T ((acquired threefold: (1) CS factor 7 (scan time: 1:20 min), (2) CS factor 10 (0:55 min), and (3) CS-AI factor 10 (0:55 min)). Two radiologists rated the image quality of seven arterial segments and overall image noise. Additionally, a pairwise forced-choice comparison was conducted. Apparent signal- (aSNR) and contrast-to-noise ratios (aCNR) were measured, and image sharpness was assessed by calculating the edge rise distance (ERD). Multiple t-tests and non-parametric tests with Bonferroni correction were performed for comparison to CS7, which was considered as the current clinical standard.
**Results or Findings:** Compared to CS7, CS10 showed lower image quality scores (p<0.001) while CS10-AI obtained higher results (p=0.010). Image noise was similar between CS7 and CS10 (p=0.138) while CS10-AI yielded a lower noise (p=0.008). Forced choice revealed preferences for CS7 over CS10 (p<0.001), but no preference between CS7 and CS10-AI (p>0.999). Compared to CS7, aSNR and aCNR were lower in CS10 (p<0.001) and the ERD was longer (p=0.004), while CS10-AI provided better aSNR and aCNR (p=0.001) and showed no difference in ERD (p=0.776).
**Conclusion:** CS-AI enables the acquisition of cervical REACT in less than one minute without compromising image quality. Further studies are required to confirm these results in patients and to evaluate the diagnostic performance regarding vascular findings such as stenosis or dissection.
**Limitations:** No pathologies were assessed. No comparison was made with established reference standards.
**Funding for this study:** Not applicable.
**Ethics committee - additional information:** Approved by our institutional review board (reference number: 20-1296_1) and registered in the national registry for clinical trials (DRKS00030210).
**Author Disclosures:**
Lenhard Pennig: Speaker: Guerbet GmbH Speaker: Philips Healthcare
Carsten H. Gietzen: Nothing to disclose
Juliana Tristram: Nothing to disclose
Roman Johannes Gertz: Speaker: Guerbet GmbH Speaker: Philips Healthcare
Kenan Kaya: Nothing to disclose
Kilian Weiss: Employee: Philips GmbH
Robert Angelo Terzis: Nothing to disclose
Jan Paul Janssen: Nothing to disclose
Lukas Goertz: Nothing to disclose

**Carotid artery assessment in dual-source photon-counting CT: impact of low-energy virtual monoenergetic imaging on image quality, vascular contrast and diagnostic assessability**

*A-I. Nica*[1], C. Booz[1], G. M. Bucolo[1], L. S. Alizadeh[1], T. Vogl[1], T. D'Angelo[2], H-L. Kaatsch[3], D. Overhoff[3], S. Waldeck[3]; [1]Frankfurt/DE, [2]Messina/IT, [3]Koblenz/DE

**Purpose or Learning Objective:** The purpose of this study is to evaluate the impact of low-energy VMI reconstructions on quantitative and qualitative image quality, vascular contrast, and diagnostic assessability of the carotid arteries in photon-counting CTA.
**Methods or Background:** A total of 122 patients (67 male) who had undergone dual-source photon-counting CTA scans of the carotid artery were retrospectively analyzed in this study. Standard 120 kV CT images and low-keV VMI series from 40 to 100 keV with an interval of 15 keV were reconstructed. Quantitative analyses included the evaluation of vascular CT numbers, signal-to-noise ratio (SNR), and contrast-to-noise ratio (CNR). CT number measurements were performed in the common, external, and internal carotid arteries. Qualitative analyses were performed by three board-certified radiologists independently using five-point scales to evaluate image quality, vascular contrast, and diagnostic assessability of the carotid arteries.
**Results or Findings:** Mean attenuation, CNR and SNR values were highest in 40 keV VMI reconstructions (HU, $1362.32 \pm 457.81$; CNR, $33.19 \pm 12.86$; SNR, $34.37 \pm 12.89$) followed by 55-keV VMI reconstructions; all three mean values at these keV levels were significantly higher compared with standard 120 kV CT series (HU, $154.43 \pm 23.69$; CNR, $16.34 \pm 5.47$; SNR, $24.44 \pm 7.14$) ($p < 0.0001$). The qualitative analysis showed highest rating scores for 55 keV VMI reconstructions followed by 40 keV and 70 keV VMI series with a significant difference compared to standard 120 kV CT images regarding image quality, vascular contrast, and diagnostic assessability of the carotid arteries (all comparisons, $p < 0.01$).
**Conclusion:** Low-keV VMI reconstructions at a level of 40–55 keV significantly improve image quality, vascular contrast, and the diagnostic assessability of the carotid arteries compared with standard CT series in photon-counting CTA.
**Limitations:** Single-center retrospective study
**Funding for this study:** No funding was received
**Ethics committee - additional information:** The local IRB approved this study.
**Author Disclosures:**
Christian Booz: Speaker: Siemens Healthineers
Thomas Vogl: Nothing to disclose
Daniel Overhoff: Nothing to disclose
Andreea-Ioana Nica: Nothing to disclose
Hanns-Leonhard Kaatsch: Nothing to disclose
Stephan Waldeck: Nothing to disclose
Tommaso D'Angelo: Speaker: Philips Speaker: Bracco
Leona Soraja Alizadeh: Nothing to disclose
Giuseppe Mauro Bucolo: Nothing to disclose

**A Novel Approach in Vascular Imaging: AI-Driven 3D Reconstruction of Carotid Arteries for Enhanced Stroke Risk Assessment**

K. Gasbarrino[1], *A. Benjamin*[1], T. Beiko[1], J. Ramirez-Garcia Luna[1], R. Khan[1], L. H. Gonzalez Torres[1], S. Levasseur[1], S. Taj[2], K. Khan[1]; [1]Montreal/CA, [2]Columbia, MD/US

**Purpose or Learning Objective:** The standard approach to assessing stroke risk via 2D carotid ultrasound is limited by operator variability, a lack of 3D vessel visualization, and subjective interpretation, resulting in a nearly 30% misclassification rate. To address these challenges, we developed AI-powered software that transforms 2D ultrasound images into precise 3D models of carotid arteries and automates vessel measurements.
**Methods or Background:** We applied a multi-class U-Net AI model, trained on ~4000 2D ultrasound images from 113 North American patients with cardiovascular risk factors. Two independent sonographers annotated these images, identifying key vascular structures, including medial-adventitial boundary, intimal-luminal boundary, and plaque. 3D reconstructions were achieved by integrating 2D image segmentations with positional data captured from an electromagnetic sensor (Northern Digital Inc, Canada) during a single B-mode sweep of the carotid artery. Algorithms were developed for automated measurement of vessel diameter, artery stenosis, and classification of disease severity. Validation was conducted using a carotid artery phantom with a predefined 70% stenosis (R.G. Shelley Ltd, Canada), along with clinical evaluation in 8 patients to compare performance against the current standard of care.
**Results or Findings:** The AI model demonstrated strong performance, achieving a DICE coefficient of 0.86 in detecting vessel structures. The software successfully generated 3D models, with vascular metrics showing a 99% agreement with the known stenosis in the phantom model. Intra-operator variability was minimal, with stenosis measurements showing only minor deviations (71.42±3.42%). In the clinical study, a 90% reduction in ultrasound scan was achieved, while maintaining diagnostic accuracy equivalent to that of a vascular radiologist with >10 years of experience.
**Conclusion:** Our software represents a significant advancement in carotid artery imaging, delivering a ten-fold improvement in scan efficiency while achieving expert-level diagnostic accuracy with minimal variability.
**Limitations:** N/A
**Funding for this study:** Ontario Brain Institute; Québec's Ministère de l'Économie, de l'Innovation et de l'Énergie
**Ethics committee - additional information:** The study was approved by Advarra IRB (Pro00068778)
**Author Disclosures:**
Luis H. Gonzalez Torres: Nothing to disclose
Rafia Khan: Consultant: Sonaro Inc
Jose Ramirez-Garcia Luna: Shareholder: Sonaro Inc
Sophie Levasseur: Nothing to disclose
Karina Gasbarrino: Founder: Sonaro Inc
Thierry Beiko: Employee: Sonaro Inc.
Kashif Khan: Founder: Sonaro Inc
Sabir Taj: Advisory Board: Sonaro Inc
Alex Benjamin: Research/Grant Support: Sonaro

**Association Between Pericarotid Fat Density and Hemorrhagic Transformation After Endovascular Therapy for Acute Extracranial Internal Carotid Artery Occlusion**

*Z. Cui*, J. Zhang; Shanghai/CN
*(510349687@qq.com)*

**Purpose or Learning Objective:** This study aimed to investigate the association between pericarotid fat density (PFD) around the occlusion and hemorrhagic transformation (HT) risk and functional outcome in acute ischemic stroke (AIS) patients with extracranial internal carotid artery (e-ICA) occlusion who underwent endovascular thrombectomy (EVT).
**Methods or Background:** This multicenter retrospective study included a cohort of patients with e-ICA occlusion after EVT between June 2019 and March 2024. PFD was assessed using semi-automated quantitative software at pre-operation neck CT angiography (CTA). The associations between PFD and HT, and functional outcome (mRS score 0-3 vs 4-6) were analyzed using multivariable logistic regression. A mediation analysis was conducted to explore whether HT mediates the relationship between PFD and functional outcome. Additionally, We sought the association between PFD and admission C-reactive protein (CRP) levels.
**Results or Findings:** 101 patients were included and divided into an HT group (n=36) and a non-HT group (n=65). PFD was independently associated with HT (adjusted odds ratio [aOR]: 1.84, 95% CI: 1.28 to 2.66, P<0.001) and unfavorable functional outcome (1.41, 1.04 to 1.91, p=0.030). The AUC values were 0.79 (95% CI, 0.70 to 0.89) and 0.68 (0.57 to 0.78), indicating a favorable predictive performance for the HT risk and unfavorable prognosis prediction. Mediation analysis revealed that HT explained more than 60% of the relationship between PFD and worse functional outcome. In addition, higher PFD was positively correlated with elevated CRP levels.
**Conclusion:** PFD is an independent predictor of HT and a worse functional outcome at 90 days in patients with AIS and e-ICA occlusion who underwent EVT. Evaluation of PFD provides opportunities for HT risk stratification and outcome prediction.
**Limitations:** Given the sample size, we were unable to apply the more stringent inclusion criteria of restricted to the extracranial ICA.
**Funding for this study:** No funding was received for this study.
**Ethics committee - additional information:** Informed consent was waived for all participants due to the retrospective nature of the study.
**Author Disclosures:**
Jun Zhang: Nothing to disclose
Zhimeng Cui: Nothing to disclose

**The influence of visual signals on blood flow in the central retinal and internal carotid arteries**

M. Beraia, D. Gachechiladze, *A. Siradze*, N. Eliava, M. Lazarashvili, N. Nikabadze, S. Siradze, J. Giorgelashvili, L. Udumashvili; Tbilisi/GE
*(anetasiradze@yahoo.com)*

**Purpose or Learning Objective:** The retina offers a unique window into brain structure and functional disorders due to its anatomical, physiological, and embryological similarities with the brain. This research explores the influence of visual-verbal/nonverbal stimuli on blood flow in both the retina and brain.
**Methods or Background:** A duplex Ultrasound study was conducted with 25 volunteers (11 males, 14 females, aged 21–35), examining blood flow in the central retinal artery (CRA) and internal carotid artery (ICA) under two types of visual stimuli: verbal irritation (Shakespeare's sonnets) and nonverbal (pictures – find the hidden figures). Blood flow parameters (Vsys, Vdia, PI, and RI) were measured in intervals 1-15 and 25-40 seconds after stimuli initiation, to assess the nature (neuro/humoral) of blood flow regulation (RBC circulation time: 20 seconds). Initial 5sec for the baseline images. CE-MRI angiography (TOF) was used to exclude vascular anomalies.
**Results or Findings:** In the CRA, Vsys increased from 7.5–11.5 cm/sec to 10.7–14.3 cm/sec, with Vdia at 4.1–4.9 cm/sec. RI rose from 0.53 to 0.64, and PI from 0.71 to 0.94. In the ICA, Vsys rose from 80–130 cm/sec to 95–170 cm/sec, with Vdia at 24–45 cm/sec. RI increased from 0.67 to 0.74, and PI from 1.01 to 1.17. Changes in the CRA and ICA were unidirectional (r = 0.7). In nonverbal cases, Vsys and RI were higher in the CRA (P < 0.05) and ICA (P < 0.01). The time of the stimuli initiation did not change the results. These details indicate the mostly sympathetic regulation of cerebral blood flow.
**Conclusion:** Quantitative blood circulation studies in these arteries may be used as the functional ultrasound diagnostics of the retina and brain.
**Limitations:** Not applicable.
**Funding for this study:** No funding was provided for this study.
**Ethics committee - additional information:** The ethics committee notification can be found under the number UID 2438.
**Author Disclosures:**
Nino Eliava: Nothing to disclose
Luarsabi Udumashvili: Nothing to disclose
Aneta Siradze: Nothing to disclose
Dudana Gachechiladze: Nothing to disclose
Mariam Lazarashvili: Nothing to disclose
Salome Siradze: Nothing to disclose
Merab Beraia: Nothing to disclose
Jano Giorgelashvili: Nothing to disclose
Nini Nikabadze: Nothing to disclose

**Intracranial arterial calcification detection; a comparison between ultra-high-resolution photon-counting CT, conventional energy-integrating CT and micro-CT**

*J. Van Der Bie*[1], B. P. Berghout[1], R. P. J. Budde[1], J. Gutierrez[2], M. Van Straten[1], D. Bos[1]; [1]Rotterdam/NL, [2]New york, NY/US
*(j.vanderbie@erasmusmc.nl)*

**Purpose or Learning Objective:** To assess the performance of photon-counting detector CT (PCD-CT) in detecting and quantifying intracranial arterial calcifications and comparing the performance to conventional energy-integrating CT (EID-CT), using micro-CT (µCT) as the reference standard.
**Methods or Background:** Thirty histopathological cross-sections of intracranial arteries were scanned with PCD-CT, EID-CT, and µCT. µCT was optimized for image quality (reference standard), while clinical protocols were used for PCD-CT and EID-CT. Various reconstruction kernels (EID-CT: Hv40/Hv49/Hv59; PCD-CT: Hv40/Hv48/Hv56/Hv64/Hv72/Hv89) were used to enhance spatial resolution. Two experienced observers independently evaluated the presence of calcifications in all acquisitions and were compared by Cohen's Kappa and concordance percentages. For objective analysis, mass scores were used to assess both the detection and mass. The objective measure was analyzed using Bland-Altman plots.

Saturday

**Results or Findings:** Observer 1 detected calcifications in 24 samples and Observer 2 in 23 samples using µCT (90% concordance, κ=0.706). EID-CT with Hv59 showed the highest interobserver agreement (97% concordance, κ=0.911), but low detection rates (observer 1: 27%, observer 2: 25%) compared to µCT. PCD-CT yielded better detection with Hv48 (observer 1: 70% detection rate, 90% concordance, κ=0.706) and Hv56 (observer 2: 80% detection rate, 77% concordance, κ=0.314). Mass scores indicated the highest detection with PCD-CT Hv64, though with increased noise. The Hv48/Hv56 kernels were deemed optimal, yielding sensitivity, and specificity (for observers 1/2) of 83%/92%, 50%/83%, respectively, and accuracy of 77%/90%.
**Conclusion:** PCD-CT outperformed EID-CT in detecting intracranial calcifications. Nevertheless, small calcifications sometimes go undetected compared to µCT. Hv48 and Hv56 kernels are recommended for optimal results, balancing detection rates and noise.
**Limitations:** In the observer study, detection was assessed on a sample basis, leading to potential oversight of smaller calcifications in segments where larger ones were present, which is reflected in the mass scores.
**Funding for this study:** This study has received funding by Smart*Light is partially funded by the Interreg V Flanders-Netherlands program with financial support from the European Regional Development Fund (ERDF).
**Ethics committee - additional information:** All brain sources were approved by the IRB at their respective institutions. The Institutional Review Board waived written informed consent:
**Author Disclosures:**
Ricardo P. J. Budde: Speaker: Siemens Healthineers Advisory Board: Bayer
Marcel Van Straten: Nothing to disclose
Daniel Bos: Nothing to disclose
Bernhard P. Berghout: Nothing to disclose
Jose Gutierrez: Nothing to disclose
Judith Van Der Bie: Nothing to disclose

### Cerebral blood flow alterations and host genetic association in individuals with long COVID: A transcriptomic-neuroimaging study

*Y. Wang*[1], F. Zhou[2]; [1]Nanchang/CN, [2]NanChang City, Jiangxi Province/CN
*(1694865434@qq.com)*

**Purpose or Learning Objective:** Neuroimaging studies have indicated that altered cerebral blood flow (CBF) was associated with the long-term symptoms of long COVID. long COVID were found to be strongly associated with host gene expression. Nevertheless, the relationships between altered CBF, clinical symptoms, and gene expression in the central nervous system (CNS) remain unclear in individuals with long COVID
**Methods or Background:** First, CBF pattern was computed from arterial spin labeling sequence in long COVID. Next, using CNS gene expression data from the AHBA transcriptomic dataset, we conducted the spatial correlation between CBF and gene expression to defined the CBF-related genes. Functional enrichment analyses were applied to understand the biological functions of CBF-related genes. The cell type-specific expression analyses is utilized to identify the CNS cell types most closely associated with long COVID-19
**Results or Findings:** Lower CBF in left frontal-temporal gyrus was associated with higher fatigue and worse cognition in long COVID. This CBF pattern was spatially associated with the expression of 2,178 genes, which significantly overlap with the genes reported to interact with SARS CoV-2 proteins (odds ratio= 1.60, P= 0.0036). Functional enrichment analyses indicated these 2,178 genes were enriched in the molecular functions and biological pathways of COVID-19. Additionally, these genes were strongly associated with the oligodendrocyte progenitor cells, astrocytes, and myelinating oligodendrocytes of the cortex. ALL above results were corrected for multiple comparisons
**Conclusion:** Lower CBF is associated with persistent clinical symptoms in long COVID individuals, possibly as a consequence of the complex interactions among multiple COVID-19-related genes, which contributes to our understanding of the impact of adverse CNS outcomes and the trajectory of development to long COVID.
**Limitations:** The heterogeneity of clinical symptoms at the time of scanning may affect gene expression in individuals with long COVID.
**Funding for this study:** The author(s) disclosed receipt of the following financial sup port for the research, authorship, and/or publication of this article: This work was supported by the COVID-19 Research Project of the leading medical discipline in Jiangxi Province, Jiangxi Province Double Thousand Talent Plan (jxsq2023201039), Clinical Research Center for Medical Imaging In Jiangxi Province (20223BCG74001) and Jiangxi Province Key Laboratory for Precision Pathology and Intelligent Diagnosis (2024SSY06281).
**Ethics committee - additional information:** The present study was approved by the Ethics Committee of the First Affiliated Hospital of Nanchang University (IIT2023018)
**Author Disclosures:**
Yao Wang: Nothing to disclose
Fuqing Zhou: Nothing to disclose

**12:30-13:30 Research Stage 3**

### Research Presentation Session: Paediatric

### RPS 1912

### Imaging the growing brain

**Moderator**
C. Carducci; Rome/IT
*(chi.carducci@gmail.com)*

### Children's brain development is linked to the trajectory of epigenetic-based inflammatory scores

J. Chuah, A. M. A. Manahan, S. Y. Chan, H. Pei, M. Fortier, M. Meaney, *A. P. Tan*; Singapore/SG
*(dnrtanap@nus.edu.sg)*

**Purpose or Learning Objective:** Dysregulation of immune activation has been consistently shown in patients with mental health disorders. In this study, we mapped the trajectories of DNA-methylation based inflammation scores and examined how these trajectories relate to exposure to maternal depression, subsequent cognitive outcomes, and brain development at multiple levels.
**Methods or Background:** Inflammation scores were calculated for 293 children from DNA methylation data based on epigenome-wide association studies of serum C-reactive proteins at ages 9 and 48 months. We stratify these children into quartiles based on their inflammation scores at baseline and map the trajectories of inflammation scores for each quartile. Next, we examined if children with different inflammation score trajectories have different exposure to maternal depression, executive function performance, and brain changes evaluated using multimodal MRI at ages 4.5, 6.0, and 7.0 years.
**Results or Findings:** We observed a decreasing trend of our DNA-methylation based inflammation scores, primarily driven by children with higher levels of inflammation scores at baseline. Children with lower levels of inflammation scores at 9M and a slower decrease in inflammation scores between 9M and 48M were exposed to higher levels of maternal depressive symptoms and showed poorer executive function performance at ages 4.5 and 7. Children with different inflammation score trajectories exhibit significantly different brain structure and function, involving predominantly brain regions involved in executive function performance, emotion, and reward processing.
**Conclusion:** Children with lower baseline inflammation scores and slower rate of decrease across childhood are exposed to higher levels of maternal depression, possibly related to a blunted immune response from chronic stress exposure. This has a significant downstream impact on cognitive and brain development at multiple levels.
**Limitations:** Evaluation of executive function performance with questionnaires
**Funding for this study:** This research was supported by grants NMRC/TCR/004-NUS/2008 and NMRC/TCR/012-NUHS/2014 from the Singapore National Research Foundation (NRF) under the Translational and Clinical Research Flagship and grant OFLCG/MOH-000504 from the Open Fund Large Collaborative Grant Programmes and administered by the Singapore Ministry of Health's National Medical Research Council (NMRC), Singapore. In RIE2025, GUSTO is supported by funding from the NRF's Human Health and Potential (HHP) Domain, under the Human Potential Programme. Additional funding was provided by the Singapore Institute for Clinical Sciences, Agency for Science Technology and Research (A*STAR), Singapore. MJM is supported by funding from the Hope for Depression Research Foundation, USA, the Toxic Stress Network of the JPB Foundation, USA, the Jacobs Foundation, Switzerland, and the NRF and A*STAR's Human Potential Programme (H22P0M0001), Singapore. SYC is supported by funding from the NMRC Open Fund – Young Individual Research Grant (MOH-001149-00). EHT is supported by the NMRC Clinician-Scientist Award (CSA) (MOH-001415). APT is supported by funding from the NMRC Transition Award (MOH-001273-00) and A*STAR (Brain-Body Initiative, iGrants call ID #21718).
**Ethics committee - additional information:** The study was approved by the National Healthcare Group Domain Specific Review Board (D/2009/021 and B/2014/00411) and the SingHealth Centralized Institutional Review Board (D/2018/2767 and A/2019/2406). All investigations were conducted according to the principles expressed in the Declaration of Helsinki. Written consent was obtained from all guardians on behalf of the enrolled children.
**Author Disclosures:**
Ai Peng Tan: Nothing to disclose
Marielle Fortier: Nothing to disclose
Michael Meaney: Nothing to disclose
Shi Yu Chan: Nothing to disclose
Jasmine Chuah: Nothing to disclose
Huang Pei: Nothing to disclose
Aisleen M. A. Manahan: Nothing to disclose

**Geometric microstructural changes of white matter in infants with periventricular white matter injury and spastic cerebral palsy**

*M. Wang*[1], H. Zhu[2], T. Huang[3], J. Cheng[2], H. Jiang[1]; [1]Wuxi/CN, [2]Beijing/CN, [3]Zhengzhou/CN
*(wyflipped@gmail.com)*

**Purpose or Learning Objective:** To investigate the geometric microstructural changes in white matter in infants with periventricular white matter injury and spastic cerebral palsy (PWMI-SCP) and facilitate early prediction.
**Methods or Background:** PWMI is a high-risk factor for SCP. However, early identification of PWMI-SCP infants remains challenging. A novel mathematical framework, “Director Field Analysis” (DFA), reflects changes in the microstructural geometry of white matter and offers new insights into the pathological mechanisms of PWMI-SCP. DFA provides specific quantitative metrics, including splay, twist, bend, and total distortion index. Analysis of variance, correlation analysis, and receiver operating characteristics analysis were performed. Corrected p-values < 0.05 were considered significant.
**Results or Findings:** The PWMI-SCP group exhibited significantly elevated DFA metrics, primarily in the corpus callosum, posterior thalamic radiata, and corona radiata, comparing to the PWMI without SCP group, which were associated with enlarged lateral ventricles, reduced deep nuclear volumes and motor dysfunction. Mediation analysis indicated that increased geometric microstructure in the corpus callosum partially mediates the relationship between the lateral ventricles and motor function. A multi-parameter model based on DFA metrics can effectively predict PWMI-SCP with an AUC of 0.95.
**Conclusion:** Abnormal increases in white matter geometric microstructure in the sensorimotor circuit may be one of the neural substrates underlying the manifestation of SCP in PWMI infants. Monitoring fiber-orientational alterations may provide new insights into early prediction of PWMI-SCP.
**Limitations:** First, this study focused on infants aged 6−36 m□□th□; th□r□f□r□, future research should investigate changes in white matter geometric microstructure in infants aged 0-6 months to improve the early diagnosis rate of PWMI-SCP. Additionally, biomolecular research is required to understand the interactions between brain morphology, tissue mechanics, and white matter geometric microstructure to elucidate the pathophysiological mechanisms of PWMI-SCP.
**Funding for this study:** This study was financially supported by grants from the STI 2030 - Major Project (Grant No. 2022ZD0209000); the Hong Kong global STEM scholar scheme; the internal fund of the Hong Kong Polytechnic University; the Wuxi Municipal Health Commission's 'Double Hundred' Medical Health Young Elite Talent Project (Grant No. BJ2023088) and the Wuxi Municipal Science and Technology Bureau's Medical and Health Tackling Project (Grant No. Y20232012); the Jiangsu Province Graduate Research Innovation Project (KYCX24_2647); National Natural Science Foundation of China (Grant No. 61971017, No. 82204933); Open Research Fund of the State Key Laboratory of Cognitive Neuroscience and Learning (Grant No. CNLZD2101).
**Ethics committee - additional information:** The study received institutional review board approval, and written informed consent was obtained from all participants.
**Author Disclosures:**
Jian Cheng: Nothing to disclose
Hua Zhu: Nothing to disclose
Tingting Huang: Nothing to disclose
Miaoyan Wang: Nothing to disclose
Haoxiang Jiang: Nothing to disclose

**Altered coupling of cerebral blood perfusion and neuronal activity in children with MRI-negative drug refractory Epilepsy**

*H. Ran*, T. Zhang; Zunyi/CN
*(haifengran_zmu@163.com)*

**Purpose or Learning Objective:** Drug refractory left temporal lobe epilepsy (DRLTLE) often give rise to neuronal activity and cerebral vascular hemodynamics changes, which may result in neurovascular decoupling. However, neuroimaging evidence on neurovascular decoupling remains scarce, this study aimed to assess the manifestation of neurovascular coupling (NVC) in childhood DRLTLE using resting-state fMRI (rs-fMRI) and arterial spin labeling imaging (ASL).
**Methods or Background:** Based on the collected rs-fMRI and ASL imaging data, degree centrality (DC) and cerebral blood flow (CBF) were calculated respectively. Across voxel CBF-DC correlations used to evaluate the NVC within whole brain, and NVC of brain region was assessed by the CBF/DC ratio. We performed correlation analysis to evaluate the relationship between the variables. Finally, we explored classification problems between DRLTLE and healthy control (HC).
**Results or Findings:** Compared HC group, the DRLTLE children with higher across voxel CBF-DC correlations. The brain regions of abnormal CBF, DC, and CBF/DC ratio in predominantly in the default mode and the executive control network, the abnormally CBF, DC values in some brain regions were significantly correlated cognitive function. The classification model using CBF/DC ratio as features achieved the 72.8% accuracy, 0.764 area under the curve, 68.5% sensitivity, 87.5% specificity, the classification accuracy were higher than the model using CBF or DC feature.
**Conclusion:** The study reveals the cerebral blood perfusion, neuronal activity, global and regional NVC alteration in children with MRI-negative DRLTLE non-invasively, associated with lower cognitive performance. These findings indicating that NVC-based study can better integrate information of neuronal activity and cerebral hemodynamics, offering a new insight into the neuropathological mechanisms of DRLTLE, and NVC may help clinical classification for childhood DRLTLE.
**Limitations:** The sample size was relatively small and the potential impact of antiepileptic drugs could not totally eliminated.
**Funding for this study:** This study was supported by National Natural Science Foundation of China (Grant Nos .82171919) and Intelligent Medical Imaging Engineering Research Center of Guizhou Higher Education Institutions project (Grant No. Qianjiaoji [2023] 038)
**Ethics committee - additional information:** The ethic committee of Zunyi Medical University reviewed and granted ethical approval of this research (Ethical Batch Number: lunshen [2021] 1-080)
**Author Disclosures:**
Tijiang Zhang: Nothing to disclose
Haifeng Ran: Nothing to disclose

**Magnetic resonance imaging of children with the use of an incubator**

P. D. Mika, J. Cydejko, *B. Rowinski*, D. Świętoń, E. Szurowska; Gdańsk/PL
*(browinski@uck.gda.pl)*

**Purpose or Learning Objective:** Important limitation of pediatric MRI imaging is requirement of general anesthesia (GA) in the youngest group of patients. Imaging in GA is not only stressful for children but also extends the time of the procedure. The solution is to use an incubator dedicated to work in a magnetic field. The MRI dedicated incubator allows a full spectrum of MRI imaging. Analysis of examinations with the use of an MRI incubator performed at the Department of Radiology of the University Clinical Center in Gdansk.
**Methods or Background:** A retrospective evaluation of MRI examinations performed using an incubator carried out in the youngest group of patients. The study includes an analysis of the tests performed in terms of the child's age and the examined area. The paper presents the benefits of using an MRI incubator. Sequences prepared specifically for test protocols in which the incubator is used are discussed Patients were tested in the "feed and sleep" protocol.
**Results or Findings:** Between 2020 and 2023, a total of 146 MRI examinations were performed. Most of the studies were brain scans without the administration of a contrast agent. Only in 1 case supplementary general sedation was necessary.
**Conclusion:** 1. The study showed a sharp increase in the number of studies using an incubator. 2. The incubator dedicated for MRI examinations allows to limit the amount of anesthesia in the youngest patients to 4 kg of body weight. 3. Increase in the cost of effectiveness of MRI examinations in the youngest group of patients due to the lack of anesthesia costs.
**Limitations:** Movement artifacts
Weight over 4kg
Implants not allowed for MRI
**Funding for this study:** No
**Ethics committee - additional information:** No
**Author Disclosures:**
Paulina Danuta Mika: Nothing to disclose
Edyta Szurowska: Nothing to disclose
Bartosz Rowinski: Nothing to disclose
Dominik Świętoń: Nothing to disclose
Joanna Cydejko: Nothing to disclose

**Brain Perfusion Imaging by Arterial Spin Labelling Predicts Postsurgical Seizure Freedom in Pediatric Focal Lesional Epilepsy**

*A. G. Gennari*, L. Gaito, D. Cserpan, R. Kottke, R. Tuura O'Gorman, G. Ramantani; Zurich/CH
*(gennari_antonio@libero.it)*

**Purpose or Learning Objective:** In children with pharmacoresistant focal lesional epilepsy, lesion-associated brain perfusion changes captured by arterial spin labelling (ASL) are an emerging imaging tool improving lesion detection. However, their correlation with postsurgical seizure outcomes is still unexplored. This study aims to determine whether including ASL-derived perfusion changes in surgical planning is associated with favorable postsurgical seizure outcomes in children with focal cortical dysplasia (FCD) or low-grade epilepsy-associated tumors (LEAT).
**Methods or Background:** We retrospectively analyzed MRI scans from 18 children (median age at MRI: 4.8 years, IQR: 1.9–11.5) who underwent surgical resection for pharmacoresistant epilepsy and had at least 1 year of post-surgical follow-up. All patients received presurgical ASL imaging along with pre- and postsurgical structural MRI. Image postprocessing, including segmentation and coregistration, was used to qualitatively and quantitatively evaluate the completeness of resection of both the anatomical lesion and the ASL-detected perfusion changes. The DICE similarity index was adopted in quantitative analysis to grade the segmentations' alignment. These findings were then correlated with seizure outcomes.
**Results or Findings:** Fourteen (78%) patients achieved complete seizure freedom. Qualitative analysis showed that complete resection of the ASL-detected perfusion changes significantly correlated with seizure freedom (p=0.009). Quantitative analysis indicated that higher degrees of alignment between perfusion and resection cavity segmentations, as measured by DICE score, were associated with seizure freedom (p=0.043), while lesion volume inclusion was not (p=0.44).
**Conclusion:** Including ASL perfusion imaging in the presurgical evaluation can help better define the epileptogenic zone, improving postsurgical seizure outcomes, and supporting it as a complementary tool in surgical planning for pharmacoresistant pediatric focal lesional epilepsy.
**Limitations:** Limitations: - small sample size; - focus on MRI-visible lesions only, limiting the generalizability to MRI-negative patients; - manual segmentation, which may limit the reproducibility of our results.
**Funding for this study:** We thank the National Science Foundation (SNSF: 208184) (to G.R.), the Anna Mueller Grocholski Foundation, and the Theodor und Ida Herzog-Egli-Stiftung (to A.G.G.) for funding. The funders had no role in the design or analysis of the study.
**Ethics committee - additional information:** The collection and analysis of patient data were approved by and performed according to the guidelines and regulations of the local ethics committee (KEK-ZH PB-2024-00298). All parents gave written, informed general consent to reuse clinical data for research.
**Author Disclosures:**
Ruth Tuura O'Gorman: Nothing to disclose
Antonio Giulio Gennari: Research/Grant Support: The Anna Mueller Grocholski Foundation and the Theodor und Ida Herzog-Egli-Stiftung funded Dr. Gennari post-doc
Dorottya Cserpan: Nothing to disclose
Georgia Ramantani: Research/Grant Support: Prof Ramantani received a grant from the National Science Foundation (SNSF: 208184)
Raimund Kottke: Nothing to disclose
Luca Gaito: Nothing to disclose

**Refining Diagnostic Accuracy in Pediatric Metabolic Brain Disorders: Integrating MRI, Proton Spectroscopy, and Diffusion-Weighted Imaging**
*R. Agarwal*[1], U. Gupta[2], N. Jha[2]; [1]Bengaluru/IN, [2]Ghaziabad/IN
*(aritika1994@gmail.com)*

**Purpose or Learning Objective:** This study aims to assess MRI signal abnormalities in pediatric metabolic brain disorders, focusing on diffusion-weighted imaging (DWI) and proton magnetic resonance spectroscopy (MRS). The goals are to identify specific imaging patterns, correlate them with clinical, biochemical, and genetic data, and enhance diagnostic accuracy for early intervention.
**Methods or Background:** Metabolic brain disorders in children are inherited conditions leading to progressive neurodegeneration, where early diagnosis is critical. This study evaluated 30 pediatric patients (aged 0-12 years) with suspected metabolic brain disorders using MRI, DWI, and proton MRS, along with biochemical and genetic testing. MRI findings were categorized based on the involvement of white matter, grey matter, or both.
**Results or Findings:** Of the 30 patients, 24 (80%) were diagnosed with metabolic brain disorders, predominantly in males (79.17%) and in the 0-3 years age group (62.5%). Common symptoms included regression of developmental milestones (70.83%) and seizures (58.33%). MRI showed white matter involvement in 10 cases, grey matter in 5, and both in 9. Diagnoses included X-linked adrenoleukodystrophy, metachromatic leukodystrophy, Leigh disease, Wilson disease, glutaric aciduria type I, and neuronal ceroid lipofuscinosis. MRI findings included symmetrical T2 hyperintensities in 66.67%, diffusion restriction in 41.67%, and distinctive MRS peaks such as elevated N-acetylaspartate in Canavan disease and lactate in Leigh disease.
**Conclusion:** DWI and proton MRS are crucial for early diagnosis of pediatric metabolic brain disorders. Identifying characteristic imaging patterns enhances diagnostic precision and facilitates timely intervention, improving patient outcomes.
**Limitations:** The study's small sample size may limit the generalizability of the findings, and the cross-sectional design does not address disease progression over time. Future research should involve larger cohorts and longitudinal studies to validate these results and assess the long-term efficacy of imaging-based diagnostic methods.
**Funding for this study:** None
**Ethics committee - additional information:** Not required
**Author Disclosures:**
Ritika Agarwal: Nothing to disclose
Narendran Jha: Nothing to disclose
Ujjwal Gupta: Nothing to disclose

**Dynamic network dysfunction in children with idiopathic generalized epilepsy and its association with cognitive impairment and gene expression profiles**
*H. Ran*, K. Huang, T. Zhang; Zunyi/CN
*(haifengran_zmu@163.com)*

**Purpose or Learning Objective:** Idiopathic generalized epilepsy(IGE) has been considered as a network disease, recurrent seizures may result in network reconfiguration and cognitive impairments. The dynamic changes in functional network in IGE children and the relationship with cognitive impairment and gene expression profiles needs to be explored.
**Methods or Background:** 26 IGE children and 35 healthy controls(HC) were recruited, the modular variability(MV) of constructed time-varying multi-layer network was calculated and compared between groups based on rs-fMRI. The correlation analysis was performed between MV and cognitive function scores and clinical variables. Allen Human Brain Atlas were used to identify gene sets associated with dynamic network remodeling in IGE. The associated biological processes, pathways were identified by gene enrichment tools.
**Results or Findings:** Compared to HC, IGE children demonstrated changed MV mainly located in the sensorimotor areas, salience/ventral attention, and default mode network, and at the sub-network level, children with IGE exhibited increased MV in the default mode network(p<0.05, FDR). MV changes in the left prefrontal, precuneus cortex were negatively correlated with the verbal IQ, full scale IQ and performance IQ scores, respectively(r=-0.400, -0.419, -0.408; p=0.042, 0.032, 0.038), while MV in the right orbitofrontal cortex was positively correlated with the verbal IQ and full scale IQ scores, respectively(r=0.488, 0.442; p=0.011, 0.023). Gene expression profiles was associated with dynamic network dysfunction in IGE(r=0.499, pperm < 0.05). Enrichment analysis indicated that the genes related to dynamic network reorganization were principally enriched in dendrite, axon, and nervous system development.
**Conclusion:** In IGE children, altered dynamic functional networks has been identified and correlated with cognitive function and gene expression, revealing the complex relationship between the dynamic changes of macroscopic modules and genetic pathological mechanisms in IGE patients.
**Limitations:** The sample size of this study was relative small.
**Funding for this study:** This study was supported by National Natural Science Foundation of China (Grant Nos .82171919) and Intelligent Medical Imaging Engineering Research Center of Guizhou Higher Education Institutions project (Grant No. Qianjiaoji [2023] 038)
**Ethics committee - additional information:** Ethics approval of this research was granted by the Ethic Committee of the Affiliated Hospital of Zunyi Medical University[KLL-2021-347]
**Author Disclosures:**
Tijiang Zhang: Nothing to disclose
Kexin Huang: Nothing to disclose
Haifeng Ran: Nothing to disclose

**12:30-13:30 Research Stage 4**

### Research Presentation Session: Imaging Informatics and Artificial Intelligence

### RPS 1905

### Artificial intelligence in abdominal and oncological imaging

**Moderator**
G. P. Raval; Rajkot/IN
*(drgaurang.raval@gmail.com)*

**Total Segmentator: Integration and validation into PACS workstation for abdominal CT scans. A feasibility study**
*G. Lappas*[1], N. Patlakas[1], P. Giannikopoulos[1], M. Triantafyllou[2], G. I. Kalaitzakis[3], M. Klontzas[2], K. Petropoulos[1]; [1]Athens/GR, [2]Crete/GR, [3]Heraklion/GR

**Purpose or Learning Objective:** This study aims to validate the performance of the open-source Total Segmentator for the segmentation of abdominal organs in CT scans and integration into the existing PACS workstation.
**Methods or Background:** The model's segmentation capability was quantified using Dice Similarity Coefficient (DSC) and Normalized Surface Distance (NSD) across five datasets (N=1243). Data variability including statistics radiomics analysis was performed. Grad-CAM and Monte-Carlo were deployed focusing on the understanding of model decision-making and robustness, respectively. One assistant professor of radiology and one senior radiology resident rated the predicted segmentations' quality from Greek hospital CT scans (N=100) and integration into the clinical routine.

**Results or Findings:** The model demonstrated high accuracy in segmenting most organs, e.g., DSC - CI: 0.85-0.97, while showed lower performance on gallbladder, pancreas and prostate, e.g., DSC – CI: 0.71-0.85. Those results depict the robustness of the model across the five datasets which present high variability depicted by normalized volume (μ±σ: 0.24±0.18) and normalized intensity (μ±σ: 0.39±0.21) coming in agreement with radiomics findings. Grad-CAM and Monte-Carlo noise results provided insights into the model's decision-making process and highlighting areas for potential improvement. The clinical feasibility study indicates a promising tool generating high quality segmentations with 97% of those requiring minimal manual adaptations.
**Conclusion:** Total Segmentator showed robust performance in segmentation of the major abdominal organs with poorer performance for gallbladder, pancreas and prostate. The integration into PACS workstation proves the model's potential for routine use in Greek hospitals marking a significant step towards more efficient radiological flow.
**Limitations:** The model had lower performance for gallbladder, pancreas and prostate while 7% of the validated cases had disagreements larger than 50% volume-wise. The available number of CT scans used for rating by radiologists was limited.
**Funding for this study:** None
**Ethics committee - additional information:** Not applicable
**Author Disclosures:**
Michail Klontzas: Nothing to disclose
Konstantinos Petropoulos: Board Member: IKnowHealth S.A.
Nektarios Patlakas: Nothing to disclose
Georgios Ioannis Kalaitzakis: Nothing to disclose
Petros Giannikopoulos: Nothing to disclose
Georgios Lappas: Nothing to disclose
Matthaios Triantafyllou: Nothing to disclose

**Results of the ULS23 Challenge on automatic 3D universal lesion segmentation in computed tomography**
M. J. J. De Grauw[1], E. Scholten[1], E. J. Smit[1], M. J. Rutten[2], B. Van Ginneken[1], M. Prokop[1], *A. Hering*[1]; [1]Nijmegen/NL, [2]'S-Hertogenbosch/NL
*(alessa.hering@radboudumc.nl)*

**Purpose or Learning Objective:** Generalizable automatic segmentation methods are well-suited for application in the diverse clinical contexts encountered during tumour follow-up in CT. The ULS23 challenge establishes the state-of-the-art in automatic 3D lesion segmentation quality, measurement accuracy, and prediction robustness.
**Methods or Background:** Current benchmarks often focus on organ-specific lesion segmentation, yet diverse clinical cases require fast, generalist models. The ULS23 challenge focuses on universal 3D lesion segmentation across chest-abdomen-pelvis CT, with 38,693 diverse lesions in the training dataset. The evaluation dataset contains 775 clinically relevant lesions from 284 patients across two Dutch tertiary care centers. We developed a strong baseline method based on the nnUnet and invited the research community to submit their solutions to the challenge. Post-challenge, we conducted experiments to explore lesion type influence, uncertainty, and robustness.
**Results or Findings:** The ULS23 challenge encouraged over 50 international researchers to develop solutions for automatic 3D lesion segmentation. During the official challenge period, 153 submissions were recorded during the development phase with seven teams submitting to the final leaderboard. The U-mamba framework achieved the highest challenge score, excelling in segmentation quality (70.8% ± 23.5% Dice), axial measurement accuracy (Long-axis 10.3%, Short-axis 11.8% Symmetric Mean Absolute Percentage Error), and robustness when evaluated using repeated segmentation (79.7% ± 24.2% Dice). Bone, pancreas and colon lesions remain challenging, and while segmentation consistency improves with performance, models still showed significant variability, affecting measurement accuracy.
**Conclusion:** The results of the ULS23 challenge demonstrate the potential of 3D universal lesion segmentation using large, aggregated datasets as a viable alternative to organ-specific models. However, significant variability in segmentation performance, particularly for bone, pancreas and colon lesions, indicates the need for further improvements. Addressing these performance inconsistencies could help reduce variance and enhance overall clinical applicability.
**Limitations:** Not applicable.
**Funding for this study:** No funding was provided for this study.
**Ethics committee - additional information:** N/A
**Author Disclosures:**
Mathias Prokop: Patent Holder: Mevis Medical Solution Consultant: Canon Medical Systems, Siemens Healthineer Shareholder: Thirona
Ernst Scholten: Nothing to disclose
Max Jacobus Johannes De Grauw: Nothing to disclose
Alessa Hering: Nothing to disclose
Bram Van Ginneken: Founder: Plain, Thirona
Ewoud J. Smit: Nothing to disclose
Matthieu J.C.M. Rutten: Nothing to disclose

**Automated Matching of Lesions in Cancer Follow-Up Using 3D Siamese Neural Networks and CT**
*A. Vergara*, A. Jimenez-Pastor, A. Alberich-Bayarri; Valencia/ES
*(alejandrovergara@quibim.com)*

**Purpose or Learning Objective:** There is a lack of automated and reliable tools to automatically track lesion changes over time, as well as to streamline treatment response reporting, such as RECIST-1.1 and others. The primary goal of this work was to address the automatic matching of lesions in the thoracoabdominal region across timepoints through 3D-Siamese Neural Networks (SNN).
**Methods or Background:** A retrospective dataset of 253 longitudinal CT exams from metastatic NSCLC patients was used, with a high variability in lesion location, including lymph nodes/lungs/liver/adrenal glands (43.87%/34.02%/11.82%/6.17%, respectively) and volume (0.1-1000cm3). A 3D-SNN architecture was used to compare the similarity between tumor pairs and matching the same lesion across consecutive scans. The final model resulted from a two-step training process, initially evaluating 144 hyperparameter settings and subsequently retraining using the top-performing ones. Different configurations were created combining input information (CT image, CT image and lesion segmentation mask, and CT image with several HU windows), learning rate (LR), LR schedulers, convolutional block complexity (1/2 convolutional layers), and loss functions (contrastive and BCE). An 80/20 training/test split ensured comparable lesion size and location heterogeneity in both sets.
**Results or Findings:** The best model employed the CT image as input, a LR of 10e-4, a step LR scheduler, BCE loss, and 1-convolutional-layer block, delivering 92.80%/91.10%/92.4% in accuracy/precision/recall in the test set. The accuracy decomposed by location was 93.79%/88.32%/92.50%/94.35% for lymph nodes, lung, liver and adrenal gland, respectively. Considering lesion volumes, the accuracy was 90.57%/93.35%/94.48% for small (<10cm3), medium (10-100cm3), and large (>100cm3) ones.
**Conclusion:** 3D SNNs are a promising and reliable technique to accurately match tumor lesions over consecutive timepoints, regardless of lesion size and region.
**Limitations:** Current work focuses on matching lesions individually, not the entire patient, which is future work.
**Funding for this study:** None
**Ethics committee - additional information:** N/A
**Author Disclosures:**
Alejandro Vergara: Nothing to disclose
Ana Jimenez-Pastor: Nothing to disclose
Angel Alberich-Bayarri: Nothing to disclose

**Comparative Analysis of Language Models for Automated Interpretation of Longitudinal TACE Reports in Hepatocellular Carcinoma**
*E. Can*[1], E. Kotter[1], K. Vogt[1], M. Brönnimann[2], A. Elkilany[3], W. Uller[1], K. Bressem[4], L. C. Adams[4]; [1]Freiburg/DE, [2]Bern/CH, [3]Leipzig/DE, [4]Munich/DE
*(elif.can@uniklinik-freiburg.de)*

**Purpose or Learning Objective:** The increasing complexity of radiology data in hepatocellular carcinoma (HCC) requires innovative solutions to ensure consistent and efficient interpretation. This study aims to evaluate the performance of four leading language models (GPT, Gemini, Llama, and Llama405b) in extracting and interpreting key clinical data from longitudinal TACE reports. By automating this process, we seek to reduce the burden on radiologists, enhance decision-making accuracy, and improve workflow efficiency in interventional radiology.
**Methods or Background:** We analyzed the performance of each model on 50 anonymized TACE reports. The models were assessed for accuracy in extracting clinical data (diagnosis, BCLC staging, mRECIST assessment), as well as vascular involvement and metastases identification. A detailed error analysis was conducted to evaluate consistency and precision across tasks. Performance metrics included diagnosis accuracy, liver segment identification, and error severity.
**Results or Findings:** All models demonstrated high accuracy (90-100%) in basic tasks such as diagnosis identification and procedure date extraction. However, in complex tasks like mRECIST assessment and lymph node status evaluation, performance varied significantly. Gemini outperformed in segment identification (4.6/5) and vascular involvement (2.9/5), while all models struggled with mRECIST accuracy (0-10%). Llama and Llama405b exhibited slightly higher error rates (7.7, 8.0) compared to GPT (6.5) and Gemini (6.6).
**Conclusion:** While language models show promise in automating TACE report interpretation, challenges remain in specialized medical tasks. Gemini showed the best overall performance, but significant improvements are needed in mRECIST assessment and anatomical reporting. Further research is required to refine these models for clinical application, potentially reducing radiologists' workload and enhancing decision-making processes.
**Limitations:** The models' generalizability across other forms of interventional radiology remains to be validated.
**Funding for this study:** No funding provided.

**Ethics committee - additional information:** Approved by the ethics committee of the University Medical Center Freiburg.
**Author Disclosures:**
Elif Can: Nothing to disclose
Lisa C. Adams: Nothing to disclose
Aboelyazid Elkilany: Nothing to disclose
Katharina Vogt: Nothing to disclose
Michael Brönnimann: Nothing to disclose
Keno Bressem: Nothing to disclose
Wibke Uller: Nothing to disclose
Elmar Kotter: Nothing to disclose

**CT-based Foundation Model Enhanced Preoperative Prediction of Microvascular Invasion in Hepatocellular Carcinoma: a multicenter study**
*L. Deng*, W. Xia, J. Xia, W. Dai, F. Yan, R. Li; Shanghai/CN
*(dl13075@rjh.com.cn)*

**Purpose or Learning Objective:** To develop CT-based foundation model and multi-instance learning (MIL) framework to preoperatively predict microvascular invasion (MVI) in hepatocellular carcinoma (HCC).
**Methods or Background:** CT-based foundation models were developed through self-supervised learning using public CT image datasets. Patients with pathologically proven HCC were included from two centers, and the CT image sequences of non-contrast, arterial and portal venous phase were acquired. The features from slices of HCC tumor region were extracted by foundation models, and the features were aggregated by MIL to predict MVI status. The predictions of all sequences were combined to obtain a final prediction by logistics regression. The performance of proposed method was evaluated by area under the receiver operating characteristic curve (AUC).
**Results or Findings:** The CT image patches of 36,811 lesions from DeepLesion, LiTS and 3D-IRCADb were used for foundation model development. A total of 617 HCC patients (median age, 69 years; IQR, 52-67 years; men 510) were included and divided into training set (center 1, n=493) and independent test set (center 2, n=124). By using a few slices of HCC tumor region (median number, 3; IQR, 2-4), the proposed method achieved AUCs of 0.80, 0.78, 0.74, and 0.83 for non-contrast, arterial phase, portal venous phase, and all sequences combined, respectively. The proposed method significantly outperformed the method without foundation model (AUC=0.72, 0.67, 0.71 and 0.64 for each sequence, P<.05) and the radiomics model (AUC=0.72, P<.05).
**Conclusion:** By leveraging a few representative CT slices and avoiding the need for full tumor delineation, the foundation model-based method achieves improved performance compared to previous methods, demonstrating the crucial role of the foundation model for accurate MVI prediction and facilitating more efficient clinical decision-making.
**Limitations:** Not applicable.
**Funding for this study:** Not applicable.
**Ethics committee - additional information:** Not applicable.
**Author Disclosures:**
Wei Xia: Nothing to disclose
Ruokun Li: Nothing to disclose
Fuhua Yan: Nothing to disclose
Ji Xia: Nothing to disclose
Wenwen Dai: Nothing to disclose
Lin Deng: Nothing to disclose

**Artificial intelligence for evaluation of magnetic resonance imaging-detected extramural vascular invasion in rectal cancer**
*H. Huang*, K. Zhao, Z. Liu, C. Liang; Guangzhou/CN
*(huanghaitao@gdph.org.cn)*

**Purpose or Learning Objective:** Extramural vascular invasion (EMVI) is a detectable magnetic resonance imaging (MRI) marker that reflects both tumor invasive and metastatic potential. Since EMVI+ is seen as an independent indicator of worse prognosis, clinicians may pursue more intensive treatment strategies for affected patients. However, EMVI assessment was influenced by observer experience and subjective factors, limiting its practical effectiveness. This study aims to develop and validate an interpretable deep learning-based approach for automated mrEMVI identification through voxel-level segmentation, providing objective and consistent detection method.
**Methods or Background:** This is a multicenter study that included a total of 2,501 rectal cancer patients, with 1,830 in the training cohort and 671 in the validation cohorts. Dice similarity score was used to measure segmentation performance, while the inter-reader agreement of mrEMVI was calculated using Cohen's Kappa (κ). The prognostic value of mrEMVI statuses identified by the artificial intelligence (AI) model was evaluated by Kaplan–Meier curves and the Cox model.
**Results or Findings:** Our model demonstrated excellent performance in identifying mrEMVI, achieving accuracy of 81.54% and 84.72% in the two validation cohorts. The model demonstrated a high level of inter-reader consistency with senior radiologists in identifying mrEMVI status (κ: 0.713–0.736). AI-mrEMVI+ patients have significantly shorter 3-year disease-free survival (DFS) and 5-year overall survival (OS) compared to AI-mrEMVI– patients (DFS: 62.23% vs 84.91%, HR=2.67 95% CI: 1.95–3.66, P<0.001; OS: 68.71% vs 87.14%, HR=2.64 95% CI: 1.75–3.97, P<0.001).
**Conclusion:** We provide a more objective and consistent approach for the detection of mrEMVI, demonstrating potential in prognostic prediction, and offering promising contributions to optimizing the treatment of rectal cancer patients.
**Limitations:** the model exhibited some false positives, primarily because the model mistakenly identified larger blood vessels in the mesorectal area as mrEMVI.
**Funding for this study:** National Science Foundation for Young Scientists of China (82202267).
**Ethics committee - additional information:** This study has obtained approval from the ethics review committees of all participating hospitals. Considering the retrospective design of the study, the requirement for written informed consent from patients was waived.
**Author Disclosures:**
Changhong Liang: Author: Administrative support, manuscript review
Zaiyi Liu: Author: Administrative support, manuscript review
Haitao Huang: Author: Study design, analyzed the data, manuscript Writing
Ke Zhao: Author: Study design, analyzed the data

**PROVIZ Proof-of-Technology: Performance of a machine learning software for detection of clinically significant prostate cancer on biparametric MRI in a prospective clinical study**
*R. Segre*, M. Sunoqrot, G. Nketiah, P. Davik, S. Langorgen, M. Elschot, T. Frost Bathen; Trondheim/NO
*(rebecca.segre@ntnu.no)*

**Purpose or Learning Objective:** PROVIZ is a machine learning software designed to detect clinically significant prostate cancer (csPCa, defined as GGG > 1) on MRI as a reference for targeted biopsy. The aim of this study is to evaluate feasibility (technical issues in < 10% of processed cases), safety (absence of Serious Adverse Device Effects, SADEs), and performance of PROVIZ.
**Methods or Background:** Prospective, proof-of-technology study on 80 consenting men. Inclusion criteria: biopsy-naive men undergoing MRI for suspicion of prostate cancer. MR images were first delineated according to PI-RADS v. 2.1 by an experienced radiologist. Subsequently, automated detection (up to three suspicious areas) was performed using PROVIZ. Delineations of all lesions were used as a reference for targeted biopsies, providing the ground truth.
**Results or Findings:** To date, 73/80 patients have completed the study. Regarding feasibility, one technical issue was experienced among the participants. In terms of safety, no SADEs were observed. As to performance, PROVIZ scored a high AUROC (92.3%) and can be retrospectively tuned to reach the same patient-level sensitivity of the radiologist at PI-RADS 3 (94.6%), while gaining an improvement in specificity (72.2% vs 52.8%). At this operating point, PROVIZ would have referred 7 less patients to biopsy than the radiologist (45/73 vs 52/73). On a lesion-level, PROVIZ showed a slightly lower sensitivity than the radiologist (42.9% vs 45.5%) but fewer false positives per case (30.1% vs 41.1%).
**Conclusion:** Preliminary results of this prospective study indicate that PROVIZ is feasible and safe to use, with a performance for detection of csPCa comparable to an experienced radiologist. As a support tool for the radiologist, PROVIZ shows potential for reducing false positive predictions, therefore minimizing unnecessary biopsies.
**Limitations:** Incomplete data collection. Single-center study.
**Funding for this study:** Norwegian University of Science and Technology (NTNU), Research Council of Norway (Grant Number 295013), The Liaison Committee between the Central Norway Regional Health Authority and the Norwegian University of Science and Technology (Grant Numbers 983005100, 982992100 and 90368401), St. Olavs Hospital - Trondheim University Hospital, Central Norway Regional Health Authority.
**Ethics committee - additional information:** REK (Regionale komiteer for medisinsk og helsefaglig forskningsetikk) approval no. 479272.
**Author Disclosures:**
Sverre Langorgen: Nothing to disclose
Matthijs Elschot: Nothing to disclose
Rebecca Segre: Nothing to disclose
Tone Frost Bathen: Nothing to disclose
Mohammed Sunoqrot: Nothing to disclose
Petter Davik: Nothing to disclose
Gabriel Nketiah: Nothing to disclose

**Diagnostic performance of a fully automated AI algorithm for lesion detection and PI-RADS classification in patients with suspected prostate cancer**

*H. Engel*[1], A. Nedelcu[1], R. Grimm[2], H. Von Busch[2], A. Sigle[3], T. Krauß[1], J. Weiß[1], M. Benndorf[4], B. Oerther[1]; [1]Freiburg im Breisgau/DE, [2]Forchheim/DE, [3]Freiburg/DE, [4]Detmold/DE

**Purpose or Learning Objective:** To evaluate the diagnostic performance of a fully automated AI algorithm with lesion detection and PI-RADS classification in a cohort of consecutive patients verified by targeted and extensive systematic biopsies.
**Methods or Background:** This retrospective, single-centre study included consecutive patients who underwent 3T multiparametric prostate magnetic resonance imaging (MRI) performed between 05/2017 and 05/2020, followed by targeted transperineal ultrasound-fusion guided and systematic biopsy. The AI algorithm (syngo.via Prostate MR, VB60S HF01, Siemens Healthineers) was described in previous publications and is based on axial T2- and diffusion-weighted imaging sequences. The results of the AI algorithm were compared with those of human readers and the diagnostic performance was determined.
**Results or Findings:** The evaluation of 272 patients resulted in 436 target lesions. 135 patients (49.5%) had clinically significant prostate cancer (csPCa), 35 (12.8%) had clinically insignificant prostate cancer (ISUP=1) and 102 (37.5%) were benign. Patient-level cancer detection rates (CDRs) of csPCa for AI versus human reading were 11%/18% for PI-RADS ≤2, 24%/11% for PI-RADS 3, 54%/41% for PI-RADS 4, and 74%/92% for PI-RADS 5. The accuracy of the AI was significantly better (0.74 versus 0.63 at a threshold of PI-RADS ≥4, p <0.01). 62 patients with human reading PI-RADS ≥3 were correctly classified as true negative by AI.
**Conclusion:** The AI algorithm proved to be a reliable and robust tool for lesion detection and classification. Furthermore, the CDRs and distribution of PI-RADS assessment categories of the AI are consistent with the results of recent meta-analyses, indicating precise risk stratification.
**Limitations:** The limitations of our study are mainly its retrospective and monocentric design. Additionally, the study design based on histopathological verification implies an under-representation of negative MRI scans and a cohort that is not fully representative of the wider patient population.
**Funding for this study:** The licence of the AI algorithm was part of an unrestricted collaboration agreement between Siemens Healthineers and the Department of Radiology, Medical Center - University of Freiburg, Faculty of Medicine, University of Freiburg. While Siemens provided technical support, the study conception and design, as well as the analysis and interpretation of the data, were conducted independently. August Sigle received research support within the Berta-Ottenstein-Programme. Other than that, the authors declare that no funds, grants, or other support were received during the preparation of this manuscript.
**Ethics committee - additional information:** Approval was granted by the Ethics Committee of the University of Freiburg (No. 20-1256).
**Author Disclosures:**
Tobias Krauß: Nothing to disclose
Benedict Oerther: Nothing to disclose
August Sigle: Nothing to disclose
Jakob Weiß: Nothing to disclose
Robert Grimm: Nothing to disclose
Andrea Nedelcu: Nothing to disclose
Matthias Benndorf: Nothing to disclose
Hannes Engel: Nothing to disclose
Heinrich Von Busch: Nothing to disclose

**14:00-15:30 Research Stage 1**

### Research Presentation Session: Cardiac

### RPS 2003

### Cardiac applications of photon-counting CT

**Moderator**
F. Catapano; Milan/IT
*(federica.catapano@humanitas.it)*

**Ultrahigh-resolution photon-counting detector CT detects a significantly lower coronary plaque volume than energy-integrating detector CT**

*M. Vecsey-Nagy*[1], G. Tremamunno[1], C. Gnasso[1], E. Zsarnóczay[2], D. Kravchenko[2], B. Szilveszter[2], P. Maurovich-Horvat[2], A. Varga-Szemes[1], T. S. Emrich[1]; [1]Charleston, SC/US, [2]Budapest/HU
*(vnagymilan@gmail.com)*

**Purpose or Learning Objective:** To evaluate the effect of photon-counting detector (PCD)-CT on coronary plaque quantification and characterization on coronary CT angiography (CCTA) series compared to energy-integrating detector (EID)-CT.
**Methods or Background:** Consecutive patients undergoing clinically indicated CCTA on EID-CT (192×0.6 mm collimation) were enrolled for an ultrahigh-spatial-resolution (UHR) PCD-CT scan (120×0.2 mm collimation) within 30 days. PCD-CT was acquired using equivalent or lower CT dose index and equivalent contrast media volume as the clinical scan. Total, calcified, fibrotic, and low-attenuation coronary plaque volumes were quantified and compared between scanners. Intra- and inter-reader reproducibility was assessed on both systems.
**Results or Findings:** A total of 164 plaques from 48 patients were segmented on both scans. Total plaque volume was lower on PCD-CT compared to EID-CT (723.5 [interquartile range: 500.6–1184.7] vs. 1084.7 [IQR: 710.7–1609.8] mm3, p<0.001). UHR-based segmentations produced lower fibrotic plaque volumes than EID-CT-based measurements (325.4 [IQR: 151.7–519.2] vs. 627.7 [IQR: 385.8–795.1] mm3, p<0.001), while low-attenuation (72.1 [IQR: 38.6–161.9] vs. 58.1 [IQR: 23.4–102.3] mm3, p=0.052) and calcified plaque volumes (IQR: 344.5 [174.3–605.7] vs. 342.1 [IQR: 180.4–607.5] mm3, p=0.50) did not differ significantly between PCD-CT and EID-CT. Total, calcified, and fibrotic plaque volumes demonstrated excellent agreement between repeated measurements and between readers for both PCD-CT and EID-CT (all intraclass correlation coefficients >0.90). While low-attenuation plaque volume had strong intra- (ICC: 0.84 [95%CI, 0.57–0.94]) and inter-reader (ICC: 0.92 [95%CI, 0.81–0.97]) agreements for PCD-CT, EID-CT showed only moderate (ICC: 0.62 [95%CI, 0.11–0.86]) and poor (ICC: 0.47 [95%CI, 0.01–0.79]) intra- and inter-reader reproducibility.
**Conclusion:** Compared to EID-CT, PCD-CT UHR imaging reduces segmented coronary plaque volume by nearly one-third and improves the reproducibility of low-attenuation plaque measurements.
**Limitations:** Lack of invasive reference.
**Funding for this study:** The study was funded by a research grant from Siemens Healthineers.
**Ethics committee - additional information:** Local Ethics Committee approved the present study.
**Author Disclosures:**
Emese Zsarnóczay: Nothing to disclose
Pál Maurovich-Horvat: Research/Grant Support: Siemens Healthineers
Milán Vecsey-Nagy: Nothing to disclose
Giuseppe Tremamunno: Nothing to disclose
Dmitrij Kravchenko: Nothing to disclose
Bálint Szilveszter: Nothing to disclose
Tilman Stephan Emrich: Research/Grant Support: Siemens Healthineers
Chiara Gnasso: Nothing to disclose
Akos Varga-Szemes: Research/Grant Support: Siemens Healthineers

**Reducing Variability in Coronary Plaque Characterization with Photon-Counting CT**

A. Choux[1], S. Sharma[2], S. Ross[2], R. Thompson[2], Z. Yu[2], *A. Pourmorteza*[1]; [1]Atlanta, GA/US, [2]Vernon Hills, IL/US
*(amir.pourmorteza@gmail.com)*

**Purpose or Learning Objective:** Photon-counting detector CT (PCD-CT) has demonstrated significant radiation dose reduction capabilities compared to energy-integrating detector CT (EID-CT), particularly for coronary artery calcium scoring (CACS). Alternatively, the dose savings from PCD-CT can be leveraged to improve the reproducibility of CACS by reconstructing images with thinner slice thicknesses to reduce variability from partial volume effects (PVE). This ex vivo study aims to evaluate and quantify the effect of PVE on reproducibility of calcified plaque volume measurements.
**Methods or Background:** Six excised human hearts with varying degrees of calcification were placed inside a chest phantom and scanned on a CdZnTe-based PCD-CT (120 kVp and 250 mAs). To simulate variability in the scan and reconstruction ranges, 10 image volumes were reconstructed for each heart using identical parameters, with the only variation being the starting slice location, which was incremented by 1/10 of the slice thickness for each reconstruction. Images were reconstructed using FBP (soft kernel, 0.3 mm in-plane pixel size) at both 3-mm (recommended for CACS) and 1.5-mm slice thicknesses. Calcium volume was measured using 130 HU threshold (as per SCCT guidelines). Variability was quantified as the ratio of std deviation and mean of calcium volume measurements for each heart and a paired t-test (alpha=0.05) was used for establishing statistical significance.
**Results or Findings:** Variability was found to be 36.2% for conventional 3-mm images, which was significantly higher than the 16.6% variability for 1.5-mm images (p<0.001).
**Conclusion:** Slight variations in scan or reconstruction range leads to significant variability in coronary plaque characterization. Taking advantage of thinner slices provided by PCD-CT (matched in radiation dose to thick EID slices) can mitigate this variability drastically.
**Limitations:** This was a small sample size ex-vivo study.
**Funding for this study:** Sponsored research agreement with Canon Medical Research USA, Inc.
**Ethics committee - additional information:** Ex vivo study, did not require ethics committee approval
**Author Disclosures:**
Zhou Yu: Employee: CMRU
Steven Ross: Employee: CMRU
Richard Thompson: Employee: CMRU
Arnaud Choux: Research/Grant Support: Canon Medical Research USA, GE HealthCare
Amir Pourmorteza: Grant Recipient: Canon Medical Research USA, GE HealthCare
Shobhit Sharma: Employee: CMRU

**Quantification of Coronary Plaque Components with Photon-counting CT: Analyzing Software Consistency and Variability**
*M. Gruber*, D. Beitzke, C. Loewe, D. Beitzke; Vienna/AT
*(manuel.gruber19@gmail.com)*

**Purpose or Learning Objective:** Coronary computed tomography angiography (CCTA) enables non-invasive quantification of plaque burden and composition. The aim of this study was to evaluate the intra-reader and inter-platform reproducibility of coronary plaque volume and composition measurements from two plaque analysis software solutions using a first-generation Photon-counting CT (PCCT) system.
**Methods or Background:** Twenty plaques from thirteen patients who underwent CCTA with a slice thickness of 0.4 mm were analyzed. Plaque quantification was performed using two dedicated software solutions (Software 1: Syngo.via Frontier CT Coronary Plaque Analysis, Siemens Healthineers; Software 2: QAngio CT, Medis Medical Imaging Systems). Volumes of coronary lumen, total plaque and plaque components were assessed at two time points with each software using the following attenuation thresholds for plaque classification in Hounsfield units (HU) : necrotic core: -30 to 30 HU, fibrotic plaque: 31 to 350 HU and calcified plaque: >351 HU.
**Results or Findings:** Intraobserver variability, determined by the Pearson correlation coefficient showed a strong positive correlation (r = 0.94, p< 0.001), indicating a high consistency in repeated measures. Furthermore, the paired-samples t-test showed no statistically significant difference between the two time points for Software 1 t(19) = 0.77, p= 0.45 or Software 2 t(19) = -0.81, p= 0.43). A repeated-measures ANOVA revealed no significant main effect for the software type, but differences were observed for lumen volume and calcified plaque volume with F(11.78, 33.85) =12.81, p< 0.001 and F(1.29, 24.56) = 13.89, p < 0.001, respectively.
**Conclusion:** Quantification measurements of plaque with PCCT are feasible and highly reproducible, indicating that software comparisons should take into account potential differences in measurements of specific components.
**Limitations:** Limitations of this study are the relatively small sample size, as well as the reliance on a single reader.
**Funding for this study:** Beitzke Daniela as a research radiographer is supported by a research grant from Siemens Healthineers.
**Ethics committee - additional information:** The study is approved by the local Ethics board. All patients gave written and informed consent.
**Author Disclosures:**
Manuel Gruber: Nothing to disclose
Dietrich Beitzke: Nothing to disclose
Christian Loewe: Nothing to disclose
Daniela Beitzke: Research/Grant Support: Siemens Healthineers

**Semiquantitative Metrics of Coronary Artery Disease Burden: Intra-Individual Comparison between Ultrahigh-Resolution Photon-Counting Detector CT and Energy-Integrating Detector CT**
*G. Tremamunno*[1], A. Varga-Szemes[2], U. J. Schoepf[2], D. Kravchenko[2], M. T. Hagar[2], A. Laghi[1], T. S. Emrich[2], M. Vecsey-Nagy[2]; [1]Rome/IT, [2]Charleston, SC/US
*(giuseppe.tremamunno@uniroma1.it)*

**Purpose or Learning Objective:** To assess the impact of ultrahigh-resolution (UHR) photon-counting detector (PCD)-CT on the semiquantitative evaluation of coronary artery disease (CAD) compared to energy-integrating detector (EID)-CT.
**Methods or Background:** Patients undergoing coronary CT angiography (CCTA) on an EID-CT system were prospectively enrolled for UHR PCD-CT scan within 30 days. Both datasets were visually evaluated using five established semiquantitative scores: Segment Involvement Score (SIS), Segment Stenosis Score (SSS), Multivessel Aggregate Stenosis Score (MVAS), CCTA-adapted Leaman score (CT-LeSc), and Coronary Artery Disease Reporting and Data System (CAD-RADS). Additionally, the total number of detected plaques and high-risk features were reported (positive remodeling, spotty calcification, low-attenuation, and napkin-ring sign).
**Results or Findings:** The cohort comprised 46 patients (37 men, 68.4±6.9 years). When assessing stenosis severity, PCD-CT showed lower SSS (3.5 [1.3-5.0] vs 6.5 [3.0-9.8], p<0.001), MVAS (5.5 [4.0-7.0] vs 7.0 [5.0-9.0], p<0.001), and CT-LeSc (10.4 [8.5-13.9] vs 11.2 [8.8-15.4], p=0.032). Furthermore, 52% (24/46) of patients were reclassified to a lower CAD-RADS category compared to EID-CT. In terms of CAD extent, PCD-CT demonstrated higher SIS (8.0 [6.0-9.0] vs 7.0 [6.0-8.8], p=0.018) and plaque count (9.0 [7.0-13.8] vs 7.0 [7.0-9.8] p<0.001). Positive remodeling was less frequent in PCD-CT datasets (2.0 [1.0-4.3] vs 1.0 [0.0-3.0], p=0.012), with no significant differences in other high-risk features.
**Conclusion:** The use of UHR PCD-CT results in the detection of less severe, but more extensive CAD in the same patient compared to EID-CT. The effect of such CCTA-based differences on individual risk stratification needs further investigation.
**Limitations:** Only a limited number of patients were enrolled and the characterization and extent of plaques were not validated against invasive reference.
**Funding for this study:** This study received funding by Siemens Healthineers
**Ethics committee - additional information:** Name of Institution: Medical University of South Carolina
Code/Number: Pro00108359
Date of Approval: 4/13/2021
**Author Disclosures:**
Muhammad Taha Hagar: Nothing to disclose
Milán Vecsey-Nagy: Nothing to disclose
Uwe Joseph Schoepf: Research/Grant Support: Siemens Healthineers
Giuseppe Tremamunno: Nothing to disclose
Dmitrij Kravchenko: Nothing to disclose
Andrea Laghi: Nothing to disclose
Tilman Stephan Emrich: Research/Grant Support: Siemens Healthineers
Akos Varga-Szemes: Research/Grant Support: Siemens Healthineers

**Cost-effectiveness of ultrahigh-resolution photon-counting detector coronary CT angiography for the evaluation of stable chest pain**
*M. Vecsey-Nagy*[1], T. S. Emrich[1], G. Tremamunno[1], D. Kravchenko[1], M. T. Hagar[1], B. Szilveszter[2], P. Maurovich-Horvat[2], A. Varga-Szemes[1], J. A. Decker[3]; [1]Charleston, SC/US, [2]Budapest/HU, [3]Augsburg/DE
*(vnagymilan@gmail.com)*

**Purpose or Learning Objective:** To simulate the cost-effectiveness of ultrahigh-resolution (UHR) photon-counting detector (PCD)-CT in stable chest pain patients undergoing coronary CT angiography (CCTA).
**Methods or Background:** A decision and simulation model was developed using Monte Carlo simulations with 1,000 bootstrap resamples to estimate the costs associated with PCD-CT in lieu of EID-CT for CCTA and the referral for subsequent testing. The model was constructed using the diagnostic accuracy metrics of 55 coronary lesions of patients who underwent CCTA on both CT systems and subsequent invasive coronary angiography (ICA). Sensitivity and specificity were defined for each Coronary Artery Disease Reporting and Data System category. The aggregate healthcare expenditures were derived from the hospital billing system.
**Results or Findings:** Assuming a projected cohort of 15,000 patients over the lifetime of the PCD-CT, its implementation resulted in a 18.9% reduction in the number of functional follow-up tests (6330.3 ± 59.5 vs. 5135.7 ± 60.6, p<0.001), a 6.0% reduction in performed ICAs (1,447.7 ± 36.2 vs. 1,360.2 ± 34.7, p<0.001), and a 9.4% decrease in major procedure-related complications. Over a 10-year expected life expectancy, PCD-CT led to an average cost saving of $794.50 ± 18.50 per patient and an overall cost difference of $11,917,500 ± 4,350,169.

**Conclusion:** PCD-CT has the potential to reduce the financial burden on healthcare systems and procedure-related complications for stable chest pain patients with coronary calcification when compared to EID-CT.
**Limitations:** Limited number of lesions included.
**Funding for this study:** The study was funded by a research grant from Siemens Healthineers.
**Ethics committee - additional information:** Local Ethics Committee approved the present study.
**Author Disclosures:**
Muhammad Taha Hagar: Nothing to disclose
Pál Maurovich-Horvat: Research/Grant Support: Siemens Healthineers
Milán Vecsey-Nagy: Nothing to disclose
Giuseppe Tremamunno: Nothing to disclose
Dmitrij Kravchenko: Nothing to disclose
Bálint Szilveszter: Nothing to disclose
Tilman Stephan Emrich: Research/Grant Support: Siemens Healthineers
Josua A. Decker: Nothing to disclose
Akos Varga-Szemes: Research/Grant Support: Siemens Healthineers

**Intra-individual differences in pericoronary fat attenuation index measurements between photon-counting and energy-integrating detector computed tomography**
G. Tremamunno[1], M. Vecsey-Nagy[1], M. T. Hagar[1], U. J. Schoepf[1], J. O'Doherty[1], J. A. Luetkens[2], A. Varga-Szemes[1], T. S. Emrich[1], *D. Kravchenko*[1]; [1]Charleston, SC/US, [2]Bonn/DE

**Purpose or Learning Objective:** Pericoronary adipose tissue (PCAT) fat attenuation index (FAI) predicts major adverse cardiac events, but is known to be influenced by multiple factors, such as kernel sharpness, slice thickness, and tube potential. The objective of this study was to explore intra-individual differences in PCAT FAI between PCD- and energy-integrating detector (EID)-CT.
**Methods or Background:** Patients were prospectively enrolled for a PCD-CT research scan after EID coronary CT angiography. Reconstructions were performed using a Qr36 kernel at 0.6 mm slice thickness (EID and PCD-down-sampled [DS]) and at 0.2 mm ultra-high resolution (UHR) for the PCD-CT. Data was processed either with no use of iterative reconstruction, using a weighted filter back projection, or set to a strength level of Advanced Modeled Iterative Reconstruction 3 for the EID-CT and Quantum Iterative Reconstruction 4 for the PCD-CT. PCAT FAI of the right coronary artery (RCA), left anterior descending artery, and left circumflex artery (LCX) was measured automatically using established thresholds of -190 to -30 HU at a set distance and radius. Statistical testing was performed using repeated-measures ANOVA and Bonferroni's multiple comparison tests (p<.003).
**Results or Findings:** 40 patients (mean age 68±8 years, 32 males [80%]) were analyzed. Absolute FAI measurements differed significantly for all vessels between all reconstructions in the ANOVA comparison (all p<.001). The mean FAI when using iterative reconstruction did not demonstrate significant differences on multiple comparisons (e.g. LCX: EID: -65.7±8.5; PCD-DS: -66.0±7.4; PCD-UHR: -67.8±7.0 HU, respectively; all p >.05).
**Conclusion:** Intra-individual absolute PCAT FAI measurements differ significantly between EID- and PCD-CT when controlling for reconstruction kernel and slice thickness. However, the use of iterative reconstruction minimizes most differences in FAI, enabling inter-scanner comparability.
**Limitations:** Small population size and not all patients underwent both examinations at the same tube potential.
**Funding for this study:** In part supported by a research grant from Siemens Healthineers.
**Ethics committee - additional information:** This HIPAA compliant single-center study received approval from the Institutional Review Board of the Medical University of South Carolina.
**Author Disclosures:**
Julian Alexander Luetkens: Nothing to disclose
Muhammad Taha Hagar: Nothing to disclose
Milán Vecsey-Nagy: Nothing to disclose
Jim O'Doherty: Employee: Siemens
Uwe Joseph Schoepf: Consultant: Keya Medical Consultant: HeartFlow Consultant: Bracco Consultant: Bayer Grant Recipient: Siemens Consultant: Elucid Consultant: Guerbert
Giuseppe Tremamunno: Nothing to disclose
Dmitrij Kravchenko: Speaker: Philips
Tilman Stephan Emrich: Speaker: Siemens
Akos Varga-Szemes: Consultant: Elucid Grant Recipient: Siemens

**Correlation between hemodynamically significant stenoses and spectral photon-counting CT first-pass myocardial perfusion imaging compared with dual-energy CT in very-high risk patients**
*G. Fahrni*[1], S. A. Si-Mohamed[2], R. Wiemker[3], D. C. Rotzinger[1], A. Houmeau[2], C. Prieur[2], P. C. Douek[2], S. Boccalini[2]; [1]Lausanne/CH, [2]Lyon/FR, [3]Hamburg/DE

**Purpose or Learning Objective:** To assess the capabilities of first-pass myocardial perfusion imaging (MPI) with Spectral Photon Counting CT (SPCCT) Coronary Angiography to detect fractional-flow reserve (FFR) positive coronary artery stenoses, compared with dual-energy CT (DECT) in a very-high cardiovascular risk population.
**Methods or Background:** 18 very-high cardiovascular risk patients referred for CCTA after diagnostic invasive coronary angiography (ICA) were prospectively included (15 men, 3 women). ECG-gated CCTA was performed with DECT (IQon CT and CT7500, Philips Healthcare) and SPCCT (Philips clinical SPCCT prototype, Philips Healthcare), within 3 days. First-pass perfusion images were reconstructed with high-resolution (DECT) and ultra-high-resolution (SPCCT) parameters. Myocardial territories were visually labelled as normal or showing hypoperfusion. These labels were then compared to hemodynamic positivity on diagnostic ICA, indicated by either by significant stenosis (>70%) or FFR-positive stenosis. Myocardial attenuation was measured in normal and hypoperfusion territories.
**Results or Findings:** A total of 54 coronary artery territories were included. At ICA, significant stenosis was found in 15 (26%) arteries (8 LAD, 3 CX, 4 RCA). 11 lesions were significant stenoses, 4 were FFR-positive. Seventeen (31%) coronary artery territories were labelled as hypoperfusion for DECT and 18 (33%) for SPCCT. There was a significant difference between normal and hypoperfusion segments both for DECT (mean: 97±43 HU versus 80±35 HU, p<0.001) and SPCCT (mean: 92±86 HU versus 76±74 HU, p<0.001). A sensitivity for hemodynamically significant stenoses detection of 73% and specificity of 69% were found for DECT, versus 60% ad 72% for SPCCT.
**Conclusion:** In very-high cardiovascular risk population, SPCCT first-pass myocardial perfusion imaging was comparable to DECT in detecting myocardial hypoperfusion in significant or FFR-positive coronary artery stenosis territories.
**Limitations:** Small cohort of patients and predominance of LAD lesions.
**Funding for this study:** This work was supported by the European Union Horizon 2020 grant No. 643694. G.F. is supported by a research grand from the Swiss Society of Radiology (SSR, Luzern, Switzerland) and Lausanne University hospital (CHUV, Lausanne, Switzerland).
**Ethics committee - additional information:** Hospices Civils de Lyon, approval number: 2019-A02945–52, SPEQUA study
**Author Disclosures:**
Salim Aymeric Si-Mohamed: Nothing to disclose
Cyril Prieur: Nothing to disclose
Angele Houmeau: Nothing to disclose
Guillaume Fahrni: Nothing to disclose
David Christian Rotzinger: Nothing to disclose
Rafael Wiemker: Employee: Philips Innovative Technologies, 22335 Hamburg, Germany
Philippe Charles Douek: Nothing to disclose
Sara Boccalini: Nothing to disclose

**Myocardial Extracellular Volume Using Photon-Counting Detector CT with Synthetic Hematocrit Derived from Virtual Non-Contrast Images**
*V. Mergen*, N. Ehrbar, L. J. Moser, R. Manka, H. Alkadhi, M. Eberhard; Zurich/CH
*(victor.mergen@usz.ch)*

**Purpose or Learning Objective:** To evaluate the accuracy of myocardial extracellular volume (ECV) calculation using a synthetic hematocrit derived from virtual non-iodine images (VNI) and virtual non-contrast images (VNC) with photon-counting detector (PCD) CT.
**Methods or Background:** In this retrospective study, 125 consecutive patients examined by a PCD coronary CT angiography (CCTA) and a cardiac late enhancement (LE) scan, and having a recent blood hematocrit were included. In the derivation cohort (first 75 patients), CCTA and LE scans were reconstructed as VNI at 60, 70, and 80keV and as VNC with quantum iterative reconstruction (QIR) strengths 2, 3, and 4. Mean blood pool attenuation (BPmean) was correlated to the blood hematocrit. In the validation cohort (next 50 patients), BPmean served to calculate a synthetic hematocrit. Myocardial ECV was computed using the synthetic hematocrit and compared with the ECV using the blood hematocrit as reference.

**Results or Findings:** In the derivation cohort (49 men, mean age 79±8 years, mean BMI 26±5 kg/m2), correlation between BPmean and blood hematocrit ranged from poor for VNI of CCTA at 80keV, QIR2 ($R2=0.12$) to moderate for VNI of LE at 60keV, QIR4; 70keV, QIR3 and 4; and VNC of LE, QIR3 and 4 (all, $R2=0.58$). In the validation cohort (29 men, age 75±14 years, mean BMI 26±5 kg/m2), BPmean from VNC of the LE scan with QIR3 served to calculate the synthetic hematocrit. Median ECV was 26.9% (interquartile range (IQR), 25.5%,28.8%) using the blood hematocrit and 26.8% (IQR, 25.4%,29.7%) using the synthetic hematocrit (VNC, QIR3; mean difference,-0.2%; limits of agreement,-2.4%,2.0%; p=0.33).
**Conclusion:** Myocardial ECV calculation with PCD-CT, using a synthetic hematocrit derived from VNC images, enables accurate ECV measurements.
**Limitations:** Limited number of patients and lack of comparison with the clinical reference standard cardiac MRI
**Funding for this study:** None
**Ethics committee - additional information:** Kantonale Ethikkommission Zürich
**Author Disclosures:**
Victor Mergen: Research/Grant Support: institutional grants from Bayer, Canon, Guerbet, and Siemens
Matthias Eberhard: Research/Grant Support: institutional grants from Bayer, Canon, Guerbet, and Siemens
Nicolas Ehrbar: Research/Grant Support: institutional grants from Bayer, Canon, Guerbet, and Siemens
Lukas Jakob Moser: Research/Grant Support: institutional grants from Bayer, Canon, Guerbet, and Siemens
Robert Manka: Research/Grant Support: institutional grants from Bayer, Canon, Guerbet, and Siemens
Hatem Alkadhi: Research/Grant Support: institutional grants from Bayer, Canon, Guerbet, and Siemens

**Quantification of extracellular volume (ECV) with Photon Counting CT to identify transthyretin-related cardiac amyloidosis**
A. Clemente, *A. Marcucci*, C. De Gori, M. Occhipinti, M. Muca, F. Pignatelli, D. Cioni, E. Neri; Pisa/IT

**Purpose or Learning Objective:** Cardiac amyloid deposition causes interstitial expansion, thereby increasing myocardial extracellular volume (ECV). The need for quantification of amyloid burden in transthyretin-related cardiac amyloidosis (TTR-CA) is currently met in part through semi-quantitative bone scintigraphy or with measurement of ECV through Cardiovascular Magnetic Resonance(CMR). Although Photon-counting CT (PCCT) provides comprehensive spectral data with every acquisition of the heart, to date few studies analysed ECV in cardiac amyloidosis by using it. We evaluated the accuracy of extracellular volume (ECV) quantification with PCCT in TTR-CA.
**Methods or Background:** We prospectively enrolled 15 patients referred to our centre for suspected cardiac amyloidosis and atypical symptoms who underwent a complete diagnostic work-out including bone scintigraphy and PCCT. Iodine maps were created by using multienergy late scan. Volumetric ROIs of at least 2 cm3 were manually positioned using a 17 segments model of the left ventricle and global ECV was calculated as average of all segments. Then, the study population was divided into TTR-CA cases and non-amyloid heart disease cases (NCA).
**Results or Findings:** One patient was excluded due to light chain (AL) amyloidosis. Among the 14 patients included (77 years±5;10 men) 7 were diagnosed with TTR-CA. All TTR-CA cases showed positive scintigraphy (Perugini score ≥2), except for one, where the diagnosis was made through fat tissue biopsy and consistent cardiac CMR findings. Global ECV was significantly higher in patients with TTR-CA (40.50±8.97%) than in NCA patients (27.00±3.03%;$P<0.01$). The accuracy of myocardial global ECV to identify occult TTR-CA was high (AUC=0.87; 95%CI, 0.65-1.00).
**Conclusion:** Preliminary results on myocardial tissue characterization based on ECV quantification with PCCT iodine maps show this is a promising method to detect TTR-CA.
**Limitations:** Preliminary results with small sample.
**Funding for this study:** No funding received for this study.
**Ethics committee - additional information:** No
**Author Disclosures:**
Francesca Pignatelli: Nothing to disclose
Mariaelena Occhipinti: Nothing to disclose
Emanuele Neri: Nothing to disclose
Matilda Muca: Nothing to disclose
Alessandro Marcucci: Nothing to disclose
Alberto Clemente: Investigator: study MYOAMY-CT
Carmelo De Gori: Nothing to disclose
Dania Cioni: Nothing to disclose

**Accuracy of Iodine Maps from Photon-Counting Detector CT for Detecting Myocardial Late Enhancement – A Comparison to LGE-MRI**
G. Tremamunno[1], A. Varga-Szemes[2], D. Kravchenko[3], A. Laghi[1], F. Bamberg[4], M. Vecsey-Nagy[5], T. S. Emrich[6], *M. T. Hagar*[4]; [1]Rome/IT, [2]Charleston, SC/US, [3]Bonn/DE, [4]Freiburg im Breisgau/DE, [5]Budapest/HU, [6]Mainz/DE
*(taha.hagar@uniklinik-freiburg.de)*

**Purpose or Learning Objective:** To assess the feasibility and determine the diagnostic accuracy of iodine maps from photon-counting detector (PCD) CT in detecting and characterizing myocardial late enhancement (LE), compared to late gadolinium enhancement (LGE) MRI.
**Methods or Background:** This IRB-approved retrospective analysis of a prospective study cohort included subjects who underwent cardiac MRI followed by late iodine enhancement (CT-LE) using a PCD-CT system (NAEOTOM Alpha, Siemens Healthineers). CT-LE scans were performed 5 minutes after administering 100 mL of contrast media (Ultravist, 370 mg I/mL; Bayer Healthcare) with an ECG-triggered sequential protocol, and full spectral capabilities. Iodine maps were reconstructed at a section thickness of 1.0 mm, using a quantitative kernel (Qr40), and iterative reconstruction (QIR level 3). Two blinded and independent radiologists interpreted all images. Diagnostic accuracy was evaluated per-patient and per-segment using LGE-MRI as the standard of reference. Inter-reader agreement was assessed with Cohen's kappa.
**Results or Findings:** The final cohort comprised 27 patients (52% female, mean age 52.9 ± 17.2 years). Twelve patients (44%) had positive LGE on MRI (3 ischemic, 9 non-ischemic). Per-patient sensitivity was 100% and 91.7%, with specificities of 73.3% and 80.0%, and accuracies of 85.2%, respectively for both readers. Per-segment sensitivity was 74.7% and 66.7%, with specificities of 94.9% and 96.4%, and accuracies of 91.1% and 90.7%. Cohen's kappa was 0.70 (patient level) and 0.63 (segment level), respectively.
**Conclusion:** Iodine maps from PCD-CT provide high diagnostic accuracy for myocardial LE detection with substantial inter-reader agreement. PCD-CT may serve as an alternative to LGE-MRI in selective cases, such as a contraindication to MRI or anxiety.
**Limitations:** Our study's limited sample size mandates confirmatory research. Our study design introduces a selection bias, so further studies are needed to evaluate the diagnostic role across diverse patient populations.
**Funding for this study:** This study was partially supported by an unrestricted research grant from Siemens Healthineers.
**Ethics committee - additional information:** The study protocol, compliant with the Health Insurance Portability and Accountability Act, received approval from the local institutional review board at the respective academic medical center, and all subjects provided written informed consent.
**Author Disclosures:**
Muhammad Taha Hagar: Speaker: Siemens Healthineers
Milán Vecsey-Nagy: Nothing to disclose
Giuseppe Tremamunno: Nothing to disclose
Fabian Bamberg: Nothing to disclose
Dmitrij Kravchenko: Nothing to disclose
Andrea Laghi: Nothing to disclose
Tilman Stephan Emrich: Nothing to disclose
Akos Varga-Szemes: Research/Grant Support: Siemens Healthineers

**Ultra-low dose (0.4 mSv) coronary computed tomography angiography using photon-counting detector computed tomography**
*S. Araki*, S. Nakamura, M. Takafuji, Y. Ichikawa, H. Sakuma, K. Kitagawa; Tsu/JP
*(s-araki@med.mie-u.ac.jp)*

**Purpose or Learning Objective:** Photon-counting detector computed tomography (PCD-CT), which allows for the exclusion of electronic noise, shows promise for significant dose reduction in coronary computed tomography angiography (CCTA). This study aimed to assess the radiation dose and image quality of CCTA using PCD-CT, combined with electrocardiogram (ECG)-triggered prospective high-pitch helical scanning and an ultra-low tube potential of 70 kVp and to investigate the effect of a sharp kernel on image quality and stenosis assessment in such an ultra-low dose CCTA setting.
**Methods or Background:** Forty patients (65% male) with stable heart rates and no prior coronary interventions were included. Data on CT dose index volume (CTDIvol) and dose-length product (DLP) were collected, with effective radiation dose estimated using a conversion factor of 0.014. Images were reconstructed using kernels of Bv64 and Bv40 for image quality and stenosis assessment.

**Results or Findings:** The mean CTDIvol, DLP, and effective dose of CCTA were 1.72±0.38 mGy, 29.1±6.8 mGy·cm, and 0.41±0.09 mSv, respectively. Image quality was similar (p=0.75) between the two kernels, with over 95% of segments achieving a rating of good image quality for both kernels. Additionally, 42% of non-calcified plaques showed an increased stenosis severity from Bv40 to Bv64 (p<0.001), while 25% of calcified plaques exhibited a decreased severity (p<0.001).
**Conclusion:** PCD-CT technology with ECG-triggered prospective high-pitch helical scanning and the tube potential of 70kVp can provide CCTA with ultra-low radiation exposure (0.4 mSv), offering a safer and more effective method for diagnosing coronary artery disease. The noise reduction capability of PCD-CT allows the use of a sharp kernel even in this low-dose CCTA setting without compromising image quality, potentially improving the evaluation of coronary artery stenosis.
**Limitations:** There is no reference standard such as coronary angiography.
**Funding for this study:** No funding was provided for this study
**Ethics committee - additional information:** Clinical Research Ethics Review Committee of Mie University Hospital (approval No. H2019-207)
**Author Disclosures:**
Kakuya Kitagawa: Nothing to disclose
Hajime Sakuma: Nothing to disclose
Suguru Araki: Nothing to disclose
Yasutaka Ichikawa: Nothing to disclose
Masafumi Takafuji: Nothing to disclose
Satoshi Nakamura: Nothing to disclose

**Benefits of photon counting CT for the assessment of native heart valves**
C. Mayard, S. A. Si-Mohamed, A. Houmeau, L. Boussel, P. C. Douek, *S. Boccalini*; Lyon/FR
*(sara.boccalini@yahoo.com)*

**Purpose or Learning Objective:** To assess the benefits of photon counting CT (PCCT) on image quality of cardiac valves as compared to conventional CT (conv-CT).
**Methods or Background:** Patients were prospectively included to undergo a clinically indicated coronary CT angiography with PCCT and conv-CT within three days. All the components of each valve were subjectively scored by two observers with a 4-point scale for sharpness and conspicuity. The number of nodules and of mitral chordae was noted. The number and the localisation of the calcifications relative to the thickness of the aortic leaflets were assessed. Furthermore, the full width at mid weight (FWMH) of the attenuation profile of a line perpendicular to the commissure between the left and right coronary cusps of the aortic valve was calculated.
**Results or Findings:** Thirty-three patients were included (88% men; 62±13 years). 33 pairs of aortic and mitral valves and 18 pairs of pulmonary valves could be analysed. Conspicuity of aortic, mitral, and pulmonary valvular structures was increased with PCCT except for one commissure of the aortic and pulmonary valves (p=0.06 and p=0.07). Sharpness was superior for all structures of the aortic and mitral valves, and for 2/3 edges of the pulmonary valve. More fine structures (nodules, chordae) and calcifications of the aortic and mitral valves were visible with PCCT. The precise localisation of the calcifications could be assessed with PCCT in most cases while it remained doubtful in many cases with conv-CT (p=0.02). FWMH was lower with PCCT (1.7(IQ=1.1) vs 2.5mm (IQ=1.3); p<0.01). The radiation dose was lower with PCCT (567.8 ±67.7 vs 681.8 ±159.6 mGy*cm; p<0.01).
**Conclusion:** PCCT yielded better objective and subjective image quality of cardiac valves as compared to conv-CT and more components of the valve structures were visible.
**Limitations:** Small cohort
**Funding for this study:** European grant H2020
**Ethics committee - additional information:** Approved
**Author Disclosures:**
Salim Aymeric Si-Mohamed: Speaker: Philips
Angele Houmeau: Nothing to disclose
Philippe Charles Douek: Speaker: Philips
Loïc Boussel: Speaker: Philips
Sara Boccalini: Speaker: Philips
Charles Mayard: Nothing to disclose

**14:00-15:30 Research Stage 2**

### Research Presentation Session: Musculoskeletal

### RPS 2010

### Imaging of injuries and instabilities of the peripheral joints

**Moderator**
A. J. Shah; Ahmedabad/IN
*(drankur203@gmail.com)*

**MRI findings of shoulder injury related to vaccine administration (SIRVA) following COVID-19 vaccination: A cross-sectional study**
*N. M. I. Obeidat*[1], R. Khasawneh[1], S. Bani Essa[1], M. Alkhatatba[1], A. Abdel Kareem[1], M. Al-Na'Asan[1], Y. Alshgerat[1], M. Aljarrah[1], L. Sawalha[2]; [1]Irbid/JO, [2]Amman/JO
*(nmobeidat8@just.edu.jo)*

**Purpose or Learning Objective:** To investigate the MRI findings of patients presenting with SIRVA after COVID-19 vaccination and to assess the associations between these findings and patient demographics, clinical symptoms, and vaccine-related factors.
**Methods or Background:** A retrospective cross-sectional study involved patients who reported shoulder disorders following COVID-19 vaccination between 1 May 2021 and 1 May 2022. Data collected included demographics, clinical symptoms, vaccination details, and MRI findings. Statistical analyses assessed associations between MRI findings and patient demographics, clinical symptoms, and vaccine-related factors. Chi-square tests and t-tests were utilized, with statistical significance set at p < 0.05 and trends noted at p < 0.1.
**Results or Findings:** 35 patients were diagnosed with SIRVA (3 had bilateral involvement; mean age 53.6 ± 9.0 years; 54.3% females). The majority developed symptoms within 24 hours post-vaccination (88%), most of them resolved within a week (84%). Pain was the predominant symptom (51.4%). MRI findings were subacromial bursitis (89.5%), enthesial erosions (63.2%), tendinopathy (47.4%), rotator cuff tears and changes of adhesive capsulitis (each 36.8%), effusion (23.7%), as well as muscle and bone marrow edema (10.5% and 7.9%, respectively). Statistically significant associations were found between tendinopathy and both, older age (p=0.024) and AstraZeneca vaccine (p=0.033), subacromial bursitis with both female gender (p=0.013) and higher BMI (p=0.023), and between changes of adhesive capsulitis and Sinopharm vaccine (p=0.029). Non-diabetics, females, and patients with tendinopathy, were more likely to have persistent symptoms after 3 years following vaccination.
**Conclusion:** SIRVA following COVID-19 vaccination results in different shoulder pathologies apparent on MRI, many of which are strongly related to patient demographics and type of vaccine administered. Awareness of SIRVA changes among radiologists is paramount, especially in seasons of vaccination campaigns such as in early winter (flu-vaccines).
**Limitations:** Retrospective study and small patient sample.
**Funding for this study:** No funding was received for this study.
**Ethics committee - additional information:** Retrospective study.
**Author Disclosures:**
Leen Sawalha: Nothing to disclose
Mohammad Al-Na'Asan: Nothing to disclose
Mohammad Alkhatatba: Nothing to disclose
Suhaib Bani Essa: Nothing to disclose
Naser Mohammad Issa Obeidat: Nothing to disclose
Majed Aljarrah: Nothing to disclose
Ruba Khasawneh: Nothing to disclose
Ali Abdel Kareem: Nothing to disclose
Yahya Alshgerat: Nothing to disclose

**Low-dose four-dimensional ct in diagnosing wrist instability**
*I. Blom*[1], N. Mathijssen[2], G. Kraan[2]; [1]Delft/NL, [2]Zoetermeer/NL
*(i.blom@rdgg.nl)*

**Purpose or Learning Objective:** To assess the ability to lower the radiation dose in four-dimensional computed tomography (4DCT) for assessment of scapholunate (SL) instability without compromising diagnostic quality.

**Methods or Background:** Scapholunate (SL) instability can be diagnosed using 4DCT. However, CT comes with increased radiation exposure compared to other diagnostic imaging tools. Therefore, six randomly chosen cadaveric human hand specimens were scanned using an automatic device simulating radial-ulnar deviation. Parameters affecting radiation dose—scanning time, scan range, tube current (mAs), and tube voltage (kVp)—were varied and compared to clinical settings. Effective dose (ED) and image noise were assessed for all performed scans. Image noise was measured in soft tissue and cortical bone, since cortical bone was used for segmentation. Three medical specialists analyzed image quality and diagnostic value using a 5-point Likert scale.
**Results or Findings:** The ED was 0.081 mSv under standard clinical settings. Reducing the scan range to 80 mm (including all carpal bones) decreased the ED to 0.038 mSv. Further reduction to 0.004 mSv was achieved by shortening the scanning time and lowering the tube current. Lowering tube current increased image noise in soft tissue, but reduced noise in cortical bone. Adjusting scanning time and scan range did not affect image noise. Image quality was deemed diagnostically acceptable for low dose 4DCT by all observers, even 22 percent of the scans were deemed as excellent image quality.
**Conclusion:** A low-dose 4DCT protocol for SL instability seems feasible without compromising diagnostic image quality compared to other imaging tools. Further research is needed to explore low-dose 4DCT for other indications.
**Limitations:** The limitations of the study are a human cadaveric study and a questionaire based on 1 question.
**Funding for this study:** No fundings were received for this study.
**Ethics committee - additional information:** None.
**Author Disclosures:**
Nina Mathijssen: Nothing to disclose
Gerald Kraan: Nothing to disclose
Ian Blom: Nothing to disclose

**Enhancing hip replacement assessment: Integrating iterative metal artefact reduction (iMAR) algorithm with cinematic volume rendering technique (cVRT) in photon-counting CT**
*X. Liu*; Zhengzhou, Henan Province, China/CN
*(278459366@qq.com)*

**Purpose or Learning Objective:** To explore the potential of combining the iterative metal artifact reduction (iMAR) algorithm with cinematic volume rendering technique (cVRT) in photon-counting CT for assessing hip replacements.
**Methods or Background:** A retrospective study was conducted on 120 patients who underwent hip arthroplasty exams using the photon-counting CT scanner (NAEOTOM Alpha). Reconstruction of CT images employing conventional methods, volume rendering (VR), and cVRT, both with and without iMAR. Measurements of CT numbers and standard deviations (SDs) in regions of interest (ROIs) were obtained. Objective image quality and subjective scores were assessed using established scales. Statistical analyses included paired T tests, Mann-Whitney U tests, and Kappa tests.
**Results or Findings:** Compared with the non-iMAR group, the iMAR group showed significantly decreased and increased CT numbers in hyperattenuating and hypoattenuating areas, respectively, as well as lowered artifact and image noise ($p<.001$). Qualitatively, the iMAR group showed superiority to the non-iMAR group in both image quality and diagnostic confidence, with scores increases of 2.70 and 2.88 points, respectively ($p<0.05$). iMAR combined with cVRT received the highest subjective score ($p<0.05$) among the four series of post-processing images, followed by iMAR with VR images($P<0.05$), cVRT and VR images in the non-iMAR group both received the lowest scores.
**Conclusion:** The iMAR algorithm in photon-counting CT effectively reduces artifacts and image noise, enhancing both image quality and diagnostic confidence in post-hip metal replacement assessments. When combined with cVRT, it provides a more intuitive visualization of metal implant stability and the relationship between implants and adjacent tissues.
**Limitations:** Not applicable.
**Funding for this study:** No funding was received for this study.
**Ethics committee - additional information:** The ethics committee notification can be found under the number 2021-KS-HNSR115
**Author Disclosures:**
Xing Liu: Nothing to disclose

**Complications of hip prostheses**
*S. L. Chung*[1], M. S. Sait[2]; [1]Oxford/UK, [2]Kings University Hospital/UK
*(auntminnie23@gmail.com)*

**Purpose or Learning Objective:** To familiarise the different types of hip prostheses. To be able to interpret normal imaging findings post surgery in mainstay modalities such as radiograph, ultrasound, CT and MRI. To understand the limitations of each imaging modalities and when to consider further imaging such as SPECT-CT scans or PET/CT. To be aware of the possible false positive findings such as positive tracer uptake in nuclear scans within 1 year post operation is still within normal physiological limits. To increase awareness the numerous types of complications from most common to least and the time intervals it occurs.
**Methods or Background:** Review of current literature of optimisation of imaging modalities to reduce artefact caused by hip prostheses enabling better assessment of the joint.
**Results or Findings:** Slice-encoding for metal artefact correction (SEMAC)-is a relatively new MRI sequence particularly STIR and T1-weighted SEMAC sequences help reduce artefacts caused by hip prosthesis best and can help exclude aseptic loosening. Understand the scopes of SPECT-CT, PET/CT and MRI including when to use adjunct imaging to diagnose.
**Conclusion:** MRI is the best imaging modality for reviewing hip prosthesis but accessibility is dependent on institution. MR SEMAC protocol should be routinely applied to reduce artefacts and increasing diagnostic assessment.
**Limitations:** Limited articles on updated nuclear studies/protocols.
**Funding for this study:** Nil
**Ethics committee - additional information:** Not Applicable
**Author Disclosures:**
Mohammed Saif Sait: Nothing to disclose
Siok Li Chung: Nothing to disclose

**How routine day-one radiographs affect patient management after hip and knee arthroplasty or internal fixation**
*A. Jonkergouw*, P. Tukker, W. De Monye; Haarlem/NL

**Purpose or Learning Objective:** This study aims to evaluate the clinical consequences of radiographs performed routinely on the first day after arthroplasty or internal fixation of the hip and knee.
**Methods or Background:** We conducted a retrospective search of our imaging database for day-one post-operative radiographs after total hip arthroplasty, hip hemi-arthroplasty, dynamic hip screw fixation, gamma nail fixation, total knee arthroplasty, and unicompartmental knee arthroplasty, starting from 1 January 2023 until 500 radiographs for hip surgeries and 500 for knee surgeries were included. For each case, the radiological report was reviewed to determine if any immediate post-operative abnormalities were detected. Additionally, we recorded the type of prosthesis, the patient's age and sex, and information on excessive pain from patient records.
**Results or Findings:** Of the 500 patients who underwent hip surgery, 388 (77.6%) received total hip arthroplasty, 27 (5.4%) hemi-arthroplasty, 34 (6.8%) dynamic hip screw fixation, and 51 (10.2%) gamma nail fixation. Of the 500 patients who underwent knee surgery, 420 (84%) received total knee arthroplasty, and 80 (16%) unicompartmental knee arthroplasty. Across the entire cohort, post-operative abnormalities were noted in 33 patients (3.3%). Five patients (0.5%) required additional medical care due to a fracture, all of whom had undergone hip surgery. Three (0.3%) underwent revision surgery, and 2 (0.2%) received weight-bearing restrictions. Of the 5 patients with a fracture, 4 (80%) reported significant pain prior to undergoing radiological examination. Gender did not influence the occurrence of post-operative abnormalities.
**Conclusion:** Given that only 0.5% of all patients required additional medical care after detection of post-operative abnormalities, the necessity for routine post-operative radiographs appears limited.
**Limitations:** Based on the available data, no definite predictive factor has been identified in relation to radiological abnormalities after hip or knee surgery.
**Funding for this study:** None
**Ethics committee - additional information:** The ACLU gave permission for this study. This committee advises the Board of Directors on new scientific studies at Spaarne Gasthuis.
**Author Disclosures:**
Anne Jonkergouw: Nothing to disclose
Wouter De Monye: Nothing to disclose
Paul Tukker: Nothing to disclose

**Increased Lateral Tibial Plateau Slope (LTPS) and extreme Intercondylar Notch Slope (INS): parameters to predict Anterior Cruciate Ligament (ACL) High-grade Injury (HgI) and Mucoid Degeneration (MD)?**
*A. Cutaia*, R. Faletti, P. Fonio, E. La Paglia; Turin/IT
*(aldo.cutaia@unito.it)*

**Purpose or Learning Objective:** To assess the reliability of LTPS and INS to predict ACL HgI and MD.
**Methods or Background:** 110 patients (Mean Age 46,1years) with MR evidence of pathological ACL were retrospectively selected. Three groups: HgI (Mean age 31,58years), Low-grade injury (LgI) (Mean age 45,85years) and MD (Mean age 59,33years). LTPS was measured on sagittal T1-TSE images: Firstly selecting a slice comprehending tibial attachment of posterior cruciate ligament and intercondylar eminence, tracking the tibial axis with craniocaudal circles method. Secondly, in a slice immediately medial to the head of the fibula, drawing a line perpendicular to the tibial axis. Thirdly, measuring the angle between this line and the one parallel to the posterior tibial plateau. LTPS was reported as increased when greater than 10°. INS was measured drawing the Blumensaat Line, then evaluating in which section of the tibial plateau it landed: anterior third type 1, middle third type 2, posterior third type 3. INS was extreme when classified as group 2 or 3. Statistical significance was measured using Z and T test for quantitative variables and ChiSquare for qualitative variables.
**Results or Findings:** General Group (GG) mean LTPS was 6,87° +-3,09. Patients with an increased LTPS (Mean LTPS 10,69° +-0,73) showed significantly higher prevalence (66% vs.36%) of ACL HgI compared to GG ($p<0.005$). HgI group's mean LTPS was 7,83° +-3,02, significantly higher than LgI group ($p<0.025$). MD group showed significantly higher prevalence (55% vs.27%) of extreme INS compared to HgI ($p<0.025$).
**Conclusion:** Increased LTPS should be added in MR reports as it could highlight a predisposition to ACL HgI. INS should be assessed as it could play a pivotal role in the onset of ACL MD on the basis of chronic impingement.
**Limitations:** The study is retrospective and monocentric.
**Funding for this study:** None.
**Ethics committee - additional information:** None.
**Author Disclosures:**
Aldo Cutaia: Nothing to disclose
Riccardo Faletti: Nothing to disclose
Ernesto La Paglia: Nothing to disclose
Paolo Fonio: Nothing to disclose

### Is It Necessary To Add Soft Tissue Injury to the Classification in Tibial Plateau Fracture Management?

M. Tunçez[1], I. Akan[1], F. Seyfettinoğlu[2], *H. Çetin Tunçez*[1], B. Dirim Mete[1], C. Kazımoğlu[1]; [1]Izmir/TR, [2]Adana/TR
*(drhulyacetintuncez@gmail.com)*

**Purpose or Learning Objective:** Current classification systems have generally been developed based on the type of fracture patterns. The most commonly used is the Schatzker classification system, which includes six types of tibial plateau fractures. While this classification evaluates the fracture in two dimensions, three-dimensional classifications were introduced after the spread of CT. To our knowledge, the classification system regarding soft tissue injuries based on MRI findings has not been established yet. Therefore, in this study, we aimed to evaluate the usability of a new modified classification by evaluating soft tissue injuries in tibial plateau fractures.
**Methods or Background:** A total of 36 patients with tibial plateau fractures were included in the study. Patients' age, gender, and affected sides were recorded. Injuries to the medial meniscus, lateral meniscus, anterior cruciate ligament, posterior cruciate ligament, medial collateral ligament, and lateral collateral ligament were examined with preoperative magnetic resonance imaging. Soft tissue injuries were arranged according to the novel modified classification based on the Schatzker classification.
**Results or Findings:** The mean age of the study participants was 45 (19-76) years; 72% of the patients were men and 28% were women. Moreover, 44% and 56% of the patients had broken the right and left tibial plateaus, respectively. At least one soft tissue injury was detected in 29 (81%) patients. In 14 (39%) patients, two or more soft tissue injuries were observed. All patients were arranged according to the novel modified classification regarding ligament and meniscus injuries.
**Conclusion:** With this novel modified classification system, we think that having better information about the preoperative condition of the soft tissue injuries can change the surgical strategy in patients with tibial plateau fractures.
**Limitations:** Patients with minimal displacement and those requiring conservative treatment were excluded.
**Funding for this study:** No funding was received for this study.
**Ethics committee - additional information:** This retrospective study was approved by the Institutional Review Board of our hospital.
**Author Disclosures:**
Hülya Çetin Tunçez: Author: Data collection Author: STUDY DESIGN
Cemal Kazımoğlu: Author: literature search
Ihsan Akan: Author: data collection
Mahmut Tunçez: Author: Study Design
Berna Dirim Mete: Author: data collection
Fırat Seyfettinoğlu: Author: literature search

### Validation of mRUST as an endpoint for fracture healing studies in lower-limb fractures

D. Deppe[1], *M. Gabriele*[1], E. K. Simşek[2], A. Ordas-Bayon[3], M. Leskovar[1], A. Trepczynski[1], S. Zachow[1], G. Duda[1], M. Heyland[1]; [1]Berlin/DE, [2]Ankara/TR, [3]Madrid/ES
*(matteo.gabriele2@studio.unibo.it)*

**Purpose or Learning Objective:** Determining the best treatment for lower limb fractures is challenging due to multiple factors affecting bone healing, requiring precise monitoring for optimal care. A radiographic assessment of the end point of bone healing is difficult, prompting the use of scores such as the modified Radiographic Union Score for Tibial fractures (mRUST) that represents one of the most used scores to evaluate radiographic fracture healing. However, the use of mRUST across different locations and treatment options has not yet been thoroughly demonstrated. The objective of this study is to validate the robustness of mRUST as a reliable measure during follow-up in lower limb fractures using various treatment modalities.
**Methods or Background:** Six international investigators (five orthopaedic surgeons and one radiologist) independently assessed the mRUST in 166 patients with extra-articular lower-limb fractures for different follow-up timepoints. Inter-rater reliability was assessed for location (femur/ tibia), treatment option (plate fixation/ nail fixation) and for different treatment options in different fracture locations using intraclass correlation coefficients (ICC).
**Results or Findings:** 166 patients (63 femur fractures, 103 tibia fractures; 32.52% female) with a total of 1136 follow up time points were included. Overall inter-rater reliability for mRUST was good (0.77), regardless of fixation method (0.79, for both nail and plate fixation) or anatomical location (0.78 in tibia fractures, 0.81 in femur fractures). On cortex level, reliability varied for different location within in the bone with highest inter-rater agreement for the medial cortex (0.70-0.74) and lowest for the posterior cortex (0.65-0.74)
**Conclusion:** The mRUST-Score proves to be a robust scoring tool for healing monitoring in lower-limb fractures treated with different fixation methods in different parts of the bone.
**Limitations:** Images were presented in chronological order, which limited the results of this study.
**Funding for this study:** None.
**Ethics committee - additional information:** Local ethics committee approval was granted for this retrospective study (EA4/099/22).
**Author Disclosures:**
Georg Duda: Nothing to disclose
Alejandro Ordas-Bayon: Nothing to disclose
Stefan Zachow: Nothing to disclose
Dominik Deppe: Nothing to disclose
Mark Heyland: Nothing to disclose
Matteo Gabriele: Nothing to disclose
Marko Leskovar: Nothing to disclose
Ekin Kaya Simşek: Nothing to disclose
Adam Trepczynski: Nothing to disclose

### Correlation of Osteochondral Lesions of the Talar Dome with Tears of the Superior and Inferior Bands of the Anterior Talofibular Ligament and the Calcaneofibular Ligament: A Retrospective Study

*S. Rajan*, J. S. Chatha, H. Mahajan; New Delhi/IN
*(drsriramrajan@gmail.com)*

**Purpose or Learning Objective:** Osteochondral lesions of the talar dome (OLT) can result from recurrent ankle microinstability, traumatic events, or anatomical abnormalities. This study aims to evaluate the correlation between OLT and single, double, or triple ligament tears within the lateral collateral ligament complex.
**Methods or Background:** A retrospective review was conducted on 212 consecutive ankle MRIs performed for pain or instability, sourced from the PACS server. Examination under anesthesia and arthroscopic confirmation were obtained in 23 cases. The data were anonymized and analyzed by two radiologists with extensive experience. The status of the superior and inferior ATFL bands, the calcaneofibular ligament (CFL), and the superficial and deep deltoid ligaments were assessed. Osteochondral lesions were classified using the Anderson classification. Statistical significance was evaluated using the Chi-square test to assess the relationship between ligament tears and OLT.
**Results or Findings:** OLT was identified in 74 patients (36.6%), while 126 patients (62.4%) had no OLT. Among those with OLT, 72 had lateral collateral ligament pathology, with only 2 cases showing no definitive tears. Of the 74 patients with OLT, 26 had tears of the superior ATFL with inferior band scarring, 36 had tears of both ATFL bands, and 19 had tears of both ATFL bands and the CFL. Statistical analysis revealed a significant correlation ($p < 0.01$) between the presence of ligament tears and the occurrence of OLT.

**Conclusion:** This study highlights a significant correlation between osteochondral lesions of the talar dome and lateral ligament tears, emphasizing the need for comprehensive assessment of ligament integrity in patients with OLT.
**Limitations:** Arthroscopic proof of ligament tears was not present in all cases
**Funding for this study:** None
**Ethics committee - additional information:** Retrospective study
**Author Disclosures:**
Jagneet Singh Chatha: Nothing to disclose
Sriram Rajan: Nothing to disclose
Harsh Mahajan: Nothing to disclose

### Evaluating peroneus tendon motion using dynamic magnetic resonance imaging – a pilot study

K. Bokwa, D. G. Mocanu, N. Solidakis, *P. Szaro*; Gothenburg/SE
*(pawel.szaro@vgregion.se)*

**Purpose or Learning Objective:** Previous studies indicate that peroneus brevis instability and split tears may be missed in up to half of patients clinically due to unclear clinical signs and on conventional magnetic resonance (MRI) because of its static nature. We hypothesize that dynamic imaging may improve the diagnosis of peroneus brevis instability and split tears. However, no studies have evaluated whether dynamic MRI can visualize peroneus tendon motion. The aim of this study is to assess whether dynamic MRI can be used to evaluate the motion of the peroneal tendons.
**Methods or Background:** Study design: observational pilot study. We performed dynamic MRI using two small flexible coils in a 3T machine, assessing the axial plane at the lateral malleolus with Dynamic Balanced Fast Field Echo (BFFE). Ten participants received movement training from a radiology nurse before imaging. Two radiologists (raters) evaluated the image quality. We included only examinations with clear tendon outlines, visible motion without artifacts. Raters assessed the position of peroneus brevis relative to peroneus longus in neutral, plantar, and dorsal flexion, reaching a consensus. Radiologists measured the distance between the tendons' central points in each position, reporting the mean values.
**Results or Findings:** All examinations were included in the analysis. Preliminary analysis revealed that dynamic MRI allows visualisation of peroneus tendons motion in good quality. The mean distance between the tendons was 2.1 mm (SD 0.3 mm) in the neutral position, 4.8 mm (SD 0.3 mm) in dorsal flexion, and 2.0 mm (SD 0.2 mm) in plantar flexion.
**Conclusion:** Dynamic magnetic resonance allows the evaluation of peroneus tendon motion, offering a novel approach for evaluating stability that may improve the accuracy of peroneus diagnostics.
**Limitations:** Small sample size, only dorsal and plantar flexion in the ankle.
**Funding for this study:** The study was founded by Stiftelsen Tornspiran 934: 2023-12-01.
**Ethics committee - additional information:** The Swedish Ethical Review Authority approved the study: 2023-07231-01.
**Author Disclosures:**
Dan Gheorghe Mocanu: Nothing to disclose
Nektarios Solidakis: Nothing to disclose
Katarzyna Bokwa: Nothing to disclose
Pawel Szaro: Nothing to disclose

### Top five MRI findings of professional soccer players in pre-season medical examinations

E. Höhne, I. Yel, A. Gökduman, S. Bernatz, *M. Dimitrova*, C. Booz, T. Vogl, S. Mahmoudi; Frankfurt/DE

**Purpose or Learning Objective:** In professional soccer extensive musculoskeletal assessments are conducted prior to player transfers to evaluate the current state and future risk of injury. Magnetic resonance imaging (MRI) is essential in this process revealing musculoskeletal findings even in the absence of symptoms. This analysis presents the five most frequent MRI findings in a cohort of professional soccer players and aims to improve understanding of the physical condition of elite athletes.
**Methods or Background:** This retrospective study included comprehensive musculoskeletal 3T MRI scans obtained during medical checks of professional soccer players from August 2019 to September 2024. Clinical data were extracted from medical records and supplemented with further functional information.
**Results or Findings:** MRI scans of 44 professional soccer players were analysed. Among the players, five were left-footed, one was two-footed, and the remaining players were right-footed. The average age at the time of examination was 22.8 years (± 4,3). The most common finding was a secondary cleft on the left side, observed in 18 players (40.9%). A bulging or protruding disc at the L5/S1 level was found in 31.8% of the cases. Degenerative changes in the labrum were identified in 27.3% of players on the left side and in another 27.3% on the right side. Chondropathy of the left knee was present in 27.3% of cases.
**Conclusion:** This retrospective analysis revealed several notable findings, particularly given the young average age of the athletes. The main findings included a secondary cleft and labral degeneration, which may be associated with the common occurence of groin pain in soccer players. A better understanding of these associations could enhance the development of more effective diagnostic and preventive strategies for musculoskeletal injuries.
**Limitations:** The limited sample size restricts the generalizability of findings.
**Funding for this study:** None.
**Ethics committee - additional information:** The local ethics committee has approved this retrospective study.
**Author Disclosures:**
Christian Booz: Nothing to disclose
Ibrahim Yel: Nothing to disclose
Mirela Dimitrova: Nothing to disclose
Thomas Vogl: Nothing to disclose
Scherwin Mahmoudi: Nothing to disclose
Aynur Gökduman: Nothing to disclose
Simon Bernatz: Nothing to disclose
Elena Höhne: Nothing to disclose

**14:00-15:30 Research Stage 3**

### Research Presentation Session: Hybrid, Molecular and Translational Imaging

### RPS 2006

### Hybrid and molecular imaging in oncology: clinical and translational studies

**Moderator**
P. M. Kazmierczak; Munich/DE

### Ga-68-FAPI PET/CT in Malignant Mesothelioma: Prospective Single-Center Observational Trial

*L. Kessler*, B. M. Schaarschmidt, J. Siveke, L. Umutlu, M. Schuler, M. Stuschke, K. Herrmann, W. Fendler, H. Hautzel; Essen/DE
*(lukas.kessler@uk-essen.de)*

**Purpose or Learning Objective:** Mesothelioma are rare tumors mostly affecting the pleura and are associated with overall poor prognosis. Mesothelioma subtypes have shown to express fibroblast-activation-protein (FAP) in tumor cells, suggesting FAP as a promising target for imaging and therapy. Thus, novel radiolabeled FAP-inhibitors (FAPI) are of interest for future theranostic approaches. The FAPI-PET observational trial (NCT04571086) evaluates Ga-68-FAPI PET imaging in cancer patients and here we present data on Ga-68-FAPI in patients with mesothelioma.
**Methods or Background:** Forty-one patients underwent Ga-68 FAPI-PET imaging and F-18-FDG PET. The primary endpoint was correlation of Ga-68-FAPI-PET uptake (SUVmax and SUVpeak) with histopathological FAP expression. Secondary objectives included detection rate and diagnostic performance (sensitivity, specificity, positive/negative predictive values and accuracy) compared to F-18-FDG PET validated by histopathology or a compound reference standard (histopathology, alternative imaging or follow-up imaging).
**Results or Findings:** SUVmax and SUVpeak values showed a significant correlation with histopathological FAP expression (SUVmax $r = 0.49$, $p = 0.04$; SUVpeak $r = 0.51$, $p = 0.03$). Overall Ga-68-FAPI showed high diagnostic performance (SE 98%, SP 81%, PPV 88% and NPV 97%). Ga-68-FAPI had similiar sensivity compared to F-18-FDG on both per-patient (100.0% vs. 97.3%) and per-region (98.0% vs. 95.9%) basis but showed increased Specificity (81.1% vs. 36.8%) and positive predictive value (87.5% vs. 66.2%) in per-region analysis, indicating superior performance. This discrepancy was attributed to a higher number of false positive regions on F-18-FDG (FAPI, N = 7 vs. FDG, N = 31).
**Conclusion:** This is the first study to show correlation between Ga-68-FAPI uptake and histopathological FAP expression and superior diagnostic value compared to F-18-FDG in mesothelioma patients. These findings highlight the potential of Ga-68-FAPI as a potential tool in clinical practice.
**Limitations:** The limitations are single center, observational cohort with heterogenous patients.
**Funding for this study:** No funding was received for this study.
**Ethics committee - additional information:** University Duisburg-Essen permits 19-8991-BO and 20-9485-BO

**Author Disclosures:**
Ken Herrmann: Consultant: Bayer, SOFIE Biosciences, SIRTEX, Adacap, Curium, Endocyte, BTG, IPSEN, Siemens Healthineers, GE Healthcare, Amgen, Novartis, ymabs, Aktis Oncology, Theragnostics, Pharma15, Debiopharm, AstraZeneca, Janssen.
Hubertus Hautzel: Consultant: Urenco Consultant: Roche
Wolfgang Fendler: Consultant: SOFIE Biosciences, Janssen (consultant, speaker), Calyx (consultant, image review), Bayer (consultant, speaker, research funding)
Martin Stuschke: Nothing to disclose
Benedikt Michael Schaarschmidt: Research/Grant Support: DFG
Jens Siveke: Consultant: AstraZeneca, Bayer, Boehringer Ingelheim, Bristol-Myers Squibb, Immunocore, MSD, Novartis, Roche/Genentech and Servier Board Member: Pharma15 Research/Grant Support: Abalos Therapeutics, Boehringer Ingelheim, Bristol-Myers Squibb, Celgene, Eisbach Bio, and Roche/Genentech
Lukas Kessler: Nothing to disclose
Lale Umutlu: Nothing to disclose
Martin Schuler: Nothing to disclose

**Diagnostic accuracy and molecular characterization of endometrial cancer using fully hybrid [18F]FDG PET/MRI**
*T. Russo*, C. Bezzi, C. Sabini, G. Candotti, G. Ironi, F. De Cobelli, P. Mapelli, A. Chiti, M. Picchio; Milan/IT
*(t.russo.1994@gmail.com)*

**Purpose or Learning Objective:** This study aims at evaluating the effectiveness of fully hybrid [18F]FDG PET/MRI in EC staging, assessing its diagnostic accuracy and prognostic role in predicting features of EC aggressiveness, including p53abn MMRd for the new molecular classification.
**Methods or Background:** This prospective study involved 80 patients with biopsy-confirmed EC who underwent preoperative [18F]FDG PET/MRI for staging purposes. The PET/MRI scans were independently reviewed by a radiologist and a nuclear medicine physician, assessing the diagnostic accuracy (ACC), sensitivity (SN), specificity (SP), and positive and negative predictive value (PPV, NPV). Imaging and clinical parameters were then investigated for their correlation (Spearman's rank correlation) and analyzed through Fisher's exact test, and ROC analysis. Kaplan-Meier survival curves, Log-rank tests and Cox proportional hazards models were used to evaluate the prognostic value of parameters for predicting tumor relapse.
**Results or Findings:** PET/MRI provided ACC=98.75%, SN=98.75%, and PPV=100% for primary tumor detection, and ACC = 92.31%, SN = 84.62%, SP = 93.85%, PPV = 73.33%, NPV = 96.83% for LN detection. PET/MR parameters were able to predict LVSI (AUC= 80.16%), deep MI, p53abn and MMRd (AUC>70%). Less accurate predictions were found for EC histotype (AUC=68.04%) and infiltration pattern (AUC=65.19%). Finally, quantitative parameters could also predict both disease relapse (AUC=81.63%), with MTV and Size_CC showing the highest prognostic value, and the need to administer post-operative adjuvant therapy (AUC=74.63%).
**Conclusion:** [18F]FDG PET/MRI show good accuracy in the staging of EC primary tumor and LN metastases. Moreover, PET and MRI-derived parameters have a potential role in the characterization tumor aggressiveness and molecular alterations, as well as tumor recurrence prediction, crucial information for an optimal patient treatment and management in clinical practice.
**Limitations:** Molecular characterization not available for all patients.
**Funding for this study:** None
**Ethics committee - additional information:** The study received approval from the Institution's Ethics Committee (protocol number 85/INT/2019) and informed consent was obtained from all patients in accordance with EC guidelines . All procedures were carried out in accordance with the Declaration of Helsinki (1964) and its later amendments.
**Author Disclosures:**
Maria Picchio: Nothing to disclose
Gabriele Ironi: Nothing to disclose
Carlotta Sabini: Nothing to disclose
Arturo Chiti: Nothing to disclose
Giorgio Candotti: Nothing to disclose
Carolina Bezzi: Nothing to disclose
Tommaso Russo: Nothing to disclose
Paola Mapelli: Nothing to disclose
Francesco De Cobelli: Nothing to disclose

**Implmentation of Diffusion Weighted Imaging for whole body staging of lymphoma patients**
*A. Milosevic*[1], M. Chodyla[1], H. Steinberg[1], L. Kessler[1], B. M. Schaarschmidt[1], L. Umutlu[1], J. Grueneisen[2]; [1]Essen/DE, [2]Munich/DE
*(aleksandar.milosevic@uk-essen.de)*

**Purpose or Learning Objective:** To asses the feasability of Diffusion Weighted Imaging (DWI) in staging of lymphoma patients to establish a radiation-free alternative to FDG-PET.
**Methods or Background:** A total of 181 lymphoma patients (mean age: 30.9 ± 19.1 years. 75 female and 106 male) undergoing clinically indicated 18F-FDG PET/MR examinations were retrospectivly assessed. 745 target lesions were assessed regarding Tracer-uptake (Standardized Uptake Values, SUV), diffusion restriction (Apparent Diffusion Coefficient, ADC), size and localization. Each of the target lesions was assigned a Deauville score. SUVs and ADC values were then compared using Spearman's rank correlation test. ROC-analysis was employed in order to find appropriate thresholds to distinguish between vital (score 4-5) and non-vital (score 1-3) manifestations in ADC-measurements,
**Results or Findings:** Calculated mean values for the ADCmin and ADCmean of targets with a Deauville score of 4 and 5 were significantly lower when compared to those lesions with a score of 1-3. Accordingly, ADCmean displayed a strong inverse correlation with the SUVs (r = -0.83). Furthermore, ROC analysis displayed an AUC of 0.91, 0.98 and 0,87 with a sensitivity of 87%, 93%, and 80% for ADCmin, ADCmean and ADCmax, respectively.
**Conclusion:** We highly recommend considering DWI an adjunct parameter for staging and restaging of lymphoma patients. DWI can be particularly helpful for individuals suffering from subtypes with low avidity to FDG and patient groups susceptible to radiation.
**Limitations:** Lack of proper gold standard for reference tissue in ADC meassurements. Thus, threshold levels were calculated manually.
**Funding for this study:** No funding.
**Ethics committee - additional information:** committee of university of Duisburg-Essen
**Author Disclosures:**
Johannes Grueneisen: Nothing to disclose
Benedikt Michael Schaarschmidt: Nothing to disclose
Lukas Kessler: Nothing to disclose
Lale Umutlu: Nothing to disclose
Aleksandar Milosevic: Nothing to disclose
Michal Chodyla: Nothing to disclose
Hannah Steinberg: Nothing to disclose

**In pursuit of an appropriate use criteria for the use of 18-F FES PET CT in the management of ER positive breast cancer : work in progress**
*P. S. Choudhury*, S. Chowdhury, M. Gupta, R. Kumar; Delhi/IN
*(pschoudhary@hotmail.com)*

**Purpose or Learning Objective:** Oestrogen receptor (ER) is highly expressed in 70-80% of breast malignancies (BC). ER expression or absence plays a central role in its oncogenesis and is a prognostic and predictive biomarker. Molecular imaging with 18-F Fluroestradiol (FES) PET-CT targets ER and may have higher incremental value in guiding management by increasing specificity.
**Methods or Background:** We enrolled 57 female and 1 male breast cancer patient during initial staging and restaging as a part of an ongoing prospective study and performed 18-F FDG and 18-F FES within 1 week. Whole body FDG and FES PET-CT scan done from base of skull to mid thigh. Image of the breasts performed in prone position by hanging technique. The study was approved by scientific committee (Res/SCM/53/2022/67) and IRB (RGCIRC/IRB-BHR/112/2022). Lesion detection sensitivity was compared for a total number of lesions by McNemar test. FES was taken as reference in indeterminate lesions. Incremental value was reported by identifying FES exclusive lesions. Spearman rank test was used to co-relate ER expression and SUV max.
**Results or Findings:** FDG was more sensitive in lesion detection (80.3% vs 61.2% p<0.001) However FES detected more lesions in lobular variety (81.5% vs 56.2% p0.09). Significant co-relation seen between ER+ve and FES uptake. Significant incremental value of FES seen in 27% of patients with indeterminate lesions characterised by FES. Overall change in management noted in 21.1% (5.2% surgical and intent of management 15.8%).
**Conclusion:** Potential clinical applications of FES PET CT could be to select appropriate patients for hormonal therapies, resolving ER status of lesions non-invasively, solving clinical dilemmas when results of other investigations are inconclusive, systemic staging of breast cancers with low metabolic activity and selecting optimal doses for current or novel ER targeted therapies.
**Limitations:** Work in progress
**Funding for this study:** Radiopharmaceuticals were procured by the institution and the equipments used belongs to the institution. No other source of funding was used

**Ethics committee - additional information:** Ethics Committee letter reference: IRB (RGCIRC/IRB-BHR/112/2022)
**Author Disclosures:**
Rajiv Kumar: Nothing to disclose
Manoj Gupta: Nothing to disclose
Suchita Chowdhury: Nothing to disclose
Partha S Choudhury: Nothing to disclose

**[1-11C]acetate PET/CT distinguishes aggressive cribriform Gleason score 7 prostate cancer and is mechanistically informed by spatial metabolomics**

*N. Sushentsev*[1], G. Hamm[1], R. Manavaki[1], D. Soloviev[2], D. Lewis[2], L. Aloj[1], R. Goodwin[1], F. A. Gallagher[1], T. Barrett[1]; [1]Cambridge/UK, [2]Glasgow/UK
*(ns784@medschl.cam.ac.uk)*

**Purpose or Learning Objective:** We aimed at identifying a clinical metabolic imaging technique to differentiate Gleason score 7 (GS7) prostate tumours with dominant cribriform and non-cribriform Gleason pattern 4 (GP4) based on their comparative metabolic pathway enrichment analysis (MPEA).
**Methods or Background:** 28 prostate cancer (PCa) patients with n=39 GS7 lesions on prostatectomy were recruited, of which n=27 and n=12 harboured non-cribriform and cribriform GP4, respectively. The patients were divided into three sub-cohorts (A, B, and C), each encompassing n=13 GS7 lesions (n=9 non-cribriform; n=4 cribriform). In cohort A, n=39 fresh-frozen tumour samples were used for spatial metabolomics imaging to enable comparative MPEA between cribriform and non-cribriform GP4 epithelium. In cohort B, formalin-fixed-paraffin-embedded samples were immunohistochemically stained for fatty acid synthase (FASN) to corroborate the findings from cohort A. In cohort C, we determined standardised uptake value (SUVbw) for [1-11C]-acetate PET/CT in cribriform and non-cribriform GS7 lesions as a marker of fatty acid synthesis.
**Results or Findings:** In cohort A, MPEA highlighted fatty acid biosynthesis as the most significantly enriched pathway in cribriform GP4 epithelium compared to non-cribriform glands (fold change 4.2; Padj<0.0001). In cohort B, this aligned with a significantly increased expression of FASN in cribriform GS7 lesions compared to non-cribriform tumours (P=0.001). In cohort C, this corresponded to a significant increase in mean SUVbw of cribriform lesions compared to non-cribriform tumours (P<0.05 for all timepoints up to 60min post-injection). Conversely, the comparison of tumour-to-urine 1H-MRI ADCratio derived from the whole cohort showed no difference between the two GS7 phenotypes (P=0.56).
**Conclusion:** Clinical imaging of lipid metabolism is a biologically informed way of characterising cribriform and non-cribriform GS7 PCa, which is a challenge for 1H-MRI.
**Limitations:** Modest sample size dictated by study complexity.
**Funding for this study:** Prostate Cancer UK, Cancer Research UK, AstraZeneca
**Ethics committee - additional information:** National Research Ethics Service Committee East of England, Cambridge South, Research Ethics Committee; study numbers: 16/EE/0205, 03/018. Cambridge University Hospitals Local Ethics Committee (CUH/15/EE/0213), and the Administration of Radioactive Substances Advisory Committee (ARSAC, certificate reference RPC/83/400/33606).
**Author Disclosures:**
Luigi Aloj: Nothing to disclose
Richard Goodwin: Employee: AstraZeneca
Roido Manavaki: Nothing to disclose
Nikita Sushentsev: Nothing to disclose
David Lewis: Nothing to disclose
Gregory Hamm: Employee: AstraZeneca
Tristan Barrett: Nothing to disclose
Ferdia Aidan Gallagher: Nothing to disclose
Dmitry Soloviev: Nothing to disclose

**The Role of [ 68 Ga]Ga FAPi PET/CT in Staging and Restaging in Breast Cancer with Low FDG Uptake**

N. Alan Selcuk, *G. Beydagi*, K. Akcay, B. B. Oven, S. Celik, L. Kabasakal; Istanbul/TR
*(gamzebeydagi@gmail.com)*

**Purpose or Learning Objective:** The aim of this study is to assess the potential efficacy of [68Ga]Ga FAPi PET/CT in staging and restaging in breast cancer patients with FDG-negative or low FDG uptake lesions.
**Methods or Background:** Between October 2020 and February 2024, 25 female patients with breast cancer were prospectively enrolled. These patients underwent [68Ga]Ga-FAPi and [18F]-FDG PET/CT imaging within one week for staging or restaging. The maximum standard uptake values (SUVmax) of the primary tumor areas and metastases in the [68Ga]Ga-FAPi and [18F]-FDG PET/CT images were recorded and statistically compared using the paired t-test.
**Results or Findings:** 25 female patients with suspicious primary malignancy recurrence or metastases but low FDG affinity were imaged with [68Ga]Ga-FAPi PET/CT. The mean age was 57.1±11.7 years. Histopathologic examination available for 20 patients revealed lobular carcinoma in 10 cases, ductal carcinoma in 8 cases, signet ring cell carcinoma in one patient and squamous cell carcinoma in one patient. In six patients (24%), neither the [18F]-FDG nor the [68Ga]Ga-FAPi PET/CT revealed any findings indicating recurrence or metastasis. Disease stage increased in 36% (n=9) of patients after [68Ga]Ga-FAPi PET/CT imaging, with 8 of them showing no pathologic findings on [18F]-FDG PET/CT. 60% (n=6) of the lobular carcinomas were upstaged after [68Ga]Ga-FAPi PET/CT. The detection of lymph nodes and distant metastases in lobular carcinoma was higher with [68Ga]Ga-FAPi PET/CT than with [18F]-FDG PET/CT. Furthermore, [68Ga]Ga-FAPi PET/CT showed a higher SUVmax in primary tumor foci and metastases (p<0.05).
**Conclusion:** [68Ga]Ga-FAPi PET/CT has been shown to be superior for staging in breast cancer, especially for lobular carcinoma with low FDG affinity. It is anticipated that [68Ga]Ga-FAPi PET/CT will be included in future guidelines for staging in breast cancer patients, especially in patients with lobular carcinoma.
**Limitations:** None
**Funding for this study:** None
**Ethics committee - additional information:** Ethics committee approval no: 1576
**Author Disclosures:**
Levent Kabasakal: Nothing to disclose
Bala Basak Oven: Nothing to disclose
Kaan Akcay: Nothing to disclose
Gamze Beydagi: Nothing to disclose
Serkan Celik: Nothing to disclose
Nalan Alan Selcuk: Nothing to disclose

**Can 18F-FDG-PET CT Predict Mediastinal Lymph Node Metastases in NSCLC Patients Undergoing Neo-Adjuvant Immuno-Chemotherapy?**

*D. Kifjak*, M. J. Hochmair, A. Korajac, S. Pochepnia, R-I. Milos, K. Sinn, A. Hoda, H. Prosch, L. Beer; Vienna/AT

**Purpose or Learning Objective:** To evaluate the association between 18F-FDG-PET CT quantitative imaging markers of surgically resected mediastinal lymph nodes and histopathologic results in patients with operable non-small cell lung cancer (NSCLC) treated with neo-adjuvant combined immuno-chemotherapy.
**Methods or Background:** This preliminary analysis of a prospective, single-center study included 20 patients (8 male, 12 female) with NSCLC who were treated with neo-adjuvant combined immune-chemotherapy and underwent pre-operative 18F-FDG-PET-CT. We semi-automatically extracted the following parameters for each mediastinal lymph node station: metabolic tumor volume (MTV) and total lesion glycolysis (TLG) at pre-operative scans. The histological results of mediastinal lymph nodes were retrieved from patients' records. A Mann-Whitney-U-Test was calculated to compare MTV/TLG and histological results for each mediastinal lymph node station.
**Results or Findings:** A total of 191 mediastinal lymph nodes were surgically removed. Four mediastinal lymph node metastases were found in three patients. In contrast 17 patients showed no tumor cells in their mediastinal lymph nodes. The median MTV for positive lymph nodes was 4.2 (range: 0-7.4) compared to 0 (range 0-4.3) for negative lymph nodes. The median TLG for positive lymph nodes was 6.3 (range 0-42) and for negative lymph nodes it was 0 (range 0-24). A statistically significant association between MTV and mediastinal lymph node metastases was observed, p=0.046. While there was a noticeable trend, no statistically significant association was identified between TLG and lymph node metastases, p=0.052.
**Conclusion:** High MTV values and to a lesser degree TLG are associated with residual lymph node metastases. However, both false positive (inflammation) and negative findings (micrometastatis) occur, thus limiting the ability of 18F-FDG-PET CT to predict histopathological response.
**Limitations:** Single-center study. Selection bias, only patients with histologically confirmed lymph nodes were part of the study.
**Funding for this study:** The Austrian Federal Ministry for Digital and Economic Affairs, the National Foundation for Research, Technology and Development and the Christian Doppler Research Association is gratefully acknowledged.
**Ethics committee - additional information:** Local IRB # 1521/2015
**Author Disclosures:**
Katharina Sinn: Nothing to disclose
Maximilian Johannes Hochmair: Nothing to disclose
Helmut Prosch: Nothing to disclose
Lucian Beer: Nothing to disclose
Aida Korajac: Nothing to disclose
Daria Kifjak: Nothing to disclose
Ruxandra-Iulia Milos: Nothing to disclose
Alireza Hoda: Nothing to disclose
Svitlana Pochepnia: Nothing to disclose

**Integrated diagnostics for survival prediction in patients with GEP-NET undergoing PRRT**

*F. Herr*, C. A. Dascalescu, M. P. Fabritius, M. Brendel, C. Auernhammer, C. Spitzweg, J. Ricke, M. Heimer, C. C. Cyran; Munich/DE
*(felix.herr@med.uni-muenchen.de)*

**Purpose or Learning Objective:** Integrated biomarkers of survival for patients with gastroenteropancreatic neuroendocrine tumors (GEP-NETs) receiving peptide receptor radionuclide therapy (PRRT) are still limited. This study aims to identify predictors of progression-free survival (PFS) in patients with GEP-NETs undergoing PRRT.
**Methods or Background:** This single-center retrospective study included 178 patients with GEP-NETs (G1 and G2) who received at least two consecutive cycles of PRRT with [177Lu]Lu-DOTA-TATE and underwent somatostatin receptor (SSTR) PET/CT before and after therapy. At baseline, an assessment was conducted in accordance with the Krenning score, and clinical parameters, including chromogranin A (CgA), neuron-specific enolase (NSE), hemoglobin, Ki-67, erythrocytes, C-reactive protein (CRP) and albumin were also collected. PFS was defined by a GEP-NET multidisciplinary team assessment.
**Results or Findings:** In univariate analysis at baseline, Krenning score 3, elevated levels of CgA (> 200 ng/dl) and NSE (>25 ng/dl) were significantly (p < 0.05) associated with shorter PFS. Ki-67 index > 5 %, primary tumor in the pancreas, levels of erythrocytes > 4 Mio/ µl, CRP > 1 mg/dl and albumin < 4.1 g/dl at baseline were also significantly (p<0.05) correlated with a shorter PFS. In multivariate analysis, Krenning score 3, CgA > 200 ng/ml, NSE > 35 ng/ml, and Ki-67 index > 5 % at baseline were significantly (p < 0.05) associated with shorter PFS. Including the Krenning score at baseline leads to a significant improvement of the cox regression model (p<0.05). Only the Ki-67 index (z=2.55) showed a higher z-score than the Krenning score at baseline (z = 2.41).
**Conclusion:** This study demonstrates the additional prognostic value of the Krenning score in conjunction with clinical parameters for patients with GEP-NET undergoing PRRT.
**Limitations:** Limitations of this study are its retrospective single-center design and the lack of multimodality imaging biomarkers.
**Funding for this study:** Wilhelm Vaillant Stiftung
**Ethics committee - additional information:** Ethics Committee of LMU-Munich – Project number: 20-1077
**Author Disclosures:**
Matthias Philipp Fabritius: Nothing to disclose
Clemens C. Cyran: Nothing to disclose
Christine Spitzweg: Nothing to disclose
Maurice Heimer: Nothing to disclose
Felix Herr: Nothing to disclose
Christoph Auernhammer: Nothing to disclose
Matthias Brendel: Nothing to disclose
Christian Alexander Dascalescu: Nothing to disclose
Jens Ricke: Nothing to disclose

**Multi-radiotracer PET/CT for the evaluation of carotid atherosclerotic plaque vulnerability: A systematic review**

*T. R. Readford*, P. Kench, M. Ugander, S. Patel, N. Giannotti; Sydney/AU
*(thomas.readford@sydney.edu.au)*

**Purpose or Learning Objective:** Carotid atherosclerosis is a major contributor to the burden of cerebrovascular diseases. Conventional imaging is limited in interrogating the biological and functional characteristics that may increase plaque vulnerability. Multiple positron emission tomography/computed tomography (PET/CT) radiotracers provide novel diagnostic insights into plaque vulnerability and identify patients at higher risk of cerebrovascular events. This systematic review investigated the clinical role of PET radiotracers in identifying vulnerable carotid atherosclerotic plaque.
**Methods or Background:** A systematic review of the existing literature was performed using the following search strategy: 'carotid', 'PET', 'atherosclerosis', 'plaque' and 'vulnerability'. Only original research articles were included. Covidence was used for data screening and data extraction.
**Results or Findings:** Thirty-nine studies were included that used 18F-fluorodeoxyglucose (18F-FDG), 18F-sodium fluoride (18F-NaF), 18F-fluoromisonidazole (18F-MISO), 68Ga-DOTATATE, 68Ga-Pentixafor and 11C-Acetate to target plaque metabolism-related inflammation, microcalcification, hypoxia, activated macrophages, C-X-C motif chemokine receptor4 and fatty acid synthesis, respectively. Seven studies used dual PET radiotracers with time intervals between scans ranging from one day to 4.8 months. Correlation between PET imaging and histology post-carotid endarterectomy was available in 17 studies. Authors noted agreement between macrophage-driven plaque inflammation by PET/CT and vulnerability-related morphological changes by MRI, suggesting complementary roles of combined MRI and PET/CT in detecting vulnerable plaque. Significant variability was observed in reported PET/CT acquisition techniques, injected radiotracer dose [18F-FDG: 185-925MBq, 18F-NaF: 125-370 MBq, 68Ga-DOTATATE: 148-157 MBq] and uptake times [18F-FDG: 50-180min ,18F-NaF: 60-180min, 68Ga-DOTATATE: 60-120min].
**Conclusion:** The use of multiple PET radiotracers may provide advanced diagnostic insights into carotid atherosclerotic plaque vulnerability. Further research is necessary to establish consensus on what constitutes a standard approach for the evaluation of vulnerable carotid plaque by PET/CT.
**Limitations:** Further quantitative analysis was limited by the variability of imaging parameters used across studies in this review.
**Funding for this study:** No funding was received for this study.
**Ethics committee - additional information:** This was a systematic review of existing literature.
**Author Disclosures:**
Peter Kench: Nothing to disclose
Martin Ugander: Nothing to disclose
Nicola Giannotti: Nothing to disclose
Thomas Ramsay Readford: Nothing to disclose
Sanjay Patel: Nothing to disclose

**Lymphoma Therapy Response Assessment with Low-Dose [18F]FDG Total-Body PET/CT**

*C. Mingels*[1], K. J. Chung[1], H. Nalbant[1], A. Willey[1], L. K. Shiyam Sundar[2], Y. G. Abdelhafez[1], R. Badawi[1], B. A. Spencer[1], L. Nardo[1];
[1]Sacramento, CA/US, [2]Vienna/AT

**Purpose or Learning Objective:** Our aim was to identify the lower limit of injected dose for [18F]FDG Total-Body (TB) PET/CT in lymphoma therapy response assessment.
**Methods or Background:** In this prospective study 24 patients with biopsy-proven lymphoma were enrolled for interim or end-of-treatment TB PET/CT after 1h and 2h of the injection of ~3.0MBq/Kg [18F]FDG. Lower injected activities (1.0 MBq/kg, 0.5MBq/kg, 0.25MBq/kg, 0.125MBq/kg) were simulated. Lesions were segmented by an artificial intelligence-aided software and confirmed by an expert. Standardized-uptake values (SUVmax/mean/peak), metabolic tumor volume (MTV) and total-lesion glycolysis (TLG) were calculated. Additionally, total MTV (TMTV) was assessed for each patient. Liver and mediastinal blood-pool were used to calculate tumor-to-background ratio (TBR) and contrast-to-noise level (CNR). Therapy response assessment was performed by Deauville criteria.
**Results or Findings:** In total, 182 lymphoma lesions were analyzed. SUVmax/mean/peak, MTV, TLG, TBR and TMTV were not significantly different between reference standard and low-dose images. Image noise increased significantly with lower doses. CNR decreased significantly. Clinical therapy response assessment by Deauville Score was significantly lower between 0.125MBq/kg and reference standard (p<0.01) for 1h p.i. imaging. All other low-dose reconstructions revealed no significant differences. For 2h p.i. there was a significant difference in Deauville Score for 0.5MBq/kg, 0.25MBq/kg and 0.125MBq/kg compared to the reference standard (p<0.01). Deauville Scores for 1MBq/kg at 2h were not significantly different to the reference standard (p=0.16).
**Conclusion:** Dose reduction in therapy response assessment with TB PET/CT is possible to a lower limit of 0.25MBq/kg for 1h p.i. imaging and 1.0MBq/kg for 2h p.i. TB PET/CT. However, lower injected activities are at risk to underestimate the metabolic activity of the lymphoma lesions due to higher noise levels. TMTV and TLG were not different in ultra-low-dose [18F]FDG TB PET/CT.
**Limitations:** Small cohort, simulated low dose images
**Funding for this study:** The work was also supported by the In Vivo Translational Imaging Shared Resources with funds from NCI P30CA093373 and by the Fred and Julia Rusch Foundation for Nuclear Medicine Research and Education. Hande Nalbant's funding is partially provided by United Imaging Health's UIH Fellowship Gift.
**Ethics committee - additional information:** This study was approved by the UC Davis institutional review board (IRB1470016). Written informed consent for inclusion was obtained. The study was performed in accordance with the Declaration of Helsinki.
**Author Disclosures:**
Hande Nalbant: Nothing to disclose
Kevin J. Chung: Nothing to disclose
Clemens Mingels: Nothing to disclose
Ramsey Badawi: Nothing to disclose
Lorenzo Nardo: Nothing to disclose
Andrew Willey: Nothing to disclose
Lalith Kumar Shiyam Sundar: Nothing to disclose
Benjamin A. Spencer: Nothing to disclose
Yasser Gaber Abdelhafez: Nothing to disclose

14:00-15:30 Research Stage 4

### Research Presentation Session: Abdominal and Gastrointestinal

### RPS 2001

### Shining a spotlight on HCC and liver transplant

**Moderator**
W. K. Jeong; Seoul/KR
*(jeongwk@gmail.com)*
**Author Disclosures:**
Woo Kyoung Jeong: Consultant: Guerbet; Research Grant/Support: GE Healthcare; Speaker: GE Healthcare

**The predictive value of iodine map histogram analysis of non-tumorous hepatic parenchyma for post-hepatectomy Liver Failure after narrow-margin hepatectomy in hepatocellular carcinoma**
*Y. Xu*, J. Liu, J. Zhou; Lanzhou City/CN
*(xuyuan961030@163.com)*

**Purpose or Learning Objective:** Post-hepatectomy liver failure (PHLF) is a severe postoperative complication with a high incidence and mortality rate, particularly in patients with narrow-margin (NM). This study aims to predict PHLF in NM-hepatocellular carcinoma (HCC) using iodine map histogram analysis of non-tumorous hepatic parenchyma.
**Methods or Background:** A retrospective analysis was conducted on the clinical and imaging data of 107 patients with NM-HCC who underwent hepatectomy, divided into those with PHLF (n=45) and without PHLF (n=62). Histogram parameters of non-tumorous hepatic parenchyma were calculated from iodine map derived from the portal venous phase of spectral CT, including Min, Max, Mean, SD, Skewness, Kurtosis, Entropy, and percentiles (V10-V95), along with the future liver remnant volume (FLV) and standardized future residual liver volume ratio (SFLV%). Logistic regression analyse was used to identify independent predictors of PHLF, and a comprehensive model was developed. The performance of the new comprehensive model was assessed using ROC curve analysis and was compared with ALBI and MELD scores.
**Results or Findings:** Significant intergroup differences were observed in the iodine map histogram analysis of non-tumorous hepatic parenchyma for Skewness, Kurtosis, V50, V75, V90, V95, FLV, and SFLV% ($P < 0.01$ to $P = 0.04$). Multivariate logistic regression analysis revealed that V95, Kurtosis, and SFLV% were independent risk factors for predicting PHLF. The comprehensive model (ModelALL), developed by combining these independent risk factors, exhibited the highest predictive efficacy for PHLF, with an AUC of 0.77 (0.67-0.87), outperforming both the ALBI and MELD scores, which had AUCs of 0.70 (0.58-0.81) and 0.62 (0.49-0.74), respectively.
**Conclusion:** The model which combines the iodine map histogram parameters of non-tumorous hepatic parenchyma (V95 and Kurtosis) with SFLV%, aids in the preoperative prediction of PHLF in NM-HCC patients and outperforms conventional scoring systems.
**Limitations:** Not applicable
**Funding for this study:** This study has received funding by grants of Natural Science Foundation of China (82260361, 82371914), Lanzhou University Second Hospital Second Hospital “Cuiying Technology Innovation Plan” (CY2022-QN-A10), Lanzhou University Second Hospital "Cuiying Postgraduate Instructor Cultivation Program Project (CYDSPY202003) and Outstanding Young Talents and Backbone Talents Project of Gansu Provincial Health Industry Research Program (GSWSQN2023-04).
**Ethics committee - additional information:** The present study was approved by our hospital ethics committee (No. 2022A-112) and performed according to the ethical guidelines of the 1975 Declaration of Helsinki.
**Author Disclosures:**
Junlin Zhou: Nothing to disclose
Yuan Xu: Nothing to disclose
Jianli Liu: Nothing to disclose

**Diagnostic performance of an abbreviated magnetic resonance protocol for surveillance of hepatocellular carcinoma in candidates for liver transplant**
*M. Mattone*, F. Quintarelli, A. Napoli, C. Catalano; Rome/IT
*(monicamattone95@gmail.com)*

**Purpose or Learning Objective:** To evaluate the per-patient diagnostic performance of an abbreviated magnetic resonance protocol for hepatocellular carcinoma (HCC) surveillance in cirrhotic liver.
**Methods or Background:** Retrospective study including cirrhotic patients with HCC who are candidates for liver transplant enrolled in a surveillance program based on the use of MRI with hepatobiliary contrast agent. Two different sets of images for each patient are provided to two radiologists, who interpret the images independently, to simulate an abbreviated protocol. Interobserver variability was assessed using Cohen's kappa coefficient. A reference standard based on histologic examination or radiologic criteria (LIRADS at least equal to 4) was used to determine the diagnostic accuracy of each set of images.
**Results or Findings:** A total of 200 patients who underwent MRI for HCC surveillance were included in this study. One set of images consisted of the complete protocol (Set1) and one set of images consisted of T2WI with fat saturation, DWI and hepatobiliary sequences (Set2).The sensitivity, specificity, and accuracy of Set of readers 1 and 2 were 91.5%/90.5%, 88.6.4%/87.6% and 87.5%/85.0%, respectively. The sensitivities of the sets were not significantly different. Inter-reader agreement was substantial. Ascites, previous surgical and interventional radiology treatments, and small tumor size are associated with lower sensitivity.
**Conclusion:** An abbreviated MRI protocol including T2WI with fat saturation, DWI and hepatobiliary sequences is highly sensitive and can be a valid method for HCC surveillance in cirrhotic liver in patients candidates to liver transplant.
**Limitations:** Low number of readers
**Funding for this study:** No founds were used
**Ethics committee - additional information:** Not applicable
**Author Disclosures:**
Monica Mattone: Nothing to disclose
Fabio Quintarelli: Nothing to disclose
Carlo Catalano: Nothing to disclose
Alessandro Napoli: Nothing to disclose

**Impact of CT-defined sarcopenia on survival in patients undergoing orthotopic liver transplant**
*D. Tore*[1], C. Guarnaccia[1], A. Depaoli[2], C. Gaetani[1], M. Anna Pia[1], M. Visciano[1], M. Dini[1], F. Tandoi[3], P. Fonio[1]; [1]Turin/IT, [2]Ivrea/IT, [3]Bari/IT

**Purpose or Learning Objective:** To evaluate the impact of CT-defined sarcopenia on survival in patients undergoing orthotropic liver transplant (OLT).
**Methods or Background:** Monocentric retrospective study. 440 patients (70 females, 370 males) who underwent OLT at our Institution within 01.01.2014 and 31.12.2019 with an abdomen CT scan acquired within six month before surgery were selected. Skeletal muscles segmentations at the level of L3 and L4 were performed using open source software 3D Slicer using thresholds (-29 to 150 HU); psoas muscle area (PMA), skeletal muscle area (SMA), psoas muscle index (PMI) and skeletal muscle index (SMI) were calculated at both levels. Optimal cut-offs to dichotomize between sarcopenic and non-sarcopenic patients divided in females and males were calculated using Youden's J statistic and ROC curves. Survival analysis was performed using Kaplan-Meyer's curves.
**Results or Findings:** Male patients classified as sarcopenic according to SMI criterion at L3 level presented an increased risk of mortality compared to non-sarcopenic with an hazard ratio of 1.63 ($p=0.03$). Male patients classified as sarcopenic according to SMA criterion at L3 level presented an increased risk of mortality compared to non-sarcopenic with an hazard ratio of 1.87 ($p=0.008$). In the female group none of the sarcopenia definitions tested highlighted an increased risk of mortality with p values always $>0.05$.
**Conclusion:** PMI and SMA CT-defined sarcopenia at L3 level represented a negative prognostic factor for male patients survival after OLT. The use of such definitions of sarcopenia may identify more fragile subjects in this setting and improve care and, possibly outcomes, for such patients. The absence of statistically significant differences between sarcopenic and non-sarcopenic female subjects may be due to the relatively limited sample size for such group of patients.
**Limitations:** Monocentric retrospective study.
**Funding for this study:** No funds were received for this work.
**Ethics committee - additional information:** Not applicable, retrospective study

**Author Disclosures:**
Micheletti Anna Pia: Nothing to disclose
Alessandro Depaoli: Nothing to disclose
Francesco Tandoi: Nothing to disclose
Carla Guarnaccia: Nothing to disclose
Massimiliano Dini: Nothing to disclose
Davide Tore: Nothing to disclose
Paolo Fonio: Nothing to disclose
Clara Gaetani: Nothing to disclose
Michele Visciano: Nothing to disclose

**Hepatic functional reserve score based on two-dimensional shear wave elastography for evaluation of preoperative hepatic functional reserve and prediction of post-hepatectomy liver failure in HCC**
*X. Zhong*; Guangzhou/CN
*(zhongx35@mail3.sysu.edu.cn)*

**Purpose or Learning Objective:** To establish a Hepatic Functional Reserve (HFR) score based on liver stiffness (LS) and liver volume, and to explore its feasibility in evaluating preoperative hepatic reserve and predicting postoperative remnant hepatic function in hepatocellular carcinoma (HCC). Additionally, the study aimed to determine a safe future liver remnant (FLR) volume threshold to prevent post-hepatectomy liver failure (PHLF).
**Methods or Background:** The study included 345 HCC patients, with 265 in the training group and 80 in the test group. Preoperative LS was measured using two-dimensional shear-wave elastography. Total liver volume (TLV), tumor volume (TuV), and FLR were simulated using the IQQA system. The median LS of patients without significant fibrosis (7.6kPa) and standard liver volume (SLV) were used as normal references for liver quality and quantity. The preoperative HFR score (HFR-PRE) was calculated as 7.6/LS×(TLV-TuV)/SLV, and its correlation with liver function indicators was assessed. The postoperative HFR score (HFR-POST) was calculated as 7.6/LS×FLR/SLV, and its effectiveness in predicting PHLF was evaluated. The optimal FLR threshold was determined using the maximum Youden index.
**Results or Findings:** HFR-PRE showed significant correlation with preoperative liver function indicators such as Child-Pugh, MELD, ALBI scores, and ICG-R15 (all $p<0.05$). The AUC for HFR-POST in predicting PHLF was 0.864 in the training group and 0.857 in the test group. HFR-POST outperformed ALBI, MELD, and ICG-R15 scores (all $p<0.05$) in predicting PHLF. The minimum FLR/SLV threshold was 5/76*LS to prevent PHLF, with a sensitivity of 81.5% and specificity of 77.3%.
**Conclusion:** The HFR score effectively evaluates preoperative liver function and predicts PHLF in HCC patients. It also helps determine a safe residual liver volume to reduce the risk of PHLF.
**Limitations:** LS measurement acquired from one liver segment might not represent the quality of the whole liver.
**Funding for this study:** None
**Ethics committee - additional information:** The study protocol received approval from the Institutional Review Board of The First Affiliated Hospital of Sun Yat-sen University (IRB approval number: [2019]046).
**Author Disclosures:**
Xian Zhong: Nothing to disclose

**Comparative Analysis of Semi-Automated CT Volumetry and Intraoperative Graft Weight in Living Donor Liver Transplantation in a tertiary care centre**
*A. Garg*, J. Chowdhary, P. K. Sukhani, S. P. Joshi; Jaipur/IN
*(ashnagarg9@gmail.com)*

**Purpose or Learning Objective:** To evaluate the accuracy of semi-automated CT volumetry (CTV) in estimating liver volume in potential living donor liver transplant (LDLT) donors, compared to the gold standard of intraoperative graft weight measurement. Aim: 1. Inform transplant surgeons and radiologists about the reliability of semi-automated CTV in LDLT donor evaluation. 2. Highlight the potential benefits of using CTV in preoperative planning, improved donor selection and reduced surgical complications. 3. Discuss the implications of accurate volume assessment on LDLT outcomes and patient safety.
**Methods or Background:** Background: Living Donor Liver Transplantation (LDLT) is a life-saving procedure for patients with end-stage liver disease. Accurate liver volume assessment is crucial in LDLT to ensure sufficient graft volume for recipient survival, prevent small-for-size graft syndrome and reduce donor morbidity and mortality. Methods: This retrospective study included 70 potential LDLT donors who underwent: Semi-automated CTV using AW VolumeShare 7 on 128 slice - GE Healthcare, Optima. Inclusion Criteria: Adult LDLT donors (>18 years) Exclusion Criteria: Previous liver surgery/disease Variant vascular/biliary anatomy, not allowing safe resection
**Results or Findings:** Primary Outcome: Strong correlation ($r = 0.92$, $p < 0.001$) Sensitivity: 93.2% (95% CI: 85.1-97.5) Specificity: 90.5% (95% CI: 81.2-95.8) Mean difference between CTV and intraoperative graft weight: 25.6 ± 57.8 grams Secondary Outcomes: Bland-Altman analysis showed good agreement between CTV and intraoperative graft weight, with 95% limits of agreement (-89.2 to 140.4 grams) CTV accurately predicted graft weight within 10% of actual weight in 85% of cases (n = 70)
**Conclusion:** Semi-automated CTV demonstrates high accuracy and reliability in estimating liver volume in LDLT donors. CTV accurately predicts graft weight with good agreement with intraoperative measurements thus helping in preoperative evaluation and surgical planning for LDLT.
**Limitations:** Single-center experience, Operator dependence, Intraoperative graft weight measurement variability
**Funding for this study:** No external funding
**Ethics committee - additional information:** Approved by the Institutional Ethics Committee, Mahatma Gandhi Medical College and Hospital, India
**Author Disclosures:**
Swati Purohit Joshi: Nothing to disclose
Jai Chowdhary: Nothing to disclose
Paresh Kumar Sukhani: Nothing to disclose
Ashna Garg: Nothing to disclose

**LI-RADS Nonradiation Treatment Response Algorithm Version 2024: Diagnostic Performance and Added Value of Ancillary Features**
*S. Zhou*, Y-C. Wang; Nanjing/CN

**Purpose or Learning Objective:** We aimed to evaluate the diagnostic performance of the MRI-based Nonradiation LR-TRA v2024 and the value of incorporating ancillary features (AFs) on improving diagnostic precision.
**Methods or Background:** This retrospective study included patients with HCC who underwent local-regional therapy (LRT) followed by curative treatments between January 2017 and December 2022. Each treated lesion was evaluated according to the LR-TRA v2024, LR-TRA v2017, and modified Response Evaluation Criteria in Solid Tumors (mRECIST) criteria, with pathologic response serving as the reference. The sensitivity, specificity, and accuracy of different treatment response criteria were compared using the McNemar test.
**Results or Findings:** A total of 231 patients (198 males; median age, 56 years; IQR, 50-63 years) with 306 treated lesions (249 incomplete pathologic necrosis) were evaluated. LR-TRA v2024-Viable (without AFs) exhibited comparable sensitivity and accuracy than LR-TRA v2017-Viable and mRECIST (sensitivity: 81.1% [95%CI: 75.8, 85.8], 79.5% [95% CI: 74.0, 84.4], and 80.3% [95% CI: 74.8, 85.1]; accuracy: 80.1% [95% CI: 75.1, 84.4], 79.4% [95% CI: 74.4, 83.8] and 75.2% [95% CI: 69.9, 79.9], respectively) for predicting incomplete pathologic tumor necrosis. Both LR-TRA v2024 (without AFs)-Viable and v2017-Viable exhibited significantly higher specificity than mRECIST (both $P \le .001$). LR-TRA v2024 (with AFs)-Viable incorporating ancillary features exhibited the highest sensitivity (85.9% [95% CI: 81.0, 90.0]) and accuracy (83.7% [95% CI: 79.0, 87.6]) among different treatment response criteria, and showed a statistic difference compared to LR-TRA v2017-Viable (both $P = .006$), without sacrificing specificity (73.7% [95% CI: 60.3, 84.5]).
**Conclusion:** LR-TRA v2024 (without AFs) demonstrates good diagnostic performance and ease of use. AFs significantly enhance diagnostic sensitivity and accuracy through category adjustments, without sacrificing specificity. LR-TRA v2024 combined with AFs is strongly recommended for use in clinical practice.
**Limitations:** Selection bias; only based on MRI
**Funding for this study:** This study has received funding by four National Natural Science Foundation of China (NSFC, No. 82271978, 92359304, 82330060, 823B2040); Zhongda Hospital Affiliated to Southeast University, Jiangsu Province High-Level Hospital Pairing Assistance Construction Funds (No. zdyyxy09).
**Ethics committee - additional information:** This study was approved by our institutional review board, and the requirement for written informed consent was waived for the retrospective data [No. 2022ZDSYLL410-P01].
**Author Disclosures:**
Yuan-Cheng Wang: Nothing to disclose
Shuwei Zhou: Nothing to disclose

**Short MRI Surveillance (SMS) for hepatocellular carcinoma screening: first results on image quality of the SMS-HCC study**
*C. Van De Braak*, F. Willemssen, F. Smits, R. De Man, A. Van Der Lugt, D. Bos, R. S. Dwarkasing; Rotterdam/NL
*(c.vandebraak@erasmusmc.nl)*

**Purpose or Learning Objective:** Current guidelines recommend biannual US screening for patients with high risk of developing hepatocellular carcinoma (HCC). It was reported that the sensitivity of US for detecting early-stage HCC in these patients is merely 47%. Our aim is to validate a Short MRI Surveillance (SMS)-protocol in current surveillance patients and compare it to US in a prospective, multicentre study. Here, we present our first results on image quality.

**Methods or Background:** From November 2023, patients from the current HCC surveillance programme were invited to undergo paired US-MRI screening. The MRI was performed on 1.5/3.0-T systems using a dedicated 8-16 channel range body coil. The protocol consisted of T1W in-out phase, T2W with fat saturation and DWI. One radiologist evaluated the US- and MRI images, while a second and third radiologist solely evaluated the MRI images. US was reported according to the LIRADS-US surveillance classification. The current analyses were restricted to the first 50 study participants and revolve around descriptive analyses of the image quality and presence of lesions.
**Results or Findings:** The most common indication for the HCC surveillance programme was Hepatitis B (35/50, 70%), followed by cirrhosis (16/50, 32%). Image quality was rated as good in 64% of the US examinations and 94% of the MRI examinations. Based on US, a total of 16 lesions were found in eight patients, whilst on MRI this total was 109 lesions in 21 patients. Reported lesions were benign (e.g. cysts), except for one lesion that was noted on SMS, but undetected on US, which proved to be LIRADS-3 on subsequent contrast-enhanced MRI.
**Conclusion:** Our preliminary results show the potential of MRI for HCC surveillance, through better image quality and a higher detection rate of focal lesions.
**Limitations:** No limitations were identified.
**Funding for this study:** Funding was provided by a grant of the Dutch Cancer Society (grant number: 2021-2/13803).
**Ethics committee - additional information:** This study was approved by the institutional review board and written consent was obtained from all participants.
**Author Disclosures:**
Aad Van Der Lugt: Nothing to disclose
Rob De Man: Nothing to disclose
Roy S. Dwarkasing: Nothing to disclose
Fokko Smits: Nothing to disclose
François Willemssen: Nothing to disclose
Daniel Bos: Nothing to disclose
Céline Van De Braak: Nothing to disclose

### Enhanced prediction of microvascular invasion in hepatocellular carcinoma: a comparative study between intraoperative ultrasound and CT radiomics

*F. Rizzetto*, S. Tortora, E. Rondi, P. Carboni, M. M. B. Barabino, A. Vanzulli; Milan/IT
*(Francesco.rizzetto@unimi.it)*

**Purpose or Learning Objective:** Intraoperative ultrasound (IOUS) can identify signs of microvascular invasion (MVI) in hepatocellular carcinoma (HCC) during surgery. We evaluated whether preoperative CT-derived radiomics can offer a less invasive alternative.
**Methods or Background:** All patients who underwent surgical resection with IOUS for histologically confirmed HCC lesions were retrospectively included. For those with available preoperative triphasic CT scans, HCC nodules were segmented across the arterial, venous, and delayed phases. Using PyRadiomics, radiomic features (RFs) were extracted from each segmentation. After dimensionality reduction, the selected RFs from each phase were used to train and validate various predictive models based on Support Vector Machine (SVM) algorithm, with histopathological confirmation of MVI as ground truth. The best performing model was tested on an independent dataset. Qualitative IOUS features were selected through multivariate regression and used to build a corresponding SVM model. Performance for detecting MVI was assessed using the area under the Receiver Operating Characteristic curve (AUC-ROC), with model comparisons made using the DeLong test.
**Results or Findings:** A total of 124 patients, each with a single HCC lesion, were selected, with preoperative CT scans acquired in nearly 30 different institutions. Of them, 86 patients were assigned to the training and validation dataset (80:20 split), while the remaining 38 served as independent test dataset. The most performing radiomic model included 10 RFs extracted from arterial phase. ROC analysis in the independent test yielded an AUC-ROC of 70% (95% confidence interval[CI]: 52-88%; p=0.030) for radiomic model and 70% (95%CI: 51-87%; p=0.020) for IOUS model, with no statistical difference (p=1.00).
**Conclusion:** Preoperative CT-derived radiomics demonstrated equivalent predictive performance to IOUS in assessing MVI in HCC, offering a non-invasive preoperative alternative for MVI risk stratification.
**Limitations:** Retrospective design is the main study limitation.
**Funding for this study:** No fundings were received for this study.
**Ethics committee - additional information:** Institutional Review Board approved the retrospective data collection in anonymous form
**Author Disclosures:**
Francesco Rizzetto: Nothing to disclose
Pierluigi Carboni: Nothing to disclose
Angelo Vanzulli: Nothing to disclose
Silvia Tortora: Nothing to disclose
Elisa Rondi: Nothing to disclose
Matteo Mario Bruno Barabino: Nothing to disclose

### A CT-based radiomics model to predict P53-mutated hepatocellular carcinoma

*Y. Shi*, M. Li, Y. Pei, W. Li; Changsha/CN
*(sytsyt1998@163.com)*

**Purpose or Learning Objective:** To evaluate the diagnostic performance of a CT-based radiomics model for predicting P53-mutated hepatocellular carcinoma (HCC).
**Methods or Background:** In this retrospective single-center study, patients with histopathologically confirmed HCC who underwent preoperative contrasted-enhanced CT examination and surgery between November 2017 and July 2022 were recruited. HCC was classified into P53-mutated HCC and non-P53-mutated HCC using the gene sequencing. Radiological features were analyzed and clinical information were collected. Radscore was based on radiomics features extracted from the plain scan, arterial phase and portal vein phase images using the random forest method. Univariable and multivariable logistic regression analyses were used to identify variables that were significantly and independently associated with P53-mutated HCC, which were furtherly to develop a model. The model performance was evaluated with the area under the receiver operating characteristic curve (AUC), and the log-rank test was used to analyze recurrence-free survival (RFS).
**Results or Findings:** A total of 109 patients were enrolled, and assigned randomly (8:2) into training (87 patients) and validation sets (22 patients). The univariable analysis showed that the presence of enhancing capsule and intratumoral artery, and the radscore were significant risk factors for P53-mutated HCC. Further multivariable analysis identified that only the radscore was the independent predictor (odds ratio (OR), 3.05 [95% CI, 2.00–4.67], p<0.001) for P53-mutated HCC, and was used to develop the radiomics model. The model showed excellent performance in predicting P53-mutated HCC, with AUC of 0.899 (95% CI: 0.836-0.962) in the training set and 0.744 (95% CI: 0.523-0.964) in the validation set, Patients with predicted P53-mutated HCC has shorter RFS than those with predicted non-P53-mutated HCC (p=0.03).
**Conclusion:** A CT-based radiomics model could accurately predict P53-mutated HCC.
**Limitations:** A single-center retrospective study with a small sample.
**Funding for this study:** Funding: This work was supported by the National Natural Science Foundation of China (82071895, Wenzheng Li; 82271984, Wenzheng Li), the Natural Science Foundation of Hunan Province (2023JJ30903, Wenzheng Li; 2022JJ30950, Yigang Pei), the Natural Science Foundation for Youth of Hunan Province (2024JJ6665, Mengsi Li; 2023JJ40970, Wenguang Liu), the Youth Science Foundation of Xiangya Hospital (2023Q06, Mengsi Li) and Postdoctoral Fellowship Programof CPSF (GZC20242047, Mengsi Li).
**Ethics committee - additional information:** Institutional Review Board approval was abtained.
**Author Disclosures:**
Wenzheng Li: Nothing to disclose
Yuting Shi: Nothing to disclose
Yigang Pei: Nothing to disclose
Mengsi Li: Nothing to disclose

### The role of gadoxetic acid-enhanced MRI-derived residual relative enhancement index (RREI) in quantifying liver function in hepatocelulary carcinoma patients

*U. Eryürük*, M. N. Tasdemir, E. Cakir, S. Aslan; Giresun/TR
*(uluhaneryuruk@gmail.com)*

**Purpose or Learning Objective:** To investigate the efficacy of the residual relative enhancement index (RREI), derived from gadoxetic acid-enhanced MRI, in estimating liver function in patients with hepatocellular carcinoma (HCC), by validating with the albumin-bilirubin (ALBI) grade.
**Methods or Background:** We retrospectively analyzed 41 patients with HCC who underwent gadoxetic acid-enhanced MRI. Enhancement ratio (ER) was calculated using the formula ER=(SI_HBP20-SI_pre)/SI_pre, where SI_HBP20 represents the signal intensity in the hepatobiliary phase and SI_pre represents the pre-contrast signal intensity. RREI was calculated as RREI=residual liver volume (RLV)×ER. Receiver operating characteristic curve analysis was performed to determine optimal cut-off values of RLV, ER, and RREI for predicting ALBI grades. Spearman's rank correlation was used to evaluate correlations between RLV, ER, RREI, and ALBI scores. Intraclass correlation coefficient (ICC) was used to assess intra-reader reliability and inter-reader agreement for RLV, ER, and RREI measurements.

Saturday

**Results or Findings:** ROC analysis showed that the optimal RREI cut-off for predicting ALBI grade 1 was 680-698, and for ALBI grade 3 was 537-496, for reader 1 and reader 2, respectively. RREI demonstrated good performance in predicting ALBI grade 1, with accuracy of 85.3%-95.1%, sensitivity of 89.4%-94.7%, and specificity of 81.8%-95.4%. In differentiating ALBI grade 3 from other grades, RREI showed excellent performance, with accuracy of 92.7%-97.6%, sensitivity of 90.6%-96.9%, and specificity of 100%. Strong correlations were observed between RREI and ALBI scores, with correlation coefficients of -0.852 and -0.839 for both readers. Intra-reader and inter-reader reliability was almost perfect, with ICC values of 0.975 and 0.937, respectively.
**Conclusion:** RREI exhibited a strong correlation with ALBI scores for assessing liver function in HCC patients and showed good accuracy in predicting ALBI grades, indicating its potential as a reliable radiological tool for evaluating liver function.
**Limitations:** This was a retrospective study.
**Funding for this study:** None.
**Ethics committee - additional information:** The study was approved by the local ethics commitee.
**Author Disclosures:**
Uluhan Eryürük: Nothing to disclose
Merve Nur Tasdemir: Nothing to disclose
Ertugrul Cakir: Nothing to disclose
Serdar Aslan: Nothing to disclose

**Sarcopenia improves after transjugular intrahepatic portosystemic shunt (TIPS) placement in patients with cirrhosis**
*J. Kittinger*, T. Müllner-Bucsics, L. Hartl, L. Reider, F. Wolf, M. Trauner, M. Mandorfer, T. Reiberger, K. Lampichler; Vienna/AT

**Purpose or Learning Objective:** Transjugular intrahepatic portosystemic shunt (TIPS) is used to treat complications of portal hypertension in patients with cirrhosis. Sarcopenia in cirrhosis has been linked to worse patient outcomes but may improve after TIPS. We aimed to evaluate the prevalence of sarcopenia in patients undergoing TIPS and its evolution after TIPS.
**Methods or Background:** Retrospective analysis of the Vienna TIPS cohort from 01/2004 to 03/2022. Transversal psoas muscle thickness (TPMT) was evaluated on cross-sectional abdominal imaging both (i) prior to TIPS (time interval < 3 months) and (ii) at follow-up time points (FU 1: 3-18 months; FU 2 > 18 months) – by two independent radiologists. Sarcopenia was defined by previously published height-corrected TPMT cut-offs at the level of L3 (men < 12mm/m; women < 8mm/m).
**Results or Findings:** 199 patients were included (mean age 55.7±11 years, 69.3% male; median MELD: 11 (9-18) ; history of variceal bleeding 33.2% and ascites 79.9%). FU imaging was available in 70 patients (FU 1) and 57 patients (FU 2), respectively. Interrater reliability of psoas muscle measurements was excellent (κ=0.985). Sarcopenia was highly prevalent in patients undergoing TIPS (42.7%, 85/199). 4/45 (8.9%) of non-sarcopenic patients at baseline developed sarcopenia after TIPS implantation at FU 1 and 5/35 (14.3%) at FU 2, respectively. Transplant-free survival (TFS) of patients without sarcopenia was favourable (median: 59 months, IQR: 15 months – ∞), while mortality was high among patients with sarcopenia at baseline (TFS: median 21, IQR 1.8 – 106 months; p < 0.001). Resolution of sarcopenia was linked to a significant survival benefit (HR 0.121; p<0.001; adjusted to age and MELD score).
**Conclusion:** Sarcopenia is prevalent in TIPS patients and linked to worse outcome.
**Limitations:** CT-based diagnosis of sarcopenia without functional testing.
**Funding for this study:** No funding was provided for this study.
**Ethics committee - additional information:** Approval of local ethics committee (Medical University of Vienna) was obtained: EK 1760/2014, 14-264-VK, EK 1943/2017.
**Author Disclosures:**
Mattias Mandorfer: Speaker: AbbVie, Gilead, Collective Acumen, and W. L. Gore & Associates, Takeda Advisory Board: AbbVie, Gilead, Collective Acumen, and W. L. Gore & Associates, Takeda
Theresa Müllner-Bucsics: Nothing to disclose
Michael Trauner: Speaker: Bristol-Myers Squibb (BMS), Falk Foundation, Gilead, Intercept and Merck Sharp & Dohme (MSD) Advisory Board: from Abbvie, Albireo, Boehringer Ingelheim, BiomX, Falk Pharma GmbH, GENFIT, Gilead, Hightide, Intercept, Janssen, MSD, Novartis, Phenex, Pliant, Regulus and Shire; travel grants from AbbVie, Falk, Gilead, Intercept and Janssen; and research grants from Albireo, Alnylam, CymaBay, Falk, Gilead, Intercept, MSD, Takeda and Ultragenyx
Katharina Lampichler: Nothing to disclose
Jakob Kittinger: Nothing to disclose
Thomas Reiberger: Speaker: AbbVie, Bayer, Boehringer Ingelheim, Gilead, Intercept, MSD, Roche, Siemens, and W. L. Gore & Associates
Florian Wolf: Nothing to disclose
Lukas Hartl: Nothing to disclose
Lukas Reider: Nothing to disclose

**14:00-15:30** **Room G1**

### Research Presentation Session: Radiographers

### RPS 2014

### Green innovations: radiographers pioneering sustainable healthcare

**Moderators**
G. D'Anna; Milan/IT
*(gennaro.danna@gmail.com)*
M. F. McEntee; Cork/IE
*(mark.mcentee@ucc.ie)*

**Sustainability in medical imaging and radiotherapy education and practice: a survey of the student perspectives in a Portuguese Allied Health School**
*J. M. Saude*, N. Adubeiro, L. Nogueira, I. Ribeiro, A. Xavier, C. Carvalhais; Porto/PT
*(miguelsaude@ess.ipp.pt)*

**Purpose or Learning Objective:** Higher education institutions play an important role as they prepare the professionals, decision-makers, and democratic citizens of the future. In Allied Health education, it is crucial to develop a professional that, in addition to the technical and clinical content, also have a critical sense regarding environmental and social aspects. This study aims to survey medical imaging and radiotherapy (MIR) students' perceptions and knowledge about environmental sustainability in MIR education and practice.
**Methods or Background:** A cross-sectional study was carried out, using a self-designed questionnaire, partially adapted and translated to Portuguese from previous studies. The final version of the questionnaire, after a pilot test, included twenty-six questions and was distributed amongst MIR bachelor's degree students attending 1st, 2nd, 3rd and 4th academic years in September and October of 2024.
**Results or Findings:** A total of 175 students participated in the study. Results showed that almost everyone believes they possess general knowledge about Sustainability, however 23,0% demonstrate a lack of awareness about the negative impact that clinical practice has on the environment. Data also revealed that students considered there is a need for more teaching about sustainability in the course (49,0%). In general, participants demonstrated little knowledge about sustainable practices in the profession (34,0%), although, in daily life reported high adoption of sustainable practices (85,0%). Strong connection between their course and SDGs 3 (91,4%), 4 (53,7%), and 8 (38,8%), was reported.
**Conclusion:** Our findings revealed that there is room for curricula adjustments. Early exposure to sustainability concepts has the potential to promote the development of environmentally conscious professionals. By integrating sustainability into their education will potentially increase the knowledge and skills to make informed decisions that can reduce the negative environmental impacts of clinical practices.
**Limitations:** No limitations were identified.
**Funding for this study:** No funding was received for this study.
**Ethics committee - additional information:** The study is educational and received a favourable statement from the institutional Data Protection Officer.
**Author Disclosures:**
Luisa Nogueira: Nothing to disclose
Nuno Adubeiro: Nothing to disclose
Jose Miguel Saude: Nothing to disclose
Inês Ribeiro: Nothing to disclose
Carlos Carvalhais: Nothing to disclose
Ana Xavier: Nothing to disclose

**European Radiology Departments: Radiographers' Perspectives on Environmental and Energy Sustainability**
*A. Roletto*[1], D. Catania[1], L. Rainford[2], A. Savio[3], M. Zanardo[1], G. R. Bonfitto[1], S. Zanoni[3]; [1]Milan/IT, [2]Dublin/IE, [3]Brescia/IT
*(rolettoandrea@yahoo.it)*

**Purpose or Learning Objective:** Energy consumption of radiology equipment, life cycle of consumables, waste generation, and CO2 emissions caused by staff travel are factors that influence the environmental impact of radiology departments. Through an international survey, the perception and knowledge

Saturday of European radiographers on environmental sustainability issues was investigated.
**Methods or Background:** In March 2024, an online survey was developed and shared with radiographers and therapeutic radiographers across Europe. The survey consisted of 43 questions, covering demographic information and participants' views and actions related to environmental sustainability, energy consumption and waste generation in healthcare.
**Results or Findings:** In total, 253 responses were collected from 27 European countries. Most respondents highlighted the importance of environmental sustainability in healthcare. According to 63.6% (n=161) of participant, the main source of environmental impact in radiology comes from energy consumption of radiological equipment. Additionally, 44.7% (n=113) suggested that conducting diagnostic exams remotely could reduce the $CO_2$ emissions caused by staff commuting. Regarding workplace practices, over 70% (n=192) reported that they turn-off devices after use. Among the possible obstacles to turning off radiological equipment, respondents identified long shutdown and/or restart times, loss of clinical/technical data and non-applicability to the radiographers' role. Although recycling is practiced, it is limited to paper, plastic, and glass. Lack of environmental sustainability strategies in the workplace was reported by 66% (n=167). Meanwhile, 96.1% (n=243) believe that radiographers could play an active role in promoting environmental sustainability in their departments.
**Conclusion:** This study offers a comprehensive analysis of European radiographers' awareness and opinions on environmental sustainability. Although radiographers recognize the importance of creating eco-friendly radiology departments, there are still gaps in implementation of sustainable practices within radiology practices.
**Limitations:** Potential biases may have been introduced in relation to the complexity of the topics covered.
**Funding for this study:** Not applicable
**Ethics committee - additional information:** Ethical exemption was granted by the host institution University College Dublin for this anonymous survey (LS-LR-24-25-Catania-Rainford)
**Author Disclosures:**
Anna Savio: Nothing to disclose
Diego Catania: Nothing to disclose
Louise Rainford: Nothing to disclose
Andrea Roletto: Nothing to disclose
Moreno Zanardo: Nothing to disclose
Giuseppe Roberto Bonfitto: Nothing to disclose
Simone Zanoni: Nothing to disclose

**State-of-Play in Artificial Intelligence Sustainability in Medical Imaging: a scoping review**
M. Champendal[1], B. Lokaj[2], J. Zaghir[3], V. Durand De Gevigney[2], C. Lovis[3], H. Müller[4], J. Schmid[2], *R. S. T. Ribeiro*[1]; [1]Lausanne/CH, [2]Genève/CH, [3]Geneva/CH, [4]Sierre/CH
*(ricardo.ribeiro@hesav.ch)*

**Purpose or Learning Objective:** To synthesize the existing literature on how the environmental sustainability of artificial intelligence (AI) in medical imaging is being addressed and to identify specific strategies that have been used.
**Methods or Background:** A scoping review was conducted following the Joanna Briggs Institute methodology. Comprehensive literature search was performed in MEDLINE, Embase, CINAHL, and Web of Science, targeting publications from 2014 to 2024 in English or French. The search used a combination of keywords and MeSH terms related to environmental sustainability, AI, and medical imaging modalities. Three independent reviewers screened abstracts, titles and full texts for eligibility.
**Results or Findings:** The search identified 2812 results, of which 11 met the inclusion criteria. The selected papers comprised 8 research articles, 3 reviews. Three key themes emerged: energy consumption (n=10), carbon footprint (n=4), and computational resources (n=4). The metrics $CO_2$ equivalent, carbon intensity, training time, power use effectiveness, equivalent distance travelled by car were proposed to assess potential AI impact on the environment. Most energy-efficient techniques involved data, AI modelling and training such as data augmentation, data quantisation, lightweight model development, reduction of parameters. Identified strategies to enhance efficiency and reduce environmental impact include (i)integrating energy and carbon metrics in AI evaluation in addition to accuracy assessments, (ii)developing an ecolabel for AI tools, (iii)transitioning to cloud computing and (iv)developing lightweight AI models.
**Conclusion:** This review identified critical metrics and actionable strategies used to assess and improve sustainable practices in AI for medical imaging which include the integration of specific sustainability-related metrics, cloud computing adoption and development of efficient AI models.
**Limitations:** The limitations of this review include not assessing the quality of the articles, which is standard practice in scoping reviews, and restricting the search to only four databases.
**Funding for this study:** Not applicable
**Ethics committee - additional information:** Not applicable
**Author Disclosures:**
Ricardo Silva Teresa Ribeiro: Nothing to disclose
Jamil Zaghir: Nothing to disclose
Henning Müller: Nothing to disclose
Christian Lovis: Nothing to disclose
Jerome Schmid: Nothing to disclose
Valentin Durand De Gevigney: Nothing to disclose
Belinda Lokaj: Nothing to disclose
Mélanie Champendal: Nothing to disclose

**A life-cycle assessment framework for quantifying the carbon footprint of diagnostic imaging**
*A. Roletto*[1], A. Savio[2], B. Marchi[2], S. Zanoni[2]; [1]Milan/IT, [2]Brescia/IT
*(rolettoandrea@yahoo.it)*

**Purpose or Learning Objective:** Environmental sustainability topic is increasingly relevant in the radiology sector, which accounts for about 10% of healthcare sector's carbon footprint. Life-cycle assessment (LCA) is one of the main tools for analysing the environmental impact of processes. This study aims to review existing LCA approaches in radiology available in literature, identify their characteristics and limitations to propose a more comprehensive LCA framework for diagnostic imaging.
**Methods or Background:** Through a literature review on the topic of LCA in diagnostic imaging, performed according to the PRISMA statement, a novel LCA framework specifically for diagnostic imaging was developed. The framework includes all the features described by the various selected articles and overcoming their limitations.
**Results or Findings:** Regarding the literature review, an LCA approach was described by 5 articles. All studies electricity consumption in their framework. Usage of consumables and auxiliary equipment was included in 80% (4/5) of the studies. Equipment production, staff commuting, and waste generation were included in only 40% (2/5). Only two articles have considered a cradle-to-grave system approach. Subsequently, a novel LCA framework was designed to overcome the limitations by covering all aspects that can be included in the three phases: 1) Preclinical, production of imaging equipment and consumables, staff commuting; 2) Clinical, use of devices and consumables during diagnostic imaging; 3) Post-clinical, waste generation and data storage for the images produced.
**Conclusion:** This literature review provides an overview of studies focusing on LCA methodology for diagnostic imaging. The results culminated in the proposal of a comprehensive framework, which aims to overcome the identified limitations by providing a complete analysis of the environmental footprint of radiological procedures.
**Limitations:** Still sparse reporting of LCA in Diagnostic imaging limited the strength of the conclusions of this study.
**Funding for this study:** Not applicable
**Ethics committee - additional information:** Not applicable
**Author Disclosures:**
Anna Savio: Nothing to disclose
Andrea Roletto: Nothing to disclose
Beatrice Marchi: Nothing to disclose
Simone Zanoni: Nothing to disclose

**Leading Radiographers into a Greener Future: A Systematic Review of Green Transformational Leadership and Sustainable Practices**
*L. Federico*[1], A. Roletto[2], D. Catania[2], S. Zanoni[3], S. Durante[1]; [1]Bologna/IT, [2]Milan/IT, [3]Brescia/IT
*(laura.federico01@gmail.com)*

**Purpose or Learning Objective:** The healthcare system requires many resources to maintain the standards necessary to care for the population. It is the task of effective leadership to ensure the environmental sustainability clinical activities. The aim of this study is to examine the role of Green Transformational Leadership (GTL) in radiography, focusing on the mitigation strategies adopted by radiographer managers and their impact on promoting environmental sustainability in radiology departments.
**Methods or Background:** A systematic literature was conducted in accordance with the PRISMA statements in several databases, targeting studies on leadership in radiography, green innovation, and environmental responsibility. The search strategy employed keywords such as "green transformational leadership," "radiographer manager," and "environmental leadership in healthcare." Two reviewers independently screened titles and abstracts to select studies based on inclusion criteria.
**Results or Findings:** Among the retrieved articles, 6 met the inclusion criteria. The analysis highlighted how GTL can influence the implementation of sustainable practices in radiography. Radiographer managers using GTL play a key role in promoting sustainable practices, such as reducing energy consumption, encouraging use of reusable imaging equipment, and minimising waste generation. Interdisciplinary collaboration could be decisive in increasing effectiveness of green initiatives. Studies reviewed highlighted the importance of successful communication, measurable goals, and staff training for adopting green practices. Radiography service managers with emotional intelligence who fostered a climate of trust were more successful in promoting a culture of sustainability.
**Conclusion:** Radiography leaders who prioritise green strategies can significantly reduce the environmental impact of radiology departments. Investing in leadership development, promoting emotional intelligence, and encouraging interdisciplinary collaboration are critical steps in creating a green healthcare system that aims towards environmental sustainability without reducing quality patient care.
**Limitations:** Sparce literature of GTL limit the strength of the conclusions of this study.
**Funding for this study:** Not needed
**Ethics committee - additional information:** Not needed
**Author Disclosures:**
Diego Catania: Nothing to disclose
Stefano Durante: Nothing to disclose
Andrea Roletto: Nothing to disclose
Simone Zanoni: Nothing to disclose
Laura Federico: Nothing to disclose

**Sustainable practices in nuclear medicine: A scoping review**

*D. Fonseca Ribeiro*[1], K. Borg Grima[2], A. Geão[3], C. Andersson[4], S. Murphy[5], P. S. Costa[6], C. Baun[7], A. Karangelis[8], M. Champendal[9]; [1]London/UK, [2]Naxxar/MT, [3]Montijo/PT, [4]Uppsala/SE, [5]Dublin/IE, [6]Porto/PT, [7]Odense/DK, [8]Patra/GR, [9]Lausanne/CH

**Purpose or Learning Objective:** Sustainable development seeks to balance economic growth, environmental impact, and social inclusion, which is critical in high-tech fields like nuclear medicine. This study reviewed published literature on sustainable practices in nuclear medicine, focusing on the three main pillars of sustainability: environmental, social, and economic.
**Methods or Background:** The scoping review was conducted in accordance with the Joanna Briggs Institute methodology. The search was performed on PubMed, Embase, Cinhal and Web of Science in November 2023 and included studies in English. The research equation combined keywords and Medical Subject Heading terms (MeSH) related to sustainability in Nuclear Medicine. Three independent review authors screened all abstracts and titles, and four reviewers conducted the data extraction and analysis.
**Results or Findings:** Thirty-two studies met the inclusion criteria for this scoping review, with most articles having been published in 2022 and 2023. Spain contributed the highest number of publications. Studies were categorised according to procedure type (n = 32; 31% therapy, 9% PET, 35% diagnostic & therapy, 19% SPECT) and study location (hospital based, non-hospital based, or both). The studies primarily focused on strategies related to the three sustainability pillars, with an emphasis on the environmental impact.
**Conclusion:** This review highlighted the growing interest in sustainability within the nuclear medicine field, especially in relation to environmental factors. However, significant knowledge gaps emerged on the impact of economic and social factors within nuclear medicine practices. Recommended strategies included proper radioactive waste management, resource optimisation, and fostering collaborative environments to ensure sustainable nuclear medicine practices.
**Limitations:** The limitations of the study are the exclusion of articles not available in the English language and the quality of the articles included was not assessed according to the methodology of a scoping review.
**Funding for this study:** No funding was provided for this study.
**Ethics committee - additional information:** This study was a scoping review.
**Author Disclosures:**
Daniela Fonseca Ribeiro: Nothing to disclose
Karen Borg Grima: Nothing to disclose
Apostolos Karangelis: Nothing to disclose
Ana Geão: Nothing to disclose
Camilla Andersson: Nothing to disclose
Christina Baun: Nothing to disclose
Pedro Silva Costa: Nothing to disclose
Shauna Murphy: Nothing to disclose
Mélanie Champendal: Nothing to disclose

**Towards sustainable radiography: exploring perceptions and overcoming barriers**

*M. A. Rawashdeh*[1], A. England[2], M. F. Mcentee[2], M. Ali[1], M. E. S. Zakaria[1]; [1]Ajman/AE, [2]Cork/IE
*(marawashdeh@just.edu.jo)*

**Purpose or Learning Objective:** Previous research has examined the attitudes and behaviors of various professions regarding environmental sustainability. However, there is a paucity of studies specifically addressing the perspectives of radiographers. This study aims to investigate radiographers' perceptions, practices, and barriers to change related to environmental sustainability within the field of radiology.
**Methods or Background:** Ethical approval was obtained and data collection was conducted using Google Forms. The survey targeted 104 practicing radiographers across multiple countries. Questions were organized into five domains to gather insights into demographics, training related to global warming and climate change. Data analysis employed both descriptive and inferential statistics.
**Results or Findings:** A total of 104 radiographers completed the survey. Female respondents exhibited a significantly higher participation rate in environmental protection campaigns (P=0.01). The majority of participants (68%) acknowledged their awareness of climate change and its impact on the natural environment. Furthermore, 74% of respondents indicated a belief in the necessity to enhance sustainability practices. The most frequently employed strategies to reduce energy consumption and emissions included low-energy lighting (60%), real-time power monitoring tools (41%), and energy-efficient heating systems (32%). A notable concern regarding sustainability emerged among respondents, with time constraints (50%) and a lack of leadership (48%) identified as prevalent barriers.
**Conclusion:** Participants recognize the significance of environmental sustainability in the field of radiology; however, obstacles such as inadequate leadership, support, authority, and facility limitations impede the adoption of sustainable practices.
**Limitations:** The present study has several limitations. It relied on voluntary participation from radiographers and employed snowball sampling, which may introduce self-selection bias. Additionally, the online survey format may limit geographical or technical access, thereby affecting the generalizability of study findings.
**Funding for this study:** Not applicable
**Ethics committee - additional information:** IRB from Gulf Medical University
**Author Disclosures:**
Mark F. Mcentee: Nothing to disclose
Mohamed El Sayed Zakaria: Nothing to disclose
Magdi Ali: Nothing to disclose
Andrew England: Nothing to disclose
Mohammad Ahmmad Rawashdeh: Nothing to disclose

**Considerations on the environmental sustainability of using ChatGPT in radiography**

*E. Scaramelli*, G. R. Bonfitto, A. Roletto, S. V. Fasulo, L. Bombelli, D. Catania; Milan/IT
*(es.elena.scaramelli00@gmail.com)*

**Purpose or Learning Objective:** ChatGPT has gained credit among radiographers, given its contribution to training and clinical activity. However, its environmental impact is overlooked. The increasing use of AI is contributing to rising Greenhouse gas emissions, water waste and energy consumption. This research aims to explore the environmental impact of using ChatGPT in radiography and raise awareness of its sustainability in daily clinical practice, underscoring the need for radiographers to engage, given the gap in AI's environmental impact studies within radiology.
**Methods or Background:** A literature review across databases (Google Scholar, PubMed) was conducted according to the PRISMA statement to find recent evidence on environmental impact of ChatGPT in radiography and, more generally, in healthcare.
**Results or Findings:** The literature shows limited evidence on the sustainability of AI in radiology, leading to an analysis of the broader healthcare sector to estimate its impact and develop insights for radiology. From this search, only 16 articles were found to be relevant to healthcare, and just one focused on radiology. The results reveal a dual nature of AI: while it's incredibly useful in healthcare, it has a significant environmental impact. Indeed, companies like Google, Microsoft, and Meta consume 2.2 billion cubic meters of water to cool their servers and produce electricity, marking a 48% increase in water usage. GPT-3 produces 8.4 metric tons of $CO_2$ annually and requires 700,000 Liters of water for training. AI is also responsible for 1-2% of global electricity consumption, raising important questions about its long-term sustainability.
**Conclusion:** Although tools such ChatGPT show usefulness in clinical practice, including radiography, it’s essential to use them more responsibly, given their substantial environmental impact on planet's resources.
**Limitations:** The lack of evidence in the literature, limits the strength of the conclusions of this study.
**Funding for this study:** Not applicable
**Ethics committee - additional information:** Not applicable
**Author Disclosures:**
Luca Bombelli: Nothing to disclose
Simone Vito Fasulo: Nothing to disclose
Diego Catania: Nothing to disclose
Andrea Roletto: Nothing to disclose
Elena Scaramelli: Nothing to disclose
Giuseppe Roberto Bonfitto: Nothing to disclose

**Green Imaging Revolution: Pioneering Sustainable Practices in Radiology for a Healthier Planet**

*A. A. Bherwani*[1], M. Sudds[2], L. Townsend-Sanders[3], A. Prieto Valero[4]; [1]Orlando, FL/US, [2]Wymondham/UK, [3]Tampa, FL/US, [4]Madrid/ES
*(anand.bherwani@gmail.com)*

**Purpose or Learning Objective:** This study highlights the critical need to incorporate sustainability in radiology, reducing the environmental impact without compromising diagnostic accuracy or patient care. Focusing on energy-efficient practices, waste reduction, and technological innovations in modalities like CT, MRI, and general radiography, the objective is to integrate eco-friendly strategies into radiological departments as part of broader healthcare sustainability goals.
**Methods or Background:** A comprehensive literature review of over 90 sources, including peer-reviewed articles, books, and case studies, was conducted. This research explores innovations such as energy-saving modes in MRI and CT, digital radiography's reduction of chemical waste, and sustainable contrast media practices. Case studies from several healthcare institutes globally are analyzed, illustrating real-world applications of green radiology practices.
**Results or Findings:** Adopting energy-efficient imaging systems and optimizing resource use led to significant environmental and financial benefits. For example, Singapore General Hospital reduced energy consumption by 30%, saving $1.2 million annually, while Mayo Clinic enhanced wastewater treatment, minimizing environmental contamination from contrast media. These practices and many others from various other institutions not only reduced greenhouse gas emissions but also operational costs.
**Conclusion:** Incorporating sustainable practices in radiology is vital for reducing healthcare's environmental footprint while maintaining high standards of patient care. This research provides a scalable framework for radiology departments worldwide, illustrating how integrating sustainability can enhance operational efficiency and contribute to global health equity and environmental conservation.
**Limitations:** This study is based on case studies from select institutions, which may not reflect the diverse resources and regulatory environments of all healthcare systems. Additionally, the long-term impacts of implementing these sustainable radiology practices require further investigation.
**Funding for this study:** Not Applicable
**Ethics committee - additional information:** Not Applicable
**Author Disclosures:**
Laurie Townsend-Sanders: Nothing to disclose
Alicia Prieto Valero: Nothing to disclose
Anand Ashok Bherwani: Employee: GE Healthcare Pharmaceutical Diagnostics
Michael Sudds: Nothing to disclose

**Centargo: Insights from the CIMROD Experiment on Contrast Consumption, Efficiency, Patient Care and environmental impact**

Y. Anquetil[1], *T. Leturgez*[2], J. E. Jacquin[1], F. Kruta[3], F. Jambon[1]; [1]Perigueux/FR, [2]La Garenne Colombes/FR, [3]Paris/FR
*(thibaut.leturgez@bayer.com)*

**Purpose or Learning Objective:** With the transition in France's contrast product supply model on March 1, 2024, the functionalities of CT injectors have been re-evaluated. This study assesses the impact of Centargo injectors on contrast media volume, patient care, preparation time, and environmental impacts at the CIMROD center, which acquired two Centargo injectors.
**Methods or Background:** To collect pertinent data, we conducted three measurements and utilized five data sources. The private hospital, equipped with two CT machines, transitioned from a single-use Stellant injector to a multi-use system, ultimately adopting the Centargo injector with enhanced features. Data sources included the volume of contrast media administered per patient, the time radiographers allocated to various tasks in their routines, protocol preparation and traceability times, and waste weight measurements. The evaluation period spanned from April to September and incorporated different injector configurations as well as a tagging system, Ubudu, to monitor workflow efficiency among radiographers.
**Results or Findings:** CIMROD aimed for an average contrast volume of 80 mL using Ultravist 370. The Centargo injector successfully achieved an average of 72.08 mL per injection across 1,099 procedures, and after further optimization, this volume decreased to 68.31 mL, resulting in a 15% reduction compared to the initial target. Workflow improvements included a 50% reduction in injector preparation time, a 14% increase in patient care time, and a 6% increase in image preparation time. Time savings of 16% per exam were noted, with waste production decreasing by 66% compared to the single-use Stellant, equating to an annual reduction of 1.4 tonnes.
**Conclusion:** The findings confirm the benefits of the Centargo injector, especially when combined with Smart Protocol and Nautilus software, RIS-Injector connectivity, and kVp optimization, leading to enhanced operational efficiency and reduced environmental impact.
**Limitations:** Single center evaluation.
**Funding for this study:** Bayer funded
**Ethics committee - additional information:** The experimentation isn't collecting patient level data so we didn't submitted it to an ethical committee.
**Author Disclosures:**
Jerome Elisabeth Jacquin: Nothing to disclose
François Kruta: Equipment Support Recipient: Ubudu
Yohan Anquetil: Equipment Support Recipient: CIMROD
Thibaut Leturgez: Employee: Bayer
François Jambon: Equipment Support Recipient: CIMROD

**Success factors for implementing an intervention using return letters for low-value MRIs**

*I. Ø. Brandsæter*, E. Kjelle, E. R. Andersen, B. M. Hofmann; Gjøvik/NO
*(Ingrid.o.brandsater@ntnu.no)*

**Purpose or Learning Objective:** The study aimed to investigate key stakeholders' experiences with and reflections on success factors for implementing an intervention using return letters to reduce the use of three low-value magnetic resonance imaging (MRI) examinations.
**Methods or Background:** An intervention to reduce low-value MRI was designed and implemented in private imaging centres in Norway in October 2022. The intervention used return letters based on Choosing Wisely recommendations for poor referrals for MRI of the lower back, brain and knee sent to imaging centres. Two semi-structured individual interviews were conducted with the medical directors of the two included imaging providers (radiologists) and two focus group interviews with nine managers from the various private imaging centres (radiographers) operated by the two imaging providers were conducted. Inductive content analysis in three steps was used to analyse the data.
**Results or Findings:** The analysis resulted in five categories: general experience, anchoring, organisation, return letter procedure and outcome. In general, the intervention was well received. Sufficient information, anchoring and support from the organisation's leaders were identified as crucial success factors. However, there were some barriers to the implementation, e.g. the medical directors in charge of the implementation found it hard to be hands-on and distribute information to the radiogpraphers at the imaging centres. Additionally, some Choosing Wisely recommendations were found vague and difficult to use by the radiographers doing the referral assessment.
**Conclusion:** This study provides insights into the practical and crucial details of implementing interventions to reduce low-value imaging using Choosing Wisely recommendations. The intervention was generally well received, and several key success factors were identified.
**Limitations:** The limitations of the study are that only managers and medical directors were included in the study, interviewing referrers and patients would have provided other important perspectives.
**Funding for this study:** Funding was provided by the Research Council of Norway (Project number 302503).
**Ethics committee - additional information:** Ethical approval is unnecessary according to national regulations in Norway (LOV-2008–06-20–44), and this study was not submitted to the regional committees for medical and health research ethics. The Norwegian Agency for Shared Services in Education and Research approved the processing and storage of personal information in this study (Ref. 974188)
**Author Disclosures:**
Ingrid Øfsti Brandsæter: Nothing to disclose
Bjørn Morten Hofmann: Nothing to disclose
Elin Kjelle: Nothing to disclose
Eivind R. Andersen: Nothing to disclose

**Quantifying Energy Savings in Radiology: A Simple Approach to Make the Radiology Department More Sustainable**

*K. Iaccarino*, D. Fazzini, S. Papa, M. Ali; Milan/IT
*(iaccarinokatia@gmail.com)*

**Purpose or Learning Objective:** To quantify the energy consumption of radiology reporting stations and explore a hypothetical scenario to mitigate energy waste in a healthcare setting.
**Methods or Background:** We monitored 10 reporting stations over a period of 90 days at the Centro Diagnostico Italiano (Milan, Italy). An energy logger was installed on each station to measure real-time power consumption, capturing data on both active and idle states. Stations were configured to enter stand-by mode after 4 hours of inactivity. We conducted a simulation to assess the impact of shutting down the stations after 1 hour of inactivity instead of allowing them to remain in stand-by mode.
**Results or Findings:** The overall power consumption of the 10 reporting stations was approximately 16,615.65 kWh, which is equivalent to about 6 households in Italy (average annual consumption of 2,700 kWh per household). We identified three main power consumption patterns: mainly-off, mainly-on, and always-off. The estimated on-mode consumption was 12,738.44 kWh per year, while stand-by consumption was 3,128.13 kWh and off-mode consumption was 749.06 kWh. By implementing the hypothetical scenario of shutting down after 1 hour of inactivity, we estimated potential energy savings of around 2,346.12 kWh/year. Considering that CDI has a total of 53 reporting stations across all sites, the estimated total annual energy savings would be approximately 12,434.44 kWh.
**Conclusion:** Optimizing energy usage in radiology departments is essential for promoting sustainability. Simple configuration changes can lead to significant reductions in energy waste, enhancing the environmental responsibility of healthcare facilities.
**Limitations:** The sample size of 10 reporting stations may not be representative of the entire radiology department, and the 90-day monitoring period might not account for seasonal variations in usage. The hypothetical scenario assumes uniform behavior across stations, which may not reflect actual workflows.
**Funding for this study:** None
**Ethics committee - additional information:** The ethics commettee approval is not applicable for this study
**Author Disclosures:**
Marco Ali: Consultant: Bracco Imaging S.p.A.
Sergio Papa: Nothing to disclose
Deborah Fazzini: Nothing to disclose
Katia Iaccarino: Nothing to disclose

**16:00-17:30** **Research Stage 1**

### Research Presentation Session: Head and Neck

### RPS 2108

### A journey through thyroid imaging

**Moderator**
E. Gotsiridze; Tbilisi/GE
*(gotsiridze.elene@gmail.com)*

### Additional Value of Pertechnetate Scintigraphy to ACR-TIRADS and EU-TIRADS for Thyroid Nodule Classification in Euthyroid Patients

L. Sollmann, M. Eveslage, M. Danzer, M. Schäfers, B. Heitplatz, D. Hescheler, B. Riemann, *B. Noto*; Münster/DE
*(benjamin.noto@ukmuenster.de)*

**Purpose or Learning Objective:** Thyroid nodules are a highly prevalent, predominantly benign finding, yet their accurate evaluation remains challenging. While ultrasound and TIRADS are now widely accepted as standard in evaluation, the utility of thyroid scintigraphy in euthyroid patients remains debated. Previous studies have investigated the diagnostic potential of TIRADS or radionuclide scanning in isolation, but an integrated approach has not been explored so far. This study aimed to evaluate if pertechnetate scintigraphy enhances the diagnostic value of TIRADS in a multimodal framework.
**Methods or Background:** The diagnostic capabilities of ACR-TIRADS, EU-TIRADS, pertechnetate scintigraphy, and multimodal models were retrospectively analyzed for 322 nodules (231 benign, 91 malignant) in 208 euthyroid patients undergoing thyroidectomy. Statistical analysis employed generalized estimating equations.
**Results or Findings:** Thyroid scintigraphy demonstrated an AUC of 0.6 (95%CI:0.55-0.66), ACR-TIRADS of 0.84 (95%CI:0.79-0.89) and EU-TIRADS of 0.78 (95%CI: 0.72-0.83). Integrating thyroid scintigraphy with ACR-TIRADS enhanced diagnostic accuracy, yielding an AUC of 0.86 (p=0.039). Similarly, combining thyroid scintigraphy with EU-TIRADS resulted in an AUC of 0.80 (p =0.008), surpassing the individual TIRADS performances. Furthermore, the integration of thyroid scintigraphy adjusted the malignancy probability among TIRADS categories. Iso- or hyperfunctioning nodules in ACR-TIRADS TR4 and hypofunctioning nodules in TR3 exhibited comparable probabilities of malignancy. Similarly, iso- or hyperfunctioning nodules in EU-TIRADS 4 showed similar malignancy probabilities to hypofunctional nodules in EU-TIRADS 3, indicating the potential for more refined risk stratification.
**Conclusion:** This study demonstrates enhanced diagnostic performance achieved by integrating thyroid scintigraphy with ACR—and EU-TIRADS for classifying thyroid nodules in euthyroid patients. Such a multimodal approach could improve risk stratification and management decisions, particularly in complex scenarios like multinodular goiter. Further research is warranted to validate these findings and explore their clinical implications.
**Limitations:** Retrospective design
**Funding for this study:** The Medical Faculty, University of Münster, Germany supported B.N. as a clinician scientist.
**Ethics committee - additional information:** The study protocol was approved by the ethics committee of the University of Munster and performed in accordance with the ethical standards as laid down in the 1964 Declaration of Helsinki and its later amendments.
**Author Disclosures:**
Michael Schäfers: Nothing to disclose
Moritz Danzer: Nothing to disclose
Barbara Heitplatz: Nothing to disclose
Lea Sollmann: Nothing to disclose
Burkhard Riemann: Nothing to disclose
Daniel Hescheler: Nothing to disclose
Benjamin Noto: Nothing to disclose
Maria Eveslage: Nothing to disclose

### Subtraction ultrasound microangiography for assessment of microvascularity patterns in diffuse thyroid disease

*A. Borlea*, D. I. Stoian; Timisoara/RO
*(andreea.brl3@gmail.com)*

**Purpose or Learning Objective:** To evaluate the use of Subtraction Ultrasound Microangiography (SUMA) in the quantitative assessment of microvascularity patterns in diffuse thyroid diseases and to compare these findings with normal thyroid tissue.
**Methods or Background:** Conventional color Doppler ultrasound is limited by its subjective nature in assessing thyroid vascularity. SUMA provides a quantitative approach through color pixel percentage (CPP) measurement. A cohort of 220 subjects was studied, including 90 with autoimmune thyroiditis, 20 with Graves' disease, and 110 normal controls. SUMA was used to quantify CPP, and results were analyzed to identify differences between the groups. The relationship between TSH levels and CPP was also explored.
**Results or Findings:** Significant differences in CPP were observed across the groups. Normal controls (euthyroid) demonstrated a median CPP of 26% (IQR 18-37%), Graves' disease patients with clinical and subclinical hyperthyroidism had a median CPP of 75% (IQR 55-82%), and those with untreated hypothyroid Hashimoto thyroiditis (hypothyroid) had a median CPP of 63% (IQR 49-75%). A U-shaped relationship was found between TSH levels and CPP, indicating alterations in vascularity in both hypo- and hyperthyroid states.
**Conclusion:** SUMA allows for a more objective and quantitative assessment of thyroid microvascularity, revealing distinct differences between normal, autoimmune thyroiditis, and Graves' disease tissues. The technique may improve diagnostic accuracy compared to conventional Doppler ultrasound.
**Limitations:** Further studies are needed to validate SUMA's diagnostic potential, including its reproducibility across different ultrasound systems and settings, as well as its sensitivity in detecting disease progression or treatment response.
**Funding for this study:** The study received no external funding
**Ethics committee - additional information:** Victor BAbes University of Medicine and Pharmacy
**Author Disclosures:**
Dana I Stoian: Nothing to disclose
Andreea Borlea: Nothing to disclose

### Ultrasound-guided microwave ablation versus thyroidectomy for the treatment of solitary nodular retrosternal goiter

*Y. Li*, Y. Luo, M. Zhang; Beijing/CN
*(Lyy19900325@sina.com)*

**Purpose or Learning Objective:** The purpose of this study was to compare the clinical outcomes of microwave ablation versus thyroidectomy for patients with solitary nodular retrosternal goiter (RSG).
**Methods or Background:** This retrospective study evaluated 243 patients with solitary nodular RSG treated with MWA (M group, n=111) or thyroidectomy (T group, n=132). Complications, thyroid function, and treatment variables, including procedure time, estimated blood loss, hospitalization, and cost, were compared. The volume, volume reduction rate, symptoms, and cosmetic score were also evaluated in the W group.
**Results or Findings:** The rate of overall complications, transient RLN injury and hypothyroidism was 35.6%, 9.1%, and 10.6% in the T group, respectively, but these complications were 7.2%, 2,7%, and 0 in the M group (all P<.05). Patients in the M group had a significantly shorter procedure time (median, 6.48 versus 95.0 minutes, P<.0001), less estimated blood loss (0 versus 50 ml, P<.0001), and lower cost (US ＄1541.25 versus ＄2839.40, P<.0001) than those treated by thyroidectomy. After MWA, the VRR was 76.31% and 89.37% at the 12 months and last follow-up time, respectively. Of all the nodules treated by MWA, 50 (45%) received additional ablation. The symptom and cosmetic scores were both significantly reduced at the last follow-up.

**Conclusion:** MWA is an effective and safe treatment for solitary nodular RSG. Moreover, MWA is associated with a faster recovery, fewer complications, and superior esthetic results relative to thyroidectomy and it may be a potential alternative to surgery in selected patients, especially for those who are ineligible or unwilling to receive surgical treatment.
**Limitations:** This study was carried out in a single center, and multicenter studies are warranted.
**Funding for this study:** This study recieved no funding.
**Ethics committee - additional information:** Ethical approval was obtained from the Institutional Ethics Committee of the Chinese PLA General Hospital.
**Author Disclosures:**
Yingying Li: Nothing to disclose
Mingbo Zhang: Nothing to disclose
Yukun Luo: Nothing to disclose

**Mid-term thyroid function alterations as predictors of long-term outcomes in radiofrequency ablation of benign thyroid nodules**
*Y-H. Chen*[1], P-L. Chiang[1], Y-H. Chang[2], C-K. Chou[2], W-C. Lin[1]; [1]Kaohsiung/TW, [2]Kaohsiung City/TW
*(yh19970718@gmail.com)*

**Purpose or Learning Objective:** Radiofrequency ablation (RFA) has gained recognition as a highly effective treatment for benign thyroid nodules. While thyroid function alterations have been observed during post-RFA follow-up, this study specifically focuses on the potential link between changes in thyroid function and volume reduction ratio of treated nodules. Additionally, it seeks to evaluate whether fluctuations in thyroid function at mid-term follow-up can serve as early indicators for the development of long-term hypothyroidism following RFA.
**Methods or Background:** In this retrospective study, 50 euthyroid individuals (mean age = 47.0 years; 43 females, 7 males) with a total of 72 benign thyroid nodules (median volume = 4.61 mL) undergoing RFA were evaluated. Comprehensive assessments, including clinical examinations, ultrasound imaging, and blood tests, were conducted at specific intervals (pre-RFA, and at 6 months, 12 months, and annually post-RFA).
**Results or Findings:** The mean follow-up period was 22.3 months. Both medium-term and long-term follow-ups revealed significant reductions in T3 levels ($p<0.001$, $p=0.005$) and elevations in TSH levels ($p<0.001$, $p<0.001$) compared to baseline measurements. A negative correlation was found between medium-term T3 levels and long-term volume reduction ratio ($r=-0.332$, $p=0.005$). Furthermore, patients with lower T3 levels during medium-term follow-up demonstrated a significantly higher long-term volume reduction ratio compared to those with higher T3 levels (0.92 vs. 0.77, $p=0.017$).
**Conclusion:** Following radiofrequency ablation, notable unexpected alterations in thyroid function were observed, without meeting the criteria of hypothyroidism. Additionally, a lower mid-term T3 level may be indicative of a better volume reduction ratio during long-term follow-up.
**Limitations:** The study is limited by biases associated with its retrospective design and a lack of short-term data within a six-month period. To address these limitations, further prospective studies with increased focus on short-term data are necessary.
**Funding for this study:** No funding was received for this study.
**Ethics committee - additional information:** This study received approval from the institutional review board under the reference number 202200189B0.
**Author Disclosures:**
Wei-Che Lin: Nothing to disclose
Yen-Hsiang Chang: Nothing to disclose
Pi-Ling Chiang: Nothing to disclose
Yi-Han Chen: Nothing to disclose
Chen-Kai Chou: Nothing to disclose

**Comparision of K-TIRADS, EU-TIRADS and ACR-TIRADS Guidelines for Malignancy Risk Determination of Thyroid Nodules**
*E. Tobcu*, E. Karavaş, G. Taşova Yılmaz, Z. Tobcu; Balıkesir/TR
*(etobcu@bandirma.edu.tr)*

**Purpose or Learning Objective:** To evaluate the performances of three internationally recognized thyroid imaging reporting and data systems (TIRADS) for risk stratification of malignancy in comparison to one another.
**Methods or Background:** A total of 225 thyroid nodules with definitive FNAB cytology or histopathological diagnoses were included in the study. Various ultrasound (US) features were classified into categories based on three TIRADS editions. The guidelines were assessed regarding sensitivity, specificity, predictive values, and diagnostic accuracy to compare diagnostic value.
**Results or Findings:** American College of Radiology (ACR)-TIRADS demonstrated the best diagnostic accuracy (63.1%), the highest specificity (58.7%), and positive predictive value (36.3%) among three different TIRADS systems. Korean (K)-TIRADS exhibited the highest sensitivity (94.2%), negative predictive value (96.1%), and the most favorable negative likelihood ratio (0.13). The European (EU)-TIRADS had a sensitivity of 90.4%, specificity of 48.6%, and diagnostic accuracy of 58.2%, ranking between the other two guidelines across most parameters.
**Conclusion:** The rigorous use of the guidelines established by each of the three TIRADS systems would have markedly reduced the number of FNABs performed. The comparison of the three guidelines in our study indicated that they are effective screening methods for identifying malignant thyroid nodules. Among them, K-TIRADS showed the most effective diagnostic performance in sensitivity, while ACR-TIRADS yielded the best specificity.
**Limitations:** The main limitation of our study was its single-center design. Another limitation of our study was the lack of histopathological diagnoses for all nodules. All nodules with malignant cytological results underwent surgery that enables us to reach histopathological diagnoses, but only two nodules with benign cytology underwent surgery due to the clinician's discretion and the patient's preference. Cytology results were used as a reference standard diagnosis in the remaining nodules.
**Funding for this study:** No funding
**Ethics committee - additional information:** The study was performed in accordance with the ethical guidelines of the Helsinki Declaration and approved by the local ethics review committee (2024-3).
**Author Disclosures:**
Eren Tobcu: Nothing to disclose
Erdal Karavaş: Nothing to disclose
Gülden Taşova Yılmaz: Nothing to disclose
Zeynep Tobcu: Nothing to disclose

**Thyroid nodule characterization: interobeserver evaluation of different TIRADS with and without AI software**
*C. Di Bella*[1], E. David[2], C. Solito[1], V. Dolcetti[1], P. Pacini[1], G. Del Gaudio[1], M. Renda[1], C. Catalano[1], V. Cantisani[1]; [1]Rome/IT, [2]Catania/IT
*(chiaradibella30@gmail.com)*

**Purpose or Learning Objective:** To evaluate the diagnostic performance of CAD compared with TI-RADS systems and to compare the performance of TI-RADS when used by operators with different levels of experience.
**Methods or Background:** Three operators with different levels of experience evaluated 484 thyroid nodules and the diagnostic accuracy of three risk stratification systems (ACR-, EU-, K-TIRADS) and CAD software (S-Detect) in characterizing the nodules. Nodules were characterized and stratified by using the three TIRADS systems; then S-detect software was applied and the data were compared with each other and with the gold standard (citology).
**Results or Findings:** The sensitivity of the human operator measurement and the negative predictive value (NPV) is 100%, for all three types of TIRADS. The positive predictive value (PPV) is 50%. The specificity is 78.4% (EU), 85.7% (ACR), 89% (K); this implies a certain proportion of "false positives", especially in the use of the tirads EU. The sensitivity of the measurement of s-detect alone is 66.7%, for all TRAIDS. This estimate implies a low certainty of the negative result. The NPV is 96.2% (ACR, K) and 96.3% (EU). The PPV is 50% (ACR-K) and 66.7% (EU). The specificity is higher than the sensitivity: 92.7% (ACR - K) and 96.3% (EU).
**Conclusion:** S-DETECT combined with EU-TIRADS has similar results as S-DETECT with ACR- and K- TIRADS in terms of sensitivity, specificity and NPV. However, it has a slightly better PPV, suggesting greater accuracy in correctly diagnosing positive cases than the ACR- and K-classification systems. S-Detect cannot yet be considered a substitute for the human operator but is a valuable tool for characterizing thyroid nodules, when integrated with radiologist evaluation and for support tool for less experienced operators and in doubtful cases
**Limitations:** Reduced patient sample. Ultrasound's operator dependence
**Funding for this study:** None
**Ethics committee - additional information:** Ethics committee "Sapienza"
**Author Disclosures:**
Giovanni Del Gaudio: Nothing to disclose
Maurizio Renda: Nothing to disclose
Patrizia Pacini: Nothing to disclose
Chiara Di Bella: Nothing to disclose
Vincenzo Dolcetti: Nothing to disclose
Carmen Solito: Nothing to disclose
Vito Cantisani: Nothing to disclose
Emanuele David: Nothing to disclose
Carlo Catalano: Nothing to disclose

**Is Shear-Wave Elastography an Accurate Tool for Evaluating Nodules in Patients with Autoimmune Thyroiditis?**

*D. I. Stoian*, A. Borlea; Timisoara/RO
*(stoian.dana@umft.ro)*

**Purpose or Learning Objective:** To evaluate the diagnostic accuracy of Shear Wave Elastography (SWE) in differentiating benign from malignant nodules in patients with chronic autoimmune thyroiditis.
**Methods or Background:** SWE has been widely studied in assessing thyroid nodules, but the background of thyroid lymphocitic infiltration and fibrosis may impact diagnostic performance. This study enrolled 130 subjects aged 18-84 years with a previous diagnosis of autoimmune thyroiditis and thyroid nodules. SWE measurements were performed on both thyroid parenchyma and nodules, assessing elasticity indices (EIs), including mean and maximum values, and the nodule-to-thyroid (N/T) ratio. Conventional ultrasound risk assessment using TIRADS was also evaluated. The relationship between elasticity indices and biochemical parameters and thyroid volume was examined.
**Results or Findings:** There were no statistically significant differences in thyroid function or autoimmunity parameters between benign and malignant nodules. Significant differences were found in TIRADS scores ($p < 0.0001$), mean nodule EI ($p < 0.0001$), and N/T shear wave ratio ($p < 0.0001$). The mean nodule EI (47.2 kPa for malignant vs. 18.1 kPa for benign nodules) had the highest diagnostic performance, significantly outperforming both the maximum EI ($p = 0.0360$) and the N/T ratio ($p = 0.0130$). The mean nodule EI also demonstrated superior diagnostic accuracy compared to TIRADS ($p = 0.0025$).
**Conclusion:** SWE demonstrates high diagnostic accuracy in evaluating nodules also in context of autoimmune thyroiditis. The mean nodule EI is the most reliable elastographic parameter, outperforming other elasticity indices and TIRADS in distinguishing malignant from benign nodules.
**Limitations:** This study did not include a control group of nodules without autoimmune thyroiditis, limiting comparisons of SWE performance in different thyroid backgrounds. Further studies are needed to assess reproducibility across other clinical settings.
**Funding for this study:** There was no funding for the study
**Ethics committee - additional information:** The studies involving humans were approved by The Ethics Committee of the Victor Babes University of Medicine and Pharmacy. The studies were conducted in accordance with the local legislation and institutional requirements. The participants provided their written informed consent to participate in this study.
**Author Disclosures:**
Dana I Stoian: Nothing to disclose
Andreea Borlea: Nothing to disclose

**Follow up or FNAB?: Malignancy Rates of ACR-TIRADS 4 and 5 Thyroid Nodules <10mm in Diameter**

*E. Tobcu*, E. Karavaş, Z. Tobcu, G. Taşova Yılmaz; Balıkesir/TR
*(etobcu@bandirma.edu.tr)*

**Purpose or Learning Objective:** The purpose of this study is to ascertain the rate of malignancy in nodules that are <10 mm in diameter in the TR-4 and TR-5 categories, as defined by the American College of Radiology Thyroid Imaging and Reporting Data System (ACR-TIRADS) 2017 whitepaper.
**Methods or Background:** Assessment of thyroid nodules was conducted in accordance with the 2017 whitepaper of the ACR. The fine-needle aspiration biopsy (FNAB) procedure was performed under the guidance of ultrasound. Lesions designated as Bethesda group 3, 4, 5, and 6 were classified as "non-benign group," while nodules defined as Bethesda group 2 were classified as "benign group". All patients underwent surgery, except for those with benign cytology.
**Results or Findings:** A total of 60 nodules were included in the study. Based on cytological analysis, 12 of the 15 nodules classified in the TR-4 category were determined to be benign thyroid nodules, while 3 nodules were classified as "suspicious for malignancy" (Bethesda-V) (%20) after cytological assessment. In the assessment of 45 TR-5 nodules, 30 were identified as non-benign (66.6%), while the remaining 15 were classified as benign thyroid nodules after cytological evaluation.
**Conclusion:** Our investigation demonstrated that <10mm thyroid nodules classified in categories TR-4 and TR-5, according to ACR-TIRADS 2017 guideline, have a malignancy rate of 55%. We think that FNAB should be performed prior to the decision of active surveillance, in order to determine the PMTCs that exhibit aggressive cytologic features and to identify those with benign cytology to avoid an unnecessary active surveillance process.
**Limitations:** The research was conducted with a small number of patients and was completed at a single center. Second, we did not exclude patients with thyroiditis that may have an impact on the accuracy of FNAB.
**Funding for this study:** The authors state that this study has not received any funding
**Ethics committee - additional information:** This prospective study was approved by the institutional review board of our in¬stitution (decision number: 2024/3-40), and any requirement of informed consent was waived.
**Author Disclosures:**
Eren Tobcu: Nothing to disclose
Erdal Karavaş: Nothing to disclose
Gülden Taşova Yılmaz: Nothing to disclose
Zeynep Tobcu: Nothing to disclose

**Evaluating the diagnostic value of arterial enhancement fraction from dual-layer spectral detector CT in lymph node metastasis of papillary thyroid carcinoma**

*L. L. Ye*[1], X. Zheng[1], Y. Liao[2]; [1]Dongguan/CN, [2]Guangzhou/CN
*(13412192434@163.com)*

**Purpose or Learning Objective:** To assess the diagnostic value of the arterial enhancement fraction (AEF) derived from dual-layer spectral detector CT scans in detecting lymph node metastasis in papillary thyroid carcinoma (PTC).
**Methods or Background:** Preoperative spectral CT images of 58 lymph nodes from 25 PTC patients (7 males, 18 females; aged 28-74) confirmed by surgery and pathology were analyzed. All patients underwent lymph node dissection. Lymph nodes were classified into metastatic (N = 24) and non-metastatic (N = 34) groups based on pathology. AEF (defined as iodine concentration in the arterial phase / iodine concentration in the venous phase) was measured. The Mann-Whitney U test was used to compare AEF between groups. ROC analysis evaluated AEF's predictive performance for lymph node metastasis.
**Results or Findings:** AEF was significantly higher in metastatic lymph nodes than in non-metastatic ones ($p<0.05$). In terms of differentiating lymph node metastasis, the AUC of AEF is 0.737 (95%CI 0.582-0.891), with an accuracy of 0.810, a sensitivity of 0.583, and a specificity of 0.971.
**Conclusion:** AEF from dual-layer spectral detector CT demonstrates significant differences between metastatic and non-metastatic lymph nodes in PTC and has diagnostic potential for identifying metastatic lymph nodes, providing a basis for precise preoperative treatment planning.
**Limitations:** Not applicable
**Funding for this study:** Not applicable
**Ethics committee - additional information:** Not applicable
**Author Disclosures:**
Xiaolin Zheng: Consultant: mentor
Li Li Ye: Speaker: speaker, main person in charge of abstract
Yuting Liao: Other: statistical researcher, unpaid

**Diagnostic of intravoxel incoherent motion diffusion-weighted imaging histogram parameters in distinguishing between benign and malignant thyroid nodules**

*X. Li*, Y. Yue, J. Ren; Beijing/CN
*(lxp17634986228@163.com)*

**Purpose or Learning Objective:** To explore the diagnostic efficacy of histogram parameters of IVIM in differentiating benign and malignant thyroid nodules.
**Methods or Background:** A total of 51 patients with thyroid nodules were retrospectively included from March 2017 to September 2022, including the benign group 24 cases and malignant group 27 cases . All results were confirmed by surgical pathology. multiple b-value small field diffusion weighted images were collected to generate true diffusion coefficient(D), pseudo diffusion coefficient(D*), and perfusion fraction(f) images. At the same time, an apparent diffusion coefficient(ADC) image with a b-value of 990s/mm2 was generated. Manually outline the volume of interest(VOI) of the entire lesion. Using a self-designed program in Matlab, calculate the histogram parameters of the D, D *, and f at VOI, and calculate the mean ADC at VOI. Compare the intergroup differences between IVIM histogram parameters and ADC mean. Using multiple logistic regression, further select the optimal parameters and establish a multi parameter joint model and verify and compare the diagnostic performance of different models.
**Results or Findings:** The statistically significant differences between benign and malignant thyroid nodules are as follows($P<0.05$): gender, ADC mean, 5th, 15th, 85th, and 95th percentile, mean, skewness, and root mean square difference in the D-plot, 5th and 15th percentile percentile, mean, skewness, and coefficient of variation in the D * plot. The AUC values for skewness, 15th percentile, and root mean square difference of the D-plot combined with gender were 0.94, with a sensitivity of 88.46% and a specificity of 90.91%. The AUC value of the mean ADC combined with gender is 0.86, with a sensitivity of 77.78% and a specificity of 87.50%.
**Conclusion:** Histogram parameters of IVIM has good diagnostic value in distinguishing benign and malignant thyroid nodules.
**Limitations:** Not applicable.

Saturday

**Funding for this study:** Not applicable.
**Ethics committee - additional information:** This is a retrospective study.
**Author Disclosures:**
Yunlong Yue: Nothing to disclose
Xingpeng Li: Nothing to disclose
Jie Ren: Nothing to disclose

**New thyroid imaging reporting and data system (TI-RADS) based on ultrasonography features for follicular thyroid neoplasms: A multicenter study**
*Y. Zhang*; Shanghai/CN
*(zhangyifeng@tongji.edu.cn)*

**Purpose or Learning Objective:** This study aimed to establish a new risk stratification system for FTN and new methods for non-invasive and practical preoperative evaluation of thyroid follicular tumors to reduce missed diagnoses and unnecessary biopsies.
**Methods or Background:** 535 FTNs of 535 patients from four hospitals were included in this retrospective study. All the nodules were randomly divided into test and validation groups. FTN-TIRADS was established based on the results of univariate analysis and logistic regression of ultrasonography features in the test group. Each nodule was evaluated and classified by existing risk stratification systems (EU-RSS, ATA-RSS, ACR-TIRADS, Chinese TIRADS [C-TIRADS]) and FTN-TIRADS. The diagnostic value of FTN-TIRADS in the validation group was verified and compared with the test group and the other four risk stratification systems. The unnecessary rates of fine needle aspiration (FNA) of FTN-TIRADS and the other four risk stratification systems were compared, too.
**Results or Findings:** Test group and validation group included 370 patients and 165 patients. The following features were independent risk factors and included in FTN-TIRADS: nodule composition, echogenicity, calcifications, halo sign, and indistinct boundary with thyroid capsule. The AUC of FTN-TIRADS was 0.855, statistically higher than EU-RSS, ATA-RSS, ACR-TIRADS, and C-TIRADS (0.759, 0.759, 0.753, 0.677, respectively). The FTN-TIRADS of the validation group had a similar diagnostic performance. The unnecessary FNA rate of the FTN-TIRADS was 26.0%, which was significantly lower than that of EU-RSS (79.9%), ATA-RSS (92.5%), ACR-TIRADS (55.8%) and C-TIRADS (62.2%).
**Conclusion:** FTN-TIRADS achieved better differential diagnosis of FTN than current risk stratification systems and significantly reduced the rate of unnecessary FNA.
**Limitations:** The study was performed retrospectively; a more conclusive validation could be achieved using a prospective design that better captured the diverse characteristics of actual clinical cases.
**Funding for this study:** This work was supported by the National Natural Science Foundation of China (Grants No. 81927801, 81725008, 81772849, 82171942, and 82371971)
**Ethics committee - additional information:** The institutional review board of the university-affiliated hospital approved this retrospective study (approval number 22K82)
**Author Disclosures:**
Yifeng Zhang: Nothing to disclose

| 16:00-17:30 | Research Stage 2 |
| --- | --- |

### Research Presentation Session: Oncologic Imaging

### RPS 2116

### Advances in imaging genitourinary cancer

**Moderator**
T. Akbas; Istanbul/TR
*(tugana.akbas@acibadem.com)*

**Total bone diffusion volume on whole-body diffusion-weighted imaging is a strong prognostic marker of disease survival in metastatic castration-resistant prostate cancer**
*L. D'Erme*[1], A. Candito[2], G. Avesani[1], S. Bottazzi[1], R. Emsley[2], D. Meo[2], J. Carmichael[2], N. Tunariu[2], D-M. Koh[2]; [1]Rome/IT, [2]London/UK
*(lucaderme@icloud.com)*

**Purpose or Learning Objective:** To investigate the relationship between total bone diffusion volume (tBDV) and global apparent diffusion coefficient (gADC) derived from whole-body diffusion-weighted MRI (WBDWI); as well as automatic bone scan index (aBSI) derived from bone scintigraphy with disease overall survival (OS) in metastatic castration-resistant prostate cancer (mCRPC).
**Methods or Background:** In this IRB approved study, we retrospectively reviewed 302 mCRPC patients (Jan 2015 - Dec 2023) who underwent baseline WBDWI before systemic anticancer treatment. Segmentation masks of bone disease on b900 WBDWI images were generated by an automated tool, and refined by a 3-year experienced oncological radiologist, to derive the tBDV and gADC values. The aBSI was derived in 265 patients with available baseline bone scintigraphy. Kaplan-Meier survival curves and log-rank tests for 5-years OS, including their hazard ratios (HR) and 95% confidence intervals (CI), were compared between the three groups, stratified by the median values of tBDV (89 mL), gADC (0.83), and aBSI (0.021). Significance was set at $p < 0.05$.
**Results or Findings:** Patients with tBDV < 89 mL demonstrated significantly longer OS compared to those with tBDV ≥ 89 mL (40.8 vs 23.7 months; $p < 0.0001$; HR 2.28, 95%CI 1.76–2.94). No significant survival difference was observed between the gADC groups (32.0 vs 27.2 months; $p = 0.4293$). OS was significantly longer in patients with aBSI < 0.021 than those with aBSI ≥ 0.021 (40.8 vs 24.4 months; $p < 0.0001$; HR 2.18, 95%CI 1.66–2.85).
**Conclusion:** WBDWI-derived tBDV is a strong independent prognostic marker for OS in mCRPC patients. The tBDV measurement is comparable if not superior to aBSI as a predictor of disease survival.
**Limitations:** Retrospective design and longitudinal imaging data were not considered.
**Funding for this study:** This study represents independent research funded by the National Institute for Health and Care Research (NIHR) Biomedical Research Centre at The Royal Marsden NHS Foundation Trust and The Institute of Cancer Research, London, and by the Royal Marsden Cancer Charity, and Cancer Research UK (CRUK) National Cancer Imaging Trials Accelerator (NCITA) and Prostate Cancer UK. The views expressed are those of the author(s) and not necessarily those of the NIHR or the Department of Health and Social Care. This work uses data provided by patients and collected by the NHS as part of their care and support.
**Ethics committee - additional information:** The study was approved by the Institutional Ethics Committee (no. 21/LO/0605).
**Author Disclosures:**
Giacomo Avesani: Nothing to disclose
Dow-Mu Koh: Nothing to disclose
Davide Meo: Nothing to disclose
Nina Tunariu: Nothing to disclose
Robby Emsley: Nothing to disclose
Silvia Bottazzi: Nothing to disclose
Antonio Candito: Nothing to disclose
Luca D'Erme: Nothing to disclose
Juliet Carmichael: Nothing to disclose

**Why we shouldn't trust CT in the evaluation of bone metastases in patients with metastatic prostate cancer: a comparison between pattern of changes on CT and bone metastases MET-RADS-P classification**
*S. Bottazzi*[1], L. Russo[1], G. Avesani[1], L. D'Erme[1], C. Messiou[2], D-M. Koh[2], E. Sala[1], N. Tunariu[2]; [1]Rome/IT, [2]Sutton/UK
*(silvia.bottazzi02@gmail.com)*

**Purpose or Learning Objective:** To compare changes on computed tomography (CT) with the MET-RADS-P response assessment categories (RAC) of bone metastases in advanced prostate cancer patients (APCb) during treatment.
**Methods or Background:** 102 patients (median age 68years, range 51-83) with APCb who underwent both CT and whole-body magnetic resonance imaging (WBMRI) within 30 days at baseline and during treatment were included. Up to five focal lesions > 10 mm per patient were selected based on one or more of the following: [1] Sclerotic, lytic or mixed lesion on CT; [2] active bone marrow lesion on WBMRI [3] newly developed CT or MR lesion. Each lesion was assigned a CT pattern of change - based on changes in size and Hounsfield Unit (HU) – and a RAC according to METRADS-P criteria. The CT patterns were corroborated with the RAC, grouped as response (RAC1-2), stable (RAC3), and progression (RAC4-5).
**Results or Findings:** 358 lesions were identified. Of these, 70% (252/358) were sclerotic (SL), 6% (21/358) lytic (LL) and 4% (13/258) mixed lesions (ML). 20% (72/358) showing MR characteristics of active bone metastases were undetectable on CT. The most frequent CT patterns of change on treatment were: stable SL (no changes in density or size) in 35.8% (128/358), increasing in size SL (>5 mm) in 11.4% (41/358), new SL (appeared during treatment) in 10.6% (38/358). Stable SL corresponded to the RAC classifications as follows: 22.3% (29/128) stable treated disease, 25.8% (33/128) responding, 23.4% progressing (30/128) and 28.1% (36/128) stable active disease. New SLs corresponded to responding disease in 28.9% (11/38).
**Conclusion:** 20% of bone metastases are occult on CT. A stable SL on CT is a poor predictor of disease status. Hence, CT appears unreliable in the assessment of bone disease response inAPCb
**Limitations:** Na

**Funding for this study:** This study represents independent research funded by the National Institute for Health and Care Research (NIHR) Biomedical Research Centre at The Royal Marsden NHS Foundation Trust and The Institute of Cancer Research, London, and by the Royal Marsden Cancer Charity, and Cancer Research UK (CRUK) National Cancer Imaging Trials Accelerator (NCITA) and Prostate Cancer UK. The views expressed are those of the author(s) and not necessarily those of the NIHR or the Department of Health and Social Care. This work uses data provided by patients and collected by the NHS as part of their care and support.
**Ethics committee - additional information:** Reference no. 21/LO/0605
**Author Disclosures:**
Giacomo Avesani: Nothing to disclose
Christina Messiou: Nothing to disclose
Dow-Mu Koh: Nothing to disclose
Nina Tunariu: Nothing to disclose
Silvia Bottazzi: Nothing to disclose
Evis Sala: Nothing to disclose
Luca D'Erme: Nothing to disclose
Luca Russo: Nothing to disclose

### Cancer Detection Rates in DWI-upgraded Transition Zone lesions align with risk assessment categories in PI-RADS v2.1: a Systematic Review and Meta-analysis

*G. Agrotis*, E. H. P. Pooch, R. G. H. Beets-Tan, I. G. Schoots; Amsterdam/NL
*(g.agrotis@hotmail.com)*

**Purpose or Learning Objective:** To assess and compare cancer detection rates (CDRs) of transition zone (TZ) lesions that were upgraded from PI-RADSv2.1 score 2 to 3("2+1") or from score 3 to 4("3+1") using diffusion-weighted imaging (DWI) and evaluate their clinical impact.
**Methods or Background:** A systematic literature search was conducted in Embase, Medline and Web of Science for studies evaluating TZ lesions with the use of DWI, with histology proven Grade Group≥2 cancer (GG≥2) as primary outcome. Pooled estimates for sensitivity, specificity, CDRs, and Odds Ratio (OR) were derived from extracted data at lesion level and quantitatively pooled using a bivariate binomial and random effects model.
**Results or Findings:** A total of 7 studies included 1,437 TZ lesions. GG≥2 CDRs for PI-RADSv2.1 scores of 1, 2, 2+1, 3, 3+1, 4, and 5 were respectively 2%[95% CI:0%-12%], 7%[4%-11%], 12%[6%-24%], 21%[18%-25%], 37%[23%-53%], 53%[33%-72%], and 86%[40%-98%]. GG≥2 CDRs of TZ scores '2+1' and '2' were statistically different, with OR 3.13[1.31-7.48],p=0.01, while '2+1' and '3' scores were not, with an OR of 0.76[0.42-1.33],p=0.34. GG≥2 CDRs of TZ score '3+1' and '3' were statistically different, with an OR of 2.3[1.07-4.95],p=0.03, while scores '3+1' and '4' were not with an OR of 0.63[0.28-1.38],p=0.25. Still, false positive rates were substantial in both subcategories ('2+1': 76%[73.8%-78.2%] and '3+1': 45%[42.4%-47.6%]).
**Conclusion:** The risk of having significant prostate cancer in ‘2+1’ and ‘3+1’ Transition Zone lesions, with an upgrading based on DWI images, is appropriately categorized within the PI-RADS v2.1 scoring system, as shown by this meta-analysis. Especially TZ lesions with score ‘3+1’ may impact individualized biopsy-decisions, as 2-in-5 harbor significant disease, similar to score ‘4’ lesions. Still, the high false positive rate in this sub-category emphasizes the need for strategies to minimize overdiagnosis.
**Limitations:** Data availability and population differences
**Funding for this study:** None
**Ethics committee - additional information:** Not applicable
**Author Disclosures:**
Georgios Agrotis: Nothing to disclose
Eduardo H. P. Pooch: Nothing to disclose
Regina G. H. Beets-Tan: Nothing to disclose
Ivo Gerardus Schoots: Nothing to disclose

### Evaluation of Arterial Enhancement Fraction and Extracellular Volume Fraction from Dual-Layer Spectral CT for Typing and Grading Renal Cell Carcinoma

*X. Zhang*[1], G. Zhang[1], H. Sun[1], Z. Jin[1], X. Lu[2], S-H. Yu[1], L. Xu[3], J. Zhang[1], X. Bai[1]; [1]Beijing/CN, [2]Shenyang/CN, [3]Hangzhou/CN
*(zhang_xiaoxiao23@163.com)*

**Purpose or Learning Objective:** To explore the value of arterial enhancement fraction (AEF) and extracellular volume fraction (ECV) obtained from dual-layer spectral CT in the typing and grading of renal cell carcinoma (RCC).
**Methods or Background:** In this retrospective study, patients with pathologically confirmed RCC who has undergone dual-layer spectral CT were included. RCC was classified into non-clear cell (non-ccRCC) and clear cell (ccRCC). The ccRCC cases were further categorized as high-grade or low-grade based on the WHO/ISUP grading system. AEF and ECV parameter maps were generated from both contrast-enhanced and iodine concentration (IC) images, producing quantitative parameters AEFHU, ECVHU, AEFIC, and ECVIC. Receiver operating characteristic curves were used to evaluate the ability of these parameters in RCC typing and grading.
**Results or Findings:** The study included 68 patients, comprising 13 with non-ccRCC and 55 with ccRCC. CcRCC showed higher values of AEFHU, ECVHU, AEFIC, and ECVIC compared with non-ccRCC. The multivariate model comprising AEFIC, and ECVIC demonstrated the highest diagnostic accuracy for ccRCC, with an area under curve (AUC) of 0.822, sensitivity of 83.6%, and specificity of 76.9%. Among the ccRCC cases, 34 were low-grade and 21 were high-grade. High-grade ccRCCs exhibited significantly higher ECVHU and ECVIC than low-grade tumors. The multivariate model with tumor diameter, and ECVIC achieved the highest diagnostic accuracy in identifying high-grade ccRCC, with an AUC of 0.909, sensitivity of 90.5%, and specificity of 76.5%.
**Conclusion:** AEF and ECV derived from dual-layer spectral CT can help distinguish ccRCC from non-ccRCC. Additionally, ECV can accurately identify high-grade ccRCC, offering valuable insights for RCC.
**Limitations:** Given the retrospective design and relatively small sample size of the present study, further studies should aim to include larger cohorts and consider prospective data collection to validate these findings.
**Funding for this study:** This study has received funding by the National High Level Hospital Clinical Research Funding [2022-PUMCH-A-033]; the Natural Science Foundation of Beijing Municipality [L232133]; the Chinese Academy of Medical Sciences Initiative for Innovative Medicine [2022-I2M-C&T-B-019]; National High Level Hospital Clinical Research Funding [2022-PUMCH-A-035]; National High Level Hospital Clinical Research Funding [2022-PUMCH-B-069].
**Ethics committee - additional information:** The study was conducted in accordance with the principles of the Declaration of Helsinki and approved by the institutional Research Ethics Committee
**Author Disclosures:**
Jiahui Zhang: Nothing to disclose
Gumuyang Zhang: Nothing to disclose
Xiaomei Lu: Nothing to disclose
Hao Sun: Nothing to disclose
Lili Xu: Nothing to disclose
Xin Bai: Nothing to disclose
Zhengyu Jin: Nothing to disclose
Xiaoxiao Zhang: Nothing to disclose
Sheng-Hui Yu: Nothing to disclose

### Prognostic role of Whole-body MRI (WB-MRI) in patients with metastatic prostate cancer receiving systemic anti-cancer therapy

*C. Sattin*[1], C. Pizzi[1], F. Arnone[1], P. Hoxha[1], D. Berloco[1], F. Zugni[1], P. Summers[1], A. R. R. Padhani[2], G. Petralia[1]; [1]Milan/IT, [2]Northwood/UK
*(caterina.sattin@unimi.it)*

**Purpose or Learning Objective:** To investigate the potential of the response assessment category (RAC) from MET-RADS-P guidelines as prognostic biomarker in metastatic castrate resistant prostate cancer (mCRPC) patients.
**Methods or Background:** We enrolled mCRPC patients who underwent whole-body MRI at baseline and at each time point (every 12 weeks disease until progression) after systemic anti-cancer therapy (SACT). We correlated the maximum RAC at time point 1 (TP1) with overall survival (OS). Patients were divided in two groups: those with a maximum RAC 1-2 (highly likely or likely to be responding, respectively) and those with a maximum RAC 3-4-5 (stable disease, likely or highly likely to be progressing) at TP1. Survival curves were depicted in Kaplan-Meier plots and compared via a log-rank test and hazard ratio (HR) using Cox regression model, with point comparisons of three-year survival and median survival duration, using R.
**Results or Findings:** Out of 31 mCRPC patients enrolled, a higher OS was observed in patients with a maximum RAC 1-2 (N=11) than in those with a maximum RAC 3-4-5 (N=21) at TP1 (log-rank test p=0.005): median 34 months (lower bound 95%CI = 27 months) vs median 12 months (95%CI 11-28 months). The HR for the RAC 3-4-5 patients was 1.34 (95%CI 0.83 – 1.85, p= 0.009). Three-year OS was 30.3% for RAC1-2 vs 5.3% for RAC 3-4-5, for a difference of 25.0% (95%CI -11.1% - 61.2%, p=0.175).
**Conclusion:** Our observations support the potential of RAC after TP1 as a prognostic biomarker in mCRPC undergoing SACT.
**Limitations:** Retrospective and monocentric study.
**Funding for this study:** No fundings
**Ethics committee - additional information:** Not applicable
**Author Disclosures:**
Fabio Zugni: Nothing to disclose
Francesca Arnone: Nothing to disclose
Giuseppe Petralia: Nothing to disclose
Caterina Pizzi: Nothing to disclose
Paolo Hoxha: Nothing to disclose
Paul Summers: Nothing to disclose
Professor Anwar R. R Padhani: Nothing to disclose
Caterina Sattin: Nothing to disclose
Donatello Berloco: Nothing to disclose

**Prognostic value of WB-MRI derived Bone Marrow Adipose Tissue (BMAT) in bone metastatic prostate cancer patients treated with androgen deprivation + enzalutamide +/- zoledronic acid**
*N. Di Meo*, C. Buizza, P. Rondi, A. Dalla Volta, A. Borghesi, M. Ravanelli, A. Berruti, D. Farina; Brescia, BS/IT
*(nunzia.dimeo@hotmail.it)*

**Purpose or Learning Objective:** To assess the prognostic significance of bone marrow adipose tissue (BMAT) in prostate cancer patients with hormone-sensitive bone metastases undergoing whole-body MRI (WB-MRI) and receiving enzalutamide treatment.
**Methods or Background:** Imaging was conducted on a 1.5T MRI scanner using a MET-RADS-P-compliant protocol. Manual single-slice segmentation of fat fraction (FF%) sequences was performed by one operator (R1) at the L3 vertebral level and across three contiguous slices at the femoral head. WB-MRI was performed at baseline and at 6 and 12 months following the initiation of therapy. Absolute BMAT values and temporal changes were recorded and correlated with survival outcomes.
**Results or Findings:** Of the 126 patients enrolled in this prospective phase 2 clinical trial, 100 were available for analysis. No correlation was found between BMAT measurements at the L3 vertebra and the femoral head, with the latter showing significantly higher values (90.7% vs. 63.9%, respectively). A significant positive correlation was identified between baseline L3 BMAT and both progression-free survival (PFS) and overall survival (OS), with hazard ratios (HR) of 0.37 and 0.33, respectively, after a median split. Additionally, early changes in L3 BMAT were inversely associated with PFS and OS, with HRs of 1.89 and 2.96, respectively. BMAT at the femoral head was not associated with survival outcomes.
**Conclusion:** L3 BMAT is a valuable prognostic and predictive biomarker that can be easily derived from WB-MRI. It may contribute to more personalized treatment strategies for patients with metastatic prostate cancer.
**Limitations:** No external validation.
**Funding for this study:** No Funding
**Ethics committee - additional information:** No
**Author Disclosures:**
Andrea Borghesi: Nothing to disclose
Chiara Buizza: Nothing to disclose
Davide Farina: Nothing to disclose
Paolo Rondi: Nothing to disclose
Marco Ravanelli: Nothing to disclose
Alberto Dalla Volta: Nothing to disclose
Nunzia Di Meo: Nothing to disclose
Alfredo Berruti: Nothing to disclose

**Conventional Parameters of Periprostatic Fat on 18F-PSMA-1007 PET/CT: A Novel Biomarker for Predicting High ISUP Grade and Short-Term Prognosis in Prostate Cancer**
*L. Chen*, Y. Yang, F. Yao; Wenzhou/CN
*(1004491518@qq.com)*

**Purpose or Learning Objective:** This study aimed to investigate the value of periprostatic fat area and 18F-PSMA-1007 uptake in predicting high ISUP grade and postoperative PSA persistence in prostate cancer patients using 18F-PSMA-1007 PET/CT.
**Methods or Background:** A retrospective analysis was conducted on clinical data and 18F-PSMA-1007 PET/CT data of 350 prostate cancer patients. 3D-Slicer and Lifex software were utilized for delineating the region of interest for periprostatic fat and measuring periprostatic fat area and 18F-PSMA-1007 uptake. The primary outcome of this study was the ISUP grade greater than 3 based on surgical pathological results of radical prostatectomy. The secondary outcome was postoperative PSA persistence, defined as routine follow-up tPSA > 0.1 ng/ml. Logistic regression analyses were performed to assess the association between characteristics and outcomes and construct predictive models. Receiver operating characteristic curves were utilized to determine optimal cutoff values and evaluate model performance.
**Results or Findings:** Larger periprostatic fat area emerged as an independent risk factor for higher ISUP grade ($p < 0.001$) and postoperative PSA persistence ($p = 0.009$) in prostate cancer patients. Higher 18F-PSMA-1007 uptake was also closely associated with higher ISUP grade ($p < 0.001$) and postoperative PSA persistence ($p < 0.001$). Models respectively established to predict higher ISUP grade and postoperative PSA persistence showed good predictive performance, with AUC values of 0.736 and 0.745.
**Conclusion:** Larger periprostatic fat area and higher 18F-PSMA-1007 uptake are independent risk factors for high ISUP grade and postoperative PSA persistence, which can be used to predict high ISUP grade and the persistence of PSA.
**Limitations:** This is a small sample study. The generalizability of the results requires further consideration.
**Funding for this study:** This study was supported by the Wenzhou Major Program of Science and Technology Innovation (Grant No. ZY2020012).
**Ethics committee - additional information:** This retrospective study has been reviewed and approved by the the first affiliated hospital of Wenzhou Medical University ethics committee.
**Author Disclosures:**
Yunjun Yang: Nothing to disclose
Fei Yao: Nothing to disclose
Lixuan Chen: Nothing to disclose

**Decrease in kidney volume predicts loss of renal function in prostate cancer patients receiving LuPSMA treatment**
*F. Jungmann*, L. Steinhelfer, M. R. Makowski, M. Eiber, R. Braren; Munich/DE
*(friederike.jungmann@tum.de)*

**Purpose or Learning Objective:** Lutetium-177 (177Lu) prostate-specific membrane antigen (PSMA) radioligand therapy (RLT) is a novel treatment option for metastatic, castration-resistant prostate cancer (mCRPC). Evidence is increasing that nephrotoxicity is a delayed side effect in a considerable fraction of patients. The purpose of this study was to identify prognostic markers for clinically significant deterioration of kidney function in patients undergoing 177Lu-PSMA RLT.
**Methods or Background:** Total kidney volume (TKV) at 3 and 6 months following 177Lu-PSMA RLT was extracted from routine clinical CT scans using deep learning. A cut-off at ≥30% eGFR decline was defined as clinically significant deterioration of kidney function, given its indication as a substantial risk of end-stage renal disease. Differences between patients developing an eGFR decline of ≥30% after 12 months and those who did not considering baseline renal parameters, their relative changes (Δ%), nephrotoxic risk factors, and the number of 177Lu-PSMA cycles were analyzed. Furthermore, distinct threshold values of significant features to differentiate between the two patient groups were identified based on ROC analysis using the Youden-Index.
**Results or Findings:** A ≥10% decrease in TKV at six months predicted a severe eGFR decline of ≥30% at 12 months with high diagnostic accuracy (ROC-AUC of 0.90), surpassing all other parameters. Baseline risk factors, the number of prior treatment regimens and 177Lu-PSMA cycles did not correlate with a higher eGFR decrease at 12 months.
**Conclusion:** Our retrospective analysis demonstrates the feasibility of fully automated kidney volume assessment from routine clinical imaging data to predicting significant deterioration of kidney function at 12-month after 177Lu-PSMA RLT in mCRPC. It is more accurate than early relative eGFR change and might contribute as a non-invasive biomarker when treatment decisions are pending including determining whether to continue/discontinue or adapt 177LuPSMA treatment.
**Limitations:** Retrospective, single-center
**Funding for this study:** None
**Ethics committee - additional information:** Ethical approval for this retrospective, HIPAA-compliant analysis was obtained from the local institutional review boards. The requirement for informed consent was waived because of its retrospective design.
**Author Disclosures:**
Rickmer Braren: Nothing to disclose
Marcus R. Makowski: Nothing to disclose
Matthias Eiber: Nothing to disclose
Friederike Jungmann: Nothing to disclose
Lisa Steinhelfer: Nothing to disclose

**Deep learning-accelerated MRI Imaging in Patients with Prostate Cancer and Benign Prostatic Hyperplasia**
*V. Koch*, T. Vogl, R. Strecker, C. Booz, S. Mahmoudi, L. D. Grünewald; Frankfurt/DE
*(vitali-koch@gmx.de)*

**Purpose or Learning Objective:** The purpose of this study was to investigate the impact of deep learning-accelerated T2-weighted MRI imaging of prostate cancer and benign prostatic hyperplasia (BPH).
**Methods or Background:** In this prospective study, adults who underwent 3-Tesla MRI of the prostate due to suspicion of prostate cancer or benign prostatic hyperplasia were included. Standard sequences were acquired according to a dedicated protocol compromising T1-, T2-, and diffusion-weighted imaging sequences. Additionally, T2-weighted imaging sequences using the deep learning algorithm (T2DL) were acquired in axial, coronal, and sagittal planes. Quantitative analysis encompassed time efficiency and objective imaging parameters, including signal-to-noise ratio (SNR) and contrast-to-noise ratio (CNR). Qualitative evaluation was independently performed by three blinded radiologists to assess diagnostic confidence, image quality, and lesion sharpness subjectively. Interreader agreement was calculated using Fleiss κ.

Saturday

**Results or Findings:** A total of 46 male patients (mean age, 70 ± 9 years) were included. The study cohort encompassed 22 patients (48%) with prostatic cancer and 24 patients (52%) with BPH. Subjective evaluation of T2DL-sequences among all three readers revealed slightly superior diagnostic confidence, image quality, and lesion sharpness when compared to standard T2w sequences. Especially regarding focal lesions, T2DL-sequences allowed for significantly sharper demarcation with higher diagnostic confidence in cancer diagnosis. Objective image analysis of T2DL revealed significantly higher SNR and CNR values when compared to conventional T2w-sequences. Acquisition times of T2w-sequences (axial, coronal, and sagittal plane) could be reduced by an average of 50 % using T2DL-sequences.
**Conclusion:** Our findings suggest that deep learning-accelerated T2w-sequences in MRI imaging of the prostate allow a relevant reduction in acquisition time while maintaining both subjective and objective image quality.
**Limitations:** Single-center study.
**Funding for this study:** No funding.
**Ethics committee - additional information:** Approval obtained.
**Author Disclosures:**
Christian Booz: Nothing to disclose
Thomas Vogl: Nothing to disclose
Vitali Koch: Nothing to disclose
Scherwin Mahmoudi: Nothing to disclose
Ralph Strecker: Nothing to disclose
Leon David Grünewald: Nothing to disclose

**Feasibility of arterial spin labelling in MRI evaluation of adnexal lesions: A comparative study with dynamic contrast enhancement imaging**
*C. Meinzer*[1], K. Zhang[1], R. Gnirs[1], O. Zivanovic[1], H-U. Kauczor[1], H-P. Schlemmer[1], F. Kurz[2], T. Mokry[1]; [1]Heidelberg/DE, [2]Geneva/CH
*(clara.meinzer@dkfz-heidelberg.de)*

**Purpose or Learning Objective:** To evaluate feasibility of arterial spin labeling (ASL) as a non-contrast MRI technique for assessing solid tissue of adnexal lesions and to compare its performance with dynamic contrast enhanced (DCE) MRI.
**Methods or Background:** We prospectively included 11 adnexal lesions with solid tissue in nine females. Regions of interest (ROIs) were annotated on DCE images, and then transferred anatomically to corresponding sites on ASL images. From these ROIs, we extracted semi-quantitative DCE parameters: area under the curve (AUC), relative area under the curve (relAUC), peak enhancement, time to peak, mean residence time, area under the first moment curve, and wash-in-rate (WiR). From ASL perfusion maps, we obtained mean adnexal blood flow (ABF). Correlation between ABF and DCE parameters was assessed using Pearson's correlation coefficient. For those parameters showing significant correlation, Bland-Altman plots were generated to evaluate agreement.
**Results or Findings:** Pearson's correlation revealed significant correlations between ABF and two DCE parameters: relAUC (r=-0.75, p=0.008), WiR (r=0.65, p=0.031). Other analysed DCE parameters showed no statistically significant correlations with ABF (p>0.05). Bland-Altman analysis was performed for the significantly correlating parameters. For relAUC, mean difference was 135.85 (SD=18.04). No data points fell outside the limits of agreement, indicating good agreement between ASL and DCE. Similarly, WiR showed a mean difference of -17.02 (SD=11.24), and no points outside the limits of agreement.
**Conclusion:** ABF demonstrated significant correlations with relAUC and WiR, indicating that ASL can provide comparable perfusion information to DCE for these metrics. The Bland-Altman analysis further suggests reasonable agreement between ASL-derived perfusion and DCE parameters for relAUC and WiR.
**Limitations:** The small sample size limits the generalisability of the findings. ASL MRI is susceptible to lower signal-to-noise ratios and variability in perfusion measurements, which can impact accuracy and reproducibility.
**Funding for this study:** No funding was received for this study.
**Ethics committee - additional information:** This study received institutional review board approval and written informed consent was obtained from all participants (S-337/2016).
**Author Disclosures:**
Oliver Zivanovic: Nothing to disclose
Clara Meinzer: Nothing to disclose
Ke Zhang: Nothing to disclose
Theresa Mokry: Nothing to disclose
Regula Gnirs: Nothing to disclose
Hans-Ulrich Kauczor: Nothing to disclose
Felix Kurz: Nothing to disclose
Heinz-Peter Schlemmer: Nothing to disclose

**16:00-17:30 Research Stage 3**

### Research Presentation Session: Breast

### RPS 2102

### How to optimise and use breast MRI

**Moderator**
N. Sharma; Leeds/UK
*(Nisha.sharma2@nhs.net)*

**Diagnostic accuracy of abbreviated magnetic resonance imaging for breast cancer screening: a multi-reader study**
*S. V. Grinsven*[1], R. Mann[2], K. M. Duvivier[3], M. De Jong[4], P. K. De Koekkoek-Doll[3], C. Loo[3], J. Veltman[5], W. B. Veldhuis[1], For The Dense Trial Study Group[1]; [1]Utrecht/NL, [2]Nijmegen/NL, [3]Amsterdam/NL, [4]Den Bosch/NL, [5]Almelo/NL
*(s.e.l.vangrinsven-2@umcutrecht.nl)*

**Purpose or Learning Objective:** Costs and time of a full multi-parametric MRI protocol may be reduced by using an abbreviated MRI (AB-MRI) protocol. The DENSE trial's multiparametric protocol provided the unique opportunity to study the accuracy of various AB-MRI protocols, to identify the minimal protocol necessary to maintain high diagnostic accuracy.
**Methods or Background:** Seven radiologists performed incremental reads of a subset of 518 MRI examinations from the DENSE trial (women with extremely dense breasts and negative mammography). Different sequences were added in four incremental steps, starting with: 1) both high resolution (hi-res) and ultra-fast T1-weighted images (T1WI), up to 120 seconds after contrast-injection only, 2) complemented by diffusion-weighted images (DWI), 3) T2-weighted images (T2WI), and 4) finally adding all remaining full protocol sequences: non-fatsat-T1-weighted pre-contrast images, all remaining dynamic phases, and curve-kinetics. Each radiologist assessed the same MRI examinations and provided BI-RADS scores for all four steps. We calculated the pooled sensitivity and specificity per incremental step by using a generalized estimating equation model, and the pooled reading time per incremental step by using a linear mixed model.
**Results or Findings:** The sensitivity and specificity of the most abbreviated MRI protocol (step 1) were not significantly different from that of the full multiparametric MRI protocol (step 4) (p=0.68, p=0.39). The pooled reading time of step 1 was almost 50% shorter than that of the full multiparametric MRI protocol (p<0.01), and the MR acquisition time was 70-80% shorter, depending on the hospital and scanner vendor.
**Conclusion:** In a screening setting, a full multiparametric MRI protocol, including pre-contrast DWI and T2WI, and delayed post-contrast T1WI, did not provide significant additional diagnostic information for making a recall/no-recall decision compared to an ultrafast bi-dynamic T1WI-only protocol.
**Limitations:** A prospective screening study should confirm these results.
**Funding for this study:** The DENSE trial is financially supported by the University Medical Center Utrecht (UMC Utrecht, Project number: UMCU DENSE), the Netherlands Organization for Health Research and Development (ZonMw, Project numbers: ZONMW-200320002-UMCU and ZonMW Preventie 50-53125-98-014), the Dutch Cancer Society (KWF Kankerbestrijding, Project numbers: DCS-UU-2009-4348, UU-2014-6859 and UU-2014-7151), the Dutch Pink Ribbon / a Sister's hope (Project number: Pink Ribbon-10074), Bayer AG Pharmaceuticals, Radiology (Project number: BSP-DENSE), and Stichting Kankerpreventie Midden-West. For research purposes, Volpara Health Technologies (Wellington, New Zealand) has provided Volpara Imaging Software 1.5 for installation on servers in the screening units of the Dutch screening program.
**Ethics committee - additional information:** On November 11, 2011, the trial was approved by the Dutch Minister of Health, Welfare, and Sport, under advisement from the Health Council of the Netherlands.
**Author Disclosures:**
Mathijn De Jong: Nothing to disclose
Petra Katharina De Koekkoek-Doll: Nothing to disclose
Wouter B. Veldhuis: Nothing to disclose
Katya M. Duvivier: Nothing to disclose
Jeroen Veltman: Nothing to disclose
For The Dense Trial Study Group: Nothing to disclose
Sophie Van Grinsven: Nothing to disclose
Claudette Loo: Nothing to disclose
Ritse Mann: Nothing to disclose

**Breast MRI protocol strategies to reduce energy consumption and carbon emissions: phantom and patient tests**

*J. T. Lee*[1], B. K. Seo[1], M. S. Bae[1], H. Choi[2], K. R. Cho[2], O. Woo[2], S. E. Song[2], S-Y. Kim[2], S. Cheon[1]; [1]Ansan/KR, [2]Seoul/KR
*(jeoungtaek@gmail.com)*

**Purpose or Learning Objective:** Environmental sustainability in healthcare is crucial, and MRI is a major energy-intensive device in radiology. We aimed to identify optimal energy-saving breast MRI protocols by comparing energy consumption and carbon emissions of abbreviated MRI, ultrafast dynamic contrast-enhanced MRI (Ultrafast-DCE), and artificial intelligence (AI)-assisted protocols against multiparametric MRI, conventional dynamic contrast-enhanced MRI (Conventional-DCE), and non-AI-assisted protocols using patient and phantom tests.
**Methods or Background:** A 3-T MRI scanner equipped with a dedicated breast coil and a power meter providing a 1-Hz sampling rate was used. We compared scan time (seconds), total energy (kW), energy consumption (kWh), and carbon emissions per scan (kgCO2e) between abbreviated (n=74) and multiparametric (n=81) protocols, Ultrafast-DCE (n=81) and Conventional-DCE (n=81), and AI-assisted (n=76) and non-AI-assisted (n=76) protocols in 307 patients. Additionally, the signal-to-noise ratio was compared between AI-assisted and non-AI-assisted protocols using a breast MRI phantom. The abbreviated protocol included T2-weighted imaging (T2), diffusion-weighted imaging (DWI) (b values: 0 and 800 s/mm²), four-phase DCE T1-weighted imaging (T1), and axillary T1. The multiparametric protocol consisted of T2, DWI (b values: 0, 800, and 1400 s/mm²), Ultrafast-DCE, five-phase DCE T1, and axillary T1.
**Results or Findings:** Abbreviated MRI reduced scan time by 40%, total energy by 36%, and energy/carbon emissions by 62% compared to multiparametric MRI. Ultrafast-DCE reduced scan time by 81%, total energy by 83%, and energy/carbon emissions by 97% compared to Conventional-DCE. AI-assisted MRI reduced scan time by 29%, total energy by 30%, and energy/carbon emissions by 52% compared to non-AI-assisted MRI, while increasing signal-to-noise ratio by 16% (all $p < 0.001$).
**Conclusion:** Abbreviated, ultrafast, and AI-assisted MRI protocols significantly reduce energy consumption and carbon emissions, supporting eco-friendly MRI practices.
**Limitations:** Not applicable.
**Funding for this study:** National Research Foundation of Korea funded by the Korea government (RS-2024-00347290).
**Ethics committee - additional information:** No
**Author Disclosures:**
Min Sun Bae: Nothing to disclose
Okhee Woo: Nothing to disclose
Soo-Yeon Kim: Nothing to disclose
Bo Kyoung Seo: Nothing to disclose
Sung Eun Song: Nothing to disclose
Jeong Taek Lee: Nothing to disclose
Sewon Cheon: Nothing to disclose
Kyu Ran Cho: Nothing to disclose
Hangseok Choi: Nothing to disclose

**Use of Diffusion-Weighted MRI in Screening High-Risk Women Under 40 for Breast Cancer**

*C. C. Arıkan*, M. A. Arıkan, M. A. Nazli; Istanbul/TR
*(ceydaceren95@gmail.com)*

**Purpose or Learning Objective:** Diffusion-weighted imaging(DWI) presents a rapid, cost-effective, and non-contrast alternative to contrast-enhanced MRI(CE-MRI).This study aims to evaluate the diagnostic performance of DWI compared to CE-MRI in breast cancer screening for high-risk women under 40 while assessing inter-rater agreement and correlating findings with biopsy outcomes and ultrasound BI-RADS categories.
**Methods or Background:** We retrospectively analyzed the DWI and CE-MR images of 112 women under the age of 40 who had breast MRI screening due to high risk. Two radiologists independently reviewed the images without knowledge of ultrasound or pathological results. Patients were classified as having "diffusion restriction present or absent" and "pathological contrast enhancement present or absent." Inter-rater agreement was assessed using Cohen’s kappa coefficient.MRI findings were compared to ultrasound-based BI-RADS reports and biopsy results to determine sensitivity and specificity.
**Results or Findings:** Lesion detection inter-rater agreement was high for DWI(kappa=0.83), and moderate for CE-MRI(kappa=0.57). Of the 112 patients, 43 underwent biopsy, with 37 benign and 6 malignant diagnoses. DWI detected 5 out of 6 malignant lesions (sensitivity 83.3%).DWI exhibited 10 false positives (specificity 73%).CE-MRI detected all malignant lesions (sensitivity 100%), but showed 30 false positives (specificity 18.9%). Among the 79 patients classified by BI-RADS with no biopsy, 57 were BI-RADS1-2, with 5(%8.7) false positives on DWI and 14(%24,5) on CE-MRI. Of the remaining 12 BI-RADS3 cases were recommended for follow-up, DWI was positive in 2(%16.6) and CE-MRI in 7(%58.3).
**Conclusion:** DWI shows high sensitivity and specificity, particularly in breast cancer screening for women at high risk below 40 years.The high inter-rater agreement shows its reliability, and the reduced recall rate suggests that DWI has the potential to reduce unnecessary treatments compared to CE-MRI.
**Limitations:** The study is limited by loss to follow-up patients and those with incomplete diagnostic tests
**Funding for this study:** No funding was provided for this study.
**Ethics committee - additional information:** Başakşehir Çam and Sakura City Hospital Ethics Committee
**Author Disclosures:**
Mehmet Ali Nazli: Nothing to disclose
Ceyda Ceren Arıkan: Nothing to disclose
Mehmet Ali Arıkan: Nothing to disclose

**Patient comfort in supine breast MRI using a wearable coil - preliminary questionnaire results**

*L. Nohava*, R. Czerny, M. Tik, E. Laistler, R. Frass-Kriegl; Vienna/AT

**Purpose or Learning Objective:** Supine positioning during breast MRI using a wearable coil at 3 T has the potential to improve patient comfort and to extend the inclusion criteria for breast MR examinations (obesity, pregnancy). While the technical performance of the wearable coil for supine breast MRI is being assessed in ongoing studies, the aim of this study was the evaluation of patient perception with the goal of ensuring comfortable patient-oriented breast MR examinations.
**Methods or Background:** A questionnaire evaluating the impact of radiofrequency coils on patient comfort in MRI as an add-on to clinical studies comparing the performance of flexible coils with standard rigid coils was developed. In an IRB-approved breast MRI study, patients underwent one exam in supine using a wearable flexible coil and one prone reference exam. After each exam, patients filled in the questionnaire with 18 items using a 7-point Likert scale. So far, 10 questionnaire sets were collected. The study population had a range of different bra sizes (70B-95D), ages (20-64 years), and BMIs (19-30 kg/m2).
**Results or Findings:** Significant improvement in patient comfort during supine breast MRI was found for the following items: “I felt comfortable before the exam.” ($p<0.009$); “I found it cumbersome or physically demanding to take the lying position.” ($p<0.026$); ”I felt comfortable during the exam.” ($p<0.043$); “I found the lying position comfortable.” ($p<0.047$). 9 patients commented on pain or discomfort in prone whereas only 1 patient commented on discomfort due to peripheral nerve stimulation in supine breast MRI.
**Conclusion:** Patients perceived supine BraCoil MRI as significantly more comfortable than prone MRI, in anticipation of and during the exam.
**Limitations:** The limitations of the study are the small sample size, and mono-center character.
**Funding for this study:** Funding was provided by by the Austrian Science Fund (FWF)/Agence Nationale de Recherche (ANR) grant FWF I-3618/ANR-17-CE19-0022 “BraCoil” and FWF grant P37189 “OPTIMAL”, the Horizon Europe Grants No. 101078393 “MRITwins” and No. 101071008 “CITRUS”, and the Austrian Society for Senology (ÖGS) support grant.
**Ethics committee - additional information:** The study was approved by the Ethics Committee of the Medical University of Vienna (EK No. 2137/2021).
**Author Disclosures:**
Lena Nohava: Nothing to disclose
Roberta Frass-Kriegl: Nothing to disclose
Elmar Laistler: Shareholder: ALSIX GmbH
Martin Tik: Nothing to disclose
Raphaela Czerny: Nothing to disclose

**A breast MRI image quality score (BreastMRI-QUAL): preliminary results**

S. Marziali, *L. Corradini*, M. Zanardo, C. Depretto, G. Della Pepa, G. Irmici, G. P. Scaperrotta, F. Sardanelli; Milan/IT
*(corradinilisa@gmail.com)*

**Purpose or Learning Objective:** Breast MRI is an established technique for diagnosing breast cancer using a multiparametric protocol, including sequences before/after contrast administration. The diagnostic performance depends on image quality, limited by misregistration artefacts due to patient movement. We propose a standardized image quality score (BreastMRI-QUAL).
**Methods or Background:** Two independent readers with 3 years of experience assigned a 4-level score to each sequence of 50 consecutive 1.5-T examinations at a tertiary cancer centre, as follows: 0 = not diagnostic for any cause; 1 = relevant artefacts/malpositioning with conserved diagnostic value for the specific case; 2 = slight artefacts/malpositioning with conserved diagnostic value; 3 = excellent image quality with full diagnostic value. The score per sequence was summed as follows: (T2-weighted*1) + (DWI-b=0*0.5) + (ADCmaps*0.5) + (T1-weighted-precontrast*1) + (T1-weighted-postcontrast*2) + (T1-weighted-subtracted*3). To obtain a global score (GS)

from 0 to 10, the sum was divided by 2.4, with scores below 6 considered as insufficient.
**Results or Findings:** The average GS between the two readers was 8.0 ± 1.0 (mean ± standard deviation), with 42 cases (84%%) receiving a score ≥ 7. Only 1 case (2%) was scored <6 by both readers. The difference between the GSs assigned by the readers was ≤ 1 for 32 cases (64%), >1 but ≤ 2 for 17 cases (34%), and > 2 for 1 case (2%). The Bland-Altman analysis showed a mean difference (bias) of 0.28, with the limits of agreement ranging from -1.82 to 2.38, indicating the level of agreement between readers. The average assessment time/examination was 3 min.
**Conclusion:** BreastMRI-QUAL is a reproducible quality score system. Breast MRI image quality at a tertiary cancer center was good-to-excellent in over 80% of cases. Multicenter-multivendor validation studies are needed.
**Limitations:** Monocentric study, limited sample size.
**Funding for this study:** No funding.
**Ethics committee - additional information:** Use of anonymized datasets outside clinical workflow.
**Author Disclosures:**
Gianmarco Della Pepa: Nothing to disclose
Sara Marziali: Nothing to disclose
Francesco Sardanelli: Research/Grant Support: Bayer AG, Bracco imaging, GE healthcare Speaker: Bayer AG, Siemens Healthineers, Esaote Advisory Board: Bayer AG, Bracco imaging, GE healthcare
Gianfranco Paride Scaperrotta: Nothing to disclose
Moreno Zanardo: Nothing to disclose
Catherine Depretto: Nothing to disclose
Lisa Corradini: Nothing to disclose
Giovanni Irmici: Nothing to disclose

**Detection of residual fibroglandular tissue on breast MRI in women treated with mastectomy and DIEP flap breast reconstruction**
*N. Smeins*, J. Rooij, Van, E. Heuts, J. B. Houwers, S. Tuinder, T. Van Nijnatten; Maastricht/NL
*(nieke.smeins@mumc.nl)*

**Purpose or Learning Objective:** After breast amputation, and especially skin sparing mastectomy, there can be residual fibroglandular tissue (RFGT). RFGT can influence the risk of breast cancer recurrence. However, women are not screened for RFGT after mastectomy. This study examines the frequency in which RFGT can be detected on breast MRI after mastectomy and DIEP flap reconstruction and the influence of RFGT on breast cancer recurrence risk.
**Methods or Background:** This retrospective, single-centre study included female patients who underwent mastectomy and DIEP flap reconstruction. Post-reconstruction breast MRI exams from 2007-2022 were reassessed by a breast radiologist to detect potential presence of RFGT. The presence of RFGT was rated according to a confidence scale (1-5), with a score of 1 indicating 'definitely no breast tissue' and 5 indicating 'definitely breast tissue'. Locations suspected of RFGT rated with a score of 4 or more were considered RFGT. RFGT prevalence was correlated with disease recurrence.
**Results or Findings:** A total of 73 patients (85 breasts) were included. RFGT was found in 15 (20.5%) patients and 16 (18.8%) breasts. Ten (13.7%) local recurrences had occurred after a mean follow-up period of 164.3 months (range: 27.0-381.0 months). Presence of RFGT resulted in a relative risk of 2.58 (95% CI 0.83-7.98) for recurring disease. Most breast MRI exams were assessed with a score of 3 on the confidence scale (39.2%).
**Conclusion:** RFGT is frequently detected on breast MRI after mastectomy and DIEP flap breast reconstruction and might have an association with disease recurrence. Future studies should focus on the clinical consequences of visualization of RFGT on breast MRI and whether there is a role for breast MRI in post-mastectomy patients.
**Limitations:** No limitations were identified.
**Funding for this study:** N. Smeins received a salary from Kankeronderzoekfonds Limburg.
**Ethics committee - additional information:** The study was approved by METC azM/UM (reference number METC 2022-3122).
**Author Disclosures:**
Joep Rooij, Van: Nothing to disclose
Thiemo Van Nijnatten: Nothing to disclose
Nieke Smeins: Nothing to disclose
Estherm. Heuts: Nothing to disclose
Stefania Tuinder: Nothing to disclose
Janneke B. Houwers: Nothing to disclose

**Breast cancer on post bilateral mastectomy surveillance MRI**
*T. Arazi Kleinman*[1], J. Lvovski[1], D. Walchok[1], G. Michal[2], T. Sella[3]; [1]Beer Yakov/IL, [2]Tel Aviv/IL, [3]Jerusalem/IL
*(t_arazikleinman@yahoo.com)*

**Purpose or Learning Objective:** Current guidelines regarding post bilateral mastectomy (BMx) follow-up indicate no need for imaging. Regardless many patients are referred for breast MRI, though this practice is not evidence based. The aim of this study to evaluate the role of MRI in detection of cancer in post BMx women.
**Methods or Background:** Retrospective analysis of surveillance breast MRI in women s/p BMx between the years 2017-2020, at a single institution. Data collected included demographic information, personal and family history of breast cancer, indication for mastectomy (prophylactic vs. therapeutic) and reconstruction type. Suspicious MRI findings underwent biopsy and were correlated with pathology. Malignancy or benignity were determined by either pathology or stability on imaging for at least 12 months. Descriptive statistics applied with p<0.05 considered significant.
**Results or Findings:** 229 asymptomatic women s/p BMx aged 29-76±8.7 years underwent 709 surveillance studies for a total of 1418 breasts examined. Reconstructions included 1324 (93.3%) silicone, 47 (3.3%) autologous flaps and 47 (3.3%) with no reconstruction. 158 (69%) women underwent risk-reducing prophylactic Mx (rr-Mx) on one side and therapeutic Mx (t-Mx) for cancer on the other, 45 (20%) underwent bilateral rr-Mx and 26 (11%) underwent bilateral t-Mx for bilateral breast cancer. Overall, 782/1418 breasts underwent rr-Mx and 184/1418 breasts t-Mx. Cancer was detected in six breasts, five post t-Mx and one post rr-Mx. Overall cancer detection rate (CDR) was 0.4 (6/1418), higher in post t-Mx (CDR= 0.78, 5/636) than post rr-Mx (CDR= 0.12, 1/782), p<0.05. No cancers were detected in women post rr-BMx.
**Conclusion:** Cancer risk in women undergoing bilateral rr-Mx is negligible and likely does not warrant MRI surveillance. In contrast, CDR in post t-Mx women was 0.78 on the side of prior cancer and surveillance MRI may be considered.
**Limitations:** Single-institution, retrospective
**Funding for this study:** No Funding for this study
**Ethics committee - additional information:** Retrospective study
**Author Disclosures:**
Guindy Michal: Nothing to disclose
Tal Arazi Kleinman: Nothing to disclose
Joana Lvovski: Nothing to disclose
Daria Walchok: Nothing to disclose
Tamar Sella: Nothing to disclose

**Percentage functional tumor volume on pre-treatment MRI within HER2+ breast cancer predicts pathologic complete response to combination neoadjuvant immunotherapy and chemotherapy**
*R. J. Weinfurtner*, S. Falcon, D. Ataya, M. Abdalah, O. Stringfield, N. Raghunand, B. Czerniecki, H. Soliman, H. Han; Tampa, FL/US
*(Tigerphage@yahoo.com)*

**Purpose or Learning Objective:** To determine if functional tumor volume (FTV) analysis of human epidermal growth factor 2 positive (HER2+) breast cancer on pre-treatment MRI can help predict pathologic complete response (pCR) in patients treated with dendritic cell vaccine (DC1) neoadjuvant immunotherapy (NAI) followed by neoadjuvant chemotherapy (NAC).
**Methods or Background:** Patients with HER2+ breast cancer in this pilot trial underwent pre-treatment MRI, followed by ultrasound-guided intratumoral and intranodal DC1 injections, and then NAC prior to post-treatment MRI and surgery. FTV was calculated on pre-treatment post-contrast T1-weighted MRI images using a percent enhancement threshold of 70% and signal enhancement ratio set to 0. The %FTV was calculated as %FTV = FTV / segmented tumor volume. These were correlated with pathologic response at surgery using unpaired t-tests where p<0.05 was considered significant. FTV analysis was also compared to post-NAI/NAC pre-surgical MRI reports for diagnostic test accuracy comparison.
**Results or Findings:** Nineteen patients aged 29-74 (average 54) were included in the study, and 11 (57%) achieved pCR. MRI complete response (mCR) was seen in 11/19 (57%). However, accuracy for mCR predicting pCR was only 38%. For %FTV, median was 71%, and patients achieving pCR had higher %FTV (78% vs 57%, respectively, p=0.007). As a diagnostic test, %FTV above median accurately predicted pCR in 79% (95% confidence interval of 54-94%) with sensitivity 75%, specificity 86%, PPV 90%, and NPV 67%.
**Conclusion:** In this pilot study of combination NAI/NAC treatment for HER2+ breast cancer, tumors with above median %FTV on pre-treatment MRI demonstrated more favorable response to treatment, achieving pCR in 78%. Given that post-treatment MRI evaluation demonstrated low accuracy in predicting pCR, pre-treatment FTV analysis may prove a more accurate predictor in future studies.

**Limitations:** This study was a pilot study with limited sample size.
**Funding for this study:** Internal institution grant
**Ethics committee - additional information:** Institutional Review Board (IRB)
**Author Disclosures:**
Robert Jared Weinfurtner: Nothing to disclose
Mahmoud Abdalah: Nothing to disclose
Hatem Soliman: Nothing to disclose
Shannon Falcon: Nothing to disclose
Dana Ataya: Nothing to disclose
Brian Czerniecki: Nothing to disclose
Hyo Han: Nothing to disclose
Olya Stringfield: Nothing to disclose
Natarajan Raghunand: Nothing to disclose

**Breast MRI: assessment of the Kaiser score in differentiation of non-mass lesions**
*M. Vukojevic*, M. M. Nadrljanski, I. B. Krušac, D. Dimitrijevic, L. J. Raspopović, A. Djajic, M. Mihajlović; Belgrade/RS
*(milosvukojevic999@gmail.com)*

**Purpose or Learning Objective:** To examine the performance of the Kaiser score in the diagnosis of nonmass breast lesions on MRI.
**Methods or Background:** There were 39 female patients with pathologically confirmed nonmass lesions on breast MRI retrospectively analyzed. For each patient, the Kaiser score was determined and the BI-RADS category assigned. All patients were examined with full diagnostic protocol (T2W-STIR, T2W-TSE, T1W-TSE, DWI-ADC, 3D-FLASH) on 1.5T and 3T. Specificity and sensitivity of the Kaiser score were computed and the correlation between the Kaiser score and the BI-RADS classification was determined.
**Results or Findings:** In the group of patients with nonmass lesions (N=39), there were 10 patients (n1) with benign lesions (25.64%) and 29 patients with malignant lesions (n2). The mean Kaiser score value in n1=3 and in n2=7. There was significant correlation between the Kaiser score and BI-RADS category: n1=0.92; n2=0.66; N=0.79. Sensitivity of the Kaiser score was 89.7% and specificity equaled 70.0%. ROC curve value reached 0.89.
**Conclusion:** Kaiser score represents a reproducible, sensitive and specific diagnostic tool for assessment of nonmass lesions on breast MRI and may contribute to the adequate BI-RADS categorization and appropriate further steps in diagnostic algorithm of the patients with nonmass lesions.
**Limitations:** A single center retrospective analysis with the limited number of patients.
**Funding for this study:** None
**Ethics committee - additional information:** No decision was required for the retrospective analysis without the patient intervention.
**Author Disclosures:**
Iva B. Krušac: Nothing to disclose
Dejan Dimitrijevic: Nothing to disclose
Luka Josif Raspopović: Nothing to disclose
Marko Mihajlović: Nothing to disclose
Andjela Djajic: Nothing to disclose
Milos Vukojevic: Nothing to disclose
Mirjan M. Nadrljanski: Nothing to disclose

**Distinguish HER2-low expression level in breast cancer: insights from qualitative and quantitative MRI analysis**
*Y. Shen*, C. You, Y. Gu; Shanghai/CN

**Purpose or Learning Objective:** To investigate whether qualitative and quantitative MRI features can reflect HER2-low expression breast cancer.
**Methods or Background:** The benefit of novel antibody-drug conjugates in HER2-low expression breast cancer suggests that the conventional binary classification HER2 status is insufficient to meet the needs of clinical diagnosis and treatment.The pre-treatment breast MRI images of 232 patients with pathologically confirmed breast cancer were retrospectively analyzed. Clinicopathologic features and MRI features were recorded. The qualitative MRI features included BI-RADS descriptors in DCE-MRI, and intratumoral T2 hyperintensity and peritumoral edema in T2WI. The quantitative features were generated by multi-b-value DKI, including mean, median, 5th, 95th percentile, skewness, kurtosis and entropy of ADC, Dapp and Kapp histogram from the mono-b and multi-b value models.
**Results or Findings:** HER2 status was categorized into HER2-zero (n=60), HER2-low (n=91) and HER2-over expression (n=81). For MRI features, the proportion of intratumoral T2 hyperintensity was higher in HER2-low than in other groups (p=0.009, p=0.008). For the lesion type, the mass lesions were more common in HER2-zero group than in HER2-low group (p=0.038). For mass lesions, mass shape (p<0.001) and margin(p<0.001) were significantly different between HER2-low and other groups, and mass shape is the independent predictive factor (HER2-low vs. HER2-zero: p=0.010, HER2-low vs. HER2-over: p=0.012). The area under the ROC curve (AUC) of qualitative features to distinguish HER2-low and -zero was 0.763 (95% CI: 0.667-0.859). Quantitative features differed between HER2-low and -overexpression groups, especially in NME-related lesions. All combined variables (Combinedall) had the best performance in predicting HER2-low, with an AUC of 0.802 (95% CI: 0.701 - 0.903).
**Conclusion:** Qualitative and quantitative MRI features are valuable for noninvasively distinguishing HER2-low expression breast cancer, and have their advantages in mass and NME lesions, respectively.
**Limitations:** Single-center retrospective study with limited samples.
**Funding for this study:** Not applicable.
**Ethics committee - additional information:** Fudan University, Shanghai Cancer Center
**Author Disclosures:**
Yajia Gu: Nothing to disclose
Chao You: Nothing to disclose
Yiyuan Shen: Nothing to disclose

**Pre- and post-contrast assessment of apparent diffusion coefficient in early tumor response assessment in patients on neoadjuvant chemotherapy**
*M. M. Nadrljanski*, I. B. Krušac, D. Dimitrijevic, L. J. Raspopović, A. Djajic, M. Mihajlović; Belgrade/RS
*(dr.m.nadrljanski@gmail.com)*

**Purpose or Learning Objective:** To assess the difference in pre- and post-contrast assessment of apparent diffusion coefficient (ADC) in early tumor response to neoadjuvant chemotherapy (NACT) after the 2nd cycle.
**Methods or Background:** There were 43 patients (N=43) included in retrospective analysis of ADC (b50, b850) in assessment of early tumor response in responders (R, n1=19) and non-responders (NR, n2=24), defined pathologically . In all patients, diffusion-weighted imaging (EPI sequence) was performed before and after application of contrast medium (gadobutrol, 1 mmol/L; 0.1 mL/kg). All patients were examined on either 1.5T or 3T unit in same institution with full diagnostic protocol (T2W-STIR, T2W-TSE, T1W-TSE, DWI b50, b850, 3D-FLASH, DWI b50, b850).
**Results or Findings:** In R, mean pre-contrast ADC: 1.17+/-0.07 mm2/s x 10-3, significantly differs from pre-contrast ADC in NR: 0.98+/-0.09 mm2/s x 10-3; p<0.0001. In R, mean post-contrast ADC value: 1.09+/-0.08 mm2/s x 10-3, significantly differs from post-contrast ADC in NR: 0.91+/-0.09 mm2/s x 10-3; p<0.0001. Significant difference was obtained for R between mean value of ADC on pre- and post-contrast DWI: 1.17+/-0.07 mm2/s x 10-3 vs. 1.09+/-0.08 mm2/s x 10-3; p<0.001. Significant difference was obtained for NR between mean value of ADC on pre- and post-contrast DWI: 0.98+/-0.97 mm2/s x 10-3 vs. 0.91+/-0.09 mm2/s x 10-3; p<0.001.
**Conclusion:** DWI before and after the application of contrast media did not clinically influence the differentiation between R and NR, although ADC was generally lower after the application of contrast medium. Although significantly different, ADC values for R before and after the application of the contrast medium did not show clinical relevance and did not interfere with the interpretation of the findings. The same applied for the ADC values for NR.
**Limitations:** Small number of patients in a single center retrospective study.
**Funding for this study:** None,
**Ethics committee - additional information:** Institutional Review Board waived the need for decision for the collection and analysis of medical record information, with no participant interaction.
**Author Disclosures:**
Iva B. Krušac: Nothing to disclose
Dejan Dimitrijevic: Nothing to disclose
Luka Josif Raspopović: Nothing to disclose
Marko Mihajlović: Nothing to disclose
Andjela Djajic: Nothing to disclose
Mirjan M. Nadrljanski: Nothing to disclose

**16:00-17:30** **Research Stage 4**

### Research Presentation Session: Imaging Informatics and Artificial Intelligence

### RPS 2105

### Artificial intelligence in chest imaging

**Moderator**
C. M. Schaefer-Prokop; Amersfoort/NL
*(cornelia.schaeferprokop@gmail.com)*

**Standardized platform to evaluate, compare, and analyze AI-based software for detection and classification of lung nodules for the purpose of CT lung cancer screening implementation**

*X. Ouyang*[1], K. Togka[2], D. Han[2], H. L. Lancaster[2], I. Schuldink[2], A. N. Walstra[2], C. Van Der Aalst[1], H. J. De Koning[1], M. Oudkerk[2]; [1]Rotterdam/NL, [2]Groningen/NL
*(x.ouyang@erasmusmc.nl)*

**Purpose or Learning Objective:** Low-dose CT detects lung nodules and consequently lung cancer (LC) at an early stage, proven to reduce LC mortality. To aid radiologists, commercially available AI-based software have been developed to analyze lung nodules. Self-reported performance metrics appear promising, however, there remains no independent, standardized platform for external validation. We aimed to develop a standardized, independent, trustworthy platform to assess and compare the performance of commercially available AI-lung nodule analysis software.
**Methods or Background:** We developed a platform using a sequestered dataset of 560 scans from the EU-funded 4-IN-THE-LUNG-RUN (4ITLR) lung cancer screening implementation trial. The platform is based on systematic Structured Query Language (SQL) database architecture. Output of AI software in different data formats was reformatted and stored to standardized SQL records, eliminating manual errors, and allowing AI software results to be compared using uniformed data analysis algorithms to the final consensus result of an expert radiologist panel. Performance is evaluated on two levels: nodule level and participant level. Nodule level compares the detection/classification of the reference nodule per participant and participant level was based on the largest-detected solid nodule.
**Results or Findings:** Performance of AI software at nodule level was reported using frequencies of agreement and discrepancies with the 4ITLR consensus result on reference nodule. At the participant level, Cohen's kappa coefficient is used to measure the agreement level with reference.
**Conclusion:** The standardized platform developed provides an independent assessment of AI software performance. Clinical users benefit from reliable comparison of outcomes for lung nodule analysis and transparency of commercial AI in radiology.
**Limitations:** No limitations have been identified yet.
**Funding for this study:** The 4-IN-THE-LUNG-RUN trial is funded by the European Union (grant number:848294)
**Ethics committee - additional information:** Not applicable
**Author Disclosures:**
Matthijs Oudkerk: Nothing to disclose
Anna N.H. Walstra: Nothing to disclose
Harriet Louise Lancaster: Nothing to disclose
Harry J. De Koning: Nothing to disclose
Katerina Togka: Nothing to disclose
Daiwei Han: Nothing to disclose
Xiaotong Ouyang: Nothing to disclose
Ilona Schuldink: Nothing to disclose
Carlijn Van Der Aalst: Nothing to disclose

**Benchmarking of Artificial Intelligence and Radiologists for Lung Cancer Screening in CT: The LUNA25 Challenge**

*D. Peeters*[1], B. Obreja[1], N. Antonissen[1], R. Dinnessen[1], Z. Saghir[2], E. Scholten[1], R. Vliegenthart[3], M. Prokop[1], C. Jacobs[1]; [1]Nijmegen/NL, [2]Hellerup/DK, [3]Groningen/NL
*(dre.peeters@radboudumc.nl)*

**Purpose or Learning Objective:** The imminent implementation of lung cancer screening and growing workload for radiologists demonstrates the need for safe and validated AI algorithms. At present, it is challenging to adequately validate and benchmark the increasing amount of AI algorithms being developed. In this study, we present the LUNA25 challenge, a public competition aiming to evaluate the diagnostic performance of AI algorithms and radiologists in lung nodule malignancy risk estimation at screening CT.
**Methods or Background:** The LUNA25 dataset will include 5051 screening CT scans from the National Lung Cancer Screening Trial (NLST), with 624 malignant and 7414 benign nodules. Participating teams can access this dataset to develop AI algorithms. For algorithm validation, a separate set of 65 malignant and 818 benign nodules from the Danish Lung Cancer Screening Trial (DLCST) will serve as a hidden test set. Additionally, a subset from DLCST with indeterminate nodules measuring 5-15mm in diameter will be assessed by a panel of radiologists with varying experience levels to benchmark radiologists' performance against AI algorithms. Performance will be measured using area under the ROC curve (AUC) and at different operating points in terms of sensitivity and specificity.
**Results or Findings:** With the NLST and DLCST cohorts collected, the challenge is ready to be introduced to the ECR audience. Preliminary results with an in-house developed AI algorithm demonstrated a mean AUC of 0.91 [0.87, 0.95] on DLCST.
**Conclusion:** The LUNA25 challenge expects to establish a worldwide benchmark for AI algorithms in estimating lung nodule malignancy risk at screening CTs and offer insights into how AI compares to radiologists across different experience levels and operating points.
**Limitations:** LUNA25 only benchmarks AI's stand-alone performance, and does not address workflow integration or radiologist-AI interaction, which are important for clinical adoption.
**Funding for this study:** Funding was provided by the Dutch Cancer Society
**Ethics committee - additional information:** The institutional review board waived the need for informed consent because of the retrospective design and data pseudonymization.
**Author Disclosures:**
Renate Dinnessen: Nothing to disclose
Dre Peeters: Nothing to disclose
Mathias Prokop: Nothing to disclose
Zaigham Saghir: Nothing to disclose
Ernst Scholten: Nothing to disclose
Rozemarijn Vliegenthart: Nothing to disclose
Noa Antonissen: Nothing to disclose
Bogdan Obreja: Nothing to disclose
Colin Jacobs: Nothing to disclose

**Systematic prioritisation of ai-detected chest x-ray abnormalities for optimised lung cancer detection: a multicentre study**

*R. Bramley*[1], A. Sharman[1], R. Duerden[2], S. Lyon[1], M. Ryan[3], E. Weber[4], L. Brown[1], M. Evison[1]; [1]Manchester/UK, [2]Stockport/UK, [3]Sydney/AU, [4]Linköping/SE
*(rhidian.bramley@nhs.net)*

**Purpose or Learning Objective:** This multicentre study aimed to establish a reproducible and data-driven method for selecting AI-detected chest X-ray (CXR) abnormalities to be prioritised for urgent reporting, supporting faster lung cancer diagnosis. By analysing cancer prevalence and clinical significance across two distinct cohorts from seven acute trusts, the study sought to maximise lung cancer detection while maintaining a high negative predictive value (NPV).
**Methods or Background:** The study involved two cohorts: a retrospective cohort of 1,282 CXR from primary care with detectable lung cancer (Cohort 1), and a prospective cohort of 13,802 consecutive primary care adult CXR (Cohort 2), with AI deployed in shadow mode. The Annalise-AI platform identified 124 distinct findings. An interactive tool was developed to assess prioritisation strategies based on the cancer prevalence ratio of each AI finding individually and in combination, combined with clinical judgement.
**Results or Findings:** The final prioritisation strategy flagged 41 AI findings which included 95.9% of cancers in Cohort 1 and 21.6% of CXR in Cohort 2 (sensitivity 95.87%, specificity 79.11%, PPV 4.43%, NPV 99.95%). A further 15 AI findings were prioritised based on clinical judgement as findings not associated with cancer, but requiring prioritisation as potentially needing prompt intervention.
**Conclusion:** This study demonstrates a reproducible and data-driven method for prioritising AI-detected CXR abnormalities, balancing the need for high sensitivity and NPV while reducing unnecessary prioritisation of low-risk cases. The shadow mode approach ensured clinical safety before deployment, and the interactive tool provided a systematic means to assess prioritisation strategies, offering a practical alternative to traditional judgement-based methods and supporting more efficient lung cancer diagnosis.
**Limitations:** The tool is designed to support assessment of AI performance in shadow mode in the referral population. Performance metrics should be validated before deployment in other populations.
**Funding for this study:** Funding was provided by the NHS England National AI Diagnostics fund (AIDF).
**Ethics committee - additional information:** The study was performed in shadow mode and did not impact on patient care.

**Author Disclosures:**
Rebecca Duerden: Nothing to disclose
Matthew Evison: Nothing to disclose
Rhidian Bramley: Nothing to disclose
Sarah Lyon: Nothing to disclose
Elodie Weber: Employee: Sectra
Melissa Ryan: Employee: Annalise.ai
Louise Brown: Nothing to disclose
Anna Sharman: Nothing to disclose

**Beyond Nodules: A Deep Learning Approach for Comprehensive Lung Tumour Segmentation on CT**
*L. Petrychenko*, V. Pugliese, R. G. H. Beets-Tan, L. Topff, K. Groot Lipman; Amsterdam/NL
*(l.petrychenko@nki.nl)*

**Purpose or Learning Objective:** Several commercially available AI applications for lung nodule analysis on chest CT are limited to the detection and segmentation of nodules up to 30 mm. There is clinical potential for AI-assisted volumetric analysis and treatment monitoring of lung tumours of any size, including masses. We aim to develop a Deep Learning model to detect and segment lung lesions, including primary cancers of all T-stages.
**Methods or Background:** In this retrospective study, we collected 1001 chest CT scans from 504 patients (mean age 66.4±10.3 years; 52% female) with histopathologically confirmed primary lung cancer, treated at the Netherlands Cancer Institute. Both the baseline and first follow-up scans after treatment were included. Patients were randomly assigned to 90% training and 10% testing sets. Two radiologists (4-7 years of experience) performed manual segmentation of all lung nodules ≥ 3mm and masses. The deep learning model development utilized a Residual Encoder nnUNet backbone. SGD was selected as the optimizer, $10^{-2}$ was set as the initial learning rate, 2 was set as Batch Size, and a nnUNet ResEnc XL architecture was selected.
**Results or Findings:** The dataset represented all T-stages (Tis/T1/T2/T3/T4: 1.6/34/21/16/28%) and major histopathological types, with lesion sizes ranging from 3 to 135 mm. DL model achieved a median Dice Similarity Coefficient (DSC) of 90.0% across all lung lesions, with a median of 1 false positive detection per scan. For primary lung tumors, detection sensitivity was 77.2%, and median DSC was 90.4%.
**Conclusion:** The DL model demonstrated very good segmentation performance for primary lung tumors of all sizes, including masses. The model has the potential to assist physicians in treatment monitoring and planning, though further improvements in detection sensitivity could enhance its clinical utility.
**Limitations:** The model requires both external and clinical validation.
**Funding for this study:** No additional funding was received; the study was conducted entirely at the Netherlands Cancer Institute.
**Ethics committee - additional information:** The study received Institutional Review Board (IRB) approval.
**Author Disclosures:**
Valerio Pugliese: Nothing to disclose
Kevin Groot Lipman: Nothing to disclose
Regina G. H. Beets-Tan: Nothing to disclose
Liliana Petrychenko: Nothing to disclose
Laurens Topff: Nothing to disclose

**Foundation Model-based Unsupervised CT Kernel Conversion for Standardizing Emphysema Quantification**
*D. Park*, J-H. Kang, J. Jeong; Seoul, Republic of Korea/KR
*(dhpark.ee@gmail.com)*

**Purpose or Learning Objective:** Emphysema quantification is crucial for evaluation and management of chronic obstructive pulmonary disease (COPD). Typically, emphysema is identified in computed tomography (CT) images reconstructed with smooth kernels. However, CT reconstruction kernels vary, and raw data are often deleted after reconstruction, making it hard to adjust the kernel retrospectively. Therefore, this study aims to develop and validate a method for kernel conversion to standardize emphysema quantification using a foundational deep learning model.
**Methods or Background:** Paired CT images from nine cases reconstructed with different kernels were used. Automated lung segmentation was performed using TotalSegmentator, a foundational deep learning model. An unsupervised kernel conversion method was then applied to transform the images to a pre-defined kernel. The kernel conversion was evaluated by comparing the emphysema score (ES), defined as the ratio of regions with HU below -950 within the lung area, before and after the conversion.
**Results or Findings:** Before kernel conversion, the mean ES difference between images reconstructed with smoother kernels (ex: B30f and STANDARD) and those with sharper kernels (ex: B60f and LUNG) was 11.00±6.85%. After conversion to the target smooth kernel, the mean ES difference was reduced to 2.30±2.65%. Although the sample size was small, this reduction was statistically significant based on a paired t-test (p=0.011).
**Conclusion:** The foundational model enables the conversion of CT images reconstructed with different kernels to a target smooth kernel, allowing for standardized emphysema quantification without the need for additional datasets for model development. This result suggests that the approach can be easily used by anyone with the appropriate software.
**Limitations:** For more rigorous validation, it is necessary to not only compare the difference of ES before and after kernel conversion but also comparative evaluation on ground-truth emphysema masks.
**Funding for this study:** Not applicable.
**Ethics committee - additional information:** We used a dataset from the Korea Testing Laboratory (KTL) challenge.
**Author Disclosures:**
Doohyun Park: Employee: VUNO Inc.
Jung-Hyun Kang: Employee: VUNO Inc.
Jonghun Jeong: Employee: VUNO Inc.

**Scientific Evidence of AI in Lung Nodule Evaluation on CT-examinations: A Systematic Review**
*J. Paramasamy*, J-W. Groen, A. Leliveld, B. Willems, J. Aerts, A. Odink, J. J. Visser; Rotterdam/NL
*(j.paramasamy@erasmusmc.nl)*

**Purpose or Learning Objective:** The purpose of this study was to systematically review the scientific evidence demonstrating the efficacy of CE-marked and/or FDA-cleared AI-applications for pulmonary nodule evaluation on CT examinations.
**Methods or Background:** Following the PRISMA guidelines, Medline, Embase, Web of Science, Cochrane, and Google Scholar databases were searched (Jan 1, 2012–Sep 30, 2024) for studies on AI-based evaluation of pulmonary nodules on CT-scans. Included articles were classified according to a hierarchical model of AI-efficacy: Radiology AI Deployment and Assessment Rubric (RADAR) framework. Additionally, the evolution of evidence over time was examined.
**Results or Findings:** A total of 98 articles encompassing AI-applications for lung nodule evaluation from 16 vendors were included, with approximately 90% of clinical questions addressed through cross-sectional studies. These publications primarily focused on automatic lung nodule detection, accounting for 61.8% of the studies. All included articles were classified based on their highest level of efficacy using RADAR, with the majority (41/98) at level 2 (diagnostic accuracy). Standalone nodule detection sensitivities in these studies ranged from 50% to 99%. No studies were identified at efficacy levels 5 (patient outcomes), 6 (cost-effectiveness), or 7 (local impact). The number of articles at levels 3 (diagnostic thinking) and 4 (therapeutic impact) was 1 between 2012-2016, and increased to 40 between 2020-2024.
**Conclusion:** Current scientific evidence for AI-applications in lung nodule evaluation primarily emphasizes diagnostic accuracy. However, there is a noticeable shift in research towards exploring the potential clinical impact of this technology.
**Limitations:** No meta-analysis was conducted due to significant heterogeneity in methods and reporting. Moreover, vendor involvement in most studies could potentially influence outcomes and introduce bias. Furthermore, as AI for lung nodule evaluation rapidly evolves, the included articles since 2012 may reflect variations in AI-application performance over time.
**Funding for this study:** Unrestricted institutional grant
**Ethics committee - additional information:** Systematic reviews of existing published literature
**Author Disclosures:**
Jasika Paramasamy: Nothing to disclose
Arlette Odink: Nothing to disclose
Joachim Aerts: Nothing to disclose
Asabi Leliveld: Nothing to disclose
Bo Willems: Nothing to disclose
Jacob Johannes Visser: Grant Recipient: Grant to institution from Qure.ai; consulting fees from Tegus; payment to institution for lectures from Roche; travel grant from Qure.ai; participation on a data safety monitoring board or advisory board from Quibim, Contextflow, Noaber Foundation, and NLC Ventures; leadership or fiduciary role on the steering committee of the PINPOINT Project (payment to institution from AstraZeneca) and RSNA Common Data Elements Steering Committee (unpaid); phantom shares in Contextflow and Quibim.
Jan-Willem Groen: Nothing to disclose

**On the effect of lesion number on the FROC performance in AI-based lung nodule detection**
*T. Escobar*, E. Oubel; Montpellier/FR
*(thibault.escobar@intrasense.fr)*

**Purpose or Learning Objective:** In the lung nodule detection context, we aimed to determine weather the number of lesions per patient acts as a confounding variable in performance evaluation, potentially affecting metrics like sensitivity and FROC, and if this factor should thus be rigorously controlled during testing.
**Methods or Background:** Two experiments were conducted using the LIDC-IDRI dataset. In both experiments, a trained model was evaluated on sub-groups of patients sorted by deacreasing lesion number. The first experiment formed cumulative sub-groups by adding and discarding 10 patients at a time, creating groups with varying patient numbers. To ensure no effect nor spurious correlation related to patient number, the second employed a sliding window of 100 patients with a step of 10. For each sub-group, FROC were computed based on 5-fold cross-validation predictions for the whole dataset. Additionally, false positives (FP/s), false negatives (FN/s), true positives per scan (TP/s), and sensitivity (Se) were evaluated to identify which parts of the FROC were affected.
**Results or Findings:** A clear inverse relationship between the number of lesions and FROC scores was observed. Pearson and Spearman correlation coefficients were significant and equal to -0.9 for both experiments. As lesion number increased, TP/s, FP/s, and FN/s increased, while Se decreased (i.e. TP/s increase did not compensate for FP/s and FN/s ones.).
**Conclusion:** The number of lesions per patient inversly affects the FROC in lung nodule detection models. The number of lesions per patient should thus be controlled and documented during model evaluation to ensure accurate performance assessments and to clarify under which conditions they are guaranteed. Further studies are required to rigorously examine these effects and validate the hypotheses.
**Limitations:** Limitations include no specific investigation of the sources of FP/s and FN/s increase with number of lesions.
**Funding for this study:** This study was totally funded by the compagny Intrasense SA as part of its research and development activity.
**Ethics committee - additional information:** Not applicable
**Author Disclosures:**
Thibault Escobar: Employee: Intrasense SA
Estanislao Oubel: Employee: Intrasense SA

**Optimizing Healthcare Sustainability through AI-Assisted Lung Cancer Detection at the time of initial CXR**
*J. Packer*, M. Storey, A. Chung, S. J. Rickaby, G. Dean, S. C. Shelmerdine, C. Malamateniou; London/UK
*(jack.packer1@nhs.net)*

**Purpose or Learning Objective:** Lung cancer is the leading cause of cancer mortality in the UK. Early diagnosis is essential but hindered by workforce shortages and limited CT access. This study evaluates whether the 'Artificial Intelligence triage to same-day CT' (AI-CT) pathway, using the Annalise CXR v2.3 model, can enhance healthcare sustainability by reducing patient visits, travel emissions, and administrative workload while improving CT access for suspected cancer.
**Methods or Background:** Sustainability indices for 26,660 patients (January 2022–October 2023) were assessed across five NHS centres in London. Key metrics included time from chest radiograph to CT report (Time to CT), AI accuracy, and cancer suspicion on CXR and CT, pre- and post-AI-CT implementation. Time to CT was measured using survival analysis, and diagnostic performance (AUC-ROC, F1 scores) was calculated based on CT-confirmed cancer. Time saved for patients and admin teams was estimated, and carbon reduction was calculated using the Carbon Trust online calculator.
**Results or Findings:** From 26,660 chest radiographs and 573 CT scans, 75 of 10,833 patients received same-day CT post-AI, compared to 13 of 8,434 pre-AI, eliminating 150 appointments. Each appointment saved ~1.5 hours per patient, totalling 225 hours, with ~37.5 additional hours saved for admin teams. With travel emissions estimated at 1 kgCO2e per patient, this potentially resulted in a 150 kgCO2e reduction. The AI model showed high sensitivity (91%) but low specificity (22%, F1 score 0.56). There was a significant increase in CT within 1 and 3 days post-suspicious CXR (HR 1.93, 1.34; $p < 0.001$).
**Conclusion:** The AI-CT pathway improved same-day CT access and reduced patient visits and emissions. However, the model's low specificity suggests a need for supervised triage to optimize performance.
**Limitations:** The AI’s low specificity and dependence on co-located facilities, limit generalizability.
**Funding for this study:** None
**Ethics committee - additional information:** Local trust clinical audit and QI registration forms approved.
**Author Disclosures:**
Mathew Storey: Nothing to disclose
Simon Joseph Rickaby: Nothing to disclose
Susan Cheng Shelmerdine: Nothing to disclose
Jack Packer: Nothing to disclose
Anthony Chung: Nothing to disclose
Geraldine Dean: Nothing to disclose
Christina Malamateniou: Nothing to disclose

**An artificial intelligence software for the detection of benign and non-typically benign pulmonary nodules on chest CT scans**
*S. Bennani*[1], N-E. Regnard[2], M. Durteste[1], V. Marty[1], R. Quilliet[1], A. Pourchot[1], L. Clovis[1], J. Ventre[1], G. Chassagnon[1]; [1]Paris/FR, [2]Lieusaint/FR

**Purpose or Learning Objective:** Detecting lung nodules on chest computed tomography (CT) is an important task that extends beyond the realm of lung cancer screening. This study aimed to compare the performance of radiologists to an AI software in identifying both non-typically benign and benign nodules on CT scans.
**Methods or Background:** We retrospectively collected thin-section chest CT scans from private practices across France focusing on patients aged 15 or older. The dataset included patients with non-typically benign (solid and sub-solid nodules), benign (granulomas and intrapulmonary lymph nodes), or no nodules. An expert thoracic radiologist defined the ground truth using past and follow-up scans as well as radiologist reports. We compared the performance of four radiologists who had access to limited clinical information with that of an AI software, LungCT (Gleamer, Paris, France). We conducted patient-wise ROC and lesion-wise FROC analyses.
**Results or Findings:** The final dataset included 250 chest CT scans (age = 66 ± 23 y, 117 women, 133 men). Among these, 128 scans contained at least one non-typically benign nodule, 40 displayed only benign nodules and 82 were nodule-free. The analysis focused on nodules with a diameter >6mm. The patient-wise AUC of the AI was 0.97 [0.93,1.00] and that of radiologists was 0.88 [0.83,0.92]. On a lesion-wise basis, the AI achieved a sensitivity of 79% [75%,83%] for 0.30 false positive (FP) per scan. Radiologists exhibited an average sensitivity of 72% [67%,76%] with a mean FP rate of 0.34 [0.25,0.45].
**Conclusion:** The AI solution demonstrated robust patient-wise performance and comparable lesion-wise detection of non-typically benign and benign nodules on CT scans to radiologists.
**Limitations:** The study’s retrospective design and limited sample size could affect the generalisability of results. Future research should evaluate the performance of AI-assisted radiologists.
**Funding for this study:** Gleamer (Paris, France) funded this study.
**Ethics committee - additional information:** Not applicable
**Author Disclosures:**
Nor-Eddine Regnard: Founder: Gleamer
Guillaume Chassagnon: Nothing to disclose
Vincent Marty: Employee: Gleamer
Lauryane Clovis: Employee: Gleamer
Rémi Quilliet: Employee: Gleamer
Souhail Bennani: Employee: Gleamer
Marion Durteste: Employee: Gleamer
Jeanne Ventre: Employee: Gleamer
Aloïs Pourchot: Employee: Gleamer

**Improving the generalisation of radiographic AI using automated data curation to mitigate shortcut learning**
*I. A. Selby*[1], E. González Solares[1], A. Breger[2], M. Roberts[1], J. Babar[1], F. J. Gilbert[1], N. Walton[1], C-B. Schönlieb[1], J. R. Weir-Mccall[3]; [1]Cambridge/UK, [2]Vienna/AT, [3]London/UK
*(iselby@doctors.org.uk)*

**Purpose or Learning Objective:** To investigate whether automated data curation pipelines for chest radiographs can improve deep-learning model performance on unseen data.
**Methods or Background:** Two public datasets, MIDRC-1A and MIDRC-R1, were used to develop diagnostic COVID-19 models using four architectures (DenseNet121/ResNet152V2/VGG16/EfficientNetB3). Each was trained four times using a different data curation workflow: WF1. Raw pixel data with partitioning stratified on dataset and COVID-19 status; WF2. DICOM-cleaned data with look-up tables applied, lateral projections and non-chest radiographs excluded, classes balanced on Manufacturer and Projection tags, and partitioning additionally stratified on the same metadata; WF3. Cases excluded using an open-source data-cleaning pipeline (AutoQC, https://gitlab.developers.cam.ac.uk/maths/cia/covid-19-projects/autoqc). Partitioning was stratified on projection and the presence of a pacemaker using AutoQC annotations; and WF4. The previous two workflows combined. COVID-19 diagnosis was inferred from laboratory tests, and model performance was assessed using five other public datasets. Generalisation from internal-to-external data was quantified using ΔAUCs.

Saturday

**Results or Findings:** 43,176 radiographs were included in WF1, with 33.2% (14,328) being COVID-19-positive. The development sets of the other workflows were up to 60% smaller. Similarly, the external test sets ranged from 24,563-to-38,417 patients, depending on workflow. The WF1 models experienced the largest fall in generalisation (mean ΔAUC = -0.15 [95%CI:-0.17,-0.14]), while models trained utilising AutoQC (WF3-4) demonstrated the most consistent performance with mean ΔAUCs = -0.04 [95%CI:-0.06,-0.02] and -0.02 [95%CI:-0.04,0.00] for WF3 and WF4 ($p<0.05$). The WF2 models had a mean ΔAUC = -0.07 [95%CI:-0.09,-0.05].
**Conclusion:** Automated data curation can improve the generalisation of deep learning models for chest radiographs, facilitating more consistent performance on data from new locations and equipment.
**Limitations:** Future work should evaluate the tool in multiclassification tasks and non-COVID-19 datasets. In addition to the current pacemaker detection, tools for a broader range of support apparatus are necessary.
**Funding for this study:** The authors wish to acknowledge support from the EU/EFPIA Innovative Medicines Initiative 2 Joint Undertaking - DRAGON (101005122) (I.S., A.B., M.R., L.E.S., J.B., C.-B.S., E.S., J.W.M., AIX-COVNET); the National Institute for Health and Care Research (NIHR) Cambridge Biomedical Research Centre (BRC-1215-20014) (I.S., L.E.S., J.H.F.R., E.S., J.W.M.); Wellcome Trust (J.H.F.R.), British Heart Foundation (J.H.F.R.); the EPSRC Cambridge Mathematics of Information in Healthcare Hub EP/T017961/1 (M.R., J.H.F.R., C.-B.S.); Cancer Research UK (CRUK) National Cancer Imaging Translational Accelerator (NCITA) [C42780/A27066] (L.E.S.); Cambridge Mathematics of Information in Healthcare (CMIH) Hub EP/T017961/1; Austrian Science Fund (FWF, project T-1307) (A.B.); and the Trinity Challenge BloodCounts! project (M.R., C.-B.S.). The AIX-COVNET collaboration is also grateful to Intel for financial support.
C.B.S. additionally acknowledges support from the Philip Leverhulme Prize, the Royal Society Wolfson Fellowship, the EPSRC advanced career fellowship EP/V029428/1, EPSRC grants EP/ S026045/1 and EP/T003553/1, EP/N014588/1, EP/T017961/1, the Wellcome Innovator Awards 215733/Z/19/Z and 221633/Z/20/Z, the European Union Horizon 2020 research and innovation program under the Marie Skodowska-Curie grant agreement No. 777826 NoMADS, the Cantab Capital Institute for the Mathematics of Information and the Alan Turing Institute.
Please note that the content of this publication reflects the authors’ views and that neither IMI nor the European Union, EFPIA, or the DRAGON consortium are responsible for any use that may be made of the information contained therein.
**Ethics committee - additional information:** The Brent Research Ethics Committee, the Health Research Authority (HRA), and Health and Care Research Wales (HCRW) provided ethical approval for our retrospective study (IRAS ID: 282705, REC No.: 20/HRA/2504, R&D No.: A095585). Informed consent was not required as data was pseudonymised.
**Author Disclosures:**
Carola-Bibiane Schönlieb: Nothing to disclose
Eduardo González Solares: Nothing to disclose
Michael Roberts: Nothing to disclose
Fiona J. Gilbert: Nothing to disclose
Jonathan R. Weir-Mccall: Nothing to disclose
Anna Breger: Nothing to disclose
Ian Andrew Selby: Nothing to disclose
Judith Babar: Nothing to disclose
Nicholas Walton: Nothing to disclose

### Sunday, March 2

**08:00-09:00** **Research Stage 1**

### Research Presentation Session: Breast

### RPS 2202

### Personalised risk prediction of breast cancer

**Moderator**
P. Clauser; Vienna/AT
*(clauser.p@hotmail.it)*
**Author Disclosures:**
Paola Clauser: Board Member: EUSOBI; Grant Recipient: Siemens

**Associations of automatically measured breast density with breast cancer risk and duration of the pre-clinical detectable phase in a Dutch screening cohort**
*J. Peters*[1], D. Van Der Waal[1], M. Smid-Geirnaerdt[1], C. Van Gils[2], M. Broeders[1]; [1]Nijmegen/NL, [2]Utrecht/NL
*(Jim.Peters@radboudumc.nl)*

**Purpose or Learning Objective:** Breast density could impact screening strategies. Women with dense breasts face higher breast cancer risk. Furthermore, lesion masking may shorten the pre-clinical detectable phase (PCDP), increasing interval cancer rates. This study examines the associations of automated breast density measures with breast cancer risk and PCDP duration.
**Methods or Background:** Digital mammograms were used from 60,739 participants in a prospective Dutch screening cohort (PRISMA study, 2014-2019). Dense volume (DV,cm3), volumetric breast density (VBD,%) and Volpara Density Grade (VDG1-4) were assessed using Volpara version 1.5.0. Breast cancer diagnoses were ascertained through linkage with the Netherlands Cancer Registry. Participants with prior breast cancer (N=73) or screen-detected breast cancer at study entry (N=401), were excluded. Information on time to screen-detected and interval cancers was used to fit a three-state (1:cancer-free, 2:pre-clinical detectable cancer, 3:clinical cancer) Markov regression model. Hazard ratios (HRs) were calculated for the effects of breast density on state transition intensities 1->2 (=breast cancer risk) and 2->3 (=1/PCDP duration).
**Results or Findings:** After a median 4.2 years (IQR 3.9–4.6) we observed 430 screen-detected and 316 interval cancers. Log-transformed VBD and DV were positively associated with increased breast cancer risk (HR 1.12 [95%CI 1.05-1.21] and HR 1.32 [95%CI 1.05-1.35] per one-standard-deviation increase, respectively). Both measures were associated with shorter PCDP (HR 1.48 [95% CI 1.30-1.69] and HR 1.19 [95%CI 1.05-1.35]). Mean PCDP duration was 1.63 years [95%CI 1.11–2.41] for women with highest density (VDG4), compared to 3.41 years [95%CI 3.31-3.52] for VDG1-3.
**Conclusion:** Breast density is associated with breast cancer risk and PCDP duration. VBD has the strongest association with PCDP, indicating a reduced sensitivity of biennial mammography, while DV contains more information on breast cancer risk.
**Limitations:** Other confounders than age, e.g. BMI, were not yet included in the model.
**Funding for this study:** The PRISMA study is funded by ZonMw and KWF.
**Ethics committee - additional information:** CMO Arnhem-Nijmegen
**Author Disclosures:**
Jim Peters: Nothing to disclose
Carla Van Gils: Nothing to disclose
Maartje Smid-Geirnaerdt: Nothing to disclose
Mireille Broeders: Nothing to disclose
Danielle Van Der Waal: Nothing to disclose

**Mammographic biomarkers of cardiovascular risk: the BAKER study**
*D. Capra*, O. Hoda, C. B. Monti, M. Zanardo, F. Sardanelli; Milan/IT
*(davide.capra@unimi.it)*

**Purpose or Learning Objective:** Mammography could offer two sex-specific biomarkers to spotlight cardiovascular risk in women: breast arterial calcifications (BAC) and breast density. We conducted a prospective case-control study evaluating the association between BAC and gynaecological and cardiovascular risk factors.
**Methods or Background:** Consecutive women showing BAC and age- and breast density-matched controls referred for annual mammography were prospectively enrolled. We recorded anthropometric variables, traditional cardiovascular risk factors and gynaecological risk factors. Breast density was classified as low breast density (BI-RADS categories A and B) or high breast density (BI-RADS C and D).
**Results or Findings:** 72 BAC patients and 72 controls were enrolled (median age 70.0 years, IQR 62.5 to 78 years). Women with BAC had a younger age at menopause (50 vs 52 years, p=0.008), and showed associations with breastfeeding (p=0.041) and parity (p=0.038). Women with BAC show a borderline significant trend towards the use of anti-ipertensive medications (p=0.092). No other differences between cases and controls were observed (p>0.101). Higher breast density was significantly associated with younger age (p<0.001), lower body weight (p<0.001), lower systolic blood pressure (p=0.003), and higher HDL cholesterol (p=0.017), whereas lower breast density was associated with longer time since menopause (p=0.003) and use of anti-ipertensive medications (p<0.001).
**Conclusion:** Women with BAC have a younger menopausal age, which represents a precocious shift towards a less favourable cardiometabolic hormonal balance. Similarly, women with low breast density show an unfavourable cardiovascular risk profile, using more often anti-ipertensive medications, having a higher systolic blood pressure and lower levels of HDL cholesterol.
**Limitations:** Age and breast density matching reduces the statistical power to observe associations between breast density and cardiovascular risk factors among women of similar age. Absence of follow up to record cardiovascular events.
**Funding for this study:** General Electric Healthcare supported this study.
**Ethics committee - additional information:** Ethics committe approval number 90/INT/2020, 08/09/2020. All participants signed informed consent.
**Author Disclosures:**
Francesco Sardanelli: Advisory Board: Bayer AG Advisory Board: Siemens Healthineers Advisory Board: Bracco Imaging Advisory Board: Esaote Advisory Board: GE Healthcare
Omar Hoda: Nothing to disclose
Caterina Beatrice Monti: Nothing to disclose
Moreno Zanardo: Nothing to disclose
Davide Capra: Nothing to disclose

**Short-term risk prediction of breast cancer comparing risk tools for digital mammography and digital breast tomosynthesis in U.S. screening populations**
E. F. Conant[1], C. Parghi[2], P. Hall[3], *M. Eriksson*[3]; [1]Philadelphia, PA/US, [2]Addison, TX/US, [3]Stockholm/SE
*(mikael.eriksson@ki.se)*

**Purpose or Learning Objective:** Image-derived AI-based risk models demonstrate ability to predict breast cancer risk using digital mammography (DM) and digital breast tomosynthesis (DBT) imaging data. However, a direct comparison of performances within the same screening population has yet to be conducted.
**Methods or Background:** We conducted a nested case-control study including women aged 35-98 from Solis and UPenn screening cohorts, between 2014 and 2021. Participants were followed for two screens, with cancer diagnosed before August 2022. Two image-based ProFound AI Risk models, one for DM and one for DBT, estimated absolute 1-year breast cancer risks at study-entry. We assessed models' discriminatory performance (AUC) controlling for screening site and classified risks according to U.S. Preventive Services Task Force (USPSTF) thresholds.
**Results or Findings:** Study included 780 women with incident breast cancer (mean age 63.4±11.2) and 7,481 controls (mean age 57.0±10.6). Cancers were diagnosed on average 1.3±0.5 years (range 4 months to 4 years) after study-entry. At study entry, AUCs of DM and DBT models were 0.71 (95% CI: 0.69-0.73) and 0.75 (95% CI: 0.73-0.77), respectively (p<0.01). Comparing UPenn and Solis data, similar estimates were observed for respective models. Based on USPSTF guidelines, 14% of women were classified as high-risk. Among this group, DM model predicted 40% (95% CI: 36-43%) of future breast cancers, compared to 48% (95% CI: 44-52%) by DBT model (p<0.01). Non-significant differences in proportions of future breast cancers were observed comparing sites.
**Conclusion:** The image-derived DM and DBT AI-risk models predicted 40-48% of future breast cancers at study-entry in two U.S. screening populations. The DBT model predicted a significantly higher proportion of future cancers compared to the DM model emphasizing the need for some women to obtain supplemental screening.
**Limitations:** Study is limited to only 2 sites in U.S.
**Funding for this study:** iCAD, Inc. Swedish Research Council Swedish Breast Cancer Association
**Ethics committee - additional information:** The study has been reviewed and approved.
**Author Disclosures:**
Mikael Eriksson: Patent Holder: iCAD, Inc.
Chirag Parghi: Nothing to disclose
Per Hall: Patent Holder: iCAD, Inc.
Emily F. Conant: Advisory Board: iCAD, Inc.

**Impact of Breast Density Metrics on Personalized Breast Cancer Screening Protocols**

*G. Gennaro*[1], L. Bucchi[2], A. Ravaioli[2], F. Caumo[1]; [1]Padova/IT, [2]Meldola/IT
*(gisella.gennaro@iov.veneto.it)*

**Purpose or Learning Objective:** To evaluate the impact of different breast density metrics on the personalization of breast cancer screening.
**Methods or Background:** The RIBBS study (ClinicalTrials.gov NCT05675085) is a personalized breast screening study targeting young women. The protocol used digital breast tomosynthesis (DBT) and double reading to stratify participants based on individual breast cancer risk and breast density. In this protocol, breast density determined the need for supplemental ultrasound (US), while breast cancer risk guided the frequency of screening. A quantitative software tool provided volumetric breast density, with a 25% threshold used to identify women who needed supplemental US. This study compares stratification based on this volumetric approach with stratification using categorical breast density metrics, both objective and human.
**Results or Findings:** A total of 10,269 women, all aged 45 years, were enrolled in the RIBBS study. Of these, 1,904 women (18.5%) had ≥25% breast volumetric density and underwent additional US. Using the categorical breast density provided by the same software, 41.1% of participants would have been categorized as having a BIRADS "d" category density, 2.2 times higher than that identified through the volumetric metric. If BIRADS categorization had been performed by human readers, considering every DBT classified as "d" by at least one reader, the percentage would have dropped to 32.3%, still 1.7 times higher than the current protocol.
**Conclusion:** This study shows that the choice of breast density metrics can significantly influence the stratification process in personalized breast cancer screening. Quantitative metrics allow more precise stratification than categorical approaches, improving the feasibility of supplemental imaging in clinical practice.
**Limitations:** Changes in screening performance due to the use of categorical breast density for supplemental US remain unassessed, as the current results are based solely on the applied protocol.
**Funding for this study:** This specific subanalysis had no funding
**Ethics committee - additional information:** The RIBBS study was approved by the Ethics Committee with the following code "RIBBS 2019/37"
**Author Disclosures:**
Francesca Caumo: Nothing to disclose
Lauro Bucchi: Nothing to disclose
Alessandra Ravaioli: Nothing to disclose
Gisella Gennaro: Nothing to disclose

**Changes in Mammographic Density for Breasts Developing and not Developing Breast Cancer**

*J. Gjesvik*, N. Moshina, S. Sagstad, M. Larsen, Å. S. Holen, M. B. Bergan, S. Hofvind; Oslo/NO
*(jogj@kreftregisteret.no)*

**Purpose or Learning Objective:** The evidence on longitudinal changes in mammographic density in breasts developing cancer is limited. We aimed to analyse mammographic density among women developing and not developing breast cancer over three consecutive screening rounds in BreastScreen Norway.
**Methods or Background:** In this retrospective cohort study, 66,696 women aged 50-69 years with three consecutive screening examinations performed in Rogaland and Hordaland counties, 2007-2020, were included. A total of 909 women were diagnosed with screen-detected and 287 with interval cancer. Mammographic density data was obtained from an automated software (Volpara 1.5.0 and 1.5.4.0) and included absolute (cm3) and percent (%) dense volume per woman and breast. A linear mixed-effects model with a fixed effect for each woman was applied on a breast level to define the changes in absolute and percent dense volume. The model was adjusted for age at entry, breast volume, and history of benign breast disease.
**Results or Findings:** Mean age for women not developing breast cancer was 62.5 years (standard deviation, SD: 5.1), while it was 62.3 (SD: 4.4) for women with screen-detected cancer and 61.9 (SD: 4.8) for interval cancer. Absolute and percent dense volume decreased over time in all women. In breasts developing cancer the rate of decrease was lower for absolute dense volume, estimate=0.004 (95% CI: 0.002-0.007, p=0.041), compared to breasts not developing cancer. The rate of decrease was also lower for percent dense volume, estimate=0.003 (95%CI 0.000-0.007, p=0.053), in breasts developing versus not developing cancer.
**Conclusion:** Absolute dense volume decreased to a lower degree in breasts developing versus not developing cancer. Longitudinal changes in absolute dense volume could be used for more precise breast cancer risk prediction and screening personalization.
**Limitations:** The study is retrospective and the population is fairly homogenous.
**Funding for this study:** No funding
**Ethics committee - additional information:** Regional Commitee for Medical and Health Research Ethics
**Author Disclosures:**
Nataliia Moshina: Nothing to disclose
Jonas Gjesvik: Nothing to disclose
Marie Burns Bergan: Nothing to disclose
Åsne Sørlien Holen: Nothing to disclose
Silje Sagstad: Nothing to disclose
Solveig Hofvind: Nothing to disclose
Marthe Larsen: Nothing to disclose

**Using Artificial Intelligence to Detect Subclinical Breast Cancer**

*J. Gjesvik*[1], N. Moshina[1], C. Lee[2], D. L. Miglioretti[3], S. Hofvind[1]; [1]Oslo/NO, [2]Seattle, WA/US, [3]Davis, CA/US
*(jogj@kreftregisteret.no)*

**Purpose or Learning Objective:** Investigate whether an artificial intelligence algorithm (AI) trained for detecting breast cancer scored the breast developing breast cancer and the breast not developing breast cancer differently years before diagnosis.
**Methods or Background:** In this retrospective cohort study, we included women aged 50-69 who attended three consecutive biennial screening rounds between 2004 and 2018, as part of BreastScreen Norway. A total of 116 495 women were included in the final study population, 1265 with screen-detected breast cancer detected at, and 342 with interval cancer diagnosed within two years after, the third screening round. We used a commercial AI algorithm to score each breast with a risk score between 0 and 100.
**Results or Findings:** For women developing screening-detected breast cancer the mean AI-score at the first screening round for the breast developing breast cancer was 19.2 (SD: 28.6), and 82.7 (SD: 26.7) after the third screening round. The score was 9.5 (SD: 19.0) in the first and 5.0 (SD: 15.7) in the third screening round for the breast not developing breast cancer. For interval cancer, the mean scores for breasts developing cancer were 17.8 (SD:26.3) and 33.1 (SD: 33.8), respectively, and mean scores for breasts not developing cancer were 10.5 (SD: 19.9) and 8.4 (SD: 18.7), respectively. For women not developing breast cancer, the mean AI score was 7.1 (SD: 15.2) in the first and 6.4 (SD: 14.5) in the third screening rounds, respectively.
**Conclusion:** AI-scores were higher in breasts developing cancer up to 6 years before it was diagnosed. The findings suggests that commercial AI algorithms for breast cancer detection might be considered for identifying women at higher risk of developing breast cancer.
**Limitations:** This is a retrospective study, and the population is mostly homogenous.
**Funding for this study:** Funded by the Norwegian Cancer Society (Pink Ribbon)
**Ethics committee - additional information:** Regional Committee for Medical and Health Research Ethics, Norway
**Author Disclosures:**
Christopher Lee: Nothing to disclose
Nataliia Moshina: Nothing to disclose
Jonas Gjesvik: Nothing to disclose
Diana L Miglioretti: Nothing to disclose
Solveig Hofvind: Nothing to disclose

**External validation of a mammographic masking prediction model in the Dutch Breast Cancer Screening Program**

*S. D. Verboom*[1], J. G. Mainprize[2], J. Peters[1], M. Broeders[1], M. Yaffe[2], I. Sechopoulos[1]; [1]Nijmegen/NL, [2]Toronto, ON/CA
*(sarah.verboom@radboudumc.nl)*

**Purpose or Learning Objective:** To externally validate a lesion masking prediction model for mammograms, Mammatus, developed on a North American cohort, in a larger retrospective breast cancer screening cohort from one screening center in The Netherlands.
**Methods or Background:** A total of 935 digital mammograms from the Dutch Breast Cancer Screening Program with a unilateral invasive breast cancer that was either screen detected or diagnosed within 24 months after a negative screening (interval cancer) were included. All mammograms were retrospectively evaluated for the visibility of malignant masses using all available diagnostic imaging and clinical information. Mammatus was applied on the contralateral mammogram to eliminate the influence of the lesion. The area under the receiver operator characteristics (ROC) curve (AUC) when using Mammatus to distinguish examinations with screen-detected cancers (assumed low masking risk) from interval cancers (assumed high masking risk) was computed. The AUC was compared to that of the original cohort and to that obtained using volumetric breast density (VBD) as a predictor. A second three-category ROC analysis was performed, with interval cancers that were retrospectively visible classified as intermediate lesion masking.

**Results or Findings:** Mammatus achieved an AUC of 0.70 (95%CI 0.67-0.74) for distinguishing between screen-detected- (n=632) and interval-cancer exams (n=303). This performance did not differ from the original study (AUC=0.75 (0.68-0.82), p=0.20), and outperformed VBD (AUC=0.66 (0.62-0.70, p<0.002). The three-category ROC analysis showed that Mammatus outperformed VBD at identifying low risk of lesion masking (AUC=0.74 (0.70-0.77)), however, not for identifying high risk (AUC=0.69 (0.65-0.74)).
**Conclusion:** Mammatus performed well in predicting breast cancer-masking risk in a Dutch screening cohort. This suggests that adding information other than density improves prediction of lesion masking.
**Limitations:** There is no ground truth of lesion masking risk, therefore the best possible approximation is used.
**Funding for this study:** aiREAD financed by the Dutch Research Council (NWO), Dutch Cancer Society (KWF), and Health Holland (HH)
**Ethics committee - additional information:** The Radboudumc ethics committee declared that this study falls outside the scope of the Dutch Medical Research involving Human Subjects Act and could be carried out without approval of an Institutional Review Board.
**Author Disclosures:**
Jim Peters: Nothing to disclose
Mireille Broeders: Research/Grant Support: Hologic, Screenpoint Medical, Sectra Benelux, Volpara Healthcare, Lunit, and iCAD Speaker: Hologic and Siemens Healthcare
Sarah Delaja Verboom: Nothing to disclose
James G. Mainprize: Founder: Calavera Surgical Design, Inc. Research/Grant Support: GE Healthcare
Martin Yaffe: Research/Grant Support: GE Healthcare
Ioannis Sechopoulos: Speaker: Canon, Siemens Healthcare Research/Grant Support: Siemens Healthcare, Canon Medical Systems, ScreenPoint Medical, Sectra Benelux, Volpara Healthcare, Lunit Advisory Board: Koning Corp.

**08:00-09:00** **Research Stage 2**

### Research Presentation Session: Neuro

### RPS 2211

### Contrast agents and energy sustainability in neuroimaging

**Moderator**
Y. Özsunar; Aydın/TR
*(yeldaozsunar@gmail.com)*

**Efficacy of gadoquatrane, a novel low-dose high relaxivity macrocyclic gadolinium-based contrast agent, at 5-, 10- and 15-min post injection in comparison to gadobutrol for CNS CE-MRI**
*C. Deuschl*[1], K. Kudo[2], A. Liu[3], M. A. Klemens[3], B. M. Hofmann[3], P. Palkowitsch[3], B. P. Liu[4]; [1]Essen/DE, [2]Sapporo/JP, [3]Berlin/DE, [4]Chicago, IL/US
*(cornelius.deuschl@uk-essen.de)*

**Purpose or Learning Objective:** This dose-finding Phase 2 study for the novel tetrameric macrocyclic GBCA gadoquatrane investigated the efficacy of 0.04 mmol Gd/kg body weight (bw) gadoquatrane in comparison to 0.1 mmol Gd/kg bw gadobutrol for CE-MRI in patients with CNS lesions at 5-, 10- and 15-min post injection (pi).
**Methods or Background:** Adult patients with CNS lesions were included in the study and underwent two CE-MRIs within an interval of 3 to 14 days, first gadobutrol, then gadoquatrane. Images were acquired at 5- (3D IR-GRE), 10- (2D-SE) and 15-min pi (3D IR-GRE). The 5-, 10- and 15-min CE-MRIs were assessed in the Blinded Independent Central Review using visualization parameters (contrast enhancement, border delineation, internal morphology) to investigate the time course of enhancement. Quantitative signal intensity measurements were also performed. Safety parameters were assessed and pharmacokinetic (PK) samples were taken to determine plasma concentrations for both GBCAs.
**Results or Findings:** The sum of the lesion visualization parameters at each point for the average reader was very similar for gabobutrol (9; 8.86; 9.09) and gadoquatrane (8.94; 8.8; 8.97), with differences close to zero (-0.06; -0.06; -0.12). These results were representative for the three individual readers and each visualization parameter. The results of the quantitative measurements supported the qualitative evaluations. The safety and concentration-time profile of gadoquatrane were very similar to gadobutrol, but dose-proportionally lower.
**Conclusion:** Gadoquatrane at a dose of 0.04 mmol Gd/kg bw shows similar efficacy as gadobutrol at 0.1 mmol Gd/kg bw on 5-, 10- and 15-min pi CE-MRI images.
**Limitations:** The limitation of the study is the small sample size of 50 evaluable patients.
**Funding for this study:** This Phase 2 study was sponsored by Bayer AG.
**Ethics committee - additional information:** Ethics vote from all sites available.
**Author Disclosures:**
Alex Liu: Employee: Bayer
Benjamin P. Liu: Nothing to disclose
Cornelius Deuschl: Nothing to disclose
Petra Palkowitsch: Employee: Bayer
Kohsuke Kudo: Nothing to disclose
Birte Maria Hofmann: Employee: Bayer
Mark Alexander Klemens: Employee: Bayer

**Clinical experience with gadopiclenol in patients with brain metastases: Single and double doses, comparison of standard and delayed scanning**
*J. Vymazal*, Z. Ryznarova, R. Liščák, A. Rulseh; Prague/CZ
*(josef.vymazal@homolka.cz)*

**Purpose or Learning Objective:** To demonstrate the role of gadopiclenol in MR imaging of brain metastases with at standard (0.05 mmol/kg) and double dose (0.1 mmol/kg), including delayed examination (ca 15 minutes after contrast administration).
**Methods or Background:** Ninety-one subjects with known brain metastases underwent MRI prior to radiosurgery (44 female, mean age 64.2 ±12 years). A standard T1W 3D SPACE sequence was acquired in all subjects (3 subjects at 1.5T, 88 at 3T) post-contrast, followed by a delayed acquisition. In roughly half of subjects (n=47), the delayed acquisition was preceded by additional contrast administration (cumulative double dose). After randomization, 3 blinded readers evaluated the number of lesions for each patient in 2 sessions, 1 month apart. A paired t-test was used to compare early and late evaluations in the two groups (subjects with cumulative single dose or double dose), the Welch t-test was used to compare differences in (late-early) single versus (late-early) double doses.
**Results or Findings:** Agreement between all three blinded readers was used for evaluation. The late single dose exam revealed 170 metastatic lesions compared to 161 metastatic lesions on the early exam. Late double-dose exam revealed 138 metastatic lesions compared to 121 metastatic lesions on the single dose early exam. The number of lesions on early versus late (single/double dose) exams did not differ significantly (p=0.4 and p=0.11, respectively). No significant difference was detected between double dose and delayed single dose.
**Conclusion:** Although relatively more lesions were detected on delayed exam (single/double dose), the difference did not reach significance, likely reflecting small effect size. Furthermore, no significant difference was found between late single and double dose.
**Limitations:** The study was performed at a single institution. Only the SPACE sequence was used for blinded reading.
**Funding for this study:** Supported by MH CZ - DRO (NNH, 00023884) No. 244302
**Ethics committee - additional information:** Approved on June, 5, 2024
**Author Disclosures:**
Roman Liščák: Author: co-author
Zuzana Ryznarova: Author: co-author
Josef Vymazal: Consultant: Bracco Consultant: GE Healthcare
Aaron Rulseh: Author: corresponding author

**Global body exposure to gadolinium after administration of the Human Equivalent Dose (HED) of gadopiclenol, in comparison to the HED of gadobutrol and gadoterate in Healthy Rats**
*M. Rasschaert*, E. Couloumy, C. Hollenbeck, E. Renard, I. Janot, N. Decout, M. Lefebvre, C. Factor, P. Robert; Roissy CdG Cedex/FR

**Purpose or Learning Objective:** Gadopiclenol is a high relaxivity macrocyclic Gadolinium-Based Contrast Agent (mGBCA) approved in 2022 in the USA and December 2023 in Europe. The aim of this study was to evaluate the time-dependent gadolinium Gd exposure in main body organs over 5 months in rats after a single injection of gadopiclenol or two mGBCAs.
**Methods or Background:** Healthy female rats were allocated to 3 groups: gadopiclenol (Elucirem®), gadobutrol (Gadovist®) and gadoterate (Dotarem®). One single administration of GBCA at the Human Equivalent Dose was realized, blinded. Selected tissues (including central (CNS) and peripheric nervous system (PNS) organs, excretion organs, bone) were collected for total Gd determination by Inductive Coupled Plasma-Mass Spectrometry at 1 day, 1 week, 1 or 5 months after injection (n=10/group and timepoint). The global exposure to Gd in all investigated organs was estimated by calculating the area under the curve (AUC).

Sunday

**Results or Findings:** After gadopiclenol administration, the Gd exposure over the 5 months compared to gadoterate and gadobutrol was respectively -43% and -41% in CNS, -28% and -32% in PNS (sciatic nerve, spinal nodes, footpads, spinal cord), -25% and -68% in skin, -1% and -26% in liver, -19% to -44% in kidney and -31% to -39% in spleen. In bone, the Gd exposure after gadopiclenol administration was +80% and +16% higher in femur diaphysis, -1% and +48% in epiphysis. In bone marrow, the AUC was -22% and -35% lower compared to gadobutrol and gadoterate respectively. Globally, the overall Gd exposition was -25% and -40% after gadopiclenol compared to gadoterate and gadobutrol, respectively.
**Conclusion:** In our experimental conditions, the measured exposure to Gd after gadopiclenol injection is significantly reduced compared to the other marketed GBCAs in most of the investigated organs.
**Limitations:** Animal Study
**Funding for this study:** None
**Ethics committee - additional information:** Research project 2015080615264829 approved by French MESR Ministry
**Author Disclosures:**
Claire Hollenbeck: Employee: Guerbet
Philippe Robert: Employee: Guerbet
Cecile Factor: Employee: Guerbet
Ilona Janot: Employee: Guerbet
Nathalie Decout: Employee: Guerbet
Mylène Lefebvre: Employee: Guerbet
Emilie Couloumy: Employee: Guerbet
Elisabeth Renard: Employee: Guerbet
Marlène Rasschaert: Employee: Guerbet

**Use of gadolinium-based contrast agents (GBCA) over the years: impact on monitoring multiple sclerosis disease activity**
S. M. Sceppacuercia[1], C. Tur[1], *A. H. M. E. Hammam*[2], W. H. E. Hamed[2], O. Sarwani[2], D. Deborah[1], C. Auger Acosta[1], T. A. Yousry[2], A. Rovira Cañellas[1]; [1]Barcelona/ES, [2]London/UK
*(zekus83@gmail.com)*

**Purpose or Learning Objective:** To describe the evolution of GBCA use, the detection of Gd-enhancing, and new T2 lesions over time in two independent cohorts of MS patients. We also examined the association between new T2 lesions and clinical features, focusing on the role of GBCA use.
**Methods or Background:** We included all MS patients clinical MRIs at the Multiple Sclerosis Centre of Catalonia, Spain, and the Queen Square MS Centre, UK, during May from 2015 to 2022. Clinical and demographic data included age, sex, disease duration, clinical phenotype, progression, relapses since the last MRI, and DMT variables. Brain MRI data included GBCA use, the number of Gd+ lesions, and new T2 lesions. Statistical analysis used logistic regression models adjusted for confounders.
**Results or Findings:** 479 patients(cohort1), and 794 (cohort2). Both cohorts showed a significant decrease in GBCA use over time, an increase in DMT exposure ($p=0.032$), and a reduction in relapses ($p<0.001$) and progression events ($p=0.006$). A significant reduction in Gd-enhancing lesions was observed in Barcelona. The detection of new lesions also decreased over time, likely due to an older population ($p<0.001$) and increased use of high-efficacy DMTs ($p<0.001$). GBCA administration was independently associated with a higher likelihood of detecting new lesions ($p=0.030$).
**Conclusion:** Over time, there was a decrease in GBCA use, Gd-enhancing lesion detection, and new/enlarging T2 lesions, suggesting a trend towards a less aggressive disease course, possibly due to more effective DMTs and an aging population. GBCA use was independently associated with higher detection of new T2 lesions, possibly reflecting heightened neuroradiologist vigilance when new inflammatory activity is suspected and the facilitation of T2 lesion detection by Gd-enhancing lesions.
**Limitations:** This includes the retrospective nature, a single month/year representation and unequal MRIs' frequencies among years and centres.
**Funding for this study:** A retrospective study with no fund needed.
**Ethics committee - additional information:** A retrospective study.
**Author Disclosures:**
Sofia Maria Sceppacuercia: Nothing to disclose
Tarek A. Yousry: Nothing to disclose
Weaam Hamed Elsayed Hamed: Nothing to disclose
Alejandro Rovira Cañellas: Other: Alex Rovira serves/ed on scientific advisory boards for Novartis, Sanofi, Synthetic MR, BMS, Roche, and Biogen, and has received speaker honoraria from Bayer, Sanofi, Merck-Serono, Teva Pharmaceutical Industries Ltd, Novartis, Roche, BMS and Biogen, and is CMO and co-founder of TensorMedical.
Ahmed Hassan Mohamed Elzeki Hammam: Nothing to disclose
Carmen Tur: Other: Carmen Tur has received honoraria from Roche and Novartis and is a steering committee member of the O'HAND trial and of the Consensus group on Follow-on DMTs.
Cristina Auger Acosta: Nothing to disclose
Omran Sarwani: Nothing to disclose
Deborah Deborah: Nothing to disclose

**Different rates and patterns of symptoms associated with gadolinium exposure (SAGE) between linear and macrocyclic gadolinium based contrast agents**
*I. Shahid*, E. Lancelot; Paris/FR
*(imran.shahid@guerbet.com)*

**Purpose or Learning Objective:** Some patients who received multiple administrations of gadolinium-based contrast agents (GBCAs) have been reported to develop "symptoms associated with gadolinium exposure" (SAGE). The aim of this study was to analyze pharmacovigilance data and to explore the various SAGE patterns of linear and macrocyclic GBCAs among patients exhibiting three or more SAGE symptoms.
**Methods or Background:** SAGE symptoms were searched by preferred terms (PTs) in different system organ class (SOC) categories in the FDA Adverse Event Reporting System (FAERS) database, over a 6-year period ranging from 2014 to 2019, for 3 linear and 3 macrocyclic GBCAs.
**Results or Findings:** The analysis of FAERS data revealed a significantly higher SAGE weight for the linear GBCAs (20-24%) than for the macrocyclic GBCAs (5-9%). For the linear GBCAs, the most prevalent combinations of 3 SAGE symptoms were reported in 152-164 occurrences, while for the macrocyclic GBCAs this range was significantly lower (1-13 occurrences). Moreover, the patterns of SAGE combinations differed significantly between both categories of GBCAs.
**Conclusion:** Linear GBCAs are associated with higher SAGE reporting rates than macrocyclic GBCAs. They also present different patterns of SAGE combinations.
**Limitations:** This study did not provide any demonstration of a causal relationship between the reported events and GBCA administration to the patients.
**Funding for this study:** None
**Ethics committee - additional information:** Not Applicable (No patients were involved)
**Author Disclosures:**
Eric Lancelot: Employee: Guerbet
Imran Shahid: Employee: Guerbet

**How Compressed-Sense acceleration technique impacts the Central Nervous System Magnetic Resonance Imaging energy consumption**
*S. Kalari*[1], I. Seimenis[1], E. Psatha[2], D. Apostolou[1], I. Loulakas[1], E. Karavasilis[2]; [1]Athens/GR, [2]Alexandroupolis/GR
*(raykalari@gmail.com)*

**Purpose or Learning Objective:** Compressed-sensing is a new acquisition technique that enables efficient signal acquisition and reconstruction reducing the scanning time. The aim of our study was to estimate the impact of CS on energy consumption during brain imaging.
**Methods or Background:** Thirty individuals underwent brain MRI using the same exam protocol including 3DT1, 3DT2 and 3DT2 Flair with and without CS on a 3.0TPhilips MRI system. Energy consumption was recorded using kilowatt-hour energy measurement sensors (0.017 Hz sampling rate) for all the above sequences. The 3DT1, 3DT2 and 3DT2 Flair with and without CS image quality was assessed quantitatively using VolBrain free available online software. Images were also reviewed from an experienced neuroradiologist who evaluated their total image quality and 3DT2 Flair lesion detection sensitivity. Sequences' signal to noise ratio (SNR), image quality indices derived from VolBrain, qualitative indices and their energy consumption were introduced to SPSS to perform paired t-test statistical analysis ($p<0.05$).
**Results or Findings:** The mean acquisition time of 3DT2 FLAIR, 3DT2 and 3DT1 with and without CS were 210sec±0.8sec vs 390sec±1.1sec, 200sec±0.7sec vs 370sec±1sec and 230sec±0.1sec vs 400sec±1.2sec respectively. We didn't find any statistical differences in both image qualitative and quantitative quality metrics. There was no substantial difference of the SNR. Concerning the energy consumption/min CS increased by 42% the energy consumption rate in 3DT2 FLAIR (0.57kWh/min versus 0.40kWh/min) therefore the total energy consumption was decreased by 10% (1,99 versus 2.2 kWh). 3DT2 and 3DT1 energy with and without CS consumption rate were 0.60kWh/min, 0.56kWh/min and 0.58kwh/min and 0.53kwh/min. Thus, the 3DT2 and 3DT1 total energy consumption were decreased 45,9% and 42,5%, respectively.
**Conclusion:** CS image acceleration technique has the potential to reduce MRI scanning time and energy required while maintaining high image quality.
**Limitations:** Not applicable
**Funding for this study:** No funding was provided for this study.
**Ethics committee - additional information:** The study received institutional review board approval and written consent was obtained from all participants.

**Author Disclosures:**
Evlampia Psatha: Nothing to disclose
Selmina Kalari: Nothing to disclose
Efstratios Karavasilis: Nothing to disclose
Ioannis Seimenis: Nothing to disclose
Ioannis Loulakas: Nothing to disclose
Dimitrios Apostolou: Nothing to disclose

**A methodology for reducing the environmental impact of the energy consumption of a neuroradiology MRI system using daily electricity emission data**

*A. Roletto*[1], M. Verga[2], G. L. Viganò[2], S. Zanoni[2]; [1]Milan/IT, [2]Brescia/IT
*(rolettoandrea@yahoo.it)*

**Purpose or Learning Objective:** Magnetic resonance imaging (MRI) scanners contribute significantly to the carbon footprint of healthcare sector, and efforts to reduce environmental impact are based on reducing energy consumption. The aim of this work was to develop a methodology to reduce carbon emissions by reorganising an MRI neuroradiology system's diagnostic activities based on daily electricity emission data.
**Methods or Background:** In July 2024, at a large public hospital of Brescia (Italy), the energy profiles of two weeks of diagnostic activity of a neuroradiology MRI system were recorded using HT GSC60 Power Meter/Analyzer (HT-Italia, Italy), calculating the total energy consumption and its distribution over days. Finally, the amount of carbon dioxide equivalent (CO2e) emitted by electricity consumption was calculated using open-source daily emission data (www.electricitymaps.com) and the amount that can be saved by rescheduling diagnostic activities to lower emission day periods.
**Results or Findings:** The total weekly energy consumption was 2305.1 kWh, spread over 5 working days, from 7am to 7pm. The most performed examinations were brain MRI (25 kWh/scan) and lumbar spine MRI (18 kWh/scan). Total emissions were 373.5 kgCO2e, equivalent to emissions from one homes' energy use for six months. Examinations performed between 4pm and 7pm have the highest carbon emission index (213.4-252.3 g$CO_2$eq/kWh). Rescheduling them earlier in the working day or at the weekend would reduce emissions by 18%, without any decrease of energy consumed.
**Conclusion:** This study shows how clinicians and managers can reduce carbon emissions from MRI diagnostic activity, not only by reducing energy consumption through cutting unnecessary examinations or optimising the acquisition technique, but also by rescheduling work activities according to daily electricity emission data, scheduling examinations during low-carbon emission times.
**Limitations:** Short period of data collection limited the strength of the conclusions of this study.
**Funding for this study:** Not applicable
**Ethics committee - additional information:** Not applicable
**Author Disclosures:**
Matteo Verga: Nothing to disclose
Andrea Roletto: Nothing to disclose
Gian Luca Viganò: Nothing to disclose
Simone Zanoni: Nothing to disclose

**08:00-09:00** **Research Stage 3**

### Research Presentation Session: Oncologic Imaging

### RPS 2216

### Structured reporting, radiomics and deep learning

**Moderator**
M. E. Mayerhöfer; Vienna/AT

**Evaluating the Impact of Structured Radiology Reporting on Clinical Practice and Decision-Making: A Survey in a Large Tertiary University Hospital – STAR Study**

*M. Mancino*, G. Avesani, A. Infante, S. Gaudino, B. Merlino, L. Natale, E. Sala; Rome/IT
*(matteomancino@gmail.com)*

**Purpose or Learning Objective:** To evaluate the impact of structured radiology reports (SRRs) on clinical decision-making and patient management, specifically focusing on improvements in diagnostic accuracy, treatment planning, patient outcomes, and data standardization. Additionally, we aim to measure clinicians' satisfaction with the clarity, comprehensiveness, and utility of SRRs in comparison to standard narrative reports.
**Methods or Background:** SRRs were introduced one and a half years ago at Fondazione Policlinico Universitario A.Gemelli, a large tertiary university italian hospital , and have since been implemented in nearly all radiological procedures . This transition was the result of a comprehensive collaboration among radiologists, surgeons, and clinicians to ensure that the structured templates met clinical needs while adhering to guidelines from major radiological societies. An extensive and anonymous survey was conducted among non-radiologist clinicians, surgeons, and residents from various specialties to gather feedback on SRRs' clarity, clinical impact, adaptability, research value, and efficiency compared to traditional reports.
**Results or Findings:** Survey responses indicate increased clinician satisfaction, improved communication, and greater workflow efficiency with SRRs. Preliminary findings suggest better data interpretation, ease of retrieval, and enhanced multidisciplinary discussions, particularly in oncology. SRRs were perceived as more effective than traditional reports in supporting clinical decisions and improving collaboration.
**Conclusion:** SRRs significantly improve clinical practice by delivering clearer, more consistent interpretations that directly enhance patient outcomes and support effective decision-making. The increased clarity and standardization foster better collaboration among clinicians, ultimately benefiting patient care. Continuous feedback from users is essential to refine SRRs and ensure they remain adaptable and impactful. Furthermore, SRRs provide a foundation for consistent data collection, crucial for advancing research and supporting evidence-based practices.
**Limitations:** The single-center survey limits the generalizability of the results. Future studies should include multiple centers to validate these findings.
**Funding for this study:** No specific funding was obtained for this study.
**Ethics committee - additional information:** This study has been notified to the Ethics Committee of Fondazione Policlinico Universitario A. Gemelli IRCCS.
**Author Disclosures:**
Giacomo Avesani: Nothing to disclose
Amato Infante: Nothing to disclose
Simona Gaudino: Nothing to disclose
Luigi Natale: Nothing to disclose
Evis Sala: Nothing to disclose
Matteo Mancino: Nothing to disclose
Biagio Merlino: Nothing to disclose

**Unraveling tumour heterogeneity with radiogenomics: comparing single instance and multiple instances learning AI approaches**

*D. I. Rodríguez Sánchez *, R. Spaans, S. Rostami, O. Maxouri, Z. Bodalal, R. G. H. Beets-Tan; Amsterdam/NL
*(i.rodriguez@nki.nl)*

**Purpose or Learning Objective:** Tumour genetic heterogeneity is a fact in cancer research. While biopsying every lesion is clinically infeasible, imaging-based methods (such as radiogenomics) promise clinicians non-invasive insights into the underlying tumour biology. This study assesses the impact of accounting for tumour heterogeneity by comparing Single Instance Learning (SIL) and Multiple Instance Learning (MIL) AI approaches.
**Methods or Background:** A cohort of 1666 routine contrast-enhanced CT scans, including over 11.000 segmented lesions, was retrospectively collected along with matched next-generation sequencing data. The morphological phenotype was quantified from each lesion by radiomic features, with subsequent feature selection using orthogonal principal feature selection (OPFA) and five-fold cross-validation. SIL and MIL-based machine learning methods were compared to predict the mutational status of TP53, KRAS, and EGFR. Interlesional morphological heterogeneity was measured using spatial distance metrics.
**Results or Findings:** For genes with established low biological variability between lesions (TP53 and KRAS), accounting for tumour heterogeneity did not improve the radiogenomic predictive performance. However, for genes with high discordance (EGFR), MIL-based machine learning methods (AUC range=0.72-0.78) performed significantly better than SIL (AUC=0.56). These findings are also supported by interlesional heterogeneity scores, which did not differ between wild-type and mutated TP53 or KRAS cases. Conversely, EGFR-mutated patients demonstrated significantly higher interlesional morphological heterogeneity than their wild-type counterparts ($p<0.0001$).
**Conclusion:** MIL models may better reflect tumour heterogeneity, particularly in cases with high biological variability. Incorporating MIL into radiogenomic models may enhance their predictive accuracy by accounting for real-world tumour heterogeneity.
**Limitations:** The study's limitations include external validation. A large-scale multicenter radiogenomics dataset is currently being finalised to validate these results.

**Funding for this study:** Funding was provided by the European Union's Horizon 2020 research and innovation program under the Marie Skłodowska-Curie grant agreement number 101034290 (EMERALD International PhD Program for Medical Doctors).
**Ethics committee - additional information:** IRB approval: IRBd19-147
**Author Disclosures:**
Zuhir Bodalal: Nothing to disclose
Robert Spaans: Nothing to disclose
Diana Ivonne Rodríguez Sánchez : Nothing to disclose
Sajjad Rostami: Nothing to disclose
Regina G. H. Beets-Tan: Nothing to disclose
Olga Maxouri: Nothing to disclose

**Enhancing speed and precision of lesion tracking in follow-up lung CT using deep-learning-based registration**
*S. Kuckertz*[1], S. Heldmann[1], F. Peisen[2], J. H. Moltz[3]; [1]Lübeck/DE, [2]Tübingen/DE, [3]Bremen/DE

**Purpose or Learning Objective:** Continuous lesion assessment in cancer patients is integral to radiologists' work. Part of this process is the tedious and time-consuming (re-)localisation and measurement of lesions. Fast and precise image registration facilitates the workflow by automatically matching previous and current observations. In this study we evaluate our deep-learning-based lung registration using a longitudinal dataset including expert lesion segmentations, comparing it to a state-of-the-art conventional non-learning approach.
**Methods or Background:** We follow a 3-level coarse-to-fine deep-learning registration approach. At each level, we input the baseline and follow-up CT scan at a different resolution into a U-Net, resulting in a deformation field that maps corresponding locations from baseline to follow-up. Combining the deformations from all levels allows accurate tracking of anatomical points. Our method is trained on 681 follow-up image pairs and evaluated on a distinct dataset consisting of 90 image pairs with 307 manually segmented lung lesions. We compare our approach to a non-learning GPU-accelerated registration. Each lesion centre in the baseline scan is propagated onto the follow-up, where we check whether it maps inside the given corresponding lesion.
**Results or Findings:** With our learning-based approach, 81.1% of the baseline lesion centres were correctly mapped to the corresponding lesion in the follow-up (conventional approach: 73.6%). The median distance between the propagated and the given lesion centre was 1.9 mm (conventional approach: 3.2 mm) and the average calculation time was 0.92 s (conventional approach: 14.56 s).
**Conclusion:** Our learning-based registration approach enhances both speed and accuracy, enabling precise relocation of all lung lesions in follow-up scans in less than a second. This facilitates radiologists' workflow, also enabling cursor synchronisation and change highlighting in viewers.
**Limitations:** CTs were cropped to the thorax area and resampled to an isotropic resolution of 1.5 mm.
**Funding for this study:** Funding was provided by the Federal Ministry of Education and Research of Germany (BMBF) as part of SPIRABENE (project number 13GW0561B).
**Ethics committee - additional information:** Only retrospective data was used for this work. The outcome had no effect on patient treatment.
**Author Disclosures:**
Sven Kuckertz: Nothing to disclose
Felix Peisen: Nothing to disclose
Stefan Heldmann: Nothing to disclose
Jan Hendrik Moltz: Nothing to disclose

**Radiomic gradient in the peritumoral tissue of liver metastases: A biomarker to drive clinical practice?**
*A. Ammirabile*[1], F. Fiz[2], E. M. Ragaini[1], S. Sirchia[3], S. Viganò[1], M. Francone[1], L. Cavinato[1], E. Lanzarone[3], L. Viganò[1]; [1]Milan/IT, [2]Genova/IT, [3]Bergamo/IT
*(angela.ammirabile@humanitas.it)*

**Purpose or Learning Objective:** To investigate the variation of three textural features (mean HU, entropy, and uniformity) in the peritumoral tissue around colorectal liver metastases (CRLM) as distance from the tumor increases.
**Methods or Background:** This retrospective study included all consecutive patients with histologically proven CRLM who underwent locoregional treatment (resection/ablation) between January 2010 and December 2022. Inclusion criteria were high-quality CT with an adequate portal phase and identifiable hypodense CRLM (>10 mm). Multiple VOIs were generated: 1) manual tumor segmentation (Tumor-VOI); 2) multiple automatic concentric rims at increasing distance from CRLM (1 to 10 millimeters); 3) manual segmentation of a virtual biopsy of non-tumoral parenchyma (Liver-VOI). Radiomic features were extracted by the LifeX software. The percentage variation of index values across different VOIs was calculated, using Liver-VOI as reference. Subgroup analyses were based on tumor size (10-30 vs. >30 mm) and chemotherapy administration (no chemotherapy vs. responders).
**Results or Findings:** 63 CRLM in 51 patients (median age 67 years, 14 females) were analyzed. Median peritumoral HU values were similar to Liver-VOI, except within the 1-mm VOI around CRLM (p=0.002). Entropy progressively decreased (from 3.11 of CRLM to 2.54 of Liver-VOI, p<0.001) while uniformity increased (from 0.135 of CRLM to 0.199 of Liver-VOI, p<0.001). At 10 mm from CRLM, entropy was similar to Liver-VOI in 62% of cases and uniformity in 46%. Smaller CRLM and responders to chemotherapy showed higher and earlier normalization of entropy and uniformity values.
**Conclusion:** The radiomic analysis of peritumoral tissue in CRLM demonstrated a peculiar gradient of decreasing entropy and increasing uniformity despite a normal radiological appearance, representing a potential biomarker for personalized clinical decision-making.
**Limitations:** Retrospective analysis; small cohort; heterogeneous CT data; missing correlation with pathologic/surgical data.
**Funding for this study:** AIRC grant #2019–23822
**Ethics committee - additional information:** Protocol 988/22
**Author Disclosures:**
Sara Sirchia: Nothing to disclose
Angela Ammirabile: Nothing to disclose
Ettore Lanzarone: Nothing to disclose
Francesco Fiz: Nothing to disclose
Samuele Viganò: Nothing to disclose
Elisa Maria Ragaini: Nothing to disclose
Marco Francone: Nothing to disclose
Lara Cavinato: Nothing to disclose
Luca Viganò: Nothing to disclose

**Integrating MRI and PET/CT Radiomics for Enhanced Survival Prediction in Esophageal Cancer**
*C. Noirot*, D. Abler, L. Haefliger, S. Mantziari, M. Schäfer, N. Vietti Violi, A. Depeursinge, C. Dromain, M. Jreige; Lausanne/CH
*(camille.noirot@chuv.ch)*

**Purpose or Learning Objective:** Prognosis evaluation in esophageal cancer remains challenging. Accurate survival prediction is crucial for treatment planning and follow-up strategies. Although MRI and 18F-FDG PET/CT provide valuable information, they have limitations in accurately predicting patient outcomes. This study aimed to develop radiomics models based on MRI and PET/CT to predict overall survival in esophageal cancer patients using baseline and follow-up imaging.
**Methods or Background:** Sixty patients (M/F: 50/10, mean age 66±9 years) with newly diagnosed esophageal cancer were prospectively included (2017-2022). Patients underwent staging with 18F-FDG PET/CT and MRI, with follow-up MRI after neoadjuvant treatment. Tumors were manually segmented using Mint™ Software, and radiomics features were extracted via QuantImage v2 platform. The dataset, including 645 features from MRI and PET/CT, was split into training (80%) and test (20%) sets. Various survival prediction algorithms were compared. Model performance was assessed with the concordance index (C-index) using bootstrapping for confidence interval (CI) estimation.
**Results or Findings:** Radiomics features were analyzed at baseline from both PET/CT and MRI for 52 patients, and at follow-up MRI for 49 patients. Mean survival was of 37 months (range: 1.8 to 78.1). The MRI model (14 features) achieved a C-index of 0.733 (95% CI: 0.718–0.756), and the PET/CT model (5 features) achieved 0.724 (95% CI: 0.707–0.746) for predicting OS. A combined model with 19 features improved the C-index to 0.868 (95% CI: 0.853–0.881), while a follow-up MRI model (16 features) reached 0.807 (95% CI: 0.790–0.827).
**Conclusion:** The radiomics model based on MRI and PET/CT demonstrated robust performance in predicting survival for esophageal cancer patients. Integrating multi-modal baseline and follow-up imaging radiomics features into survival models could enhance prognostic accuracy, improving personalized management strategies in esophageal cancer.
**Limitations:** The limitations of the study are the number of patients.
**Funding for this study:** No funding was received.
**Ethics committee - additional information:** The study was approved by "Commission Cantonale d'Ethique de la Recherche sur l'être humain" à Lausanne (CER-VD 2017-00388)
**Author Disclosures:**
Markus Schäfer: Nothing to disclose
Camille Noirot: Nothing to disclose
Adrien Depeursinge: Nothing to disclose
Laura Haefliger: Nothing to disclose
Daniel Abler: Nothing to disclose
Styliani Mantziari: Nothing to disclose
Naïk Vietti Violi: Nothing to disclose
Mario Jreige: Nothing to disclose
Clarisse Dromain: Nothing to disclose

**AI-Assisted Annotation for Improving Federated Learning in Automated RCC Image Segmentation**

K. S. Younis[1], *J. Garrett*[2], A. Elhanashi[3], A. Gentili[4], S. Faghani[5], S. Kuanar[5], Y. Singh[5], Y. Huo[6], G-M. Conte[5], J. Yacoub[7], O. Unal[2]; [1]Cleveland, OH/US, [2]Madison, WI/US, [3]Pisa/IT, [4]San DIego, CA/US, [5]Rochester, MN/US, [6]Nashville, TN/US, [7]Washington, DC/US
*(jgarrett@uwhealth.org)*

**Purpose or Learning Objective:** To evaluate the impact of AI-assisted annotation tools on improving consistency in dataset labeling for federated learning, focusing on image segmentation tasks for renal cell carcinoma (RCC) in CT and MR images.
**Methods or Background:** One of the major challenges in distributed learning is variability in data labels across sites, especially in image segmentation tasks where mask generation methods can differ significantly. This study leverages a publicly available dataset from The Cancer Imaging Archive (TCGA-KIRC) for RCC, which contains rich imaging and clinical data. AI-assisted annotation tools standardize data labeling before training models such as Unet and Swin UNETr. The primary goal is to assess if these tools enhance model performance or reduce the number of cases needed for effective training in federated learning environments. Radiologists from various institutions across multiple states in the US manually annotated the images and federated learning was conducted using NVIDIA nvFLARE.
**Results or Findings:** Preliminary results suggest that AI-assisted annotation improves model consistency, with segmentation accuracy increasing by approximately 12% when compared to non-standardized data labels. The model efficiency was also reflected in reduced data redundancy and higher annotation agreement between annotators. This enhancement allowed for more precise radiomic analysis across datasets. Additionally, the number of samples required for training decreased by 20%, indicating the efficiency of AI-assisted annotation in generating reliable training datasets.
**Conclusion:** AI-assisted annotation holds promise for improving performance and efficiency in federated learning environments, particularly for automated image segmentation tasks like RCC detection. The enhanced consistency in label generation helps to reduce the variability introduced by multiple sites, thereby improving model generalizability.
**Limitations:** The study does not include external validation across diverse imaging platforms, and annotation tools were not evaluated for real-time performance during federated learning.
**Funding for this study:** Nvidia Education grant
**Ethics committee - additional information:** N/A
**Author Disclosures:**
Yashbir Singh: Nothing to disclose
Abdussalam Elhanashi: Nothing to disclose
Orhan Unal: Nothing to disclose
Amilcare Gentili: Nothing to disclose
Shahriar Faghani: Nothing to disclose
Joseph Yacoub: Consultant: Microsoft Nuance
Khaled Salem Younis: Nothing to disclose
Yuankai Huo: Nothing to disclose
Shiba Kuanar: Nothing to disclose
Gian-Marco Conte: Nothing to disclose
John Garrett: Grant Recipient: Research Support from the NIH, GE Healthcare, and the American Cancer Society Advisory Board: RadUnity Corp. Shareholder: NVIDIA, Inc. and RadUnity Corp.

**08:00-09:00 Research Stage 4**

### Research Presentation Session: Imaging Informatics and Artificial Intelligence

### RPS 2205

### Imaging informatics, quality and new techniques

**Moderator**
L. Lofino; Milan/IT
*(Ludovica.Lofino@humanitas.it)*

**Universal Medical Imaging Encoding Datasets - A new standard for creating large-scale diagnostic imaging datasets**

*B. O. Klaudel*[1], A. Obuchowski[2], A. Komor[1], P. Frąckowski[1], K. Rogala[1], K. Knitter[1]; [1]Gdańsk/PL, [2]Banino/PL
*(klaudel.b@gmail.com)*

**Purpose or Learning Objective:** We present a new standard for creating and unifying large-scale diagnostic imaging datasets. It aims to address the challenges in medical AI, particularly the lack of standardized, comprehensive data for training foundation models in medical imaging. By combining multiple open-source datasets, unifying them to a common ontology and providing standardized preprocessing pipelines, we seek to accelerate the development of more generalized and robust medical AI models.
**Methods or Background:** We combined over 20 open-source datasets, resulting in more than one million annotated medical images, including CT, MRI, and X-ray modalities. We created reusable preprocessing pipelines to transform diverse source datasets into a unified format. A key innovation is the adoption of the RadLex ontology, developed by the Radiological Society of North America, to standardize labels and masks across all included datasets. It addresses issues of inconsistent labeling, regional variations, and differing granularity in existing medical imaging datasets.
**Results or Findings:** The project has created the largest publicly available dataset of annotated radiological imaging to date, with over one million images, 40+ labels, and 15 annotation masks. The datasets are accompanied by ready-to-use preprocessing pipelines that can be easily adapted to incorporate new data sources. The unified ontology based on RadLex enables consistent labeling, facilitating more effective model training and cross-dataset compatibility.
**Conclusion:** UMIE datasets represent a significant advancement in medical imaging AI, providing a standardized foundation for developing more generalized models. By addressing the challenges of data scarcity, inconsistent formatting, and labeling discrepancies, it paves the way for more robust and widely applicable AI solutions The open-source nature of the project encourages collaboration and further expansion of the datasets.
**Limitations:** UMIE datasets inherits potential biases and incomplete labelling coverage from source datasets, and limitations of the RadLex ontology.
**Funding for this study:** No funding was required.
**Ethics committee - additional information:** All data used in this study come from existing opensource datasets.
**Author Disclosures:**
Andrzej Komor: Nothing to disclose
Kacper Rogala: Nothing to disclose
Barbara Olga Klaudel: Nothing to disclose
Aleksander Obuchowski: Nothing to disclose
Kacper Knitter: Nothing to disclose
Piotr Frąckowski: Nothing to disclose

**Analysis of key principles for improving the efficiency of medical data annotation processes for machine learning**

*P. Pilius*[1], N. Smirnov[2]; [1]Almaty/KZ, [2]Haar/DE
*(polishka1903@gmail.com)*

**Purpose or Learning Objective:** The aim of this work is to analyze the fundamental principles of project management and evaluate potential opportunities for its implementation for improving efficiency in the preparation of medical data for machine learning: for data collection, working with annotators, and results validation.
**Methods or Background:** We analyzed personal experiences from 9 projects conducted during 2022-2024 years, aimed at creating annotated medical datasets with various levels of complexity and across different fields. The clients were private companies developing artificial intelligence technologies, and the annotators included certified radiologists, residents, and radiographers.

Sunday

To enhance efficiency, we studied and applied principles from an advanced training course on the foundations of project management.
**Results or Findings:** The duration of the projects ranged from 3 weeks to 3 months. The number of annotated datasets included: 75 MRI, 3720 CT, 55,500 X-rays, 6000 angiograms, 1700 mammograms, and 3200 radiological text report annotations. All projects were completed on time and met quality requirements. Based on practical experience and the implementation of fundamental project management concepts, we've formulated recommendations and standards for conducting similar projects.
**Conclusion:** The organization of medical data annotation processes for machine learning presents numerous specific challenges. To optimize the workflow and achieve desired outcomes, it is essential to have not only sufficient experience in handling medical images but also a solid understanding of fundamental project management principles.
**Limitations:** The work is based on personal experience and observations and is primarily of a recommendatory nature. It is not possible to objectively compare the outcomes of projects where basic management principles were and were not applied, as the projects were not repeated under identical conditions. However, the variability and uniqueness of each project allowed for a comprehensive analysis, leading to the formulation of universal standards.
**Funding for this study:** No funding
**Ethics committee - additional information:** Kazakh National Scientific and Research Center of Oncology and Radiology
**Author Disclosures:**
Polina Pilius: Nothing to disclose
Nikolai Smirnov: Nothing to disclose

**Data Interoperability in a Clinical Pathway From Free-Text Reports**
*K. Nairz*, N. Cihoric, F. Dennstädt, M. Schmerder, H. Bonel,
H. Von Tengg-Kobligk; Bern/CH
*(knud.nairz@insel.ch)*

**Purpose or Learning Objective:** Structured reporting (SR) in radiology has been shown to be the most favorable scheme to provide imaging information to referrers. Implementation of SR is associated with additional effort for the radiologists, but there are recent advances with Large Language Models (LLMs) that are prompted to convert free text into a structured format. We aimed at killing two birds with a stone by leveraging LLMs to generate structured data that enhances interoperability as well. As a proof of concept we we selected breast cancer patient pathways for documentation. We structured the free-text information from various sources, including health interviews, mammography reports, biopsy results, pathology findings, and tumor board discussions, to ensure that the data could be effectively transmitted and support therapeutic decision-making.
**Methods or Background:** Our approach is based on the use of Common Data Elements (CDEs), which are minimal information units or precisely defined questions associated with a set of standardized answers, each having explicitly defined values. Building on in-depth analyses of reports and guidelines such as BI-RADS, we defined corresponding sets of Common Data Elements and created templates to establish a structured format. To ensure data protection we utilized a locally installed LLM (Llama 3), which was prompted to answer CDE-based questions. This process enabled the mapping of free-text content to a structured format, which was ultimately stored in FHIR compatible JSON format.
**Results or Findings:** By defining key CDE values, or specific combinations of these values, clinicians can identify critical insights that may suggest a particular therapeutic course or provide predictive indicators for patient outcomes.
**Conclusion:** Prompting LLMs to answer CDE-based structures proves to be a viable approach to promote data interoperability in complex medical settings.
**Limitations:** The study focuses specifically on breast cancer pathways.
**Funding for this study:** Innosuisse 59228.1
**Ethics committee - additional information:** Kantonale Ethikkommission Bern
**Author Disclosures:**
Harald Bonel: Nothing to disclose
Max Schmerder: Nothing to disclose
Nikola Cihoric: Nothing to disclose
Knud Nairz: Nothing to disclose
Fabio Dennstädt: Nothing to disclose
Hendrik Von Tengg-Kobligk: Nothing to disclose

**Deep learning based automated field of view positioning for prostate magnetic resonance imaging**
*A. S. Quinsten*[1], A. Wetter[2], M. Raczkowski[3], L. Trembecki[3], R. Buchkremer[4], D. Matusiewicz[1], K. Nassenstein[1], M. Forsting[1], A. Demircioglu[1]; [1]Essen/DE, [2]Hamburg/DE, [3]Wrocław/PL, [4]Düsseldorf/DE

**Purpose or Learning Objective:** Prostate magnetic resonance imaging (MRI) is typically conducted according to manual prescriptions by radiographers. This approach is time-consuming, error-prone, inconsistent due to rater variability, and has low reproducibility. The aim of the study was to develop a deep learning-based framework for the automatic planning of the field of view (FoV) in the oblique coronal and axial planes in prostate MRI according to Prostate Imaging Reporting and Data System (PI-RADS) guidelines.
**Methods or Background:** The retrospective multicentre study included 2109 patients from diagnostic (Sites I and III) and radiotherapy (Site II) centres. The variability within and between raters was evaluated by three assessors. Three distinct deep neural networks were developed with the objective of predicting the oblique coronal and axial FoV. The optimal network was evaluated on three external cohorts using a non-inferiority test, and its clinical utility was assessed.
**Results or Findings:** The optimal model demonstrated non-inferior performance, with slice position differences ranging from 0.21 ± 0.99 and 0.37 ± 0.48. At Sites I and III, the predictions were predominantly non-inferior, with FoV overlaps of 86.6 ± 5.8% and 88.7 ± 6.0% and angle differences of 4.66 ± 4.89° (Site I) as well as 3.46 ± 2.80° (Site III). In contrast, the predictions for Site II demonstrated inferior overlap (67.0 ± 9.7% and 63.6 ± 8.8%) and higher angle differences (9.18 ± 9.49°). Consequently, the clinical utility was excellent for Sites I and III (97.9–100%) but lower for Site II (85.3–89.0%).
**Conclusion:** The utilisation of a deep learning-based framework for the automated positioning of the FoV in oblique coronal and axial planes for prostate MRI is a viable approach, exhibiting high clinical utility.
**Limitations:** The present study did not include images acquired with the endorectal coil.
**Funding for this study:** No funding was provided for this study
**Ethics committee - additional information:** The ethics committee notification can be found under the number 22-10740-BO.
**Author Disclosures:**
Michael Forsting: Nothing to disclose
Kai Nassenstein: Nothing to disclose
Anton Sheahan Quinsten: Nothing to disclose
Rüdiger Buchkremer: Nothing to disclose
David Matusiewicz: Nothing to disclose
Axel Wetter: Nothing to disclose
Lukasz Trembecki: Nothing to disclose
Aydin Demircioglu: Nothing to disclose
Maciej Raczkowski: Nothing to disclose

**RADAR - real-time automated detection and analysis of radiopaque devices using CT topograms**
*C. S. Schmidt*, M. Walter, J. Haubold, F. Nensa, R. Hosch; Essen/DE

**Purpose or Learning Objective:** The aim of this study was to develop a deep learning (DL) model for the automatic detection and localisation of medical devices known to cause metal artefacts in CT images, utilising their corresponding topograms.
**Methods or Background:** A dataset of 943 CT topograms with radiopaque medical devices was manually annotated via box labelling by a radiology resident with three years of experience in CT imaging. The following classes were defined: cochlear implant, cardiac conduction device (pacemaker, defibrillator, stimulator), implanted port, prosthetic heart valve, (embolisation) coil, osteosynthesis (nail-, plate-, screw-, and wire-fixation, spinal instrumentation hardware), sternal wires, external fixation hardware, hip prosthesis, shoulder prosthesis, knee prosthesis, denture (prosthesis, implant). An 80/10/10% split for training, validation and testing was performed and the YOLO11X model was trained for 100 epochs. The model was evaluated using mAP50 scores, precision (P) and recall (R).
**Results or Findings:** The model achieved an average mAP50 score of 0.83, Precision of 0.86 and Recall of 0.79 over all classes and the following (mAP50/P/R) scores for the respective classes: cochlear implant (0.92/0.93/0.85), cardiac conduction device (0.90/0.82/0.93), implanted port (0.91/0.97/0.79), prosthetic heart valve (0.86/0.82/0.78), coil (0.99/0.98/1), osteosynthesis (0.63/1/0.55), sternal wires (0.51/0.74/0.57), external fixation hardware (0.52/0.51/0.33), hip prosthesis (0.99/0.89/1), shoulder prosthesis (0.88/0.88/0.86), knee prosthesis (0.99/0.95/1), denture (0.88/0.81/0.76).
**Conclusion:** The presented model demonstrates an accurate detection of most radiopaque medical devices in CT scout images. It could thus be utilised as an efficient orchestration tool for selecting a cohort of high quality imaging studies without interfering artefacts.
**Limitations:** The limitations of the study are its small sample size and that scout images were annotated by a single observer. Additionally, certain medical devices can be challenging to identify and localise on topograms, which could cause relevant features to go undetected.
**Funding for this study:** No funding was received for this study.
**Ethics committee - additional information:** Informed consent was waived by the ethics committee due to the retrospective setting.
**Author Disclosures:**
Johannes Haubold: Nothing to disclose
Cynthia Sabrina Schmidt: Nothing to disclose
Marie Walter: Nothing to disclose
René Hosch: Nothing to disclose
Felix Nensa: Nothing to disclose

**Evaluating the Impact of Quantum Technology on Radiomics: A Comparative Study of Classical and Quantum Random Forest Models**

*F. Mariotti*, A. Agostini, A. Borgheresi, L. Pierpaoli, F. Ricciardiello, A. Zannotti, D. Nicolini, A. B. Galosi, A. Giovagnoni; Ancona/IT
*(Dottfrancescomariotti@gmail.com)*

**Purpose or Learning Objective:** This study aims to evaluate the impact of quantum technology on the performance of radiomics random forest (RF) models for medical imaging. We simulated a semi-quantum approach, involving quantum embedding followed by classical RF, and a fully quantum approach using a quantum random forest (QRF) model.
**Methods or Background:** We used three radiomic datasets: 1. Perineural infiltration of peripancreatic fat in pancreatic adenocarcinoma on CT, 2. Characterization of renal nodules in CT, 3. Prediction of LI-RADS category on abbreviated MRI protocols. For the quantum approaches, we compared the original random forest (RF) models with simulated quantum-embedded RF and quantum random forest (QRF) algorithms, implemented in Python using an 8-qubit configuration. The comparison involved analyzing the accuracy and the receiver operating characteristic (ROC) curves using statistical significance set at p-values < 0,05
**Results or Findings:** The classical RF achieved the highest accuracy for the pancreas (0.9167) and kidney (0.8571) datasets. For the liver dataset, both the quantum embedding RF and QRF outperformed the classical approach (0.8462 vs. 0.7692), with the ROC curves showing statistically significant improvement ($p < 0.01$). In the pancreas dataset, quantum methods showed slightly lower accuracy (0.8333), and for the kidney, they also performed worse (0.7857). This indicates that the benefits of quantum approaches may be data-dependent, providing advantages in some cases but not yielding consistent improvements across all datasets.
**Conclusion:** Quantum machine learning is a feasible approach for radiomic datasets, showing variable results and the potential to outperform classical methods. However, the variability in performance suggests that fine-tuning of quantum algorithms may be necessary depending on the specific characteristics of each dataset.
**Limitations:** Small datasets used and simulation of quantum processes with a 8-qubit setup. Further research should involve larger datasets and physical quantum devices.
**Funding for this study:** This study did not receive any specific funding from public, commercial, or not-for-profit sectors. The research was conducted without external financial support.
**Ethics committee - additional information:** Not Applicable
**Author Disclosures:**
Daniele Nicolini: Nothing to disclose
Alice Zannotti: Nothing to disclose
Luca Pierpaoli: Nothing to disclose
Andrea Agostini: Nothing to disclose
Francesco Ricciardiello: Nothing to disclose
Francesco Mariotti: Nothing to disclose
Andrea Benedetto Galosi: Nothing to disclose
Alessandra Borgheresi: Nothing to disclose
Andrea Giovagnoni: Nothing to disclose

**A new framework for 3D data representation in Extended Reality (XR) on iPhone, iPad and Apple Vision Pro**

*A. M. C. Boehner*, A. Jacob, A. Isaak, C. C. Pieper, J. A. Luetkens, D. Kütting; Bonn/DE
*(boehner.amc@gmail.com)*

**Purpose or Learning Objective:** 3D data is rarely spatially displayed in routine. However, patient-clinician and clinician-clinician interaction may benefit from such representation in Extended Reality (XR). Additionally, radiologists may aid surgeons during surgery via audiovisual communication to demonstrate 3D data if needed.
**Methods or Background:** We developed and tested a workflow integrating different software platforms (e.g.'Medical Imaging XR', 'Fiji') to display DICOM images on iPhone and iPad (n=35) and Apple Vision Pro (AVP, n=10). The system enables fused XR visualization of CT, MRI, PET. Handheld devices were utilized to aid sonographic correlations of hepatic lesions (n=11); by surgeons during surgery preparations (n=10); and for patient information (n=14). Integrated systems were tested in a mock audiovisual call from the operating room via the AVP to the other devices located on and off campus.
**Results or Findings:** Our framework allowed for fast integration of 3D datasets across devices with low computational burden. XR during sonographic correlation of hepatic lesions significantly reduced the time needed to identify lesions from 4:50min to 2:45min ($P<0.05$). Patient reported full acceptance of XR usage. AVP allowed real-time image-data and view sharing between the radiologist and surgeon.
**Conclusion:** Integration of XR across smartphones, tablets and AVP enhanced medical imaging communication between all parties, reducing time to locate lesions and improving patient-physician interactions. AVP further facilitates sterile audiovisual communication between surgeons and radiologists during procedures, allowing for remote and swift consultation without leaving the sterile field.
**Limitations:** Our method was tested exclusively on Apple products, limiting its generalizability to other platforms.
**Funding for this study:** This project was part of the ISMC, funded by the Ministry of Economic Affairs, Innovation, Digitalization and Energy of the state of North Rhine-Westphalia
**Ethics committee - additional information:** Ethics University Hospital Bonn, Germany (2024-228-BO)
**Author Disclosures:**
Julian Alexander Luetkens: Nothing to disclose
Alexander Isaak: Nothing to disclose
Alexander Marc Christian Boehner: Nothing to disclose
Alice Jacob: Nothing to disclose
Daniel Kütting: Nothing to disclose
Claus Christian Pieper: Nothing to disclose

**08:00-09:00** **Room G1**

### Research Presentation Session: Radiographers

### RPS 2214

### Advancing radiography through education and research: innovations, challenges, and future directions

**Moderators**
B. Horehledova; Heerlen/NL
*(barbora.horehledova@gmail.com)*
J. Santos; Coimbra/PT
*(joanasantos@estesc.ipc.pt)*

**The College of Radiographers' Education and Career Framework (fourth edition): Exploring the guideline implementation gap across England using Normalisation Process Theory**

*H. L. Spencer*[1], K. Williamson[2], A. Robertson[2], M. N. K. Anudjo[1], C. Burton[3]; [1]Bournemouth/UK, [2]London/UK, [3]Norwich/UK
*(HSpencer@aecc.ac.uk)*

**Purpose or Learning Objective:** In 2022, the College of Radiographers (CoR) published the fourth edition of their Education and Career Framework (ECF). This essential document provides a professional blueprint for the radiography career trajectory with the overarching aim of improving patient outcomes. However, publication does not guarantee implementation; there often exists a dissonance between policy intent and policy in-action. Therefore, if we are to access the full benefits of the ECF, its implementation requires careful consideration.
**Methods or Background:** To advance our understanding of the translational gap between policy and practice, this observational mixed-methods study employed Normalisation Process Theory (NPT) as a theoretical frame. Focusing on the diagnostic radiography profession in England, a national consultation survey was deployed, alongside four consultation workshops. The data collection methods were underpinned by NPT. The framework approach was adapted for the qualitative data analysis. The quantitative survey data, meanwhile, was analysed using descriptive and inferential statistics.
**Results or Findings:** The data collection took place between April-June 2023. In total, 142 survey responses were returned. Each workshop was comprised of 7-11 participants. The findings were deductively interpreted through the lens of NPT, from which five core themes emerged: making sense of complexity (coherence); bringing people together (cognitive participation); being strategic (collective action); evaluating complexity (reflexive monitoring); implementation in the 'real world' (barriers and enablers).
**Conclusion:** By furthering our understanding of the guideline implementation gap, it was then possible to propose recommendations to enhance the ECF's adoption. The recommendations were study-derived, linked to responsible stakeholders, and grouped into four strategic priorities, aligned with the NPT domains. Through these evidence-based recommendations, it is hoped the ECF can be translated more fully from page to the 'real world' for the benefit of the profession and its service users.
**Limitations:** Nonapplicable.
**Funding for this study:** This study was undertaken as part of a Clinical Education Improvement Fellowship secondment, supported by NHS England (South East) Workforce, Training, and Education, Canterbury Christ Church

Sunday

University, and the Florence Nightingale Foundation. However, no direct funding was received for this study.
**Ethics committee - additional information:** Canterbury Christ Church University Faculty of Medicine, Health, and Social Care Ethics Panel (Reference: ETH2223-0262).
**Author Disclosures:**
Christopher Burton: Nothing to disclose
Messiah Narh Kwame Anudjo: Nothing to disclose
Holly Louise Spencer: Nothing to disclose
Kathryn Williamson: Employee: Society and College of Radiographers
Amy Robertson: Employee: Society and College of Radiographers

**Newly qualified radiographers' perception of the induction programme in a radiology department: a survey study**
*L. Bombelli*, G. R. Bonfitto, A. Roletto, E. Scaramelli, S. V. Fasulo, D. Catania; Milan/IT
*(bombelli.luca@virgilio.it)*

**Purpose or Learning Objective:** The role of radiographers is rapidly evolving, putting them in a key role in a contest of increasing complexities in patient care. With the rising demand for specialized skills, it is crucial to implement structured induction programs for newly qualified radiographers (NQR), also to avoid poor workplace performance and even the dismissal of workers. This study aims to explore the perceptions of NQR who have already experienced an induction programme.
**Methods or Background:** A survey was distributed to NQR in a large university hospital in Italy. The questionnaire gathered demographic data and by using a 5-point Likert scale assessed 29 sentences concerning issues in their work, including patient management, decision-making, work organization, self-confidence development and relationships with other team members.
**Results or Findings:** Twenty-two NQR participated in the survey. Among these, 32% (n=7) graduated within the last year and 46% (n=10) reported that they had their first experience in a healthcare setting. Regarding Likert scale evaluation, only 10 out of 29 sentences received scores of 4 or higher, indicating “Agreement”. Participants with prior work experiences reported greater self-confidence in their skills once the induction program has been completed. Conversely, radiographers with no work experience indicated feeling more supported by management.
**Conclusion:** In conclusion, NQR involved in this study felt adequately prepared for clinical practice after completing the induction program, despite some differences between radiographers with different levels of experiences. A successful induction program for NQR is essential to foster a proactive mindset, promote appropriate work methods, enhance collaboration among team members, reduce radiographers’ stress, turnover and ensure a high quality of patient care.
**Limitations:** Quantitative study design and limited sample size may have caused limitations.
**Funding for this study:** No funding for the study.
**Ethics committee - additional information:** No ethic committe
**Author Disclosures:**
Luca Bombelli: Nothing to disclose
Simone Vito Fasulo: Nothing to disclose
Diego Catania: Nothing to disclose
Andrea Roletto: Nothing to disclose
Elena Scaramelli: Nothing to disclose
Giuseppe Roberto Bonfitto: Nothing to disclose

**Patient, Public and Practitioner Partnership within Imaging and Radiotherapy: An exploration of the implementation and use of the College of Radiographers Guiding Principles**
*R. M. Strudwick*[1], A. Ramlaul[2], P. Shuttleworth[3], C. Fiyebor[1]; [1]Ipswich/UK, [2]High Wycombe/UK, [3]Leeds/UK
*(r.strudwick@uos.ac.uk)*

**Purpose or Learning Objective:** In 2014 the National Health Service (NHS) released the Five Year Forward plan1, envisioning a shift in power from health professionals to patients and the public. In response the Society and College of Radiographers (SCoR) produced the “Patient, Public and Practitioner Partnership within Imaging and Radiotherapy: Guiding Principles” (P4) document which was implemented within four domains of radiography practice; service delivery, service development, education and research2. This project explored how these guidelines were implemented; and whether improvement to the quality and scope were needed, leading to making recommendations for updating the document.
**Methods or Background:** A qualitative methodological framework was adopted with two phases. Phase 1 – a survey exploring use of the P4 document’s guiding principles. There was no maximum number of participants to ensure inclusivity. Phase 2 - six focus groups from the four domains3.
**Results or Findings:** 626 participants completed the phase 1 survey. 18.85% (n=118) of participants were aware of the document and used it as a reference tool for practice, teaching, and research. 81.15% (n=508) of participants stated they were unaware of the document. Themes from phase 2; importance of service user involvement in service delivery and evaluation, resources to ensure service user involvement, suggestions to update the P4 document and use of the P4 document in radiographer education. Participants acknowledged guidance in the document was best practice. They reported more awareness of patients’ needs and the effect this has on radiographers in supporting their needs.
**Conclusion:** Participants recommended the document be given greater visibility. The voices of patients and the public must be heard within radiography practice.
**Limitations:** Small sample size
**Funding for this study:** Feedback from this study can be used for the future development of the P4 document.
**Ethics committee - additional information:** University of Suffolk Ethics committee approval
**Author Disclosures:**
Pamela Shuttleworth: Nothing to disclose
Aarthi Ramlaul: Nothing to disclose
Ruth Mary Strudwick: Grant Recipient: CoRIPS funded project
Chioma Fiyebor: Nothing to disclose

**Patients’ perception of Radiographers’ communication skills during planar X-ray imaging: a single centre study**
*F. Zarb*, P. Bezzina, D. Ciantar; Msida/MT
*(francis.zarb@um.edu.mt)*

**Purpose or Learning Objective:** To explore patients’ perception of radiographers’ communication skills during planar x-ray imaging examinations at a general hospital in Malta
**Methods or Background:** A random sample of patients attending for planar x-ray examinations at a medical imaging department at a general hospital in Malta filled out a self-designed questionnaire consisting of demographic data and scored a series of statements on a Likert scale of 1-4, with 1 being ‘Very unsatisfied’, 2 being ‘Unsatisfied’, 3 being ‘Satisfied’ and 4 being ‘Very satisfied’. Data was collected and analysed to identify trends and understand patients’ perception of radiographers’ communication skills. The Friedman test and Kruskal Wallis test were used to analyse the gathered data.
**Results or Findings:** Mean scores were either 3-Satisfied or 4-Very Satisfied. There were no significant ($p>0.05$) discrepancies when comparing the different demographics. Females provided significantly ($p<0.05$) higher mean rating scores than males. Patients’ scores varied the most in the statement ‘Radiographers informed me of what they had to do next’.
**Conclusion:** This is the first study of its kind performed locally evaluating patients’ perceptions of Radiographers communication skills. The mechanism in place locally for training radiographers in patient communication appears to be effective, as patients are given instructions clearly and without undue discomfort. To make it simpler for patients once they have completed the x-ray and put their minds at ease during their entire stay at the hospital, a clearer pathway should be implemented. Having radiographers with good communication skills improves the patients’ experience when attending for imaging services.
**Limitations:** Limited sample size, honesty of participants filling the questionnaire and a number of radiographers being foreigners making communication a challenge.
**Funding for this study:** No funding was received for this study.
**Ethics committee - additional information:** Ethical permission for this study was sought and obtained from the Faculty of Health Science Research Ethics Committee (FREC), University of Malta.
**Author Disclosures:**
Francis Zarb: Nothing to disclose
Paul Bezzina: Nothing to disclose
Dean Ciantar: Nothing to disclose

**A post-graduation expectation analysis of Italian Radiographers. The OPEN project**
*A. Masperi*; Abbiategrasso/IT
*(andrea.masperi1992@gmail.com)*

**Purpose or Learning Objective:** The aim of this study was to explore student satisfaction with the OPEN project via a survey and to identify factors influencing radiographers' post-graduation decisions.
**Methods or Background:** Radiography undergraduates face changing career paths, influenced by technology, aspirations, financial gain and post-graduate education choices. In January 2024 the Radiographer bachelor’s degree course at the University of Milan launched the OPEN project aimed at creating a new postgraduate orientation programme. Following AMEE guidelines, a semi-structured survey was sent to 17 undergraduate students in the OPEN project to identify factors influencing their post-graduation decisions. University of Milan protocols ensured consent, anonymity, and confidentiality. The survey, in three parts with Likert scale questions, showed good internal consistency via Cronbach's alpha.
**Results or Findings:** Out of 17 participants, 16 consented to participate (94%), with all attending more than 50% of meetings. The internal consistency of the survey was excellent (αC = 0.83551) and results were expressed in terms of median and interquartile range. Students prioritise careers in research-oriented hospitals with opportunities for research and academic advancement. They seek facilities that offer permanent contracts, incentivise clinical activities for salary growth and a balanced working environment.
**Conclusion:** The Radiography degree programme at the University of Milan has demonstrated the effectiveness of postgraduate orientation programmes that bridge academic and career gaps.
**Limitations:** It should be noted that the results of this new project are based on a limited cohort of subjects from a single university. Furthermore, gender preferences were not explored, which could have provided valuable insights to the survey by broadening the field of interview.
**Funding for this study:** None
**Ethics committee - additional information:** None
**Author Disclosures:**
Andrea Masperi: Nothing to disclose

**Radiation Awareness and Occupational Concerns Among Radiographers and Students**

K. Brage[1], J. Jensen[1], O. Brage[1], *M. W. Kusk*[2], P. L. Hansen[1], M. Roland Pedersen[3], H. Precht[4]; [1]Odense/DK, [2]Esbjerg/DK, [3]Vejle/DK, [4]Kolding/DK
*(martin.weber.kusk@rsyd.dk)*

**Purpose or Learning Objective:** To assess the knowledge, perceptions, and concerns of professionals working with ionizing radiation (IR) regarding their occupational exposure and its implications on their health, fertility, and offspring.
**Methods or Background:** This cross-sectional survey included European radiographers and students and was distributed via social media and professional bodies (EFRS and Danish Society of Radiographers) from March to July 2024. The questionnaire covered demographics, knowledge of IR, and concerns, using a five-point Likert scale.
**Results or Findings:** A total of 629 participants from 32 countries were included: 414 women (mean age 34.34 ± 12.18), 208 men (mean age 38.27 ± 11.81), and 7 non-binary or undisclosed individuals. Of these 28.30% were students. Mean exposure time was 10.69 years ± 10.68. Overall, 29.77% of respondents agreed that their radiation exposure could negatively impact their health, 23.75% expressed concerns about fertility, and 18.59% about their children's health. No significant differences were observed between sex in these responses. Of the radiographers 0.44% of did not fully understand the occupational risks of IR, 5.77% were dissatisfied with their radiation protection education, and 4.66% felt unqualified to inform patients. Additionally, 1.99% were unsure about self-protection, and 4.00% felt they lacked the necessary protection means. Regarding the International Basic Safety Standard, a total of 28.95% felt not up to date while the number was 12.42% for the National Legislation.
**Conclusion:** This study highlights concerns regarding IR and related health with up to 30% expressing concerns. While only a minority of respondents felt they needed more education or resources to protect themselves, a third lacked knowledge on the International Basic Safety Standards.
**Limitations:** Selection bias could be present as this topic may have attracted respondents with greater concerns.
**Funding for this study:** None
**Ethics committee - additional information:** The University of Southern Denmark Research Ethics Committee (23/70920) approved this project on 8 December 2023. Before accessing the questionnaire, participants were informed about the study's purpose and were assured of the confidentiality of their responses. Only those who provided their informed consent proceeded to complete the questionnaire.
**Author Disclosures:**
Helle Precht: Nothing to disclose
Karen Brage: Nothing to disclose
Martin Weber Kusk: Nothing to disclose
Pernille Lund Hansen: Nothing to disclose
Janni Jensen: Nothing to disclose
Malene Roland Pedersen: Nothing to disclose
Oliver Brage: Nothing to disclose

**Abdominal Ultrasound Simulation based on CT examinations as an educational tool for enhancing Ultrasound acquisition competences of radiography students**

R. S. T. Ribeiro, C. Schiesser, *C. Campeanu*, C. S. D. Reis; Lausanne/CH
*(cosmin.campeanu@hesav.ch)*

**Purpose or Learning Objective:** To assess the effectiveness of simulate d CT examinations as an educational intervention for enhancing radiography students' competencies in ultrasound(US).
**Methods or Background:** A pilot study was conducted with third-year students enrolled in a four-week US module. Participants had prior knowledge of physics but no US practical experience. The educational intervention utilised abdominal CT examinations to enhance competences in US image acquisition, analysis and transducer positioning. A 20 images set comprising abdominal anatomy was administered before and after the module. Assessments were made using a Likert-scale across 4 categories: anatomical identification, anatomical topographical correlation, sectional planes recognition, transducer positioning. Data were analysed using measures of central tendency and dispersion to assess improvements.
**Results or Findings:** The simulated US based on CT examinations improved students' US competencies. Anatomical Identification scores increased by 49.8%, mainly in the gallbladder (2.30-point increase), liver (1.85-points), and pancreas (1.55-points). Anatomical Topographical Correlation improved by 34.2%, particularly for the pancreas (1.80-point) and gallbladder (1.50-points), reflecting enhanced spatial understanding. Sectional Planes Recognition was enhanced by 46.5%, for gallbladder (2.40-point) and kidneys (1.86 ), demonstrating better comprehension of cross-sectional anatomy. Transducer Positioning presented a a gain of 66.1%, with improvements for gallbladder (2.60) and kidneys (2.27). Overall, the intervention effectively addressed initial skill gaps, leading to advancements in both specific anatomical structures analysis and hands-on practical competencies in US.
**Conclusion:** The use of CT examinations proved to be a valuable tool to improve not only anatomical identification but also students' spatial understanding and practical application skills in US. The greatest improvements were observed in areas that were initially the most challenging, such as pancreas and gallbladder identification and transducer positioning.
**Limitations:** Only abdominal anatomy was tested. Reduced student panel (5 students)
**Funding for this study:** Not applicable
**Ethics committee - additional information:** Not applicable
**Author Disclosures:**
Cosmin Campeanu: Nothing to disclose
Claudia Sa Dos Reis: Nothing to disclose
Ricardo Silva Teresa Ribeiro: Nothing to disclose
Claire Schiesser: Nothing to disclose

**Evaluating internship guides: Can student opinion create a virtuous circle?**

*A. Devetti*[1], S. Da Dalt[1], F. R. Fabris[2], L. Cereser[1], M. G. Belgrano[2], R. Girometti[1], C. Zuiani[1]; [1]Udine/IT, [2]Trieste/IT
*(angie.devetti@uniud.it)*

**Purpose or Learning Objective:** This study aimed to evaluate the efficacy of a peer-assessment tool designed to enhance the quality of clinical tutoring experiences for undergraduate students of a Radiographer Bachelor Degree internship program. By collecting student feedback on their tutors' performance, the institution sought to identify strengths, weaknesses, and implement targeted improvement strategies.
**Methods or Background:** Over three academic years, 940 student evaluations were collected from 180 clinical tutors across four departments (Radiotherapy, Nuclear Medicine, Medical Physics, and Diagnostics). Tutors were evaluated on seven dimensions using a 10-point Likert scale. The reliability of the evaluation tool was assessed using Cronbach's alpha, which consistently yielded values between 0.92 and 0.94.
**Results or Findings:** The results indicate a high overall level of student satisfaction with the clinical tutoring experience. The mean overall rating was 8.87 out of 10, with a standard deviation of 0.97. While slight variations were observed across departments, these differences were not statistically significant. The high Cronbach's alpha values suggest that the evaluation tool is reliable and internally consistent.
**Conclusion:** The implementation of a peer-assessment tool has proven to be an effective method for gathering valuable feedback on the quality of clinical tutoring. By providing tutors with individualized feedback, the institution has fostered a culture of continuous improvement. The aggregated data has also allowed for department-wide analysis, enabling the identification of areas where additional training or support may be needed.
**Limitations:** The evaluation tool was designed specifically for the Radiographer Bachelor Degree internship program and may not be generalizable to other healthcare settings. Additionally, the study relied solely on student perceptions of tutor performance.
**Funding for this study:** None

Ethics committee - additional information: None
**Author Disclosures:**
Stefano Da Dalt: Nothing to disclose
Angie Devetti: Nothing to disclose
Chiara Zuiani: Nothing to disclose
Manuel Gianvalerio Belgrano: Nothing to disclose
Rossano Girometti: Nothing to disclose
Lorenzo Cereser: Nothing to disclose
Francesca Romana Fabris: Nothing to disclose

**09:30-11:00** **Research Stage 1**

### Research Presentation Session: Musculoskeletal

### RPS 2310

### Imaging of the various pathologies of the spine

**Moderator**
C. Loupatatzis; Männedorf/CH
*(c.loupatatzis@spitalmaennedorf.ch)*

### 3D Ultrashort Echo Time MRI for Assessing the Cartilaginous Endplate of the lumbar intervertebral discs: Correlation with Disc Degeneration and Modic Changes in Conventional Fast Spin Echo Sequences

*Y. Kim*[1], J. G. Cha[2], S. Lee[1]; [1]Seoul/KR, [2]Bucheon/KR
*(kimyeoju@hanyang.ac.kr)*

**Purpose or Learning Objective:** To investigate the association between cartilaginous endplate (CEP) abnormalities on 3-dimensional ultrashort echo time MRI with cone trajectory technique (3D UTE) and disc degeneration and endplate Modic change on conventional MRI.
**Methods or Background:** Ninety one patients (44 men, 47 women, mean age: 55.75 years, range: 19-85 years) underwent MRI of the lumbar spine with conventional sagittal T1, T2-weighted and fat-suppressed T2 weighted fast spin echo sequence and sagittal 3D UTE cone trajectory technique (TR = 16.1 ms, TE = 0.032 ms and 6.6 ms) in 3T MRI. Two musculoskeletal radiologists assessed CEP abnormalities (irregularity, thickening, thinning and defects) of the superior and inferior endplates of the L3-4, L4-5 and L5-S1 discs on 3D UTE and disc degeneration with the Pfirrmann grading system, and presence of Modic change of the endplate on conventional MRI by consensus. The relationship of CEP abnormalities with the disc degeneration and Modic change was tested using Pearson's chi-square test and Spearman's correlation analysis.
**Results or Findings:** All CEP abnormalities were positively correlated with Pfirrmann grading system (Spearman ρ, 0.31-0.47) and Modic change (Spearman ρ, 0.24-0.50) with statistical significance ($p < 0.001$ for all Pearson's chi-square and Spearman's correlation analysis).
**Conclusion:** The CEP abnormality in 3D UTE MRI may be associated with the severity of disc degeneration and the presence of Modic change.
**Limitations:** The number of patients in the study is relatively small. MRI findings did not correlate with pathological findings. The MRI findings did not correlate with the patient's clinical symptoms or prognosis. This is a cross-sectional study that does not allow for a longitudinal study of the patient.
**Funding for this study:** None
**Ethics committee - additional information:** This study was designed prospectively, and was approved by the Inha University hospital's review board, and informed consent was obtained from all the participating patients.
**Author Disclosures:**
Seunghun Lee: Nothing to disclose
Yeoju Kim: Nothing to disclose
Jang Gyu Cha: Nothing to disclose

### Added Value of Color-Coded Fat-Calcium Dual-Energy CT in the Detection of Spine Occult Bone Metastasis – a Pilot Study

*J. Li*[1], J. Liu[2]; [1]Fujian/CN, [2]Xiamen/CN
*(1508883851@qq.com)*

**Purpose or Learning Objective:** To assess the capability of color-coded Fat-Calcium dual-energy CT (DECT) in identifying spinal occult bone metastases (S-OBMs).
**Methods or Background:** DECT images of a consecutive series of lung cancer patients were retrospectively analyzed. Two radiologists reviewed conventional CT images and color-coded Fat-Calcium images, recording the locations (diffuse infiltration, focal involvement of vertebral cancellous, vertebral edges, basivertebral venous plexus, and appendages) and number of occult bone metastases (OBMs) identified on the color-coded Fat-Calcium images. Diagnostic performance measures (sensitivity, specificity, positive predictive value (PPV), negative predictive value (NPV), and accuracy) were then assessed.
**Results or Findings:** A total of 24 patients were included, comprising 80 spinal occult bone metastases (S-OBMs). Color-coded DECT images show 100% sensitivity, PPV, and accuracy in diagnosing diffusely invasive S-OBMs. The overall sensitivity of color-coded DECT images for focal occult metastases was measured at 96.1%. However, the PPV and accuracy of DECT for focal OBMs were influenced by the lesion's location. The PPV and accuracy of OBMs in vertebral trabecular regions were higher than those in vertebral edges, basivertebral venous plexus, and attachments (PPV: 81%, 7.7%, 2.0%, and 6.0%, respectively; accuracy: 95.2%, 67.2%, 47.4%, and 14%, respectively). The diagnostic performance for vertebral cancellous regions was the highest, with sensitivity, specificity, PPV, NPV, and accuracy of 94%, 95.5%, 81%, 98.7%, and 95.2%, respectively.
**Conclusion:** Color-coded Fat-Calcium DECT significantly improves the detection of OBM in the spine.
**Limitations:** Firstly, it was a retrospective study with a small sample size. Secondly, this study was based on non-enhanced DECT, and whether enhanced DECT could improve diagnostic efficiency requires further study.
**Funding for this study:** Natural Science Foundation of Fujian Province, China (grant numbers: 2023J01181)
**Ethics committee - additional information:** Fujian Cancer Hospital Ethics Committee (K2023-198-01)
**Author Disclosures:**
Jianfang Liu: Nothing to disclose
Jie Li: Nothing to disclose

### Ex vivo and in vivo validation of dual-layer detector spectral-CT fat quantification of vertebrae bone marrow

*Y. F. Melzer*, G. Campbell, N. F. Schubert, I. Fiedler, B. Busse, I. Molwitz; Hamburg/DE
*(f.schwietzer@uke.de)*

**Purpose or Learning Objective:** To evaluate and validate dual-layer detector spectral-CT fat quantification (dlCT) of the vertebrae bone marrow.
**Methods or Background:** Isolated human cadaver vertebrae (n=14) of 10 body donors were scanned within 72-96 hours after death using dlCT (CT7500) at 120 kV and a 3T MRI (Ingenia) (Philips Healthcare, the Netherlands). Spherical volumes of interest (VOIs, 11 mm diameter) were placed in the center of all vertebral bodies. Additionally, n=13 patients were prospectively included (mean age 57±9 years; three females) who underwent prior to liver transplantation multiphase dlCT and MR imaging. VOIs were defined at the third lumbar vertebrae. Within the VOIs fat was quantified in dlCT scans without contrast agent using three-material decomposition for hydroxyapatite, red bone marrow, and fat. Reference values for red bone marrow were generated from the blood pool. MRI fat quantification was performed using mDIXONquant sequences (TE shortest, TR shortest, FA 3°). For statistics, Pearson's correlations and Bland Altman analysis were employed.
**Results or Findings:** Ex vivo correlation between dlCT and MRI was high ($r2=0.94$, $p<0.001$) with a mean difference of -0.55 [95% intervals of agreement -11.0, 9.9]. In vivo, correlation between dlCT and MRI was moderate ($r2=0.47$, $p=0.01$). The mean difference amounted to 14.4 [95% intervals of agreement -3.9, 32.7].
**Conclusion:** Ex vivo dlCT fat quantification of the vertebral bone marrow delivers reproducable results. In vivo measurements require further calibration of dlCT using MRI and - due to challenges of MRI fat quantification in the presences of bone - preferably histology as a reference.
**Limitations:** Small sample size due to ongoing recruitment and necessary further calibration of dlCT fat quantification with histological analyses, for which the vertebra are currently prepared by formalin fixation.
**Funding for this study:** None.
**Ethics committee - additional information:** 2023-300414-WF (Ärztekammer Hamburg)
**Author Disclosures:**
Björn Busse: Nothing to disclose
Isabel Molwitz: Nothing to disclose
Graeme Campbell: Nothing to disclose
Imke Fiedler: Nothing to disclose
Niklas Ferdinand Schubert: Nothing to disclose
Yasmin Fede Melzer: Nothing to disclose

**VERIFACT: Revealing the Hidden Epidemic of Undiagnosed Vertebral Fractures in Routine CT Scans**

H. P. Dimai, J. Igrec, *J. Steiner*, R. Riedl, M. Fuchsjäger; Graz/AT
*(jakob.steiner@medunigraz.at)*

**Purpose or Learning Objective:** Osteoporosis is a condition characterized by low bone mass and increased fracture risk. Vertebral fractures are the most common, often undetected despite serious health consequences. Studies reveal that many fractures are missed in radiographic and CT reports, with false-negative rates ranging from 30% to 84%, highlighting significant underreporting in clinical practice. The study aims to assess recognition rates in chest and abdominal CTs.
**Methods or Background:** This retrospective analysis included 1,500 CT images from 1,380 patients. Two independent board-certified radiologist with multi-year-experience reviewed each scan for vertebral fractures, noting the number, location, and type of fracture, and classifying them based on the Genant classification system (Grades 2 and 3). Rater agreement was assessed using the Kappa coefficient and AC1 statistic. Fracture documentation rates were calculated based on whether fractures were noted in the formal radiology report summary or only in the free-text narrative. Stratified analysis was performed by patient gender, fracture location, and the presence of multiple fractures.
**Results or Findings:** Vertebral fractures were found in 11.5% of patients. Agreement between the raters was excellent, with a Kappa value of 0.94 (95% CI: 0.92–0.97) and AC1 of 0.99 (95% CI: 0.98–0.99). However, fractures were documented in the summary in only 14.7% of cases (95% CI: 9.8%–20.9%), while 35.3% were mentioned only in the narrative portion (95% CI: 28.1%–43.0%). Overall, 50% of the fractures were reported in either the summary or the narrative (95% CI: 42.3%–57.8%).
**Conclusion:** While interrater agreement on fracture detection was high, the rate of documentation, particularly in the structured summary, was low. These findings suggest a need for better reporting protocols to ensure vertebral fractures are clearly communicated, improving patient outcomes and clinical decision-making.
**Limitations:** n/a
**Funding for this study:** n/a
**Ethics committee - additional information:** Approved by local ethics committee
**Author Disclosures:**
Jakob Steiner: Nothing to disclose
Regina Riedl: Nothing to disclose
Michael Fuchsjäger: Nothing to disclose
Hans Peter Dimai: Nothing to disclose
Jasminka Igrec: Nothing to disclose

**Spectral Collagen Imaging: Assessment of Thoracic Disk Herniation and Degeneration**

*M. Dimitrova*, C. Booz, S. Mahmoudi, A. Gökduman, L. D. Grünewald, S. Bernatz, E. Höhne, T. Vogl, I. Yel; Frankfurt/DE

**Purpose or Learning Objective:** This study investigates the diagnostic efficacy of Dual-Energy CT (DECT)-derived collagen maps in evaluating thoracic disk herniation and degeneration.
**Methods or Background:** A retrospective analysis was conducted involving 51 patients who underwent dual-source DECT (Somatom Force; Siemens Healthineers) and MRI of the thoracic spine within a two-week timeframe. Two blinded radiologists assessed the presence and type of herniation using the North American Spine Society's classification for intervertebral disk pathology, evaluating both conventional grayscale CT and DECT collagen maps. Diagnostic accuracy, sensitivity, and specificity were calculated with MRI as the reference standard. Additionally, subjective assessments of diagnostic confidence and image quality were performed, and inter-reader reliability was evaluated using the intraclass correlation coefficient.
**Results or Findings:** Analysis of 612 intervertebral disks showed that DECT collagen maps achieved significantly higher sensitivity (98.0%), specificity (97.7%), and diagnostic accuracy (97.7%) compared to conventional CT (sensitivity: 72.0%, specificity: 97.0%, diagnostic accuracy: 96.0%; $p < 0.001$). Substantial inter-reader reliability was noted ($\kappa=0.76$, $p < 0.001$), with DECT collagen maps providing enhanced diagnostic confidence and image quality ($p < 0.001$). For assessing disk degeneration, DECT collagen maps demonstrated high sensitivity (83.0%), specificity (81.9%), and diagnostic accuracy (83.4%) in distinguishing non/mild from moderate/severe degeneration, with inter-reader reliability also showing strong agreement ($\kappa=0.82$, $p < 0.001$). Subjective evaluations reported moderate to high diagnostic confidence (median 3.5) and moderate to good image quality (median 3.5).
**Conclusion:** DECT-derived collagen maps significantly enhance the detection of thoracic disk herniation and degeneration, offering improved diagnostic accuracy, reliability, confidence, and image quality over conventional CT. This imaging technique serves as a valuable alternative for patients who cannot undergo MRI.
**Limitations:** retrospective study single-centre Study small patient group CT system from a specific vendor
**Funding for this study:** No funding.
**Ethics committee - additional information:** Approval by local ethics committee.
**Author Disclosures:**
Christian Booz: Nothing to disclose
Ibrahim Yel: Nothing to disclose
Mirela Dimitrova: Nothing to disclose
Thomas Vogl: Nothing to disclose
Scherwin Mahmoudi: Nothing to disclose
Aynur Gökduman: Nothing to disclose
Leon David Grünewald: Nothing to disclose
Simon Bernatz: Nothing to disclose
Elena Höhne: Nothing to disclose

**Cervical spine motion in dynamic X-rays – approach, results, conclusions**

*M. J. Łubiński*, A. Majos, P. Kowalski; Lodz/PL
*(marcin.lubinski92@gmail.com)*

**Purpose or Learning Objective:** Dynamic X-ray is very usefull tool to evaluate cevical spine motion. The aim of this study is to find reference ranges, correlations and practical tips which radiologists can use in cervical spine mobility assesment.
**Methods or Background:** 288 patients aged 19-78 years old without cervical spine osteoarthritis or suffering from first degree osteoarthritis in Kellgren-Lawrance classtification were examined. We performed lateral X-rays in three projections – neutral, flexion and extension. In the functional X-ray examinations of the cervical spine, the following parameters were assessed: the Cobb angle C2-C7, angular and horizontal segmental mobility and segmental cervical curvature in flexion and extension.
**Results or Findings:** Reference ranges for extension and flexion C2-C7 Cobb angle, angular and horizontal segmental mobility were found. Analysis showed the biggest mobility of C4-C5 segment. Correlations suggest that horizontal displacement index is the most universal parameter in cervical spine mobility assessment.
**Conclusion:** A large group of patients and multitude measurements made it possible to find reliable and clinically useful reference ranges and parameters which can be used in routine evaluation of cervical spine functional tests.
**Limitations:** Not applicable.
**Funding for this study:** No funding was provided for this study.
**Ethics committee - additional information:** Not applicable
**Author Disclosures:**
Agata Majos: Author: Co-researcher
Marcin Janusz Łubiński: Author: Main researcher
Piotr Kowalski: Author: Co-researcher

**Longitudinal assessment of structural abnormalities in the lumbar spine of adolescent competitive alpine skiers over 48 months**

*G. C. Feuerriegel*, D. Meyer, D. Fitze, J. Hanimann, C. Stern, S. Fröhlich, J. Scherr, J. Spörri, R. Sutter; Zurich/CH

**Purpose or Learning Objective:** To longitudinally assess and compare spinal structural abnormalities in adolescent competitive alpine skiers over 48 months and to compare MRI findings in asymptomatic and symptomatic skiers and explore their clinical relevance.
**Methods or Background:** Adolescent competitive alpine skiers recruited for a cross-sectional MRI investigation underwent a 3T MR imaging of the lumbar spine at baseline and after 48 months. All MR images were assessed for structural changes occurring in the intervertebral disc, vertebral body and facet joints. At both baseline and follow-up, athletes' low back pain (LBP) symptoms were assessed and Athletes were classified as symptomatic if at least one 'substantial' episode of health problems related to back overuse had occurred in the 12 months prior to the MRI examination. The Wilcoxon signed-rank test and Pearson's chi-squared test were used to compare the measurements.
**Results or Findings:** A total of 63 athletes (mean age at follow-up 19.6±1.2 years, 25 female) were included in the study. A significant increase in LBP affecting training and competition was observed at follow-up ($P = 0.04$). Of the athletes with LBP, 63% (n=16) reported recurrent LBP, 14% (n=4) reported permanent backpain, and 26% (n=7) reported one-time LBP since baseline. Assessment of structural changes revealed a significant increase in the number of athletes with disc dehydration ($P < 0.001$), disc protrusions ($P = 0.002$) or disc extrusions ($P = 0.04$). Overall, structural abnormalities did not correlate with LBP ($P>0.05$).
**Conclusion:** Overuse related structural changes progress during adolescence and are not self-limiting. However, structural changes are not directly correlated with LBP. This finding may facilitate the development of appropriate treatment and prevention strategies that do not focus solely on spinal changes.
**Limitations:** Structural abnormalities were only assessed by MRI and not confirmed by other modalities.

**Funding for this study:** This study was generously supported by the Balgrist Foundation.
**Ethics committee - additional information:** Cantonal Ethics Committee Zurich
**Author Disclosures:**
Johannes Scherr: Nothing to disclose
Jörg Spörri: Nothing to disclose
Stefan Fröhlich: Nothing to disclose
Christoph Stern: Nothing to disclose
Jonas Hanimann: Nothing to disclose
Reto Sutter: Nothing to disclose
Georg Constantin Feuerriegel: Nothing to disclose
Daniela Meyer: Nothing to disclose
Daniel Fitze: Nothing to disclose

**Multiparametric quantitative MRI in Charcot-Marie-Tooth 1A inherited neuropathy: correlation with motor function and balance performance**
*D. Bianco*, F. Zaottini, S. Rinaldi, M. Pansecchi, M. Hamedani, S. Massucco, E. Rovetta, C. Martinoli; Genova/IT
*(deborahbianco@yahoo.it)*

**Purpose or Learning Objective:** We aimed to evaluate the feasibility of a quantitative multiparametric MRI protocol of lumbo-sacral plexus and proximal sciatic nerve to differentiate patients affected by Charcot-Marie-Tooth type 1A (CMT1A) neuropathy from controls and to correlate these imaging parameters with clinical grading scale of disease's severity.
**Methods or Background:** Patients with clinical, electrophysiological and genetical proven CMT1a were prospectively enrolled. The 3T MRI protocol included the following sequences: Diffusion Tensor Imaging, 2 points T2 DIXON, T1 mapping and T2 mapping. The MR parameters were independently measured by two radiologists. The same day of MRI examination, the CMT1a patients were clinically assessed using CMTNS score and Berg Balance Score (BBS). An age and sex matched control group without clinical signs of neuropathy (NN) was enrolled.
**Results or Findings:** n=11 patients (7 f,4 m), 47.57 yo ± 14.39 and n= 8 NN controls (5 f, 3 m) 46.5 yo ± 14.39 underwent MRI. The interobserver reliability of measurements was good (ICC=0,65). Lumbosacral plexus roots and sciatic nerve cross sectional area, Fractional Anisotropy (FA), T1 and T2 relaxation time were significantly different between the two groups (p<0,05). Bilateral L5 and S1 T1 relaxation values and FA significantly correlated with CMTNS (respectively R=0.86, p=0.013 and R=0,77, p=0,04) and BBS (respectively R=0.62, p=0.041 and R= 0.75, p=0,042). Sciatic Nerve FA demonstrated strong correlations with both CMTNS (R=0.92, p=0.010) and BBS (R=0.89, p=0.019).
**Conclusion:** These findings suggest that FA and T1 relaxation time of the lumbosacral plexus and sciatic nerve are the MRI parameters that better correlate with balance performance and overall functional disability in CMT1A patients, representing potential biomarker for disease severity and longitudinal evaluation.
**Limitations:** Small sample size.
**Funding for this study:** The study was funded by the Italian Ministry of health trough the public grant BANDO RICERCA FINALIZZATA 2021.
**Ethics committee - additional information:** Comitato Etico Territoriale - Regione Liguria
**Author Disclosures:**
Deborah Bianco: Nothing to disclose
Simone Rinaldi: Nothing to disclose
Federico Zaottini: Nothing to disclose
Carlo Martinoli: Nothing to disclose
Mehrnaz Hamedani: Nothing to disclose
Edoardo Rovetta: Nothing to disclose
Michelle Pansecchi: Nothing to disclose
Sara Massucco: Nothing to disclose

**T2 relaxation times of the pubic symphysis in osteitis pubis**
*N. Andjelic*[1], N. Holl[2], B. Klaan[2], M-A. Weber[2]; [1]Sremska Kamenica/RS, [2]Rostock/DE
*(nikola.andjelic92@gmail.com)*

**Purpose or Learning Objective:** This study aimed to evaluate the T2 relaxation times of the cartilage layer of the pubic symphysis in male athletes with osteitis pubis and examine the correlation between these values, pubic bone marrow edema (BME), the pubic symphysis width, and the presence of cleft injuries.
**Methods or Background:** Sixty-two male athletes (median age, 28 years) presenting with groin pain were examined using a 3-Tesla MRI system. T2 mapping was applied using a T2W sequence to assess the pubic symphysis in three ways: interpubic disc, articular cartilage, and the entire hyaline-fibrocartilage complex. T2 relaxation times were measured, and BME and cleft injuries were identified. Correlations between T2 values, symphyseal width, and the presence of BME and pubic cleft injuries were explored.
**Results or Findings:** The median T2 relaxation times for the hyaline-fibrocartilage complex, interpubic disc, and articular cartilage were 49.7 ms, 54.6 ms, and 46.2 ms, respectively, with significant differences between (p-value < 0.001), and a notable distinction between the right and left sides of the articular cartilage. Athletes with BME had higher T2 relaxation times for the hyaline-fibrocartilage complex and interpubic disc (p-value < 0.01) but not for the articular cartilage. A moderate positive correlation (r = 0.4) was found between symphysis width and T2 relaxation times.
**Conclusion:** T2 mapping provides valuable insights into the structural changes in the pubic symphysis in athletes with osteitis pubis. Higher T2 relaxation times in the interpubic disc and whole symphyseal hyaline/fibrocartilage complex are associated with BME, suggesting their potential use in evaluating osteitis pubis.
**Limitations:** The study was limited to male patients, and the absence of an asymptomatic control group is a notable limitation. Additionally, the pre-screening of all participants introduces a potential selection bias due to the study design.
**Funding for this study:** This research project was part of the ESOR Bracco Research Fellowship 2024
**Ethics committee - additional information:** The study was approved by the Ethical Committee of Rostock University (approval No. A 2020-0040)
**Author Disclosures:**
Norman Holl: Nothing to disclose
Marc-André Weber: Nothing to disclose
Bastian Klaan: Nothing to disclose
Nikola Andjelic: Nothing to disclose

**Ultrasound-guided navigation system for spine surgery**
*A. Lubansu*, P. Pandin; Brussels/BE
*(alphonse.lubansu@hubruxelles.be)*

**Purpose or Learning Objective:** Current image-guided navigation systems in spine surgery rely on ionizing radiation from intraoperative fluoroscopy or CT scans. This study evaluates the feasibility and utility of fusing intraoperative ultrasound (US) imaging with preoperative lumbar CT scans to create a novel US-guided spinal navigation system, aiming to reduce radiation exposure.
**Methods or Background:** Over one year, 25 patients undergoing lumbar spine surgery participated in this study. Cortical borders of spinal structures were co-registered and fused with preoperative CT scans. The accuracy and time required for co-registration were assessed. When navigation accuracy was within 2mm, various spinal procedures, including screw removal or placement and canal or foraminal recalibration, were performed using the system.
**Results or Findings:** Accurate co-registration was achieved in under 10 minutes for all cases. Anatomical landmarks for co-registration varied depending on the target region (sacrum, sacroiliac joint, lumbosacral junction, or lumbar vertebrae). The system facilitated percutaneous screw placement, intradural lesion localization, and optimal neural structure decompression. No complications related to US-guided navigation occurred. The total radiation dose was reduced compared to conventional non-navigated procedures.
**Conclusion:** This preliminary experience suggests that US-guided navigation for spinal procedures is feasible, accurate, safe, and potentially beneficial in reducing radiation exposure. The system demonstrated versatility across various spinal regions and procedures. Further research with larger cohorts and more indications is needed to fully understand the potential advantages and limitations of this innovative navigation system.
**Limitations:** This study is limited by its small sample size and single-center design. A direct comparison with conventional navigation techniques was not assessed. The learning curve for implementing this new technique was not evaluated.
**Funding for this study:** No
**Ethics committee - additional information:** Not applicable
**Author Disclosures:**
Alphonse Lubansu: Nothing to disclose
Pierre Pandin: Nothing to disclose

09:30-11:00 **Research Stage 2**

### Research Presentation Session: Imaging Informatics and Artificial Intelligence

### RPS 2305

### Healthy aging, body composition and prevention: the true potential of AI?

**Moderator**
M. Pop; Tg.Mures/RO

**Automated coronary calcification assessment on ungated unenhanced chest CT using an optimised nnUNet framework for patient prognostication in non-small-cell lung cancer**
*J. Y. Anifowose*, Z. Li, G. Agarwal, E. Aboagye, B. Ariff, S. Copley, M. Chen; London/UK
*(anifowoseolayinka68@gmail.com)*

**Purpose or Learning Objective:** To develop an automated software for assessing coronary calcification in non-small cell lung cancer (NSCLC) patients using an optimised deep learning nnUNet framework for disease prognostication.
**Methods or Background:** Cardiovascular risk is higher in NSCLC patients than in the general population, but is often underdiagnosed in clinical practice. Attenuation correction CTs from routinely acquired PET-CT staging scans are ungated unenhanced studies which offer an opportunity to assess this risk without incurring additional radiation exposure or radiology workload. nnUNet is a state-of-the-art deep learning architecture demonstrating superior performance in medical image segmentation applications. We trained nnUNet models for coronary calcification on ungated unenhanced chest CTs (n = 100) from a public domain dataset (Stanford AIMI) and tested them on independent data: attenuation correction CTs of PET-CT scans of NSCLC patients acquired at our multi-centre institution between 2012 and 2018 (n = 287, age: 66.8 ± 10.1, male: female 174:113). The reference truth segmentations were drawn and verified by two radiologists of 8 and 2 years of experience. Models with varying batch sizes and convolutional filters were developed and benchmarked; with the best performing one selected to develop a composite prognostic predictor, based on model-derived coronary calcification score and significant NSCLC features.
**Results or Findings:** The best performing nnUNet has a 3D_fullres configuration with batch size of 4 and patch size 28x224x224. All cases of coronary calcifications were successfully detected. Multivariable Cox showed statistical significance of disease histology and stage on patient survival. The composite predictor achieved statistically significant prognostic risk stratification (p-value < 0.05).
**Conclusion:** An optimised nnUNet framework can facilitate automated coronary calcification assessment on ungated unenhanced CT to support a composite prognostic predictor in NSCLC patients.
**Limitations:** Retrospective study. Single external validation cohort.
**Funding for this study:** Academy of Medical Sciences award SGL026\1024.
**Ethics committee - additional information:** Retrospective observational study IRAS: 243592 REC: 18HH4616
**Author Disclosures:**
Zechen Li: Nothing to disclose
Susan Copley: Nothing to disclose
Ben Ariff: Nothing to disclose
Mitchell Chen: Nothing to disclose
Eric Aboagye: Nothing to disclose
Girija Agarwal: Nothing to disclose
Jubril Yinka Anifowose: Nothing to disclose

**Deep learning-based biological age estimation from MRI predicts cardiometabolic events in the general population**
*M. Jung*[1], M. Reisert[2], H. Rieder[2], S. Rospleszcz[2], M. T. Lu[1], F. Bamberg[2], V. Raghu[1], J. Weiß[2]; [1]Boston MA/US, [2]Freiburg/DE
*(matthias.jung@uniklinik-freiburg.de)*

**Purpose or Learning Objective:** Chronological age is one of the cornerstones of medical decision-making, but it's an imperfect measure of health. We propose a deep learning framework (MRI-Age) for estimating biological age from MRI and investigated its value in predicting cardiometabolic outcomes in the general population beyond chronological age.
**Methods or Background:** We used 30,389 individuals from the German National Cohort (NAKO) to develop MRI-Age, which takes MRI-derived volumetric body composition, including subcutaneous (SAT), visceral (VAT), intramuscular adipose tissue (IMAT), and skeletal muscle (SM) from the 1st to 5th lumbar vertebra as input and outputs an age estimate in years. For downstream analyses, we calculated MRI-Age acceleration, defined as an age-specific z-score of the age estimate. We then validated this framework in an external testing set of 36,317 individuals from the UK Biobank (UKBB). Incident outcomes were diabetes, MACE, and all-cause mortality. Multivariable Cox regression assessed the association between MRI-Age acceleration categories "negative" (MRI-Age acceleration <-1), "reference" (MRI-Age acceleration -1 to 1), and "positive" (MRI-Age acceleration >1) and outcomes adjusted for traditional cardiometabolic risk factors in the UKBB.
**Results or Findings:** In 36,317 UKBB participants (65.1±7.8 years, 51.7% female; median follow-up 4.8 years), we found a higher incidence of diabetes, MACE, and death in individuals with positive MRI-acceleration. In multivariable-adjusted Cox regression, we observed a significant positive association between positive MRI-Age acceleration and diabetes (aHR: 1.87, 95% CI [1.56-2.25], p<0.001), MACE (aHR: 1.26, 95% CI [1.01-1.57], p=0.038), and all-cause mortality (aHR: 1.37, 95% CI [1.09-1.72], p=0.007).
**Conclusion:** Deep learning-derived biological age from MRI predicts cardiometabolic outcomes in the general population beyond chronological age and cardiometabolic risk factors. Individuals at high MRI-Age could benefit from personalized prevention strategies, lifestyle interventions, and treatment planning.
**Limitations:** Limited age-range. Predominantly white population.
**Funding for this study:** This project was conducted with data from the German National Cohort (NAKO) (www.nako.de). The NAKO is funded by the Federal Ministry of Education and Research (BMBF) [project funding reference numbers: 01ER1301A/B/C, 01ER1511D, and 01ER1801A/B/C/D], federal states of Germany, and the Helmholtz Association, the participating universities and the institutes of the Leibniz Association. This research has been conducted using the UK Biobank Resource under Application Number 80337. We thank all participants who took part in the NAKO and UKBB study and the staff of these research initiatives. MJ was funded by the Deutsche Forschungsgemeinschaft (DFG, German Research Foundation) - 518480401. VKR was funded by Norn Group Longevity Impetus Grant, NHLBI K01HL168231, and AHA Career Development Award 935176.
**Ethics committee - additional information:** Informed consent was obtained from all participants in the UK Biobank and the German National Cohort study. In addition, we received local IRB approval (IRB of the University of Freiburg: 23-1316-S1-retro and 24-1099-S1-retro).
**Author Disclosures:**
Susanne Rospleszcz: Nothing to disclose
Marco Reisert: Nothing to disclose
Jakob Weiß: Nothing to disclose
Matthias Jung: Nothing to disclose
Fabian Bamberg: Nothing to disclose
Hanna Rieder: Nothing to disclose
Vineet Raghu: Nothing to disclose
Michael T. Lu: Nothing to disclose

**Body Composition in the general population: MRI-derived reference curves from over 66,000 individuals and their association with cardiometabolic outcomes**
*M. Jung*[1], M. Reisert[2], H. Rieder[2], S. Rospleszcz[2], M. Russe[2], M. T. Lu[1], F. Bamberg[2], V. Raghu[1], J. Weiß[2]; [1]Boston MA/US, [2]Freiburg/DE
*(matthias.jung@uniklinik-freiburg.de)*

**Purpose or Learning Objective:** Body composition (BC) plays an important role in risk estimation in patients with cardiometabolic disease and cancer, but reference curves are missing to place individual measurements in context. We developed a deep learning framework to quantify BC from MRI to calculate reference curves and investigated its value for predicting cardiometabolic outcomes.
**Methods or Background:** BC extracted from MRI data of the UK Biobank (UKBB) and German National Cohort included 1) subcutaneous (SAT), 2) visceral (VAT), 3) intramuscular adipose tissue (IMAT), 4) skeletal muscle (SM), and 5) SM fat fraction (SMFF). Reference curves were generated using generalized additive models for each BC metric to calculate age, sex, and height-specific z-scores. Multivariable Cox regression assessed the association between z-score categories (low: z<-1; middle: z=-1-1; high: z>1) and outcomes (incident diabetes; major adverse cardiovascular events [MACE]; and all-cause mortality) adjusted for traditional cardiometabolic risk factors in the UKBB.
**Results or Findings:** Among 66,608 individuals (57.7±12.9 years; BMI: 26.2±4.5 kg/m2, 48.3% female), we observed sex differences in BC volumes and distributions with SAT, VAT, SMFF, and IMAT positively and SM negatively associated with age. We found graded associations between BC z-score categories and health outcomes in the UKBB. In multivariable adjusted Cox regression, z-score risk categories had hazard ratios of up to 2.69 for incident diabetes (high VAT), 1.41 for incident MACE (high IMAT), and 1.49 for all-cause mortality (low SM) compared to middle categories.

**Conclusion:** BC measures normalized for age, sex, and height are associated with cardiometabolic outcomes beyond traditional risk factors in the general population. We will provide open-source BC reference curves, which may accelerate the clinical translation of BC-based risk assessment for cardiometabolic disease and support future BC research.
**Limitations:** Study population is predominantly white Western European adults.
**Funding for this study:** This project was conducted with data from the German National Cohort (NAKO) (www.nako.de). The NAKO is funded by the Federal Ministry of Education and Research (BMBF) [project funding reference numbers: 01ER1301A/B/C, 01ER1511D, and 01ER1801A/B/C/D], federal states of Germany, and the Helmholtz Association, the participating universities and the institutes of the Leibniz Association. This research has been conducted using the UK Biobank Resource under Application Number 80337. We thank all participants who took part in the NAKO and UKBB study and the staff of these research initiatives. MJ was funded by the Deutsche Forschungsgemeinschaft (DFG, German Research Foundation) - 518480401. VKR was funded by Norn Group Longevity Impetus Grant, NHLBI K01HL168231, and AHA Career Development Award 935176.
**Ethics committee - additional information:** Informed consent was obtained from all participants in the UK Biobank and the German National Cohort study. In addition, we received local IRB approval (IRB of the University of Freiburg: 23-1316-S1-retro and 24-1099-S1-retro).
**Author Disclosures:**
Susanne Rospleszcz: Nothing to disclose
Marco Reisert: Nothing to disclose
Jakob Weiß: Nothing to disclose
Matthias Jung: Nothing to disclose
Maximilian Russe: Nothing to disclose
Fabian Bamberg: Nothing to disclose
Hanna Rieder: Nothing to disclose
Vineet Raghu: Nothing to disclose
Michael T. Lu: Nothing to disclose

### AI-Driven MRI Biomarker Extraction and Machine Learning Analysis of Their Association with Diabetes: A UK Biobank Study

*S. Kim*[1], D. W. Kim[1], C. Han[1], D. Kim[2], D. Yoon[1]; [1]Seoul/KR, [2]Daegu/KR
*(crown7699@yuhs.ac)*

**Purpose or Learning Objective:** To evaluate AI-derived imaging biomarkers from whole-body MRI in detecting and predicting diabetes mellitus (DM).
**Methods or Background:** An open-source multi-label segmentation model was applied to Dixon whole-body MRIs from the UK Biobank to segment organs and body compositions. Volume indices (volume/m³) and fat fractions of each structure were calculated automatically. For DM detection at the time of MRI, logistic regression was performed. Excluding baseline DM, random survival forest analysis was performed for predicting future DM. Area under curve (AUC) and Harrell's C-index was used. Performance of imaging biomarkers was compared to the Leicester Diabetes Risk Score.
**Results or Findings:** Among the 2,924 participants, 149 had DM at baseline. Of the 2,775 participants without baseline DM, 28 developed DM and were included in the survival analysis (median follow-up 4.1 years, up to 8.9 years). For DM detection, adrenal gland volume index, kidney volume index, and pancreatic fat fraction (AUC 0.748, 0.716, and 0.710 respectively) were the top classifiers. The multivariate model, using 10 selected imaging features, achieved AUC of 0.802. In survival analysis, pancreatic fat fraction, adrenal gland volume index, and torso fat volume index (C-index 0.713, 0.685, and 0.678 respectively) were the top predictors. The multivariate model with six selected imaging features achieved C-index 0.780, outperforming the Leicester Diabetes Risk Score (C-index 0.651). When imaging features were combined with clinical features, performance further improved (C-index 0.794).
**Conclusion:** AI-derived MRI biomarkers demonstrated strong performance in detecting current DM and predicting future onset, highlighting their potential utility in opportunistic screening.
**Limitations:** Further validation of the open-source segmentation model is necessary to assess its quantitative and qualitative performance.
**Funding for this study:** MD-PhD/Medical Scientist Training Program through the Korea Health Industry Development Institute, funded by the Ministry of Health & Welfare, Republic of Korea
**Ethics committee - additional information:** Our institution has received IRB approval for UK Biobank-related research, and any additional ethical considerations are adhered to in accordance with this approval.
**Author Disclosures:**
Songsoo Kim: Nothing to disclose
Donghyun Kim: Nothing to disclose
Dong Won Kim: Nothing to disclose
Changho Han: Nothing to disclose
Dukyong Yoon: Nothing to disclose

### CompositIA: an open-source pipeline for automated quantification of body composition scores from thoraco-abdominal CT scans

*R. F. Cabini*, A. Cozzi, S. Leu, B. Thelen, R. Krause, F. Del Grande, S. Rizzo, D. U. Pizzagalli; Lugano/CH
*(raffaella.fiamma.cabini@usi.ch)*

**Purpose or Learning Objective:** This study aims to develop and validate CompositIA, an automated, open-source pipeline for quantifying body composition scores from thoraco-abdominal CT scans.
**Methods or Background:** CompositIA consists of three main steps: automatic identification of the L1 and L3 vertebrae, segmentation of image slices at these spinal levels, and quantification of body composition indices. Two Deep Learning models were used: MultiResUNet for detecting CT slices intersecting the L1 and L3 vertebrae, and UNet for segmenting the corresponding axial slices. The pipeline was trained on 205 contrast-enhanced thoraco-abdominal CT scans and tested on an independent dataset of 54 scans. Manual segmentation was performed by two radiology residents, who identified the centers of the L1 and L3 vertebrae and segmented the corresponding axial slices. Performance was evaluated via mean absolute error (MAE) for L1/L3 detection, volumetric Dice similarity coefficient (vDSC) for segmentation, and mean percentage relative error (PRE), regression analysis, and Bland–Altman plots for body composition indices estimation.
**Results or Findings:** On the independent dataset CompositIA achieved a MAE of about 5 mm in detecting slices intersecting the L1 and L3 vertebrae, with a MAE < 10 mm in at least 85% of cases, and a vDSC greater than 0.85 in segmenting axial slices. Regression and Bland–Altman analyses demonstrated a strong linear relationship and good agreement between automated and manual scores (p values < 0.001 for all indices), with mean PREs ranging from 5.13% to 15.18%.
**Conclusion:** CompositIA facilitated automated quantification of body composition scores, achieving high precision in independent testing.
**Limitations:** The main limitation of the study is the small size of the training set.
**Funding for this study:** Raffaella F. Cabini, Benedikt Thelen, Rolf Krause and Diego U. Pizzagalli were supported financially by the grants ExaTrain (to Rolf Krause), and FIR (to Diego U. Pizzagalli).
**Ethics committee - additional information:** This study was approved by the local Ethics Committee (Comitato Etico Cantonale, Repubblica e Cantone Ticino, Switzerland; protocol code 2021-00943). All patients whose CT scans were included in the training set provided informed consent for the participation in the study.
**Author Disclosures:**
Andrea Cozzi: Nothing to disclose
Rolf Krause: Nothing to disclose
Stefania Rizzo: Nothing to disclose
Filippo Del Grande: Nothing to disclose
Diego Ulisse Pizzagalli: Nothing to disclose
Raffaella Fiamma Cabini: Nothing to disclose
Benedikt Thelen: Nothing to disclose
Svenja Leu: Nothing to disclose

### A novel CT-based biological age model, based on automated abdominal CT biomarkers for accurate longevity prediction

*J. Garrett*[1], M. Lee[1], A. Pyrros[2], R. Summers[3], M. Kattan[4], P. J. Pickhardt[1]; [1]Madison, WI/US, [2]Downers Grove, IL/US, [3]Bethesda, MD/US, [4]Cleveland, OH/US
*(jgarrett@uwhealth.org)*

**Purpose or Learning Objective:** To derive and test a CT-biological age (CTBA) model using explainable fully automated abdominal CT-based tissue biomarkers predictive of survival in a large adult population.
**Methods or Background:** In this retrospective cohort study, an automated suite of explainable CT-based AI algorithms quantifying skeletal muscle (L3 level), fat (L3 level), aortic calcification, bone density, and solid organs (liver/spleen/kidney volume) was applied to a large adult cohort undergoing abdominal CT between January 2001-January 2021. Multivariable Cox proportional hazards regression survival analysis was performed to determine final CT biomarker selection based on index of prediction accuracy (IPA). Using all-cause mortality as a primary outcome, the CTBA model informed only by CT biomarkers and blinded to demographics was compared to a model based on demographic data (chronological age/sex/race). The model was also applied to an external validation cohort of 40,718 adults.
**Results or Findings:** 123,281 adults (mean age, 53.6 years [SD 17.4]; 47% women) underwent abdominal CT during the study interval. Median post-CT follow-up was 5.3 years (IQR,1.9-10.4 years). CT biomarkers of greatest importance to the model were (in descending order): muscle attenuation, aortic calcification, visceral fat attenuation, and bone density. The CTBA model significantly outperformed demographic data for predicting longevity (IPA=29.2 vs. 21.7; 10-year AUC=0.880 vs. 0.779; p<0.001). Age- and sex-corrected survival HR for highest-vs-lowest risk CTBA quartile was 8.73 (95% CI,8.14-9.36); HR for highest-risk vs remaining quartiles was 3.13 (95% CI,3.04-3.23).

CTBA model performed well in the external validation cohort (IPA=28.6; AUC=0.888).
**Conclusion:** A novel CTBA model informed only by objective fully automated "opportunistically" derived abdominal CT biomarkers outperformed a demographics (CA/sex/race) based model and improves survival prediction.
**Limitations:** Data from a single large academic medical center were used for model training.
**Funding for this study:** None
**Ethics committee - additional information:** IRB Waiver of consent; retrospective analysis.
**Author Disclosures:**
Michael Kattan: Nothing to disclose
Perry J. Pickhardt: Advisory Board: Nanox AI
Matthew Lee: Nothing to disclose
John Garrett: Shareholder: NVIDIA Advisory Board: RadUnity
Ayis Pyrros: Nothing to disclose
Ronald Summers: Nothing to disclose

**Enabling Exchange of Quantitative CT-Assessed Body Composition Data using FHIR: A First Step into Interoperable Body Composition Profiling**
*Y. Wen*, J. H. Eil, K. A. Borys, J. Kohnke, K. Arzideh, J. Haubold, F. Nensa, O. Pelka, R. Hosch; Essen/DE

**Purpose or Learning Objective:** This study aims to demonstrate the integration of AI-generated body composition and organ measurements from CT images with Fast Healthcare Interoperability Resources (FHIR) to standardise and enhance CT-derived biomarker interoperability.
**Methods or Background:** FHIR is a widely used interoperability standard that enables health information exchange across different healthcare systems. With the development of AI models, modern AI applications cannot only analyse the data but also generate relevant data for patient monitoring and assessment, such as models for body composition analysis. The missing step in advancing personalised medicine is combining AI-generated healthcare results with an interoperable and standardised format. Therefore, this study integrated the results of the Body and Organ Analysis (BOA) model into FHIR profiles, including measurements of 11 semantic body regions, seven tissues, and 104 landmarks, to streamline and provide interoperability of CT-derived biomarkers in radiology.
**Results or Findings:** Two FHIR profiles, Body Composition Analysis Observation and Body Structure Volume Observation profiles, have been developed to capture body composition measurements and record the volume of body structure generated from the BOA model, incorporating terminology coding and references to related FHIR resources.
**Conclusion:** The presented FHIR profiles provide an interoperable format for AI-generated body composition data, standardising the storage and exchange of AI-generated biomarkers derived from CT images. The contributed profiles can also be extended in future work to support other radiological modalities (e.g. MRI) or other image-based AI model biomarkers (e.g. CT-based bone mineral density).
**Limitations:** The created profiles focus on tissue and organ volumetrics and should be enhanced to include other available image-based markers and imaging modalities.
**Funding for this study:** No funding was received for this study.
**Ethics committee - additional information:** This study does not require ethics committee approval, since no identifiable or sensitive patient data was used.
**Author Disclosures:**
Katarzyna Anna Borys: Nothing to disclose
Jan Horst Eil: Nothing to disclose
Johannes Haubold: Nothing to disclose
Judith Kohnke: Nothing to disclose
Yutong Wen: Nothing to disclose
Kamyar Arzideh: Nothing to disclose
René Hosch: Nothing to disclose
Felix Nensa: Nothing to disclose
Obioma Pelka: Nothing to disclose

**Transfer of a CT-based 3D body composition analysis segmentation model to MRI T2-weighted sequences using a generative adversarial network**
*C. Bojahr*[1], J. Haubold[1], O. Pollok[1], C. S. Schmidt[1], K. A. Borys[1], M. Mancino[2], L. Umutlu[1], F. Nensa[1], R. Hosch[1]; [1]Essen/DE, [2]Rome/IT

**Purpose or Learning Objective:** This study aims to adapt CT-based deep learning (DL) models using CycleGAN-based style transfer, enabling accurate body composition analysis (BCA) without extensive manual annotation on T2-weighted MRI sequences.
**Methods or Background:** This study analyzed data from 120 patients (96 train, 24 test) who underwent whole-body CT and MRI within 48 hours. A CycleGAN was trained to convert CT images to T2-weighted MRIs, producing synthetic MRIs that preserve CT structures with MRI styling. BCA was assessed on the corresponding CT scans using the Body and Organ Analysis (BOA) framework, and 10 body-region class segmentations were transferred to synthetic MRIs to train an initial nnU-Netv2 3D segmentation model. This model was used to generate proposals for all 120 MRIs, which two trainees under guidance of a radiologists (with 8 years of experience) refined. A second model was then trained on the refined segmentations, and evaluated by comparing both models to expert annotations using the Sørensen-DICE score.
**Results or Findings:** The comparison between the two models (style transfer vs. expert refined) revealed the following DICE-scores: subcutaneous tissue (0.835 vs. 0.978), muscle (0.845 vs. 0.965), abdominal cavity (0.943 vs. 0.988), thoracic cavity (0.895 vs. 0.977), bone (0.774 vs. 0.919), glands (0.576 vs. 0. 899), pericardium (0.697 vs. 0.945), mediastinum (0.731 vs. 0.914), brain (0.894 vs. 0.965), spinal canal (0.886 vs. 0.970) and the average of all classes (0.808 vs. 0.952).
**Conclusion:** The presented approach shows rapid adaptation of CT BCA models to MRI without manual annotation, achieving notable segmentation performance. When refined by experts, these metrics are further enhanced, enabling precise body composition analysis with reduced annotation effort.
**Limitations:** Validation of different MRI scanners is necessary to ensure the generalizability and robustness of the proposed method.
**Funding for this study:** No funding was provided for this study.
**Ethics committee - additional information:** Informed consent was waived by the ethics committee due to the retrospective setting.
**Author Disclosures:**
Christian Bojahr: Nothing to disclose
Katarzyna Anna Borys: Nothing to disclose
Olivia Pollok: Nothing to disclose
Johannes Haubold: Nothing to disclose
Lale Umutlu: Nothing to disclose
Cynthia Sabrina Schmidt: Nothing to disclose
Matteo Mancino: Nothing to disclose
René Hosch: Nothing to disclose
Felix Nensa: Nothing to disclose

**Deep Learning-Based Fully Automated Body Composition Analysis as a Prognostic Factor in ARDS Patients using CT-Based Opportunistic Biomarkers**
*J. Kohnke*, K. Schmidt, F. Espeter, K. Pattberg, J. Haubold, F. Nensa, R. Hosch; Essen/DE

**Purpose or Learning Objective:** Acute Respiratory Distress Syndrome (ARDS) is a severe condition with high morbidity and mortality. Early risk assessment is crucial for improving outcomes and guiding treatment. While body composition parameters have recently emerged as prognostic factors, they are not commonly considered. However, image-based Body Composition Analysis (BCA) can help extract relevant information about patients. By leveraging deep learning, these features can be effectively used for enhanced risk stratification using detailed body information.
**Methods or Background:** Thoracic CT scans from 960 ARDS patients (37.4 % female; median age = 54.7; interquartile range 43.0 - 64.6), were analyzed. The scans were obtained within two days before or after ICU admission. Extracted BCA features include lung volume and sarcopenia marker (muscle volume / bone volume). Based on the features, tertiles were determined separately for both genders (lower tertile vs. others). Kaplan-Meier, Log-Rank, and Cox-regression analyses compared 30-days survival between the tertiles.
**Results or Findings:** Kaplan-Meier analysis revealed significant differences in survival based on lung volume markers ($p = 0.02$ for male; $p = 0.09$ female) and sarcopenia ($p = 0.01$ male; $p = 0.52$ female). Cox regression indicated that Lung volume ($p = 0.02$) and gender ($p = 0.01$) had significant effects on survival, while sarcopenia ($p = 0.07$) was slightly not statistically significant for survival.
**Conclusion:** The results suggest that image based BCA from routine CT imaging could improve risk predictions in ARDS patients by using additional information of the patient's body.
**Limitations:** Although BCA parameters show promise, generalizability is limited as all data were from a single center, highlighting the need for validation in broader clinical settings. Furthermore, the differences between the results depending on gender require further investigation.
**Funding for this study:** None
**Ethics committee - additional information:** This study adhered to all guidelines defined by the approving institutional review board of the investigating hospital. The Institutional Review Board waived written informed consent due to the study's retrospective nature. Complete anonymization of all data was performed before inclusion in the study.

**Author Disclosures:**
Karsten Schmidt: Nothing to disclose
Johannes Haubold: Nothing to disclose
Judith Kohnke: Nothing to disclose
René Hosch: Nothing to disclose
Kevin Pattberg: Nothing to disclose
Felix Nensa: Nothing to disclose
Florian Espeter: Nothing to disclose

**Enhancing Autopsy Evaluations with AI-Driven Body Composition Biomarkers from Post-Mortem CT Scans**

*J. Garrett*[1], M. Golden[1], M. Lee[1], S. Berry[2], N. Appel[3], H. Edgar[3], P. J. Pickhardt[1]; [1]Madison, WI/US, [2]Kalamazoo, MI/US, [3]Albuquerque, NM/US
*(jgarrett@uwhealth.org)*

**Purpose or Learning Objective:** To correlate fully automated post-mortem CT (PMCT)-based measures of aortic calcification, skeletal muscle, and intra-abdominal fat of decedents with causes of death and comorbidities.
**Methods or Background:** Retrospective study of the New Mexico Decedent Image Database (NMDID) with non-contrast PMCT scans between 2010-2017. An automated pipeline of AI-driven algorithms for quantifying skeletal muscle, subcutaneous fat, visceral fat, and aortic calcification (Agatston score) from the abdominal component of PMCT scans was used. Scans with more than minimal decomposition were excluded. Cause of death was categorized as acute or chronic. CT-based biological age was derived using a predetermined model.
**Results or Findings:** The final cohort included 6638 decedents (mean age 50 ± 18 years; 74% male). Deaths were classified as 80% acute, 10% chronic, and 10% uncertain. Muscle density and area at the L3 level were higher in the acute group compared to the chronic group (26 HU vs. 18 HU, $p<0.001$; 192 $cm^2$ vs. 183 $cm^2$, $p<0.001$) and higher in those without cancer (25 HU vs. 16 HU, $p<0.001$; 190 $cm^2$ vs. 169 $cm^2$, $p<0.01$). Aortic Agatston scores were higher in heart disease deaths (5120 vs. 2098, $p<0.001$). Diabetic patients had higher L3 visceral fat area (227 $cm^2$ vs. 175 $cm^2$, $p<0.001$) and lower muscle density (17 HU vs. 25 HU, $p<0.001$). The chronic group had a larger biological-chronological age gap than the acute group (median age gap, 19 years vs. 10 years; $p<0.001$).
**Conclusion:** Fully automated quantitative CT-based tissue biomarkers from PMCT scans match expectations based on previous studies on living patients and correlate with acuity of death and chronic co-morbidities.
**Limitations:** The process imperfect of categorizing decedents into “acute” or “chronic” causes of death based on death certificates is imperfect without accounting for all potential medical confounders.
**Funding for this study:** None
**Ethics committee - additional information:** IRB Exempt study; non-human subjects per HIPAA
**Author Disclosures:**
Perry J. Pickhardt: Advisory Board: Nanox AI
Heather Edgar: Nothing to disclose
Matthew Lee: Nothing to disclose
Shamsi Berry: Nothing to disclose
John Garrett: Shareholder: NVIDIA Advisory Board: RadUnity
Nicollette Appel: Nothing to disclose
Max Golden: Nothing to disclose

**Deep Learning Models for Cardiomegaly Detection Enables Assessment of Cardiomegaly Prevalence in an International CT Data Repository: Insights from AICT Consortium**

*U. Zidan*[1], N. Bi[1], A. Chandrashekar[1], M. Bown[2], E. Joviliano[3], V. Grau[1], E. R. Ranschaert[4], R. Lee[1]; [1]Oxford/UK, [2]leicester/UK, [3]São Paulo/BR, [4]Ghent/BE
*(usama.zidan@nds.ox.ac.uk)*

**Purpose or Learning Objective:** To develop high-performance ML/DL pipelines for the detection and characterization of cardiomegaly in a diverse international repository of CT scans.
**Methods or Background:** The AICT consortium (www.aict.ai) consists of 10 clinical sites across 3 continents, contributing CT scans in an agnostic fashion to a common research repository. The ultimate goal is to collect 1 million CT studies, enabling ML/DL training at an unprecedented scale. . This pilot analysis includes the first 5487 unique individuals encompassing 1978 chest CT scans performed from March 2017 to September 2024. Two published models (Superem Total Segmentor) were used to detect cardiomegaly.
**Results or Findings:** Here we report the findings on cardiomegaly (defined as a cardiothoracic ratio [CTR] > 0.50). Of the 1978 individuals, 1577 did not exhibit cardiomegaly (784 males and 793 females), and 401 had cardiomegaly (178 males and 223 females). The overall prevalence of cardiomegaly is 20%. The prevalence is higher among females (22%) compared to males (19%) ($p<0.05$). The average age of those with cardiomegaly is on average 70 years old (range: 21-96) [m:65,(21-96); f:75,(36-95); $p<0.05$]. The mean CTR in those with cardiomegaly is 0.55 (±0.05) [m: 0.55 (±0.05); f: 0.56 (±0.06); p=ns].
**Conclusion:** The AICT Consortium repository, combined with high-throughput ML/DL analytic pipelines, provides novel insights into the prevalence and demographic distribution of cardiomegaly in a contemporary international cohort. This data enhances our understanding of cardiomegaly epidemiology and supports the development of advanced detection methods.
**Limitations:** [To be added based on study outcomes]
**Funding for this study:** Horizon Europe and UK Research Innovation
**Ethics committee - additional information:** The study was approved by HRA (22/HRA/2302)
**Author Disclosures:**
Vicente Grau: Nothing to disclose
Anirudh Chandrashekar: Nothing to disclose
Edwaldo Joviliano: Nothing to disclose
Regent Lee: Nothing to disclose
Usama Zidan: Nothing to disclose
Matt Bown: Nothing to disclose
Ning Bi: Nothing to disclose
Erik R. Ranschaert: Nothing to disclose

**Detection of osteoporotic vertebral body compression fracture in computed tomography scans of the chest and abdomen using artificial intelligence Nanox.AI**

*V. Mathew*, D. Pearce, N. Kate Rose, S. Saini, E. Bogoch; Toronto, ON/CA
*(vinumathew123@gmail.com)*

**Purpose or Learning Objective:** The detection of undiagnosed vertebral compression fractures (VCFs) is critical due to their association with increased risk of future fragility fractures. Primary objective is to evaluate the performance of Nanox.AI HealthOST in identifying incidental VCFs on outpatient chest and abdomen CT scans by assessing sensitivity, specificity, PPV, and NPV. Secondary objective is to quantify missed VCFs on by initial reporting radiologist.
**Methods or Background:** HealthOST is an AI solution from Nanox.AI, providing automatic image analysis of the spine from CT images to support clinicians in the evaluation and assessment of indicators of osteoporosis. Retrospective analysis on 590 outpatient CT cases from St. Michael’s Hospital at Unity Health Toronto. Two radiologists, including a senior musculoskeletal radiologist established a consensus “gold standard” for comparison with AI results. Two AI thresholds for vertebral height reduction were examined: mild (>20%) and moderate (>25%). Original radiologist reports were reviewed to quantify missed VCFs on these scans.
**Results or Findings:** At the 20% threshold, AI showed a sensitivity of 91.1%, specificity of 52.7%, PPV of 17.1%, and NPV of 98.2%. At the 25% threshold, sensitivity decreased to 79.9%, while specificity improved to 94.2%, with a PPV of 50.7% and NPV of 98.4%. AI increased fracture detection by 88% compared to initial radiologist findings at the 20% threshold and 92% at the 25% threshold.
**Conclusion:** Nanox.AI HealthOST shows potential as an effective tool for VCF screening, with high sensitivity at the 20% threshold and improved specificity at 25%. Given the variable specificity and substantial rate of false positives, a secondary review by radiologists is recommended for accuracy. Increased detection rate by the AI in comparison to the initial radiologist report highlights the AI's capability to assist in fracture detection and enhancing diagnostic accuracy.
**Limitations:** None
**Funding for this study:** AMGEN Inc.
**Ethics committee - additional information:** Ethics committee approval REB# 21-183
**Author Disclosures:**
Noah Kate Rose: Nothing to disclose
Vinu Mathew: Nothing to disclose
Dawn Pearce: Nothing to disclose
Earl Bogoch: Research/Grant Support: Amgen
Sidharth Saini: Nothing to disclose

**09:30-11:00** **Research Stage 3**

### Research Presentation Session: Chest

### RPS 2304

### Lung cancer imaging: characterisation and prognosis

**Moderator**
R.-I. Milos; Vienna/AT
*(ruxandra-iulia.milos@meduniwien.ac.at)*

**Pulmonary adenocarcinoma: Correlation of Pathological Growth Pattern and Radiological Morphology on Computed Tomography**

L. Biggemann, H. Bohnenberger, J. Vincke, P. Kraus, T. Overbeck, A. Hammerstein-Eqourd, *J. Uhlig*; Göttingen/DE
*(johannes.uhlig@med.uni-goettingen.de)*

**Purpose or Learning Objective:** Pulmonary adenocarcinoma (AC) can present with different pathological growth patterns. This study evaluates whether these growth patterns correlate with the radiological morphology of the tumor on computed tomography (CT).
**Methods or Background:** Patients with surgically resected pulmonary AC and preoperative CT imaging were retrospectively included. Cases were sampled to distribute growth patterns approximately evenly. Pathological growth patterns were assessed on a representative pathological slice. The predominant growth pattern was defined as >=60%. Radiological morphology was assessed using preoperative thoracic CT scans and compared across growth patterns using the chi-square test.
**Results or Findings:** A total of n=345 patients were included (43.8% female; median age 68 years). Pathological growth patterns were acinar (n=68), lepidic (n=51), micropapillary (n=58), papillary (n=50), mucinous (n=51), and solid (n=67). While age was balanced across AC growth patterns, lepidic and micropapillary ACs were more common in women (56.9% / 51.7%); and solid and acinar ACs more likely in men (71.6% / 57.4%; overall p <0.05). Pathological growth patterns demonstrated specific morphologies on CT regarding nodule type, margin, ground glass opacities, central low attenuation, air bronchogram, associated lymphadenopathy and location of distant metastases (each variable p<0.05). For example, lepidic ACs commonly presented as ground-glass or partially solid nodules (13.7% / 35%); acinar ACs with associated ground-glass opacities (77.9%); solid ACs with contact to pulmonary fissures (50.7%) and central low attenuation (58.2%); and mucinous ACs with air bronchogram (41.2%).
**Conclusion:** Radiological morphology of pulmonary ACs on CT correlates well with pathological growth patterns, which could aid guiding diagnostic and treatment patterns.
**Limitations:** Limitations include that only a representative pathological slice of the pulmonary AC was assessed, whereas growth patterns might vary in the full tumor volume; and that CT-imaging was performed on different CT-scanners, introducing heterogeneity.
**Funding for this study:** This study has been supported by Siemens Healthineers.
**Ethics committee - additional information:** Ehtics committee of the University Medical Center Goettingen
**Author Disclosures:**
Hanibal Bohnenberger: Nothing to disclose
Tobias Overbeck: Nothing to disclose
Paul Kraus: Nothing to disclose
Johannes Uhlig: Investigator: Siemens Healthineers Advisory Board: Bayer
Jan Vincke: Nothing to disclose
Lorenz Biggemann: Nothing to disclose
Alexander Hammerstein-Eqourd: Nothing to disclose

**AI-Based Computer-Aided Volumetry for Invasiveness Evaluation in Lung Adenocarcinoma: Influence of Radiation Dose Reduction and Reconstruction Algorithms on High-Definition CT**

*Y. Ozawa*, D. Takenaka, H. Nagata, T. Ueda, M. Nomura, T. Yoshikawa, Y. Ohno; Toyoake/JP
*(ykiooster@gmail.com)*

**Purpose or Learning Objective:** To determine the influence of radiation dose and reconstruction method on artificial intelligence (AI)-based computer-aided volumetry (CADv) for nodule component measurement and diagnostic performance to evaluate invasiveness in lung adenocarcinoma on high-definition CT (HDCT).
**Methods or Background:** 112 consecutive patients with 181 lung adenocarcinomas underwent thin-section HDCTs at standard-dose (SDCT: 9.0±1.8 mGy), reduced-dose (RDCT: 1.7±0.2 mGy) and ultra-low-dose (ULDCT: 0.8±0.1 mGy) levels. All HDCT data were reconstructed with hybrid-type iterative reconstruction (IR) and deep learning reconstruction (DLR). Then, standard references for solid and GGO components and consolidation-to-tumor ratio (CTR) were computationally determined with the simultaneous truth and performance level estimation (STAPLE) method from annotated CT data by three board-certified chest radiologists. Then, each component volume and CTR on all HDCT data were measured by AI-based CADv software. Each component volume and consolidation-to-tumor ratio (CTR) were correlated between CADv measurement on each CT data and standard reference. Then, Measurement differences of each index between standard reference and each CADv measurement were compared among all CT data by Tukey's HSD test. Finally, diagnostic performance of invasiveness was compared among all CTR measurements by ROC analysis.
**Results or Findings:** There were significant correlations for each component and volume and CTR on all HDCTs (hybrid-type IR: 0.71≤r≤0.88, p<0.0001; DLR: 0.71≤r≤0.88, p<0.0001). Mean differences between each CADv measurement and standard reference had no significant differences among all HDCTs (p>0.05). Area under the curve (AUC) of each HDCT with DLR (SDCT: AUC=0.98, RDCT: AUC=0.98, ULDCT: AUC=0.97) was significantly larger than all HDCTs with hybrid-type IR (AUC=0.95, p<0.05).
**Conclusion:** Reconstruction method rather than radiation dose reduction had significantly affected to diagnostic performance of AI-based CADv for invasiveness evaluation in lung adenocarcinoma on HDCT.
**Limitations:** Number of subjects
**Funding for this study:** Reconstruction method is more important than radiation dose reduction for keeping diagnostic performance of AI-based CADv for invasiveness evaluation in lung ademocarcinoma on high-definition CT.
**Ethics committee - additional information:** Research grants from Canon Medical Systems Corporation and Smoking Research Foundation
**Author Disclosures:**
Yoshiyuki Ozawa: Research/Grant Support: Grants-in-Aid for Scientific Research from the Japanese Ministry of Education, Culture, Sports, Science and Technology Research/Grant Support: Smoking Research Foundation
Masahiko Nomura: Nothing to disclose
Takahiro Ueda: Research/Grant Support: Grants-in-Aid for Scientific Research from the Japanese Ministry of Education, Culture, Sports, Science and Technology
Daisuke Takenaka: Nothing to disclose
Hiroyuki Nagata: Research/Grant Support: Grants-in-Aid for Scientific Research from the Japanese Ministry of Education, Culture, Sports, Science and Technology Research/Grant Support: Canon Medical Systems Corporation
Takeshi Yoshikawa: Nothing to disclose
Yoshiharu Ohno: Research/Grant Support: Smoking Research Foundation Research/Grant Support: Canon Medical Systems Corporation

**Variability of Enlarged Mediastinal Lymph Node Measurement in CT: Manual vs. Automatic Assessment**

A. Olesinski, R. Lederman, Y. Azraq, J. Sosna, *L. Joskowicz*; Jerusalem/IL
*(josko@cs.huji.ac.il)*

**Purpose or Learning Objective:** Detection of enlarged mediastinal lymph nodes (LNs) in CECT chest scans is necessary for the assessment of oncology patients. It is performed by manually measuring the short axis length (SAL) of the LNs. We aimed to quantify the interobserver agreement and variability of the SAL measurements and to compare them to automatically computed SALs from volumetric LN delineations.
**Methods or Background:** We retrospectively analyzed 40 CECT chest scans from patients with mediastinal lymphadenopathy. Two radiologists with 25 years and 35 years of experience independently measured the SALs of 471 LNs by: identifying the scan slice in which the LN appears largest, drawing the line segment along its short axis; and classifying the LN as normal (SAL<10mm) or enlarged (SAL≥10mm). Four weeks later, one of the radiologists manually segmented the volume of the LN's along its contours. The LNs were automatically classified as normal/enlarged based on their SALs computed from the delineation (COMP). Confusion matrices were computed, as well as the differences between the SALs.
**Results or Findings:** The normal/enlarged LN overall agreement (371 normal LNs, 49 enlarged LNs) between both radiologists was 94.8% (420/471). For agreement/disagreement, the SAL differences (std) were 1.2(1.1)mm and 3.5(3.2)mm. Note that the SALs difference is nearly twice as large for disagreement as for agreement. The normal/enlarged overall agreement between the manual and the automatically computed SALs and both radiologists was 94.8%(421/471) and 92.4%(411/471).
**Conclusion:** Identification of enlarged mediastinum lymph nodes in chest CECT based on short axis measurements derived manually or computed from lymph nodes delineations has high accuracy. The agreement of the computed SALs from LN contour delineations is within the manual SALs interobserver variability. Accurate automatic LN segmentations can be obtained with existing methods.
**Limitations:** Single institution and single contour delineation.
**Funding for this study:** None.
**Ethics committee - additional information:** Yes, Helsinki Committee of the Hadassah University Medical Center
**Author Disclosures:**
Richard Lederman: Consultant: HighRAD
Leo Joskowicz: Consultant: HighRAD
Jacob Sosna: Consultant: HighRAD
Alon Olesinski: Consultant: HighRAD
Yusef Azraq: Nothing to disclose

**Multiparametric 18F-FDG PET/MRI based on restrictive spectrum imaging and amide proton transfer-weighted imaging facilitates the assessment of lymph node metastases in non-small cell lung cancer**
*N. Meng*[1], X. Liu[1], J. Pan[1], X. Yu[1], Y. Wu[1], Y. Yang[2], Z. Wang[3], M. Wang[1]; [1]Zhengzhou/CN, [2]Bei Jing/CN, [3]Shanghai/CN
*(821157922@qq.com)*

**Purpose or Learning Objective:** To investigate the value of multiparametric 18F-FDG PET/MRI based on tri-compartmental restrictive spectrum imaging (RSI), amide proton transfer-weighted imaging (APTWI), and diffusion-weighted imaging (DWI) in the assessment of lymph node metastases (LNM) of non-small cell lung cancer (NSCLC)
**Methods or Background:** A total of 152 NSCLC patients were enrolled. 18F-FDG PET- derived parameter (SUVmax), RSI-derived parameters (f1, f2, and f3), APTWI-derived parameter (MTRasym(3.5ppm)), DWI-derived parameter (ADC), and were calculated. Logistic regression analysis was used to identify independent predictors, and combined diagnostics. AUC, calibration curves and decision curve analysis (DCA) were employed to assess the performance of the combined diagnostics.
**Results or Findings:** MTRasym(3.5ppm), SUVmax, f2, and f3 were higher and ADC and f1 were lower in LNM-positive group than in LNM-negative group (all $P < 0.05$). Maximum lesion diameter, f1, MTRasym(3.5ppm) , SUVmax, and ADC were independent predictors of LNM status in NSCLC patients, and the combination of them had an optimal diagnostic efficacy (AUC = 0.978; sensitivity = 95.35 %; specificity = 90.91 %), which was significantly higher than maximum lesion diameter, f1, MTRasym(3.5ppm), SUVmax, and ADC (AUC = 0.774, 0.810, 0.832, 0.834, and 0.783, respectively, and all $P < 0.01$). The combined diagnosis showed a good performance (AUC = 0.968) in the bootstrap (1000 samples)-based internal validation. Calibration curves and DCA demonstrated that the combined diagnosis not only provided better stability, but also resulted in a higher net benefit for the patients involved.
**Conclusion:** Multiparametric 18F-FDG PET/MRI based on RSI, APTWI, and DWI is beneficial for the non-invasive assessment of LNM status in NSCLC, and the combination of maximum diameter, f1, MTRasym(3.5ppm), SUVmax, and ADC may serve as a promising biomarker.
**Limitations:** This study was conducted at a single institution with a relatively small sample size
**Funding for this study:** The National Key R&D Program of China (2023YFC2414200), the National Natural Science Foundation of China (82371934), the Joint Fund of Henan Province Science and Technology R&D Program (225200810062).
**Ethics committee - additional information:** The current study received approval from the local ethics committee, and all participants provided written informed consent for participation.
**Author Disclosures:**
Xue Liu: Nothing to disclose
Yang Yang: Nothing to disclose
Jiayin Pan: Nothing to disclose
Nan Meng: Nothing to disclose
Meiyun Wang: Nothing to disclose
Yaping Wu: Nothing to disclose
Zhe Wang: Nothing to disclose
Xuan Yu: Nothing to disclose

**Diagnostic Accuracy in NSCLC Lymph Node Staging with Total-Body and Conventional PET/CT**
*C. Mingels*[1], M. H. Madani[1], F. Sen[1], H. Nalbant[1], Y. G. Abdelhafez[1], M. Guindani[2], R. Badawi[1], B. A. Spencer[1], L. Nardo[1]; [1]Sacramento/US, [2]Los Angeles, CA/US

**Purpose or Learning Objective:** To characterize diagnostic accuracy for nodal (N)-staging with [18F]FDG Total-Body (TB) and short-axial field-of-view (SAFOV) PET/CT in non-small cell lung cancer (NSCLC) patients.
**Methods or Background:** In this prospective, randomized, single center head-to-head comparative study 48 patients underwent TB and SAFOV PET/CT. 700 nodal levels (1R/L, 2R/L, 3a/p, 4R/L, 5, 6, 7, 8R/L, 9R/L, 10-14R/L) of 28 patients could be associated to histopathological findings, imaging after localized or systemic treatment, which allowed calculation of sensitivity, specificity, positive (PPV) and negative predictive value (NPV). Thresholds for maximum standardized uptake value (SUVmax), tumor-to-background ratio (TBR), metabolic tumor volume (MTV) and total-lesion glycolysis (TLG) were calculated.
**Results or Findings:** TB and SAFOV PET/CT showed high diagnostic accuracy indices for patient-based N-staging. Sensitivity and specificity were 86.0% (CI: 77.0-95.0%) and 98.3% (CI: 97.3-99.3%) for TB; 77.2% (CI: 66.3-88.1%) and 97.4% (CI: 96.1-98.6%) for SAFOV PET. PPV was higher for TB (81.7%, CI: 71.9-91.5%) compared to SAFOV PET (72.1%, CI: 60.9-83.4%), however, this finding was not statistically significant (p=0.08). NPV for TB (98.6%, CI: 97.9-99.6%) and SAFOV PET/CT (98.0%, CI: 96.9-99.1%) were comparable (p=0.22). Overall, NSCLC N-staging was affected in six cases on SAFOV and only in one case on TB PET/CT. Semi-quantitative analysis revealed a SUVmax-threshold of 3.0 to detect TP lesions on both scanners. TBR, MTV and TLG thresholds were lower on TB compared to SAFOV PET (TBR: 1.2 vs. 1.7, MTV: 0.5 ml vs. 1.0 ml and TLG: 1.0 ml vs. 3.0 ml).
**Conclusion:** TB and SAFOV PET/CT showed high diagnostic accuracy for N-staging in NSCLC. Sensitivity and PPV on TB PET/CT were slightly higher compared to SAFOV PET/CT. TB PET/CT showed lower rate of incorrect N-staging and lower semi-quantitative thresholds.
**Limitations:** Small sample size, composite reference standard with imaging
**Funding for this study:** Research reported in this publication was supported by the National Institutes of Health under award number R01CA249422. The work was also supported by the In Vivo Translational Imaging Shared Resources with funds from NCI P30CA093373 and by the Fred and Julia Rusch Foundation for Nuclear Medicine Research and Education. Hande Nalbant's funding is partially provided by United Imaging Health's UIH Fellowship Gift.
**Ethics committee - additional information:** This study was approved by the UC Davis institutional review board (IRB 1506448). Written informed consent for inclusion was obtained. The study was performed in accordance with the Declaration of Helsinki.
**Author Disclosures:**
Fatma Sen: Nothing to disclose
Hande Nalbant: Nothing to disclose
Clemens Mingels: Nothing to disclose
Michele Guindani: Nothing to disclose
Mohammad H. Madani: Nothing to disclose
Ramsey Badawi: Nothing to disclose
Lorenzo Nardo: Nothing to disclose
Benjamin A. Spencer: Nothing to disclose
Yasser Gaber Abdelhafez: Nothing to disclose

**Whole-lesion iodine map histogram analysis versus single-slice spectral CT parameters for determining of visceral Pleural Invasion in NSCLC**
*K. Zhu*, J. Zhou; Lanzhou/CN
*(18835359804@163.com)*

**Purpose or Learning Objective:** To evaluate and compare the performances of whole-lesion iodine map histogram analysis to those of single-slice spectral CT parameters in discriminating of visceral pleural invasion in NSCLC.
**Methods or Background:** A total of 99 NSCLC patients underwent preoperative spectral CT and were divided into two groups: VPI and non-VPI.There were 65 men and 34 women with a mean age of 59.33 ± 8.62 (standard deviation) years(range:37-79 years) .The whole-lesion iodine map histogram parameterswere measured for each NSCLC patient. By placing regions of interest at representative levels of the tumor and normalizing them, spectral CT parameters IC and NIC were obtained. Discriminating capabilities of spectral CT and histogram parameters were assessed and compared using area under the ROC curve (AUC)and logistic regression models.
**Results or Findings:** The SD, Variance and CV of the iodine map histogram analysis,and iodine concentration and normalized iodine concentration of single-slice spectral CT parameters were significantly different of visceral pleural invasion in NSCLC ($P < 0.001$ to $P = 0.03$). The CV of histogram parameters (AUC=0.65; 95% [CI]: 0.54-0.76) and normalized iodine concentration (AUC=0.75; 95% CI: 0.64-0.85) from spectral CT parameters had the best performance for distinguishing whether visceral pleural invasion occurred in NSCLC. At ROC curve analysis no significant differences in AUC were found between histogram parameters (AUC = 0.84; 95% CI: 0.76-0.91) and spectral CT parameters (AUC = 0.66; 95% CI: 0.55-0.77) ($P = 0.24$).
**Conclusion:** Both whole-lesion iodine map histogram analysis and single-slice spectral CT parameters help discriminate whether of visceral pleural invasion in NSCLC, and the single-slice spectral CT parameters performed better in terms of diagnostic efficacy.
**Limitations:** This finding has not been validated in an independent population, limiting their generalizability. Future prospective studieswith larger and external patient cohorts are warranted.
**Funding for this study:** This research has been supported by the National Natural Science Foundation of China (82371914).

**Ethics committee - additional information:** The study was approved by the Institutional Ethics Committee (2021A-498) and exempted from patient informed consent.
**Author Disclosures:**
Junlin Zhou: Nothing to disclose
Kaibo Zhu: Nothing to disclose

**MRI-Based Molecular Imaging vs. FDG-PET/CT: Capability for Postoperative Recurrence Prediction with FDG-PET/CT in Stage I NSCLC Patients**
*Y. Ozawa*, H. Nagata, T. Ueda, M. Nomura, T. Yoshikawa, D. Takenaka, Y. Ohno; Toyoake/JP
*(ykiooster@gmail.com)*

**Purpose or Learning Objective:** To compare the prediction capability for postoperative recurrence among FDG-PET/CT and MRI-based molecular information from chemical exchange saturation transfer (CEST) imaging and diffusion-weighted imaging (DWI) in stage I non-small cell lung cancer (NSCLC) patients.
**Methods or Background:** 79 pathologically diagnosed and surgically treated NSCLC patients who underwent CEST imaging, DWI and FDG-PET/CT, follow-up and pathological examinations were included in this study. According to the follow-up and pathological examination results, all patients were divided as recurrence (n=13) and non-recurrence (n=66) groups. In each lesion, magnetization transfer ratio asymmetry at 3.5ppm (MTRasym), apparent diffusion coefficient (ADC) and SUVmax of each nodule were assessed by ROI measurements. To compare all indexes between two groups, Student's t-test was performed. To determine the significant predictors, multiple logistic regression analysis was performed. Then, ROC analysis was performed to compare distinguishing two groups among all indexes and combined significant predictors. Finally, sensitivity (SE), specificity (SP) and accuracy (AC) were compared among all methods by McNemar's test.
**Results or Findings:** There was significant difference of each index between two groups (p<0.05). Multiple logistic regression analyses determined MTRasym (Odds ratio [OR]: 1.31, p=0.03) and ADC (OR: 0.002, p=0.008) as significant predictors. When applied each threshold value, SPs and ACs of MTRasym (SP: 81.8%, AC: 82.2%), ADC (SP: 87.9%, AC: 86.1%) and combined predictors (SP: 89.4%, AC: 89.9%) were significantly higher than those of SUVmax (SP: 69.7%, p<0.05; AC: 72. %, p<0.05). Moreover, AC of combined predictors was significantly higher than that of MTRasym (p=0.03).
**Conclusion:** MRI-based molecular information has better prediction capability for postoperative recurrence than FDG-PET/CT in stage I NSCLC patients.
**Limitations:** Limited study cohort number and follow-up periods in some patients are considered as limitations in his study.
**Funding for this study:** Canon Medical Systems Corporation
**Ethics committee - additional information:** Fujita Health University Hospital
**Author Disclosures:**
Yoshiyuki Ozawa: Research/Grant Support: Grants-in-Aid for Scientific Research from the Japanese Ministry of Education, Culture, Sports, Science and Technology Research/Grant Support: Smoking Research Foundation
Masahiko Nomura: Nothing to disclose
Takahiro Ueda: Research/Grant Support: Grant-in-Aid for Scientific Research from the Japanese Ministry of Education, Culture, Sports, Science and Technology
Daisuke Takenaka: Nothing to disclose
Hiroyuki Nagata: Research/Grant Support: Canon Medical Systems Corporation Research/Grant Support: Grants-in-Aid for Scientific Research from the Japanese Ministry of Education, Culture, Sports, Science and Technology
Takeshi Yoshikawa: Nothing to disclose
Yoshiharu Ohno: Research/Grant Support: Canon Medical Systems Corporation Research/Grant Support: Smoking Research Foundation

**Interstitial lung abnormalities are significant poor prognostic factors in resected clinical stage Ⅰ non-small cell lung cancer**
*T. Hino*, T. Akamine, T. Hida, K. Sagiyama, Y. Yamasaki, K. Tabata, K. Ishigami; Fukuoka/JP
*(hino.takuya.372@m.kyushu-u.ac.jp)*

**Purpose or Learning Objective:** Interstitial lung abnormalities (ILA) are known to be associated with increased mortality; however, the impact of ILA on post-operative prognosis of non-small cell lung cancer (NSCLC) remains unclear. The aim of study is to assess the association between mortality in post-operative clinical stage Ⅰ NSCLC patients and the presence of ILA.
**Methods or Background:** We retrospectively evaluated the patients who underwent chest CT, followed by radical resection for clinical stage Ⅰ NSCLC from 2006 to 2018. The presence of ILA was evaluated on high-resolution CT images by two radiologists and one thoracic surgeon. Five-year overall survival (OS), recurrence-free survival (RFS), and cumulative incidence of other cause of death stratified by the presence of ILA were assessed.
**Results or Findings:** Among 709 patients included in this study, 80 patients had ILA (11.2%). Five-year OS and RFS were significantly lower in patients with ILA than those without ILA (43.6% vs. 90.1 %, log-rank test p<0.001; 44.7% vs. 80.6 %, log-rank test p<0.001). Multivariable analysis demonstrated that the presence of ILA was an independent poor prognostic factor in both OS and RFS (HR: 1.45, 95% CI: 1.14–1.84, p=0.003; HR: 1.37, 95% CI: 1.08–1.74, p=0.010, respectively). Five-year cumulative incidence of other cause of death was significantly higher in patients with ILA than those with non-ILA (21.1% vs. 5.4%, Gray's test p<0.001).
**Conclusion:** The presence of ILA affected the cause of other death and was an independent poor prognostic factor in clinical stage Ⅰ NSCLC.
**Limitations:** All the participants were retrospectively collected. ILA was not conformed with pathological specimens.
**Funding for this study:** N/A
**Ethics committee - additional information:** Kyushu University Hospital IRB No. 23240-00
**Author Disclosures:**
Kosuke Tabata: Nothing to disclose
Kousei Ishigami: Nothing to disclose
Yuzo Yamasaki: Nothing to disclose
Akamine Takaki Akamine: Nothing to disclose
Koji Sagiyama: Nothing to disclose
Takuya Hino: Nothing to disclose
Tomoyuki Hida: Nothing to disclose

**Intratumoral and peritumoral CT radiomics in predicting anaplastic lymphoma kinase mutations and survival in patients with lung adenocarcinoma: a multicenter study**
*G. Lin*, W. Chen, J. Ji; Lishui/CN

**Purpose or Learning Objective:** To explore the value of intratumoral and peritumoral radiomics in preoperative prediction of anaplastic lymphoma kinase (ALK) mutation status and survival in patients with lung adenocarcinoma.
**Methods or Background:** We retrospectively collected data from 505 eligible patients with lung adenocarcinoma from four hospitals (training and external validation sets 1–3). The CT-based radiomics features were extracted separately from the gross tumor volume (GTV) and GTV incorporating peritumoral 3-, 6-, 9-, 12-, and 15-mm regions (GPTV3, GPTV6, GPTV9, GPTV12, and GPTV15), and screened the most relevant features to construct radiomics models to predict ALK (+). The combined model incorporated radiomics scores (Rad-scores) of the best radiomics model and clinical predictors was constructed. Performance was evaluated using receiver operating characteristic (ROC) analysis. Survival outcomes were examined using the Cox proportional hazards model.
**Results or Findings:** The GPTV3 radiomics model using a support vector machine (SVM) algorithm achieved the best predictive performance, with the highest average AUC of 0.811 in the validation sets. Clinical TNM stage and pleural indentation were independent predictors. The combined model incorporating the GPTV3-Rad-score and clinical predictors achieved higher performance than the clinical model alone in predicting ALK (+) in three validation sets (AUC: 0.855 vs. 0.648, 0.882 vs. 0.634, 0.810 vs. 0.663). The prediction score of the combined model could stratify survival outcome in patients receiving ALK-TKI therapy (P=0.026) and immunotherapy (P=0.012).
**Conclusion:** The presented combined model based on GPTV3 effectively mined tumor features to predict ALK mutation status and stratify survival in patients with lung adenocarcinoma.
**Limitations:** This is a retrospective study and may have varying degrees of selection bias.
**Funding for this study:** This research was funded by the National Natural Science Foundation of China (Grant No.82072026 to Jiansong Ji), Key Project of Joint Construction by Provincial and Ministerial Authorities (Grant No.WKJ-ZJ-2452 to Minjiang Chen), Medical and Health General Project of Zhejiang Province (Grant No. 2024KY568 to Weiyue Chen, and No. 2023KY425 to Guihan Lin).
**Ethics committee - additional information:** This study was approved by the Institutional Review Boards, and the requirement for informed consent was waived due to retrospective nature.
**Author Disclosures:**
Jiansong Ji: Nothing to disclose
Weiyue Chen: Nothing to disclose
Guihan Lin: Nothing to disclose

**09:30-11:00** **Research Stage 4**

### Research Presentation Session: Genitourinary

### RPS 2307

### Malignant lesions of the female pelvis: advances in imaging techniques, diagnostic and follow-up

**Moderator**
A. M. Hötker; Zurich/CH

### A Machine Learning Model Based on Endometrium MRI Radiomics to Predict Histological Diagnosis From Biopsy in Subjects at Risk of Endometrial Cancer: Pilot study

*R. V. Ninkova*, M. Gennarini, V. Miceli, A. Cupertino, F. Curti, C. Catalano, L. Manganaro; Rome/IT
*(roberta.ninkova@gmail.com)*

**Purpose or Learning Objective:** Aim of this study was to develop a machine learning model based on Magnetic Resonance Imaging (MRI) to stratify the single-subject risk of endometrial cancer (EC).
**Methods or Background:** From September 2023 to July 2024, we collected MRI images from 41 patients. Among these subjects, 15 patients (36.6%) belonged to class "microsatellite instability (MSI)" and 26 patients (63.4%) belonged to class "microsatellite stability (MSS)", according to histological diagnosis from biopsy. This image set was used for the training and cross-validation of different machine learning models. A robust radiomic approach was applied, under the hypothesis that radiomic feature could be able to capture the disease heterogeneity among the two groups.
**Results or Findings:** Three models consisting of 3 ensembles of machine-learning classifiers (random forests, support vector machines and k-nearest neighbor classifiers) were developed for the binary classification task of interest ("MSI" vs. "MSS"), based on supervised learning, using histological diagnosis from biopsy as reference standard. The best model showed ROC-AUC mean value of 84 % [78.4-89.8], accuracy mean value of 75.6% [69.6-81.7], sensitivity mean value of 68.9 % [59.3-78.5], specificity mean value of 79.5 % [74-85], PPV mean value of 71.66 %[58.1-73.9], and NPV mean value of 81.6% [76.3-86.9] (p <0.005 mean value).
**Conclusion:** The radiomics-based machine learning model achieved a high diagnostic performance in the molecular stratification of patients with EC, which could improve risk stratification and support clinical therapeutic decision. This is a preliminary study which could provide a new perspective for patients with EC, allowing a complete and accurate identification of the disease and promoting personalized treatment.
**Limitations:** The primary limitation of this study is the small patient cohort. Expanding the sample size and investigating additional molecular pathological profiles will be necessary for further validation.
**Funding for this study:** None
**Ethics committee - additional information:** The study was performed in line with the principles of the Declaration of Helsinki.
**Author Disclosures:**
Marco Gennarini: Nothing to disclose
Valentina Miceli: Nothing to disclose
Federica Curti: Nothing to disclose
Angelica Cupertino: Nothing to disclose
Roberta Valerieva Ninkova: Nothing to disclose
Carlo Catalano: Nothing to disclose
Lucia Manganaro: Nothing to disclose

### FIGO 2023 staging of endometrial cancer: is there still a role for radiology?

*A. Rame*[1], S. Bottazzi[1], G. Avesani[1], M. Bonatti[2], V. Celli[1], E. Perrone[1], T. Pasciuto[1], B. Gui[1], E. Sala[1]; [1]Rome/IT, [2]Bolzano/IT
*(annecopper95@gmail.com)*

**Purpose or Learning Objective:** To investigate the role of MRI in the preoperative evaluation of endometrial cancer (EC) considering the new FIGO 2023 staging system.
**Methods or Background:** Patients diagnosed with EC from two institutions between 2019 and 2023 were retrospectively included. Inclusion criteria were the availability of preoperative MRI and biopsy, molecular data and definitive histopathological data obtained after surgery. Two radiologists retrieved MRI findings from reports and combined them with biopsy results (grading and histology) to determine the FIGO 2023 preoperative stage. Definitive histopathological and molecular data served as the gold standard (final staging). The preoperative evaluation was compared with final staging (FIGO stages I, II, III and IV as independent categories) and discrepancies were recorded.
**Results or Findings:** 231 patients were included. The agreement between preoperative evaluation and final staging was 74% (171/231), while the FIGO stage was discordant at 26% (60/231). Causes of discordance were: lymph node (LN) involvement in 26.7% (16/60), changes in grading or histological subtype between biopsy and surgical specimen in 23.3% (14/60), metastases and carcinosis in 15% (9/60: 5 in the upper abdominal peritoneum, 1 in the pelvic peritoneum and 3 distant), the presence of substantial lymphovascular space invasion (LVSI) in 18.2% (10/55). Less common causes of discrepancies were myometrial invasion (6.7%; 4/60), cervical stroma invasion (5%; 3/60), ovarian involvement (3.3%; 2/60) and vaginal invasion (1.7%; 1/60). Overall, parameters not assessable preoperatively (LVSI and definitive grading and histological subtype) accounted for staging discrepancies in 10.4% (24/231) of cases.
**Conclusion:** Our study showed that the inclusion of parameters not assessable preoperatively in the new FIGO 2023 staging system does not significantly diminish the role of MRI in the preoperative evaluation of EC, resulting in staging discrepancy in only approximately 10% of cases.
**Limitations:** No limitations.
**Funding for this study:** No funding was received for this study.
**Ethics committee - additional information:** ID 6194, approved on 7/12/2023
**Author Disclosures:**
Matteo Bonatti: Nothing to disclose
Tina Pasciuto: Nothing to disclose
Giacomo Avesani: Nothing to disclose
Emanuele Perrone: Nothing to disclose
Benedetta Gui: Nothing to disclose
Veronica Celli: Nothing to disclose
Silvia Bottazzi: Nothing to disclose
Evis Sala: Nothing to disclose
Anna Rame: Nothing to disclose

### Value of enhanced t1 mapping MR imaging in the evaluation of the depth of myometrial invasion in endometrial cancer: compared with dynamic contrast-enhanced MR imaging

*X. Liu*, Z. Yuan, Y. Li, J. Ren, Y. He, H. Xue, Z. Jin; Beijing/CN
*(liuxinyu960815@gmail.com)*

**Purpose or Learning Objective:** To compare the diagnostic efficiency of enhanced T1 mapping MR imaging and dynamic contrast-enhanced (DCE) imaging for assessing the depth of myometrial invasion in patients with endometrial cancer.
**Methods or Background:** 46 women diagnosed with endometrial cancer underwent preoperative MR imaging. Two radiologists independently assessed the depth of myometrial invasion, categorized as no myometrial invasion, superficial myometrial invasion, or deep myometrial invasion, on T2WI+DWI+DCE MR imaging, followed by T2WI+DWI+enhanced T1 mapping MR imaging, four weeks later. The findings were then compared to histopathological examinations. The diagnostic performance comparison was conducted using the chi-square test.
**Results or Findings:** The overall accuracy for accessing depth of myometrial invasion on T2WI+DW+DCE and T2WI+DW+enhanced T1 mapping were 76.1%, 80.4% for reader 1, and 78.3%, 80.4% for reader 2, respectively. The increment was not statistically significant for either reader. While assessing the absence of myometrial invasion, the precision, sensitivity, and specificity achieved by both radiologists using DCE were 100%, 16.7%, and 100%, whereas for enhanced T1 mapping the precisions were 100%, with sensitivities of 50% and 33%, and specificities of 100%. In evaluating superficial myometrial invasion, these values using DCE were 82.4%/82.8%, 84.8%/87.9%, and 53.8%; using enhanced T1 mapping, these values were 87.5%/85.3%, 84.8%/87.9%, and 69.2%/61.5%. Assessment of deep myometrial invasion achieved these values of 54.5%/60%, 85.7%, and 87.2%/89.7% with DCE; 54.5%/60%, 85.7%, 87.2%/89.7% with enhanced T1 mapping. Inter-reader agreement, measured with kappa values, was 0.831 with DCE-MRI and 0.899 with enhanced T1 mapping. Both radiologists concurred that enhanced T1 mapping substantially improved diagnostic confidence over DCE-MRI.
**Conclusion:** Enhanced T1 mapping demonstrates superior diagnostic efficiency in the evaluation of myometrial invasion in endometrial cancer compared with DCE MR imaging.
**Limitations:** This was a pilot study with a small sample size.
**Funding for this study:** This work was supported by grants from Natural Science Foundation of China (grant No. 82271886).
**Ethics committee - additional information:** This retrospective study was approved by the institutional review board, which waived the requirement of informed consent.

**Author Disclosures:**
Jing Ren: Nothing to disclose
Yuan Li: Nothing to disclose
Zhilin Yuan: Nothing to disclose
Zhengyu Jin: Nothing to disclose
Huadan Xue: Nothing to disclose
Yonglan He: Nothing to disclose
Xinyu Liu: Nothing to disclose

**MRI in early-stage cervical cancer after cone biopsy: can it predict the presence of residual tumor?**
*A. Amerighi*, M. Dolciami, A. Napoletano, I. Isufi, G. Avesani, E. Sala, B. Gui; Rome/IT

**Purpose or Learning Objective:** Cervical cancer (CC) is increasingly diagnosed at earlier stages due to cancer screening programs. Cone biopsy is often necessary to rule out invasive disease and can sometimes suffice for treatment; however, determining residual disease is essential for treatment planning. Despite its crucial role in managing CC, the role of Magnetic Resonance Imaging (MRI) in determining residual disease in patients after conization remains poorly understood, with only a few studies focusing on this topic. We aimed to assess MRI accuracy in detecting residual disease after cervical conization for early-stage cancer and compare the diagnostic performance of MRI with and without a contrast agent.
**Methods or Background:** We retrospectively enrolled all patients with early-stage CC who received conization before MRI and then surgically treated (hysterectomy, trachelectomy, or re-conization). Two radiologists evaluated MRI scans for residual disease in the cervix, blinded to surgical outcomes.
**Results or Findings:** 119 patients were included in the study. MRI showed an accuracy of 78,18%, sensitivity of 65.45%, specificity of 90.91%, positive predictive value (PPV) of 87.80%, and negative predictive value (NPV) of 72.46%. There was no significant change in the MRI performance with and without contrast medium, in accuracy (81,05% vs 75,80%, respectively), sensitivity (76,92% vs 55,17%), specificity (85,19% vs 96,43%), PPV (83,33% vs 94,12%), and NPV (79,31% vs 67,50%).
**Conclusion:** MRI showed good accuracy in assessing residual tumor after conization with high specificity and PPV; however, the main problem remains the high number of false negatives. The use of contrast medium did not significantly affect the MRI's performance, as it led to a slight increase in true negatives but also an increase in false positives.
**Limitations:** Retrospective study
**Funding for this study:** None
**Ethics committee - additional information:** ID prot. 7073 prot. 0022234/24
**Author Disclosures:**
Andrea Amerighi: Nothing to disclose
Giacomo Avesani: Nothing to disclose
Miriam Dolciami: Nothing to disclose
Ina Isufi: Nothing to disclose
Anna Napoletano: Nothing to disclose
Benedetta Gui: Nothing to disclose
Evis Sala: Nothing to disclose

**Diagnostic performance of DWI and ADC in characterizing the N parameter classified according to the Node-RADS score in patients with cervical cancer**
*M. Gennarini*, R. V. Ninkova, V. Miceli, A. Cupertino, S. Riccardi, F. Curti, C. Cutonilli, C. Catalano, L. Manganaro; Rome/IT
*(marco.gennarini@hotmail.it)*

**Purpose or Learning Objective:** This study evaluates the diagnostic accuracy of the Node-RADS score in Magnetic Resonance Imaging (MRI) and assesses the significance of the Apparent Diffusion Coefficient (ADC) in identifying actual lymph node neoplastic involvement in patients with cervical cancer (CC).
**Methods or Background:** A retrospective analysis was performed on cervical cancer patients who underwent preoperative MRI and radical surgery with lymphadenectomy from February 2018 to July 2024. Lymph node involvement risk was evaluated for the primary pelvic lymph nodes, assigning scores ranging from 1 to 5: 1 (very low), 2 (low), 3 (unclear), 4 (high), and 5 (very high). The mean ADC, relative ADC (rADC), and corrected ADC (cADC) for lymph nodes rated as Node-RADS 3, 4, and 5 were measured and compared against lymph node histology results.
**Results or Findings:** In this study, 156 lymph nodes from 54 patients, with a Node-RADS score greater than 2, were included. Of these, 108/156 (69.2%) were histologically confirmed as positive, while 48/156 (30.8%) were negative. The mean ADC value proved most statistically significant, showing a sensitivity of 87.0%, specificity of 82.6%, a positive predictive value (PPV) of 92.2%, and a negative predictive value (NPV) of 73.1%. The area under the curve (AUC) values for Node-RADS >2 and Node-RADS 3 were 0.892 and 0.677, respectively, with ADC thresholds of <0.963×10^-3 mm^2/s and <0.983×10^-3 mm^2/s.
**Conclusion:** The ADC measurement of lymph nodes provides crucial data that aids in the accurate classification of patients with cervical cancer. Utilizing an ADC cut-off of 0.963×10^-3 mm^2/s, the MRI demonstrated high diagnostic sensitivity. Thus, ADC serves as a valuable tool for enhancing the diagnostic process in cervical cancer management.
**Limitations:** Single center retrospective design study.
**Funding for this study:** None
**Ethics committee - additional information:** N/A
**Author Disclosures:**
Marco Gennarini: Nothing to disclose
Claudia Cutonilli: Nothing to disclose
Valentina Miceli: Nothing to disclose
Federica Curti: Nothing to disclose
Sandrine Riccardi: Nothing to disclose
Angelica Cupertino: Nothing to disclose
Roberta Valerieva Ninkova: Nothing to disclose
Carlo Catalano: Nothing to disclose
Lucia Manganaro: Nothing to disclose

**Assessing the feasibility of Magnetic Resonance Imaging Compilation for determining the treatment strategies and predicting the recurrence risk factors and short-term efficacy in cervical cancer**
*X. Ou*, Y. Li, Y. Pei, W. Li; Hunan/CN
*(2631141379@qq.com)*

**Purpose or Learning Objective:** To investigate the feasibility of MAGiC (sy-T2WI; sy-T1, sy-T2, and sy-PD maps) to determine treatment plan and predict recurrence risk factors (RRF)， short-term treatment efficacy (STE) in cervical cancer (CC) patients using high-resolution T2-weighted (hr-T2WI） and diffusion-weighted imaging (DWI) as reference standards.
**Methods or Background:** 194 patients suspected of CC were enrolled. For CC underwent CCRT, hrT2WI performed in 2 months for evaluating STE (completed response and no-CR ). MAGiC can generate synthetic morphologic images (syT2WI) and quantitative synthetic images (sy-T1, T2 and PD maps). For syT2WI, The evaluation of image quality and staging using sy-T2WI and hr-T2WI was conducted. The accuracy, sensitivity and specificity of sy-T2WI were analyzed for making treatment strategies (IB-IIA: surgery; IIB-IVA: CCRT). The AUC was used to predict RRF and STE, use quantitative sy-T1, T2, PD maps.
**Results or Findings:** 69 out of 119 CC(IIB－IVA) received CCRT . 50 out of 119 CC(IA-IIA) received surgery. SyT2WI was no significant differences with hrT2WI in four aspects (P＞0.05). The accuracy, sensitivity and specificity of sy-T2WI was 0.908, 0.908 ,0.999 for differentiating IB-IIA from IIB-IVA, and an excellent agreement between them (k = 0.935; p < 0.001). T2, T1 and ADC values had a significant differences to identified CR from no-CR and identified RRF from no-RRF (P < 0.05) but PD.The diagnostic performance of ADC was inferior to T2 for STE, which was similar to T2 for RRF. Furthermore, T1 ＋T2 was superior to ADC for predicting RRF (AUC: 0.980 vs. 0.776; p = 0.005) and forecasting STE (AUC: 0.982 vs. 0.737; p < 0.001) .
**Conclusion:** MAGiC is a promising technique for deciding therapeutic planning and predicting RRF and STE in CC , which is similar and even superior to hr-T2WI and DWI.
**Limitations:** Not applicable
**Funding for this study:** Not applicable
**Ethics committee - additional information:** the local Research Ethics Committee
**Author Disclosures:**
Wenzheng Li: Nothing to disclose
Yigang Pei: Nothing to disclose
Yue Li: Nothing to disclose
Xiaorong Ou: Nothing to disclose

**A retrospective comparative study of diagnostic algorithms for the differential diagnosis of uterine leiomyoma and sarcoma: preliminary results**
*C. Vercelli*[1], F. Rosa[2], C. Martinetti[1], D. Schettini[1], A. Gastaldo[2], N. Gandolfo[1]; [1]Genova/IT, [2]Savona/IT
*(catevercelli@gmail.com)*

**Purpose or Learning Objective:** In 2022, an important Consensus Statement and diagnostic algorithm for assessing the risk of uterine leiomyosarcoma (LMS) using MRI was published (Hindman et al.). The aim of our study is to compare the diagnostic performance of the recently proposed algorithms available in the literature for the differential diagnosis of uterine mesenchymal tumors on MRI.
**Methods or Background:** After a literature review, three diagnostic algorithms were identified: Hindman et al. (2022), Rosa et al. (2023), and Wahab et al. (2020). A retrospective evaluation was conducted on 50 MRIs performed for suspected uterine mesenchymal masses. Each lesion was categorized in a double-blind manner by an expert Radiologist in gynecological imaging according to the three algorithms. Histological examination or appropriate follow-up was used as reference standard for the final diagnosis.
**Results or Findings:** The study included 52 uterine masses: 37 benign lesions (leiomyomas) and 15 malignant lesions (7 LMS, 2 STUMP, 2 endometrial stromal sarcomas, 1 adenosarcoma, and 3 non-sarcomatous malignant lesions). The three algorithms demonstrated equal specificity in diagnosing LMS (97.3%). However, when analyzing the performance in the differential diagnosis between malignant and benign lesions (including rarer histotypes), the Rosa et al. algorithm showed superior sensitivity (93.33% vs 73.33%) and diagnostic accuracy (96.15% vs 90.38%).
**Conclusion:** The differential diagnosis of uterine mesenchymal lesions presents a diagnostic challenge with significant implications for management and outcome. The three algorithms demonstrated high diagnostic accuracy; in particular, the Rosa et al. algorithm proved more effective in identifying also rarer malignant histotypes
**Limitations:** Retrospective study; Small cohort with high prevalence of sarcomas.
**Funding for this study:** No funds
**Ethics committee - additional information:** N. Registro CER Liguria: 78/2023 - DB id 12883
N. CET - Liguria: 104/2024 - DB id 13756
**Author Disclosures:**
Nicoletta Gandolfo: Nothing to disclose
Daria Schettini: Nothing to disclose
Carola Martinetti: Nothing to disclose
Alessandro Gastaldo: Nothing to disclose
Francesca Rosa: Nothing to disclose
Caterina Vercelli: Nothing to disclose

**Clues for a new MR scoring system of uterine mesenchymal tumors**
E. Zlotykamien Taieb, *D. Gherman*; Vincennes/FR
*(dia.gherman@yahoo.com)*

**Purpose or Learning Objective:** To externally validate a previous MRI-based expert consensus algorithm and evaluate the potential improvement of an MR-scoring system's accuracy in diagnosing uterine mesenchymal tumors.
**Methods or Background:** A bicentric retrospective observational cohort study was conducted from January 2018 to December 2023 including women with a pathological diagnosis of myometrial tumor following a pelvic MRI within six months. Clinical and MR criteria were recorded blindly by two radiologists. Continuous variables were analyzed using a Mann–Whitney test, and categorical variables using Fisher's exact test. Odds ratios for predicting malignancy were calculated with 95% confidence intervals and p-values.
**Results or Findings:** The cohort included 455 women with mesenchymal tumors: 437 leiomyomas, 2 STUMPs, and 16 leiomyosarcomas. Using initial criteria (pelvic hypertrophic lymph nodes, T2W signal intensity, DW signal intensity compared to endometrium, and ADC cutoff value of $0.9 \times 10^{-3}$ $mm^2/sec$), the model accurately classified 420 out of 455 cases (Accuracy: 80.9%, sensitivity was 61.1% and specificity 93.6%. A refined approach added "irregular tumor margins" and menopausal status, modified DW signal compared to bladder, and an ADC cutoff value of $1.23 \times 10^{-3}$ $mm^2/s$, improving classification to 445 out of 455 cases (Accuracy: 92.5%; sensitivity: 83.3% ; specificity: 98.4%. The refined algorithm significantly improved accuracy, allowing the development of a 5-category scoring system.
**Conclusion:** MR imaging effectively differentiates leiomyosarcoma from other uterine tumors. The new algorithm increases diagnostic accuracy, helping prevent morcellation risks in women with uterine leiomyosarcoma
**Limitations:** Our study is retrospective, which does not permit avoidance of all biases. Even though the cohort was bicentric, the prevalence of malignancy remained low. Due to the sample size, differentiation between STUMP and frankly invasive UMT was not possible. No external validation of our modified score is yet available, necessitating further studies.
**Funding for this study:** None
**Ethics committee - additional information:** Institutional ethics committee approval and waiver of informed consent (CRM-2405-410)
**Author Disclosures:**
Diana Gherman: Nothing to disclose
Eva Zlotykamien Taieb: Author: conceiving and writing this study

**Magnetic resonance spectroscopy integration with multiparametric MRI: Enhancing diagnostic precision in sonographically indeterminate adnexal masses**
*D. Garg*, R. Kaur, R. Bedi, R. Gupta, B. Goel, U. Handa; Chandigarh/IN
*(gargdollphy@gmail.com)*

**Purpose or Learning Objective:** Proton magnetic resonance spectroscopy ($^1$H-MRS) is a non-invasive imaging technique that offers insights into biochemical metabolism. While its role in brain and prostate malignancies is well-established, its application in evaluating adnexal masses is still in its early stages. This study investigates the role of $^1$H-MRS in characterising adnexal masses and its value, in conjunction with dynamic contrast-enhanced MRI (DCE-MRI) and diffusion-weighted imaging (DWI), in enhancing the diagnostic accuracy of conventional MRI for differentiating adnexal masses.
**Methods or Background:** We conducted a prospective study including 62 histologically confirmed adnexal masses (19 benign and 43 malignant), which were indeterminate on ultrasound. Patients underwent conventional MRI, DWI (apparent diffusion coefficient), DCE-MRI (time intensity curves), and $^1$H-MRS. Single-voxel spectroscopy analysed resonance peaks for choline, N-acetyl aspartate (NAA), creatine, lactate, and lipids. Choline-to-creatine ratios were compared between benign and malignant tumours, and ROC curves were used to define optimal thresholds.
**Results or Findings:** Conventional MRI showed sensitivity, specificity, positive predictive value (PPV), and negative predictive value (NPV) of 97.67%, 57.89%, 84%, and 91.67%, respectively. We detected choline peak in 100% of malignant and 47.4% of benign masses, NAA in 79.1% of malignant and 31.6% of benign masses, as well as lipid peaks in 36.8% of benign and 20.9% of malignant masses. The mean choline-to-creatine ratio is 1.05+0.55 in benign and 12.18+12.38 in malignant tumours, statistically significant ($p<0.05$). With a choline-to-creatine threshold of 2.01, sensitivity, specificity of 100% is achieved. The addition of $^1$H-MRS, DWI, and DCE-MRI improved the diagnostic accuracy of conventional MRI, with 100% sensitivity, 94.74% specificity, 97.73% PPV, and 100% NPV.
**Conclusion:** 1H-MRS has promising role in characterising adnexal masses and in conjunction with DWI and DCE-MRI, enhances the diagnostic accuracy of conventional MRI.
**Limitations:** The sample size is limited.
**Funding for this study:** No funding was provided for this study.
**Ethics committee - additional information:** The ethics committee notification can be found under the number ECR/658/Inst/PB/2014/RR-20
**Author Disclosures:**
Uma Handa: Nothing to disclose
Rekha Gupta: Nothing to disclose
Raveena Bedi: Nothing to disclose
Dollphy Garg: Nothing to disclose
Ravinder Kaur: Nothing to disclose
Bharti Goel: Nothing to disclose

**11:30-12:30** **Research Stage 1**

### Research Presentation Session: Abdominal and Gastrointestinal

### RPS 2401

### Acute abdominal diseases and imaging of the bowel

**Moderator**
M. Zins; Paris/FR
*(mzins@hpsj.fr)*

**0.5-mSv ultra-low dose appendiceal CT using deep learning–based denoising algorithm: a comparison with conventional 2.0-mSv low dose CT**
*B. J. Choi*, W. Chang, J. H. Hwang, J. Cho, Y. J. Lee, Y. H. Kim, S. H. Park, J. Y. Choi; Seongnam-si/KR

**Purpose or Learning Objective:** To demonstrate that 0.5-mSv ultra-low dose CT using deep learning–based denoising algorithm (DLA) has non-inferiority in diagnosing acute appendicitis, compared to conventional 2.0-mSv low dose CT.
**Methods or Background:** We used 2.0-mSv CT images of 30 patients with suspected appendicitis from the prior prospective study. The original 2.0-mSv CT were reconstructed using iterative model reconstruction (IMR). We simulated 0.5-mSv CT images from the original 2.0-mSv CT. Then we applied IMR and DLA, resulting in three CT image groups per patient (IMR 2.0-mSv, IMR 0.5-mSv, and DLA 0.5-mSv groups). Six radiologists (three abdominal and three non-abdominal radiologists) rated the likelihood of appendicitis on a five-point Likert scale. Primary end point was comparison of the pooled area under the receiver operating characteristic curve (AUC) between DLA 0.5-mSv CT and IMR 2.0-mSv CT, with a non-inferiority margin of 0.06. Secondary end points included comparison of AUC between DLA 0.5-mSv CT and IMR 0.5-mSv CT, diagnostic sensitivity/specificity.
**Results or Findings:** The AUC of DLA 0.5-mSv CT was non-inferior to that of IMR 2.0-mSv CT [AUC difference: 0.003 (95% CI: -0.011, 0.017)]. The AUC of

DLA 0.5-mSv CT was slightly higher among the non-abdominal radiologists compared to IMR 0.5-mSv CT [AUC difference: 0.034 (95% CI: -0.130, 0.198)]. Diagnostic sensitivity/specificity were 100% (9/9)/95% (20/21) for all readers with both DLA 0.5-mSv CT and IMR 2.0-mSv CT. However, the sensitivities of two non-abdominal radiologists with IMR 0.5-mSv CT were mildly compromised [78% (7/9) and 89% (8/9), respectively].
**Conclusion:** 0.5-mSv ultra-low dose CT using DLA was non-inferior to conventional 2.0-mSv low dose CT using IMR in diagnosing acute appendicitis.
**Limitations:** The limitations of the study are 1) 0.5-mSv CT images were simulations, not real data and 2) small sample size, collected from a single tertiary hospital.
**Funding for this study:** Funding was provided by the National Research Foundation of Korea(NRF) grant funded by the Korea government(MSIT) (NRF-2022R1F1A1072570).
**Ethics committee - additional information:** The institutional review board approved this study, and the requirement for informed consent was waived (B-2401-879-112).
**Author Disclosures:**
So Hyun Park: Nothing to disclose
Jungheum Cho: Nothing to disclose
Ji Young Choi: Nothing to disclose
Yoon Jin Lee: Nothing to disclose
Won Chang: Nothing to disclose
Young Hoon Kim: Nothing to disclose
Byung Jin Choi: Nothing to disclose
Jin Hee Hwang: Nothing to disclose

### Upright CT vs. Supine CT: Diagnostic Capabilities for Inguinal Hernias and Subtypes in Emergency Department

*T. Yoshikawa*, H. Nagata, T. Ueda, M. Nomura, D. Takenaka, Y. Ozawa, Y. Ohno; Toyoake/JP
*(yoshikawa0816@aol.com)*

**Purpose or Learning Objective:** Upright CT is recently and clinically set in our institution, and the purpose of this study was to directly compare capabilities for diagnosis and subtype classification of inguinal herniation between upright CT (uCT) and conventional supine CT (sCT).
**Methods or Background:** 258 consecutive patients who suspected inguinal hernia underwent sCT and uCT within a week, surgical treatment or follow-up examination. From this cohort, 120 inguinal hernias and computationally selected 120 out of 396 non-inguinal hernia were visually assessed by two board certified general and abdominal radiologists by 5-point scales as well as subtypes of hernia. Inter-observer agreements for probability of hernia and subtype were assessed by kappa statistics with $\chi 2$ test. Then, ROC analysis was performed to compare diagnostic performance between two CTs. Then, sensitivity (SE), specificity (SP) and accuracy (AC) were compared each other by McNemar's test. Moreover, subtype classification accuracy (SAC) was also compared between uCT and sCT by McNemar's test.
**Results or Findings:** Inter-observer agreement for probability of hernia were determined as significant and almost perfect on uCT (κ=0.84, p<0.0001) and substantial on sCT (κ=0.77, p<0.0001), and that for subtype classification were also significant and almost perfect on both CTs (uCT: κ=0.83, p<0.0001; sCT: κ=0.81, p<0.0001). Area under the curve (AUC) of uCT (AUC=0.99) were significantly larger than that of sCT (AUC=0.97, p<0.05). SE and AC of uCT (SE=92.5%, AC=96.3%) were significantly higher than those of sCT (SE: 80.8%, p<0.0001; AC: 90.4%, p<0.0001). SAC of uCT (87.5%) was significantly higher than that of sCT (73.3%, p<0.0001).
**Conclusion:** Upright CT has better diagnostic performance for inguinal hernia and subtype classification than conventional supine CT in routine clinical practice.
**Limitations:** Lack of clinical outcome evaluation
**Funding for this study:** Research grant from Canon Medical Systems Corporation
**Ethics committee - additional information:** Fujita Health University Hospital
**Author Disclosures:**
Yoshiyuki Ozawa: Research/Grant Support: Grant-in-Aid for Scientific Research from the Japanese Ministry of Education, Culture, Sports, Science and Technology Research/Grant Support: Smoking Research Foundation
Masahiko Nomura: Nothing to disclose
Takahiro Ueda: Research/Grant Support: Grant-in-Aid for Scientific Research from the Japanese Ministry of Education, Culture, Sports, Science and Technology
Daisuke Takenaka: Nothing to disclose
Hiroyuki Nagata: Research/Grant Support: Grants-in-Aid for Scientific Research from the Japanese Ministry of Education, Culture, Sports, Science and Technology Research/Grant Support: Canon Medical Systems Corporation
Takeshi Yoshikawa: Nothing to disclose
Yoshiharu Ohno: Research/Grant Support: Smoking Research Foundation Research/Grant Support: Canon Medical Systems Corporation

### Dual-Energy CT of Gastrointestinal Bleeding - Influence on diagnostic accuracy and reader confidence

*M. Oberparleiter*, H-C. Breit, J. Vosshenrich, P. Hehenkamp, A. C. Seifert, A. Kobe, C. J. Zech, M. Obmann; Basel/CH

**Purpose or Learning Objective:** Current guidelines suggest replacing unenhanced images with DECT-derived virtual non-contrast images (VNC) in suspected upper GI bleeding based on only two clinical studies. Our study compares diagnostic accuracy, reader confidence, and reading time of a conventional triphasic versus a dual-energy CT protocol in patients with upper and lower GI bleeding.
**Methods or Background:** This retrospective study included 52 patients with active GI bleeding (22 upper, 30 lower) and 52 controls who underwent non-contrast, arterial, and portal-venous phase abdominal CT. For each case, a triphasic conventional CT dataset and a DECT dataset with VNC, iodine images, and arterial and portal venous phase images were created. Two residents and two fellowship-trained abdominal radiologists evaluated all cases for active GI bleeding. Radiation dose and reading time were recorded. Diagnostic confidence was rated on a 5-point Likert scale. Inter-reader agreement was assessed using Fleiss' kappa. Sensitivity and specificity were compared using McNemar's test, reading time, and reader confidence with the Wilcoxon signed-rank test.
**Results or Findings:** Inter-reader agreement was substantial ($\kappa$=0.80). Sensitivity and specificity for detecting GI bleeding using conventional CT did not differ from DECT (91% and 95%, vs. 93% and 96%, p=0.30 and p=0.77, respectively). Subgroup analysis of lower GI bleeding showed a sensitivity of 88% in conventional CT versus 93% in DECT (p=0.18). Diagnostic confidence increased from 4(IQR, 4-5) to 5(IQR, 4-5) when using DECT (p<0.01). Mean reading time per case was 102 s for both datasets (p=0.62). Total DLP without true unenhanced images was 21% lower.
**Conclusion:** DECT-derived VNC and iodine images can replace true non-contrast images when searching for GI-bleeding. Guidelines should be extended to include lower-GI-bleeding.
**Limitations:** Sample size was moderate, the study had a single-center design, and only dual-source and split-beam DECT scanners were used.
**Funding for this study:** This research received no specific grant from any funding agency in the public, commercial, or not-for-profit sectors.
**Ethics committee - additional information:** The need for informed consent was waived due to the retrospective nature of this study.
**Author Disclosures:**
Markus Obmann: Nothing to disclose
Hanns-Christian Breit: Nothing to disclose
Christoph Johannes Zech: Nothing to disclose
Adrian Kobe: Nothing to disclose
Paul Hehenkamp: Nothing to disclose
Jan Vosshenrich: Nothing to disclose
Alina Carolin Seifert: Nothing to disclose
Moritz Oberparleiter: Nothing to disclose

### Correlation of CT-derived Quantitative Image Features and Inflammatory Laboratory in Pyelonephritis

*A. W. Marka*, M. Graf, S. Ziegelmayer, M. R. Makowski, A. Sauter, T. Huber; Munich/DE
*(alexander.marka@gmail.com)*

**Purpose or Learning Objective:** To investigate the relationship between inflammatory laboratory markers and quantitative CT-derived imaging features in patients with acute pyelonephritis (APN).
**Methods or Background:** In this single-center retrospective study, we evaluated patients with clinical symptoms of APN at our institution from December 2018 to April 2024. Inclusion criteria comprised APN symptoms, elevated inflammatory markers (CRP and/or WBC), and CT morphologic signs of APN. Exclusion criteria included concomitant acute pathology and poor image quality. A total of 102 patients (mean age 60.1±18.8) were initially identified; 14 were excluded due to acute pathology and 5 due to poor image quality, leaving 83 for final analysis. CT scans followed a standardized protocol, and two radiologists blinded to clinical and lab data conducted image analysis. Inflammatory markers were collected on the scan day. Statistical analyses included Spearman correlation, Mann-Whitney-U tests, and linear regression.
**Results or Findings:** Spearman correlation analysis revealed strong positive correlations between CRP levels and both total volume (r=0.76, p<0.001) and percentage (r=0.71, p<0.001) of renal perfusion deficit. Multivariate linear regression showed total perfusion deficit volume explained 54.5% of CRP variability (p<0.001). WBC count also correlated significantly with total volume (r=0.379, p<0.001) and percentage (r=0.374, p<0.001) of perfusion deficit. Procalcitonin levels moderately correlated with fat stranding area (r=0.482, p=0.0014) but not other CT features. Locoregional lymphadenopathy was significantly associated with elevated CRP and WBC counts, but not procalcitonin levels.

**Conclusion:** Quantitative CT-derived features, particularly renal perfusion deficits, are significantly associated with inflammatory markers in APN. These findings suggest CT imaging can serve as a surrogate for inflammation severity, potentially guiding clinical management. Further research is needed to explore the clinical implications of these associations.
**Limitations:** -Small cohort -Scarcity of procalcitonin levels -No correlation with duration of a patient's hospitalization
**Funding for this study:** None
**Ethics committee - additional information:** Data collection, processing, and analysis were approved by the institutional review board (protocol number 180/17S), and informed consent was waived.
**Author Disclosures:**
Andreas Sauter: Nothing to disclose
Markus Graf: Nothing to disclose
Thomas Huber: Nothing to disclose
Marcus R. Makowski: Nothing to disclose
Alexander Wolfgang Marka: Nothing to disclose
Sebastian Ziegelmayer: Nothing to disclose

### Fast abdominopelvic T2-weighted imaging with deep learning reconstruction for acute abdomen: feasibility study

J. Xu, L. Zhu, W. Liu, *Y. Lu*, J. Liu, C. Ma, Y. Zhang, X. Wang, F. Feng; Beijing/CN
*(lytong9815@163.com)*

**Purpose or Learning Objective:** To evaluate the image quality and diagnostic performance of single-shot fast spin-echo T2 weighted imaging with deep learning reconstruction (SSFSE-DL) in volunteers and patients with acute abdomen, in comparison to SSFSE without deep learning reconstruction (SSFSE-nonDL) and conventional PROPELLER sequences.
**Methods or Background:** Thirty-five healthy volunteers, as well as 35 patients with acute abdominal pain from emergency room were prospectively enrolled. Abdominopelvic MRI at 3T was performed using three T2-weighted imaging sequences: SSFSE-DL (acquisition time: 34s), SSFSE-nonDL, and conventional PROPELLER (acquisition time: 2-3min), in random order. Two blinded radiologists independently evaluated the overall image quality, noise, motion artifacts and clarity of major abdominopelvic organs. Diagnostic confidence for the presence or absence of common abdominopelvic diseases was rated on a 1-5 Likert scale. Signal-to-noise ratio (SNR), contrast-to-noise ratio (CNR) as well as image noise for the liver, pancreas and spleen were also quantified. Intra- and inter- observer agreement were assessed, and comparisons of image quality and diagnostic performance between the three sequences were made.
**Results or Findings:** Intra- and inter- observer agreement for the qualitative analysis and diagnostic performance were good to excellent (0.776-0.967). SSFSE-DL yielded significant higher SNR and CNR, and lower noise than SSFSE-nonDL and PROPELLER in both volunteers and patients (all $P<0.05$). SSFSE-DL obtained significantly higher image quality and lower noise than SSFSE-nonDL and PROPELLER (both $P<0.05$). SSFSE-DL and SSFSE-nonDL had significantly lower motion artifacts and better clarity of major abdominopelvic organs than PROPELLER (both $P<0.05$). The AUC for detecting common abdominopelvic diseases in SSFSE-DL (0.977-1) and SSFSE-nonDL (0.887-1) were significantly higher than PROPELLER (0.585-0.953).
**Conclusion:** SSFSE-DL achieved superior image quality and diagnosis performance for volunteers and patients with acute abdomen.
**Limitations:** The number of patients with positive diagnosis for each specific disease was relatively small.
**Funding for this study:** This study was funded by the National Natural Science Foundation of China (82371950)
**Ethics committee - additional information:** This prospective single-center study was approved by the local institutional review board, and informed consent was obtained from all participants prior to inclusion in the study.
**Author Disclosures:**
Wei Liu: Nothing to disclose
Feng Feng: Nothing to disclose
Yitong Lu: Nothing to disclose
Chenxue Ma: Nothing to disclose
Xuan Wang: Nothing to disclose
Jia Xu: Nothing to disclose
Liang Zhu: Nothing to disclose
Jingjuan Liu: Nothing to disclose
Yifei Zhang: Nothing to disclose

### Diagnostic performance of low-dose abdominal CT with artificial intelligence iterative reconstruction for acute pancreatitis

*X. Zhang*[1], S. Zhong[2], G. Zhang[2], X. Zhou[1]; [1]Chongqing/CN, [2]Shanghai/CN
*(db421521@163.com)*

**Purpose or Learning Objective:** To characterize the diagnostic performance of low-dose (LD) abdominal CT combined with artificial intelligence iterative reconstruction (AIIR) for assessing acute pancreatitis based on CT severity index (CTSI).
**Methods or Background:** A total of 30 patients with acute pancreatitis who underwent follow-up CT examination were prospectively enrolled. All patients underwent standard-dose (SD) CT followed by LD-CT in the same breath hold, where an immediate LD-scan was added in the portal venous phase. The SD-protocol was 120 kVp, ref. 141 mAs, and hybrid iterative reconstruction (HIR), whereas the LD-protocol was 120kVp, ref. 50mAs and AIIR reconstruction. To obtain the CTSI, SD- and LD-CT images at portal venous phase were independently scored by two radiologists for assessing pancreatic inflammation, necrosis, and extrapancreatic complications. Signal-to-noise ratio (SNR) and contrast-to-noise ratio (CNR) of healthy and inflamed parenchyma were measured and calculated.
**Results or Findings:** Compared to SD-CT, LD-CT examination achieved a 65.18% reduction in effective radiation dose for the portal venous phase (6.06 ± 1.28 mSv vs. 2.11 ± 0.45 mSv, $p < 0.05$). Based on CTSI scoring, LD-AIIR was found comparable to SD-HIR in evaluating the severity of acute pancreatitis (5.02 ± 1.42 vs. 5.11 ± 1.78, $p = 0.86$). Inter-observer agreement for assessing the severity of acute pancreatitis was excellent (k = 0.89). LD-AIIR showed superior conspicuity compared to SD-HIR for both the inflamed (SNR: 1.96 ± 0.83 vs. 1.74 ± 0.98; CNR: 4.17 ± 1.58 vs. 2.11 ± 0.87; both $p < 0.05$) and the healthy parenchyma (SNR: 8.03 ± 1.71 vs. 4.91 ± 1.04; CNR: 3.06 ± 1.84 vs. 1.78 ± 1.03; both $p < 0.05$).
**Conclusion:** AIIR allows for significant radiation dose reduction without compromising image quality or diagnostic performance for the evaluation of acute pancreatitis.
**Limitations:** n/a
**Funding for this study:** n/a
**Ethics committee - additional information:** Ethics committee of Chongqing University Jiangjin Hospital
**Author Disclosures:**
Xue Zhou: Nothing to disclose
Xiufu Zhang: Nothing to disclose
Guozhi Zhang: Nothing to disclose
Sihua Zhong: Nothing to disclose

### SBOM-AI TRIAL: Set up and validation of AI-based automatic total Small Bowel length Measurement using CT and MRI in Obese patients candidates for metabolic surgery

*M. Zerunian*[1], S. Nardacci[1], N. Petrucciani[1], I. Toniolo[2], D. De Santis[1], D. Caruso[1], C. G. Fontanella[2], G. Silecchia[1], A. Laghi[1]; [1]Rome/IT, [2]Padova/IT
*(marta.zerunian@gmail.com)*

**Purpose or Learning Objective:** Total small bowel length(TSBL) is crucial to achieve successful metabolic/bariatric surgery. A non-invasive measurement of the TSBL will impact on surgical strategy to avoid short-bowel syndrome after surgery. Cross-sectional imaging(CSI) might play an important role by measuring TSBL non-invasively. We aimed to set up a reliable AI-based automatic method using preoperative CSI to measure the TSBL in candidate to bariatric/metabolic surgery.
**Methods or Background:** This multicentre prospective TRIAL included patients eligible for bariatric surgery(BMI >35 kg/m2 and at least one obesity-related comorbidity,BMI>40 kg/m2) underwent the same day MRI-and CT-enterography before bariatric surgery. TSLB assessed right before the surgery at the operation table and, TSLB <250 cm considered the cut-off as risk for developing short-bowel syndrome. TSLB obtained on MRI and CT by manual segmentation(Slicer3D). A Convolutional Neural Network with U-NET developed, consisting in a contracting path followed by an expansive path, RELU activation and a softmax activation function to reduce the feature map. The Adam optimization algorithm with a constant learning rate was used for the learning process. Training stopped after 100 epochs. DICE coefficient were calculated to quantify the accuracy of the prediction compared to annotated-by-radiologist images.
**Results or Findings:** Fifty patients enrolled (27 female,age range 26-54 years old,mean BMI 41.47). Patients underwent surgery showed a TSBL mean measured intraoperatively of 652.85±87.37cm. CSI showed good concordance with TSBL mean of 589.08±82.95cm(k= 0.70).DICE coefficient of the training set showed a DICE score ranging between 35%-45%, confirmed in the validation set. All methods correctly categorized the patients according to the cut-off considered as risk factor to develop short-bowel syndrome.
**Conclusion:** Automatic AI-based segmentation of small bowel on non-invasive CSI might be a useful tool to assess obese patients to personalize the treatment and reduce complications.
**Limitations:** Small sample size

**Funding for this study:** Italian Ministry of University and Research (MUR) Research Projects of Significant National Interest – PRIN (ID: MUR 2022MPAE29_003)
**Ethics committee - additional information:** Multicenter interventional study
**Author Disclosures:**
Niccolò Petrucciani: Nothing to disclose
Damiano Caruso: Nothing to disclose
Marta Zerunian: Nothing to disclose
Domenico De Santis: Nothing to disclose
Ilaria Toniolo: Nothing to disclose
Gianfranco Silecchia: Nothing to disclose
Andrea Laghi: Nothing to disclose
Chiara Giulia Fontanella: Nothing to disclose
Stefano Nardacci: Nothing to disclose

**Vascular enhancement in single-pass abdominal CT: Effects of a fixed injection duration in patients with non-traumatic acute abdomen**
*A. Stanzione*, V. Arpaia, A. E. Antonini, R. Liuzzi, L. Sommella, L. Mannacio, A. Brunetti, L. Camera; Naples/IT
*(arnaldostanzione@yahoo.it)*

**Purpose or Learning Objective:** To evaluate the effects of a fixed injection duration (FID) on vascular enhancement in a Single-Pass (SP) abdominal CT performed in patients with nontraumatic acute abdomen (ANTA).
**Methods or Background:** 100 patients (58M, 42F; aged 52±20 yrs ) with ANTA underwent a SP contrast-enhanced CT (Somatom Drive, Siemens) performed using a Single Source at either 80 kVp (Group A; BMI 19±3), 100 Kvp (Group B; BMI 25±4) or 120 Kvp (Group C; BMI 30±2.5) . In all groups a non-ionic iodinated contrast media (370 mgI/ml) was administered as follows: Group A (0.37 grI/Kg); Group B (0.52 grI/Kg); Group C (0.63 grI/Kg). All patients underwent a SP protocol with a FID (50 sec) and a tailored scan delay (SD). In all patients Signal- (SNR) and Contrast-to-Noise Ratios (CNR) were calculated for the abdominal aorta (AA) and the main portal vein (MPV) using the psoas muscles as reference tissue. Statistical analysis was performed with ANOVA ($p < 0.05$).
**Results or Findings:** No significant differences were observed in the demographics of either Group A (20M/12F; 47±20 yrs) or B (36M/22F; 54±19 yrs) whereas a female preponderance was observed in Group C (2M/8F; 50±18 yrs). Despite significant differences ($p < 0.001$) were observed in both the volumes (53±11 vs 103±17 vs 141±19 ml) as well as the injection rates (1.1±0.2 vs 2.1±0.4 vs 2.8±0.4 ml/sec) of the contrast media for Group A, B and C, respectively, SNR and CNR were not significantly different for both AA and MPV.
**Conclusion:** SP performed with a FID results in a consistent vascular enhancement.
**Limitations:** Unbalanced sample size
**Funding for this study:** None
**Ethics committee - additional information:** Local IRB approval
**Author Disclosures:**
Luigi Mannacio: Nothing to disclose
Laura Sommella: Nothing to disclose
Valerio Arpaia: Nothing to disclose
Luigi Camera: Nothing to disclose
Raffaele Liuzzi: Nothing to disclose
Andrea Ennio Antonini: Nothing to disclose
Arnaldo Stanzione: Nothing to disclose
Arturo Brunetti: Nothing to disclose

**11:30-12:30** **Research Stage 2**

### Research Presentation Session: Cardiac

### RPS 2403

### Cardiac imaging: insights from trials

**Moderator**
N. Lama; Athens/GR
*(niklampatr@gmail.com)*

**Association between metabolic phenotypes and MRI-derived cardiac function parameters, major adverse cardiovascular events, and mortality: Insights from the UK Biobank**
*B. Bogner*, M. Jung, M. Reisert, J. Maushagen, S. Rospleszcz, C. L. Schlett, F. Bamberg, J. Weiß, J. Taron; Freiburg/DE

**Purpose or Learning Objective:** To evaluate the association between metabolic phenotypes, MRI-derived cardiac function, major adverse cardiovascular events (MACE), and all-cause mortality.
**Methods or Background:** We analyzed 22,348 UK Biobank (UKBB) participants who underwent cardiac MRI. Obesity was defined as BMI ≥30kg/m²; metabolically unhealthy as the presence of >=1 metabolic syndrome components or diabetes resulting in four phenotypes: metabolically healthy non-obese (MHN), metabolically healthy obese (MHO), metabolically unhealthy non-obese (MUN), metabolically unhealthy obese (MUO). Associations between MRI-derived left ventricular ejection fraction (LVEF, %), cardiac index (cardiac output/body surface area [L/min/m2]), MACE, and all-cause mortality were investigated using uni-/multivariable linear and Cox regression analyses adjusted for age, sex, and smoking status.
**Results or Findings:** Among 22,348 UKBB participants (mean age 64.1±7.5 years, 48.8% male), 45.4% were MHN, 6.0% MHO, 36.5% MUN and 12.1% MUO. Over a median follow-up of 5.2 years, 52 MACE events and 371 deaths occurred. Compared to MHN, both obese phenotypes showed a significant negative association with LVEF whereas a positive association was found for MUN ($p<=0.02$). For cardiac index, only MUO showed a significant positive association compared to MHN ($p=0.03$). Cox regression revealed a >5-fold higher risk of MACE for both obese phenotypes (MHO (HR 5.53 [95% CI 2.10-14.58], $p<0.001$; MUO (HR 5.24 [95% CI 2.37-11.57], $p<0.001$) compared to MHN. A similar pattern was seen for all-cause mortality (MHO (1.57 [1.04, 2.36], $p=0.03$; MUO (1.61 [1.20, 2.17]; $p=0.001$).
**Conclusion:** Alterations in metabolic health are linked to differences in cardiac function, MACE and all-cause mortality risk independent of age, sex, and smoking. This suggests distinct mechanisms affecting cardiac health and emphasizes the need to consider both metabolic health and obesity for personalized risk assessment.
**Limitations:** Findings may not be generalizable to non-UK populations or ethnic groups underrepresented in the UKBB.
**Funding for this study:** None
**Ethics committee - additional information:** Approved by the UK Biobank.
**Author Disclosures:**
Susanne Rospleszcz: Nothing to disclose
Christopher L. Schlett: Nothing to disclose
Marco Reisert: Nothing to disclose
Jakob Weiß: Nothing to disclose
Balazs Bogner: Nothing to disclose
Matthias Jung: Nothing to disclose
Jana Taron: Nothing to disclose
Juliane Maushagen: Nothing to disclose
Fabian Bamberg: Nothing to disclose

**The aging heart: Associations between cardiac structure, function, and demographic factors in a population-based study**
*B. J. Kerber*[1], T. Küstner[2], S. Gatidis[3]; [1]Zurich/CH, [2]Tübingen/DE, [3]Stanford, CA/US

**Purpose or Learning Objective:** Exploring the relationship between demographic factors and quantitative imaging phenotypes based on cardiac MR (CMR) in the NAKO population study to provide insights into the aging heart and advance the understanding of cardiovascular disease.
**Methods or Background:** Steady-state free precession short-axis CMR full-cycle sequences from 29,104 participants of the NAKO study, aged 19 to 74 years (16,201 male and 12,903 female, aged 47.9 +/- 12.4, resp. 48.7 +/- 12.2 years), were analyzed. A custom-trained nnU-Net model was used to segment images into left ventricle (LV), leftventricular myocardium, and right ventricle (RV). From these segmentations, key cardiac metrics including LV mass (LVM), LV and RV end-diastolic and end-systolic volumes (LVEDV/RVEDV/LVESV/RVESV), ejection fractions (LVEF/RVEF), stroke volumes (LVSV/RVSV) and cardiac output (LVCO) were calculated. Correlation analysis and group comparisons were performed to examine associations between the participants' demographic and quantitative imaging phenotypes.
**Results or Findings:** In this study, LVEDV/RVEDV (r=-0.22/r=-0.23), LVESV/RVESV (r=-0.17/r=-0.19), LVSV/RVSV, (r=-0.21/r=-0.20) and LVCO (r=-0.19) significantly decreased with age, with males showing a higher baseline and steeper decline. LVM and LVEF were nearly stable (r=0.01/r=0.01). LVEF was consistently higher in females (p<0.001). RVEF increased with age for females and decreased for males (r=0.07, r=-0.02). Hypertension, diabetes, smoking and high blood lipids were associated with significantly higher LVM and lower LVEF/RVEF (p<0.001), while high HDL was negatively correlated with LVM (r=-0.36), LVEDV/RVEDV (r=-0.20/r=-0.22) and positively with LVEF/RVEF (r=0.08/r=0.14).
**Conclusion:** A CMR full-cycle segmentation model was developed and applied to a large population study spanning a broad age range. The resulting imaging phenotypes revealed strong associations with age, disease, and demographic factors.
**Limitations:** The analysis was performed automatically using only basic quality control.
**Funding for this study:** No specific funding.
**Ethics committee - additional information:** The study protocol, participant information, and consent forms for the NAKO study were reviewed by all local ethics committees of the participating institutions.
**Author Disclosures:**
Sergios Gatidis: Nothing to disclose
Bjarne Jonas Kerber: Nothing to disclose
Thomas Küstner: Nothing to disclose

### Variability of coronary artery calcium score in the multicentre DISCHARGE trial: Agreement between readings at the core laboratory and the clinical centres

*F. Biavati*[1], M. Mohamed[1], S. Tsogias[1], B. Föllmer[1], M. Bosserdt[1], J. Dodd[2], M. Dewey[1]; [1]Berlin/DE, [2]Dublin/IE

**Purpose or Learning Objective:** To assess the agreement between core laboratory and the clinical centre measurement of coronary artery calcium (CAC) score using the Agatston method.
**Methods or Background:** At each of the 26 clinical centres across 16 European countries [NCT02400229], radiologists measured the patients' CAC score based on noncontrast coronary computed tomography (CT). Two readers measured the CAC score at the core laboratory blinded to the clinical centre reading. Bland-Altman analysis of the CAC scores values and Cohen's kappa of the CAC score risk categories (I: 0, II: 1-400, III: >400) was performed.
**Results or Findings:** 1550 patients (mean age, 59 years ± 10 [SD], 56.3% women) were included. The Bland-Altman analysis showed a mean absolute difference of 2.0 and limits of agreement of ± 93.3 between the core laboratory and the clinical centres. There was agreement in the CAC score risk categories in 96.6% (1498 of 1550) of patients between the core laboratory and the clinical centre reading. Discrepancies between CAC score risk categories occurred mostly between categories I and II (88.5%, 46 of 52). Agreement according to Cohen's kappa was excellent (0.94, 95% CI: 0.93, 0.96; p<.001). Most disagreements in the assignment of risk categories were between category I and II (88.5%).
**Conclusion:** CAC score measurements had good agreement between the core laboratory and clinical centres in a Pan-European multicentre trial suggesting that CAC score measurements can be widely implemented as part of cardiac CT based on its reproducibility.
**Limitations:** Our study has limitations. It included only stable chest pain patients. The population was from 26 European centres, limiting global applicability. Some CT scans were excluded due to missing data or non-matching reconstruction parameters, and only filtered back projection reconstruction was used.
**Funding for this study:** Funding was provided by grants from the EU-FP7 Framework Program (FP 2007-2013, EC-GA 603266).
**Ethics committee - additional information:** The study was approved by The German Federal Office for Radiation Protection and the local or national authorities at each trial site. The reference number is: EA1/294/13.
**Author Disclosures:**
Jonathan Dodd: Author: Receives royalties as a co-author of book chapters in the Stat-Dx book Series Diagnostic Imaging – Cardiovascular and the textbook CT and MRI in Cardiology, Elsevier. Other: Associate Editor for Radiology, a member of the Editorial Board for Radiology Cardiothoracic Imaging, and an Associate Editor for the Quarterly Journal of Medicine. All non-paid. Grant Recipient: Received funding from EU-FP7 Framework Program (DISCHARGE EU FP EC-GA 603266).
Mahmoud Mohamed: Nothing to disclose
Marc Dewey: Board Member: M.D. is European Society of Radiology (ESR) Publications Chair (2022-2025); the opinions expressed in this presentation are the author's own and do not represent the view of ESR. Other: Hands-on cardiac CT courses (www.ct-kurs.de) Author: Cardiac CT (Springer Nature). Grant Recipient: EU (EC-GA 603266 in HEALTH.2013.2.4.2-2) DFG (DE 1361/14-1, DE 1361/18-1, BIOQIC GRK 2260/1, Radiomics DE 1361/19-1 [428222922] and 20-1 [428223139] in SPP 2177/1), GUIDE-IT (DE 1361/24-1), Berlin University Alliance (GC_SC_PC 27), G-BA (01NVF23002), Berlin Institute of Health (Digital Health Accelerator). Research/Grant Support: Siemens, General Electric, Philips, Canon. Patent Holder: Patent on fractal analysis of perfusion imaging (jointly with Florian Michallek, EPO 2022 EP3350773A1, and USPTO 2021 10,991,109, approved).
Sotirios Tsogias: Nothing to disclose
Federico Biavati: Nothing to disclose
Maria Bosserdt: Nothing to disclose
Bernhard Föllmer: Nothing to disclose

### Patient Acceptance of Coronary CT Angiography Versus Invasive Coronary Angiography in Patients with Stable Chest Pain

M. Bosserdt, *K. Schulze*, M. Mohamed, A-M. Stantien, M. Dewey, E. Schöneberger; Berlin/DE

**Purpose or Learning Objective:** Patient preference between coronary computed tomography (CT) and invasive coronary angiography (ICA) in a multicentre analysis in Europe is unknown. Therefore, we compare patient preference for CT and ICA in a European multicentre randomised controlled trial.
**Methods or Background:** A total of 3561 patients with a clinical indication for ICA with stable chest pain and an intermediate likelihood of obstructive coronary artery disease were analysed in this prespecified secondary analysis from the randomised DISCHARGE trial (NCT02400229) conducted between October 2015 and April 2019 in 26 European centres. Patient preference using a previously validated questionnaire completed at least 24 hours after CT or ICA, including preparation for the tests, anxiety, comfort, level of helplessness, pain, willingness to undergo the tests again, overall satisfaction and preference.
**Results or Findings:** The questionnaire was completed by 89.7% in the CT group (1622/1808) and 89.4% (1567/1753, P=.75) in the ICA group. Patients reported significantly higher satisfaction with CT (mean (SD): CT: 1.37 (0.53) vs. 1.48 (0.60); score: 1-5; P<.0001), were more willing to undergo CT again (mean (SD): CT: 1404/1622 (86.6%) vs. ICA: 1127/1567 (71.9%); P<.0001), and were better prepared to CT (mean (SD): CT: 1.40 (0.56) vs. 1.49 (0.64); score: 1-5; P=.0005). They were less anxious before CT (mean (SD): CT: 1.07 (1.17) vs. ICA: 1.62 (1.33); score: 1-4; P<.0001), felt less helpless during CT (mean (SD): CT: 0.54 (0.72) vs. ICA: 0.91 (0.90); score: 0-4; P<.0001), and felt more comfortable (mean(SD): CT: 1.64 [0.66] vs. ICA: 1.90 [0.80]; score: 1-5; P<.0001).
**Conclusion:** In this multicentre, randomised trial of patients referred for ICA with stable chest pain and an intermediate likelihood of obstructive coronary artery disease, patient preference was in favour of coronary CT angiography.
**Limitations:** Not applicable.
**Funding for this study:** This study was funded by grants from the EU-FP7 Framework Program (FP 2007-2013, EC-GA 603266).
**Ethics committee - additional information:** The study was approved by ethics committee at Charité (EA1/294/13).
**Author Disclosures:**
Kenrick Schulze: Nothing to disclose
Mahmoud Mohamed: Nothing to disclose
Eva Schöneberger: Nothing to disclose
Anne-Marieke Stantien: Nothing to disclose
Marc Dewey: Board Member: M.D. is European Society of Radiology (ESR) Publications Chair (2022-2025); the opinions expressed in this presentation are the author's own and do not represent the view of ESR. Grant Recipient: EU (EC-GA 603266 in HEALTH.2013.2.4.2-2) DFG (DE 1361/14-1, DE 1361/18-1, BIOQIC GRK 2260/1, Radiomics DE 1361/19-1 [428222922] and 20-1 [428223139] in SPP 2177/1), GUIDE-IT (DE 1361/24-1), Berlin University Alliance (GC_SC_PC 27), G-BA (01NVF23002), Berlin Institute of Health (Digital Health Accelerator). Author: Cardiac CT (Springer Nature) Patent Holder: Patent on fractal analysis of perfusion imaging (jointly with Florian Michallek, EPO 2022 EP3350773A1, and USPTO 2021 10,991,109, approved) Research/Grant Support: Siemens, General Electric, Philips, Canon. Other: Hands-on cardiac CT courses (www.ct-kurs.de) Institutional research agreements: Siemens, General Electric, Philips, Canon. Patent on fractal analysis of perfusion imaging (jointly with Florian Michallek, EPO 2022 EP3350773A1, and USPTO 2021 10,991,109, approved)
Maria Bosserdt: Nothing to disclose

**CAD-Man EXTEND: Long-term clinical results of a single centre randomised controlled trial comparing CT with ICA**

*A-M. Stantien*[1], F. Biavati[1], A-C. Stahl[1], S. Chimed[1], M. Mohamed[1], L. M. Serna Higuita[2], M. Bosserdt[1], M. Dewey[1]; [1]Berlin/DE, [2]Tübingen/DE

**Purpose or Learning Objective:** To investigate major adverse cardiovascular events (MACE) after 10 years of follow-up in patients with an intermediate probability of coronary artery disease (CAD) undergoing computed tomography (CT) or invasive coronary angiography (ICA).
**Methods or Background:** This is the 10-year long-term clinical follow-up of the single centre randomised CAD-Man (Coronary Artery Disease Management) trial comparing CT with ICA in patients with atypical angina or chest pain clinically referred for ICA. Clinical follow-up was done by interviewing patients using questionnaires asking about MACE (myocardial infarction, stroke, unstable angina, (surgical) re-/revascularization, cardiac death) in the past 10 years. Additionally, every patient was offered a cardiac CT scan and blood samples were taken. The association between randomisation group and MACE was assessed using a multivariate Cox proportional hazards model.
**Results or Findings:** Out of 329 patients included in CAD-Man 106 patients completed the clinical long-term follow-up resulting in a total median follow-up of 4.9 years. Additional 18 MACE cases occurred, 7/49 (14%) of which in the CT group and 11/57 (19%) in the ICA group. There was no statistically significant difference in MACE between the two randomisation arms. The HR was 0.86 (95% CI 0.42–1.74) in the CT group.
**Conclusion:** After 10 years of follow-up, the survival and the occurrence of MACE was similar in the CT group compared to the ICA group for patients referred for ICA because of atypical angina or stable chest pain and an intermediate pretest probability of CAD.
**Limitations:** The limitations of the study are the single centre design and the low number of MACE, limiting the generalisability of our findings.
**Funding for this study:** This study was funded by a grant of the Heisenberg programme.
**Ethics committee - additional information:** The study was approved by ethics committee at Charité (EA1/124/23).
**Author Disclosures:**
Mahmoud Mohamed: Nothing to disclose
Anne-Marieke Stantien: Nothing to disclose
Lina Maria Serna Higuita: Nothing to disclose
Surenjav Chimed: Nothing to disclose
Marc Dewey: Research/Grant Support: Institutional research agreements with Siemens, General Electric, Philips, Canon. Grant Recipient: EU (EC-GA 603266 in HEALTH.2013.2.4.2-2) DFG (DE 1361/14-1, DE 1361/18-1, BIOQIC GRK 2260/1, Radiomics DE 1361/19-1 [428222922] and 20-1 [428223139] in SPP 2177/1), GUIDE-IT (DE 1361/24-1), Berlin University Alliance (GC_SC_PC 27), G-BA (01NVF23002), Berlin Institute of Health (Digital Health Accelerator). Patent Holder: Patent on fractal analysis of perfusion imaging (jointly with Florian Michallek, EPO 2022 EP3350773A1, and USPTO 2021 10,991,109, approved) Author: Cardiac CT (Springer Nature). Other: Hands-on cardiac CT-couses (www.ct-kurs.de) Board Member: European Society of Radiology (ESR) Publications Chair (2022-2025)
Ann-Christine Stahl: Nothing to disclose
Federico Biavati: Nothing to disclose
Maria Bosserdt: Nothing to disclose

**Improved Prediction of Obstructive Coronary Artery Disease by DISCHARGE Trial Pretest Calculator Combined with Cardiac CT**

*M. Mohamed*[1], M. Dewey[1], V. Wieske[1], P. Schlattmann[2], R. Haase[1], J. Dodd[3]; [1]Berlin/DE, [2]Jena/DE, [3]Dublin/IE

**Purpose or Learning Objective:** To evaluate the accuracy of pretest probability (PTP) calculations alone and in combination with computed tomography angiography (CTA) results for the diagnosis of CAD in stable chest pain.
**Methods or Background:** Individual patient data (IPD) meta-analysis of 65 prospective diagnostic accuracy studies of patients clinically referred to invasive coronary angiography (ICA) with stable chest pain in 22 countries. Three clinical probability models a PTP model based on age, sex and chest pain type (termed the updated DISCHARGE trial PTP calculator), a CTA alone model and the updated DISCHARGE Trial PTP calculator and CTA model combined were constructed. The models were built by multivariable logistic regressions with a dataset-specific random intercept and were compared using the area under the receiver-operating-characteristic curve (AUC) and the decision curve analysis (DCA).
**Results or Findings:** 5332 stable chest pain patients with clinically indicated ICA were included. The updated DISCHARGE pretest probability calculator was more accurate than the original Diamond-Forrester model (AUC: 0.68, 95% CI: 0.66-0.69 versus 0.63, 95% CI: 0.62-0.65). The combination of DISCHARGE pretest probability calculator with CTA findings significantly improved accuracy compared with either model alone (AUC: 0.86, 95% CI: 0.85-0.87 versus 0.81, 95% CI: 0.80-0.82). The improved prediction of CAD by combining CTA with the updated DISCHARGE Trial PTP calculator prediction model was consistent in the DCA with an increased net benefit for all chest pain types and was almost equally seen in patients with typical or atypical angina (0.85, 95% CI: 0.84-0.86) and nonanginal or other chest discomfort (0.88, 95% CI: 0.86-0.89).
**Conclusion:** Combining the updated DISCHARGE trial PTP calculator with CTA provides more accurate prediction than CTA alone for the diagnosis of obstructive CAD.
**Limitations:** The ICA indication resulted in a relatively high CAD prevalence of 48.3%.
**Funding for this study:** The COME-CCT Consortium is funded by a joint program of the German Research Foundation and the German Federal Ministry of Education and Research (01KG1110) and the Digital Health Accelerator of the Berlin Institute of Health to Marc Dewey. All researchers are independent of the funding bodies. The funding bodies had no role in the study design; in the collection, analysis and interpretation of data; in the writing of the report; and in the decision to submit the manuscript for publication.
**Ethics committee - additional information:** This study was approved by the local research ethics committee of Charité (EA-1-080-08) and the German Federal Office for Radiation Protection (Z5-22462/2-2008-048). All patients gave written informed consent before randomisation.
**Author Disclosures:**
Jonathan Dodd: Nothing to disclose
Mahmoud Mohamed: Nothing to disclose
Peter Schlattmann: Nothing to disclose
Viktoria Wieske: Nothing to disclose
Marc Dewey: Nothing to disclose
Robert Haase: Nothing to disclose

**Pericoronary adipose tissue volume but not attenuation is associated with quantitative coronary plaque metrics on coronary CT angiography: Insights from the PROMISE trial**

*M. C. Langenbach*[1], I. Hadzic[1], T. Mayrhofer[1], J. Karady[1], S. Shah[2], M. T. Lu[1], M. Ferencik[1], P. Douglas[2], B. Foldyna[1]; [1]Boston, MA/US, [2]Durham/US
*(marcel.langenbach@me.com)*

**Purpose or Learning Objective:** Pericoronary adipose tissue (PCAT) is related to pericoronary inflammation and contributes to atherogenesis and adverse outcomes. We investigated the association between PCAT and advanced plaque characteristics in patients with stable chest pain.
**Methods or Background:** PCAT was quantified around all three major epicardial vessels on non-contrast CT images from the PROMISE trial using a validated deep-learning algorithm. Quantitative coronary plaque metrics included total plaque volume and burden (TPV, $mm^3$; TPB, %), and plaque composition (calcified plaque (CP), non-calcified plaque (NCP), and low-density NCP (LD-NCP; <30HU). Multivariable linear regression analyses related global PCAT density (per 10HU) and BSA-indexed PCAT volume (per 10 $cm^3/m^2$) to plaque metrics (per 10$mm^3$/1%), adjusted for signal-to-noise ratio, tube voltage, risk score, and BMI.
**Results or Findings:** In 3,620 participants (age: 60±8 years; women: 1,945(51.2%), mean total heart PCAT volume and density were 12.9±3.1$cm^3/m^2$ and -81.4±6.4HU. Greater PCAT volume related to higher CP volume (Coef. 2.24, 95%CI:0.40–4.08, p=0.017), LD-NCP volume (Coef. 0.28, 95%CI:0.05–0.52, p=0.018), and TPV (Coef. 3.84, 95%CI:-0.21–7.88, p=0.06). A 10 $cm^3/m^2$ increase in PCAT volume was associated with a 7% higher TPB (Coef. 6.93, 95%CI:3.58–10.28, p<0.001), 4% higher CP burden (Coef. 3.97, 95%CI:2.42–5.51, p<0.001), 3% higher NCP burden (Coef. 2.96, 95%CI:0.33–5.59, p=0.027), and 1% higher LD-NCP burden (Coef. 0.68, 95%CI:0.34–1.01, p<0.001). PCAT density showed no significant association with plaque volume and burden including composition.
**Conclusion:** PCAT volume is associated with plaque volume, burden, and composition suggesting a relationship with both atherogenesis and plaque architecture. In contrast, PCAT density, a known measure of inflammation, was not associated with either. These findings emphasize the complex physiology of pericoronary fat and underscore the need to further investigate PCAT's potential role as a target for treatment interventions.
**Limitations:** Secondary analysis
**Funding for this study:** DFG (project number: 502109212)
**Ethics committee - additional information:** Massachusetts General Hospital (2009P002231)
**Author Disclosures:**
Ibrahim Hadzic: Nothing to disclose
Pamela Douglas: Nothing to disclose
Thomas Mayrhofer: Nothing to disclose
Borek Foldyna: Nothing to disclose
Maros Ferencik: Nothing to disclose
Marcel Christian Langenbach: Grant Recipient: DFG research grant
Julia Karady: Nothing to disclose
Michael T. Lu: Nothing to disclose
Svati Shah: Nothing to disclose

**Evaluating Radiation Exposure in Patients with Stable chest Pain in the DISCHARGE trial**

M. Bosserdt[1], M. Mohamed[1], M. C. Williams[2], M. Dewey[1], *J. Knape*[1];
[1]Berlin/DE, [2]Edinburgh/UK

**Purpose or Learning Objective:** To assess 3.5 years of cumulative radiation doses of cardiovascular imaging to participants undergoing computed tomography (CT) or invasive coronary angiography (ICA) for suspected coronary artery disease.
**Methods or Background:** This is a prespecified analysis of a multicentre, randomised DISCHARGE trial involving 3561 participants with stable chest and who were referred for ICA, conducted between October 2015 and April 2019 in 26 European centres. Participants were randomised to either CT (1808) or ICA (1753). Radiation dose from CT (dose-length product), SPECT (injected activity), PET-CT (injected activity) and ICA (kern-area product) was assessed for 3.5 years after randomisation. Effective dose was calculated using conversion factors appropriate for the imaging modality. Missing data were imputed using the mean. Wilcoxon rank sum test was used to assess group differences.
**Results or Findings:** Over a median follow-up period of 3.5 years, a total of 1845 (CT: 1796 vs. ICA: 49) CT scans, 1939 (CT: 344 vs. ICA: 1595) ICA without PCI, 584 (CT: 269 vs. ICA: 315) ICA with PCI, 75 (CT: 46 vs. ICA: 29) SPECT and 81 (CT: 66 vs. ICA: 15) PET-CT were performed. Total per-participant cumulative dose was higher in the CT group (median, 6.1 mSv; IQR, 3.9-10.3 mSv) compared with ICA group (median, 4.4 mSv; IQR, 2.2-9.1 mSv, P<0.001). The cumulative dose varied across participating centre, with a mean 9-fold for CT-group and 4-fold for ICA-group variation between the highest and lowest dose.
**Conclusion:** The cumulative radiation dose over 3.5 years was higher in the CT group compared to the ICA group. However, the total radiation dose for the CT and ICA group differed greatly among the participating centres.
**Limitations:** At a follow-up radiation dose was not available for all procedures
**Funding for this study:** This study was funded by grants from the EU-FP7 Framework Program (FP 2007-2013, EC-GA 603266)
**Ethics committee - additional information:** The study was approved by ethics committee at Charité (EA1/294/13)).
**Author Disclosures:**
Mahmoud Mohamed: Nothing to disclose
Marc Dewey: Grant Recipient: EU (EC-GA 603266 in HEALTH.2013.2.4.2-2) DFG (DE 1361/14-1, DE 1361/18-1, BIOQIC GRK 2260/1, Radiomics DE 1361/19-1 [428222922] and 20-1 [428223139] in SPP 2177/1), GUIDE-IT (DE 1361/24-1), Berlin University Alliance (GC_SC_PC 27), G-BA (01NVF23002), Berlin Institute of Health (Digital Health Accelerator). Other: Editor: Cardiac CT (Springer Nature). Hands-on cardiac CT courses (www.ct-kurs.de) Institutional research agreements: Siemens, General Electric, Philips, Canon. Patent on fractal analysis of perfusion imaging (jointly with Florian Michallek, EPO 2022 EP3350773A1, and USPTO 2021 10,991,109, approved) M.D. is European Society of Radiology (ESR) Publications Chair (2022-2025); the opinions expressed in this presentation are the author's own and do not represent the view of ESR.
Michelle Claire Williams: Nothing to disclose
Maria Bosserdt: Nothing to disclose
Jakob Knape: Nothing to disclose

**11:30-12:30 Research Stage 3**

### Research Presentation Session: Interventional Radiology

### RPS 2409

### Hepato-biliary interventions in benign conditions

**Moderator**
G. Maleux; Leuven/BE
*(Geert.Maleux@uzleuven.be)*

**Multicenter outcomes analysis of self-expandable biodegradable stents for the management of benign biliary strictures in 81 patients with pediatric liver transplantation**

*P. Marra*[1], D. Barnés Navarro[2], L. F. Fernández Rodríguez[3], G. Barbiero[4], S. Mcguirk[5], C. Gonzalez-Junyent[2], T. Hernández Cabrero[3], M. C. Minà[4], S. Sironi[1]; [1]Bergamo/IT, [2]Barcelona/ES, [3]Madrid/ES, [4]Padova/IT, [5]Birmingham/UK
*(pmarra@asst-pg23.it)*

**Purpose or Learning Objective:** Percutaneous transhepatic cholangiography (PTC), billioplasty and biliary drainage are routine treatments for benign strictures after pediatric liver transplantation (pLT). This multicentric study evaluated the efficacy and safety of biodegradable biliary stents.
**Methods or Background:** ELLA (Ella-CS Ltd) is self-expandable stent made of polydioxanone which degrades in 3-6 months. We evaluated a total of 81 patients (39 females, median age 4 y/o; 42 males, median age 5 y/o) with benign biliary strictures developed after pLT in five European centers. All the patients underwent percutaneous bilioplasty followed by ELLA stent placement between October 2014 and March 2024. Stricture features and treatment timing were assessed. Efficacy in terms of freedom from stricture recurrence and safety in terms of complications were analyzed.
**Results or Findings:** Regarding stricture features, 42.7% of strictures had and extension <1 cm, 57.3% ≥1 cm; 69.7% of patients had strictures at the hepaticojejunostomy, 2.5% had intrahepatic strictures and 28% had both intrahepatic and anastomotic strictures. The time between PTC and stent placement varied from 0 to 744 days (median of 36 days, IQR 61 days) with maintainance of a drainage; 53/81 (65.4%) patients underwent additional bilioplasty sessions before ELLA placement. Successful stent placement was achieved in 100% of cases and complications occurred in 23.5% of cases, all minor, mostly infectious cholangitis. Eighteen patients had a stricture recurrence (22.2%) during a median follow-up of 784 days (IQR 1200 days); of these, 55.6% were retreated with ELLA.
**Conclusion:** Biodegradable self-expandable biliary stent are be safe and effective for the treatment of benign biliary strictures after pLT. Further studies investigating factor predisposing to stent failure with standardized protocols are required to define the ideal candidates and the best timing for stenting.
**Limitations:** Retrospective design; variable protocols among centers
**Funding for this study:** None
**Ethics committee - additional information:** Comitato Etico of Bergamo - Multicenter ELLA
**Author Disclosures:**
Sandro Sironi: Nothing to disclose
Teresa Hernández Cabrero: Nothing to disclose
Giulio Barbiero: Nothing to disclose
Paolo Marra: Nothing to disclose
Simon Mcguirk: Nothing to disclose
Lucia Fernández Fernández Rodríguez: Nothing to disclose
Carla Gonzalez-Junyent: Nothing to disclose
Maria Carla Minà: Nothing to disclose
Daniel Barnés Navarro: Nothing to disclose

**Percutaneous portal vein recanalization of non-cirrhotic extrahepatic portal vein obstruction: technical considerations and clinical outcomes in 15 children**

*P. Marra*, R. Muglia, F. S. Carbone, L. Dulcetta, L. D'Antiga, S. Sironi; Bergamo/IT
*(pmarra@asst-pg23.it)*

**Purpose or Learning Objective:** Portal hypertension resulting from extrahepatic portal vein obstruction (EHPVO) in children has been managed primarily through Meso-Rex bypass. The aim of the study is to report a preliminary series of patients who underwent attempts at portal vein recanalization (PVR) prior to other types of intervention.
**Methods or Background:** A cohort of consecutive patients with EHPVO from 2021-2024 was retrospectively collected. After transjugular wedge hepatic venography for the study of the native intrahepatic portal system, percutaneous PVR was attempted via transhepatic and/or transplenic access. Clinical and procedural data, technical and clinical success, complications and follow up data were recorded. Technical success was considered at least the partial revascularization of the native portal system.
**Results or Findings:** Fifteen patients (7 males; median age 8 years) with severe portal hypertension due to EHPVO underwent 20 percutaneous transhepatic (n=1), transplenic (n=12) or simultaneous transhepatic/transplenic (n=7) attempts at portal vein recanalization. Rex vein was judged patent ad wedge hepatic venography in 2/15 (13%). Successful recanalization was achieved in 9/15 patients (60%). No major adverse events were observed. After successful angioplasty, 8/9 patients required metal stenting to obtain sustained patency. None of the failed patients was considered eligible for Meso-Rex bypass and underwent TIPS (n=2), splenectomy (n=1), surgical shunt (n=1). Two patients were followed-up without further interventions. After a median follow-up of 6 months patency of the main portal vein was demonstrated in all the patients who achieved PVR, with clinical and laboratory improvement of portal hypertension.
**Conclusion:** Our preliminary experience suggests that 60% of children with EHPVO can restore the portal flow by endovascular treatment, even with obliterated Rex vein. Thanks to its low invasiveness, PVR may be regarded as the primary intervention, before considering surgery.
**Limitations:** Retrospective study with limited sample
**Funding for this study:** None
**Ethics committee - additional information:** EC of Bergamo - Portal-01
**Author Disclosures:**
Sandro Sironi: Nothing to disclose
Paolo Marra: Nothing to disclose
Francesco Saverio Carbone: Nothing to disclose
Riccardo Muglia: Nothing to disclose
Lorenzo D'Antiga: Nothing to disclose
Ludovico Dulcetta: Nothing to disclose

**Comparison of different techniques for transjugular intrahepatic portosystemic shunt creation in a retrospective series of 51 paediatric patients**

*A. Princi*, P. Marra, F. S. Carbone, L. Dulcetta, R. Muglia, S. Sironi; Bergamo/IT
*(angeloprinci1@gmail.com)*

**Purpose or Learning Objective:** TIPS creation with the standard technique may be challenging in children with low body weight, unusual anatomy, and liver grafts. This study analyses different approaches for TIPS creation in a retrospective cohort of paediatric patients.
**Methods or Background:** The retrospective single-centre cohort included 47 patients who received TIPS either with the standard (sTIPS; n:30 median age 10 years IQR8-16) or with a transhepatic/transplenic ''hybrid'' (hTIPS; hTIPS n:17 13y IQR7-16) approaches from 2005 to 2023. Inclusion criteria were age ≤ 18 years or liver graft transplanted in paediatric age. All the variables between the sTIPS and the hTIPS group were compared.
**Results or Findings:** Technical, hemodynamic, and clinical success were 100%, 100%, and 83% in sTIPS (8 permanent, 22 as bridge) while 100%, 97%, and 81% in hTIPS (10 permanent, three bridge). Patients with liver grafts were 4 (13%) in the sTIPS and 5 in the hTIPS (30%) groups, respectively. Portal cavernoma, acute portal vein thrombosis, and Budd-Chiari syndrome were significantly more prevalent in the hTIPS group (76% vs 33%, p<0.05). Indications were comparable. Covered stents were respectively employed in 90% sTIPS and 88% hTIPS, with primary-assisted patency of 100% and 93% after a median follow-up of 34 and 14 months (IQR 21-52 and 12-20). Complications included one hemoperitoneum in each group conservatively managed, one liver failure (hTIPS group) requiring urgent transplantation and one septic shock (sTIPS group). Eight shunt dysfunctions in sTIPS and 3 in hTIPS were all successfully treated.
**Conclusion:** In a retrospective cohort of children, hybrid techniques for TIPS creation achieved high success rates comparable to the standard technique despite unfavourable baseline characteristics. Further studies may investigate the role of these approaches in expanding TIPS indication for complex scenarios in paediatric patients.
**Limitations:** Retrospective nature.
**Funding for this study:** None.
**Ethics committee - additional information:** Not applicable.
**Author Disclosures:**
Sandro Sironi: Nothing to disclose
Paolo Marra: Nothing to disclose
Francesco Saverio Carbone: Nothing to disclose
Angelo Princi: Nothing to disclose
Riccardo Muglia: Nothing to disclose
Ludovico Dulcetta: Nothing to disclose

**Transjugular Intrahepatic Portosystemic Shunt (TIPS): early laboratory changes and correlations with short-term mortality**

*F. Schön*, T. Helmberger, M. Berning, S. F. U. Blum, C. Radosa, S. Löck, R-T. Hoffmann, J-P. Kühn; Dresden/DE

**Purpose or Learning Objective:** To investigate early laboratory changes following Transjugular Intrahepatic Portosystemic Shunt (TIPS) and their associations with short-term mortality.
**Methods or Background:** TIPS procedures from 2017 to 2023 were enrolled retrospectively. Laboratory parameters (INR, ALAT, ASAT, GGT and bilirubin) were assessed once pre-procedurally, on post-procedural days 1 or 2, and once again between days 5 and 7. Percentage changes from baseline were calculated for each parameter. Temporal changes of the parameters were assessed using Kruskal-Wallis tests, and comparisons regarding 30-day mortality were evaluated using Mann-Whitney U tests.
**Results or Findings:** A total of 245 TIPS procedures (161 men, mean age 59.8 +/- 10.9 years) were enrolled, with a technical success rate of 95.5% (234/245). All laboratory parameters significantly increased post-procedurally (p < 0.001). ALAT and ASAT revealed the highest increase within the first two post-procedural days (+374 +/- 1118%, and +450 +/- 1079%, respectively), followed by decreases on days 5-7 (+279 +/- 568%, and +125 +/- 233%, respectively). Patients who died within 30 days (n = 17) had significantly higher baseline INR (p = 0.009), ASAT (p = 0.014) and bilirubin (p = 0.011), while GGT was lower (p = 0.012). 30-day mortality was associated with a higher increase of ASAT and GGT on days 1/2 (+1361 +/- 2202% vs. +415 +/- 954%, p = 0.029; and +56 +/- 101% vs. +21 +/- 51%, p = 0.034, respectively).
**Conclusion:** TIPS significantly impacts liver function, with marked early increases of ALAT and ASAT levels. Pronounced increase of laboratory parameters within the first two post-procedural days might help to identify high-risk patients in terms of short-term mortality.
**Limitations:** Retrospective study design with a relatively small number of patients.
**Funding for this study:** Not applicable.
**Ethics committee - additional information:** The present study was approved by the local ethics committee (BO-EK-501122023).
**Author Disclosures:**
Ralf-Thorsten Hoffmann: Nothing to disclose
Marco Berning: Nothing to disclose
Jens-Peter Kühn: Nothing to disclose
Felix Schön: Nothing to disclose
Christoph Radosa: Nothing to disclose
Sophia Freya Ulrike Blum: Nothing to disclose
Thomas Helmberger: Nothing to disclose
Steffen Löck: Nothing to disclose

**Successful approach to giant hydatid cysts of liver**

*U. Koç*, C. Aydın, M. Özdemir; Ankara/TR

**Purpose or Learning Objective:** We aimed to evaluate cases of giant liver hydatid cysts.
**Methods or Background:** Between December 2020 and January 2023, out of 100 liver hydatid cysts treated with the percutaneous approach in our interventional radiology department, 35 cases were more than 10 cm in one of the diameters. These giant hydatid cysts were treated with the catheterization approach which includes trochar style puncture, aspiration of the cavity, and installation with hypertonic saline solution. Then, ethanol installation of the cyst is the next step if the cavity is not connected with the biliary tree.
**Results or Findings:** Out of 35 patients, 27 (78%) patients were treated successfully with catheter approach solely without the need of ERCP related interventions. 8 patients (22%) needed further interventions with ERCP because of cystobiliary fistula; 2 out of 8 patients had biliary passage confirmed after contrast installation at catheterization. Other 6 patients did not have opaque drainage into the biliary system, rather they were suspected of having a fistula because of yellow content or biochemistry showing high bilirubin. These 8 patients had extended days of external catheter duration, about 20 days on average; while other patients had the external catheter removed approximately 2 days later. 1 patient had to go through cystectomy

Sunday operation because of insufficient drainage after repeated ERCP interventions, and this patient had the longest hospitalization duration of 100 days; whilst other ERCP patients had 20 days of hospitalization on average.
**Conclusion:** Giant hydatid cysts are manageable with a percutaneous approach. Cystobiliary fistula must be kept in mind especially for the giant hydatid cysts, since size is an important predictor for this communication.
**Limitations:** As a limitation, this study did not compare the method with others, but a future study will address this.
**Funding for this study:** None
**Ethics committee - additional information:** None
**Author Disclosures:**
Mustafa Özdemir: Nothing to disclose
Ural Koç: Nothing to disclose
Ceren Aydın: Nothing to disclose

**Incidence of Bleeding Between Percutaneous vs. Endoscopic Biliary Diversion in Patients with Biliary Tract Obstruction: A Systematic Review and Meta-Analysis**
*E. D. L. A. Salazar Perez*, E. E. Lozada Hernandez,
B. E. E. Retamoza Rojas; Leon/MX
*(estrella.asp.16.02@gmail.com)*

**Purpose or Learning Objective:** This meta-analysis examines the incidence of bleeding in patients undergoing biliary diversion via two alternatives to ERCP: percutaneous and endoscopic methods. The analysis aims to establish a foundation for identifying the most suitable treatment by evaluating effectiveness and safety. The primary goal of managing biliary tract obstruction is to achieve safe and effective drainage, with proper patient selection and diagnosis being essential for determining the best procedure.
**Methods or Background:** Eleven studies were analyzed regarding the incidence of bleeding, pancreatitis, and reinterventions associated with endoscopic or percutaneous biliary drainage procedures from January 2010 to 2023. The evaluated aspects included sensitivity, risk of bias ratio, odds ratio, and their 95% confidence interval using a random-effects model, with effects considered statistically significant if the confidence interval was at 95%. The $I^2$ statistic was calculated to assess heterogeneity. Bias analysis was reported using funnel plot tables. Data processing was performed using the R programming language within the RStudio environment (version 4.1.0 CRAN). Any p-value less than 0.05 was considered statistically significant.
**Results or Findings:** The study evaluated complications and reinterventions for two techniques of biliary diversion: the percutaneous method, involving 2,058 patients from 11 studies, and the endoscopic method, with 7,959 patients. The overall odds ratio for bleeding was 1.81 (95% CI 0.43-7.60), with heterogeneity ($I^2$ = 74%). For acute pancreatitis, the overall odds ratio was 0.15 (95% CI 0.05-0.47) with p = 0.03. Reinterventions showed an odds ratio of 0.25, with a wide confidence interval [0.06; 1.51], reflecting high heterogeneity and variability among the studies.
**Conclusion:** The percutaneous technique, as a first-line option compared to many other emerging techniques, remains the ideal choice in many referral centers for diseases presenting with biliary tract obstruction, showing minimal complication rates.
**Limitations:** Heterogeneity.
**Funding for this study:** There is no funding or conflict of interest.
**Ethics committee - additional information:** The registration for the approval of the hospital was the CEI-004-2022
**Author Disclosures:**
Beatriz Elena Elena Retamoza Rojas: Nothing to disclose
Estrella De Los Angeles Salazar Perez: Nothing to disclose
Edgard Efren Lozada Hernandez: Nothing to disclose

**Clinical Outcomes of Separate versus Single Tract Techniques in Percutaneous Radiologic Gastrostomy with Single Gastropexy: A Multi-Center Retrospective Analysis**
*H. N. Lee*[1], S-J. Park[2], Y. Cho[3], S. Lee[1]; [1]Cheonan/KR, [2]Ansan/KR, [3]Gangneung/KR
*(kenshin_007_@naver.com)*

**Purpose or Learning Objective:** To compare the clinical outcomes of separate versus single tract techniques and to investigate predictors of complications during percutaneous radiologic gastrostomy with single gastropexy.
**Methods or Background:** Between January 2018 and January 2024, 241 consecutive patients (mean age: 68.8 ± 13.5 years; male: 73.4%) who underwent percutaneous radiologic gastrostomy with single gastropexy were enrolled. The patients were divided into two groups based on the anchoring method: the separate tract group (n = 136) and the single tract group (n = 105).
**Results or Findings:** The technical success rate was 99.3% in the separate tract group and 98.1% in the single tract group (p = 0.582). Four patients (3.81%) in the single tract group experienced intra-procedural anchor dislodgment. In 3 of these cases, technical success was achieved by deploying a second anchor. The 30-day complication rate was 12.5% in the separate tract group and 15.2% in the single tract group (p = 0.324). There was no procedure-related mortality. BMI (odds ratio 0.885, p = 0.021) was a negative predictor, while unfavorable anatomy on CT (odds ratio 2.4, p = 0.033) was a positive predictor for complication.
**Conclusion:** Although anchor dislodgment was a complication unique to the single tract technique, the two groups showed no significant difference in overall clinical outcomes. This study highlights that BMI and CT findings are more crucial predictors of complications than the choice of technique.
**Limitations:** The retrospective nature of the study leads to several biases.
**Funding for this study:** This study was not supported by any funding.
**Ethics committee - additional information:** The Institutional Review Board of tertiary care hospitals approved this retrospective study and waived written informed consent for using clinical and imaging data.
**Author Disclosures:**
Sung-Joon Park: Nothing to disclose
Youngjong Cho: Nothing to disclose
Hyoung Nam Lee: Nothing to disclose
Sangjoon Lee: Nothing to disclose

**11:30-12:30** **Research Stage 4**

### Research Presentation Session: Musculoskeletal

### RPS 2410

### Imaging of musculoskeletal tumours

**Moderator**
I.-M. Noebauer-Huhmann; Vienna/AT
*(iris.noebauer@meduniwien.ac.at)*

**Dual-energy computed tomography parameters for the differentiation of vertebral small osteolytic metastases (SOMs) and SOM-mimics**
*J. Li*[1], J. Liu[2]; [1]Fujian/CN, [2]Xiamen/CN
*(1508883851@qq.com)*

**Purpose or Learning Objective:** To evaluate the value of dual energy computed tomography (DECT) quantitative parameters for the differentiation of small osteolytic metastases (SOMs) and SOM-mimics such as osteopenia, osteoporosis, and Schmorl's nodule.
**Methods or Background:** Fat(HAP), fat(calcium), hydroxyapatite(fat), and calcium(fat) densities [Dfat(HAP), Dfat(calcium), DHAP(fat), and Dcalcium(fat)], as well as CT value were collected. Comparisons were made using the independent sample T test. Diagnostic performance was assessed in terms of area under the receiver operating characteristic curve (AUC). The sensitivity, specificity, positive predictive value (PPV), negative predictive value (NPV), and accuracy of each parameter was assessed as well.
**Results or Findings:** A total of 106 patients were included, of whom 24 had SOMs (lesion, n = 48), while 82 had SOM-mimics (lesion, n = 202). SOMs associated with significantly higher CT value, Dfat(calcium), and Dfat(HAP) compared to SOM-mimics (P < .001). The AUCs were 0.674, 0.879, and 0.887, respectively. The sensitivity, specificity, PPV, NPV, and accuracy of fat(calcium) were 77.1%, 85.1%, 55.2%, 94.0%, 83.6%, respectively; while those for Dfat(HAP) were 83.3%, 80.7%, 50.6%, 95.3%, 81.2%, respectively. The optimal diagnostic cutoffs for Dfat(calcium) and Dfat(HAP) were ≥ 1000.0 mg/cm3 and ≥ 966.9 mg/cm3, respectively, which achieved consistent diagnostic results among 89.6% lesions (n = 224). The combined use of Dfat(HAP) and Dfat(calcium) achieved significantly better diagnostic performance, with AUC, sensitivity, specificity, PPV, NPV, and accuracy of 0.910, 82.2%, 87.2%, 61.7%, 95.1%, and 86.2%, respectively.
**Conclusion:** Dfat(calcium) and Dfat(HAP) on DECT carry the potential as parameters for the discrimination of SOMs from SOM-mimics
**Limitations:** This was a retrospective study with a relatively small sample size. In addition, the focus on thoracolumbar lesions limits the generalizability of our results.
**Funding for this study:** Natural Science Foundation of Fujian Province, China (grant numbers: 2023J01181)
**Ethics committee - additional information:** Fujian Cancer Hospital Ethics Committee (K2023-198-01)
**Author Disclosures:**
Jianfang Liu: Nothing to disclose
Jie Li: Nothing to disclose

**Multimodal machine learning method for the identification of prognostic and predictive biomarkers in Adolescent and Young Adults (AYA) sarcoma: a pilot study**
*S. Lusi*, R. Romanelli, A. Marzullo, A. Laffi, A. Santoro, L. Balzarini, M. Francone, A. F. Bertuzzi; Milan/IT

**Purpose or Learning Objective:** This study relies on the use of Artificial Intelligence (AI) to develop and validate a multimodal machine learning method that could provide a prognostic model in AYA patients affected by sarcomas, exploring the clinical, radiomic and pathological features that may be predictive of disease outcome.
**Methods or Background:** The study is a monocentric retrospective cohort study involving 245 patients with sarcomas. Our preliminary and full results were performed on a smaller cohort of 22 patients with soft tissue sarcoma of the extremities (13 non-AYA and 9 AYA) for whom clinical data (using Excel form), radiomic features (from a pre-treatment MRI) and histopathological features (extracted using a foundation model) were collected. All this data was then used to match similar patient profiles in the two groups using logistic regression propensity scores. Disease-free survival of matched patients was described using a Kaplan-Meier curve.
**Results or Findings:** Statistical analysis didn't identify any correlation between clinical and radiological features that could explain the differences in prognosis between the two groups, probably due to the small cohort size. However, AI analysis using a Kaplan-Meier curve showed that AYA patients had a worse prognosis than non-AYA patients ($p < 0.05$), confirming for the first time, to our knowledge, by deep machine learning what is observed in clinical practice.
**Conclusion:** Despite the limitations of these preliminary results based on a small cohort of patients, our findings provide valuable insights into the differential prognosis that characterises these two groups. AI holds promise for uncovering hidden characteristics, with future research potentially incorporating biological markers to further explore therapeutic targets.
**Limitations:** The small cohort size of the study limits its statistical power. The retrospective design may introduce selection bias. Future studies in larger populations are needed.
**Funding for this study:** No funding was received for this study.
**Ethics committee - additional information:** The study is retrospective.
**Author Disclosures:**
Alexia Francesca Bertuzzi: Nothing to disclose
Armando Santoro: Nothing to disclose
Aldo Marzullo: Nothing to disclose
Alice Laffi: Nothing to disclose
Marco Francone: Nothing to disclose
Roberta Romanelli: Nothing to disclose
Stefano Lusi: Nothing to disclose
Luca Balzarini: Nothing to disclose

**Radiomics in MRI to improve the characterization of cartilaginous bone tumours**
*Q. Bui*, M. Lacroix, L. S. Fournier, F. Larousserie, A. Feydy; Paris/FR

**Purpose or Learning Objective:** In long bones, distinguishing between enchondromas and chondrosarcomas before surgery is often challenging and may require invasive biopsy for accurate diagnosis. The purpose of this work was to assess the performance of MRI radiomics-based machine learning in classifying enchondromas and chondrosarcomas in long bones.
**Methods or Background:** Ninety-eight patients with pathology-proven cartilaginous tumours of long bones were retrospectively included from a tertiary bone tumour centre. The training set consisted of 81 MRI (n = 33 enchondromas; n = 48 chondrosarcomas). The internal test set consisted of 17 MRI (n = 7 enchondromas; n = 10 chondrosarcomas). 3D segmentation was performed on T1-weighted and fat-suppressed T2-weighted MRI images and radiomics features were extracted. Dimensionality reduction was performed based on reproducibility, redundancy and feature importance. Different models were tested, including multiparametric, single sequence and sequential. A Random Forest classifier was tuned on the training set using five-fold cross-validation and tested on the internal test set.
**Results or Findings:** The Random Forest classifier with the T2 then T1 sequential model, which was the best-performing model, achieved an AUC of 0.943 [0.832 – 1.000] on the internal test set. Its accuracy in correctly classifying enchondromas and chondrosarcomas was 71% (5/7) and 100% (10/10), respectively.
**Conclusion:** This work shows that MRI radiomics can accurately differentiate between benign and malignant cartilaginous tumours. Although comparisons among various models did not achieve statistical significance, the data suggest that a sequential approach using single sequence models might outperform a multiparametric model. Further studies with larger sample sizes are needed to integrate these findings into clinical practice and improve preoperative diagnosis of cartilaginous tumours within the context of personalised medicine.
**Limitations:** Limitations of the study were the small sample size and the lack of an external test set.
**Funding for this study:** The author received a research grant from Societé Française de Radiologie and Assistance Publique - Hôpitaux de Paris.
**Ethics committee - additional information:** This study involved a retrospective analysis of anonymised data collected as part of routine care.
**Author Disclosures:**
Laure S. Fournier: Nothing to disclose
Antoine Feydy: Nothing to disclose
Quentin Bui: Research/Grant Support: Société Française de Radiologie Research/Grant Support: Assistance Publique - Hôpitaux de Paris
Frederique Larousserie: Nothing to disclose
Maxime Lacroix: Nothing to disclose

**Optimizing Cryoablation Outcomes in Desmoid Tumors: A Machine Learning-Driven Radiomic Analysis**
*M. E. Chevasco Hanze*, L. Ponsa Cobas, J. A. Narváez García, D. A. Sandoval Díaz, J. Hernández Gañan, J. C. Sardiñas Barrero; Barcelona/ES
*(Miguelemilioch27@gmail.com)*

**Purpose or Learning Objective:** Desmoid tumors (DT) are locally aggressive, infiltrative neoplasms with a high risk of local recurrence. Recently, percutaneous cryoablation has emerged as an alternative therapy, though its role as a salvage treatment remains unclear. This study aimed to evaluate disease progression after cryoablation at a 1-year follow-up and develop a predictive model using clinical and radiomic variables.
**Methods or Background:** We performed a retrospective analysis of patients treated with cryoablation for extra-abdominal DT from January 2018 to September 2023. Pre- and post-cryoablation contrast-enhanced MRIs were reviewed, and disease progression was defined as less than 90% necrosis or non-enhancement at follow-up. Radiomics features were extracted from T2-weighted pre-cryoablation MRIs, and data were filtered based on correlation matrices and statistical tests (T-Student, Mann-Whitney U, Chi-square). Prediction models, including LASSO, Random Forest, XGBoost, SVM, and KNN, were evaluated using ROC analysis and 5-fold cross-validation to determine the optimal approach.
**Results or Findings:** Twenty-eight patients were included (median age 43; 67% women), with a no disease progression rate of 60.71%, significantly associated with partial response on mRECIST criteria ($p = 0.022$). The Random Forest model showed the best performance (AUC = 0.77). Key predictive features included tumor diameter, sphericity, major axis length, minimum intensity, kurtosis, interquartile range, and tumor location. Tumors >61 mm, with ellipsoid shape (major axis length >79 mm) and regular form (sphericity <0.7), predicted no disease progression. Similarly, tumors <61 mm with a regular shape (sphericity <0.5) and fibrous matrix (minimum <175) predicted favorable outcomes.
**Conclusion:** Cryoablation therapy has demonstrated a good therapy for DT treatment. Radiomics shape and first order features have shown their relevance in cryoablation therapy planning as it serve as a patient selection tool.
**Limitations:** Small sample size.
No split train-test approach.
**Funding for this study:** No funding was received
**Ethics committee - additional information:** No intervention, just an observational study
**Author Disclosures:**
Daniel Alejandro Sandoval Díaz: Nothing to disclose
Laia Ponsa Cobas: Nothing to disclose
Miguel Emilio Chevasco Hanze: Nothing to disclose
Javier Hernández Gañan: Nothing to disclose
José Antonio Narváez García: Nothing to disclose
Juan Carlos Sardiñas Barrero: Nothing to disclose

**“Pseudo-CT” MRI sequences and detection of lytic lesions in multiple myeloma**
*C. Chabot*; Brussels/BE
*(caroline.chabot@hotmail.com)*

**Purpose or Learning Objective:** To assess the diagnostic accuracy, repeatability, and reproducibility of pseudo-CT MRI sequences (ZTE, BB) in detecting osteolytic lesions in MM using WB-CT as the reference standard.
**Methods or Background:** In this prospective study, consecutive patients were enrolled in our academic hospital. Inclusion criteria were newly diagnosed MM, monoclonal gammopathy of undetermined significance at high risk for MM, or suspicion of progressive MM. Participants underwent ZTE and BB sequences covering the lumbar spine, pelvis, and proximal femurs as part of 3T WB-MRI examinations, as well as clinically indicated 18F-FDG PET/CT examination that included optimized WB-CT. Ten bone regions and two scores (categorical score/semiquantitative score) were assessed by three radiologists on the ZTE, BB, and WB-CT images. The accuracy, repeatability, and reproducibility of categorical scores (Gwet agreement coefficients AC1 and AC2) and

Sunday differences in semiquantitative scores were assessed at per-sequence, per-region, and per-patient levels.
**Results or Findings:** 47 participants were included. In experienced readers, BB and ZTE showed 98% accuracy per-patient, while BB accuracy ranged from 83%–100% and ZTE from 74%–94% per-region. Increased false-negative findings in the spine ranging from 17%-23%, according to the lumbar vertebra, was observed using ZTE(P<.013). Regardless of the region (except coxal bones), differences in the BB score minus the ZTE score were positively skewed(P<.021). Repeatability was very good(AC1≥0.87), while reproducibility was at least good(AC2≥0.63).
**Conclusion:** Both MRI-based ZTE and BB pseudo-CT sequences of the lumbar spine, pelvis, and femurs demonstrated high diagnostic accuracy in detecting osteolytic lesions in MM. Compared with BB, the ZTE sequence yielded more FN findings in the spine.
**Limitations:** Pseudo-CT sequences were limited to the lumbar spine, pelvis, and femurs; the reference CT required optimization from 18F-FDG PET/CT; focus was on detecting osteolytic lesions, including nonactive ones that may persist post-treatment.
**Funding for this study:** None
**Ethics committee - additional information:** This prospective study was approved by the institutional ethics committee (2020/27JUL/380) and is registered on ClinicalTrials.gov (no. NCT05381077). Written informed consent was obtained from all participants.
**Author Disclosures:**
Caroline Chabot: Nothing to disclose

**Implementing tin prefiltration in routine clinical CT scans of the lower extremity: Impact on radiation dose**
*T. Marth*, C. Stern, R. Sutter; Zürich/CH
*(thomas-marth@hotmail.com)*

**Purpose or Learning Objective:** Several studies have demonstrated the potential of tin prefiltration to reduce the radiation dose while maintaining diagnostic quality for musculoskeletal imaging. Still, no study has reported data on the impact of radiation dose reduction on clinical routine scanning.
**Methods or Background:** Retrospective inclusion of 300 patients who underwent clinically indicated CT scans of the pelvis, knee, or ankle on a single CT scanner (SOMATOM Definition AS, Siemens Healthineers) before January 2020 (without tin filter) and after December 2020 (with tin filter). For each joint, 50 patients with tin prefiltration and 50 patients without tin prefiltration were selected.
**Results or Findings:** The CTDIvol, DLP, and effective dose were reduced significantly in all tin-prefiltered examinations compared to the conventional, non-tin-prefiltered examinations (p<.001). Tin-prefiltered CT scans had a significantly lower CTDIvol and mean effective dose (all p<.001): CTDIvol was 65% lower in the pelvis, 73% in the knee, and 54% in the ankle. This resulted in a reduction of the effective dose of 61%, 71%, and 60%, respectively. In absolute numbers, the reduction of the median effective dose delivered in a single scan of the pelvis was 2.29 mSv, 0.15 mSv for the knee, and 0.03 mSv in the ankle.
**Conclusion:** The implementation of tin prefiltration in clinical routine scan protocols significantly reduced the effective radiation dose for unenhanced CT scans of the pelvis (61% reduction, 2.29 mSv), the knee (71% reduction, 0.15 mSv), and the ankle (60% reduction, 0.03 mSv).
**Limitations:** It would be possible to reduce radiation dose even more by applying deep learning-based denoising algorithms, however this was not yet available in clinical routine during the data acquisition.
**Funding for this study:** No specific funding.
**Ethics committee - additional information:** BASEC-ID 2024-01094 Kantonale Ethikkommission Zürich
**Author Disclosures:**
Thomas Marth: Other: Balgrist University Hospital and Balgrist Campus each have an academic research collaboration with Siemens Healthineers. Balgrist University Hospital also has an academic research collaboration with Bayer.
Christoph Stern: Other: Balgrist University Hospital has an academic research collaboration with Siemens Healthineers. Balgrist University Hospital also has an academic research collaboration with Bayer.
Reto Sutter: Other: Balgrist University Hospital has an academic research collaboration with Siemens Healthineers. Balgrist University Hospital also has an academic research collaboration with Bayer.

**Evaluation of the diagnostic potential of a Tomosynthesis system for MSK**
Y. Beer[1], *N. Shabshin*[2], L. Copel[1], Y. Kimmel[3], R. Ophir[1], Y. S. Schiffenbauer[3], S. Tal[1]; [1]Zrifin/IL, [2]Afula/IL, [3]Petach Tikva/IL
*(nogah.shabshin@gmail.com)*

**Purpose or Learning Objective:** Digital tomosynthesis (DTS) is a well-established technology that has become the gold standard for breast mammography. In recent years its benefits in musculoskeletal (MSK) imaging have been acknowledged, leading to a rapid increase in its utilization. It improves detection, localization and characterization of subtle fractures. In some patients DTS can alleviate the need for CT with lower complexity. Recently a new technology based on Cold Cathode Xray tube with a multi tube set-up has made this technology more affordable and accessible. The purpose of this study is to evaluate the diagnostic potential of the cold-cathode multi-tube DTS
**Methods or Background:** The study included 19 patients with suspected fractures who underwent CT and radiographs (XR). Patients were scanned using the cold-cathode DTS . 15 patients had imaging performed with Cast or metal. Images were evaluated by an MSK radiologist and orthopedic surgeon in consensus with CT as the gold standard. Studies were evaluated for presence, location, intraarticular involvement, displaced fragments and incidental lesions. The surgeon was asked whether DTS provided valuable information and whether it increased the confidence of the final diagnosis.
**Results or Findings:** In 17/19 studies DTS added value to the XR. In 7, DTS found fractures occult in XR. In 3, DTS was able to clear a suspected fracture. in 5, DTS was able to better localize the fracture. In 1, DTS was able to determine fracture age and in 1 study DTS found a sclerotic lesion obscured in XR. In addition, on XR the cast limited evaluation of fine bony details, however there was no such limitation with DTS
**Conclusion:** Cold cathode DTS provides high quality tomography of anatomies enabling depiction of occult pathologies, localization, characterization and resolution of questionable findings
**Limitations:** Initial study results
**Funding for this study:** Study was funded by Nanox-x Imaging Ltd.
**Ethics committee - additional information:** Study was approved by the local Ethics committee in the institution and each patient underwent informed consent
**Author Disclosures:**
Robenpour Ophir: Nothing to disclose
Laurian Copel: Nothing to disclose
Yiftah Beer: Nothing to disclose
Yotam Kimmel: Nothing to disclose
Yael S Schiffenbauer: Nothing to disclose
Nogah Shabshin: Consultant: Nano-x imaging
Sigal Tal: Nothing to disclose
